# Below the Mesophotic

**DOI:** 10.1038/s41598-018-23067-1

**Published:** 2018-03-20

**Authors:** C. C. Baldwin, L. Tornabene, D. R. Robertson

**Affiliations:** 1 Department of Vertebrate Zoology, National Museum of Natural History, Smithsonian Institution, Washington, DC 20560 USA; 2 School of Aquatic and Fishery Sciences, Burke Museum of Natural History and Culture, University of Washington, Seattle, WA 98195 USA; 3 Smithsonian Tropical Research Institute, Balboa, Republic of Panama

## Abstract

Mesophotic coral ecosystems, which occur at depths of ~40 to 150 m, have received recent scientific attention as potential refugia for organisms inhabiting deteriorating shallow reefs. These ecosystems merit research in their own right, as they harbor both depth-generalist species and a distinctive reef-fish fauna. Reef ecosystems just below the mesophotic are globally underexplored, and the scant recent literature that mentions them often suggests that mesophotic ecosystems transition directly into those of the deep sea. Through submersible-based surveys in the Caribbean Sea, we amassed the most extensive database to date on reef-fish diversity between ~40 and 309 m at any single tropical location. Our data reveal a unique reef-fish assemblage living between ~130 and 309 m that, while taxonomically distinct from shallower faunas, shares strong evolutionary affinities with them. Lacking an existing name for this reef-faunal zone immediately below the mesophotic but above the deep aphotic, we propose “rariphotic.” Together with the “altiphotic,” proposed here for the shallowest reef-faunal zone, and the mesophotic, the rariphotic is part of a depth continuum of discrete faunal zones of tropical reef fishes, and perhaps of reef ecosystems in general, all of which warrant further study in light of global declines of shallow reefs.

## Introduction

Studies of deep tropical-reef ecosystems have surged during the past decade^1–10^. This is due in part to the global decline of shallow coral reefs having sparked interest in the potential for deep reefs to act as refugia for shallow-water organisms stressed by warming surface waters or deteriorating reefs. Variously called the “coral-reef twilight zone” or “deep reefs,” mesophotic coral ecosystems are light-dependent coral communities at tropical and some higher latitudes that generally are considered to extend from 30–40 m to as deep as 150 m^2,3,7,9,11^. Although the boundary between shallow and mesophotic ecosystems initially was established based on the lower limit for conventional scuba diving rather than substantial turnovers in species composition^2,4,11–13^, there is supporting biological evidence for both corals^14^ and reef fishes^15^. The lower limit of the mesophotic is defined as the maximum depth at which there is sufficient penetration of sunlight to support photosynthesis and, hence, the growth of zooxanthellate coral reefs^1,2^. Some coral biologists divide the mesophotic into upper and lower portions, with a faunal transition of species around 60 m^1,9,16^.

Fish biologists have adopted the term “mesophotic” for tropical reef fishes (demersal and cryptic species that live on or visit coral or rocky bottoms) at similar depths just below shallow areas, at ~40 to 150 m depending on the method of study and maximum depth investigated^3,7,10,13^. A faunal break among reef fishes occurs between ~60 and 90 m, thus also resulting in the recognition of upper and lower mesophotic zones for such fishes^3,7,8,10,17,18^. The species composition of reef fishes inhabiting the upper mesophotic is similar to that on shallow coral reefs, whereas species inhabiting the lower mesophotic constitute an assemblage that is taxonomically distinct from shallow-reef taxa^3,7,8,11,17^. Abundance and biomass of fish species in the upper and lower mesophotic typically vary significantly^3^.

Maximum depths of investigation between 70 and 150 m in most studies of tropical mesophotic fish communities have precluded recognition of the lower boundary of the mesophotic zone, the nature of the reef-fish fauna below that zone, and the location of any deeper faunal breaks^8,18–21^. Two studies that recorded fishes to depths of 300 m only distinguished shallow (<30 m) from “deep-reef” (>50 m) faunas^22,23^, and several recent publications depict the mesophotic transitioning directly into aphotic deep-sea ecosystems^11,24–26^. Tropical ecosystems below 150 m thus have received little targeted scientific attention, a deficiency attributable to depth limits imposed by the rebreather/mixed-gas technology commonly used to explore mesophotic reefs, to the scarcity and high costs of deep-diving ROVs and submersibles, and to the inability of trawls to sample deep rocky habitat.

Our intensive efforts over the past six years using a manned submersible to study the diversity of mesophotic and deeper tropical fish communities at Curaçao Island in the Caribbean Sea have resulted in the most extensive database to date on the diversity and depth distributions of reef fishes between ~40 and 309 m at a single location anywhere in the tropics. This data set provided the opportunity to analyze variation in faunal assemblages (species composition and abundance) at mesophotic and greater depths, the results of which we report here.

## Results and Discussion

**Figure 1:**
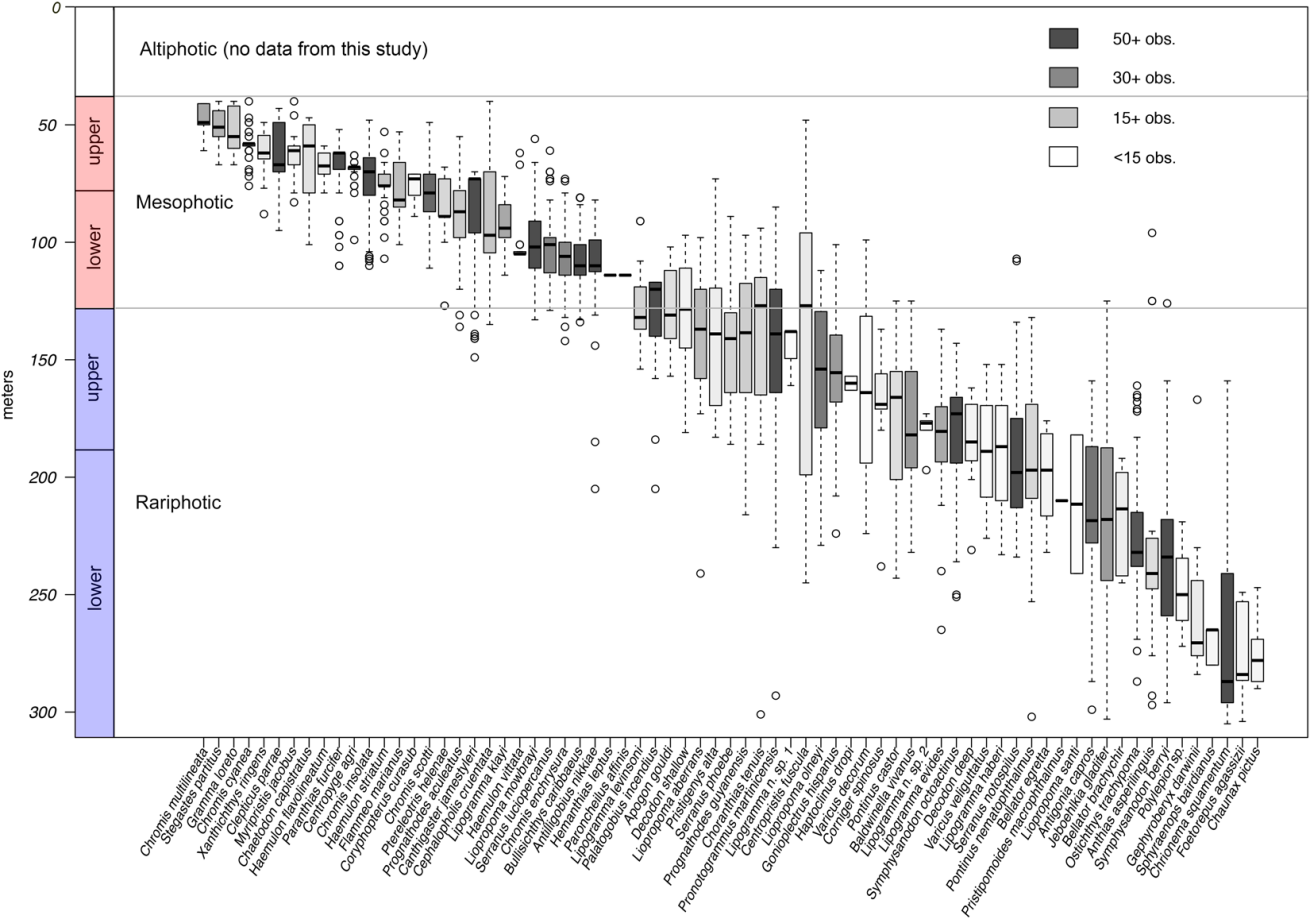
Depth distributions of 71 mesophotic and rariphotic fishes derived from 4,436 depth observations between 40 and 309 m off Curaçao, southern Caribbean. Boxes indicate the 25^th^ and 75th quantiles, whiskers are 1.5 the interquartile, and circles are outliers.

**Figure 2:**
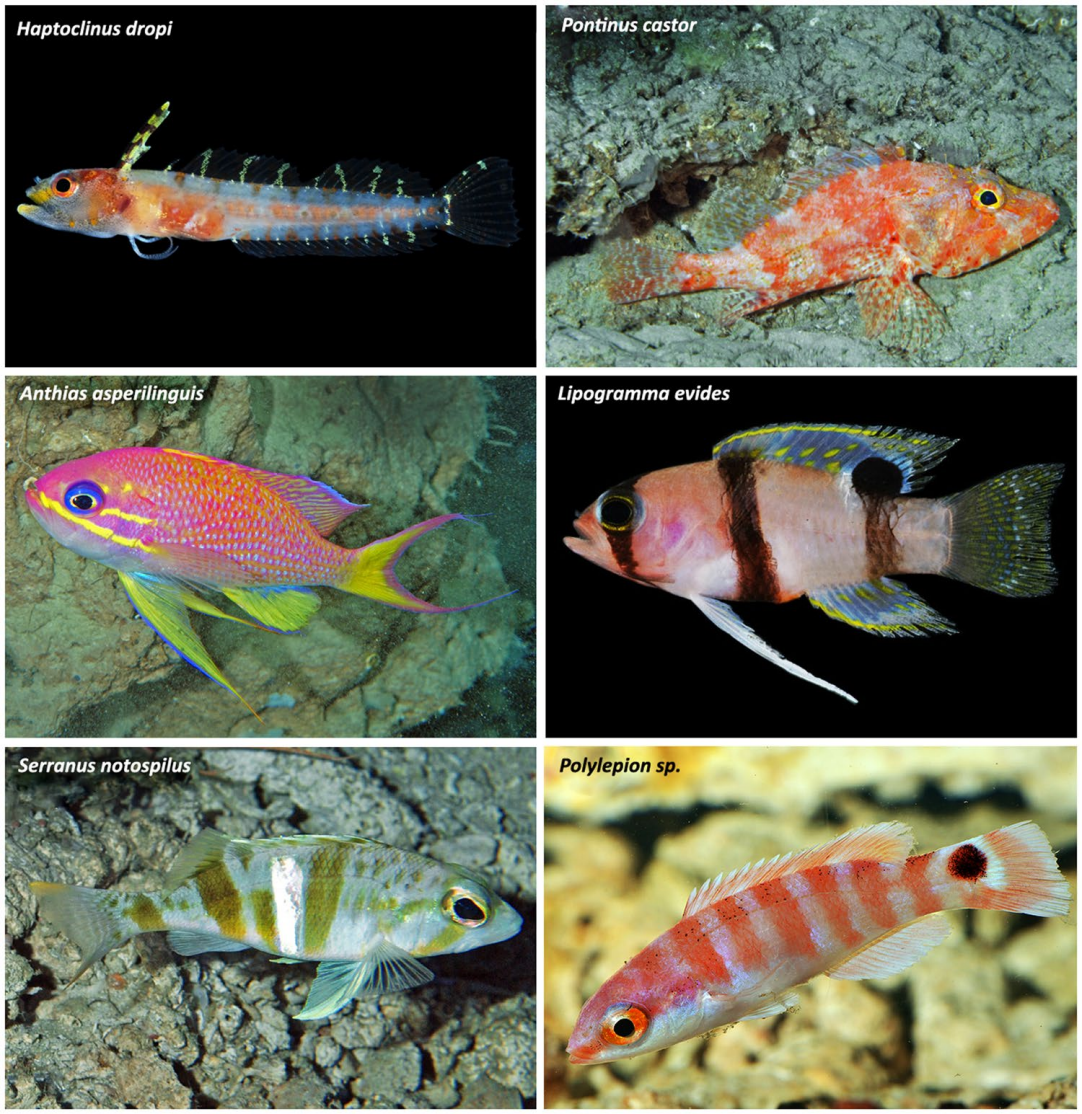
Representative Caribbean fishes inhabiting the rariphotic zone off Curaçao. *Haptoclinus dropi* (Labrisomidae); *Pontinus castor* (Scorpaenidae); *Anthias asperilinguis* (Serranidae); *Lipogramma evides* (Grammatidae); *Serranus notospilus* (Serranidae); *Polylepion sp*. (Labridae). Photograph of *A. asperilinguis* by Patrick Colin, other photographs by C. C. Baldwin and D. R. Robertson.

Our data set comprises 4,436 depth observations of 71 reef-fish species observed and unambiguously identified between 40 and 309 m off Curaçao (Fig. 1, Tables 1–3). As described herein, results of our cluster analyses of these data reveal strong faunal breaks along the depth gradient that clearly point to the existence of a faunal zone immediately below the mesophotic that extends down to at least 309 m and that is home to a unique reef-fish fauna (Fig. 2). Many component species of this fauna have been described as new species as part of our investigation^27–30^ or remain undescribed. As there is no appropriate term in existing coral-reef literature for a demersal zone that is immediately below the mesophotic but above the deep aphotic regions, we propose “rariphotic” (*rarus* = scarce), to reflect the scarcity of light at sub-mesophotic depths. Below we discuss the boundaries and taxonomic composition of this fish-faunal zone off Curaçao. Providing a name for this faunal zone, as biologists have for the mesophotic, draws attention to its existence, emphasizes that the mesophotic is not the only “deep-reef” faunal zone below shallow coral reefs, and provides terminology with which to discuss it. As there is also no equivalent term in general use for shallow coral reefs above the mesophotic that follows the same naming scheme, we propose “altiphotic” (*altus* = high) to complete this classification of coral-reef faunal zones: altiphotic, mesophotic, rariphotic.

**Table 1:** Fishes belonging primarily to the altiphotic (0–39 m) and mesophotic (40–129 m) zones based on visual observations and specimens collected between 40 and 309 m off Curaçao.

Family	Genus	species	N	% in Mesophotic	% in Rariphotic	Faunal Zone Assignment off Curaçao	Depth Range off Curaçao (m)	Global Depth Range (m)
Chaetodontidae	*Chaetodon*	*capistratus*	17	100%	0%	A/M	<40–101	0–101
Gobiidae	*Ptereleotris*	*helenae*	23	100%	0%	A/M	<40––127	3–160
Grammatidae	*Gramma*	*loreto*	27	100%	0%	A/M	<40–67	2–100
Haemulidae	*Haemulon*	*flavolineatum*	12	100%	0%	A/M	<40–79	0–79
	*Haemulon*	*vittatum*	17	100%	0%	A/M	<40–105	0–105
Holocentridae	*Myripristis*	*jacobus*	13	100%	0%	A/M	<40–83	1–210
Labridae	*Clepticus*	*parrae*	287	100%	0%	A/M	<40–95	0–145
Pomacentridae	*Chromis*	*cyanea*	42	100%	0%	A/M	<40–76	3–76
	*Chromis*	*multilineata*	49	100%	0%	A/M	<40–61	1–91
	*Stegastes*	*partitus*	42	100%	0%	A/M	<40–67	0–111
Serranidae	*Paranthias*	*furcifer*	247	100%	0%	A/M	<40–110	0–128
	*Cephalopholis*	*cruentata*	32	97%	3%	A/M	<40–135	1–170
Apogonidae	*Paroncheilus*	*affinis*	5	100%	0%	M	114	15–300
Balistidae	*Xanthichthys*	*ringens*	15	100%	0%	M	49–88	0–190
Chaetodontidae	*Prognathodes*	*aculeatus*	29	93%	7%	M	55–136	1–177
Gobiidae	*Antilligobius*	*nikkiae*	255	97%	3%	M	82–205	73–205
	*Coryphopterus*	*curasub*	5	100%	0%	M	70–97	70–97
Grammatidae	*Lipogramma*	*klayi*	49	100%	0%	M	72–114	40–150
Haemulidae	*Haemulon*	*striatum*	33	100%	0%	M	53–107	1–210
Holocentridae	*Flammeo*	*marianus*	37	100%	0%	M	53–101	15–128
Pomacanthidae	*Centropyge*	*argi*	39	100%	0%	M	63–99	5–170
Pomacentridae	*Chromis*	*insolata*	213	100%	0%	M	40–110	20–152
	*Chromis*	*scotti*	85	100%	0%	M	49–111	5–172
	*Chromis*	*cf. enchrysura*	68	94%	6%	M	73–142	70–172
Serranidae	*Bullisichthys*	*caribbaeus*	262	87%	13%	M	81–134	81–548
	*Hemanthias*	*leptus*	2	100%	0%	M	114	35–640
	*Liopropoma*	*mowbrayi*	111	98%	2%	M	56–133	24–133
	*Serranus*	*luciopercanus*	69	100%	0%	M	61–129	60–300
Tetraodontidae	*Canthigaster*	*jamestyleri*	116	94%	6%	M	70–149	25–152

A species was assigned to the mesophotic zone (M) if >75% of occurrences are in that zone, to altiphotic/mesophotic zones (A/M) if it commonly occurs at depths <40 m^32–34^. N = number of depth observations from combined visual and collection data. Global depth ranges are from data generated in this study and elsewhere^32–34^.

**Table 2:** Fishes that primarily overlap the mesophotic (40–129 m) and rariphotic (130 to at least 309 m) zones based on visual observations and specimens collected between 40 and 309 m off Curaçao.

Family	Genus	species	N	% in Mesophotic	% in Rariphotic	Faunal Zone Assignment off Curaçao	Depth Range off Curaçao (m)	Global Depth Range (m)
Apogonidae	*Apogon*	*gouldi*	18	44%	56%	R/M	102–157	55–262
Gobiidae	*Palatogobius*	*incendius*	316	70%	30%	R/M	117–205	117–158
	*Varicus*	*decorum*	3	33%	66%	R/M	99–220	99–251
Chaetodontidae	*Prognathodes*	*guyanensis*	28	36%	64%	R/M	97–216	60–250
Grammatidae	*Lipogramma*	*levinsoni*	25	40%	60%	R/M	91–154	91–154
Labridae	*Decodon*	*n. sp*.	8	50%	50%	R/M	97–181	97–181
Priacanthidae	*Pristigenys*	*alta*	11	36%	64%	R/M	73–183	5–300
Serranidae	*Centropristis*	*fuscula*	13	62%	38%	R/M	48–245	20–308
	*Choranthias*	*tenuis*	22	59%	41%	R/M	94–301	55–915
	*Liopropoma*	*aberrans*	38	42%	58%	R/M	98–241	89–241
	*Pronotogrammus*	*martinicensis*	559	38%	62%	R/M	85–293	55–300

All species listed here have <75% of occurrences in either the rariphotic (R) or mesophotic (M) zones. N = number of depth observations from combined visual and collection data. Global depth ranges are from data generated in this study and elsewhere^32–34^.

**Table 3:** Fishes belonging primarily to the rariphotic zone (130 to at least 309 m) zone based on visual observations and specimens collected between 40 and 309 m off Curaçao.

Family	Genus	species	N	% in Mesophotic	% in Rariphotic	Faunal Zone Assignment off Curaçao	Depth Range off Curaçao (m)	Global Depth Range (m)
Gobiidae	*Varicus*	*veliguttatus*	5	0%	100%	R	152–289	152–293
Grammatidae	*Lipogramma*	*evides*	48	0%	100%	R	137–265	133–365
	*Lipogramma*	*haberi*	3	0%	100%	R	153–233	153–233
	*Lipogramma*	*n. sp. 1*	3	0%	100%	R	138–161	138–161
	*Lipogramma*	*n. sp. 2*	5	0%	100%	R	173–197	173–197
Holocentridae	*Corniger*	*spinosus*	10	0%	100%	R	137–238	45–275
Labridae	*Decodon*	*puellaris*	7	0%	100%	R	162–231	18–275
	*Polylepion*	*n. sp*.	3	0%	83%	R	68–274	68–457
Labrisomidae	*Haptoclinus*	*dropi*	2	0%	100%	R	157–167	157–275
Percophidae	*Chrionema*	*squamentum*	181	0%	100%	R	159–305	115–305
Scorpaenidae	*Pontinus*	*castor*	19	10%	90%	R	125–243	32–549
	*Pontinus*	*nematophthalmus*	23	0%	100%	R	132–302	82–410
Serranidae	*Anthias*	*asperilinguis*	19	16%	84%	R	96–297	69–393
	*Baldwinella*	*cf. vivanus*	57	3%	97%	R	125–232	125–232
	*Gonioplectrus*	*hispanus*	56	9%	91%	R	101–224	35–460
	*Jeboehlkia*	*gladifer*	55	2%	98%	R	125–303	100–395
	*Liopropoma*	*olneyi*	76	25%	75%	R	112–229	112–229
	*Liopropoma*	*santi*	5	0%	100%	R	182–241	182–275
	*Serranus*	*notospilus*	101	2%	98%	R	107–234	7–234
	*Serranus*	*phoebe*	25	24%	76%	R	89–186	15–402
Symphysanodontidae	*Symphysanodon*	*berryi*	117	1%	99%	R	126–296	100–500
	*Symphysanodon*	*octoactinus*	153	0%	100%	R	143–251	130–640
Triglidae	*Bellator*	*egretta*	7	0%	100%	R	176–232	40–232
	*Bellator*	*brachychir*	10	0%	100%	R	192–245	27–366
Callionymidae	*Foetorepus*	*agassizii*	7	0%	100%	R	236–304	91–650
Caproidae	*Antigonia*	*capros*	78	0%	100%	R	159–299	50–900
Chaunacidae	*Chaunax*	*pictus*	6	0%	100%*	R	247–307	200–1183
Epigonidae	*Sphyraenops*	*bairdianus*	6	0%	100%*	R	265–280	200–1750
Holocentridae	*Ostichthys*	*trachypoma*	95	0%	100%	R	161–287	37–550
Lutjanidae	*Pristipomoides*	*macrophthalmus*	1	0%	100%	R	210–290	100–550
Trachichthyidae	*Gephyroberyx*	*darwinii*	12	0%	100%*	R	167–284	75–1250

All species listed here have >75% of occurrences in the rariphotic zone (R). N = number of depth observations from combined visual and collection data. Global depth ranges are from data generated in this study and elsewhere^32–34^. *Indicates species that also frequently inhabit the aphotic zone at depths >500 m^32–34^.

### Defining the boundaries of the rariphotic faunal zone

**Figure 3:**
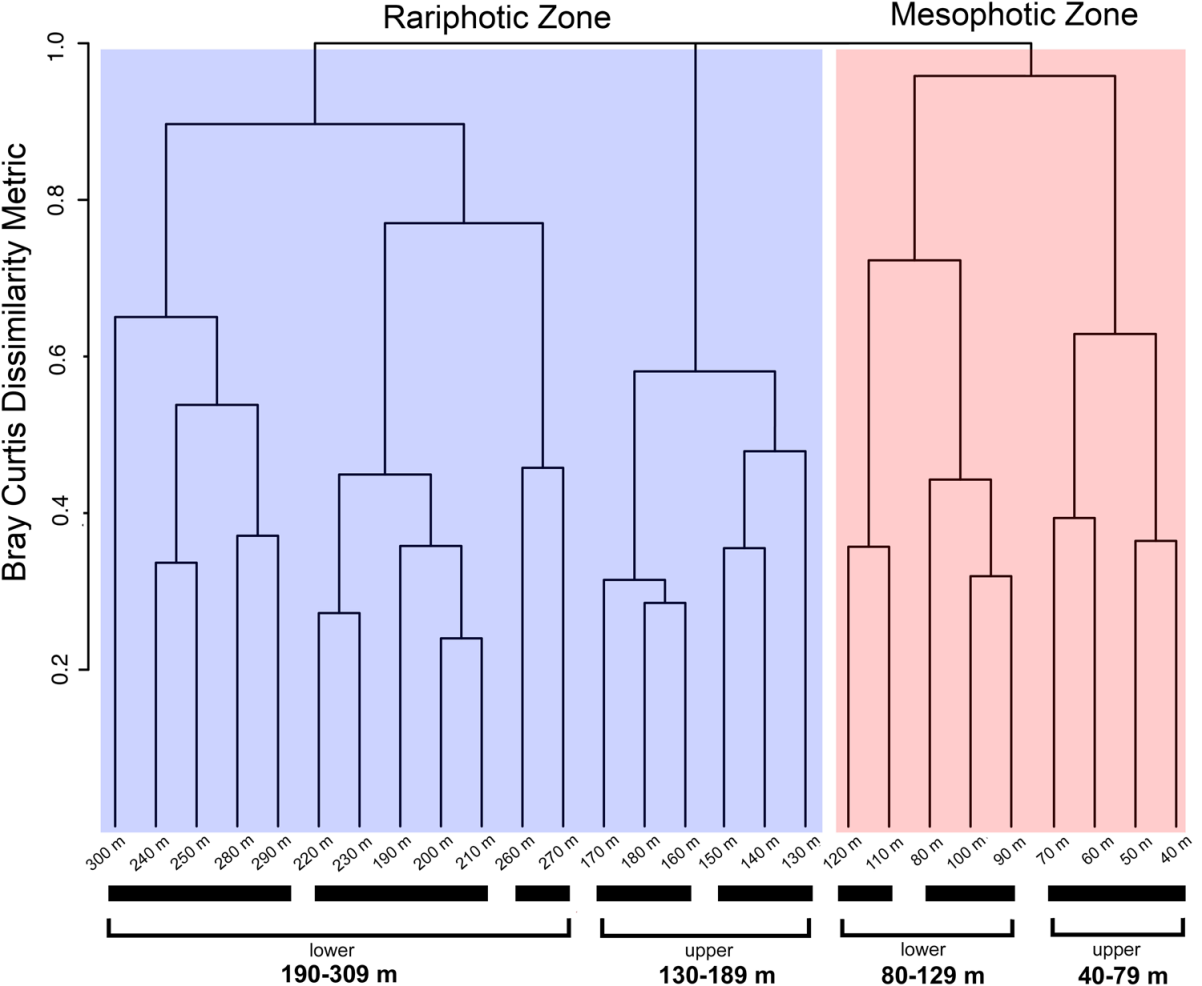
Hierarchical clustering dendrogram from the Bray-Curtis dissimilarity analysis of 4,436 depth observations of fishes between 40 and 309 m off Curaçao. Thick solid black lines below clusters indicate groups that have significantly (p < 10^−7^) distinct faunal communities based on a SIMPROF analysis. Depth bins are 10-m intervals labeled by the minimum depth in each interval (e.g., “100 m” = 100–109 m).

**Figure 4:**
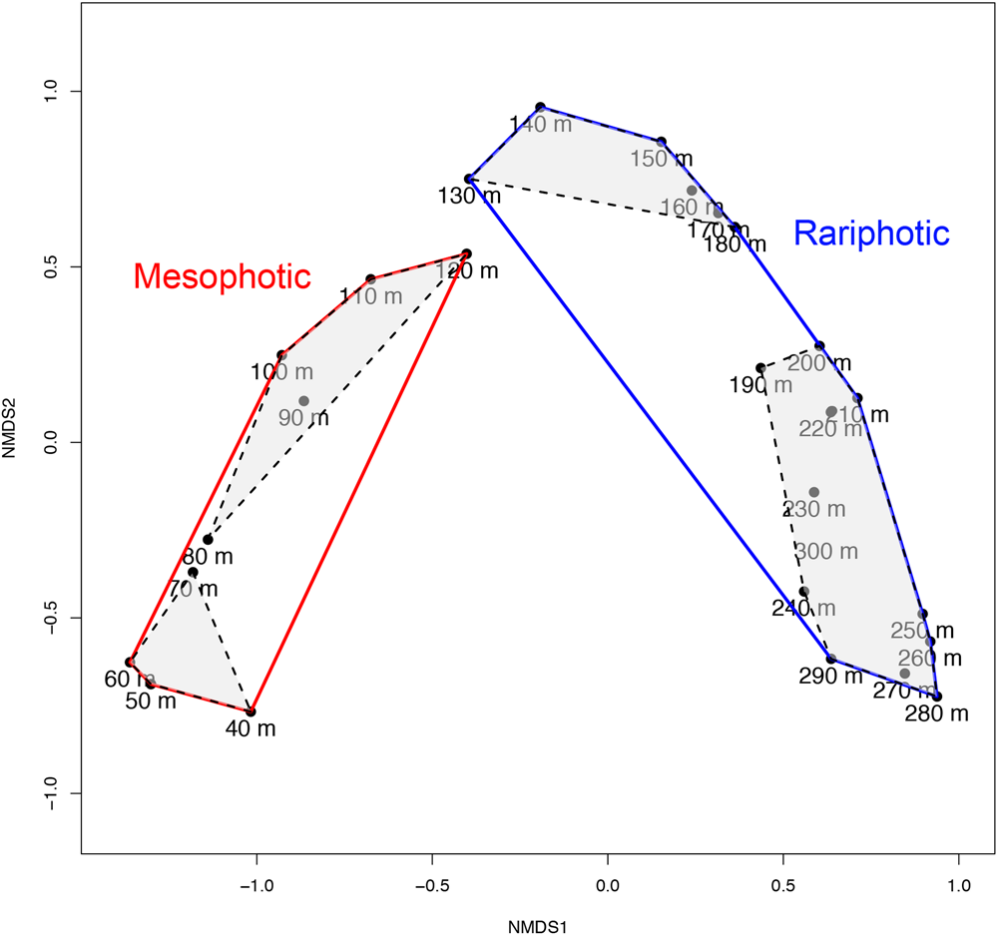
Nonmetric multidimensional scaling ordination (MDS) plot derived from the Bray-Curtis dissimilarity analysis. Red and blue polygons represent mesophotic and rariphotic zones, respectively. Shaded polygons represent upper and lower mesophotic and rariphotic sections. The two zones and four sections were derived from eight significantly different (p < 10^−7^) clusters in the SIMPROV analysis. Each *a posteriori* zone and section is significantly distinct (PERMANOVA, p < 0.01).

We first recognized the rariphotic fish assemblage from our anecdotal fish-observation and capture data, and a cluster analysis of our fish-depth observations shows that it is distinct from the mesophotic fish assemblage off Curaçao. A similarity profile (SIMPROF) analysis based on the Bray-Curtis dissimilarity metric revealed eight clusters, each significantly different (at p < 10^−7^) from the others (Figs 3 and 4): 40–79 m, 80–109 m, 110–129 m, 130–159 m, 160–189 m, 190–239, 260–279 m, and a cluster combining 240–259 m and 280–309 m depth bins. The shallowest three clusters can be combined into a ~40–129 m mesophotic zone, with a faunal break at ~80 m separating its upper (40–79) and lower (80–129) parts and providing further support for the ~80–85 m faunal break among mesophotic reef fishes previously identified by a rebreather study at Curaçao^7^. We group the remaining clusters into the rariphotic zone (~130–309 m), with a division between the two most dissimilar sections at ~190 m delineating upper (130–189) and lower (190–309) sections. Faunal breaks in reef-fish assemblages at ~120–130 m also have been identified off Hawaii and the Marshall Islands in the Pacific, as well as in the Gulf of Mexico^8,10,31^, and the ~190 m break between upper and lower rariphotic faunas at Curaçao is at the same depth as a faunal break recently identified in a study of Gulf of Mexico fishes based on depth maxima and minima^10^. The displacement of the 260–279 m depth group outside the 240–309 m cluster (Fig. 3) likely is an artefact attributable to low numbers in the 260–279 m bin of species that are common in adjacent shallower and deeper bins, and the sensitivity of the analysis to small differences, due to relatively small sample sizes (Fig. 1).

Although the data could be interpreted as supporting between three and eight distinct faunal zones, the four clusters (40–79 m upper mesophotic, 80–129 m lower mesophotic, 130–189 m upper rariphotic, and 190–309 m lower rariphotic) are significantly distinct (p < 0.01) and highly dissimilar (95–99%) from one another (Figs 3 and 4). The strongest breaks among all of the partitions occur between the mesophotic and rariphotic clusters, at 129–130 m, and between the upper and lower rariphotic clusters, at 189–190 m (Figs 3 and 4). Hence two zones, mesophotic and rariphotic—each with upper and lower sections—adequately explain the data and provide the simplest classification of faunal zones.

The key fish species responsible for divisions between the upper and lower mesophotic, lower mesophotic and upper rariphotic, and upper and lower rariphotic at Curaçao are listed in Table 4. *Clepticus parrae* (Labridae)*, Paranthias furcifer* (Serranidae), *Chromis cyanea*, and *C. multilineata* (Pomacentridae), all of which are common altiphotic species, dominate the upper mesophotic relative to the lower mesophotic. *Bullisichthys caribbaeus, Pronotogrammus martinicensis, Liopropoma mowbrayi* (Serranidae), *Antilligobius nikkiae* (Gobiidae), and *Chromis cf. enchrysura* predominantly define the lower mesophotic relative to the upper mesophotic. *Pronotogrammus martinicensis* becomes more abundant in the upper rariphotic and, along with *Palatogobius incendius* (Gobiidae) and *Symphysanodon octoactinus* (Symphysanodontidae), drives the separation between the upper rariphotic relative to the lower mesophotic. Key lower-mesophotic species driving the separation from the upper rariphotic include some of the same species that separate the upper and lower mesophotic (*B. carribaeus, A. nikkiae, L. mowbrayi*, and *C. cf. enchrysura*) but also *C. insolata, C. scotti*, and *Serranus luciopercanus* (Serranidae). Relative to the lower rariphotic, recognition of the upper rariphotic is driven by some species that also distinguish the upper rariphotic and lower mesophotic (*P. martinicensis, P. incendius*, and *S. octoactinus*) but also by several additional species of Serranidae (*Liopropoma aberrans, L. olneyi, Gonioplectrus hispanus, Serranus notospilus*, and *Baldwinella vivanus*). *Chrionema squamentum* (Percophidae) and *Symphysanodon berryi* drive the separation of the lower and upper rariphotic.

**Table 4:** SIMPER analysis results showing the relative Bray-Curtis contributions of top ten species driving differences between adjacent depth zones.

Species	Contribution to Bray-Curtis	Avg. abundance shallower zones (transformed)	Avg. abundance deeper zones (transformed)
**Upper mesophotic vs Lower mesophotic**			
*Bullisichthys caribbaeus*	0.060	0.000	12.643
*Clepticus parrae*	0.054	13.028	1.616
*Antilligobius nikkiae*	0.054	0.000	11.701
*Paranthias furcifer*	0.039	9.918	1.911
*Pronotogrammus martinicensis*	0.036	0.000	8.049
*Liopropoma mowbrayi*	0.030	2.329	8.344
*Chromis cf. enchrysura*	0.027	0.750	6.210
*Chromis cyanea*	0.026	5.114	0.000
*Chromis multilineata*	0.025	4.672	0.000
*Canthigaster jamestyleri*	0.025	3.464	4.189
**Lower mesophotic vs Upper rariphotic**			
*Bullisichthys caribbaeus*	0.052	12.643	1.850
*Antilligobius nikkiae*	0.047	11.701	1.980
*Palatogobius incendius*	0.038	5.124	5.804
*Liopropoma mowbrayi*	0.037	8.344	0.779
*Pronotogrammus martinicensis*	0.035	8.049	12.683
*Chromis insolata*	0.032	6.137	0.000
*Serranus luciopercanus*	0.026	5.884	0.655
*Symphysanodon octoactinus*	0.026	0.000	5.208
*Chromis scotti*	0.026	4.984	0.000
*Chromis cf enchrysura*	0.025	6.210	1.171
**Upper rariphotic vs. Lower rariphotic**			
*Pronotogrammus martinicensis*	0.087	12.683	1.764
*Palatogobius incendius*	0.045	5.804	0.144
*Chrionema squamentum*	0.039	0.845	5.482
*Symphysanodon octoactinus*	0.039	5.208	2.266
*Liopropoma olneyi*	0.037	5.169	0.493
*Gonioplectrus hispanus*	0.034	4.594	0.349
*Symphysanodon berryi*	0.028	1.477	4.710
*Serranus notospilus*	0.026	3.588	2.495
*Liopropoma aberrans*	0.024	3.087	0.144
*Baldwinella vivanus*	0.023	3.403	1.433

Although we lack the data necessary to identify the depth of a faunal break between rariphotic reef fishes and deep-sea fishes at Curaçao, the lower limit of the rariphotic may lie below 309 m. The global depth ranges of many of the Curaçao rariphotic reef fishes extend to 300–500 m, whereas few occur much deeper (Table 3 and references therein). Further, 500 m represents the approximate upper limit of the depth ranges of species in many Caribbean deep-sea families. For example, in the northeastern Caribbean, representatives of the demersal deep-sea fish families Halosauridae, Nettastomatidae, Ateleopodidae, Polymixiidae, Berycidae, Zeniontidae, and Draconettidae were observed at minimum depths of approximately 500 m during recent extensive ROV surveys^24^.

### Taxonomic composition of the rariphotic fish assemblage

**Figure 5:**
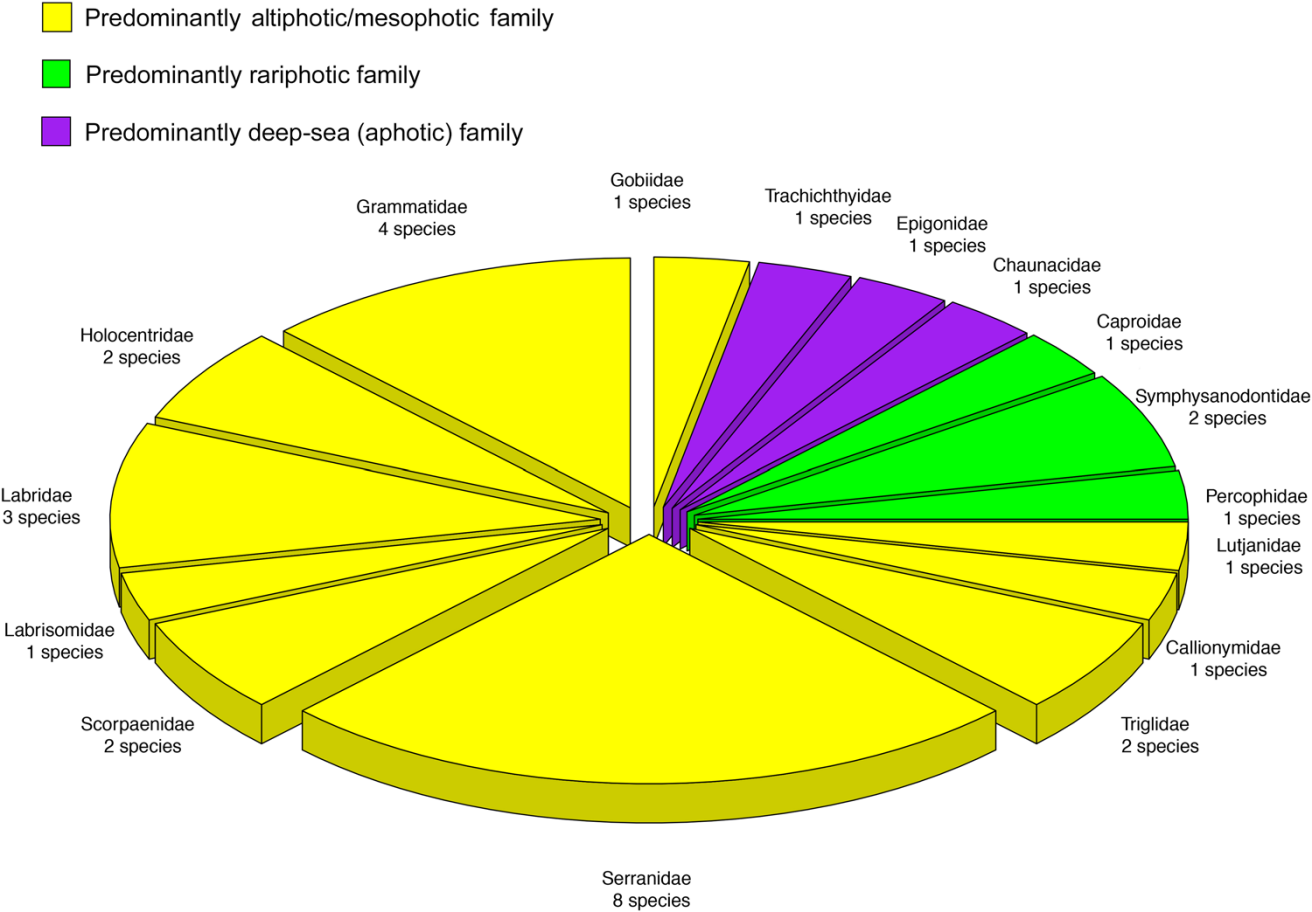
Families of rariphotic reef-fish species off Curaçao analyzed in this study and the predominant depth category to which each can be assigned. Altiphotic/mesophotic families are those for which depth ranges of members are predominantly shallower than 130 m^32–34^. Rariphotic families are those predominantly inhabiting depths > 130 to as deep as 500 m^32–34^. Deep-sea (aphotic) families are those typically inhabiting depths > 500 m^24,32–34^.

The mesophotic reef-fish fauna at Curaçao does not transition directly to one composed of deep-sea taxa. Forty-two of the 71 species of demersal Caribbean fishes in our data set have at least 25% of their depth distribution in the rariphotic—i.e., >130 m (Tables 1–3, Fig. 1). Eleven of those are mesophotic/rariphotic overlap species (Table 2), and 31 are primarily or exclusively rariphotic (Table 3). Those 31 species belong to 16 families, 10 of which (Callionymidae, Gobiidae, Grammatidae, Holocentridae, Labridae, Labrisomidae, Lutjanidae, Scorpaenidae, Serranidae, and Triglidae) primarily comprise species that inhabit altiphotic and mesophotic depths^32–34^ (Fig. 5). Three of the 16 families (Caproidae, Percophidae, and Symphysanodontidae) typically are found at rariphotic depths (~130 to as deep as 500 m or more^32–34^ – Fig. 5). Only three families (Chaunacidae, Epigonidae, and Trachichthyidae), each with a single species at Curaçao, primarily comprise species that are typical deep-sea inhabitants whose depth ranges extend far deeper than 500 m^32–34^ (Fig. 5). The Serranidae, with eight rariphotic species, the Grammatidae (4), and the Labridae (3) are the most common rariphotic families (Fig. 5).

The 31 predominantly rariphotic species at Curaçao belong to 23 genera (Table 3), only three of which—*Chaunax*, *Gephyroberyx*, and *Sphyraenops*—are primarily aphotic, deep-sea taxa^32–34^. *Lipogramma*, with four rariphotic species, *Bellator* (2), *Liopropoma* (2), *Pontinus* (2), *Serranus* (2), and *Symphyanodon* (2) are the most common rariphotic genera (Table 3).

Thus, deep-sea taxa contribute little to the rariphotic reef-fish assemblage at Curaçao, which is dominated overwhelmingly by primarily altiphotic and mesophotic families. Furthermore, at least some rariphotic fishes not only are members of predominantly altiphotic taxa but are evolutionarily derived from shallow-reef ancestors. A recent study of ours indicates that several Caribbean rariphotic goby lineages represent recent (~10–14 mya) evolutionary transitions from shallow ancestors, with subsequent species radiations by rariphotic lineages in some genera^35^. Our inspection of relationships in other recently published phylogenies suggests that similar shallow-to-deep habitat transitions likely occurred in other Caribbean fish genera that include rariphotic species, including *Lipogramma* (Grammatidae), *Haptoclinus* (Labrisomidae), and both *Decodon* and *Polylepion* (Labridae). In each case, the rariphotic species are nested deep within clades of altiphotic or mesophotic species^30,36–38^ (supplemented with our unpublished sequence data for the rariphotic genus *Haptoclinus*). Other genera of rariphotic reef fishes, such as *Pristipomoides* (and closely related species in the Lutjanidae subfamily Etelinae), and *Corniger* and *Ostichthys* (Holocentridae subfamily Myripristinae), belong to clades primarily containing rariphotic and mesophotic taxa that, collectively, are sister groups to clades of altiphotic taxa^37,39,40^. Few studies have investigated historical depth transitions between shallow- and deep-reef taxa. Most research on the general structure of tropical reef-fish faunas at the regional level has focused on the phylogeography of shallow faunas and the relationship between faunal structure and the usage of reefs vs. other habitats^41–44^. However, research attention needs to be expanded to resolving phylogenetic relationships among reef-fish assemblages inhabiting different depth zones, as that is central to understanding the evolutionary origins and current composition of local and regional reef-fish faunas inhabiting shallow as well as deep reefs.

The rariphotic reef-fish fauna described here is not restricted to Curaçao. Similarly large data sets are needed to determine the depth breaks between mesophotic and rariphotic fish faunas at other Caribbean sites, and establish the lower limit of the rariphotic zone. However, preliminary observations and collections between ~40 and 300 m off Bonaire, Dominica, Honduras, and St. Eustatius indicate that much of the Curaçao rariphotic fauna is widespread throughout the Caribbean. Not surprisingly, we and others^3,7,13,22,23^ (and see species range-maps^34^) have observed the same for the Caribbean mesophotic fish fauna. Other ocean basins also appear to accommodate a rariphotic fish fauna; for example, in the Hawaiian Archipelago and Indo-west Pacific, some species of *Chromis* (Pomacentridae)*, Parapercis* (Pinguipedidae), *Bodianus* (Labridae), *Scorpaena* (Scorpaenidae), and *Anthias, Odontanthias*, and *Sacura* (Serranidae) have the upper limits of their depth ranges between 130 and 180 m^8,32^.

### Faunal depth boundaries

**Figure 6:**
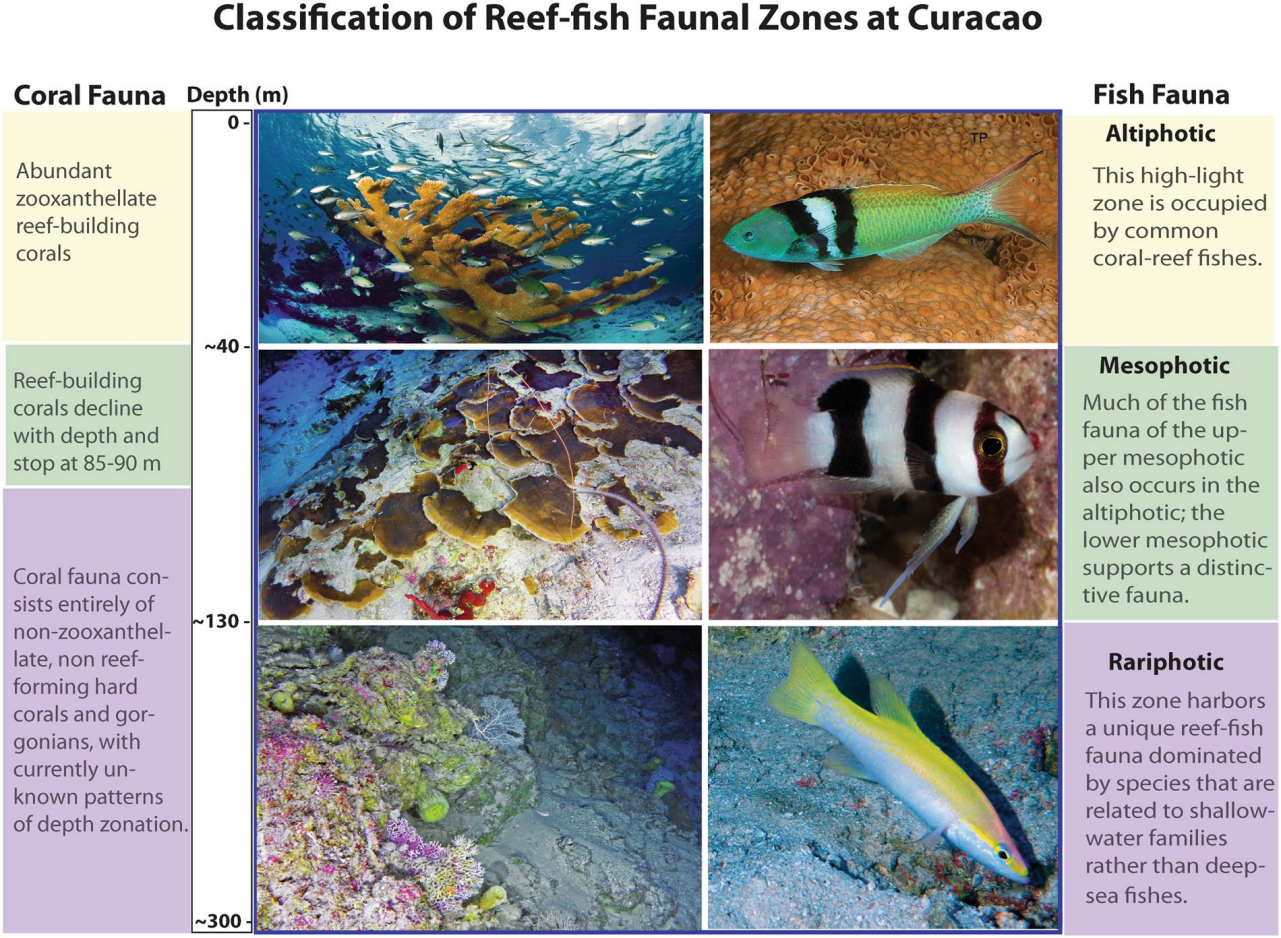
Classification of faunal zones above the aphotic based on analysis of fish assemblages at Curaçao. Representative coral and fish species are depicted for each zone. Altiphotic: (left) the zooxanthellate coral, *Acropora palmata*, and (right) *Thalassoma bifasciatum* (depth off Curaçao 0-35 m); Mesophotic: (left) a zooxanthellate plate coral, *Agaricia* sp., and (right) *Lipogramma levinsoni* (depth off Curaçao 91–154 m); Rariphotic: (left) the lace coral *Stylaster* sp. and gorgonian *Nicella* sp., and (right) *Liopropoma olneyi* (depth off Curaçao 112–229 m). Photos by Federico Cabello (upper left), Kevin Bryant (upper right), C. C. Baldwin, D. R. Robertson, and L. Tornabene.

The boundary between mesophotic and rariphotic fish communities occurs at Curaçao at ~130 m, shallower than the 150-m depth typically considered as the lower boundary of the mesophotic zone^2,3,7,9,11^. However, the lower limits of zooxanthellate coral-reef development (which has traditionally been used to define the lower boundary of the mesophotic zone) range from ~55 to 133 m^15,45,46^ in the wider Caribbean area, and the lower limits of such reef corals in our study area at Curaçao are ~85–90 m^16,47^ (Fig. 6). The latter depth range is similar to the ~80–85 m faunal break between upper and lower mesophotic reef-fish assemblages at Curaçao revealed in our analysis and elsewhere^7^. At our study site on the leeward coast of Curaçao, in addition to a steep drop-off in bottom topography beginning at 7–15 m, a second vertical drop-off begins at ~80–90 m^7,48,49^, thus dramatically reducing light levels^7^, both of which may influence faunal boundaries of corals and fishes. Beyond that drop-off to ~309 m at Curaçao, the slope consists of a series of vertical to moderately sloped cliffs and ridges interrupted by rubble and sand beaches. Faunal assemblages of benthic invertebrates have not been characterized at Curaçao below the ~85–90 m limits of zooxanthellate coral growth, but we have observed non-zooxanthellate, non-reef-building hard corals and a diversity of gorgonians below that depth zone (Fig. 6). Other than the correlation between the depths of the lower limit of zooxanthellate coral growth and the faunal break between upper and lower mesophotic fish assemblages, there are no obvious associations between fish-faunal boundaries and benthos/bottom topography at Curaçao. In the northern Gulf of Mexico, typical mesophotic reef fishes extend their depth ranges well below the shallow (~50 m) lower limit of zooxanthellate coral growth^18^, another indication of a lack of a strong association between the depth zonations of reef fishes and reef-building corals, at least in the wider Caribbean. A general ~200-m faunal changeover in the Gulf of Mexico based on the depth ranges of fishes and other taxa there may be related to environmental differences (productivity and water-current systems) between the edge of the continental shelf and slope^10^. However, while that Gulf possesses a broad continental shelf, no equivalent exists at Curaçao, where the reef drops off rapidly from near the surface to at least 300 m.

**Figure 7:**
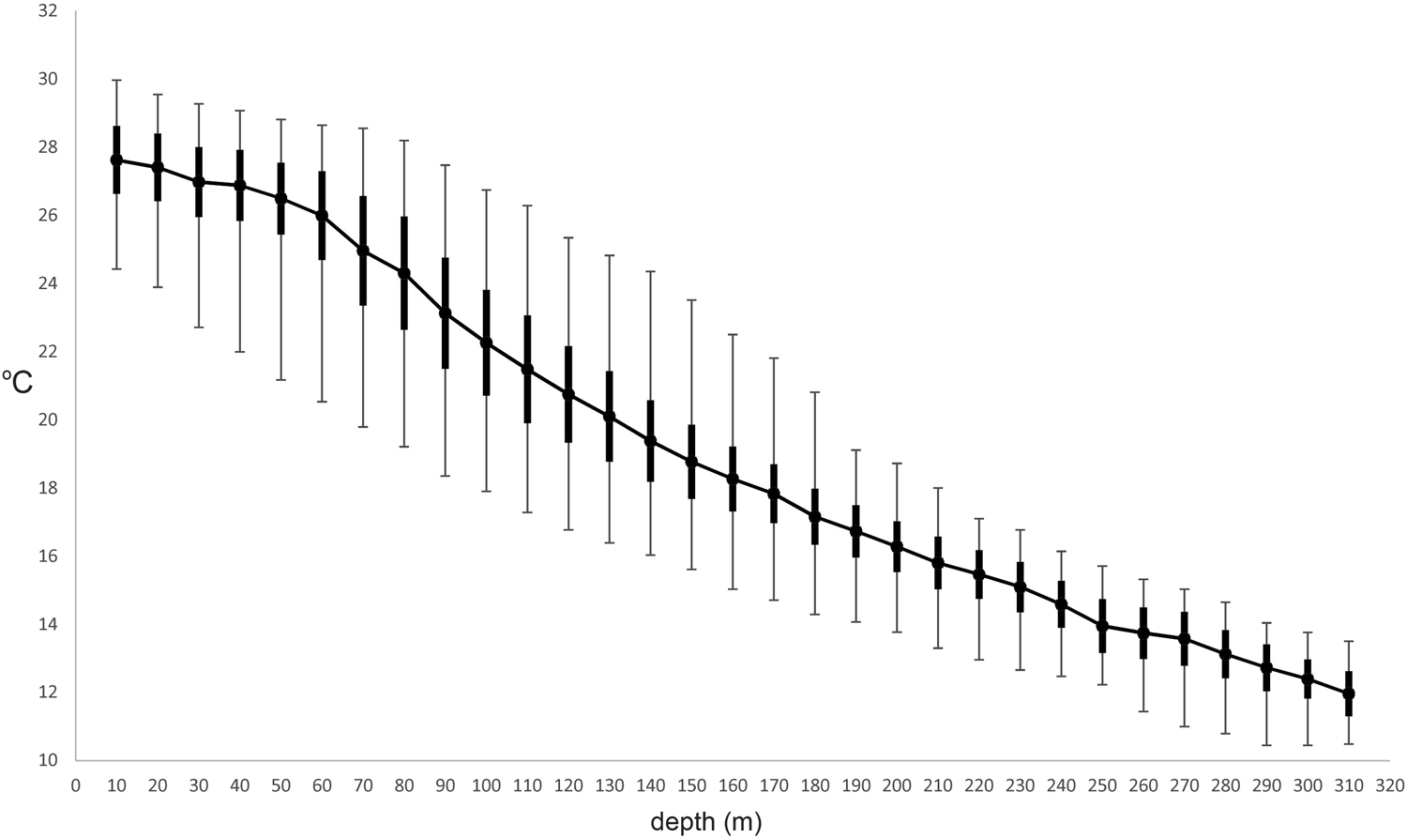
Mean (with range and +/− one standard deviation) temperatures at 10-m intervals between the surface and 309 m recorded from 319 submersible dives off Curaçao.

Other physical water-mass features such as temperature, pressure, and dissolved oxygen also may play a role in the structuring of reef communities by depth^24^. We assessed potential correlations between temperature and fish-faunal structure from temperature data recorded during 319 sub dives to depths as great as 309 m in 2011–2014 (Fig. 7). Mean temperature declines gradually to ~50 m, where, during submersible dives, we often observed the upper limit of the thermocline as a shimmering layer of water. The rate of decline in mean temperature increases between ~50 and 130 m, a zone that exhibits the greatest variability in temperatures. That variability likely is due to both day-to-day fluctuations off Curaçao^16^ and seasonal upwelling^49^. Mean temperatures continue to decline gradually to 309 m, providing the 130–309 m zone with the coldest, most stable temperatures. The top of the thermocline at ~50–60 m coincides with only a minor fish-faunal break within the upper mesophotic (Fig. 3), while the zone of most rapid decline and greatest variability in temperatures is associated with the mesophotic (~40–130 m), and the coldest and most stable temperatures with the rariphotic (~130–309 m). However, any conclusions about those relationships remain tentative in light of temperature variability. Future research efforts need to scrutinize relationships between the zonation of reef fishes and temperature patterns on shallow-to-deep reef slopes at other Caribbean locations.

Faunal depth boundaries also vary by location, as indicated by differences not only between local and global depth ranges of fish species (Tables 1–3) but also between depth ranges of zooxanthellate coral reefs in different parts of the Greater Caribbean^2,15,16,18,46,50,51^. If local depth ranges vary geographically, then using species’ local depth distributions, as done in this study, is likely to produce better estimates of faunal breaks than using species’ global depth ranges. Furthermore, studies at different locations with different environmental regimes that combine information on species’ local depth distributions with that on changes in environmental variables along depth gradients offer the opportunity to assess how such variables affect depth distributions of not only tropical reef fishes but also corals and other invertebrate reef taxa.

### New classification of tropical reef-faunal zones

A graphical representation of reef-faunal zones based on the present study at Curaçao is shown in Fig. 6. Existing classifications for benthic faunal zones along continental shores, shelves, and slopes (i.e., littoral [intertidal], sublittoral [intertidal to 200 m], archibenthal [60–1,000 m], bathyal [200–1,000 m], abyssal [4,000–6,000 m], hadal [>6,000 m]^52,53^) do not delineate tropical altiphotic, mesophotic, and rariphotic faunal zones and cannot be adapted to fully describe major depth-related changes in the Curaçao reef-fish assemblage between the surface and 309 m. Open-ocean ecosystems have yet another, different classification (i.e., epipelagic/euphotic [0–200 m], mesopelagic/disphotic [200–1,000 m], bathypelagic [1,000–4,000 m], and abyssopelagic [>4,000 m]^54^) that is not appropriate for benthic communities. Our classification, derived from analyzing fish-faunal assemblages between 40 and 309 m, accommodates a broad rariphotic zone below the mesophotic that is populated largely by deep-living representatives of shallow-water higher taxa at Curaçao and elsewhere in the Caribbean Sea. The extent to which this classification can accommodate depth changes in assemblages of zooxanthellate and other types of corals, as well as other reef taxa at Curaçao and elsewhere, remains to be investigated. Formally classifying mesophotic and rariphotic assemblages improves the ability of scientists and the public to communicate about tropical ocean biodiversity and directs attention to one of the poorly studied marine environments, in this case a highly diverse one. Recent investigations of just a few Caribbean mesophotic and rariphotic reef ecosystems have revealed so many new species^27–30,55–62^ that the true amount of biodiversity in both of those zones is likely far from adequately known. Dedicated global biodiversity exploration of such ecosystems should be a major research priority. Because altiphotic, mesophotic, and rariphotic zones form a tropical depth continuum, further study of their biological and ecological interactions is needed in light of changing global ocean conditions. As mesophotic depths may serve as refugia for altiphotic inhabitants, so may rariphotic depths for mesophotic life.

## Methods

### Site description

Observations and collections of deep-reef fishes were conducted off the coast of Curaçao near 12.083197N, 68.899058W. The study site was chosen based on accessibility to deep-reef ecosystems very close to shore, obviating the need (and associated costs) of a research support vessel for submersible operations. The general reef characteristics of Curaçao and Bonaire are very similar and have been summarized elsewhere^49^. That summary agrees well with our observations from sub dives at both Islands. Coral-cover estimates for shallow reefs (<20 m) on the leeward side of Curaçao range between 16–40%^63^. Overall, gradually sloping shallow-reef areas drop more steeply at ~7–15 m, followed by additional vertical drop-offs beginning at 70–90 m depending on location^7,16,48,64–66^. Most of the deep-reef area to ~300 m at Curaçao is characterized by steep to moderately sloped cliffs interrupted by rubble and sand beaches. Cliffs were largely formed by erosion of terraces formed during periods of low sea-level during Pleistocene^67^. Curaçao experiences less than 500 mm of annual precipitation (http://www.meteo.cw/climate.php), and, in general, water visibility is high year-round. There were no observable differences in visibility along the deep-reef slope during our study period.

### Fish observations

Observations and collections of deep-reef fishes were a major objective of ~80 dives between 2011 and 2016 made by the manned submersible *Curasub* (http://www.substation-curacao.com/) off the coast of Curaçao. Each dive typically lasted ~4 hours and many reached a depth near 310 m (the maximum to which *Curasub* is rated). Fish-sighting records and collections commenced at 40 m on each dive. Dives generally involved roving surveys, with the submersible facing the reef and moving slowly (<2 knots) laterally while simultaneously descending very slowly down the slope to a variable maximum depth. Periodic pauses were made throughout each dive for collecting specimens. Most species depth records were obtained by a pair of us seated in the front of the submersible linking our sightings of identifiable fishes to readouts from an internal digital depth gauge. These observations were supplemented with representative specimens captured using an anesthetic ejection system coupled to a suction hose that empties into a collecting chamber^55^. A total of 202 specimens was collected, with a special emphasis on species that are difficult to identify visually (e.g. cryptobenthic species), unfamiliar species likely to be new, and species for which tissue samples were needed for ongoing studies involving genetic analyses. Identifications of captured specimens were made using both morphology and DNA barcoding^67^. Fishes that could not be identified confidently visually from the sub or from investigation of captured specimens (e.g., those with problematic taxonomy) were excluded from the analysis presented here. We estimated relative sampling effort by examining the time spent at each 10-m depth band on 53 dives in 2011–2013 (Supplemental Fig. 1), and differences in sampling efforts at each depth are accounted for below under “Fish-Assemblage structure.” *Guidelines for the Use of Fishes in Research* co-established by the American Society of Ichthyologists and Herpetologists (http://www.asih.org/sites/default/files/documents/publications/asf-guidelines-use-of-fishes-in-research-2013.pdf) were followed for all field-collecting activities, and fish specimens collected as part of this study were done so under Smithsonian Animal Care and Use Committee (ACUC) approval to C. C. Baldwin (ACUC #2011–07 and #2014–13).

### Fish-Assemblage structure

The depth structure of fish assemblages was examined using the Bray-Curtis dissimilarity metric^7,24,68,69^. The 4,436 observations on 71 species were separated into 10-m bins from 40 to 309 m. The number of observations per species ranged from 1 to 559 (mean = 62.4). Sampling effort (dive time) across each 10-m depth interval was relatively uniform across most depths, but somewhat shorter at the deepest intervals. We therefore standardized species observations in each depth interval by applying a multiplier equal to the average dive time in the most heavily sampled depth interval divided by the average dive time in each respective interval. In some cases, few observations of certain species may be due to sampling bias, as small, solitary, cryptobenthic species are often overlooked in visual surveys. To avoid overemphasizing the importance of rare species in our analysis, while also controlling for extremely abundant species that sometimes occur in large schools, we applied a square-root transformation to our raw abundance data. The clustering analysis based on the Bray-Curtis metrics used the complete-linkage clustering algorithm, which seeks to maximize distance between clusters by calculating the proximity between clusters based on the two most distant objects in each cluster. Non-metric multidimensional scaling (NMDS) ordination and a hierarchical cluster dendrogram were used to visualize community structure. The number of significantly distinct depth clusters was determined using Analysis of Similarity Profiles (SIMPROF) with a conservative value of alpha = 10^−7^ (see^24,70–72^). To confirm the statistical significance of depth zones that were delineated *a posteriori* (e.g. the upper- and lower- mesophotic and rariphotic zones, which combined clusters from the SIMPROF analysis), we used PERMANOVA^73^. A similarity percentage analysis (SIMPER)^7,24,69,74^ was used to determine which species contributed most to differences between adjacent depth zones (upper mesophotic-lower mesophotic, lower mesophotic-upper rariphotic, upper rariphotic-lower rariphotic) (Table 4).

### Temperature

The *Curasub* is equipped with a Sensus Ultra dive data logger that records temperature at 10-second intervals during a sub dive to 0.01C with an accuracy of +/− 0.8 C. We analyzed temperature data from 319 dives made during three years (2011–2014) to as deep as 309 m.

### Data availability

Raw fish-depth data analyzed during this study are included in Supplementary Table 1, and raw temperature data in Supplementary Table 2. Data used to produce temperature graph in Fig. 7 are tabulated in Supplementary Table 3.

## Electronic supplementary material

Supplementary Materials
