# Supplementary material for: Below the Mesophotic
Source: Sci Rep. 2018 Mar 20;8:4920. doi: 10.1038/s41598-018-23067-1 (PMC5861108; doi:10.1038/s41598-018-23067-1)
Supplement: Supplementary file 1 — Supplementary Materials [file 41598_2018_23067_MOESM1_ESM.pdf]

SUPPLEMENTARY MATERIALS

Manuscript Number: SREP-17-37764A

Title: Below the Mesophotic

Authors: Carole C. Baldwin

Luke Tornabene

D. Ross Robertson

Supplementary Fig. 1. Relative sampling effort expressed as time spent at each 10-m depth interval on 53 dives made off Curaçao in 2011-2013.

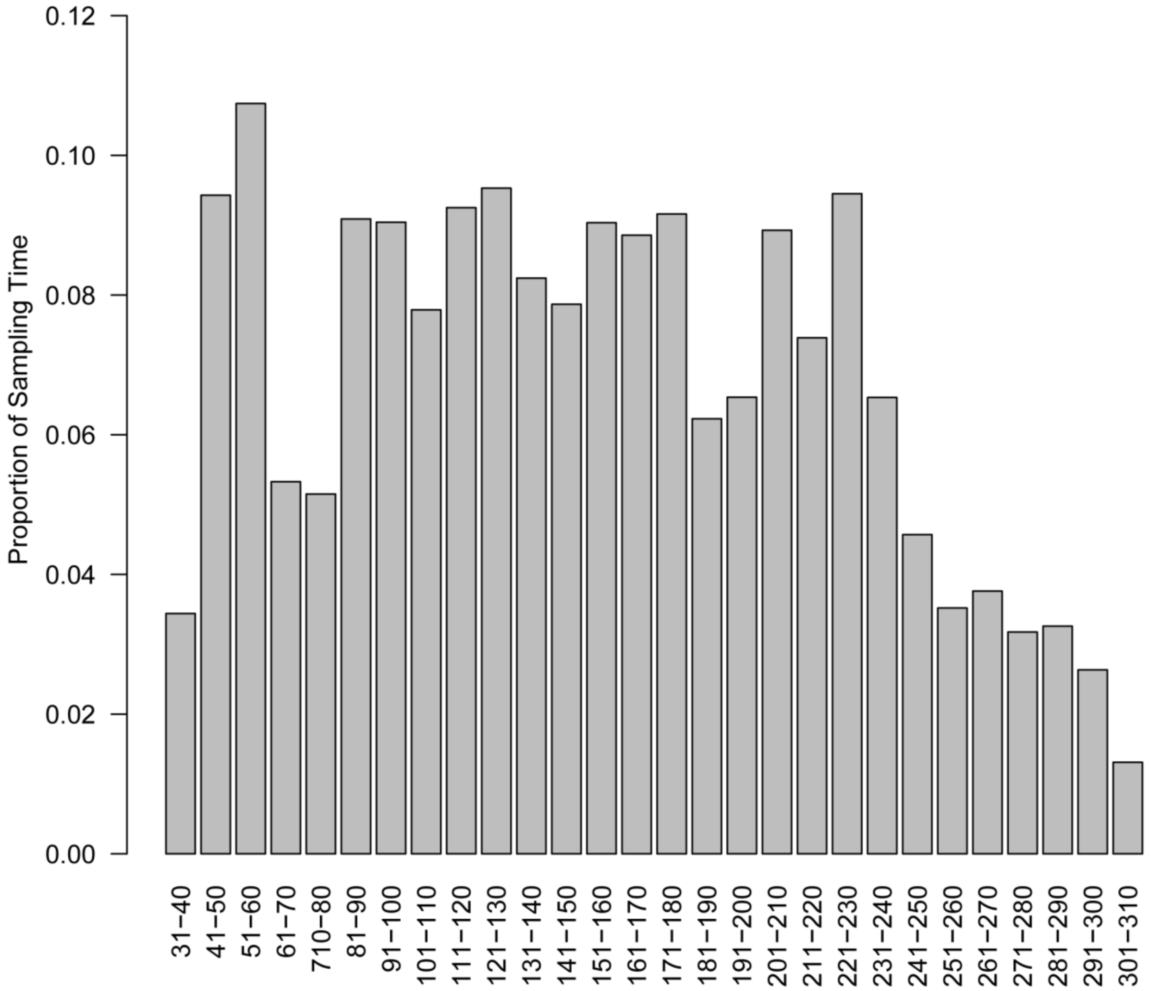

Supplementary Table 1. Raw depth data analyzed in this study.

Genus	Species	Depth (m)	Description
Liopropoma	aberrans	98	Specimen
Liopropoma	aberrans	98	visual
Liopropoma	aberrans	110	visual
Liopropoma	aberrans	113	visual
Liopropoma	aberrans	113	visual
Liopropoma	aberrans	114	visual
Liopropoma	aberrans	116	visual
Liopropoma	aberrans	116	Specimen
Liopropoma	aberrans	119	visual
Liopropoma	aberrans	120	visual
Liopropoma	aberrans	124	visual
Liopropoma	aberrans	125	visual
Liopropoma	aberrans	126	visual
Liopropoma	aberrans	128	visual
Liopropoma	aberrans	128	visual
Liopropoma	aberrans	128	visual
Liopropoma	aberrans	134	visual
Liopropoma	aberrans	137	Specimen
Liopropoma	aberrans	137	visual
Liopropoma	aberrans	137	visual
Liopropoma	aberrans	142	visual
Liopropoma	aberrans	144	visual
Liopropoma	aberrans	149	Specimen
Liopropoma	aberrans	149	visual
Liopropoma	aberrans	151	visual
Liopropoma	aberrans	152	visual
Liopropoma	aberrans	154	visual
Liopropoma	aberrans	155	visual
Liopropoma	aberrans	158	visual
Liopropoma	aberrans	158	visual
Liopropoma	aberrans	158	visual
Liopropoma	aberrans	159	visual
Liopropoma	aberrans	160	visual
Liopropoma	aberrans	170	visual
Liopropoma	aberrans	170	visual
Liopropoma	aberrans	172	visual
Liopropoma	aberrans	173	visual
Liopropoma	aberrans	241	visual
Prognathodes	aculeatus	55	visual
Prognathodes	aculeatus	57	visual
Prognathodes	aculeatus	60	visual
Prognathodes	aculeatus	61	visual
Prognathodes	aculeatus	62	visual
Prognathodes	aculeatus	62	visual

Prognathodes	aculeatus	78	visual
Prognathodes	aculeatus	78	visual
Prognathodes	aculeatus	78	visual
Prognathodes	aculeatus	79	visual
Prognathodes	aculeatus	79	visual
Prognathodes	aculeatus	80	visual
Prognathodes	aculeatus	82	visual
Prognathodes	aculeatus	84	visual
Prognathodes	aculeatus	87	visual
Prognathodes	aculeatus	88	visual
Prognathodes	aculeatus	91	visual
Prognathodes	aculeatus	91	visual
Prognathodes	aculeatus	91	visual
Prognathodes	aculeatus	91	visual
Prognathodes	aculeatus	95	visual
Prognathodes	aculeatus	98	visual
Prognathodes	aculeatus	98	visual
Prognathodes	aculeatus	99	Specimen
Prognathodes	aculeatus	103	visual
Prognathodes	aculeatus	107	visual
Prognathodes	aculeatus	120	visual
Prognathodes	aculeatus	131	visual
Prognathodes	aculeatus	136	visual
Centropyge	agri	63	visual
Centropyge	agri	63	visual
Centropyge	agri	66	visual
Centropyge	agri	67	visual
Centropyge	agri	68	visual
Centropyge	agri	68	visual
Centropyge	agri	68	visual
Centropyge	agri	68	visual
Centropyge	agri	68	visual
Centropyge	agri	68	visual
Centropyge	agri	68	visual
Centropyge	agri	68	visual
Centropyge	agri	68	visual
Centropyge	agri	68	visual
Centropyge	agri	68	visual
Centropyge	agri	68	visual
Centropyge	agri	68	visual
Centropyge	agri	68	visual
Centropyge	agri	68	visual
Centropyge	agri	68	visual
Centropyge	agri	68	visual
Centropyge	agri	68	visual
Centropyge	agri	68	visual
Centropyge	agri	68	visual

Centropyge	agri	68	visual
Centropyge	agri	68	visual
Centropyge	agri	69	visual
Centropyge	agri	69	visual
Centropyge	agri	69	visual
Centropyge	agri	69	visual
Centropyge	agri	70	visual
Centropyge	agri	70	visual
Centropyge	agri	70	visual
Centropyge	agri	70	visual
Centropyge	agri	72	visual
Centropyge	agri	76	visual
Centropyge	agri	76	visual
Centropyge	agri	79	visual
Centropyge	agri	99	visual
Pristigenys	alta	73	visual
Pristigenys	alta	98	visual
Pristigenys	alta	113	visual
Pristigenys	alta	126	visual
Pristigenys	alta	133	visual
Pristigenys	alta	139	visual
Pristigenys	alta	168	visual
Pristigenys	alta	168	visual
Pristigenys	alta	171	visual
Pristigenys	alta	181	visual
Pristigenys	alta	183	visual
Anthias	asperilinguis	96	visual
Anthias	asperilinguis	125	Specimen
Anthias	asperilinguis	125	Specimen
Anthias	asperilinguis	223	visual
Anthias	asperilinguis	226	Specimen
Anthias	asperilinguis	226	Specimen
Anthias	asperilinguis	230	visual
Anthias	asperilinguis	235	Specimen
Anthias	asperilinguis	241	Specimen
Anthias	asperilinguis	241	visual
Anthias	asperilinguis	244	visual
Anthias	asperilinguis	244	visual
Anthias	asperilinguis	244	Specimen
Anthias	asperilinguis	244	Specimen
Anthias	asperilinguis	251	visual
Anthias	asperilinguis	251	Specimen
Anthias	asperilinguis	276	Specimen
Anthias	asperilinguis	293	Specimen
Anthias	asperilinguis	297	Specimen
Symphysanodon	berryi	126	visual
Symphysanodon	berryi	159	visual

Symphysanodon	berryi	165	visual
Symphysanodon	berryi	166	Specimen
Symphysanodon	berryi	166	Specimen
Symphysanodon	berryi	168	visual
Symphysanodon	berryi	169	visual
Symphysanodon	berryi	183	visual
Symphysanodon	berryi	184	visual
Symphysanodon	berryi	189	visual
Symphysanodon	berryi	192	visual
Symphysanodon	berryi	192	visual
Symphysanodon	berryi	192	visual
Symphysanodon	berryi	193	visual
Symphysanodon	berryi	194	visual
Symphysanodon	berryi	205	visual
Symphysanodon	berryi	205	visual
Symphysanodon	berryi	205	visual
Symphysanodon	berryi	205	visual
Symphysanodon	berryi	205	visual
Symphysanodon	berryi	206	visual
Symphysanodon	berryi	207	visual
Symphysanodon	berryi	211	visual
Symphysanodon	berryi	211	visual
Symphysanodon	berryi	213	visual
Symphysanodon	berryi	213	visual
Symphysanodon	berryi	213	visual
Symphysanodon	berryi	216	visual
Symphysanodon	berryi	218	visual
Symphysanodon	berryi	218	visual
Symphysanodon	berryi	219	visual
Symphysanodon	berryi	219	visual
Symphysanodon	berryi	222	visual
Symphysanodon	berryi	226	visual
Symphysanodon	berryi	226	visual
Symphysanodon	berryi	226	visual
Symphysanodon	berryi	226	visual
Symphysanodon	berryi	226	visual
Symphysanodon	berryi	226	visual
Symphysanodon	berryi	226	visual
Symphysanodon	berryi	226	visual
Symphysanodon	berryi	226	visual
Symphysanodon	berryi	226	visual
Symphysanodon	berryi	226	visual
Symphysanodon	berryi	226	visual
Symphysanodon	berryi	227	visual
Symphysanodon	berryi	230	visual
Symphysanodon	berryi	231	visual
Symphysanodon	berryi	232	visual

Symphysanodon	berryi	232	visual
Symphysanodon	berryi	232	visual
Symphysanodon	berryi	232	visual
Symphysanodon	berryi	232	visual
Symphysanodon	berryi	233	visual
Symphysanodon	berryi	234	visual
Symphysanodon	berryi	234	visual
Symphysanodon	berryi	234	visual
Symphysanodon	berryi	234	visual
Symphysanodon	berryi	234	visual
Symphysanodon	berryi	234	visual
Symphysanodon	berryi	234	visual
Symphysanodon	berryi	234	visual
Symphysanodon	berryi	234	visual
Symphysanodon	berryi	237	visual
Symphysanodon	berryi	238	visual
Symphysanodon	berryi	238	visual
Symphysanodon	berryi	240	visual
Symphysanodon	berryi	240	visual
Symphysanodon	berryi	242	visual
Symphysanodon	berryi	247	visual
Symphysanodon	berryi	247	visual
Symphysanodon	berryi	247	visual
Symphysanodon	berryi	247	visual
Symphysanodon	berryi	247	visual
Symphysanodon	berryi	247	visual
Symphysanodon	berryi	247	visual
Symphysanodon	berryi	247	visual
Symphysanodon	berryi	247	visual
Symphysanodon	berryi	247	visual
Symphysanodon	berryi	249	visual
Symphysanodon	berryi	253	visual
Symphysanodon	berryi	253	visual
Symphysanodon	berryi	253	Specimen
Symphysanodon	berryi	259	visual
Symphysanodon	berryi	259	visual
Symphysanodon	berryi	259	visual
Symphysanodon	berryi	259	visual
Symphysanodon	berryi	259	visual
Symphysanodon	berryi	259	visual
Symphysanodon	berryi	259	visual
Symphysanodon	berryi	259	visual
Symphysanodon	berryi	259	visual
Symphysanodon	berryi	259	visual
Symphysanodon	berryi	259	visual
Symphysanodon	berryi	263	visual
Symphysanodon	berryi	265	visual

Symphysanodon	berryi	265	visual
Symphysanodon	berryi	269	visual
Symphysanodon	berryi	270	visual
Symphysanodon	berryi	274	visual
Symphysanodon	berryi	277	visual
Symphysanodon	berryi	280	visual
Symphysanodon	berryi	280	visual
Symphysanodon	berryi	293	visual
Symphysanodon	berryi	294	Specimen
Symphysanodon	berryi	294	Specimen
Symphysanodon	berryi	296	visual
Symphysanodon	berryi	296	visual
Symphysanodon	berryi	296	visual
Symphysanodon	berryi	296	visual
Symphysanodon	berryi	296	visual
Symphysanodon	berryi	296	visual
Symphysanodon	berryi	296	visual
Symphysanodon	berryi	296	visual
Symphysanodon	berryi	296	visual
Symphysanodon	berryi	296	visual
Symphysanodon	berryi	296	visual
Bellator	brachychir	192	visual
Bellator	brachychir	194	Specimen
Bellator	brachychir	198	visual
Bellator	brachychir	198	visual
Bellator	brachychir	198	visual
Bellator	brachychir	229	visual
Bellator	brachychir	233	visual
Bellator	brachychir	242	visual
Bellator	brachychir	243	visual
Bellator	brachychir	245	Specimen
Chaetodon	capistratus	47	visual
Chaetodon	capistratus	47	visual
Chaetodon	capistratus	47	visual
Chaetodon	capistratus	49	visual
Chaetodon	capistratus	50	visual
Chaetodon	capistratus	50	visual
Chaetodon	capistratus	53	visual
Chaetodon	capistratus	55	visual
Chaetodon	capistratus	59	visual
Chaetodon	capistratus	72	visual
Chaetodon	capistratus	72	visual
Chaetodon	capistratus	79	visual
Chaetodon	capistratus	79	visual
Chaetodon	capistratus	82	visual
Chaetodon	capistratus	82	visual
Chaetodon	capistratus	88	visual

Chaetodon	capistratus	101	visual
Antigonia	capros	159	visual
Antigonia	capros	165	visual
Antigonia	capros	165	visual
Antigonia	capros	168	visual
Antigonia	capros	168	visual
Antigonia	capros	174	visual
Antigonia	capros	174	Specimen
Antigonia	capros	178	visual
Antigonia	capros	178	visual
Antigonia	capros	180	visual
Antigonia	capros	183	visual
Antigonia	capros	183	visual
Antigonia	capros	183	visual
Antigonia	capros	183	visual
Antigonia	capros	184	visual
Antigonia	capros	184	visual
Antigonia	capros	185	visual
Antigonia	capros	186	visual
Antigonia	capros	187	visual
Antigonia	capros	187	visual
Antigonia	capros	189	visual
Antigonia	capros	193	visual
Antigonia	capros	196	visual
Antigonia	capros	201	visual
Antigonia	capros	204	visual
Antigonia	capros	204	visual
Antigonia	capros	204	visual
Antigonia	capros	204	Specimen
Antigonia	capros	205	visual
Antigonia	capros	206	visual
Antigonia	capros	206	visual
Antigonia	capros	207	visual
Antigonia	capros	210	visual
Antigonia	capros	215	Specimen
Antigonia	capros	215	visual
Antigonia	capros	215	visual
Antigonia	capros	216	visual
Antigonia	capros	216	visual
Antigonia	capros	218	visual
Antigonia	capros	219	visual
Antigonia	capros	219	visual
Antigonia	capros	219	visual
Antigonia	capros	219	visual
Antigonia	capros	221	visual
Antigonia	capros	221	visual
Antigonia	capros	222	visual

Antigonia	capros	223	visual
Antigonia	capros	223	visual
Antigonia	capros	223	visual
Antigonia	capros	224	visual
Antigonia	capros	224	visual
Antigonia	capros	224	visual
Antigonia	capros	224	visual
Antigonia	capros	224	Specimen
Antigonia	capros	224	visual
Antigonia	capros	225	visual
Antigonia	capros	227	visual
Antigonia	capros	227	visual
Antigonia	capros	228	visual
Antigonia	capros	230	visual
Antigonia	capros	231	visual
Antigonia	capros	232	visual
Antigonia	capros	233	visual
Antigonia	capros	234	visual
Antigonia	capros	236	visual
Antigonia	capros	236	visual
Antigonia	capros	237	visual
Antigonia	capros	237	visual
Antigonia	capros	246	visual
Antigonia	capros	247	visual
Antigonia	capros	250	visual
Antigonia	capros	251	visual
Antigonia	capros	251	visual
Antigonia	capros	277	visual
Antigonia	capros	279	visual
Antigonia	capros	286	visual
Antigonia	capros	287	visual
Antigonia	capros	299	visual
Bullisichthys	caribbaeus	81	visual
Bullisichthys	caribbaeus	81	visual
Bullisichthys	caribbaeus	81	visual
Bullisichthys	caribbaeus	81	visual
Bullisichthys	caribbaeus	81	visual
Bullisichthys	caribbaeus	81	visual
Bullisichthys	caribbaeus	81	visual
Bullisichthys	caribbaeus	81	visual
Bullisichthys	caribbaeus	81	visual
Bullisichthys	caribbaeus	81	visual
Bullisichthys	caribbaeus	84	visual
Bullisichthys	caribbaeus	84	visual
Bullisichthys	caribbaeus	84	visual
Bullisichthys	caribbaeus	84	visual
Bullisichthys	caribbaeus	84	visual

Bullisichthys	caribbaeus	84	visual
Bullisichthys	caribbaeus	84	visual
Bullisichthys	caribbaeus	84	visual
Bullisichthys	caribbaeus	84	visual
Bullisichthys	caribbaeus	84	visual
Bullisichthys	caribbaeus	84	visual
Bullisichthys	caribbaeus	84	visual
Bullisichthys	caribbaeus	84	visual
Bullisichthys	caribbaeus	84	visual
Bullisichthys	caribbaeus	84	visual
Bullisichthys	caribbaeus	84	visual
Bullisichthys	caribbaeus	84	visual
Bullisichthys	caribbaeus	84	visual
Bullisichthys	caribbaeus	84	visual
Bullisichthys	caribbaeus	84	visual
Bullisichthys	caribbaeus	92	visual
Bullisichthys	caribbaeus	92	visual
Bullisichthys	caribbaeus	92	visual
Bullisichthys	caribbaeus	92	visual
Bullisichthys	caribbaeus	92	visual
Bullisichthys	caribbaeus	92	visual
Bullisichthys	caribbaeus	94	Specimen
Bullisichthys	caribbaeus	94	Specimen
Bullisichthys	caribbaeus	96	visual
Bullisichthys	caribbaeus	96	visual
Bullisichthys	caribbaeus	96	visual
Bullisichthys	caribbaeus	96	visual
Bullisichthys	caribbaeus	96	visual
Bullisichthys	caribbaeus	96	visual
Bullisichthys	caribbaeus	96	visual
Bullisichthys	caribbaeus	96	visual
Bullisichthys	caribbaeus	96	visual
Bullisichthys	caribbaeus	96	visual
Bullisichthys	caribbaeus	96	visual
Bullisichthys	caribbaeus	96	visual
Bullisichthys	caribbaeus	96	visual
Bullisichthys	caribbaeus	96	visual
Bullisichthys	caribbaeus	96	visual
Bullisichthys	caribbaeus	96	visual
Bullisichthys	caribbaeus	96	visual
Bullisichthys	caribbaeus	96	visual
Bullisichthys	caribbaeus	96	visual
Bullisichthys	caribbaeus	97	visual
Bullisichthys	caribbaeus	97	visual
Bullisichthys	caribbaeus	97	visual
Bullisichthys	caribbaeus	97	visual
Bullisichthys	caribbaeus	97	visual

Bullisichthys	caribbaeus	99	visual
Bullisichthys	caribbaeus	101	visual
Bullisichthys	caribbaeus	101	visual
Bullisichthys	caribbaeus	101	visual
Bullisichthys	caribbaeus	101	visual
Bullisichthys	caribbaeus	101	visual
Bullisichthys	caribbaeus	101	visual
Bullisichthys	caribbaeus	101	visual
Bullisichthys	caribbaeus	101	visual
Bullisichthys	caribbaeus	101	visual
Bullisichthys	caribbaeus	101	visual
Bullisichthys	caribbaeus	101	visual
Bullisichthys	caribbaeus	101	visual
Bullisichthys	caribbaeus	101	visual
Bullisichthys	caribbaeus	101	visual
Bullisichthys	caribbaeus	101	visual
Bullisichthys	caribbaeus	101	visual
Bullisichthys	caribbaeus	101	visual
Bullisichthys	caribbaeus	101	visual
Bullisichthys	caribbaeus	101	visual
Bullisichthys	caribbaeus	101	visual
Bullisichthys	caribbaeus	101	visual
Bullisichthys	caribbaeus	102	visual
Bullisichthys	caribbaeus	102	Specimen
Bullisichthys	caribbaeus	103	visual
Bullisichthys	caribbaeus	103	visual
Bullisichthys	caribbaeus	103	visual
Bullisichthys	caribbaeus	103	visual
Bullisichthys	caribbaeus	103	visual
Bullisichthys	caribbaeus	103	visual
Bullisichthys	caribbaeus	103	visual
Bullisichthys	caribbaeus	103	visual
Bullisichthys	caribbaeus	103	visual
Bullisichthys	caribbaeus	103	visual
Bullisichthys	caribbaeus	103	visual
Bullisichthys	caribbaeus	103	visual
Bullisichthys	caribbaeus	103	visual
Bullisichthys	caribbaeus	103	visual
Bullisichthys	caribbaeus	103	visual
Bullisichthys	caribbaeus	103	visual
Bullisichthys	caribbaeus	103	visual
Bullisichthys	caribbaeus	103	visual
Bullisichthys	caribbaeus	103	visual
Bullisichthys	caribbaeus	103	visual
Bullisichthys	caribbaeus	104	visual
Bullisichthys	caribbaeus	104	visual
Bullisichthys	caribbaeus	107	visual

Bullisichthys	caribbaeus	107	visual
Bullisichthys	caribbaeus	107	visual
Bullisichthys	caribbaeus	107	visual
Bullisichthys	caribbaeus	107	visual
Bullisichthys	caribbaeus	107	visual
Bullisichthys	caribbaeus	107	visual
Bullisichthys	caribbaeus	107	visual
Bullisichthys	caribbaeus	107	visual
Bullisichthys	caribbaeus	107	visual
Bullisichthys	caribbaeus	107	visual
Bullisichthys	caribbaeus	107	visual
Bullisichthys	caribbaeus	109	visual
Bullisichthys	caribbaeus	109	visual
Bullisichthys	caribbaeus	110	visual
Bullisichthys	caribbaeus	110	visual
Bullisichthys	caribbaeus	110	visual
Bullisichthys	caribbaeus	110	visual
Bullisichthys	caribbaeus	110	visual
Bullisichthys	caribbaeus	110	visual
Bullisichthys	caribbaeus	110	visual
Bullisichthys	caribbaeus	110	visual
Bullisichthys	caribbaeus	110	visual
Bullisichthys	caribbaeus	110	visual
Bullisichthys	caribbaeus	110	visual
Bullisichthys	caribbaeus	110	visual
Bullisichthys	caribbaeus	110	visual
Bullisichthys	caribbaeus	110	visual
Bullisichthys	caribbaeus	111	visual
Bullisichthys	caribbaeus	111	visual
Bullisichthys	caribbaeus	111	visual
Bullisichthys	caribbaeus	113	visual
Bullisichthys	caribbaeus	113	visual
Bullisichthys	caribbaeus	113	visual
Bullisichthys	caribbaeus	113	visual
Bullisichthys	caribbaeus	113	visual
Bullisichthys	caribbaeus	113	visual
Bullisichthys	caribbaeus	113	visual
Bullisichthys	caribbaeus	113	visual
Bullisichthys	caribbaeus	113	visual
Bullisichthys	caribbaeus	113	visual
Bullisichthys	caribbaeus	113	visual
Bullisichthys	caribbaeus	113	visual
Bullisichthys	caribbaeus	113	visual
Bullisichthys	caribbaeus	113	visual
Bullisichthys	caribbaeus	113	visual
Bullisichthys	caribbaeus	113	visual
Bullisichthys	caribbaeus	113	visual

Bullisichthys	caribbaeus	113	visual
Bullisichthys	caribbaeus	113	visual
Bullisichthys	caribbaeus	113	visual
Bullisichthys	caribbaeus	113	visual
Bullisichthys	caribbaeus	113	visual
Bullisichthys	caribbaeus	113	visual
Bullisichthys	caribbaeus	113	visual
Bullisichthys	caribbaeus	113	visual
Bullisichthys	caribbaeus	113	visual
Bullisichthys	caribbaeus	113	visual
Bullisichthys	caribbaeus	113	visual
Bullisichthys	caribbaeus	113	visual
Bullisichthys	caribbaeus	113	visual
Bullisichthys	caribbaeus	113	visual
Bullisichthys	caribbaeus	113	visual
Bullisichthys	caribbaeus	113	visual
Bullisichthys	caribbaeus	113	visual
Bullisichthys	caribbaeus	113	visual
Bullisichthys	caribbaeus	113	visual
Bullisichthys	caribbaeus	113	visual
Bullisichthys	caribbaeus	113	visual
Bullisichthys	caribbaeus	113	visual
Bullisichthys	caribbaeus	113	visual
Bullisichthys	caribbaeus	113	visual
Bullisichthys	caribbaeus	113	visual
Bullisichthys	caribbaeus	113	visual
Bullisichthys	caribbaeus	113	visual
Bullisichthys	caribbaeus	113	visual
Bullisichthys	caribbaeus	113	visual
Bullisichthys	caribbaeus	113	visual
Bullisichthys	caribbaeus	113	visual
Bullisichthys	caribbaeus	114	visual
Bullisichthys	caribbaeus	114	visual
Bullisichthys	caribbaeus	114	visual
Bullisichthys	caribbaeus	114	visual
Bullisichthys	caribbaeus	114	visual
Bullisichthys	caribbaeus	114	visual
Bullisichthys	caribbaeus	114	visual
Bullisichthys	caribbaeus	114	visual
Bullisichthys	caribbaeus	114	visual
Bullisichthys	caribbaeus	114	visual
Bullisichthys	caribbaeus	114	visual
Bullisichthys	caribbaeus	114	visual
Bullisichthys	caribbaeus	114	visual
Bullisichthys	caribbaeus	114	visual
Bullisichthys	caribbaeus	114	visual
Bullisichthys	caribbaeus	114	visual

Bullisichthys	caribbaeus	114	visual
Bullisichthys	caribbaeus	114	visual
Bullisichthys	caribbaeus	114	visual
Bullisichthys	caribbaeus	114	visual
Bullisichthys	caribbaeus	114	visual
Bullisichthys	caribbaeus	115	visual
Bullisichthys	caribbaeus	115	visual
Bullisichthys	caribbaeus	115	visual
Bullisichthys	caribbaeus	115	visual
Bullisichthys	caribbaeus	115	visual
Bullisichthys	caribbaeus	115	visual
Bullisichthys	caribbaeus	115	visual
Bullisichthys	caribbaeus	115	visual
Bullisichthys	caribbaeus	115	visual
Bullisichthys	caribbaeus	115	visual
Bullisichthys	caribbaeus	115	visual
Bullisichthys	caribbaeus	115	visual
Bullisichthys	caribbaeus	117	visual
Bullisichthys	caribbaeus	117	visual
Bullisichthys	caribbaeus	117	visual
Bullisichthys	caribbaeus	117	visual
Bullisichthys	caribbaeus	117	visual
Bullisichthys	caribbaeus	117	visual
Bullisichthys	caribbaeus	117	visual
Bullisichthys	caribbaeus	117	visual
Bullisichthys	caribbaeus	122	visual
Bullisichthys	caribbaeus	123	visual
Bullisichthys	caribbaeus	123	visual
Bullisichthys	caribbaeus	125	visual
Bullisichthys	caribbaeus	131	visual
Bullisichthys	caribbaeus	131	visual
Bullisichthys	caribbaeus	131	visual
Bullisichthys	caribbaeus	131	visual
Bullisichthys	caribbaeus	131	visual
Bullisichthys	caribbaeus	131	visual
Bullisichthys	caribbaeus	131	visual
Bullisichthys	caribbaeus	131	visual
Bullisichthys	caribbaeus	131	visual
Bullisichthys	caribbaeus	131	visual
Bullisichthys	caribbaeus	131	visual
Bullisichthys	caribbaeus	131	visual
Bullisichthys	caribbaeus	131	visual
Bullisichthys	caribbaeus	131	visual
Bullisichthys	caribbaeus	131	visual
Bullisichthys	caribbaeus	131	visual
Bullisichthys	caribbaeus	131	visual
Bullisichthys	caribbaeus	131	visual

Bullisichthys	caribbaeus	131	visual
Bullisichthys	caribbaeus	131	visual
Bullisichthys	caribbaeus	131	visual
Bullisichthys	caribbaeus	131	visual
Bullisichthys	caribbaeus	131	visual
Bullisichthys	caribbaeus	131	visual
Bullisichthys	caribbaeus	131	visual
Bullisichthys	caribbaeus	131	visual
Bullisichthys	caribbaeus	131	visual
Bullisichthys	caribbaeus	131	visual
Bullisichthys	caribbaeus	133	visual
Bullisichthys	caribbaeus	134	visual
Pontinus	castor	125	Specimen
Pontinus	castor	128	visual
Pontinus	castor	130	visual
Pontinus	castor	155	visual
Pontinus	castor	155	visual
Pontinus	castor	155	visual
Pontinus	castor	156	visual
Pontinus	castor	157	visual
Pontinus	castor	163	visual
Pontinus	castor	166	visual
Pontinus	castor	184	visual
Pontinus	castor	189	Specimen
Pontinus	castor	194	visual
Pontinus	castor	198	Specimen
Pontinus	castor	204	visual
Pontinus	castor	208	visual
Pontinus	castor	211	Specimen
Pontinus	castor	215	visual
Pontinus	castor	243	visual
Cephalopholis	cruentata	40	visual
Cephalopholis	cruentata	46	visual
Cephalopholis	cruentata	58	visual
Cephalopholis	cruentata	62	visual
Cephalopholis	cruentata	66	visual
Cephalopholis	cruentata	67	visual
Cephalopholis	cruentata	67	visual
Cephalopholis	cruentata	70	visual
Cephalopholis	cruentata	70	visual
Cephalopholis	cruentata	72	visual
Cephalopholis	cruentata	79	visual
Cephalopholis	cruentata	82	visual
Cephalopholis	cruentata	84	visual
Cephalopholis	cruentata	92	visual
Cephalopholis	cruentata	95	visual
Cephalopholis	cruentata	96	visual

Cephalopholis	cruentata	98	visual
Cephalopholis	cruentata	99	visual
Cephalopholis	cruentata	100	visual
Cephalopholis	cruentata	100	visual
Cephalopholis	cruentata	101	visual
Cephalopholis	cruentata	101	visual
Cephalopholis	cruentata	103	visual
Cephalopholis	cruentata	104	visual
Cephalopholis	cruentata	105	visual
Cephalopholis	cruentata	108	visual
Cephalopholis	cruentata	109	visual
Cephalopholis	cruentata	110	visual
Cephalopholis	cruentata	114	visual
Cephalopholis	cruentata	117	visual
Cephalopholis	cruentata	127	visual
Cephalopholis	cruentata	135	visual
Chromis	cyanea	40	visual
Chromis	cyanea	47	visual
Chromis	cyanea	49	visual
Chromis	cyanea	49	visual
Chromis	cyanea	49	visual
Chromis	cyanea	53	visual
Chromis	cyanea	53	visual
Chromis	cyanea	55	visual
Chromis	cyanea	58	visual
Chromis	cyanea	58	visual
Chromis	cyanea	58	visual
Chromis	cyanea	58	visual
Chromis	cyanea	58	visual
Chromis	cyanea	58	visual
Chromis	cyanea	58	visual
Chromis	cyanea	58	visual
Chromis	cyanea	58	visual
Chromis	cyanea	58	visual
Chromis	cyanea	58	visual
Chromis	cyanea	58	visual
Chromis	cyanea	58	visual
Chromis	cyanea	58	visual
Chromis	cyanea	58	visual
Chromis	cyanea	58	visual
Chromis	cyanea	58	visual
Chromis	cyanea	58	visual
Chromis	cyanea	58	visual
Chromis	cyanea	58	visual
Chromis	cyanea	58	visual
Chromis	cyanea	58	visual
Chromis	cyanea	58	visual

Chromis	cyanea	59	visual
Chromis	cyanea	61	visual
Chromis	cyanea	61	visual
Chromis	cyanea	61	visual
Chromis	cyanea	61	visual
Chromis	cyanea	61	visual
Chromis	cyanea	64	visual
Chromis	cyanea	69	visual
Chromis	cyanea	70	visual
Chromis	cyanea	72	visual
Chromis	cyanea	76	visual
Gephyroberyx	darwinii	167	visual
Gephyroberyx	darwinii	230	visual
Gephyroberyx	darwinii	230	Specimen
Gephyroberyx	darwinii	258	Specimen
Gephyroberyx	darwinii	268	Specimen
Gephyroberyx	darwinii	268	Specimen
Gephyroberyx	darwinii	273	visual
Gephyroberyx	darwinii	275	visual
Gephyroberyx	darwinii	276	Specimen
Gephyroberyx	darwinii	276	Specimen
Gephyroberyx	darwinii	276	Specimen
Gephyroberyx	darwinii	284	visual
Chromis	enchrysura	73	visual
Chromis	enchrysura	74	visual
Chromis	enchrysura	79	visual
Chromis	enchrysura	83	visual
Chromis	enchrysura	84	visual
Chromis	enchrysura	88	visual
Chromis	enchrysura	88	visual
Chromis	enchrysura	91	visual
Chromis	enchrysura	91	visual
Chromis	enchrysura	91	visual
Chromis	enchrysura	94	visual
Chromis	enchrysura	94	visual
Chromis	enchrysura	96	visual
Chromis	enchrysura	96	visual
Chromis	enchrysura	98	visual
Chromis	enchrysura	99	visual
Chromis	enchrysura	100	visual
Chromis	enchrysura	100	visual
Chromis	enchrysura	101	visual
Chromis	enchrysura	101	visual
Chromis	enchrysura	101	visual
Chromis	enchrysura	101	visual
Chromis	enchrysura	102	visual
Chromis	enchrysura	104	visual

Chromis	enchrysura	105	visual
Chromis	enchrysura	105	visual
Chromis	enchrysura	106	visual
Chromis	enchrysura	106	visual
Chromis	enchrysura	106	visual
Chromis	enchrysura	106	visual
Chromis	enchrysura	106	visual
Chromis	enchrysura	106	visual
Chromis	enchrysura	106	visual
Chromis	enchrysura	106	visual
Chromis	enchrysura	106	visual
Chromis	enchrysura	106	visual
Chromis	enchrysura	106	visual
Chromis	enchrysura	107	visual
Chromis	enchrysura	107	visual
Chromis	enchrysura	107	visual
Chromis	enchrysura	107	visual
Chromis	enchrysura	109	visual
Chromis	enchrysura	110	visual
Chromis	enchrysura	112	visual
Chromis	enchrysura	113	visual
Chromis	enchrysura	113	visual
Chromis	enchrysura	114	visual
Chromis	enchrysura	114	visual
Chromis	enchrysura	114	visual
Chromis	enchrysura	114	visual
Chromis	enchrysura	114	visual
Chromis	enchrysura	114	visual
Chromis	enchrysura	114	visual
Chromis	enchrysura	116	visual
Chromis	enchrysura	116	visual
Chromis	enchrysura	116	visual
Chromis	enchrysura	117	visual
Chromis	enchrysura	117	visual
Chromis	enchrysura	118	visual
Chromis	enchrysura	118	visual
Chromis	enchrysura	122	visual
Chromis	enchrysura	123	visual
Chromis	enchrysura	125	visual
Chromis	enchrysura	129	visual
Chromis	enchrysura	132	visual
Chromis	enchrysura	132	visual
Chromis	enchrysura	136	visual
Chromis	enchrysura	142	visual
Lipogramma	evides	137	visual
Lipogramma	evides	137	visual
Lipogramma	evides	152	Specimen

Lipogramma	evides	152	Specimen
Lipogramma	evides	152	Specimen
Lipogramma	evides	152	Specimen
Lipogramma	evides	157	visual
Lipogramma	evides	162	Specimen
Lipogramma	evides	162	Specimen
Lipogramma	evides	164	visual
Lipogramma	evides	166	visual
Lipogramma	evides	169	visual
Lipogramma	evides	171	Specimen
Lipogramma	evides	172	visual
Lipogramma	evides	172	visual
Lipogramma	evides	172	Specimen
Lipogramma	evides	172	Specimen
Lipogramma	evides	174	Specimen
Lipogramma	evides	175	visual
Lipogramma	evides	177	Specimen
Lipogramma	evides	177	Specimen
Lipogramma	evides	177	visual
Lipogramma	evides	179	Specimen
Lipogramma	evides	180	visual
Lipogramma	evides	181	visual
Lipogramma	evides	183	visual
Lipogramma	evides	187	visual
Lipogramma	evides	187	Specimen
Lipogramma	evides	187	visual
Lipogramma	evides	187	Specimen
Lipogramma	evides	187	Specimen
Lipogramma	evides	188	visual
Lipogramma	evides	191	visual
Lipogramma	evides	192	visual
Lipogramma	evides	192	visual
Lipogramma	evides	192	visual
Lipogramma	evides	195	visual
Lipogramma	evides	195	visual
Lipogramma	evides	197	visual
Lipogramma	evides	198	visual
Lipogramma	evides	199	visual
Lipogramma	evides	201	visual
Lipogramma	evides	202	visual
Lipogramma	evides	207	visual
Lipogramma	evides	212	visual
Lipogramma	evides	212	visual
Lipogramma	evides	240	visual
Lipogramma	evides	265	visual
Paranthias	furcifer	52	visual
Paranthias	furcifer	52	visual

Paranthias	furcifer	52	visual
Paranthias	furcifer	53	visual
Paranthias	furcifer	56	visual
Paranthias	furcifer	58	visual
Paranthias	furcifer	61	visual
Paranthias	furcifer	61	visual
Paranthias	furcifer	61	visual
Paranthias	furcifer	61	visual
Paranthias	furcifer	61	visual
Paranthias	furcifer	61	visual
Paranthias	furcifer	61	visual
Paranthias	furcifer	61	visual
Paranthias	furcifer	61	visual
Paranthias	furcifer	61	visual
Paranthias	furcifer	61	visual
Paranthias	furcifer	61	visual
Paranthias	furcifer	61	visual
Paranthias	furcifer	61	visual
Paranthias	furcifer	61	visual
Paranthias	furcifer	61	visual
Paranthias	furcifer	61	visual
Paranthias	furcifer	61	visual
Paranthias	furcifer	61	visual
Paranthias	furcifer	61	visual
Paranthias	furcifer	61	visual
Paranthias	furcifer	61	visual
Paranthias	furcifer	61	visual
Paranthias	furcifer	62	visual
Paranthias	furcifer	62	visual
Paranthias	furcifer	62	visual
Paranthias	furcifer	62	visual
Paranthias	furcifer	62	visual
Paranthias	furcifer	62	visual
Paranthias	furcifer	62	visual
Paranthias	furcifer	62	visual
Paranthias	furcifer	62	visual
Paranthias	furcifer	62	visual
Paranthias	furcifer	62	visual
Paranthias	furcifer	62	visual
Paranthias	furcifer	62	visual
Paranthias	furcifer	62	visual
Paranthias	furcifer	62	visual
Paranthias	furcifer	62	visual
Paranthias	furcifer	62	visual
Paranthias	furcifer	62	visual
Paranthias	furcifer	62	visual
Paranthias	furcifer	62	visual

Paranthias	furcifer	62	visual
Paranthias	furcifer	62	visual
Paranthias	furcifer	62	visual
Paranthias	furcifer	62	visual
Paranthias	furcifer	62	visual
Paranthias	furcifer	62	visual
Paranthias	furcifer	62	visual
Paranthias	furcifer	62	visual
Paranthias	furcifer	62	visual
Paranthias	furcifer	62	visual
Paranthias	furcifer	62	visual
Paranthias	furcifer	62	visual
Paranthias	furcifer	62	visual
Paranthias	furcifer	62	visual
Paranthias	furcifer	62	visual
Paranthias	furcifer	62	visual
Paranthias	furcifer	62	visual
Paranthias	furcifer	62	visual
Paranthias	furcifer	62	visual
Paranthias	furcifer	62	visual
Paranthias	furcifer	62	visual
Paranthias	furcifer	62	visual
Paranthias	furcifer	62	visual
Paranthias	furcifer	62	visual
Paranthias	furcifer	62	visual
Paranthias	furcifer	62	visual
Paranthias	furcifer	62	visual
Paranthias	furcifer	62	visual
Paranthias	furcifer	62	visual
Paranthias	furcifer	62	visual
Paranthias	furcifer	62	visual
Paranthias	furcifer	62	visual
Paranthias	furcifer	62	visual
Paranthias	furcifer	62	visual
Paranthias	furcifer	62	visual
Paranthias	furcifer	62	visual
Paranthias	furcifer	62	visual
Paranthias	furcifer	62	visual
Paranthias	furcifer	62	visual
Paranthias	furcifer	62	visual
Paranthias	furcifer	62	visual
Paranthias	furcifer	62	visual
Paranthias	furcifer	62	visual
Paranthias	furcifer	62	visual
Paranthias	furcifer	62	visual
Paranthias	furcifer	62	visual
Paranthias	furcifer	62	visual

Paranthias	furcifer	62	visual
Paranthias	furcifer	62	visual
Paranthias	furcifer	62	visual
Paranthias	furcifer	62	visual
Paranthias	furcifer	62	visual
Paranthias	furcifer	62	visual
Paranthias	furcifer	62	visual
Paranthias	furcifer	62	visual
Paranthias	furcifer	62	visual
Paranthias	furcifer	62	visual
Paranthias	furcifer	62	visual
Paranthias	furcifer	62	visual
Paranthias	furcifer	62	visual
Paranthias	furcifer	62	visual
Paranthias	furcifer	62	visual
Paranthias	furcifer	62	visual
Paranthias	furcifer	62	visual
Paranthias	furcifer	62	visual
Paranthias	furcifer	62	visual
Paranthias	furcifer	62	visual
Paranthias	furcifer	62	visual
Paranthias	furcifer	62	visual
Paranthias	furcifer	62	visual
Paranthias	furcifer	62	visual
Paranthias	furcifer	62	visual
Paranthias	furcifer	62	visual
Paranthias	furcifer	62	visual
Paranthias	furcifer	62	visual
Paranthias	furcifer	62	visual
Paranthias	furcifer	62	visual
Paranthias	furcifer	62	visual
Paranthias	furcifer	62	visual
Paranthias	furcifer	62	visual
Paranthias	furcifer	62	visual
Paranthias	furcifer	64	visual
Paranthias	furcifer	64	visual
Paranthias	furcifer	64	visual
Paranthias	furcifer	64	visual
Paranthias	furcifer	64	visual
Paranthias	furcifer	66	visual
Paranthias	furcifer	66	visual
Paranthias	furcifer	66	visual
Paranthias	furcifer	66	visual
Paranthias	furcifer	66	visual
Paranthias	furcifer	66	visual
Paranthias	furcifer	66	visual
Paranthias	furcifer	66	visual

Paranthias	furcifer	66	visual
Paranthias	furcifer	66	visual
Paranthias	furcifer	66	visual
Paranthias	furcifer	66	visual
Paranthias	furcifer	66	visual
Paranthias	furcifer	66	visual
Paranthias	furcifer	66	visual
Paranthias	furcifer	66	visual
Paranthias	furcifer	66	visual
Paranthias	furcifer	66	visual
Paranthias	furcifer	66	visual
Paranthias	furcifer	66	visual
Paranthias	furcifer	66	visual
Paranthias	furcifer	66	visual
Paranthias	furcifer	66	visual
Paranthias	furcifer	67	visual
Paranthias	furcifer	67	visual
Paranthias	furcifer	67	visual
Paranthias	furcifer	67	visual
Paranthias	furcifer	67	visual
Paranthias	furcifer	67	visual
Paranthias	furcifer	67	visual
Paranthias	furcifer	69	visual
Paranthias	furcifer	69	visual
Paranthias	furcifer	69	visual
Paranthias	furcifer	69	visual
Paranthias	furcifer	69	visual
Paranthias	furcifer	69	visual
Paranthias	furcifer	69	visual
Paranthias	furcifer	69	visual
Paranthias	furcifer	69	visual
Paranthias	furcifer	69	visual
Paranthias	furcifer	69	visual
Paranthias	furcifer	69	visual
Paranthias	furcifer	69	visual
Paranthias	furcifer	69	visual
Paranthias	furcifer	69	visual
Paranthias	furcifer	69	visual
Paranthias	furcifer	69	visual
Paranthias	furcifer	69	visual
Paranthias	furcifer	69	visual
Paranthias	furcifer	69	visual
Paranthias	furcifer	69	visual
Paranthias	furcifer	69	visual
Paranthias	furcifer	69	visual
Paranthias	furcifer	69	visual
Paranthias	furcifer	69	visual

Paranthias	furcifer	69	visual
Paranthias	furcifer	69	visual
Paranthias	furcifer	69	visual
Paranthias	furcifer	69	visual
Paranthias	furcifer	69	visual
Paranthias	furcifer	70	visual
Paranthias	furcifer	70	visual
Paranthias	furcifer	70	visual
Paranthias	furcifer	70	visual
Paranthias	furcifer	70	visual
Paranthias	furcifer	70	visual
Paranthias	furcifer	71	visual
Paranthias	furcifer	73	visual
Paranthias	furcifer	73	visual
Paranthias	furcifer	73	visual
Paranthias	furcifer	74	visual
Paranthias	furcifer	76	visual
Paranthias	furcifer	76	visual
Paranthias	furcifer	76	visual
Paranthias	furcifer	77	visual
Paranthias	furcifer	77	visual
Paranthias	furcifer	77	visual
Paranthias	furcifer	77	visual
Paranthias	furcifer	77	visual
Paranthias	furcifer	77	visual
Paranthias	furcifer	77	visual
Paranthias	furcifer	77	visual
Paranthias	furcifer	77	visual
Paranthias	furcifer	77	visual
Paranthias	furcifer	79	visual
Paranthias	furcifer	79	visual
Paranthias	furcifer	79	visual
Paranthias	furcifer	79	visual
Paranthias	furcifer	79	visual
Paranthias	furcifer	79	visual
Paranthias	furcifer	79	visual
Paranthias	furcifer	79	visual
Paranthias	furcifer	79	visual
Paranthias	furcifer	79	visual
Paranthias	furcifer	79	visual
Paranthias	furcifer	79	visual
Paranthias	furcifer	79	visual
Paranthias	furcifer	79	visual
Paranthias	furcifer	79	visual
Paranthias	furcifer	79	visual
Paranthias	furcifer	79	visual
Paranthias	furcifer	79	visual

Paranthias	furcifer	79	visual
Paranthias	furcifer	79	visual
Paranthias	furcifer	79	visual
Paranthias	furcifer	91	visual
Paranthias	furcifer	91	visual
Paranthias	furcifer	97	visual
Paranthias	furcifer	97	visual
Paranthias	furcifer	102	visual
Paranthias	furcifer	110	visual
Paranthias	furcifer	110	visual
Centropristis	fuscula	48	Specimen
Centropristis	fuscula	72	Specimen
Centropristis	fuscula	74	Visual
Centropristis	fuscula	96	visual
Centropristis	fuscula	119	visual
Centropristis	fuscula	122	visual
Centropristis	fuscula	127	visual
Centropristis	fuscula	128	visual
Centropristis	fuscula	197	visual
Centropristis	fuscula	199	visual
Centropristis	fuscula	234	visual
Centropristis	fuscula	236	visual
Centropristis	fuscula	245	visual
Jeboehlkia	gladifer	125	Specimen
Jeboehlkia	gladifer	171	visual
Jeboehlkia	gladifer	172	Specimen
Jeboehlkia	gladifer	174	visual
Jeboehlkia	gladifer	175	visual
Jeboehlkia	gladifer	175	visual
Jeboehlkia	gladifer	176	visual
Jeboehlkia	gladifer	177	visual
Jeboehlkia	gladifer	178	visual
Jeboehlkia	gladifer	179	visual
Jeboehlkia	gladifer	180	visual
Jeboehlkia	gladifer	180	visual
Jeboehlkia	gladifer	183	visual
Jeboehlkia	gladifer	185	visual
Jeboehlkia	gladifer	190	visual
Jeboehlkia	gladifer	191	visual
Jeboehlkia	gladifer	192	visual
Jeboehlkia	gladifer	192	visual
Jeboehlkia	gladifer	192	visual
Jeboehlkia	gladifer	198	visual
Jeboehlkia	gladifer	198	visual
Jeboehlkia	gladifer	206	visual
Jeboehlkia	gladifer	206	visual
Jeboehlkia	gladifer	208	visual

Jeboehlkia	gladifer	213	visual
Jeboehlkia	gladifer	215	visual
Jeboehlkia	gladifer	217	visual
Jeboehlkia	gladifer	218	visual
Jeboehlkia	gladifer	219	visual
Jeboehlkia	gladifer	221	visual
Jeboehlkia	gladifer	223	visual
Jeboehlkia	gladifer	227	visual
Jeboehlkia	gladifer	230	visual
Jeboehlkia	gladifer	232	Specimen
Jeboehlkia	gladifer	233	visual
Jeboehlkia	gladifer	234	visual
Jeboehlkia	gladifer	235	visual
Jeboehlkia	gladifer	236	visual
Jeboehlkia	gladifer	236	visual
Jeboehlkia	gladifer	238	visual
Jeboehlkia	gladifer	244	visual
Jeboehlkia	gladifer	244	visual
Jeboehlkia	gladifer	244	Specimen
Jeboehlkia	gladifer	245	visual
Jeboehlkia	gladifer	250	visual
Jeboehlkia	gladifer	251	visual
Jeboehlkia	gladifer	257	visual
Jeboehlkia	gladifer	257	visual
Jeboehlkia	gladifer	259	visual
Jeboehlkia	gladifer	262	visual
Jeboehlkia	gladifer	265	visual
Jeboehlkia	gladifer	272	visual
Jeboehlkia	gladifer	273	visual
Jeboehlkia	gladifer	287	visual
Jeboehlkia	gladifer	303	visual
Apogon	gouldi	102	visual
Apogon	gouldi	108	Specimen
Apogon	gouldi	108	visual
Apogon	gouldi	110	Specimen
Apogon	gouldi	112	visual
Apogon	gouldi	119	Specimen
Apogon	gouldi	122	visual
Apogon	gouldi	123	visual
Apogon	gouldi	130	visual
Apogon	gouldi	132	visual
Apogon	gouldi	134	visual
Apogon	gouldi	141	Specimen
Apogon	gouldi	141	Specimen
Apogon	gouldi	141	Specimen
Apogon	gouldi	147	visual
Apogon	gouldi	148	Specimen

Apogon	gouldi	151	Specimen
Apogon	gouldi	157	visual
Prognathodes	guyanensis	97	visual
Prognathodes	guyanensis	102	visual
Prognathodes	guyanensis	106	visual
Prognathodes	guyanensis	108	visual
Prognathodes	guyanensis	108	visual
Prognathodes	guyanensis	115	visual
Prognathodes	guyanensis	115	visual
Prognathodes	guyanensis	120	visual
Prognathodes	guyanensis	122	visual
Prognathodes	guyanensis	125	visual
Prognathodes	guyanensis	130	visual
Prognathodes	guyanensis	132	visual
Prognathodes	guyanensis	136	visual
Prognathodes	guyanensis	137	visual
Prognathodes	guyanensis	140	visual
Prognathodes	guyanensis	141	Specimen
Prognathodes	guyanensis	143	visual
Prognathodes	guyanensis	153	visual
Prognathodes	guyanensis	155	visual
Prognathodes	guyanensis	162	visual
Prognathodes	guyanensis	163	visual
Prognathodes	guyanensis	165	visual
Prognathodes	guyanensis	165	visual
Prognathodes	guyanensis	165	visual
Prognathodes	guyanensis	169	visual
Prognathodes	guyanensis	178	visual
Prognathodes	guyanensis	203	visual
Prognathodes	guyanensis	216	visual
Ptereleotris	helenae	68	visual
Ptereleotris	helenae	70	visual
Ptereleotris	helenae	70	visual
Ptereleotris	helenae	70	visual
Ptereleotris	helenae	73	visual
Ptereleotris	helenae	73	visual
Ptereleotris	helenae	73	visual
Ptereleotris	helenae	76	visual
Ptereleotris	helenae	76	visual
Ptereleotris	helenae	88	visual
Ptereleotris	helenae	88	visual
Ptereleotris	helenae	89	visual
Ptereleotris	helenae	89	visual
Ptereleotris	helenae	89	visual
Ptereleotris	helenae	89	visual
Ptereleotris	helenae	89	visual
Ptereleotris	helenae	89	visual

Ptereleotris	helenae	89	visual
Ptereleotris	helenae	89	visual
Ptereleotris	helenae	91	visual
Ptereleotris	helenae	100	visual
Ptereleotris	helenae	127	visual
Ptereleotris	helenae	127	visual
Gonioplectrus	hispanus	101	visual
Gonioplectrus	hispanus	124	visual
Gonioplectrus	hispanus	124	visual
Gonioplectrus	hispanus	126	visual
Gonioplectrus	hispanus	128	visual
Gonioplectrus	hispanus	133	visual
Gonioplectrus	hispanus	133	visual
Gonioplectrus	hispanus	134	visual
Gonioplectrus	hispanus	134	visual
Gonioplectrus	hispanus	136	visual
Gonioplectrus	hispanus	136	visual
Gonioplectrus	hispanus	137	visual
Gonioplectrus	hispanus	137	visual
Gonioplectrus	hispanus	139	visual
Gonioplectrus	hispanus	140	visual
Gonioplectrus	hispanus	141	visual
Gonioplectrus	hispanus	142	visual
Gonioplectrus	hispanus	142	visual
Gonioplectrus	hispanus	145	visual
Gonioplectrus	hispanus	145	visual
Gonioplectrus	hispanus	146	visual
Gonioplectrus	hispanus	147	visual
Gonioplectrus	hispanus	151	visual
Gonioplectrus	hispanus	151	visual
Gonioplectrus	hispanus	152	visual
Gonioplectrus	hispanus	153	visual
Gonioplectrus	hispanus	153	visual
Gonioplectrus	hispanus	155	visual
Gonioplectrus	hispanus	156	visual
Gonioplectrus	hispanus	156	Specimen
Gonioplectrus	hispanus	156	visual
Gonioplectrus	hispanus	156	visual
Gonioplectrus	hispanus	158	visual
Gonioplectrus	hispanus	159	visual
Gonioplectrus	hispanus	159	visual
Gonioplectrus	hispanus	162	visual
Gonioplectrus	hispanus	163	visual
Gonioplectrus	hispanus	164	visual
Gonioplectrus	hispanus	165	visual
Gonioplectrus	hispanus	167	visual
Gonioplectrus	hispanus	168	visual

Gonioplectrus	hispanus	168	visual
Gonioplectrus	hispanus	168	visual
Gonioplectrus	hispanus	168	visual
Gonioplectrus	hispanus	169	visual
Gonioplectrus	hispanus	171	visual
Gonioplectrus	hispanus	171	visual
Gonioplectrus	hispanus	171	visual
Gonioplectrus	hispanus	178	visual
Gonioplectrus	hispanus	178	visual
Gonioplectrus	hispanus	180	visual
Gonioplectrus	hispanus	187	visual
Gonioplectrus	hispanus	189	visual
Gonioplectrus	hispanus	205	visual
Gonioplectrus	hispanus	208	visual
Gonioplectrus	hispanus	224	visual
Palatogobius	incendius	117	visual
Palatogobius	incendius	117	visual
Palatogobius	incendius	117	visual
Palatogobius	incendius	117	visual
Palatogobius	incendius	117	visual
Palatogobius	incendius	117	visual
Palatogobius	incendius	117	visual
Palatogobius	incendius	117	visual
Palatogobius	incendius	117	visual
Palatogobius	incendius	117	visual
Palatogobius	incendius	117	visual
Palatogobius	incendius	117	visual
Palatogobius	incendius	117	visual
Palatogobius	incendius	117	visual
Palatogobius	incendius	117	visual
Palatogobius	incendius	117	visual
Palatogobius	incendius	117	visual
Palatogobius	incendius	117	visual
Palatogobius	incendius	117	visual
Palatogobius	incendius	117	visual
Palatogobius	incendius	117	visual
Palatogobius	incendius	117	visual
Palatogobius	incendius	117	visual
Palatogobius	incendius	117	visual
Palatogobius	incendius	117	visual
Palatogobius	incendius	117	visual
Palatogobius	incendius	117	visual
Palatogobius	incendius	117	visual
Palatogobius	incendius	117	visual
Palatogobius	incendius	117	visual
Palatogobius	incendius	117	visual

Palatogobius	incendius	117	visual
Palatogobius	incendius	117	visual
Palatogobius	incendius	117	visual
Palatogobius	incendius	117	visual
Palatogobius	incendius	117	visual
Palatogobius	incendius	117	visual
Palatogobius	incendius	117	visual
Palatogobius	incendius	117	visual
Palatogobius	incendius	117	visual
Palatogobius	incendius	117	visual
Palatogobius	incendius	117	visual
Palatogobius	incendius	117	visual
Palatogobius	incendius	117	visual
Palatogobius	incendius	117	visual
Palatogobius	incendius	117	visual
Palatogobius	incendius	117	visual
Palatogobius	incendius	117	visual
Palatogobius	incendius	117	visual
Palatogobius	incendius	117	visual
Palatogobius	incendius	117	visual
Palatogobius	incendius	117	visual
Palatogobius	incendius	117	visual
Palatogobius	incendius	117	visual
Palatogobius	incendius	117	visual
Palatogobius	incendius	117	visual
Palatogobius	incendius	117	visual
Palatogobius	incendius	117	visual
Palatogobius	incendius	117	visual
Palatogobius	incendius	117	visual
Palatogobius	incendius	117	visual
Palatogobius	incendius	117	visual
Palatogobius	incendius	117	visual
Palatogobius	incendius	117	visual
Palatogobius	incendius	117	visual
Palatogobius	incendius	117	visual
Palatogobius	incendius	117	visual
Palatogobius	incendius	117	visual
Palatogobius	incendius	117	visual
Palatogobius	incendius	117	visual
Palatogobius	incendius	117	visual
Palatogobius	incendius	117	visual
Palatogobius	incendius	117	visual
Palatogobius	incendius	117	visual
Palatogobius	incendius	117	visual
Palatogobius	incendius	117	visual
Palatogobius	incendius	117	visual
Palatogobius	incendius	117	visual

Palatogobius	incendius	117	visual
Palatogobius	incendius	117	visual
Palatogobius	incendius	117	visual
Palatogobius	incendius	117	visual
Palatogobius	incendius	117	visual
Palatogobius	incendius	117	visual
Palatogobius	incendius	117	visual
Palatogobius	incendius	117	visual
Palatogobius	incendius	117	visual
Palatogobius	incendius	117	visual
Palatogobius	incendius	117	visual
Palatogobius	incendius	117	visual
Palatogobius	incendius	117	visual
Palatogobius	incendius	117	visual
Palatogobius	incendius	117	visual
Palatogobius	incendius	117	visual
Palatogobius	incendius	117	visual
Palatogobius	incendius	117	visual
Palatogobius	incendius	117	visual
Palatogobius	incendius	117	visual
Palatogobius	incendius	117	visual
Palatogobius	incendius	119	visual
Palatogobius	incendius	119	visual
Palatogobius	incendius	119	visual
Palatogobius	incendius	119	visual
Palatogobius	incendius	119	visual
Palatogobius	incendius	119	visual
Palatogobius	incendius	119	visual
Palatogobius	incendius	119	visual
Palatogobius	incendius	119	visual
Palatogobius	incendius	119	visual
Palatogobius	incendius	119	visual
Palatogobius	incendius	119	visual
Palatogobius	incendius	119	visual
Palatogobius	incendius	119	visual
Palatogobius	incendius	119	visual
Palatogobius	incendius	119	visual
Palatogobius	incendius	119	visual
Palatogobius	incendius	119	visual
Palatogobius	incendius	119	visual
Palatogobius	incendius	119	visual
Palatogobius	incendius	119	visual
Palatogobius	incendius	119	visual
Palatogobius	incendius	119	visual
Palatogobius	incendius	119	visual
Palatogobius	incendius	119	visual
Palatogobius	incendius	119	visual

Palatogobius	incendius	119	visual
Palatogobius	incendius	119	visual
Palatogobius	incendius	119	visual
Palatogobius	incendius	119	visual
Palatogobius	incendius	119	visual
Palatogobius	incendius	119	visual
Palatogobius	incendius	119	visual
Palatogobius	incendius	119	visual
Palatogobius	incendius	119	visual
Palatogobius	incendius	119	visual
Palatogobius	incendius	119	visual
Palatogobius	incendius	119	visual
Palatogobius	incendius	119	visual
Palatogobius	incendius	119	visual
Palatogobius	incendius	120	visual
Palatogobius	incendius	120	visual
Palatogobius	incendius	120	visual
Palatogobius	incendius	120	visual
Palatogobius	incendius	120	visual
Palatogobius	incendius	120	visual
Palatogobius	incendius	120	visual
Palatogobius	incendius	120	visual
Palatogobius	incendius	120	visual
Palatogobius	incendius	120	visual
Palatogobius	incendius	120	visual
Palatogobius	incendius	120	visual
Palatogobius	incendius	120	visual
Palatogobius	incendius	120	visual
Palatogobius	incendius	120	visual
Palatogobius	incendius	120	visual
Palatogobius	incendius	120	visual
Palatogobius	incendius	120	visual
Palatogobius	incendius	120	visual
Palatogobius	incendius	120	visual
Palatogobius	incendius	120	Specimen
Palatogobius	incendius	120	Specimen
Palatogobius	incendius	122	visual
Palatogobius	incendius	122	visual
Palatogobius	incendius	122	visual
Palatogobius	incendius	122	visual
Palatogobius	incendius	122	visual
Palatogobius	incendius	122	visual
Palatogobius	incendius	122	visual
Palatogobius	incendius	122	visual
Palatogobius	incendius	122	visual
Palatogobius	incendius	122	visual
Palatogobius	incendius	122	visual

Palatogobius	incendius	122	visual
Palatogobius	incendius	122	visual
Palatogobius	incendius	122	visual
Palatogobius	incendius	122	visual
Palatogobius	incendius	122	visual
Palatogobius	incendius	122	visual
Palatogobius	incendius	122	visual
Palatogobius	incendius	122	visual
Palatogobius	incendius	122	visual
Palatogobius	incendius	126	Specimen
Palatogobius	incendius	128	Specimen
Palatogobius	incendius	128	Specimen
Palatogobius	incendius	128	Specimen
Palatogobius	incendius	128	visual
Palatogobius	incendius	128	visual
Palatogobius	incendius	128	visual
Palatogobius	incendius	128	visual
Palatogobius	incendius	128	visual
Palatogobius	incendius	128	visual
Palatogobius	incendius	128	visual
Palatogobius	incendius	128	visual
Palatogobius	incendius	128	visual
Palatogobius	incendius	128	visual
Palatogobius	incendius	128	visual
Palatogobius	incendius	128	visual
Palatogobius	incendius	128	visual
Palatogobius	incendius	128	visual
Palatogobius	incendius	128	visual
Palatogobius	incendius	128	visual
Palatogobius	incendius	128	visual
Palatogobius	incendius	128	visual
Palatogobius	incendius	128	visual
Palatogobius	incendius	128	visual
Palatogobius	incendius	128	visual
Palatogobius	incendius	128	visual
Palatogobius	incendius	128	visual
Palatogobius	incendius	128	visual
Palatogobius	incendius	128	visual
Palatogobius	incendius	128	visual
Palatogobius	incendius	128	visual
Palatogobius	incendius	128	visual
Palatogobius	incendius	128	visual
Palatogobius	incendius	128	visual
Palatogobius	incendius	128	Specimen
Palatogobius	incendius	128	Specimen
Palatogobius	incendius	128	Specimen
Palatogobius	incendius	128	Specimen

Palatogobius	incendius	128	Specimen
Palatogobius	incendius	128	Specimen
Palatogobius	incendius	140	visual
Palatogobius	incendius	140	visual
Palatogobius	incendius	140	visual
Palatogobius	incendius	140	visual
Palatogobius	incendius	140	visual
Palatogobius	incendius	140	visual
Palatogobius	incendius	140	visual
Palatogobius	incendius	140	visual
Palatogobius	incendius	140	visual
Palatogobius	incendius	140	visual
Palatogobius	incendius	140	visual
Palatogobius	incendius	140	visual
Palatogobius	incendius	140	visual
Palatogobius	incendius	140	visual
Palatogobius	incendius	140	visual
Palatogobius	incendius	140	visual
Palatogobius	incendius	140	visual
Palatogobius	incendius	140	visual
Palatogobius	incendius	140	visual
Palatogobius	incendius	140	visual
Palatogobius	incendius	140	visual
Palatogobius	incendius	140	visual
Palatogobius	incendius	140	visual
Palatogobius	incendius	140	visual
Palatogobius	incendius	140	visual
Palatogobius	incendius	140	visual
Palatogobius	incendius	140	visual
Palatogobius	incendius	140	visual
Palatogobius	incendius	140	visual
Palatogobius	incendius	140	visual
Palatogobius	incendius	140	visual
Palatogobius	incendius	140	visual
Palatogobius	incendius	140	visual
Palatogobius	incendius	140	visual
Palatogobius	incendius	140	visual
Palatogobius	incendius	140	visual
Palatogobius	incendius	140	visual
Palatogobius	incendius	140	visual
Palatogobius	incendius	140	visual
Palatogobius	incendius	140	visual
Palatogobius	incendius	140	visual
Palatogobius	incendius	140	visual
Palatogobius	incendius	140	visual
Palatogobius	incendius	140	visual
Palatogobius	incendius	140	visual

Palatogobius	incendius	140	visual
Palatogobius	incendius	140	visual
Palatogobius	incendius	140	visual
Palatogobius	incendius	140	visual
Palatogobius	incendius	140	visual
Palatogobius	incendius	140	visual
Palatogobius	incendius	140	visual
Palatogobius	incendius	140	visual
Palatogobius	incendius	140	visual
Palatogobius	incendius	140	visual
Palatogobius	incendius	140	visual
Palatogobius	incendius	140	visual
Palatogobius	incendius	140	visual
Palatogobius	incendius	140	visual
Palatogobius	incendius	140	visual
Palatogobius	incendius	152	Specimen
Palatogobius	incendius	152	Specimen
Palatogobius	incendius	158	visual
Palatogobius	incendius	158	visual
Palatogobius	incendius	158	visual
Palatogobius	incendius	158	visual
Palatogobius	incendius	158	visual
Palatogobius	incendius	158	visual
Palatogobius	incendius	158	visual
Palatogobius	incendius	158	visual
Palatogobius	incendius	158	visual
Palatogobius	incendius	158	visual
Palatogobius	incendius	158	visual
Palatogobius	incendius	158	visual
Palatogobius	incendius	158	visual
Palatogobius	incendius	158	visual
Palatogobius	incendius	158	visual
Palatogobius	incendius	158	visual
Palatogobius	incendius	158	visual
Palatogobius	incendius	158	visual
Palatogobius	incendius	158	visual
Palatogobius	incendius	158	visual
Palatogobius	incendius	158	visual
Palatogobius	incendius	158	visual
Palatogobius	incendius	158	visual
Palatogobius	incendius	158	visual
Palatogobius	incendius	158	visual
Palatogobius	incendius	158	visual
Palatogobius	incendius	158	visual
Palatogobius	incendius	158	visual
Palatogobius	incendius	158	visual
Palatogobius	incendius	158	visual

Palatogobius	incendius	184	Specimen
Palatogobius	incendius	205	Specimen
Chromis	insolata	48	visual
Chromis	insolata	49	visual
Chromis	insolata	49	visual
Chromis	insolata	49	visual
Chromis	insolata	51	visual
Chromis	insolata	52	visual
Chromis	insolata	53	visual
Chromis	insolata	55	visual
Chromis	insolata	55	visual
Chromis	insolata	56	visual
Chromis	insolata	56	visual
Chromis	insolata	56	visual
Chromis	insolata	56	visual
Chromis	insolata	58	visual
Chromis	insolata	58	visual
Chromis	insolata	58	visual
Chromis	insolata	58	visual
Chromis	insolata	58	visual
Chromis	insolata	58	visual
Chromis	insolata	59	visual
Chromis	insolata	59	visual
Chromis	insolata	59	visual
Chromis	insolata	59	visual
Chromis	insolata	59	visual
Chromis	insolata	59	visual
Chromis	insolata	59	visual
Chromis	insolata	59	visual
Chromis	insolata	59	visual
Chromis	insolata	59	visual
Chromis	insolata	59	visual
Chromis	insolata	59	visual
Chromis	insolata	59	visual
Chromis	insolata	59	visual
Chromis	insolata	59	visual
Chromis	insolata	59	visual
Chromis	insolata	59	visual
Chromis	insolata	59	visual
Chromis	insolata	59	visual
Chromis	insolata	59	visual
Chromis	insolata	59	visual
Chromis	insolata	59	visual
Chromis	insolata	61	visual
Chromis	insolata	61	visual
Chromis	insolata	61	visual
Chromis	insolata	61	visual

Chromis	insolata	64	visual
Chromis	insolata	64	visual
Chromis	insolata	64	visual
Chromis	insolata	64	visual
Chromis	insolata	64	visual
Chromis	insolata	64	visual
Chromis	insolata	64	visual
Chromis	insolata	64	visual
Chromis	insolata	64	visual
Chromis	insolata	64	visual
Chromis	insolata	64	visual
Chromis	insolata	64	visual
Chromis	insolata	64	visual
Chromis	insolata	64	visual
Chromis	insolata	64	visual
Chromis	insolata	65	visual
Chromis	insolata	66	visual
Chromis	insolata	66	visual
Chromis	insolata	66	visual
Chromis	insolata	66	visual
Chromis	insolata	66	visual
Chromis	insolata	66	visual
Chromis	insolata	66	visual
Chromis	insolata	66	visual
Chromis	insolata	66	visual
Chromis	insolata	66	visual
Chromis	insolata	66	visual
Chromis	insolata	67	visual
Chromis	insolata	67	visual
Chromis	insolata	67	visual
Chromis	insolata	67	visual
Chromis	insolata	67	visual
Chromis	insolata	67	visual
Chromis	insolata	67	visual
Chromis	insolata	67	visual
Chromis	insolata	67	visual
Chromis	insolata	67	visual
Chromis	insolata	67	visual
Chromis	insolata	67	visual
Chromis	insolata	67	visual
Chromis	insolata	67	visual
Chromis	insolata	67	visual
Chromis	insolata	67	visual
Chromis	insolata	67	visual
Chromis	insolata	67	visual
Chromis	insolata	67	visual
Chromis	insolata	67	visual

Chromis	insolata	67	visual
Chromis	insolata	67	visual
Chromis	insolata	67	visual
Chromis	insolata	67	visual
Chromis	insolata	67	visual
Chromis	insolata	67	visual
Chromis	insolata	67	visual
Chromis	insolata	70	visual
Chromis	insolata	70	visual
Chromis	insolata	70	visual
Chromis	insolata	70	visual
Chromis	insolata	70	visual
Chromis	insolata	70	visual
Chromis	insolata	70	visual
Chromis	insolata	70	visual
Chromis	insolata	70	visual
Chromis	insolata	70	visual
Chromis	insolata	70	visual
Chromis	insolata	71	visual
Chromis	insolata	71	visual
Chromis	insolata	72	visual
Chromis	insolata	73	visual
Chromis	insolata	73	visual
Chromis	insolata	73	visual
Chromis	insolata	73	visual
Chromis	insolata	73	visual
Chromis	insolata	73	visual
Chromis	insolata	75	visual
Chromis	insolata	75	visual
Chromis	insolata	75	visual
Chromis	insolata	75	visual
Chromis	insolata	76	visual
Chromis	insolata	76	visual
Chromis	insolata	76	visual
Chromis	insolata	76	visual
Chromis	insolata	76	visual
Chromis	insolata	76	visual
Chromis	insolata	76	visual
Chromis	insolata	76	visual
Chromis	insolata	76	visual
Chromis	insolata	76	visual
Chromis	insolata	76	visual
Chromis	insolata	76	visual
Chromis	insolata	76	visual
Chromis	insolata	76	visual
Chromis	insolata	76	visual
Chromis	insolata	78	visual

Chromis	insolata	78	visual
Chromis	insolata	78	visual
Chromis	insolata	78	visual
Chromis	insolata	78	visual
Chromis	insolata	78	visual
Chromis	insolata	78	visual
Chromis	insolata	79	visual
Chromis	insolata	80	visual
Chromis	insolata	80	visual
Chromis	insolata	80	visual
Chromis	insolata	80	visual
Chromis	insolata	80	visual
Chromis	insolata	80	visual
Chromis	insolata	80	visual
Chromis	insolata	80	visual
Chromis	insolata	80	visual
Chromis	insolata	80	visual
Chromis	insolata	80	visual
Chromis	insolata	80	visual
Chromis	insolata	80	visual
Chromis	insolata	80	visual
Chromis	insolata	80	visual
Chromis	insolata	80	visual
Chromis	insolata	80	visual
Chromis	insolata	80	visual
Chromis	insolata	80	visual
Chromis	insolata	80	visual
Chromis	insolata	80	visual
Chromis	insolata	81	visual
Chromis	insolata	81	visual
Chromis	insolata	81	visual
Chromis	insolata	81	visual
Chromis	insolata	81	visual
Chromis	insolata	82	visual
Chromis	insolata	83	visual
Chromis	insolata	84	visual
Chromis	insolata	84	visual
Chromis	insolata	84	visual
Chromis	insolata	84	visual
Chromis	insolata	84	visual
Chromis	insolata	84	visual
Chromis	insolata	84	visual
Chromis	insolata	84	visual
Chromis	insolata	84	visual
Chromis	insolata	84	visual
Chromis	insolata	84	visual
Chromis	insolata	85	visual

Chromis	insolata	87	visual
Chromis	insolata	89	visual
Chromis	insolata	89	visual
Chromis	insolata	89	visual
Chromis	insolata	89	visual
Chromis	insolata	90	visual
Chromis	insolata	90	visual
Chromis	insolata	90	visual
Chromis	insolata	90	visual
Chromis	insolata	90	visual
Chromis	insolata	91	visual
Chromis	insolata	91	visual
Chromis	insolata	91	visual
Chromis	insolata	92	visual
Chromis	insolata	100	visual
Chromis	insolata	100	visual
Chromis	insolata	101	visual
Chromis	insolata	104	visual
Chromis	insolata	104	visual
Chromis	insolata	106	visual
Chromis	insolata	106	visual
Chromis	insolata	106	visual
Chromis	insolata	107	visual
Chromis	insolata	107	visual
Chromis	insolata	107	visual
Chromis	insolata	108	visual
Chromis	insolata	110	visual
Myripristis	jacobus	40	visual
Myripristis	jacobus	46	visual
Myripristis	jacobus	55	visual
Myripristis	jacobus	59	visual
Myripristis	jacobus	61	visual
Myripristis	jacobus	61	visual
Myripristis	jacobus	61	visual
Myripristis	jacobus	62	visual
Myripristis	jacobus	64	visual
Myripristis	jacobus	67	visual
Myripristis	jacobus	76	visual
Myripristis	jacobus	79	visual
Myripristis	jacobus	83	visual
Canthigaster	jamestyleri	70	visual
Canthigaster	jamestyleri	73	visual
Canthigaster	jamestyleri	73	visual
Canthigaster	jamestyleri	73	visual
Canthigaster	jamestyleri	73	visual
Canthigaster	jamestyleri	73	visual
Canthigaster	jamestyleri	73	visual

Canthigaster	jamestyleri	73	visual
Canthigaster	jamestyleri	73	visual
Canthigaster	jamestyleri	73	visual
Canthigaster	jamestyleri	73	visual
Canthigaster	jamestyleri	73	visual
Canthigaster	jamestyleri	73	visual
Canthigaster	jamestyleri	73	visual
Canthigaster	jamestyleri	73	visual
Canthigaster	jamestyleri	73	visual
Canthigaster	jamestyleri	73	visual
Canthigaster	jamestyleri	73	visual
Canthigaster	jamestyleri	73	visual
Canthigaster	jamestyleri	73	visual
Canthigaster	jamestyleri	73	visual
Canthigaster	jamestyleri	73	visual
Canthigaster	jamestyleri	73	visual
Canthigaster	jamestyleri	73	visual
Canthigaster	jamestyleri	73	visual
Canthigaster	jamestyleri	73	visual
Canthigaster	jamestyleri	73	visual
Canthigaster	jamestyleri	73	visual
Canthigaster	jamestyleri	73	visual
Canthigaster	jamestyleri	73	visual
Canthigaster	jamestyleri	73	visual
Canthigaster	jamestyleri	73	visual
Canthigaster	jamestyleri	73	visual
Canthigaster	jamestyleri	73	visual
Canthigaster	jamestyleri	73	visual
Canthigaster	jamestyleri	73	visual
Canthigaster	jamestyleri	73	visual
Canthigaster	jamestyleri	73	visual
Canthigaster	jamestyleri	73	visual
Canthigaster	jamestyleri	73	visual
Canthigaster	jamestyleri	73	visual
Canthigaster	jamestyleri	73	visual
Canthigaster	jamestyleri	73	visual
Canthigaster	jamestyleri	73	visual
Canthigaster	jamestyleri	73	visual
Canthigaster	jamestyleri	73	visual
Canthigaster	jamestyleri	73	visual
Canthigaster	jamestyleri	73	visual
Canthigaster	jamestyleri	73	visual
Canthigaster	jamestyleri	73	visual
Canthigaster	jamestyleri	73	visual
Canthigaster	jamestyleri	73	visual
Canthigaster	jamestyleri	73	visual
Canthigaster	jamestyleri	73	visual

Canthigaster	jamestyleri	73	visual
Canthigaster	jamestyleri	73	visual
Canthigaster	jamestyleri	73	visual
Canthigaster	jamestyleri	73	visual
Canthigaster	jamestyleri	73	visual
Canthigaster	jamestyleri	73	visual
Canthigaster	jamestyleri	73	visual
Canthigaster	jamestyleri	73	visual
Canthigaster	jamestyleri	75	visual
Canthigaster	jamestyleri	79	visual
Canthigaster	jamestyleri	87	visual
Canthigaster	jamestyleri	87	visual
Canthigaster	jamestyleri	91	Specimen
Canthigaster	jamestyleri	91	Specimen
Canthigaster	jamestyleri	92	visual
Canthigaster	jamestyleri	92	visual
Canthigaster	jamestyleri	92	visual
Canthigaster	jamestyleri	92	visual
Canthigaster	jamestyleri	92	visual
Canthigaster	jamestyleri	92	visual
Canthigaster	jamestyleri	92	visual
Canthigaster	jamestyleri	92	visual
Canthigaster	jamestyleri	95	visual
Canthigaster	jamestyleri	96	visual
Canthigaster	jamestyleri	96	visual
Canthigaster	jamestyleri	96	visual
Canthigaster	jamestyleri	96	visual
Canthigaster	jamestyleri	96	visual
Canthigaster	jamestyleri	96	visual
Canthigaster	jamestyleri	96	visual
Canthigaster	jamestyleri	96	visual
Canthigaster	jamestyleri	96	visual
Canthigaster	jamestyleri	96	visual
Canthigaster	jamestyleri	96	visual
Canthigaster	jamestyleri	96	visual
Canthigaster	jamestyleri	112	visual
Canthigaster	jamestyleri	114	visual
Canthigaster	jamestyleri	114	visual
Canthigaster	jamestyleri	114	visual
Canthigaster	jamestyleri	116	visual
Canthigaster	jamestyleri	116	visual
Canthigaster	jamestyleri	116	visual
Canthigaster	jamestyleri	117	visual
Canthigaster	jamestyleri	117	visual
Canthigaster	jamestyleri	117	visual
Canthigaster	jamestyleri	117	visual
Canthigaster	jamestyleri	117	visual

Canthigaster	jamestyleri	120	visual
Canthigaster	jamestyleri	121	visual
Canthigaster	jamestyleri	122	visual
Canthigaster	jamestyleri	122	visual
Canthigaster	jamestyleri	126	visual
Canthigaster	jamestyleri	126	visual
Canthigaster	jamestyleri	128	visual
Canthigaster	jamestyleri	128	visual
Canthigaster	jamestyleri	131	visual
Canthigaster	jamestyleri	139	visual
Canthigaster	jamestyleri	140	visual
Canthigaster	jamestyleri	141	visual
Canthigaster	jamestyleri	141	visual
Canthigaster	jamestyleri	141	visual
Canthigaster	jamestyleri	149	visual
Lipogramma	klayi	72	visual
Lipogramma	klayi	72	visual
Lipogramma	klayi	73	visual
Lipogramma	klayi	73	visual
Lipogramma	klayi	75	visual
Lipogramma	klayi	79	visual
Lipogramma	klayi	79	Specimen
Lipogramma	klayi	79	Specimen
Lipogramma	klayi	79	Specimen
Lipogramma	klayi	79	Specimen
Lipogramma	klayi	80	visual
Lipogramma	klayi	82	visual
Lipogramma	klayi	84	visual
Lipogramma	klayi	87	visual
Lipogramma	klayi	89	visual
Lipogramma	klayi	90	visual
Lipogramma	klayi	91	visual
Lipogramma	klayi	91	visual
Lipogramma	klayi	92	visual
Lipogramma	klayi	93	visual
Lipogramma	klayi	94	visual
Lipogramma	klayi	94	visual
Lipogramma	klayi	94	visual
Lipogramma	klayi	94	visual
Lipogramma	klayi	94	visual
Lipogramma	klayi	94	visual
Lipogramma	klayi	94	visual
Lipogramma	klayi	94	visual
Lipogramma	klayi	94	visual
Lipogramma	klayi	94	visual
Lipogramma	klayi	94	visual
Lipogramma	klayi	94	visual

Lipogramma	klayi	95	visual
Lipogramma	klayi	96	visual
Lipogramma	klayi	96	visual
Lipogramma	klayi	98	visual
Lipogramma	klayi	98	visual
Lipogramma	klayi	98	visual
Lipogramma	klayi	99	visual
Lipogramma	klayi	99	visual
Lipogramma	klayi	99	visual
Lipogramma	klayi	99	visual
Lipogramma	klayi	101	visual
Lipogramma	klayi	101	visual
Lipogramma	klayi	101	visual
Lipogramma	klayi	102	visual
Lipogramma	klayi	107	visual
Lipogramma	klayi	113	visual
Lipogramma	klayi	114	visual
Lipogramma	levinsoni	91	visual
Lipogramma	levinsoni	91	visual
Lipogramma	levinsoni	108	visual
Lipogramma	levinsoni	114	visual
Lipogramma	levinsoni	118	Specimen
Lipogramma	levinsoni	118	Specimen
Lipogramma	levinsoni	119	visual
Lipogramma	levinsoni	122	visual
Lipogramma	levinsoni	123	visual
Lipogramma	levinsoni	128	visual
Lipogramma	levinsoni	130	Specimen
Lipogramma	levinsoni	130	Specimen
Lipogramma	levinsoni	132	Specimen
Lipogramma	levinsoni	132	Specimen
Lipogramma	levinsoni	135	visual
Lipogramma	levinsoni	137	visual
Lipogramma	levinsoni	137	visual
Lipogramma	levinsoni	137	visual
Lipogramma	levinsoni	137	visual
Lipogramma	levinsoni	137	visual
Lipogramma	levinsoni	137	visual
Lipogramma	levinsoni	140	visual
Lipogramma	levinsoni	141	Specimen
Lipogramma	levinsoni	141	Specimen
Lipogramma	levinsoni	154	visual
Gramma	loreto	40	visual
Gramma	loreto	41	visual
Gramma	loreto	42	visual
Gramma	loreto	42	visual
Gramma	loreto	42	visual

Gramma	loreto	42	visual
Gramma	loreto	42	visual
Gramma	loreto	42	visual
Gramma	loreto	42	visual
Gramma	loreto	42	visual
Gramma	loreto	42	visual
Gramma	loreto	42	visual
Gramma	loreto	46	visual
Gramma	loreto	55	visual
Gramma	loreto	56	visual
Gramma	loreto	58	visual
Gramma	loreto	58	visual
Gramma	loreto	59	visual
Gramma	loreto	59	visual
Gramma	loreto	59	visual
Gramma	loreto	61	visual
Gramma	loreto	61	visual
Gramma	loreto	61	visual
Gramma	loreto	61	visual
Gramma	loreto	62	visual
Gramma	loreto	63	visual
Gramma	loreto	67	visual
Serranus	luciopercanus	61	visual
Serranus	luciopercanus	70	visual
Serranus	luciopercanus	70	visual
Serranus	luciopercanus	70	visual
Serranus	luciopercanus	73	visual
Serranus	luciopercanus	73	visual
Serranus	luciopercanus	73	visual
Serranus	luciopercanus	74	visual
Serranus	luciopercanus	82	visual
Serranus	luciopercanus	90	visual
Serranus	luciopercanus	95	visual
Serranus	luciopercanus	95	visual
Serranus	luciopercanus	95	visual
Serranus	luciopercanus	95	visual
Serranus	luciopercanus	96	visual
Serranus	luciopercanus	97	visual
Serranus	luciopercanus	98	visual
Serranus	luciopercanus	98	visual
Serranus	luciopercanus	99	visual
Serranus	luciopercanus	99	visual
Serranus	luciopercanus	99	visual
Serranus	luciopercanus	99	visual
Serranus	luciopercanus	99	visual
Serranus	luciopercanus	99	visual
Serranus	luciopercanus	99	visual

Serranus	luciopercanus	99	visual
Serranus	luciopercanus	99	visual
Serranus	luciopercanus	99	visual
Serranus	luciopercanus	99	visual
Serranus	luciopercanus	100	visual
Serranus	luciopercanus	100	visual
Serranus	luciopercanus	100	visual
Serranus	luciopercanus	101	visual
Serranus	luciopercanus	101	visual
Serranus	luciopercanus	101	visual
Serranus	luciopercanus	101	visual
Serranus	luciopercanus	102	visual
Serranus	luciopercanus	104	visual
Serranus	luciopercanus	104	visual
Serranus	luciopercanus	104	visual
Serranus	luciopercanus	105	visual
Serranus	luciopercanus	105	visual
Serranus	luciopercanus	105	visual
Serranus	luciopercanus	105	visual
Serranus	luciopercanus	105	visual
Serranus	luciopercanus	105	visual
Serranus	luciopercanus	108	visual
Serranus	luciopercanus	110	visual
Serranus	luciopercanus	110	visual
Serranus	luciopercanus	110	visual
Serranus	luciopercanus	111	visual
Serranus	luciopercanus	113	visual
Serranus	luciopercanus	113	visual
Serranus	luciopercanus	114	visual
Serranus	luciopercanus	114	visual
Serranus	luciopercanus	116	visual
Serranus	luciopercanus	116	visual
Serranus	luciopercanus	116	visual
Serranus	luciopercanus	117	visual
Serranus	luciopercanus	118	visual
Serranus	luciopercanus	119	visual
Serranus	luciopercanus	119	visual
Serranus	luciopercanus	120	visual
Serranus	luciopercanus	120	visual
Serranus	luciopercanus	125	visual
Serranus	luciopercanus	126	visual
Serranus	luciopercanus	127	visual
Serranus	luciopercanus	129	Specimen
Serranus	luciopercanus	129	Specimen
Flammeo	marianus	53	visual
Flammeo	marianus	58	visual
Flammeo	marianus	58	visual

Flammeo	marianus	58	visual
Flammeo	marianus	59	visual
Flammeo	marianus	61	visual
Flammeo	marianus	61	visual
Flammeo	marianus	66	visual
Flammeo	marianus	66	visual
Flammeo	marianus	66	visual
Flammeo	marianus	69	visual
Flammeo	marianus	69	visual
Flammeo	marianus	69	visual
Flammeo	marianus	69	visual
Flammeo	marianus	69	visual
Flammeo	marianus	73	visual
Flammeo	marianus	74	visual
Flammeo	marianus	81	visual
Flammeo	marianus	82	visual
Flammeo	marianus	82	visual
Flammeo	marianus	84	visual
Flammeo	marianus	84	visual
Flammeo	marianus	84	visual
Flammeo	marianus	84	visual
Flammeo	marianus	85	visual
Flammeo	marianus	85	visual
Flammeo	marianus	85	visual
Flammeo	marianus	85	visual
Flammeo	marianus	85	visual
Flammeo	marianus	85	visual
Flammeo	marianus	85	visual
Flammeo	marianus	87	visual
Flammeo	marianus	87	Specimen
Flammeo	marianus	88	visual
Flammeo	marianus	91	visual
Flammeo	marianus	98	visual
Flammeo	marianus	101	visual
Pronotogrammus	martinicensis	85	visual
Pronotogrammus	martinicensis	92	visual
Pronotogrammus	martinicensis	93	visual
Pronotogrammus	martinicensis	94	visual
Pronotogrammus	martinicensis	94	visual
Pronotogrammus	martinicensis	94	visual
Pronotogrammus	martinicensis	94	visual
Pronotogrammus	martinicensis	94	visual
Pronotogrammus	martinicensis	94	visual
Pronotogrammus	martinicensis	94	visual
Pronotogrammus	martinicensis	94	visual
Pronotogrammus	martinicensis	94	visual
Pronotogrammus	martinicensis	94	visual

Pronotogrammus	martinicensis	94	visual
Pronotogrammus	martinicensis	96	Specimen
Pronotogrammus	martinicensis	96	Specimen
Pronotogrammus	martinicensis	96	Specimen
Pronotogrammus	martinicensis	97	visual
Pronotogrammus	martinicensis	102	visual
Pronotogrammus	martinicensis	102	visual
Pronotogrammus	martinicensis	102	visual
Pronotogrammus	martinicensis	104	visual
Pronotogrammus	martinicensis	104	visual
Pronotogrammus	martinicensis	106	visual
Pronotogrammus	martinicensis	107	visual
Pronotogrammus	martinicensis	107	visual
Pronotogrammus	martinicensis	107	visual
Pronotogrammus	martinicensis	107	visual
Pronotogrammus	martinicensis	108	visual
Pronotogrammus	martinicensis	109	visual
Pronotogrammus	martinicensis	110	visual
Pronotogrammus	martinicensis	111	visual
Pronotogrammus	martinicensis	111	visual
Pronotogrammus	martinicensis	111	visual
Pronotogrammus	martinicensis	111	visual
Pronotogrammus	martinicensis	111	visual
Pronotogrammus	martinicensis	112	visual
Pronotogrammus	martinicensis	112	visual
Pronotogrammus	martinicensis	113	visual
Pronotogrammus	martinicensis	113	visual
Pronotogrammus	martinicensis	113	visual
Pronotogrammus	martinicensis	113	visual
Pronotogrammus	martinicensis	113	visual
Pronotogrammus	martinicensis	113	visual
Pronotogrammus	martinicensis	113	visual
Pronotogrammus	martinicensis	113	visual
Pronotogrammus	martinicensis	113	visual
Pronotogrammus	martinicensis	113	visual
Pronotogrammus	martinicensis	113	visual
Pronotogrammus	martinicensis	113	visual
Pronotogrammus	martinicensis	113	visual
Pronotogrammus	martinicensis	113	visual
Pronotogrammus	martinicensis	113	visual
Pronotogrammus	martinicensis	113	visual
Pronotogrammus	martinicensis	113	visual
Pronotogrammus	martinicensis	113	visual
Pronotogrammus	martinicensis	113	visual
Pronotogrammus	martinicensis	113	visual
Pronotogrammus	martinicensis	113	visual
Pronotogrammus	martinicensis	113	visual

Pronotogrammus	martinicensis	113	visual
Pronotogrammus	martinicensis	113	visual
Pronotogrammus	martinicensis	113	visual
Pronotogrammus	martinicensis	114	visual
Pronotogrammus	martinicensis	114	visual
Pronotogrammus	martinicensis	114	visual
Pronotogrammus	martinicensis	114	visual
Pronotogrammus	martinicensis	114	visual
Pronotogrammus	martinicensis	114	visual
Pronotogrammus	martinicensis	115	visual
Pronotogrammus	martinicensis	115	visual
Pronotogrammus	martinicensis	115	visual
Pronotogrammus	martinicensis	116	visual
Pronotogrammus	martinicensis	116	visual
Pronotogrammus	martinicensis	116	visual
Pronotogrammus	martinicensis	116	visual
Pronotogrammus	martinicensis	116	visual
Pronotogrammus	martinicensis	116	visual
Pronotogrammus	martinicensis	116	visual
Pronotogrammus	martinicensis	116	visual
Pronotogrammus	martinicensis	116	visual
Pronotogrammus	martinicensis	116	visual
Pronotogrammus	martinicensis	116	visual
Pronotogrammus	martinicensis	116	visual
Pronotogrammus	martinicensis	116	visual
Pronotogrammus	martinicensis	116	visual
Pronotogrammus	martinicensis	116	visual
Pronotogrammus	martinicensis	116	visual
Pronotogrammus	martinicensis	116	visual
Pronotogrammus	martinicensis	116	visual
Pronotogrammus	martinicensis	116	visual
Pronotogrammus	martinicensis	116	visual
Pronotogrammus	martinicensis	116	visual
Pronotogrammus	martinicensis	116	visual
Pronotogrammus	martinicensis	116	visual
Pronotogrammus	martinicensis	116	visual
Pronotogrammus	martinicensis	116	visual
Pronotogrammus	martinicensis	116	visual
Pronotogrammus	martinicensis	116	visual
Pronotogrammus	martinicensis	116	visual
Pronotogrammus	martinicensis	116	visual
Pronotogrammus	martinicensis	116	visual
Pronotogrammus	martinicensis	116	visual
Pronotogrammus	martinicensis	118	visual
Pronotogrammus	martinicensis	118	visual
Pronotogrammus	martinicensis	118	visual
Pronotogrammus	martinicensis	118	visual

Pronotogrammus	martinicensis	119	visual
Pronotogrammus	martinicensis	119	visual
Pronotogrammus	martinicensis	119	visual
Pronotogrammus	martinicensis	119	visual
Pronotogrammus	martinicensis	119	visual
Pronotogrammus	martinicensis	119	visual
Pronotogrammus	martinicensis	119	visual
Pronotogrammus	martinicensis	119	visual
Pronotogrammus	martinicensis	119	visual
Pronotogrammus	martinicensis	119	visual
Pronotogrammus	martinicensis	119	visual
Pronotogrammus	martinicensis	119	visual
Pronotogrammus	martinicensis	119	visual
Pronotogrammus	martinicensis	119	visual
Pronotogrammus	martinicensis	119	visual
Pronotogrammus	martinicensis	119	visual
Pronotogrammus	martinicensis	119	visual
Pronotogrammus	martinicensis	119	visual
Pronotogrammus	martinicensis	119	visual
Pronotogrammus	martinicensis	119	visual
Pronotogrammus	martinicensis	119	visual
Pronotogrammus	martinicensis	119	visual
Pronotogrammus	martinicensis	119	visual
Pronotogrammus	martinicensis	120	visual
Pronotogrammus	martinicensis	120	visual
Pronotogrammus	martinicensis	120	visual
Pronotogrammus	martinicensis	120	visual
Pronotogrammus	martinicensis	120	visual
Pronotogrammus	martinicensis	120	visual
Pronotogrammus	martinicensis	120	visual
Pronotogrammus	martinicensis	120	visual
Pronotogrammus	martinicensis	120	visual
Pronotogrammus	martinicensis	120	visual
Pronotogrammus	martinicensis	120	visual
Pronotogrammus	martinicensis	120	visual
Pronotogrammus	martinicensis	120	visual
Pronotogrammus	martinicensis	120	visual
Pronotogrammus	martinicensis	120	visual
Pronotogrammus	martinicensis	120	visual
Pronotogrammus	martinicensis	120	visual
Pronotogrammus	martinicensis	120	visual
Pronotogrammus	martinicensis	120	visual
Pronotogrammus	martinicensis	120	visual
Pronotogrammus	martinicensis	120	visual
Pronotogrammus	martinicensis	120	visual
Pronotogrammus	martinicensis	120	visual
Pronotogrammus	martinicensis	120	visual

Pronotogrammus	martinicensis	121	visual
Pronotogrammus	martinicensis	121	visual
Pronotogrammus	martinicensis	121	visual
Pronotogrammus	martinicensis	122	visual
Pronotogrammus	martinicensis	122	visual
Pronotogrammus	martinicensis	122	visual
Pronotogrammus	martinicensis	122	visual
Pronotogrammus	martinicensis	122	visual
Pronotogrammus	martinicensis	122	visual
Pronotogrammus	martinicensis	122	visual
Pronotogrammus	martinicensis	122	visual
Pronotogrammus	martinicensis	122	visual
Pronotogrammus	martinicensis	122	visual
Pronotogrammus	martinicensis	122	visual
Pronotogrammus	martinicensis	122	visual
Pronotogrammus	martinicensis	122	visual
Pronotogrammus	martinicensis	122	visual
Pronotogrammus	martinicensis	122	visual
Pronotogrammus	martinicensis	122	visual
Pronotogrammus	martinicensis	122	visual
Pronotogrammus	martinicensis	122	visual
Pronotogrammus	martinicensis	122	visual
Pronotogrammus	martinicensis	122	visual
Pronotogrammus	martinicensis	122	visual
Pronotogrammus	martinicensis	122	visual
Pronotogrammus	martinicensis	122	visual
Pronotogrammus	martinicensis	122	visual
Pronotogrammus	martinicensis	122	visual
Pronotogrammus	martinicensis	122	visual
Pronotogrammus	martinicensis	122	visual
Pronotogrammus	martinicensis	122	visual
Pronotogrammus	martinicensis	122	visual
Pronotogrammus	martinicensis	122	visual
Pronotogrammus	martinicensis	122	visual
Pronotogrammus	martinicensis	122	visual
Pronotogrammus	martinicensis	125	visual
Pronotogrammus	martinicensis	125	visual
Pronotogrammus	martinicensis	125	Specimen
Pronotogrammus	martinicensis	125	Specimen
Pronotogrammus	martinicensis	125	Specimen
Pronotogrammus	martinicensis	125	Specimen
Pronotogrammus	martinicensis	126	visual
Pronotogrammus	martinicensis	127	visual
Pronotogrammus	martinicensis	127	visual
Pronotogrammus	martinicensis	128	visual
Pronotogrammus	martinicensis	128	visual
Pronotogrammus	martinicensis	128	visual

Pronotogrammus	martinicensis	129	visual
Pronotogrammus	martinicensis	129	visual
Pronotogrammus	martinicensis	129	visual
Pronotogrammus	martinicensis	129	visual
Pronotogrammus	martinicensis	129	visual
Pronotogrammus	martinicensis	129	visual
Pronotogrammus	martinicensis	129	visual
Pronotogrammus	martinicensis	129	visual
Pronotogrammus	martinicensis	129	visual
Pronotogrammus	martinicensis	130	visual
Pronotogrammus	martinicensis	130	visual
Pronotogrammus	martinicensis	130	visual
Pronotogrammus	martinicensis	130	visual
Pronotogrammus	martinicensis	131	visual
Pronotogrammus	martinicensis	131	visual
Pronotogrammus	martinicensis	131	visual
Pronotogrammus	martinicensis	131	visual
Pronotogrammus	martinicensis	132	visual
Pronotogrammus	martinicensis	132	visual
Pronotogrammus	martinicensis	132	visual
Pronotogrammus	martinicensis	132	visual
Pronotogrammus	martinicensis	132	visual
Pronotogrammus	martinicensis	132	visual
Pronotogrammus	martinicensis	132	visual
Pronotogrammus	martinicensis	132	visual
Pronotogrammus	martinicensis	132	visual
Pronotogrammus	martinicensis	132	visual
Pronotogrammus	martinicensis	132	visual
Pronotogrammus	martinicensis	133	visual
Pronotogrammus	martinicensis	133	visual
Pronotogrammus	martinicensis	133	visual
Pronotogrammus	martinicensis	133	visual
Pronotogrammus	martinicensis	133	visual
Pronotogrammus	martinicensis	134	visual
Pronotogrammus	martinicensis	134	visual
Pronotogrammus	martinicensis	134	visual
Pronotogrammus	martinicensis	134	visual
Pronotogrammus	martinicensis	134	visual
Pronotogrammus	martinicensis	134	visual
Pronotogrammus	martinicensis	134	visual
Pronotogrammus	martinicensis	134	visual
Pronotogrammus	martinicensis	136	visual
Pronotogrammus	martinicensis	137	visual
Pronotogrammus	martinicensis	137	visual
Pronotogrammus	martinicensis	137	visual
Pronotogrammus	martinicensis	137	visual
Pronotogrammus	martinicensis	137	visual

Pronotogrammus	martinicensis	137	visual
Pronotogrammus	martinicensis	137	visual
Pronotogrammus	martinicensis	137	visual
Pronotogrammus	martinicensis	137	visual
Pronotogrammus	martinicensis	137	visual
Pronotogrammus	martinicensis	137	visual
Pronotogrammus	martinicensis	137	visual
Pronotogrammus	martinicensis	137	visual
Pronotogrammus	martinicensis	137	visual
Pronotogrammus	martinicensis	137	visual
Pronotogrammus	martinicensis	137	visual
Pronotogrammus	martinicensis	137	visual
Pronotogrammus	martinicensis	137	visual
Pronotogrammus	martinicensis	137	visual
Pronotogrammus	martinicensis	137	visual
Pronotogrammus	martinicensis	137	visual
Pronotogrammus	martinicensis	137	visual
Pronotogrammus	martinicensis	137	visual
Pronotogrammus	martinicensis	137	visual
Pronotogrammus	martinicensis	138	visual
Pronotogrammus	martinicensis	138	visual
Pronotogrammus	martinicensis	138	visual
Pronotogrammus	martinicensis	138	visual
Pronotogrammus	martinicensis	138	visual
Pronotogrammus	martinicensis	138	visual
Pronotogrammus	martinicensis	138	visual
Pronotogrammus	martinicensis	138	visual
Pronotogrammus	martinicensis	138	visual
Pronotogrammus	martinicensis	138	visual
Pronotogrammus	martinicensis	138	visual
Pronotogrammus	martinicensis	138	visual
Pronotogrammus	martinicensis	139	visual
Pronotogrammus	martinicensis	139	visual
Pronotogrammus	martinicensis	139	visual
Pronotogrammus	martinicensis	139	visual
Pronotogrammus	martinicensis	139	visual
Pronotogrammus	martinicensis	139	visual
Pronotogrammus	martinicensis	139	visual
Pronotogrammus	martinicensis	140	visual
Pronotogrammus	martinicensis	140	visual
Pronotogrammus	martinicensis	140	visual
Pronotogrammus	martinicensis	140	visual
Pronotogrammus	martinicensis	142	visual
Pronotogrammus	martinicensis	142	visual
Pronotogrammus	martinicensis	142	visual
Pronotogrammus	martinicensis	142	visual
Pronotogrammus	martinicensis	143	visual

Pronotogrammus	martinicensis	143	Specimen
Pronotogrammus	martinicensis	143	visual
Pronotogrammus	martinicensis	143	visual
Pronotogrammus	martinicensis	143	visual
Pronotogrammus	martinicensis	145	visual
Pronotogrammus	martinicensis	145	visual
Pronotogrammus	martinicensis	145	visual
Pronotogrammus	martinicensis	146	visual
Pronotogrammus	martinicensis	146	visual
Pronotogrammus	martinicensis	146	visual
Pronotogrammus	martinicensis	146	visual
Pronotogrammus	martinicensis	146	visual
Pronotogrammus	martinicensis	147	visual
Pronotogrammus	martinicensis	148	visual
Pronotogrammus	martinicensis	149	visual
Pronotogrammus	martinicensis	149	visual
Pronotogrammus	martinicensis	149	visual
Pronotogrammus	martinicensis	149	visual
Pronotogrammus	martinicensis	151	visual
Pronotogrammus	martinicensis	151	visual
Pronotogrammus	martinicensis	151	visual
Pronotogrammus	martinicensis	151	visual
Pronotogrammus	martinicensis	151	visual
Pronotogrammus	martinicensis	152	visual
Pronotogrammus	martinicensis	152	visual
Pronotogrammus	martinicensis	152	visual
Pronotogrammus	martinicensis	152	visual
Pronotogrammus	martinicensis	154	visual
Pronotogrammus	martinicensis	154	visual
Pronotogrammus	martinicensis	155	visual
Pronotogrammus	martinicensis	155	visual
Pronotogrammus	martinicensis	155	visual
Pronotogrammus	martinicensis	156	visual
Pronotogrammus	martinicensis	156	visual
Pronotogrammus	martinicensis	157	visual
Pronotogrammus	martinicensis	157	visual
Pronotogrammus	martinicensis	157	visual
Pronotogrammus	martinicensis	157	visual
Pronotogrammus	martinicensis	157	visual
Pronotogrammus	martinicensis	158	visual
Pronotogrammus	martinicensis	158	visual
Pronotogrammus	martinicensis	158	visual
Pronotogrammus	martinicensis	158	visual
Pronotogrammus	martinicensis	158	visual
Pronotogrammus	martinicensis	158	visual
Pronotogrammus	martinicensis	158	visual
Pronotogrammus	martinicensis	158	visual

Pronotogrammus	martinicensis	158	visual
Pronotogrammus	martinicensis	159	visual
Pronotogrammus	martinicensis	159	visual
Pronotogrammus	martinicensis	159	visual
Pronotogrammus	martinicensis	159	visual
Pronotogrammus	martinicensis	159	visual
Pronotogrammus	martinicensis	159	visual
Pronotogrammus	martinicensis	159	visual
Pronotogrammus	martinicensis	159	visual
Pronotogrammus	martinicensis	159	visual
Pronotogrammus	martinicensis	159	visual
Pronotogrammus	martinicensis	159	visual
Pronotogrammus	martinicensis	159	visual
Pronotogrammus	martinicensis	159	visual
Pronotogrammus	martinicensis	159	visual
Pronotogrammus	martinicensis	159	visual
Pronotogrammus	martinicensis	159	visual
Pronotogrammus	martinicensis	159	visual
Pronotogrammus	martinicensis	159	visual
Pronotogrammus	martinicensis	159	visual
Pronotogrammus	martinicensis	159	visual
Pronotogrammus	martinicensis	159	visual
Pronotogrammus	martinicensis	159	visual
Pronotogrammus	martinicensis	160	visual
Pronotogrammus	martinicensis	160	visual
Pronotogrammus	martinicensis	160	visual
Pronotogrammus	martinicensis	160	visual
Pronotogrammus	martinicensis	161	visual
Pronotogrammus	martinicensis	161	visual
Pronotogrammus	martinicensis	161	visual
Pronotogrammus	martinicensis	162	visual
Pronotogrammus	martinicensis	162	visual
Pronotogrammus	martinicensis	162	visual
Pronotogrammus	martinicensis	162	visual
Pronotogrammus	martinicensis	162	visual
Pronotogrammus	martinicensis	162	visual
Pronotogrammus	martinicensis	162	visual
Pronotogrammus	martinicensis	162	visual
Pronotogrammus	martinicensis	162	visual
Pronotogrammus	martinicensis	162	visual
Pronotogrammus	martinicensis	162	visual
Pronotogrammus	martinicensis	162	visual
Pronotogrammus	martinicensis	162	visual
Pronotogrammus	martinicensis	162	visual
Pronotogrammus	martinicensis	162	visual
Pronotogrammus	martinicensis	162	visual
Pronotogrammus	martinicensis	162	visual

Pronotogrammus	martinicensis	162	visual
Pronotogrammus	martinicensis	162	visual
Pronotogrammus	martinicensis	162	visual
Pronotogrammus	martinicensis	162	visual
Pronotogrammus	martinicensis	162	visual
Pronotogrammus	martinicensis	162	visual
Pronotogrammus	martinicensis	162	visual
Pronotogrammus	martinicensis	162	visual
Pronotogrammus	martinicensis	162	visual
Pronotogrammus	martinicensis	162	visual
Pronotogrammus	martinicensis	162	visual
Pronotogrammus	martinicensis	162	visual
Pronotogrammus	martinicensis	162	visual
Pronotogrammus	martinicensis	162	visual
Pronotogrammus	martinicensis	162	visual
Pronotogrammus	martinicensis	162	visual
Pronotogrammus	martinicensis	162	visual
Pronotogrammus	martinicensis	162	visual
Pronotogrammus	martinicensis	162	visual
Pronotogrammus	martinicensis	162	visual
Pronotogrammus	martinicensis	162	visual
Pronotogrammus	martinicensis	163	visual
Pronotogrammus	martinicensis	163	visual
Pronotogrammus	martinicensis	163	visual
Pronotogrammus	martinicensis	163	visual
Pronotogrammus	martinicensis	163	visual
Pronotogrammus	martinicensis	164	visual
Pronotogrammus	martinicensis	164	visual
Pronotogrammus	martinicensis	164	visual
Pronotogrammus	martinicensis	164	visual
Pronotogrammus	martinicensis	164	visual
Pronotogrammus	martinicensis	165	visual
Pronotogrammus	martinicensis	165	visual
Pronotogrammus	martinicensis	165	visual
Pronotogrammus	martinicensis	165	visual
Pronotogrammus	martinicensis	165	visual
Pronotogrammus	martinicensis	165	visual
Pronotogrammus	martinicensis	165	visual
Pronotogrammus	martinicensis	165	visual
Pronotogrammus	martinicensis	165	visual
Pronotogrammus	martinicensis	165	visual
Pronotogrammus	martinicensis	165	visual
Pronotogrammus	martinicensis	165	visual
Pronotogrammus	martinicensis	165	visual
Pronotogrammus	martinicensis	165	visual
Pronotogrammus	martinicensis	166	visual
Pronotogrammus	martinicensis	166	visual

Pronotogrammus	martinicensis	166	visual
Pronotogrammus	martinicensis	166	visual
Pronotogrammus	martinicensis	166	visual
Pronotogrammus	martinicensis	166	visual
Pronotogrammus	martinicensis	166	visual
Pronotogrammus	martinicensis	166	visual
Pronotogrammus	martinicensis	167	visual
Pronotogrammus	martinicensis	168	visual
Pronotogrammus	martinicensis	168	visual
Pronotogrammus	martinicensis	168	visual
Pronotogrammus	martinicensis	168	visual
Pronotogrammus	martinicensis	169	visual
Pronotogrammus	martinicensis	169	visual
Pronotogrammus	martinicensis	169	visual
Pronotogrammus	martinicensis	169	visual
Pronotogrammus	martinicensis	169	visual
Pronotogrammus	martinicensis	169	visual
Pronotogrammus	martinicensis	169	visual
Pronotogrammus	martinicensis	169	visual
Pronotogrammus	martinicensis	169	visual
Pronotogrammus	martinicensis	169	visual
Pronotogrammus	martinicensis	169	visual
Pronotogrammus	martinicensis	171	visual
Pronotogrammus	martinicensis	171	visual
Pronotogrammus	martinicensis	171	visual
Pronotogrammus	martinicensis	171	visual
Pronotogrammus	martinicensis	171	visual
Pronotogrammus	martinicensis	171	visual
Pronotogrammus	martinicensis	171	visual
Pronotogrammus	martinicensis	171	visual
Pronotogrammus	martinicensis	171	visual
Pronotogrammus	martinicensis	172	visual
Pronotogrammus	martinicensis	172	visual
Pronotogrammus	martinicensis	173	visual
Pronotogrammus	martinicensis	174	visual
Pronotogrammus	martinicensis	174	visual
Pronotogrammus	martinicensis	174	visual
Pronotogrammus	martinicensis	175	visual
Pronotogrammus	martinicensis	175	visual
Pronotogrammus	martinicensis	177	visual
Pronotogrammus	martinicensis	177	visual
Pronotogrammus	martinicensis	177	visual
Pronotogrammus	martinicensis	177	visual
Pronotogrammus	martinicensis	179	visual
Pronotogrammus	martinicensis	180	visual
Pronotogrammus	martinicensis	180	visual
Pronotogrammus	martinicensis	180	visual

Pronotogrammus	martinicensis	180	visual
Pronotogrammus	martinicensis	180	visual
Pronotogrammus	martinicensis	182	visual
Pronotogrammus	martinicensis	183	visual
Pronotogrammus	martinicensis	183	visual
Pronotogrammus	martinicensis	183	visual
Pronotogrammus	martinicensis	183	visual
Pronotogrammus	martinicensis	183	visual
Pronotogrammus	martinicensis	183	visual
Pronotogrammus	martinicensis	183	visual
Pronotogrammus	martinicensis	183	visual
Pronotogrammus	martinicensis	183	visual
Pronotogrammus	martinicensis	183	visual
Pronotogrammus	martinicensis	183	visual
Pronotogrammus	martinicensis	183	visual
Pronotogrammus	martinicensis	183	visual
Pronotogrammus	martinicensis	183	visual
Pronotogrammus	martinicensis	183	visual
Pronotogrammus	martinicensis	183	visual
Pronotogrammus	martinicensis	183	visual
Pronotogrammus	martinicensis	183	visual
Pronotogrammus	martinicensis	183	visual
Pronotogrammus	martinicensis	183	visual
Pronotogrammus	martinicensis	183	visual
Pronotogrammus	martinicensis	183	visual
Pronotogrammus	martinicensis	183	visual
Pronotogrammus	martinicensis	183	visual
Pronotogrammus	martinicensis	183	visual
Pronotogrammus	martinicensis	183	visual
Pronotogrammus	martinicensis	184	visual
Pronotogrammus	martinicensis	185	visual
Pronotogrammus	martinicensis	185	visual
Pronotogrammus	martinicensis	185	visual
Pronotogrammus	martinicensis	186	visual
Pronotogrammus	martinicensis	186	visual
Pronotogrammus	martinicensis	186	visual
Pronotogrammus	martinicensis	186	visual
Pronotogrammus	martinicensis	187	visual
Pronotogrammus	martinicensis	187	visual
Pronotogrammus	martinicensis	187	visual
Pronotogrammus	martinicensis	187	visual
Pronotogrammus	martinicensis	188	visual
Pronotogrammus	martinicensis	189	visual
Pronotogrammus	martinicensis	189	visual
Pronotogrammus	martinicensis	190	visual
Pronotogrammus	martinicensis	190	visual
Pronotogrammus	martinicensis	190	visual

Pronotogrammus	martinicensis	190	visual
Pronotogrammus	martinicensis	191	visual
Pronotogrammus	martinicensis	192	visual
Pronotogrammus	martinicensis	193	visual
Pronotogrammus	martinicensis	194	visual
Pronotogrammus	martinicensis	194	visual
Pronotogrammus	martinicensis	194	visual
Pronotogrammus	martinicensis	194	visual
Pronotogrammus	martinicensis	194	visual
Pronotogrammus	martinicensis	194	visual
Pronotogrammus	martinicensis	194	visual
Pronotogrammus	martinicensis	194	visual
Pronotogrammus	martinicensis	198	visual
Pronotogrammus	martinicensis	198	visual
Pronotogrammus	martinicensis	201	visual
Pronotogrammus	martinicensis	201	visual
Pronotogrammus	martinicensis	203	visual
Pronotogrammus	martinicensis	203	visual
Pronotogrammus	martinicensis	207	visual
Pronotogrammus	martinicensis	208	visual
Pronotogrammus	martinicensis	219	visual
Pronotogrammus	martinicensis	219	visual
Pronotogrammus	martinicensis	220	Specimen
Pronotogrammus	martinicensis	221	visual
Pronotogrammus	martinicensis	221	visual
Pronotogrammus	martinicensis	221	visual
Pronotogrammus	martinicensis	221	visual
Pronotogrammus	martinicensis	230	visual
Pronotogrammus	martinicensis	293	visual
Liopropoma	mowbrayi	56	visual
Liopropoma	mowbrayi	66	visual
Liopropoma	mowbrayi	67	visual
Liopropoma	mowbrayi	69	visual
Liopropoma	mowbrayi	71	visual
Liopropoma	mowbrayi	73	visual
Liopropoma	mowbrayi	76	visual
Liopropoma	mowbrayi	76	visual
Liopropoma	mowbrayi	78	visual
Liopropoma	mowbrayi	78	visual
Liopropoma	mowbrayi	79	visual
Liopropoma	mowbrayi	80	visual
Liopropoma	mowbrayi	81	visual
Liopropoma	mowbrayi	82	visual
Liopropoma	mowbrayi	84	visual
Liopropoma	mowbrayi	84	visual
Liopropoma	mowbrayi	84	visual
Liopropoma	mowbrayi	84	visual

Liopropoma	mowbrayi	84	visual
Liopropoma	mowbrayi	85	visual
Liopropoma	mowbrayi	87	visual
Liopropoma	mowbrayi	88	visual
Liopropoma	mowbrayi	88	visual
Liopropoma	mowbrayi	89	visual
Liopropoma	mowbrayi	90	visual
Liopropoma	mowbrayi	90	visual
Liopropoma	mowbrayi	91	visual
Liopropoma	mowbrayi	91	visual
Liopropoma	mowbrayi	91	visual
Liopropoma	mowbrayi	93	visual
Liopropoma	mowbrayi	94	visual
Liopropoma	mowbrayi	94	visual
Liopropoma	mowbrayi	94	visual
Liopropoma	mowbrayi	94	visual
Liopropoma	mowbrayi	94	visual
Liopropoma	mowbrayi	95	visual
Liopropoma	mowbrayi	96	visual
Liopropoma	mowbrayi	96	Specimen
Liopropoma	mowbrayi	98	visual
Liopropoma	mowbrayi	98	visual
Liopropoma	mowbrayi	98	visual
Liopropoma	mowbrayi	99	visual
Liopropoma	mowbrayi	99	visual
Liopropoma	mowbrayi	99	visual
Liopropoma	mowbrayi	99	visual
Liopropoma	mowbrayi	99	visual
Liopropoma	mowbrayi	99	visual
Liopropoma	mowbrayi	99	visual
Liopropoma	mowbrayi	101	visual
Liopropoma	mowbrayi	101	visual
Liopropoma	mowbrayi	101	visual
Liopropoma	mowbrayi	101	visual
Liopropoma	mowbrayi	101	visual
Liopropoma	mowbrayi	101	visual
Liopropoma	mowbrayi	102	visual
Liopropoma	mowbrayi	102	visual
Liopropoma	mowbrayi	102	visual
Liopropoma	mowbrayi	102	visual
Liopropoma	mowbrayi	102	visual
Liopropoma	mowbrayi	103	visual
Liopropoma	mowbrayi	103	visual
Liopropoma	mowbrayi	103	visual
Liopropoma	mowbrayi	103	visual
Liopropoma	mowbrayi	103	visual
Liopropoma	mowbrayi	103	visual

Liopropoma	mowbrayi	103	visual
Liopropoma	mowbrayi	104	visual
Liopropoma	mowbrayi	105	visual
Liopropoma	mowbrayi	105	visual
Liopropoma	mowbrayi	105	visual
Liopropoma	mowbrayi	106	visual
Liopropoma	mowbrayi	106	visual
Liopropoma	mowbrayi	106	visual
Liopropoma	mowbrayi	106	visual
Liopropoma	mowbrayi	107	visual
Liopropoma	mowbrayi	107	visual
Liopropoma	mowbrayi	107	visual
Liopropoma	mowbrayi	108	visual
Liopropoma	mowbrayi	109	visual
Liopropoma	mowbrayi	110	visual
Liopropoma	mowbrayi	110	visual
Liopropoma	mowbrayi	110	visual
Liopropoma	mowbrayi	111	visual
Liopropoma	mowbrayi	111	visual
Liopropoma	mowbrayi	111	visual
Liopropoma	mowbrayi	111	visual
Liopropoma	mowbrayi	111	visual
Liopropoma	mowbrayi	112	visual
Liopropoma	mowbrayi	112	visual
Liopropoma	mowbrayi	113	visual
Liopropoma	mowbrayi	113	visual
Liopropoma	mowbrayi	113	visual
Liopropoma	mowbrayi	113	visual
Liopropoma	mowbrayi	113	visual
Liopropoma	mowbrayi	113	visual
Liopropoma	mowbrayi	113	visual
Liopropoma	mowbrayi	113	Specimen
Liopropoma	mowbrayi	114	visual
Liopropoma	mowbrayi	115	visual
Liopropoma	mowbrayi	116	visual
Liopropoma	mowbrayi	117	visual
Liopropoma	mowbrayi	117	visual
Liopropoma	mowbrayi	117	visual
Liopropoma	mowbrayi	118	visual
Liopropoma	mowbrayi	118	visual
Liopropoma	mowbrayi	118	visual
Liopropoma	mowbrayi	128	visual
Liopropoma	mowbrayi	128	visual
Liopropoma	mowbrayi	129	visual
Liopropoma	mowbrayi	130	visual
Liopropoma	mowbrayi	133	visual
Chromis	multilineata	41	visual

Chromis	multilineata	41	visual
Chromis	multilineata	41	visual
Chromis	multilineata	41	visual
Chromis	multilineata	41	visual
Chromis	multilineata	41	visual
Chromis	multilineata	41	visual
Chromis	multilineata	41	visual
Chromis	multilineata	41	visual
Chromis	multilineata	41	visual
Chromis	multilineata	41	visual
Chromis	multilineata	41	visual
Chromis	multilineata	41	visual
Chromis	multilineata	41	visual
Chromis	multilineata	41	visual
Chromis	multilineata	41	visual
Chromis	multilineata	41	visual
Chromis	multilineata	41	visual
Chromis	multilineata	41	visual
Chromis	multilineata	41	visual
Chromis	multilineata	41	visual
Chromis	multilineata	41	visual
Chromis	multilineata	41	visual
Chromis	multilineata	46	visual
Chromis	multilineata	49	visual
Chromis	multilineata	49	visual
Chromis	multilineata	50	visual
Chromis	multilineata	50	visual
Chromis	multilineata	50	visual
Chromis	multilineata	50	visual
Chromis	multilineata	50	visual
Chromis	multilineata	50	visual
Chromis	multilineata	50	visual
Chromis	multilineata	50	visual
Chromis	multilineata	50	visual
Chromis	multilineata	50	visual
Chromis	multilineata	50	visual
Chromis	multilineata	50	visual
Chromis	multilineata	50	visual
Chromis	multilineata	50	visual
Chromis	multilineata	50	visual
Chromis	multilineata	50	visual
Chromis	multilineata	50	visual
Chromis	multilineata	50	visual
Chromis	multilineata	50	visual
Chromis	multilineata	54	visual
Chromis	multilineata	55	visual
Chromis	multilineata	59	visual

Chromis	multilineata	61	visual
Antilligobius	nikkiae	82	visual
Antilligobius	nikkiae	82	visual
Antilligobius	nikkiae	82	visual
Antilligobius	nikkiae	82	visual
Antilligobius	nikkiae	91	visual
Antilligobius	nikkiae	91	Specimen
Antilligobius	nikkiae	91	Specimen
Antilligobius	nikkiae	91	Specimen
Antilligobius	nikkiae	94	visual
Antilligobius	nikkiae	95	visual
Antilligobius	nikkiae	95	visual
Antilligobius	nikkiae	95	visual
Antilligobius	nikkiae	95	visual
Antilligobius	nikkiae	95	visual
Antilligobius	nikkiae	95	visual
Antilligobius	nikkiae	95	visual
Antilligobius	nikkiae	95	visual
Antilligobius	nikkiae	95	visual
Antilligobius	nikkiae	95	visual
Antilligobius	nikkiae	95	visual
Antilligobius	nikkiae	95	visual
Antilligobius	nikkiae	95	visual
Antilligobius	nikkiae	95	visual
Antilligobius	nikkiae	96	visual
Antilligobius	nikkiae	97	visual
Antilligobius	nikkiae	98	visual
Antilligobius	nikkiae	98	visual
Antilligobius	nikkiae	98	visual
Antilligobius	nikkiae	98	visual
Antilligobius	nikkiae	98	visual
Antilligobius	nikkiae	98	visual
Antilligobius	nikkiae	98	visual
Antilligobius	nikkiae	98	visual
Antilligobius	nikkiae	98	visual
Antilligobius	nikkiae	98	visual
Antilligobius	nikkiae	98	visual
Antilligobius	nikkiae	98	visual
Antilligobius	nikkiae	98	visual
Antilligobius	nikkiae	98	visual
Antilligobius	nikkiae	98	visual
Antilligobius	nikkiae	98	visual
Antilligobius	nikkiae	98	visual
Antilligobius	nikkiae	98	visual
Antilligobius	nikkiae	98	visual
Antilligobius	nikkiae	98	visual
Antilligobius	nikkiae	98	visual

Antilligobius	nikkiae	98	Specimen
Antilligobius	nikkiae	99	visual
Antilligobius	nikkiae	99	visual
Antilligobius	nikkiae	99	visual
Antilligobius	nikkiae	99	visual
Antilligobius	nikkiae	99	visual
Antilligobius	nikkiae	99	visual
Antilligobius	nikkiae	99	visual
Antilligobius	nikkiae	99	visual
Antilligobius	nikkiae	99	visual
Antilligobius	nikkiae	99	visual
Antilligobius	nikkiae	99	visual
Antilligobius	nikkiae	99	visual
Antilligobius	nikkiae	99	visual
Antilligobius	nikkiae	99	visual
Antilligobius	nikkiae	99	visual
Antilligobius	nikkiae	99	visual
Antilligobius	nikkiae	99	visual
Antilligobius	nikkiae	99	visual
Antilligobius	nikkiae	99	visual
Antilligobius	nikkiae	99	visual
Antilligobius	nikkiae	99	visual
Antilligobius	nikkiae	99	visual
Antilligobius	nikkiae	100	visual
Antilligobius	nikkiae	101	visual
Antilligobius	nikkiae	101	visual
Antilligobius	nikkiae	101	visual
Antilligobius	nikkiae	101	visual
Antilligobius	nikkiae	101	visual
Antilligobius	nikkiae	101	visual
Antilligobius	nikkiae	101	visual
Antilligobius	nikkiae	101	visual
Antilligobius	nikkiae	101	visual
Antilligobius	nikkiae	101	visual
Antilligobius	nikkiae	101	visual
Antilligobius	nikkiae	101	visual
Antilligobius	nikkiae	101	visual
Antilligobius	nikkiae	102	visual
Antilligobius	nikkiae	102	visual
Antilligobius	nikkiae	103	visual
Antilligobius	nikkiae	103	visual
Antilligobius	nikkiae	103	visual
Antilligobius	nikkiae	104	visual
Antilligobius	nikkiae	105	visual
Antilligobius	nikkiae	105	visual
Antilligobius	nikkiae	107	visual
Antilligobius	nikkiae	107	visual

Antilligobius	nikkiae	107	visual
Antilligobius	nikkiae	108	visual
Antilligobius	nikkiae	108	visual
Antilligobius	nikkiae	108	visual
Antilligobius	nikkiae	108	visual
Antilligobius	nikkiae	110	visual
Antilligobius	nikkiae	110	visual
Antilligobius	nikkiae	110	visual
Antilligobius	nikkiae	110	visual
Antilligobius	nikkiae	110	visual
Antilligobius	nikkiae	110	visual
Antilligobius	nikkiae	110	visual
Antilligobius	nikkiae	110	visual
Antilligobius	nikkiae	110	visual
Antilligobius	nikkiae	110	visual
Antilligobius	nikkiae	110	visual
Antilligobius	nikkiae	110	visual
Antilligobius	nikkiae	110	visual
Antilligobius	nikkiae	110	visual
Antilligobius	nikkiae	110	visual
Antilligobius	nikkiae	110	visual
Antilligobius	nikkiae	110	visual
Antilligobius	nikkiae	110	visual
Antilligobius	nikkiae	110	visual
Antilligobius	nikkiae	110	visual
Antilligobius	nikkiae	110	visual
Antilligobius	nikkiae	110	visual
Antilligobius	nikkiae	110	visual
Antilligobius	nikkiae	110	visual
Antilligobius	nikkiae	110	visual
Antilligobius	nikkiae	110	visual
Antilligobius	nikkiae	110	visual
Antilligobius	nikkiae	110	visual
Antilligobius	nikkiae	110	visual
Antilligobius	nikkiae	110	visual
Antilligobius	nikkiae	110	visual
Antilligobius	nikkiae	110	visual
Antilligobius	nikkiae	110	visual
Antilligobius	nikkiae	110	visual
Antilligobius	nikkiae	110	visual
Antilligobius	nikkiae	111	visual
Antilligobius	nikkiae	111	visual
Antilligobius	nikkiae	111	visual
Antilligobius	nikkiae	111	visual
Antilligobius	nikkiae	111	visual
Antilligobius	nikkiae	111	visual
Antilligobius	nikkiae	111	visual

Antilligobius	nikkiae	111	visual
Antilligobius	nikkiae	111	visual
Antilligobius	nikkiae	111	visual
Antilligobius	nikkiae	111	visual
Antilligobius	nikkiae	111	visual
Antilligobius	nikkiae	111	visual
Antilligobius	nikkiae	111	visual
Antilligobius	nikkiae	111	visual
Antilligobius	nikkiae	111	visual
Antilligobius	nikkiae	111	visual
Antilligobius	nikkiae	111	visual
Antilligobius	nikkiae	111	visual
Antilligobius	nikkiae	111	visual
Antilligobius	nikkiae	111	visual
Antilligobius	nikkiae	111	visual
Antilligobius	nikkiae	111	visual
Antilligobius	nikkiae	111	Specimen
Antilligobius	nikkiae	112	visual
Antilligobius	nikkiae	112	visual
Antilligobius	nikkiae	112	visual
Antilligobius	nikkiae	112	visual
Antilligobius	nikkiae	112	visual
Antilligobius	nikkiae	112	visual
Antilligobius	nikkiae	112	visual
Antilligobius	nikkiae	112	visual
Antilligobius	nikkiae	112	visual
Antilligobius	nikkiae	112	visual
Antilligobius	nikkiae	112	visual
Antilligobius	nikkiae	112	visual
Antilligobius	nikkiae	112	visual
Antilligobius	nikkiae	112	visual
Antilligobius	nikkiae	112	visual
Antilligobius	nikkiae	112	visual
Antilligobius	nikkiae	112	visual
Antilligobius	nikkiae	112	visual
Antilligobius	nikkiae	112	visual
Antilligobius	nikkiae	112	visual
Antilligobius	nikkiae	112	visual
Antilligobius	nikkiae	112	visual
Antilligobius	nikkiae	112	visual
Antilligobius	nikkiae	112	visual
Antilligobius	nikkiae	112	visual
Antilligobius	nikkiae	112	visual
Antilligobius	nikkiae	112	visual
Antilligobius	nikkiae	112	visual
Antilligobius	nikkiae	112	visual
Antilligobius	nikkiae	112	visual

Antilligobius	nikkiae	112	visual
Antilligobius	nikkiae	112	visual
Antilligobius	nikkiae	112	visual
Antilligobius	nikkiae	112	visual
Antilligobius	nikkiae	113	visual
Antilligobius	nikkiae	113	visual
Antilligobius	nikkiae	113	visual
Antilligobius	nikkiae	113	visual
Antilligobius	nikkiae	113	visual
Antilligobius	nikkiae	113	visual
Antilligobius	nikkiae	113	visual
Antilligobius	nikkiae	113	visual
Antilligobius	nikkiae	113	visual
Antilligobius	nikkiae	113	visual
Antilligobius	nikkiae	113	visual
Antilligobius	nikkiae	113	visual
Antilligobius	nikkiae	113	visual
Antilligobius	nikkiae	113	visual
Antilligobius	nikkiae	113	visual
Antilligobius	nikkiae	113	visual
Antilligobius	nikkiae	115	visual
Antilligobius	nikkiae	116	visual
Antilligobius	nikkiae	118	visual
Antilligobius	nikkiae	118	visual
Antilligobius	nikkiae	119	visual
Antilligobius	nikkiae	119	visual
Antilligobius	nikkiae	119	visual
Antilligobius	nikkiae	119	visual
Antilligobius	nikkiae	119	visual
Antilligobius	nikkiae	119	visual
Antilligobius	nikkiae	119	visual
Antilligobius	nikkiae	119	visual
Antilligobius	nikkiae	119	visual
Antilligobius	nikkiae	119	visual
Antilligobius	nikkiae	119	visual
Antilligobius	nikkiae	119	visual
Antilligobius	nikkiae	119	visual
Antilligobius	nikkiae	119	visual
Antilligobius	nikkiae	119	visual
Antilligobius	nikkiae	119	visual
Antilligobius	nikkiae	119	visual
Antilligobius	nikkiae	119	visual
Antilligobius	nikkiae	119	visual
Antilligobius	nikkiae	119	visual
Antilligobius	nikkiae	119	visual
Antilligobius	nikkiae	122	visual
Antilligobius	nikkiae	122	visual

Antilligobius	nikkiae	124	visual
Antilligobius	nikkiae	124	visual
Antilligobius	nikkiae	124	visual
Antilligobius	nikkiae	124	visual
Antilligobius	nikkiae	124	visual
Antilligobius	nikkiae	124	visual
Antilligobius	nikkiae	124	visual
Antilligobius	nikkiae	124	visual
Antilligobius	nikkiae	124	visual
Antilligobius	nikkiae	124	visual
Antilligobius	nikkiae	124	visual
Antilligobius	nikkiae	125	visual
Antilligobius	nikkiae	128	visual
Antilligobius	nikkiae	128	Specimen
Antilligobius	nikkiae	130	visual
Antilligobius	nikkiae	130	visual
Antilligobius	nikkiae	131	Specimen
Antilligobius	nikkiae	131	Specimen
Antilligobius	nikkiae	144	visual
Antilligobius	nikkiae	185	visual
Antilligobius	nikkiae	205	visual
Serranus	notospilus	107	visual
Serranus	notospilus	108	visual
Serranus	notospilus	134	visual
Serranus	notospilus	137	visual
Serranus	notospilus	140	visual
Serranus	notospilus	140	visual
Serranus	notospilus	141	visual
Serranus	notospilus	142	visual
Serranus	notospilus	142	visual
Serranus	notospilus	142	visual
Serranus	notospilus	148	visual
Serranus	notospilus	149	visual
Serranus	notospilus	150	visual
Serranus	notospilus	150	visual
Serranus	notospilus	152	visual
Serranus	notospilus	154	visual
Serranus	notospilus	154	visual
Serranus	notospilus	158	visual
Serranus	notospilus	158	visual
Serranus	notospilus	161	visual
Serranus	notospilus	162	visual
Serranus	notospilus	162	visual
Serranus	notospilus	162	visual
Serranus	notospilus	165	visual
Serranus	notospilus	166	visual
Serranus	notospilus	175	visual

Serranus	notospilus	175	visual
Serranus	notospilus	175	visual
Serranus	notospilus	179	visual
Serranus	notospilus	180	visual
Serranus	notospilus	180	visual
Serranus	notospilus	183	visual
Serranus	notospilus	183	visual
Serranus	notospilus	183	visual
Serranus	notospilus	185	visual
Serranus	notospilus	186	visual
Serranus	notospilus	187	visual
Serranus	notospilus	187	visual
Serranus	notospilus	189	visual
Serranus	notospilus	191	visual
Serranus	notospilus	191	visual
Serranus	notospilus	191	visual
Serranus	notospilus	193	visual
Serranus	notospilus	193	visual
Serranus	notospilus	194	visual
Serranus	notospilus	195	visual
Serranus	notospilus	195	visual
Serranus	notospilus	197	visual
Serranus	notospilus	198	visual
Serranus	notospilus	198	visual
Serranus	notospilus	198	visual
Serranus	notospilus	198	visual
Serranus	notospilus	198	visual
Serranus	notospilus	199	visual
Serranus	notospilus	200	visual
Serranus	notospilus	201	visual
Serranus	notospilus	201	visual
Serranus	notospilus	201	visual
Serranus	notospilus	201	visual
Serranus	notospilus	201	visual
Serranus	notospilus	201	visual
Serranus	notospilus	202	visual
Serranus	notospilus	203	visual
Serranus	notospilus	203	visual
Serranus	notospilus	204	visual
Serranus	notospilus	206	visual
Serranus	notospilus	206	visual
Serranus	notospilus	206	visual
Serranus	notospilus	206	visual
Serranus	notospilus	206	visual
Serranus	notospilus	207	visual
Serranus	notospilus	207	visual
Serranus	notospilus	208	visual

Serranus	notospilus	209	visual
Serranus	notospilus	213	visual
Serranus	notospilus	213	visual
Serranus	notospilus	216	visual
Serranus	notospilus	217	visual
Serranus	notospilus	218	visual
Serranus	notospilus	218	visual
Serranus	notospilus	218	visual
Serranus	notospilus	221	visual
Serranus	notospilus	221	visual
Serranus	notospilus	222	visual
Serranus	notospilus	223	visual
Serranus	notospilus	223	visual
Serranus	notospilus	224	visual
Serranus	notospilus	224	visual
Serranus	notospilus	224	visual
Serranus	notospilus	226	visual
Serranus	notospilus	227	visual
Serranus	notospilus	229	visual
Serranus	notospilus	229	visual
Serranus	notospilus	230	visual
Serranus	notospilus	230	visual
Serranus	notospilus	231	visual
Serranus	notospilus	232	visual
Serranus	notospilus	232	visual
Serranus	notospilus	233	visual
Serranus	notospilus	233	visual
Serranus	notospilus	234	visual
Symphysanodon	octoactinus	143	visual
Symphysanodon	octoactinus	152	visual
Symphysanodon	octoactinus	155	visual
Symphysanodon	octoactinus	156	visual
Symphysanodon	octoactinus	158	visual
Symphysanodon	octoactinus	158	visual
Symphysanodon	octoactinus	158	visual
Symphysanodon	octoactinus	158	visual
Symphysanodon	octoactinus	158	visual
Symphysanodon	octoactinus	158	Specimen
Symphysanodon	octoactinus	158	Specimen
Symphysanodon	octoactinus	160	visual
Symphysanodon	octoactinus	160	visual
Symphysanodon	octoactinus	160	visual
Symphysanodon	octoactinus	162	visual
Symphysanodon	octoactinus	162	Specimen
Symphysanodon	octoactinus	162	Specimen
Symphysanodon	octoactinus	162	Specimen
Symphysanodon	octoactinus	162	visual

Symphysanodon	octoactinus	162	visual
Symphysanodon	octoactinus	162	visual
Symphysanodon	octoactinus	163	visual
Symphysanodon	octoactinus	163	visual
Symphysanodon	octoactinus	163	visual
Symphysanodon	octoactinus	163	visual
Symphysanodon	octoactinus	163	visual
Symphysanodon	octoactinus	163	visual
Symphysanodon	octoactinus	164	visual
Symphysanodon	octoactinus	164	visual
Symphysanodon	octoactinus	164	visual
Symphysanodon	octoactinus	165	visual
Symphysanodon	octoactinus	165	visual
Symphysanodon	octoactinus	166	visual
Symphysanodon	octoactinus	166	visual
Symphysanodon	octoactinus	166	visual
Symphysanodon	octoactinus	166	visual
Symphysanodon	octoactinus	166	visual
Symphysanodon	octoactinus	166	visual
Symphysanodon	octoactinus	166	visual
Symphysanodon	octoactinus	166	visual
Symphysanodon	octoactinus	166	visual
Symphysanodon	octoactinus	166	visual
Symphysanodon	octoactinus	168	visual
Symphysanodon	octoactinus	168	visual
Symphysanodon	octoactinus	170	visual
Symphysanodon	octoactinus	171	visual
Symphysanodon	octoactinus	171	visual
Symphysanodon	octoactinus	171	visual
Symphysanodon	octoactinus	172	visual
Symphysanodon	octoactinus	173	visual
Symphysanodon	octoactinus	173	visual
Symphysanodon	octoactinus	173	visual
Symphysanodon	octoactinus	173	visual
Symphysanodon	octoactinus	173	visual
Symphysanodon	octoactinus	173	visual
Symphysanodon	octoactinus	173	visual
Symphysanodon	octoactinus	173	visual
Symphysanodon	octoactinus	173	visual
Symphysanodon	octoactinus	173	visual
Symphysanodon	octoactinus	173	visual
Symphysanodon	octoactinus	173	visual
Symphysanodon	octoactinus	173	visual
Symphysanodon	octoactinus	173	visual
Symphysanodon	octoactinus	173	visual
Symphysanodon	octoactinus	173	visual
Symphysanodon	octoactinus	173	visual

Symphysanodon	octoactinus	173	visual
Symphysanodon	octoactinus	173	visual
Symphysanodon	octoactinus	173	visual
Symphysanodon	octoactinus	173	visual
Symphysanodon	octoactinus	173	visual
Symphysanodon	octoactinus	173	visual
Symphysanodon	octoactinus	173	visual
Symphysanodon	octoactinus	173	visual
Symphysanodon	octoactinus	173	visual
Symphysanodon	octoactinus	173	visual
Symphysanodon	octoactinus	173	visual
Symphysanodon	octoactinus	173	visual
Symphysanodon	octoactinus	173	visual
Symphysanodon	octoactinus	173	visual
Symphysanodon	octoactinus	175	visual
Symphysanodon	octoactinus	175	visual
Symphysanodon	octoactinus	176	Specimen
Symphysanodon	octoactinus	176	visual
Symphysanodon	octoactinus	177	visual
Symphysanodon	octoactinus	177	visual
Symphysanodon	octoactinus	178	visual
Symphysanodon	octoactinus	180	visual
Symphysanodon	octoactinus	180	visual
Symphysanodon	octoactinus	180	visual
Symphysanodon	octoactinus	181	visual
Symphysanodon	octoactinus	182	visual
Symphysanodon	octoactinus	183	visual
Symphysanodon	octoactinus	183	visual
Symphysanodon	octoactinus	183	Specimen
Symphysanodon	octoactinus	183	visual
Symphysanodon	octoactinus	184	visual
Symphysanodon	octoactinus	185	visual
Symphysanodon	octoactinus	185	visual
Symphysanodon	octoactinus	187	visual
Symphysanodon	octoactinus	187	visual
Symphysanodon	octoactinus	187	visual
Symphysanodon	octoactinus	187	visual
Symphysanodon	octoactinus	187	visual
Symphysanodon	octoactinus	188	visual
Symphysanodon	octoactinus	188	visual
Symphysanodon	octoactinus	189	visual
Symphysanodon	octoactinus	189	visual
Symphysanodon	octoactinus	191	visual
Symphysanodon	octoactinus	191	visual
Symphysanodon	octoactinus	191	visual
Symphysanodon	octoactinus	192	visual
Symphysanodon	octoactinus	192	visual

Symphysanodon	octoactinus	192	visual
Symphysanodon	octoactinus	194	visual
Symphysanodon	octoactinus	202	visual
Symphysanodon	octoactinus	202	visual
Symphysanodon	octoactinus	205	visual
Symphysanodon	octoactinus	208	visual
Symphysanodon	octoactinus	208	visual
Symphysanodon	octoactinus	208	visual
Symphysanodon	octoactinus	215	visual
Symphysanodon	octoactinus	217	visual
Symphysanodon	octoactinus	217	visual
Symphysanodon	octoactinus	217	visual
Symphysanodon	octoactinus	220	visual
Symphysanodon	octoactinus	221	visual
Symphysanodon	octoactinus	223	visual
Symphysanodon	octoactinus	223	visual
Symphysanodon	octoactinus	224	visual
Symphysanodon	octoactinus	224	visual
Symphysanodon	octoactinus	224	visual
Symphysanodon	octoactinus	224	visual
Symphysanodon	octoactinus	224	visual
Symphysanodon	octoactinus	224	visual
Symphysanodon	octoactinus	224	visual
Symphysanodon	octoactinus	224	visual
Symphysanodon	octoactinus	224	visual
Symphysanodon	octoactinus	224	visual
Symphysanodon	octoactinus	227	visual
Symphysanodon	octoactinus	232	visual
Symphysanodon	octoactinus	232	visual
Symphysanodon	octoactinus	232	visual
Symphysanodon	octoactinus	232	visual
Symphysanodon	octoactinus	232	visual
Symphysanodon	octoactinus	232	visual
Symphysanodon	octoactinus	232	visual
Symphysanodon	octoactinus	233	visual
Symphysanodon	octoactinus	233	visual
Symphysanodon	octoactinus	235	visual
Symphysanodon	octoactinus	236	visual
Symphysanodon	octoactinus	250	visual
Symphysanodon	octoactinus	251	visual
Liopropoma	olneyi	112	visual
Liopropoma	olneyi	113	visual
Liopropoma	olneyi	113	visual
Liopropoma	olneyi	115	visual
Liopropoma	olneyi	115	visual
Liopropoma	olneyi	119	visual
Liopropoma	olneyi	123	visual

Liopropoma	olneyi	123	visual
Liopropoma	olneyi	124	visual
Liopropoma	olneyi	124	visual
Liopropoma	olneyi	124	visual
Liopropoma	olneyi	125	visual
Liopropoma	olneyi	126	visual
Liopropoma	olneyi	126	visual
Liopropoma	olneyi	126	visual
Liopropoma	olneyi	127	visual
Liopropoma	olneyi	128	visual
Liopropoma	olneyi	128	visual
Liopropoma	olneyi	129	visual
Liopropoma	olneyi	130	visual
Liopropoma	olneyi	131	visual
Liopropoma	olneyi	131	visual
Liopropoma	olneyi	132	visual
Liopropoma	olneyi	133	Specimen
Liopropoma	olneyi	134	visual
Liopropoma	olneyi	137	visual
Liopropoma	olneyi	138	visual
Liopropoma	olneyi	140	visual
Liopropoma	olneyi	140	visual
Liopropoma	olneyi	140	Specimen
Liopropoma	olneyi	143	visual
Liopropoma	olneyi	147	Specimen
Liopropoma	olneyi	147	Specimen
Liopropoma	olneyi	148	visual
Liopropoma	olneyi	151	visual
Liopropoma	olneyi	152	visual
Liopropoma	olneyi	152	visual
Liopropoma	olneyi	154	visual
Liopropoma	olneyi	154	Specimen
Liopropoma	olneyi	154	visual
Liopropoma	olneyi	155	visual
Liopropoma	olneyi	155	visual
Liopropoma	olneyi	155	visual
Liopropoma	olneyi	156	visual
Liopropoma	olneyi	158	visual
Liopropoma	olneyi	158	visual
Liopropoma	olneyi	162	visual
Liopropoma	olneyi	162	visual
Liopropoma	olneyi	163	visual
Liopropoma	olneyi	163	visual
Liopropoma	olneyi	165	visual
Liopropoma	olneyi	167	visual
Liopropoma	olneyi	168	visual
Liopropoma	olneyi	171	visual

Liopropoma	olneyi	177	visual
Liopropoma	olneyi	179	visual
Liopropoma	olneyi	179	visual
Liopropoma	olneyi	179	visual
Liopropoma	olneyi	180	visual
Liopropoma	olneyi	180	visual
Liopropoma	olneyi	180	visual
Liopropoma	olneyi	180	visual
Liopropoma	olneyi	180	visual
Liopropoma	olneyi	181	visual
Liopropoma	olneyi	182	Specimen
Liopropoma	olneyi	183	visual
Liopropoma	olneyi	184	visual
Liopropoma	olneyi	185	visual
Liopropoma	olneyi	187	visual
Liopropoma	olneyi	187	visual
Liopropoma	olneyi	192	visual
Liopropoma	olneyi	192	Specimen
Liopropoma	olneyi	193	visual
Liopropoma	olneyi	194	visual
Liopropoma	olneyi	229	visual
Liopropoma	olneyi	229	visual
Clepticus	parrae	43	visual
Clepticus	parrae	43	visual
Clepticus	parrae	43	visual
Clepticus	parrae	43	visual
Clepticus	parrae	43	visual
Clepticus	parrae	43	visual
Clepticus	parrae	43	visual
Clepticus	parrae	43	visual
Clepticus	parrae	43	visual
Clepticus	parrae	43	visual
Clepticus	parrae	43	visual
Clepticus	parrae	43	visual
Clepticus	parrae	43	visual
Clepticus	parrae	43	visual
Clepticus	parrae	43	visual
Clepticus	parrae	43	visual
Clepticus	parrae	43	visual
Clepticus	parrae	43	visual
Clepticus	parrae	43	visual
Clepticus	parrae	43	visual
Clepticus	parrae	43	visual
Clepticus	parrae	43	visual
Clepticus	parrae	43	visual
Clepticus	parrae	43	visual
Clepticus	parrae	43	visual

Clepticus	parrae	43	visual
Clepticus	parrae	43	visual
Clepticus	parrae	43	visual
Clepticus	parrae	43	visual
Clepticus	parrae	43	visual
Clepticus	parrae	43	visual
Clepticus	parrae	43	visual
Clepticus	parrae	43	visual
Clepticus	parrae	43	visual
Clepticus	parrae	43	visual
Clepticus	parrae	43	visual
Clepticus	parrae	43	visual
Clepticus	parrae	43	visual
Clepticus	parrae	43	visual
Clepticus	parrae	43	visual
Clepticus	parrae	43	visual
Clepticus	parrae	43	visual
Clepticus	parrae	43	visual
Clepticus	parrae	43	visual
Clepticus	parrae	43	visual
Clepticus	parrae	43	visual
Clepticus	parrae	43	visual
Clepticus	parrae	43	visual
Clepticus	parrae	43	visual
Clepticus	parrae	43	visual
Clepticus	parrae	43	visual
Clepticus	parrae	43	visual
Clepticus	parrae	43	visual
Clepticus	parrae	43	visual
Clepticus	parrae	43	visual
Clepticus	parrae	43	visual
Clepticus	parrae	43	visual
Clepticus	parrae	43	visual
Clepticus	parrae	43	visual
Clepticus	parrae	43	visual
Clepticus	parrae	43	visual
Clepticus	parrae	43	visual
Clepticus	parrae	43	visual
Clepticus	parrae	43	visual
Clepticus	parrae	43	visual
Clepticus	parrae	46	visual
Clepticus	parrae	49	visual
Clepticus	parrae	49	visual
Clepticus	parrae	49	visual
Clepticus	parrae	49	visual
Clepticus	parrae	49	visual
Clepticus	parrae	49	visual

Clepticus	parrae	49	visual
Clepticus	parrae	49	visual
Clepticus	parrae	49	visual
Clepticus	parrae	49	visual
Clepticus	parrae	49	visual
Clepticus	parrae	55	visual
Clepticus	parrae	58	visual
Clepticus	parrae	61	visual
Clepticus	parrae	61	visual
Clepticus	parrae	61	visual
Clepticus	parrae	61	visual
Clepticus	parrae	61	visual
Clepticus	parrae	61	visual
Clepticus	parrae	61	visual
Clepticus	parrae	61	visual
Clepticus	parrae	61	visual
Clepticus	parrae	61	visual
Clepticus	parrae	61	visual
Clepticus	parrae	61	visual
Clepticus	parrae	61	visual
Clepticus	parrae	61	visual
Clepticus	parrae	61	visual
Clepticus	parrae	61	visual
Clepticus	parrae	61	visual
Clepticus	parrae	61	visual
Clepticus	parrae	61	visual
Clepticus	parrae	61	visual
Clepticus	parrae	61	visual
Clepticus	parrae	61	visual
Clepticus	parrae	61	visual
Clepticus	parrae	61	visual
Clepticus	parrae	67	visual
Clepticus	parrae	67	visual
Clepticus	parrae	67	visual
Clepticus	parrae	67	visual
Clepticus	parrae	67	visual
Clepticus	parrae	67	visual
Clepticus	parrae	67	visual
Clepticus	parrae	67	visual
Clepticus	parrae	67	visual
Clepticus	parrae	67	visual
Clepticus	parrae	67	visual
Clepticus	parrae	67	visual
Clepticus	parrae	67	visual
Clepticus	parrae	67	visual
Clepticus	parrae	67	visual
Clepticus	parrae	67	visual

Clepticus	parrae	67	visual
Clepticus	parrae	67	visual
Clepticus	parrae	67	visual
Clepticus	parrae	67	visual
Clepticus	parrae	67	visual
Clepticus	parrae	67	visual
Clepticus	parrae	67	visual
Clepticus	parrae	67	visual
Clepticus	parrae	67	visual
Clepticus	parrae	67	visual
Clepticus	parrae	67	visual
Clepticus	parrae	67	visual
Clepticus	parrae	67	visual
Clepticus	parrae	67	visual
Clepticus	parrae	67	visual
Clepticus	parrae	67	visual
Clepticus	parrae	67	visual
Clepticus	parrae	67	visual
Clepticus	parrae	67	visual
Clepticus	parrae	67	visual
Clepticus	parrae	67	visual
Clepticus	parrae	67	visual
Clepticus	parrae	67	visual
Clepticus	parrae	67	visual
Clepticus	parrae	67	visual
Clepticus	parrae	67	visual
Clepticus	parrae	67	visual
Clepticus	parrae	67	visual
Clepticus	parrae	67	visual
Clepticus	parrae	67	visual
Clepticus	parrae	67	visual
Clepticus	parrae	67	visual
Clepticus	parrae	67	visual
Clepticus	parrae	67	visual
Clepticus	parrae	67	visual
Clepticus	parrae	67	visual
Clepticus	parrae	67	visual
Clepticus	parrae	67	visual
Clepticus	parrae	67	visual
Clepticus	parrae	67	visual
Clepticus	parrae	67	visual
Clepticus	parrae	67	visual
Clepticus	parrae	67	visual
Clepticus	parrae	67	visual
Clepticus	parrae	67	visual
Clepticus	parrae	67	visual
Clepticus	parrae	67	visual

Clepticus	parrae	67	visual
Clepticus	parrae	67	visual
Clepticus	parrae	67	visual
Clepticus	parrae	67	visual
Clepticus	parrae	67	visual
Clepticus	parrae	67	visual
Clepticus	parrae	67	visual
Clepticus	parrae	67	visual
Clepticus	parrae	67	visual
Clepticus	parrae	67	visual
Clepticus	parrae	67	visual
Clepticus	parrae	67	visual
Clepticus	parrae	67	visual
Clepticus	parrae	67	visual
Clepticus	parrae	67	visual
Clepticus	parrae	67	visual
Clepticus	parrae	67	visual
Clepticus	parrae	67	visual
Clepticus	parrae	67	visual
Clepticus	parrae	67	visual
Clepticus	parrae	67	visual
Clepticus	parrae	67	visual
Clepticus	parrae	67	visual
Clepticus	parrae	67	visual
Clepticus	parrae	67	visual
Clepticus	parrae	67	visual
Clepticus	parrae	67	visual
Clepticus	parrae	67	visual
Clepticus	parrae	67	visual
Clepticus	parrae	67	visual
Clepticus	parrae	67	visual
Clepticus	parrae	67	visual
Clepticus	parrae	67	visual
Clepticus	parrae	67	visual
Clepticus	parrae	67	visual
Clepticus	parrae	67	visual
Clepticus	parrae	67	visual
Clepticus	parrae	67	visual
Clepticus	parrae	70	visual
Clepticus	parrae	70	visual
Clepticus	parrae	70	visual
Clepticus	parrae	70	visual
Clepticus	parrae	70	visual
Clepticus	parrae	70	visual
Clepticus	parrae	70	visual
Clepticus	parrae	70	visual
Clepticus	parrae	70	visual

Clepticus	parrae	70	visual
Clepticus	parrae	70	visual
Clepticus	parrae	70	visual
Clepticus	parrae	70	visual
Clepticus	parrae	70	visual
Clepticus	parrae	70	visual
Clepticus	parrae	70	visual
Clepticus	parrae	70	visual
Clepticus	parrae	70	visual
Clepticus	parrae	70	visual
Clepticus	parrae	70	visual
Clepticus	parrae	70	visual
Clepticus	parrae	70	visual
Clepticus	parrae	70	visual
Clepticus	parrae	70	visual
Clepticus	parrae	70	visual
Clepticus	parrae	73	visual
Clepticus	parrae	73	visual
Clepticus	parrae	73	visual
Clepticus	parrae	73	visual
Clepticus	parrae	73	visual
Clepticus	parrae	73	visual
Clepticus	parrae	73	visual
Clepticus	parrae	73	visual
Clepticus	parrae	73	visual
Clepticus	parrae	73	visual
Clepticus	parrae	73	visual
Clepticus	parrae	73	visual
Clepticus	parrae	73	visual
Clepticus	parrae	73	visual
Clepticus	parrae	73	visual
Clepticus	parrae	73	visual
Clepticus	parrae	73	visual
Clepticus	parrae	73	visual
Clepticus	parrae	73	visual
Clepticus	parrae	73	visual
Clepticus	parrae	73	visual
Clepticus	parrae	73	visual
Clepticus	parrae	73	visual
Clepticus	parrae	73	visual
Clepticus	parrae	73	visual
Clepticus	parrae	73	visual
Clepticus	parrae	73	visual
Clepticus	parrae	73	visual
Clepticus	parrae	73	visual
Clepticus	parrae	73	visual
Clepticus	parrae	73	visual

Clepticus	parrae	73	visual
Clepticus	parrae	73	visual
Clepticus	parrae	73	visual
Clepticus	parrae	73	visual
Clepticus	parrae	73	visual
Clepticus	parrae	73	visual
Clepticus	parrae	73	visual
Clepticus	parrae	73	visual
Clepticus	parrae	73	visual
Clepticus	parrae	73	visual
Clepticus	parrae	73	visual
Clepticus	parrae	73	visual
Clepticus	parrae	73	visual
Clepticus	parrae	73	visual
Clepticus	parrae	73	visual
Clepticus	parrae	73	visual
Clepticus	parrae	75	visual
Clepticus	parrae	75	visual
Clepticus	parrae	75	visual
Clepticus	parrae	75	visual
Clepticus	parrae	82	visual
Clepticus	parrae	84	visual
Clepticus	parrae	87	visual
Clepticus	parrae	91	visual
Clepticus	parrae	92	visual
Clepticus	parrae	93	visual
Clepticus	parrae	95	visual
Stegastes	partitus	40	visual
Stegastes	partitus	44	visual
Stegastes	partitus	44	visual
Stegastes	partitus	44	visual
Stegastes	partitus	44	visual
Stegastes	partitus	44	visual
Stegastes	partitus	44	visual
Stegastes	partitus	44	visual
Stegastes	partitus	44	visual
Stegastes	partitus	44	visual
Stegastes	partitus	44	visual
Stegastes	partitus	44	visual
Stegastes	partitus	46	visual
Stegastes	partitus	47	visual
Stegastes	partitus	47	visual
Stegastes	partitus	47	visual
Stegastes	partitus	49	visual
Stegastes	partitus	49	visual
Stegastes	partitus	49	visual
Stegastes	partitus	50	visual

Stegastes	partitus	51	visual
Stegastes	partitus	51	visual
Stegastes	partitus	52	visual
Stegastes	partitus	52	visual
Stegastes	partitus	52	visual
Stegastes	partitus	52	visual
Stegastes	partitus	52	visual
Stegastes	partitus	53	visual
Stegastes	partitus	53	visual
Stegastes	partitus	53	visual
Stegastes	partitus	54	visual
Stegastes	partitus	55	visual
Stegastes	partitus	55	visual
Stegastes	partitus	57	visual
Stegastes	partitus	58	visual
Stegastes	partitus	58	visual
Stegastes	partitus	58	visual
Stegastes	partitus	61	visual
Stegastes	partitus	63	visual
Stegastes	partitus	66	visual
Stegastes	partitus	67	visual
Stegastes	partitus	67	visual
Serranus	phoebe	89	visual
Serranus	phoebe	89	visual
Serranus	phoebe	103	visual
Serranus	phoebe	126	visual
Serranus	phoebe	127	visual
Serranus	phoebe	129	visual
Serranus	phoebe	130	visual
Serranus	phoebe	132	visual
Serranus	phoebe	137	visual
Serranus	phoebe	138	visual
Serranus	phoebe	140	visual
Serranus	phoebe	140	visual
Serranus	phoebe	141	Specimen
Serranus	phoebe	141	Specimen
Serranus	phoebe	142	visual
Serranus	phoebe	143	visual
Serranus	phoebe	144	visual
Serranus	phoebe	146	visual
Serranus	phoebe	164	visual
Serranus	phoebe	169	visual
Serranus	phoebe	170	visual
Serranus	phoebe	171	visual
Serranus	phoebe	173	visual
Serranus	phoebe	173	visual
Serranus	phoebe	186	visual

Xanthichthys	ringens	49	visual
Xanthichthys	ringens	49	visual
Xanthichthys	ringens	53	visual
Xanthichthys	ringens	53	visual
Xanthichthys	ringens	56	visual
Xanthichthys	ringens	59	visual
Xanthichthys	ringens	62	visual
Xanthichthys	ringens	62	visual
Xanthichthys	ringens	62	visual
Xanthichthys	ringens	63	visual
Xanthichthys	ringens	63	visual
Xanthichthys	ringens	66	visual
Xanthichthys	ringens	68	visual
Xanthichthys	ringens	77	visual
Xanthichthys	ringens	88	visual
Chromis	scotti	49	visual
Chromis	scotti	56	visual
Chromis	scotti	61	visual
Chromis	scotti	68	visual
Chromis	scotti	69	visual
Chromis	scotti	69	visual
Chromis	scotti	70	visual
Chromis	scotti	70	visual
Chromis	scotti	70	visual
Chromis	scotti	70	visual
Chromis	scotti	70	visual
Chromis	scotti	70	visual
Chromis	scotti	70	visual
Chromis	scotti	70	visual
Chromis	scotti	70	visual
Chromis	scotti	70	visual
Chromis	scotti	71	visual
Chromis	scotti	71	visual
Chromis	scotti	71	visual
Chromis	scotti	71	visual
Chromis	scotti	71	visual
Chromis	scotti	71	visual
Chromis	scotti	71	visual
Chromis	scotti	71	visual
Chromis	scotti	71	visual
Chromis	scotti	71	visual
Chromis	scotti	71	visual
Chromis	scotti	72	visual
Chromis	scotti	73	visual
Chromis	scotti	73	visual
Chromis	scotti	73	visual
Chromis	scotti	73	visual

Chromis	scotti	73	visual
Chromis	scotti	73	visual
Chromis	scotti	75	visual
Chromis	scotti	76	visual
Chromis	scotti	78	visual
Chromis	scotti	78	visual
Chromis	scotti	78	visual
Chromis	scotti	78	visual
Chromis	scotti	79	visual
Chromis	scotti	79	visual
Chromis	scotti	79	visual
Chromis	scotti	79	visual
Chromis	scotti	80	visual
Chromis	scotti	80	visual
Chromis	scotti	81	visual
Chromis	scotti	81	visual
Chromis	scotti	82	visual
Chromis	scotti	82	visual
Chromis	scotti	83	visual
Chromis	scotti	84	visual
Chromis	scotti	84	visual
Chromis	scotti	84	visual
Chromis	scotti	84	visual
Chromis	scotti	84	visual
Chromis	scotti	84	visual
Chromis	scotti	84	visual
Chromis	scotti	84	visual
Chromis	scotti	84	visual
Chromis	scotti	84	visual
Chromis	scotti	84	visual
Chromis	scotti	84	visual
Chromis	scotti	87	visual
Chromis	scotti	87	visual
Chromis	scotti	87	visual
Chromis	scotti	88	visual
Chromis	scotti	89	visual
Chromis	scotti	91	visual
Chromis	scotti	91	visual
Chromis	scotti	91	visual
Chromis	scotti	94	visual
Chromis	scotti	95	visual
Chromis	scotti	98	visual
Chromis	scotti	101	visual
Chromis	scotti	101	visual
Chromis	scotti	101	visual
Chromis	scotti	102	visual
Chromis	scotti	102	visual

Chromis	scotti	103	visual
Chromis	scotti	104	visual
Chromis	scotti	104	visual
Chromis	scotti	104	visual
Chromis	scotti	104	visual
Chromis	scotti	111	visual
Corniger	spinosus	137	visual
Corniger	spinosus	144	Specimen
Corniger	spinosus	156	Specimen
Corniger	spinosus	158	visual
Corniger	spinosus	169	Specimen
Corniger	spinosus	169	visual
Corniger	spinosus	171	visual
Corniger	spinosus	180	visual
Corniger	spinosus	238	visual
Chrionema	squamentum	159	Specimen
Chrionema	squamentum	160	visual
Chrionema	squamentum	183	visual
Chrionema	squamentum	185	visual
Chrionema	squamentum	190	visual
Chrionema	squamentum	191	visual
Chrionema	squamentum	198	visual
Chrionema	squamentum	206	visual
Chrionema	squamentum	210	visual
Chrionema	squamentum	215	visual
Chrionema	squamentum	215	visual
Chrionema	squamentum	216	Specimen
Chrionema	squamentum	216	Specimen
Chrionema	squamentum	216	visual
Chrionema	squamentum	217	visual
Chrionema	squamentum	221	visual
Chrionema	squamentum	223	visual
Chrionema	squamentum	223	visual
Chrionema	squamentum	226	visual
Chrionema	squamentum	226	visual
Chrionema	squamentum	226	visual
Chrionema	squamentum	226	Specimen
Chrionema	squamentum	226	visual
Chrionema	squamentum	226	visual
Chrionema	squamentum	227	visual
Chrionema	squamentum	227	visual
Chrionema	squamentum	228	visual
Chrionema	squamentum	228	visual
Chrionema	squamentum	229	visual
Chrionema	squamentum	229	visual
Chrionema	squamentum	230	visual
Chrionema	squamentum	230	visual

Chrionema	squamentum	231	visual
Chrionema	squamentum	232	visual
Chrionema	squamentum	233	visual
Chrionema	squamentum	234	visual
Chrionema	squamentum	234	visual
Chrionema	squamentum	234	visual
Chrionema	squamentum	235	visual
Chrionema	squamentum	237	visual
Chrionema	squamentum	237	visual
Chrionema	squamentum	238	visual
Chrionema	squamentum	238	visual
Chrionema	squamentum	238	visual
Chrionema	squamentum	240	visual
Chrionema	squamentum	241	visual
Chrionema	squamentum	241	visual
Chrionema	squamentum	241	visual
Chrionema	squamentum	243	visual
Chrionema	squamentum	244	visual
Chrionema	squamentum	245	visual
Chrionema	squamentum	250	visual
Chrionema	squamentum	252	visual
Chrionema	squamentum	256	visual
Chrionema	squamentum	259	visual
Chrionema	squamentum	267	visual
Chrionema	squamentum	269	Specimen
Chrionema	squamentum	282	Specimen
Chrionema	squamentum	283	visual
Chrionema	squamentum	283	visual
Chrionema	squamentum	283	visual
Chrionema	squamentum	283	visual
Chrionema	squamentum	283	visual
Chrionema	squamentum	283	visual
Chrionema	squamentum	283	visual
Chrionema	squamentum	283	visual
Chrionema	squamentum	283	visual
Chrionema	squamentum	283	visual
Chrionema	squamentum	283	visual
Chrionema	squamentum	283	visual
Chrionema	squamentum	283	visual
Chrionema	squamentum	283	visual
Chrionema	squamentum	283	visual
Chrionema	squamentum	283	visual
Chrionema	squamentum	283	visual
Chrionema	squamentum	283	visual
Chrionema	squamentum	283	visual
Chrionema	squamentum	283	visual

Chrionema	squamentum	283	visual
Chrionema	squamentum	283	visual
Chrionema	squamentum	283	Specimen
Chrionema	squamentum	287	visual
Chrionema	squamentum	287	visual
Chrionema	squamentum	287	visual
Chrionema	squamentum	287	visual
Chrionema	squamentum	287	visual
Chrionema	squamentum	287	visual
Chrionema	squamentum	287	visual
Chrionema	squamentum	287	visual
Chrionema	squamentum	287	visual
Chrionema	squamentum	287	visual
Chrionema	squamentum	287	visual
Chrionema	squamentum	287	visual
Chrionema	squamentum	287	visual
Chrionema	squamentum	287	visual
Chrionema	squamentum	287	visual
Chrionema	squamentum	287	visual
Chrionema	squamentum	287	visual
Chrionema	squamentum	287	visual
Chrionema	squamentum	287	visual
Chrionema	squamentum	287	visual
Chrionema	squamentum	287	visual
Chrionema	squamentum	287	visual
Chrionema	squamentum	287	visual
Chrionema	squamentum	290	visual
Chrionema	squamentum	290	visual
Chrionema	squamentum	290	visual
Chrionema	squamentum	290	visual
Chrionema	squamentum	290	visual
Chrionema	squamentum	290	visual
Chrionema	squamentum	290	visual
Chrionema	squamentum	290	visual
Chrionema	squamentum	290	visual
Chrionema	squamentum	290	visual
Chrionema	squamentum	290	visual
Chrionema	squamentum	290	visual
Chrionema	squamentum	290	visual
Chrionema	squamentum	290	visual
Chrionema	squamentum	290	visual
Chrionema	squamentum	290	visual
Chrionema	squamentum	290	visual
Chrionema	squamentum	290	visual
Chrionema	squamentum	290	visual
Chrionema	squamentum	290	visual
Chrionema	squamentum	290	visual

Chrionema	squamentum	290	visual
Chrionema	squamentum	290	visual
Chrionema	squamentum	293	visual
Chrionema	squamentum	296	visual
Chrionema	squamentum	296	visual
Chrionema	squamentum	296	visual
Chrionema	squamentum	296	visual
Chrionema	squamentum	296	visual
Chrionema	squamentum	296	visual
Chrionema	squamentum	296	visual
Chrionema	squamentum	296	visual
Chrionema	squamentum	296	visual
Chrionema	squamentum	296	visual
Chrionema	squamentum	296	visual
Chrionema	squamentum	296	visual
Chrionema	squamentum	296	visual
Chrionema	squamentum	296	visual
Chrionema	squamentum	296	visual
Chrionema	squamentum	296	visual
Chrionema	squamentum	296	visual
Chrionema	squamentum	296	visual
Chrionema	squamentum	296	visual
Chrionema	squamentum	296	visual
Chrionema	squamentum	296	visual
Chrionema	squamentum	296	visual
Chrionema	squamentum	296	visual
Chrionema	squamentum	297	visual
Chrionema	squamentum	297	visual
Chrionema	squamentum	297	visual
Chrionema	squamentum	297	visual
Chrionema	squamentum	297	visual
Chrionema	squamentum	299	visual
Chrionema	squamentum	299	visual
Chrionema	squamentum	299	visual
Chrionema	squamentum	301	visual
Chrionema	squamentum	301	visual
Chrionema	squamentum	301	visual
Chrionema	squamentum	301	visual
Chrionema	squamentum	302	visual
Chrionema	squamentum	302	visual
Chrionema	squamentum	303	visual
Chrionema	squamentum	303	visual
Chrionema	squamentum	304	visual
Chrionema	squamentum	305	visual
Chrionema	squamentum	305	visual
Chrionema	squamentum	305	visual
Chrionema	squamentum	305	visual

Chrionema	squamentum	305	visual
Chrionema	squamentum	305	visual
Chrionema	squamentum	305	visual
Chrionema	squamentum	305	visual
Chrionema	squamentum	305	visual
Chrionema	squamentum	305	visual
Chrionema	squamentum	305	visual
Chrionema	squamentum	305	visual
Choranthias	tenuis	94	Specimen
Choranthias	tenuis	113	visual
Choranthias	tenuis	114	Specimen
Choranthias	tenuis	115	visual
Choranthias	tenuis	115	visual
Choranthias	tenuis	115	visual
Choranthias	tenuis	115	visual
Choranthias	tenuis	116	Specimen
Choranthias	tenuis	125	visual
Choranthias	tenuis	127	Specimen
Choranthias	tenuis	127	Specimen
Choranthias	tenuis	127	visual
Choranthias	tenuis	128	visual
Choranthias	tenuis	140	visual
Choranthias	tenuis	141	Specimen
Choranthias	tenuis	155	visual
Choranthias	tenuis	165	Specimen
Choranthias	tenuis	174	Specimen
Choranthias	tenuis	183	visual
Choranthias	tenuis	186	Specimen
Choranthias	tenuis	186	Specimen
Choranthias	tenuis	301	visual
Ostichthys	trachypoma	161	visual
Ostichthys	trachypoma	165	visual
Ostichthys	trachypoma	166	visual
Ostichthys	trachypoma	168	visual
Ostichthys	trachypoma	171	visual
Ostichthys	trachypoma	172	visual
Ostichthys	trachypoma	172	visual
Ostichthys	trachypoma	183	visual
Ostichthys	trachypoma	184	visual
Ostichthys	trachypoma	185	visual
Ostichthys	trachypoma	187	visual
Ostichthys	trachypoma	187	visual
Ostichthys	trachypoma	187	visual
Ostichthys	trachypoma	189	visual
Ostichthys	trachypoma	191	visual
Ostichthys	trachypoma	201	visual
Ostichthys	trachypoma	202	visual

Ostichthys	trachypoma	202	visual
Ostichthys	trachypoma	202	Specimen
Ostichthys	trachypoma	204	Specimen
Ostichthys	trachypoma	208	visual
Ostichthys	trachypoma	209	visual
Ostichthys	trachypoma	214	visual
Ostichthys	trachypoma	215	visual
Ostichthys	trachypoma	215	Specimen
Ostichthys	trachypoma	219	visual
Ostichthys	trachypoma	219	visual
Ostichthys	trachypoma	219	visual
Ostichthys	trachypoma	221	visual
Ostichthys	trachypoma	223	visual
Ostichthys	trachypoma	223	visual
Ostichthys	trachypoma	223	visual
Ostichthys	trachypoma	223	visual
Ostichthys	trachypoma	224	visual
Ostichthys	trachypoma	224	visual
Ostichthys	trachypoma	224	visual
Ostichthys	trachypoma	226	visual
Ostichthys	trachypoma	227	visual
Ostichthys	trachypoma	229	visual
Ostichthys	trachypoma	230	visual
Ostichthys	trachypoma	230	visual
Ostichthys	trachypoma	231	visual
Ostichthys	trachypoma	231	visual
Ostichthys	trachypoma	232	visual
Ostichthys	trachypoma	232	visual
Ostichthys	trachypoma	232	visual
Ostichthys	trachypoma	232	visual
Ostichthys	trachypoma	232	visual
Ostichthys	trachypoma	232	visual
Ostichthys	trachypoma	232	visual
Ostichthys	trachypoma	232	visual
Ostichthys	trachypoma	233	visual
Ostichthys	trachypoma	233	visual
Ostichthys	trachypoma	233	visual
Ostichthys	trachypoma	233	visual
Ostichthys	trachypoma	233	visual
Ostichthys	trachypoma	234	visual
Ostichthys	trachypoma	235	visual
Ostichthys	trachypoma	235	visual
Ostichthys	trachypoma	235	visual
Ostichthys	trachypoma	235	visual
Ostichthys	trachypoma	236	visual
Ostichthys	trachypoma	237	visual
Ostichthys	trachypoma	237	visual

Ostichthys	trachypoma	237	visual
Ostichthys	trachypoma	237	visual
Ostichthys	trachypoma	238	visual
Ostichthys	trachypoma	238	visual
Ostichthys	trachypoma	238	visual
Ostichthys	trachypoma	238	visual
Ostichthys	trachypoma	238	visual
Ostichthys	trachypoma	238	visual
Ostichthys	trachypoma	239	visual
Ostichthys	trachypoma	239	visual
Ostichthys	trachypoma	240	visual
Ostichthys	trachypoma	240	visual
Ostichthys	trachypoma	247	visual
Ostichthys	trachypoma	250	visual
Ostichthys	trachypoma	250	visual
Ostichthys	trachypoma	253	visual
Ostichthys	trachypoma	255	visual
Ostichthys	trachypoma	255	visual
Ostichthys	trachypoma	257	visual
Ostichthys	trachypoma	260	visual
Ostichthys	trachypoma	260	visual
Ostichthys	trachypoma	263	visual
Ostichthys	trachypoma	264	visual
Ostichthys	trachypoma	265	visual
Ostichthys	trachypoma	266	visual
Ostichthys	trachypoma	267	visual
Ostichthys	trachypoma	267	visual
Ostichthys	trachypoma	269	visual
Ostichthys	trachypoma	269	visual
Ostichthys	trachypoma	274	visual
Ostichthys	trachypoma	287	visual
Baldwinella	vivanus	125	visual
Baldwinella	vivanus	129	visual
Baldwinella	vivanus	141	Specimen
Baldwinella	vivanus	141	Specimen
Baldwinella	vivanus	141	Specimen
Baldwinella	vivanus	141	Specimen
Baldwinella	vivanus	144	visual
Baldwinella	vivanus	144	visual
Baldwinella	vivanus	146	Specimen
Baldwinella	vivanus	148	visual
Baldwinella	vivanus	148	visual
Baldwinella	vivanus	149	visual
Baldwinella	vivanus	149	visual
Baldwinella	vivanus	155	visual
Baldwinella	vivanus	155	Specimen
Baldwinella	vivanus	155	visual

Baldwinella	vivanus	157	visual
Baldwinella	vivanus	162	Specimen
Baldwinella	vivanus	162	Specimen
Baldwinella	vivanus	162	Specimen
Baldwinella	vivanus	162	Specimen
Baldwinella	vivanus	164	visual
Baldwinella	vivanus	166	visual
Baldwinella	vivanus	168	visual
Baldwinella	vivanus	172	visual
Baldwinella	vivanus	173	visual
Baldwinella	vivanus	178	visual
Baldwinella	vivanus	180	visual
Baldwinella	vivanus	182	visual
Baldwinella	vivanus	183	visual
Baldwinella	vivanus	183	visual
Baldwinella	vivanus	185	visual
Baldwinella	vivanus	187	visual
Baldwinella	vivanus	189	visual
Baldwinella	vivanus	192	visual
Baldwinella	vivanus	192	visual
Baldwinella	vivanus	192	Specimen
Baldwinella	vivanus	193	visual
Baldwinella	vivanus	194	visual
Baldwinella	vivanus	195	visual
Baldwinella	vivanus	195	visual
Baldwinella	vivanus	195	Specimen
Baldwinella	vivanus	196	visual
Baldwinella	vivanus	198	visual
Baldwinella	vivanus	198	Specimen
Baldwinella	vivanus	199	Specimen
Baldwinella	vivanus	203	visual
Baldwinella	vivanus	204	visual
Baldwinella	vivanus	207	visual
Baldwinella	vivanus	208	visual
Baldwinella	vivanus	211	visual
Baldwinella	vivanus	213	visual
Baldwinella	vivanus	219	Specimen
Baldwinella	vivanus	221	visual
Baldwinella	vivanus	223	visual
Baldwinella	vivanus	223	Specimen
Baldwinella	vivanus	232	visual
Pontinus	nematophthalmus	160	Specimen
Pontinus	nematophthalmus	200	Specimen
Pontinus	nematophthalmus	200	Specimen
Pontinus	nematophthalmus	200	Specimen
Pontinus	nematophthalmus	200	Specimen
Pontinus	nematophthalmus	161	Specimen

Pontinus	nematophthalmus	133	Specimen
Pontinus	nematophthalmus	302	Specimen
Pontinus	nematophthalmus	242	Specimen
Pontinus	nematophthalmus	197	Specimen
Pontinus	nematophthalmus	197	Specimen
Pontinus	nematophthalmus	185	Specimen
Pontinus	nematophthalmus	189	Specimen
Pontinus	nematophthalmus	177	Specimen
Pontinus	nematophthalmus	177	Specimen
Pontinus	nematophthalmus	150	Specimen
Pontinus	nematophthalmus	253	Specimen
Pontinus	nematophthalmus	218	Specimen
Pontinus	nematophthalmus	219	Specimen
Pontinus	nematophthalmus	181	Specimen
Pontinus	nematophthalmus	237	Specimen
Pontinus	nematophthalmus	152	Visual
Pontinus	nematophthalmus	132	Visual
Haemulon	flavolineatum	67	Visual
Haemulon	flavolineatum	79	Visual
Haemulon	flavolineatum	70	Visual
Haemulon	flavolineatum	79	Visual
Haemulon	flavolineatum	34	Visual
Haemulon	flavolineatum	69	Visual
Haemulon	flavolineatum	72	Visual
Haemulon	flavolineatum	59	Visual
Haemulon	flavolineatum	67	Visual
Haemulon	flavolineatum	68	Visual
Haemulon	flavolineatum	59	Visual
Haemulon	flavolineatum	65	Visual
Haemulon	striatum	53	Visual
Haemulon	striatum	91	Visual
Haemulon	striatum	87	Visual
Haemulon	striatum	70	Visual
Haemulon	striatum	76	Visual
Haemulon	striatum	81	Visual
Haemulon	striatum	73	Visual
Haemulon	striatum	91	Visual
Haemulon	striatum	75	Visual
Haemulon	striatum	71	Visual
Haemulon	striatum	98	Visual
Haemulon	striatum	66	Visual
Haemulon	striatum	84	Visual
Haemulon	striatum	107	Visual
Haemulon	striatum	62	Visual
Haemulon	striatum	76	Visual
Haemulon	striatum	76	Visual
Haemulon	striatum	76	Visual

Haemulon	striatum	76	Visual
Haemulon	striatum	76	Visual
Haemulon	striatum	76	Visual
Haemulon	striatum	76	Visual
Haemulon	striatum	76	Visual
Haemulon	striatum	76	Visual
Haemulon	striatum	73	Visual
Haemulon	striatum	71	Visual
Haemulon	striatum	71	Visual
Haemulon	striatum	71	Visual
Haemulon	striatum	71	Visual
Haemulon	striatum	71	Visual
Haemulon	striatum	71	Visual
Haemulon	striatum	71	Visual
Haemulon	striatum	71	Visual
Haemulon	vittata	105	Visual
Haemulon	vittata	104	Visual
Haemulon	vittata	104	Visual
Haemulon	vittata	104	Visual
Haemulon	vittata	105	Visual
Haemulon	vittata	105	Visual
Haemulon	vittata	105	Visual
Haemulon	vittata	105	Visual
Haemulon	vittata	105	Visual
Haemulon	vittata	105	Visual
Haemulon	vittata	105	Visual
Haemulon	vittata	105	Visual
Haemulon	vittata	105	Visual
Haemulon	vittata	101	Visual
Haemulon	vittata	101	Visual
Haemulon	vittata	62	Visual
Haemulon	vittata	67	Visual
Bellator	egretta	176	specimen
Bellator	egretta	181	specimen
Bellator	egretta	182	specimen
Bellator	egretta	212	specimen
Bellator	egretta	232	visual
Bellator	egretta	221	visual
Bellator	egretta	197	visual
Chaunax	pictus	247	specimen
Chaunax	pictus	269	specimen
Chaunax	pictus	287	visual
Chaunax	pictus	290	visual
Chaunax	pictus	286	visual
Chaunax	pictus	270	visual
Coryphopterus	curasub	89	specimen
Coryphopterus	curasub	71	specimen

Coryphopterus	curasub	71	specimen
Coryphopterus	curasub	73	specimen
Coryphopterus	curasub	80	specimen
Decodon	shallow	122	specimen
Decodon	shallow	122	specimen
Decodon	deep	169	specimen
Decodon	shallow	155	specimen
Decodon	shallow	97	specimen
Decodon	shallow	135	specimen
Decodon	shallow	135	specimen
Decodon	deep	185	specimen
Decodon	deep	231	specimen
Decodon	deep	201	specimen
Decodon	deep	185	specimen
Decodon	deep	169	specimen
Decodon	shallow	181	specimen
Decodon	deep	162	specimen
Decodon	shallow	100	specimen
Foetorepus	agassizii	287	specimen
Foetorepus	agassizii	284	specimen
Foetorepus	agassizii	249	specimen
Foetorepus	agassizii	304	visual
Foetorepus	agassizii	253	visual
Foetorepus	agassizii	253	visual
Foetorepus	agassizii	286	visual
Liopropoma	santi	241	specimen
Liopropoma	santi	182	specimen
Lipogramma	few	177	specimen
Lipogramma	few	197	specimen
Lipogramma	few	173	specimen
Lipogramma	few	180	visual
Lipogramma	few	176	visual
Lipogramma	haberi	233	specimen
Lipogramma	haberi	152	specimen
Lipogramma	haberi	187	specimen
Lipogramma	many	138	specimen
Lipogramma	many	138	specimen
Lipogramma	many	161	specimen
Paroncheilus	affinis	114	specimen
Paroncheilus	affinis	114	specimen
Paroncheilus	affinis	114	specimen
Paroncheilus	affinis	114	specimen
Paroncheilus	affinis	114	specimen
Polylepion	polylepion	250	specimen
Polylepion	polylepion	272	specimen
Polylepion	polylepion	219	specimen
Pristipomoides	macrophthalmus	210	specimen

Sphyraenops	bairdianus	265	specimen
Sphyraenops	bairdianus	265	specimen
Sphyraenops	bairdianus	265	specimen
Sphyraenops	bairdianus	265	specimen
Sphyraenops	bairdianus	280	specimen
Sphyraenops	bairdianus	280	specimen
Varicus	decorum	224	specimen
Varicus	veliguttatus	152	specimen
Varicus	decorum	99	specimen
Varicus	veliguttatus	226	specimen
Varicus	veliguttatus	187	specimen
Varicus	decorum	164	specimen
Varicus	veliguttatus	191	specimen
Hemanthias	leptus	114	specimen
Hemanthias	leptus	114	specimen
Haptoclinus	dropi	163	specimen
Haptoclinus	dropi	157	specimen

Supplementary Table 2. Raw temperature data from 319 submersible dives to depths up to 309 m off

Dive File Nam	Year	Month	Day	Time at depth (s)	Depth (m)	Temp (°C)
19699403	2011	5	19	0	0.92	29.29
19699403	2011	5	19	10	1.63	29.28
19699403	2011	5	19	20	2.72	29.28
19699403	2011	5	19	30	2.78	29.28
19699403	2011	5	19	40	2.96	29.28
19699403	2011	5	19	50	2.99	29.28
19699403	2011	5	19	60	2.95	29.28
19699403	2011	5	19	70	2.88	29.28
19699403	2011	5	19	80	3.03	29.28
19699403	2011	5	19	90	3.26	29.27
19699403	2011	5	19	100	3.74	29.26
19699403	2011	5	19	110	5.23	29.25
19699403	2011	5	19	120	7.9	29.21
19699403	2011	5	19	130	9.83	29.19
19699403	2011	5	19	140	10.62	29.16
19699403	2011	5	19	150	11.33	29.14
19699403	2011	5	19	160	11.14	29.12
19699403	2011	5	19	170	11.32	29.1
19699403	2011	5	19	180	11.58	29.08
19699403	2011	5	19	190	11.85	29.07
19699403	2011	5	19	200	11.99	29.05
19699403	2011	5	19	210	11.98	29.03
19699403	2011	5	19	220	11.94	29.03
19699403	2011	5	19	230	12.16	29
19699403	2011	5	19	240	12.07	28.99
19699403	2011	5	19	250	12.2	28.98
19699403	2011	5	19	260	12.32	28.96
19699403	2011	5	19	270	12.71	28.95
19699403	2011	5	19	280	13.21	28.92
19699403	2011	5	19	290	13.2	28.92
19699403	2011	5	19	300	12.7	28.92
19699403	2011	5	19	310	12.64	28.91
19699403	2011	5	19	320	12.96	28.89
19699403	2011	5	19	330	13.46	28.88
19699403	2011	5	19	340	14.64	28.85
19699403	2011	5	19	350	16.21	28.82
19699403	2011	5	19	360	18.01	28.79
19699403	2011	5	19	370	19.16	28.78
19699403	2011	5	19	380	19.56	28.77
19699403	2011	5	19	390	19.89	28.76
19699403	2011	5	19	400	20.07	28.75
19699403	2011	5	19	410	21.01	28.71
19699403	2011	5	19	420	23.47	28.64
19699403	2011	5	19	430	26.05	28.53
19699403	2011	5	19	440	26.91	28.48

19699403	2011	5	19	450	28.57	28.39
19699403	2011	5	19	460	30.87	28.31
19699403	2011	5	19	470	33.48	28.23
19699403	2011	5	19	480	35.69	28.14
19699403	2011	5	19	490	38.14	28.03
19699403	2011	5	19	500	40.84	28.01
19699403	2011	5	19	510	43.14	27.96
19699403	2011	5	19	520	44.25	27.94
19699403	2011	5	19	530	45.91	27.92
19699403	2011	5	19	540	48.78	27.83
19699403	2011	5	19	550	50.85	27.78
19699403	2011	5	19	560	51.16	27.76
19699403	2011	5	19	570	51.39	27.73
19699403	2011	5	19	580	51.28	27.71
19699403	2011	5	19	590	51.38	27.68
19699403	2011	5	19	600	51.12	27.66
19699403	2011	5	19	610	51.32	27.64
19699403	2011	5	19	620	51.23	27.6
19699403	2011	5	19	630	51.13	27.58
19699403	2011	5	19	640	51.26	27.56
19699403	2011	5	19	650	51.26	27.55
19699403	2011	5	19	660	51.2	27.52
19699403	2011	5	19	670	51.29	27.5
19699403	2011	5	19	680	51.18	27.47
19699403	2011	5	19	690	51.21	27.46
19699403	2011	5	19	700	51.09	27.44
19699403	2011	5	19	710	51.19	27.42
19699403	2011	5	19	720	51.26	27.39
19699403	2011	5	19	730	51.25	27.39
19699403	2011	5	19	740	51.2	27.36
19699403	2011	5	19	750	51.2	27.34
19699403	2011	5	19	760	51.21	27.33
19699403	2011	5	19	770	51.18	27.32
19699403	2011	5	19	780	51.21	27.31
19699403	2011	5	19	790	51.3	27.3
19699403	2011	5	19	800	51.17	27.28
19699403	2011	5	19	810	51.21	27.27
19699403	2011	5	19	820	51.14	27.26
19699403	2011	5	19	830	51.09	27.25
19699403	2011	5	19	840	51.15	27.25
19699403	2011	5	19	850	51.17	27.24
19699403	2011	5	19	860	51.22	27.23
19699403	2011	5	19	870	51.31	27.22
19699403	2011	5	19	880	51.13	27.21
19699403	2011	5	19	890	51.19	27.21
19699403	2011	5	19	900	51.12	27.2
19699403	2011	5	19	910	51.14	27.19

19699403	2011	5	19	920	51.13	27.18
19699403	2011	5	19	930	51.14	27.17
19699403	2011	5	19	940	51.1	27.17
19699403	2011	5	19	950	51.15	27.17
19699403	2011	5	19	960	51.05	27.16
19699403	2011	5	19	970	51.23	27.15
19699403	2011	5	19	980	51.19	27.15
19699403	2011	5	19	990	51.2	27.14
19699403	2011	5	19	1000	51.21	27.14
19699403	2011	5	19	1010	51.28	27.12
19699403	2011	5	19	1020	51.22	27.12
19699403	2011	5	19	1030	51.29	27.12
19699403	2011	5	19	1040	51.2	27.11
19699403	2011	5	19	1050	50.67	27.1
19699403	2011	5	19	1060	50.94	27.1
19699403	2011	5	19	1070	50.78	27.1
19699403	2011	5	19	1080	50.83	27.1
19699403	2011	5	19	1090	50.8	27.1
19699403	2011	5	19	1100	50.81	27.09
19699403	2011	5	19	1110	50.78	27.1
19699403	2011	5	19	1120	50.91	27.1
19699403	2011	5	19	1130	51.5	27.09
19699403	2011	5	19	1140	52.22	27.09
19699403	2011	5	19	1150	52.84	27.08
19699403	2011	5	19	1160	53.41	27.07
19699403	2011	5	19	1170	54.05	27.07
19699403	2011	5	19	1180	54.01	27.07
19699403	2011	5	19	1190	54.05	27.07
19699403	2011	5	19	1200	54.4	27.07
19699403	2011	5	19	1210	54.34	27.07
19699403	2011	5	19	1220	54.23	27.06
19699403	2011	5	19	1230	54.29	27.06
19699403	2011	5	19	1240	54.25	27.06
19699403	2011	5	19	1250	54.45	27.06
19699403	2011	5	19	1260	54.28	27.07
19699403	2011	5	19	1270	54.28	27.05
19699403	2011	5	19	1280	54.18	27.05
19699403	2011	5	19	1290	54.21	27.06
19699403	2011	5	19	1300	54.23	27.06
19699403	2011	5	19	1310	54.1	27.05
19699403	2011	5	19	1320	53.99	27.05
19699403	2011	5	19	1330	53.9	27.05
19699403	2011	5	19	1340	53.86	27.05
19699403	2011	5	19	1350	53.99	27.05
19699403	2011	5	19	1360	54.37	27.05
19699403	2011	5	19	1370	54.67	27.04
19699403	2011	5	19	1380	54.37	27.04

19699403	2011	5	19	1390	54.1	27.05
19699403	2011	5	19	1400	53.88	27.04
19699403	2011	5	19	1410	54.06	27.04
19699403	2011	5	19	1420	54.47	27.04
19699403	2011	5	19	1430	53.31	27.05
19699403	2011	5	19	1440	52.12	27.05
19699403	2011	5	19	1450	51.07	27.07
19699403	2011	5	19	1460	49.43	27.07
19699403	2011	5	19	1470	47.3	27.09
19699403	2011	5	19	1480	46.38	27.1
19699403	2011	5	19	1490	45.48	27.1
19699403	2011	5	19	1500	45.16	27.1
19699403	2011	5	19	1510	44.43	27.1
19699403	2011	5	19	1520	43.84	27.11
19699403	2011	5	19	1530	43.03	27.12
19699403	2011	5	19	1540	42.3	27.13
19699403	2011	5	19	1550	41.52	27.14
19699403	2011	5	19	1560	41.47	27.14
19699403	2011	5	19	1570	42.09	27.14
19699403	2011	5	19	1580	42.63	27.15
19699403	2011	5	19	1590	43.36	27.15
19699403	2011	5	19	1600	43.67	27.15
19699403	2011	5	19	1610	43.52	27.16
19699403	2011	5	19	1620	43.57	27.16
19699403	2011	5	19	1630	43.51	27.16
19699403	2011	5	19	1640	43.43	27.16
19699403	2011	5	19	1650	43.75	27.17
19699403	2011	5	19	1660	43.95	27.17
19699403	2011	5	19	1670	44.24	27.16
19699403	2011	5	19	1680	44.54	27.17
19699403	2011	5	19	1690	44.79	27.17
19699403	2011	5	19	1700	44.84	27.17
19699403	2011	5	19	1710	44.64	27.17
19699403	2011	5	19	1720	44.83	27.17
19699403	2011	5	19	1730	45.26	27.17
19699403	2011	5	19	1740	45.52	27.17
19699403	2011	5	19	1750	45.63	27.16
19699403	2011	5	19	1760	45.36	27.17
19699403	2011	5	19	1770	45.18	27.17
19699403	2011	5	19	1780	45.06	27.16
19699403	2011	5	19	1790	44.98	27.17
19699403	2011	5	19	1800	44.86	27.16
19699403	2011	5	19	1810	44.82	27.16
19699403	2011	5	19	1820	45.15	27.16
19699403	2011	5	19	1830	45.46	27.16
19699403	2011	5	19	1840	45.24	27.17
19699403	2011	5	19	1850	45.16	27.16

19699403	2011	5	19	1860	45.31	27.16
19699403	2011	5	19	1870	45.29	27.15
19699403	2011	5	19	1880	45.46	27.15
19699403	2011	5	19	1890	45.31	27.16
19699403	2011	5	19	1900	45.36	27.16
19699403	2011	5	19	1910	45.42	27.15
19699403	2011	5	19	1920	45.46	27.15
19699403	2011	5	19	1930	45.39	27.16
19699403	2011	5	19	1940	45.37	27.15
19699403	2011	5	19	1950	45.38	27.15
19699403	2011	5	19	1960	45.49	27.15
19699403	2011	5	19	1970	45.74	27.14
19699403	2011	5	19	1980	46.19	27.14
19699403	2011	5	19	1990	46.72	27.14
19699403	2011	5	19	2000	46.87	27.14
19699403	2011	5	19	2010	47.09	27.14
19699403	2011	5	19	2020	47.11	27.14
19699403	2011	5	19	2030	47.29	27.13
19699403	2011	5	19	2040	47.58	27.13
19699403	2011	5	19	2050	47.92	27.12
19699403	2011	5	19	2060	47.86	27.13
19699403	2011	5	19	2070	47.67	27.13
19699403	2011	5	19	2080	47.64	27.13
19699403	2011	5	19	2090	47.77	27.13
19699403	2011	5	19	2100	47.64	27.12
19699403	2011	5	19	2110	47.89	27.13
19699403	2011	5	19	2120	48.13	27.12
19699403	2011	5	19	2130	48.39	27.12
19699403	2011	5	19	2140	47.72	27.11
19699403	2011	5	19	2150	46.87	27.12
19699403	2011	5	19	2160	46.26	27.12
19699403	2011	5	19	2170	46.22	27.13
19699403	2011	5	19	2180	46.07	27.13
19699403	2011	5	19	2190	45.75	27.13
19699403	2011	5	19	2200	46.04	27.14
19699403	2011	5	19	2210	46.58	27.13
19699403	2011	5	19	2220	47.36	27.12
19699403	2011	5	19	2230	48.09	27.11
19699403	2011	5	19	2240	48.69	27.1
19699403	2011	5	19	2250	49.95	27.1
19699403	2011	5	19	2260	50.46	27.1
19699403	2011	5	19	2270	49.15	27.1
19699403	2011	5	19	2280	49	27.1
19699403	2011	5	19	2290	49.99	27.09
19699403	2011	5	19	2300	50.83	27.09
19699403	2011	5	19	2310	51.62	27.08
19699403	2011	5	19	2320	51.8	27.08

19699403	2011	5	19	2330	51.86	27.08
19699403	2011	5	19	2340	52.34	27.08
19699403	2011	5	19	2350	52.75	27.07
19699403	2011	5	19	2360	52.43	27.05
19699403	2011	5	19	2370	52.46	27.04
19699403	2011	5	19	2380	52.28	27.05
19699403	2011	5	19	2390	52.29	27.04
19699403	2011	5	19	2400	52.49	27.04
19699403	2011	5	19	2410	52.84	27.03
19699403	2011	5	19	2420	53	27.03
19699403	2011	5	19	2430	52.81	27.03
19699403	2011	5	19	2440	52.04	27.02
19699403	2011	5	19	2450	51.11	27.02
19699403	2011	5	19	2460	50.75	27.02
19699403	2011	5	19	2470	50.79	27.02
19699403	2011	5	19	2480	51	27.01
19699403	2011	5	19	2490	51.21	27
19699403	2011	5	19	2500	51.32	27
19699403	2011	5	19	2510	52.46	26.99
19699403	2011	5	19	2520	55.22	26.96
19699403	2011	5	19	2530	58.47	26.95
19699403	2011	5	19	2540	61.54	26.9
19699403	2011	5	19	2550	64.68	26.85
19699403	2011	5	19	2560	67.9	26.77
19699403	2011	5	19	2570	70.93	26.71
19699403	2011	5	19	2580	71.96	26.67
19699403	2011	5	19	2590	72.79	26.61
19699403	2011	5	19	2600	74.55	26.56
19699403	2011	5	19	2610	76.94	26.46
19699403	2011	5	19	2620	79.47	26.34
19699403	2011	5	19	2630	82.27	26.2
19699403	2011	5	19	2640	84.13	26.1
19699403	2011	5	19	2650	84.85	25.94
19699403	2011	5	19	2660	85.32	25.85
19699403	2011	5	19	2670	85.67	25.76
19699403	2011	5	19	2680	85.96	25.63
19699403	2011	5	19	2690	86.27	25.53
19699403	2011	5	19	2700	86.53	25.47
19699403	2011	5	19	2710	86.83	25.39
19699403	2011	5	19	2720	87.06	25.32
19699403	2011	5	19	2730	86.48	25.21
19699403	2011	5	19	2740	85.97	25.1
19699403	2011	5	19	2750	85.5	25
19699403	2011	5	19	2760	85.43	24.93
19699403	2011	5	19	2770	85.55	24.79
19699403	2011	5	19	2780	85.43	24.71
19699403	2011	5	19	2790	85.44	24.66

19699403	2011	5	19	2800	85.47	24.6
19699403	2011	5	19	2810	85.49	24.56
19699403	2011	5	19	2820	85.36	24.47
19699403	2011	5	19	2830	85.33	24.41
19699403	2011	5	19	2840	85.35	24.35
19699403	2011	5	19	2850	85.28	24.31
19699403	2011	5	19	2860	85.27	24.26
19699403	2011	5	19	2870	85.27	24.21
19699403	2011	5	19	2880	85.22	24.17
19699403	2011	5	19	2890	85.18	24.1
19699403	2011	5	19	2900	85.21	24.07
19699403	2011	5	19	2910	85.17	24
19699403	2011	5	19	2920	85.06	23.97
19699403	2011	5	19	2930	84.96	23.93
19699403	2011	5	19	2940	84.88	23.91
19699403	2011	5	19	2950	84.83	23.89
19699403	2011	5	19	2960	84.81	23.85
19699403	2011	5	19	2970	84.77	23.82
19699403	2011	5	19	2980	84.78	23.78
19699403	2011	5	19	2990	85.88	23.73
19699403	2011	5	19	3000	86.67	23.69
19699403	2011	5	19	3010	87.4	23.65
19699403	2011	5	19	3020	88.41	23.6
19699403	2011	5	19	3030	90.17	23.46
19699403	2011	5	19	3040	91.38	23.42
19699403	2011	5	19	3050	91.69	23.39
19699403	2011	5	19	3060	91.72	23.28
19699403	2011	5	19	3070	93.35	23.14
19699403	2011	5	19	3080	95.89	23.07
19699403	2011	5	19	3090	96.97	23.02
19699403	2011	5	19	3100	97.63	22.99
19699403	2011	5	19	3110	99.41	22.87
19699403	2011	5	19	3120	101.49	22.78
19699403	2011	5	19	3130	103.68	22.65
19699403	2011	5	19	3140	106.16	22.55
19699403	2011	5	19	3150	108.51	22.46
19699403	2011	5	19	3160	109.55	22.42
19699403	2011	5	19	3170	110.94	22.39
19699403	2011	5	19	3180	112.84	22.28
19699403	2011	5	19	3190	115.17	22.11
19699403	2011	5	19	3200	117.47	21.85
19699403	2011	5	19	3210	118.51	21.8
19699403	2011	5	19	3220	119.4	21.8
19699403	2011	5	19	3230	120.51	21.63
19699403	2011	5	19	3240	121.01	21.57
19699403	2011	5	19	3250	121.14	21.45
19699403	2011	5	19	3260	122.24	21.35

19699403	2011	5	19	3270	124.14	21.21
19699403	2011	5	19	3280	124.82	21.17
19699403	2011	5	19	3290	125.02	21.14
19699403	2011	5	19	3300	125.16	21.08
19699403	2011	5	19	3310	125.41	21
19699403	2011	5	19	3320	126.54	20.92
19699403	2011	5	19	3330	127.89	20.83
19699403	2011	5	19	3340	128.41	20.81
19699403	2011	5	19	3350	129.78	20.77
19699403	2011	5	19	3360	131.43	20.71
19699403	2011	5	19	3370	132.1	20.7
19699403	2011	5	19	3380	132.97	20.64
19699403	2011	5	19	3390	133.98	20.6
19699403	2011	5	19	3400	135.5	20.57
19699403	2011	5	19	3410	136.79	20.52
19699403	2011	5	19	3420	136.02	20.49
19699403	2011	5	19	3430	135.84	20.46
19699403	2011	5	19	3440	136.02	20.41
19699403	2011	5	19	3450	135.99	20.38
19699403	2011	5	19	3460	136	20.35
19699403	2011	5	19	3470	135.98	20.3
19699403	2011	5	19	3480	135.87	20.27
19699403	2011	5	19	3490	135.82	20.22
19699403	2011	5	19	3500	135.92	20.19
19699403	2011	5	19	3510	136.04	20.17
19699403	2011	5	19	3520	135.99	20.12
19699403	2011	5	19	3530	135.94	20.08
19699403	2011	5	19	3540	135.95	20.02
19699403	2011	5	19	3550	135.96	20
19699403	2011	5	19	3560	135.97	19.96
19699403	2011	5	19	3570	135.99	19.95
19699403	2011	5	19	3580	135.99	19.92
19699403	2011	5	19	3590	135.98	19.9
19699403	2011	5	19	3600	135.9	19.86
19699403	2011	5	19	3610	135.86	19.82
19699403	2011	5	19	3620	135.86	19.76
19699403	2011	5	19	3630	135.81	19.71
19699403	2011	5	19	3640	135.42	19.67
19699403	2011	5	19	3650	133.77	19.66
19699403	2011	5	19	3660	131.53	19.64
19699403	2011	5	19	3670	129.25	19.64
19699403	2011	5	19	3680	127.02	19.64
19699403	2011	5	19	3690	126.34	19.66
19699403	2011	5	19	3700	126.24	19.67
19699403	2011	5	19	3710	126.17	19.67
19699403	2011	5	19	3720	125.81	19.68
19699403	2011	5	19	3730	125.54	19.68

19699403	2011	5	19	3740	125.28	19.69
19699403	2011	5	19	3750	125.11	19.7
19699403	2011	5	19	3760	124.95	19.7
19699403	2011	5	19	3770	124.98	19.73
19699403	2011	5	19	3780	125.11	19.74
19699403	2011	5	19	3790	124.34	19.75
19699403	2011	5	19	3800	122.45	19.77
19699403	2011	5	19	3810	120.4	19.78
19699403	2011	5	19	3820	119.94	19.79
19699403	2011	5	19	3830	119.79	19.8
19699403	2011	5	19	3840	118.49	19.81
19699403	2011	5	19	3850	116.93	19.82
19699403	2011	5	19	3860	114.7	19.83
19699403	2011	5	19	3870	112.12	19.85
19699403	2011	5	19	3880	109.92	19.87
19699403	2011	5	19	3890	107.9	19.9
19699403	2011	5	19	3900	107.01	19.92
19699403	2011	5	19	3910	105.59	19.95
19699403	2011	5	19	3920	103.28	20
19699403	2011	5	19	3930	101.2	20.05
19699403	2011	5	19	3940	99.24	20.17
19699403	2011	5	19	3950	97.26	20.25
19699403	2011	5	19	3960	95.47	20.32
19699403	2011	5	19	3970	93.69	20.39
19699403	2011	5	19	3980	91.7	20.46
19699403	2011	5	19	3990	89.42	20.53
19699403	2011	5	19	4000	86.98	20.64
19699403	2011	5	19	4010	84.36	20.74
19699403	2011	5	19	4020	81.62	20.86
19699403	2011	5	19	4030	79.58	21
19699403	2011	5	19	4040	78.73	21.1
19699403	2011	5	19	4050	76.75	21.21
19699403	2011	5	19	4060	74.25	21.35
19699403	2011	5	19	4070	72.31	21.51
19699403	2011	5	19	4080	72.03	21.67
19699403	2011	5	19	4090	71.48	21.85
19699403	2011	5	19	4100	70.41	21.98
19699403	2011	5	19	4110	69.49	22.15
19699403	2011	5	19	4120	68.47	22.33
19699403	2011	5	19	4130	67.21	22.49
19699403	2011	5	19	4140	66.07	22.63
19699403	2011	5	19	4150	64.19	22.78
19699403	2011	5	19	4160	62.37	22.95
19699403	2011	5	19	4170	61.35	23.11
19699403	2011	5	19	4180	61.59	23.32
19699403	2011	5	19	4190	60.58	23.49
19699403	2011	5	19	4200	58.47	23.64

19699403	2011	5	19	4210	55.95	23.77
19699403	2011	5	19	4220	54.91	23.88
19699403	2011	5	19	4230	54.61	24.05
19699403	2011	5	19	4240	53.79	24.17
19699403	2011	5	19	4250	52.25	24.28
19699403	2011	5	19	4260	50.09	24.4
19699403	2011	5	19	4270	48.82	24.5
19699403	2011	5	19	4280	49.29	24.71
19699403	2011	5	19	4290	49.45	24.84
19699403	2011	5	19	4300	49.29	25
19699403	2011	5	19	4310	47.75	25.1
19699403	2011	5	19	4320	46.11	25.19
19699403	2011	5	19	4330	44.28	25.29
19699403	2011	5	19	4340	42.98	25.38
19699403	2011	5	19	4350	42.47	25.46
19699403	2011	5	19	4360	40.77	25.57
19699403	2011	5	19	4370	38.85	25.66
19699403	2011	5	19	4380	36.84	25.77
19699403	2011	5	19	4390	35.18	25.84
19699403	2011	5	19	4400	34.64	25.92
19699403	2011	5	19	4410	34.8	26
19699403	2011	5	19	4420	34.75	26.11
19699403	2011	5	19	4430	34.9	26.16
19699403	2011	5	19	4440	35.51	26.21
19699403	2011	5	19	4450	36.04	26.28
19699403	2011	5	19	4460	36.04	26.31
19699403	2011	5	19	4470	35.89	26.35
19699403	2011	5	19	4480	36.02	26.39
19699403	2011	5	19	4490	35.89	26.42
19699403	2011	5	19	4500	35.19	26.47
19699403	2011	5	19	4510	35.13	26.51
19699403	2011	5	19	4520	35.27	26.54
19699403	2011	5	19	4530	34.3	26.6
19699403	2011	5	19	4540	34.16	26.64
19699403	2011	5	19	4550	34.32	26.67
19699403	2011	5	19	4560	34.17	26.71
19699403	2011	5	19	4570	33.87	26.74
19699403	2011	5	19	4580	33.83	26.78
19699403	2011	5	19	4590	33.88	26.8
19699403	2011	5	19	4600	34.05	26.83
19699403	2011	5	19	4610	33.97	26.85
19699403	2011	5	19	4620	33.87	26.89
19699403	2011	5	19	4630	33.87	26.91
19699403	2011	5	19	4640	33.85	26.95
19699403	2011	5	19	4650	33.3	26.96
19699403	2011	5	19	4660	33.02	26.99
19699403	2011	5	19	4670	31.65	27.01

19699403	2011	5	19	4680	30.12	27.04
19699403	2011	5	19	4690	28.66	27.07
19699403	2011	5	19	4700	28.74	27.09
19699403	2011	5	19	4710	28.86	27.1
19699403	2011	5	19	4720	28.77	27.1
19699403	2011	5	19	4730	28.75	27.14
19699403	2011	5	19	4740	28.71	27.14
19699403	2011	5	19	4750	28.81	27.16
19699403	2011	5	19	4760	28.66	27.17
19699403	2011	5	19	4770	28.73	27.17
19699403	2011	5	19	4780	28.69	27.19
19699403	2011	5	19	4790	28.79	27.2
19699403	2011	5	19	4800	28.75	27.22
19699403	2011	5	19	4810	28.69	27.22
19699403	2011	5	19	4820	28.8	27.24
19699403	2011	5	19	4830	28.79	27.24
19699403	2011	5	19	4840	28.8	27.25
19699403	2011	5	19	4850	28.79	27.25
19699403	2011	5	19	4860	28.92	27.25
19699403	2011	5	19	4870	28.72	27.26
19699403	2011	5	19	4880	28.88	27.27
19699403	2011	5	19	4890	28.85	27.28
19699403	2011	5	19	4900	28.79	27.28
19699403	2011	5	19	4910	28.72	27.29
19699403	2011	5	19	4920	28.82	27.29
19699403	2011	5	19	4930	28.66	27.31
19699403	2011	5	19	4940	28.74	27.3
19699403	2011	5	19	4950	28.64	27.32
19699403	2011	5	19	4960	27.18	27.33
19699403	2011	5	19	4970	25.51	27.35
19699403	2011	5	19	4980	25.02	27.35
19699403	2011	5	19	4990	24.86	27.36
19699403	2011	5	19	5000	24.06	27.38
19699403	2011	5	19	5010	24.22	27.38
19699403	2011	5	19	5020	24.64	27.39
19699403	2011	5	19	5030	24.81	27.39
19699403	2011	5	19	5040	24.72	27.39
19699403	2011	5	19	5050	24.73	27.39
19699403	2011	5	19	5060	24.85	27.39
19699403	2011	5	19	5070	24.69	27.4
19699403	2011	5	19	5080	24.16	27.41
19699403	2011	5	19	5090	24.23	27.41
19699403	2011	5	19	5100	24.54	27.4
19699403	2011	5	19	5110	24.06	27.41
19699403	2011	5	19	5120	24.06	27.42
19699403	2011	5	19	5130	24.26	27.42
19699403	2011	5	19	5140	24.23	27.42

19699403	2011	5	19	5150	24.19	27.42
19699403	2011	5	19	5160	24.08	27.42
19699403	2011	5	19	5170	24.18	27.43
19699403	2011	5	19	5180	24.14	27.42
19699403	2011	5	19	5190	24.05	27.43
19699403	2011	5	19	5200	24.09	27.44
19699403	2011	5	19	5210	24.05	27.45
19699403	2011	5	19	5220	24.09	27.46
19699403	2011	5	19	5230	24.06	27.46
19699403	2011	5	19	5240	24.05	27.46
19699403	2011	5	19	5250	24.15	27.46
19699403	2011	5	19	5260	24.05	27.46
19699403	2011	5	19	5270	23.64	27.48
19699403	2011	5	19	5280	22.07	27.49
19699403	2011	5	19	5290	20.64	27.5
19699403	2011	5	19	5300	19.87	27.51
19699403	2011	5	19	5310	19.12	27.52
19699403	2011	5	19	5320	18.88	27.52
19699403	2011	5	19	5330	19.23	27.53
19699403	2011	5	19	5340	18.95	27.54
19699403	2011	5	19	5350	17.17	27.55
19699403	2011	5	19	5360	15.27	27.57
19699403	2011	5	19	5370	13.75	27.6
19699403	2011	5	19	5380	12.7	27.64
19699403	2011	5	19	5390	10.63	27.69
19699403	2011	5	19	5400	8.24	27.75
19699403	2011	5	19	5410	6.46	27.82
19699403	2011	5	19	5420	4.77	27.88
19699403	2011	5	19	5430	3.58	27.93
19699403	2011	5	19	5440	3.08	27.98
19699403	2011	5	19	5450	2.93	28.01
19699403	2011	5	19	5460	2.24	28.07
19699403	2011	5	19	5470	2.3	28.09
19699403	2011	5	19	5480	2.79	28.12
19699403	2011	5	19	5490	3	28.17
19699403	2011	5	19	5500	2.13	28.21
19699403	2011	5	19	5510	0.59	28.32
19699403	2011	5	19	5520	0.59	28.35
19699403	2011	5	19	5530	0.6	28.37
19699403	2011	5	19	5540	0.6	28.41
19699403	2011	5	19	5550	0.58	28.5
19699403	2011	5	19	5560	0.59	28.53
19699403	2011	5	19	5570	0.58	28.58
19699403	2011	5	19	5580	0.59	28.61
19699403	2011	5	19	5590	0.58	28.65
19699403	2011	5	19	5600	0.57	28.68
19699403	2011	5	19	5610	0.53	28.71

19699403	2011	5	19	5620	0.54	28.75
19699403	2011	5	19	5630	0.51	28.77
19699403	2011	5	19	5640	0.54	28.79
19699403	2011	5	19	5650	0.53	28.82
19779043	2011	5	20	0	1.2	28.76
19779043	2011	5	20	10	1.54	28.75
19779043	2011	5	20	20	1.45	28.75
19779043	2011	5	20	30	1.42	28.75
19779043	2011	5	20	40	2.14	28.75
19779043	2011	5	20	50	2.44	28.75
19779043	2011	5	20	60	2.67	28.75
19779043	2011	5	20	70	2.79	28.75
19779043	2011	5	20	80	2.99	28.75
19779043	2011	5	20	90	3.39	28.74
19779043	2011	5	20	100	4.62	28.71
19779043	2011	5	20	110	6.58	28.68
19779043	2011	5	20	120	8.86	28.65
19779043	2011	5	20	130	11.19	28.62
19779043	2011	5	20	140	12.61	28.6
19779043	2011	5	20	150	12.8	28.59
19779043	2011	5	20	160	13.21	28.59
19779043	2011	5	20	170	13.64	28.57
19779043	2011	5	20	180	13.66	28.57
19779043	2011	5	20	190	13.79	28.57
19779043	2011	5	20	200	13.82	28.57
19779043	2011	5	20	210	13.78	28.56
19779043	2011	5	20	220	13.99	28.56
19779043	2011	5	20	230	14.23	28.54
19779043	2011	5	20	240	14.55	28.53
19779043	2011	5	20	250	14.75	28.53
19779043	2011	5	20	260	14.51	28.53
19779043	2011	5	20	270	14.41	28.53
19779043	2011	5	20	280	14.43	28.52
19779043	2011	5	20	290	14.67	28.51
19779043	2011	5	20	300	14.96	28.51
19779043	2011	5	20	310	14.91	28.51
19779043	2011	5	20	320	14.5	28.5
19779043	2011	5	20	330	14.58	28.5
19779043	2011	5	20	340	15.94	28.49
19779043	2011	5	20	350	17.06	28.47
19779043	2011	5	20	360	17.94	28.46
19779043	2011	5	20	370	19.08	28.44
19779043	2011	5	20	380	19.67	28.44
19779043	2011	5	20	390	21.11	28.41
19779043	2011	5	20	400	23.39	28.38
19779043	2011	5	20	410	25.96	28.32
19779043	2011	5	20	420	28.53	28.28

19779043	2011	5	20	430	31.2	28.23
19779043	2011	5	20	440	33.64	28.18
19779043	2011	5	20	450	36.24	28.13
19779043	2011	5	20	460	38.41	28.08
19779043	2011	5	20	470	39.32	28.05
19779043	2011	5	20	480	40.14	28.03
19779043	2011	5	20	490	41.17	27.98
19779043	2011	5	20	500	42.48	27.93
19779043	2011	5	20	510	44.64	27.88
19779043	2011	5	20	520	45.97	27.85
19779043	2011	5	20	530	46.92	27.82
19779043	2011	5	20	540	48.5	27.74
19779043	2011	5	20	550	50.11	27.69
19779043	2011	5	20	560	52.5	27.62
19779043	2011	5	20	570	55.25	27.55
19779043	2011	5	20	580	57.21	27.5
19779043	2011	5	20	590	57.98	27.48
19779043	2011	5	20	600	58.5	27.46
19779043	2011	5	20	610	59.43	27.42
19779043	2011	5	20	620	61.31	27.32
19779043	2011	5	20	630	63.5	27.24
19779043	2011	5	20	640	65.34	27.18
19779043	2011	5	20	650	67.22	27.12
19779043	2011	5	20	660	69.31	27.09
19779043	2011	5	20	670	71.19	27.03
19779043	2011	5	20	680	73.32	26.99
19779043	2011	5	20	690	74.35	26.95
19779043	2011	5	20	700	75.65	26.91
19779043	2011	5	20	710	77.41	26.82
19779043	2011	5	20	720	79.85	26.74
19779043	2011	5	20	730	82.46	26.69
19779043	2011	5	20	740	83.42	26.66
19779043	2011	5	20	750	83.91	26.61
19779043	2011	5	20	760	84.38	26.6
19779043	2011	5	20	770	84.83	26.57
19779043	2011	5	20	780	85.27	26.53
19779043	2011	5	20	790	85.66	26.5
19779043	2011	5	20	800	85.92	26.45
19779043	2011	5	20	810	86.27	26.4
19779043	2011	5	20	820	86.56	26.35
19779043	2011	5	20	830	86.86	26.32
19779043	2011	5	20	840	87.2	26.28
19779043	2011	5	20	850	87.58	26.21
19779043	2011	5	20	860	88.06	26.17
19779043	2011	5	20	870	89.13	26.13
19779043	2011	5	20	880	90.8	26.03
19779043	2011	5	20	890	92.96	25.81

19779043	2011	5	20	900	95.14	25.62
19779043	2011	5	20	910	96.71	25.57
19779043	2011	5	20	920	98.06	25.51
19779043	2011	5	20	930	99.5	25.38
19779043	2011	5	20	940	100.9	25.2
19779043	2011	5	20	950	102.94	25.11
19779043	2011	5	20	960	104.56	25.01
19779043	2011	5	20	970	105.83	24.92
19779043	2011	5	20	980	107.55	24.7
19779043	2011	5	20	990	107.89	24.53
19779043	2011	5	20	1000	107.9	24.42
19779043	2011	5	20	1010	107.77	24.28
19779043	2011	5	20	1020	107.48	24.19
19779043	2011	5	20	1030	107.64	24.11
19779043	2011	5	20	1040	108.01	23.95
19779043	2011	5	20	1050	108.22	23.86
19779043	2011	5	20	1060	108.39	23.8
19779043	2011	5	20	1070	110.23	23.71
19779043	2011	5	20	1080	111.57	23.62
19779043	2011	5	20	1090	113.19	23.53
19779043	2011	5	20	1100	114.26	23.44
19779043	2011	5	20	1110	115.57	23.34
19779043	2011	5	20	1120	117.75	23.18
19779043	2011	5	20	1130	119.41	23.1
19779043	2011	5	20	1140	120.46	23.02
19779043	2011	5	20	1150	121.3	22.94
19779043	2011	5	20	1160	121.75	22.83
19779043	2011	5	20	1170	122.87	22.73
19779043	2011	5	20	1180	124.66	22.6
19779043	2011	5	20	1190	126.94	22.42
19779043	2011	5	20	1200	128.19	22.3
19779043	2011	5	20	1210	128.62	22.2
19779043	2011	5	20	1220	130.34	22.1
19779043	2011	5	20	1230	131.76	21.96
19779043	2011	5	20	1240	132.94	21.83
19779043	2011	5	20	1250	134.53	21.56
19779043	2011	5	20	1260	135.26	21.39
19779043	2011	5	20	1270	136.61	21.31
19779043	2011	5	20	1280	138.01	21.2
19779043	2011	5	20	1290	138.71	21.08
19779043	2011	5	20	1300	138.89	20.96
19779043	2011	5	20	1310	138.9	20.85
19779043	2011	5	20	1320	139.42	20.69
19779043	2011	5	20	1330	139.49	20.58
19779043	2011	5	20	1340	139.33	20.51
19779043	2011	5	20	1350	139.36	20.39
19779043	2011	5	20	1360	139.21	20.32

19779043	2011	5	20	1370	139.41	20.26
19779043	2011	5	20	1380	140.11	20.18
19779043	2011	5	20	1390	141.62	19.96
19779043	2011	5	20	1400	142.56	19.93
19779043	2011	5	20	1410	143.35	19.86
19779043	2011	5	20	1420	143.34	19.79
19779043	2011	5	20	1430	143.51	19.75
19779043	2011	5	20	1440	144.55	19.68
19779043	2011	5	20	1450	145.27	19.64
19779043	2011	5	20	1460	146.69	19.57
19779043	2011	5	20	1470	147.53	19.5
19779043	2011	5	20	1480	147.57	19.47
19779043	2011	5	20	1490	147.45	19.44
19779043	2011	5	20	1500	147.75	19.41
19779043	2011	5	20	1510	147.83	19.37
19779043	2011	5	20	1520	147.74	19.34
19779043	2011	5	20	1530	147.64	19.31
19779043	2011	5	20	1540	148.1	19.24
19779043	2011	5	20	1550	148.44	19.18
19779043	2011	5	20	1560	148.56	19.16
19779043	2011	5	20	1570	148.78	19.13
19779043	2011	5	20	1580	148.85	19.1
19779043	2011	5	20	1590	148.9	19.07
19779043	2011	5	20	1600	149.48	19
19779043	2011	5	20	1610	150.12	18.92
19779043	2011	5	20	1620	151.48	18.87
19779043	2011	5	20	1630	153.31	18.82
19779043	2011	5	20	1640	154.15	18.79
19779043	2011	5	20	1650	154.51	18.8
19779043	2011	5	20	1660	155.64	18.76
19779043	2011	5	20	1670	155.86	18.75
19779043	2011	5	20	1680	155.59	18.73
19779043	2011	5	20	1690	156.45	18.7
19779043	2011	5	20	1700	156.53	18.66
19779043	2011	5	20	1710	156.48	18.64
19779043	2011	5	20	1720	156.56	18.59
19779043	2011	5	20	1730	156.4	18.54
19779043	2011	5	20	1740	156.07	18.52
19779043	2011	5	20	1750	156.23	18.49
19779043	2011	5	20	1760	156.2	18.46
19779043	2011	5	20	1770	156.02	18.43
19779043	2011	5	20	1780	156.98	18.4
19779043	2011	5	20	1790	157.47	18.38
19779043	2011	5	20	1800	157.64	18.35
19779043	2011	5	20	1810	159.2	18.31
19779043	2011	5	20	1820	160.01	18.25
19779043	2011	5	20	1830	160.47	18.23

19779043	2011	5	20	1840	160.66	18.21
19779043	2011	5	20	1850	160.79	18.2
19779043	2011	5	20	1860	161.35	18.17
19779043	2011	5	20	1870	162.77	18.03
19779043	2011	5	20	1880	164.84	17.79
19779043	2011	5	20	1890	166.16	17.79
19779043	2011	5	20	1900	167.51	17.75
19779043	2011	5	20	1910	168.02	17.75
19779043	2011	5	20	1920	167.98	17.75
19779043	2011	5	20	1930	168.56	17.73
19779043	2011	5	20	1940	169.4	17.7
19779043	2011	5	20	1950	170.53	17.59
19779043	2011	5	20	1960	171.63	17.47
19779043	2011	5	20	1970	172.69	17.39
19779043	2011	5	20	1980	173.47	17.38
19779043	2011	5	20	1990	173.82	17.39
19779043	2011	5	20	2000	173.98	17.39
19779043	2011	5	20	2010	174.17	17.38
19779043	2011	5	20	2020	174.19	17.36
19779043	2011	5	20	2030	173.99	17.35
19779043	2011	5	20	2040	173.82	17.32
19779043	2011	5	20	2050	173.9	17.31
19779043	2011	5	20	2060	173.93	17.26
19779043	2011	5	20	2070	174.22	17.24
19779043	2011	5	20	2080	174.83	17.21
19779043	2011	5	20	2090	175.08	17.18
19779043	2011	5	20	2100	175.16	17.16
19779043	2011	5	20	2110	175.57	17.14
19779043	2011	5	20	2120	175.66	17.09
19779043	2011	5	20	2130	175.66	17.07
19779043	2011	5	20	2140	175.81	17.03
19779043	2011	5	20	2150	175.68	17
19779043	2011	5	20	2160	175.28	16.97
19779043	2011	5	20	2170	174.97	16.92
19779043	2011	5	20	2180	175.65	16.89
19779043	2011	5	20	2190	175.76	16.86
19779043	2011	5	20	2200	175.75	16.85
19779043	2011	5	20	2210	176.09	16.82
19779043	2011	5	20	2220	176.68	16.8
19779043	2011	5	20	2230	177.72	16.78
19779043	2011	5	20	2240	177.84	16.76
19779043	2011	5	20	2250	178.38	16.75
19779043	2011	5	20	2260	178.8	16.72
19779043	2011	5	20	2270	179.47	16.7
19779043	2011	5	20	2280	179.95	16.69
19779043	2011	5	20	2290	180.05	16.68
19779043	2011	5	20	2300	180.23	16.67

19779043	2011	5	20	2310	181.1	16.65
19779043	2011	5	20	2320	181.52	16.64
19779043	2011	5	20	2330	181.56	16.64
19779043	2011	5	20	2340	181.4	16.63
19779043	2011	5	20	2350	181.36	16.63
19779043	2011	5	20	2360	181.47	16.62
19779043	2011	5	20	2370	181.45	16.62
19779043	2011	5	20	2380	181.47	16.6
19779043	2011	5	20	2390	181.4	16.6
19779043	2011	5	20	2400	181.82	16.59
19779043	2011	5	20	2410	181.46	16.58
19779043	2011	5	20	2420	180.78	16.57
19779043	2011	5	20	2430	179.78	16.57
19779043	2011	5	20	2440	179.96	16.57
19779043	2011	5	20	2450	180.48	16.57
19779043	2011	5	20	2460	180.38	16.57
19779043	2011	5	20	2470	179.79	16.57
19779043	2011	5	20	2480	178.2	16.57
19779043	2011	5	20	2490	177.43	16.58
19779043	2011	5	20	2500	177.36	16.58
19779043	2011	5	20	2510	177.23	16.58
19779043	2011	5	20	2520	176.15	16.58
19779043	2011	5	20	2530	173.95	16.59
19779043	2011	5	20	2540	171.73	16.59
19779043	2011	5	20	2550	170.53	16.6
19779043	2011	5	20	2560	169.21	16.6
19779043	2011	5	20	2570	167.54	16.6
19779043	2011	5	20	2580	165.83	16.62
19779043	2011	5	20	2590	165.15	16.63
19779043	2011	5	20	2600	164.83	16.64
19779043	2011	5	20	2610	164.47	16.67
19779043	2011	5	20	2620	162.69	16.7
19779043	2011	5	20	2630	160.43	16.72
19779043	2011	5	20	2640	158.43	16.75
19779043	2011	5	20	2650	156.25	16.78
19779043	2011	5	20	2660	155.41	16.82
19779043	2011	5	20	2670	153.96	16.89
19779043	2011	5	20	2680	152.3	16.9
19779043	2011	5	20	2690	150.03	16.97
19779043	2011	5	20	2700	147.27	17.03
19779043	2011	5	20	2710	145.09	17.09
19779043	2011	5	20	2720	143.06	17.17
19779043	2011	5	20	2730	142.07	17.23
19779043	2011	5	20	2740	140.52	17.29
19779043	2011	5	20	2750	139.08	17.34
19779043	2011	5	20	2760	138.4	17.41
19779043	2011	5	20	2770	137.43	17.48

19779043	2011	5	20	2780	134.96	17.56
19779043	2011	5	20	2790	132.71	17.6
19779043	2011	5	20	2800	131.61	17.65
19779043	2011	5	20	2810	129.3	17.71
19779043	2011	5	20	2820	128.13	17.76
19779043	2011	5	20	2830	127.24	17.82
19779043	2011	5	20	2840	126.52	17.86
19779043	2011	5	20	2850	127.21	17.9
19779043	2011	5	20	2860	127.69	17.96
19779043	2011	5	20	2870	128.17	18.03
19779043	2011	5	20	2880	128.35	18.09
19779043	2011	5	20	2890	128.58	18.15
19779043	2011	5	20	2900	128.51	18.2
19779043	2011	5	20	2910	128.17	18.25
19779043	2011	5	20	2920	126.87	18.31
19779043	2011	5	20	2930	124.67	18.35
19779043	2011	5	20	2940	122.16	18.41
19779043	2011	5	20	2950	119.6	18.49
19779043	2011	5	20	2960	117.21	18.6
19779043	2011	5	20	2970	114.69	18.65
19779043	2011	5	20	2980	112.35	18.71
19779043	2011	5	20	2990	111.13	18.78
19779043	2011	5	20	3000	110.72	18.96
19779043	2011	5	20	3010	109.93	19.05
19779043	2011	5	20	3020	108.13	19.14
19779043	2011	5	20	3030	105.89	19.25
19779043	2011	5	20	3040	104.1	19.54
19779043	2011	5	20	3050	102.33	19.69
19779043	2011	5	20	3060	100.9	19.75
19779043	2011	5	20	3070	100.69	19.82
19779043	2011	5	20	3080	100.39	19.95
19779043	2011	5	20	3090	98.73	20.08
19779043	2011	5	20	3100	97.18	20.15
19779043	2011	5	20	3110	95.7	20.4
19779043	2011	5	20	3120	95.18	20.48
19779043	2011	5	20	3130	94.3	20.52
19779043	2011	5	20	3140	93.42	20.59
19779043	2011	5	20	3150	91.84	20.71
19779043	2011	5	20	3160	89.93	20.8
19779043	2011	5	20	3170	88.19	20.89
19779043	2011	5	20	3180	86.75	21.02
19779043	2011	5	20	3190	86.37	21.17
19779043	2011	5	20	3200	85.85	21.31
19779043	2011	5	20	3210	84.46	21.44
19779043	2011	5	20	3220	83.72	21.59
19779043	2011	5	20	3230	83.9	21.72
19779043	2011	5	20	3240	84.25	21.86

19779043	2011	5	20	3250	83.66	22.09
19779043	2011	5	20	3260	83.23	22.24
19779043	2011	5	20	3270	82.74	22.37
19779043	2011	5	20	3280	81.67	22.51
19779043	2011	5	20	3290	81.08	22.64
19779043	2011	5	20	3300	79.54	22.78
19779043	2011	5	20	3310	77.92	22.9
19779043	2011	5	20	3320	76.26	23.1
19779043	2011	5	20	3330	74.35	23.21
19779043	2011	5	20	3340	72.03	23.32
19779043	2011	5	20	3350	70.65	23.44
19779043	2011	5	20	3360	69.57	23.57
19779043	2011	5	20	3370	67.76	23.74
19779043	2011	5	20	3380	65.59	23.88
19779043	2011	5	20	3390	63.27	23.96
19779043	2011	5	20	3400	60.8	24.09
19779043	2011	5	20	3410	58.05	24.21
19779043	2011	5	20	3420	56.17	24.38
19779043	2011	5	20	3430	53.63	24.5
19779043	2011	5	20	3440	51.98	24.6
19779043	2011	5	20	3450	52.3	24.71
19779043	2011	5	20	3460	52.28	24.79
19779043	2011	5	20	3470	51.97	24.91
19779043	2011	5	20	3480	51.77	24.98
19779043	2011	5	20	3490	51.65	25.04
19779043	2011	5	20	3500	51.77	25.13
19779043	2011	5	20	3510	52	25.22
19779043	2011	5	20	3520	52.94	25.35
19779043	2011	5	20	3530	52.25	25.41
19779043	2011	5	20	3540	51.57	25.47
19779043	2011	5	20	3550	51.54	25.52
19779043	2011	5	20	3560	51.67	25.57
19779043	2011	5	20	3570	51.64	25.6
19779043	2011	5	20	3580	51.4	25.67
19779043	2011	5	20	3590	51.4	25.75
19779043	2011	5	20	3600	51.12	25.8
19779043	2011	5	20	3610	51.41	25.9
19779043	2011	5	20	3620	51.77	25.95
19779043	2011	5	20	3630	51.57	26
19779043	2011	5	20	3640	50.29	26.05
19779043	2011	5	20	3650	49.74	26.06
19779043	2011	5	20	3660	48.5	26.08
19779043	2011	5	20	3670	46.73	26.13
19779043	2011	5	20	3680	46.38	26.16
19779043	2011	5	20	3690	46.73	26.21
19779043	2011	5	20	3700	47.22	26.25
19779043	2011	5	20	3710	47.13	26.28

19779043	2011	5	20	3720	46.83	26.32
19779043	2011	5	20	3730	46.95	26.37
19779043	2011	5	20	3740	47.14	26.39
19779043	2011	5	20	3750	47.42	26.41
19779043	2011	5	20	3760	47.55	26.43
19779043	2011	5	20	3770	47.35	26.47
19779043	2011	5	20	3780	47.36	26.48
19779043	2011	5	20	3790	47.24	26.5
19779043	2011	5	20	3800	47.18	26.53
19779043	2011	5	20	3810	47.06	26.56
19779043	2011	5	20	3820	46.27	26.59
19779043	2011	5	20	3830	45.92	26.6
19779043	2011	5	20	3840	45.77	26.63
19779043	2011	5	20	3850	45.68	26.64
19779043	2011	5	20	3860	45.64	26.66
19779043	2011	5	20	3870	45.82	26.68
19779043	2011	5	20	3880	45.98	26.69
19779043	2011	5	20	3890	45.82	26.71
19779043	2011	5	20	3900	45.97	26.71
19779043	2011	5	20	3910	46.24	26.72
19779043	2011	5	20	3920	46.24	26.73
19779043	2011	5	20	3930	46.09	26.74
19779043	2011	5	20	3940	44.67	26.76
19779043	2011	5	20	3950	42.71	26.8
19779043	2011	5	20	3960	40.78	26.82
19779043	2011	5	20	3970	40.22	26.83
19779043	2011	5	20	3980	39.51	26.85
19779043	2011	5	20	3990	38.38	26.86
19779043	2011	5	20	4000	36.89	26.89
19779043	2011	5	20	4010	36.48	26.91
19779043	2011	5	20	4020	36.62	26.93
19779043	2011	5	20	4030	37.25	26.94
19779043	2011	5	20	4040	38.05	26.95
19779043	2011	5	20	4050	38.9	26.96
19779043	2011	5	20	4060	40.62	26.98
19779043	2011	5	20	4070	41.52	27
19779043	2011	5	20	4080	42.24	27
19779043	2011	5	20	4090	42.86	27
19779043	2011	5	20	4100	44.6	27.01
19779043	2011	5	20	4110	45.34	27
19779043	2011	5	20	4120	45.79	27
19779043	2011	5	20	4130	46.4	27
19779043	2011	5	20	4140	48.15	26.96
19779043	2011	5	20	4150	49.29	26.95
19779043	2011	5	20	4160	50.48	26.95
19779043	2011	5	20	4170	51.69	26.93
19779043	2011	5	20	4180	52.32	26.92

19779043	2011	5	20	4190	52.5	26.92
19779043	2011	5	20	4200	53	26.91
19779043	2011	5	20	4210	53.28	26.89
19779043	2011	5	20	4220	53.84	26.89
19779043	2011	5	20	4230	54.07	26.88
19779043	2011	5	20	4240	54.31	26.85
19779043	2011	5	20	4250	54.25	26.84
19779043	2011	5	20	4260	54.31	26.82
19779043	2011	5	20	4270	54.2	26.82
19779043	2011	5	20	4280	53.83	26.79
19779043	2011	5	20	4290	54.02	26.78
19779043	2011	5	20	4300	53.66	26.75
19779043	2011	5	20	4310	52.36	26.75
19779043	2011	5	20	4320	50.44	26.75
19779043	2011	5	20	4330	48.42	26.76
19779043	2011	5	20	4340	46.31	26.78
19779043	2011	5	20	4350	44.29	26.8
19779043	2011	5	20	4360	42.19	26.82
19779043	2011	5	20	4370	40.24	26.84
19779043	2011	5	20	4380	38.05	26.87
19779043	2011	5	20	4390	35.62	26.89
19779043	2011	5	20	4400	33.88	26.94
19779043	2011	5	20	4410	33.47	26.95
19779043	2011	5	20	4420	33.06	26.97
19779043	2011	5	20	4430	31.97	27
19779043	2011	5	20	4440	30.21	27.02
19779043	2011	5	20	4450	28.32	27.06
19779043	2011	5	20	4460	27.42	27.07
19779043	2011	5	20	4470	27.81	27.09
19779043	2011	5	20	4480	27.44	27.11
19779043	2011	5	20	4490	26.92	27.13
19779043	2011	5	20	4500	27.12	27.14
19779043	2011	5	20	4510	26.16	27.17
19779043	2011	5	20	4520	24.54	27.2
19779043	2011	5	20	4530	22.79	27.22
19779043	2011	5	20	4540	21.83	27.25
19779043	2011	5	20	4550	21.63	27.26
19779043	2011	5	20	4560	21.15	27.29
19779043	2011	5	20	4570	20.94	27.32
19779043	2011	5	20	4580	21.1	27.32
19779043	2011	5	20	4590	21.03	27.33
19779043	2011	5	20	4600	20.99	27.35
19779043	2011	5	20	4610	20.85	27.36
19779043	2011	5	20	4620	20.55	27.38
19779043	2011	5	20	4630	20.47	27.39
19779043	2011	5	20	4640	20.58	27.42
19779043	2011	5	20	4650	20.44	27.43

19779043	2011	5	20	4660	20.4	27.44
19779043	2011	5	20	4670	20.58	27.45
19779043	2011	5	20	4680	20.34	27.46
19779043	2011	5	20	4690	19.98	27.47
19779043	2011	5	20	4700	19.96	27.49
19779043	2011	5	20	4710	19.23	27.5
19779043	2011	5	20	4720	18.67	27.53
19779043	2011	5	20	4730	18.28	27.55
19779043	2011	5	20	4740	18.06	27.57
19779043	2011	5	20	4750	17.48	27.6
19779043	2011	5	20	4760	16.83	27.62
19779043	2011	5	20	4770	16.25	27.64
19779043	2011	5	20	4780	15.72	27.67
19779043	2011	5	20	4790	15.51	27.71
19779043	2011	5	20	4800	15.21	27.74
19779043	2011	5	20	4810	15.05	27.77
19779043	2011	5	20	4820	14.69	27.79
19779043	2011	5	20	4830	13.42	27.82
19779043	2011	5	20	4840	11.13	27.88
19779043	2011	5	20	4850	9.78	27.92
19779043	2011	5	20	4860	7.83	27.96
19779043	2011	5	20	4870	5.61	28
19779043	2011	5	20	4880	4.18	28.03
19779043	2011	5	20	4890	2.93	28.07
19779043	2011	5	20	4900	2.73	28.11
19779043	2011	5	20	4910	2.19	28.15
19779043	2011	5	20	4920	2.21	28.21
19779043	2011	5	20	4930	0.61	28.25
19779043	2011	5	20	4940	0.59	28.28
19779043	2011	5	20	4950	0.61	28.29
19779043	2011	5	20	4960	0.61	28.33
19779043	2011	5	20	4970	0.57	28.36
19779043	2011	5	20	4980	0.6	28.39
19779043	2011	5	20	4990	0.58	28.42
19779043	2011	5	20	5000	0.6	28.43
19779043	2011	5	20	5010	0.5	28.46
19779043	2011	5	20	5020	0.44	28.48
19779043	2011	5	20	5030	0.47	28.5
19779043	2011	5	20	5040	0.48	28.52
19779043	2011	5	20	5050	0.48	28.53
19779043	2011	5	20	5060	0.47	28.57
19779043	2011	5	20	5070	0.47	28.57
19787843	2011	5	20	0	1	29.03
19787843	2011	5	20	10	1.57	29.03
19787843	2011	5	20	20	2.75	29.02
19787843	2011	5	20	30	2.52	29.01
19787843	2011	5	20	40	2.4	29.01

19787843	2011	5	20	50	2.03	29.02
19787843	2011	5	20	60	2.54	29.01
19787843	2011	5	20	70	3.08	28.99
19787843	2011	5	20	80	3.31	28.99
19787843	2011	5	20	90	3.84	28.98
19787843	2011	5	20	100	5.53	28.95
19787843	2011	5	20	110	6.25	28.93
19787843	2011	5	20	120	8.01	28.92
19787843	2011	5	20	130	9.69	28.89
19787843	2011	5	20	140	10.41	28.87
19787843	2011	5	20	150	10.86	28.85
19787843	2011	5	20	160	11.11	28.85
19787843	2011	5	20	170	11.16	28.82
19787843	2011	5	20	180	11.06	28.8
19787843	2011	5	20	190	10.64	28.79
19787843	2011	5	20	200	10.58	28.78
19787843	2011	5	20	210	10.92	28.76
19787843	2011	5	20	220	10.76	28.74
19787843	2011	5	20	230	10.7	28.73
19787843	2011	5	20	240	10.72	28.71
19787843	2011	5	20	250	10.76	28.71
19787843	2011	5	20	260	10.69	28.7
19787843	2011	5	20	270	10.75	28.69
19787843	2011	5	20	280	10.78	28.67
19787843	2011	5	20	290	10.72	28.67
19787843	2011	5	20	300	10.68	28.66
19787843	2011	5	20	310	10.76	28.64
19787843	2011	5	20	320	10.68	28.64
19787843	2011	5	20	330	10.65	28.64
19787843	2011	5	20	340	10.72	28.62
19787843	2011	5	20	350	10.68	28.61
19787843	2011	5	20	360	10.59	28.6
19787843	2011	5	20	370	10.73	28.6
19787843	2011	5	20	380	10.78	28.59
19787843	2011	5	20	390	11.47	28.56
19787843	2011	5	20	400	13.35	28.54
19787843	2011	5	20	410	15.41	28.5
19787843	2011	5	20	420	16.99	28.46
19787843	2011	5	20	430	17.55	28.46
19787843	2011	5	20	440	19	28.44
19787843	2011	5	20	450	20.77	28.35
19787843	2011	5	20	460	22.65	28.24
19787843	2011	5	20	470	24.74	28.17
19787843	2011	5	20	480	26.68	28.12
19787843	2011	5	20	490	29.04	28.05
19787843	2011	5	20	500	31.58	27.99
19787843	2011	5	20	510	33.81	27.93

19787843	2011	5	20	520	35.67	27.87
19787843	2011	5	20	530	37.78	27.8
19787843	2011	5	20	540	40.17	27.75
19787843	2011	5	20	550	42.52	27.67
19787843	2011	5	20	560	44.77	27.6
19787843	2011	5	20	570	46.96	27.53
19787843	2011	5	20	580	49.12	27.5
19787843	2011	5	20	590	51.74	27.45
19787843	2011	5	20	600	54.22	27.39
19787843	2011	5	20	610	56.22	27.29
19787843	2011	5	20	620	58.25	27.25
19787843	2011	5	20	630	60.49	27.2
19787843	2011	5	20	640	63.13	27.16
19787843	2011	5	20	650	66.09	27.12
19787843	2011	5	20	660	69.1	27.07
19787843	2011	5	20	670	71.79	26.99
19787843	2011	5	20	680	73.37	26.96
19787843	2011	5	20	690	73.95	26.93
19787843	2011	5	20	700	73.99	26.9
19787843	2011	5	20	710	73.8	26.87
19787843	2011	5	20	720	73.58	26.85
19787843	2011	5	20	730	73.62	26.79
19787843	2011	5	20	740	74.5	26.67
19787843	2011	5	20	750	75.18	26.56
19787843	2011	5	20	760	75.68	26.53
19787843	2011	5	20	770	75.96	26.48
19787843	2011	5	20	780	76.01	26.49
19787843	2011	5	20	790	75.73	26.47
19787843	2011	5	20	800	75.28	26.46
19787843	2011	5	20	810	74.99	26.44
19787843	2011	5	20	820	74.9	26.42
19787843	2011	5	20	830	75.26	26.4
19787843	2011	5	20	840	75.26	26.39
19787843	2011	5	20	850	75.08	26.35
19787843	2011	5	20	860	75.07	26.33
19787843	2011	5	20	870	75.01	26.31
19787843	2011	5	20	880	74.74	26.28
19787843	2011	5	20	890	74.36	26.28
19787843	2011	5	20	900	74.19	26.25
19787843	2011	5	20	910	74.17	26.24
19787843	2011	5	20	920	74.46	26.2
19787843	2011	5	20	930	74.79	26.19
19787843	2011	5	20	940	74.84	26.17
19787843	2011	5	20	950	74.87	26.14
19787843	2011	5	20	960	74.71	26.14
19787843	2011	5	20	970	74.52	26.13
19787843	2011	5	20	980	74.33	26.13

19787843	2011	5	20	990	74.22	26.12
19787843	2011	5	20	1000	73.36	26.11
19787843	2011	5	20	1010	72.61	26.1
19787843	2011	5	20	1020	72.07	26.09
19787843	2011	5	20	1030	71.67	26.09
19787843	2011	5	20	1040	71.26	26.08
19787843	2011	5	20	1050	70.94	26.07
19787843	2011	5	20	1060	70.74	26.06
19787843	2011	5	20	1070	70.27	26.05
19787843	2011	5	20	1080	68.97	26.05
19787843	2011	5	20	1090	68.16	26.04
19787843	2011	5	20	1100	66.5	26.05
19787843	2011	5	20	1110	64.29	26.06
19787843	2011	5	20	1120	62.49	26.06
19787843	2011	5	20	1130	61.57	26.07
19787843	2011	5	20	1140	59.9	26.08
19787843	2011	5	20	1150	58.32	26.12
19787843	2011	5	20	1160	58.93	26.16
19787843	2011	5	20	1170	59.41	26.18
19787843	2011	5	20	1180	59.33	26.19
19787843	2011	5	20	1190	57.96	26.21
19787843	2011	5	20	1200	55.66	26.25
19787843	2011	5	20	1210	53.71	26.27
19787843	2011	5	20	1220	51.93	26.28
19787843	2011	5	20	1230	50.53	26.32
19787843	2011	5	20	1240	50.27	26.35
19787843	2011	5	20	1250	49.82	26.35
19787843	2011	5	20	1260	49.59	26.38
19787843	2011	5	20	1270	49.11	26.42
19787843	2011	5	20	1280	48.61	26.44
19787843	2011	5	20	1290	48.4	26.46
19787843	2011	5	20	1300	49.74	26.47
19787843	2011	5	20	1310	51.22	26.49
19787843	2011	5	20	1320	52.16	26.5
19787843	2011	5	20	1330	53.35	26.5
19787843	2011	5	20	1340	53.95	26.5
19787843	2011	5	20	1350	54.09	26.5
19787843	2011	5	20	1360	54.05	26.51
19787843	2011	5	20	1370	53.98	26.5
19787843	2011	5	20	1380	53.76	26.5
19787843	2011	5	20	1390	53.78	26.51
19787843	2011	5	20	1400	53.78	26.51
19787843	2011	5	20	1410	53.66	26.52
19787843	2011	5	20	1420	53.48	26.52
19787843	2011	5	20	1430	53.8	26.52
19787843	2011	5	20	1440	53.97	26.52
19787843	2011	5	20	1450	54.22	26.52

19787843	2011	5	20	1460	55.09	26.52
19787843	2011	5	20	1470	55.22	26.51
19787843	2011	5	20	1480	54.74	26.52
19787843	2011	5	20	1490	54.83	26.52
19787843	2011	5	20	1500	54.83	26.52
19787843	2011	5	20	1510	54.68	26.52
19787843	2011	5	20	1520	54.66	26.52
19787843	2011	5	20	1530	54.67	26.53
19787843	2011	5	20	1540	54.85	26.53
19787843	2011	5	20	1550	54.78	26.53
19787843	2011	5	20	1560	54.63	26.53
19787843	2011	5	20	1570	54.55	26.53
19787843	2011	5	20	1580	54.2	26.53
19787843	2011	5	20	1590	52.57	26.54
19787843	2011	5	20	1600	50.57	26.56
19787843	2011	5	20	1610	49.85	26.57
19787843	2011	5	20	1620	49.55	26.58
19787843	2011	5	20	1630	48.39	26.6
19787843	2011	5	20	1640	48.87	26.61
19787843	2011	5	20	1650	48.99	26.62
19787843	2011	5	20	1660	48.91	26.64
19787843	2011	5	20	1670	48.7	26.64
19787843	2011	5	20	1680	48.48	26.64
19787843	2011	5	20	1690	47.06	26.66
19787843	2011	5	20	1700	46.53	26.67
19787843	2011	5	20	1710	45.59	26.68
19787843	2011	5	20	1720	45.44	26.7
19787843	2011	5	20	1730	45.64	26.71
19787843	2011	5	20	1740	45.37	26.71
19787843	2011	5	20	1750	45.15	26.71
19787843	2011	5	20	1760	44.86	26.72
19787843	2011	5	20	1770	44.58	26.74
19787843	2011	5	20	1780	44.86	26.73
19787843	2011	5	20	1790	44.72	26.74
19787843	2011	5	20	1800	44.52	26.75
19787843	2011	5	20	1810	44.29	26.75
19787843	2011	5	20	1820	44.15	26.75
19787843	2011	5	20	1830	44.82	26.75
19787843	2011	5	20	1840	44.95	26.76
19787843	2011	5	20	1850	45.07	26.76
19787843	2011	5	20	1860	45.32	26.76
19787843	2011	5	20	1870	45.55	26.77
19787843	2011	5	20	1880	45.62	26.77
19787843	2011	5	20	1890	45.38	26.77
19787843	2011	5	20	1900	45.02	26.78
19787843	2011	5	20	1910	45.76	26.78
19787843	2011	5	20	1920	45.84	26.78

19787843	2011	5	20	1930	45.54	26.78
19787843	2011	5	20	1940	45.35	26.78
19787843	2011	5	20	1950	45.38	26.78
19787843	2011	5	20	1960	45.48	26.78
19787843	2011	5	20	1970	45.67	26.79
19787843	2011	5	20	1980	45.74	26.79
19787843	2011	5	20	1990	46	26.79
19787843	2011	5	20	2000	46.06	26.79
19787843	2011	5	20	2010	46.05	26.79
19787843	2011	5	20	2020	46.04	26.8
19787843	2011	5	20	2030	46.04	26.79
19787843	2011	5	20	2040	46.07	26.8
19787843	2011	5	20	2050	45.89	26.81
19787843	2011	5	20	2060	45.69	26.81
19787843	2011	5	20	2070	45.6	26.81
19787843	2011	5	20	2080	45.56	26.81
19787843	2011	5	20	2090	45.27	26.82
19787843	2011	5	20	2100	44.95	26.82
19787843	2011	5	20	2110	45.03	26.82
19787843	2011	5	20	2120	45.13	26.82
19787843	2011	5	20	2130	45.96	26.82
19787843	2011	5	20	2140	46.99	26.82
19787843	2011	5	20	2150	46.85	26.81
19787843	2011	5	20	2160	47.03	26.81
19787843	2011	5	20	2170	47.08	26.82
19787843	2011	5	20	2180	47.22	26.82
19787843	2011	5	20	2190	47.51	26.82
19787843	2011	5	20	2200	47.62	26.82
19787843	2011	5	20	2210	47.66	26.82
19787843	2011	5	20	2220	47.76	26.82
19787843	2011	5	20	2230	49.32	26.81
19787843	2011	5	20	2240	51.53	26.79
19787843	2011	5	20	2250	52.89	26.78
19787843	2011	5	20	2260	53.2	26.78
19787843	2011	5	20	2270	53.01	26.78
19787843	2011	5	20	2280	51.32	26.78
19787843	2011	5	20	2290	51.75	26.78
19787843	2011	5	20	2300	52.21	26.78
19787843	2011	5	20	2310	51.98	26.78
19787843	2011	5	20	2320	51.35	26.79
19787843	2011	5	20	2330	51.45	26.78
19787843	2011	5	20	2340	51.45	26.78
19787843	2011	5	20	2350	51.26	26.78
19787843	2011	5	20	2360	51.47	26.78
19787843	2011	5	20	2370	51.51	26.78
19787843	2011	5	20	2380	51.88	26.78
19787843	2011	5	20	2390	52.43	26.78

19787843	2011	5	20	2400	52.18	26.78
19787843	2011	5	20	2410	52.08	26.77
19787843	2011	5	20	2420	51.98	26.78
19787843	2011	5	20	2430	51.87	26.78
19787843	2011	5	20	2440	51.86	26.77
19787843	2011	5	20	2450	51.98	26.77
19787843	2011	5	20	2460	52.26	26.77
19787843	2011	5	20	2470	52.37	26.77
19787843	2011	5	20	2480	52.32	26.76
19787843	2011	5	20	2490	51.93	26.77
19787843	2011	5	20	2500	51.7	26.77
19787843	2011	5	20	2510	52.09	26.76
19787843	2011	5	20	2520	52.22	26.76
19787843	2011	5	20	2530	53.09	26.75
19787843	2011	5	20	2540	55.37	26.75
19787843	2011	5	20	2550	57.91	26.72
19787843	2011	5	20	2560	60.5	26.7
19787843	2011	5	20	2570	63.3	26.67
19787843	2011	5	20	2580	66.16	26.65
19787843	2011	5	20	2590	69.21	26.62
19787843	2011	5	20	2600	71.63	26.57
19787843	2011	5	20	2610	73.75	26.53
19787843	2011	5	20	2620	75.44	26.48
19787843	2011	5	20	2630	77.46	26.39
19787843	2011	5	20	2640	80.05	26.35
19787843	2011	5	20	2650	82.59	26.31
19787843	2011	5	20	2660	83.34	26.28
19787843	2011	5	20	2670	83.38	26.21
19787843	2011	5	20	2680	83.63	26.17
19787843	2011	5	20	2690	85.49	26.09
19787843	2011	5	20	2700	87.16	26.02
19787843	2011	5	20	2710	86.58	25.99
19787843	2011	5	20	2720	85.18	25.95
19787843	2011	5	20	2730	84.66	25.88
19787843	2011	5	20	2740	85.26	25.76
19787843	2011	5	20	2750	85.81	25.73
19787843	2011	5	20	2760	85.81	25.68
19787843	2011	5	20	2770	85.61	25.64
19787843	2011	5	20	2780	85.55	25.6
19787843	2011	5	20	2790	85.65	25.55
19787843	2011	5	20	2800	85.41	25.5
19787843	2011	5	20	2810	85.12	25.45
19787843	2011	5	20	2820	85.11	25.43
19787843	2011	5	20	2830	85.74	25.38
19787843	2011	5	20	2840	87.21	25.32
19787843	2011	5	20	2850	87.75	25.28
19787843	2011	5	20	2860	88.52	25.22

19787843	2011	5	20	2870	90.35	25.17
19787843	2011	5	20	2880	90.86	25.11
19787843	2011	5	20	2890	90.88	25.08
19787843	2011	5	20	2900	91.22	25.03
19787843	2011	5	20	2910	91.41	24.98
19787843	2011	5	20	2920	92.44	24.91
19787843	2011	5	20	2930	92.76	24.85
19787843	2011	5	20	2940	93.2	24.81
19787843	2011	5	20	2950	93.29	24.74
19787843	2011	5	20	2960	93.17	24.67
19787843	2011	5	20	2970	93.67	24.62
19787843	2011	5	20	2980	93.97	24.57
19787843	2011	5	20	2990	94.02	24.54
19787843	2011	5	20	3000	94.52	24.5
19787843	2011	5	20	3010	95.01	24.46
19787843	2011	5	20	3020	95.57	24.38
19787843	2011	5	20	3030	95.67	24.32
19787843	2011	5	20	3040	95.79	24.24
19787843	2011	5	20	3050	96.05	24.16
19787843	2011	5	20	3060	97.14	24.12
19787843	2011	5	20	3070	97.52	24.07
19787843	2011	5	20	3080	97.71	24.02
19787843	2011	5	20	3090	98.14	23.81
19787843	2011	5	20	3100	98.34	23.75
19787843	2011	5	20	3110	99.17	23.67
19787843	2011	5	20	3120	99.68	23.57
19787843	2011	5	20	3130	100.13	23.48
19787843	2011	5	20	3140	100.62	23.42
19787843	2011	5	20	3150	100.85	23.42
19787843	2011	5	20	3160	101.15	23.34
19787843	2011	5	20	3170	102.08	23.32
19787843	2011	5	20	3180	102.76	23.26
19787843	2011	5	20	3190	103.15	23.2
19787843	2011	5	20	3200	103.27	23.06
19787843	2011	5	20	3210	103.66	22.96
19787843	2011	5	20	3220	104.03	22.84
19787843	2011	5	20	3230	104.46	22.75
19787843	2011	5	20	3240	104.44	22.66
19787843	2011	5	20	3250	104.43	22.63
19787843	2011	5	20	3260	105.26	22.55
19787843	2011	5	20	3270	105.3	22.51
19787843	2011	5	20	3280	104.95	22.46
19787843	2011	5	20	3290	104.78	22.4
19787843	2011	5	20	3300	105.26	22.33
19787843	2011	5	20	3310	104.97	22.3
19787843	2011	5	20	3320	105.09	22.25
19787843	2011	5	20	3330	105.02	22.21

19787843	2011	5	20	3340	105.15	22.17
19787843	2011	5	20	3350	104.9	22.13
19787843	2011	5	20	3360	104.72	22.09
19787843	2011	5	20	3370	104.75	22.05
19787843	2011	5	20	3380	104.71	22.01
19787843	2011	5	20	3390	104.98	21.97
19787843	2011	5	20	3400	105.07	21.95
19787843	2011	5	20	3410	105.1	21.91
19787843	2011	5	20	3420	105.05	21.89
19787843	2011	5	20	3430	104.77	21.85
19787843	2011	5	20	3440	104.45	21.84
19787843	2011	5	20	3450	104.62	21.8
19787843	2011	5	20	3460	104.6	21.79
19787843	2011	5	20	3470	105.57	21.76
19787843	2011	5	20	3480	106.55	21.71
19787843	2011	5	20	3490	107.34	21.71
19787843	2011	5	20	3500	107.98	21.68
19787843	2011	5	20	3510	108.04	21.66
19787843	2011	5	20	3520	108.68	21.64
19787843	2011	5	20	3530	109.82	21.6
19787843	2011	5	20	3540	110.42	21.59
19787843	2011	5	20	3550	110.4	21.57
19787843	2011	5	20	3560	111.1	21.52
19787843	2011	5	20	3570	111.49	21.51
19787843	2011	5	20	3580	111.74	21.5
19787843	2011	5	20	3590	111.78	21.49
19787843	2011	5	20	3600	112.52	21.47
19787843	2011	5	20	3610	112.89	21.46
19787843	2011	5	20	3620	112.92	21.44
19787843	2011	5	20	3630	112.43	21.44
19787843	2011	5	20	3640	112.64	21.42
19787843	2011	5	20	3650	113.37	21.39
19787843	2011	5	20	3660	114.04	21.38
19787843	2011	5	20	3670	114.41	21.37
19787843	2011	5	20	3680	114.91	21.35
19787843	2011	5	20	3690	115.88	21.32
19787843	2011	5	20	3700	116.64	21.25
19787843	2011	5	20	3710	116.6	21.21
19787843	2011	5	20	3720	116.8	21.16
19787843	2011	5	20	3730	117.47	21.13
19787843	2011	5	20	3740	117.88	21.07
19787843	2011	5	20	3750	118.72	20.89
19787843	2011	5	20	3760	119.57	20.81
19787843	2011	5	20	3770	119.64	20.75
19787843	2011	5	20	3780	120.22	20.68
19787843	2011	5	20	3790	121.52	20.56
19787843	2011	5	20	3800	122.1	20.5

19787843	2011	5	20	3810	122.37	20.45
19787843	2011	5	20	3820	122.24	20.39
19787843	2011	5	20	3830	122.15	20.36
19787843	2011	5	20	3840	122.12	20.31
19787843	2011	5	20	3850	122.03	20.25
19787843	2011	5	20	3860	121.86	20.21
19787843	2011	5	20	3870	121.67	20.15
19787843	2011	5	20	3880	121.5	20.11
19787843	2011	5	20	3890	121.27	20.08
19787843	2011	5	20	3900	121.08	20.06
19787843	2011	5	20	3910	120.85	20.03
19787843	2011	5	20	3920	120.5	20.03
19787843	2011	5	20	3930	120.01	20.02
19787843	2011	5	20	3940	119.61	20
19787843	2011	5	20	3950	119.36	19.98
19787843	2011	5	20	3960	119.19	19.99
19787843	2011	5	20	3970	119.02	20
19787843	2011	5	20	3980	118.98	20
19787843	2011	5	20	3990	119.54	20.01
19787843	2011	5	20	4000	119.44	20
19787843	2011	5	20	4010	119.15	20
19787843	2011	5	20	4020	118.73	20
19787843	2011	5	20	4030	118.29	20
19787843	2011	5	20	4040	117.91	19.99
19787843	2011	5	20	4050	117.5	20
19787843	2011	5	20	4060	117.19	20.03
19787843	2011	5	20	4070	116.82	20.07
19787843	2011	5	20	4080	116.42	20.11
19787843	2011	5	20	4090	115.96	20.16
19787843	2011	5	20	4100	115.49	20.21
19787843	2011	5	20	4110	115.37	20.22
19787843	2011	5	20	4120	115.41	20.29
19787843	2011	5	20	4130	115.38	20.33
19787843	2011	5	20	4140	115.83	20.38
19787843	2011	5	20	4150	117.1	20.41
19787843	2011	5	20	4160	117.71	20.43
19787843	2011	5	20	4170	117.83	20.47
19787843	2011	5	20	4180	118.49	20.49
19787843	2011	5	20	4190	119.67	20.5
19787843	2011	5	20	4200	121.2	20.5
19787843	2011	5	20	4210	121.9	20.46
19787843	2011	5	20	4220	121.7	20.39
19787843	2011	5	20	4230	121.72	20.33
19787843	2011	5	20	4240	122.07	20.28
19787843	2011	5	20	4250	122.04	20.24
19787843	2011	5	20	4260	121.91	20.21
19787843	2011	5	20	4270	122	20.19

19787843	2011	5	20	4280	122.02	20.15
19787843	2011	5	20	4290	122.26	20.12
19787843	2011	5	20	4300	122.33	20.09
19787843	2011	5	20	4310	122.35	20.05
19787843	2011	5	20	4320	122.44	20.03
19787843	2011	5	20	4330	121.71	20
19787843	2011	5	20	4340	121.08	19.96
19787843	2011	5	20	4350	121.43	19.92
19787843	2011	5	20	4360	121.24	19.91
19787843	2011	5	20	4370	120.93	19.91
19787843	2011	5	20	4380	120.55	19.89
19787843	2011	5	20	4390	120.33	19.91
19787843	2011	5	20	4400	120.36	19.92
19787843	2011	5	20	4410	120.26	19.92
19787843	2011	5	20	4420	120.12	19.92
19787843	2011	5	20	4430	120.07	19.93
19787843	2011	5	20	4440	119.62	19.96
19787843	2011	5	20	4450	119.18	20
19787843	2011	5	20	4460	118.22	20.02
19787843	2011	5	20	4470	115.86	20.07
19787843	2011	5	20	4480	113.11	20.1
19787843	2011	5	20	4490	109.71	20.14
19787843	2011	5	20	4500	107.46	20.19
19787843	2011	5	20	4510	106.53	20.24
19787843	2011	5	20	4520	104.47	20.29
19787843	2011	5	20	4530	102.71	20.33
19787843	2011	5	20	4540	101.29	20.38
19787843	2011	5	20	4550	99.11	20.42
19787843	2011	5	20	4560	97.96	20.48
19787843	2011	5	20	4570	97.58	20.6
19787843	2011	5	20	4580	97.2	20.75
19787843	2011	5	20	4590	97.38	20.89
19787843	2011	5	20	4600	97.6	21.06
19787843	2011	5	20	4610	97.49	21.14
19787843	2011	5	20	4620	97.15	21.21
19787843	2011	5	20	4630	96.65	21.27
19787843	2011	5	20	4640	97.34	21.36
19787843	2011	5	20	4650	97.82	21.43
19787843	2011	5	20	4660	97.55	21.53
19787843	2011	5	20	4670	97.3	21.6
19787843	2011	5	20	4680	97.45	21.7
19787843	2011	5	20	4690	97.55	21.76
19787843	2011	5	20	4700	97.36	21.82
19787843	2011	5	20	4710	97.27	21.89
19787843	2011	5	20	4720	97.5	21.94
19787843	2011	5	20	4730	97.54	22
19787843	2011	5	20	4740	97.52	22.06

19787843	2011	5	20	4750	97.52	22.11
19787843	2011	5	20	4760	97.65	22.16
19787843	2011	5	20	4770	97.63	22.25
19787843	2011	5	20	4780	98.2	22.32
19787843	2011	5	20	4790	97.63	22.37
19787843	2011	5	20	4800	97.42	22.4
19787843	2011	5	20	4810	97.72	22.44
19787843	2011	5	20	4820	97.72	22.49
19787843	2011	5	20	4830	97.72	22.53
19787843	2011	5	20	4840	97.65	22.56
19787843	2011	5	20	4850	97.45	22.58
19787843	2011	5	20	4860	97.5	22.6
19787843	2011	5	20	4870	97.6	22.64
19787843	2011	5	20	4880	97.55	22.67
19787843	2011	5	20	4890	97.35	22.68
19787843	2011	5	20	4900	95.55	22.74
19787843	2011	5	20	4910	92.92	22.8
19787843	2011	5	20	4920	90.19	22.87
19787843	2011	5	20	4930	87.59	22.92
19787843	2011	5	20	4940	85.09	22.99
19787843	2011	5	20	4950	82.62	23.11
19787843	2011	5	20	4960	80.18	23.21
19787843	2011	5	20	4970	77.77	23.33
19787843	2011	5	20	4980	75.53	23.54
19787843	2011	5	20	4990	73.13	23.67
19787843	2011	5	20	5000	71.37	23.78
19787843	2011	5	20	5010	69.25	23.89
19787843	2011	5	20	5020	66.81	24.03
19787843	2011	5	20	5030	64.32	24.19
19787843	2011	5	20	5040	61.94	24.3
19787843	2011	5	20	5050	59.63	24.44
19787843	2011	5	20	5060	58.79	24.59
19787843	2011	5	20	5070	58.58	24.74
19787843	2011	5	20	5080	57.15	24.83
19787843	2011	5	20	5090	56.57	24.89
19787843	2011	5	20	5100	55.86	24.97
19787843	2011	5	20	5110	55.13	25.07
19787843	2011	5	20	5120	54.13	25.15
19787843	2011	5	20	5130	53.71	25.27
19787843	2011	5	20	5140	53.46	25.34
19787843	2011	5	20	5150	53.34	25.42
19787843	2011	5	20	5160	52.74	25.49
19787843	2011	5	20	5170	51.83	25.56
19787843	2011	5	20	5180	50.57	25.62
19787843	2011	5	20	5190	50.11	25.69
19787843	2011	5	20	5200	50.61	25.77
19787843	2011	5	20	5210	50.25	25.85

19787843	2011	5	20	5220	48.73	25.92
19787843	2011	5	20	5230	47.59	25.97
19787843	2011	5	20	5240	46.8	26.01
19787843	2011	5	20	5250	45.83	26.07
19787843	2011	5	20	5260	44.36	26.12
19787843	2011	5	20	5270	42.8	26.18
19787843	2011	5	20	5280	41.71	26.24
19787843	2011	5	20	5290	40.06	26.3
19787843	2011	5	20	5300	37.81	26.35
19787843	2011	5	20	5310	35.63	26.42
19787843	2011	5	20	5320	33.56	26.48
19787843	2011	5	20	5330	31.52	26.53
19787843	2011	5	20	5340	29.49	26.6
19787843	2011	5	20	5350	28.01	26.67
19787843	2011	5	20	5360	26.73	26.71
19787843	2011	5	20	5370	26.24	26.75
19787843	2011	5	20	5380	26.07	26.81
19787843	2011	5	20	5390	24.97	26.86
19787843	2011	5	20	5400	25.13	26.93
19787843	2011	5	20	5410	25.48	27.03
19787843	2011	5	20	5420	24.66	27.07
19787843	2011	5	20	5430	23.17	27.13
19787843	2011	5	20	5440	20.89	27.2
19787843	2011	5	20	5450	18.21	27.28
19787843	2011	5	20	5460	16.49	27.34
19787843	2011	5	20	5470	15.53	27.38
19787843	2011	5	20	5480	14.64	27.43
19787843	2011	5	20	5490	13.86	27.47
19787843	2011	5	20	5500	11.81	27.54
19787843	2011	5	20	5510	10.38	27.6
19787843	2011	5	20	5520	9.11	27.64
19787843	2011	5	20	5530	8.13	27.69
19787843	2011	5	20	5540	7.23	27.75
19787843	2011	5	20	5550	6.56	27.8
19787843	2011	5	20	5560	4.7	27.85
19787843	2011	5	20	5570	4.19	27.89
19787843	2011	5	20	5580	4.59	27.93
19787843	2011	5	20	5590	4.6	27.96
19787843	2011	5	20	5600	4.05	27.99
19787843	2011	5	20	5610	3.45	28.01
19787843	2011	5	20	5620	2.86	28.05
19787843	2011	5	20	5630	2.43	28.08
19787843	2011	5	20	5640	2.15	28.12
19787843	2011	5	20	5650	2.56	28.19
19787843	2011	5	20	5660	2.49	28.21
19787843	2011	5	20	5670	2.41	28.21
19787843	2011	5	20	5680	2.31	28.24

19787843	2011	5	20	5690	0.94	28.26
19787843	2011	5	20	5700	0.63	28.3
19787843	2011	5	20	5710	0.59	28.32
19787843	2011	5	20	5720	0.59	28.35
19787843	2011	5	20	5730	0.6	28.38
19787843	2011	5	20	5740	0.59	28.42
19787843	2011	5	20	5750	0.65	28.47
19787843	2011	5	20	5760	0.6	28.49
19787843	2011	5	20	5770	0.61	28.51
19787843	2011	5	20	5780	0.57	28.54
19787843	2011	5	20	5790	0.65	28.56
19787843	2011	5	20	5800	0.65	28.57
19787843	2011	5	20	5810	0.56	28.6
19787843	2011	5	20	5820	0.49	28.63
19787843	2011	5	20	5830	0.48	28.65
19787843	2011	5	20	5840	0.48	28.67
20027853	2011	5	23	0	1.41	28.6
20027853	2011	5	23	10	1.86	28.64
20027853	2011	5	23	20	2.57	28.63
20027853	2011	5	23	30	2.53	28.63
20027853	2011	5	23	40	2.22	28.64
20027853	2011	5	23	50	2.11	28.63
20027853	2011	5	23	60	2.27	28.64
20027853	2011	5	23	70	2.63	28.63
20027853	2011	5	23	80	2.35	28.64
20027853	2011	5	23	90	2.66	28.62
20027853	2011	5	23	100	3.15	28.63
20027853	2011	5	23	110	4.12	28.61
20027853	2011	5	23	120	6.3	28.6
20027853	2011	5	23	130	7.59	28.59
20027853	2011	5	23	140	8.03	28.59
20027853	2011	5	23	150	9.45	28.56
20027853	2011	5	23	160	11.18	28.54
20027853	2011	5	23	170	12.66	28.52
20027853	2011	5	23	180	14.39	28.5
20027853	2011	5	23	190	16.67	28.48
20027853	2011	5	23	200	18.85	28.46
20027853	2011	5	23	210	20.74	28.44
20027853	2011	5	23	220	22.55	28.41
20027853	2011	5	23	230	24.44	28.39
20027853	2011	5	23	240	26.77	28.37
20027853	2011	5	23	250	28.93	28.35
20027853	2011	5	23	260	30.94	28.32
20027853	2011	5	23	270	33.24	28.29
20027853	2011	5	23	280	35.77	28.28
20027853	2011	5	23	290	37.85	28.22
20027853	2011	5	23	300	40.01	28.14

20027853	2011	5	23	310	42.34	28.1
20027853	2011	5	23	320	44.65	28.04
20027853	2011	5	23	330	46.86	27.99
20027853	2011	5	23	340	49.07	27.94
20027853	2011	5	23	350	51.38	27.87
20027853	2011	5	23	360	53.75	27.81
20027853	2011	5	23	370	56.2	27.73
20027853	2011	5	23	380	58.52	27.64
20027853	2011	5	23	390	60.5	27.59
20027853	2011	5	23	400	62.69	27.53
20027853	2011	5	23	410	64.68	27.47
20027853	2011	5	23	420	66.59	27.28
20027853	2011	5	23	430	68.15	27.09
20027853	2011	5	23	440	70.54	27.03
20027853	2011	5	23	450	72.86	26.97
20027853	2011	5	23	460	75.15	26.82
20027853	2011	5	23	470	77.83	26.71
20027853	2011	5	23	480	80.25	26.63
20027853	2011	5	23	490	82.17	26.38
20027853	2011	5	23	500	84.35	26.31
20027853	2011	5	23	510	86.76	26.27
20027853	2011	5	23	520	89.6	26.17
20027853	2011	5	23	530	92.43	26.09
20027853	2011	5	23	540	95.06	25.96
20027853	2011	5	23	550	97.9	25.85
20027853	2011	5	23	560	100.98	25.71
20027853	2011	5	23	570	103.96	25.53
20027853	2011	5	23	580	106.95	25.38
20027853	2011	5	23	590	110.07	25.21
20027853	2011	5	23	600	112.92	24.87
20027853	2011	5	23	610	115.7	24.47
20027853	2011	5	23	620	117.97	24.36
20027853	2011	5	23	630	120.35	24.2
20027853	2011	5	23	640	122.9	24.03
20027853	2011	5	23	650	125.13	23.87
20027853	2011	5	23	660	125.42	23.68
20027853	2011	5	23	670	125.94	23.48
20027853	2011	5	23	680	127.36	23.32
20027853	2011	5	23	690	128.06	23.11
20027853	2011	5	23	700	128.15	22.99
20027853	2011	5	23	710	128.09	22.88
20027853	2011	5	23	720	128.09	22.73
20027853	2011	5	23	730	129.16	22.38
20027853	2011	5	23	740	129.25	22.32
20027853	2011	5	23	750	128.92	22.25
20027853	2011	5	23	760	128.91	22.13
20027853	2011	5	23	770	129.05	22.04

20027853	2011	5	23	780	129.08	21.97
20027853	2011	5	23	790	128.96	21.89
20027853	2011	5	23	800	129.27	21.8
20027853	2011	5	23	810	129.26	21.69
20027853	2011	5	23	820	129.45	21.61
20027853	2011	5	23	830	129.49	21.53
20027853	2011	5	23	840	129.22	21.44
20027853	2011	5	23	850	128.8	21.35
20027853	2011	5	23	860	128.38	21.28
20027853	2011	5	23	870	128.01	21.21
20027853	2011	5	23	880	128.81	21.14
20027853	2011	5	23	890	129.63	21.08
20027853	2011	5	23	900	130.05	21.01
20027853	2011	5	23	910	130.46	20.94
20027853	2011	5	23	920	130.5	20.85
20027853	2011	5	23	930	130.24	20.8
20027853	2011	5	23	940	130.25	20.74
20027853	2011	5	23	950	129.93	20.69
20027853	2011	5	23	960	130.13	20.64
20027853	2011	5	23	970	130.52	20.59
20027853	2011	5	23	980	130.17	20.53
20027853	2011	5	23	990	129.47	20.47
20027853	2011	5	23	1000	129.01	20.41
20027853	2011	5	23	1010	128.65	20.36
20027853	2011	5	23	1020	128.84	20.31
20027853	2011	5	23	1030	128.68	20.26
20027853	2011	5	23	1040	129.11	20.23
20027853	2011	5	23	1050	129.42	20.19
20027853	2011	5	23	1060	129.18	20.16
20027853	2011	5	23	1070	128.64	20.14
20027853	2011	5	23	1080	128.75	20.11
20027853	2011	5	23	1090	128.89	20.09
20027853	2011	5	23	1100	128.66	20.07
20027853	2011	5	23	1110	128.41	20.04
20027853	2011	5	23	1120	128.03	20.03
20027853	2011	5	23	1130	127.35	20.01
20027853	2011	5	23	1140	127.29	19.99
20027853	2011	5	23	1150	127.37	19.96
20027853	2011	5	23	1160	127.42	19.96
20027853	2011	5	23	1170	127.32	19.95
20027853	2011	5	23	1180	127.18	19.94
20027853	2011	5	23	1190	127.11	19.93
20027853	2011	5	23	1200	127.19	19.92
20027853	2011	5	23	1210	126.62	19.92
20027853	2011	5	23	1220	125.92	19.91
20027853	2011	5	23	1230	125.32	19.91
20027853	2011	5	23	1240	124.82	19.91

20027853	2011	5	23	1250	125.17	19.9
20027853	2011	5	23	1260	125.47	19.91
20027853	2011	5	23	1270	125.33	19.9
20027853	2011	5	23	1280	124.78	19.9
20027853	2011	5	23	1290	124.08	19.89
20027853	2011	5	23	1300	123.75	19.91
20027853	2011	5	23	1310	123.43	19.91
20027853	2011	5	23	1320	124.19	19.91
20027853	2011	5	23	1330	124.31	19.91
20027853	2011	5	23	1340	123.48	19.91
20027853	2011	5	23	1350	123.05	19.92
20027853	2011	5	23	1360	123.32	19.92
20027853	2011	5	23	1370	123.57	19.92
20027853	2011	5	23	1380	123.69	19.92
20027853	2011	5	23	1390	123.96	19.91
20027853	2011	5	23	1400	124.49	19.92
20027853	2011	5	23	1410	124.63	19.91
20027853	2011	5	23	1420	124.62	19.92
20027853	2011	5	23	1430	124.87	19.91
20027853	2011	5	23	1440	125.1	19.9
20027853	2011	5	23	1450	125.07	19.9
20027853	2011	5	23	1460	124.81	19.9
20027853	2011	5	23	1470	124.72	19.89
20027853	2011	5	23	1480	126.24	19.89
20027853	2011	5	23	1490	127.95	19.88
20027853	2011	5	23	1500	129.04	19.88
20027853	2011	5	23	1510	129.34	19.87
20027853	2011	5	23	1520	129.56	19.86
20027853	2011	5	23	1530	129.84	19.85
20027853	2011	5	23	1540	129.85	19.82
20027853	2011	5	23	1550	129.92	19.81
20027853	2011	5	23	1560	130.68	19.78
20027853	2011	5	23	1570	131.03	19.77
20027853	2011	5	23	1580	131.44	19.75
20027853	2011	5	23	1590	132.2	19.74
20027853	2011	5	23	1600	132.44	19.71
20027853	2011	5	23	1610	131.69	19.69
20027853	2011	5	23	1620	132.2	19.67
20027853	2011	5	23	1630	132.3	19.66
20027853	2011	5	23	1640	132.12	19.64
20027853	2011	5	23	1650	131.95	19.61
20027853	2011	5	23	1660	131.65	19.6
20027853	2011	5	23	1670	131.27	19.6
20027853	2011	5	23	1680	131.06	19.59
20027853	2011	5	23	1690	131.53	19.57
20027853	2011	5	23	1700	131.46	19.54
20027853	2011	5	23	1710	131.31	19.52

20027853	2011	5	23	1720	131.71	19.51
20027853	2011	5	23	1730	131.51	19.5
20027853	2011	5	23	1740	131.23	19.49
20027853	2011	5	23	1750	131.79	19.47
20027853	2011	5	23	1760	131.91	19.46
20027853	2011	5	23	1770	131.74	19.45
20027853	2011	5	23	1780	131.78	19.46
20027853	2011	5	23	1790	131.76	19.44
20027853	2011	5	23	1800	131.89	19.43
20027853	2011	5	23	1810	131.55	19.43
20027853	2011	5	23	1820	131.49	19.42
20027853	2011	5	23	1830	131.66	19.42
20027853	2011	5	23	1840	131.85	19.4
20027853	2011	5	23	1850	131.64	19.39
20027853	2011	5	23	1860	131.46	19.39
20027853	2011	5	23	1870	130.73	19.39
20027853	2011	5	23	1880	129.95	19.38
20027853	2011	5	23	1890	129.43	19.38
20027853	2011	5	23	1900	130.18	19.38
20027853	2011	5	23	1910	130.59	19.38
20027853	2011	5	23	1920	130.79	19.37
20027853	2011	5	23	1930	130.85	19.37
20027853	2011	5	23	1940	130.55	19.37
20027853	2011	5	23	1950	129.82	19.36
20027853	2011	5	23	1960	128.96	19.37
20027853	2011	5	23	1970	128.38	19.37
20027853	2011	5	23	1980	127.82	19.38
20027853	2011	5	23	1990	128.85	19.38
20027853	2011	5	23	2000	129.12	19.37
20027853	2011	5	23	2010	129.29	19.37
20027853	2011	5	23	2020	129.18	19.37
20027853	2011	5	23	2030	128.86	19.37
20027853	2011	5	23	2040	128.81	19.38
20027853	2011	5	23	2050	128.81	19.37
20027853	2011	5	23	2060	128.51	19.38
20027853	2011	5	23	2070	128.77	19.38
20027853	2011	5	23	2080	128.7	19.38
20027853	2011	5	23	2090	128.34	19.38
20027853	2011	5	23	2100	128.44	19.38
20027853	2011	5	23	2110	128.54	19.39
20027853	2011	5	23	2120	128.4	19.39
20027853	2011	5	23	2130	128.47	19.39
20027853	2011	5	23	2140	128.43	19.39
20027853	2011	5	23	2150	128.31	19.39
20027853	2011	5	23	2160	128.1	19.39
20027853	2011	5	23	2170	127.68	19.39
20027853	2011	5	23	2180	127.31	19.39

20027853	2011	5	23	2190	126.84	19.39
20027853	2011	5	23	2200	126.52	19.39
20027853	2011	5	23	2210	126.5	19.39
20027853	2011	5	23	2220	126.78	19.4
20027853	2011	5	23	2230	126.69	19.39
20027853	2011	5	23	2240	127.2	19.39
20027853	2011	5	23	2250	127.17	19.39
20027853	2011	5	23	2260	126.96	19.4
20027853	2011	5	23	2270	127.19	19.39
20027853	2011	5	23	2280	126.92	19.4
20027853	2011	5	23	2290	126.68	19.4
20027853	2011	5	23	2300	126.22	19.4
20027853	2011	5	23	2310	125.77	19.4
20027853	2011	5	23	2320	125.24	19.4
20027853	2011	5	23	2330	124.91	19.4
20027853	2011	5	23	2340	124.36	19.4
20027853	2011	5	23	2350	124.38	19.4
20027853	2011	5	23	2360	124.12	19.4
20027853	2011	5	23	2370	122.79	19.4
20027853	2011	5	23	2380	123.05	19.44
20027853	2011	5	23	2390	123.02	19.46
20027853	2011	5	23	2400	122.45	19.49
20027853	2011	5	23	2410	121.71	19.5
20027853	2011	5	23	2420	121.66	19.52
20027853	2011	5	23	2430	121.28	19.54
20027853	2011	5	23	2440	121.18	19.57
20027853	2011	5	23	2450	121.05	19.6
20027853	2011	5	23	2460	121	19.64
20027853	2011	5	23	2470	121.06	19.66
20027853	2011	5	23	2480	121.15	19.68
20027853	2011	5	23	2490	121.09	19.7
20027853	2011	5	23	2500	121.12	19.72
20027853	2011	5	23	2510	120.72	19.75
20027853	2011	5	23	2520	120.14	19.76
20027853	2011	5	23	2530	120.16	19.78
20027853	2011	5	23	2540	120.2	19.8
20027853	2011	5	23	2550	119.99	19.82
20027853	2011	5	23	2560	120.41	19.82
20027853	2011	5	23	2570	120.14	19.84
20027853	2011	5	23	2580	119.73	19.85
20027853	2011	5	23	2590	119.71	19.86
20027853	2011	5	23	2600	119.7	19.88
20027853	2011	5	23	2610	119.1	19.89
20027853	2011	5	23	2620	119.2	19.89
20027853	2011	5	23	2630	119.31	19.9
20027853	2011	5	23	2640	118.75	19.92
20027853	2011	5	23	2650	118.83	19.92

20027853	2011	5	23	2660	118.66	19.93
20027853	2011	5	23	2670	118.2	19.95
20027853	2011	5	23	2680	117.94	19.96
20027853	2011	5	23	2690	117.95	19.96
20027853	2011	5	23	2700	117.92	19.98
20027853	2011	5	23	2710	117.41	19.99
20027853	2011	5	23	2720	116.91	19.99
20027853	2011	5	23	2730	116.36	20
20027853	2011	5	23	2740	115.08	20.02
20027853	2011	5	23	2750	113.85	20.03
20027853	2011	5	23	2760	113.52	20.04
20027853	2011	5	23	2770	113.46	20.05
20027853	2011	5	23	2780	113.03	20.07
20027853	2011	5	23	2790	113.98	20.11
20027853	2011	5	23	2800	113.25	20.13
20027853	2011	5	23	2810	113.16	20.13
20027853	2011	5	23	2820	113.82	20.16
20027853	2011	5	23	2830	114.31	20.17
20027853	2011	5	23	2840	114.22	20.19
20027853	2011	5	23	2850	113.99	20.21
20027853	2011	5	23	2860	113.83	20.23
20027853	2011	5	23	2870	114.01	20.25
20027853	2011	5	23	2880	114.06	20.27
20027853	2011	5	23	2890	114	20.28
20027853	2011	5	23	2900	114.1	20.31
20027853	2011	5	23	2910	113.85	20.32
20027853	2011	5	23	2920	113.96	20.35
20027853	2011	5	23	2930	113.98	20.36
20027853	2011	5	23	2940	113.95	20.39
20027853	2011	5	23	2950	113.4	20.41
20027853	2011	5	23	2960	112.82	20.42
20027853	2011	5	23	2970	112.6	20.46
20027853	2011	5	23	2980	112.62	20.48
20027853	2011	5	23	2990	112.29	20.5
20027853	2011	5	23	3000	112.48	20.53
20027853	2011	5	23	3010	112.1	20.55
20027853	2011	5	23	3020	111.96	20.57
20027853	2011	5	23	3030	111.87	20.6
20027853	2011	5	23	3040	112.26	20.64
20027853	2011	5	23	3050	112.18	20.67
20027853	2011	5	23	3060	112.28	20.7
20027853	2011	5	23	3070	112.02	20.71
20027853	2011	5	23	3080	112.54	20.73
20027853	2011	5	23	3090	112.79	20.76
20027853	2011	5	23	3100	112.76	20.78
20027853	2011	5	23	3110	112.48	20.79
20027853	2011	5	23	3120	112.72	20.82

20027853	2011	5	23	3130	112.69	20.84
20027853	2011	5	23	3140	112.19	20.85
20027853	2011	5	23	3150	112.2	20.86
20027853	2011	5	23	3160	112.48	20.89
20027853	2011	5	23	3170	112.23	20.89
20027853	2011	5	23	3180	111.94	20.9
20027853	2011	5	23	3190	112.03	20.92
20027853	2011	5	23	3200	112.18	20.92
20027853	2011	5	23	3210	111.47	20.92
20027853	2011	5	23	3220	111.6	20.93
20027853	2011	5	23	3230	111.73	20.94
20027853	2011	5	23	3240	111.25	20.94
20027853	2011	5	23	3250	111.6	20.94
20027853	2011	5	23	3260	112.43	20.94
20027853	2011	5	23	3270	112.61	20.94
20027853	2011	5	23	3280	111.84	20.92
20027853	2011	5	23	3290	111.64	20.91
20027853	2011	5	23	3300	111.49	20.9
20027853	2011	5	23	3310	111.5	20.89
20027853	2011	5	23	3320	111.63	20.89
20027853	2011	5	23	3330	111.64	20.87
20027853	2011	5	23	3340	111.6	20.86
20027853	2011	5	23	3350	111.73	20.86
20027853	2011	5	23	3360	111.81	20.85
20027853	2011	5	23	3370	111.6	20.85
20027853	2011	5	23	3380	111.99	20.85
20027853	2011	5	23	3390	113.16	20.85
20027853	2011	5	23	3400	113.62	20.84
20027853	2011	5	23	3410	113.22	20.84
20027853	2011	5	23	3420	113.64	20.83
20027853	2011	5	23	3430	113.66	20.82
20027853	2011	5	23	3440	113.64	20.8
20027853	2011	5	23	3450	113.62	20.79
20027853	2011	5	23	3460	113.57	20.77
20027853	2011	5	23	3470	113.73	20.75
20027853	2011	5	23	3480	113.75	20.75
20027853	2011	5	23	3490	113.87	20.74
20027853	2011	5	23	3500	114.72	20.72
20027853	2011	5	23	3510	114.45	20.71
20027853	2011	5	23	3520	113.7	20.69
20027853	2011	5	23	3530	113.13	20.69
20027853	2011	5	23	3540	112.72	20.7
20027853	2011	5	23	3550	112.4	20.71
20027853	2011	5	23	3560	111.96	20.74
20027853	2011	5	23	3570	112.06	20.75
20027853	2011	5	23	3580	111.99	20.77
20027853	2011	5	23	3590	111.71	20.78

20027853	2011	5	23	3600	111.6	20.81
20027853	2011	5	23	3610	111.56	20.85
20027853	2011	5	23	3620	111.75	20.86
20027853	2011	5	23	3630	111.64	20.88
20027853	2011	5	23	3640	111.7	20.92
20027853	2011	5	23	3650	111.47	20.94
20027853	2011	5	23	3660	111.86	20.94
20027853	2011	5	23	3670	111.93	20.96
20027853	2011	5	23	3680	111.36	20.96
20027853	2011	5	23	3690	110.87	20.99
20027853	2011	5	23	3700	110.65	21
20027853	2011	5	23	3710	110.48	21.01
20027853	2011	5	23	3720	110.35	21.03
20027853	2011	5	23	3730	110.23	21.04
20027853	2011	5	23	3740	110.68	21.05
20027853	2011	5	23	3750	111.05	21.07
20027853	2011	5	23	3760	111.33	21.08
20027853	2011	5	23	3770	111.79	21.09
20027853	2011	5	23	3780	110.19	21.1
20027853	2011	5	23	3790	107.84	21.14
20027853	2011	5	23	3800	105.78	21.16
20027853	2011	5	23	3810	103.82	21.17
20027853	2011	5	23	3820	102.56	21.19
20027853	2011	5	23	3830	101.94	21.22
20027853	2011	5	23	3840	100.55	21.25
20027853	2011	5	23	3850	99.21	21.28
20027853	2011	5	23	3860	98.25	21.31
20027853	2011	5	23	3870	98.2	21.35
20027853	2011	5	23	3880	97.15	21.4
20027853	2011	5	23	3890	97.51	21.43
20027853	2011	5	23	3900	97.32	21.5
20027853	2011	5	23	3910	97.21	21.54
20027853	2011	5	23	3920	96.94	21.59
20027853	2011	5	23	3930	97.67	21.61
20027853	2011	5	23	3940	97.55	21.64
20027853	2011	5	23	3950	97.3	21.67
20027853	2011	5	23	3960	97.25	21.71
20027853	2011	5	23	3970	97.16	21.74
20027853	2011	5	23	3980	96.91	21.78
20027853	2011	5	23	3990	96.76	21.8
20027853	2011	5	23	4000	96.87	21.83
20027853	2011	5	23	4010	96.65	21.85
20027853	2011	5	23	4020	96.86	21.89
20027853	2011	5	23	4030	96.58	21.92
20027853	2011	5	23	4040	96.7	21.92
20027853	2011	5	23	4050	96.56	21.94
20027853	2011	5	23	4060	96.61	21.98

20027853	2011	5	23	4070	96.3	22
20027853	2011	5	23	4080	96.43	22.03
20027853	2011	5	23	4090	95.31	22.04
20027853	2011	5	23	4100	93.5	22.07
20027853	2011	5	23	4110	91.96	22.1
20027853	2011	5	23	4120	90.32	22.14
20027853	2011	5	23	4130	88.01	22.2
20027853	2011	5	23	4140	86.39	22.24
20027853	2011	5	23	4150	85.77	22.26
20027853	2011	5	23	4160	85.46	22.32
20027853	2011	5	23	4170	85.16	22.39
20027853	2011	5	23	4180	85.81	22.42
20027853	2011	5	23	4190	85.45	22.48
20027853	2011	5	23	4200	85.28	22.53
20027853	2011	5	23	4210	85.96	22.58
20027853	2011	5	23	4220	85.87	22.64
20027853	2011	5	23	4230	85.79	22.68
20027853	2011	5	23	4240	85.49	22.73
20027853	2011	5	23	4250	84.99	22.78
20027853	2011	5	23	4260	84.93	22.82
20027853	2011	5	23	4270	84.29	22.89
20027853	2011	5	23	4280	83.5	22.93
20027853	2011	5	23	4290	82.28	22.99
20027853	2011	5	23	4300	81.2	23.05
20027853	2011	5	23	4310	80.81	23.09
20027853	2011	5	23	4320	81.08	23.14
20027853	2011	5	23	4330	81.25	23.19
20027853	2011	5	23	4340	81.04	23.23
20027853	2011	5	23	4350	81.04	23.27
20027853	2011	5	23	4360	81.22	23.31
20027853	2011	5	23	4370	81.15	23.35
20027853	2011	5	23	4380	81.07	23.39
20027853	2011	5	23	4390	81.37	23.44
20027853	2011	5	23	4400	81.42	23.49
20027853	2011	5	23	4410	81.67	23.53
20027853	2011	5	23	4420	81.6	23.57
20027853	2011	5	23	4430	81.66	23.6
20027853	2011	5	23	4440	80.92	23.65
20027853	2011	5	23	4450	81.7	23.71
20027853	2011	5	23	4460	81.29	23.74
20027853	2011	5	23	4470	79.94	23.78
20027853	2011	5	23	4480	78.65	23.81
20027853	2011	5	23	4490	78.3	23.84
20027853	2011	5	23	4500	77.96	23.89
20027853	2011	5	23	4510	78.18	23.91
20027853	2011	5	23	4520	78.12	23.94
20027853	2011	5	23	4530	77.86	23.96

20027853	2011	5	23	4540	77.61	23.98
20027853	2011	5	23	4550	78.27	24
20027853	2011	5	23	4560	78.39	24.03
20027853	2011	5	23	4570	78.21	24.05
20027853	2011	5	23	4580	77.67	24.07
20027853	2011	5	23	4590	76.84	24.14
20027853	2011	5	23	4600	76.48	24.16
20027853	2011	5	23	4610	76.58	24.2
20027853	2011	5	23	4620	76.65	24.23
20027853	2011	5	23	4630	76.63	24.24
20027853	2011	5	23	4640	76.49	24.26
20027853	2011	5	23	4650	76.65	24.28
20027853	2011	5	23	4660	76.43	24.31
20027853	2011	5	23	4670	76.3	24.34
20027853	2011	5	23	4680	76.33	24.36
20027853	2011	5	23	4690	76.76	24.37
20027853	2011	5	23	4700	76.74	24.39
20027853	2011	5	23	4710	76.9	24.4
20027853	2011	5	23	4720	76.39	24.44
20027853	2011	5	23	4730	76.65	24.46
20027853	2011	5	23	4740	76.56	24.46
20027853	2011	5	23	4750	76.92	24.48
20027853	2011	5	23	4760	76.74	24.49
20027853	2011	5	23	4770	77.05	24.5
20027853	2011	5	23	4780	77.15	24.5
20027853	2011	5	23	4790	77.11	24.52
20027853	2011	5	23	4800	76.51	24.53
20027853	2011	5	23	4810	76.87	24.55
20027853	2011	5	23	4820	77.65	24.57
20027853	2011	5	23	4830	76.91	24.58
20027853	2011	5	23	4840	76.39	24.6
20027853	2011	5	23	4850	76.54	24.6
20027853	2011	5	23	4860	76.09	24.61
20027853	2011	5	23	4870	74.98	24.63
20027853	2011	5	23	4880	74.08	24.64
20027853	2011	5	23	4890	72.15	24.67
20027853	2011	5	23	4900	71.57	24.71
20027853	2011	5	23	4910	71.37	24.8
20027853	2011	5	23	4920	71.27	24.82
20027853	2011	5	23	4930	71.67	24.87
20027853	2011	5	23	4940	72	24.89
20027853	2011	5	23	4950	71.79	24.92
20027853	2011	5	23	4960	71.46	24.94
20027853	2011	5	23	4970	71.74	24.97
20027853	2011	5	23	4980	71.91	25.03
20027853	2011	5	23	4990	71.83	25.07
20027853	2011	5	23	5000	71.9	25.09

20027853	2011	5	23	5010	72.24	25.11
20027853	2011	5	23	5020	72.41	25.14
20027853	2011	5	23	5030	72.17	25.19
20027853	2011	5	23	5040	71.01	25.22
20027853	2011	5	23	5050	69.39	25.27
20027853	2011	5	23	5060	67.54	25.32
20027853	2011	5	23	5070	65.7	25.39
20027853	2011	5	23	5080	64.9	25.45
20027853	2011	5	23	5090	63.03	25.5
20027853	2011	5	23	5100	61.71	25.54
20027853	2011	5	23	5110	59.88	25.59
20027853	2011	5	23	5120	58.34	25.64
20027853	2011	5	23	5130	56	25.71
20027853	2011	5	23	5140	54.45	25.76
20027853	2011	5	23	5150	52.38	25.83
20027853	2011	5	23	5160	50.21	25.91
20027853	2011	5	23	5170	48.35	25.97
20027853	2011	5	23	5180	47.35	26.03
20027853	2011	5	23	5190	45.71	26.1
20027853	2011	5	23	5200	45.23	26.16
20027853	2011	5	23	5210	43.79	26.23
20027853	2011	5	23	5220	42.69	26.28
20027853	2011	5	23	5230	41	26.35
20027853	2011	5	23	5240	39.58	26.42
20027853	2011	5	23	5250	39.4	26.46
20027853	2011	5	23	5260	38.79	26.54
20027853	2011	5	23	5270	38.23	26.59
20027853	2011	5	23	5280	38.12	26.61
20027853	2011	5	23	5290	37.47	26.64
20027853	2011	5	23	5300	36.74	26.75
20027853	2011	5	23	5310	36.44	26.78
20027853	2011	5	23	5320	36.38	26.88
20027853	2011	5	23	5330	36.25	26.96
20027853	2011	5	23	5340	36.44	27.01
20027853	2011	5	23	5350	36.51	27.07
20027853	2011	5	23	5360	36.76	27.11
20027853	2011	5	23	5370	37.02	27.17
20027853	2011	5	23	5380	35.82	27.19
20027853	2011	5	23	5390	34.02	27.24
20027853	2011	5	23	5400	31.93	27.28
20027853	2011	5	23	5410	29.95	27.32
20027853	2011	5	23	5420	27.53	27.37
20027853	2011	5	23	5430	25.34	27.43
20027853	2011	5	23	5440	23.09	27.48
20027853	2011	5	23	5450	21.38	27.53
20027853	2011	5	23	5460	20.36	27.57
20027853	2011	5	23	5470	18.13	27.63

20027853	2011	5	23	5480	16.53	27.67
20027853	2011	5	23	5490	16.6	27.73
20027853	2011	5	23	5500	16.38	27.77
20027853	2011	5	23	5510	15.96	27.8
20027853	2011	5	23	5520	15.37	27.82
20027853	2011	5	23	5530	13.5	27.87
20027853	2011	5	23	5540	11.08	27.93
20027853	2011	5	23	5550	8.66	27.95
20027853	2011	5	23	5560	6.26	27.99
20027853	2011	5	23	5570	4.21	28.03
20027853	2011	5	23	5580	2.68	28.07
20027853	2011	5	23	5590	2.28	28.1
20027853	2011	5	23	5600	2.2	28.11
20027853	2011	5	23	5610	2.27	28.14
20027853	2011	5	23	5620	2.14	28.17
20027853	2011	5	23	5630	2.25	28.19
20027853	2011	5	23	5640	1.66	28.22
20027853	2011	5	23	5650	0.67	28.25
20027853	2011	5	23	5660	0.65	28.28
20027853	2011	5	23	5670	0.67	28.29
20027853	2011	5	23	5680	0.62	28.32
20027853	2011	5	23	5690	0.65	28.33
20027853	2011	5	23	5700	0.69	28.35
20027853	2011	5	23	5710	0.65	28.38
20027853	2011	5	23	5720	0.63	28.4
20027853	2011	5	23	5730	0.63	28.42
20027853	2011	5	23	5740	0.63	28.43
20027853	2011	5	23	5750	0.62	28.46
20027853	2011	5	23	5760	0.66	28.48
20027853	2011	5	23	5770	0.64	28.5
20027853	2011	5	23	5780	0.64	28.52
20027853	2011	5	23	5790	0.51	28.53
20038703	2011	5	23	0	1.43	28.92
20038703	2011	5	23	10	2.31	28.92
20038703	2011	5	23	20	2.52	28.92
20038703	2011	5	23	30	2.45	28.92
20038703	2011	5	23	40	2.79	28.92
20038703	2011	5	23	50	3.34	28.92
20038703	2011	5	23	60	4.67	28.89
20038703	2011	5	23	70	6.06	28.85
20038703	2011	5	23	80	6.89	28.84
20038703	2011	5	23	90	7.72	28.83
20038703	2011	5	23	100	9.74	28.78
20038703	2011	5	23	110	12.02	28.74
20038703	2011	5	23	120	14.88	28.69
20038703	2011	5	23	130	18.03	28.64
20038703	2011	5	23	140	21.52	28.61

20038703	2011	5	23	150	25.17	28.57
20038703	2011	5	23	160	28.62	28.51
20038703	2011	5	23	170	31.28	28.49
20038703	2011	5	23	180	34.22	28.45
20038703	2011	5	23	190	37.27	28.42
20038703	2011	5	23	200	40.35	28.38
20038703	2011	5	23	210	43.42	28.34
20038703	2011	5	23	220	46.37	28.25
20038703	2011	5	23	230	48.76	28.16
20038703	2011	5	23	240	51.3	28.05
20038703	2011	5	23	250	53.93	27.97
20038703	2011	5	23	260	56.58	27.92
20038703	2011	5	23	270	59.34	27.85
20038703	2011	5	23	280	61.85	27.81
20038703	2011	5	23	290	63.63	27.78
20038703	2011	5	23	300	66.07	27.67
20038703	2011	5	23	310	68.67	27.6
20038703	2011	5	23	320	70	27.55
20038703	2011	5	23	330	70.89	27.53
20038703	2011	5	23	340	72.92	27.47
20038703	2011	5	23	350	74.07	27.4
20038703	2011	5	23	360	75.37	27.35
20038703	2011	5	23	370	76.57	27.28
20038703	2011	5	23	380	77.15	27.23
20038703	2011	5	23	390	78.08	27.14
20038703	2011	5	23	400	79	27.07
20038703	2011	5	23	410	79.62	27.02
20038703	2011	5	23	420	80.06	26.96
20038703	2011	5	23	430	81.57	26.84
20038703	2011	5	23	440	82.2	26.76
20038703	2011	5	23	450	81.74	26.7
20038703	2011	5	23	460	81.53	26.65
20038703	2011	5	23	470	81.65	26.59
20038703	2011	5	23	480	81.9	26.51
20038703	2011	5	23	490	82.19	26.42
20038703	2011	5	23	500	82.77	26.35
20038703	2011	5	23	510	83.57	26.28
20038703	2011	5	23	520	83.98	26.19
20038703	2011	5	23	530	84.45	26.1
20038703	2011	5	23	540	84.93	26.03
20038703	2011	5	23	550	84.89	25.96
20038703	2011	5	23	560	84.8	25.91
20038703	2011	5	23	570	85.38	25.83
20038703	2011	5	23	580	85.98	25.77
20038703	2011	5	23	590	86.16	25.72
20038703	2011	5	23	600	86.77	25.65
20038703	2011	5	23	610	87.51	25.59

20038703	2011	5	23	620	87.87	25.53
20038703	2011	5	23	630	87.92	25.47
20038703	2011	5	23	640	88.24	25.4
20038703	2011	5	23	650	88.99	25.34
20038703	2011	5	23	660	89.33	25.28
20038703	2011	5	23	670	89.43	25.22
20038703	2011	5	23	680	89.32	25.15
20038703	2011	5	23	690	89.62	25.1
20038703	2011	5	23	700	89.69	25.05
20038703	2011	5	23	710	89.83	25
20038703	2011	5	23	720	89.68	24.96
20038703	2011	5	23	730	89.32	24.9
20038703	2011	5	23	740	89.19	24.86
20038703	2011	5	23	750	89.62	24.81
20038703	2011	5	23	760	90.49	24.75
20038703	2011	5	23	770	91.5	24.7
20038703	2011	5	23	780	92.78	24.61
20038703	2011	5	23	790	94.01	24.56
20038703	2011	5	23	800	95.62	24.42
20038703	2011	5	23	810	96.37	24.39
20038703	2011	5	23	820	97.7	24.3
20038703	2011	5	23	830	99.3	24.25
20038703	2011	5	23	840	99.94	24.22
20038703	2011	5	23	850	100.54	24.16
20038703	2011	5	23	860	100.97	24.09
20038703	2011	5	23	870	101.41	24
20038703	2011	5	23	880	101.43	23.95
20038703	2011	5	23	890	101.49	23.88
20038703	2011	5	23	900	101.62	23.79
20038703	2011	5	23	910	101.44	23.69
20038703	2011	5	23	920	101.67	23.58
20038703	2011	5	23	930	101.78	23.49
20038703	2011	5	23	940	101.31	23.38
20038703	2011	5	23	950	101.92	23.28
20038703	2011	5	23	960	101.93	23.21
20038703	2011	5	23	970	101.86	23.14
20038703	2011	5	23	980	101.68	23.08
20038703	2011	5	23	990	101.54	23.03
20038703	2011	5	23	1000	101.43	22.97
20038703	2011	5	23	1010	101.77	22.89
20038703	2011	5	23	1020	101.68	22.84
20038703	2011	5	23	1030	102.06	22.75
20038703	2011	5	23	1040	101.75	22.67
20038703	2011	5	23	1050	101.81	22.64
20038703	2011	5	23	1060	101.7	22.61
20038703	2011	5	23	1070	101.79	22.57
20038703	2011	5	23	1080	102.45	22.52

20038703	2011	5	23	1090	103.23	22.49
20038703	2011	5	23	1100	104.03	22.43
20038703	2011	5	23	1110	105.36	22.39
20038703	2011	5	23	1120	106.32	22.34
20038703	2011	5	23	1130	108.3	22.26
20038703	2011	5	23	1140	109.37	22.24
20038703	2011	5	23	1150	110.39	22.22
20038703	2011	5	23	1160	110.97	22.18
20038703	2011	5	23	1170	111.65	22.14
20038703	2011	5	23	1180	112.16	22.11
20038703	2011	5	23	1190	112.99	22.07
20038703	2011	5	23	1200	113.96	22.01
20038703	2011	5	23	1210	115.42	21.98
20038703	2011	5	23	1220	116.05	21.96
20038703	2011	5	23	1230	116.32	21.94
20038703	2011	5	23	1240	116.83	21.92
20038703	2011	5	23	1250	116.79	21.91
20038703	2011	5	23	1260	117.37	21.86
20038703	2011	5	23	1270	118.35	21.82
20038703	2011	5	23	1280	118.49	21.81
20038703	2011	5	23	1290	118.44	21.8
20038703	2011	5	23	1300	117.58	21.78
20038703	2011	5	23	1310	117.16	21.76
20038703	2011	5	23	1320	117.28	21.75
20038703	2011	5	23	1330	118.29	21.71
20038703	2011	5	23	1340	117.95	21.69
20038703	2011	5	23	1350	118	21.69
20038703	2011	5	23	1360	117.91	21.67
20038703	2011	5	23	1370	117.87	21.65
20038703	2011	5	23	1380	118.48	21.64
20038703	2011	5	23	1390	118.44	21.64
20038703	2011	5	23	1400	118.24	21.61
20038703	2011	5	23	1410	118.58	21.6
20038703	2011	5	23	1420	118.46	21.59
20038703	2011	5	23	1430	118.07	21.59
20038703	2011	5	23	1440	118.87	21.56
20038703	2011	5	23	1450	118.76	21.54
20038703	2011	5	23	1460	118.41	21.54
20038703	2011	5	23	1470	118.68	21.53
20038703	2011	5	23	1480	118.65	21.52
20038703	2011	5	23	1490	118.22	21.52
20038703	2011	5	23	1500	117.43	21.51
20038703	2011	5	23	1510	117.15	21.5
20038703	2011	5	23	1520	117.4	21.5
20038703	2011	5	23	1530	117.66	21.48
20038703	2011	5	23	1540	118.06	21.48
20038703	2011	5	23	1550	118.41	21.47

20038703	2011	5	23	1560	118.23	21.46
20038703	2011	5	23	1570	119.04	21.45
20038703	2011	5	23	1580	119.72	21.44
20038703	2011	5	23	1590	120.09	21.44
20038703	2011	5	23	1600	120.4	21.42
20038703	2011	5	23	1610	120.34	21.4
20038703	2011	5	23	1620	120.29	21.4
20038703	2011	5	23	1630	120.87	21.39
20038703	2011	5	23	1640	121.11	21.37
20038703	2011	5	23	1650	120.89	21.37
20038703	2011	5	23	1660	120.85	21.36
20038703	2011	5	23	1670	120.73	21.36
20038703	2011	5	23	1680	120.77	21.35
20038703	2011	5	23	1690	120.71	21.35
20038703	2011	5	23	1700	120.79	21.34
20038703	2011	5	23	1710	121	21.33
20038703	2011	5	23	1720	121.71	21.32
20038703	2011	5	23	1730	121.98	21.31
20038703	2011	5	23	1740	121.03	21.3
20038703	2011	5	23	1750	120.85	21.3
20038703	2011	5	23	1760	120.64	21.29
20038703	2011	5	23	1770	120.15	21.29
20038703	2011	5	23	1780	120.1	21.28
20038703	2011	5	23	1790	119.77	21.28
20038703	2011	5	23	1800	119.65	21.28
20038703	2011	5	23	1810	119.73	21.28
20038703	2011	5	23	1820	119.62	21.28
20038703	2011	5	23	1830	120.05	21.28
20038703	2011	5	23	1840	122.03	21.28
20038703	2011	5	23	1850	123.13	21.27
20038703	2011	5	23	1860	124.21	21.27
20038703	2011	5	23	1870	124.96	21.26
20038703	2011	5	23	1880	125.94	21.25
20038703	2011	5	23	1890	127.56	21.23
20038703	2011	5	23	1900	128.54	21.22
20038703	2011	5	23	1910	129.31	21.18
20038703	2011	5	23	1920	129.53	21.16
20038703	2011	5	23	1930	130.36	21.11
20038703	2011	5	23	1940	130.24	21.06
20038703	2011	5	23	1950	130.41	21.03
20038703	2011	5	23	1960	130.39	21
20038703	2011	5	23	1970	130.16	20.98
20038703	2011	5	23	1980	130.15	20.92
20038703	2011	5	23	1990	130.28	20.89
20038703	2011	5	23	2000	130.26	20.86
20038703	2011	5	23	2010	130.05	20.83
20038703	2011	5	23	2020	129.72	20.82

20038703	2011	5	23	2030	130.21	20.78
20038703	2011	5	23	2040	130.11	20.74
20038703	2011	5	23	2050	130.1	20.71
20038703	2011	5	23	2060	129.93	20.69
20038703	2011	5	23	2070	130.01	20.66
20038703	2011	5	23	2080	130.29	20.64
20038703	2011	5	23	2090	130.01	20.62
20038703	2011	5	23	2100	129.63	20.6
20038703	2011	5	23	2110	130.03	20.57
20038703	2011	5	23	2120	129.73	20.53
20038703	2011	5	23	2130	129.34	20.52
20038703	2011	5	23	2140	129.54	20.49
20038703	2011	5	23	2150	130.21	20.46
20038703	2011	5	23	2160	131.28	20.44
20038703	2011	5	23	2170	132.69	20.4
20038703	2011	5	23	2180	133.38	20.39
20038703	2011	5	23	2190	134.19	20.34
20038703	2011	5	23	2200	135.07	20.3
20038703	2011	5	23	2210	135.31	20.28
20038703	2011	5	23	2220	136.3	20.25
20038703	2011	5	23	2230	136.59	20.22
20038703	2011	5	23	2240	136.79	20.18
20038703	2011	5	23	2250	137.46	20.14
20038703	2011	5	23	2260	137.38	20.11
20038703	2011	5	23	2270	136.81	20.1
20038703	2011	5	23	2280	136.52	20.08
20038703	2011	5	23	2290	136.17	20.06
20038703	2011	5	23	2300	136.02	20.03
20038703	2011	5	23	2310	135.88	20.02
20038703	2011	5	23	2320	135.28	19.99
20038703	2011	5	23	2330	134.62	19.97
20038703	2011	5	23	2340	133.94	19.96
20038703	2011	5	23	2350	133.25	19.94
20038703	2011	5	23	2360	133.91	19.9
20038703	2011	5	23	2370	134.39	19.89
20038703	2011	5	23	2380	134.48	19.89
20038703	2011	5	23	2390	134.44	19.87
20038703	2011	5	23	2400	134.26	19.87
20038703	2011	5	23	2410	133.9	19.87
20038703	2011	5	23	2420	133.87	19.86
20038703	2011	5	23	2430	134.63	19.85
20038703	2011	5	23	2440	135.18	19.85
20038703	2011	5	23	2450	136.02	19.85
20038703	2011	5	23	2460	136.17	19.85
20038703	2011	5	23	2470	136.02	19.86
20038703	2011	5	23	2480	136.44	19.85
20038703	2011	5	23	2490	136.48	19.85

20038703	2011	5	23	2500	136.54	19.85
20038703	2011	5	23	2510	137.04	19.86
20038703	2011	5	23	2520	136.66	19.86
20038703	2011	5	23	2530	136.98	19.86
20038703	2011	5	23	2540	137.15	19.86
20038703	2011	5	23	2550	136.94	19.86
20038703	2011	5	23	2560	136.71	19.86
20038703	2011	5	23	2570	136.48	19.86
20038703	2011	5	23	2580	136.63	19.86
20038703	2011	5	23	2590	136.61	19.86
20038703	2011	5	23	2600	136.63	19.87
20038703	2011	5	23	2610	136.39	19.87
20038703	2011	5	23	2620	135.99	19.88
20038703	2011	5	23	2630	136.08	19.88
20038703	2011	5	23	2640	136.25	19.89
20038703	2011	5	23	2650	136.41	19.88
20038703	2011	5	23	2660	136.49	19.89
20038703	2011	5	23	2670	136.68	19.89
20038703	2011	5	23	2680	136.46	19.89
20038703	2011	5	23	2690	136.45	19.91
20038703	2011	5	23	2700	136.95	19.92
20038703	2011	5	23	2710	137.66	19.92
20038703	2011	5	23	2720	137.8	19.93
20038703	2011	5	23	2730	137.71	19.94
20038703	2011	5	23	2740	137.4	19.94
20038703	2011	5	23	2750	137.29	19.96
20038703	2011	5	23	2760	137.93	19.96
20038703	2011	5	23	2770	137.78	19.96
20038703	2011	5	23	2780	137.87	19.96
20038703	2011	5	23	2790	137.73	19.96
20038703	2011	5	23	2800	137.43	19.96
20038703	2011	5	23	2810	137.51	19.96
20038703	2011	5	23	2820	137.26	19.96
20038703	2011	5	23	2830	136.67	19.96
20038703	2011	5	23	2840	136.5	19.97
20038703	2011	5	23	2850	137.41	19.98
20038703	2011	5	23	2860	138.47	19.98
20038703	2011	5	23	2870	138.83	19.99
20038703	2011	5	23	2880	138.77	19.99
20038703	2011	5	23	2890	138.65	20
20038703	2011	5	23	2900	138.68	20
20038703	2011	5	23	2910	138.5	20
20038703	2011	5	23	2920	138.41	20
20038703	2011	5	23	2930	138.47	20
20038703	2011	5	23	2940	138.54	20
20038703	2011	5	23	2950	138.45	20.01
20038703	2011	5	23	2960	138.48	20.01

20038703	2011	5	23	2970	139.13	20.03
20038703	2011	5	23	2980	139.03	20.03
20038703	2011	5	23	2990	138.54	20.03
20038703	2011	5	23	3000	138.47	20.03
20038703	2011	5	23	3010	138.58	20.03
20038703	2011	5	23	3020	138.61	20.04
20038703	2011	5	23	3030	138.31	20.05
20038703	2011	5	23	3040	138.36	20.05
20038703	2011	5	23	3050	138.51	20.05
20038703	2011	5	23	3060	138.06	20.05
20038703	2011	5	23	3070	137.22	20.06
20038703	2011	5	23	3080	136.62	20.07
20038703	2011	5	23	3090	137.86	20.07
20038703	2011	5	23	3100	138.81	20.07
20038703	2011	5	23	3110	139.02	20.07
20038703	2011	5	23	3120	138.9	20.07
20038703	2011	5	23	3130	138.64	20.07
20038703	2011	5	23	3140	138.67	20.07
20038703	2011	5	23	3150	138.3	20.07
20038703	2011	5	23	3160	138.2	20.08
20038703	2011	5	23	3170	138.63	20.09
20038703	2011	5	23	3180	138.65	20.08
20038703	2011	5	23	3190	138.54	20.09
20038703	2011	5	23	3200	138.92	20.08
20038703	2011	5	23	3210	139.54	20.08
20038703	2011	5	23	3220	139.64	20.08
20038703	2011	5	23	3230	139.89	20.07
20038703	2011	5	23	3240	139.72	20.07
20038703	2011	5	23	3250	139.87	20.06
20038703	2011	5	23	3260	139.74	20.05
20038703	2011	5	23	3270	140.12	20.04
20038703	2011	5	23	3280	140.87	20.03
20038703	2011	5	23	3290	140.6	20.03
20038703	2011	5	23	3300	140.33	20.03
20038703	2011	5	23	3310	140.41	20.03
20038703	2011	5	23	3320	140.27	20.02
20038703	2011	5	23	3330	140.49	20.01
20038703	2011	5	23	3340	140.23	20.01
20038703	2011	5	23	3350	140.11	20
20038703	2011	5	23	3360	140.1	19.99
20038703	2011	5	23	3370	140.8	19.97
20038703	2011	5	23	3380	140.35	19.96
20038703	2011	5	23	3390	140.34	19.95
20038703	2011	5	23	3400	140.95	19.92
20038703	2011	5	23	3410	140.97	19.9
20038703	2011	5	23	3420	140.8	19.89
20038703	2011	5	23	3430	140.59	19.89

20038703	2011	5	23	3440	140.56	19.86
20038703	2011	5	23	3450	141.22	19.85
20038703	2011	5	23	3460	141.51	19.84
20038703	2011	5	23	3470	141.49	19.82
20038703	2011	5	23	3480	141.64	19.81
20038703	2011	5	23	3490	141.33	19.79
20038703	2011	5	23	3500	141.16	19.77
20038703	2011	5	23	3510	141.42	19.75
20038703	2011	5	23	3520	141.63	19.74
20038703	2011	5	23	3530	142.28	19.72
20038703	2011	5	23	3540	142.58	19.7
20038703	2011	5	23	3550	142.53	19.68
20038703	2011	5	23	3560	142.65	19.67
20038703	2011	5	23	3570	142.96	19.65
20038703	2011	5	23	3580	142.84	19.64
20038703	2011	5	23	3590	142.97	19.61
20038703	2011	5	23	3600	142.96	19.59
20038703	2011	5	23	3610	142.76	19.57
20038703	2011	5	23	3620	142.56	19.55
20038703	2011	5	23	3630	142.83	19.53
20038703	2011	5	23	3640	142.89	19.52
20038703	2011	5	23	3650	143.67	19.51
20038703	2011	5	23	3660	143.53	19.5
20038703	2011	5	23	3670	143.31	19.5
20038703	2011	5	23	3680	143.33	19.49
20038703	2011	5	23	3690	143.18	19.48
20038703	2011	5	23	3700	143.5	19.48
20038703	2011	5	23	3710	143.3	19.47
20038703	2011	5	23	3720	143.01	19.46
20038703	2011	5	23	3730	143.06	19.46
20038703	2011	5	23	3740	143.11	19.46
20038703	2011	5	23	3750	143.2	19.45
20038703	2011	5	23	3760	143.1	19.45
20038703	2011	5	23	3770	142.74	19.44
20038703	2011	5	23	3780	143.13	19.44
20038703	2011	5	23	3790	143.41	19.44
20038703	2011	5	23	3800	143.64	19.42
20038703	2011	5	23	3810	143.64	19.42
20038703	2011	5	23	3820	143.91	19.42
20038703	2011	5	23	3830	144.24	19.42
20038703	2011	5	23	3840	144.53	19.41
20038703	2011	5	23	3850	144.58	19.41
20038703	2011	5	23	3860	144.53	19.4
20038703	2011	5	23	3870	144.75	19.39
20038703	2011	5	23	3880	144.5	19.39
20038703	2011	5	23	3890	144.59	19.37
20038703	2011	5	23	3900	144.18	19.35

20038703	2011	5	23	3910	143.35	19.35
20038703	2011	5	23	3920	143.25	19.34
20038703	2011	5	23	3930	142.66	19.33
20038703	2011	5	23	3940	141.84	19.32
20038703	2011	5	23	3950	141.22	19.32
20038703	2011	5	23	3960	141.38	19.31
20038703	2011	5	23	3970	141.44	19.31
20038703	2011	5	23	3980	141.1	19.32
20038703	2011	5	23	3990	140.47	19.32
20038703	2011	5	23	4000	139.73	19.32
20038703	2011	5	23	4010	140.11	19.32
20038703	2011	5	23	4020	140.37	19.33
20038703	2011	5	23	4030	140.92	19.34
20038703	2011	5	23	4040	140.96	19.34
20038703	2011	5	23	4050	140.66	19.35
20038703	2011	5	23	4060	141.21	19.35
20038703	2011	5	23	4070	141.45	19.35
20038703	2011	5	23	4080	141.44	19.35
20038703	2011	5	23	4090	141.85	19.36
20038703	2011	5	23	4100	141.96	19.35
20038703	2011	5	23	4110	141.92	19.36
20038703	2011	5	23	4120	141.7	19.36
20038703	2011	5	23	4130	141.33	19.37
20038703	2011	5	23	4140	141.31	19.37
20038703	2011	5	23	4150	140.73	19.38
20038703	2011	5	23	4160	139.88	19.38
20038703	2011	5	23	4170	139.47	19.38
20038703	2011	5	23	4180	139.16	19.39
20038703	2011	5	23	4190	138.85	19.39
20038703	2011	5	23	4200	140.12	19.39
20038703	2011	5	23	4210	141.65	19.39
20038703	2011	5	23	4220	142.63	19.39
20038703	2011	5	23	4230	142.65	19.39
20038703	2011	5	23	4240	142.81	19.39
20038703	2011	5	23	4250	142.88	19.39
20038703	2011	5	23	4260	143.38	19.39
20038703	2011	5	23	4270	143.47	19.39
20038703	2011	5	23	4280	143.84	19.39
20038703	2011	5	23	4290	143.6	19.39
20038703	2011	5	23	4300	143.41	19.39
20038703	2011	5	23	4310	143.16	19.38
20038703	2011	5	23	4320	143.21	19.38
20038703	2011	5	23	4330	143.18	19.38
20038703	2011	5	23	4340	143.25	19.38
20038703	2011	5	23	4350	143.32	19.37
20038703	2011	5	23	4360	144.38	19.36
20038703	2011	5	23	4370	144.38	19.35

20038703	2011	5	23	4380	143.98	19.36
20038703	2011	5	23	4390	143.47	19.35
20038703	2011	5	23	4400	142.62	19.36
20038703	2011	5	23	4410	142.56	19.36
20038703	2011	5	23	4420	142.7	19.35
20038703	2011	5	23	4430	142.81	19.36
20038703	2011	5	23	4440	142.46	19.35
20038703	2011	5	23	4450	141.93	19.35
20038703	2011	5	23	4460	140.33	19.36
20038703	2011	5	23	4470	139.68	19.35
20038703	2011	5	23	4480	139.3	19.36
20038703	2011	5	23	4490	138.29	19.36
20038703	2011	5	23	4500	137.9	19.38
20038703	2011	5	23	4510	137.8	19.4
20038703	2011	5	23	4520	137.81	19.42
20038703	2011	5	23	4530	137.74	19.44
20038703	2011	5	23	4540	137.63	19.44
20038703	2011	5	23	4550	138.04	19.48
20038703	2011	5	23	4560	138.04	19.49
20038703	2011	5	23	4570	137.95	19.5
20038703	2011	5	23	4580	139.01	19.52
20038703	2011	5	23	4590	140.19	19.51
20038703	2011	5	23	4600	141.49	19.51
20038703	2011	5	23	4610	142.39	19.5
20038703	2011	5	23	4620	142.98	19.48
20038703	2011	5	23	4630	143.6	19.48
20038703	2011	5	23	4640	143.52	19.46
20038703	2011	5	23	4650	144.3	19.43
20038703	2011	5	23	4660	144.9	19.42
20038703	2011	5	23	4670	145.59	19.37
20038703	2011	5	23	4680	146.11	19.37
20038703	2011	5	23	4690	146.41	19.35
20038703	2011	5	23	4700	146.04	19.32
20038703	2011	5	23	4710	145.57	19.29
20038703	2011	5	23	4720	146.08	19.25
20038703	2011	5	23	4730	146.59	19.23
20038703	2011	5	23	4740	147.73	19.17
20038703	2011	5	23	4750	149.3	19.12
20038703	2011	5	23	4760	149.8	19.09
20038703	2011	5	23	4770	150.13	19.07
20038703	2011	5	23	4780	150.83	19
20038703	2011	5	23	4790	150.77	18.87
20038703	2011	5	23	4800	150.43	18.8
20038703	2011	5	23	4810	150.92	18.74
20038703	2011	5	23	4820	152.69	18.67
20038703	2011	5	23	4830	153.7	18.57
20038703	2011	5	23	4840	153.85	18.53

20038703	2011	5	23	4850	154	18.48
20038703	2011	5	23	4860	154.38	18.42
20038703	2011	5	23	4870	154.86	18.35
20038703	2011	5	23	4880	155.36	18.3
20038703	2011	5	23	4890	155.55	18.25
20038703	2011	5	23	4900	156.03	18.17
20038703	2011	5	23	4910	156.17	18.13
20038703	2011	5	23	4920	156.15	18.07
20038703	2011	5	23	4930	155.84	18.05
20038703	2011	5	23	4940	155.57	18.01
20038703	2011	5	23	4950	156.18	17.95
20038703	2011	5	23	4960	156.25	17.91
20038703	2011	5	23	4970	156.14	17.89
20038703	2011	5	23	4980	155.55	17.86
20038703	2011	5	23	4990	154.88	17.83
20038703	2011	5	23	5000	154.4	17.81
20038703	2011	5	23	5010	155.39	17.72
20038703	2011	5	23	5020	156.67	17.69
20038703	2011	5	23	5030	158.58	17.62
20038703	2011	5	23	5040	159.49	17.6
20038703	2011	5	23	5050	160.58	17.6
20038703	2011	5	23	5060	161.57	17.56
20038703	2011	5	23	5070	161.44	17.56
20038703	2011	5	23	5080	161.89	17.55
20038703	2011	5	23	5090	162	17.51
20038703	2011	5	23	5100	162.82	17.49
20038703	2011	5	23	5110	163.49	17.46
20038703	2011	5	23	5120	164.5	17.46
20038703	2011	5	23	5130	164.71	17.42
20038703	2011	5	23	5140	165.48	17.38
20038703	2011	5	23	5150	166.15	17.35
20038703	2011	5	23	5160	166.23	17.33
20038703	2011	5	23	5170	166.22	17.32
20038703	2011	5	23	5180	166.37	17.3
20038703	2011	5	23	5190	166.47	17.28
20038703	2011	5	23	5200	166.41	17.25
20038703	2011	5	23	5210	166.31	17.21
20038703	2011	5	23	5220	166.48	17.19
20038703	2011	5	23	5230	166.56	17.17
20038703	2011	5	23	5240	166.54	17.14
20038703	2011	5	23	5250	166.6	17.11
20038703	2011	5	23	5260	166.76	17.1
20038703	2011	5	23	5270	167.27	17.07
20038703	2011	5	23	5280	166.95	17.05
20038703	2011	5	23	5290	167.03	17.04
20038703	2011	5	23	5300	167.06	17.03
20038703	2011	5	23	5310	166.95	17.02

20038703	2011	5	23	5320	166.96	17.02
20038703	2011	5	23	5330	166.51	17
20038703	2011	5	23	5340	165.71	17
20038703	2011	5	23	5350	164.49	17
20038703	2011	5	23	5360	163.01	17
20038703	2011	5	23	5370	161.4	17
20038703	2011	5	23	5380	160	16.99
20038703	2011	5	23	5390	158.82	17
20038703	2011	5	23	5400	158.33	17
20038703	2011	5	23	5410	158.16	17
20038703	2011	5	23	5420	157.92	17.01
20038703	2011	5	23	5430	157.73	17
20038703	2011	5	23	5440	157.43	17.01
20038703	2011	5	23	5450	157.05	17.02
20038703	2011	5	23	5460	157.31	17.03
20038703	2011	5	23	5470	157.46	17.03
20038703	2011	5	23	5480	157.44	17.05
20038703	2011	5	23	5490	157.78	17.06
20038703	2011	5	23	5500	157.69	17.06
20038703	2011	5	23	5510	157.16	17.07
20038703	2011	5	23	5520	156.4	17.07
20038703	2011	5	23	5530	155.85	17.07
20038703	2011	5	23	5540	156.76	17.08
20038703	2011	5	23	5550	157.33	17.08
20038703	2011	5	23	5560	157.1	17.09
20038703	2011	5	23	5570	156.33	17.09
20038703	2011	5	23	5580	155.79	17.1
20038703	2011	5	23	5590	155.87	17.1
20038703	2011	5	23	5600	155.92	17.11
20038703	2011	5	23	5610	155.58	17.11
20038703	2011	5	23	5620	155.59	17.11
20038703	2011	5	23	5630	155.74	17.12
20038703	2011	5	23	5640	156	17.12
20038703	2011	5	23	5650	155.94	17.14
20038703	2011	5	23	5660	155.62	17.14
20038703	2011	5	23	5670	155.37	17.14
20038703	2011	5	23	5680	155.03	17.16
20038703	2011	5	23	5690	155.08	17.17
20038703	2011	5	23	5700	154.53	17.18
20038703	2011	5	23	5710	153.66	17.21
20038703	2011	5	23	5720	152.93	17.21
20038703	2011	5	23	5730	152.04	17.23
20038703	2011	5	23	5740	151.76	17.26
20038703	2011	5	23	5750	151.9	17.32
20038703	2011	5	23	5760	151.84	17.36
20038703	2011	5	23	5770	151.36	17.4
20038703	2011	5	23	5780	151.89	17.42

20038703	2011	5	23	5790	152.27	17.46
20038703	2011	5	23	5800	152.29	17.48
20038703	2011	5	23	5810	151.81	17.51
20038703	2011	5	23	5820	150.47	17.54
20038703	2011	5	23	5830	148.81	17.58
20038703	2011	5	23	5840	147.81	17.61
20038703	2011	5	23	5850	146.45	17.64
20038703	2011	5	23	5860	144.42	17.69
20038703	2011	5	23	5870	142.74	17.75
20038703	2011	5	23	5880	140.36	17.81
20038703	2011	5	23	5890	140.13	17.94
20038703	2011	5	23	5900	140.87	18.14
20038703	2011	5	23	5910	139.8	18.16
20038703	2011	5	23	5920	137.6	18.19
20038703	2011	5	23	5930	136.1	18.23
20038703	2011	5	23	5940	134.11	18.31
20038703	2011	5	23	5950	132.45	18.36
20038703	2011	5	23	5960	130.96	18.44
20038703	2011	5	23	5970	129.56	18.5
20038703	2011	5	23	5980	128.23	18.53
20038703	2011	5	23	5990	126.9	18.59
20038703	2011	5	23	6000	126.09	18.65
20038703	2011	5	23	6010	125.4	18.78
20038703	2011	5	23	6020	124.85	18.85
20038703	2011	5	23	6030	123.97	18.92
20038703	2011	5	23	6040	122.11	19
20038703	2011	5	23	6050	121.05	19.07
20038703	2011	5	23	6060	119.45	19.14
20038703	2011	5	23	6070	118.6	19.19
20038703	2011	5	23	6080	117.96	19.26
20038703	2011	5	23	6090	116.34	19.35
20038703	2011	5	23	6100	114.92	19.39
20038703	2011	5	23	6110	112.9	19.46
20038703	2011	5	23	6120	110.52	19.53
20038703	2011	5	23	6130	109.17	19.64
20038703	2011	5	23	6140	107.37	19.76
20038703	2011	5	23	6150	105.05	19.83
20038703	2011	5	23	6160	103.21	19.92
20038703	2011	5	23	6170	101.44	20.03
20038703	2011	5	23	6180	99.52	20.14
20038703	2011	5	23	6190	96.73	20.21
20038703	2011	5	23	6200	94.02	20.32
20038703	2011	5	23	6210	91	20.46
20038703	2011	5	23	6220	88.51	20.64
20038703	2011	5	23	6230	85.9	20.79
20038703	2011	5	23	6240	83.09	20.96
20038703	2011	5	23	6250	81.23	21.08

20038703	2011	5	23	6260	81.32	21.21
20038703	2011	5	23	6270	80.7	21.39
20038703	2011	5	23	6280	79.37	21.53
20038703	2011	5	23	6290	77.03	21.64
20038703	2011	5	23	6300	74.19	21.82
20038703	2011	5	23	6310	71.22	21.98
20038703	2011	5	23	6320	68.63	22.18
20038703	2011	5	23	6330	67.85	22.33
20038703	2011	5	23	6340	68.11	22.75
20038703	2011	5	23	6350	69.06	22.99
20038703	2011	5	23	6360	68.51	23.03
20038703	2011	5	23	6370	66.51	23.1
20038703	2011	5	23	6380	64.21	23.22
20038703	2011	5	23	6390	62.05	23.35
20038703	2011	5	23	6400	59.76	23.5
20038703	2011	5	23	6410	57.02	23.6
20038703	2011	5	23	6420	54.43	23.75
20038703	2011	5	23	6430	52.11	23.89
20038703	2011	5	23	6440	50.23	24.06
20038703	2011	5	23	6450	48.05	24.21
20038703	2011	5	23	6460	45.38	24.34
20038703	2011	5	23	6470	42.82	24.56
20038703	2011	5	23	6480	40.57	24.64
20038703	2011	5	23	6490	38.18	24.78
20038703	2011	5	23	6500	35.69	24.92
20038703	2011	5	23	6510	33.3	25.06
20038703	2011	5	23	6520	30.74	25.21
20038703	2011	5	23	6530	28.72	25.38
20038703	2011	5	23	6540	27.09	25.64
20038703	2011	5	23	6550	25.12	25.75
20038703	2011	5	23	6560	23.46	25.93
20038703	2011	5	23	6570	21.45	26.03
20038703	2011	5	23	6580	19.12	26.12
20038703	2011	5	23	6590	17.14	26.25
20038703	2011	5	23	6600	15.7	26.38
20038703	2011	5	23	6610	15.2	26.5
20038703	2011	5	23	6620	13.33	26.64
20038703	2011	5	23	6630	11.53	26.77
20038703	2011	5	23	6640	10.1	26.87
20038703	2011	5	23	6650	7.68	26.96
20038703	2011	5	23	6660	5.06	27.03
20038703	2011	5	23	6670	3.22	27.11
20038703	2011	5	23	6680	2.55	27.22
20038703	2011	5	23	6690	2.13	27.3
20038703	2011	5	23	6700	2.13	27.35
20038703	2011	5	23	6710	1.71	27.41
20038703	2011	5	23	6720	0.6	27.53

20038703	2011	5	23	6730	0.6	27.59
20038703	2011	5	23	6740	0.62	27.66
20038703	2011	5	23	6750	0.62	27.75
20038703	2011	5	23	6760	0.59	27.82
20038703	2011	5	23	6770	0.57	27.89
20038703	2011	5	23	6780	0.57	27.94
20038703	2011	5	23	6790	0.58	28
20038703	2011	5	23	6800	0.58	28.05
20038703	2011	5	23	6810	0.57	28.09
20038703	2011	5	23	6820	0.6	28.15
20038703	2011	5	23	6830	0.59	28.21
20038703	2011	5	23	6840	0.54	28.24
20038703	2011	5	23	6850	0.48	28.28
20038703	2011	5	23	6860	0.48	28.32
20125503	2011	5	24	0	1.01	28.99
20125503	2011	5	24	10	1.83	29
20125503	2011	5	24	20	2.2	28.98
20125503	2011	5	24	30	2.55	28.98
20125503	2011	5	24	40	2.68	28.98
20125503	2011	5	24	50	2.45	28.98
20125503	2011	5	24	60	2.67	28.98
20125503	2011	5	24	70	2.96	28.97
20125503	2011	5	24	80	4.22	28.95
20125503	2011	5	24	90	5.89	28.92
20125503	2011	5	24	100	8.18	28.89
20125503	2011	5	24	110	9.44	28.87
20125503	2011	5	24	120	10.41	28.85
20125503	2011	5	24	130	11.65	28.82
20125503	2011	5	24	140	12.92	28.8
20125503	2011	5	24	150	13.1	28.78
20125503	2011	5	24	160	13.31	28.78
20125503	2011	5	24	170	13.86	28.76
20125503	2011	5	24	180	14.12	28.75
20125503	2011	5	24	190	14.44	28.75
20125503	2011	5	24	200	14.37	28.73
20125503	2011	5	24	210	14.42	28.73
20125503	2011	5	24	220	14.37	28.72
20125503	2011	5	24	230	14.35	28.71
20125503	2011	5	24	240	14.34	28.71
20125503	2011	5	24	250	14.42	28.69
20125503	2011	5	24	260	15.12	28.68
20125503	2011	5	24	270	16.11	28.67
20125503	2011	5	24	280	16.94	28.65
20125503	2011	5	24	290	18.1	28.64
20125503	2011	5	24	300	20.37	28.59
20125503	2011	5	24	310	23	28.57
20125503	2011	5	24	320	26.04	28.52

20125503	2011	5	24	330	28.43	28.5
20125503	2011	5	24	340	30.61	28.46
20125503	2011	5	24	350	32.09	28.4
20125503	2011	5	24	360	33.92	28.37
20125503	2011	5	24	370	35.66	28.33
20125503	2011	5	24	380	37.27	28.29
20125503	2011	5	24	390	38.83	28.25
20125503	2011	5	24	400	41.57	28.09
20125503	2011	5	24	410	44.65	28
20125503	2011	5	24	420	47.74	27.95
20125503	2011	5	24	430	50.75	27.89
20125503	2011	5	24	440	54.04	27.71
20125503	2011	5	24	450	56.53	27.67
20125503	2011	5	24	460	59.15	27.54
20125503	2011	5	24	470	62.09	27.32
20125503	2011	5	24	480	64.9	27.26
20125503	2011	5	24	490	66.25	27.25
20125503	2011	5	24	500	67.65	27.22
20125503	2011	5	24	510	69.38	27.16
20125503	2011	5	24	520	71.15	27.07
20125503	2011	5	24	530	72.75	27
20125503	2011	5	24	540	74.43	26.95
20125503	2011	5	24	550	75.71	26.93
20125503	2011	5	24	560	76.81	26.89
20125503	2011	5	24	570	77.84	26.84
20125503	2011	5	24	580	79.53	26.73
20125503	2011	5	24	590	81.11	26.62
20125503	2011	5	24	600	82.32	26.5
20125503	2011	5	24	610	82.78	26.46
20125503	2011	5	24	620	83.34	26.39
20125503	2011	5	24	630	83.79	26.32
20125503	2011	5	24	640	84.17	26.26
20125503	2011	5	24	650	84.76	26.21
20125503	2011	5	24	660	85.31	26.16
20125503	2011	5	24	670	85.85	26.1
20125503	2011	5	24	680	86.11	26.03
20125503	2011	5	24	690	86.56	25.98
20125503	2011	5	24	700	86.98	25.92
20125503	2011	5	24	710	87.39	25.86
20125503	2011	5	24	720	87.71	25.82
20125503	2011	5	24	730	88.1	25.77
20125503	2011	5	24	740	88.65	25.72
20125503	2011	5	24	750	89.29	25.68
20125503	2011	5	24	760	90.69	25.59
20125503	2011	5	24	770	91.86	25.52
20125503	2011	5	24	780	92.81	25.47
20125503	2011	5	24	790	94.15	25.39

20125503	2011	5	24	800	95.92	25.26
20125503	2011	5	24	810	96.87	25.19
20125503	2011	5	24	820	98.13	25.14
20125503	2011	5	24	830	99.93	25.01
20125503	2011	5	24	840	101.13	24.95
20125503	2011	5	24	850	102.24	24.78
20125503	2011	5	24	860	103.13	24.72
20125503	2011	5	24	870	104.37	24.65
20125503	2011	5	24	880	105.03	24.57
20125503	2011	5	24	890	104.67	24.5
20125503	2011	5	24	900	104.4	24.44
20125503	2011	5	24	910	105.03	24.37
20125503	2011	5	24	920	106.46	24.21
20125503	2011	5	24	930	108.28	24.1
20125503	2011	5	24	940	109.91	24.03
20125503	2011	5	24	950	111.82	23.92
20125503	2011	5	24	960	113.62	23.85
20125503	2011	5	24	970	114.41	23.78
20125503	2011	5	24	980	114.62	23.71
20125503	2011	5	24	990	114.88	23.58
20125503	2011	5	24	1000	115.14	23.5
20125503	2011	5	24	1010	115.24	23.43
20125503	2011	5	24	1020	115.44	23.38
20125503	2011	5	24	1030	116.67	23.29
20125503	2011	5	24	1040	118.14	23.21
20125503	2011	5	24	1050	118.7	23.13
20125503	2011	5	24	1060	119.9	23.04
20125503	2011	5	24	1070	120.72	22.98
20125503	2011	5	24	1080	121.59	22.91
20125503	2011	5	24	1090	122.35	22.85
20125503	2011	5	24	1100	123.67	22.6
20125503	2011	5	24	1110	125.1	22.42
20125503	2011	5	24	1120	127.28	22.1
20125503	2011	5	24	1130	128.75	22.05
20125503	2011	5	24	1140	129.22	22.02
20125503	2011	5	24	1150	129.12	21.99
20125503	2011	5	24	1160	129	21.93
20125503	2011	5	24	1170	128.45	21.84
20125503	2011	5	24	1180	128.37	21.78
20125503	2011	5	24	1190	128.56	21.72
20125503	2011	5	24	1200	128.67	21.65
20125503	2011	5	24	1210	128.64	21.55
20125503	2011	5	24	1220	128.71	21.46
20125503	2011	5	24	1230	128.78	21.42
20125503	2011	5	24	1240	128.83	21.37
20125503	2011	5	24	1250	128.87	21.32
20125503	2011	5	24	1260	129.14	21.28

20125503	2011	5	24	1270	129.34	21.24
20125503	2011	5	24	1280	129.39	21.2
20125503	2011	5	24	1290	129.46	21.16
20125503	2011	5	24	1300	129.95	21.11
20125503	2011	5	24	1310	130.39	21.06
20125503	2011	5	24	1320	130.9	21.03
20125503	2011	5	24	1330	130.83	20.98
20125503	2011	5	24	1340	131.06	20.95
20125503	2011	5	24	1350	131.11	20.92
20125503	2011	5	24	1360	130.12	20.87
20125503	2011	5	24	1370	130.08	20.85
20125503	2011	5	24	1380	131.03	20.81
20125503	2011	5	24	1390	132.6	20.71
20125503	2011	5	24	1400	133.71	20.71
20125503	2011	5	24	1410	135.25	20.64
20125503	2011	5	24	1420	136.12	20.6
20125503	2011	5	24	1430	137.22	20.55
20125503	2011	5	24	1440	138.42	20.54
20125503	2011	5	24	1450	140.19	20.47
20125503	2011	5	24	1460	141.16	20.45
20125503	2011	5	24	1470	142	20.41
20125503	2011	5	24	1480	142.2	20.39
20125503	2011	5	24	1490	144.07	20.31
20125503	2011	5	24	1500	146.61	20.29
20125503	2011	5	24	1510	147.45	20.28
20125503	2011	5	24	1520	147.98	20.25
20125503	2011	5	24	1530	148.54	20.18
20125503	2011	5	24	1540	149.83	20.1
20125503	2011	5	24	1550	151.69	20
20125503	2011	5	24	1560	153.95	19.92
20125503	2011	5	24	1570	155.04	19.89
20125503	2011	5	24	1580	155.6	19.85
20125503	2011	5	24	1590	155.18	19.8
20125503	2011	5	24	1600	155.88	19.74
20125503	2011	5	24	1610	156.16	19.66
20125503	2011	5	24	1620	156.13	19.61
20125503	2011	5	24	1630	156.29	19.53
20125503	2011	5	24	1640	156.43	19.5
20125503	2011	5	24	1650	156.58	19.46
20125503	2011	5	24	1660	156.89	19.41
20125503	2011	5	24	1670	156.55	19.36
20125503	2011	5	24	1680	156.53	19.32
20125503	2011	5	24	1690	156.29	19.26
20125503	2011	5	24	1700	155.91	19.23
20125503	2011	5	24	1710	156.13	19.17
20125503	2011	5	24	1720	156.17	19.11
20125503	2011	5	24	1730	156.06	19.08

20125503	2011	5	24	1740	156.18	19.06
20125503	2011	5	24	1750	156.09	19.02
20125503	2011	5	24	1760	156.09	18.97
20125503	2011	5	24	1770	156.13	18.92
20125503	2011	5	24	1780	156.16	18.89
20125503	2011	5	24	1790	156.03	18.86
20125503	2011	5	24	1800	156.23	18.82
20125503	2011	5	24	1810	156.8	18.8
20125503	2011	5	24	1820	157.49	18.78
20125503	2011	5	24	1830	157.71	18.76
20125503	2011	5	24	1840	158.08	18.74
20125503	2011	5	24	1850	158.74	18.71
20125503	2011	5	24	1860	158.82	18.67
20125503	2011	5	24	1870	158.75	18.64
20125503	2011	5	24	1880	158.57	18.62
20125503	2011	5	24	1890	158.27	18.59
20125503	2011	5	24	1900	157.07	18.55
20125503	2011	5	24	1910	155.68	18.51
20125503	2011	5	24	1920	153.93	18.48
20125503	2011	5	24	1930	151.94	18.47
20125503	2011	5	24	1940	150.88	18.45
20125503	2011	5	24	1950	150	18.43
20125503	2011	5	24	1960	148.67	18.43
20125503	2011	5	24	1970	147.47	18.46
20125503	2011	5	24	1980	146.76	18.48
20125503	2011	5	24	1990	146.58	18.5
20125503	2011	5	24	2000	146.42	18.53
20125503	2011	5	24	2010	146.46	18.56
20125503	2011	5	24	2020	146.56	18.58
20125503	2011	5	24	2030	146.42	18.62
20125503	2011	5	24	2040	145.57	18.64
20125503	2011	5	24	2050	144.2	18.67
20125503	2011	5	24	2060	143.54	18.69
20125503	2011	5	24	2070	141.88	18.74
20125503	2011	5	24	2080	139.75	18.77
20125503	2011	5	24	2090	137.54	18.81
20125503	2011	5	24	2100	136.37	18.85
20125503	2011	5	24	2110	135.35	18.9
20125503	2011	5	24	2120	134.23	18.94
20125503	2011	5	24	2130	132.88	19
20125503	2011	5	24	2140	131.22	19.04
20125503	2011	5	24	2150	129.86	19.1
20125503	2011	5	24	2160	129.76	19.15
20125503	2011	5	24	2170	130.11	19.19
20125503	2011	5	24	2180	129.31	19.25
20125503	2011	5	24	2190	127.67	19.32
20125503	2011	5	24	2200	126.28	19.36

20125503	2011	5	24	2210	125.61	19.39
20125503	2011	5	24	2220	124.9	19.43
20125503	2011	5	24	2230	123.97	19.48
20125503	2011	5	24	2240	123.25	19.52
20125503	2011	5	24	2250	122.54	19.6
20125503	2011	5	24	2260	122.05	19.64
20125503	2011	5	24	2270	120.07	19.67
20125503	2011	5	24	2280	117.56	19.71
20125503	2011	5	24	2290	115.09	19.75
20125503	2011	5	24	2300	113.05	19.8
20125503	2011	5	24	2310	110.84	19.85
20125503	2011	5	24	2320	108.56	19.95
20125503	2011	5	24	2330	106.77	20.07
20125503	2011	5	24	2340	104.87	20.21
20125503	2011	5	24	2350	103.06	20.29
20125503	2011	5	24	2360	101.07	20.38
20125503	2011	5	24	2370	98.65	20.56
20125503	2011	5	24	2380	96.41	20.68
20125503	2011	5	24	2390	94.31	20.79
20125503	2011	5	24	2400	92.87	20.92
20125503	2011	5	24	2410	91.71	21.09
20125503	2011	5	24	2420	90.71	21.24
20125503	2011	5	24	2430	90.5	21.42
20125503	2011	5	24	2440	90.25	21.54
20125503	2011	5	24	2450	89.5	21.68
20125503	2011	5	24	2460	89.26	21.82
20125503	2011	5	24	2470	89.51	21.94
20125503	2011	5	24	2480	89.81	22.03
20125503	2011	5	24	2490	89.08	22.14
20125503	2011	5	24	2500	88.71	22.25
20125503	2011	5	24	2510	88.19	22.35
20125503	2011	5	24	2520	87.7	22.5
20125503	2011	5	24	2530	87.17	22.58
20125503	2011	5	24	2540	86.82	22.67
20125503	2011	5	24	2550	86.4	22.75
20125503	2011	5	24	2560	86.12	22.82
20125503	2011	5	24	2570	85.97	22.89
20125503	2011	5	24	2580	85.69	22.96
20125503	2011	5	24	2590	85.73	23.01
20125503	2011	5	24	2600	85.54	23.11
20125503	2011	5	24	2610	85.58	23.17
20125503	2011	5	24	2620	85.42	23.25
20125503	2011	5	24	2630	85.48	23.3
20125503	2011	5	24	2640	85.47	23.38
20125503	2011	5	24	2650	85.45	23.45
20125503	2011	5	24	2660	85.13	23.54
20125503	2011	5	24	2670	83.4	23.62

20125503	2011	5	24	2680	81.19	23.67
20125503	2011	5	24	2690	79.07	23.75
20125503	2011	5	24	2700	77.39	23.81
20125503	2011	5	24	2710	76.08	23.89
20125503	2011	5	24	2720	74.33	23.98
20125503	2011	5	24	2730	72.56	24.07
20125503	2011	5	24	2740	70.89	24.22
20125503	2011	5	24	2750	69.5	24.32
20125503	2011	5	24	2760	67.95	24.39
20125503	2011	5	24	2770	66.37	24.53
20125503	2011	5	24	2780	64.87	24.61
20125503	2011	5	24	2790	63.65	24.69
20125503	2011	5	24	2800	62.09	24.75
20125503	2011	5	24	2810	60.54	24.83
20125503	2011	5	24	2820	58.84	24.89
20125503	2011	5	24	2830	56.8	24.96
20125503	2011	5	24	2840	54.9	25
20125503	2011	5	24	2850	53.49	25.09
20125503	2011	5	24	2860	52.22	25.15
20125503	2011	5	24	2870	51.67	25.18
20125503	2011	5	24	2880	51.81	25.22
20125503	2011	5	24	2890	51.71	25.27
20125503	2011	5	24	2900	51.66	25.35
20125503	2011	5	24	2910	51.78	25.39
20125503	2011	5	24	2920	51.88	25.42
20125503	2011	5	24	2930	52.06	25.45
20125503	2011	5	24	2940	51.9	25.49
20125503	2011	5	24	2950	51.8	25.54
20125503	2011	5	24	2960	51.75	25.58
20125503	2011	5	24	2970	51.37	25.64
20125503	2011	5	24	2980	51.53	25.67
20125503	2011	5	24	2990	51.28	25.72
20125503	2011	5	24	3000	50.8	25.78
20125503	2011	5	24	3010	50.95	25.8
20125503	2011	5	24	3020	51.31	25.83
20125503	2011	5	24	3030	51.63	25.88
20125503	2011	5	24	3040	51.41	25.91
20125503	2011	5	24	3050	50.89	25.95
20125503	2011	5	24	3060	49.97	25.98
20125503	2011	5	24	3070	48.75	26.03
20125503	2011	5	24	3080	47.71	26.07
20125503	2011	5	24	3090	46.78	26.11
20125503	2011	5	24	3100	46.38	26.15
20125503	2011	5	24	3110	46.66	26.19
20125503	2011	5	24	3120	46.58	26.23
20125503	2011	5	24	3130	46.68	26.28
20125503	2011	5	24	3140	46.84	26.28

20125503	2011	5	24	3150	46.81	26.32
20125503	2011	5	24	3160	46.66	26.34
20125503	2011	5	24	3170	46.81	26.36
20125503	2011	5	24	3180	46.75	26.39
20125503	2011	5	24	3190	46.66	26.41
20125503	2011	5	24	3200	46.43	26.43
20125503	2011	5	24	3210	45.98	26.46
20125503	2011	5	24	3220	45.86	26.47
20125503	2011	5	24	3230	45.64	26.5
20125503	2011	5	24	3240	45.21	26.51
20125503	2011	5	24	3250	44.93	26.53
20125503	2011	5	24	3260	44.93	26.54
20125503	2011	5	24	3270	45.61	26.55
20125503	2011	5	24	3280	46.1	26.57
20125503	2011	5	24	3290	46.14	26.58
20125503	2011	5	24	3300	45.85	26.59
20125503	2011	5	24	3310	45.24	26.61
20125503	2011	5	24	3320	44.79	26.63
20125503	2011	5	24	3330	45.1	26.64
20125503	2011	5	24	3340	45.57	26.65
20125503	2011	5	24	3350	46.06	26.65
20125503	2011	5	24	3360	46.69	26.65
20125503	2011	5	24	3370	47.3	26.65
20125503	2011	5	24	3380	47.18	26.67
20125503	2011	5	24	3390	47.02	26.67
20125503	2011	5	24	3400	46.81	26.68
20125503	2011	5	24	3410	46.95	26.68
20125503	2011	5	24	3420	47.11	26.69
20125503	2011	5	24	3430	47.26	26.7
20125503	2011	5	24	3440	47.12	26.71
20125503	2011	5	24	3450	46.97	26.71
20125503	2011	5	24	3460	46.91	26.72
20125503	2011	5	24	3470	47.17	26.72
20125503	2011	5	24	3480	47.44	26.72
20125503	2011	5	24	3490	48.24	26.72
20125503	2011	5	24	3500	49.7	26.71
20125503	2011	5	24	3510	50.44	26.71
20125503	2011	5	24	3520	51.89	26.69
20125503	2011	5	24	3530	52.7	26.68
20125503	2011	5	24	3540	53.23	26.68
20125503	2011	5	24	3550	53.28	26.67
20125503	2011	5	24	3560	53.51	26.67
20125503	2011	5	24	3570	53	26.65
20125503	2011	5	24	3580	52.43	26.65
20125503	2011	5	24	3590	52.23	26.65
20125503	2011	5	24	3600	52.15	26.65
20125503	2011	5	24	3610	51.5	26.65

20125503	2011	5	24	3620	51.54	26.64
20125503	2011	5	24	3630	51.7	26.64
20125503	2011	5	24	3640	51.72	26.65
20125503	2011	5	24	3650	51.68	26.65
20125503	2011	5	24	3660	51.49	26.64
20125503	2011	5	24	3670	51.44	26.64
20125503	2011	5	24	3680	51.31	26.64
20125503	2011	5	24	3690	52.07	26.64
20125503	2011	5	24	3700	52.12	26.63
20125503	2011	5	24	3710	52.27	26.63
20125503	2011	5	24	3720	52.24	26.64
20125503	2011	5	24	3730	51.79	26.64
20125503	2011	5	24	3740	51.61	26.63
20125503	2011	5	24	3750	51.79	26.64
20125503	2011	5	24	3760	52.16	26.63
20125503	2011	5	24	3770	52.8	26.62
20125503	2011	5	24	3780	53.43	26.62
20125503	2011	5	24	3790	53.71	26.63
20125503	2011	5	24	3800	53.84	26.63
20125503	2011	5	24	3810	53.99	26.62
20125503	2011	5	24	3820	53.96	26.61
20125503	2011	5	24	3830	53.88	26.61
20125503	2011	5	24	3840	53.98	26.61
20125503	2011	5	24	3850	53.9	26.61
20125503	2011	5	24	3860	53.95	26.61
20125503	2011	5	24	3870	53.96	26.6
20125503	2011	5	24	3880	54	26.61
20125503	2011	5	24	3890	54.09	26.6
20125503	2011	5	24	3900	54.01	26.6
20125503	2011	5	24	3910	53.34	26.6
20125503	2011	5	24	3920	51.22	26.61
20125503	2011	5	24	3930	48.98	26.62
20125503	2011	5	24	3940	46.62	26.65
20125503	2011	5	24	3950	44.08	26.67
20125503	2011	5	24	3960	41.78	26.69
20125503	2011	5	24	3970	39.72	26.72
20125503	2011	5	24	3980	37.62	26.75
20125503	2011	5	24	3990	35.33	26.78
20125503	2011	5	24	4000	33.14	26.8
20125503	2011	5	24	4010	30.9	26.85
20125503	2011	5	24	4020	28.72	26.89
20125503	2011	5	24	4030	26.56	26.95
20125503	2011	5	24	4040	26.06	26.99
20125503	2011	5	24	4050	25.47	27.03
20125503	2011	5	24	4060	25.2	27.07
20125503	2011	5	24	4070	25.33	27.1
20125503	2011	5	24	4080	25.33	27.12

20125503	2011	5	24	4090	25.07	27.17
20125503	2011	5	24	4100	24.97	27.2
20125503	2011	5	24	4110	24.93	27.23
20125503	2011	5	24	4120	24.96	27.26
20125503	2011	5	24	4130	24.9	27.28
20125503	2011	5	24	4140	24.97	27.29
20125503	2011	5	24	4150	24.87	27.32
20125503	2011	5	24	4160	24.96	27.34
20125503	2011	5	24	4170	25.2	27.35
20125503	2011	5	24	4180	25.67	27.37
20125503	2011	5	24	4190	25.94	27.4
20125503	2011	5	24	4200	25.51	27.42
20125503	2011	5	24	4210	25.53	27.43
20125503	2011	5	24	4220	25.12	27.46
20125503	2011	5	24	4230	25.25	27.48
20125503	2011	5	24	4240	25.13	27.5
20125503	2011	5	24	4250	25.07	27.52
20125503	2011	5	24	4260	24.43	27.53
20125503	2011	5	24	4270	23.96	27.57
20125503	2011	5	24	4280	23.29	27.59
20125503	2011	5	24	4290	23.26	27.61
20125503	2011	5	24	4300	23.28	27.64
20125503	2011	5	24	4310	23.09	27.7
20125503	2011	5	24	4320	22.82	27.74
20125503	2011	5	24	4330	22.98	27.75
20125503	2011	5	24	4340	23.2	27.79
20125503	2011	5	24	4350	23.29	27.82
20125503	2011	5	24	4360	23.37	27.86
20125503	2011	5	24	4370	23.26	27.89
20125503	2011	5	24	4380	23.37	27.92
20125503	2011	5	24	4390	23.62	27.93
20125503	2011	5	24	4400	23.85	27.94
20125503	2011	5	24	4410	23.58	27.98
20125503	2011	5	24	4420	23.31	28.01
20125503	2011	5	24	4430	22.99	28.03
20125503	2011	5	24	4440	22.1	28.07
20125503	2011	5	24	4450	21.16	28.1
20125503	2011	5	24	4460	19.02	28.12
20125503	2011	5	24	4470	17.21	28.15
20125503	2011	5	24	4480	14.88	28.19
20125503	2011	5	24	4490	13.3	28.21
20125503	2011	5	24	4500	12.16	28.25
20125503	2011	5	24	4510	10.84	28.28
20125503	2011	5	24	4520	8.91	28.3
20125503	2011	5	24	4530	7.18	28.33
20125503	2011	5	24	4540	5.51	28.36
20125503	2011	5	24	4550	3.75	28.39

20125503	2011	5	24	4560	3	28.42
20125503	2011	5	24	4570	2.87	28.43
20125503	2011	5	24	4580	2.44	28.45
20125503	2011	5	24	4590	2.65	28.46
20125503	2011	5	24	4600	2.51	28.48
20125503	2011	5	24	4610	2.33	28.5
20125503	2011	5	24	4620	2.28	28.51
20125503	2011	5	24	4630	0.58	28.54
20125503	2011	5	24	4640	0.57	28.57
20125503	2011	5	24	4650	0.57	28.58
20125503	2011	5	24	4660	0.57	28.6
20125503	2011	5	24	4670	0.58	28.63
20125503	2011	5	24	4680	0.56	28.64
20125503	2011	5	24	4690	0.59	28.66
20125503	2011	5	24	4700	0.57	28.67
20125503	2011	5	24	4710	0.6	28.69
20125503	2011	5	24	4720	0.58	28.71
20125503	2011	5	24	4730	0.6	28.72
20125503	2011	5	24	4740	0.56	28.74
20125503	2011	5	24	4750	0.56	28.75
20125503	2011	5	24	4760	0.47	28.78
20125503	2011	5	24	4770	0.47	28.79
20133213	2011	5	24	0	1.63	29.05
20133213	2011	5	24	10	2.48	29.03
20133213	2011	5	24	20	2.55	29.03
20133213	2011	5	24	30	2.55	29.03
20133213	2011	5	24	40	1.58	29.03
20133213	2011	5	24	50	2.04	29.04
20133213	2011	5	24	60	2.43	29.03
20133213	2011	5	24	70	2.37	29.03
20133213	2011	5	24	80	2.66	29.03
20133213	2011	5	24	90	2.44	29.03
20133213	2011	5	24	100	2.3	29.01
20133213	2011	5	24	110	2.48	29
20133213	2011	5	24	120	3.02	29
20133213	2011	5	24	130	3.84	28.97
20133213	2011	5	24	140	4.61	28.96
20133213	2011	5	24	150	5.65	28.93
20133213	2011	5	24	160	6.62	28.92
20133213	2011	5	24	170	7.32	28.92
20133213	2011	5	24	180	7.72	28.89
20133213	2011	5	24	190	8	28.88
20133213	2011	5	24	200	8.57	28.85
20133213	2011	5	24	210	10.25	28.82
20133213	2011	5	24	220	12.88	28.78
20133213	2011	5	24	230	16.31	28.74
20133213	2011	5	24	240	19.23	28.7

20133213	2011	5	24	250	21.69	28.67
20133213	2011	5	24	260	24.19	28.61
20133213	2011	5	24	270	26.63	28.6
20133213	2011	5	24	280	29.02	28.55
20133213	2011	5	24	290	30.21	28.52
20133213	2011	5	24	300	32.23	28.31
20133213	2011	5	24	310	34.3	28.17
20133213	2011	5	24	320	36.52	28.16
20133213	2011	5	24	330	39.09	28.14
20133213	2011	5	24	340	41.53	27.98
20133213	2011	5	24	350	43.94	27.87
20133213	2011	5	24	360	46.51	27.78
20133213	2011	5	24	370	49.42	27.71
20133213	2011	5	24	380	52.62	27.67
20133213	2011	5	24	390	55.25	27.61
20133213	2011	5	24	400	56.45	27.57
20133213	2011	5	24	410	57.63	27.43
20133213	2011	5	24	420	59.16	27.32
20133213	2011	5	24	430	60.15	27.26
20133213	2011	5	24	440	61.01	27.24
20133213	2011	5	24	450	62.06	27.17
20133213	2011	5	24	460	63.09	27.14
20133213	2011	5	24	470	63.92	27.08
20133213	2011	5	24	480	64.56	27.02
20133213	2011	5	24	490	65.19	26.96
20133213	2011	5	24	500	65.91	26.9
20133213	2011	5	24	510	66.42	26.86
20133213	2011	5	24	520	67.06	26.81
20133213	2011	5	24	530	67.24	26.77
20133213	2011	5	24	540	67.65	26.75
20133213	2011	5	24	550	68.1	26.69
20133213	2011	5	24	560	68.86	26.66
20133213	2011	5	24	570	69.97	26.62
20133213	2011	5	24	580	71.04	26.52
20133213	2011	5	24	590	71.71	26.5
20133213	2011	5	24	600	72.26	26.46
20133213	2011	5	24	610	72.82	26.4
20133213	2011	5	24	620	74.51	26.32
20133213	2011	5	24	630	76.71	26.2
20133213	2011	5	24	640	79.24	26.12
20133213	2011	5	24	650	81.91	26.03
20133213	2011	5	24	660	84.55	25.95
20133213	2011	5	24	670	87.34	25.85
20133213	2011	5	24	680	89.53	25.78
20133213	2011	5	24	690	91.93	25.56
20133213	2011	5	24	700	94.4	25.4
20133213	2011	5	24	710	95.74	25.43

20133213	2011	5	24	720	96.37	25.32
20133213	2011	5	24	730	97.04	25.14
20133213	2011	5	24	740	97.9	24.95
20133213	2011	5	24	750	98.7	24.87
20133213	2011	5	24	760	99.89	24.64
20133213	2011	5	24	770	102	24.21
20133213	2011	5	24	780	103.72	24.21
20133213	2011	5	24	790	105.01	24.21
20133213	2011	5	24	800	106.19	24.11
20133213	2011	5	24	810	108.57	24.02
20133213	2011	5	24	820	110.76	23.97
20133213	2011	5	24	830	111.76	23.94
20133213	2011	5	24	840	111.98	23.89
20133213	2011	5	24	850	112.46	23.85
20133213	2011	5	24	860	112.75	23.78
20133213	2011	5	24	870	112.51	23.71
20133213	2011	5	24	880	112.2	23.65
20133213	2011	5	24	890	112.13	23.6
20133213	2011	5	24	900	112.13	23.51
20133213	2011	5	24	910	111.95	23.42
20133213	2011	5	24	920	112.07	23.38
20133213	2011	5	24	930	112.23	23.32
20133213	2011	5	24	940	112.36	23.27
20133213	2011	5	24	950	112.6	23.19
20133213	2011	5	24	960	112.71	23.11
20133213	2011	5	24	970	112.76	23.05
20133213	2011	5	24	980	112.67	23.02
20133213	2011	5	24	990	112.59	22.99
20133213	2011	5	24	1000	112.53	22.96
20133213	2011	5	24	1010	112.46	22.9
20133213	2011	5	24	1020	112.75	22.86
20133213	2011	5	24	1030	113.25	22.83
20133213	2011	5	24	1040	113.1	22.8
20133213	2011	5	24	1050	112.92	22.77
20133213	2011	5	24	1060	112.54	22.75
20133213	2011	5	24	1070	111.86	22.71
20133213	2011	5	24	1080	111.01	22.69
20133213	2011	5	24	1090	110.41	22.67
20133213	2011	5	24	1100	110.18	22.64
20133213	2011	5	24	1110	110.34	22.61
20133213	2011	5	24	1120	110.39	22.6
20133213	2011	5	24	1130	110.42	22.57
20133213	2011	5	24	1140	110.72	22.56
20133213	2011	5	24	1150	111.17	22.54
20133213	2011	5	24	1160	111.79	22.52
20133213	2011	5	24	1170	112.41	22.51
20133213	2011	5	24	1180	113.15	22.5

20133213	2011	5	24	1190	113.41	22.5
20133213	2011	5	24	1200	113.24	22.46
20133213	2011	5	24	1210	113.01	22.44
20133213	2011	5	24	1220	112.91	22.42
20133213	2011	5	24	1230	113.83	22.38
20133213	2011	5	24	1240	115.74	22.35
20133213	2011	5	24	1250	118.11	22.32
20133213	2011	5	24	1260	119.46	22.31
20133213	2011	5	24	1270	120.31	22.29
20133213	2011	5	24	1280	120.6	22.28
20133213	2011	5	24	1290	120.31	22.27
20133213	2011	5	24	1300	120	22.25
20133213	2011	5	24	1310	119.73	22.25
20133213	2011	5	24	1320	119.84	22.23
20133213	2011	5	24	1330	119.63	22.21
20133213	2011	5	24	1340	119.44	22.2
20133213	2011	5	24	1350	119.63	22.18
20133213	2011	5	24	1360	119.4	22.17
20133213	2011	5	24	1370	119.04	22.14
20133213	2011	5	24	1380	118.5	22.14
20133213	2011	5	24	1390	118.01	22.13
20133213	2011	5	24	1400	117.58	22.13
20133213	2011	5	24	1410	117.76	22.11
20133213	2011	5	24	1420	118	22.1
20133213	2011	5	24	1430	117.77	22.1
20133213	2011	5	24	1440	117.51	22.1
20133213	2011	5	24	1450	117.16	22.09
20133213	2011	5	24	1460	116.85	22.07
20133213	2011	5	24	1470	117.3	22.06
20133213	2011	5	24	1480	117.53	22.05
20133213	2011	5	24	1490	117.37	22.05
20133213	2011	5	24	1500	117.22	22.05
20133213	2011	5	24	1510	117.07	22.04
20133213	2011	5	24	1520	117.1	22.04
20133213	2011	5	24	1530	117.31	22.03
20133213	2011	5	24	1540	117.33	22.03
20133213	2011	5	24	1550	117.06	22.02
20133213	2011	5	24	1560	116.71	22.02
20133213	2011	5	24	1570	116.71	22.01
20133213	2011	5	24	1580	116.72	22
20133213	2011	5	24	1590	116.66	22
20133213	2011	5	24	1600	116.72	22
20133213	2011	5	24	1610	117.07	21.99
20133213	2011	5	24	1620	117.33	21.99
20133213	2011	5	24	1630	117.25	21.99
20133213	2011	5	24	1640	117.1	21.98
20133213	2011	5	24	1650	117.12	21.97

20133213	2011	5	24	1660	117.24	21.97
20133213	2011	5	24	1670	117.14	21.97
20133213	2011	5	24	1680	117.25	21.97
20133213	2011	5	24	1690	117.14	21.96
20133213	2011	5	24	1700	117.38	21.96
20133213	2011	5	24	1710	117.38	21.96
20133213	2011	5	24	1720	117.21	21.96
20133213	2011	5	24	1730	117.5	21.95
20133213	2011	5	24	1740	117.29	21.96
20133213	2011	5	24	1750	117.35	21.95
20133213	2011	5	24	1760	117.29	21.95
20133213	2011	5	24	1770	117.43	21.95
20133213	2011	5	24	1780	117.65	21.94
20133213	2011	5	24	1790	117.45	21.94
20133213	2011	5	24	1800	117.39	21.94
20133213	2011	5	24	1810	117.15	21.94
20133213	2011	5	24	1820	117.15	21.93
20133213	2011	5	24	1830	116.99	21.93
20133213	2011	5	24	1840	116.93	21.93
20133213	2011	5	24	1850	116.48	21.93
20133213	2011	5	24	1860	116.46	21.93
20133213	2011	5	24	1870	116.42	21.93
20133213	2011	5	24	1880	116.26	21.93
20133213	2011	5	24	1890	116.55	21.94
20133213	2011	5	24	1900	116.68	21.93
20133213	2011	5	24	1910	116.38	21.93
20133213	2011	5	24	1920	116.78	21.94
20133213	2011	5	24	1930	117.14	21.93
20133213	2011	5	24	1940	117.66	21.93
20133213	2011	5	24	1950	117.52	21.92
20133213	2011	5	24	1960	117.48	21.92
20133213	2011	5	24	1970	117.26	21.92
20133213	2011	5	24	1980	116.98	21.92
20133213	2011	5	24	1990	116.97	21.92
20133213	2011	5	24	2000	116.72	21.92
20133213	2011	5	24	2010	116.66	21.92
20133213	2011	5	24	2020	116.89	21.92
20133213	2011	5	24	2030	116.56	21.92
20133213	2011	5	24	2040	116.53	21.92
20133213	2011	5	24	2050	116.25	21.92
20133213	2011	5	24	2060	115.93	21.93
20133213	2011	5	24	2070	115.78	21.92
20133213	2011	5	24	2080	116.1	21.93
20133213	2011	5	24	2090	116.22	21.93
20133213	2011	5	24	2100	116.27	21.92
20133213	2011	5	24	2110	115.88	21.93
20133213	2011	5	24	2120	115.98	21.93

20133213	2011	5	24	2130	116.59	21.92
20133213	2011	5	24	2140	117.38	21.93
20133213	2011	5	24	2150	117.64	21.93
20133213	2011	5	24	2160	117.72	21.92
20133213	2011	5	24	2170	117.46	21.93
20133213	2011	5	24	2180	117.27	21.92
20133213	2011	5	24	2190	117.11	21.93
20133213	2011	5	24	2200	117.22	21.92
20133213	2011	5	24	2210	117.49	21.92
20133213	2011	5	24	2220	117.43	21.92
20133213	2011	5	24	2230	117.22	21.92
20133213	2011	5	24	2240	117	21.92
20133213	2011	5	24	2250	116.76	21.92
20133213	2011	5	24	2260	116.28	21.93
20133213	2011	5	24	2270	115.73	21.92
20133213	2011	5	24	2280	115.66	21.92
20133213	2011	5	24	2290	115.52	21.93
20133213	2011	5	24	2300	115.48	21.92
20133213	2011	5	24	2310	116.15	21.92
20133213	2011	5	24	2320	116.35	21.92
20133213	2011	5	24	2330	116.2	21.92
20133213	2011	5	24	2340	116.1	21.92
20133213	2011	5	24	2350	115.98	21.92
20133213	2011	5	24	2360	115.9	21.92
20133213	2011	5	24	2370	116.56	21.92
20133213	2011	5	24	2380	116.84	21.92
20133213	2011	5	24	2390	117	21.92
20133213	2011	5	24	2400	117.13	21.92
20133213	2011	5	24	2410	117.12	21.92
20133213	2011	5	24	2420	117.12	21.92
20133213	2011	5	24	2430	116.84	21.92
20133213	2011	5	24	2440	116.85	21.92
20133213	2011	5	24	2450	116.76	21.92
20133213	2011	5	24	2460	116.77	21.92
20133213	2011	5	24	2470	116.59	21.92
20133213	2011	5	24	2480	116.54	21.92
20133213	2011	5	24	2490	116.85	21.91
20133213	2011	5	24	2500	117.21	21.92
20133213	2011	5	24	2510	117.27	21.91
20133213	2011	5	24	2520	117.45	21.92
20133213	2011	5	24	2530	117.29	21.92
20133213	2011	5	24	2540	117.33	21.92
20133213	2011	5	24	2550	117.72	21.92
20133213	2011	5	24	2560	117.44	21.92
20133213	2011	5	24	2570	117.37	21.92
20133213	2011	5	24	2580	117.26	21.92
20133213	2011	5	24	2590	116.87	21.92

20133213	2011	5	24	2600	116.56	21.92
20133213	2011	5	24	2610	117.09	21.92
20133213	2011	5	24	2620	117.51	21.92
20133213	2011	5	24	2630	117.66	21.92
20133213	2011	5	24	2640	117.36	21.94
20133213	2011	5	24	2650	116.84	21.95
20133213	2011	5	24	2660	116.67	21.94
20133213	2011	5	24	2670	117.5	21.95
20133213	2011	5	24	2680	118.58	21.95
20133213	2011	5	24	2690	119.43	21.94
20133213	2011	5	24	2700	120.13	21.93
20133213	2011	5	24	2710	120.73	21.93
20133213	2011	5	24	2720	120.93	21.93
20133213	2011	5	24	2730	120.74	21.93
20133213	2011	5	24	2740	120.73	21.92
20133213	2011	5	24	2750	121.15	21.92
20133213	2011	5	24	2760	122.22	21.91
20133213	2011	5	24	2770	122.65	21.86
20133213	2011	5	24	2780	122.86	21.8
20133213	2011	5	24	2790	122.98	21.76
20133213	2011	5	24	2800	122.89	21.72
20133213	2011	5	24	2810	123.27	21.65
20133213	2011	5	24	2820	123.3	21.6
20133213	2011	5	24	2830	122.92	21.55
20133213	2011	5	24	2840	122.84	21.53
20133213	2011	5	24	2850	123.1	21.47
20133213	2011	5	24	2860	123.17	21.43
20133213	2011	5	24	2870	123.36	21.37
20133213	2011	5	24	2880	123.32	21.32
20133213	2011	5	24	2890	123.57	21.26
20133213	2011	5	24	2900	123.46	21.23
20133213	2011	5	24	2910	123.51	21.19
20133213	2011	5	24	2920	123.36	21.17
20133213	2011	5	24	2930	123.34	21.14
20133213	2011	5	24	2940	123.49	21.1
20133213	2011	5	24	2950	123.94	21.06
20133213	2011	5	24	2960	124.28	21.02
20133213	2011	5	24	2970	124.31	20.99
20133213	2011	5	24	2980	124.34	20.97
20133213	2011	5	24	2990	124.31	20.94
20133213	2011	5	24	3000	124.5	20.9
20133213	2011	5	24	3010	124.12	20.86
20133213	2011	5	24	3020	123.8	20.85
20133213	2011	5	24	3030	123.72	20.82
20133213	2011	5	24	3040	122.91	20.81
20133213	2011	5	24	3050	122.62	20.79
20133213	2011	5	24	3060	123.26	20.76

20133213	2011	5	24	3070	124.6	20.71
20133213	2011	5	24	3080	125.72	20.69
20133213	2011	5	24	3090	126.68	20.66
20133213	2011	5	24	3100	127.27	20.64
20133213	2011	5	24	3110	127.73	20.63
20133213	2011	5	24	3120	127.98	20.61
20133213	2011	5	24	3130	128.35	20.6
20133213	2011	5	24	3140	128.5	20.6
20133213	2011	5	24	3150	128.78	20.59
20133213	2011	5	24	3160	128.64	20.57
20133213	2011	5	24	3170	128.6	20.57
20133213	2011	5	24	3180	128.44	20.55
20133213	2011	5	24	3190	128.52	20.54
20133213	2011	5	24	3200	128.45	20.53
20133213	2011	5	24	3210	128.71	20.52
20133213	2011	5	24	3220	128.65	20.51
20133213	2011	5	24	3230	128.77	20.49
20133213	2011	5	24	3240	128.71	20.48
20133213	2011	5	24	3250	128.46	20.48
20133213	2011	5	24	3260	129.17	20.46
20133213	2011	5	24	3270	130.83	20.45
20133213	2011	5	24	3280	131.66	20.43
20133213	2011	5	24	3290	133.39	20.42
20133213	2011	5	24	3300	134.45	20.41
20133213	2011	5	24	3310	134.88	20.4
20133213	2011	5	24	3320	134.91	20.39
20133213	2011	5	24	3330	134.67	20.39
20133213	2011	5	24	3340	134.43	20.38
20133213	2011	5	24	3350	135.54	20.36
20133213	2011	5	24	3360	137.08	20.35
20133213	2011	5	24	3370	138.88	20.34
20133213	2011	5	24	3380	139.6	20.35
20133213	2011	5	24	3390	139.72	20.33
20133213	2011	5	24	3400	139.85	20.33
20133213	2011	5	24	3410	139.8	20.32
20133213	2011	5	24	3420	140.13	20.32
20133213	2011	5	24	3430	140.12	20.32
20133213	2011	5	24	3440	140.35	20.31
20133213	2011	5	24	3450	141.21	20.29
20133213	2011	5	24	3460	142.23	20.28
20133213	2011	5	24	3470	143.23	20.27
20133213	2011	5	24	3480	145.08	20.25
20133213	2011	5	24	3490	146.96	20.25
20133213	2011	5	24	3500	148.37	20.22
20133213	2011	5	24	3510	148.77	20.21
20133213	2011	5	24	3520	149.48	20.21
20133213	2011	5	24	3530	150.57	20.18

20133213	2011	5	24	3540	151.61	20.17
20133213	2011	5	24	3550	153.01	20.14
20133213	2011	5	24	3560	153.51	20.11
20133213	2011	5	24	3570	154.09	20.09
20133213	2011	5	24	3580	154.23	20.08
20133213	2011	5	24	3590	154.25	20.07
20133213	2011	5	24	3600	153.94	20.06
20133213	2011	5	24	3610	154.3	20.05
20133213	2011	5	24	3620	154.7	20.03
20133213	2011	5	24	3630	154.75	20.01
20133213	2011	5	24	3640	154.36	19.98
20133213	2011	5	24	3650	154.45	19.97
20133213	2011	5	24	3660	154.72	19.95
20133213	2011	5	24	3670	154.9	19.93
20133213	2011	5	24	3680	155.11	19.92
20133213	2011	5	24	3690	154.86	19.9
20133213	2011	5	24	3700	155.22	19.89
20133213	2011	5	24	3710	155.2	19.89
20133213	2011	5	24	3720	155.09	19.87
20133213	2011	5	24	3730	154.99	19.86
20133213	2011	5	24	3740	155.21	19.85
20133213	2011	5	24	3750	155.12	19.84
20133213	2011	5	24	3760	155.43	19.82
20133213	2011	5	24	3770	155.58	19.78
20133213	2011	5	24	3780	155.74	19.78
20133213	2011	5	24	3790	155.62	19.75
20133213	2011	5	24	3800	155.16	19.73
20133213	2011	5	24	3810	154.87	19.72
20133213	2011	5	24	3820	154.84	19.71
20133213	2011	5	24	3830	154.7	19.68
20133213	2011	5	24	3840	154.17	19.67
20133213	2011	5	24	3850	153.64	19.66
20133213	2011	5	24	3860	153.54	19.64
20133213	2011	5	24	3870	153.22	19.64
20133213	2011	5	24	3880	152.63	19.64
20133213	2011	5	24	3890	152.4	19.63
20133213	2011	5	24	3900	152.01	19.63
20133213	2011	5	24	3910	152.06	19.63
20133213	2011	5	24	3920	151.64	19.63
20133213	2011	5	24	3930	151.21	19.64
20133213	2011	5	24	3940	151.15	19.63
20133213	2011	5	24	3950	150.99	19.63
20133213	2011	5	24	3960	151.1	19.63
20133213	2011	5	24	3970	151.33	19.63
20133213	2011	5	24	3980	151.11	19.61
20133213	2011	5	24	3990	151.18	19.62
20133213	2011	5	24	4000	151.19	19.62

20133213	2011	5	24	4010	150.46	19.6
20133213	2011	5	24	4020	150.47	19.61
20133213	2011	5	24	4030	150.26	19.6
20133213	2011	5	24	4040	150.02	19.6
20133213	2011	5	24	4050	149.87	19.6
20133213	2011	5	24	4060	150.32	19.6
20133213	2011	5	24	4070	150.12	19.59
20133213	2011	5	24	4080	149.6	19.59
20133213	2011	5	24	4090	149.05	19.59
20133213	2011	5	24	4100	149.67	19.6
20133213	2011	5	24	4110	150.46	19.6
20133213	2011	5	24	4120	150.82	19.6
20133213	2011	5	24	4130	151.28	19.6
20133213	2011	5	24	4140	151.51	19.6
20133213	2011	5	24	4150	151.45	19.6
20133213	2011	5	24	4160	151.24	19.6
20133213	2011	5	24	4170	150.83	19.6
20133213	2011	5	24	4180	151.34	19.59
20133213	2011	5	24	4190	151.77	19.57
20133213	2011	5	24	4200	151.67	19.54
20133213	2011	5	24	4210	151.37	19.53
20133213	2011	5	24	4220	151.91	19.53
20133213	2011	5	24	4230	152.57	19.52
20133213	2011	5	24	4240	152.28	19.51
20133213	2011	5	24	4250	151.82	19.5
20133213	2011	5	24	4260	152	19.5
20133213	2011	5	24	4270	152.28	19.5
20133213	2011	5	24	4280	152.81	19.49
20133213	2011	5	24	4290	153.04	19.49
20133213	2011	5	24	4300	153.67	19.48
20133213	2011	5	24	4310	154.34	19.47
20133213	2011	5	24	4320	154.5	19.46
20133213	2011	5	24	4330	154.36	19.44
20133213	2011	5	24	4340	154.44	19.44
20133213	2011	5	24	4350	154.63	19.42
20133213	2011	5	24	4360	154.72	19.41
20133213	2011	5	24	4370	154.42	19.39
20133213	2011	5	24	4380	154.33	19.38
20133213	2011	5	24	4390	154.53	19.36
20133213	2011	5	24	4400	154.68	19.35
20133213	2011	5	24	4410	154.81	19.33
20133213	2011	5	24	4420	154.3	19.32
20133213	2011	5	24	4430	154.66	19.3
20133213	2011	5	24	4440	154.71	19.28
20133213	2011	5	24	4450	154.59	19.28
20133213	2011	5	24	4460	154.71	19.26
20133213	2011	5	24	4470	154.71	19.26

20133213	2011	5	24	4480	154.59	19.25
20133213	2011	5	24	4490	154.82	19.24
20133213	2011	5	24	4500	154.6	19.22
20133213	2011	5	24	4510	154.77	19.21
20133213	2011	5	24	4520	154.68	19.21
20133213	2011	5	24	4530	154.71	19.2
20133213	2011	5	24	4540	154.61	19.19
20133213	2011	5	24	4550	154.48	19.19
20133213	2011	5	24	4560	154.3	19.18
20133213	2011	5	24	4570	154.54	19.17
20133213	2011	5	24	4580	154.85	19.17
20133213	2011	5	24	4590	154.81	19.17
20133213	2011	5	24	4600	155.43	19.16
20133213	2011	5	24	4610	156.13	19.17
20133213	2011	5	24	4620	157.41	19.16
20133213	2011	5	24	4630	157.96	19.15
20133213	2011	5	24	4640	158.01	19.14
20133213	2011	5	24	4650	158.07	19.14
20133213	2011	5	24	4660	158.01	19.12
20133213	2011	5	24	4670	157.83	19.12
20133213	2011	5	24	4680	158.09	19.11
20133213	2011	5	24	4690	157.81	19.1
20133213	2011	5	24	4700	157.41	19.1
20133213	2011	5	24	4710	157.65	19.08
20133213	2011	5	24	4720	157.98	19.07
20133213	2011	5	24	4730	157.93	19.06
20133213	2011	5	24	4740	158.06	19.05
20133213	2011	5	24	4750	157.87	19.03
20133213	2011	5	24	4760	157.56	19.03
20133213	2011	5	24	4770	157.85	19.02
20133213	2011	5	24	4780	157.58	19.03
20133213	2011	5	24	4790	157.45	19.02
20133213	2011	5	24	4800	157.24	19.01
20133213	2011	5	24	4810	157.45	19
20133213	2011	5	24	4820	157.75	19
20133213	2011	5	24	4830	157.77	18.97
20133213	2011	5	24	4840	158.45	18.97
20133213	2011	5	24	4850	158.5	18.96
20133213	2011	5	24	4860	158.27	18.95
20133213	2011	5	24	4870	158.67	18.95
20133213	2011	5	24	4880	158.37	18.93
20133213	2011	5	24	4890	158.51	18.93
20133213	2011	5	24	4900	158.21	18.92
20133213	2011	5	24	4910	158.23	18.92
20133213	2011	5	24	4920	158.43	18.92
20133213	2011	5	24	4930	158.45	18.91
20133213	2011	5	24	4940	158.83	18.91

20133213	2011	5	24	4950	158.73	18.89
20133213	2011	5	24	4960	158.28	18.89
20133213	2011	5	24	4970	157.76	18.89
20133213	2011	5	24	4980	157.63	18.88
20133213	2011	5	24	4990	158.19	18.87
20133213	2011	5	24	5000	158.71	18.86
20133213	2011	5	24	5010	158.48	18.85
20133213	2011	5	24	5020	158.62	18.85
20133213	2011	5	24	5030	158.49	18.84
20133213	2011	5	24	5040	158.31	18.83
20133213	2011	5	24	5050	158.18	18.83
20133213	2011	5	24	5060	158.16	18.82
20133213	2011	5	24	5070	158.02	18.82
20133213	2011	5	24	5080	157.89	18.82
20133213	2011	5	24	5090	158.45	18.82
20133213	2011	5	24	5100	158.36	18.82
20133213	2011	5	24	5110	157.82	18.82
20133213	2011	5	24	5120	157.85	18.81
20133213	2011	5	24	5130	158.25	18.8
20133213	2011	5	24	5140	159.16	18.79
20133213	2011	5	24	5150	158.57	18.78
20133213	2011	5	24	5160	157.93	18.78
20133213	2011	5	24	5170	157.83	18.78
20133213	2011	5	24	5180	158.12	18.78
20133213	2011	5	24	5190	158.17	18.78
20133213	2011	5	24	5200	158.28	18.77
20133213	2011	5	24	5210	158.5	18.77
20133213	2011	5	24	5220	158.79	18.76
20133213	2011	5	24	5230	158.57	18.75
20133213	2011	5	24	5240	158.53	18.75
20133213	2011	5	24	5250	158.75	18.75
20133213	2011	5	24	5260	158.57	18.75
20133213	2011	5	24	5270	158.64	18.73
20133213	2011	5	24	5280	158.95	18.72
20133213	2011	5	24	5290	158.74	18.72
20133213	2011	5	24	5300	158.52	18.71
20133213	2011	5	24	5310	158.57	18.71
20133213	2011	5	24	5320	158.3	18.69
20133213	2011	5	24	5330	158.44	18.68
20133213	2011	5	24	5340	158.5	18.67
20133213	2011	5	24	5350	158.46	18.67
20133213	2011	5	24	5360	158.43	18.66
20133213	2011	5	24	5370	158.54	18.65
20133213	2011	5	24	5380	158.37	18.64
20133213	2011	5	24	5390	158.68	18.63
20133213	2011	5	24	5400	158.77	18.62
20133213	2011	5	24	5410	159.12	18.6

20133213	2011	5	24	5420	158.97	18.6
20133213	2011	5	24	5430	159.21	18.59
20133213	2011	5	24	5440	159.38	18.58
20133213	2011	5	24	5450	159.32	18.57
20133213	2011	5	24	5460	158.79	18.54
20133213	2011	5	24	5470	159.02	18.53
20133213	2011	5	24	5480	159.37	18.52
20133213	2011	5	24	5490	159.17	18.5
20133213	2011	5	24	5500	159.16	18.48
20133213	2011	5	24	5510	159.6	18.46
20133213	2011	5	24	5520	159.39	18.42
20133213	2011	5	24	5530	158.64	18.42
20133213	2011	5	24	5540	158.25	18.41
20133213	2011	5	24	5550	158.11	18.39
20133213	2011	5	24	5560	157.82	18.39
20133213	2011	5	24	5570	157.53	18.37
20133213	2011	5	24	5580	157.75	18.38
20133213	2011	5	24	5590	157.26	18.37
20133213	2011	5	24	5600	156.64	18.36
20133213	2011	5	24	5610	155.76	18.37
20133213	2011	5	24	5620	155.15	18.37
20133213	2011	5	24	5630	154.86	18.38
20133213	2011	5	24	5640	154.74	18.38
20133213	2011	5	24	5650	154.37	18.39
20133213	2011	5	24	5660	153.99	18.41
20133213	2011	5	24	5670	153.58	18.42
20133213	2011	5	24	5680	153.26	18.43
20133213	2011	5	24	5690	153.52	18.46
20133213	2011	5	24	5700	153.64	18.47
20133213	2011	5	24	5710	153.72	18.48
20133213	2011	5	24	5720	153.67	18.51
20133213	2011	5	24	5730	153.37	18.53
20133213	2011	5	24	5740	153.02	18.55
20133213	2011	5	24	5750	152.44	18.57
20133213	2011	5	24	5760	152.36	18.58
20133213	2011	5	24	5770	152.3	18.6
20133213	2011	5	24	5780	152.48	18.61
20133213	2011	5	24	5790	153.39	18.65
20133213	2011	5	24	5800	154.12	18.67
20133213	2011	5	24	5810	154.76	18.67
20133213	2011	5	24	5820	155.43	18.69
20133213	2011	5	24	5830	155.95	18.7
20133213	2011	5	24	5840	156.14	18.69
20133213	2011	5	24	5850	156.27	18.68
20133213	2011	5	24	5860	156.21	18.67
20133213	2011	5	24	5870	155.93	18.67
20133213	2011	5	24	5880	155.68	18.67

20133213	2011	5	24	5890	155.8	18.66
20133213	2011	5	24	5900	155.92	18.65
20133213	2011	5	24	5910	155.77	18.65
20133213	2011	5	24	5920	155.66	18.65
20133213	2011	5	24	5930	155.13	18.64
20133213	2011	5	24	5940	154.69	18.64
20133213	2011	5	24	5950	155.23	18.64
20133213	2011	5	24	5960	155.23	18.64
20133213	2011	5	24	5970	155.38	18.64
20133213	2011	5	24	5980	155.62	18.64
20133213	2011	5	24	5990	155.42	18.64
20133213	2011	5	24	6000	155.4	18.64
20133213	2011	5	24	6010	155.24	18.64
20133213	2011	5	24	6020	154.74	18.64
20133213	2011	5	24	6030	153.94	18.64
20133213	2011	5	24	6040	153.59	18.65
20133213	2011	5	24	6050	153.17	18.66
20133213	2011	5	24	6060	153.36	18.67
20133213	2011	5	24	6070	153.52	18.67
20133213	2011	5	24	6080	153.31	18.67
20133213	2011	5	24	6090	153.25	18.67
20133213	2011	5	24	6100	153.41	18.68
20133213	2011	5	24	6110	153.42	18.68
20133213	2011	5	24	6120	153.26	18.69
20133213	2011	5	24	6130	153.46	18.7
20133213	2011	5	24	6140	153.79	18.71
20133213	2011	5	24	6150	153.91	18.72
20133213	2011	5	24	6160	153.83	18.72
20133213	2011	5	24	6170	153.21	18.75
20133213	2011	5	24	6180	151.95	18.75
20133213	2011	5	24	6190	151.09	18.78
20133213	2011	5	24	6200	150.69	18.78
20133213	2011	5	24	6210	150.4	18.8
20133213	2011	5	24	6220	149.98	18.82
20133213	2011	5	24	6230	149.29	18.87
20133213	2011	5	24	6240	147.94	18.89
20133213	2011	5	24	6250	146.76	18.93
20133213	2011	5	24	6260	145.97	18.96
20133213	2011	5	24	6270	145.09	19.02
20133213	2011	5	24	6280	143.63	19.06
20133213	2011	5	24	6290	143.22	19.1
20133213	2011	5	24	6300	141.79	19.15
20133213	2011	5	24	6310	140.56	19.18
20133213	2011	5	24	6320	139.25	19.22
20133213	2011	5	24	6330	137.64	19.27
20133213	2011	5	24	6340	135.92	19.3
20133213	2011	5	24	6350	134.5	19.35

20133213	2011	5	24	6360	133.31	19.37
20133213	2011	5	24	6370	131.02	19.42
20133213	2011	5	24	6380	128.81	19.46
20133213	2011	5	24	6390	126.79	19.5
20133213	2011	5	24	6400	124.42	19.57
20133213	2011	5	24	6410	121.61	19.6
20133213	2011	5	24	6420	119.01	19.64
20133213	2011	5	24	6430	116.52	19.69
20133213	2011	5	24	6440	114.09	19.74
20133213	2011	5	24	6450	111.45	19.82
20133213	2011	5	24	6460	108.58	19.9
20133213	2011	5	24	6470	105.96	20.02
20133213	2011	5	24	6480	103.61	20.11
20133213	2011	5	24	6490	101.18	20.2
20133213	2011	5	24	6500	98.76	20.29
20133213	2011	5	24	6510	96.32	20.4
20133213	2011	5	24	6520	93.51	20.51
20133213	2011	5	24	6530	90.38	20.6
20133213	2011	5	24	6540	87.33	20.71
20133213	2011	5	24	6550	84.54	20.82
20133213	2011	5	24	6560	81.73	20.96
20133213	2011	5	24	6570	78.94	21.12
20133213	2011	5	24	6580	76.12	21.3
20133213	2011	5	24	6590	73.07	21.58
20133213	2011	5	24	6600	70.94	21.7
20133213	2011	5	24	6610	68.9	21.89
20133213	2011	5	24	6620	66.53	22.07
20133213	2011	5	24	6630	64.22	22.25
20133213	2011	5	24	6640	61.94	22.43
20133213	2011	5	24	6650	59.55	22.59
20133213	2011	5	24	6660	57.1	22.78
20133213	2011	5	24	6670	54.71	23.01
20133213	2011	5	24	6680	52.39	23.17
20133213	2011	5	24	6690	49.95	23.35
20133213	2011	5	24	6700	47.53	23.56
20133213	2011	5	24	6710	45.15	23.73
20133213	2011	5	24	6720	43.73	23.89
20133213	2011	5	24	6730	42.85	24.03
20133213	2011	5	24	6740	41.26	24.21
20133213	2011	5	24	6750	38.88	24.35
20133213	2011	5	24	6760	36.57	24.52
20133213	2011	5	24	6770	33.91	24.7
20133213	2011	5	24	6780	31.2	24.81
20133213	2011	5	24	6790	28.4	24.95
20133213	2011	5	24	6800	26.25	25.13
20133213	2011	5	24	6810	24.02	25.24
20133213	2011	5	24	6820	22.89	25.34

20133213	2011	5	24	6830	20.99	25.49
20133213	2011	5	24	6840	18.16	25.64
20133213	2011	5	24	6850	15.07	25.81
20133213	2011	5	24	6860	11.92	25.96
20133213	2011	5	24	6870	9.25	26.09
20133213	2011	5	24	6880	7.19	26.28
20133213	2011	5	24	6890	4.27	26.4
20133213	2011	5	24	6900	1.94	26.56
20133213	2011	5	24	6910	1.78	26.85
20133213	2011	5	24	6920	1.67	26.95
20133213	2011	5	24	6930	1.76	27
20133213	2011	5	24	6940	1.9	27.06
20133213	2011	5	24	6950	2.04	27.12
20133213	2011	5	24	6960	1.76	27.17
20133213	2011	5	24	6970	0.58	27.32
20133213	2011	5	24	6980	0.57	27.39
20133213	2011	5	24	6990	0.58	27.48
20133213	2011	5	24	7000	0.58	27.54
20133213	2011	5	24	7010	0.59	27.68
20133213	2011	5	24	7020	0.57	27.73
20133213	2011	5	24	7030	0.6	27.78
20133213	2011	5	24	7040	0.57	27.84
20133213	2011	5	24	7050	0.56	27.89
20133213	2011	5	24	7060	0.53	27.93
20133213	2011	5	24	7070	0.46	27.99
20133213	2011	5	24	7080	0.46	28.03
20133213	2011	5	24	7090	0.44	28.09
20133213	2011	5	24	7100	0.47	28.15
20133213	2011	5	24	7110	0.47	28.18
20283843	2011	5	26	0	1.01	28.63
20283843	2011	5	26	10	1.85	28.62
20283843	2011	5	26	20	2.7	28.69
20283843	2011	5	26	30	2.86	28.67
20283843	2011	5	26	40	2.78	28.68
20283843	2011	5	26	50	2.87	28.68
20283843	2011	5	26	60	2.74	28.67
20283843	2011	5	26	70	2.59	28.68
20283843	2011	5	26	80	2.89	28.68
20283843	2011	5	26	90	3.18	28.68
20283843	2011	5	26	100	4.17	28.67
20283843	2011	5	26	110	5.36	28.67
20283843	2011	5	26	120	6.33	28.66
20283843	2011	5	26	130	8.24	28.64
20283843	2011	5	26	140	9.76	28.64
20283843	2011	5	26	150	11.03	28.62
20283843	2011	5	26	160	12.01	28.61
20283843	2011	5	26	170	13.04	28.6

20283843	2011	5	26	180	13.6	28.6
20283843	2011	5	26	190	14.02	28.6
20283843	2011	5	26	200	14.14	28.6
20283843	2011	5	26	210	13.99	28.6
20283843	2011	5	26	220	13.89	28.6
20283843	2011	5	26	230	14.1	28.6
20283843	2011	5	26	240	14.37	28.6
20283843	2011	5	26	250	14.24	28.6
20283843	2011	5	26	260	14.07	28.6
20283843	2011	5	26	270	14.29	28.6
20283843	2011	5	26	280	14.55	28.6
20283843	2011	5	26	290	15.09	28.6
20283843	2011	5	26	300	15.8	28.6
20283843	2011	5	26	310	15.86	28.6
20283843	2011	5	26	320	15.44	28.59
20283843	2011	5	26	330	15.47	28.6
20283843	2011	5	26	340	15.77	28.6
20283843	2011	5	26	350	16.1	28.6
20283843	2011	5	26	360	16.58	28.59
20283843	2011	5	26	370	17.09	28.59
20283843	2011	5	26	380	17.71	28.58
20283843	2011	5	26	390	18.16	28.58
20283843	2011	5	26	400	18.51	28.57
20283843	2011	5	26	410	19.69	28.56
20283843	2011	5	26	420	21.67	28.54
20283843	2011	5	26	430	23.06	28.53
20283843	2011	5	26	440	25.23	28.51
20283843	2011	5	26	450	27.25	28.5
20283843	2011	5	26	460	29	28.49
20283843	2011	5	26	470	31.21	28.46
20283843	2011	5	26	480	33.64	28.45
20283843	2011	5	26	490	35.73	28.42
20283843	2011	5	26	500	37.72	28.41
20283843	2011	5	26	510	39.82	28.39
20283843	2011	5	26	520	42.64	28.37
20283843	2011	5	26	530	45.94	28.28
20283843	2011	5	26	540	48.7	28.14
20283843	2011	5	26	550	51.4	28.07
20283843	2011	5	26	560	54.58	28.06
20283843	2011	5	26	570	57.62	28
20283843	2011	5	26	580	59.28	27.94
20283843	2011	5	26	590	60.83	27.91
20283843	2011	5	26	600	61.4	27.88
20283843	2011	5	26	610	61.7	27.85
20283843	2011	5	26	620	61.91	27.82
20283843	2011	5	26	630	61.96	27.8
20283843	2011	5	26	640	61.41	27.77

20283843	2011	5	26	650	61.4	27.72
20283843	2011	5	26	660	61.53	27.67
20283843	2011	5	26	670	61.79	27.64
20283843	2011	5	26	680	61.75	27.58
20283843	2011	5	26	690	61.44	27.53
20283843	2011	5	26	700	61.24	27.48
20283843	2011	5	26	710	59.97	27.45
20283843	2011	5	26	720	58.9	27.42
20283843	2011	5	26	730	58.18	27.39
20283843	2011	5	26	740	58.06	27.35
20283843	2011	5	26	750	58.47	27.32
20283843	2011	5	26	760	58.92	27.29
20283843	2011	5	26	770	58.84	27.28
20283843	2011	5	26	780	58.77	27.28
20283843	2011	5	26	790	58.72	27.26
20283843	2011	5	26	800	58.88	27.25
20283843	2011	5	26	810	58.57	27.25
20283843	2011	5	26	820	58.26	27.24
20283843	2011	5	26	830	56.59	27.24
20283843	2011	5	26	840	54.32	27.24
20283843	2011	5	26	850	51.89	27.26
20283843	2011	5	26	860	49.89	27.26
20283843	2011	5	26	870	49.99	27.26
20283843	2011	5	26	880	50.47	27.25
20283843	2011	5	26	890	51.71	27.24
20283843	2011	5	26	900	52.48	27.23
20283843	2011	5	26	910	52.65	27.23
20283843	2011	5	26	920	51.41	27.24
20283843	2011	5	26	930	50.51	27.24
20283843	2011	5	26	940	50.65	27.24
20283843	2011	5	26	950	51.96	27.22
20283843	2011	5	26	960	53.3	27.21
20283843	2011	5	26	970	52.31	27.21
20283843	2011	5	26	980	51.03	27.22
20283843	2011	5	26	990	51.04	27.21
20283843	2011	5	26	1000	51.94	27.2
20283843	2011	5	26	1010	51.79	27.2
20283843	2011	5	26	1020	51.84	27.2
20283843	2011	5	26	1030	51.76	27.21
20283843	2011	5	26	1040	51.73	27.2
20283843	2011	5	26	1050	51.83	27.2
20283843	2011	5	26	1060	51.89	27.19
20283843	2011	5	26	1070	51.71	27.19
20283843	2011	5	26	1080	51.88	27.2
20283843	2011	5	26	1090	51.84	27.18
20283843	2011	5	26	1100	52.1	27.18
20283843	2011	5	26	1110	52.51	27.18

20283843	2011	5	26	1120	52.87	27.17
20283843	2011	5	26	1130	53.61	27.17
20283843	2011	5	26	1140	54.01	27.17
20283843	2011	5	26	1150	54.26	27.17
20283843	2011	5	26	1160	54.48	27.17
20283843	2011	5	26	1170	54.55	27.17
20283843	2011	5	26	1180	54.63	27.17
20283843	2011	5	26	1190	54.7	27.16
20283843	2011	5	26	1200	54.77	27.17
20283843	2011	5	26	1210	54.63	27.16
20283843	2011	5	26	1220	54.47	27.16
20283843	2011	5	26	1230	54.39	27.16
20283843	2011	5	26	1240	54.42	27.16
20283843	2011	5	26	1250	54.54	27.16
20283843	2011	5	26	1260	54.5	27.16
20283843	2011	5	26	1270	54.76	27.15
20283843	2011	5	26	1280	54.87	27.14
20283843	2011	5	26	1290	54.97	27.15
20283843	2011	5	26	1300	54.83	27.15
20283843	2011	5	26	1310	55.09	27.15
20283843	2011	5	26	1320	54.69	27.15
20283843	2011	5	26	1330	54.37	27.14
20283843	2011	5	26	1340	54.07	27.15
20283843	2011	5	26	1350	53.61	27.15
20283843	2011	5	26	1360	53.59	27.15
20283843	2011	5	26	1370	54.04	27.14
20283843	2011	5	26	1380	54.54	27.14
20283843	2011	5	26	1390	53.89	27.15
20283843	2011	5	26	1400	53.83	27.14
20283843	2011	5	26	1410	54.3	27.14
20283843	2011	5	26	1420	54.43	27.14
20283843	2011	5	26	1430	54.54	27.14
20283843	2011	5	26	1440	54.79	27.14
20283843	2011	5	26	1450	54.85	27.14
20283843	2011	5	26	1460	54.82	27.14
20283843	2011	5	26	1470	54.79	27.14
20283843	2011	5	26	1480	54.49	27.14
20283843	2011	5	26	1490	54.39	27.14
20283843	2011	5	26	1500	54.37	27.14
20283843	2011	5	26	1510	54.32	27.13
20283843	2011	5	26	1520	54.24	27.14
20283843	2011	5	26	1530	54.12	27.13
20283843	2011	5	26	1540	52.95	27.14
20283843	2011	5	26	1550	52.26	27.14
20283843	2011	5	26	1560	51.15	27.15
20283843	2011	5	26	1570	49.2	27.16
20283843	2011	5	26	1580	47.53	27.17

20283843	2011	5	26	1590	46.3	27.18
20283843	2011	5	26	1600	45.57	27.2
20283843	2011	5	26	1610	45	27.21
20283843	2011	5	26	1620	44.55	27.21
20283843	2011	5	26	1630	44.6	27.24
20283843	2011	5	26	1640	44.69	27.26
20283843	2011	5	26	1650	44.4	27.27
20283843	2011	5	26	1660	44.35	27.28
20283843	2011	5	26	1670	44.57	27.28
20283843	2011	5	26	1680	44.65	27.3
20283843	2011	5	26	1690	44.53	27.32
20283843	2011	5	26	1700	44.33	27.34
20283843	2011	5	26	1710	44.13	27.35
20283843	2011	5	26	1720	44.15	27.36
20283843	2011	5	26	1730	43.59	27.37
20283843	2011	5	26	1740	43.36	27.38
20283843	2011	5	26	1750	43.6	27.39
20283843	2011	5	26	1760	43.88	27.39
20283843	2011	5	26	1770	43.89	27.39
20283843	2011	5	26	1780	43.99	27.41
20283843	2011	5	26	1790	44.01	27.41
20283843	2011	5	26	1800	43.87	27.43
20283843	2011	5	26	1810	43.88	27.43
20283843	2011	5	26	1820	43.93	27.43
20283843	2011	5	26	1830	43.81	27.44
20283843	2011	5	26	1840	44.12	27.44
20283843	2011	5	26	1850	44.59	27.45
20283843	2011	5	26	1860	44.96	27.45
20283843	2011	5	26	1870	45.92	27.46
20283843	2011	5	26	1880	46.2	27.45
20283843	2011	5	26	1890	46.44	27.46
20283843	2011	5	26	1900	46.47	27.46
20283843	2011	5	26	1910	46.56	27.46
20283843	2011	5	26	1920	45.99	27.46
20283843	2011	5	26	1930	45.88	27.47
20283843	2011	5	26	1940	45.97	27.46
20283843	2011	5	26	1950	46.14	27.47
20283843	2011	5	26	1960	46.15	27.47
20283843	2011	5	26	1970	45.63	27.47
20283843	2011	5	26	1980	45.56	27.48
20283843	2011	5	26	1990	45.71	27.48
20283843	2011	5	26	2000	45.59	27.48
20283843	2011	5	26	2010	45.61	27.48
20283843	2011	5	26	2020	46.02	27.47
20283843	2011	5	26	2030	46.45	27.47
20283843	2011	5	26	2040	46.29	27.48
20283843	2011	5	26	2050	46.24	27.48

20283843	2011	5	26	2060	46.58	27.47
20283843	2011	5	26	2070	47.08	27.47
20283843	2011	5	26	2080	47.38	27.47
20283843	2011	5	26	2090	47.36	27.47
20283843	2011	5	26	2100	47.45	27.47
20283843	2011	5	26	2110	47.75	27.47
20283843	2011	5	26	2120	48.13	27.46
20283843	2011	5	26	2130	48.18	27.46
20283843	2011	5	26	2140	48.74	27.46
20283843	2011	5	26	2150	48.81	27.46
20283843	2011	5	26	2160	48.6	27.46
20283843	2011	5	26	2170	48.13	27.46
20283843	2011	5	26	2180	48.12	27.46
20283843	2011	5	26	2190	48.54	27.46
20283843	2011	5	26	2200	48.88	27.46
20283843	2011	5	26	2210	49.03	27.46
20283843	2011	5	26	2220	48.89	27.46
20283843	2011	5	26	2230	48.78	27.45
20283843	2011	5	26	2240	48.6	27.46
20283843	2011	5	26	2250	48.43	27.46
20283843	2011	5	26	2260	47.17	27.46
20283843	2011	5	26	2270	46.81	27.47
20283843	2011	5	26	2280	46.82	27.46
20283843	2011	5	26	2290	46.88	27.47
20283843	2011	5	26	2300	47.24	27.46
20283843	2011	5	26	2310	48.11	27.47
20283843	2011	5	26	2320	48.4	27.46
20283843	2011	5	26	2330	47.85	27.46
20283843	2011	5	26	2340	48.05	27.46
20283843	2011	5	26	2350	49.29	27.44
20283843	2011	5	26	2360	49.82	27.45
20283843	2011	5	26	2370	50.26	27.44
20283843	2011	5	26	2380	50.76	27.42
20283843	2011	5	26	2390	51.22	27.42
20283843	2011	5	26	2400	51.55	27.41
20283843	2011	5	26	2410	51.85	27.39
20283843	2011	5	26	2420	51.89	27.39
20283843	2011	5	26	2430	51.84	27.39
20283843	2011	5	26	2440	51.7	27.38
20283843	2011	5	26	2450	51.9	27.37
20283843	2011	5	26	2460	51.9	27.36
20283843	2011	5	26	2470	51.88	27.35
20283843	2011	5	26	2480	52.18	27.35
20283843	2011	5	26	2490	52.11	27.34
20283843	2011	5	26	2500	52.03	27.34
20283843	2011	5	26	2510	52	27.34
20283843	2011	5	26	2520	51.97	27.33

20283843	2011	5	26	2530	52.19	27.32
20283843	2011	5	26	2540	52.33	27.32
20283843	2011	5	26	2550	53.27	27.31
20283843	2011	5	26	2560	53.12	27.3
20283843	2011	5	26	2570	52.89	27.29
20283843	2011	5	26	2580	52.94	27.28
20283843	2011	5	26	2590	53.36	27.28
20283843	2011	5	26	2600	53.53	27.25
20283843	2011	5	26	2610	53.69	27.25
20283843	2011	5	26	2620	53.51	27.25
20283843	2011	5	26	2630	53.5	27.23
20283843	2011	5	26	2640	53.3	27.22
20283843	2011	5	26	2650	52.61	27.22
20283843	2011	5	26	2660	52.49	27.22
20283843	2011	5	26	2670	52.22	27.21
20283843	2011	5	26	2680	51.87	27.21
20283843	2011	5	26	2690	51.76	27.2
20283843	2011	5	26	2700	51.87	27.19
20283843	2011	5	26	2710	51.99	27.19
20283843	2011	5	26	2720	51.85	27.2
20283843	2011	5	26	2730	51.67	27.19
20283843	2011	5	26	2740	51.72	27.19
20283843	2011	5	26	2750	51.8	27.19
20283843	2011	5	26	2760	51.64	27.19
20283843	2011	5	26	2770	51.75	27.17
20283843	2011	5	26	2780	51.99	27.18
20283843	2011	5	26	2790	52.24	27.17
20283843	2011	5	26	2800	52.62	27.17
20283843	2011	5	26	2810	53.14	27.17
20283843	2011	5	26	2820	53.6	27.17
20283843	2011	5	26	2830	54.91	27.16
20283843	2011	5	26	2840	55.76	27.14
20283843	2011	5	26	2850	56.28	27.13
20283843	2011	5	26	2860	57.27	27.09
20283843	2011	5	26	2870	59.27	27.08
20283843	2011	5	26	2880	61.42	27.06
20283843	2011	5	26	2890	63.96	27.02
20283843	2011	5	26	2900	66.62	27
20283843	2011	5	26	2910	68.67	26.97
20283843	2011	5	26	2920	70.37	26.95
20283843	2011	5	26	2930	72.55	26.89
20283843	2011	5	26	2940	74.78	26.74
20283843	2011	5	26	2950	77.3	26.65
20283843	2011	5	26	2960	80.46	26.6
20283843	2011	5	26	2970	83.75	26.53
20283843	2011	5	26	2980	85.47	26.46
20283843	2011	5	26	2990	86.26	26.41

20283843	2011	5	26	3000	86.16	26.35
20283843	2011	5	26	3010	86.31	26.3
20283843	2011	5	26	3020	86.39	26.25
20283843	2011	5	26	3030	86.4	26.18
20283843	2011	5	26	3040	86.3	26.12
20283843	2011	5	26	3050	86.13	26.07
20283843	2011	5	26	3060	85.95	26
20283843	2011	5	26	3070	85.97	25.93
20283843	2011	5	26	3080	85.98	25.9
20283843	2011	5	26	3090	85.99	25.85
20283843	2011	5	26	3100	86.03	25.82
20283843	2011	5	26	3110	85.98	25.77
20283843	2011	5	26	3120	85.91	25.71
20283843	2011	5	26	3130	85.86	25.67
20283843	2011	5	26	3140	85.72	25.64
20283843	2011	5	26	3150	85.54	25.58
20283843	2011	5	26	3160	85.36	25.54
20283843	2011	5	26	3170	86.47	25.49
20283843	2011	5	26	3180	88.14	25.45
20283843	2011	5	26	3190	90.28	25.39
20283843	2011	5	26	3200	91.91	25.32
20283843	2011	5	26	3210	92.89	25.28
20283843	2011	5	26	3220	93.83	25.2
20283843	2011	5	26	3230	94.43	25.15
20283843	2011	5	26	3240	94.71	25.1
20283843	2011	5	26	3250	94.85	25.06
20283843	2011	5	26	3260	95.01	24.99
20283843	2011	5	26	3270	94.95	24.94
20283843	2011	5	26	3280	94.96	24.88
20283843	2011	5	26	3290	95.36	24.81
20283843	2011	5	26	3300	95.63	24.76
20283843	2011	5	26	3310	95.53	24.7
20283843	2011	5	26	3320	95.14	24.6
20283843	2011	5	26	3330	95.23	24.57
20283843	2011	5	26	3340	95.31	24.53
20283843	2011	5	26	3350	95.37	24.48
20283843	2011	5	26	3360	95.31	24.45
20283843	2011	5	26	3370	95.34	24.39
20283843	2011	5	26	3380	95.93	24.32
20283843	2011	5	26	3390	97.08	24.26
20283843	2011	5	26	3400	97.66	24.22
20283843	2011	5	26	3410	98.22	24.17
20283843	2011	5	26	3420	98.96	24.12
20283843	2011	5	26	3430	99.6	24.07
20283843	2011	5	26	3440	99.84	24.01
20283843	2011	5	26	3450	100.15	23.96
20283843	2011	5	26	3460	100.58	23.92

20283843	2011	5	26	3470	100.82	23.88
20283843	2011	5	26	3480	101.12	23.84
20283843	2011	5	26	3490	101.55	23.79
20283843	2011	5	26	3500	101.72	23.75
20283843	2011	5	26	3510	101.56	23.72
20283843	2011	5	26	3520	101.23	23.68
20283843	2011	5	26	3530	101.35	23.63
20283843	2011	5	26	3540	102.48	23.6
20283843	2011	5	26	3550	102.82	23.57
20283843	2011	5	26	3560	103.56	23.53
20283843	2011	5	26	3570	104.21	23.5
20283843	2011	5	26	3580	104.41	23.46
20283843	2011	5	26	3590	104.53	23.38
20283843	2011	5	26	3600	104.88	23.32
20283843	2011	5	26	3610	105.88	23.2
20283843	2011	5	26	3620	107.01	23.1
20283843	2011	5	26	3630	108.22	23.01
20283843	2011	5	26	3640	109.51	22.94
20283843	2011	5	26	3650	110.57	22.85
20283843	2011	5	26	3660	111.59	22.75
20283843	2011	5	26	3670	112.52	22.75
20283843	2011	5	26	3680	113.77	22.61
20283843	2011	5	26	3690	114.96	22.6
20283843	2011	5	26	3700	116.12	22.52
20283843	2011	5	26	3710	117.36	22.44
20283843	2011	5	26	3720	118.59	22.36
20283843	2011	5	26	3730	119.64	22.29
20283843	2011	5	26	3740	120.92	22.27
20283843	2011	5	26	3750	122.15	22.17
20283843	2011	5	26	3760	123.38	22.06
20283843	2011	5	26	3770	124.69	21.96
20283843	2011	5	26	3780	126.5	21.68
20283843	2011	5	26	3790	127.07	21.74
20283843	2011	5	26	3800	127.92	21.7
20283843	2011	5	26	3810	129.31	21.6
20283843	2011	5	26	3820	130.3	21.59
20283843	2011	5	26	3830	131.87	21.45
20283843	2011	5	26	3840	132.12	21.44
20283843	2011	5	26	3850	132.29	21.42
20283843	2011	5	26	3860	132.87	21.37
20283843	2011	5	26	3870	133.12	21.35
20283843	2011	5	26	3880	133.02	21.33
20283843	2011	5	26	3890	132.97	21.3
20283843	2011	5	26	3900	133.11	21.25
20283843	2011	5	26	3910	133.09	21.21
20283843	2011	5	26	3920	133.16	21.11
20283843	2011	5	26	3930	133.23	21.07

20283843	2011	5	26	3940	132.93	21.06
20283843	2011	5	26	3950	132.47	21.03
20283843	2011	5	26	3960	131.92	21.01
20283843	2011	5	26	3970	131.19	20.99
20283843	2011	5	26	3980	130.65	20.96
20283843	2011	5	26	3990	130.3	20.94
20283843	2011	5	26	4000	131.21	20.89
20283843	2011	5	26	4010	132.23	20.85
20283843	2011	5	26	4020	132.12	20.82
20283843	2011	5	26	4030	132.35	20.78
20283843	2011	5	26	4040	132.26	20.75
20283843	2011	5	26	4050	131.68	20.73
20283843	2011	5	26	4060	131.16	20.71
20283843	2011	5	26	4070	131.19	20.7
20283843	2011	5	26	4080	130.77	20.68
20283843	2011	5	26	4090	130.54	20.67
20283843	2011	5	26	4100	129.84	20.65
20283843	2011	5	26	4110	129.61	20.64
20283843	2011	5	26	4120	130.13	20.62
20283843	2011	5	26	4130	130.18	20.6
20283843	2011	5	26	4140	130.31	20.59
20283843	2011	5	26	4150	130.46	20.57
20283843	2011	5	26	4160	130.31	20.57
20283843	2011	5	26	4170	129.31	20.57
20283843	2011	5	26	4180	127.03	20.56
20283843	2011	5	26	4190	125.82	20.56
20283843	2011	5	26	4200	126.07	20.56
20283843	2011	5	26	4210	126.01	20.57
20283843	2011	5	26	4220	125.53	20.57
20283843	2011	5	26	4230	125.36	20.57
20283843	2011	5	26	4240	125.26	20.57
20283843	2011	5	26	4250	123.64	20.58
20283843	2011	5	26	4260	122.71	20.58
20283843	2011	5	26	4270	122.14	20.61
20283843	2011	5	26	4280	121.07	20.62
20283843	2011	5	26	4290	120.05	20.65
20283843	2011	5	26	4300	119.49	20.67
20283843	2011	5	26	4310	119.09	20.75
20283843	2011	5	26	4320	118.8	20.78
20283843	2011	5	26	4330	118.62	20.79
20283843	2011	5	26	4340	118.55	20.83
20283843	2011	5	26	4350	118.45	20.88
20283843	2011	5	26	4360	118.22	20.9
20283843	2011	5	26	4370	116.41	20.93
20283843	2011	5	26	4380	114.01	20.96
20283843	2011	5	26	4390	111.59	21
20283843	2011	5	26	4400	109.17	21.06

20283843	2011	5	26	4410	106.95	21.09
20283843	2011	5	26	4420	105.02	21.14
20283843	2011	5	26	4430	102.92	21.21
20283843	2011	5	26	4440	100.91	21.32
20283843	2011	5	26	4450	98.85	21.42
20283843	2011	5	26	4460	96.27	21.5
20283843	2011	5	26	4470	94.14	21.61
20283843	2011	5	26	4480	92.04	21.71
20283843	2011	5	26	4490	89.96	21.82
20283843	2011	5	26	4500	87.5	21.96
20283843	2011	5	26	4510	85.01	22.1
20283843	2011	5	26	4520	82.81	22.33
20283843	2011	5	26	4530	80.54	22.49
20283843	2011	5	26	4540	79.3	22.61
20283843	2011	5	26	4550	77.16	22.76
20283843	2011	5	26	4560	74.9	22.87
20283843	2011	5	26	4570	73.48	22.99
20283843	2011	5	26	4580	71.53	23.1
20283843	2011	5	26	4590	69.41	23.25
20283843	2011	5	26	4600	67.15	23.42
20283843	2011	5	26	4610	66.44	23.55
20283843	2011	5	26	4620	66.66	23.7
20283843	2011	5	26	4630	66.83	23.82
20283843	2011	5	26	4640	66.7	23.91
20283843	2011	5	26	4650	66.15	24.01
20283843	2011	5	26	4660	64.32	24.13
20283843	2011	5	26	4670	61.95	24.21
20283843	2011	5	26	4680	60.73	24.3
20283843	2011	5	26	4690	59.09	24.43
20283843	2011	5	26	4700	57.2	24.53
20283843	2011	5	26	4710	56.05	24.66
20283843	2011	5	26	4720	54.31	24.78
20283843	2011	5	26	4730	53.75	24.92
20283843	2011	5	26	4740	53.37	25.1
20283843	2011	5	26	4750	52.45	25.21
20283843	2011	5	26	4760	51.93	25.3
20283843	2011	5	26	4770	51.26	25.38
20283843	2011	5	26	4780	50.16	25.51
20283843	2011	5	26	4790	49.51	25.57
20283843	2011	5	26	4800	49.43	25.67
20283843	2011	5	26	4810	49.19	25.79
20283843	2011	5	26	4820	48.79	25.89
20283843	2011	5	26	4830	48.75	26
20283843	2011	5	26	4840	48.81	26.07
20283843	2011	5	26	4850	48.54	26.15
20283843	2011	5	26	4860	48.15	26.22
20283843	2011	5	26	4870	47.93	26.27

20283843	2011	5	26	4880	47.58	26.35
20283843	2011	5	26	4890	47.22	26.44
20283843	2011	5	26	4900	46.09	26.48
20283843	2011	5	26	4910	45.82	26.55
20283843	2011	5	26	4920	45.95	26.6
20283843	2011	5	26	4930	46.02	26.66
20283843	2011	5	26	4940	45.84	26.7
20283843	2011	5	26	4950	45.83	26.73
20283843	2011	5	26	4960	46.12	26.76
20283843	2011	5	26	4970	46.46	26.79
20283843	2011	5	26	4980	46.27	26.83
20283843	2011	5	26	4990	44.54	26.86
20283843	2011	5	26	5000	42.32	26.9
20283843	2011	5	26	5010	39.96	26.94
20283843	2011	5	26	5020	38.04	26.98
20283843	2011	5	26	5030	36.28	27.03
20283843	2011	5	26	5040	34.31	27.07
20283843	2011	5	26	5050	32.56	27.12
20283843	2011	5	26	5060	30.65	27.2
20283843	2011	5	26	5070	29.05	27.26
20283843	2011	5	26	5080	28.28	27.35
20283843	2011	5	26	5090	27.04	27.42
20283843	2011	5	26	5100	25.78	27.48
20283843	2011	5	26	5110	24.07	27.54
20283843	2011	5	26	5120	21.87	27.6
20283843	2011	5	26	5130	19.31	27.67
20283843	2011	5	26	5140	17.2	27.74
20283843	2011	5	26	5150	16.34	27.78
20283843	2011	5	26	5160	16.11	27.82
20283843	2011	5	26	5170	15.44	27.86
20283843	2011	5	26	5180	15.12	27.92
20283843	2011	5	26	5190	13.44	27.94
20283843	2011	5	26	5200	11.25	27.99
20283843	2011	5	26	5210	8.82	28.04
20283843	2011	5	26	5220	6.75	28.1
20283843	2011	5	26	5230	4.6	28.15
20283843	2011	5	26	5240	3.68	28.17
20283843	2011	5	26	5250	2.83	28.22
20283843	2011	5	26	5260	2.7	28.27
20283843	2011	5	26	5270	2.65	28.28
20283843	2011	5	26	5280	2.64	28.3
20283843	2011	5	26	5290	2.86	28.33
20283843	2011	5	26	5300	2.5	28.35
20283843	2011	5	26	5310	0.65	28.39
20283843	2011	5	26	5320	0.67	28.4
20283843	2011	5	26	5330	0.65	28.42
20283843	2011	5	26	5340	0.63	28.44

20283843	2011	5	26	5350	0.63	28.46
20283843	2011	5	26	5360	0.68	28.48
20283843	2011	5	26	5370	0.62	28.5
20283843	2011	5	26	5380	0.67	28.52
20283843	2011	5	26	5390	0.64	28.53
20283843	2011	5	26	5400	0.67	28.55
20283843	2011	5	26	5410	0.61	28.57
20283843	2011	5	26	5420	0.56	28.58
20283843	2011	5	26	5430	0.59	28.6
20283843	2011	5	26	5440	0.58	28.6
20283843	2011	5	26	5450	0.6	28.63
20293083	2011	5	26	0	1.01	29.05
20293083	2011	5	26	10	1.74	29.05
20293083	2011	5	26	20	2.3	29.04
20293083	2011	5	26	30	2.79	29.03
20293083	2011	5	26	40	2.75	29.04
20293083	2011	5	26	50	2.32	29.03
20293083	2011	5	26	60	2.4	29.03
20293083	2011	5	26	70	2.61	29.03
20293083	2011	5	26	80	3	29.03
20293083	2011	5	26	90	3.41	29.02
20293083	2011	5	26	100	3.62	29
20293083	2011	5	26	110	4.84	28.97
20293083	2011	5	26	120	7.2	28.92
20293083	2011	5	26	130	8.74	28.9
20293083	2011	5	26	140	9.78	28.89
20293083	2011	5	26	150	11	28.87
20293083	2011	5	26	160	11.36	28.85
20293083	2011	5	26	170	11.38	28.85
20293083	2011	5	26	180	11.83	28.84
20293083	2011	5	26	190	12.25	28.82
20293083	2011	5	26	200	12.4	28.82
20293083	2011	5	26	210	11.77	28.8
20293083	2011	5	26	220	11.85	28.79
20293083	2011	5	26	230	12.25	28.78
20293083	2011	5	26	240	12.85	28.77
20293083	2011	5	26	250	13.65	28.75
20293083	2011	5	26	260	14.54	28.75
20293083	2011	5	26	270	15.2	28.73
20293083	2011	5	26	280	15.5	28.71
20293083	2011	5	26	290	15.83	28.71
20293083	2011	5	26	300	16.46	28.7
20293083	2011	5	26	310	17.07	28.68
20293083	2011	5	26	320	17.16	28.68
20293083	2011	5	26	330	17.31	28.67
20293083	2011	5	26	340	17.63	28.67
20293083	2011	5	26	350	17.84	28.65

20293083	2011	5	26	360	18.07	28.64
20293083	2011	5	26	370	18.53	28.64
20293083	2011	5	26	380	20.16	28.61
20293083	2011	5	26	390	21.59	28.6
20293083	2011	5	26	400	22.49	28.58
20293083	2011	5	26	410	24.22	28.57
20293083	2011	5	26	420	26.22	28.53
20293083	2011	5	26	430	28.34	28.51
20293083	2011	5	26	440	30.41	28.49
20293083	2011	5	26	450	32.3	28.46
20293083	2011	5	26	460	34.38	28.44
20293083	2011	5	26	470	36.4	28.42
20293083	2011	5	26	480	39	28.39
20293083	2011	5	26	490	41.63	28.34
20293083	2011	5	26	500	44.68	28.21
20293083	2011	5	26	510	48.13	28.14
20293083	2011	5	26	520	51.29	28.05
20293083	2011	5	26	530	53.64	27.93
20293083	2011	5	26	540	55.45	27.91
20293083	2011	5	26	550	57.86	27.82
20293083	2011	5	26	560	60.07	27.71
20293083	2011	5	26	570	62.03	27.53
20293083	2011	5	26	580	63.67	27.53
20293083	2011	5	26	590	66.15	27.42
20293083	2011	5	26	600	68.75	27.35
20293083	2011	5	26	610	70.56	27.33
20293083	2011	5	26	620	71.72	27.32
20293083	2011	5	26	630	72.32	27.27
20293083	2011	5	26	640	72.42	27.19
20293083	2011	5	26	650	72.5	27.12
20293083	2011	5	26	660	72.65	27.09
20293083	2011	5	26	670	72.79	27.05
20293083	2011	5	26	680	72.97	27.01
20293083	2011	5	26	690	73.2	26.96
20293083	2011	5	26	700	73.47	26.93
20293083	2011	5	26	710	73.76	26.89
20293083	2011	5	26	720	74.12	26.83
20293083	2011	5	26	730	75.28	26.73
20293083	2011	5	26	740	78.19	26.66
20293083	2011	5	26	750	81.41	26.56
20293083	2011	5	26	760	84.85	26.46
20293083	2011	5	26	770	88.41	26.39
20293083	2011	5	26	780	92	26.29
20293083	2011	5	26	790	95.5	26.16
20293083	2011	5	26	800	98.96	26.02
20293083	2011	5	26	810	102.08	25.85
20293083	2011	5	26	820	104.88	25.78

20293083	2011	5	26	830	107.06	25.59
20293083	2011	5	26	840	108.94	25.34
20293083	2011	5	26	850	110.47	24.91
20293083	2011	5	26	860	111.82	24.78
20293083	2011	5	26	870	113.91	24.46
20293083	2011	5	26	880	116.27	24.28
20293083	2011	5	26	890	118.66	24.11
20293083	2011	5	26	900	121.15	23.95
20293083	2011	5	26	910	123.62	23.68
20293083	2011	5	26	920	124.54	23.61
20293083	2011	5	26	930	124.81	23.6
20293083	2011	5	26	940	124.77	23.45
20293083	2011	5	26	950	125.44	23.23
20293083	2011	5	26	960	125.93	23.14
20293083	2011	5	26	970	126.32	23.09
20293083	2011	5	26	980	126.38	23
20293083	2011	5	26	990	126.3	22.92
20293083	2011	5	26	1000	126.24	22.82
20293083	2011	5	26	1010	126.23	22.73
20293083	2011	5	26	1020	126.3	22.57
20293083	2011	5	26	1030	126.32	22.44
20293083	2011	5	26	1040	126.68	22.35
20293083	2011	5	26	1050	126.54	22.3
20293083	2011	5	26	1060	126.14	22.24
20293083	2011	5	26	1070	125.76	22.19
20293083	2011	5	26	1080	125.32	22.14
20293083	2011	5	26	1090	124.81	22.07
20293083	2011	5	26	1100	124.13	22
20293083	2011	5	26	1110	123.89	21.95
20293083	2011	5	26	1120	123.65	21.91
20293083	2011	5	26	1130	123.62	21.85
20293083	2011	5	26	1140	123.4	21.8
20293083	2011	5	26	1150	123.04	21.78
20293083	2011	5	26	1160	122.7	21.71
20293083	2011	5	26	1170	122.13	21.67
20293083	2011	5	26	1180	121.23	21.66
20293083	2011	5	26	1190	120.99	21.63
20293083	2011	5	26	1200	121.9	21.59
20293083	2011	5	26	1210	123.66	21.53
20293083	2011	5	26	1220	124.19	21.52
20293083	2011	5	26	1230	123.36	21.51
20293083	2011	5	26	1240	122.59	21.51
20293083	2011	5	26	1250	121.67	21.5
20293083	2011	5	26	1260	120.75	21.49
20293083	2011	5	26	1270	120.02	21.49
20293083	2011	5	26	1280	119.3	21.48
20293083	2011	5	26	1290	118.55	21.47

20293083	2011	5	26	1300	118.04	21.46
20293083	2011	5	26	1310	117.68	21.46
20293083	2011	5	26	1320	117.51	21.46
20293083	2011	5	26	1330	118.16	21.46
20293083	2011	5	26	1340	119.49	21.46
20293083	2011	5	26	1350	121.45	21.45
20293083	2011	5	26	1360	123.65	21.41
20293083	2011	5	26	1370	125.65	21.3
20293083	2011	5	26	1380	127.58	21.22
20293083	2011	5	26	1390	129.55	21.17
20293083	2011	5	26	1400	130.95	21.13
20293083	2011	5	26	1410	132.37	21.05
20293083	2011	5	26	1420	133.46	21.04
20293083	2011	5	26	1430	133.9	21.01
20293083	2011	5	26	1440	132.64	20.99
20293083	2011	5	26	1450	131.93	20.96
20293083	2011	5	26	1460	131.46	20.95
20293083	2011	5	26	1470	130.98	20.93
20293083	2011	5	26	1480	130.83	20.9
20293083	2011	5	26	1490	130.98	20.88
20293083	2011	5	26	1500	131.17	20.84
20293083	2011	5	26	1510	131.17	20.81
20293083	2011	5	26	1520	130.9	20.79
20293083	2011	5	26	1530	130.75	20.78
20293083	2011	5	26	1540	130.92	20.74
20293083	2011	5	26	1550	130.96	20.71
20293083	2011	5	26	1560	131.21	20.7
20293083	2011	5	26	1570	131.39	20.67
20293083	2011	5	26	1580	131.5	20.66
20293083	2011	5	26	1590	131.44	20.64
20293083	2011	5	26	1600	131.68	20.64
20293083	2011	5	26	1610	131.93	20.63
20293083	2011	5	26	1620	132.12	20.61
20293083	2011	5	26	1630	132.22	20.59
20293083	2011	5	26	1640	132.18	20.57
20293083	2011	5	26	1650	132.17	20.56
20293083	2011	5	26	1660	132.13	20.55
20293083	2011	5	26	1670	131.96	20.54
20293083	2011	5	26	1680	131.88	20.53
20293083	2011	5	26	1690	131.77	20.53
20293083	2011	5	26	1700	131.58	20.52
20293083	2011	5	26	1710	131.28	20.51
20293083	2011	5	26	1720	130.91	20.5
20293083	2011	5	26	1730	131.25	20.5
20293083	2011	5	26	1740	131.46	20.48
20293083	2011	5	26	1750	131.4	20.48
20293083	2011	5	26	1760	131.65	20.47

20293083	2011	5	26	1770	131.98	20.46
20293083	2011	5	26	1780	132.04	20.46
20293083	2011	5	26	1790	131.79	20.45
20293083	2011	5	26	1800	131.41	20.43
20293083	2011	5	26	1810	130.95	20.42
20293083	2011	5	26	1820	130.51	20.42
20293083	2011	5	26	1830	130.12	20.42
20293083	2011	5	26	1840	129.61	20.42
20293083	2011	5	26	1850	129.47	20.42
20293083	2011	5	26	1860	130.15	20.41
20293083	2011	5	26	1870	131.41	20.4
20293083	2011	5	26	1880	132.89	20.39
20293083	2011	5	26	1890	134.97	20.38
20293083	2011	5	26	1900	136.69	20.35
20293083	2011	5	26	1910	137.96	20.34
20293083	2011	5	26	1920	139.04	20.31
20293083	2011	5	26	1930	139.94	20.24
20293083	2011	5	26	1940	141.93	20.11
20293083	2011	5	26	1950	143.58	20.1
20293083	2011	5	26	1960	145.28	20.06
20293083	2011	5	26	1970	147.54	20
20293083	2011	5	26	1980	149.34	19.96
20293083	2011	5	26	1990	150.75	19.92
20293083	2011	5	26	2000	150.64	19.89
20293083	2011	5	26	2010	150.73	19.84
20293083	2011	5	26	2020	150.82	19.72
20293083	2011	5	26	2030	150.61	19.69
20293083	2011	5	26	2040	150.47	19.67
20293083	2011	5	26	2050	151.06	19.62
20293083	2011	5	26	2060	151.58	19.6
20293083	2011	5	26	2070	153.23	19.54
20293083	2011	5	26	2080	154.16	19.51
20293083	2011	5	26	2090	154.58	19.46
20293083	2011	5	26	2100	154.84	19.43
20293083	2011	5	26	2110	154.85	19.39
20293083	2011	5	26	2120	154.41	19.38
20293083	2011	5	26	2130	153.55	19.34
20293083	2011	5	26	2140	152.29	19.3
20293083	2011	5	26	2150	151.07	19.28
20293083	2011	5	26	2160	150.01	19.24
20293083	2011	5	26	2170	149.17	19.22
20293083	2011	5	26	2180	148.36	19.19
20293083	2011	5	26	2190	147.25	19.16
20293083	2011	5	26	2200	146.12	19.14
20293083	2011	5	26	2210	144.6	19.14
20293083	2011	5	26	2220	144.25	19.16
20293083	2011	5	26	2230	143.32	19.18

20293083	2011	5	26	2240	142.21	19.19
20293083	2011	5	26	2250	141.45	19.2
20293083	2011	5	26	2260	140.93	19.22
20293083	2011	5	26	2270	140.76	19.25
20293083	2011	5	26	2280	140.35	19.27
20293083	2011	5	26	2290	140.22	19.29
20293083	2011	5	26	2300	140.05	19.3
20293083	2011	5	26	2310	139.63	19.34
20293083	2011	5	26	2320	138.96	19.35
20293083	2011	5	26	2330	138.28	19.38
20293083	2011	5	26	2340	137.72	19.39
20293083	2011	5	26	2350	136.93	19.43
20293083	2011	5	26	2360	136.45	19.44
20293083	2011	5	26	2370	137	19.5
20293083	2011	5	26	2380	136.96	19.52
20293083	2011	5	26	2390	136.13	19.55
20293083	2011	5	26	2400	134.65	19.57
20293083	2011	5	26	2410	132.75	19.62
20293083	2011	5	26	2420	131.97	19.65
20293083	2011	5	26	2430	131.48	19.69
20293083	2011	5	26	2440	131.68	19.71
20293083	2011	5	26	2450	131.85	19.74
20293083	2011	5	26	2460	132.02	19.77
20293083	2011	5	26	2470	131.62	19.79
20293083	2011	5	26	2480	131.08	19.82
20293083	2011	5	26	2490	130.34	19.84
20293083	2011	5	26	2500	129.36	19.86
20293083	2011	5	26	2510	128.75	19.89
20293083	2011	5	26	2520	127.72	19.91
20293083	2011	5	26	2530	125.84	19.94
20293083	2011	5	26	2540	124.71	19.97
20293083	2011	5	26	2550	123.49	20
20293083	2011	5	26	2560	123.63	20.03
20293083	2011	5	26	2570	123.71	20.07
20293083	2011	5	26	2580	123.68	20.1
20293083	2011	5	26	2590	123.55	20.11
20293083	2011	5	26	2600	123.1	20.14
20293083	2011	5	26	2610	122.66	20.16
20293083	2011	5	26	2620	122.37	20.18
20293083	2011	5	26	2630	122.1	20.2
20293083	2011	5	26	2640	121.66	20.22
20293083	2011	5	26	2650	121.17	20.26
20293083	2011	5	26	2660	120.28	20.29
20293083	2011	5	26	2670	118.29	20.33
20293083	2011	5	26	2680	117.23	20.39
20293083	2011	5	26	2690	116.95	20.42
20293083	2011	5	26	2700	116.94	20.46

20293083	2011	5	26	2710	116.98	20.5
20293083	2011	5	26	2720	117.27	20.57
20293083	2011	5	26	2730	117.62	20.62
20293083	2011	5	26	2740	117.8	20.68
20293083	2011	5	26	2750	117.78	20.71
20293083	2011	5	26	2760	117.55	20.75
20293083	2011	5	26	2770	117.27	20.78
20293083	2011	5	26	2780	116.96	20.81
20293083	2011	5	26	2790	116.69	20.84
20293083	2011	5	26	2800	116.48	20.86
20293083	2011	5	26	2810	116.3	20.89
20293083	2011	5	26	2820	116.17	20.92
20293083	2011	5	26	2830	116.07	20.96
20293083	2011	5	26	2840	115.84	21
20293083	2011	5	26	2850	114.59	21.05
20293083	2011	5	26	2860	112.51	21.08
20293083	2011	5	26	2870	110.07	21.12
20293083	2011	5	26	2880	108.47	21.16
20293083	2011	5	26	2890	107.5	21.19
20293083	2011	5	26	2900	106.63	21.22
20293083	2011	5	26	2910	106.07	21.27
20293083	2011	5	26	2920	106.01	21.35
20293083	2011	5	26	2930	105.87	21.42
20293083	2011	5	26	2940	105.26	21.46
20293083	2011	5	26	2950	103.4	21.56
20293083	2011	5	26	2960	101.59	21.61
20293083	2011	5	26	2970	100.5	21.71
20293083	2011	5	26	2980	98.43	21.77
20293083	2011	5	26	2990	97.65	21.84
20293083	2011	5	26	3000	97.1	21.93
20293083	2011	5	26	3010	94.96	22.03
20293083	2011	5	26	3020	92.61	22.11
20293083	2011	5	26	3030	90.74	22.22
20293083	2011	5	26	3040	89.8	22.32
20293083	2011	5	26	3050	88.02	22.46
20293083	2011	5	26	3060	87.07	22.53
20293083	2011	5	26	3070	86.59	22.62
20293083	2011	5	26	3080	86.42	22.81
20293083	2011	5	26	3090	85.67	22.9
20293083	2011	5	26	3100	83.95	23
20293083	2011	5	26	3110	82.5	23.12
20293083	2011	5	26	3120	80.24	23.28
20293083	2011	5	26	3130	77.8	23.45
20293083	2011	5	26	3140	75.19	23.51
20293083	2011	5	26	3150	72.54	23.64
20293083	2011	5	26	3160	70.18	23.74
20293083	2011	5	26	3170	70.14	23.86

20293083	2011	5	26	3180	70.3	23.95
20293083	2011	5	26	3190	70.15	24.05
20293083	2011	5	26	3200	70.07	24.15
20293083	2011	5	26	3210	70.04	24.21
20293083	2011	5	26	3220	70.17	24.26
20293083	2011	5	26	3230	69.94	24.32
20293083	2011	5	26	3240	70.03	24.37
20293083	2011	5	26	3250	70.04	24.44
20293083	2011	5	26	3260	69.93	24.5
20293083	2011	5	26	3270	69.63	24.57
20293083	2011	5	26	3280	69.08	24.65
20293083	2011	5	26	3290	68.27	24.71
20293083	2011	5	26	3300	67.62	24.76
20293083	2011	5	26	3310	66.34	24.85
20293083	2011	5	26	3320	65.65	24.89
20293083	2011	5	26	3330	64.78	24.96
20293083	2011	5	26	3340	63.52	25
20293083	2011	5	26	3350	62.69	25.04
20293083	2011	5	26	3360	60.79	25.1
20293083	2011	5	26	3370	58.81	25.16
20293083	2011	5	26	3380	56.81	25.27
20293083	2011	5	26	3390	54.76	25.36
20293083	2011	5	26	3400	52.38	25.44
20293083	2011	5	26	3410	49.69	25.51
20293083	2011	5	26	3420	46.96	25.65
20293083	2011	5	26	3430	45.78	25.75
20293083	2011	5	26	3440	44.91	25.85
20293083	2011	5	26	3450	42.86	25.92
20293083	2011	5	26	3460	41.75	25.96
20293083	2011	5	26	3470	41.21	26.02
20293083	2011	5	26	3480	39.81	26.11
20293083	2011	5	26	3490	39.27	26.18
20293083	2011	5	26	3500	38.34	26.29
20293083	2011	5	26	3510	37.29	26.39
20293083	2011	5	26	3520	35.61	26.48
20293083	2011	5	26	3530	33.5	26.57
20293083	2011	5	26	3540	31.49	26.68
20293083	2011	5	26	3550	29.5	26.78
20293083	2011	5	26	3560	27.59	26.88
20293083	2011	5	26	3570	25.82	26.96
20293083	2011	5	26	3580	24.22	27.07
20293083	2011	5	26	3590	22.36	27.16
20293083	2011	5	26	3600	20.85	27.25
20293083	2011	5	26	3610	20.56	27.31
20293083	2011	5	26	3620	20.67	27.35
20293083	2011	5	26	3630	20.55	27.42
20293083	2011	5	26	3640	20.22	27.47

20293083	2011	5	26	3650	20.45	27.52
20293083	2011	5	26	3660	20.76	27.58
20293083	2011	5	26	3670	20.02	27.63
20293083	2011	5	26	3680	19.06	27.68
20293083	2011	5	26	3690	17.71	27.72
20293083	2011	5	26	3700	17.22	27.78
20293083	2011	5	26	3710	17.45	27.82
20293083	2011	5	26	3720	17.39	27.85
20293083	2011	5	26	3730	17.17	27.88
20293083	2011	5	26	3740	16.89	27.91
20293083	2011	5	26	3750	17.15	27.94
20293083	2011	5	26	3760	16.95	27.96
20293083	2011	5	26	3770	17.25	27.98
20293083	2011	5	26	3780	17.61	28
20293083	2011	5	26	3790	17.69	28.03
20293083	2011	5	26	3800	17.8	28.06
20293083	2011	5	26	3810	17.38	28.07
20293083	2011	5	26	3820	16.88	28.1
20293083	2011	5	26	3830	16.66	28.13
20293083	2011	5	26	3840	16.49	28.14
20293083	2011	5	26	3850	16.25	28.18
20293083	2011	5	26	3860	15.97	28.2
20293083	2011	5	26	3870	15.5	28.24
20293083	2011	5	26	3880	15.32	28.25
20293083	2011	5	26	3890	15.3	28.27
20293083	2011	5	26	3900	15.57	28.28
20293083	2011	5	26	3910	14.99	28.31
20293083	2011	5	26	3920	14.28	28.32
20293083	2011	5	26	3930	14.14	28.34
20293083	2011	5	26	3940	14.45	28.35
20293083	2011	5	26	3950	15.07	28.35
20293083	2011	5	26	3960	15.66	28.36
20293083	2011	5	26	3970	16.29	28.37
20293083	2011	5	26	3980	17.08	28.38
20293083	2011	5	26	3990	17.47	28.39
20293083	2011	5	26	4000	17.33	28.41
20293083	2011	5	26	4010	17.07	28.42
20293083	2011	5	26	4020	16.52	28.43
20293083	2011	5	26	4030	16.34	28.45
20293083	2011	5	26	4040	15.79	28.46
20293083	2011	5	26	4050	15.07	28.48
20293083	2011	5	26	4060	14.01	28.5
20293083	2011	5	26	4070	12.22	28.52
20293083	2011	5	26	4080	10.39	28.54
20293083	2011	5	26	4090	8.52	28.57
20293083	2011	5	26	4100	6.85	28.6
20293083	2011	5	26	4110	5.32	28.61

20293083	2011	5	26	4120	3.64	28.64
20293083	2011	5	26	4130	3.07	28.65
20293083	2011	5	26	4140	2.77	28.67
20293083	2011	5	26	4150	2.5	28.68
20293083	2011	5	26	4160	2.82	28.68
20293083	2011	5	26	4170	2.72	28.69
20293083	2011	5	26	4180	0.6	28.74
20293083	2011	5	26	4190	0.58	28.75
20293083	2011	5	26	4200	0.6	28.75
20293083	2011	5	26	4210	0.58	28.77
20293083	2011	5	26	4220	0.59	28.79
20293083	2011	5	26	4230	0.64	28.83
20293083	2011	5	26	4240	0.62	28.85
20293083	2011	5	26	4250	0.58	28.88
20293083	2011	5	26	4260	0.66	28.89
20293083	2011	5	26	4270	0.62	28.92
20293083	2011	5	26	4280	0.64	28.92
20293083	2011	5	26	4290	0.55	28.92
20293083	2011	5	26	4300	0.51	28.95
20293083	2011	5	26	4310	0.53	28.96
20293083	2011	5	26	4320	0.5	28.99
20301553	2011	5	26	0	1.47	29.36
20301553	2011	5	26	10	2.15	29.35
20301553	2011	5	26	20	2.59	29.35
20301553	2011	5	26	30	2.62	29.35
20301553	2011	5	26	40	2.39	29.35
20301553	2011	5	26	50	2.48	29.34
20301553	2011	5	26	60	2.67	29.32
20301553	2011	5	26	70	2.73	29.31
20301553	2011	5	26	80	2.98	29.28
20301553	2011	5	26	90	3.36	29.25
20301553	2011	5	26	100	5.14	29.17
20301553	2011	5	26	110	7.4	29.12
20301553	2011	5	26	120	9.14	29.1
20301553	2011	5	26	130	10.1	29.09
20301553	2011	5	26	140	10.49	29.07
20301553	2011	5	26	150	10.56	29.05
20301553	2011	5	26	160	10.87	29.04
20301553	2011	5	26	170	11.05	29.03
20301553	2011	5	26	180	11.13	29
20301553	2011	5	26	190	11.17	28.98
20301553	2011	5	26	200	11.26	28.96
20301553	2011	5	26	210	11.56	28.96
20301553	2011	5	26	220	11.67	28.94
20301553	2011	5	26	230	11.76	28.92
20301553	2011	5	26	240	12.11	28.92
20301553	2011	5	26	250	12.29	28.92

20301553	2011	5	26	260	12.17	28.91
20301553	2011	5	26	270	12.35	28.9
20301553	2011	5	26	280	12.73	28.89
20301553	2011	5	26	290	13.05	28.88
20301553	2011	5	26	300	13.29	28.86
20301553	2011	5	26	310	13.68	28.84
20301553	2011	5	26	320	14.18	28.83
20301553	2011	5	26	330	15.62	28.79
20301553	2011	5	26	340	17.71	28.75
20301553	2011	5	26	350	19.85	28.73
20301553	2011	5	26	360	22.08	28.71
20301553	2011	5	26	370	23.6	28.69
20301553	2011	5	26	380	25.32	28.67
20301553	2011	5	26	390	26.09	28.67
20301553	2011	5	26	400	27.4	28.62
20301553	2011	5	26	410	29.39	28.6
20301553	2011	5	26	420	31.69	28.57
20301553	2011	5	26	430	34.16	28.55
20301553	2011	5	26	440	36.58	28.53
20301553	2011	5	26	450	39.09	28.5
20301553	2011	5	26	460	41.6	28.46
20301553	2011	5	26	470	43.72	28.42
20301553	2011	5	26	480	46.29	28.29
20301553	2011	5	26	490	48.81	28.23
20301553	2011	5	26	500	50.01	28.18
20301553	2011	5	26	510	49.82	28.16
20301553	2011	5	26	520	50.32	28.1
20301553	2011	5	26	530	50.93	28.04
20301553	2011	5	26	540	51.35	28.01
20301553	2011	5	26	550	51.56	27.97
20301553	2011	5	26	560	51.75	27.93
20301553	2011	5	26	570	51.97	27.87
20301553	2011	5	26	580	52.2	27.82
20301553	2011	5	26	590	51.55	27.76
20301553	2011	5	26	600	51.04	27.73
20301553	2011	5	26	610	50.83	27.67
20301553	2011	5	26	620	50.87	27.64
20301553	2011	5	26	630	51.16	27.6
20301553	2011	5	26	640	51.47	27.56
20301553	2011	5	26	650	51.77	27.53
20301553	2011	5	26	660	52.06	27.5
20301553	2011	5	26	670	52.28	27.46
20301553	2011	5	26	680	52.08	27.42
20301553	2011	5	26	690	51.25	27.41
20301553	2011	5	26	700	50.48	27.4
20301553	2011	5	26	710	49.93	27.38
20301553	2011	5	26	720	49.3	27.36

20301553	2011	5	26	730	49.51	27.34
20301553	2011	5	26	740	49.44	27.31
20301553	2011	5	26	750	49.28	27.29
20301553	2011	5	26	760	49.1	27.28
20301553	2011	5	26	770	49.14	27.28
20301553	2011	5	26	780	49.35	27.28
20301553	2011	5	26	790	50.1	27.25
20301553	2011	5	26	800	50.86	27.24
20301553	2011	5	26	810	51.29	27.23
20301553	2011	5	26	820	51.72	27.21
20301553	2011	5	26	830	52.05	27.2
20301553	2011	5	26	840	52.39	27.19
20301553	2011	5	26	850	52.64	27.17
20301553	2011	5	26	860	52.95	27.16
20301553	2011	5	26	870	53.37	27.14
20301553	2011	5	26	880	53.52	27.13
20301553	2011	5	26	890	53.62	27.11
20301553	2011	5	26	900	53.83	27.1
20301553	2011	5	26	910	53.98	27.08
20301553	2011	5	26	920	54.15	27.07
20301553	2011	5	26	930	54.22	27.06
20301553	2011	5	26	940	54.17	27.04
20301553	2011	5	26	950	54.1	27.04
20301553	2011	5	26	960	54.11	27.03
20301553	2011	5	26	970	54.16	27.03
20301553	2011	5	26	980	54.49	27.01
20301553	2011	5	26	990	54.28	27
20301553	2011	5	26	1000	54	27
20301553	2011	5	26	1010	53.89	26.99
20301553	2011	5	26	1020	53.93	26.98
20301553	2011	5	26	1030	53.84	26.97
20301553	2011	5	26	1040	53.95	26.96
20301553	2011	5	26	1050	53.99	26.96
20301553	2011	5	26	1060	53.66	26.96
20301553	2011	5	26	1070	51.95	26.95
20301553	2011	5	26	1080	49.76	26.96
20301553	2011	5	26	1090	48.29	26.96
20301553	2011	5	26	1100	47.64	26.96
20301553	2011	5	26	1110	46.14	26.98
20301553	2011	5	26	1120	45.74	26.98
20301553	2011	5	26	1130	44.91	27
20301553	2011	5	26	1140	44.4	27.02
20301553	2011	5	26	1150	44.31	27.03
20301553	2011	5	26	1160	43.84	27.05
20301553	2011	5	26	1170	43.62	27.07
20301553	2011	5	26	1180	43.28	27.09
20301553	2011	5	26	1190	43.32	27.1

20301553	2011	5	26	1200	43.49	27.13
20301553	2011	5	26	1210	43.54	27.15
20301553	2011	5	26	1220	43.45	27.17
20301553	2011	5	26	1230	43.36	27.18
20301553	2011	5	26	1240	43.54	27.21
20301553	2011	5	26	1250	43.37	27.21
20301553	2011	5	26	1260	43.61	27.23
20301553	2011	5	26	1270	43.93	27.25
20301553	2011	5	26	1280	44.31	27.25
20301553	2011	5	26	1290	45.1	27.26
20301553	2011	5	26	1300	46.24	27.28
20301553	2011	5	26	1310	46.52	27.28
20301553	2011	5	26	1320	46.52	27.29
20301553	2011	5	26	1330	45.95	27.31
20301553	2011	5	26	1340	45.69	27.31
20301553	2011	5	26	1350	45.91	27.31
20301553	2011	5	26	1360	46.08	27.31
20301553	2011	5	26	1370	46.03	27.32
20301553	2011	5	26	1380	46.21	27.32
20301553	2011	5	26	1390	46.45	27.32
20301553	2011	5	26	1400	46.03	27.33
20301553	2011	5	26	1410	45.51	27.33
20301553	2011	5	26	1420	46.05	27.34
20301553	2011	5	26	1430	46.57	27.33
20301553	2011	5	26	1440	46.66	27.34
20301553	2011	5	26	1450	46.92	27.34
20301553	2011	5	26	1460	46.95	27.34
20301553	2011	5	26	1470	46.71	27.34
20301553	2011	5	26	1480	46.58	27.35
20301553	2011	5	26	1490	46.78	27.35
20301553	2011	5	26	1500	46.89	27.34
20301553	2011	5	26	1510	46.82	27.34
20301553	2011	5	26	1520	46.49	27.34
20301553	2011	5	26	1530	46.46	27.34
20301553	2011	5	26	1540	46.62	27.33
20301553	2011	5	26	1550	46.95	27.32
20301553	2011	5	26	1560	47.35	27.32
20301553	2011	5	26	1570	47.81	27.32
20301553	2011	5	26	1580	48.39	27.31
20301553	2011	5	26	1590	48.89	27.3
20301553	2011	5	26	1600	49.58	27.28
20301553	2011	5	26	1610	50.92	27.25
20301553	2011	5	26	1620	51.76	27.24
20301553	2011	5	26	1630	51.8	27.24
20301553	2011	5	26	1640	51.85	27.23
20301553	2011	5	26	1650	51.99	27.22
20301553	2011	5	26	1660	52.11	27.21

20301553	2011	5	26	1670	52.16	27.21
20301553	2011	5	26	1680	52.28	27.2
20301553	2011	5	26	1690	52.36	27.2
20301553	2011	5	26	1700	52.16	27.18
20301553	2011	5	26	1710	52	27.17
20301553	2011	5	26	1720	51.88	27.17
20301553	2011	5	26	1730	51.86	27.16
20301553	2011	5	26	1740	51.77	27.15
20301553	2011	5	26	1750	51.77	27.15
20301553	2011	5	26	1760	51.75	27.14
20301553	2011	5	26	1770	51.82	27.13
20301553	2011	5	26	1780	52.77	27.1
20301553	2011	5	26	1790	54.78	27.04
20301553	2011	5	26	1800	56.92	27.02
20301553	2011	5	26	1810	59.73	26.98
20301553	2011	5	26	1820	63.03	26.95
20301553	2011	5	26	1830	66.12	26.9
20301553	2011	5	26	1840	68.1	26.82
20301553	2011	5	26	1850	70.53	26.7
20301553	2011	5	26	1860	73.74	26.64
20301553	2011	5	26	1870	77.18	26.57
20301553	2011	5	26	1880	79.51	26.5
20301553	2011	5	26	1890	81.47	26.46
20301553	2011	5	26	1900	83.89	26.35
20301553	2011	5	26	1910	85.69	26.29
20301553	2011	5	26	1920	86.07	26.27
20301553	2011	5	26	1930	86.42	26.25
20301553	2011	5	26	1940	86.68	26.21
20301553	2011	5	26	1950	86.76	26.16
20301553	2011	5	26	1960	86.26	26.11
20301553	2011	5	26	1970	86.1	26.08
20301553	2011	5	26	1980	86.05	26.05
20301553	2011	5	26	1990	85.98	26.01
20301553	2011	5	26	2000	85.99	25.99
20301553	2011	5	26	2010	86.01	25.96
20301553	2011	5	26	2020	85.79	25.92
20301553	2011	5	26	2030	85.72	25.89
20301553	2011	5	26	2040	85.66	25.86
20301553	2011	5	26	2050	85.53	25.83
20301553	2011	5	26	2060	85.84	25.79
20301553	2011	5	26	2070	86.72	25.73
20301553	2011	5	26	2080	87.16	25.71
20301553	2011	5	26	2090	87.38	25.69
20301553	2011	5	26	2100	87.51	25.68
20301553	2011	5	26	2110	88.86	25.6
20301553	2011	5	26	2120	90.89	25.46
20301553	2011	5	26	2130	93.02	25.34

20301553	2011	5	26	2140	95.6	25.35
20301553	2011	5	26	2150	96.8	25.33
20301553	2011	5	26	2160	97.04	25.3
20301553	2011	5	26	2170	98.19	25.24
20301553	2011	5	26	2180	100.72	25.06
20301553	2011	5	26	2190	103.27	24.9
20301553	2011	5	26	2200	105.74	24.68
20301553	2011	5	26	2210	107.9	24.43
20301553	2011	5	26	2220	109.74	24.21
20301553	2011	5	26	2230	111.78	24.02
20301553	2011	5	26	2240	113.61	23.81
20301553	2011	5	26	2250	115.88	23.67
20301553	2011	5	26	2260	118.68	23.72
20301553	2011	5	26	2270	121.89	23.64
20301553	2011	5	26	2280	125.25	23.56
20301553	2011	5	26	2290	128.56	23.52
20301553	2011	5	26	2300	131.81	23.36
20301553	2011	5	26	2310	134.87	23.21
20301553	2011	5	26	2320	138.08	23.09
20301553	2011	5	26	2330	141.25	22.98
20301553	2011	5	26	2340	144.54	22.84
20301553	2011	5	26	2350	147.82	22.65
20301553	2011	5	26	2360	150.83	22.5
20301553	2011	5	26	2370	152.44	22.35
20301553	2011	5	26	2380	153.22	22.27
20301553	2011	5	26	2390	153.55	22.17
20301553	2011	5	26	2400	153.62	22.09
20301553	2011	5	26	2410	153.48	22
20301553	2011	5	26	2420	153.14	21.89
20301553	2011	5	26	2430	152.59	21.8
20301553	2011	5	26	2440	151.89	21.71
20301553	2011	5	26	2450	151.01	21.6
20301553	2011	5	26	2460	150.06	21.5
20301553	2011	5	26	2470	149.41	21.39
20301553	2011	5	26	2480	149.06	21.26
20301553	2011	5	26	2490	148.8	21.15
20301553	2011	5	26	2500	148.66	21.04
20301553	2011	5	26	2510	148.57	20.93
20301553	2011	5	26	2520	148.48	20.8
20301553	2011	5	26	2530	148.29	20.56
20301553	2011	5	26	2540	148.2	20.49
20301553	2011	5	26	2550	148.15	20.42
20301553	2011	5	26	2560	147.92	20.34
20301553	2011	5	26	2570	147.57	20.28
20301553	2011	5	26	2580	147.12	20.23
20301553	2011	5	26	2590	146.52	20.16
20301553	2011	5	26	2600	146.04	20.09

20301553	2011	5	26	2610	145.98	20
20301553	2011	5	26	2620	145.92	19.92
20301553	2011	5	26	2630	145.83	19.9
20301553	2011	5	26	2640	145.78	19.85
20301553	2011	5	26	2650	145.68	19.83
20301553	2011	5	26	2660	145.35	19.78
20301553	2011	5	26	2670	145	19.75
20301553	2011	5	26	2680	144.74	19.72
20301553	2011	5	26	2690	144.54	19.7
20301553	2011	5	26	2700	144.33	19.68
20301553	2011	5	26	2710	144.02	19.68
20301553	2011	5	26	2720	143.72	19.67
20301553	2011	5	26	2730	142.77	19.67
20301553	2011	5	26	2740	142.2	19.67
20301553	2011	5	26	2750	142.42	19.67
20301553	2011	5	26	2760	142.9	19.67
20301553	2011	5	26	2770	142.97	19.68
20301553	2011	5	26	2780	142.83	19.68
20301553	2011	5	26	2790	142.56	19.69
20301553	2011	5	26	2800	142.19	19.69
20301553	2011	5	26	2810	141.88	19.68
20301553	2011	5	26	2820	141.68	19.69
20301553	2011	5	26	2830	141.51	19.7
20301553	2011	5	26	2840	141.25	19.71
20301553	2011	5	26	2850	140.94	19.71
20301553	2011	5	26	2860	140.71	19.72
20301553	2011	5	26	2870	140.64	19.74
20301553	2011	5	26	2880	140.63	19.74
20301553	2011	5	26	2890	140.5	19.76
20301553	2011	5	26	2900	139.49	19.76
20301553	2011	5	26	2910	138.52	19.78
20301553	2011	5	26	2920	137.02	19.79
20301553	2011	5	26	2930	135.47	19.82
20301553	2011	5	26	2940	134.33	19.83
20301553	2011	5	26	2950	132.22	19.87
20301553	2011	5	26	2960	130.45	19.91
20301553	2011	5	26	2970	128.56	19.94
20301553	2011	5	26	2980	127.47	19.98
20301553	2011	5	26	2990	126.56	20.03
20301553	2011	5	26	3000	125.16	20.07
20301553	2011	5	26	3010	124.49	20.12
20301553	2011	5	26	3020	124.03	20.18
20301553	2011	5	26	3030	123.73	20.23
20301553	2011	5	26	3040	123.28	20.29
20301553	2011	5	26	3050	122.93	20.35
20301553	2011	5	26	3060	122.8	20.46
20301553	2011	5	26	3070	122.68	20.53

20301553	2011	5	26	3080	122.56	20.55
20301553	2011	5	26	3090	122.37	20.59
20301553	2011	5	26	3100	120.76	20.67
20301553	2011	5	26	3110	119.62	20.71
20301553	2011	5	26	3120	119.17	20.78
20301553	2011	5	26	3130	118.2	20.81
20301553	2011	5	26	3140	116.15	20.85
20301553	2011	5	26	3150	113.88	20.89
20301553	2011	5	26	3160	111.44	20.96
20301553	2011	5	26	3170	109.05	21.02
20301553	2011	5	26	3180	106.57	21.1
20301553	2011	5	26	3190	104.08	21.17
20301553	2011	5	26	3200	101.4	21.21
20301553	2011	5	26	3210	98.6	21.29
20301553	2011	5	26	3220	96.08	21.4
20301553	2011	5	26	3230	93.83	21.58
20301553	2011	5	26	3240	91.69	21.7
20301553	2011	5	26	3250	89.6	21.85
20301553	2011	5	26	3260	87.55	21.96
20301553	2011	5	26	3270	85.59	22.12
20301553	2011	5	26	3280	83.65	22.31
20301553	2011	5	26	3290	81.58	22.44
20301553	2011	5	26	3300	79.57	22.59
20301553	2011	5	26	3310	77.46	22.7
20301553	2011	5	26	3320	76.7	22.79
20301553	2011	5	26	3330	76.36	22.88
20301553	2011	5	26	3340	76.38	22.99
20301553	2011	5	26	3350	76.62	23.09
20301553	2011	5	26	3360	76.9	23.17
20301553	2011	5	26	3370	77.63	23.36
20301553	2011	5	26	3380	76.23	23.5
20301553	2011	5	26	3390	74.25	23.62
20301553	2011	5	26	3400	73.36	23.69
20301553	2011	5	26	3410	71.83	23.76
20301553	2011	5	26	3420	69.64	23.83
20301553	2011	5	26	3430	67.68	23.93
20301553	2011	5	26	3440	65.62	24.04
20301553	2011	5	26	3450	63.2	24.13
20301553	2011	5	26	3460	60.76	24.25
20301553	2011	5	26	3470	58.17	24.32
20301553	2011	5	26	3480	55.75	24.42
20301553	2011	5	26	3490	53.45	24.55
20301553	2011	5	26	3500	51.08	24.66
20301553	2011	5	26	3510	48.64	24.76
20301553	2011	5	26	3520	46.28	24.9
20301553	2011	5	26	3530	44.15	25.04
20301553	2011	5	26	3540	43.35	25.16

20301553	2011	5	26	3550	43.43	25.37
20301553	2011	5	26	3560	43.14	25.44
20301553	2011	5	26	3570	43.16	25.55
20301553	2011	5	26	3580	42.93	25.71
20301553	2011	5	26	3590	42.82	25.76
20301553	2011	5	26	3600	42.78	25.8
20301553	2011	5	26	3610	42.5	25.89
20301553	2011	5	26	3620	40.91	25.98
20301553	2011	5	26	3630	39.28	26.07
20301553	2011	5	26	3640	37.94	26.13
20301553	2011	5	26	3650	37.41	26.3
20301553	2011	5	26	3660	36.81	26.43
20301553	2011	5	26	3670	36.04	26.54
20301553	2011	5	26	3680	35.46	26.6
20301553	2011	5	26	3690	35.05	26.67
20301553	2011	5	26	3700	34.13	26.76
20301553	2011	5	26	3710	33.55	26.88
20301553	2011	5	26	3720	33.99	26.97
20301553	2011	5	26	3730	33.63	27
20301553	2011	5	26	3740	33.43	27.12
20301553	2011	5	26	3750	32.74	27.14
20301553	2011	5	26	3760	32.47	27.17
20301553	2011	5	26	3770	31.58	27.25
20301553	2011	5	26	3780	31.39	27.3
20301553	2011	5	26	3790	31.52	27.35
20301553	2011	5	26	3800	31.91	27.39
20301553	2011	5	26	3810	31.96	27.45
20301553	2011	5	26	3820	31.79	27.54
20301553	2011	5	26	3830	31.25	27.6
20301553	2011	5	26	3840	31.35	27.64
20301553	2011	5	26	3850	30.86	27.72
20301553	2011	5	26	3860	30.48	27.74
20301553	2011	5	26	3870	30.49	27.75
20301553	2011	5	26	3880	30.67	27.79
20301553	2011	5	26	3890	30.47	27.82
20301553	2011	5	26	3900	29.96	27.87
20301553	2011	5	26	3910	29.36	27.93
20301553	2011	5	26	3920	28.48	27.95
20301553	2011	5	26	3930	27.96	27.97
20301553	2011	5	26	3940	26.91	28
20301553	2011	5	26	3950	25.59	28.02
20301553	2011	5	26	3960	25.08	28.04
20301553	2011	5	26	3970	24.79	28.07
20301553	2011	5	26	3980	23.94	28.1
20301553	2011	5	26	3990	22.69	28.13
20301553	2011	5	26	4000	21.54	28.14
20301553	2011	5	26	4010	21.19	28.17

20301553	2011	5	26	4020	20.86	28.2
20301553	2011	5	26	4030	20.6	28.22
20301553	2011	5	26	4040	20.51	28.24
20301553	2011	5	26	4050	20.09	28.25
20301553	2011	5	26	4060	19.6	28.28
20301553	2011	5	26	4070	19.69	28.29
20301553	2011	5	26	4080	20.14	28.3
20301553	2011	5	26	4090	20.46	28.31
20301553	2011	5	26	4100	20.31	28.32
20301553	2011	5	26	4110	20	28.33
20301553	2011	5	26	4120	19.32	28.35
20301553	2011	5	26	4130	18.41	28.36
20301553	2011	5	26	4140	18.22	28.38
20301553	2011	5	26	4150	17.65	28.39
20301553	2011	5	26	4160	17.09	28.41
20301553	2011	5	26	4170	17.16	28.42
20301553	2011	5	26	4180	17.36	28.42
20301553	2011	5	26	4190	17.31	28.43
20301553	2011	5	26	4200	16.83	28.45
20301553	2011	5	26	4210	16.3	28.46
20301553	2011	5	26	4220	15.28	28.46
20301553	2011	5	26	4230	13.44	28.49
20301553	2011	5	26	4240	11.14	28.51
20301553	2011	5	26	4250	8.83	28.54
20301553	2011	5	26	4260	6.61	28.57
20301553	2011	5	26	4270	4.87	28.59
20301553	2011	5	26	4280	3.54	28.6
20301553	2011	5	26	4290	3.18	28.62
20301553	2011	5	26	4300	2.73	28.63
20301553	2011	5	26	4310	2.37	28.64
20301553	2011	5	26	4320	2.68	28.64
20301553	2011	5	26	4330	2.58	28.67
20301553	2011	5	26	4340	2.39	28.69
20301553	2011	5	26	4350	2.11	28.7
20301553	2011	5	26	4360	0.59	28.78
20301553	2011	5	26	4370	0.6	28.78
20301553	2011	5	26	4380	0.6	28.8
20301553	2011	5	26	4390	0.59	28.82
20301553	2011	5	26	4400	0.59	28.85
20301553	2011	5	26	4410	0.58	28.89
20301553	2011	5	26	4420	0.59	28.9
20301553	2011	5	26	4430	0.58	28.92
20301553	2011	5	26	4440	0.58	28.93
20301553	2011	5	26	4450	0.58	28.96
20301553	2011	5	26	4460	0.56	28.99
20301553	2011	5	26	4470	0.57	29.01
20301553	2011	5	26	4480	0.47	29.03

20301553	2011	5	26	4490	0.47	29.06
20301553	2011	5	26	4500	0.48	29.08
20376853	2011	5	27	0	1.24	28.91
20376853	2011	5	27	10	1.54	28.91
20376853	2011	5	27	20	2.18	28.9
20376853	2011	5	27	30	2.89	28.9
20376853	2011	5	27	40	2.75	28.9
20376853	2011	5	27	50	2.75	28.91
20376853	2011	5	27	60	2.77	28.9
20376853	2011	5	27	70	2.55	28.91
20376853	2011	5	27	80	2.76	28.92
20376853	2011	5	27	90	3.28	28.9
20376853	2011	5	27	100	4.25	28.89
20376853	2011	5	27	110	6.08	28.87
20376853	2011	5	27	120	7.83	28.85
20376853	2011	5	27	130	10.15	28.82
20376853	2011	5	27	140	11.83	28.79
20376853	2011	5	27	150	12.84	28.78
20376853	2011	5	27	160	13.21	28.77
20376853	2011	5	27	170	13.34	28.75
20376853	2011	5	27	180	13.16	28.75
20376853	2011	5	27	190	13.47	28.74
20376853	2011	5	27	200	13.75	28.73
20376853	2011	5	27	210	12.98	28.73
20376853	2011	5	27	220	12.11	28.74
20376853	2011	5	27	230	12.38	28.73
20376853	2011	5	27	240	12.67	28.71
20376853	2011	5	27	250	12.64	28.71
20376853	2011	5	27	260	12.57	28.71
20376853	2011	5	27	270	12.42	28.71
20376853	2011	5	27	280	12.19	28.71
20376853	2011	5	27	290	11.91	28.71
20376853	2011	5	27	300	12.01	28.7
20376853	2011	5	27	310	12.44	28.69
20376853	2011	5	27	320	12.43	28.69
20376853	2011	5	27	330	12.53	28.68
20376853	2011	5	27	340	12.79	28.68
20376853	2011	5	27	350	13.22	28.67
20376853	2011	5	27	360	13.64	28.67
20376853	2011	5	27	370	14.19	28.66
20376853	2011	5	27	380	14.74	28.65
20376853	2011	5	27	390	15.2	28.64
20376853	2011	5	27	400	15.7	28.64
20376853	2011	5	27	410	16.71	28.63
20376853	2011	5	27	420	17.7	28.61
20376853	2011	5	27	430	18.36	28.61
20376853	2011	5	27	440	19.16	28.6

20376853	2011	5	27	450	20.46	28.59
20376853	2011	5	27	460	22.11	28.57
20376853	2011	5	27	470	23.43	28.55
20376853	2011	5	27	480	24.77	28.53
20376853	2011	5	27	490	25.77	28.54
20376853	2011	5	27	500	26.62	28.53
20376853	2011	5	27	510	27.98	28.52
20376853	2011	5	27	520	29.33	28.5
20376853	2011	5	27	530	31.66	28.48
20376853	2011	5	27	540	34.02	28.46
20376853	2011	5	27	550	35.22	28.44
20376853	2011	5	27	560	37.34	28.42
20376853	2011	5	27	570	39.71	28.41
20376853	2011	5	27	580	40.48	28.39
20376853	2011	5	27	590	40.98	28.39
20376853	2011	5	27	600	41.59	28.38
20376853	2011	5	27	610	42.11	28.35
20376853	2011	5	27	620	42.42	28.28
20376853	2011	5	27	630	42.73	28.25
20376853	2011	5	27	640	43.54	28.23
20376853	2011	5	27	650	44.75	28.17
20376853	2011	5	27	660	45.6	28.07
20376853	2011	5	27	670	47.16	27.98
20376853	2011	5	27	680	49.4	27.85
20376853	2011	5	27	690	50.71	27.82
20376853	2011	5	27	700	51.41	27.78
20376853	2011	5	27	710	52.02	27.71
20376853	2011	5	27	720	52.05	27.61
20376853	2011	5	27	730	51.74	27.57
20376853	2011	5	27	740	51.98	27.51
20376853	2011	5	27	750	52.18	27.46
20376853	2011	5	27	760	52.84	27.42
20376853	2011	5	27	770	52.27	27.39
20376853	2011	5	27	780	51.08	27.36
20376853	2011	5	27	790	49.9	27.32
20376853	2011	5	27	800	50.03	27.29
20376853	2011	5	27	810	49.91	27.26
20376853	2011	5	27	820	49.99	27.22
20376853	2011	5	27	830	50.03	27.18
20376853	2011	5	27	840	50.04	27.17
20376853	2011	5	27	850	49.93	27.13
20376853	2011	5	27	860	49.9	27.1
20376853	2011	5	27	870	49.91	27.1
20376853	2011	5	27	880	49.9	27.08
20376853	2011	5	27	890	49.93	27.05
20376853	2011	5	27	900	49.89	27.05
20376853	2011	5	27	910	49.93	27.06

20376853	2011	5	27	920	49.86	27.03
20376853	2011	5	27	930	49.85	27.01
20376853	2011	5	27	940	49.95	27
20376853	2011	5	27	950	49.9	26.97
20376853	2011	5	27	960	49.92	26.96
20376853	2011	5	27	970	49.89	26.95
20376853	2011	5	27	980	49.84	26.94
20376853	2011	5	27	990	49.94	26.94
20376853	2011	5	27	1000	49.97	26.92
20376853	2011	5	27	1010	49.97	26.92
20376853	2011	5	27	1020	49.94	26.92
20376853	2011	5	27	1030	49.05	26.92
20376853	2011	5	27	1040	48.75	26.92
20376853	2011	5	27	1050	49.23	26.91
20376853	2011	5	27	1060	49.37	26.9
20376853	2011	5	27	1070	49.18	26.9
20376853	2011	5	27	1080	49.35	26.89
20376853	2011	5	27	1090	49.42	26.89
20376853	2011	5	27	1100	49.34	26.88
20376853	2011	5	27	1110	49.18	26.88
20376853	2011	5	27	1120	49.19	26.88
20376853	2011	5	27	1130	49.34	26.87
20376853	2011	5	27	1140	49.21	26.86
20376853	2011	5	27	1150	49.19	26.85
20376853	2011	5	27	1160	49.15	26.86
20376853	2011	5	27	1170	49.09	26.85
20376853	2011	5	27	1180	49.15	26.84
20376853	2011	5	27	1190	49.08	26.84
20376853	2011	5	27	1200	49.14	26.85
20376853	2011	5	27	1210	49.97	26.82
20376853	2011	5	27	1220	51.91	26.8
20376853	2011	5	27	1230	52.62	26.8
20376853	2011	5	27	1240	52.78	26.8
20376853	2011	5	27	1250	52.86	26.78
20376853	2011	5	27	1260	53.02	26.78
20376853	2011	5	27	1270	53.77	26.78
20376853	2011	5	27	1280	53.96	26.76
20376853	2011	5	27	1290	53.67	26.75
20376853	2011	5	27	1300	53.7	26.76
20376853	2011	5	27	1310	53.66	26.75
20376853	2011	5	27	1320	53.42	26.75
20376853	2011	5	27	1330	53.52	26.75
20376853	2011	5	27	1340	53.41	26.74
20376853	2011	5	27	1350	53.34	26.74
20376853	2011	5	27	1360	53.44	26.73
20376853	2011	5	27	1370	53.46	26.72
20376853	2011	5	27	1380	53.41	26.72

20376853	2011	5	27	1390	53.36	26.72
20376853	2011	5	27	1400	53.48	26.72
20376853	2011	5	27	1410	53.45	26.71
20376853	2011	5	27	1420	53.51	26.71
20376853	2011	5	27	1430	53.18	26.71
20376853	2011	5	27	1440	51.96	26.71
20376853	2011	5	27	1450	50.19	26.72
20376853	2011	5	27	1460	48.54	26.73
20376853	2011	5	27	1470	46.97	26.75
20376853	2011	5	27	1480	45.98	26.75
20376853	2011	5	27	1490	44.78	26.76
20376853	2011	5	27	1500	43.81	26.78
20376853	2011	5	27	1510	43.46	26.77
20376853	2011	5	27	1520	43.08	26.78
20376853	2011	5	27	1530	42.61	26.79
20376853	2011	5	27	1540	42.57	26.78
20376853	2011	5	27	1550	42.19	26.8
20376853	2011	5	27	1560	42.18	26.82
20376853	2011	5	27	1570	42.75	26.84
20376853	2011	5	27	1580	42.77	26.85
20376853	2011	5	27	1590	42.5	26.87
20376853	2011	5	27	1600	42.6	26.89
20376853	2011	5	27	1610	42.86	26.92
20376853	2011	5	27	1620	43.1	26.92
20376853	2011	5	27	1630	43.3	26.92
20376853	2011	5	27	1640	43.31	26.93
20376853	2011	5	27	1650	43.32	26.94
20376853	2011	5	27	1660	42.95	26.96
20376853	2011	5	27	1670	43.03	26.95
20376853	2011	5	27	1680	43.14	26.96
20376853	2011	5	27	1690	43.67	26.97
20376853	2011	5	27	1700	43.96	26.99
20376853	2011	5	27	1710	44.27	27
20376853	2011	5	27	1720	44.56	27
20376853	2011	5	27	1730	45.01	27
20376853	2011	5	27	1740	45.05	27
20376853	2011	5	27	1750	45.29	27
20376853	2011	5	27	1760	45.18	26.98
20376853	2011	5	27	1770	44.73	26.99
20376853	2011	5	27	1780	44.76	26.97
20376853	2011	5	27	1790	44.64	26.96
20376853	2011	5	27	1800	44.94	26.95
20376853	2011	5	27	1810	44.77	26.95
20376853	2011	5	27	1820	44.68	26.96
20376853	2011	5	27	1830	44.69	26.96
20376853	2011	5	27	1840	44.77	26.94
20376853	2011	5	27	1850	44.81	26.93

20376853	2011	5	27	1860	45.24	26.9
20376853	2011	5	27	1870	45.73	26.9
20376853	2011	5	27	1880	45.81	26.9
20376853	2011	5	27	1890	46.63	26.88
20376853	2011	5	27	1900	46.81	26.87
20376853	2011	5	27	1910	47.09	26.86
20376853	2011	5	27	1920	47.15	26.86
20376853	2011	5	27	1930	46.98	26.85
20376853	2011	5	27	1940	47.11	26.85
20376853	2011	5	27	1950	47.14	26.84
20376853	2011	5	27	1960	46.93	26.84
20376853	2011	5	27	1970	46.6	26.83
20376853	2011	5	27	1980	46.23	26.83
20376853	2011	5	27	1990	46.48	26.82
20376853	2011	5	27	2000	47.29	26.79
20376853	2011	5	27	2010	47.54	26.79
20376853	2011	5	27	2020	47.54	26.78
20376853	2011	5	27	2030	46.35	26.79
20376853	2011	5	27	2040	45.61	26.8
20376853	2011	5	27	2050	45.16	26.79
20376853	2011	5	27	2060	44.97	26.8
20376853	2011	5	27	2070	44.85	26.79
20376853	2011	5	27	2080	44.83	26.8
20376853	2011	5	27	2090	44.65	26.8
20376853	2011	5	27	2100	44.51	26.8
20376853	2011	5	27	2110	44.64	26.8
20376853	2011	5	27	2120	44.74	26.82
20376853	2011	5	27	2130	44.73	26.84
20376853	2011	5	27	2140	45.3	26.84
20376853	2011	5	27	2150	47.66	26.83
20376853	2011	5	27	2160	49.38	26.79
20376853	2011	5	27	2170	50.13	26.78
20376853	2011	5	27	2180	51.78	26.76
20376853	2011	5	27	2190	52.02	26.75
20376853	2011	5	27	2200	51.78	26.76
20376853	2011	5	27	2210	52.15	26.75
20376853	2011	5	27	2220	52.06	26.74
20376853	2011	5	27	2230	52.2	26.74
20376853	2011	5	27	2240	52.19	26.73
20376853	2011	5	27	2250	51.77	26.73
20376853	2011	5	27	2260	51.66	26.73
20376853	2011	5	27	2270	51.6	26.73
20376853	2011	5	27	2280	51.61	26.72
20376853	2011	5	27	2290	51.63	26.73
20376853	2011	5	27	2300	51.66	26.72
20376853	2011	5	27	2310	51.88	26.71
20376853	2011	5	27	2320	51.94	26.71

20376853	2011	5	27	2330	51.85	26.7
20376853	2011	5	27	2340	51.82	26.7
20376853	2011	5	27	2350	52.72	26.69
20376853	2011	5	27	2360	52.98	26.67
20376853	2011	5	27	2370	52.69	26.67
20376853	2011	5	27	2380	52.51	26.67
20376853	2011	5	27	2390	52.48	26.67
20376853	2011	5	27	2400	52.59	26.67
20376853	2011	5	27	2410	52.81	26.67
20376853	2011	5	27	2420	52.84	26.66
20376853	2011	5	27	2430	52.65	26.66
20376853	2011	5	27	2440	52.34	26.65
20376853	2011	5	27	2450	52.31	26.65
20376853	2011	5	27	2460	54.15	26.64
20376853	2011	5	27	2470	56.46	26.62
20376853	2011	5	27	2480	58.65	26.6
20376853	2011	5	27	2490	60.91	26.58
20376853	2011	5	27	2500	63.47	26.56
20376853	2011	5	27	2510	66.02	26.54
20376853	2011	5	27	2520	68.47	26.5
20376853	2011	5	27	2530	70.13	26.46
20376853	2011	5	27	2540	71.57	26.44
20376853	2011	5	27	2550	74.01	26.4
20376853	2011	5	27	2560	76.14	26.36
20376853	2011	5	27	2570	78.71	26.33
20376853	2011	5	27	2580	81.72	26.27
20376853	2011	5	27	2590	84.51	26.11
20376853	2011	5	27	2600	86.23	26.04
20376853	2011	5	27	2610	86.59	26.02
20376853	2011	5	27	2620	86.56	25.96
20376853	2011	5	27	2630	86.9	25.96
20376853	2011	5	27	2640	87.33	25.88
20376853	2011	5	27	2650	86.99	25.81
20376853	2011	5	27	2660	86.44	25.78
20376853	2011	5	27	2670	86.28	25.75
20376853	2011	5	27	2680	86.39	25.72
20376853	2011	5	27	2690	86.46	25.67
20376853	2011	5	27	2700	86.23	25.62
20376853	2011	5	27	2710	85.93	25.59
20376853	2011	5	27	2720	85.78	25.56
20376853	2011	5	27	2730	85.69	25.53
20376853	2011	5	27	2740	85.74	25.5
20376853	2011	5	27	2750	85.74	25.46
20376853	2011	5	27	2760	85.92	25.45
20376853	2011	5	27	2770	86.02	25.43
20376853	2011	5	27	2780	86.09	25.39
20376853	2011	5	27	2790	85.65	25.37

20376853	2011	5	27	2800	85.46	25.35
20376853	2011	5	27	2810	84.99	25.33
20376853	2011	5	27	2820	84.76	25.3
20376853	2011	5	27	2830	84.82	25.28
20376853	2011	5	27	2840	85.52	25.26
20376853	2011	5	27	2850	86.46	25.24
20376853	2011	5	27	2860	88.67	25.15
20376853	2011	5	27	2870	90.27	25.12
20376853	2011	5	27	2880	91.06	25.1
20376853	2011	5	27	2890	93.23	25.02
20376853	2011	5	27	2900	94.83	24.92
20376853	2011	5	27	2910	95.54	24.91
20376853	2011	5	27	2920	97.16	24.82
20376853	2011	5	27	2930	99.44	24.75
20376853	2011	5	27	2940	101.79	24.61
20376853	2011	5	27	2950	104.22	24.44
20376853	2011	5	27	2960	106.74	24.27
20376853	2011	5	27	2970	109.36	24.07
20376853	2011	5	27	2980	110.96	23.85
20376853	2011	5	27	2990	112.07	23.82
20376853	2011	5	27	3000	113.88	23.66
20376853	2011	5	27	3010	116.1	23.56
20376853	2011	5	27	3020	118.45	23.42
20376853	2011	5	27	3030	120.95	23.28
20376853	2011	5	27	3040	123.36	23.18
20376853	2011	5	27	3050	125.25	23.1
20376853	2011	5	27	3060	127.57	22.92
20376853	2011	5	27	3070	129.32	22.7
20376853	2011	5	27	3080	131.06	22.6
20376853	2011	5	27	3090	133.43	22.44
20376853	2011	5	27	3100	136.2	22.17
20376853	2011	5	27	3110	138.45	22.12
20376853	2011	5	27	3120	140.73	22.09
20376853	2011	5	27	3130	143.64	21.9
20376853	2011	5	27	3140	146.26	21.6
20376853	2011	5	27	3150	147.48	21.46
20376853	2011	5	27	3160	147.99	21.24
20376853	2011	5	27	3170	148.07	21.15
20376853	2011	5	27	3180	148.18	21
20376853	2011	5	27	3190	148.8	20.95
20376853	2011	5	27	3200	149.69	20.93
20376853	2011	5	27	3210	150.43	20.92
20376853	2011	5	27	3220	150.92	20.81
20376853	2011	5	27	3230	151.04	20.63
20376853	2011	5	27	3240	151.02	20.6
20376853	2011	5	27	3250	151.03	20.57
20376853	2011	5	27	3260	151.05	20.55

20376853	2011	5	27	3270	151.13	20.5
20376853	2011	5	27	3280	151.08	20.42
20376853	2011	5	27	3290	151.07	20.38
20376853	2011	5	27	3300	151	20.32
20376853	2011	5	27	3310	151.06	20.15
20376853	2011	5	27	3320	151.04	20.1
20376853	2011	5	27	3330	151.07	19.98
20376853	2011	5	27	3340	151	19.92
20376853	2011	5	27	3350	151.01	19.85
20376853	2011	5	27	3360	150.95	19.81
20376853	2011	5	27	3370	150.92	19.79
20376853	2011	5	27	3380	150.96	19.72
20376853	2011	5	27	3390	150.8	19.71
20376853	2011	5	27	3400	150.56	19.67
20376853	2011	5	27	3410	150.49	19.65
20376853	2011	5	27	3420	150.04	19.59
20376853	2011	5	27	3430	148.11	19.57
20376853	2011	5	27	3440	146.26	19.53
20376853	2011	5	27	3450	144.67	19.52
20376853	2011	5	27	3460	143.21	19.51
20376853	2011	5	27	3470	141.75	19.5
20376853	2011	5	27	3480	139.58	19.49
20376853	2011	5	27	3490	137.19	19.47
20376853	2011	5	27	3500	135.21	19.51
20376853	2011	5	27	3510	133.68	19.52
20376853	2011	5	27	3520	132.52	19.59
20376853	2011	5	27	3530	131.26	19.63
20376853	2011	5	27	3540	130.27	19.66
20376853	2011	5	27	3550	130.58	19.71
20376853	2011	5	27	3560	131.47	19.74
20376853	2011	5	27	3570	131.56	19.76
20376853	2011	5	27	3580	132.76	19.82
20376853	2011	5	27	3590	133.98	19.85
20376853	2011	5	27	3600	133.02	19.85
20376853	2011	5	27	3610	131.52	19.85
20376853	2011	5	27	3620	130.07	19.87
20376853	2011	5	27	3630	128.57	19.89
20376853	2011	5	27	3640	127.02	19.91
20376853	2011	5	27	3650	125.19	19.92
20376853	2011	5	27	3660	123.52	19.99
20376853	2011	5	27	3670	122.26	20.02
20376853	2011	5	27	3680	120.9	20.09
20376853	2011	5	27	3690	119.43	20.14
20376853	2011	5	27	3700	117.78	20.21
20376853	2011	5	27	3710	116.16	20.25
20376853	2011	5	27	3720	114.89	20.37
20376853	2011	5	27	3730	113.52	20.45

20376853	2011	5	27	3740	112.02	20.5
20376853	2011	5	27	3750	110.47	20.54
20376853	2011	5	27	3760	108.47	20.58
20376853	2011	5	27	3770	106.33	20.64
20376853	2011	5	27	3780	104.43	20.69
20376853	2011	5	27	3790	102.87	20.76
20376853	2011	5	27	3800	101.48	20.86
20376853	2011	5	27	3810	99.94	20.96
20376853	2011	5	27	3820	98.45	21.01
20376853	2011	5	27	3830	97.2	21.11
20376853	2011	5	27	3840	96.19	21.32
20376853	2011	5	27	3850	94.82	21.43
20376853	2011	5	27	3860	94.12	21.75
20376853	2011	5	27	3870	93.2	21.89
20376853	2011	5	27	3880	91.41	21.96
20376853	2011	5	27	3890	89.85	22.25
20376853	2011	5	27	3900	88.33	22.42
20376853	2011	5	27	3910	86.62	22.57
20376853	2011	5	27	3920	85.08	22.67
20376853	2011	5	27	3930	83.55	22.83
20376853	2011	5	27	3940	81.97	23.06
20376853	2011	5	27	3950	80.41	23.15
20376853	2011	5	27	3960	79.89	23.22
20376853	2011	5	27	3970	80.57	23.25
20376853	2011	5	27	3980	81.12	23.48
20376853	2011	5	27	3990	81.17	23.58
20376853	2011	5	27	4000	80.91	23.6
20376853	2011	5	27	4010	79.34	23.66
20376853	2011	5	27	4020	77.73	23.82
20376853	2011	5	27	4030	76.42	23.96
20376853	2011	5	27	4040	75.31	24.03
20376853	2011	5	27	4050	73.96	24.12
20376853	2011	5	27	4060	72.76	24.23
20376853	2011	5	27	4070	71.61	24.32
20376853	2011	5	27	4080	70.4	24.4
20376853	2011	5	27	4090	69.27	24.49
20376853	2011	5	27	4100	67.88	24.55
20376853	2011	5	27	4110	66.25	24.64
20376853	2011	5	27	4120	64.75	24.71
20376853	2011	5	27	4130	63.36	24.8
20376853	2011	5	27	4140	61.94	24.88
20376853	2011	5	27	4150	60.52	24.97
20376853	2011	5	27	4160	60	25.1
20376853	2011	5	27	4170	59.15	25.19
20376853	2011	5	27	4180	58.39	25.28
20376853	2011	5	27	4190	57.86	25.36
20376853	2011	5	27	4200	56.3	25.42

20376853	2011	5	27	4210	54.55	25.46
20376853	2011	5	27	4220	53.12	25.58
20376853	2011	5	27	4230	51.43	25.6
20376853	2011	5	27	4240	50.41	25.7
20376853	2011	5	27	4250	50.41	25.75
20376853	2011	5	27	4260	50.67	25.81
20376853	2011	5	27	4270	50.47	25.86
20376853	2011	5	27	4280	50.52	25.91
20376853	2011	5	27	4290	50.5	25.96
20376853	2011	5	27	4300	50.54	25.99
20376853	2011	5	27	4310	50.49	26.03
20376853	2011	5	27	4320	50.55	26.06
20376853	2011	5	27	4330	50.51	26.11
20376853	2011	5	27	4340	50.41	26.12
20376853	2011	5	27	4350	50.59	26.16
20376853	2011	5	27	4360	50.43	26.18
20376853	2011	5	27	4370	50.39	26.21
20376853	2011	5	27	4380	49.53	26.23
20376853	2011	5	27	4390	47.92	26.26
20376853	2011	5	27	4400	46.01	26.28
20376853	2011	5	27	4410	44.08	26.32
20376853	2011	5	27	4420	42.22	26.38
20376853	2011	5	27	4430	41.17	26.42
20376853	2011	5	27	4440	41.39	26.57
20376853	2011	5	27	4450	41.29	26.76
20376853	2011	5	27	4460	41.3	26.85
20376853	2011	5	27	4470	41.44	26.95
20376853	2011	5	27	4480	41.28	27.02
20376853	2011	5	27	4490	41.28	27.07
20376853	2011	5	27	4500	42.24	27.16
20376853	2011	5	27	4510	42.27	27.15
20376853	2011	5	27	4520	41.32	27.17
20376853	2011	5	27	4530	40.88	27.19
20376853	2011	5	27	4540	41.01	27.23
20376853	2011	5	27	4550	41.41	27.31
20376853	2011	5	27	4560	41.83	27.39
20376853	2011	5	27	4570	42.18	27.43
20376853	2011	5	27	4580	42.61	27.48
20376853	2011	5	27	4590	41.93	27.58
20376853	2011	5	27	4600	41.39	27.59
20376853	2011	5	27	4610	41.27	27.64
20376853	2011	5	27	4620	39.94	27.7
20376853	2011	5	27	4630	37.91	27.75
20376853	2011	5	27	4640	36.84	27.75
20376853	2011	5	27	4650	35.84	27.81
20376853	2011	5	27	4660	34.51	27.83
20376853	2011	5	27	4670	33.5	27.85

20376853	2011	5	27	4680	33.81	27.94
20376853	2011	5	27	4690	33.99	27.96
20376853	2011	5	27	4700	33.89	27.97
20376853	2011	5	27	4710	33.8	28.01
20376853	2011	5	27	4720	33.91	28.03
20376853	2011	5	27	4730	33.75	28.03
20376853	2011	5	27	4740	33.74	28.05
20376853	2011	5	27	4750	33.9	28.06
20376853	2011	5	27	4760	33.91	28.07
20376853	2011	5	27	4770	33.75	28.1
20376853	2011	5	27	4780	33.84	28.1
20376853	2011	5	27	4790	34.04	28.12
20376853	2011	5	27	4800	33.74	28.14
20376853	2011	5	27	4810	33.62	28.15
20376853	2011	5	27	4820	33.82	28.17
20376853	2011	5	27	4830	33.89	28.17
20376853	2011	5	27	4840	33.02	28.2
20376853	2011	5	27	4850	30.73	28.22
20376853	2011	5	27	4860	28.07	28.25
20376853	2011	5	27	4870	25.48	28.28
20376853	2011	5	27	4880	23.19	28.31
20376853	2011	5	27	4890	21.63	28.32
20376853	2011	5	27	4900	20.03	28.35
20376853	2011	5	27	4910	19.15	28.36
20376853	2011	5	27	4920	18.29	28.38
20376853	2011	5	27	4930	17.93	28.39
20376853	2011	5	27	4940	16.68	28.41
20376853	2011	5	27	4950	15.21	28.43
20376853	2011	5	27	4960	13.41	28.46
20376853	2011	5	27	4970	11.83	28.48
20376853	2011	5	27	4980	10.59	28.5
20376853	2011	5	27	4990	8.75	28.52
20376853	2011	5	27	5000	6.46	28.54
20376853	2011	5	27	5010	4.38	28.57
20376853	2011	5	27	5020	3.11	28.59
20376853	2011	5	27	5030	2.76	28.59
20376853	2011	5	27	5040	2.86	28.6
20376853	2011	5	27	5050	2.73	28.6
20376853	2011	5	27	5060	2.44	28.62
20376853	2011	5	27	5070	2.74	28.63
20376853	2011	5	27	5080	2.48	28.64
20376853	2011	5	27	5090	0.66	28.67
20376853	2011	5	27	5100	0.62	28.69
20376853	2011	5	27	5110	0.67	28.71
20376853	2011	5	27	5120	0.6	28.71
20376853	2011	5	27	5130	0.59	28.75
20376853	2011	5	27	5140	0.62	28.76

20376853	2011	5	27	5150	0.6	28.79
20376853	2011	5	27	5160	0.6	28.81
20376853	2011	5	27	5170	0.58	28.82
20376853	2011	5	27	5180	0.48	28.85
20376853	2011	5	27	5190	0.48	28.87
20376853	2011	5	27	5200	0.49	28.88
20376853	2011	5	27	5210	0.48	28.89
20376853	2011	5	27	5220	0.46	28.9
20376853	2011	5	27	5230	0.45	28.92
20386083	2011	5	27	0	1.47	29.32
20386083	2011	5	27	10	2.63	29.32
20386083	2011	5	27	20	2.71	29.31
20386083	2011	5	27	30	2.13	29.32
20386083	2011	5	27	40	1.26	29.32
20386083	2011	5	27	50	0.54	29.33
20386083	2011	5	27	60	0.56	29.34
20386083	2011	5	27	70	0.6	29.34
20386083	2011	5	27	80	0.58	29.34
20386083	2011	5	27	90	0.55	29.35
20386083	2011	5	27	100	0.58	29.35
20386083	2011	5	27	110	0.57	29.34
20386083	2011	5	27	120	0.6	29.33
20386083	2011	5	27	130	0.58	29.33
20386083	2011	5	27	140	0.6	29.33
20386083	2011	5	27	150	0.6	29.33
20386083	2011	5	27	160	0.65	29.32
20386083	2011	5	27	170	0.98	29.32
20386083	2011	5	27	180	1.47	29.32
20386083	2011	5	27	190	2.34	29.29
20386083	2011	5	27	200	3.07	29.28
20386083	2011	5	27	210	2.5	29.28
20386083	2011	5	27	220	2.48	29.28
20386083	2011	5	27	230	2.64	29.28
20386083	2011	5	27	240	2.43	29.26
20386083	2011	5	27	250	2.7	29.26
20386083	2011	5	27	260	3.11	29.25
20386083	2011	5	27	270	4.21	29.21
20386083	2011	5	27	280	5.21	29.18
20386083	2011	5	27	290	6.18	29.15
20386083	2011	5	27	300	7.2	29.13
20386083	2011	5	27	310	8.25	29.1
20386083	2011	5	27	320	9.61	29.05
20386083	2011	5	27	330	10.81	29.03
20386083	2011	5	27	340	11.87	29
20386083	2011	5	27	350	12.84	28.97
20386083	2011	5	27	360	13.66	28.96
20386083	2011	5	27	370	14.58	28.92

20386083	2011	5	27	380	15.3	28.91
20386083	2011	5	27	390	15.48	28.9
20386083	2011	5	27	400	15.84	28.88
20386083	2011	5	27	410	15.69	28.87
20386083	2011	5	27	420	15.13	28.86
20386083	2011	5	27	430	14.4	28.85
20386083	2011	5	27	440	13.92	28.85
20386083	2011	5	27	450	14.28	28.83
20386083	2011	5	27	460	14.92	28.81
20386083	2011	5	27	470	15.84	28.78
20386083	2011	5	27	480	16.4	28.77
20386083	2011	5	27	490	16.26	28.75
20386083	2011	5	27	500	15.94	28.75
20386083	2011	5	27	510	15.4	28.75
20386083	2011	5	27	520	15.44	28.75
20386083	2011	5	27	530	16.43	28.73
20386083	2011	5	27	540	16.8	28.71
20386083	2011	5	27	550	17.48	28.71
20386083	2011	5	27	560	18.23	28.68
20386083	2011	5	27	570	18.95	28.67
20386083	2011	5	27	580	19.05	28.67
20386083	2011	5	27	590	18.83	28.67
20386083	2011	5	27	600	19.17	28.66
20386083	2011	5	27	610	19.21	28.66
20386083	2011	5	27	620	19.03	28.65
20386083	2011	5	27	630	18.85	28.65
20386083	2011	5	27	640	18.9	28.64
20386083	2011	5	27	650	18.87	28.64
20386083	2011	5	27	660	18.78	28.64
20386083	2011	5	27	670	18.89	28.64
20386083	2011	5	27	680	19	28.64
20386083	2011	5	27	690	18.76	28.63
20386083	2011	5	27	700	18.44	28.62
20386083	2011	5	27	710	18.14	28.62
20386083	2011	5	27	720	17.82	28.62
20386083	2011	5	27	730	17.69	28.62
20386083	2011	5	27	740	17.48	28.62
20386083	2011	5	27	750	17.21	28.62
20386083	2011	5	27	760	17.28	28.62
20386083	2011	5	27	770	16.97	28.61
20386083	2011	5	27	780	17.09	28.61
20386083	2011	5	27	790	17.17	28.6
20386083	2011	5	27	800	16.97	28.6
20386083	2011	5	27	810	16.71	28.61
20386083	2011	5	27	820	16.32	28.6
20386083	2011	5	27	830	15.87	28.61
20386083	2011	5	27	840	16.12	28.6

20386083	2011	5	27	850	17.65	28.6
20386083	2011	5	27	860	20.26	28.57
20386083	2011	5	27	870	23.74	28.54
20386083	2011	5	27	880	27.7	28.51
20386083	2011	5	27	890	31.51	28.48
20386083	2011	5	27	900	34.03	28.45
20386083	2011	5	27	910	35.92	28.42
20386083	2011	5	27	920	37.8	28.4
20386083	2011	5	27	930	39.66	28.35
20386083	2011	5	27	940	41.17	28.3
20386083	2011	5	27	950	42.98	28.02
20386083	2011	5	27	960	44.73	27.93
20386083	2011	5	27	970	46.52	27.8
20386083	2011	5	27	980	48.39	27.69
20386083	2011	5	27	990	50.2	27.57
20386083	2011	5	27	1000	51.99	27.47
20386083	2011	5	27	1010	53.81	27.43
20386083	2011	5	27	1020	55.72	27.33
20386083	2011	5	27	1030	57.86	27.28
20386083	2011	5	27	1040	60.6	27.24
20386083	2011	5	27	1050	64.16	27.25
20386083	2011	5	27	1060	67.44	27.17
20386083	2011	5	27	1070	69.9	27.03
20386083	2011	5	27	1080	71.92	26.95
20386083	2011	5	27	1090	74.3	26.91
20386083	2011	5	27	1100	77.57	26.8
20386083	2011	5	27	1110	81.31	26.77
20386083	2011	5	27	1120	84.83	26.64
20386083	2011	5	27	1130	88.3	26.53
20386083	2011	5	27	1140	91.8	26.42
20386083	2011	5	27	1150	94.86	26.21
20386083	2011	5	27	1160	97.76	26.06
20386083	2011	5	27	1170	100.05	25.84
20386083	2011	5	27	1180	102.04	25.51
20386083	2011	5	27	1190	103.93	25.14
20386083	2011	5	27	1200	105.57	24.95
20386083	2011	5	27	1210	107.5	24.68
20386083	2011	5	27	1220	109.14	24.33
20386083	2011	5	27	1230	110.8	23.99
20386083	2011	5	27	1240	112.42	23.93
20386083	2011	5	27	1250	114.77	23.94
20386083	2011	5	27	1260	117.63	23.93
20386083	2011	5	27	1270	120.54	23.85
20386083	2011	5	27	1280	123.64	23.68
20386083	2011	5	27	1290	127.02	23.55
20386083	2011	5	27	1300	130.43	23.38
20386083	2011	5	27	1310	133.68	23.11

20386083	2011	5	27	1320	136.72	22.93
20386083	2011	5	27	1330	139.7	22.85
20386083	2011	5	27	1340	142.8	22.63
20386083	2011	5	27	1350	146.09	22.49
20386083	2011	5	27	1360	149.71	22.29
20386083	2011	5	27	1370	153.28	22.06
20386083	2011	5	27	1380	156.85	21.93
20386083	2011	5	27	1390	159.46	21.65
20386083	2011	5	27	1400	161.95	21.26
20386083	2011	5	27	1410	164.7	21.24
20386083	2011	5	27	1420	166.99	21.2
20386083	2011	5	27	1430	167.96	21.07
20386083	2011	5	27	1440	168.75	20.85
20386083	2011	5	27	1450	169.18	20.68
20386083	2011	5	27	1460	169.48	20.57
20386083	2011	5	27	1470	169.65	20.46
20386083	2011	5	27	1480	169.89	20.28
20386083	2011	5	27	1490	170.18	20.15
20386083	2011	5	27	1500	170.36	20.03
20386083	2011	5	27	1510	170.11	19.91
20386083	2011	5	27	1520	169.97	19.81
20386083	2011	5	27	1530	170.42	19.64
20386083	2011	5	27	1540	170.63	19.47
20386083	2011	5	27	1550	170.54	19.32
20386083	2011	5	27	1560	170.88	19.27
20386083	2011	5	27	1570	171.1	19.18
20386083	2011	5	27	1580	171	19.06
20386083	2011	5	27	1590	170.79	18.97
20386083	2011	5	27	1600	170.74	18.91
20386083	2011	5	27	1610	170.74	18.84
20386083	2011	5	27	1620	170.72	18.72
20386083	2011	5	27	1630	170.55	18.65
20386083	2011	5	27	1640	170.53	18.61
20386083	2011	5	27	1650	170.61	18.56
20386083	2011	5	27	1660	170.54	18.46
20386083	2011	5	27	1670	170.69	18.41
20386083	2011	5	27	1680	170.52	18.35
20386083	2011	5	27	1690	170.5	18.3
20386083	2011	5	27	1700	170.47	18.25
20386083	2011	5	27	1710	170.34	18.19
20386083	2011	5	27	1720	170.06	18.13
20386083	2011	5	27	1730	169.99	18.1
20386083	2011	5	27	1740	169.82	18.06
20386083	2011	5	27	1750	169.87	18.03
20386083	2011	5	27	1760	170.07	18
20386083	2011	5	27	1770	170.06	17.95
20386083	2011	5	27	1780	169.91	17.92

20386083	2011	5	27	1790	169.77	17.89
20386083	2011	5	27	1800	169.47	17.85
20386083	2011	5	27	1810	169.65	17.81
20386083	2011	5	27	1820	169.7	17.76
20386083	2011	5	27	1830	169.77	17.74
20386083	2011	5	27	1840	169.79	17.71
20386083	2011	5	27	1850	169.77	17.69
20386083	2011	5	27	1860	169.68	17.65
20386083	2011	5	27	1870	169.5	17.64
20386083	2011	5	27	1880	168.84	17.61
20386083	2011	5	27	1890	168.37	17.59
20386083	2011	5	27	1900	168.53	17.56
20386083	2011	5	27	1910	169.21	17.54
20386083	2011	5	27	1920	169.63	17.53
20386083	2011	5	27	1930	169.53	17.52
20386083	2011	5	27	1940	169.76	17.5
20386083	2011	5	27	1950	169.51	17.48
20386083	2011	5	27	1960	168.78	17.46
20386083	2011	5	27	1970	168.75	17.46
20386083	2011	5	27	1980	168.72	17.44
20386083	2011	5	27	1990	168.87	17.43
20386083	2011	5	27	2000	168.96	17.42
20386083	2011	5	27	2010	168.84	17.4
20386083	2011	5	27	2020	168.71	17.39
20386083	2011	5	27	2030	168.37	17.38
20386083	2011	5	27	2040	167.72	17.35
20386083	2011	5	27	2050	167.37	17.35
20386083	2011	5	27	2060	167.8	17.36
20386083	2011	5	27	2070	168.55	17.35
20386083	2011	5	27	2080	168.79	17.35
20386083	2011	5	27	2090	168.81	17.35
20386083	2011	5	27	2100	168.74	17.34
20386083	2011	5	27	2110	169.08	17.33
20386083	2011	5	27	2120	169.23	17.32
20386083	2011	5	27	2130	169.23	17.32
20386083	2011	5	27	2140	169.44	17.32
20386083	2011	5	27	2150	170.96	17.3
20386083	2011	5	27	2160	172.48	17.29
20386083	2011	5	27	2170	173.48	17.28
20386083	2011	5	27	2180	173.56	17.28
20386083	2011	5	27	2190	173.58	17.26
20386083	2011	5	27	2200	173.57	17.25
20386083	2011	5	27	2210	173.53	17.24
20386083	2011	5	27	2220	174.06	17.24
20386083	2011	5	27	2230	174.58	17.23
20386083	2011	5	27	2240	175.04	17.22
20386083	2011	5	27	2250	175.98	17.21

20386083	2011	5	27	2260	177.05	17.2
20386083	2011	5	27	2270	177.57	17.19
20386083	2011	5	27	2280	177.72	17.17
20386083	2011	5	27	2290	177.65	17.16
20386083	2011	5	27	2300	177.65	17.15
20386083	2011	5	27	2310	177.92	17.14
20386083	2011	5	27	2320	177.94	17.12
20386083	2011	5	27	2330	177.95	17.1
20386083	2011	5	27	2340	177.91	17.09
20386083	2011	5	27	2350	177.74	17.06
20386083	2011	5	27	2360	178.05	17.03
20386083	2011	5	27	2370	178.39	17
20386083	2011	5	27	2380	178.35	16.97
20386083	2011	5	27	2390	178.03	16.9
20386083	2011	5	27	2400	177.79	16.87
20386083	2011	5	27	2410	178.35	16.85
20386083	2011	5	27	2420	178.06	16.82
20386083	2011	5	27	2430	177.47	16.8
20386083	2011	5	27	2440	177.27	16.79
20386083	2011	5	27	2450	177.53	16.78
20386083	2011	5	27	2460	177.93	16.76
20386083	2011	5	27	2470	178.27	16.75
20386083	2011	5	27	2480	179.08	16.74
20386083	2011	5	27	2490	180.01	16.72
20386083	2011	5	27	2500	179.94	16.7
20386083	2011	5	27	2510	180.42	16.67
20386083	2011	5	27	2520	180.27	16.64
20386083	2011	5	27	2530	180.37	16.62
20386083	2011	5	27	2540	180.29	16.6
20386083	2011	5	27	2550	180.34	16.59
20386083	2011	5	27	2560	179.99	16.57
20386083	2011	5	27	2570	179.8	16.56
20386083	2011	5	27	2580	180.04	16.54
20386083	2011	5	27	2590	180.78	16.53
20386083	2011	5	27	2600	181.46	16.51
20386083	2011	5	27	2610	181.86	16.5
20386083	2011	5	27	2620	182.2	16.47
20386083	2011	5	27	2630	182.35	16.45
20386083	2011	5	27	2640	182.41	16.42
20386083	2011	5	27	2650	182.44	16.39
20386083	2011	5	27	2660	182.32	16.38
20386083	2011	5	27	2670	182.31	16.36
20386083	2011	5	27	2680	182.6	16.33
20386083	2011	5	27	2690	182.83	16.32
20386083	2011	5	27	2700	183.14	16.27
20386083	2011	5	27	2710	183.67	16.21
20386083	2011	5	27	2720	184.39	16.21

20386083	2011	5	27	2730	185.75	16.16
20386083	2011	5	27	2740	186.59	16.14
20386083	2011	5	27	2750	187.68	16.11
20386083	2011	5	27	2760	188.91	16.07
20386083	2011	5	27	2770	189.67	16.05
20386083	2011	5	27	2780	190.03	16.04
20386083	2011	5	27	2790	190.2	16.03
20386083	2011	5	27	2800	190.38	16.02
20386083	2011	5	27	2810	190.77	16.01
20386083	2011	5	27	2820	190.85	15.99
20386083	2011	5	27	2830	190.55	15.97
20386083	2011	5	27	2840	190.07	15.96
20386083	2011	5	27	2850	190	15.94
20386083	2011	5	27	2860	190.58	15.94
20386083	2011	5	27	2870	191.65	15.89
20386083	2011	5	27	2880	192.16	15.87
20386083	2011	5	27	2890	192.58	15.86
20386083	2011	5	27	2900	192.94	15.85
20386083	2011	5	27	2910	193.32	15.84
20386083	2011	5	27	2920	193.22	15.83
20386083	2011	5	27	2930	193.58	15.82
20386083	2011	5	27	2940	193.83	15.81
20386083	2011	5	27	2950	193.77	15.8
20386083	2011	5	27	2960	193.65	15.78
20386083	2011	5	27	2970	193.52	15.78
20386083	2011	5	27	2980	193.88	15.78
20386083	2011	5	27	2990	194.11	15.77
20386083	2011	5	27	3000	194.12	15.75
20386083	2011	5	27	3010	194.08	15.74
20386083	2011	5	27	3020	193.99	15.73
20386083	2011	5	27	3030	193.84	15.73
20386083	2011	5	27	3040	193.82	15.72
20386083	2011	5	27	3050	194.01	15.72
20386083	2011	5	27	3060	194.04	15.71
20386083	2011	5	27	3070	193.7	15.7
20386083	2011	5	27	3080	193.66	15.71
20386083	2011	5	27	3090	193.87	15.7
20386083	2011	5	27	3100	193.25	15.69
20386083	2011	5	27	3110	192.71	15.69
20386083	2011	5	27	3120	192.55	15.68
20386083	2011	5	27	3130	192.4	15.67
20386083	2011	5	27	3140	192.86	15.68
20386083	2011	5	27	3150	193.36	15.67
20386083	2011	5	27	3160	193.1	15.67
20386083	2011	5	27	3170	193.25	15.67
20386083	2011	5	27	3180	193.29	15.67
20386083	2011	5	27	3190	193.29	15.65

20386083	2011	5	27	3200	193.22	15.65
20386083	2011	5	27	3210	193.1	15.65
20386083	2011	5	27	3220	193.28	15.65
20386083	2011	5	27	3230	193.48	15.64
20386083	2011	5	27	3240	193.54	15.65
20386083	2011	5	27	3250	193.16	15.64
20386083	2011	5	27	3260	192.94	15.64
20386083	2011	5	27	3270	192.81	15.64
20386083	2011	5	27	3280	192.68	15.64
20386083	2011	5	27	3290	192.6	15.64
20386083	2011	5	27	3300	192.37	15.64
20386083	2011	5	27	3310	191.99	15.63
20386083	2011	5	27	3320	191.54	15.64
20386083	2011	5	27	3330	191.25	15.64
20386083	2011	5	27	3340	190.98	15.63
20386083	2011	5	27	3350	190.78	15.64
20386083	2011	5	27	3360	190.64	15.64
20386083	2011	5	27	3370	190.56	15.63
20386083	2011	5	27	3380	190.53	15.64
20386083	2011	5	27	3390	190.46	15.63
20386083	2011	5	27	3400	190.97	15.64
20386083	2011	5	27	3410	191.28	15.63
20386083	2011	5	27	3420	191.12	15.63
20386083	2011	5	27	3430	191.02	15.63
20386083	2011	5	27	3440	190.56	15.63
20386083	2011	5	27	3450	190.44	15.63
20386083	2011	5	27	3460	190.51	15.63
20386083	2011	5	27	3470	190.7	15.63
20386083	2011	5	27	3480	191	15.63
20386083	2011	5	27	3490	191.47	15.63
20386083	2011	5	27	3500	191.85	15.64
20386083	2011	5	27	3510	192.12	15.64
20386083	2011	5	27	3520	192.47	15.64
20386083	2011	5	27	3530	193.11	15.63
20386083	2011	5	27	3540	193.1	15.63
20386083	2011	5	27	3550	193.01	15.64
20386083	2011	5	27	3560	193.15	15.64
20386083	2011	5	27	3570	192.82	15.64
20386083	2011	5	27	3580	192.7	15.64
20386083	2011	5	27	3590	192.71	15.64
20386083	2011	5	27	3600	192.75	15.63
20386083	2011	5	27	3610	192.68	15.63
20386083	2011	5	27	3620	192.48	15.63
20386083	2011	5	27	3630	192.2	15.63
20386083	2011	5	27	3640	191.93	15.64
20386083	2011	5	27	3650	191.94	15.63
20386083	2011	5	27	3660	191.84	15.63

20386083	2011	5	27	3670	191.77	15.64
20386083	2011	5	27	3680	191.81	15.63
20386083	2011	5	27	3690	192.4	15.63
20386083	2011	5	27	3700	193.71	15.64
20386083	2011	5	27	3710	195.56	15.64
20386083	2011	5	27	3720	196.64	15.64
20386083	2011	5	27	3730	197.44	15.64
20386083	2011	5	27	3740	198.03	15.64
20386083	2011	5	27	3750	198.51	15.64
20386083	2011	5	27	3760	198.83	15.63
20386083	2011	5	27	3770	198.46	15.64
20386083	2011	5	27	3780	198.23	15.64
20386083	2011	5	27	3790	197.97	15.63
20386083	2011	5	27	3800	197.91	15.64
20386083	2011	5	27	3810	198.43	15.64
20386083	2011	5	27	3820	198.72	15.64
20386083	2011	5	27	3830	198.38	15.64
20386083	2011	5	27	3840	198.05	15.64
20386083	2011	5	27	3850	197.64	15.64
20386083	2011	5	27	3860	197.84	15.64
20386083	2011	5	27	3870	196.98	15.63
20386083	2011	5	27	3880	196.13	15.64
20386083	2011	5	27	3890	196.02	15.64
20386083	2011	5	27	3900	196.35	15.63
20386083	2011	5	27	3910	196.95	15.64
20386083	2011	5	27	3920	197.66	15.64
20386083	2011	5	27	3930	198.68	15.64
20386083	2011	5	27	3940	199.5	15.64
20386083	2011	5	27	3950	199.98	15.64
20386083	2011	5	27	3960	200.24	15.64
20386083	2011	5	27	3970	200.43	15.64
20386083	2011	5	27	3980	200.38	15.64
20386083	2011	5	27	3990	200.23	15.64
20386083	2011	5	27	4000	200.37	15.65
20386083	2011	5	27	4010	200.3	15.64
20386083	2011	5	27	4020	200.32	15.64
20386083	2011	5	27	4030	200.1	15.64
20386083	2011	5	27	4040	199.89	15.65
20386083	2011	5	27	4050	199.7	15.64
20386083	2011	5	27	4060	199.78	15.64
20386083	2011	5	27	4070	199.97	15.65
20386083	2011	5	27	4080	200.14	15.64
20386083	2011	5	27	4090	200.53	15.64
20386083	2011	5	27	4100	200.54	15.64
20386083	2011	5	27	4110	200.39	15.64
20386083	2011	5	27	4120	200.25	15.64
20386083	2011	5	27	4130	200.27	15.65

20386083	2011	5	27	4140	200.36	15.64
20386083	2011	5	27	4150	200.67	15.64
20386083	2011	5	27	4160	201.15	15.64
20386083	2011	5	27	4170	201.39	15.64
20386083	2011	5	27	4180	201.29	15.64
20386083	2011	5	27	4190	201.09	15.64
20386083	2011	5	27	4200	201.08	15.63
20386083	2011	5	27	4210	200.9	15.64
20386083	2011	5	27	4220	201.05	15.63
20386083	2011	5	27	4230	201.84	15.63
20386083	2011	5	27	4240	202.71	15.61
20386083	2011	5	27	4250	203.21	15.61
20386083	2011	5	27	4260	204	15.6
20386083	2011	5	27	4270	205.51	15.58
20386083	2011	5	27	4280	206.63	15.57
20386083	2011	5	27	4290	207.74	15.56
20386083	2011	5	27	4300	208.45	15.55
20386083	2011	5	27	4310	209.06	15.55
20386083	2011	5	27	4320	209.74	15.54
20386083	2011	5	27	4330	211.1	15.53
20386083	2011	5	27	4340	211.52	15.53
20386083	2011	5	27	4350	211.95	15.53
20386083	2011	5	27	4360	212.31	15.53
20386083	2011	5	27	4370	212.68	15.51
20386083	2011	5	27	4380	212.84	15.51
20386083	2011	5	27	4390	212.84	15.5
20386083	2011	5	27	4400	212.75	15.5
20386083	2011	5	27	4410	212.75	15.48
20386083	2011	5	27	4420	212.94	15.47
20386083	2011	5	27	4430	212.94	15.47
20386083	2011	5	27	4440	212.96	15.45
20386083	2011	5	27	4450	212.75	15.44
20386083	2011	5	27	4460	212.61	15.44
20386083	2011	5	27	4470	212.77	15.43
20386083	2011	5	27	4480	212.94	15.41
20386083	2011	5	27	4490	213.05	15.4
20386083	2011	5	27	4500	213	15.36
20386083	2011	5	27	4510	212.7	15.34
20386083	2011	5	27	4520	212.72	15.32
20386083	2011	5	27	4530	212.87	15.31
20386083	2011	5	27	4540	212.8	15.29
20386083	2011	5	27	4550	212.74	15.28
20386083	2011	5	27	4560	212.5	15.26
20386083	2011	5	27	4570	211.97	15.25
20386083	2011	5	27	4580	211.46	15.23
20386083	2011	5	27	4590	211.04	15.21
20386083	2011	5	27	4600	211.04	15.2

20386083	2011	5	27	4610	211.82	15.17
20386083	2011	5	27	4620	212.1	15.17
20386083	2011	5	27	4630	212.14	15.14
20386083	2011	5	27	4640	212.22	15.14
20386083	2011	5	27	4650	212.22	15.13
20386083	2011	5	27	4660	212.59	15.12
20386083	2011	5	27	4670	213.05	15.1
20386083	2011	5	27	4680	213.17	15.09
20386083	2011	5	27	4690	212.86	15.07
20386083	2011	5	27	4700	212.77	15.07
20386083	2011	5	27	4710	212.85	15.06
20386083	2011	5	27	4720	212.85	15.04
20386083	2011	5	27	4730	212.71	15.03
20386083	2011	5	27	4740	212.53	15.02
20386083	2011	5	27	4750	212.32	15.01
20386083	2011	5	27	4760	212.11	15
20386083	2011	5	27	4770	212.41	15
20386083	2011	5	27	4780	212.53	14.99
20386083	2011	5	27	4790	212.65	14.98
20386083	2011	5	27	4800	212.66	14.98
20386083	2011	5	27	4810	212.34	14.97
20386083	2011	5	27	4820	212	14.97
20386083	2011	5	27	4830	211.69	14.96
20386083	2011	5	27	4840	211.61	14.96
20386083	2011	5	27	4850	211.53	14.96
20386083	2011	5	27	4860	211.63	14.96
20386083	2011	5	27	4870	212.38	14.96
20386083	2011	5	27	4880	212.59	14.97
20386083	2011	5	27	4890	212.19	14.96
20386083	2011	5	27	4900	212.36	14.97
20386083	2011	5	27	4910	212.58	14.98
20386083	2011	5	27	4920	212.31	14.98
20386083	2011	5	27	4930	212.46	14.98
20386083	2011	5	27	4940	212.82	14.98
20386083	2011	5	27	4950	212.67	14.99
20386083	2011	5	27	4960	212.85	15
20386083	2011	5	27	4970	212.87	15
20386083	2011	5	27	4980	212.61	15.01
20386083	2011	5	27	4990	212.35	15.01
20386083	2011	5	27	5000	212.39	15.01
20386083	2011	5	27	5010	212.35	15.03
20386083	2011	5	27	5020	212.13	15.02
20386083	2011	5	27	5030	211.97	15.03
20386083	2011	5	27	5040	212.12	15.02
20386083	2011	5	27	5050	211.97	15.02
20386083	2011	5	27	5060	211.85	15.02
20386083	2011	5	27	5070	212.2	15.03

20386083	2011	5	27	5080	212.44	15.03
20386083	2011	5	27	5090	212.44	15.03
20386083	2011	5	27	5100	212.29	15.03
20386083	2011	5	27	5110	212.56	15.03
20386083	2011	5	27	5120	212.94	15.03
20386083	2011	5	27	5130	212.85	15.03
20386083	2011	5	27	5140	212.96	15.03
20386083	2011	5	27	5150	213.01	15.03
20386083	2011	5	27	5160	212.91	15.03
20386083	2011	5	27	5170	213.12	15.03
20386083	2011	5	27	5180	213.54	15.03
20386083	2011	5	27	5190	212.99	15.02
20386083	2011	5	27	5200	212.71	15.02
20386083	2011	5	27	5210	213.35	15.02
20386083	2011	5	27	5220	213.15	15
20386083	2011	5	27	5230	212.86	15.01
20386083	2011	5	27	5240	212.82	15
20386083	2011	5	27	5250	212.79	15
20386083	2011	5	27	5260	212.7	15
20386083	2011	5	27	5270	212.38	14.98
20386083	2011	5	27	5280	211.93	14.97
20386083	2011	5	27	5290	211.76	14.97
20386083	2011	5	27	5300	211.98	14.96
20386083	2011	5	27	5310	212.37	14.95
20386083	2011	5	27	5320	212.38	14.95
20386083	2011	5	27	5330	212.57	14.94
20386083	2011	5	27	5340	212.51	14.94
20386083	2011	5	27	5350	212.3	14.92
20386083	2011	5	27	5360	212.12	14.92
20386083	2011	5	27	5370	211.96	14.92
20386083	2011	5	27	5380	211.69	14.9
20386083	2011	5	27	5390	211.41	14.9
20386083	2011	5	27	5400	211.43	14.85
20386083	2011	5	27	5410	211.43	14.84
20386083	2011	5	27	5420	211.53	14.84
20386083	2011	5	27	5430	211.78	14.83
20386083	2011	5	27	5440	211.82	14.82
20386083	2011	5	27	5450	211.72	14.82
20386083	2011	5	27	5460	211.8	14.82
20386083	2011	5	27	5470	211.83	14.82
20386083	2011	5	27	5480	212.3	14.81
20386083	2011	5	27	5490	212.29	14.8
20386083	2011	5	27	5500	212.12	14.8
20386083	2011	5	27	5510	212.03	14.8
20386083	2011	5	27	5520	212	14.79
20386083	2011	5	27	5530	211.97	14.79
20386083	2011	5	27	5540	211.81	14.78

20386083	2011	5	27	5550	212.02	14.78
20386083	2011	5	27	5560	212.13	14.78
20386083	2011	5	27	5570	212.01	14.78
20386083	2011	5	27	5580	211.9	14.78
20386083	2011	5	27	5590	211.89	14.77
20386083	2011	5	27	5600	212.13	14.77
20386083	2011	5	27	5610	212.19	14.78
20386083	2011	5	27	5620	212.09	14.76
20386083	2011	5	27	5630	211.84	14.75
20386083	2011	5	27	5640	212.16	14.75
20386083	2011	5	27	5650	211.64	14.75
20386083	2011	5	27	5660	211.34	14.75
20386083	2011	5	27	5670	211.17	14.75
20386083	2011	5	27	5680	210.81	14.74
20386083	2011	5	27	5690	210.47	14.73
20386083	2011	5	27	5700	210.01	14.73
20386083	2011	5	27	5710	209.52	14.74
20386083	2011	5	27	5720	209.28	14.73
20386083	2011	5	27	5730	209.13	14.71
20386083	2011	5	27	5740	208.94	14.72
20386083	2011	5	27	5750	208.5	14.71
20386083	2011	5	27	5760	208.06	14.71
20386083	2011	5	27	5770	207.73	14.71
20386083	2011	5	27	5780	207.45	14.72
20386083	2011	5	27	5790	207.09	14.72
20386083	2011	5	27	5800	206.69	14.74
20386083	2011	5	27	5810	206.42	14.74
20386083	2011	5	27	5820	206.18	14.75
20386083	2011	5	27	5830	205.76	14.76
20386083	2011	5	27	5840	206.01	14.78
20386083	2011	5	27	5850	207.16	14.8
20386083	2011	5	27	5860	207.53	14.81
20386083	2011	5	27	5870	207.47	14.82
20386083	2011	5	27	5880	207.34	14.83
20386083	2011	5	27	5890	207.06	14.83
20386083	2011	5	27	5900	207	14.9
20386083	2011	5	27	5910	206.99	14.9
20386083	2011	5	27	5920	206.9	14.92
20386083	2011	5	27	5930	206.61	14.92
20386083	2011	5	27	5940	206.69	14.93
20386083	2011	5	27	5950	207.34	14.94
20386083	2011	5	27	5960	207.63	14.96
20386083	2011	5	27	5970	207.56	14.97
20386083	2011	5	27	5980	207.73	14.97
20386083	2011	5	27	5990	207.93	14.97
20386083	2011	5	27	6000	207.96	14.98
20386083	2011	5	27	6010	207.73	14.99

20386083	2011	5	27	6020	207.83	15
20386083	2011	5	27	6030	207.24	15
20386083	2011	5	27	6040	206.78	15.01
20386083	2011	5	27	6050	206.34	15.02
20386083	2011	5	27	6060	205.89	15.02
20386083	2011	5	27	6070	205.54	15.03
20386083	2011	5	27	6080	205.08	15.03
20386083	2011	5	27	6090	204.56	15.03
20386083	2011	5	27	6100	204	15.03
20386083	2011	5	27	6110	203.83	15.04
20386083	2011	5	27	6120	204.02	15.05
20386083	2011	5	27	6130	204.52	15.06
20386083	2011	5	27	6140	205.05	15.07
20386083	2011	5	27	6150	206.05	15.07
20386083	2011	5	27	6160	206.86	15.09
20386083	2011	5	27	6170	206.8	15.09
20386083	2011	5	27	6180	206.47	15.09
20386083	2011	5	27	6190	206.31	15.1
20386083	2011	5	27	6200	206.26	15.1
20386083	2011	5	27	6210	206.13	15.11
20386083	2011	5	27	6220	206.27	15.12
20386083	2011	5	27	6230	206.17	15.12
20386083	2011	5	27	6240	206.2	15.12
20386083	2011	5	27	6250	206.16	15.12
20386083	2011	5	27	6260	205.97	15.13
20386083	2011	5	27	6270	206.03	15.14
20386083	2011	5	27	6280	206.01	15.13
20386083	2011	5	27	6290	205.72	15.14
20386083	2011	5	27	6300	205.3	15.14
20386083	2011	5	27	6310	204.81	15.14
20386083	2011	5	27	6320	204.92	15.14
20386083	2011	5	27	6330	205.2	15.14
20386083	2011	5	27	6340	205.16	15.14
20386083	2011	5	27	6350	204.81	15.14
20386083	2011	5	27	6360	204.72	15.15
20386083	2011	5	27	6370	204.58	15.15
20386083	2011	5	27	6380	204.49	15.15
20386083	2011	5	27	6390	204.44	15.15
20386083	2011	5	27	6400	204.56	15.15
20386083	2011	5	27	6410	204.61	15.16
20386083	2011	5	27	6420	204.67	15.16
20386083	2011	5	27	6430	204.87	15.15
20386083	2011	5	27	6440	204.77	15.15
20386083	2011	5	27	6450	204.39	15.14
20386083	2011	5	27	6460	204.37	15.14
20386083	2011	5	27	6470	204.42	15.14
20386083	2011	5	27	6480	204.71	15.14

20386083	2011	5	27	6490	204.66	15.13
20386083	2011	5	27	6500	204.27	15.12
20386083	2011	5	27	6510	204.1	15.13
20386083	2011	5	27	6520	204.75	15.12
20386083	2011	5	27	6530	205.79	15.11
20386083	2011	5	27	6540	206.31	15.11
20386083	2011	5	27	6550	206.47	15.11
20386083	2011	5	27	6560	206.55	15.11
20386083	2011	5	27	6570	206.38	15.1
20386083	2011	5	27	6580	206.11	15.09
20386083	2011	5	27	6590	205.75	15.09
20386083	2011	5	27	6600	205.47	15.09
20386083	2011	5	27	6610	205.48	15.09
20386083	2011	5	27	6620	205.49	15.07
20386083	2011	5	27	6630	206.11	15.07
20386083	2011	5	27	6640	205.99	15.07
20386083	2011	5	27	6650	206.06	15.05
20386083	2011	5	27	6660	206.3	15.05
20386083	2011	5	27	6670	206.19	15.03
20386083	2011	5	27	6680	206.09	15.02
20386083	2011	5	27	6690	205.86	15
20386083	2011	5	27	6700	205.46	15.01
20386083	2011	5	27	6710	204.36	15
20386083	2011	5	27	6720	203.03	14.99
20386083	2011	5	27	6730	203.97	15
20386083	2011	5	27	6740	204.85	14.99
20386083	2011	5	27	6750	206.07	15
20386083	2011	5	27	6760	206.9	15.01
20386083	2011	5	27	6770	207.32	15.01
20386083	2011	5	27	6780	207.17	15.02
20386083	2011	5	27	6790	207.33	15.03
20386083	2011	5	27	6800	207.09	15.02
20386083	2011	5	27	6810	207.02	15.02
20386083	2011	5	27	6820	206.88	15.02
20386083	2011	5	27	6830	206.81	15.02
20386083	2011	5	27	6840	206.8	15.01
20386083	2011	5	27	6850	206.92	15.01
20386083	2011	5	27	6860	206.76	15.01
20386083	2011	5	27	6870	206.87	15.01
20386083	2011	5	27	6880	206.93	15
20386083	2011	5	27	6890	206.92	15
20386083	2011	5	27	6900	206.95	15
20386083	2011	5	27	6910	206.82	14.99
20386083	2011	5	27	6920	206.71	15
20386083	2011	5	27	6930	206.79	14.98
20386083	2011	5	27	6940	207.04	14.98
20386083	2011	5	27	6950	207.02	14.97

20386083	2011	5	27	6960	206.93	14.96
20386083	2011	5	27	6970	207.01	14.96
20386083	2011	5	27	6980	206.97	14.96
20386083	2011	5	27	6990	207.02	14.96
20386083	2011	5	27	7000	206.8	14.95
20386083	2011	5	27	7010	206.47	14.95
20386083	2011	5	27	7020	206.04	14.95
20386083	2011	5	27	7030	205.56	14.95
20386083	2011	5	27	7040	204.96	14.96
20386083	2011	5	27	7050	204.29	14.95
20386083	2011	5	27	7060	203.37	14.96
20386083	2011	5	27	7070	202.54	14.96
20386083	2011	5	27	7080	202.01	14.97
20386083	2011	5	27	7090	202	14.97
20386083	2011	5	27	7100	202.31	14.98
20386083	2011	5	27	7110	202.14	15
20386083	2011	5	27	7120	201.88	15
20386083	2011	5	27	7130	201.52	15.03
20386083	2011	5	27	7140	201.14	15.03
20386083	2011	5	27	7150	200.56	15.04
20386083	2011	5	27	7160	199.64	15.06
20386083	2011	5	27	7170	198.88	15.07
20386083	2011	5	27	7180	198.74	15.07
20386083	2011	5	27	7190	198.25	15.09
20386083	2011	5	27	7200	196.81	15.1
20386083	2011	5	27	7210	196.39	15.1
20386083	2011	5	27	7220	196.69	15.11
20386083	2011	5	27	7230	196.69	15.12
20386083	2011	5	27	7240	196.53	15.13
20386083	2011	5	27	7250	196.62	15.14
20386083	2011	5	27	7260	196.87	15.15
20386083	2011	5	27	7270	196.86	15.15
20386083	2011	5	27	7280	196.68	15.17
20386083	2011	5	27	7290	196.47	15.18
20386083	2011	5	27	7300	196.31	15.18
20386083	2011	5	27	7310	196.64	15.2
20386083	2011	5	27	7320	196.85	15.21
20386083	2011	5	27	7330	196.91	15.21
20386083	2011	5	27	7340	196.52	15.21
20386083	2011	5	27	7350	195.25	15.22
20386083	2011	5	27	7360	194.37	15.22
20386083	2011	5	27	7370	193.77	15.21
20386083	2011	5	27	7380	193.64	15.22
20386083	2011	5	27	7390	193.86	15.23
20386083	2011	5	27	7400	193.68	15.22
20386083	2011	5	27	7410	193.38	15.23
20386083	2011	5	27	7420	193.38	15.24

20386083	2011	5	27	7430	193.47	15.24
20386083	2011	5	27	7440	193.8	15.24
20386083	2011	5	27	7450	194.48	15.25
20386083	2011	5	27	7460	194.66	15.24
20386083	2011	5	27	7470	194.6	15.25
20386083	2011	5	27	7480	194.41	15.25
20386083	2011	5	27	7490	194.43	15.25
20386083	2011	5	27	7500	194.64	15.25
20386083	2011	5	27	7510	194.86	15.25
20386083	2011	5	27	7520	195.07	15.25
20386083	2011	5	27	7530	194.81	15.25
20386083	2011	5	27	7540	194.33	15.25
20386083	2011	5	27	7550	193.96	15.26
20386083	2011	5	27	7560	193.69	15.26
20386083	2011	5	27	7570	192.82	15.26
20386083	2011	5	27	7580	192.31	15.27
20386083	2011	5	27	7590	193.21	15.28
20386083	2011	5	27	7600	193.51	15.28
20386083	2011	5	27	7610	193.65	15.28
20386083	2011	5	27	7620	194.34	15.29
20386083	2011	5	27	7630	195.07	15.31
20386083	2011	5	27	7640	195.27	15.31
20386083	2011	5	27	7650	195.17	15.31
20386083	2011	5	27	7660	195.21	15.32
20386083	2011	5	27	7670	196.06	15.33
20386083	2011	5	27	7680	196.5	15.34
20386083	2011	5	27	7690	196.71	15.33
20386083	2011	5	27	7700	197.41	15.34
20386083	2011	5	27	7710	199.18	15.35
20386083	2011	5	27	7720	199.18	15.35
20386083	2011	5	27	7730	199.08	15.35
20386083	2011	5	27	7740	198.94	15.35
20386083	2011	5	27	7750	199.36	15.35
20386083	2011	5	27	7760	199.86	15.35
20386083	2011	5	27	7770	200.47	15.36
20386083	2011	5	27	7780	201.08	15.35
20386083	2011	5	27	7790	201.58	15.35
20386083	2011	5	27	7800	201.39	15.36
20386083	2011	5	27	7810	202.77	15.36
20386083	2011	5	27	7820	203.26	15.36
20386083	2011	5	27	7830	203.73	15.35
20386083	2011	5	27	7840	205.62	15.35
20386083	2011	5	27	7850	207.68	15.33
20386083	2011	5	27	7860	210.52	15.3
20386083	2011	5	27	7870	213.63	15.27
20386083	2011	5	27	7880	214.97	15.25
20386083	2011	5	27	7890	215.2	15.21

20386083	2011	5	27	7900	215.15	15.18
20386083	2011	5	27	7910	215.65	15.17
20386083	2011	5	27	7920	215.87	15.13
20386083	2011	5	27	7930	216	15.09
20386083	2011	5	27	7940	216.63	15.07
20386083	2011	5	27	7950	217.25	15.04
20386083	2011	5	27	7960	218.08	15
20386083	2011	5	27	7970	219.17	14.99
20386083	2011	5	27	7980	220.25	14.96
20386083	2011	5	27	7990	221.31	14.94
20386083	2011	5	27	8000	221.31	14.85
20386083	2011	5	27	8010	221.57	14.84
20386083	2011	5	27	8020	221.63	14.82
20386083	2011	5	27	8030	221.85	14.78
20386083	2011	5	27	8040	222.04	14.75
20386083	2011	5	27	8050	222.49	14.72
20386083	2011	5	27	8060	223.83	14.7
20386083	2011	5	27	8070	223.98	14.66
20386083	2011	5	27	8080	223.63	14.64
20386083	2011	5	27	8090	223.84	14.61
20386083	2011	5	27	8100	224.64	14.59
20386083	2011	5	27	8110	224.95	14.57
20386083	2011	5	27	8120	225.06	14.54
20386083	2011	5	27	8130	225.07	14.55
20386083	2011	5	27	8140	225.06	14.54
20386083	2011	5	27	8150	224.8	14.52
20386083	2011	5	27	8160	224.58	14.5
20386083	2011	5	27	8170	224.46	14.49
20386083	2011	5	27	8180	225.09	14.48
20386083	2011	5	27	8190	225.13	14.46
20386083	2011	5	27	8200	225.07	14.45
20386083	2011	5	27	8210	224.95	14.44
20386083	2011	5	27	8220	224.74	14.42
20386083	2011	5	27	8230	224.43	14.42
20386083	2011	5	27	8240	224.48	14.39
20386083	2011	5	27	8250	224.53	14.39
20386083	2011	5	27	8260	224.56	14.39
20386083	2011	5	27	8270	224.67	14.37
20386083	2011	5	27	8280	224.72	14.35
20386083	2011	5	27	8290	224.61	14.35
20386083	2011	5	27	8300	224.77	14.34
20386083	2011	5	27	8310	225.02	14.33
20386083	2011	5	27	8320	225.05	14.32
20386083	2011	5	27	8330	225.19	14.32
20386083	2011	5	27	8340	225.02	14.31
20386083	2011	5	27	8350	225.25	14.28
20386083	2011	5	27	8360	225.04	14.29

20386083	2011	5	27	8370	224.96	14.28
20386083	2011	5	27	8380	224.8	14.28
20386083	2011	5	27	8390	224.44	14.28
20386083	2011	5	27	8400	224.24	14.27
20386083	2011	5	27	8410	224.16	14.27
20386083	2011	5	27	8420	223.93	14.26
20386083	2011	5	27	8430	224.4	14.26
20386083	2011	5	27	8440	224.92	14.25
20386083	2011	5	27	8450	225.01	14.25
20386083	2011	5	27	8460	225	14.25
20386083	2011	5	27	8470	225.02	14.24
20386083	2011	5	27	8480	224.7	14.24
20386083	2011	5	27	8490	224.26	14.23
20386083	2011	5	27	8500	224.12	14.23
20386083	2011	5	27	8510	224.23	14.22
20386083	2011	5	27	8520	224.37	14.22
20386083	2011	5	27	8530	225.78	14.22
20386083	2011	5	27	8540	226.11	14.21
20386083	2011	5	27	8550	225.16	14.21
20386083	2011	5	27	8560	224.78	14.21
20386083	2011	5	27	8570	224.4	14.21
20386083	2011	5	27	8580	224.47	14.21
20386083	2011	5	27	8590	225.12	14.21
20386083	2011	5	27	8600	226.06	14.2
20386083	2011	5	27	8610	226.3	14.2
20386083	2011	5	27	8620	226.39	14.2
20386083	2011	5	27	8630	226.06	14.2
20386083	2011	5	27	8640	225.55	14.19
20386083	2011	5	27	8650	225.71	14.19
20386083	2011	5	27	8660	225.73	14.19
20386083	2011	5	27	8670	225.71	14.19
20386083	2011	5	27	8680	225.52	14.18
20386083	2011	5	27	8690	225.61	14.19
20386083	2011	5	27	8700	226.04	14.19
20386083	2011	5	27	8710	225.46	14.18
20386083	2011	5	27	8720	225.14	14.19
20386083	2011	5	27	8730	224.69	14.17
20386083	2011	5	27	8740	224.43	14.18
20386083	2011	5	27	8750	224.51	14.17
20386083	2011	5	27	8760	225.13	14.18
20386083	2011	5	27	8770	226.14	14.17
20386083	2011	5	27	8780	225.77	14.17
20386083	2011	5	27	8790	225.4	14.17
20386083	2011	5	27	8800	225.65	14.17
20386083	2011	5	27	8810	226.61	14.17
20386083	2011	5	27	8820	226.96	14.18
20386083	2011	5	27	8830	226.73	14.17

20386083	2011	5	27	8840	226.69	14.17
20386083	2011	5	27	8850	226.55	14.17
20386083	2011	5	27	8860	226.45	14.17
20386083	2011	5	27	8870	226.46	14.17
20386083	2011	5	27	8880	226.82	14.17
20386083	2011	5	27	8890	226.99	14.17
20386083	2011	5	27	8900	226.94	14.17
20386083	2011	5	27	8910	226.88	14.18
20386083	2011	5	27	8920	227.28	14.17
20386083	2011	5	27	8930	227.49	14.17
20386083	2011	5	27	8940	227.79	14.17
20386083	2011	5	27	8950	228.36	14.18
20386083	2011	5	27	8960	228.62	14.18
20386083	2011	5	27	8970	228.63	14.17
20386083	2011	5	27	8980	228.22	14.17
20386083	2011	5	27	8990	227.06	14.17
20386083	2011	5	27	9000	226.61	14.17
20386083	2011	5	27	9010	226.71	14.17
20386083	2011	5	27	9020	226.65	14.17
20386083	2011	5	27	9030	226.46	14.17
20386083	2011	5	27	9040	226.52	14.18
20386083	2011	5	27	9050	227.34	14.17
20386083	2011	5	27	9060	228.2	14.18
20386083	2011	5	27	9070	228.58	14.17
20386083	2011	5	27	9080	228.63	14.17
20386083	2011	5	27	9090	230.36	14.18
20386083	2011	5	27	9100	232.79	14.18
20386083	2011	5	27	9110	235.06	14.19
20386083	2011	5	27	9120	236.15	14.19
20386083	2011	5	27	9130	236.52	14.19
20386083	2011	5	27	9140	236.73	14.19
20386083	2011	5	27	9150	236.88	14.19
20386083	2011	5	27	9160	237.91	14.19
20386083	2011	5	27	9170	238.18	14.19
20386083	2011	5	27	9180	237.65	14.18
20386083	2011	5	27	9190	237.44	14.19
20386083	2011	5	27	9200	237.23	14.19
20386083	2011	5	27	9210	237.55	14.18
20386083	2011	5	27	9220	238.14	14.18
20386083	2011	5	27	9230	238.43	14.19
20386083	2011	5	27	9240	238.85	14.18
20386083	2011	5	27	9250	239.93	14.17
20386083	2011	5	27	9260	241.45	14.18
20386083	2011	5	27	9270	243.02	14.17
20386083	2011	5	27	9280	244.49	14.17
20386083	2011	5	27	9290	245.61	14.17
20386083	2011	5	27	9300	247.4	14.17

20386083	2011	5	27	9310	249.19	14.17
20386083	2011	5	27	9320	250.55	14.15
20386083	2011	5	27	9330	252.19	14.14
20386083	2011	5	27	9340	253.79	14.13
20386083	2011	5	27	9350	254.98	14.13
20386083	2011	5	27	9360	255.11	14.1
20386083	2011	5	27	9370	254.85	14.1
20386083	2011	5	27	9380	254.9	14.1
20386083	2011	5	27	9390	255.02	14.07
20386083	2011	5	27	9400	254.88	14.04
20386083	2011	5	27	9410	255.32	14.03
20386083	2011	5	27	9420	255.76	14.01
20386083	2011	5	27	9430	255.74	13.99
20386083	2011	5	27	9440	255.46	13.97
20386083	2011	5	27	9450	255.32	13.95
20386083	2011	5	27	9460	255.41	13.94
20386083	2011	5	27	9470	255.03	13.91
20386083	2011	5	27	9480	254.61	13.89
20386083	2011	5	27	9490	254.4	13.88
20386083	2011	5	27	9500	255.23	13.88
20386083	2011	5	27	9510	255.52	13.87
20386083	2011	5	27	9520	255.86	13.87
20386083	2011	5	27	9530	256.06	13.86
20386083	2011	5	27	9540	255.84	13.8
20386083	2011	5	27	9550	255.88	13.78
20386083	2011	5	27	9560	255.98	13.77
20386083	2011	5	27	9570	256.22	13.78
20386083	2011	5	27	9580	256.11	13.75
20386083	2011	5	27	9590	256.3	13.75
20386083	2011	5	27	9600	256.22	13.74
20386083	2011	5	27	9610	256.42	13.74
20386083	2011	5	27	9620	256.79	13.71
20386083	2011	5	27	9630	256.84	13.7
20386083	2011	5	27	9640	256.86	13.68
20386083	2011	5	27	9650	256.87	13.69
20386083	2011	5	27	9660	257.18	13.68
20386083	2011	5	27	9670	257.14	13.66
20386083	2011	5	27	9680	257.31	13.66
20386083	2011	5	27	9690	257.47	13.64
20386083	2011	5	27	9700	256.85	13.62
20386083	2011	5	27	9710	256.6	13.61
20386083	2011	5	27	9720	256.51	13.6
20386083	2011	5	27	9730	256.55	13.61
20386083	2011	5	27	9740	256.61	13.6
20386083	2011	5	27	9750	256.56	13.6
20386083	2011	5	27	9760	256.49	13.6
20386083	2011	5	27	9770	256.29	13.6

20386083	2011	5	27	9780	256.45	13.61
20386083	2011	5	27	9790	256.6	13.61
20386083	2011	5	27	9800	256.81	13.61
20386083	2011	5	27	9810	256.83	13.61
20386083	2011	5	27	9820	256.78	13.61
20386083	2011	5	27	9830	256.74	13.6
20386083	2011	5	27	9840	256.54	13.6
20386083	2011	5	27	9850	256.13	13.59
20386083	2011	5	27	9860	256.19	13.6
20386083	2011	5	27	9870	257.22	13.6
20386083	2011	5	27	9880	258.31	13.6
20386083	2011	5	27	9890	258.77	13.6
20386083	2011	5	27	9900	258.89	13.6
20386083	2011	5	27	9910	259.49	13.6
20386083	2011	5	27	9920	259.42	13.57
20386083	2011	5	27	9930	259.42	13.57
20386083	2011	5	27	9940	259.69	13.55
20386083	2011	5	27	9950	259.67	13.55
20386083	2011	5	27	9960	259.69	13.55
20386083	2011	5	27	9970	259.63	13.53
20386083	2011	5	27	9980	259.28	13.53
20386083	2011	5	27	9990	259.38	13.51
20386083	2011	5	27	10000	259.36	13.51
20386083	2011	5	27	10010	259.3	13.51
20386083	2011	5	27	10020	259.34	13.5
20386083	2011	5	27	10030	259.32	13.49
20386083	2011	5	27	10040	259.1	13.49
20386083	2011	5	27	10050	259.13	13.47
20386083	2011	5	27	10060	259.42	13.46
20386083	2011	5	27	10070	259.29	13.45
20386083	2011	5	27	10080	259.19	13.44
20386083	2011	5	27	10090	258.8	13.44
20386083	2011	5	27	10100	258.33	13.42
20386083	2011	5	27	10110	258.03	13.42
20386083	2011	5	27	10120	258.13	13.42
20386083	2011	5	27	10130	258.71	13.42
20386083	2011	5	27	10140	258.89	13.41
20386083	2011	5	27	10150	259.49	13.4
20386083	2011	5	27	10160	260.85	13.39
20386083	2011	5	27	10170	261.14	13.4
20386083	2011	5	27	10180	261.27	13.4
20386083	2011	5	27	10190	261.34	13.39
20386083	2011	5	27	10200	260.8	13.39
20386083	2011	5	27	10210	260.6	13.39
20386083	2011	5	27	10220	260.72	13.39
20386083	2011	5	27	10230	260.69	13.38
20386083	2011	5	27	10240	260.64	13.37

20386083	2011	5	27	10250	260.38	13.37
20386083	2011	5	27	10260	260.12	13.36
20386083	2011	5	27	10270	259.73	13.37
20386083	2011	5	27	10280	259.36	13.36
20386083	2011	5	27	10290	258.96	13.35
20386083	2011	5	27	10300	258.75	13.35
20386083	2011	5	27	10310	259.49	13.35
20386083	2011	5	27	10320	260.07	13.34
20386083	2011	5	27	10330	260.11	13.35
20386083	2011	5	27	10340	259.8	13.34
20386083	2011	5	27	10350	259.61	13.34
20386083	2011	5	27	10360	259.61	13.34
20386083	2011	5	27	10370	259.39	13.33
20386083	2011	5	27	10380	259.44	13.32
20386083	2011	5	27	10390	259.54	13.33
20386083	2011	5	27	10400	259.5	13.33
20386083	2011	5	27	10410	259.69	13.32
20386083	2011	5	27	10420	259.72	13.32
20386083	2011	5	27	10430	259.65	13.32
20386083	2011	5	27	10440	259.6	13.32
20386083	2011	5	27	10450	259.49	13.32
20386083	2011	5	27	10460	259.49	13.32
20386083	2011	5	27	10470	259.43	13.31
20386083	2011	5	27	10480	259.78	13.32
20386083	2011	5	27	10490	259.68	13.32
20386083	2011	5	27	10500	259.44	13.31
20386083	2011	5	27	10510	259.47	13.31
20386083	2011	5	27	10520	259.56	13.31
20386083	2011	5	27	10530	259.79	13.31
20386083	2011	5	27	10540	259.8	13.3
20386083	2011	5	27	10550	259.7	13.31
20386083	2011	5	27	10560	259.61	13.31
20386083	2011	5	27	10570	259.49	13.3
20386083	2011	5	27	10580	259.61	13.3
20386083	2011	5	27	10590	259.42	13.29
20386083	2011	5	27	10600	259.56	13.3
20386083	2011	5	27	10610	259.67	13.29
20386083	2011	5	27	10620	259.5	13.29
20386083	2011	5	27	10630	259.93	13.29
20386083	2011	5	27	10640	259.76	13.29
20386083	2011	5	27	10650	259.53	13.28
20386083	2011	5	27	10660	259.36	13.28
20386083	2011	5	27	10670	259.56	13.28
20386083	2011	5	27	10680	259.65	13.28
20386083	2011	5	27	10690	259.59	13.28
20386083	2011	5	27	10700	259.33	13.28
20386083	2011	5	27	10710	258.96	13.27

20386083	2011	5	27	10720	258.67	13.27
20386083	2011	5	27	10730	259.14	13.28
20386083	2011	5	27	10740	259.14	13.28
20386083	2011	5	27	10750	259.13	13.28
20386083	2011	5	27	10760	259.15	13.27
20386083	2011	5	27	10770	259.16	13.27
20386083	2011	5	27	10780	259.13	13.26
20386083	2011	5	27	10790	259.12	13.27
20386083	2011	5	27	10800	259.31	13.26
20386083	2011	5	27	10810	259.12	13.26
20386083	2011	5	27	10820	258.34	13.26
20386083	2011	5	27	10830	257.5	13.25
20386083	2011	5	27	10840	256.66	13.25
20386083	2011	5	27	10850	256.15	13.25
20386083	2011	5	27	10860	255.92	13.25
20386083	2011	5	27	10870	255.68	13.25
20386083	2011	5	27	10880	255	13.25
20386083	2011	5	27	10890	254.14	13.25
20386083	2011	5	27	10900	253.05	13.25
20386083	2011	5	27	10910	251.45	13.25
20386083	2011	5	27	10920	250.8	13.25
20386083	2011	5	27	10930	250.1	13.25
20386083	2011	5	27	10940	249.18	13.25
20386083	2011	5	27	10950	248.63	13.25
20386083	2011	5	27	10960	248.13	13.25
20386083	2011	5	27	10970	247.6	13.27
20386083	2011	5	27	10980	248.03	13.29
20386083	2011	5	27	10990	248.87	13.32
20386083	2011	5	27	11000	248.74	13.32
20386083	2011	5	27	11010	248.33	13.33
20386083	2011	5	27	11020	247.3	13.35
20386083	2011	5	27	11030	246.51	13.36
20386083	2011	5	27	11040	245.44	13.37
20386083	2011	5	27	11050	243.99	13.39
20386083	2011	5	27	11060	242.97	13.41
20386083	2011	5	27	11070	241.47	13.44
20386083	2011	5	27	11080	240.14	13.46
20386083	2011	5	27	11090	239.5	13.48
20386083	2011	5	27	11100	238.94	13.5
20386083	2011	5	27	11110	237.51	13.51
20386083	2011	5	27	11120	236.25	13.53
20386083	2011	5	27	11130	234.47	13.57
20386083	2011	5	27	11140	233.81	13.6
20386083	2011	5	27	11150	232.11	13.63
20386083	2011	5	27	11160	230	13.65
20386083	2011	5	27	11170	227.9	13.66
20386083	2011	5	27	11180	226.12	13.69

20386083	2011	5	27	11190	224.02	13.71
20386083	2011	5	27	11200	221.89	13.73
20386083	2011	5	27	11210	220.04	13.75
20386083	2011	5	27	11220	219.25	13.78
20386083	2011	5	27	11230	217.53	13.8
20386083	2011	5	27	11240	215.55	13.88
20386083	2011	5	27	11250	213.4	13.91
20386083	2011	5	27	11260	211.2	13.95
20386083	2011	5	27	11270	208.88	13.98
20386083	2011	5	27	11280	207.82	14.03
20386083	2011	5	27	11290	206.61	14.08
20386083	2011	5	27	11300	205.46	14.09
20386083	2011	5	27	11310	203.87	14.1
20386083	2011	5	27	11320	202.27	14.16
20386083	2011	5	27	11330	201.11	14.17
20386083	2011	5	27	11340	199.99	14.26
20386083	2011	5	27	11350	198.31	14.28
20386083	2011	5	27	11360	195.86	14.35
20386083	2011	5	27	11370	193.82	14.39
20386083	2011	5	27	11380	192.03	14.43
20386083	2011	5	27	11390	189.61	14.47
20386083	2011	5	27	11400	188.46	14.5
20386083	2011	5	27	11410	186.43	14.57
20386083	2011	5	27	11420	183.56	14.61
20386083	2011	5	27	11430	181.04	14.67
20386083	2011	5	27	11440	179.15	14.73
20386083	2011	5	27	11450	177.83	14.76
20386083	2011	5	27	11460	176.6	14.8
20386083	2011	5	27	11470	175.48	14.85
20386083	2011	5	27	11480	174.26	14.93
20386083	2011	5	27	11490	173.07	14.96
20386083	2011	5	27	11500	171.93	15
20386083	2011	5	27	11510	171.39	15.05
20386083	2011	5	27	11520	171.73	15.28
20386083	2011	5	27	11530	171.79	15.28
20386083	2011	5	27	11540	171.75	15.3
20386083	2011	5	27	11550	170.39	15.37
20386083	2011	5	27	11560	167.78	15.42
20386083	2011	5	27	11570	166.47	15.58
20386083	2011	5	27	11580	165.24	15.62
20386083	2011	5	27	11590	164.02	15.67
20386083	2011	5	27	11600	163.13	15.71
20386083	2011	5	27	11610	162.24	15.75
20386083	2011	5	27	11620	161.41	15.78
20386083	2011	5	27	11630	159.73	15.88
20386083	2011	5	27	11640	157.91	16
20386083	2011	5	27	11650	156.69	16.21

20386083	2011	5	27	11660	154.98	16.31
20386083	2011	5	27	11670	152.65	16.39
20386083	2011	5	27	11680	150.36	16.49
20386083	2011	5	27	11690	147.81	16.59
20386083	2011	5	27	11700	145.75	16.71
20386083	2011	5	27	11710	143.59	16.89
20386083	2011	5	27	11720	141.2	17.02
20386083	2011	5	27	11730	138.88	17.11
20386083	2011	5	27	11740	136.49	17.23
20386083	2011	5	27	11750	135.8	17.53
20386083	2011	5	27	11760	135.79	17.82
20386083	2011	5	27	11770	134.55	17.87
20386083	2011	5	27	11780	132.53	17.88
20386083	2011	5	27	11790	131.93	18.19
20386083	2011	5	27	11800	129.86	18.21
20386083	2011	5	27	11810	129.45	18.53
20386083	2011	5	27	11820	130.1	18.68
20386083	2011	5	27	11830	130.05	18.66
20386083	2011	5	27	11840	129.63	18.74
20386083	2011	5	27	11850	129.53	18.72
20386083	2011	5	27	11860	129.36	18.77
20386083	2011	5	27	11870	129.7	18.85
20386083	2011	5	27	11880	130	18.91
20386083	2011	5	27	11890	130.01	18.95
20386083	2011	5	27	11900	130.17	19.06
20386083	2011	5	27	11910	130.08	19.15
20386083	2011	5	27	11920	128.14	19.17
20386083	2011	5	27	11930	125.39	19.2
20386083	2011	5	27	11940	122.56	19.28
20386083	2011	5	27	11950	119.99	19.39
20386083	2011	5	27	11960	117.64	19.44
20386083	2011	5	27	11970	115.1	19.5
20386083	2011	5	27	11980	112.13	19.56
20386083	2011	5	27	11990	109.44	19.67
20386083	2011	5	27	12000	106.56	19.79
20386083	2011	5	27	12010	103.83	19.87
20386083	2011	5	27	12020	101.68	19.99
20386083	2011	5	27	12030	99.7	20.25
20386083	2011	5	27	12040	97.38	20.3
20386083	2011	5	27	12050	95	20.46
20386083	2011	5	27	12060	92.51	20.62
20386083	2011	5	27	12070	90.5	20.75
20386083	2011	5	27	12080	88.41	20.97
20386083	2011	5	27	12090	86.15	21.19
20386083	2011	5	27	12100	84.26	21.5
20386083	2011	5	27	12110	82.21	21.72
20386083	2011	5	27	12120	79.74	21.84

20386083	2011	5	27	12130	77.63	22.14
20386083	2011	5	27	12140	75.97	22.3
20386083	2011	5	27	12150	73.71	22.44
20386083	2011	5	27	12160	72.21	22.52
20386083	2011	5	27	12170	71.86	22.68
20386083	2011	5	27	12180	71.17	22.88
20386083	2011	5	27	12190	69.82	22.99
20386083	2011	5	27	12200	67.93	23.16
20386083	2011	5	27	12210	65.44	23.35
20386083	2011	5	27	12220	62.87	23.47
20386083	2011	5	27	12230	60.33	23.57
20386083	2011	5	27	12240	57.84	23.67
20386083	2011	5	27	12250	55.38	23.8
20386083	2011	5	27	12260	53.12	24
20386083	2011	5	27	12270	51.49	24.16
20386083	2011	5	27	12280	49.91	24.42
20386083	2011	5	27	12290	47.76	24.46
20386083	2011	5	27	12300	45.53	24.54
20386083	2011	5	27	12310	43.26	24.67
20386083	2011	5	27	12320	40.99	24.82
20386083	2011	5	27	12330	38.81	24.9
20386083	2011	5	27	12340	36.63	25.07
20386083	2011	5	27	12350	34.42	25.19
20386083	2011	5	27	12360	32.21	25.37
20386083	2011	5	27	12370	30.28	25.52
20386083	2011	5	27	12380	29.74	25.84
20386083	2011	5	27	12390	29.75	25.87
20386083	2011	5	27	12400	29.47	25.93
20386083	2011	5	27	12410	29.02	26.21
20386083	2011	5	27	12420	28.83	26.28
20386083	2011	5	27	12430	28.75	26.41
20386083	2011	5	27	12440	28.85	26.49
20386083	2011	5	27	12450	28.99	26.57
20386083	2011	5	27	12460	29.19	26.63
20386083	2011	5	27	12470	29.43	26.68
20386083	2011	5	27	12480	29.68	26.76
20386083	2011	5	27	12490	29.94	26.82
20386083	2011	5	27	12500	30.18	26.91
20386083	2011	5	27	12510	30.4	26.96
20386083	2011	5	27	12520	30.63	27.01
20386083	2011	5	27	12530	30.83	27.09
20386083	2011	5	27	12540	31.07	27.15
20386083	2011	5	27	12550	31.36	27.2
20386083	2011	5	27	12560	31.44	27.28
20386083	2011	5	27	12570	31.31	27.34
20386083	2011	5	27	12580	31.21	27.39
20386083	2011	5	27	12590	31.21	27.46

20386083	2011	5	27	12600	31.38	27.5
20386083	2011	5	27	12610	31.56	27.53
20386083	2011	5	27	12620	31.81	27.57
20386083	2011	5	27	12630	32.1	27.6
20386083	2011	5	27	12640	32.38	27.64
20386083	2011	5	27	12650	32.1	27.69
20386083	2011	5	27	12660	31.95	27.73
20386083	2011	5	27	12670	31.52	27.77
20386083	2011	5	27	12680	31.35	27.81
20386083	2011	5	27	12690	31.34	27.85
20386083	2011	5	27	12700	31.44	27.87
20386083	2011	5	27	12710	31.36	27.89
20386083	2011	5	27	12720	30.74	27.93
20386083	2011	5	27	12730	30.21	27.95
20386083	2011	5	27	12740	28.78	27.99
20386083	2011	5	27	12750	27.46	28.03
20386083	2011	5	27	12760	26.2	28.06
20386083	2011	5	27	12770	24.86	28.09
20386083	2011	5	27	12780	23.04	28.11
20386083	2011	5	27	12790	22.14	28.16
20386083	2011	5	27	12800	21.79	28.17
20386083	2011	5	27	12810	21.14	28.2
20386083	2011	5	27	12820	20.25	28.21
20386083	2011	5	27	12830	19.87	28.22
20386083	2011	5	27	12840	19.7	28.23
20386083	2011	5	27	12850	19.44	28.25
20386083	2011	5	27	12860	18.99	28.28
20386083	2011	5	27	12870	18.19	28.3
20386083	2011	5	27	12880	17.8	28.31
20386083	2011	5	27	12890	16.34	28.35
20386083	2011	5	27	12900	13.77	28.37
20386083	2011	5	27	12910	11.19	28.4
20386083	2011	5	27	12920	8.44	28.43
20386083	2011	5	27	12930	5.47	28.46
20386083	2011	5	27	12940	3.5	28.5
20386083	2011	5	27	12950	2.74	28.53
20386083	2011	5	27	12960	2.29	28.54
20386083	2011	5	27	12970	1.94	28.56
20386083	2011	5	27	12980	1.67	28.59
20386083	2011	5	27	12990	2.14	28.64
20386083	2011	5	27	13000	0.88	28.67
20386083	2011	5	27	13010	0.58	28.69
20386083	2011	5	27	13020	0.61	28.68
20386083	2011	5	27	13030	0.59	28.69
20386083	2011	5	27	13040	0.6	28.78
20386083	2011	5	27	13050	0.57	28.81
20386083	2011	5	27	13060	0.55	28.86

20386083	2011	5	27	13070	0.6	28.89
20386083	2011	5	27	13080	0.59	28.9
20386083	2011	5	27	13090	0.62	28.92
20386083	2011	5	27	13100	0.6	28.96
20386083	2011	5	27	13110	0.49	29
20386083	2011	5	27	13120	0.48	29.02
20386083	2011	5	27	13130	0.46	29.06
20386083	2011	5	27	13140	0.47	29.06
20640423	2011	5	30	0	1.08	28.67
20640423	2011	5	30	10	1.69	28.67
20640423	2011	5	30	20	2.25	28.66
20640423	2011	5	30	30	2.37	28.66
20640423	2011	5	30	40	2.66	28.66
20640423	2011	5	30	50	2.78	28.65
20640423	2011	5	30	60	2.62	28.66
20640423	2011	5	30	70	2.96	28.66
20640423	2011	5	30	80	3.58	28.65
20640423	2011	5	30	90	4.51	28.64
20640423	2011	5	30	100	6.37	28.63
20640423	2011	5	30	110	7.81	28.6
20640423	2011	5	30	120	9.07	28.6
20640423	2011	5	30	130	9.75	28.59
20640423	2011	5	30	140	9.37	28.59
20640423	2011	5	30	150	8.85	28.6
20640423	2011	5	30	160	8.53	28.59
20640423	2011	5	30	170	9.03	28.59
20640423	2011	5	30	180	8.79	28.58
20640423	2011	5	30	190	8.67	28.59
20640423	2011	5	30	200	8.62	28.58
20640423	2011	5	30	210	8.77	28.59
20640423	2011	5	30	220	8.81	28.59
20640423	2011	5	30	230	8.56	28.58
20640423	2011	5	30	240	8.94	28.57
20640423	2011	5	30	250	8.86	28.58
20640423	2011	5	30	260	8.97	28.58
20640423	2011	5	30	270	8.72	28.57
20640423	2011	5	30	280	9.04	28.57
20640423	2011	5	30	290	11.25	28.55
20640423	2011	5	30	300	13.13	28.54
20640423	2011	5	30	310	14.89	28.52
20640423	2011	5	30	320	16.46	28.51
20640423	2011	5	30	330	17.81	28.5
20640423	2011	5	30	340	18.76	28.49
20640423	2011	5	30	350	19.56	28.48
20640423	2011	5	30	360	21.17	28.46
20640423	2011	5	30	370	23.56	28.44
20640423	2011	5	30	380	25.39	28.42

20640423	2011	5	30	390	27.85	28.41
20640423	2011	5	30	400	30.2	28.39
20640423	2011	5	30	410	31.82	28.37
20640423	2011	5	30	420	33.29	28.35
20640423	2011	5	30	430	34.78	28.34
20640423	2011	5	30	440	36.23	28.33
20640423	2011	5	30	450	38.96	28.31
20640423	2011	5	30	460	42.09	28.28
20640423	2011	5	30	470	45.53	28.26
20640423	2011	5	30	480	48.8	28.23
20640423	2011	5	30	490	51.55	28.21
20640423	2011	5	30	500	53.83	28.2
20640423	2011	5	30	510	55.94	28.18
20640423	2011	5	30	520	57.85	28.14
20640423	2011	5	30	530	60.38	28.08
20640423	2011	5	30	540	63.16	27.98
20640423	2011	5	30	550	65.76	27.71
20640423	2011	5	30	560	67.81	27.56
20640423	2011	5	30	570	69.09	27.45
20640423	2011	5	30	580	69.82	27.38
20640423	2011	5	30	590	71.37	27.23
20640423	2011	5	30	600	73.6	27.02
20640423	2011	5	30	610	75.23	26.92
20640423	2011	5	30	620	77.24	26.81
20640423	2011	5	30	630	80.34	26.6
20640423	2011	5	30	640	82.41	26.49
20640423	2011	5	30	650	83.4	26.42
20640423	2011	5	30	660	85.01	26.35
20640423	2011	5	30	670	86.85	26.23
20640423	2011	5	30	680	88.39	26.19
20640423	2011	5	30	690	89.05	26.12
20640423	2011	5	30	700	89.57	26.05
20640423	2011	5	30	710	90.07	25.96
20640423	2011	5	30	720	90.39	25.92
20640423	2011	5	30	730	90.74	25.86
20640423	2011	5	30	740	91.37	25.79
20640423	2011	5	30	750	91.73	25.68
20640423	2011	5	30	760	91.92	25.61
20640423	2011	5	30	770	92.22	25.54
20640423	2011	5	30	780	93.04	25.48
20640423	2011	5	30	790	94.03	25.42
20640423	2011	5	30	800	94.14	25.35
20640423	2011	5	30	810	93.86	25.28
20640423	2011	5	30	820	94.06	25.22
20640423	2011	5	30	830	94.58	25.14
20640423	2011	5	30	840	95.18	25.08
20640423	2011	5	30	850	95.59	25.02

20640423	2011	5	30	860	95.7	24.98
20640423	2011	5	30	870	95.56	24.94
20640423	2011	5	30	880	95.74	24.89
20640423	2011	5	30	890	97.07	24.8
20640423	2011	5	30	900	98.05	24.76
20640423	2011	5	30	910	99.37	24.72
20640423	2011	5	30	920	100.13	24.67
20640423	2011	5	30	930	100.19	24.63
20640423	2011	5	30	940	99.95	24.6
20640423	2011	5	30	950	99.57	24.57
20640423	2011	5	30	960	99.4	24.54
20640423	2011	5	30	970	99.74	24.5
20640423	2011	5	30	980	99.8	24.47
20640423	2011	5	30	990	99.47	24.43
20640423	2011	5	30	1000	98.3	24.4
20640423	2011	5	30	1010	96.51	24.38
20640423	2011	5	30	1020	95.27	24.36
20640423	2011	5	30	1030	94.55	24.34
20640423	2011	5	30	1040	94.52	24.32
20640423	2011	5	30	1050	94.39	24.29
20640423	2011	5	30	1060	93.36	24.27
20640423	2011	5	30	1070	93.03	24.26
20640423	2011	5	30	1080	93.82	24.23
20640423	2011	5	30	1090	94.39	24.21
20640423	2011	5	30	1100	94.22	24.2
20640423	2011	5	30	1110	93.04	24.21
20640423	2011	5	30	1120	91.76	24.21
20640423	2011	5	30	1130	90.71	24.2
20640423	2011	5	30	1140	90.01	24.19
20640423	2011	5	30	1150	90.82	24.17
20640423	2011	5	30	1160	91.5	24.17
20640423	2011	5	30	1170	91.55	24.17
20640423	2011	5	30	1180	91.79	24.16
20640423	2011	5	30	1190	92.45	24.16
20640423	2011	5	30	1200	92.5	24.15
20640423	2011	5	30	1210	92.44	24.15
20640423	2011	5	30	1220	91.47	24.15
20640423	2011	5	30	1230	90.73	24.14
20640423	2011	5	30	1240	89.89	24.15
20640423	2011	5	30	1250	89.05	24.15
20640423	2011	5	30	1260	89.32	24.15
20640423	2011	5	30	1270	89.86	24.14
20640423	2011	5	30	1280	89.87	24.15
20640423	2011	5	30	1290	89.32	24.15
20640423	2011	5	30	1300	88.49	24.16
20640423	2011	5	30	1310	88.19	24.17
20640423	2011	5	30	1320	88.73	24.17

20640423	2011	5	30	1330	87.97	24.19
20640423	2011	5	30	1340	87.26	24.2
20640423	2011	5	30	1350	86.13	24.21
20640423	2011	5	30	1360	85.73	24.22
20640423	2011	5	30	1370	86.43	24.23
20640423	2011	5	30	1380	86.39	24.25
20640423	2011	5	30	1390	86.2	24.26
20640423	2011	5	30	1400	86.53	24.28
20640423	2011	5	30	1410	87.25	24.27
20640423	2011	5	30	1420	87.3	24.28
20640423	2011	5	30	1430	87.35	24.3
20640423	2011	5	30	1440	87.77	24.3
20640423	2011	5	30	1450	88.55	24.31
20640423	2011	5	30	1460	89.18	24.31
20640423	2011	5	30	1470	89.76	24.32
20640423	2011	5	30	1480	90.38	24.32
20640423	2011	5	30	1490	91.59	24.32
20640423	2011	5	30	1500	92.63	24.3
20640423	2011	5	30	1510	94.07	24.28
20640423	2011	5	30	1520	95.67	24.27
20640423	2011	5	30	1530	96.5	24.25
20640423	2011	5	30	1540	97.21	24.24
20640423	2011	5	30	1550	97.46	24.22
20640423	2011	5	30	1560	97.73	24.21
20640423	2011	5	30	1570	98.27	24.2
20640423	2011	5	30	1580	98.72	24.17
20640423	2011	5	30	1590	98.82	24.14
20640423	2011	5	30	1600	98.66	24.12
20640423	2011	5	30	1610	98.96	24.1
20640423	2011	5	30	1620	99.55	24.07
20640423	2011	5	30	1630	100.25	24.05
20640423	2011	5	30	1640	100.76	24.03
20640423	2011	5	30	1650	100.91	24.01
20640423	2011	5	30	1660	101	23.98
20640423	2011	5	30	1670	101.28	23.97
20640423	2011	5	30	1680	101.33	23.96
20640423	2011	5	30	1690	101.4	23.93
20640423	2011	5	30	1700	101.88	23.92
20640423	2011	5	30	1710	102.44	23.91
20640423	2011	5	30	1720	102.64	23.89
20640423	2011	5	30	1730	102.63	23.87
20640423	2011	5	30	1740	102.8	23.85
20640423	2011	5	30	1750	102.89	23.85
20640423	2011	5	30	1760	102.83	23.83
20640423	2011	5	30	1770	102.77	23.82
20640423	2011	5	30	1780	103.07	23.81
20640423	2011	5	30	1790	102.93	23.8

20640423	2011	5	30	1800	103.02	23.79
20640423	2011	5	30	1810	102.84	23.78
20640423	2011	5	30	1820	103.06	23.78
20640423	2011	5	30	1830	102.91	23.77
20640423	2011	5	30	1840	102.92	23.76
20640423	2011	5	30	1850	102.82	23.75
20640423	2011	5	30	1860	102.99	23.75
20640423	2011	5	30	1870	102.88	23.74
20640423	2011	5	30	1880	102.91	23.73
20640423	2011	5	30	1890	102.48	23.73
20640423	2011	5	30	1900	102.81	23.71
20640423	2011	5	30	1910	102.81	23.71
20640423	2011	5	30	1920	102.81	23.71
20640423	2011	5	30	1930	102.78	23.7
20640423	2011	5	30	1940	102.67	23.69
20640423	2011	5	30	1950	102.68	23.68
20640423	2011	5	30	1960	102.37	23.68
20640423	2011	5	30	1970	102.08	23.68
20640423	2011	5	30	1980	101.95	23.68
20640423	2011	5	30	1990	102.27	23.67
20640423	2011	5	30	2000	102.89	23.66
20640423	2011	5	30	2010	102.94	23.66
20640423	2011	5	30	2020	102.84	23.66
20640423	2011	5	30	2030	103.07	23.66
20640423	2011	5	30	2040	103.66	23.66
20640423	2011	5	30	2050	104.27	23.65
20640423	2011	5	30	2060	104.66	23.64
20640423	2011	5	30	2070	105.02	23.64
20640423	2011	5	30	2080	104.89	23.64
20640423	2011	5	30	2090	105.06	23.63
20640423	2011	5	30	2100	104.95	23.64
20640423	2011	5	30	2110	105.38	23.63
20640423	2011	5	30	2120	105.74	23.62
20640423	2011	5	30	2130	105.67	23.62
20640423	2011	5	30	2140	105.6	23.62
20640423	2011	5	30	2150	106.08	23.61
20640423	2011	5	30	2160	106.16	23.61
20640423	2011	5	30	2170	106.06	23.61
20640423	2011	5	30	2180	105.71	23.61
20640423	2011	5	30	2190	105.5	23.6
20640423	2011	5	30	2200	105.63	23.6
20640423	2011	5	30	2210	105.5	23.6
20640423	2011	5	30	2220	105.25	23.6
20640423	2011	5	30	2230	104.96	23.6
20640423	2011	5	30	2240	104.5	23.6
20640423	2011	5	30	2250	104.79	23.6
20640423	2011	5	30	2260	105.09	23.59

20640423	2011	5	30	2270	105.36	23.59
20640423	2011	5	30	2280	105.35	23.58
20640423	2011	5	30	2290	105.09	23.59
20640423	2011	5	30	2300	104.91	23.59
20640423	2011	5	30	2310	105.65	23.59
20640423	2011	5	30	2320	106.26	23.57
20640423	2011	5	30	2330	106.79	23.58
20640423	2011	5	30	2340	106.95	23.57
20640423	2011	5	30	2350	106.64	23.58
20640423	2011	5	30	2360	106.85	23.57
20640423	2011	5	30	2370	106.73	23.58
20640423	2011	5	30	2380	106.35	23.57
20640423	2011	5	30	2390	106.55	23.57
20640423	2011	5	30	2400	106.57	23.57
20640423	2011	5	30	2410	106.74	23.57
20640423	2011	5	30	2420	107.5	23.57
20640423	2011	5	30	2430	107.99	23.57
20640423	2011	5	30	2440	108.21	23.57
20640423	2011	5	30	2450	108.04	23.57
20640423	2011	5	30	2460	108.53	23.57
20640423	2011	5	30	2470	109.29	23.57
20640423	2011	5	30	2480	109.97	23.57
20640423	2011	5	30	2490	111.05	23.56
20640423	2011	5	30	2500	111.67	23.55
20640423	2011	5	30	2510	112.17	23.55
20640423	2011	5	30	2520	112.19	23.55
20640423	2011	5	30	2530	111.87	23.56
20640423	2011	5	30	2540	111.59	23.55
20640423	2011	5	30	2550	111.46	23.55
20640423	2011	5	30	2560	111.25	23.56
20640423	2011	5	30	2570	111.37	23.54
20640423	2011	5	30	2580	111.15	23.54
20640423	2011	5	30	2590	110.94	23.53
20640423	2011	5	30	2600	110.47	23.53
20640423	2011	5	30	2610	110.9	23.52
20640423	2011	5	30	2620	111.34	23.51
20640423	2011	5	30	2630	111.24	23.5
20640423	2011	5	30	2640	111.22	23.5
20640423	2011	5	30	2650	111.26	23.48
20640423	2011	5	30	2660	111.31	23.49
20640423	2011	5	30	2670	111.15	23.48
20640423	2011	5	30	2680	111.06	23.48
20640423	2011	5	30	2690	111.77	23.47
20640423	2011	5	30	2700	111.73	23.46
20640423	2011	5	30	2710	111.78	23.46
20640423	2011	5	30	2720	111.86	23.45
20640423	2011	5	30	2730	111.72	23.44

20640423	2011	5	30	2740	111.71	23.43
20640423	2011	5	30	2750	111.94	23.42
20640423	2011	5	30	2760	112.18	23.42
20640423	2011	5	30	2770	111.9	23.41
20640423	2011	5	30	2780	111.43	23.41
20640423	2011	5	30	2790	111.05	23.41
20640423	2011	5	30	2800	111	23.41
20640423	2011	5	30	2810	110.98	23.4
20640423	2011	5	30	2820	110.74	23.41
20640423	2011	5	30	2830	110.74	23.4
20640423	2011	5	30	2840	110.74	23.4
20640423	2011	5	30	2850	110.43	23.4
20640423	2011	5	30	2860	109.9	23.4
20640423	2011	5	30	2870	109.35	23.41
20640423	2011	5	30	2880	109	23.41
20640423	2011	5	30	2890	108.38	23.41
20640423	2011	5	30	2900	108.26	23.41
20640423	2011	5	30	2910	108.22	23.42
20640423	2011	5	30	2920	108.05	23.42
20640423	2011	5	30	2930	107.66	23.42
20640423	2011	5	30	2940	107.25	23.43
20640423	2011	5	30	2950	107.4	23.44
20640423	2011	5	30	2960	107.61	23.44
20640423	2011	5	30	2970	107.61	23.44
20640423	2011	5	30	2980	107.54	23.45
20640423	2011	5	30	2990	107.99	23.46
20640423	2011	5	30	3000	108.25	23.46
20640423	2011	5	30	3010	108.15	23.47
20640423	2011	5	30	3020	109.08	23.46
20640423	2011	5	30	3030	109.31	23.47
20640423	2011	5	30	3040	109.76	23.47
20640423	2011	5	30	3050	110.37	23.48
20640423	2011	5	30	3060	110.55	23.48
20640423	2011	5	30	3070	110.73	23.48
20640423	2011	5	30	3080	111.8	23.49
20640423	2011	5	30	3090	112.27	23.48
20640423	2011	5	30	3100	112.29	23.48
20640423	2011	5	30	3110	112.61	23.48
20640423	2011	5	30	3120	113.3	23.46
20640423	2011	5	30	3130	113.69	23.46
20640423	2011	5	30	3140	113.64	23.46
20640423	2011	5	30	3150	113.34	23.45
20640423	2011	5	30	3160	113.21	23.45
20640423	2011	5	30	3170	113.73	23.42
20640423	2011	5	30	3180	113.5	23.41
20640423	2011	5	30	3190	113.19	23.4
20640423	2011	5	30	3200	113.02	23.39

20640423	2011	5	30	3210	113.16	23.39
20640423	2011	5	30	3220	113.26	23.38
20640423	2011	5	30	3230	113.07	23.37
20640423	2011	5	30	3240	113.31	23.37
20640423	2011	5	30	3250	112.87	23.36
20640423	2011	5	30	3260	112.31	23.36
20640423	2011	5	30	3270	112.34	23.35
20640423	2011	5	30	3280	111.28	23.35
20640423	2011	5	30	3290	109.34	23.36
20640423	2011	5	30	3300	107.23	23.35
20640423	2011	5	30	3310	104.52	23.37
20640423	2011	5	30	3320	102.86	23.39
20640423	2011	5	30	3330	103.48	23.41
20640423	2011	5	30	3340	103.88	23.41
20640423	2011	5	30	3350	104.31	23.42
20640423	2011	5	30	3360	104.49	23.42
20640423	2011	5	30	3370	104.45	23.44
20640423	2011	5	30	3380	103.36	23.45
20640423	2011	5	30	3390	101.37	23.46
20640423	2011	5	30	3400	99.42	23.48
20640423	2011	5	30	3410	97.1	23.5
20640423	2011	5	30	3420	95.48	23.52
20640423	2011	5	30	3430	93.55	23.53
20640423	2011	5	30	3440	92.03	23.55
20640423	2011	5	30	3450	90.54	23.58
20640423	2011	5	30	3460	89.14	23.6
20640423	2011	5	30	3470	87.81	23.63
20640423	2011	5	30	3480	87.44	23.66
20640423	2011	5	30	3490	87.43	23.71
20640423	2011	5	30	3500	87.46	23.75
20640423	2011	5	30	3510	87.5	23.76
20640423	2011	5	30	3520	87.38	23.8
20640423	2011	5	30	3530	87.39	23.82
20640423	2011	5	30	3540	87.43	23.85
20640423	2011	5	30	3550	87.44	23.88
20640423	2011	5	30	3560	87.49	23.9
20640423	2011	5	30	3570	87.21	23.92
20640423	2011	5	30	3580	87.06	23.95
20640423	2011	5	30	3590	87.09	23.97
20640423	2011	5	30	3600	87.25	23.99
20640423	2011	5	30	3610	87.32	24
20640423	2011	5	30	3620	87.23	24.03
20640423	2011	5	30	3630	87.47	24.05
20640423	2011	5	30	3640	87.41	24.07
20640423	2011	5	30	3650	87.42	24.09
20640423	2011	5	30	3660	87.38	24.1
20640423	2011	5	30	3670	87.44	24.12

20640423	2011	5	30	3680	86.91	24.13
20640423	2011	5	30	3690	86.7	24.14
20640423	2011	5	30	3700	86.62	24.16
20640423	2011	5	30	3710	86.47	24.17
20640423	2011	5	30	3720	85.54	24.18
20640423	2011	5	30	3730	85.35	24.2
20640423	2011	5	30	3740	85.51	24.21
20640423	2011	5	30	3750	85.68	24.21
20640423	2011	5	30	3760	85.89	24.23
20640423	2011	5	30	3770	85.92	24.24
20640423	2011	5	30	3780	85.76	24.25
20640423	2011	5	30	3790	85.7	24.25
20640423	2011	5	30	3800	85.72	24.26
20640423	2011	5	30	3810	85.72	24.28
20640423	2011	5	30	3820	85.81	24.28
20640423	2011	5	30	3830	85.88	24.28
20640423	2011	5	30	3840	85.94	24.29
20640423	2011	5	30	3850	85.9	24.3
20640423	2011	5	30	3860	85.7	24.31
20640423	2011	5	30	3870	85.95	24.31
20640423	2011	5	30	3880	86.02	24.32
20640423	2011	5	30	3890	85.67	24.32
20640423	2011	5	30	3900	85.79	24.33
20640423	2011	5	30	3910	85.76	24.33
20640423	2011	5	30	3920	85.12	24.34
20640423	2011	5	30	3930	84.24	24.35
20640423	2011	5	30	3940	82.4	24.36
20640423	2011	5	30	3950	80.97	24.37
20640423	2011	5	30	3960	81.01	24.39
20640423	2011	5	30	3970	81.35	24.4
20640423	2011	5	30	3980	81.08	24.41
20640423	2011	5	30	3990	80.16	24.41
20640423	2011	5	30	4000	79.93	24.42
20640423	2011	5	30	4010	79.98	24.42
20640423	2011	5	30	4020	79.87	24.44
20640423	2011	5	30	4030	78.95	24.45
20640423	2011	5	30	4040	78.33	24.46
20640423	2011	5	30	4050	77.45	24.46
20640423	2011	5	30	4060	77.36	24.47
20640423	2011	5	30	4070	77.52	24.48
20640423	2011	5	30	4080	77.91	24.49
20640423	2011	5	30	4090	78.11	24.49
20640423	2011	5	30	4100	78.3	24.5
20640423	2011	5	30	4110	78.59	24.5
20640423	2011	5	30	4120	78.63	24.51
20640423	2011	5	30	4130	78.65	24.52
20640423	2011	5	30	4140	78.69	24.52

20640423	2011	5	30	4150	78.8	24.52
20640423	2011	5	30	4160	78.8	24.53
20640423	2011	5	30	4170	78.88	24.53
20640423	2011	5	30	4180	78.78	24.53
20640423	2011	5	30	4190	78.67	24.53
20640423	2011	5	30	4200	78.59	24.55
20640423	2011	5	30	4210	78.55	24.54
20640423	2011	5	30	4220	78.44	24.55
20640423	2011	5	30	4230	78.59	24.55
20640423	2011	5	30	4240	78.72	24.56
20640423	2011	5	30	4250	78.56	24.56
20640423	2011	5	30	4260	78.34	24.57
20640423	2011	5	30	4270	78.04	24.57
20640423	2011	5	30	4280	78.02	24.57
20640423	2011	5	30	4290	78.05	24.57
20640423	2011	5	30	4300	78.16	24.59
20640423	2011	5	30	4310	78.36	24.58
20640423	2011	5	30	4320	78.24	24.59
20640423	2011	5	30	4330	77.97	24.59
20640423	2011	5	30	4340	77.57	24.6
20640423	2011	5	30	4350	77.43	24.6
20640423	2011	5	30	4360	77.41	24.6
20640423	2011	5	30	4370	77.38	24.62
20640423	2011	5	30	4380	77.51	24.63
20640423	2011	5	30	4390	77.36	24.63
20640423	2011	5	30	4400	77.21	24.64
20640423	2011	5	30	4410	77.19	24.64
20640423	2011	5	30	4420	77.4	24.64
20640423	2011	5	30	4430	77.84	24.64
20640423	2011	5	30	4440	77.65	24.64
20640423	2011	5	30	4450	77.57	24.64
20640423	2011	5	30	4460	77.34	24.64
20640423	2011	5	30	4470	77.23	24.65
20640423	2011	5	30	4480	77.39	24.65
20640423	2011	5	30	4490	77.43	24.65
20640423	2011	5	30	4500	77.33	24.65
20640423	2011	5	30	4510	76.97	24.66
20640423	2011	5	30	4520	76.98	24.67
20640423	2011	5	30	4530	77.1	24.67
20640423	2011	5	30	4540	77.07	24.67
20640423	2011	5	30	4550	77.33	24.67
20640423	2011	5	30	4560	77.67	24.67
20640423	2011	5	30	4570	77.09	24.67
20640423	2011	5	30	4580	77.32	24.67
20640423	2011	5	30	4590	76.98	24.67
20640423	2011	5	30	4600	76.95	24.67
20640423	2011	5	30	4610	77.1	24.67

20640423	2011	5	30	4620	77.54	24.67
20640423	2011	5	30	4630	77.91	24.67
20640423	2011	5	30	4640	78.05	24.67
20640423	2011	5	30	4650	77.89	24.67
20640423	2011	5	30	4660	77.72	24.67
20640423	2011	5	30	4670	77.64	24.67
20640423	2011	5	30	4680	77.7	24.67
20640423	2011	5	30	4690	77.83	24.67
20640423	2011	5	30	4700	78.16	24.67
20640423	2011	5	30	4710	78.91	24.66
20640423	2011	5	30	4720	78.88	24.66
20640423	2011	5	30	4730	78.82	24.66
20640423	2011	5	30	4740	78.87	24.66
20640423	2011	5	30	4750	78.84	24.66
20640423	2011	5	30	4760	78.76	24.66
20640423	2011	5	30	4770	78.83	24.66
20640423	2011	5	30	4780	79.04	24.66
20640423	2011	5	30	4790	79.53	24.65
20640423	2011	5	30	4800	80.15	24.64
20640423	2011	5	30	4810	80.96	24.64
20640423	2011	5	30	4820	81.21	24.64
20640423	2011	5	30	4830	81.55	24.64
20640423	2011	5	30	4840	81.59	24.64
20640423	2011	5	30	4850	81.53	24.64
20640423	2011	5	30	4860	81.54	24.64
20640423	2011	5	30	4870	81.69	24.63
20640423	2011	5	30	4880	81.76	24.63
20640423	2011	5	30	4890	81.69	24.63
20640423	2011	5	30	4900	81.65	24.63
20640423	2011	5	30	4910	81.74	24.63
20640423	2011	5	30	4920	82.34	24.63
20640423	2011	5	30	4930	83.11	24.61
20640423	2011	5	30	4940	83.57	24.62
20640423	2011	5	30	4950	84.09	24.6
20640423	2011	5	30	4960	84.5	24.6
20640423	2011	5	30	4970	84.71	24.6
20640423	2011	5	30	4980	84.85	24.6
20640423	2011	5	30	4990	84.99	24.6
20640423	2011	5	30	5000	85.29	24.6
20640423	2011	5	30	5010	85.26	24.6
20640423	2011	5	30	5020	85.15	24.6
20640423	2011	5	30	5030	85.29	24.59
20640423	2011	5	30	5040	85.83	24.59
20640423	2011	5	30	5050	85.8	24.58
20640423	2011	5	30	5060	85.74	24.58
20640423	2011	5	30	5070	86.13	24.58
20640423	2011	5	30	5080	86.2	24.57

20640423	2011	5	30	5090	86.13	24.57
20640423	2011	5	30	5100	85.42	24.58
20640423	2011	5	30	5110	85.07	24.57
20640423	2011	5	30	5120	85.15	24.57
20640423	2011	5	30	5130	85.16	24.57
20640423	2011	5	30	5140	84.7	24.57
20640423	2011	5	30	5150	84.96	24.57
20640423	2011	5	30	5160	85.24	24.57
20640423	2011	5	30	5170	85.35	24.57
20640423	2011	5	30	5180	85.75	24.57
20640423	2011	5	30	5190	85.83	24.56
20640423	2011	5	30	5200	85.95	24.55
20640423	2011	5	30	5210	86.07	24.55
20640423	2011	5	30	5220	85.81	24.55
20640423	2011	5	30	5230	85.78	24.53
20640423	2011	5	30	5240	85.74	24.53
20640423	2011	5	30	5250	85.98	24.53
20640423	2011	5	30	5260	86.12	24.53
20640423	2011	5	30	5270	86.11	24.53
20640423	2011	5	30	5280	86.12	24.53
20640423	2011	5	30	5290	86.19	24.52
20640423	2011	5	30	5300	86.39	24.53
20640423	2011	5	30	5310	86.68	24.52
20640423	2011	5	30	5320	87.02	24.53
20640423	2011	5	30	5330	87.33	24.51
20640423	2011	5	30	5340	87.28	24.51
20640423	2011	5	30	5350	87.32	24.5
20640423	2011	5	30	5360	87.45	24.5
20640423	2011	5	30	5370	87.5	24.5
20640423	2011	5	30	5380	87.46	24.5
20640423	2011	5	30	5390	87.35	24.51
20640423	2011	5	30	5400	87.23	24.5
20640423	2011	5	30	5410	87.15	24.5
20640423	2011	5	30	5420	86.74	24.5
20640423	2011	5	30	5430	86.44	24.5
20640423	2011	5	30	5440	87.21	24.49
20640423	2011	5	30	5450	87.53	24.49
20640423	2011	5	30	5460	87.61	24.49
20640423	2011	5	30	5470	87.34	24.49
20640423	2011	5	30	5480	86.42	24.5
20640423	2011	5	30	5490	86.71	24.49
20640423	2011	5	30	5500	87.22	24.48
20640423	2011	5	30	5510	87.2	24.49
20640423	2011	5	30	5520	87.34	24.48
20640423	2011	5	30	5530	87.59	24.48
20640423	2011	5	30	5540	87.22	24.48
20640423	2011	5	30	5550	87.44	24.48

20640423	2011	5	30	5560	87.49	24.48
20640423	2011	5	30	5570	87.49	24.48
20640423	2011	5	30	5580	87.37	24.48
20640423	2011	5	30	5590	87.37	24.49
20640423	2011	5	30	5600	87.28	24.48
20640423	2011	5	30	5610	87.2	24.49
20640423	2011	5	30	5620	87.08	24.49
20640423	2011	5	30	5630	87.13	24.49
20640423	2011	5	30	5640	87.08	24.49
20640423	2011	5	30	5650	86.92	24.48
20640423	2011	5	30	5660	86.81	24.49
20640423	2011	5	30	5670	87.13	24.48
20640423	2011	5	30	5680	87.14	24.48
20640423	2011	5	30	5690	86.77	24.49
20640423	2011	5	30	5700	86.89	24.49
20640423	2011	5	30	5710	87.12	24.48
20640423	2011	5	30	5720	87.12	24.48
20640423	2011	5	30	5730	87.04	24.49
20640423	2011	5	30	5740	86.86	24.48
20640423	2011	5	30	5750	87	24.49
20640423	2011	5	30	5760	86.97	24.5
20640423	2011	5	30	5770	86.62	24.48
20640423	2011	5	30	5780	86.41	24.49
20640423	2011	5	30	5790	86.92	24.49
20640423	2011	5	30	5800	87.26	24.49
20640423	2011	5	30	5810	86.49	24.49
20640423	2011	5	30	5820	86.86	24.49
20640423	2011	5	30	5830	87.39	24.49
20640423	2011	5	30	5840	87.8	24.49
20640423	2011	5	30	5850	88.43	24.49
20640423	2011	5	30	5860	89.64	24.47
20640423	2011	5	30	5870	90.2	24.48
20640423	2011	5	30	5880	90.47	24.47
20640423	2011	5	30	5890	91.12	24.46
20640423	2011	5	30	5900	91.83	24.46
20640423	2011	5	30	5910	92.18	24.46
20640423	2011	5	30	5920	92.37	24.45
20640423	2011	5	30	5930	92.78	24.45
20640423	2011	5	30	5940	93.1	24.44
20640423	2011	5	30	5950	93.21	24.43
20640423	2011	5	30	5960	93.2	24.44
20640423	2011	5	30	5970	93.14	24.44
20640423	2011	5	30	5980	92.85	24.43
20640423	2011	5	30	5990	92.49	24.42
20640423	2011	5	30	6000	91.81	24.43
20640423	2011	5	30	6010	91.48	24.42
20640423	2011	5	30	6020	91.38	24.42

20640423	2011	5	30	6030	91.82	24.42
20640423	2011	5	30	6040	92.88	24.41
20640423	2011	5	30	6050	93.1	24.4
20640423	2011	5	30	6060	92.87	24.39
20640423	2011	5	30	6070	93.37	24.38
20640423	2011	5	30	6080	93.94	24.35
20640423	2011	5	30	6090	93.87	24.35
20640423	2011	5	30	6100	93.84	24.34
20640423	2011	5	30	6110	93.77	24.34
20640423	2011	5	30	6120	93.14	24.32
20640423	2011	5	30	6130	92.24	24.32
20640423	2011	5	30	6140	91.91	24.31
20640423	2011	5	30	6150	91.75	24.3
20640423	2011	5	30	6160	91.54	24.3
20640423	2011	5	30	6170	91.35	24.29
20640423	2011	5	30	6180	90.48	24.29
20640423	2011	5	30	6190	91.2	24.28
20640423	2011	5	30	6200	91.72	24.28
20640423	2011	5	30	6210	91.72	24.28
20640423	2011	5	30	6220	91.41	24.28
20640423	2011	5	30	6230	91.38	24.27
20640423	2011	5	30	6240	91.81	24.27
20640423	2011	5	30	6250	92.04	24.27
20640423	2011	5	30	6260	92.2	24.27
20640423	2011	5	30	6270	93.14	24.25
20640423	2011	5	30	6280	92.85	24.25
20640423	2011	5	30	6290	91.54	24.25
20640423	2011	5	30	6300	91.07	24.25
20640423	2011	5	30	6310	90.33	24.25
20640423	2011	5	30	6320	90.06	24.25
20640423	2011	5	30	6330	90.64	24.25
20640423	2011	5	30	6340	91.45	24.25
20640423	2011	5	30	6350	91.72	24.25
20640423	2011	5	30	6360	91.7	24.25
20640423	2011	5	30	6370	91.61	24.24
20640423	2011	5	30	6380	91.83	24.24
20640423	2011	5	30	6390	91.8	24.23
20640423	2011	5	30	6400	91.85	24.22
20640423	2011	5	30	6410	92.01	24.22
20640423	2011	5	30	6420	91.94	24.21
20640423	2011	5	30	6430	92.04	24.21
20640423	2011	5	30	6440	92.02	24.22
20640423	2011	5	30	6450	91.98	24.21
20640423	2011	5	30	6460	92.01	24.21
20640423	2011	5	30	6470	91.94	24.19
20640423	2011	5	30	6480	91.93	24.18
20640423	2011	5	30	6490	91.86	24.18

20640423	2011	5	30	6500	91.64	24.17
20640423	2011	5	30	6510	91.52	24.17
20640423	2011	5	30	6520	91.52	24.17
20640423	2011	5	30	6530	91.98	24.17
20640423	2011	5	30	6540	92.33	24.16
20640423	2011	5	30	6550	92.24	24.15
20640423	2011	5	30	6560	91.76	24.15
20640423	2011	5	30	6570	91.5	24.14
20640423	2011	5	30	6580	91.25	24.14
20640423	2011	5	30	6590	90.96	24.14
20640423	2011	5	30	6600	90.68	24.14
20640423	2011	5	30	6610	90.59	24.14
20640423	2011	5	30	6620	91.31	24.14
20640423	2011	5	30	6630	91.47	24.14
20640423	2011	5	30	6640	91.59	24.14
20640423	2011	5	30	6650	91.88	24.14
20640423	2011	5	30	6660	92.79	24.14
20640423	2011	5	30	6670	93.09	24.13
20640423	2011	5	30	6680	93.11	24.13
20640423	2011	5	30	6690	93.03	24.13
20640423	2011	5	30	6700	93.01	24.11
20640423	2011	5	30	6710	92.78	24.11
20640423	2011	5	30	6720	92.59	24.1
20640423	2011	5	30	6730	92.74	24.11
20640423	2011	5	30	6740	92.95	24.1
20640423	2011	5	30	6750	93.03	24.1
20640423	2011	5	30	6760	92.36	24.09
20640423	2011	5	30	6770	92.1	24.09
20640423	2011	5	30	6780	92.25	24.09
20640423	2011	5	30	6790	92.72	24.07
20640423	2011	5	30	6800	93.61	24.07
20640423	2011	5	30	6810	93.53	24.06
20640423	2011	5	30	6820	93.5	24.06
20640423	2011	5	30	6830	93.55	24.06
20640423	2011	5	30	6840	93.62	24.05
20640423	2011	5	30	6850	93.75	24.04
20640423	2011	5	30	6860	93.39	24.05
20640423	2011	5	30	6870	92.84	24.05
20640423	2011	5	30	6880	92.7	24.04
20640423	2011	5	30	6890	92.73	24.03
20640423	2011	5	30	6900	93.05	24.03
20640423	2011	5	30	6910	92.79	24.03
20640423	2011	5	30	6920	92.64	24.03
20640423	2011	5	30	6930	92.89	24.02
20640423	2011	5	30	6940	92.92	24.03
20640423	2011	5	30	6950	93.48	24.02
20640423	2011	5	30	6960	93.83	24.03

20640423	2011	5	30	6970	94.15	24.01
20640423	2011	5	30	6980	95.4	24
20640423	2011	5	30	6990	96.38	24
20640423	2011	5	30	7000	96.63	24
20640423	2011	5	30	7010	96.77	24
20640423	2011	5	30	7020	96.72	24
20640423	2011	5	30	7030	96.64	24
20640423	2011	5	30	7040	96.76	23.99
20640423	2011	5	30	7050	96.55	23.99
20640423	2011	5	30	7060	96.25	23.99
20640423	2011	5	30	7070	96.29	23.99
20640423	2011	5	30	7080	96.23	23.98
20640423	2011	5	30	7090	96.11	23.98
20640423	2011	5	30	7100	96.3	23.98
20640423	2011	5	30	7110	96.38	23.98
20640423	2011	5	30	7120	96.55	23.97
20640423	2011	5	30	7130	96.13	23.98
20640423	2011	5	30	7140	96.11	23.97
20640423	2011	5	30	7150	96.29	23.97
20640423	2011	5	30	7160	96.52	23.97
20640423	2011	5	30	7170	96.72	23.96
20640423	2011	5	30	7180	96.94	23.96
20640423	2011	5	30	7190	96.89	23.96
20640423	2011	5	30	7200	97.39	23.96
20640423	2011	5	30	7210	97.67	23.94
20640423	2011	5	30	7220	97.25	23.94
20640423	2011	5	30	7230	96.78	23.94
20640423	2011	5	30	7240	96.47	23.93
20640423	2011	5	30	7250	96.79	23.92
20640423	2011	5	30	7260	96.91	23.92
20640423	2011	5	30	7270	96.75	23.92
20640423	2011	5	30	7280	96.64	23.92
20640423	2011	5	30	7290	96.37	23.92
20640423	2011	5	30	7300	96.13	23.9
20640423	2011	5	30	7310	95.88	23.91
20640423	2011	5	30	7320	95.55	23.91
20640423	2011	5	30	7330	96.02	23.91
20640423	2011	5	30	7340	95.7	23.91
20640423	2011	5	30	7350	95.39	23.92
20640423	2011	5	30	7360	94.88	23.92
20640423	2011	5	30	7370	94.29	23.92
20640423	2011	5	30	7380	93.88	23.92
20640423	2011	5	30	7390	93.81	23.92
20640423	2011	5	30	7400	93.71	23.93
20640423	2011	5	30	7410	93.64	23.92
20640423	2011	5	30	7420	93.62	23.93
20640423	2011	5	30	7430	94.82	23.93

20640423	2011	5	30	7440	95.63	23.93
20640423	2011	5	30	7450	97.22	23.92
20640423	2011	5	30	7460	98.4	23.92
20640423	2011	5	30	7470	98.66	23.9
20640423	2011	5	30	7480	99.73	23.89
20640423	2011	5	30	7490	101.24	23.88
20640423	2011	5	30	7500	103.56	23.86
20640423	2011	5	30	7510	106.5	23.85
20640423	2011	5	30	7520	108.55	23.82
20640423	2011	5	30	7530	109.99	23.81
20640423	2011	5	30	7540	111.03	23.81
20640423	2011	5	30	7550	112.41	23.79
20640423	2011	5	30	7560	114.51	23.75
20640423	2011	5	30	7570	115.45	23.71
20640423	2011	5	30	7580	116.97	23.62
20640423	2011	5	30	7590	119.35	23.5
20640423	2011	5	30	7600	122.11	23.43
20640423	2011	5	30	7610	123.82	23.41
20640423	2011	5	30	7620	126.17	23.32
20640423	2011	5	30	7630	127.55	23.1
20640423	2011	5	30	7640	129.29	22.92
20640423	2011	5	30	7650	130.56	22.83
20640423	2011	5	30	7660	131.13	22.8
20640423	2011	5	30	7670	131.3	22.72
20640423	2011	5	30	7680	131.36	22.64
20640423	2011	5	30	7690	131.68	22.56
20640423	2011	5	30	7700	131.68	22.46
20640423	2011	5	30	7710	131.68	22.38
20640423	2011	5	30	7720	131.59	22.31
20640423	2011	5	30	7730	131.59	22.23
20640423	2011	5	30	7740	131.59	22.14
20640423	2011	5	30	7750	132.5	21.99
20640423	2011	5	30	7760	134.07	21.87
20640423	2011	5	30	7770	135.87	21.82
20640423	2011	5	30	7780	138.2	21.74
20640423	2011	5	30	7790	140.4	21.67
20640423	2011	5	30	7800	142.44	21.58
20640423	2011	5	30	7810	144.03	21.47
20640423	2011	5	30	7820	145.98	21.28
20640423	2011	5	30	7830	147.15	21.22
20640423	2011	5	30	7840	147.89	21.14
20640423	2011	5	30	7850	148.96	21.04
20640423	2011	5	30	7860	149.92	20.96
20640423	2011	5	30	7870	151.06	20.84
20640423	2011	5	30	7880	153.12	20.68
20640423	2011	5	30	7890	154.76	20.62
20640423	2011	5	30	7900	156.18	20.42

20640423	2011	5	30	7910	156.81	20.35
20640423	2011	5	30	7920	157.59	20.23
20640423	2011	5	30	7930	159.05	20.14
20640423	2011	5	30	7940	159.41	20.07
20640423	2011	5	30	7950	160.32	20.01
20640423	2011	5	30	7960	160.36	19.9
20640423	2011	5	30	7970	160.59	19.81
20640423	2011	5	30	7980	160.9	19.74
20640423	2011	5	30	7990	161.17	19.64
20640423	2011	5	30	8000	162.01	19.57
20640423	2011	5	30	8010	162.3	19.5
20640423	2011	5	30	8020	162.24	19.43
20640423	2011	5	30	8030	162.39	19.35
20640423	2011	5	30	8040	163.63	19.24
20640423	2011	5	30	8050	164.7	19.14
20640423	2011	5	30	8060	165.13	19.11
20640423	2011	5	30	8070	165.7	19.07
20640423	2011	5	30	8080	166.24	19.03
20640423	2011	5	30	8090	166.63	18.96
20640423	2011	5	30	8100	166.86	18.91
20640423	2011	5	30	8110	166.72	18.86
20640423	2011	5	30	8120	166.39	18.81
20640423	2011	5	30	8130	165.92	18.76
20640423	2011	5	30	8140	165.38	18.71
20640423	2011	5	30	8150	164.75	18.68
20640423	2011	5	30	8160	163.91	18.65
20640423	2011	5	30	8170	163.19	18.61
20640423	2011	5	30	8180	162.57	18.59
20640423	2011	5	30	8190	161.97	18.56
20640423	2011	5	30	8200	162.82	18.52
20640423	2011	5	30	8210	163.93	18.47
20640423	2011	5	30	8220	164.09	18.43
20640423	2011	5	30	8230	163.97	18.41
20640423	2011	5	30	8240	163.78	18.38
20640423	2011	5	30	8250	162.92	18.35
20640423	2011	5	30	8260	162.38	18.32
20640423	2011	5	30	8270	162.23	18.29
20640423	2011	5	30	8280	162.18	18.26
20640423	2011	5	30	8290	162.23	18.24
20640423	2011	5	30	8300	162.77	18.2
20640423	2011	5	30	8310	162.82	18.17
20640423	2011	5	30	8320	163.19	18.15
20640423	2011	5	30	8330	163.49	18.14
20640423	2011	5	30	8340	163.96	18.13
20640423	2011	5	30	8350	164.34	18.11
20640423	2011	5	30	8360	164.53	18.1
20640423	2011	5	30	8370	165	18.08

20640423	2011	5	30	8380	165.45	18.07
20640423	2011	5	30	8390	166.01	18.06
20640423	2011	5	30	8400	166.18	18.03
20640423	2011	5	30	8410	166.12	18.02
20640423	2011	5	30	8420	166.25	18
20640423	2011	5	30	8430	166.27	17.99
20640423	2011	5	30	8440	166.14	17.96
20640423	2011	5	30	8450	165.89	17.95
20640423	2011	5	30	8460	165.79	17.93
20640423	2011	5	30	8470	165.97	17.92
20640423	2011	5	30	8480	166.57	17.88
20640423	2011	5	30	8490	166.53	17.88
20640423	2011	5	30	8500	166.83	17.87
20640423	2011	5	30	8510	165.89	17.85
20640423	2011	5	30	8520	164.86	17.85
20640423	2011	5	30	8530	164.19	17.84
20640423	2011	5	30	8540	163.75	17.82
20640423	2011	5	30	8550	164.33	17.82
20640423	2011	5	30	8560	165.92	17.8
20640423	2011	5	30	8570	166.88	17.79
20640423	2011	5	30	8580	166.9	17.78
20640423	2011	5	30	8590	166.04	17.78
20640423	2011	5	30	8600	165.67	17.78
20640423	2011	5	30	8610	165.62	17.77
20640423	2011	5	30	8620	165.67	17.77
20640423	2011	5	30	8630	165.18	17.76
20640423	2011	5	30	8640	164.88	17.75
20640423	2011	5	30	8650	164.66	17.75
20640423	2011	5	30	8660	164.44	17.74
20640423	2011	5	30	8670	164.11	17.73
20640423	2011	5	30	8680	163.92	17.73
20640423	2011	5	30	8690	163.94	17.72
20640423	2011	5	30	8700	163.94	17.71
20640423	2011	5	30	8710	163.89	17.71
20640423	2011	5	30	8720	163.36	17.71
20640423	2011	5	30	8730	161.51	17.71
20640423	2011	5	30	8740	159.41	17.71
20640423	2011	5	30	8750	157.75	17.71
20640423	2011	5	30	8760	156.73	17.72
20640423	2011	5	30	8770	157.03	17.75
20640423	2011	5	30	8780	156.56	17.77
20640423	2011	5	30	8790	156.64	17.78
20640423	2011	5	30	8800	156.56	17.79
20640423	2011	5	30	8810	156.9	17.81
20640423	2011	5	30	8820	156.61	17.84
20640423	2011	5	30	8830	156.67	17.85
20640423	2011	5	30	8840	156.62	17.86

20640423	2011	5	30	8850	156.4	17.89
20640423	2011	5	30	8860	156.11	17.89
20640423	2011	5	30	8870	155.7	17.91
20640423	2011	5	30	8880	156.16	17.92
20640423	2011	5	30	8890	157.18	17.95
20640423	2011	5	30	8900	157.16	17.96
20640423	2011	5	30	8910	157.27	17.99
20640423	2011	5	30	8920	157.72	18
20640423	2011	5	30	8930	157.25	18.01
20640423	2011	5	30	8940	156.35	18.03
20640423	2011	5	30	8950	154.64	18.04
20640423	2011	5	30	8960	153.47	18.07
20640423	2011	5	30	8970	152.92	18.07
20640423	2011	5	30	8980	153.43	18.09
20640423	2011	5	30	8990	154.68	18.1
20640423	2011	5	30	9000	155.2	18.11
20640423	2011	5	30	9010	155.15	18.13
20640423	2011	5	30	9020	155.29	18.14
20640423	2011	5	30	9030	155.65	18.14
20640423	2011	5	30	9040	155.59	18.15
20640423	2011	5	30	9050	155.47	18.15
20640423	2011	5	30	9060	155.13	18.17
20640423	2011	5	30	9070	155.77	18.17
20640423	2011	5	30	9080	156.09	18.17
20640423	2011	5	30	9090	156.14	18.17
20640423	2011	5	30	9100	156.1	18.18
20640423	2011	5	30	9110	155.85	18.19
20640423	2011	5	30	9120	154.55	18.21
20640423	2011	5	30	9130	152.68	18.21
20640423	2011	5	30	9140	151.15	18.23
20640423	2011	5	30	9150	150.05	18.24
20640423	2011	5	30	9160	149.46	18.26
20640423	2011	5	30	9170	149.15	18.29
20640423	2011	5	30	9180	148.91	18.34
20640423	2011	5	30	9190	148.91	18.38
20640423	2011	5	30	9200	148.6	18.42
20640423	2011	5	30	9210	146.84	18.43
20640423	2011	5	30	9220	144.71	18.48
20640423	2011	5	30	9230	143.17	18.51
20640423	2011	5	30	9240	142.45	18.55
20640423	2011	5	30	9250	141.93	18.6
20640423	2011	5	30	9260	140.93	18.67
20640423	2011	5	30	9270	139.38	18.71
20640423	2011	5	30	9280	139.02	18.77
20640423	2011	5	30	9290	138.89	18.82
20640423	2011	5	30	9300	137.22	18.87
20640423	2011	5	30	9310	135.71	18.91

20640423	2011	5	30	9320	133.81	18.94
20640423	2011	5	30	9330	132.38	19.01
20640423	2011	5	30	9340	131.49	19.1
20640423	2011	5	30	9350	129.75	19.17
20640423	2011	5	30	9360	128.48	19.22
20640423	2011	5	30	9370	127.61	19.28
20640423	2011	5	30	9380	126.86	19.35
20640423	2011	5	30	9390	125.55	19.43
20640423	2011	5	30	9400	123.9	19.5
20640423	2011	5	30	9410	122.9	19.55
20640423	2011	5	30	9420	122.98	19.64
20640423	2011	5	30	9430	123.23	19.78
20640423	2011	5	30	9440	122.66	19.86
20640423	2011	5	30	9450	121.82	19.96
20640423	2011	5	30	9460	121.43	20.03
20640423	2011	5	30	9470	120.74	20.15
20640423	2011	5	30	9480	119.5	20.23
20640423	2011	5	30	9490	117.72	20.29
20640423	2011	5	30	9500	116.68	20.38
20640423	2011	5	30	9510	115.42	20.5
20640423	2011	5	30	9520	114.1	20.57
20640423	2011	5	30	9530	112.78	20.64
20640423	2011	5	30	9540	111.04	20.79
20640423	2011	5	30	9550	109.11	20.9
20640423	2011	5	30	9560	107.74	21.11
20640423	2011	5	30	9570	105.83	21.22
20640423	2011	5	30	9580	104.58	21.34
20640423	2011	5	30	9590	104.05	21.46
20640423	2011	5	30	9600	103.72	21.57
20640423	2011	5	30	9610	103.59	21.67
20640423	2011	5	30	9620	102.94	21.78
20640423	2011	5	30	9630	101.97	21.86
20640423	2011	5	30	9640	99.82	21.92
20640423	2011	5	30	9650	97.66	22.04
20640423	2011	5	30	9660	95.69	22.14
20640423	2011	5	30	9670	93.8	22.22
20640423	2011	5	30	9680	92.39	22.32
20640423	2011	5	30	9690	92.24	22.38
20640423	2011	5	30	9700	92.58	22.45
20640423	2011	5	30	9710	93.08	22.53
20640423	2011	5	30	9720	93.62	22.59
20640423	2011	5	30	9730	93.99	22.66
20640423	2011	5	30	9740	94.29	22.73
20640423	2011	5	30	9750	93.66	22.85
20640423	2011	5	30	9760	92.36	22.94
20640423	2011	5	30	9770	91.97	23
20640423	2011	5	30	9780	92.06	23.04

20640423	2011	5	30	9790	92.4	23.07
20640423	2011	5	30	9800	92.29	23.14
20640423	2011	5	30	9810	90.72	23.2
20640423	2011	5	30	9820	88.8	23.27
20640423	2011	5	30	9830	86.45	23.33
20640423	2011	5	30	9840	85.29	23.39
20640423	2011	5	30	9850	84.21	23.45
20640423	2011	5	30	9860	83.74	23.51
20640423	2011	5	30	9870	83.69	23.6
20640423	2011	5	30	9880	82.41	23.68
20640423	2011	5	30	9890	80.39	23.7
20640423	2011	5	30	9900	78.68	23.74
20640423	2011	5	30	9910	76.2	23.78
20640423	2011	5	30	9920	73.93	23.83
20640423	2011	5	30	9930	73.06	23.86
20640423	2011	5	30	9940	71.84	23.89
20640423	2011	5	30	9950	69.99	23.93
20640423	2011	5	30	9960	68.98	23.97
20640423	2011	5	30	9970	67.17	24.01
20640423	2011	5	30	9980	67.19	24.08
20640423	2011	5	30	9990	67.81	24.14
20640423	2011	5	30	10000	68.09	24.17
20640423	2011	5	30	10010	68.11	24.18
20640423	2011	5	30	10020	68.02	24.22
20640423	2011	5	30	10030	67.66	24.24
20640423	2011	5	30	10040	67.28	24.26
20640423	2011	5	30	10050	67.23	24.28
20640423	2011	5	30	10060	66.17	24.32
20640423	2011	5	30	10070	66.29	24.35
20640423	2011	5	30	10080	66.51	24.36
20640423	2011	5	30	10090	66.81	24.38
20640423	2011	5	30	10100	67.09	24.41
20640423	2011	5	30	10110	67.59	24.42
20640423	2011	5	30	10120	67.99	24.46
20640423	2011	5	30	10130	68.34	24.46
20640423	2011	5	30	10140	68.64	24.48
20640423	2011	5	30	10150	68.94	24.49
20640423	2011	5	30	10160	69.24	24.5
20640423	2011	5	30	10170	69.6	24.52
20640423	2011	5	30	10180	69.94	24.53
20640423	2011	5	30	10190	69.93	24.54
20640423	2011	5	30	10200	69.47	24.56
20640423	2011	5	30	10210	69.22	24.57
20640423	2011	5	30	10220	69.28	24.57
20640423	2011	5	30	10230	69.4	24.57
20640423	2011	5	30	10240	69.39	24.58
20640423	2011	5	30	10250	69.2	24.59

20640423	2011	5	30	10260	68.95	24.6
20640423	2011	5	30	10270	68.61	24.61
20640423	2011	5	30	10280	68.38	24.62
20640423	2011	5	30	10290	68.34	24.63
20640423	2011	5	30	10300	68.32	24.64
20640423	2011	5	30	10310	67.91	24.64
20640423	2011	5	30	10320	66.94	24.67
20640423	2011	5	30	10330	66.35	24.67
20640423	2011	5	30	10340	66.12	24.67
20640423	2011	5	30	10350	65.88	24.68
20640423	2011	5	30	10360	65.54	24.7
20640423	2011	5	30	10370	65.42	24.72
20640423	2011	5	30	10380	65.56	24.72
20640423	2011	5	30	10390	65	24.74
20640423	2011	5	30	10400	64.96	24.75
20640423	2011	5	30	10410	65.2	24.76
20640423	2011	5	30	10420	65.59	24.77
20640423	2011	5	30	10430	66.08	24.78
20640423	2011	5	30	10440	65.8	24.79
20640423	2011	5	30	10450	65.56	24.79
20640423	2011	5	30	10460	65.38	24.81
20640423	2011	5	30	10470	64.93	24.82
20640423	2011	5	30	10480	63.69	24.83
20640423	2011	5	30	10490	62.8	24.84
20640423	2011	5	30	10500	62.08	24.85
20640423	2011	5	30	10510	61.4	24.86
20640423	2011	5	30	10520	60.14	24.88
20640423	2011	5	30	10530	58.29	24.9
20640423	2011	5	30	10540	56.38	24.94
20640423	2011	5	30	10550	54.99	24.96
20640423	2011	5	30	10560	53.35	24.97
20640423	2011	5	30	10570	51.54	25.04
20640423	2011	5	30	10580	50	25.19
20640423	2011	5	30	10590	48.34	25.35
20640423	2011	5	30	10600	46.85	25.5
20640423	2011	5	30	10610	44.94	25.66
20640423	2011	5	30	10620	43.2	25.79
20640423	2011	5	30	10630	41.69	25.96
20640423	2011	5	30	10640	40.38	26.07
20640423	2011	5	30	10650	38.57	26.21
20640423	2011	5	30	10660	36.86	26.32
20640423	2011	5	30	10670	35.19	26.45
20640423	2011	5	30	10680	33.61	26.55
20640423	2011	5	30	10690	31.8	26.67
20640423	2011	5	30	10700	29.45	26.77
20640423	2011	5	30	10710	27.36	26.87
20640423	2011	5	30	10720	25.29	26.96

20640423	2011	5	30	10730	23.23	27.03
20640423	2011	5	30	10740	20.98	27.12
20640423	2011	5	30	10750	18.8	27.21
20640423	2011	5	30	10760	17.23	27.28
20640423	2011	5	30	10770	15.68	27.34
20640423	2011	5	30	10780	13.69	27.43
20640423	2011	5	30	10790	11.68	27.52
20640423	2011	5	30	10800	10.01	27.57
20640423	2011	5	30	10810	7.36	27.64
20640423	2011	5	30	10820	5.01	27.71
20640423	2011	5	30	10830	3.31	27.79
20640423	2011	5	30	10840	2.71	27.87
20640423	2011	5	30	10850	2.42	27.9
20640423	2011	5	30	10860	1.91	27.93
20640423	2011	5	30	10870	1.44	27.95
20640423	2011	5	30	10880	1.4	28
20640423	2011	5	30	10890	0.63	28.04
20640423	2011	5	30	10900	0.56	28.09
20640423	2011	5	30	10910	0.56	28.1
20640423	2011	5	30	10920	0.56	28.16
20640423	2011	5	30	10930	0.54	28.2
20640423	2011	5	30	10940	0.55	28.25
20640423	2011	5	30	10950	0.51	28.28
20640423	2011	5	30	10960	0.59	28.32
20640423	2011	5	30	10970	0.58	28.36
20640423	2011	5	30	10980	0.57	28.39
20640423	2011	5	30	10990	0.49	28.41
20640423	2011	5	30	11000	0.51	28.45
20640423	2011	5	30	11010	0.51	28.46
20640423	2011	5	30	11020	0.49	28.48
20640423	2011	5	30	11030	0.5	28.51
20802781	2011	6	1	0	1.06	28.63
20802781	2011	6	1	10	2.33	28.62
20802781	2011	6	1	20	2.73	28.67
20802781	2011	6	1	30	2.6	28.66
20802781	2011	6	1	40	2.76	28.67
20802781	2011	6	1	50	2.68	28.67
20802781	2011	6	1	60	3	28.66
20802781	2011	6	1	70	3.67	28.65
20802781	2011	6	1	80	5.34	28.64
20802781	2011	6	1	90	7.49	28.61
20802781	2011	6	1	100	8.46	28.6
20802781	2011	6	1	110	8.88	28.6
20802781	2011	6	1	120	9.44	28.6
20802781	2011	6	1	130	10.39	28.6
20802781	2011	6	1	140	11.62	28.57
20802781	2011	6	1	150	12.68	28.57

20802781	2011	6	1	160	13.53	28.55
20802781	2011	6	1	170	14.32	28.55
20802781	2011	6	1	180	14.36	28.55
20802781	2011	6	1	190	14.31	28.54
20802781	2011	6	1	200	14.3	28.54
20802781	2011	6	1	210	14.4	28.54
20802781	2011	6	1	220	14.34	28.53
20802781	2011	6	1	230	13.71	28.53
20802781	2011	6	1	240	13.87	28.54
20802781	2011	6	1	250	14.31	28.53
20802781	2011	6	1	260	14.64	28.53
20802781	2011	6	1	270	15.25	28.53
20802781	2011	6	1	280	15.77	28.53
20802781	2011	6	1	290	16.2	28.53
20802781	2011	6	1	300	17.26	28.51
20802781	2011	6	1	310	18.92	28.5
20802781	2011	6	1	320	20.33	28.48
20802781	2011	6	1	330	21.06	28.48
20802781	2011	6	1	340	22.64	28.46
20802781	2011	6	1	350	24.62	28.44
20802781	2011	6	1	360	26.39	28.42
20802781	2011	6	1	370	28.74	28.39
20802781	2011	6	1	380	31.79	28.37
20802781	2011	6	1	390	34.12	28.35
20802781	2011	6	1	400	36.34	28.34
20802781	2011	6	1	410	39.03	28.31
20802781	2011	6	1	420	40.88	28.29
20802781	2011	6	1	430	41.87	28.28
20802781	2011	6	1	440	43.2	28.28
20802781	2011	6	1	450	45.26	28.25
20802781	2011	6	1	460	48.32	28.23
20802781	2011	6	1	470	50.68	28.17
20802781	2011	6	1	480	51.99	28.03
20802781	2011	6	1	490	52.46	27.89
20802781	2011	6	1	500	52.77	27.77
20802781	2011	6	1	510	54.29	27.61
20802781	2011	6	1	520	55.64	27.49
20802781	2011	6	1	530	57.57	27.23
20802781	2011	6	1	540	59.06	27.14
20802781	2011	6	1	550	59.44	27.07
20802781	2011	6	1	560	59.56	26.96
20802781	2011	6	1	570	59.5	26.87
20802781	2011	6	1	580	60.54	26.64
20802781	2011	6	1	590	62.17	26.53
20802781	2011	6	1	600	64.48	26.46
20802781	2011	6	1	610	66.19	26.4
20802781	2011	6	1	620	68.27	26.32

20802781	2011	6	1	630	69.56	26.23
20802781	2011	6	1	640	69.58	26.14
20802781	2011	6	1	650	69.69	26.08
20802781	2011	6	1	660	69.75	26.02
20802781	2011	6	1	670	69.79	25.95
20802781	2011	6	1	680	70.01	25.88
20802781	2011	6	1	690	70.23	25.82
20802781	2011	6	1	700	70.68	25.71
20802781	2011	6	1	710	72.66	25.61
20802781	2011	6	1	720	74.98	25.55
20802781	2011	6	1	730	77	25.47
20802781	2011	6	1	740	77.92	25.42
20802781	2011	6	1	750	79.42	25.35
20802781	2011	6	1	760	80.75	25.31
20802781	2011	6	1	770	82.02	25.26
20802781	2011	6	1	780	83.21	25.21
20802781	2011	6	1	790	84.37	25.16
20802781	2011	6	1	800	85.23	25.11
20802781	2011	6	1	810	86.23	25.07
20802781	2011	6	1	820	88.03	24.97
20802781	2011	6	1	830	90.12	24.93
20802781	2011	6	1	840	91.83	24.9
20802781	2011	6	1	850	94.39	24.85
20802781	2011	6	1	860	96.7	24.79
20802781	2011	6	1	870	98.52	24.76
20802781	2011	6	1	880	99.88	24.7
20802781	2011	6	1	890	100.9	24.65
20802781	2011	6	1	900	101.71	24.61
20802781	2011	6	1	910	102.45	24.57
20802781	2011	6	1	920	103.57	24.5
20802781	2011	6	1	930	105.34	24.32
20802781	2011	6	1	940	107.33	24.09
20802781	2011	6	1	950	109.31	23.97
20802781	2011	6	1	960	110.57	23.92
20802781	2011	6	1	970	113.21	23.68
20802781	2011	6	1	980	116.51	23.6
20802781	2011	6	1	990	118.76	23.52
20802781	2011	6	1	1000	120.77	23.35
20802781	2011	6	1	1010	123.12	23.27
20802781	2011	6	1	1020	124.99	23.19
20802781	2011	6	1	1030	125.91	23.12
20802781	2011	6	1	1040	126.68	23.04
20802781	2011	6	1	1050	128.3	22.83
20802781	2011	6	1	1060	129.08	22.78
20802781	2011	6	1	1070	129.08	22.7
20802781	2011	6	1	1080	129.13	22.64
20802781	2011	6	1	1090	129.2	22.57

20802781	2011	6	1	1100	129.39	22.5
20802781	2011	6	1	1110	129.41	22.42
20802781	2011	6	1	1120	129.49	22.34
20802781	2011	6	1	1130	129.56	22.26
20802781	2011	6	1	1140	129.57	22.21
20802781	2011	6	1	1150	129.45	22.14
20802781	2011	6	1	1160	129.56	22.08
20802781	2011	6	1	1170	129.48	22.03
20802781	2011	6	1	1180	129.49	21.96
20802781	2011	6	1	1190	129.65	21.91
20802781	2011	6	1	1200	129.59	21.85
20802781	2011	6	1	1210	129.05	21.82
20802781	2011	6	1	1220	127.09	21.75
20802781	2011	6	1	1230	124.81	21.71
20802781	2011	6	1	1240	123.02	21.69
20802781	2011	6	1	1250	121.38	21.63
20802781	2011	6	1	1260	119.42	21.61
20802781	2011	6	1	1270	117.14	21.6
20802781	2011	6	1	1280	114.89	21.57
20802781	2011	6	1	1290	112.39	21.57
20802781	2011	6	1	1300	110.81	21.56
20802781	2011	6	1	1310	108.49	21.57
20802781	2011	6	1	1320	105.93	21.59
20802781	2011	6	1	1330	103.71	21.61
20802781	2011	6	1	1340	101.5	21.65
20802781	2011	6	1	1350	99.38	21.7
20802781	2011	6	1	1360	96.84	21.76
20802781	2011	6	1	1370	94.33	21.82
20802781	2011	6	1	1380	92.25	21.88
20802781	2011	6	1	1390	91.2	21.96
20802781	2011	6	1	1400	90.21	22.03
20802781	2011	6	1	1410	88.54	22.13
20802781	2011	6	1	1420	86.14	22.24
20802781	2011	6	1	1430	84.04	22.33
20802781	2011	6	1	1440	81.84	22.42
20802781	2011	6	1	1450	80.34	22.51
20802781	2011	6	1	1460	78.84	22.6
20802781	2011	6	1	1470	77.64	22.68
20802781	2011	6	1	1480	77.11	22.76
20802781	2011	6	1	1490	77.08	22.85
20802781	2011	6	1	1500	76.97	22.91
20802781	2011	6	1	1510	77.12	22.97
20802781	2011	6	1	1520	77.59	23.02
20802781	2011	6	1	1530	77.31	23.08
20802781	2011	6	1	1540	76.28	23.15
20802781	2011	6	1	1550	75.76	23.21
20802781	2011	6	1	1560	76.75	23.29

20802781	2011	6	1	1570	77.24	23.35
20802781	2011	6	1	1580	77.37	23.39
20802781	2011	6	1	1590	77.74	23.42
20802781	2011	6	1	1600	77.78	23.46
20802781	2011	6	1	1610	77.75	23.5
20802781	2011	6	1	1620	79.35	23.55
20802781	2011	6	1	1630	81.9	23.58
20802781	2011	6	1	1640	84.72	23.6
20802781	2011	6	1	1650	87.41	23.64
20802781	2011	6	1	1660	90.44	23.64
20802781	2011	6	1	1670	93.59	23.66
20802781	2011	6	1	1680	96.67	23.66
20802781	2011	6	1	1690	99.47	23.67
20802781	2011	6	1	1700	102.53	23.6
20802781	2011	6	1	1710	105.76	23.55
20802781	2011	6	1	1720	108.86	23.45
20802781	2011	6	1	1730	111.94	23.37
20802781	2011	6	1	1740	114.01	23.31
20802781	2011	6	1	1750	114.12	23.24
20802781	2011	6	1	1760	114.32	23.2
20802781	2011	6	1	1770	114.5	23.17
20802781	2011	6	1	1780	114.61	23.13
20802781	2011	6	1	1790	114.38	23.1
20802781	2011	6	1	1800	114.04	23.07
20802781	2011	6	1	1810	113.69	23.04
20802781	2011	6	1	1820	113.75	23.02
20802781	2011	6	1	1830	114.17	22.97
20802781	2011	6	1	1840	115.56	22.89
20802781	2011	6	1	1850	118.16	22.78
20802781	2011	6	1	1860	120.13	22.71
20802781	2011	6	1	1870	121.9	22.58
20802781	2011	6	1	1880	124.16	22.47
20802781	2011	6	1	1890	126.14	22.43
20802781	2011	6	1	1900	127.53	22.36
20802781	2011	6	1	1910	128.57	22.32
20802781	2011	6	1	1920	129.13	22.28
20802781	2011	6	1	1930	130.35	22.19
20802781	2011	6	1	1940	131.05	22.14
20802781	2011	6	1	1950	131.68	22.05
20802781	2011	6	1	1960	133.53	21.87
20802781	2011	6	1	1970	134.7	21.81
20802781	2011	6	1	1980	135.62	21.76
20802781	2011	6	1	1990	136.93	21.71
20802781	2011	6	1	2000	137.94	21.65
20802781	2011	6	1	2010	138.99	21.6
20802781	2011	6	1	2020	139.74	21.55
20802781	2011	6	1	2030	140.21	21.52

20802781	2011	6	1	2040	140.34	21.44
20802781	2011	6	1	2050	140.49	21.38
20802781	2011	6	1	2060	140.95	21.32
20802781	2011	6	1	2070	140.85	21.27
20802781	2011	6	1	2080	140.73	21.19
20802781	2011	6	1	2090	140.4	21.11
20802781	2011	6	1	2100	140.4	21.07
20802781	2011	6	1	2110	140.39	21.02
20802781	2011	6	1	2120	140.46	20.96
20802781	2011	6	1	2130	140.38	20.92
20802781	2011	6	1	2140	140.55	20.87
20802781	2011	6	1	2150	140.46	20.82
20802781	2011	6	1	2160	140.3	20.78
20802781	2011	6	1	2170	140.62	20.75
20802781	2011	6	1	2180	140.46	20.69
20802781	2011	6	1	2190	140.87	20.65
20802781	2011	6	1	2200	142.04	20.55
20802781	2011	6	1	2210	142.11	20.53
20802781	2011	6	1	2220	142.5	20.5
20802781	2011	6	1	2230	142.76	20.46
20802781	2011	6	1	2240	142.77	20.44
20802781	2011	6	1	2250	142.19	20.41
20802781	2011	6	1	2260	142.55	20.33
20802781	2011	6	1	2270	142.64	20.29
20802781	2011	6	1	2280	142.73	20.28
20802781	2011	6	1	2290	142.91	20.25
20802781	2011	6	1	2300	143.19	20.22
20802781	2011	6	1	2310	143.61	20.19
20802781	2011	6	1	2320	144.15	20.12
20802781	2011	6	1	2330	144.19	20.09
20802781	2011	6	1	2340	144.27	20.07
20802781	2011	6	1	2350	144.25	20.03
20802781	2011	6	1	2360	144.1	20.01
20802781	2011	6	1	2370	143.82	19.98
20802781	2011	6	1	2380	144.18	19.95
20802781	2011	6	1	2390	144.7	19.93
20802781	2011	6	1	2400	146	19.89
20802781	2011	6	1	2410	146.57	19.86
20802781	2011	6	1	2420	147.53	19.85
20802781	2011	6	1	2430	147.69	19.8
20802781	2011	6	1	2440	148.03	19.78
20802781	2011	6	1	2450	148.43	19.75
20802781	2011	6	1	2460	148.14	19.74
20802781	2011	6	1	2470	148.46	19.71
20802781	2011	6	1	2480	148.26	19.69
20802781	2011	6	1	2490	148.22	19.67
20802781	2011	6	1	2500	148.16	19.66

20802781	2011	6	1	2510	148.23	19.64
20802781	2011	6	1	2520	148.33	19.64
20802781	2011	6	1	2530	148.71	19.61
20802781	2011	6	1	2540	148.99	19.6
20802781	2011	6	1	2550	149.21	19.58
20802781	2011	6	1	2560	149.36	19.56
20802781	2011	6	1	2570	149.83	19.54
20802781	2011	6	1	2580	150.36	19.52
20802781	2011	6	1	2590	150.72	19.51
20802781	2011	6	1	2600	151.03	19.5
20802781	2011	6	1	2610	151.4	19.48
20802781	2011	6	1	2620	151.7	19.46
20802781	2011	6	1	2630	151.78	19.45
20802781	2011	6	1	2640	151.76	19.43
20802781	2011	6	1	2650	152.04	19.42
20802781	2011	6	1	2660	151.72	19.4
20802781	2011	6	1	2670	152.25	19.39
20802781	2011	6	1	2680	152.5	19.36
20802781	2011	6	1	2690	152.54	19.36
20802781	2011	6	1	2700	152.56	19.35
20802781	2011	6	1	2710	152.76	19.34
20802781	2011	6	1	2720	152.74	19.32
20802781	2011	6	1	2730	152.6	19.3
20802781	2011	6	1	2740	152.6	19.31
20802781	2011	6	1	2750	152.61	19.29
20802781	2011	6	1	2760	152.78	19.29
20802781	2011	6	1	2770	152.95	19.28
20802781	2011	6	1	2780	152.35	19.25
20802781	2011	6	1	2790	151.86	19.25
20802781	2011	6	1	2800	151.86	19.25
20802781	2011	6	1	2810	152.11	19.24
20802781	2011	6	1	2820	152.54	19.23
20802781	2011	6	1	2830	152.9	19.22
20802781	2011	6	1	2840	153.28	19.22
20802781	2011	6	1	2850	152.96	19.21
20802781	2011	6	1	2860	152.84	19.21
20802781	2011	6	1	2870	152.84	19.21
20802781	2011	6	1	2880	152.87	19.2
20802781	2011	6	1	2890	152.95	19.19
20802781	2011	6	1	2900	152.87	19.18
20802781	2011	6	1	2910	152.82	19.17
20802781	2011	6	1	2920	152.77	19.18
20802781	2011	6	1	2930	152.77	19.17
20802781	2011	6	1	2940	152.95	19.17
20802781	2011	6	1	2950	152.67	19.17
20802781	2011	6	1	2960	152.53	19.16
20802781	2011	6	1	2970	152.79	19.15

20802781	2011	6	1	2980	151.86	19.15
20802781	2011	6	1	2990	151.18	19.15
20802781	2011	6	1	3000	150.63	19.15
20802781	2011	6	1	3010	150.15	19.15
20802781	2011	6	1	3020	151.37	19.14
20802781	2011	6	1	3030	152.23	19.14
20802781	2011	6	1	3040	152.58	19.14
20802781	2011	6	1	3050	152.52	19.14
20802781	2011	6	1	3060	152.54	19.14
20802781	2011	6	1	3070	152.74	19.14
20802781	2011	6	1	3080	153.88	19.14
20802781	2011	6	1	3090	154.91	19.14
20802781	2011	6	1	3100	155.45	19.14
20802781	2011	6	1	3110	156.02	19.13
20802781	2011	6	1	3120	156.13	19.12
20802781	2011	6	1	3130	156.43	19.11
20802781	2011	6	1	3140	156.57	19.1
20802781	2011	6	1	3150	156.56	19.1
20802781	2011	6	1	3160	156.53	19.1
20802781	2011	6	1	3170	156.09	19.09
20802781	2011	6	1	3180	155.89	19.1
20802781	2011	6	1	3190	155.75	19.1
20802781	2011	6	1	3200	155.57	19.08
20802781	2011	6	1	3210	155.58	19.09
20802781	2011	6	1	3220	156.21	19.08
20802781	2011	6	1	3230	156.28	19.08
20802781	2011	6	1	3240	155.78	19.07
20802781	2011	6	1	3250	155.07	19.07
20802781	2011	6	1	3260	154.61	19.07
20802781	2011	6	1	3270	154.22	19.07
20802781	2011	6	1	3280	153.94	19.07
20802781	2011	6	1	3290	154.67	19.07
20802781	2011	6	1	3300	155.42	19.07
20802781	2011	6	1	3310	155.78	19.07
20802781	2011	6	1	3320	156.16	19.07
20802781	2011	6	1	3330	156.48	19.05
20802781	2011	6	1	3340	156.76	19.06
20802781	2011	6	1	3350	157.3	19.06
20802781	2011	6	1	3360	158.74	19.04
20802781	2011	6	1	3370	159.29	19.04
20802781	2011	6	1	3380	159.6	19.03
20802781	2011	6	1	3390	159.76	19.03
20802781	2011	6	1	3400	159.98	19
20802781	2011	6	1	3410	160.3	18.97
20802781	2011	6	1	3420	161.1	18.95
20802781	2011	6	1	3430	161.31	18.93
20802781	2011	6	1	3440	161.66	18.92

20802781	2011	6	1	3450	162.19	18.92
20802781	2011	6	1	3460	162.59	18.89
20802781	2011	6	1	3470	162.81	18.85
20802781	2011	6	1	3480	162.87	18.82
20802781	2011	6	1	3490	162.71	18.78
20802781	2011	6	1	3500	162.64	18.77
20802781	2011	6	1	3510	162.6	18.75
20802781	2011	6	1	3520	162.52	18.73
20802781	2011	6	1	3530	162.39	18.71
20802781	2011	6	1	3540	162.39	18.69
20802781	2011	6	1	3550	162.69	18.66
20802781	2011	6	1	3560	162.3	18.64
20802781	2011	6	1	3570	162.67	18.62
20802781	2011	6	1	3580	162.29	18.6
20802781	2011	6	1	3590	162.6	18.57
20802781	2011	6	1	3600	162.53	18.55
20802781	2011	6	1	3610	162.51	18.53
20802781	2011	6	1	3620	162.39	18.52
20802781	2011	6	1	3630	162.48	18.5
20802781	2011	6	1	3640	161.98	18.48
20802781	2011	6	1	3650	161.17	18.45
20802781	2011	6	1	3660	161.05	18.44
20802781	2011	6	1	3670	161.25	18.43
20802781	2011	6	1	3680	161.62	18.42
20802781	2011	6	1	3690	161.76	18.41
20802781	2011	6	1	3700	161.85	18.39
20802781	2011	6	1	3710	162.23	18.39
20802781	2011	6	1	3720	163.42	18.35
20802781	2011	6	1	3730	163.76	18.35
20802781	2011	6	1	3740	163.79	18.34
20802781	2011	6	1	3750	164.04	18.32
20802781	2011	6	1	3760	164.09	18.31
20802781	2011	6	1	3770	164.18	18.28
20802781	2011	6	1	3780	164.1	18.26
20802781	2011	6	1	3790	163.92	18.25
20802781	2011	6	1	3800	164.22	18.24
20802781	2011	6	1	3810	164.05	18.22
20802781	2011	6	1	3820	164.24	18.22
20802781	2011	6	1	3830	164.39	18.21
20802781	2011	6	1	3840	164.81	18.2
20802781	2011	6	1	3850	164.91	18.19
20802781	2011	6	1	3860	164.98	18.17
20802781	2011	6	1	3870	165.17	18.17
20802781	2011	6	1	3880	165.46	18.16
20802781	2011	6	1	3890	165.83	18.16
20802781	2011	6	1	3900	166.08	18.14
20802781	2011	6	1	3910	165.89	18.14

20802781	2011	6	1	3920	165.82	18.12
20802781	2011	6	1	3930	165.77	18.11
20802781	2011	6	1	3940	165.7	18.1
20802781	2011	6	1	3950	165.65	18.09
20802781	2011	6	1	3960	165.54	18.09
20802781	2011	6	1	3970	165.41	18.07
20802781	2011	6	1	3980	165.42	18.06
20802781	2011	6	1	3990	165.51	18.05
20802781	2011	6	1	4000	165.47	18.05
20802781	2011	6	1	4010	164.61	18.03
20802781	2011	6	1	4020	163.82	18.03
20802781	2011	6	1	4030	164.25	18.02
20802781	2011	6	1	4040	163.74	18.01
20802781	2011	6	1	4050	165.04	18.01
20802781	2011	6	1	4060	166.03	18
20802781	2011	6	1	4070	166.15	18
20802781	2011	6	1	4080	166.27	18
20802781	2011	6	1	4090	166.42	18
20802781	2011	6	1	4100	166.17	17.99
20802781	2011	6	1	4110	165.66	17.99
20802781	2011	6	1	4120	165.34	17.99
20802781	2011	6	1	4130	165.08	17.99
20802781	2011	6	1	4140	164.91	17.98
20802781	2011	6	1	4150	164.64	17.98
20802781	2011	6	1	4160	164.18	17.98
20802781	2011	6	1	4170	163.61	17.98
20802781	2011	6	1	4180	162.98	17.98
20802781	2011	6	1	4190	162.39	17.98
20802781	2011	6	1	4200	162.45	17.99
20802781	2011	6	1	4210	162.43	17.99
20802781	2011	6	1	4220	161.82	18
20802781	2011	6	1	4230	161.47	18
20802781	2011	6	1	4240	161.24	18
20802781	2011	6	1	4250	160.91	18
20802781	2011	6	1	4260	160.68	18.01
20802781	2011	6	1	4270	161.03	18.01
20802781	2011	6	1	4280	161.32	18.03
20802781	2011	6	1	4290	161.62	18.03
20802781	2011	6	1	4300	162.19	18.04
20802781	2011	6	1	4310	162.33	18.06
20802781	2011	6	1	4320	162.48	18.07
20802781	2011	6	1	4330	162.5	18.07
20802781	2011	6	1	4340	161.82	18.08
20802781	2011	6	1	4350	161.86	18.1
20802781	2011	6	1	4360	162.17	18.1
20802781	2011	6	1	4370	162.36	18.11
20802781	2011	6	1	4380	162.38	18.13

20802781	2011	6	1	4390	162.48	18.14
20802781	2011	6	1	4400	162.31	18.14
20802781	2011	6	1	4410	161.76	18.17
20802781	2011	6	1	4420	161.51	18.19
20802781	2011	6	1	4430	161.41	18.21
20802781	2011	6	1	4440	161.28	18.22
20802781	2011	6	1	4450	161.15	18.23
20802781	2011	6	1	4460	161.32	18.25
20802781	2011	6	1	4470	161.54	18.26
20802781	2011	6	1	4480	161.78	18.26
20802781	2011	6	1	4490	161.95	18.27
20802781	2011	6	1	4500	161.93	18.27
20802781	2011	6	1	4510	161.94	18.28
20802781	2011	6	1	4520	162.08	18.28
20802781	2011	6	1	4530	162.2	18.27
20802781	2011	6	1	4540	161.99	18.28
20802781	2011	6	1	4550	161.93	18.27
20802781	2011	6	1	4560	162.09	18.27
20802781	2011	6	1	4570	161.63	18.26
20802781	2011	6	1	4580	161.55	18.25
20802781	2011	6	1	4590	161.49	18.24
20802781	2011	6	1	4600	161.42	18.24
20802781	2011	6	1	4610	161.38	18.24
20802781	2011	6	1	4620	161.26	18.24
20802781	2011	6	1	4630	161.5	18.23
20802781	2011	6	1	4640	161.56	18.23
20802781	2011	6	1	4650	161.57	18.22
20802781	2011	6	1	4660	161.95	18.21
20802781	2011	6	1	4670	161.94	18.21
20802781	2011	6	1	4680	162.17	18.21
20802781	2011	6	1	4690	162.74	18.2
20802781	2011	6	1	4700	162.82	18.2
20802781	2011	6	1	4710	161.97	18.19
20802781	2011	6	1	4720	161.82	18.17
20802781	2011	6	1	4730	161.76	18.17
20802781	2011	6	1	4740	161.68	18.16
20802781	2011	6	1	4750	161.62	18.16
20802781	2011	6	1	4760	161.5	18.15
20802781	2011	6	1	4770	161.36	18.15
20802781	2011	6	1	4780	161.28	18.15
20802781	2011	6	1	4790	161.35	18.15
20802781	2011	6	1	4800	161.57	18.14
20802781	2011	6	1	4810	161.89	18.14
20802781	2011	6	1	4820	161.29	18.14
20802781	2011	6	1	4830	160.65	18.14
20802781	2011	6	1	4840	160.43	18.14
20802781	2011	6	1	4850	160.64	18.14

20802781	2011	6	1	4860	160.6	18.15
20802781	2011	6	1	4870	160.42	18.16
20802781	2011	6	1	4880	160.51	18.16
20802781	2011	6	1	4890	160.76	18.16
20802781	2011	6	1	4900	161.12	18.15
20802781	2011	6	1	4910	161.73	18.15
20802781	2011	6	1	4920	162.73	18.13
20802781	2011	6	1	4930	163.37	18.11
20802781	2011	6	1	4940	163.81	18.12
20802781	2011	6	1	4950	164.18	18.11
20802781	2011	6	1	4960	165.39	18.09
20802781	2011	6	1	4970	165.83	18.08
20802781	2011	6	1	4980	165.69	18.07
20802781	2011	6	1	4990	166.12	18.06
20802781	2011	6	1	5000	166.26	18.03
20802781	2011	6	1	5010	166.52	18.02
20802781	2011	6	1	5020	166.74	18
20802781	2011	6	1	5030	166.72	18
20802781	2011	6	1	5040	166.78	17.99
20802781	2011	6	1	5050	166.72	17.98
20802781	2011	6	1	5060	166.76	17.95
20802781	2011	6	1	5070	166.76	17.95
20802781	2011	6	1	5080	166.4	17.94
20802781	2011	6	1	5090	166.38	17.93
20802781	2011	6	1	5100	166.35	17.92
20802781	2011	6	1	5110	166.36	17.92
20802781	2011	6	1	5120	166.53	17.92
20802781	2011	6	1	5130	166.63	17.9
20802781	2011	6	1	5140	166.85	17.89
20802781	2011	6	1	5150	166.8	17.89
20802781	2011	6	1	5160	166.32	17.89
20802781	2011	6	1	5170	166.51	17.88
20802781	2011	6	1	5180	166.4	17.88
20802781	2011	6	1	5190	166.42	17.88
20802781	2011	6	1	5200	166.72	17.87
20802781	2011	6	1	5210	166.43	17.86
20802781	2011	6	1	5220	166.72	17.86
20802781	2011	6	1	5230	166.67	17.85
20802781	2011	6	1	5240	166.65	17.85
20802781	2011	6	1	5250	166.6	17.85
20802781	2011	6	1	5260	166.26	17.85
20802781	2011	6	1	5270	166.06	17.85
20802781	2011	6	1	5280	166.45	17.84
20802781	2011	6	1	5290	166.81	17.84
20802781	2011	6	1	5300	166.38	17.84
20802781	2011	6	1	5310	166.38	17.84
20802781	2011	6	1	5320	166.54	17.84

20802781	2011	6	1	5330	166.56	17.84
20802781	2011	6	1	5340	166.28	17.84
20802781	2011	6	1	5350	165.45	17.84
20802781	2011	6	1	5360	164.95	17.83
20802781	2011	6	1	5370	164.64	17.84
20802781	2011	6	1	5380	165.08	17.83
20802781	2011	6	1	5390	165.95	17.83
20802781	2011	6	1	5400	166.15	17.83
20802781	2011	6	1	5410	166.63	17.83
20802781	2011	6	1	5420	166.74	17.84
20802781	2011	6	1	5430	166.37	17.83
20802781	2011	6	1	5440	166.73	17.84
20802781	2011	6	1	5450	166.83	17.83
20802781	2011	6	1	5460	166.48	17.84
20802781	2011	6	1	5470	166.67	17.84
20802781	2011	6	1	5480	166.74	17.84
20802781	2011	6	1	5490	166.9	17.84
20802781	2011	6	1	5500	167.28	17.84
20802781	2011	6	1	5510	167.51	17.83
20802781	2011	6	1	5520	167.84	17.83
20802781	2011	6	1	5530	168.14	17.83
20802781	2011	6	1	5540	167.9	17.82
20802781	2011	6	1	5550	168.18	17.82
20802781	2011	6	1	5560	168.55	17.82
20802781	2011	6	1	5570	168.09	17.82
20802781	2011	6	1	5580	167.8	17.82
20802781	2011	6	1	5590	168.6	17.82
20802781	2011	6	1	5600	168.67	17.82
20802781	2011	6	1	5610	168.69	17.82
20802781	2011	6	1	5620	168.51	17.81
20802781	2011	6	1	5630	168.35	17.81
20802781	2011	6	1	5640	168.4	17.81
20802781	2011	6	1	5650	167.58	17.82
20802781	2011	6	1	5660	167.09	17.81
20802781	2011	6	1	5670	166.88	17.81
20802781	2011	6	1	5680	166.73	17.81
20802781	2011	6	1	5690	166.65	17.81
20802781	2011	6	1	5700	166.71	17.82
20802781	2011	6	1	5710	167.05	17.8
20802781	2011	6	1	5720	167.78	17.8
20802781	2011	6	1	5730	168.25	17.8
20802781	2011	6	1	5740	168.32	17.8
20802781	2011	6	1	5750	169.17	17.8
20802781	2011	6	1	5760	169.65	17.8
20802781	2011	6	1	5770	169.86	17.79
20802781	2011	6	1	5780	169.98	17.8
20802781	2011	6	1	5790	170.07	17.79

20802781	2011	6	1	5800	170.08	17.78
20802781	2011	6	1	5810	170.15	17.79
20802781	2011	6	1	5820	170.58	17.78
20802781	2011	6	1	5830	172.43	17.76
20802781	2011	6	1	5840	173.88	17.76
20802781	2011	6	1	5850	175.55	17.74
20802781	2011	6	1	5860	176.39	17.72
20802781	2011	6	1	5870	176.78	17.7
20802781	2011	6	1	5880	177.44	17.64
20802781	2011	6	1	5890	178.35	17.6
20802781	2011	6	1	5900	178.54	17.59
20802781	2011	6	1	5910	178.62	17.57
20802781	2011	6	1	5920	178.45	17.56
20802781	2011	6	1	5930	178.5	17.53
20802781	2011	6	1	5940	178.64	17.51
20802781	2011	6	1	5950	178.5	17.5
20802781	2011	6	1	5960	178.05	17.47
20802781	2011	6	1	5970	177.65	17.46
20802781	2011	6	1	5980	177.65	17.44
20802781	2011	6	1	5990	177.75	17.42
20802781	2011	6	1	6000	177.61	17.41
20802781	2011	6	1	6010	177.5	17.39
20802781	2011	6	1	6020	177.6	17.38
20802781	2011	6	1	6030	177.56	17.36
20802781	2011	6	1	6040	177.54	17.35
20802781	2011	6	1	6050	177.44	17.34
20802781	2011	6	1	6060	177.22	17.33
20802781	2011	6	1	6070	176.87	17.32
20802781	2011	6	1	6080	176.22	17.32
20802781	2011	6	1	6090	175.88	17.3
20802781	2011	6	1	6100	175.8	17.29
20802781	2011	6	1	6110	175.78	17.28
20802781	2011	6	1	6120	175.42	17.28
20802781	2011	6	1	6130	174.96	17.28
20802781	2011	6	1	6140	174.35	17.27
20802781	2011	6	1	6150	174	17.26
20802781	2011	6	1	6160	173.87	17.26
20802781	2011	6	1	6170	173.42	17.27
20802781	2011	6	1	6180	173.7	17.27
20802781	2011	6	1	6190	173.89	17.27
20802781	2011	6	1	6200	173.87	17.27
20802781	2011	6	1	6210	173.9	17.28
20802781	2011	6	1	6220	173.89	17.28
20802781	2011	6	1	6230	173.95	17.28
20802781	2011	6	1	6240	173.94	17.29
20802781	2011	6	1	6250	174.26	17.29
20802781	2011	6	1	6260	174.72	17.29

20802781	2011	6	1	6270	175.02	17.3
20802781	2011	6	1	6280	175.16	17.3
20802781	2011	6	1	6290	175.21	17.3
20802781	2011	6	1	6300	176.32	17.3
20802781	2011	6	1	6310	176.88	17.29
20802781	2011	6	1	6320	177.1	17.29
20802781	2011	6	1	6330	177.21	17.29
20802781	2011	6	1	6340	177.24	17.3
20802781	2011	6	1	6350	177.35	17.29
20802781	2011	6	1	6360	177.54	17.29
20802781	2011	6	1	6370	177.76	17.28
20802781	2011	6	1	6380	177.98	17.28
20802781	2011	6	1	6390	178.22	17.28
20802781	2011	6	1	6400	178.34	17.28
20802781	2011	6	1	6410	178.35	17.27
20802781	2011	6	1	6420	178.38	17.27
20802781	2011	6	1	6430	178.35	17.26
20802781	2011	6	1	6440	178.38	17.26
20802781	2011	6	1	6450	178.34	17.25
20802781	2011	6	1	6460	178.26	17.25
20802781	2011	6	1	6470	178.06	17.25
20802781	2011	6	1	6480	177.77	17.25
20802781	2011	6	1	6490	178.42	17.25
20802781	2011	6	1	6500	178.37	17.25
20802781	2011	6	1	6510	178.11	17.24
20802781	2011	6	1	6520	177.68	17.25
20802781	2011	6	1	6530	177.69	17.25
20802781	2011	6	1	6540	177.67	17.25
20802781	2011	6	1	6550	177.69	17.25
20802781	2011	6	1	6560	177.88	17.26
20802781	2011	6	1	6570	177.46	17.25
20802781	2011	6	1	6580	177.59	17.26
20802781	2011	6	1	6590	177.39	17.27
20802781	2011	6	1	6600	177.18	17.28
20802781	2011	6	1	6610	177.66	17.28
20802781	2011	6	1	6620	177.37	17.28
20802781	2011	6	1	6630	177.06	17.29
20802781	2011	6	1	6640	175.78	17.3
20802781	2011	6	1	6650	174.25	17.3
20802781	2011	6	1	6660	173.64	17.3
20802781	2011	6	1	6670	173.22	17.32
20802781	2011	6	1	6680	173.33	17.32
20802781	2011	6	1	6690	173.91	17.32
20802781	2011	6	1	6700	174.6	17.33
20802781	2011	6	1	6710	174.94	17.34
20802781	2011	6	1	6720	175.38	17.35
20802781	2011	6	1	6730	176.74	17.37

20802781	2011	6	1	6740	177.26	17.37
20802781	2011	6	1	6750	177.59	17.39
20802781	2011	6	1	6760	177.36	17.39
20802781	2011	6	1	6770	177.43	17.39
20802781	2011	6	1	6780	177.39	17.39
20802781	2011	6	1	6790	177.55	17.39
20802781	2011	6	1	6800	177.73	17.39
20802781	2011	6	1	6810	177.75	17.39
20802781	2011	6	1	6820	179.02	17.39
20802781	2011	6	1	6830	179.97	17.38
20802781	2011	6	1	6840	180.21	17.37
20802781	2011	6	1	6850	180.74	17.36
20802781	2011	6	1	6860	181.94	17.33
20802781	2011	6	1	6870	182.4	17.31
20802781	2011	6	1	6880	182.48	17.3
20802781	2011	6	1	6890	182.75	17.29
20802781	2011	6	1	6900	182.9	17.27
20802781	2011	6	1	6910	182.95	17.25
20802781	2011	6	1	6920	182.9	17.25
20802781	2011	6	1	6930	182.7	17.24
20802781	2011	6	1	6940	182.84	17.23
20802781	2011	6	1	6950	183.07	17.21
20802781	2011	6	1	6960	183.03	17.2
20802781	2011	6	1	6970	183.98	17.18
20802781	2011	6	1	6980	185.1	17.17
20802781	2011	6	1	6990	185.43	17.17
20802781	2011	6	1	7000	185.33	17.15
20802781	2011	6	1	7010	185.39	17.14
20802781	2011	6	1	7020	185.44	17.14
20802781	2011	6	1	7030	184.72	17.14
20802781	2011	6	1	7040	184.44	17.13
20802781	2011	6	1	7050	184.34	17.12
20802781	2011	6	1	7060	184.48	17.1
20802781	2011	6	1	7070	184.58	17.09
20802781	2011	6	1	7080	184.91	17.09
20802781	2011	6	1	7090	186.34	17.07
20802781	2011	6	1	7100	187.1	17.08
20802781	2011	6	1	7110	186.97	17.07
20802781	2011	6	1	7120	186.73	17.07
20802781	2011	6	1	7130	186.78	17.07
20802781	2011	6	1	7140	186.97	17.06
20802781	2011	6	1	7150	187.32	17.06
20802781	2011	6	1	7160	187.46	17.05
20802781	2011	6	1	7170	187.68	17.03
20802781	2011	6	1	7180	188.45	17.03
20802781	2011	6	1	7190	188.64	17.03
20802781	2011	6	1	7200	188.85	17.03

20802781	2011	6	1	7210	188.94	17.03
20802781	2011	6	1	7220	188.95	17.02
20802781	2011	6	1	7230	188.68	17.02
20802781	2011	6	1	7240	188.92	17.01
20802781	2011	6	1	7250	188.56	17
20802781	2011	6	1	7260	188.24	17
20802781	2011	6	1	7270	188.15	17
20802781	2011	6	1	7280	188.57	17
20802781	2011	6	1	7290	189.2	16.98
20802781	2011	6	1	7300	189.45	16.97
20802781	2011	6	1	7310	189.77	16.97
20802781	2011	6	1	7320	190.55	16.96
20802781	2011	6	1	7330	190.77	16.92
20802781	2011	6	1	7340	191.11	16.91
20802781	2011	6	1	7350	191.57	16.9
20802781	2011	6	1	7360	191.64	16.89
20802781	2011	6	1	7370	192.03	16.87
20802781	2011	6	1	7380	192.16	16.86
20802781	2011	6	1	7390	191.86	16.85
20802781	2011	6	1	7400	192.11	16.84
20802781	2011	6	1	7410	192.55	16.82
20802781	2011	6	1	7420	192.81	16.81
20802781	2011	6	1	7430	192.98	16.8
20802781	2011	6	1	7440	192.81	16.79
20802781	2011	6	1	7450	193.11	16.78
20802781	2011	6	1	7460	193.43	16.76
20802781	2011	6	1	7470	193.4	16.75
20802781	2011	6	1	7480	193.05	16.74
20802781	2011	6	1	7490	192.82	16.73
20802781	2011	6	1	7500	192.66	16.71
20802781	2011	6	1	7510	192.59	16.71
20802781	2011	6	1	7520	192.57	16.71
20802781	2011	6	1	7530	192.82	16.71
20802781	2011	6	1	7540	193.29	16.69
20802781	2011	6	1	7550	193.45	16.69
20802781	2011	6	1	7560	193.52	16.68
20802781	2011	6	1	7570	193.76	16.67
20802781	2011	6	1	7580	193.59	16.66
20802781	2011	6	1	7590	193.32	16.66
20802781	2011	6	1	7600	192.02	16.64
20802781	2011	6	1	7610	191.68	16.64
20802781	2011	6	1	7620	191.08	16.64
20802781	2011	6	1	7630	190.25	16.64
20802781	2011	6	1	7640	189.25	16.64
20802781	2011	6	1	7650	189.54	16.64
20802781	2011	6	1	7660	190.02	16.64
20802781	2011	6	1	7670	190.34	16.64

20802781	2011	6	1	7680	190.82	16.67
20802781	2011	6	1	7690	191.13	16.66
20802781	2011	6	1	7700	191.38	16.66
20802781	2011	6	1	7710	192	16.63
20802781	2011	6	1	7720	193.34	16.63
20802781	2011	6	1	7730	194.16	16.61
20802781	2011	6	1	7740	194.43	16.6
20802781	2011	6	1	7750	194.09	16.6
20802781	2011	6	1	7760	193.61	16.59
20802781	2011	6	1	7770	194.6	16.58
20802781	2011	6	1	7780	194.79	16.57
20802781	2011	6	1	7790	195.06	16.56
20802781	2011	6	1	7800	195.31	16.55
20802781	2011	6	1	7810	195.59	16.54
20802781	2011	6	1	7820	195.89	16.53
20802781	2011	6	1	7830	196.18	16.53
20802781	2011	6	1	7840	196.33	16.52
20802781	2011	6	1	7850	196.38	16.5
20802781	2011	6	1	7860	196.6	16.5
20802781	2011	6	1	7870	197.31	16.49
20802781	2011	6	1	7880	197.61	16.48
20802781	2011	6	1	7890	197.38	16.47
20802781	2011	6	1	7900	197.45	16.46
20802781	2011	6	1	7910	197.2	16.46
20802781	2011	6	1	7920	195.95	16.46
20802781	2011	6	1	7930	195.09	16.44
20802781	2011	6	1	7940	193.95	16.44
20802781	2011	6	1	7950	193.31	16.43
20802781	2011	6	1	7960	194.29	16.42
20802781	2011	6	1	7970	194.34	16.42
20802781	2011	6	1	7980	193.74	16.42
20802781	2011	6	1	7990	192.91	16.42
20802781	2011	6	1	8000	193.13	16.42
20802781	2011	6	1	8010	193.37	16.42
20802781	2011	6	1	8020	193.31	16.41
20802781	2011	6	1	8030	193.43	16.41
20802781	2011	6	1	8040	192.83	16.4
20802781	2011	6	1	8050	192.68	16.39
20802781	2011	6	1	8060	192.56	16.39
20802781	2011	6	1	8070	192.35	16.39
20802781	2011	6	1	8080	192.17	16.39
20802781	2011	6	1	8090	192.22	16.39
20802781	2011	6	1	8100	192.22	16.39
20802781	2011	6	1	8110	192.54	16.38
20802781	2011	6	1	8120	192.6	16.38
20802781	2011	6	1	8130	192.69	16.37
20802781	2011	6	1	8140	192.31	16.37

20802781	2011	6	1	8150	191.71	16.36
20802781	2011	6	1	8160	190.81	16.35
20802781	2011	6	1	8170	190.12	16.36
20802781	2011	6	1	8180	188.92	16.37
20802781	2011	6	1	8190	189.98	16.38
20802781	2011	6	1	8200	190.73	16.38
20802781	2011	6	1	8210	191.42	16.39
20802781	2011	6	1	8220	191.84	16.41
20802781	2011	6	1	8230	192.44	16.42
20802781	2011	6	1	8240	193.22	16.43
20802781	2011	6	1	8250	194.08	16.44
20802781	2011	6	1	8260	194.69	16.43
20802781	2011	6	1	8270	194.44	16.42
20802781	2011	6	1	8280	193.02	16.42
20802781	2011	6	1	8290	192.65	16.41
20802781	2011	6	1	8300	192.91	16.4
20802781	2011	6	1	8310	193.61	16.4
20802781	2011	6	1	8320	193.39	16.41
20802781	2011	6	1	8330	192.92	16.39
20802781	2011	6	1	8340	192.07	16.39
20802781	2011	6	1	8350	191.89	16.4
20802781	2011	6	1	8360	191.9	16.39
20802781	2011	6	1	8370	192.05	16.39
20802781	2011	6	1	8380	192.57	16.39
20802781	2011	6	1	8390	192.21	16.39
20802781	2011	6	1	8400	191.85	16.39
20802781	2011	6	1	8410	191.72	16.4
20802781	2011	6	1	8420	190.54	16.39
20802781	2011	6	1	8430	189.77	16.39
20802781	2011	6	1	8440	188.2	16.4
20802781	2011	6	1	8450	186.47	16.41
20802781	2011	6	1	8460	184.73	16.43
20802781	2011	6	1	8470	183.13	16.46
20802781	2011	6	1	8480	182.26	16.48
20802781	2011	6	1	8490	182.04	16.5
20802781	2011	6	1	8500	182.05	16.51
20802781	2011	6	1	8510	182.2	16.55
20802781	2011	6	1	8520	181.98	16.58
20802781	2011	6	1	8530	180.74	16.6
20802781	2011	6	1	8540	179.95	16.62
20802781	2011	6	1	8550	179.93	16.65
20802781	2011	6	1	8560	180.16	16.67
20802781	2011	6	1	8570	179.37	16.69
20802781	2011	6	1	8580	179.44	16.71
20802781	2011	6	1	8590	179.36	16.73
20802781	2011	6	1	8600	178.46	16.75
20802781	2011	6	1	8610	178.09	16.76

20802781	2011	6	1	8620	177.85	16.78
20802781	2011	6	1	8630	177.97	16.79
20802781	2011	6	1	8640	178.17	16.8
20802781	2011	6	1	8650	178.16	16.82
20802781	2011	6	1	8660	178	16.84
20802781	2011	6	1	8670	176.92	16.86
20802781	2011	6	1	8680	177.04	16.88
20802781	2011	6	1	8690	176.96	16.9
20802781	2011	6	1	8700	176.95	16.91
20802781	2011	6	1	8710	176.69	16.92
20802781	2011	6	1	8720	176.39	16.98
20802781	2011	6	1	8730	176.11	16.99
20802781	2011	6	1	8740	175.88	17
20802781	2011	6	1	8750	175.82	17.01
20802781	2011	6	1	8760	175.94	17.01
20802781	2011	6	1	8770	176.19	17.03
20802781	2011	6	1	8780	176.18	17.03
20802781	2011	6	1	8790	176.06	17.04
20802781	2011	6	1	8800	176.2	17.06
20802781	2011	6	1	8810	176.45	17.07
20802781	2011	6	1	8820	176.77	17.07
20802781	2011	6	1	8830	176.29	17.08
20802781	2011	6	1	8840	175.5	17.1
20802781	2011	6	1	8850	175.02	17.1
20802781	2011	6	1	8860	174.55	17.13
20802781	2011	6	1	8870	174.11	17.14
20802781	2011	6	1	8880	172.99	17.14
20802781	2011	6	1	8890	172.51	17.16
20802781	2011	6	1	8900	172.35	17.17
20802781	2011	6	1	8910	172.4	17.19
20802781	2011	6	1	8920	171.89	17.21
20802781	2011	6	1	8930	171.13	17.21
20802781	2011	6	1	8940	172.03	17.22
20802781	2011	6	1	8950	172.26	17.25
20802781	2011	6	1	8960	172.31	17.26
20802781	2011	6	1	8970	172.3	17.27
20802781	2011	6	1	8980	172.2	17.28
20802781	2011	6	1	8990	172.06	17.29
20802781	2011	6	1	9000	170.75	17.3
20802781	2011	6	1	9010	170.41	17.32
20802781	2011	6	1	9020	171.36	17.33
20802781	2011	6	1	9030	171.26	17.35
20802781	2011	6	1	9040	171.09	17.36
20802781	2011	6	1	9050	170.89	17.36
20802781	2011	6	1	9060	170.64	17.36
20802781	2011	6	1	9070	170.55	17.38
20802781	2011	6	1	9080	169.01	17.39

20802781	2011	6	1	9090	167.49	17.4
20802781	2011	6	1	9100	165.99	17.42
20802781	2011	6	1	9110	164.1	17.43
20802781	2011	6	1	9120	162.2	17.44
20802781	2011	6	1	9130	160.42	17.46
20802781	2011	6	1	9140	158.57	17.47
20802781	2011	6	1	9150	156.58	17.5
20802781	2011	6	1	9160	154.37	17.53
20802781	2011	6	1	9170	152.35	17.57
20802781	2011	6	1	9180	150.46	17.64
20802781	2011	6	1	9190	148.35	17.71
20802781	2011	6	1	9200	146.33	17.76
20802781	2011	6	1	9210	145.08	17.82
20802781	2011	6	1	9220	143.63	17.88
20802781	2011	6	1	9230	141.65	17.94
20802781	2011	6	1	9240	140.04	18.02
20802781	2011	6	1	9250	138.41	18.1
20802781	2011	6	1	9260	136.43	18.17
20802781	2011	6	1	9270	134.1	18.25
20802781	2011	6	1	9280	131.76	18.36
20802781	2011	6	1	9290	129.8	18.47
20802781	2011	6	1	9300	128.96	18.55
20802781	2011	6	1	9310	127.75	18.7
20802781	2011	6	1	9320	125.79	18.78
20802781	2011	6	1	9330	123.69	18.85
20802781	2011	6	1	9340	121.32	18.94
20802781	2011	6	1	9350	118.67	19.06
20802781	2011	6	1	9360	115.92	19.13
20802781	2011	6	1	9370	113.3	19.24
20802781	2011	6	1	9380	111.09	19.35
20802781	2011	6	1	9390	108.89	19.46
20802781	2011	6	1	9400	106.63	19.57
20802781	2011	6	1	9410	104.28	19.71
20802781	2011	6	1	9420	103.03	19.83
20802781	2011	6	1	9430	101.42	19.99
20802781	2011	6	1	9440	99.46	20.16
20802781	2011	6	1	9450	97.01	20.3
20802781	2011	6	1	9460	94.25	20.49
20802781	2011	6	1	9470	92.14	20.67
20802781	2011	6	1	9480	89.52	20.88
20802781	2011	6	1	9490	86.88	21.03
20802781	2011	6	1	9500	84.18	21.18
20802781	2011	6	1	9510	81.35	21.34
20802781	2011	6	1	9520	78.5	21.51
20802781	2011	6	1	9530	75.83	21.78
20802781	2011	6	1	9540	73.49	21.92
20802781	2011	6	1	9550	70.57	22.03

20802781	2011	6	1	9560	67.58	22.17
20802781	2011	6	1	9570	65.74	22.28
20802781	2011	6	1	9580	64.92	22.44
20802781	2011	6	1	9590	64.17	22.57
20802781	2011	6	1	9600	64.07	22.65
20802781	2011	6	1	9610	63.89	22.76
20802781	2011	6	1	9620	64.06	22.85
20802781	2011	6	1	9630	64.35	22.93
20802781	2011	6	1	9640	63.71	23.04
20802781	2011	6	1	9650	63.17	23.16
20802781	2011	6	1	9660	61.76	23.28
20802781	2011	6	1	9670	60.46	23.39
20802781	2011	6	1	9680	60.07	23.46
20802781	2011	6	1	9690	58.91	23.53
20802781	2011	6	1	9700	57.83	23.67
20802781	2011	6	1	9710	56.08	23.74
20802781	2011	6	1	9720	54.12	23.82
20802781	2011	6	1	9730	52.97	23.91
20802781	2011	6	1	9740	51.51	23.99
20802781	2011	6	1	9750	50.13	24.09
20802781	2011	6	1	9760	48.78	24.15
20802781	2011	6	1	9770	47.67	24.22
20802781	2011	6	1	9780	46.68	24.37
20802781	2011	6	1	9790	46.46	24.52
20802781	2011	6	1	9800	45.64	24.7
20802781	2011	6	1	9810	45.33	24.81
20802781	2011	6	1	9820	45.54	24.93
20802781	2011	6	1	9830	45.57	25.09
20802781	2011	6	1	9840	45.4	25.27
20802781	2011	6	1	9850	47.19	25.39
20802781	2011	6	1	9860	48.89	25.53
20802781	2011	6	1	9870	49.2	25.64
20802781	2011	6	1	9880	50.3	25.73
20802781	2011	6	1	9890	49.94	25.76
20802781	2011	6	1	9900	50.16	25.78
20802781	2011	6	1	9910	50.11	25.82
20802781	2011	6	1	9920	50.07	25.82
20802781	2011	6	1	9930	50.02	25.86
20802781	2011	6	1	9940	50.05	25.89
20802781	2011	6	1	9950	50.14	25.92
20802781	2011	6	1	9960	49.91	25.94
20802781	2011	6	1	9970	49.78	25.96
20802781	2011	6	1	9980	50.14	25.99
20802781	2011	6	1	9990	50.18	26
20802781	2011	6	1	10000	50.16	26.02
20802781	2011	6	1	10010	50.21	26.01
20802781	2011	6	1	10020	50.42	26.02

20802781	2011	6	1	10030	50.2	26.03
20802781	2011	6	1	10040	49.62	26.03
20802781	2011	6	1	10050	49.66	26.04
20802781	2011	6	1	10060	49.6	26.05
20802781	2011	6	1	10070	49.94	26.06
20802781	2011	6	1	10080	49.81	26.07
20802781	2011	6	1	10090	50.03	26.07
20802781	2011	6	1	10100	49.92	26.08
20802781	2011	6	1	10110	49.6	26.08
20802781	2011	6	1	10120	49.21	26.09
20802781	2011	6	1	10130	49.65	26.1
20802781	2011	6	1	10140	50.07	26.09
20802781	2011	6	1	10150	49.82	26.1
20802781	2011	6	1	10160	49.81	26.09
20802781	2011	6	1	10170	49.49	26.1
20802781	2011	6	1	10180	49.56	26.09
20802781	2011	6	1	10190	48.39	26.1
20802781	2011	6	1	10200	46.26	26.13
20802781	2011	6	1	10210	43.46	26.17
20802781	2011	6	1	10220	40.59	26.26
20802781	2011	6	1	10230	39.01	26.37
20802781	2011	6	1	10240	36.46	26.47
20802781	2011	6	1	10250	33.61	26.58
20802781	2011	6	1	10260	30.59	26.69
20802781	2011	6	1	10270	27.85	26.82
20802781	2011	6	1	10280	24.87	26.92
20802781	2011	6	1	10290	22.41	27.03
20802781	2011	6	1	10300	19.92	27.1
20802781	2011	6	1	10310	17.26	27.2
20802781	2011	6	1	10320	15.06	27.31
20802781	2011	6	1	10330	12.51	27.38
20802781	2011	6	1	10340	9.88	27.46
20802781	2011	6	1	10350	7.13	27.53
20802781	2011	6	1	10360	4.83	27.62
20802781	2011	6	1	10370	2.81	27.68
20802781	2011	6	1	10380	1.63	27.76
20802781	2011	6	1	10390	1.03	27.84
20802781	2011	6	1	10400	0.94	27.88
20802781	2011	6	1	10410	0.89	27.9
20802781	2011	6	1	10420	0.88	27.93
20802781	2011	6	1	10430	0.9	27.97
20802781	2011	6	1	10440	0.68	28.01
20802781	2011	6	1	10450	0.59	28.07
20802781	2011	6	1	10460	0.61	28.11
20802781	2011	6	1	10470	0.58	28.19
20802781	2011	6	1	10480	0.59	28.22
20802781	2011	6	1	10490	0.57	28.28

20802781	2011	6	1	10500	0.63	28.31
20802781	2011	6	1	10510	0.58	28.37
20802781	2011	6	1	10520	0.59	28.4
20802781	2011	6	1	10530	0.6	28.43
20802781	2011	6	1	10540	0.61	28.45
20802781	2011	6	1	10550	0.56	28.46
20802781	2011	6	1	10560	0.49	28.5
20802781	2011	6	1	10570	0.49	28.52
20802781	2011	6	1	10580	0.48	28.53
20817391	2011	6	1	0	1.38	29.14
20817391	2011	6	1	10	2.73	29.14
20817391	2011	6	1	20	3.15	29.14
20817391	2011	6	1	30	2.96	29.14
20817391	2011	6	1	40	2.81	29.14
20817391	2011	6	1	50	3.27	29.14
20817391	2011	6	1	60	2.65	29.14
20817391	2011	6	1	70	2.65	29.14
20817391	2011	6	1	80	2.97	29.13
20817391	2011	6	1	90	2.57	29.13
20817391	2011	6	1	100	2.74	29.11
20817391	2011	6	1	110	2.88	29.11
20817391	2011	6	1	120	3.52	29.08
20817391	2011	6	1	130	5.09	29.07
20817391	2011	6	1	140	7.17	29.02
20817391	2011	6	1	150	8.57	28.99
20817391	2011	6	1	160	11.14	28.93
20817391	2011	6	1	170	12.85	28.92
20817391	2011	6	1	180	13.57	28.9
20817391	2011	6	1	190	13.96	28.88
20817391	2011	6	1	200	14.55	28.85
20817391	2011	6	1	210	16.95	28.82
20817391	2011	6	1	220	18.82	28.79
20817391	2011	6	1	230	19.87	28.77
20817391	2011	6	1	240	20.01	28.75
20817391	2011	6	1	250	19.68	28.74
20817391	2011	6	1	260	19.93	28.73
20817391	2011	6	1	270	20.07	28.71
20817391	2011	6	1	280	19.94	28.7
20817391	2011	6	1	290	19.56	28.69
20817391	2011	6	1	300	18.8	28.68
20817391	2011	6	1	310	17.96	28.67
20817391	2011	6	1	320	17.58	28.66
20817391	2011	6	1	330	18.79	28.64
20817391	2011	6	1	340	20.15	28.64
20817391	2011	6	1	350	20.27	28.62
20817391	2011	6	1	360	20	28.62
20817391	2011	6	1	370	19.06	28.63

20817391	2011	6	1	380	18.56	28.63
20817391	2011	6	1	390	17.95	28.62
20817391	2011	6	1	400	16.56	28.64
20817391	2011	6	1	410	14.51	28.64
20817391	2011	6	1	420	12.84	28.66
20817391	2011	6	1	430	12.4	28.64
20817391	2011	6	1	440	13.03	28.64
20817391	2011	6	1	450	14.91	28.61
20817391	2011	6	1	460	17.83	28.59
20817391	2011	6	1	470	20.22	28.56
20817391	2011	6	1	480	21.5	28.54
20817391	2011	6	1	490	22.07	28.55
20817391	2011	6	1	500	22.6	28.54
20817391	2011	6	1	510	23.42	28.53
20817391	2011	6	1	520	24.38	28.52
20817391	2011	6	1	530	26.32	28.5
20817391	2011	6	1	540	28.83	28.47
20817391	2011	6	1	550	30.59	28.45
20817391	2011	6	1	560	31.65	28.45
20817391	2011	6	1	570	33.11	28.43
20817391	2011	6	1	580	35.3	28.41
20817391	2011	6	1	590	37.13	28.39
20817391	2011	6	1	600	39.56	28.37
20817391	2011	6	1	610	41.11	28.36
20817391	2011	6	1	620	43.41	28.35
20817391	2011	6	1	630	45.9	28.32
20817391	2011	6	1	640	47.17	28.31
20817391	2011	6	1	650	48	28.31
20817391	2011	6	1	660	48.98	28.28
20817391	2011	6	1	670	50.75	28.23
20817391	2011	6	1	680	51.35	28.21
20817391	2011	6	1	690	51.16	28.19
20817391	2011	6	1	700	51.03	28.12
20817391	2011	6	1	710	51.18	27.98
20817391	2011	6	1	720	51.14	27.9
20817391	2011	6	1	730	51.14	27.79
20817391	2011	6	1	740	51.19	27.72
20817391	2011	6	1	750	51.2	27.63
20817391	2011	6	1	760	51.14	27.55
20817391	2011	6	1	770	51.12	27.47
20817391	2011	6	1	780	51.24	27.4
20817391	2011	6	1	790	51.3	27.34
20817391	2011	6	1	800	51.2	27.28
20817391	2011	6	1	810	51.24	27.21
20817391	2011	6	1	820	51.26	27.14
20817391	2011	6	1	830	51.2	27.07
20817391	2011	6	1	840	51.24	27.03

20817391	2011	6	1	850	51.16	27
20817391	2011	6	1	860	50.7	26.97
20817391	2011	6	1	870	50.88	26.94
20817391	2011	6	1	880	50.88	26.9
20817391	2011	6	1	890	50.82	26.88
20817391	2011	6	1	900	50.91	26.83
20817391	2011	6	1	910	50.86	26.79
20817391	2011	6	1	920	50.83	26.75
20817391	2011	6	1	930	50.77	26.71
20817391	2011	6	1	940	50.84	26.68
20817391	2011	6	1	950	50.79	26.64
20817391	2011	6	1	960	50.87	26.61
20817391	2011	6	1	970	50.77	26.59
20817391	2011	6	1	980	50.82	26.57
20817391	2011	6	1	990	51.44	26.52
20817391	2011	6	1	1000	52.08	26.5
20817391	2011	6	1	1010	52.71	26.48
20817391	2011	6	1	1020	53.44	26.44
20817391	2011	6	1	1030	54.02	26.4
20817391	2011	6	1	1040	54.17	26.38
20817391	2011	6	1	1050	54.19	26.35
20817391	2011	6	1	1060	54.14	26.32
20817391	2011	6	1	1070	54.27	26.3
20817391	2011	6	1	1080	54.1	26.26
20817391	2011	6	1	1090	53.94	26.24
20817391	2011	6	1	1100	53.91	26.21
20817391	2011	6	1	1110	53.79	26.17
20817391	2011	6	1	1120	53.68	26.13
20817391	2011	6	1	1130	53.64	26.1
20817391	2011	6	1	1140	53.62	26.07
20817391	2011	6	1	1150	53.68	26.05
20817391	2011	6	1	1160	53.53	26.03
20817391	2011	6	1	1170	53.43	26.02
20817391	2011	6	1	1180	53.68	26
20817391	2011	6	1	1190	54.19	25.98
20817391	2011	6	1	1200	54.71	25.96
20817391	2011	6	1	1210	54.27	25.94
20817391	2011	6	1	1220	52.75	25.94
20817391	2011	6	1	1230	52.15	25.92
20817391	2011	6	1	1240	52.52	25.91
20817391	2011	6	1	1250	52.91	25.89
20817391	2011	6	1	1260	53.03	25.87
20817391	2011	6	1	1270	52.93	25.86
20817391	2011	6	1	1280	51.32	25.87
20817391	2011	6	1	1290	49.25	25.87
20817391	2011	6	1	1300	47.34	25.88
20817391	2011	6	1	1310	45.73	25.92

20817391	2011	6	1	1320	45.02	26
20817391	2011	6	1	1330	44.14	26.07
20817391	2011	6	1	1340	44.19	26.12
20817391	2011	6	1	1350	43.88	26.22
20817391	2011	6	1	1360	42.95	26.32
20817391	2011	6	1	1370	42.64	26.4
20817391	2011	6	1	1380	42.94	26.47
20817391	2011	6	1	1390	43.32	26.61
20817391	2011	6	1	1400	43.36	26.65
20817391	2011	6	1	1410	43.42	26.71
20817391	2011	6	1	1420	44.08	26.81
20817391	2011	6	1	1430	43.89	26.9
20817391	2011	6	1	1440	43.71	26.95
20817391	2011	6	1	1450	44.12	27.03
20817391	2011	6	1	1460	44.11	27.1
20817391	2011	6	1	1470	43.86	27.15
20817391	2011	6	1	1480	44.19	27.22
20817391	2011	6	1	1490	44.22	27.28
20817391	2011	6	1	1500	44.15	27.32
20817391	2011	6	1	1510	44.18	27.35
20817391	2011	6	1	1520	44.04	27.39
20817391	2011	6	1	1530	43.99	27.46
20817391	2011	6	1	1540	44.17	27.5
20817391	2011	6	1	1550	44.25	27.54
20817391	2011	6	1	1560	44.46	27.57
20817391	2011	6	1	1570	44.96	27.61
20817391	2011	6	1	1580	45.31	27.64
20817391	2011	6	1	1590	45.15	27.67
20817391	2011	6	1	1600	44.9	27.7
20817391	2011	6	1	1610	44.71	27.73
20817391	2011	6	1	1620	44.73	27.75
20817391	2011	6	1	1630	45	27.77
20817391	2011	6	1	1640	45.04	27.81
20817391	2011	6	1	1650	44.78	27.82
20817391	2011	6	1	1660	45	27.83
20817391	2011	6	1	1670	45.63	27.85
20817391	2011	6	1	1680	46.07	27.88
20817391	2011	6	1	1690	46.61	27.89
20817391	2011	6	1	1700	47.08	27.91
20817391	2011	6	1	1710	47.17	27.92
20817391	2011	6	1	1720	47.36	27.93
20817391	2011	6	1	1730	47.29	27.94
20817391	2011	6	1	1740	47.24	27.96
20817391	2011	6	1	1750	47.56	27.96
20817391	2011	6	1	1760	47.97	27.97
20817391	2011	6	1	1770	48.02	27.99
20817391	2011	6	1	1780	48.04	28

20817391	2011	6	1	1790	48.3	28
20817391	2011	6	1	1800	48.62	28.01
20817391	2011	6	1	1810	48.4	27.99
20817391	2011	6	1	1820	47.1	27.98
20817391	2011	6	1	1830	46.96	27.98
20817391	2011	6	1	1840	47.27	27.97
20817391	2011	6	1	1850	46.61	27.97
20817391	2011	6	1	1860	45.36	27.99
20817391	2011	6	1	1870	45.05	27.99
20817391	2011	6	1	1880	45.45	28
20817391	2011	6	1	1890	46.19	28
20817391	2011	6	1	1900	47.72	27.98
20817391	2011	6	1	1910	50.12	27.94
20817391	2011	6	1	1920	50.99	27.93
20817391	2011	6	1	1930	51.22	27.89
20817391	2011	6	1	1940	50.76	27.82
20817391	2011	6	1	1950	50.94	27.73
20817391	2011	6	1	1960	51.24	27.63
20817391	2011	6	1	1970	51.77	27.45
20817391	2011	6	1	1980	52.07	27.39
20817391	2011	6	1	1990	52.22	27.32
20817391	2011	6	1	2000	52.01	27.26
20817391	2011	6	1	2010	52.19	27.21
20817391	2011	6	1	2020	52.25	27.14
20817391	2011	6	1	2030	52.2	27.08
20817391	2011	6	1	2040	51.86	27.03
20817391	2011	6	1	2050	51.62	26.97
20817391	2011	6	1	2060	51.57	26.93
20817391	2011	6	1	2070	51.64	26.89
20817391	2011	6	1	2080	51.55	26.86
20817391	2011	6	1	2090	51.66	26.82
20817391	2011	6	1	2100	51.57	26.79
20817391	2011	6	1	2110	51.53	26.78
20817391	2011	6	1	2120	51.5	26.75
20817391	2011	6	1	2130	52.04	26.71
20817391	2011	6	1	2140	52.19	26.7
20817391	2011	6	1	2150	52.35	26.67
20817391	2011	6	1	2160	52.86	26.66
20817391	2011	6	1	2170	53.39	26.57
20817391	2011	6	1	2180	53.79	26.52
20817391	2011	6	1	2190	54.01	26.4
20817391	2011	6	1	2200	55.62	26.35
20817391	2011	6	1	2210	56.6	26.32
20817391	2011	6	1	2220	56.74	26.23
20817391	2011	6	1	2230	55.98	26.21
20817391	2011	6	1	2240	55.79	26.2
20817391	2011	6	1	2250	56	26.14

20817391	2011	6	1	2260	56.46	26.08
20817391	2011	6	1	2270	56.25	26.04
20817391	2011	6	1	2280	55.85	26.03
20817391	2011	6	1	2290	55.8	26.01
20817391	2011	6	1	2300	56.09	25.99
20817391	2011	6	1	2310	56.05	25.96
20817391	2011	6	1	2320	55.88	25.95
20817391	2011	6	1	2330	55.28	25.92
20817391	2011	6	1	2340	54.75	25.91
20817391	2011	6	1	2350	53.89	25.89
20817391	2011	6	1	2360	54.04	25.86
20817391	2011	6	1	2370	54.09	25.85
20817391	2011	6	1	2380	54.03	25.85
20817391	2011	6	1	2390	53.93	25.83
20817391	2011	6	1	2400	54.15	25.82
20817391	2011	6	1	2410	56.48	25.79
20817391	2011	6	1	2420	59.61	25.75
20817391	2011	6	1	2430	63.14	25.69
20817391	2011	6	1	2440	66.63	25.58
20817391	2011	6	1	2450	69.59	25.5
20817391	2011	6	1	2460	72.84	25.42
20817391	2011	6	1	2470	76.17	25.3
20817391	2011	6	1	2480	79.35	25.22
20817391	2011	6	1	2490	81.89	25.17
20817391	2011	6	1	2500	84.3	25.12
20817391	2011	6	1	2510	85.98	25.1
20817391	2011	6	1	2520	86.77	25.07
20817391	2011	6	1	2530	86.96	25.04
20817391	2011	6	1	2540	87.47	25.02
20817391	2011	6	1	2550	87.64	24.99
20817391	2011	6	1	2560	87.16	24.96
20817391	2011	6	1	2570	87.11	24.94
20817391	2011	6	1	2580	87.03	24.9
20817391	2011	6	1	2590	86.43	24.89
20817391	2011	6	1	2600	86.08	24.86
20817391	2011	6	1	2610	85.95	24.83
20817391	2011	6	1	2620	85.95	24.81
20817391	2011	6	1	2630	86	24.78
20817391	2011	6	1	2640	85.94	24.77
20817391	2011	6	1	2650	85.92	24.74
20817391	2011	6	1	2660	85.89	24.73
20817391	2011	6	1	2670	85.87	24.7
20817391	2011	6	1	2680	85.84	24.68
20817391	2011	6	1	2690	85.86	24.66
20817391	2011	6	1	2700	85.82	24.65
20817391	2011	6	1	2710	85.78	24.64
20817391	2011	6	1	2720	85.84	24.61

20817391	2011	6	1	2730	85.89	24.6
20817391	2011	6	1	2740	85.81	24.59
20817391	2011	6	1	2750	85.79	24.57
20817391	2011	6	1	2760	85.73	24.56
20817391	2011	6	1	2770	85.71	24.55
20817391	2011	6	1	2780	85.78	24.53
20817391	2011	6	1	2790	85.94	24.51
20817391	2011	6	1	2800	85.72	24.5
20817391	2011	6	1	2810	85.81	24.5
20817391	2011	6	1	2820	86.13	24.48
20817391	2011	6	1	2830	86.09	24.46
20817391	2011	6	1	2840	86.01	24.45
20817391	2011	6	1	2850	86.08	24.45
20817391	2011	6	1	2860	86.33	24.43
20817391	2011	6	1	2870	87.45	24.42
20817391	2011	6	1	2880	88.79	24.4
20817391	2011	6	1	2890	91.39	24.36
20817391	2011	6	1	2900	93.22	24.35
20817391	2011	6	1	2910	93.94	24.33
20817391	2011	6	1	2920	94.22	24.3
20817391	2011	6	1	2930	95.66	24.28
20817391	2011	6	1	2940	96.57	24.25
20817391	2011	6	1	2950	96.96	24.22
20817391	2011	6	1	2960	97.24	24.14
20817391	2011	6	1	2970	97.81	24.11
20817391	2011	6	1	2980	99.9	24.07
20817391	2011	6	1	2990	102.35	24.01
20817391	2011	6	1	3000	104.86	23.92
20817391	2011	6	1	3010	107.44	23.8
20817391	2011	6	1	3020	110.08	23.68
20817391	2011	6	1	3030	112.71	23.57
20817391	2011	6	1	3040	115.58	23.46
20817391	2011	6	1	3050	118.52	23.36
20817391	2011	6	1	3060	121.26	23.31
20817391	2011	6	1	3070	123.9	23.21
20817391	2011	6	1	3080	126.59	23.07
20817391	2011	6	1	3090	129.41	22.95
20817391	2011	6	1	3100	131.21	22.78
20817391	2011	6	1	3110	133.3	22.6
20817391	2011	6	1	3120	135.45	22.45
20817391	2011	6	1	3130	137.54	22.28
20817391	2011	6	1	3140	139.62	22.2
20817391	2011	6	1	3150	141.91	22.06
20817391	2011	6	1	3160	143.57	21.99
20817391	2011	6	1	3170	145.73	21.92
20817391	2011	6	1	3180	147.52	21.82
20817391	2011	6	1	3190	148.58	21.74

20817391	2011	6	1	3200	149.08	21.62
20817391	2011	6	1	3210	149.45	21.53
20817391	2011	6	1	3220	149.22	21.44
20817391	2011	6	1	3230	147.77	21.35
20817391	2011	6	1	3240	146.68	21.27
20817391	2011	6	1	3250	145.86	21.18
20817391	2011	6	1	3260	144.95	21.1
20817391	2011	6	1	3270	144.57	21.06
20817391	2011	6	1	3280	144.47	21
20817391	2011	6	1	3290	145.35	20.94
20817391	2011	6	1	3300	146.64	20.85
20817391	2011	6	1	3310	146.74	20.8
20817391	2011	6	1	3320	146.9	20.77
20817391	2011	6	1	3330	146.94	20.73
20817391	2011	6	1	3340	146.81	20.67
20817391	2011	6	1	3350	146.79	20.64
20817391	2011	6	1	3360	146.46	20.61
20817391	2011	6	1	3370	146.12	20.55
20817391	2011	6	1	3380	146.04	20.47
20817391	2011	6	1	3390	146.66	20.44
20817391	2011	6	1	3400	148.5	20.41
20817391	2011	6	1	3410	149.33	20.36
20817391	2011	6	1	3420	149.92	20.3
20817391	2011	6	1	3430	150.21	20.28
20817391	2011	6	1	3440	150.58	20.25
20817391	2011	6	1	3450	150.92	20.24
20817391	2011	6	1	3460	151.46	20.21
20817391	2011	6	1	3470	151.91	20.17
20817391	2011	6	1	3480	153.13	20.11
20817391	2011	6	1	3490	153.64	20.07
20817391	2011	6	1	3500	153.78	20.01
20817391	2011	6	1	3510	155.07	19.98
20817391	2011	6	1	3520	156.35	19.95
20817391	2011	6	1	3530	157.58	19.9
20817391	2011	6	1	3540	158.69	19.86
20817391	2011	6	1	3550	159.38	19.8
20817391	2011	6	1	3560	159.68	19.75
20817391	2011	6	1	3570	159.84	19.71
20817391	2011	6	1	3580	159.87	19.67
20817391	2011	6	1	3590	159.93	19.63
20817391	2011	6	1	3600	160.01	19.6
20817391	2011	6	1	3610	160.14	19.56
20817391	2011	6	1	3620	160.35	19.53
20817391	2011	6	1	3630	160.53	19.49
20817391	2011	6	1	3640	160.54	19.45
20817391	2011	6	1	3650	159.35	19.4
20817391	2011	6	1	3660	159.01	19.38

20817391	2011	6	1	3670	159.33	19.35
20817391	2011	6	1	3680	159.3	19.32
20817391	2011	6	1	3690	159.23	19.3
20817391	2011	6	1	3700	159.3	19.27
20817391	2011	6	1	3710	159.45	19.25
20817391	2011	6	1	3720	159.49	19.24
20817391	2011	6	1	3730	159.48	19.22
20817391	2011	6	1	3740	159.27	19.19
20817391	2011	6	1	3750	159.29	19.17
20817391	2011	6	1	3760	159.25	19.15
20817391	2011	6	1	3770	159.21	19.14
20817391	2011	6	1	3780	159.24	19.12
20817391	2011	6	1	3790	159.3	19.1
20817391	2011	6	1	3800	159.39	19.08
20817391	2011	6	1	3810	159.37	19.05
20817391	2011	6	1	3820	159.42	19.02
20817391	2011	6	1	3830	159.34	18.96
20817391	2011	6	1	3840	159.3	18.93
20817391	2011	6	1	3850	159.28	18.9
20817391	2011	6	1	3860	159.22	18.87
20817391	2011	6	1	3870	159.27	18.84
20817391	2011	6	1	3880	159.35	18.81
20817391	2011	6	1	3890	159.26	18.77
20817391	2011	6	1	3900	159.2	18.74
20817391	2011	6	1	3910	159.21	18.72
20817391	2011	6	1	3920	159.31	18.68
20817391	2011	6	1	3930	159.24	18.65
20817391	2011	6	1	3940	159.21	18.64
20817391	2011	6	1	3950	159.31	18.62
20817391	2011	6	1	3960	159.25	18.59
20817391	2011	6	1	3970	159.12	18.58
20817391	2011	6	1	3980	159.09	18.57
20817391	2011	6	1	3990	159.25	18.55
20817391	2011	6	1	4000	159.28	18.54
20817391	2011	6	1	4010	159.17	18.54
20817391	2011	6	1	4020	158.72	18.53
20817391	2011	6	1	4030	157.81	18.54
20817391	2011	6	1	4040	157.9	18.54
20817391	2011	6	1	4050	158.28	18.54
20817391	2011	6	1	4060	158.38	18.55
20817391	2011	6	1	4070	158.04	18.55
20817391	2011	6	1	4080	157.66	18.56
20817391	2011	6	1	4090	157.49	18.57
20817391	2011	6	1	4100	156.93	18.58
20817391	2011	6	1	4110	155.77	18.6
20817391	2011	6	1	4120	153.83	18.6
20817391	2011	6	1	4130	153.31	18.61

20817391	2011	6	1	4140	152.69	18.63
20817391	2011	6	1	4150	151.71	18.66
20817391	2011	6	1	4160	150	18.69
20817391	2011	6	1	4170	148.25	18.74
20817391	2011	6	1	4180	146.58	18.78
20817391	2011	6	1	4190	145	18.82
20817391	2011	6	1	4200	143.3	18.87
20817391	2011	6	1	4210	141.82	18.95
20817391	2011	6	1	4220	140.3	19.07
20817391	2011	6	1	4230	138.8	19.13
20817391	2011	6	1	4240	138.21	19.16
20817391	2011	6	1	4250	137.82	19.22
20817391	2011	6	1	4260	136.35	19.31
20817391	2011	6	1	4270	134.76	19.37
20817391	2011	6	1	4280	133.15	19.42
20817391	2011	6	1	4290	131.78	19.49
20817391	2011	6	1	4300	130.96	19.52
20817391	2011	6	1	4310	130.59	19.55
20817391	2011	6	1	4320	130.02	19.6
20817391	2011	6	1	4330	129.04	19.63
20817391	2011	6	1	4340	128.38	19.67
20817391	2011	6	1	4350	127.6	19.7
20817391	2011	6	1	4360	126.56	19.73
20817391	2011	6	1	4370	124.94	19.78
20817391	2011	6	1	4380	124.45	19.8
20817391	2011	6	1	4390	125.15	19.83
20817391	2011	6	1	4400	125.67	19.86
20817391	2011	6	1	4410	125.63	19.89
20817391	2011	6	1	4420	124.12	19.95
20817391	2011	6	1	4430	121.77	19.97
20817391	2011	6	1	4440	119.18	20.01
20817391	2011	6	1	4450	116.6	20.09
20817391	2011	6	1	4460	114.13	20.16
20817391	2011	6	1	4470	111.72	20.24
20817391	2011	6	1	4480	109.16	20.32
20817391	2011	6	1	4490	106.78	20.41
20817391	2011	6	1	4500	105.15	20.58
20817391	2011	6	1	4510	103.65	20.71
20817391	2011	6	1	4520	101.97	20.83
20817391	2011	6	1	4530	100.25	20.98
20817391	2011	6	1	4540	98.58	21.1
20817391	2011	6	1	4550	97.14	21.21
20817391	2011	6	1	4560	96.76	21.35
20817391	2011	6	1	4570	96.7	21.45
20817391	2011	6	1	4580	96.2	21.57
20817391	2011	6	1	4590	94.99	21.66
20817391	2011	6	1	4600	93.62	21.75

20817391	2011	6	1	4610	92.14	21.85
20817391	2011	6	1	4620	90.52	21.93
20817391	2011	6	1	4630	88.78	22.03
20817391	2011	6	1	4640	86.95	22.13
20817391	2011	6	1	4650	85.47	22.21
20817391	2011	6	1	4660	85.2	22.34
20817391	2011	6	1	4670	83.86	22.44
20817391	2011	6	1	4680	82.07	22.53
20817391	2011	6	1	4690	80.3	22.64
20817391	2011	6	1	4700	78.69	22.75
20817391	2011	6	1	4710	77.1	22.85
20817391	2011	6	1	4720	75.47	22.93
20817391	2011	6	1	4730	73.79	23.02
20817391	2011	6	1	4740	72.09	23.12
20817391	2011	6	1	4750	70.43	23.18
20817391	2011	6	1	4760	68.62	23.25
20817391	2011	6	1	4770	66.81	23.3
20817391	2011	6	1	4780	65.12	23.37
20817391	2011	6	1	4790	63.58	23.46
20817391	2011	6	1	4800	61.89	23.53
20817391	2011	6	1	4810	60.28	23.58
20817391	2011	6	1	4820	58.74	23.66
20817391	2011	6	1	4830	57.1	23.71
20817391	2011	6	1	4840	55.41	23.77
20817391	2011	6	1	4850	53.63	23.85
20817391	2011	6	1	4860	51.94	23.93
20817391	2011	6	1	4870	50.56	24.03
20817391	2011	6	1	4880	49.28	24.15
20817391	2011	6	1	4890	49.09	24.32
20817391	2011	6	1	4900	49.45	24.52
20817391	2011	6	1	4910	49.92	24.75
20817391	2011	6	1	4920	50.49	24.88
20817391	2011	6	1	4930	50.58	25.01
20817391	2011	6	1	4940	50.36	25.04
20817391	2011	6	1	4950	50.58	25.06
20817391	2011	6	1	4960	49.96	25.11
20817391	2011	6	1	4970	49.38	25.17
20817391	2011	6	1	4980	49.2	25.22
20817391	2011	6	1	4990	48.99	25.32
20817391	2011	6	1	5000	49.14	25.46
20817391	2011	6	1	5010	48.98	25.58
20817391	2011	6	1	5020	48.89	25.71
20817391	2011	6	1	5030	48.98	25.82
20817391	2011	6	1	5040	49.6	25.92
20817391	2011	6	1	5050	50.36	25.99
20817391	2011	6	1	5060	51.05	26.03
20817391	2011	6	1	5070	51.41	26.06

20817391	2011	6	1	5080	51.06	26.07
20817391	2011	6	1	5090	51.24	26.1
20817391	2011	6	1	5100	51.27	26.08
20817391	2011	6	1	5110	50.88	26.03
20817391	2011	6	1	5120	49.41	26
20817391	2011	6	1	5130	47.74	26.02
20817391	2011	6	1	5140	46.28	26.11
20817391	2011	6	1	5150	44.93	26.21
20817391	2011	6	1	5160	43.6	26.33
20817391	2011	6	1	5170	42.27	26.46
20817391	2011	6	1	5180	40.88	26.59
20817391	2011	6	1	5190	39.46	26.73
20817391	2011	6	1	5200	37.98	26.82
20817391	2011	6	1	5210	37.36	26.89
20817391	2011	6	1	5220	37.12	27
20817391	2011	6	1	5230	36	27.09
20817391	2011	6	1	5240	34.58	27.16
20817391	2011	6	1	5250	33.85	27.21
20817391	2011	6	1	5260	33.14	27.28
20817391	2011	6	1	5270	32.16	27.34
20817391	2011	6	1	5280	32.22	27.38
20817391	2011	6	1	5290	32.55	27.42
20817391	2011	6	1	5300	32.57	27.47
20817391	2011	6	1	5310	32.35	27.53
20817391	2011	6	1	5320	32.37	27.59
20817391	2011	6	1	5330	32.53	27.63
20817391	2011	6	1	5340	32.48	27.67
20817391	2011	6	1	5350	32.48	27.71
20817391	2011	6	1	5360	32.25	27.75
20817391	2011	6	1	5370	31.83	27.78
20817391	2011	6	1	5380	31.59	27.82
20817391	2011	6	1	5390	31.48	27.85
20817391	2011	6	1	5400	31.98	27.88
20817391	2011	6	1	5410	31.9	27.9
20817391	2011	6	1	5420	31.91	27.93
20817391	2011	6	1	5430	31.89	27.94
20817391	2011	6	1	5440	31.84	27.95
20817391	2011	6	1	5450	31.78	27.98
20817391	2011	6	1	5460	31.93	28
20817391	2011	6	1	5470	31.78	28.02
20817391	2011	6	1	5480	31.81	28.03
20817391	2011	6	1	5490	31.77	28.04
20817391	2011	6	1	5500	31.81	28.07
20817391	2011	6	1	5510	31.78	28.08
20817391	2011	6	1	5520	31.75	28.11
20817391	2011	6	1	5530	31.95	28.12
20817391	2011	6	1	5540	31.84	28.14

20817391	2011	6	1	5550	31.7	28.15
20817391	2011	6	1	5560	31.88	28.17
20817391	2011	6	1	5570	32.03	28.18
20817391	2011	6	1	5580	31.85	28.19
20817391	2011	6	1	5590	31.87	28.2
20817391	2011	6	1	5600	31.87	28.2
20817391	2011	6	1	5610	31.86	28.21
20817391	2011	6	1	5620	31.85	28.22
20817391	2011	6	1	5630	31.71	28.22
20817391	2011	6	1	5640	31.88	28.24
20817391	2011	6	1	5650	31.89	28.25
20817391	2011	6	1	5660	31.75	28.26
20817391	2011	6	1	5670	31.86	28.25
20817391	2011	6	1	5680	31.85	28.26
20817391	2011	6	1	5690	31.9	28.27
20817391	2011	6	1	5700	31.87	28.28
20817391	2011	6	1	5710	31.91	28.28
20817391	2011	6	1	5720	31.73	28.29
20817391	2011	6	1	5730	31.88	28.29
20817391	2011	6	1	5740	31.9	28.29
20817391	2011	6	1	5750	31.98	28.3
20817391	2011	6	1	5760	31.84	28.31
20817391	2011	6	1	5770	31.88	28.32
20817391	2011	6	1	5780	31.91	28.32
20817391	2011	6	1	5790	31.94	28.32
20817391	2011	6	1	5800	31.9	28.33
20817391	2011	6	1	5810	31.74	28.33
20817391	2011	6	1	5820	31.84	28.33
20817391	2011	6	1	5830	32.47	28.33
20817391	2011	6	1	5840	33.1	28.32
20817391	2011	6	1	5850	32.77	28.33
20817391	2011	6	1	5860	31.5	28.34
20817391	2011	6	1	5870	29.88	28.35
20817391	2011	6	1	5880	28.31	28.37
20817391	2011	6	1	5890	26.67	28.38
20817391	2011	6	1	5900	25.17	28.4
20817391	2011	6	1	5910	23.69	28.42
20817391	2011	6	1	5920	22.27	28.42
20817391	2011	6	1	5930	20.84	28.44
20817391	2011	6	1	5940	19.36	28.46
20817391	2011	6	1	5950	17.93	28.47
20817391	2011	6	1	5960	16.47	28.48
20817391	2011	6	1	5970	14.68	28.5
20817391	2011	6	1	5980	12.84	28.52
20817391	2011	6	1	5990	10.89	28.53
20817391	2011	6	1	6000	8.88	28.56
20817391	2011	6	1	6010	6.73	28.57

20817391	2011	6	1	6020	4.69	28.6
20817391	2011	6	1	6030	3.12	28.63
20817391	2011	6	1	6040	2.07	28.65
20817391	2011	6	1	6050	1.84	28.66
20817391	2011	6	1	6060	1.85	28.67
20817391	2011	6	1	6070	1.64	28.69
20817391	2011	6	1	6080	1.75	28.7
20817391	2011	6	1	6090	1.78	28.72
20817391	2011	6	1	6100	0.6	28.77
20817391	2011	6	1	6110	0.58	28.79
20817391	2011	6	1	6120	0.57	28.81
20817391	2011	6	1	6130	0.56	28.83
20817391	2011	6	1	6140	0.58	28.86
20817391	2011	6	1	6150	0.55	28.9
20817391	2011	6	1	6160	0.56	28.92
20817391	2011	6	1	6170	0.58	28.93
20817391	2011	6	1	6180	0.53	28.96
20817391	2011	6	1	6190	0.56	28.97
20817391	2011	6	1	6200	0.48	29
20817391	2011	6	1	6210	0.5	29.02
20817391	2011	6	1	6220	0.51	29.04
20817391	2011	6	1	6230	0.5	29.06
20817391	2011	6	1	6240	0.49	29.07
20990381	2011	6	3	0	1.16	29.21
20990381	2011	6	3	10	1.65	29.22
20990381	2011	6	3	20	2.2	29.22
20990381	2011	6	3	30	2.36	29.22
20990381	2011	6	3	40	2.26	29.21
20990381	2011	6	3	50	2.34	29.21
20990381	2011	6	3	60	2.78	29.21
20990381	2011	6	3	70	3.01	29.21
20990381	2011	6	3	80	2.96	29.2
20990381	2011	6	3	90	2.74	29.2
20990381	2011	6	3	100	2.73	29.2
20990381	2011	6	3	110	2.94	29.19
20990381	2011	6	3	120	2.75	29.18
20990381	2011	6	3	130	2.43	29.19
20990381	2011	6	3	140	2.65	29.18
20990381	2011	6	3	150	3.13	29.17
20990381	2011	6	3	160	3.55	29.15
20990381	2011	6	3	170	5.15	29.12
20990381	2011	6	3	180	7.46	29.08
20990381	2011	6	3	190	9.25	29.04
20990381	2011	6	3	200	9.78	29.03
20990381	2011	6	3	210	10.41	29
20990381	2011	6	3	220	10.87	28.99
20990381	2011	6	3	230	11.57	28.97

20990381	2011	6	3	240	12.45	28.95
20990381	2011	6	3	250	13.28	28.94
20990381	2011	6	3	260	13.14	28.92
20990381	2011	6	3	270	13.01	28.92
20990381	2011	6	3	280	13.2	28.92
20990381	2011	6	3	290	13.61	28.9
20990381	2011	6	3	300	13.98	28.89
20990381	2011	6	3	310	14.46	28.87
20990381	2011	6	3	320	15.07	28.86
20990381	2011	6	3	330	15.56	28.85
20990381	2011	6	3	340	15.69	28.83
20990381	2011	6	3	350	15.97	28.82
20990381	2011	6	3	360	16.89	28.8
20990381	2011	6	3	370	18.8	28.77
20990381	2011	6	3	380	21.21	28.73
20990381	2011	6	3	390	23.1	28.69
20990381	2011	6	3	400	24.98	28.67
20990381	2011	6	3	410	27.31	28.64
20990381	2011	6	3	420	29.82	28.6
20990381	2011	6	3	430	32.17	28.57
20990381	2011	6	3	440	34.74	28.54
20990381	2011	6	3	450	37.31	28.51
20990381	2011	6	3	460	39.66	28.5
20990381	2011	6	3	470	41.74	28.46
20990381	2011	6	3	480	43.55	28.37
20990381	2011	6	3	490	45.91	28.23
20990381	2011	6	3	500	48.38	28
20990381	2011	6	3	510	51.14	27.85
20990381	2011	6	3	520	53.64	27.79
20990381	2011	6	3	530	55.29	27.67
20990381	2011	6	3	540	56.87	27.53
20990381	2011	6	3	550	59.67	27.39
20990381	2011	6	3	560	61.94	27.25
20990381	2011	6	3	570	64.07	27.1
20990381	2011	6	3	580	64.87	27.07
20990381	2011	6	3	590	66.44	26.92
20990381	2011	6	3	600	68.62	26.78
20990381	2011	6	3	610	71.02	26.57
20990381	2011	6	3	620	73.36	26.42
20990381	2011	6	3	630	75.64	26.29
20990381	2011	6	3	640	78.33	26.17
20990381	2011	6	3	650	81.61	26.1
20990381	2011	6	3	660	84.91	25.99
20990381	2011	6	3	670	87.99	25.83
20990381	2011	6	3	680	90.7	25.71
20990381	2011	6	3	690	93.73	25.65
20990381	2011	6	3	700	96.58	25.57

20990381	2011	6	3	710	99.1	25.45
20990381	2011	6	3	720	102.11	25.21
20990381	2011	6	3	730	105.47	25.13
20990381	2011	6	3	740	108.62	24.85
20990381	2011	6	3	750	111.71	24.76
20990381	2011	6	3	760	114.74	24.64
20990381	2011	6	3	770	117.38	24.51
20990381	2011	6	3	780	120.24	24.25
20990381	2011	6	3	790	123.02	24.07
20990381	2011	6	3	800	125	23.94
20990381	2011	6	3	810	126.78	23.8
20990381	2011	6	3	820	129.39	23.52
20990381	2011	6	3	830	131.16	23.44
20990381	2011	6	3	840	133.67	23.29
20990381	2011	6	3	850	136.61	23.06
20990381	2011	6	3	860	139.58	22.95
20990381	2011	6	3	870	141.89	22.81
20990381	2011	6	3	880	143.74	22.41
20990381	2011	6	3	890	144.85	22.42
20990381	2011	6	3	900	147.01	22.21
20990381	2011	6	3	910	149.38	22.11
20990381	2011	6	3	920	151.38	22
20990381	2011	6	3	930	153.38	21.83
20990381	2011	6	3	940	153.99	21.76
20990381	2011	6	3	950	154.25	21.51
20990381	2011	6	3	960	154.59	21.31
20990381	2011	6	3	970	155.38	21.02
20990381	2011	6	3	980	156.73	20.93
20990381	2011	6	3	990	158.07	20.86
20990381	2011	6	3	1000	159.62	20.79
20990381	2011	6	3	1010	161.27	20.71
20990381	2011	6	3	1020	162.44	20.64
20990381	2011	6	3	1030	164.15	20.5
20990381	2011	6	3	1040	166.03	20.42
20990381	2011	6	3	1050	166.83	20.28
20990381	2011	6	3	1060	168.35	20.13
20990381	2011	6	3	1070	170.37	20.01
20990381	2011	6	3	1080	172.47	19.83
20990381	2011	6	3	1090	174.01	19.7
20990381	2011	6	3	1100	174.86	19.67
20990381	2011	6	3	1110	175.1	19.64
20990381	2011	6	3	1120	175.3	19.57
20990381	2011	6	3	1130	176.23	19.4
20990381	2011	6	3	1140	177.31	19.17
20990381	2011	6	3	1150	179.2	19.1
20990381	2011	6	3	1160	180.97	19.07
20990381	2011	6	3	1170	181.81	19.02

20990381	2011	6	3	1180	183.11	18.78
20990381	2011	6	3	1190	184.47	18.66
20990381	2011	6	3	1200	186.37	18.62
20990381	2011	6	3	1210	187.65	18.53
20990381	2011	6	3	1220	187.59	18.52
20990381	2011	6	3	1230	187.94	18.48
20990381	2011	6	3	1240	189.39	18.39
20990381	2011	6	3	1250	190.01	18.33
20990381	2011	6	3	1260	190.68	18.27
20990381	2011	6	3	1270	191.24	18.21
20990381	2011	6	3	1280	192.2	18.14
20990381	2011	6	3	1290	192.95	18.1
20990381	2011	6	3	1300	194.62	18.05
20990381	2011	6	3	1310	195.13	18
20990381	2011	6	3	1320	195.27	17.93
20990381	2011	6	3	1330	195.79	17.88
20990381	2011	6	3	1340	196.1	17.83
20990381	2011	6	3	1350	196.24	17.79
20990381	2011	6	3	1360	196.17	17.74
20990381	2011	6	3	1370	196.06	17.69
20990381	2011	6	3	1380	195.96	17.66
20990381	2011	6	3	1390	195.89	17.62
20990381	2011	6	3	1400	196.09	17.58
20990381	2011	6	3	1410	196.13	17.55
20990381	2011	6	3	1420	196.13	17.51
20990381	2011	6	3	1430	196.12	17.49
20990381	2011	6	3	1440	195.92	17.46
20990381	2011	6	3	1450	195.58	17.42
20990381	2011	6	3	1460	195.97	17.39
20990381	2011	6	3	1470	195.87	17.35
20990381	2011	6	3	1480	195.58	17.32
20990381	2011	6	3	1490	195.4	17.3
20990381	2011	6	3	1500	195.52	17.28
20990381	2011	6	3	1510	195.53	17.25
20990381	2011	6	3	1520	195.59	17.24
20990381	2011	6	3	1530	195.65	17.22
20990381	2011	6	3	1540	195.5	17.21
20990381	2011	6	3	1550	195.76	17.17
20990381	2011	6	3	1560	195.87	17.16
20990381	2011	6	3	1570	195.86	17.12
20990381	2011	6	3	1580	195.78	17.11
20990381	2011	6	3	1590	195.81	17.1
20990381	2011	6	3	1600	195.86	17.08
20990381	2011	6	3	1610	195.9	17.06
20990381	2011	6	3	1620	195.83	17.06
20990381	2011	6	3	1630	195.7	17.03
20990381	2011	6	3	1640	196.33	17.01

20990381	2011	6	3	1650	196.6	16.99
20990381	2011	6	3	1660	197.02	16.97
20990381	2011	6	3	1670	196.94	16.92
20990381	2011	6	3	1680	196.9	16.92
20990381	2011	6	3	1690	196.67	16.92
20990381	2011	6	3	1700	196.74	16.92
20990381	2011	6	3	1710	196.78	16.9
20990381	2011	6	3	1720	197.06	16.89
20990381	2011	6	3	1730	197.36	16.88
20990381	2011	6	3	1740	197.21	16.87
20990381	2011	6	3	1750	197.01	16.86
20990381	2011	6	3	1760	197.01	16.85
20990381	2011	6	3	1770	197.01	16.84
20990381	2011	6	3	1780	196.83	16.84
20990381	2011	6	3	1790	196.65	16.83
20990381	2011	6	3	1800	196.65	16.82
20990381	2011	6	3	1810	196.84	16.82
20990381	2011	6	3	1820	196.87	16.82
20990381	2011	6	3	1830	196.76	16.81
20990381	2011	6	3	1840	196.45	16.81
20990381	2011	6	3	1850	196.3	16.81
20990381	2011	6	3	1860	196.61	16.8
20990381	2011	6	3	1870	196.81	16.8
20990381	2011	6	3	1880	197.09	16.8
20990381	2011	6	3	1890	197.03	16.8
20990381	2011	6	3	1900	196.96	16.78
20990381	2011	6	3	1910	197.55	16.78
20990381	2011	6	3	1920	197.88	16.78
20990381	2011	6	3	1930	197.91	16.77
20990381	2011	6	3	1940	197.84	16.77
20990381	2011	6	3	1950	197.85	16.77
20990381	2011	6	3	1960	197.93	16.77
20990381	2011	6	3	1970	197.83	16.76
20990381	2011	6	3	1980	197.58	16.76
20990381	2011	6	3	1990	197.71	16.76
20990381	2011	6	3	2000	197.58	16.76
20990381	2011	6	3	2010	197.41	16.75
20990381	2011	6	3	2020	197.96	16.75
20990381	2011	6	3	2030	198.31	16.75
20990381	2011	6	3	2040	198.28	16.75
20990381	2011	6	3	2050	198.01	16.75
20990381	2011	6	3	2060	198.64	16.75
20990381	2011	6	3	2070	199.18	16.75
20990381	2011	6	3	2080	199.34	16.75
20990381	2011	6	3	2090	200.54	16.75
20990381	2011	6	3	2100	202.02	16.75
20990381	2011	6	3	2110	203.23	16.74

20990381	2011	6	3	2120	204.74	16.71
20990381	2011	6	3	2130	206.39	16.71
20990381	2011	6	3	2140	207.58	16.67
20990381	2011	6	3	2150	208.98	16.66
20990381	2011	6	3	2160	210.43	16.61
20990381	2011	6	3	2170	212.2	16.58
20990381	2011	6	3	2180	212.03	16.58
20990381	2011	6	3	2190	212.14	16.57
20990381	2011	6	3	2200	212.2	16.55
20990381	2011	6	3	2210	211.97	16.53
20990381	2011	6	3	2220	211.61	16.51
20990381	2011	6	3	2230	211.29	16.5
20990381	2011	6	3	2240	211.58	16.48
20990381	2011	6	3	2250	211.8	16.46
20990381	2011	6	3	2260	211.61	16.44
20990381	2011	6	3	2270	211.72	16.43
20990381	2011	6	3	2280	211.57	16.42
20990381	2011	6	3	2290	211.47	16.4
20990381	2011	6	3	2300	211.67	16.4
20990381	2011	6	3	2310	211.74	16.39
20990381	2011	6	3	2320	211.5	16.38
20990381	2011	6	3	2330	211.47	16.37
20990381	2011	6	3	2340	212.27	16.36
20990381	2011	6	3	2350	212.32	16.35
20990381	2011	6	3	2360	212.17	16.35
20990381	2011	6	3	2370	211.87	16.34
20990381	2011	6	3	2380	211.53	16.34
20990381	2011	6	3	2390	211.54	16.33
20990381	2011	6	3	2400	210.88	16.32
20990381	2011	6	3	2410	210.9	16.32
20990381	2011	6	3	2420	211.24	16.32
20990381	2011	6	3	2430	211.57	16.31
20990381	2011	6	3	2440	211.79	16.3
20990381	2011	6	3	2450	212.47	16.29
20990381	2011	6	3	2460	212.54	16.29
20990381	2011	6	3	2470	212.34	16.29
20990381	2011	6	3	2480	212.21	16.28
20990381	2011	6	3	2490	212.44	16.27
20990381	2011	6	3	2500	212.33	16.27
20990381	2011	6	3	2510	212.28	16.27
20990381	2011	6	3	2520	212.31	16.26
20990381	2011	6	3	2530	212.33	16.25
20990381	2011	6	3	2540	212.1	16.25
20990381	2011	6	3	2550	212.05	16.24
20990381	2011	6	3	2560	212.08	16.24
20990381	2011	6	3	2570	212.1	16.23
20990381	2011	6	3	2580	212.4	16.23

20990381	2011	6	3	2590	212.39	16.24
20990381	2011	6	3	2600	212.51	16.24
20990381	2011	6	3	2610	212.44	16.23
20990381	2011	6	3	2620	212.19	16.23
20990381	2011	6	3	2630	211.75	16.23
20990381	2011	6	3	2640	211.43	16.23
20990381	2011	6	3	2650	211.28	16.22
20990381	2011	6	3	2660	210.69	16.22
20990381	2011	6	3	2670	209.98	16.22
20990381	2011	6	3	2680	209.81	16.22
20990381	2011	6	3	2690	210.27	16.22
20990381	2011	6	3	2700	210.2	16.22
20990381	2011	6	3	2710	209.84	16.21
20990381	2011	6	3	2720	209.45	16.21
20990381	2011	6	3	2730	208.85	16.22
20990381	2011	6	3	2740	208.97	16.22
20990381	2011	6	3	2750	209.54	16.23
20990381	2011	6	3	2760	209.9	16.23
20990381	2011	6	3	2770	210.1	16.23
20990381	2011	6	3	2780	209.38	16.23
20990381	2011	6	3	2790	207.43	16.22
20990381	2011	6	3	2800	205.37	16.23
20990381	2011	6	3	2810	203.68	16.24
20990381	2011	6	3	2820	201.18	16.25
20990381	2011	6	3	2830	199.4	16.25
20990381	2011	6	3	2840	197.26	16.28
20990381	2011	6	3	2850	195.51	16.3
20990381	2011	6	3	2860	193.61	16.32
20990381	2011	6	3	2870	192.16	16.35
20990381	2011	6	3	2880	191.37	16.39
20990381	2011	6	3	2890	190.99	16.46
20990381	2011	6	3	2900	191.19	16.47
20990381	2011	6	3	2910	191.64	16.5
20990381	2011	6	3	2920	191.84	16.53
20990381	2011	6	3	2930	191.73	16.55
20990381	2011	6	3	2940	191.64	16.57
20990381	2011	6	3	2950	191.61	16.6
20990381	2011	6	3	2960	191.79	16.62
20990381	2011	6	3	2970	191.56	16.64
20990381	2011	6	3	2980	191.35	16.66
20990381	2011	6	3	2990	191.38	16.67
20990381	2011	6	3	3000	191.35	16.69
20990381	2011	6	3	3010	190.91	16.7
20990381	2011	6	3	3020	189.2	16.72
20990381	2011	6	3	3030	187.96	16.75
20990381	2011	6	3	3040	186.26	16.75
20990381	2011	6	3	3050	184.42	16.78

20990381	2011	6	3	3060	182.89	16.79
20990381	2011	6	3	3070	181.87	16.81
20990381	2011	6	3	3080	180.97	16.83
20990381	2011	6	3	3090	180.2	16.85
20990381	2011	6	3	3100	179.12	16.87
20990381	2011	6	3	3110	177.71	16.89
20990381	2011	6	3	3120	176.95	16.9
20990381	2011	6	3	3130	176.81	16.92
20990381	2011	6	3	3140	176.39	16.96
20990381	2011	6	3	3150	176.28	16.99
20990381	2011	6	3	3160	175.89	17
20990381	2011	6	3	3170	175.41	17.02
20990381	2011	6	3	3180	175.03	17.03
20990381	2011	6	3	3190	175.02	17.04
20990381	2011	6	3	3200	174.99	17.05
20990381	2011	6	3	3210	174.83	17.07
20990381	2011	6	3	3220	174.74	17.08
20990381	2011	6	3	3230	174.68	17.1
20990381	2011	6	3	3240	174.69	17.1
20990381	2011	6	3	3250	175.03	17.11
20990381	2011	6	3	3260	175.01	17.13
20990381	2011	6	3	3270	174.87	17.14
20990381	2011	6	3	3280	174.51	17.14
20990381	2011	6	3	3290	174.74	17.15
20990381	2011	6	3	3300	174.9	17.17
20990381	2011	6	3	3310	174.65	17.17
20990381	2011	6	3	3320	174.32	17.17
20990381	2011	6	3	3330	173.81	17.18
20990381	2011	6	3	3340	173.51	17.19
20990381	2011	6	3	3350	173.3	17.19
20990381	2011	6	3	3360	172.75	17.2
20990381	2011	6	3	3370	171.95	17.21
20990381	2011	6	3	3380	172.13	17.21
20990381	2011	6	3	3390	172.12	17.21
20990381	2011	6	3	3400	172.14	17.22
20990381	2011	6	3	3410	172.04	17.22
20990381	2011	6	3	3420	171.89	17.24
20990381	2011	6	3	3430	171.35	17.24
20990381	2011	6	3	3440	170.8	17.24
20990381	2011	6	3	3450	170.42	17.25
20990381	2011	6	3	3460	170.02	17.25
20990381	2011	6	3	3470	170.08	17.26
20990381	2011	6	3	3480	169.99	17.26
20990381	2011	6	3	3490	169.63	17.26
20990381	2011	6	3	3500	169.3	17.27
20990381	2011	6	3	3510	169.52	17.28
20990381	2011	6	3	3520	168.47	17.28

20990381	2011	6	3	3530	167.84	17.28
20990381	2011	6	3	3540	167.42	17.29
20990381	2011	6	3	3550	167.97	17.31
20990381	2011	6	3	3560	168.07	17.32
20990381	2011	6	3	3570	168.05	17.33
20990381	2011	6	3	3580	167.81	17.35
20990381	2011	6	3	3590	167.61	17.36
20990381	2011	6	3	3600	167.75	17.38
20990381	2011	6	3	3610	167.81	17.39
20990381	2011	6	3	3620	167.87	17.41
20990381	2011	6	3	3630	166.41	17.42
20990381	2011	6	3	3640	164.99	17.45
20990381	2011	6	3	3650	164.59	17.48
20990381	2011	6	3	3660	164.75	17.5
20990381	2011	6	3	3670	164.75	17.54
20990381	2011	6	3	3680	164.2	17.57
20990381	2011	6	3	3690	162.6	17.61
20990381	2011	6	3	3700	162.13	17.67
20990381	2011	6	3	3710	162.18	17.73
20990381	2011	6	3	3720	162.06	17.75
20990381	2011	6	3	3730	161.64	17.8
20990381	2011	6	3	3740	160.51	17.82
20990381	2011	6	3	3750	159	17.87
20990381	2011	6	3	3760	157.89	17.9
20990381	2011	6	3	3770	157.38	17.93
20990381	2011	6	3	3780	157.27	18
20990381	2011	6	3	3790	157.18	18.03
20990381	2011	6	3	3800	157.02	18.06
20990381	2011	6	3	3810	157.21	18.08
20990381	2011	6	3	3820	157.03	18.11
20990381	2011	6	3	3830	157.03	18.15
20990381	2011	6	3	3840	156.86	18.17
20990381	2011	6	3	3850	156.98	18.21
20990381	2011	6	3	3860	157.04	18.24
20990381	2011	6	3	3870	156.76	18.26
20990381	2011	6	3	3880	156.3	18.28
20990381	2011	6	3	3890	154.37	18.32
20990381	2011	6	3	3900	152.43	18.35
20990381	2011	6	3	3910	151.77	18.39
20990381	2011	6	3	3920	151.89	18.42
20990381	2011	6	3	3930	151.97	18.43
20990381	2011	6	3	3940	152.06	18.46
20990381	2011	6	3	3950	152.03	18.49
20990381	2011	6	3	3960	151.83	18.51
20990381	2011	6	3	3970	151.5	18.53
20990381	2011	6	3	3980	151.47	18.55
20990381	2011	6	3	3990	151.58	18.57

20990381	2011	6	3	4000	151.33	18.6
20990381	2011	6	3	4010	151.52	18.62
20990381	2011	6	3	4020	151.63	18.64
20990381	2011	6	3	4030	151.38	18.67
20990381	2011	6	3	4040	151.1	18.68
20990381	2011	6	3	4050	150.91	18.7
20990381	2011	6	3	4060	150.57	18.71
20990381	2011	6	3	4070	150.21	18.73
20990381	2011	6	3	4080	150.11	18.75
20990381	2011	6	3	4090	149.13	18.77
20990381	2011	6	3	4100	148.41	18.79
20990381	2011	6	3	4110	147.33	18.82
20990381	2011	6	3	4120	146.69	18.83
20990381	2011	6	3	4130	146.34	18.85
20990381	2011	6	3	4140	145.9	18.89
20990381	2011	6	3	4150	145.29	18.9
20990381	2011	6	3	4160	145.2	18.92
20990381	2011	6	3	4170	145.35	18.94
20990381	2011	6	3	4180	145.05	18.96
20990381	2011	6	3	4190	145.04	19
20990381	2011	6	3	4200	144.86	19.02
20990381	2011	6	3	4210	144.52	19.04
20990381	2011	6	3	4220	144.01	19.07
20990381	2011	6	3	4230	143.48	19.08
20990381	2011	6	3	4240	141.79	19.11
20990381	2011	6	3	4250	140.12	19.14
20990381	2011	6	3	4260	139.6	19.17
20990381	2011	6	3	4270	139.53	19.23
20990381	2011	6	3	4280	139.04	19.28
20990381	2011	6	3	4290	138.43	19.31
20990381	2011	6	3	4300	137.94	19.34
20990381	2011	6	3	4310	137.07	19.39
20990381	2011	6	3	4320	136.16	19.42
20990381	2011	6	3	4330	135.51	19.49
20990381	2011	6	3	4340	135.99	19.56
20990381	2011	6	3	4350	135.85	19.58
20990381	2011	6	3	4360	135.24	19.61
20990381	2011	6	3	4370	134.77	19.64
20990381	2011	6	3	4380	133.14	19.7
20990381	2011	6	3	4390	132.03	19.72
20990381	2011	6	3	4400	131.62	19.75
20990381	2011	6	3	4410	131.27	19.78
20990381	2011	6	3	4420	130.88	19.8
20990381	2011	6	3	4430	130.35	19.83
20990381	2011	6	3	4440	129.75	19.86
20990381	2011	6	3	4450	129.3	19.89
20990381	2011	6	3	4460	128.99	19.91

20990381	2011	6	3	4470	128.34	19.94
20990381	2011	6	3	4480	128.32	19.96
20990381	2011	6	3	4490	128.44	19.99
20990381	2011	6	3	4500	128.45	20
20990381	2011	6	3	4510	128.34	20.03
20990381	2011	6	3	4520	128.09	20.04
20990381	2011	6	3	4530	128	20.07
20990381	2011	6	3	4540	128.21	20.09
20990381	2011	6	3	4550	128.31	20.1
20990381	2011	6	3	4560	128.66	20.13
20990381	2011	6	3	4570	128.68	20.14
20990381	2011	6	3	4580	128.5	20.16
20990381	2011	6	3	4590	128.28	20.17
20990381	2011	6	3	4600	128.29	20.18
20990381	2011	6	3	4610	128.12	20.2
20990381	2011	6	3	4620	128.14	20.21
20990381	2011	6	3	4630	128.39	20.22
20990381	2011	6	3	4640	128.45	20.23
20990381	2011	6	3	4650	128.28	20.25
20990381	2011	6	3	4660	127.91	20.26
20990381	2011	6	3	4670	127.72	20.27
20990381	2011	6	3	4680	127.66	20.28
20990381	2011	6	3	4690	127.85	20.3
20990381	2011	6	3	4700	128.44	20.32
20990381	2011	6	3	4710	128.6	20.32
20990381	2011	6	3	4720	128.64	20.32
20990381	2011	6	3	4730	127.82	20.32
20990381	2011	6	3	4740	127.22	20.34
20990381	2011	6	3	4750	127.13	20.34
20990381	2011	6	3	4760	127.43	20.35
20990381	2011	6	3	4770	127.43	20.35
20990381	2011	6	3	4780	126.85	20.35
20990381	2011	6	3	4790	126.27	20.37
20990381	2011	6	3	4800	126.02	20.37
20990381	2011	6	3	4810	125.86	20.38
20990381	2011	6	3	4820	125.96	20.39
20990381	2011	6	3	4830	126.18	20.39
20990381	2011	6	3	4840	126.1	20.4
20990381	2011	6	3	4850	125.98	20.4
20990381	2011	6	3	4860	125.89	20.41
20990381	2011	6	3	4870	125.82	20.41
20990381	2011	6	3	4880	125.8	20.42
20990381	2011	6	3	4890	125.58	20.43
20990381	2011	6	3	4900	125.38	20.43
20990381	2011	6	3	4910	124.62	20.44
20990381	2011	6	3	4920	123.9	20.46
20990381	2011	6	3	4930	123.01	20.48

20990381	2011	6	3	4940	121.7	20.5
20990381	2011	6	3	4950	120.76	20.54
20990381	2011	6	3	4960	119.99	20.57
20990381	2011	6	3	4970	119.6	20.6
20990381	2011	6	3	4980	118.07	20.63
20990381	2011	6	3	4990	116.87	20.66
20990381	2011	6	3	5000	116.24	20.68
20990381	2011	6	3	5010	114.66	20.73
20990381	2011	6	3	5020	113.97	20.77
20990381	2011	6	3	5030	114.41	20.84
20990381	2011	6	3	5040	114.25	20.89
20990381	2011	6	3	5050	114.29	20.92
20990381	2011	6	3	5060	113.53	20.96
20990381	2011	6	3	5070	112.81	20.99
20990381	2011	6	3	5080	111.61	21.03
20990381	2011	6	3	5090	110.6	21.07
20990381	2011	6	3	5100	109.42	21.11
20990381	2011	6	3	5110	108.92	21.15
20990381	2011	6	3	5120	108.75	21.2
20990381	2011	6	3	5130	108.86	21.25
20990381	2011	6	3	5140	108.89	21.28
20990381	2011	6	3	5150	108.83	21.32
20990381	2011	6	3	5160	108.92	21.35
20990381	2011	6	3	5170	108.89	21.38
20990381	2011	6	3	5180	108.79	21.42
20990381	2011	6	3	5190	108.66	21.46
20990381	2011	6	3	5200	106.97	21.53
20990381	2011	6	3	5210	104.46	21.57
20990381	2011	6	3	5220	102.04	21.64
20990381	2011	6	3	5230	99.6	21.67
20990381	2011	6	3	5240	97.89	21.72
20990381	2011	6	3	5250	96.54	21.77
20990381	2011	6	3	5260	94.88	21.82
20990381	2011	6	3	5270	93.58	21.88
20990381	2011	6	3	5280	92.98	21.95
20990381	2011	6	3	5290	92.07	22.02
20990381	2011	6	3	5300	91.27	22.1
20990381	2011	6	3	5310	90.65	22.21
20990381	2011	6	3	5320	89.26	22.3
20990381	2011	6	3	5330	88.61	22.37
20990381	2011	6	3	5340	88.31	22.42
20990381	2011	6	3	5350	88.07	22.49
20990381	2011	6	3	5360	87.37	22.57
20990381	2011	6	3	5370	85.64	22.67
20990381	2011	6	3	5380	84.56	22.75
20990381	2011	6	3	5390	83.57	22.83
20990381	2011	6	3	5400	82.43	22.9

20990381	2011	6	3	5410	81.58	22.97
20990381	2011	6	3	5420	79.89	23.03
20990381	2011	6	3	5430	78.01	23.17
20990381	2011	6	3	5440	75.91	23.23
20990381	2011	6	3	5450	73.84	23.32
20990381	2011	6	3	5460	71.93	23.38
20990381	2011	6	3	5470	71.37	23.5
20990381	2011	6	3	5480	70.95	23.55
20990381	2011	6	3	5490	70.77	23.57
20990381	2011	6	3	5500	70.65	23.6
20990381	2011	6	3	5510	69.46	23.64
20990381	2011	6	3	5520	67.32	23.71
20990381	2011	6	3	5530	64.68	23.8
20990381	2011	6	3	5540	62.38	23.86
20990381	2011	6	3	5550	59.73	23.92
20990381	2011	6	3	5560	57.02	23.98
20990381	2011	6	3	5570	54.58	24.03
20990381	2011	6	3	5580	52.5	24.1
20990381	2011	6	3	5590	50.71	24.16
20990381	2011	6	3	5600	49.65	24.25
20990381	2011	6	3	5610	49.19	24.31
20990381	2011	6	3	5620	48.96	24.38
20990381	2011	6	3	5630	48.32	24.5
20990381	2011	6	3	5640	47.1	24.58
20990381	2011	6	3	5650	46.53	24.63
20990381	2011	6	3	5660	46.37	24.67
20990381	2011	6	3	5670	46.09	24.74
20990381	2011	6	3	5680	46.95	24.81
20990381	2011	6	3	5690	47.62	24.86
20990381	2011	6	3	5700	48.05	24.9
20990381	2011	6	3	5710	48.26	24.93
20990381	2011	6	3	5720	48.55	24.97
20990381	2011	6	3	5730	48.57	25.01
20990381	2011	6	3	5740	48.15	25.07
20990381	2011	6	3	5750	47.53	25.1
20990381	2011	6	3	5760	47.07	25.14
20990381	2011	6	3	5770	46.29	25.18
20990381	2011	6	3	5780	45.77	25.21
20990381	2011	6	3	5790	45.96	25.25
20990381	2011	6	3	5800	45.91	25.27
20990381	2011	6	3	5810	45.53	25.31
20990381	2011	6	3	5820	45.98	25.33
20990381	2011	6	3	5830	45.32	25.36
20990381	2011	6	3	5840	44.97	25.39
20990381	2011	6	3	5850	44.89	25.42
20990381	2011	6	3	5860	44.95	25.44
20990381	2011	6	3	5870	45.01	25.46

20990381	2011	6	3	5880	45.05	25.47
20990381	2011	6	3	5890	45.12	25.48
20990381	2011	6	3	5900	45.54	25.51
20990381	2011	6	3	5910	45.92	25.53
20990381	2011	6	3	5920	46.21	25.55
20990381	2011	6	3	5930	46.82	25.56
20990381	2011	6	3	5940	47.33	25.57
20990381	2011	6	3	5950	48	25.58
20990381	2011	6	3	5960	48.37	25.58
20990381	2011	6	3	5970	48.28	25.6
20990381	2011	6	3	5980	47.98	25.61
20990381	2011	6	3	5990	47.83	25.63
20990381	2011	6	3	6000	47.51	25.64
20990381	2011	6	3	6010	47.59	25.65
20990381	2011	6	3	6020	47.3	25.66
20990381	2011	6	3	6030	47.27	25.67
20990381	2011	6	3	6040	47.09	25.67
20990381	2011	6	3	6050	46.99	25.69
20990381	2011	6	3	6060	46.89	25.7
20990381	2011	6	3	6070	46.76	25.71
20990381	2011	6	3	6080	46.45	25.71
20990381	2011	6	3	6090	46.63	25.71
20990381	2011	6	3	6100	46.42	25.72
20990381	2011	6	3	6110	46.67	25.73
20990381	2011	6	3	6120	47.08	25.73
20990381	2011	6	3	6130	46.99	25.74
20990381	2011	6	3	6140	47	25.75
20990381	2011	6	3	6150	47.29	25.75
20990381	2011	6	3	6160	47.76	25.75
20990381	2011	6	3	6170	48.3	25.75
20990381	2011	6	3	6180	48.85	25.75
20990381	2011	6	3	6190	49.42	25.74
20990381	2011	6	3	6200	50.07	25.74
20990381	2011	6	3	6210	50.61	25.74
20990381	2011	6	3	6220	50.84	25.74
20990381	2011	6	3	6230	50.86	25.74
20990381	2011	6	3	6240	50.99	25.74
20990381	2011	6	3	6250	51.37	25.74
20990381	2011	6	3	6260	51.48	25.73
20990381	2011	6	3	6270	51.06	25.73
20990381	2011	6	3	6280	50.73	25.74
20990381	2011	6	3	6290	50.97	25.74
20990381	2011	6	3	6300	51.26	25.74
20990381	2011	6	3	6310	51.5	25.73
20990381	2011	6	3	6320	51.61	25.74
20990381	2011	6	3	6330	51.42	25.73
20990381	2011	6	3	6340	51.56	25.73

20990381	2011	6	3	6350	52.1	25.73
20990381	2011	6	3	6360	52.2	25.73
20990381	2011	6	3	6370	51.78	25.73
20990381	2011	6	3	6380	51.3	25.73
20990381	2011	6	3	6390	51.22	25.73
20990381	2011	6	3	6400	51.55	25.74
20990381	2011	6	3	6410	51.91	25.74
20990381	2011	6	3	6420	51.87	25.73
20990381	2011	6	3	6430	51.93	25.73
20990381	2011	6	3	6440	51.4	25.73
20990381	2011	6	3	6450	49.75	25.75
20990381	2011	6	3	6460	49.23	25.75
20990381	2011	6	3	6470	49.19	25.75
20990381	2011	6	3	6480	49.37	25.75
20990381	2011	6	3	6490	49.18	25.76
20990381	2011	6	3	6500	49.14	25.76
20990381	2011	6	3	6510	49.21	25.76
20990381	2011	6	3	6520	49.47	25.77
20990381	2011	6	3	6530	49.41	25.77
20990381	2011	6	3	6540	49.56	25.78
20990381	2011	6	3	6550	49.83	25.78
20990381	2011	6	3	6560	50.21	25.78
20990381	2011	6	3	6570	50.55	25.78
20990381	2011	6	3	6580	50.82	25.78
20990381	2011	6	3	6590	50.77	25.78
20990381	2011	6	3	6600	50.66	25.78
20990381	2011	6	3	6610	50.21	25.78
20990381	2011	6	3	6620	49.86	25.78
20990381	2011	6	3	6630	49.77	25.79
20990381	2011	6	3	6640	49.7	25.79
20990381	2011	6	3	6650	48.51	25.8
20990381	2011	6	3	6660	47.28	25.8
20990381	2011	6	3	6670	46.32	25.82
20990381	2011	6	3	6680	45.52	25.82
20990381	2011	6	3	6690	45.18	25.83
20990381	2011	6	3	6700	44.8	25.84
20990381	2011	6	3	6710	43.77	25.85
20990381	2011	6	3	6720	43.52	25.86
20990381	2011	6	3	6730	42.77	25.88
20990381	2011	6	3	6740	41.38	25.89
20990381	2011	6	3	6750	40.88	25.9
20990381	2011	6	3	6760	40.78	25.92
20990381	2011	6	3	6770	39.67	25.93
20990381	2011	6	3	6780	39.24	25.96
20990381	2011	6	3	6790	38.2	25.97
20990381	2011	6	3	6800	38.08	26
20990381	2011	6	3	6810	37.91	26.04

20990381	2011	6	3	6820	37.68	26.07
20990381	2011	6	3	6830	36.42	26.13
20990381	2011	6	3	6840	35.92	26.17
20990381	2011	6	3	6850	35.55	26.33
20990381	2011	6	3	6860	34.94	26.46
20990381	2011	6	3	6870	35.15	26.57
20990381	2011	6	3	6880	34.75	26.68
20990381	2011	6	3	6890	34.22	26.78
20990381	2011	6	3	6900	34.62	26.88
20990381	2011	6	3	6910	34.97	26.96
20990381	2011	6	3	6920	34.67	27.06
20990381	2011	6	3	6930	35.17	27.11
20990381	2011	6	3	6940	35.11	27.17
20990381	2011	6	3	6950	35.1	27.24
20990381	2011	6	3	6960	35.16	27.3
20990381	2011	6	3	6970	35.15	27.38
20990381	2011	6	3	6980	34.67	27.42
20990381	2011	6	3	6990	34.87	27.51
20990381	2011	6	3	7000	35.03	27.55
20990381	2011	6	3	7010	34.82	27.6
20990381	2011	6	3	7020	33.91	27.64
20990381	2011	6	3	7030	33.88	27.67
20990381	2011	6	3	7040	34.25	27.7
20990381	2011	6	3	7050	34.42	27.78
20990381	2011	6	3	7060	33.56	27.8
20990381	2011	6	3	7070	32.28	27.82
20990381	2011	6	3	7080	32.02	27.85
20990381	2011	6	3	7090	30.73	27.89
20990381	2011	6	3	7100	28.9	27.92
20990381	2011	6	3	7110	27.23	27.97
20990381	2011	6	3	7120	25.62	28
20990381	2011	6	3	7130	23.77	28.05
20990381	2011	6	3	7140	21.98	28.08
20990381	2011	6	3	7150	19.87	28.11
20990381	2011	6	3	7160	18.01	28.15
20990381	2011	6	3	7170	15.89	28.18
20990381	2011	6	3	7180	14.5	28.21
20990381	2011	6	3	7190	13.83	28.24
20990381	2011	6	3	7200	12.52	28.27
20990381	2011	6	3	7210	10.57	28.3
20990381	2011	6	3	7220	8.78	28.34
20990381	2011	6	3	7230	6.56	28.38
20990381	2011	6	3	7240	4.28	28.41
20990381	2011	6	3	7250	3.02	28.43
20990381	2011	6	3	7260	3	28.45
20990381	2011	6	3	7270	2.68	28.48
20990381	2011	6	3	7280	2.36	28.5

20990381	2011	6	3	7290	2.5	28.51
20990381	2011	6	3	7300	2.86	28.53
20990381	2011	6	3	7310	0.69	28.56
20990381	2011	6	3	7320	0.58	28.63
20990381	2011	6	3	7330	0.61	28.64
20990381	2011	6	3	7340	0.58	28.66
20990381	2011	6	3	7350	0.6	28.7
20990381	2011	6	3	7360	0.58	28.73
20990381	2011	6	3	7370	0.56	28.75
20990381	2011	6	3	7380	0.61	28.78
20990381	2011	6	3	7390	0.58	28.82
20990381	2011	6	3	7400	0.43	28.85
20990381	2011	6	3	7410	0.4	28.89
20990381	2011	6	3	7420	0.43	28.92
20990381	2011	6	3	7430	0.43	28.95
20990381	2011	6	3	7440	0.41	28.98
20990381	2011	6	3	7450	0.44	29
21067271	2011	6	4	0	1	28.92
21067271	2011	6	4	10	1.86	28.92
21067271	2011	6	4	20	2.26	28.92
21067271	2011	6	4	30	2.48	28.92
21067271	2011	6	4	40	2.61	28.92
21067271	2011	6	4	50	2.53	28.92
21067271	2011	6	4	60	2.37	28.92
21067271	2011	6	4	70	2.56	28.92
21067271	2011	6	4	80	2.76	28.92
21067271	2011	6	4	90	2.48	28.92
21067271	2011	6	4	100	2.73	28.92
21067271	2011	6	4	110	3.14	28.92
21067271	2011	6	4	120	3.93	28.92
21067271	2011	6	4	130	5.49	28.89
21067271	2011	6	4	140	7.78	28.87
21067271	2011	6	4	150	9.18	28.85
21067271	2011	6	4	160	9.61	28.84
21067271	2011	6	4	170	9.8	28.84
21067271	2011	6	4	180	9.93	28.83
21067271	2011	6	4	190	10.02	28.82
21067271	2011	6	4	200	10.06	28.82
21067271	2011	6	4	210	10.14	28.82
21067271	2011	6	4	220	10.3	28.81
21067271	2011	6	4	230	10.53	28.82
21067271	2011	6	4	240	10.78	28.8
21067271	2011	6	4	250	11.03	28.79
21067271	2011	6	4	260	11.29	28.78
21067271	2011	6	4	270	11.53	28.78
21067271	2011	6	4	280	11.77	28.78
21067271	2011	6	4	290	12.01	28.78

21067271	2011	6	4	300	11.71	28.77
21067271	2011	6	4	310	11.54	28.76
21067271	2011	6	4	320	11.53	28.76
21067271	2011	6	4	330	11.54	28.77
21067271	2011	6	4	340	11.65	28.76
21067271	2011	6	4	350	11.62	28.75
21067271	2011	6	4	360	11.66	28.75
21067271	2011	6	4	370	12.55	28.74
21067271	2011	6	4	380	13.19	28.74
21067271	2011	6	4	390	14.2	28.71
21067271	2011	6	4	400	16.05	28.7
21067271	2011	6	4	410	17.78	28.68
21067271	2011	6	4	420	19.81	28.66
21067271	2011	6	4	430	21.28	28.64
21067271	2011	6	4	440	22.9	28.63
21067271	2011	6	4	450	24.81	28.6
21067271	2011	6	4	460	26.57	28.6
21067271	2011	6	4	470	28.45	28.58
21067271	2011	6	4	480	30.75	28.56
21067271	2011	6	4	490	32.85	28.54
21067271	2011	6	4	500	34.94	28.53
21067271	2011	6	4	510	37.31	28.5
21067271	2011	6	4	520	40.14	28.48
21067271	2011	6	4	530	42.94	28.46
21067271	2011	6	4	540	45.6	28.44
21067271	2011	6	4	550	47.91	28.4
21067271	2011	6	4	560	49.14	28.34
21067271	2011	6	4	570	50.52	28.23
21067271	2011	6	4	580	52.99	28.03
21067271	2011	6	4	590	54.16	27.91
21067271	2011	6	4	600	54.55	27.87
21067271	2011	6	4	610	54.74	27.85
21067271	2011	6	4	620	55.03	27.8
21067271	2011	6	4	630	54.84	27.71
21067271	2011	6	4	640	54.99	27.65
21067271	2011	6	4	650	55.61	27.48
21067271	2011	6	4	660	57.35	27.26
21067271	2011	6	4	670	58.07	27.22
21067271	2011	6	4	680	57.46	27.16
21067271	2011	6	4	690	56.98	27.14
21067271	2011	6	4	700	55.36	27.1
21067271	2011	6	4	710	54	27.05
21067271	2011	6	4	720	54.34	26.95
21067271	2011	6	4	730	54.75	26.92
21067271	2011	6	4	740	54.4	26.86
21067271	2011	6	4	750	54.23	26.82
21067271	2011	6	4	760	52.82	26.78

21067271	2011	6	4	770	51.45	26.76
21067271	2011	6	4	780	51.31	26.69
21067271	2011	6	4	790	51.28	26.66
21067271	2011	6	4	800	50.69	26.64
21067271	2011	6	4	810	50.23	26.6
21067271	2011	6	4	820	50.13	26.58
21067271	2011	6	4	830	50.36	26.56
21067271	2011	6	4	840	50.66	26.52
21067271	2011	6	4	850	50.95	26.49
21067271	2011	6	4	860	51.14	26.47
21067271	2011	6	4	870	51.29	26.46
21067271	2011	6	4	880	51.58	26.45
21067271	2011	6	4	890	51.88	26.44
21067271	2011	6	4	900	52.24	26.42
21067271	2011	6	4	910	52.03	26.39
21067271	2011	6	4	920	52.23	26.36
21067271	2011	6	4	930	52.73	26.35
21067271	2011	6	4	940	53.15	26.32
21067271	2011	6	4	950	53.47	26.29
21067271	2011	6	4	960	53.39	26.28
21067271	2011	6	4	970	51.75	26.28
21067271	2011	6	4	980	51.37	26.25
21067271	2011	6	4	990	51.79	26.22
21067271	2011	6	4	1000	51.58	26.21
21067271	2011	6	4	1010	51.71	26.21
21067271	2011	6	4	1020	51.57	26.19
21067271	2011	6	4	1030	51.73	26.2
21067271	2011	6	4	1040	51.48	26.17
21067271	2011	6	4	1050	51.67	26.17
21067271	2011	6	4	1060	51.61	26.15
21067271	2011	6	4	1070	51.67	26.15
21067271	2011	6	4	1080	51.59	26.14
21067271	2011	6	4	1090	52.37	26.13
21067271	2011	6	4	1100	53.37	26.1
21067271	2011	6	4	1110	53.73	26.1
21067271	2011	6	4	1120	53.75	26.07
21067271	2011	6	4	1130	53.97	26.07
21067271	2011	6	4	1140	54.05	26.07
21067271	2011	6	4	1150	54.07	26.05
21067271	2011	6	4	1160	54.2	26.04
21067271	2011	6	4	1170	54.07	26.03
21067271	2011	6	4	1180	54.08	26.03
21067271	2011	6	4	1190	54.3	26.03
21067271	2011	6	4	1200	54.54	26.02
21067271	2011	6	4	1210	54.91	26.01
21067271	2011	6	4	1220	54.88	26
21067271	2011	6	4	1230	54.85	26

21067271	2011	6	4	1240	54.76	26
21067271	2011	6	4	1250	54.7	25.98
21067271	2011	6	4	1260	54.39	25.97
21067271	2011	6	4	1270	54.44	25.97
21067271	2011	6	4	1280	54.53	25.96
21067271	2011	6	4	1290	54.5	25.96
21067271	2011	6	4	1300	54.29	25.96
21067271	2011	6	4	1310	54.2	25.95
21067271	2011	6	4	1320	54.4	25.94
21067271	2011	6	4	1330	54.59	25.92
21067271	2011	6	4	1340	54.5	25.92
21067271	2011	6	4	1350	54.42	25.93
21067271	2011	6	4	1360	54.4	25.92
21067271	2011	6	4	1370	54.55	25.92
21067271	2011	6	4	1380	54.83	25.92
21067271	2011	6	4	1390	54.47	25.91
21067271	2011	6	4	1400	54.05	25.91
21067271	2011	6	4	1410	53.7	25.92
21067271	2011	6	4	1420	53.25	25.92
21067271	2011	6	4	1430	52.1	25.92
21067271	2011	6	4	1440	51.22	25.93
21067271	2011	6	4	1450	50.09	25.94
21067271	2011	6	4	1460	48.71	25.96
21067271	2011	6	4	1470	47.49	25.96
21067271	2011	6	4	1480	47.4	25.99
21067271	2011	6	4	1490	46.55	26.03
21067271	2011	6	4	1500	45.72	26.07
21067271	2011	6	4	1510	45.07	26.1
21067271	2011	6	4	1520	44.67	26.13
21067271	2011	6	4	1530	44.54	26.18
21067271	2011	6	4	1540	44.21	26.22
21067271	2011	6	4	1550	44.34	26.25
21067271	2011	6	4	1560	44.23	26.3
21067271	2011	6	4	1570	43.86	26.36
21067271	2011	6	4	1580	43.73	26.4
21067271	2011	6	4	1590	43.88	26.44
21067271	2011	6	4	1600	44.09	26.48
21067271	2011	6	4	1610	44.23	26.52
21067271	2011	6	4	1620	44.36	26.57
21067271	2011	6	4	1630	44.6	26.63
21067271	2011	6	4	1640	44.65	26.69
21067271	2011	6	4	1650	44.63	26.73
21067271	2011	6	4	1660	44.34	26.77
21067271	2011	6	4	1670	44.59	26.82
21067271	2011	6	4	1680	44.93	26.85
21067271	2011	6	4	1690	45.28	26.86
21067271	2011	6	4	1700	45.71	26.86

21067271	2011	6	4	1710	45.94	26.85
21067271	2011	6	4	1720	46.23	26.85
21067271	2011	6	4	1730	46.79	26.84
21067271	2011	6	4	1740	47	26.83
21067271	2011	6	4	1750	47.03	26.83
21067271	2011	6	4	1760	47.14	26.82
21067271	2011	6	4	1770	46.79	26.82
21067271	2011	6	4	1780	46.52	26.82
21067271	2011	6	4	1790	46.63	26.81
21067271	2011	6	4	1800	46.07	26.82
21067271	2011	6	4	1810	45.38	26.81
21067271	2011	6	4	1820	45.34	26.82
21067271	2011	6	4	1830	45.46	26.81
21067271	2011	6	4	1840	45.34	26.82
21067271	2011	6	4	1850	45.12	26.82
21067271	2011	6	4	1860	45.39	26.82
21067271	2011	6	4	1870	45.89	26.82
21067271	2011	6	4	1880	45.98	26.82
21067271	2011	6	4	1890	46.09	26.82
21067271	2011	6	4	1900	46.26	26.82
21067271	2011	6	4	1910	46.2	26.82
21067271	2011	6	4	1920	46.28	26.82
21067271	2011	6	4	1930	46.42	26.82
21067271	2011	6	4	1940	46.28	26.83
21067271	2011	6	4	1950	46.17	26.82
21067271	2011	6	4	1960	46.16	26.83
21067271	2011	6	4	1970	46.58	26.82
21067271	2011	6	4	1980	47.1	26.82
21067271	2011	6	4	1990	47.35	26.81
21067271	2011	6	4	2000	47.51	26.81
21067271	2011	6	4	2010	47.42	26.8
21067271	2011	6	4	2020	47.47	26.8
21067271	2011	6	4	2030	47.5	26.8
21067271	2011	6	4	2040	47.22	26.79
21067271	2011	6	4	2050	47.06	26.8
21067271	2011	6	4	2060	47.17	26.78
21067271	2011	6	4	2070	47.32	26.78
21067271	2011	6	4	2080	47.25	26.78
21067271	2011	6	4	2090	47.35	26.77
21067271	2011	6	4	2100	47.39	26.78
21067271	2011	6	4	2110	47.5	26.77
21067271	2011	6	4	2120	47.5	26.77
21067271	2011	6	4	2130	47.51	26.76
21067271	2011	6	4	2140	47.67	26.76
21067271	2011	6	4	2150	47.77	26.75
21067271	2011	6	4	2160	47.55	26.75
21067271	2011	6	4	2170	47.02	26.75

21067271	2011	6	4	2180	47.1	26.75
21067271	2011	6	4	2190	47.67	26.75
21067271	2011	6	4	2200	48.32	26.74
21067271	2011	6	4	2210	48.31	26.73
21067271	2011	6	4	2220	48.26	26.72
21067271	2011	6	4	2230	48.76	26.72
21067271	2011	6	4	2240	48.87	26.71
21067271	2011	6	4	2250	49.07	26.71
21067271	2011	6	4	2260	49.07	26.71
21067271	2011	6	4	2270	49.24	26.7
21067271	2011	6	4	2280	49.4	26.7
21067271	2011	6	4	2290	48.55	26.69
21067271	2011	6	4	2300	48.17	26.69
21067271	2011	6	4	2310	47.87	26.67
21067271	2011	6	4	2320	47.77	26.68
21067271	2011	6	4	2330	47.85	26.67
21067271	2011	6	4	2340	47.4	26.67
21067271	2011	6	4	2350	47.13	26.67
21067271	2011	6	4	2360	46.66	26.68
21067271	2011	6	4	2370	46.24	26.69
21067271	2011	6	4	2380	46.44	26.69
21067271	2011	6	4	2390	46.55	26.69
21067271	2011	6	4	2400	45.93	26.71
21067271	2011	6	4	2410	45.38	26.72
21067271	2011	6	4	2420	45.08	26.73
21067271	2011	6	4	2430	45.21	26.74
21067271	2011	6	4	2440	45.52	26.76
21067271	2011	6	4	2450	45.87	26.78
21067271	2011	6	4	2460	46.1	26.79
21067271	2011	6	4	2470	46.48	26.8
21067271	2011	6	4	2480	47.84	26.78
21067271	2011	6	4	2490	48.72	26.77
21067271	2011	6	4	2500	49.28	26.77
21067271	2011	6	4	2510	49.69	26.76
21067271	2011	6	4	2520	50.17	26.75
21067271	2011	6	4	2530	50.78	26.72
21067271	2011	6	4	2540	51.36	26.7
21067271	2011	6	4	2550	51.74	26.67
21067271	2011	6	4	2560	51.93	26.65
21067271	2011	6	4	2570	51.8	26.63
21067271	2011	6	4	2580	51.58	26.61
21067271	2011	6	4	2590	51.48	26.6
21067271	2011	6	4	2600	51.38	26.58
21067271	2011	6	4	2610	51.38	26.57
21067271	2011	6	4	2620	51.38	26.56
21067271	2011	6	4	2630	51.51	26.55
21067271	2011	6	4	2640	51.69	26.55

21067271	2011	6	4	2650	51.71	26.53
21067271	2011	6	4	2660	51.69	26.52
21067271	2011	6	4	2670	51.7	26.51
21067271	2011	6	4	2680	51.81	26.5
21067271	2011	6	4	2690	51.91	26.48
21067271	2011	6	4	2700	51.77	26.47
21067271	2011	6	4	2710	51.6	26.46
21067271	2011	6	4	2720	51.72	26.45
21067271	2011	6	4	2730	51.82	26.44
21067271	2011	6	4	2740	52.71	26.42
21067271	2011	6	4	2750	52.62	26.41
21067271	2011	6	4	2760	52.62	26.4
21067271	2011	6	4	2770	52.01	26.4
21067271	2011	6	4	2780	51.76	26.41
21067271	2011	6	4	2790	51.89	26.4
21067271	2011	6	4	2800	52.1	26.4
21067271	2011	6	4	2810	52.2	26.39
21067271	2011	6	4	2820	51.4	26.4
21067271	2011	6	4	2830	51.25	26.39
21067271	2011	6	4	2840	51.19	26.4
21067271	2011	6	4	2850	51.12	26.39
21067271	2011	6	4	2860	51.24	26.39
21067271	2011	6	4	2870	51.3	26.39
21067271	2011	6	4	2880	51.23	26.39
21067271	2011	6	4	2890	51.13	26.39
21067271	2011	6	4	2900	51.02	26.39
21067271	2011	6	4	2910	50.92	26.39
21067271	2011	6	4	2920	51.04	26.39
21067271	2011	6	4	2930	51.29	26.39
21067271	2011	6	4	2940	51.52	26.39
21067271	2011	6	4	2950	51.66	26.38
21067271	2011	6	4	2960	51.4	26.36
21067271	2011	6	4	2970	51.24	26.36
21067271	2011	6	4	2980	51.88	26.35
21067271	2011	6	4	2990	52.4	26.35
21067271	2011	6	4	3000	52.61	26.34
21067271	2011	6	4	3010	52.3	26.33
21067271	2011	6	4	3020	52.52	26.32
21067271	2011	6	4	3030	53.71	26.31
21067271	2011	6	4	3040	55.24	26.28
21067271	2011	6	4	3050	57.56	26.24
21067271	2011	6	4	3060	60.72	26.2
21067271	2011	6	4	3070	63.88	26.15
21067271	2011	6	4	3080	66.74	26.1
21067271	2011	6	4	3090	69.5	26.07
21067271	2011	6	4	3100	71.96	26
21067271	2011	6	4	3110	74.14	25.96

21067271	2011	6	4	3120	76.76	25.85
21067271	2011	6	4	3130	79.73	25.74
21067271	2011	6	4	3140	82.09	25.67
21067271	2011	6	4	3150	83.17	25.67
21067271	2011	6	4	3160	83.91	25.65
21067271	2011	6	4	3170	84.59	25.62
21067271	2011	6	4	3180	85.16	25.6
21067271	2011	6	4	3190	85.56	25.57
21067271	2011	6	4	3200	85.87	25.53
21067271	2011	6	4	3210	85.85	25.49
21067271	2011	6	4	3220	85.71	25.46
21067271	2011	6	4	3230	85.54	25.43
21067271	2011	6	4	3240	85.68	25.39
21067271	2011	6	4	3250	85.89	25.35
21067271	2011	6	4	3260	85.9	25.33
21067271	2011	6	4	3270	85.84	25.31
21067271	2011	6	4	3280	85.81	25.28
21067271	2011	6	4	3290	86.7	25.24
21067271	2011	6	4	3300	88.07	25.17
21067271	2011	6	4	3310	88.38	25.14
21067271	2011	6	4	3320	88.65	25.1
21067271	2011	6	4	3330	89.32	25.04
21067271	2011	6	4	3340	89.88	25.03
21067271	2011	6	4	3350	90.03	25.03
21067271	2011	6	4	3360	89.98	25
21067271	2011	6	4	3370	90.03	25
21067271	2011	6	4	3380	90.02	24.98
21067271	2011	6	4	3390	89.9	24.97
21067271	2011	6	4	3400	89.64	24.96
21067271	2011	6	4	3410	89.63	24.93
21067271	2011	6	4	3420	89.58	24.92
21067271	2011	6	4	3430	89.55	24.89
21067271	2011	6	4	3440	89.74	24.87
21067271	2011	6	4	3450	89.99	24.84
21067271	2011	6	4	3460	90.16	24.82
21067271	2011	6	4	3470	90.24	24.81
21067271	2011	6	4	3480	90.19	24.79
21067271	2011	6	4	3490	90.03	24.78
21067271	2011	6	4	3500	89.88	24.78
21067271	2011	6	4	3510	89.7	24.76
21067271	2011	6	4	3520	89.62	24.75
21067271	2011	6	4	3530	89.52	24.74
21067271	2011	6	4	3540	89.62	24.74
21067271	2011	6	4	3550	89.91	24.71
21067271	2011	6	4	3560	90.09	24.71
21067271	2011	6	4	3570	90.39	24.68
21067271	2011	6	4	3580	91.1	24.67

21067271	2011	6	4	3590	91.71	24.67
21067271	2011	6	4	3600	92.26	24.65
21067271	2011	6	4	3610	93.39	24.64
21067271	2011	6	4	3620	94.53	24.61
21067271	2011	6	4	3630	94.98	24.6
21067271	2011	6	4	3640	95.78	24.6
21067271	2011	6	4	3650	96.82	24.57
21067271	2011	6	4	3660	98.47	24.54
21067271	2011	6	4	3670	99.37	24.5
21067271	2011	6	4	3680	100.36	24.47
21067271	2011	6	4	3690	101.55	24.45
21067271	2011	6	4	3700	102.84	24.42
21067271	2011	6	4	3710	104.65	24.41
21067271	2011	6	4	3720	106.33	24.35
21067271	2011	6	4	3730	107.53	24.32
21067271	2011	6	4	3740	108.88	24.28
21067271	2011	6	4	3750	110.5	24.23
21067271	2011	6	4	3760	112.17	24.17
21067271	2011	6	4	3770	112.81	24.12
21067271	2011	6	4	3780	112.94	24.07
21067271	2011	6	4	3790	112.93	24.04
21067271	2011	6	4	3800	112.76	23.99
21067271	2011	6	4	3810	113.46	23.94
21067271	2011	6	4	3820	114.45	23.87
21067271	2011	6	4	3830	115.07	23.84
21067271	2011	6	4	3840	115.57	23.8
21067271	2011	6	4	3850	116.03	23.73
21067271	2011	6	4	3860	116.5	23.68
21067271	2011	6	4	3870	116.9	23.63
21067271	2011	6	4	3880	117.18	23.56
21067271	2011	6	4	3890	117.47	23.5
21067271	2011	6	4	3900	118.38	23.42
21067271	2011	6	4	3910	118.76	23.39
21067271	2011	6	4	3920	118.81	23.35
21067271	2011	6	4	3930	118.7	23.31
21067271	2011	6	4	3940	119.32	23.25
21067271	2011	6	4	3950	119.94	23.19
21067271	2011	6	4	3960	120.22	23.16
21067271	2011	6	4	3970	120.71	23.14
21067271	2011	6	4	3980	121.13	23.09
21067271	2011	6	4	3990	121.53	23.05
21067271	2011	6	4	4000	121.96	23.01
21067271	2011	6	4	4010	123.07	22.98
21067271	2011	6	4	4020	124.38	22.93
21067271	2011	6	4	4030	124.72	22.91
21067271	2011	6	4	4040	124.8	22.9
21067271	2011	6	4	4050	124.69	22.87

21067271	2011	6	4	4060	124.92	22.85
21067271	2011	6	4	4070	124.98	22.8
21067271	2011	6	4	4080	124.9	22.78
21067271	2011	6	4	4090	124.81	22.75
21067271	2011	6	4	4100	124.78	22.71
21067271	2011	6	4	4110	124.63	22.7
21067271	2011	6	4	4120	124.64	22.67
21067271	2011	6	4	4130	124.5	22.64
21067271	2011	6	4	4140	124.35	22.63
21067271	2011	6	4	4150	124.16	22.61
21067271	2011	6	4	4160	123.91	22.59
21067271	2011	6	4	4170	124.23	22.56
21067271	2011	6	4	4180	125.5	22.53
21067271	2011	6	4	4190	126.18	22.52
21067271	2011	6	4	4200	126.11	22.5
21067271	2011	6	4	4210	126.23	22.5
21067271	2011	6	4	4220	126.77	22.46
21067271	2011	6	4	4230	127.1	22.43
21067271	2011	6	4	4240	127.32	22.41
21067271	2011	6	4	4250	127.41	22.39
21067271	2011	6	4	4260	127.41	22.37
21067271	2011	6	4	4270	127.54	22.35
21067271	2011	6	4	4280	127.52	22.33
21067271	2011	6	4	4290	127.33	22.32
21067271	2011	6	4	4300	126.84	22.3
21067271	2011	6	4	4310	126.51	22.28
21067271	2011	6	4	4320	127.28	22.27
21067271	2011	6	4	4330	127.67	22.25
21067271	2011	6	4	4340	127.41	22.24
21067271	2011	6	4	4350	127.27	22.23
21067271	2011	6	4	4360	127.3	22.22
21067271	2011	6	4	4370	127.77	22.2
21067271	2011	6	4	4380	128.42	22.18
21067271	2011	6	4	4390	128.33	22.17
21067271	2011	6	4	4400	127.89	22.17
21067271	2011	6	4	4410	127.08	22.16
21067271	2011	6	4	4420	126.93	22.14
21067271	2011	6	4	4430	127.11	22.14
21067271	2011	6	4	4440	127.19	22.13
21067271	2011	6	4	4450	127.16	22.12
21067271	2011	6	4	4460	127.29	22.11
21067271	2011	6	4	4470	127.26	22.11
21067271	2011	6	4	4480	127	22.1
21067271	2011	6	4	4490	125.36	22.1
21067271	2011	6	4	4500	123.76	22.1
21067271	2011	6	4	4510	122.27	22.1
21067271	2011	6	4	4520	120.7	22.12

21067271	2011	6	4	4530	118.77	22.14
21067271	2011	6	4	4540	117.08	22.15
21067271	2011	6	4	4550	115.2	22.17
21067271	2011	6	4	4560	113.25	22.21
21067271	2011	6	4	4570	111.84	22.27
21067271	2011	6	4	4580	111.39	22.32
21067271	2011	6	4	4590	112.6	22.37
21067271	2011	6	4	4600	113.5	22.41
21067271	2011	6	4	4610	113.52	22.45
21067271	2011	6	4	4620	114.25	22.47
21067271	2011	6	4	4630	114.32	22.51
21067271	2011	6	4	4640	114.37	22.52
21067271	2011	6	4	4650	114.19	22.55
21067271	2011	6	4	4660	114.43	22.57
21067271	2011	6	4	4670	113.15	22.6
21067271	2011	6	4	4680	111.6	22.63
21067271	2011	6	4	4690	110.63	22.66
21067271	2011	6	4	4700	108.87	22.71
21067271	2011	6	4	4710	107.71	22.75
21067271	2011	6	4	4720	107.33	22.78
21067271	2011	6	4	4730	107.69	22.81
21067271	2011	6	4	4740	107.76	22.85
21067271	2011	6	4	4750	107.58	22.86
21067271	2011	6	4	4760	107.48	22.89
21067271	2011	6	4	4770	107.67	22.9
21067271	2011	6	4	4780	107.54	22.92
21067271	2011	6	4	4790	107.51	22.96
21067271	2011	6	4	4800	107.52	22.96
21067271	2011	6	4	4810	107.66	22.98
21067271	2011	6	4	4820	107.07	23
21067271	2011	6	4	4830	105.22	23.03
21067271	2011	6	4	4840	102.77	23.06
21067271	2011	6	4	4850	101.16	23.1
21067271	2011	6	4	4860	99.43	23.16
21067271	2011	6	4	4870	96.96	23.17
21067271	2011	6	4	4880	95.2	23.23
21067271	2011	6	4	4890	93.41	23.29
21067271	2011	6	4	4900	91.09	23.35
21067271	2011	6	4	4910	90.15	23.4
21067271	2011	6	4	4920	90.34	23.44
21067271	2011	6	4	4930	88.56	23.5
21067271	2011	6	4	4940	86.42	23.56
21067271	2011	6	4	4950	83.8	23.64
21067271	2011	6	4	4960	81.34	23.69
21067271	2011	6	4	4970	80.09	23.74
21067271	2011	6	4	4980	78.97	23.81
21067271	2011	6	4	4990	77.54	23.89

21067271	2011	6	4	5000	76.6	23.95
21067271	2011	6	4	5010	76.77	24
21067271	2011	6	4	5020	76.69	24.07
21067271	2011	6	4	5030	75.93	24.13
21067271	2011	6	4	5040	74.38	24.2
21067271	2011	6	4	5050	73.49	24.25
21067271	2011	6	4	5060	72.2	24.28
21067271	2011	6	4	5070	71.05	24.33
21067271	2011	6	4	5080	69.53	24.36
21067271	2011	6	4	5090	68.22	24.4
21067271	2011	6	4	5100	66.38	24.47
21067271	2011	6	4	5110	64.56	24.53
21067271	2011	6	4	5120	63.26	24.58
21067271	2011	6	4	5130	61.71	24.63
21067271	2011	6	4	5140	59.84	24.7
21067271	2011	6	4	5150	59.09	24.75
21067271	2011	6	4	5160	58.74	24.81
21067271	2011	6	4	5170	57.29	24.86
21067271	2011	6	4	5180	56.99	24.93
21067271	2011	6	4	5190	57.08	24.98
21067271	2011	6	4	5200	57.17	25.01
21067271	2011	6	4	5210	57.4	25.05
21067271	2011	6	4	5220	57.68	25.07
21067271	2011	6	4	5230	58.03	25.1
21067271	2011	6	4	5240	58.15	25.13
21067271	2011	6	4	5250	58.24	25.15
21067271	2011	6	4	5260	58.44	25.19
21067271	2011	6	4	5270	58.54	25.21
21067271	2011	6	4	5280	58.43	25.23
21067271	2011	6	4	5290	58.2	25.25
21067271	2011	6	4	5300	58.3	25.28
21067271	2011	6	4	5310	57.8	25.3
21067271	2011	6	4	5320	57.54	25.32
21067271	2011	6	4	5330	57.54	25.34
21067271	2011	6	4	5340	57.5	25.36
21067271	2011	6	4	5350	57.27	25.39
21067271	2011	6	4	5360	57.1	25.42
21067271	2011	6	4	5370	56.94	25.43
21067271	2011	6	4	5380	56.84	25.45
21067271	2011	6	4	5390	56.93	25.46
21067271	2011	6	4	5400	56.31	25.48
21067271	2011	6	4	5410	56.32	25.5
21067271	2011	6	4	5420	57.25	25.51
21067271	2011	6	4	5430	58.15	25.52
21067271	2011	6	4	5440	58.57	25.52
21067271	2011	6	4	5450	58.09	25.53
21067271	2011	6	4	5460	56.61	25.55

21067271	2011	6	4	5470	55.99	25.57
21067271	2011	6	4	5480	55.49	25.58
21067271	2011	6	4	5490	55.4	25.59
21067271	2011	6	4	5500	54.95	25.6
21067271	2011	6	4	5510	54.87	25.62
21067271	2011	6	4	5520	55.22	25.63
21067271	2011	6	4	5530	55.32	25.64
21067271	2011	6	4	5540	54.79	25.65
21067271	2011	6	4	5550	54.05	25.66
21067271	2011	6	4	5560	53.86	25.67
21067271	2011	6	4	5570	53.62	25.67
21067271	2011	6	4	5580	53.62	25.68
21067271	2011	6	4	5590	53.64	25.69
21067271	2011	6	4	5600	53.39	25.71
21067271	2011	6	4	5610	52.54	25.71
21067271	2011	6	4	5620	52.46	25.72
21067271	2011	6	4	5630	52.75	25.72
21067271	2011	6	4	5640	53.08	25.73
21067271	2011	6	4	5650	53.75	25.73
21067271	2011	6	4	5660	53.94	25.73
21067271	2011	6	4	5670	53.97	25.74
21067271	2011	6	4	5680	53.91	25.75
21067271	2011	6	4	5690	53.9	25.74
21067271	2011	6	4	5700	53.96	25.75
21067271	2011	6	4	5710	53.98	25.75
21067271	2011	6	4	5720	53.83	25.75
21067271	2011	6	4	5730	53.83	25.76
21067271	2011	6	4	5740	53.84	25.75
21067271	2011	6	4	5750	53.89	25.76
21067271	2011	6	4	5760	53.26	25.77
21067271	2011	6	4	5770	52.38	25.78
21067271	2011	6	4	5780	52.03	25.78
21067271	2011	6	4	5790	51.08	25.8
21067271	2011	6	4	5800	50.77	25.81
21067271	2011	6	4	5810	49.49	25.83
21067271	2011	6	4	5820	48.64	25.85
21067271	2011	6	4	5830	48.71	25.89
21067271	2011	6	4	5840	48.69	25.93
21067271	2011	6	4	5850	48.43	25.96
21067271	2011	6	4	5860	48.54	25.99
21067271	2011	6	4	5870	48.28	26.02
21067271	2011	6	4	5880	48.1	26.03
21067271	2011	6	4	5890	47.08	26.07
21067271	2011	6	4	5900	47.08	26.11
21067271	2011	6	4	5910	46.24	26.15
21067271	2011	6	4	5920	45.25	26.21
21067271	2011	6	4	5930	43.82	26.25

21067271	2011	6	4	5940	43.29	26.32
21067271	2011	6	4	5950	42.85	26.46
21067271	2011	6	4	5960	42.04	26.56
21067271	2011	6	4	5970	41.42	26.67
21067271	2011	6	4	5980	40.15	26.8
21067271	2011	6	4	5990	39.05	26.85
21067271	2011	6	4	6000	37.99	26.95
21067271	2011	6	4	6010	36.26	27
21067271	2011	6	4	6020	35.03	27.09
21067271	2011	6	4	6030	35.41	27.18
21067271	2011	6	4	6040	35.45	27.26
21067271	2011	6	4	6050	35.34	27.3
21067271	2011	6	4	6060	35.18	27.33
21067271	2011	6	4	6070	34.68	27.39
21067271	2011	6	4	6080	33.75	27.44
21067271	2011	6	4	6090	31.85	27.51
21067271	2011	6	4	6100	29.73	27.57
21067271	2011	6	4	6110	27.71	27.64
21067271	2011	6	4	6120	25.7	27.71
21067271	2011	6	4	6130	23.72	27.78
21067271	2011	6	4	6140	21.66	27.82
21067271	2011	6	4	6150	19.8	27.87
21067271	2011	6	4	6160	17.49	27.93
21067271	2011	6	4	6170	16.19	27.97
21067271	2011	6	4	6180	14.49	28.03
21067271	2011	6	4	6190	12.93	28.07
21067271	2011	6	4	6200	11.12	28.11
21067271	2011	6	4	6210	9.16	28.16
21067271	2011	6	4	6220	6.82	28.2
21067271	2011	6	4	6230	4.72	28.25
21067271	2011	6	4	6240	3.1	28.28
21067271	2011	6	4	6250	2.93	28.3
21067271	2011	6	4	6260	2.92	28.34
21067271	2011	6	4	6270	2.5	28.37
21067271	2011	6	4	6280	2.82	28.4
21067271	2011	6	4	6290	2.43	28.42
21067271	2011	6	4	6300	2.18	28.43
21067271	2011	6	4	6310	0.66	28.48
21067271	2011	6	4	6320	0.64	28.52
21067271	2011	6	4	6330	0.58	28.53
21067271	2011	6	4	6340	0.61	28.55
21067271	2011	6	4	6350	0.61	28.57
21067271	2011	6	4	6360	0.59	28.6
21067271	2011	6	4	6370	0.61	28.62
21067271	2011	6	4	6380	0.58	28.64
21067271	2011	6	4	6390	0.56	28.67
21067271	2011	6	4	6400	0.65	28.68

21067271	2011	6	4	6410	0.6	28.7
21067271	2011	6	4	6420	0.64	28.71
21067271	2011	6	4	6430	0.67	28.73
21067271	2011	6	4	6440	0.6	28.75
21067271	2011	6	4	6450	0.48	28.76
21233721	2011	6	6	0	1.06	29.01
21233721	2011	6	6	10	2.48	29
21233721	2011	6	6	20	2.81	29
21233721	2011	6	6	30	2.81	29
21233721	2011	6	6	40	3.22	29
21233721	2011	6	6	50	3.24	28.99
21233721	2011	6	6	60	3.25	28.99
21233721	2011	6	6	70	1.36	29
21233721	2011	6	6	80	0.68	29.01
21233721	2011	6	6	90	0.7	29.01
21233721	2011	6	6	100	0.72	29.01
21233721	2011	6	6	110	0.68	29.02
21233721	2011	6	6	120	0.72	29.01
21233721	2011	6	6	130	0.7	29.01
21233721	2011	6	6	140	0.71	29.01
21233721	2011	6	6	150	0.72	29.01
21233721	2011	6	6	160	0.7	29.01
21233721	2011	6	6	170	0.78	29.01
21233721	2011	6	6	180	1.13	29
21233721	2011	6	6	190	1.77	29
21233721	2011	6	6	200	2.22	29
21233721	2011	6	6	210	2.54	29
21233721	2011	6	6	220	2.82	28.99
21233721	2011	6	6	230	2.5	29
21233721	2011	6	6	240	2.9	29
21233721	2011	6	6	250	3.46	28.99
21233721	2011	6	6	260	4.23	28.98
21233721	2011	6	6	270	4.59	28.98
21233721	2011	6	6	280	4.98	28.97
21233721	2011	6	6	290	5.6	28.97
21233721	2011	6	6	300	7.28	28.96
21233721	2011	6	6	310	8.56	28.94
21233721	2011	6	6	320	9.91	28.92
21233721	2011	6	6	330	11.48	28.92
21233721	2011	6	6	340	12.74	28.91
21233721	2011	6	6	350	13.23	28.91
21233721	2011	6	6	360	13.62	28.9
21233721	2011	6	6	370	13.46	28.9
21233721	2011	6	6	380	13.98	28.9
21233721	2011	6	6	390	14.25	28.89
21233721	2011	6	6	400	14.14	28.89
21233721	2011	6	6	410	13.75	28.89

21233721	2011	6	6	420	13.69	28.9
21233721	2011	6	6	430	13.46	28.9
21233721	2011	6	6	440	13.53	28.9
21233721	2011	6	6	450	13.56	28.9
21233721	2011	6	6	460	14.82	28.89
21233721	2011	6	6	470	16.14	28.88
21233721	2011	6	6	480	17.8	28.85
21233721	2011	6	6	490	19.84	28.84
21233721	2011	6	6	500	22.13	28.8
21233721	2011	6	6	510	24.43	28.76
21233721	2011	6	6	520	26.85	28.72
21233721	2011	6	6	530	29.02	28.69
21233721	2011	6	6	540	31.19	28.65
21233721	2011	6	6	550	32.65	28.64
21233721	2011	6	6	560	33.92	28.64
21233721	2011	6	6	570	35.09	28.63
21233721	2011	6	6	580	36.39	28.6
21233721	2011	6	6	590	37.75	28.58
21233721	2011	6	6	600	39.32	28.55
21233721	2011	6	6	610	40.74	28.48
21233721	2011	6	6	620	41.41	28.42
21233721	2011	6	6	630	42.96	28.31
21233721	2011	6	6	640	45.07	28.12
21233721	2011	6	6	650	47.28	27.91
21233721	2011	6	6	660	49.61	27.7
21233721	2011	6	6	670	51.75	27.47
21233721	2011	6	6	680	53.99	27.4
21233721	2011	6	6	690	56.58	27.32
21233721	2011	6	6	700	59.89	27.26
21233721	2011	6	6	710	63.05	27.21
21233721	2011	6	6	720	65.45	27.14
21233721	2011	6	6	730	66.82	27.01
21233721	2011	6	6	740	67.98	26.96
21233721	2011	6	6	750	68.77	26.91
21233721	2011	6	6	760	69.52	26.86
21233721	2011	6	6	770	70.35	26.83
21233721	2011	6	6	780	72.02	26.75
21233721	2011	6	6	790	73.03	26.64
21233721	2011	6	6	800	74.36	26.59
21233721	2011	6	6	810	76.17	26.53
21233721	2011	6	6	820	77.35	26.5
21233721	2011	6	6	830	78.14	26.43
21233721	2011	6	6	840	79.66	26.41
21233721	2011	6	6	850	80.55	26.35
21233721	2011	6	6	860	81.44	26.33
21233721	2011	6	6	870	82.64	26.3
21233721	2011	6	6	880	84.86	26.18

21233721	2011	6	6	890	86.22	26.1
21233721	2011	6	6	900	87.97	25.99
21233721	2011	6	6	910	88.85	25.92
21233721	2011	6	6	920	88.43	25.85
21233721	2011	6	6	930	88.22	25.79
21233721	2011	6	6	940	88.32	25.68
21233721	2011	6	6	950	88.5	25.6
21233721	2011	6	6	960	89.72	25.53
21233721	2011	6	6	970	90.69	25.47
21233721	2011	6	6	980	92.04	25.4
21233721	2011	6	6	990	92.87	25.26
21233721	2011	6	6	1000	93.39	25.18
21233721	2011	6	6	1010	95.56	24.97
21233721	2011	6	6	1020	97.81	24.92
21233721	2011	6	6	1030	99.6	24.89
21233721	2011	6	6	1040	101.37	24.83
21233721	2011	6	6	1050	102.97	24.73
21233721	2011	6	6	1060	104.23	24.67
21233721	2011	6	6	1070	105.66	24.6
21233721	2011	6	6	1080	106.54	24.54
21233721	2011	6	6	1090	108.33	24.37
21233721	2011	6	6	1100	109.31	24.32
21233721	2011	6	6	1110	109.95	24.25
21233721	2011	6	6	1120	110.26	24.19
21233721	2011	6	6	1130	110.49	24.12
21233721	2011	6	6	1140	110.29	24.05
21233721	2011	6	6	1150	110.14	24
21233721	2011	6	6	1160	110.37	23.95
21233721	2011	6	6	1170	110.87	23.87
21233721	2011	6	6	1180	111.58	23.84
21233721	2011	6	6	1190	112.07	23.81
21233721	2011	6	6	1200	112.39	23.76
21233721	2011	6	6	1210	112.82	23.71
21233721	2011	6	6	1220	113.34	23.67
21233721	2011	6	6	1230	113.68	23.63
21233721	2011	6	6	1240	113.94	23.57
21233721	2011	6	6	1250	114.4	23.52
21233721	2011	6	6	1260	114.98	23.49
21233721	2011	6	6	1270	115.56	23.45
21233721	2011	6	6	1280	117.84	23.39
21233721	2011	6	6	1290	120.08	23.32
21233721	2011	6	6	1300	121.79	23.27
21233721	2011	6	6	1310	122.86	23.23
21233721	2011	6	6	1320	124.09	23.17
21233721	2011	6	6	1330	124.66	23.11
21233721	2011	6	6	1340	125.18	23.08
21233721	2011	6	6	1350	125.45	23.03

21233721	2011	6	6	1360	125.34	23
21233721	2011	6	6	1370	125.42	22.98
21233721	2011	6	6	1380	125.46	22.94
21233721	2011	6	6	1390	125.45	22.92
21233721	2011	6	6	1400	125.58	22.9
21233721	2011	6	6	1410	125.52	22.82
21233721	2011	6	6	1420	125.56	22.82
21233721	2011	6	6	1430	125.48	22.77
21233721	2011	6	6	1440	125.55	22.76
21233721	2011	6	6	1450	125.73	22.74
21233721	2011	6	6	1460	125.8	22.71
21233721	2011	6	6	1470	125.37	22.7
21233721	2011	6	6	1480	125.49	22.68
21233721	2011	6	6	1490	125.4	22.67
21233721	2011	6	6	1500	125.38	22.64
21233721	2011	6	6	1510	125.54	22.61
21233721	2011	6	6	1520	125.58	22.6
21233721	2011	6	6	1530	125.65	22.59
21233721	2011	6	6	1540	125.7	22.56
21233721	2011	6	6	1550	125.81	22.53
21233721	2011	6	6	1560	125.74	22.53
21233721	2011	6	6	1570	125.72	22.52
21233721	2011	6	6	1580	125.6	22.5
21233721	2011	6	6	1590	125.74	22.5
21233721	2011	6	6	1600	125.77	22.48
21233721	2011	6	6	1610	125.9	22.47
21233721	2011	6	6	1620	126.18	22.46
21233721	2011	6	6	1630	126.51	22.45
21233721	2011	6	6	1640	126.56	22.44
21233721	2011	6	6	1650	126.39	22.43
21233721	2011	6	6	1660	126.39	22.42
21233721	2011	6	6	1670	126.25	22.41
21233721	2011	6	6	1680	125.7	22.41
21233721	2011	6	6	1690	125.66	22.41
21233721	2011	6	6	1700	125.79	22.39
21233721	2011	6	6	1710	125.75	22.39
21233721	2011	6	6	1720	125.74	22.39
21233721	2011	6	6	1730	126.06	22.39
21233721	2011	6	6	1740	126.42	22.37
21233721	2011	6	6	1750	126.77	22.36
21233721	2011	6	6	1760	127.85	22.35
21233721	2011	6	6	1770	130.18	22.33
21233721	2011	6	6	1780	131.89	22.29
21233721	2011	6	6	1790	132.31	22.25
21233721	2011	6	6	1800	132.53	22.25
21233721	2011	6	6	1810	133.37	22.21
21233721	2011	6	6	1820	134.22	22.17

21233721	2011	6	6	1830	134.98	22.14
21233721	2011	6	6	1840	135.47	22.11
21233721	2011	6	6	1850	137.15	22.07
21233721	2011	6	6	1860	137.54	22.05
21233721	2011	6	6	1870	137.39	22.02
21233721	2011	6	6	1880	137.33	21.98
21233721	2011	6	6	1890	137.36	21.93
21233721	2011	6	6	1900	137.2	21.91
21233721	2011	6	6	1910	137.39	21.88
21233721	2011	6	6	1920	137.44	21.84
21233721	2011	6	6	1930	137.78	21.82
21233721	2011	6	6	1940	137.71	21.77
21233721	2011	6	6	1950	137.72	21.73
21233721	2011	6	6	1960	137.79	21.71
21233721	2011	6	6	1970	137.95	21.69
21233721	2011	6	6	1980	137.87	21.66
21233721	2011	6	6	1990	138	21.64
21233721	2011	6	6	2000	138.07	21.62
21233721	2011	6	6	2010	139.24	21.58
21233721	2011	6	6	2020	139.99	21.57
21233721	2011	6	6	2030	140.4	21.55
21233721	2011	6	6	2040	140.18	21.53
21233721	2011	6	6	2050	140.23	21.51
21233721	2011	6	6	2060	140.22	21.49
21233721	2011	6	6	2070	140.34	21.47
21233721	2011	6	6	2080	140.24	21.45
21233721	2011	6	6	2090	140.26	21.43
21233721	2011	6	6	2100	140.48	21.42
21233721	2011	6	6	2110	140.35	21.41
21233721	2011	6	6	2120	139.77	21.39
21233721	2011	6	6	2130	139.73	21.39
21233721	2011	6	6	2140	140.18	21.37
21233721	2011	6	6	2150	140.78	21.35
21233721	2011	6	6	2160	141.02	21.33
21233721	2011	6	6	2170	141.16	21.33
21233721	2011	6	6	2180	141.1	21.32
21233721	2011	6	6	2190	140.93	21.3
21233721	2011	6	6	2200	141.19	21.29
21233721	2011	6	6	2210	141.46	21.29
21233721	2011	6	6	2220	141.28	21.28
21233721	2011	6	6	2230	141.2	21.28
21233721	2011	6	6	2240	141.22	21.27
21233721	2011	6	6	2250	141.18	21.25
21233721	2011	6	6	2260	141.17	21.25
21233721	2011	6	6	2270	141.45	21.25
21233721	2011	6	6	2280	141.61	21.24
21233721	2011	6	6	2290	142.01	21.2

21233721	2011	6	6	2300	142.28	21.19
21233721	2011	6	6	2310	142.59	21.17
21233721	2011	6	6	2320	143.91	21.16
21233721	2011	6	6	2330	146.52	21.12
21233721	2011	6	6	2340	147.34	21.08
21233721	2011	6	6	2350	147.9	21.05
21233721	2011	6	6	2360	148.11	21
21233721	2011	6	6	2370	148.14	20.96
21233721	2011	6	6	2380	147.96	20.87
21233721	2011	6	6	2390	147.97	20.84
21233721	2011	6	6	2400	147.93	20.75
21233721	2011	6	6	2410	148.3	20.72
21233721	2011	6	6	2420	147.79	20.7
21233721	2011	6	6	2430	147.6	20.64
21233721	2011	6	6	2440	147.86	20.61
21233721	2011	6	6	2450	148.35	20.59
21233721	2011	6	6	2460	149.16	20.54
21233721	2011	6	6	2470	149.82	20.49
21233721	2011	6	6	2480	150.38	20.45
21233721	2011	6	6	2490	150.99	20.39
21233721	2011	6	6	2500	151.92	20.32
21233721	2011	6	6	2510	153.4	20.22
21233721	2011	6	6	2520	154.54	20.2
21233721	2011	6	6	2530	155.33	20.14
21233721	2011	6	6	2540	155.73	20.11
21233721	2011	6	6	2550	156.01	20.08
21233721	2011	6	6	2560	155.96	20.06
21233721	2011	6	6	2570	156.15	20.03
21233721	2011	6	6	2580	156.05	20.02
21233721	2011	6	6	2590	155.96	19.99
21233721	2011	6	6	2600	155.29	19.95
21233721	2011	6	6	2610	155.04	19.92
21233721	2011	6	6	2620	154.66	19.89
21233721	2011	6	6	2630	153.33	19.87
21233721	2011	6	6	2640	152.6	19.85
21233721	2011	6	6	2650	152.34	19.83
21233721	2011	6	6	2660	153.65	19.79
21233721	2011	6	6	2670	155.07	19.76
21233721	2011	6	6	2680	155.87	19.75
21233721	2011	6	6	2690	156.26	19.74
21233721	2011	6	6	2700	156.67	19.72
21233721	2011	6	6	2710	156.17	19.71
21233721	2011	6	6	2720	155.18	19.68
21233721	2011	6	6	2730	154.98	19.66
21233721	2011	6	6	2740	154.9	19.65
21233721	2011	6	6	2750	155.15	19.62
21233721	2011	6	6	2760	155.47	19.6

21233721	2011	6	6	2770	155.52	19.58
21233721	2011	6	6	2780	155.65	19.57
21233721	2011	6	6	2790	155.67	19.56
21233721	2011	6	6	2800	155.59	19.57
21233721	2011	6	6	2810	155.26	19.55
21233721	2011	6	6	2820	155.28	19.54
21233721	2011	6	6	2830	155.07	19.53
21233721	2011	6	6	2840	155.1	19.53
21233721	2011	6	6	2850	155.5	19.52
21233721	2011	6	6	2860	155.75	19.51
21233721	2011	6	6	2870	155.96	19.5
21233721	2011	6	6	2880	157.03	19.49
21233721	2011	6	6	2890	157.48	19.49
21233721	2011	6	6	2900	157.49	19.48
21233721	2011	6	6	2910	157.37	19.48
21233721	2011	6	6	2920	157.94	19.46
21233721	2011	6	6	2930	159.34	19.43
21233721	2011	6	6	2940	160.24	19.42
21233721	2011	6	6	2950	160.75	19.39
21233721	2011	6	6	2960	160.95	19.38
21233721	2011	6	6	2970	161.83	19.36
21233721	2011	6	6	2980	162.07	19.35
21233721	2011	6	6	2990	162.15	19.35
21233721	2011	6	6	3000	162.58	19.34
21233721	2011	6	6	3010	162.89	19.32
21233721	2011	6	6	3020	163.28	19.31
21233721	2011	6	6	3030	162.91	19.28
21233721	2011	6	6	3040	162.76	19.26
21233721	2011	6	6	3050	162.33	19.23
21233721	2011	6	6	3060	161.96	19.21
21233721	2011	6	6	3070	161.96	19.21
21233721	2011	6	6	3080	162.28	19.2
21233721	2011	6	6	3090	162.51	19.18
21233721	2011	6	6	3100	162.41	19.17
21233721	2011	6	6	3110	162.35	19.14
21233721	2011	6	6	3120	162.31	19.1
21233721	2011	6	6	3130	162.35	19.09
21233721	2011	6	6	3140	162.46	19.08
21233721	2011	6	6	3150	162.76	19.06
21233721	2011	6	6	3160	162.9	19.04
21233721	2011	6	6	3170	162.8	19.03
21233721	2011	6	6	3180	162.74	19.01
21233721	2011	6	6	3190	162.87	19
21233721	2011	6	6	3200	162.65	18.97
21233721	2011	6	6	3210	162.56	18.97
21233721	2011	6	6	3220	162.37	18.96
21233721	2011	6	6	3230	162.79	18.95

21233721	2011	6	6	3240	162.94	18.91
21233721	2011	6	6	3250	163.01	18.89
21233721	2011	6	6	3260	162.97	18.87
21233721	2011	6	6	3270	162.98	18.85
21233721	2011	6	6	3280	162.95	18.83
21233721	2011	6	6	3290	163.19	18.81
21233721	2011	6	6	3300	163.01	18.79
21233721	2011	6	6	3310	162.65	18.78
21233721	2011	6	6	3320	162.92	18.77
21233721	2011	6	6	3330	162.87	18.74
21233721	2011	6	6	3340	162.98	18.73
21233721	2011	6	6	3350	162.81	18.73
21233721	2011	6	6	3360	163.05	18.72
21233721	2011	6	6	3370	163.08	18.71
21233721	2011	6	6	3380	163.05	18.71
21233721	2011	6	6	3390	163	18.7
21233721	2011	6	6	3400	162.74	18.7
21233721	2011	6	6	3410	162.75	18.69
21233721	2011	6	6	3420	162.94	18.69
21233721	2011	6	6	3430	162.76	18.69
21233721	2011	6	6	3440	162.44	18.68
21233721	2011	6	6	3450	163.69	18.71
21233721	2011	6	6	3460	165.48	18.69
21233721	2011	6	6	3470	166.14	18.64
21233721	2011	6	6	3480	167.16	18.59
21233721	2011	6	6	3490	167.81	18.55
21233721	2011	6	6	3500	168.53	18.53
21233721	2011	6	6	3510	169.21	18.51
21233721	2011	6	6	3520	170.26	18.49
21233721	2011	6	6	3530	170.31	18.45
21233721	2011	6	6	3540	170.62	18.41
21233721	2011	6	6	3550	172.29	18.38
21233721	2011	6	6	3560	173.06	18.36
21233721	2011	6	6	3570	173.54	18.35
21233721	2011	6	6	3580	174.24	18.31
21233721	2011	6	6	3590	174.23	18.3
21233721	2011	6	6	3600	174.06	18.28
21233721	2011	6	6	3610	174	18.28
21233721	2011	6	6	3620	174.04	18.27
21233721	2011	6	6	3630	173.97	18.26
21233721	2011	6	6	3640	173.72	18.25
21233721	2011	6	6	3650	173.32	18.23
21233721	2011	6	6	3660	172.77	18.22
21233721	2011	6	6	3670	172.13	18.21
21233721	2011	6	6	3680	171.39	18.21
21233721	2011	6	6	3690	171.42	18.2
21233721	2011	6	6	3700	171.77	18.17

21233721	2011	6	6	3710	172.28	18.15
21233721	2011	6	6	3720	172.66	18.15
21233721	2011	6	6	3730	172.55	18.14
21233721	2011	6	6	3740	172.21	18.14
21233721	2011	6	6	3750	172.04	18.12
21233721	2011	6	6	3760	173.54	18.11
21233721	2011	6	6	3770	174.33	18.1
21233721	2011	6	6	3780	174.48	18.1
21233721	2011	6	6	3790	174.14	18.09
21233721	2011	6	6	3800	173.64	18.08
21233721	2011	6	6	3810	173.2	18.07
21233721	2011	6	6	3820	173.76	18.07
21233721	2011	6	6	3830	175.3	18.06
21233721	2011	6	6	3840	176.09	18.05
21233721	2011	6	6	3850	176.2	18.04
21233721	2011	6	6	3860	176.38	18.02
21233721	2011	6	6	3870	177.54	18.02
21233721	2011	6	6	3880	177.95	18.01
21233721	2011	6	6	3890	178.46	18
21233721	2011	6	6	3900	179.14	17.98
21233721	2011	6	6	3910	179.89	17.94
21233721	2011	6	6	3920	180.03	17.93
21233721	2011	6	6	3930	180	17.92
21233721	2011	6	6	3940	179.9	17.92
21233721	2011	6	6	3950	179.89	17.91
21233721	2011	6	6	3960	179.41	17.88
21233721	2011	6	6	3970	179.24	17.87
21233721	2011	6	6	3980	180.2	17.85
21233721	2011	6	6	3990	181.4	17.85
21233721	2011	6	6	4000	181.52	17.82
21233721	2011	6	6	4010	182.14	17.79
21233721	2011	6	6	4020	181.93	17.75
21233721	2011	6	6	4030	181.49	17.74
21233721	2011	6	6	4040	181.78	17.73
21233721	2011	6	6	4050	182.12	17.69
21233721	2011	6	6	4060	182.47	17.62
21233721	2011	6	6	4070	182.71	17.6
21233721	2011	6	6	4080	184.07	17.56
21233721	2011	6	6	4090	184.55	17.53
21233721	2011	6	6	4100	185.27	17.53
21233721	2011	6	6	4110	185.83	17.53
21233721	2011	6	6	4120	186.68	17.5
21233721	2011	6	6	4130	185.31	17.46
21233721	2011	6	6	4140	184.1	17.45
21233721	2011	6	6	4150	184.06	17.42
21233721	2011	6	6	4160	184.88	17.41
21233721	2011	6	6	4170	186.58	17.39

21233721	2011	6	6	4180	186.81	17.35
21233721	2011	6	6	4190	188.12	17.33
21233721	2011	6	6	4200	189.35	17.32
21233721	2011	6	6	4210	190.79	17.29
21233721	2011	6	6	4220	191.24	17.28
21233721	2011	6	6	4230	191.7	17.26
21233721	2011	6	6	4240	192.2	17.23
21233721	2011	6	6	4250	192.27	17.2
21233721	2011	6	6	4260	192.42	17.17
21233721	2011	6	6	4270	192.75	17.08
21233721	2011	6	6	4280	194.53	17.05
21233721	2011	6	6	4290	195.66	17.03
21233721	2011	6	6	4300	196.71	17.01
21233721	2011	6	6	4310	197.02	16.98
21233721	2011	6	6	4320	196.85	16.91
21233721	2011	6	6	4330	196.66	16.9
21233721	2011	6	6	4340	197.38	16.89
21233721	2011	6	6	4350	197.89	16.87
21233721	2011	6	6	4360	198.16	16.85
21233721	2011	6	6	4370	198.49	16.84
21233721	2011	6	6	4380	198.64	16.81
21233721	2011	6	6	4390	198.72	16.77
21233721	2011	6	6	4400	198.66	16.75
21233721	2011	6	6	4410	198.84	16.75
21233721	2011	6	6	4420	198.92	16.73
21233721	2011	6	6	4430	198.85	16.71
21233721	2011	6	6	4440	199.12	16.69
21233721	2011	6	6	4450	199.18	16.66
21233721	2011	6	6	4460	199.28	16.66
21233721	2011	6	6	4470	199.39	16.64
21233721	2011	6	6	4480	199.37	16.63
21233721	2011	6	6	4490	199.38	16.61
21233721	2011	6	6	4500	198.9	16.58
21233721	2011	6	6	4510	199.22	16.57
21233721	2011	6	6	4520	199.22	16.56
21233721	2011	6	6	4530	199.31	16.56
21233721	2011	6	6	4540	199.19	16.54
21233721	2011	6	6	4550	199.08	16.53
21233721	2011	6	6	4560	199.12	16.53
21233721	2011	6	6	4570	199.42	16.52
21233721	2011	6	6	4580	199.18	16.51
21233721	2011	6	6	4590	199.07	16.51
21233721	2011	6	6	4600	199.26	16.5
21233721	2011	6	6	4610	199.27	16.5
21233721	2011	6	6	4620	199.19	16.5
21233721	2011	6	6	4630	199.19	16.5
21233721	2011	6	6	4640	199.17	16.5

21233721	2011	6	6	4650	199.13	16.5
21233721	2011	6	6	4660	198.91	16.49
21233721	2011	6	6	4670	198.86	16.47
21233721	2011	6	6	4680	198.68	16.48
21233721	2011	6	6	4690	198.58	16.47
21233721	2011	6	6	4700	198.58	16.46
21233721	2011	6	6	4710	198.63	16.47
21233721	2011	6	6	4720	198.61	16.46
21233721	2011	6	6	4730	198.38	16.46
21233721	2011	6	6	4740	198.79	16.46
21233721	2011	6	6	4750	198.87	16.46
21233721	2011	6	6	4760	198.76	16.46
21233721	2011	6	6	4770	198.7	16.46
21233721	2011	6	6	4780	199.09	16.46
21233721	2011	6	6	4790	199.04	16.46
21233721	2011	6	6	4800	198.91	16.46
21233721	2011	6	6	4810	198.88	16.45
21233721	2011	6	6	4820	199.04	16.46
21233721	2011	6	6	4830	199.19	16.45
21233721	2011	6	6	4840	199.07	16.46
21233721	2011	6	6	4850	198.88	16.45
21233721	2011	6	6	4860	198.96	16.46
21233721	2011	6	6	4870	199.45	16.46
21233721	2011	6	6	4880	200.03	16.46
21233721	2011	6	6	4890	200.34	16.46
21233721	2011	6	6	4900	200.91	16.46
21233721	2011	6	6	4910	202.31	16.46
21233721	2011	6	6	4920	202.63	16.46
21233721	2011	6	6	4930	202.8	16.46
21233721	2011	6	6	4940	202.85	16.46
21233721	2011	6	6	4950	202.95	16.45
21233721	2011	6	6	4960	203.29	16.44
21233721	2011	6	6	4970	203.52	16.42
21233721	2011	6	6	4980	203.84	16.42
21233721	2011	6	6	4990	204.36	16.4
21233721	2011	6	6	5000	204.18	16.39
21233721	2011	6	6	5010	204.08	16.39
21233721	2011	6	6	5020	203.95	16.38
21233721	2011	6	6	5030	203.75	16.38
21233721	2011	6	6	5040	203.47	16.36
21233721	2011	6	6	5050	203.13	16.36
21233721	2011	6	6	5060	202.73	16.35
21233721	2011	6	6	5070	203.06	16.34
21233721	2011	6	6	5080	203.71	16.34
21233721	2011	6	6	5090	204.15	16.33
21233721	2011	6	6	5100	204.42	16.33
21233721	2011	6	6	5110	203.97	16.32

21233721	2011	6	6	5120	203.62	16.32
21233721	2011	6	6	5130	203.26	16.31
21233721	2011	6	6	5140	202.92	16.32
21233721	2011	6	6	5150	202.93	16.31
21233721	2011	6	6	5160	203.88	16.31
21233721	2011	6	6	5170	204.41	16.3
21233721	2011	6	6	5180	204.82	16.29
21233721	2011	6	6	5190	204.97	16.29
21233721	2011	6	6	5200	204.85	16.28
21233721	2011	6	6	5210	204.96	16.28
21233721	2011	6	6	5220	204.55	16.28
21233721	2011	6	6	5230	203.91	16.27
21233721	2011	6	6	5240	203.4	16.26
21233721	2011	6	6	5250	203.08	16.26
21233721	2011	6	6	5260	203.53	16.26
21233721	2011	6	6	5270	205.23	16.26
21233721	2011	6	6	5280	205.75	16.26
21233721	2011	6	6	5290	205.89	16.26
21233721	2011	6	6	5300	205.85	16.25
21233721	2011	6	6	5310	205.08	16.25
21233721	2011	6	6	5320	204.38	16.25
21233721	2011	6	6	5330	203.76	16.25
21233721	2011	6	6	5340	203.48	16.25
21233721	2011	6	6	5350	202.49	16.24
21233721	2011	6	6	5360	201.1	16.24
21233721	2011	6	6	5370	199.53	16.24
21233721	2011	6	6	5380	199.51	16.25
21233721	2011	6	6	5390	199.83	16.26
21233721	2011	6	6	5400	200.33	16.27
21233721	2011	6	6	5410	200.3	16.28
21233721	2011	6	6	5420	200.23	16.28
21233721	2011	6	6	5430	200.52	16.29
21233721	2011	6	6	5440	200.43	16.29
21233721	2011	6	6	5450	200.3	16.31
21233721	2011	6	6	5460	200.15	16.32
21233721	2011	6	6	5470	199.86	16.33
21233721	2011	6	6	5480	199.58	16.34
21233721	2011	6	6	5490	199.29	16.34
21233721	2011	6	6	5500	199.02	16.35
21233721	2011	6	6	5510	199.36	16.37
21233721	2011	6	6	5520	199.69	16.39
21233721	2011	6	6	5530	199.61	16.39
21233721	2011	6	6	5540	199.8	16.4
21233721	2011	6	6	5550	200.05	16.42
21233721	2011	6	6	5560	200.1	16.42
21233721	2011	6	6	5570	200.08	16.44
21233721	2011	6	6	5580	199.79	16.44

21233721	2011	6	6	5590	198.97	16.46
21233721	2011	6	6	5600	197.34	16.46
21233721	2011	6	6	5610	195.77	16.47
21233721	2011	6	6	5620	194.97	16.49
21233721	2011	6	6	5630	194.58	16.5
21233721	2011	6	6	5640	193.18	16.53
21233721	2011	6	6	5650	192.22	16.54
21233721	2011	6	6	5660	192.53	16.58
21233721	2011	6	6	5670	192.6	16.6
21233721	2011	6	6	5680	192.68	16.64
21233721	2011	6	6	5690	192.57	16.64
21233721	2011	6	6	5700	192.37	16.65
21233721	2011	6	6	5710	192.44	16.67
21233721	2011	6	6	5720	192.49	16.68
21233721	2011	6	6	5730	192.61	16.7
21233721	2011	6	6	5740	192.59	16.71
21233721	2011	6	6	5750	192.69	16.72
21233721	2011	6	6	5760	192.46	16.74
21233721	2011	6	6	5770	192.44	16.75
21233721	2011	6	6	5780	192.66	16.77
21233721	2011	6	6	5790	192.71	16.78
21233721	2011	6	6	5800	192.46	16.79
21233721	2011	6	6	5810	192.3	16.8
21233721	2011	6	6	5820	192.48	16.82
21233721	2011	6	6	5830	192.6	16.82
21233721	2011	6	6	5840	192.73	16.83
21233721	2011	6	6	5850	192.65	16.84
21233721	2011	6	6	5860	192.9	16.85
21233721	2011	6	6	5870	192.79	16.85
21233721	2011	6	6	5880	192.62	16.86
21233721	2011	6	6	5890	192.73	16.87
21233721	2011	6	6	5900	192.84	16.88
21233721	2011	6	6	5910	192.83	16.89
21233721	2011	6	6	5920	192.71	16.89
21233721	2011	6	6	5930	192.59	16.89
21233721	2011	6	6	5940	192.33	16.89
21233721	2011	6	6	5950	192.75	16.92
21233721	2011	6	6	5960	192.03	16.92
21233721	2011	6	6	5970	192.81	16.92
21233721	2011	6	6	5980	193.17	16.96
21233721	2011	6	6	5990	193.09	16.96
21233721	2011	6	6	6000	192.93	16.97
21233721	2011	6	6	6010	192.77	16.98
21233721	2011	6	6	6020	192.97	16.98
21233721	2011	6	6	6030	192.98	17
21233721	2011	6	6	6040	192.82	17
21233721	2011	6	6	6050	192.76	17

21233721	2011	6	6	6060	192.97	17.01
21233721	2011	6	6	6070	193.14	17.02
21233721	2011	6	6	6080	192.89	17.02
21233721	2011	6	6	6090	192.75	17.03
21233721	2011	6	6	6100	192.45	17.03
21233721	2011	6	6	6110	191.91	17.03
21233721	2011	6	6	6120	191.35	17.04
21233721	2011	6	6	6130	190.77	17.04
21233721	2011	6	6	6140	190.18	17.04
21233721	2011	6	6	6150	189.4	17.05
21233721	2011	6	6	6160	188.65	17.05
21233721	2011	6	6	6170	188	17.06
21233721	2011	6	6	6180	187.42	17.07
21233721	2011	6	6	6190	186.85	17.09
21233721	2011	6	6	6200	185.62	17.1
21233721	2011	6	6	6210	185.39	17.14
21233721	2011	6	6	6220	184.75	17.15
21233721	2011	6	6	6230	183.18	17.17
21233721	2011	6	6	6240	181.89	17.19
21233721	2011	6	6	6250	180.39	17.21
21233721	2011	6	6	6260	178.11	17.23
21233721	2011	6	6	6270	175.74	17.25
21233721	2011	6	6	6280	173.6	17.27
21233721	2011	6	6	6290	172.14	17.3
21233721	2011	6	6	6300	170.27	17.32
21233721	2011	6	6	6310	168.5	17.35
21233721	2011	6	6	6320	167.37	17.38
21233721	2011	6	6	6330	166.71	17.42
21233721	2011	6	6	6340	166	17.44
21233721	2011	6	6	6350	164.3	17.46
21233721	2011	6	6	6360	162.38	17.48
21233721	2011	6	6	6370	159.87	17.51
21233721	2011	6	6	6380	157.44	17.54
21233721	2011	6	6	6390	155.17	17.61
21233721	2011	6	6	6400	153.16	17.71
21233721	2011	6	6	6410	150.65	17.75
21233721	2011	6	6	6420	148.26	17.85
21233721	2011	6	6	6430	145.87	17.94
21233721	2011	6	6	6440	143.29	18.05
21233721	2011	6	6	6450	140.54	18.14
21233721	2011	6	6	6460	137.87	18.25
21233721	2011	6	6	6470	134.97	18.44
21233721	2011	6	6	6480	132.74	18.6
21233721	2011	6	6	6490	130.36	18.72
21233721	2011	6	6	6500	127.76	18.82
21233721	2011	6	6	6510	125.58	18.96
21233721	2011	6	6	6520	123.26	19.12

21233721	2011	6	6	6530	120.93	19.25
21233721	2011	6	6	6540	118.91	19.44
21233721	2011	6	6	6550	116.5	19.58
21233721	2011	6	6	6560	114.09	19.74
21233721	2011	6	6	6570	111.26	19.87
21233721	2011	6	6	6580	108.26	20.04
21233721	2011	6	6	6590	105.25	20.18
21233721	2011	6	6	6600	102.83	20.27
21233721	2011	6	6	6610	101.1	20.41
21233721	2011	6	6	6620	98.75	20.51
21233721	2011	6	6	6630	96.23	20.64
21233721	2011	6	6	6640	93.45	20.8
21233721	2011	6	6	6650	90.67	21.02
21233721	2011	6	6	6660	87.53	21.19
21233721	2011	6	6	6670	84.39	21.35
21233721	2011	6	6	6680	81.28	21.54
21233721	2011	6	6	6690	78.27	21.67
21233721	2011	6	6	6700	75.47	21.81
21233721	2011	6	6	6710	72.58	22.06
21233721	2011	6	6	6720	70.47	22.2
21233721	2011	6	6	6730	69.1	22.36
21233721	2011	6	6	6740	68.08	22.52
21233721	2011	6	6	6750	66.59	22.69
21233721	2011	6	6	6760	64.19	22.89
21233721	2011	6	6	6770	61.58	23.07
21233721	2011	6	6	6780	58.74	23.28
21233721	2011	6	6	6790	55.79	23.44
21233721	2011	6	6	6800	52.59	23.55
21233721	2011	6	6	6810	49.43	23.71
21233721	2011	6	6	6820	46.36	23.85
21233721	2011	6	6	6830	43.09	23.97
21233721	2011	6	6	6840	40.67	24.1
21233721	2011	6	6	6850	37.84	24.21
21233721	2011	6	6	6860	35.03	24.44
21233721	2011	6	6	6870	31.98	24.66
21233721	2011	6	6	6880	28.89	24.84
21233721	2011	6	6	6890	26.32	24.99
21233721	2011	6	6	6900	24.12	25.16
21233721	2011	6	6	6910	20.85	25.37
21233721	2011	6	6	6920	17.94	25.54
21233721	2011	6	6	6930	16.67	25.72
21233721	2011	6	6	6940	15.38	25.91
21233721	2011	6	6	6950	13.3	26.03
21233721	2011	6	6	6960	10.49	26.24
21233721	2011	6	6	6970	7.86	26.38
21233721	2011	6	6	6980	5.29	26.54
21233721	2011	6	6	6990	3.69	26.67

21233721	2011	6	6	7000	2.82	26.78
21233721	2011	6	6	7010	2.25	26.86
21233721	2011	6	6	7020	1.75	26.96
21233721	2011	6	6	7030	1.35	27.05
21233721	2011	6	6	7040	0.72	27.16
21233721	2011	6	6	7050	0.63	27.26
21233721	2011	6	6	7060	0.65	27.37
21233721	2011	6	6	7070	0.69	27.53
21233721	2011	6	6	7080	0.62	27.64
21233721	2011	6	6	7090	0.61	27.69
21233721	2011	6	6	7100	0.69	27.77
21233721	2011	6	6	7110	0.61	27.82
21233721	2011	6	6	7120	0.5	27.92
21233721	2011	6	6	7130	0.52	27.97
21233721	2011	6	6	7140	0.51	28.01
21233721	2011	6	6	7150	0.51	28.06
21233721	2011	6	6	7160	0.52	28.1
21233721	2011	6	6	7170	0.52	28.15
21233721	2011	6	6	7180	0.51	28.18
21243681	2011	6	6	0	1.33	29.25
21243681	2011	6	6	10	2.25	29.24
21243681	2011	6	6	20	2.75	29.25
21243681	2011	6	6	30	2.4	29.24
21243681	2011	6	6	40	2.5	29.25
21243681	2011	6	6	50	2.45	29.24
21243681	2011	6	6	60	2.41	29.23
21243681	2011	6	6	70	2.72	29.22
21243681	2011	6	6	80	3.06	29.21
21243681	2011	6	6	90	4.48	29.16
21243681	2011	6	6	100	6.25	29.11
21243681	2011	6	6	110	7.99	29.07
21243681	2011	6	6	120	9.05	29.05
21243681	2011	6	6	130	9.73	29.04
21243681	2011	6	6	140	10.65	29.01
21243681	2011	6	6	150	11.72	28.97
21243681	2011	6	6	160	12.37	28.96
21243681	2011	6	6	170	12.54	28.94
21243681	2011	6	6	180	12.99	28.94
21243681	2011	6	6	190	14.04	28.92
21243681	2011	6	6	200	14.75	28.9
21243681	2011	6	6	210	15.14	28.89
21243681	2011	6	6	220	15.64	28.88
21243681	2011	6	6	230	15.64	28.85
21243681	2011	6	6	240	16.4	28.84
21243681	2011	6	6	250	17.89	28.79
21243681	2011	6	6	260	19	28.78
21243681	2011	6	6	270	20.72	28.74

21243681	2011	6	6	280	23.01	28.68
21243681	2011	6	6	290	24.74	28.67
21243681	2011	6	6	300	26.32	28.67
21243681	2011	6	6	310	28.78	28.63
21243681	2011	6	6	320	31.47	28.57
21243681	2011	6	6	330	33.83	28.54
21243681	2011	6	6	340	36.24	28.52
21243681	2011	6	6	350	38.7	28.49
21243681	2011	6	6	360	41.15	28.46
21243681	2011	6	6	370	43.66	28.44
21243681	2011	6	6	380	46.89	28.4
21243681	2011	6	6	390	50.49	28.35
21243681	2011	6	6	400	53.95	28.21
21243681	2011	6	6	410	56.82	27.99
21243681	2011	6	6	420	59.25	27.75
21243681	2011	6	6	430	61.5	27.53
21243681	2011	6	6	440	63.65	27.39
21243681	2011	6	6	450	65.96	27.31
21243681	2011	6	6	460	68.16	27.21
21243681	2011	6	6	470	70.28	27.07
21243681	2011	6	6	480	72.3	26.93
21243681	2011	6	6	490	74.28	26.78
21243681	2011	6	6	500	76.34	26.65
21243681	2011	6	6	510	78.54	26.61
21243681	2011	6	6	520	80.73	26.5
21243681	2011	6	6	530	83.34	26.39
21243681	2011	6	6	540	86.34	26.27
21243681	2011	6	6	550	88.69	26.17
21243681	2011	6	6	560	91.94	26.02
21243681	2011	6	6	570	94.98	25.92
21243681	2011	6	6	580	97.97	25.71
21243681	2011	6	6	590	101.56	25.66
21243681	2011	6	6	600	105.37	25.5
21243681	2011	6	6	610	109.17	25.32
21243681	2011	6	6	620	112.89	25.18
21243681	2011	6	6	630	116.57	24.97
21243681	2011	6	6	640	120.24	24.82
21243681	2011	6	6	650	123.61	24.77
21243681	2011	6	6	660	126.7	24.69
21243681	2011	6	6	670	129.49	24.57
21243681	2011	6	6	680	131.81	24.35
21243681	2011	6	6	690	133.41	24.21
21243681	2011	6	6	700	133.86	24
21243681	2011	6	6	710	134.6	23.8
21243681	2011	6	6	720	136.64	23.71
21243681	2011	6	6	730	138.66	23.58
21243681	2011	6	6	740	140.85	23.51

21243681	2011	6	6	750	142.74	23.32
21243681	2011	6	6	760	143.76	23.28
21243681	2011	6	6	770	144.39	23.14
21243681	2011	6	6	780	144.7	22.99
21243681	2011	6	6	790	144.97	22.85
21243681	2011	6	6	800	145.23	22.78
21243681	2011	6	6	810	145.67	22.64
21243681	2011	6	6	820	147.52	22.46
21243681	2011	6	6	830	149.01	22.41
21243681	2011	6	6	840	151.07	22.34
21243681	2011	6	6	850	153.07	22.17
21243681	2011	6	6	860	155.01	22.01
21243681	2011	6	6	870	157.21	21.88
21243681	2011	6	6	880	159.45	21.67
21243681	2011	6	6	890	161.55	21.5
21243681	2011	6	6	900	162.8	21.39
21243681	2011	6	6	910	163.92	21.32
21243681	2011	6	6	920	164.71	21.21
21243681	2011	6	6	930	164.78	21.12
21243681	2011	6	6	940	164.88	21.04
21243681	2011	6	6	950	165.23	20.98
21243681	2011	6	6	960	165.6	20.89
21243681	2011	6	6	970	165.82	20.77
21243681	2011	6	6	980	165.82	20.69
21243681	2011	6	6	990	166.02	20.59
21243681	2011	6	6	1000	165.87	20.5
21243681	2011	6	6	1010	165.38	20.41
21243681	2011	6	6	1020	165.24	20.32
21243681	2011	6	6	1030	165.37	20.25
21243681	2011	6	6	1040	165.12	20.15
21243681	2011	6	6	1050	164.7	20.09
21243681	2011	6	6	1060	164.72	20
21243681	2011	6	6	1070	164.48	19.92
21243681	2011	6	6	1080	164.17	19.85
21243681	2011	6	6	1090	163.96	19.78
21243681	2011	6	6	1100	164.15	19.73
21243681	2011	6	6	1110	164.46	19.65
21243681	2011	6	6	1120	164.72	19.58
21243681	2011	6	6	1130	164.96	19.53
21243681	2011	6	6	1140	165.11	19.48
21243681	2011	6	6	1150	165.19	19.44
21243681	2011	6	6	1160	165.39	19.39
21243681	2011	6	6	1170	165.17	19.36
21243681	2011	6	6	1180	164.89	19.33
21243681	2011	6	6	1190	165.03	19.29
21243681	2011	6	6	1200	164.93	19.25
21243681	2011	6	6	1210	165.07	19.22

21243681	2011	6	6	1220	165.16	19.2
21243681	2011	6	6	1230	165.3	19.17
21243681	2011	6	6	1240	165.33	19.14
21243681	2011	6	6	1250	165.41	19.12
21243681	2011	6	6	1260	165.41	19.1
21243681	2011	6	6	1270	165.43	19.06
21243681	2011	6	6	1280	165.39	19.04
21243681	2011	6	6	1290	165.21	19.01
21243681	2011	6	6	1300	165.06	18.96
21243681	2011	6	6	1310	164.87	18.96
21243681	2011	6	6	1320	164.44	18.95
21243681	2011	6	6	1330	163.71	18.93
21243681	2011	6	6	1340	163.48	18.92
21243681	2011	6	6	1350	163.46	18.92
21243681	2011	6	6	1360	163.46	18.92
21243681	2011	6	6	1370	163.17	18.91
21243681	2011	6	6	1380	162.92	18.91
21243681	2011	6	6	1390	163.45	18.92
21243681	2011	6	6	1400	163.75	18.92
21243681	2011	6	6	1410	163.82	18.92
21243681	2011	6	6	1420	163.85	18.91
21243681	2011	6	6	1430	163.93	18.92
21243681	2011	6	6	1440	164	18.92
21243681	2011	6	6	1450	163.99	18.9
21243681	2011	6	6	1460	164.06	18.9
21243681	2011	6	6	1470	164.07	18.9
21243681	2011	6	6	1480	164.23	18.9
21243681	2011	6	6	1490	164.35	18.91
21243681	2011	6	6	1500	164.37	18.9
21243681	2011	6	6	1510	164.32	18.9
21243681	2011	6	6	1520	164.31	18.9
21243681	2011	6	6	1530	164.32	18.9
21243681	2011	6	6	1540	164.22	18.91
21243681	2011	6	6	1550	164.18	18.9
21243681	2011	6	6	1560	164.18	18.9
21243681	2011	6	6	1570	164.18	18.9
21243681	2011	6	6	1580	164.24	18.9
21243681	2011	6	6	1590	164.32	18.89
21243681	2011	6	6	1600	164.28	18.89
21243681	2011	6	6	1610	164.37	18.89
21243681	2011	6	6	1620	164.4	18.89
21243681	2011	6	6	1630	164.41	18.89
21243681	2011	6	6	1640	164.33	18.89
21243681	2011	6	6	1650	164.14	18.9
21243681	2011	6	6	1660	164.12	18.9
21243681	2011	6	6	1670	164.27	18.89
21243681	2011	6	6	1680	164.27	18.89

21243681	2011	6	6	1690	164.24	18.9
21243681	2011	6	6	1700	164.14	18.9
21243681	2011	6	6	1710	163.74	18.9
21243681	2011	6	6	1720	163.15	18.9
21243681	2011	6	6	1730	162.61	18.91
21243681	2011	6	6	1740	161.95	18.92
21243681	2011	6	6	1750	161.55	18.91
21243681	2011	6	6	1760	161.29	18.91
21243681	2011	6	6	1770	161.04	18.92
21243681	2011	6	6	1780	160.62	18.92
21243681	2011	6	6	1790	160.19	18.92
21243681	2011	6	6	1800	159.95	18.92
21243681	2011	6	6	1810	159.81	18.92
21243681	2011	6	6	1820	159.66	18.93
21243681	2011	6	6	1830	159.71	18.94
21243681	2011	6	6	1840	159.95	18.94
21243681	2011	6	6	1850	160.43	18.95
21243681	2011	6	6	1860	161.37	18.95
21243681	2011	6	6	1870	161.84	18.95
21243681	2011	6	6	1880	162.56	18.95
21243681	2011	6	6	1890	162.64	18.94
21243681	2011	6	6	1900	162.79	18.93
21243681	2011	6	6	1910	163.05	18.92
21243681	2011	6	6	1920	163.12	18.93
21243681	2011	6	6	1930	163.31	18.92
21243681	2011	6	6	1940	163.7	18.92
21243681	2011	6	6	1950	163.82	18.92
21243681	2011	6	6	1960	164.18	18.92
21243681	2011	6	6	1970	164.72	18.91
21243681	2011	6	6	1980	164.75	18.9
21243681	2011	6	6	1990	164.7	18.9
21243681	2011	6	6	2000	164.56	18.9
21243681	2011	6	6	2010	164.32	18.9
21243681	2011	6	6	2020	164.03	18.89
21243681	2011	6	6	2030	164.01	18.89
21243681	2011	6	6	2040	164.21	18.89
21243681	2011	6	6	2050	164.33	18.89
21243681	2011	6	6	2060	164.5	18.89
21243681	2011	6	6	2070	164.84	18.89
21243681	2011	6	6	2080	164.76	18.88
21243681	2011	6	6	2090	164.66	18.89
21243681	2011	6	6	2100	164.57	18.88
21243681	2011	6	6	2110	164.7	18.88
21243681	2011	6	6	2120	164.66	18.88
21243681	2011	6	6	2130	164.52	18.88
21243681	2011	6	6	2140	164.37	18.88
21243681	2011	6	6	2150	164.34	18.88

21243681	2011	6	6	2160	164.24	18.87
21243681	2011	6	6	2170	163.97	18.85
21243681	2011	6	6	2180	163.58	18.85
21243681	2011	6	6	2190	163.43	18.85
21243681	2011	6	6	2200	163.34	18.84
21243681	2011	6	6	2210	163.38	18.85
21243681	2011	6	6	2220	163.4	18.84
21243681	2011	6	6	2230	163.53	18.85
21243681	2011	6	6	2240	163.61	18.85
21243681	2011	6	6	2250	163.64	18.85
21243681	2011	6	6	2260	163.64	18.85
21243681	2011	6	6	2270	163.71	18.84
21243681	2011	6	6	2280	164.02	18.84
21243681	2011	6	6	2290	164.02	18.84
21243681	2011	6	6	2300	164.09	18.84
21243681	2011	6	6	2310	163.86	18.84
21243681	2011	6	6	2320	162.89	18.84
21243681	2011	6	6	2330	162.08	18.85
21243681	2011	6	6	2340	161.65	18.85
21243681	2011	6	6	2350	161.78	18.84
21243681	2011	6	6	2360	161.96	18.84
21243681	2011	6	6	2370	162.02	18.85
21243681	2011	6	6	2380	161.91	18.84
21243681	2011	6	6	2390	161.85	18.84
21243681	2011	6	6	2400	162.17	18.85
21243681	2011	6	6	2410	162.2	18.85
21243681	2011	6	6	2420	161.96	18.84
21243681	2011	6	6	2430	161.67	18.84
21243681	2011	6	6	2440	161.33	18.84
21243681	2011	6	6	2450	161	18.85
21243681	2011	6	6	2460	160.76	18.85
21243681	2011	6	6	2470	160.63	18.84
21243681	2011	6	6	2480	160.35	18.85
21243681	2011	6	6	2490	160.4	18.85
21243681	2011	6	6	2500	160.64	18.85
21243681	2011	6	6	2510	160.6	18.85
21243681	2011	6	6	2520	160.59	18.85
21243681	2011	6	6	2530	160.76	18.85
21243681	2011	6	6	2540	161.51	18.85
21243681	2011	6	6	2550	161.59	18.85
21243681	2011	6	6	2560	161.46	18.85
21243681	2011	6	6	2570	161.29	18.85
21243681	2011	6	6	2580	161.07	18.85
21243681	2011	6	6	2590	160.75	18.85
21243681	2011	6	6	2600	160.7	18.85
21243681	2011	6	6	2610	160.97	18.85
21243681	2011	6	6	2620	161.07	18.85

21243681	2011	6	6	2630	160.89	18.85
21243681	2011	6	6	2640	160.74	18.85
21243681	2011	6	6	2650	160.88	18.86
21243681	2011	6	6	2660	161.1	18.86
21243681	2011	6	6	2670	161.18	18.86
21243681	2011	6	6	2680	161.46	18.86
21243681	2011	6	6	2690	161.69	18.85
21243681	2011	6	6	2700	161.49	18.87
21243681	2011	6	6	2710	161.22	18.86
21243681	2011	6	6	2720	161.33	18.87
21243681	2011	6	6	2730	161.35	18.86
21243681	2011	6	6	2740	161.27	18.86
21243681	2011	6	6	2750	161.42	18.86
21243681	2011	6	6	2760	161.43	18.86
21243681	2011	6	6	2770	161.35	18.85
21243681	2011	6	6	2780	161.15	18.86
21243681	2011	6	6	2790	160.84	18.86
21243681	2011	6	6	2800	160.69	18.86
21243681	2011	6	6	2810	160.54	18.85
21243681	2011	6	6	2820	160.5	18.86
21243681	2011	6	6	2830	160.6	18.87
21243681	2011	6	6	2840	160.67	18.86
21243681	2011	6	6	2850	160.96	18.87
21243681	2011	6	6	2860	161.11	18.87
21243681	2011	6	6	2870	161.02	18.87
21243681	2011	6	6	2880	160.84	18.88
21243681	2011	6	6	2890	160.62	18.87
21243681	2011	6	6	2900	160.93	18.87
21243681	2011	6	6	2910	161.24	18.88
21243681	2011	6	6	2920	161.16	18.88
21243681	2011	6	6	2930	161	18.87
21243681	2011	6	6	2940	160.86	18.88
21243681	2011	6	6	2950	161.06	18.88
21243681	2011	6	6	2960	161.12	18.89
21243681	2011	6	6	2970	161.06	18.88
21243681	2011	6	6	2980	160.92	18.88
21243681	2011	6	6	2990	160.92	18.88
21243681	2011	6	6	3000	160.89	18.89
21243681	2011	6	6	3010	160.61	18.89
21243681	2011	6	6	3020	160.84	18.89
21243681	2011	6	6	3030	161.28	18.89
21243681	2011	6	6	3040	161.5	18.89
21243681	2011	6	6	3050	161.53	18.89
21243681	2011	6	6	3060	161.17	18.89
21243681	2011	6	6	3070	160.62	18.89
21243681	2011	6	6	3080	160.24	18.89
21243681	2011	6	6	3090	160.02	18.89

21243681	2011	6	6	3100	160.05	18.89
21243681	2011	6	6	3110	160.51	18.89
21243681	2011	6	6	3120	160.77	18.89
21243681	2011	6	6	3130	160.93	18.89
21243681	2011	6	6	3140	161.29	18.9
21243681	2011	6	6	3150	161.8	18.9
21243681	2011	6	6	3160	161.83	18.9
21243681	2011	6	6	3170	161.64	18.91
21243681	2011	6	6	3180	161.47	18.91
21243681	2011	6	6	3190	161.24	18.92
21243681	2011	6	6	3200	161.07	18.92
21243681	2011	6	6	3210	161.27	18.92
21243681	2011	6	6	3220	161.94	18.91
21243681	2011	6	6	3230	162.24	18.91
21243681	2011	6	6	3240	162.38	18.91
21243681	2011	6	6	3250	162.45	18.91
21243681	2011	6	6	3260	162.68	18.91
21243681	2011	6	6	3270	162.39	18.91
21243681	2011	6	6	3280	162.08	18.9
21243681	2011	6	6	3290	161.92	18.9
21243681	2011	6	6	3300	162.03	18.9
21243681	2011	6	6	3310	161.92	18.9
21243681	2011	6	6	3320	161.72	18.9
21243681	2011	6	6	3330	161.57	18.91
21243681	2011	6	6	3340	161.15	18.9
21243681	2011	6	6	3350	160.23	18.9
21243681	2011	6	6	3360	159.16	18.9
21243681	2011	6	6	3370	159.2	18.9
21243681	2011	6	6	3380	159.26	18.92
21243681	2011	6	6	3390	159.52	18.91
21243681	2011	6	6	3400	159.42	18.91
21243681	2011	6	6	3410	159.33	18.92
21243681	2011	6	6	3420	159.29	18.92
21243681	2011	6	6	3430	159.21	18.93
21243681	2011	6	6	3440	158.95	18.93
21243681	2011	6	6	3450	158.39	18.95
21243681	2011	6	6	3460	158.05	18.94
21243681	2011	6	6	3470	158.01	18.95
21243681	2011	6	6	3480	158.25	18.95
21243681	2011	6	6	3490	158.3	18.96
21243681	2011	6	6	3500	158.29	18.96
21243681	2011	6	6	3510	158.13	18.97
21243681	2011	6	6	3520	157.92	19.01
21243681	2011	6	6	3530	157.82	19.01
21243681	2011	6	6	3540	157.86	19.03
21243681	2011	6	6	3550	158.06	19.03
21243681	2011	6	6	3560	157.88	19.05

21243681	2011	6	6	3570	157.61	19.06
21243681	2011	6	6	3580	157.35	19.07
21243681	2011	6	6	3590	156.87	19.07
21243681	2011	6	6	3600	155.9	19.08
21243681	2011	6	6	3610	155.14	19.08
21243681	2011	6	6	3620	153.7	19.1
21243681	2011	6	6	3630	151.98	19.12
21243681	2011	6	6	3640	151.07	19.14
21243681	2011	6	6	3650	150.55	19.16
21243681	2011	6	6	3660	150.52	19.19
21243681	2011	6	6	3670	150.51	19.23
21243681	2011	6	6	3680	150.53	19.27
21243681	2011	6	6	3690	150.79	19.29
21243681	2011	6	6	3700	150.96	19.32
21243681	2011	6	6	3710	150.96	19.35
21243681	2011	6	6	3720	151.04	19.4
21243681	2011	6	6	3730	150.94	19.45
21243681	2011	6	6	3740	150.72	19.5
21243681	2011	6	6	3750	150.49	19.55
21243681	2011	6	6	3760	150.31	19.6
21243681	2011	6	6	3770	150.32	19.64
21243681	2011	6	6	3780	150.68	19.68
21243681	2011	6	6	3790	151.31	19.74
21243681	2011	6	6	3800	151.08	19.78
21243681	2011	6	6	3810	150.58	19.83
21243681	2011	6	6	3820	150.04	19.87
21243681	2011	6	6	3830	148.74	19.9
21243681	2011	6	6	3840	147.86	19.95
21243681	2011	6	6	3850	147.99	20
21243681	2011	6	6	3860	148.1	20.05
21243681	2011	6	6	3870	148.19	20.07
21243681	2011	6	6	3880	148.4	20.09
21243681	2011	6	6	3890	148.77	20.1
21243681	2011	6	6	3900	149.06	20.13
21243681	2011	6	6	3910	149.21	20.14
21243681	2011	6	6	3920	149.27	20.17
21243681	2011	6	6	3930	150.23	20.21
21243681	2011	6	6	3940	150.59	20.24
21243681	2011	6	6	3950	150.5	20.26
21243681	2011	6	6	3960	150.24	20.28
21243681	2011	6	6	3970	150.09	20.29
21243681	2011	6	6	3980	150.19	20.3
21243681	2011	6	6	3990	150.22	20.31
21243681	2011	6	6	4000	150.29	20.32
21243681	2011	6	6	4010	150.31	20.32
21243681	2011	6	6	4020	150.42	20.33
21243681	2011	6	6	4030	150.92	20.34

21243681	2011	6	6	4040	150.85	20.35
21243681	2011	6	6	4050	150.68	20.35
21243681	2011	6	6	4060	150.5	20.36
21243681	2011	6	6	4070	150.34	20.38
21243681	2011	6	6	4080	150.2	20.38
21243681	2011	6	6	4090	150.26	20.39
21243681	2011	6	6	4100	150.17	20.4
21243681	2011	6	6	4110	149.86	20.4
21243681	2011	6	6	4120	149.62	20.42
21243681	2011	6	6	4130	149.32	20.42
21243681	2011	6	6	4140	149.19	20.42
21243681	2011	6	6	4150	148.99	20.43
21243681	2011	6	6	4160	148.78	20.44
21243681	2011	6	6	4170	148.81	20.45
21243681	2011	6	6	4180	149.16	20.46
21243681	2011	6	6	4190	149.13	20.46
21243681	2011	6	6	4200	148.45	20.48
21243681	2011	6	6	4210	148.33	20.49
21243681	2011	6	6	4220	148.69	20.5
21243681	2011	6	6	4230	149.14	20.51
21243681	2011	6	6	4240	149.48	20.52
21243681	2011	6	6	4250	149.84	20.52
21243681	2011	6	6	4260	149.99	20.52
21243681	2011	6	6	4270	150.19	20.53
21243681	2011	6	6	4280	149.73	20.54
21243681	2011	6	6	4290	149.34	20.56
21243681	2011	6	6	4300	149.66	20.57
21243681	2011	6	6	4310	149.46	20.57
21243681	2011	6	6	4320	149.46	20.58
21243681	2011	6	6	4330	149.16	20.58
21243681	2011	6	6	4340	149.2	20.59
21243681	2011	6	6	4350	149.15	20.6
21243681	2011	6	6	4360	148	20.61
21243681	2011	6	6	4370	147.33	20.61
21243681	2011	6	6	4380	147.19	20.62
21243681	2011	6	6	4390	147.09	20.61
21243681	2011	6	6	4400	146.59	20.63
21243681	2011	6	6	4410	146.37	20.63
21243681	2011	6	6	4420	146.05	20.64
21243681	2011	6	6	4430	145.59	20.64
21243681	2011	6	6	4440	145.32	20.64
21243681	2011	6	6	4450	145.11	20.66
21243681	2011	6	6	4460	145.11	20.67
21243681	2011	6	6	4470	145.15	20.67
21243681	2011	6	6	4480	145.18	20.67
21243681	2011	6	6	4490	145.12	20.69
21243681	2011	6	6	4500	144.98	20.7

21243681	2011	6	6	4510	144.85	20.71
21243681	2011	6	6	4520	144.65	20.71
21243681	2011	6	6	4530	144.6	20.72
21243681	2011	6	6	4540	144.66	20.72
21243681	2011	6	6	4550	145.17	20.74
21243681	2011	6	6	4560	145.55	20.74
21243681	2011	6	6	4570	145.83	20.75
21243681	2011	6	6	4580	145.97	20.75
21243681	2011	6	6	4590	145.96	20.75
21243681	2011	6	6	4600	146.94	20.76
21243681	2011	6	6	4610	147.59	20.76
21243681	2011	6	6	4620	147.81	20.75
21243681	2011	6	6	4630	147.94	20.75
21243681	2011	6	6	4640	148.01	20.75
21243681	2011	6	6	4650	147.91	20.75
21243681	2011	6	6	4660	147.6	20.76
21243681	2011	6	6	4670	147.44	20.76
21243681	2011	6	6	4680	147.59	20.76
21243681	2011	6	6	4690	147.53	20.77
21243681	2011	6	6	4700	147.46	20.77
21243681	2011	6	6	4710	147.2	20.77
21243681	2011	6	6	4720	146.7	20.76
21243681	2011	6	6	4730	146.15	20.77
21243681	2011	6	6	4740	145.79	20.77
21243681	2011	6	6	4750	145.33	20.76
21243681	2011	6	6	4760	145.02	20.77
21243681	2011	6	6	4770	145.09	20.78
21243681	2011	6	6	4780	145	20.78
21243681	2011	6	6	4790	144.82	20.78
21243681	2011	6	6	4800	144.93	20.78
21243681	2011	6	6	4810	145.34	20.79
21243681	2011	6	6	4820	145.49	20.8
21243681	2011	6	6	4830	145.27	20.8
21243681	2011	6	6	4840	144.97	20.81
21243681	2011	6	6	4850	144.72	20.81
21243681	2011	6	6	4860	144.7	20.82
21243681	2011	6	6	4870	144.59	20.82
21243681	2011	6	6	4880	145.14	20.82
21243681	2011	6	6	4890	145.41	20.82
21243681	2011	6	6	4900	145.32	20.83
21243681	2011	6	6	4910	144.94	20.83
21243681	2011	6	6	4920	144.67	20.84
21243681	2011	6	6	4930	145.07	20.84
21243681	2011	6	6	4940	144.81	20.85
21243681	2011	6	6	4950	144.65	20.85
21243681	2011	6	6	4960	144.57	20.85
21243681	2011	6	6	4970	144.74	20.85

21243681	2011	6	6	4980	144.82	20.86
21243681	2011	6	6	4990	144.88	20.86
21243681	2011	6	6	5000	145.32	20.86
21243681	2011	6	6	5010	145.94	20.86
21243681	2011	6	6	5020	146.19	20.87
21243681	2011	6	6	5030	146.11	20.88
21243681	2011	6	6	5040	146.15	20.88
21243681	2011	6	6	5050	146.33	20.88
21243681	2011	6	6	5060	146.17	20.89
21243681	2011	6	6	5070	146.28	20.88
21243681	2011	6	6	5080	146.34	20.89
21243681	2011	6	6	5090	146.3	20.89
21243681	2011	6	6	5100	146.14	20.89
21243681	2011	6	6	5110	146.16	20.88
21243681	2011	6	6	5120	146.18	20.87
21243681	2011	6	6	5130	146.07	20.88
21243681	2011	6	6	5140	145.44	20.88
21243681	2011	6	6	5150	145.3	20.87
21243681	2011	6	6	5160	145.16	20.87
21243681	2011	6	6	5170	145.09	20.87
21243681	2011	6	6	5180	145.23	20.87
21243681	2011	6	6	5190	144.76	20.88
21243681	2011	6	6	5200	144.16	20.88
21243681	2011	6	6	5210	143.29	20.89
21243681	2011	6	6	5220	141.51	20.89
21243681	2011	6	6	5230	139.83	20.89
21243681	2011	6	6	5240	138.29	20.89
21243681	2011	6	6	5250	135.83	20.92
21243681	2011	6	6	5260	134.24	20.92
21243681	2011	6	6	5270	131.72	20.95
21243681	2011	6	6	5280	128.88	20.99
21243681	2011	6	6	5290	126.09	21.02
21243681	2011	6	6	5300	123.39	21.06
21243681	2011	6	6	5310	120.76	21.13
21243681	2011	6	6	5320	117.94	21.19
21243681	2011	6	6	5330	115.25	21.28
21243681	2011	6	6	5340	112.53	21.35
21243681	2011	6	6	5350	109.88	21.43
21243681	2011	6	6	5360	107.04	21.53
21243681	2011	6	6	5370	104.28	21.63
21243681	2011	6	6	5380	101.38	21.71
21243681	2011	6	6	5390	98.71	21.82
21243681	2011	6	6	5400	95.86	21.95
21243681	2011	6	6	5410	93.11	22.1
21243681	2011	6	6	5420	90.27	22.24
21243681	2011	6	6	5430	87.42	22.39
21243681	2011	6	6	5440	84.49	22.56

21243681	2011	6	6	5450	81.52	22.71
21243681	2011	6	6	5460	78.59	22.85
21243681	2011	6	6	5470	75.69	23
21243681	2011	6	6	5480	72.73	23.1
21243681	2011	6	6	5490	69.8	23.28
21243681	2011	6	6	5500	66.76	23.43
21243681	2011	6	6	5510	63.99	23.59
21243681	2011	6	6	5520	61.38	23.7
21243681	2011	6	6	5530	58.81	23.8
21243681	2011	6	6	5540	56.24	23.91
21243681	2011	6	6	5550	53.66	24
21243681	2011	6	6	5560	51.06	24.17
21243681	2011	6	6	5570	48.52	24.27
21243681	2011	6	6	5580	46.2	24.37
21243681	2011	6	6	5590	43.58	24.46
21243681	2011	6	6	5600	41.13	24.56
21243681	2011	6	6	5610	38.62	24.68
21243681	2011	6	6	5620	36.03	24.88
21243681	2011	6	6	5630	33.45	25.03
21243681	2011	6	6	5640	32.04	25.21
21243681	2011	6	6	5650	29.56	25.43
21243681	2011	6	6	5660	26.69	25.63
21243681	2011	6	6	5670	24.71	25.78
21243681	2011	6	6	5680	23.43	25.93
21243681	2011	6	6	5690	22.19	26.06
21243681	2011	6	6	5700	20.96	26.17
21243681	2011	6	6	5710	19.66	26.31
21243681	2011	6	6	5720	18.17	26.42
21243681	2011	6	6	5730	16.35	26.55
21243681	2011	6	6	5740	14.12	26.67
21243681	2011	6	6	5750	10.46	26.82
21243681	2011	6	6	5760	7.45	26.94
21243681	2011	6	6	5770	5.17	27.08
21243681	2011	6	6	5780	3.89	27.19
21243681	2011	6	6	5790	2.6	27.28
21243681	2011	6	6	5800	2.58	27.37
21243681	2011	6	6	5810	2.06	27.45
21243681	2011	6	6	5820	1.95	27.51
21243681	2011	6	6	5830	1.44	27.6
21243681	2011	6	6	5840	0.59	27.71
21243681	2011	6	6	5850	0.58	27.8
21243681	2011	6	6	5860	0.6	27.87
21243681	2011	6	6	5870	0.59	27.93
21243681	2011	6	6	5880	0.59	28.03
21243681	2011	6	6	5890	0.59	28.05
21243681	2011	6	6	5900	0.62	28.12
21243681	2011	6	6	5910	0.6	28.16

21243681	2011	6	6	5920	0.59	28.19
21243681	2011	6	6	5930	0.58	28.25
21243681	2011	6	6	5940	0.59	28.31
21243681	2011	6	6	5950	0.59	28.35
21243681	2011	6	6	5960	0.48	28.39
21243681	2011	6	6	5970	0.5	28.45
21243681	2011	6	6	5980	0.48	28.5
21254471	2011	6	6	0	0.9	29.77
21254471	2011	6	6	10	1.3	29.75
21254471	2011	6	6	20	2.27	29.74
21254471	2011	6	6	30	2.53	29.73
21254471	2011	6	6	40	2.21	29.73
21254471	2011	6	6	50	2.26	29.73
21254471	2011	6	6	60	2.46	29.71
21254471	2011	6	6	70	2.44	29.66
21254471	2011	6	6	80	2.46	29.64
21254471	2011	6	6	90	2.6	29.6
21254471	2011	6	6	100	2.81	29.58
21254471	2011	6	6	110	3.4	29.57
21254471	2011	6	6	120	5.16	29.42
21254471	2011	6	6	130	6.65	29.4
21254471	2011	6	6	140	7.22	29.39
21254471	2011	6	6	150	7.47	29.38
21254471	2011	6	6	160	7.75	29.36
21254471	2011	6	6	170	8.05	29.34
21254471	2011	6	6	180	8.39	29.32
21254471	2011	6	6	190	8.77	29.3
21254471	2011	6	6	200	9.2	29.27
21254471	2011	6	6	210	9.69	29.25
21254471	2011	6	6	220	10.3	29.21
21254471	2011	6	6	230	10.97	29.19
21254471	2011	6	6	240	11.65	29.16
21254471	2011	6	6	250	12.07	29.12
21254471	2011	6	6	260	12.29	29.08
21254471	2011	6	6	270	12.49	29.06
21254471	2011	6	6	280	12.58	29.04
21254471	2011	6	6	290	12.56	29.03
21254471	2011	6	6	300	12.55	29.01
21254471	2011	6	6	310	12.92	29
21254471	2011	6	6	320	14.26	28.94
21254471	2011	6	6	330	15.93	28.9
21254471	2011	6	6	340	17.76	28.85
21254471	2011	6	6	350	19.94	28.83
21254471	2011	6	6	360	22.33	28.79
21254471	2011	6	6	370	24.78	28.77
21254471	2011	6	6	380	26.84	28.74
21254471	2011	6	6	390	29.31	28.7

21254471	2011	6	6	400	32.37	28.63
21254471	2011	6	6	410	35.1	28.32
21254471	2011	6	6	420	37.88	28.1
21254471	2011	6	6	430	40.41	28.03
21254471	2011	6	6	440	43.21	27.93
21254471	2011	6	6	450	45.38	27.92
21254471	2011	6	6	460	47.23	27.88
21254471	2011	6	6	470	49.88	27.72
21254471	2011	6	6	480	51.59	27.67
21254471	2011	6	6	490	52.62	27.61
21254471	2011	6	6	500	53.55	27.59
21254471	2011	6	6	510	54.37	27.52
21254471	2011	6	6	520	53.36	27.46
21254471	2011	6	6	530	51.81	27.39
21254471	2011	6	6	540	51.06	27.32
21254471	2011	6	6	550	51.11	27.28
21254471	2011	6	6	560	50.16	27.22
21254471	2011	6	6	570	50.44	27.17
21254471	2011	6	6	580	51.1	27.14
21254471	2011	6	6	590	51.62	27.1
21254471	2011	6	6	600	52.2	27.05
21254471	2011	6	6	610	52.99	27
21254471	2011	6	6	620	53.42	26.96
21254471	2011	6	6	630	54.05	26.91
21254471	2011	6	6	640	55.07	26.84
21254471	2011	6	6	650	55.62	26.81
21254471	2011	6	6	660	56.54	26.77
21254471	2011	6	6	670	57.04	26.73
21254471	2011	6	6	680	56.89	26.7
21254471	2011	6	6	690	55.35	26.68
21254471	2011	6	6	700	53.52	26.66
21254471	2011	6	6	710	51.77	26.65
21254471	2011	6	6	720	50.83	26.64
21254471	2011	6	6	730	50.33	26.61
21254471	2011	6	6	740	50.05	26.59
21254471	2011	6	6	750	50.13	26.58
21254471	2011	6	6	760	50.66	26.57
21254471	2011	6	6	770	51.26	26.55
21254471	2011	6	6	780	51.29	26.53
21254471	2011	6	6	790	51.93	26.51
21254471	2011	6	6	800	52.7	26.5
21254471	2011	6	6	810	52.27	26.46
21254471	2011	6	6	820	50.84	26.46
21254471	2011	6	6	830	50.58	26.45
21254471	2011	6	6	840	51.13	26.43
21254471	2011	6	6	850	52	26.41
21254471	2011	6	6	860	52.92	26.39

21254471	2011	6	6	870	53.56	26.39
21254471	2011	6	6	880	53.87	26.36
21254471	2011	6	6	890	53.91	26.35
21254471	2011	6	6	900	53.77	26.35
21254471	2011	6	6	910	54	26.34
21254471	2011	6	6	920	54.09	26.33
21254471	2011	6	6	930	54.21	26.32
21254471	2011	6	6	940	54.21	26.31
21254471	2011	6	6	950	54.13	26.3
21254471	2011	6	6	960	53.94	26.29
21254471	2011	6	6	970	54.02	26.28
21254471	2011	6	6	980	54.04	26.28
21254471	2011	6	6	990	54.02	26.27
21254471	2011	6	6	1000	53.92	26.26
21254471	2011	6	6	1010	53.8	26.26
21254471	2011	6	6	1020	53.61	26.26
21254471	2011	6	6	1030	53.29	26.25
21254471	2011	6	6	1040	52.89	26.25
21254471	2011	6	6	1050	52.73	26.24
21254471	2011	6	6	1060	53.44	26.23
21254471	2011	6	6	1070	53.82	26.21
21254471	2011	6	6	1080	54.01	26.21
21254471	2011	6	6	1090	53.96	26.22
21254471	2011	6	6	1100	53.81	26.21
21254471	2011	6	6	1110	53.26	26.21
21254471	2011	6	6	1120	51.51	26.22
21254471	2011	6	6	1130	49.71	26.23
21254471	2011	6	6	1140	47.93	26.24
21254471	2011	6	6	1150	46.12	26.24
21254471	2011	6	6	1160	45.32	26.25
21254471	2011	6	6	1170	45.63	26.24
21254471	2011	6	6	1180	46.12	26.24
21254471	2011	6	6	1190	46.57	26.24
21254471	2011	6	6	1200	46.34	26.24
21254471	2011	6	6	1210	45.55	26.25
21254471	2011	6	6	1220	45.22	26.25
21254471	2011	6	6	1230	44.74	26.25
21254471	2011	6	6	1240	43.68	26.25
21254471	2011	6	6	1250	43.36	26.25
21254471	2011	6	6	1260	43.57	26.26
21254471	2011	6	6	1270	43.55	26.25
21254471	2011	6	6	1280	42.84	26.27
21254471	2011	6	6	1290	42.36	26.28
21254471	2011	6	6	1300	42.32	26.28
21254471	2011	6	6	1310	42.66	26.28
21254471	2011	6	6	1320	43.15	26.28
21254471	2011	6	6	1330	43.71	26.28

21254471	2011	6	6	1340	44.23	26.29
21254471	2011	6	6	1350	44.68	26.29
21254471	2011	6	6	1360	44.64	26.3
21254471	2011	6	6	1370	44.45	26.3
21254471	2011	6	6	1380	45.26	26.29
21254471	2011	6	6	1390	45.63	26.28
21254471	2011	6	6	1400	45.69	26.28
21254471	2011	6	6	1410	46.01	26.28
21254471	2011	6	6	1420	46.1	26.28
21254471	2011	6	6	1430	46.02	26.28
21254471	2011	6	6	1440	46.08	26.28
21254471	2011	6	6	1450	45.88	26.28
21254471	2011	6	6	1460	45.29	26.28
21254471	2011	6	6	1470	45.03	26.28
21254471	2011	6	6	1480	45.04	26.28
21254471	2011	6	6	1490	44.97	26.27
21254471	2011	6	6	1500	45.54	26.27
21254471	2011	6	6	1510	46.39	26.26
21254471	2011	6	6	1520	46.68	26.26
21254471	2011	6	6	1530	46.62	26.26
21254471	2011	6	6	1540	46.49	26.25
21254471	2011	6	6	1550	46.52	26.26
21254471	2011	6	6	1560	46.54	26.25
21254471	2011	6	6	1570	46.54	26.25
21254471	2011	6	6	1580	46.49	26.25
21254471	2011	6	6	1590	46.67	26.25
21254471	2011	6	6	1600	46.89	26.25
21254471	2011	6	6	1610	46.97	26.25
21254471	2011	6	6	1620	46.95	26.25
21254471	2011	6	6	1630	46.68	26.25
21254471	2011	6	6	1640	46.35	26.25
21254471	2011	6	6	1650	46.2	26.25
21254471	2011	6	6	1660	46.13	26.25
21254471	2011	6	6	1670	46.18	26.25
21254471	2011	6	6	1680	46.66	26.24
21254471	2011	6	6	1690	46.83	26.25
21254471	2011	6	6	1700	46.72	26.25
21254471	2011	6	6	1710	46.32	26.25
21254471	2011	6	6	1720	45.61	26.25
21254471	2011	6	6	1730	45.07	26.26
21254471	2011	6	6	1740	45.47	26.26
21254471	2011	6	6	1750	47.65	26.25
21254471	2011	6	6	1760	49.8	26.25
21254471	2011	6	6	1770	51.92	26.23
21254471	2011	6	6	1780	53.62	26.22
21254471	2011	6	6	1790	54.46	26.22
21254471	2011	6	6	1800	53.47	26.22

21254471	2011	6	6	1810	52.45	26.22
21254471	2011	6	6	1820	52.73	26.21
21254471	2011	6	6	1830	52.48	26.21
21254471	2011	6	6	1840	52.02	26.21
21254471	2011	6	6	1850	51.92	26.22
21254471	2011	6	6	1860	51.91	26.21
21254471	2011	6	6	1870	51.84	26.21
21254471	2011	6	6	1880	51.86	26.21
21254471	2011	6	6	1890	51.71	26.21
21254471	2011	6	6	1900	51.68	26.21
21254471	2011	6	6	1910	51.59	26.21
21254471	2011	6	6	1920	51.67	26.21
21254471	2011	6	6	1930	51.59	26.21
21254471	2011	6	6	1940	51.38	26.2
21254471	2011	6	6	1950	51.15	26.2
21254471	2011	6	6	1960	51.01	26.21
21254471	2011	6	6	1970	50.78	26.21
21254471	2011	6	6	1980	50.85	26.21
21254471	2011	6	6	1990	51.23	26.2
21254471	2011	6	6	2000	51.31	26.2
21254471	2011	6	6	2010	51.37	26.2
21254471	2011	6	6	2020	51.56	26.2
21254471	2011	6	6	2030	52.74	26.18
21254471	2011	6	6	2040	54.55	26.17
21254471	2011	6	6	2050	56.66	26.15
21254471	2011	6	6	2060	58.45	26.12
21254471	2011	6	6	2070	60.41	26.09
21254471	2011	6	6	2080	63.01	26.06
21254471	2011	6	6	2090	65.84	26.03
21254471	2011	6	6	2100	68.73	26
21254471	2011	6	6	2110	71.77	25.98
21254471	2011	6	6	2120	75.07	25.94
21254471	2011	6	6	2130	78.58	25.9
21254471	2011	6	6	2140	82.14	25.85
21254471	2011	6	6	2150	84.94	25.82
21254471	2011	6	6	2160	86.01	25.82
21254471	2011	6	6	2170	86.76	25.78
21254471	2011	6	6	2180	87.42	25.75
21254471	2011	6	6	2190	87.14	25.74
21254471	2011	6	6	2200	86.67	25.72
21254471	2011	6	6	2210	86.24	25.69
21254471	2011	6	6	2220	85.91	25.67
21254471	2011	6	6	2230	85.73	25.64
21254471	2011	6	6	2240	85.56	25.62
21254471	2011	6	6	2250	85.37	25.6
21254471	2011	6	6	2260	85.28	25.58
21254471	2011	6	6	2270	85.15	25.57

21254471	2011	6	6	2280	85.2	25.56
21254471	2011	6	6	2290	85.47	25.52
21254471	2011	6	6	2300	85.33	25.5
21254471	2011	6	6	2310	85.08	25.46
21254471	2011	6	6	2320	85.53	25.46
21254471	2011	6	6	2330	86.99	25.42
21254471	2011	6	6	2340	88.31	25.36
21254471	2011	6	6	2350	89.66	25.32
21254471	2011	6	6	2360	91.16	25.3
21254471	2011	6	6	2370	92.72	25.26
21254471	2011	6	6	2380	94.81	25.23
21254471	2011	6	6	2390	96.49	25.17
21254471	2011	6	6	2400	98.12	25.14
21254471	2011	6	6	2410	100.4	25.08
21254471	2011	6	6	2420	102.42	25.04
21254471	2011	6	6	2430	104.13	24.94
21254471	2011	6	6	2440	105.9	24.89
21254471	2011	6	6	2450	107.32	24.87
21254471	2011	6	6	2460	108.65	24.82
21254471	2011	6	6	2470	110.32	24.7
21254471	2011	6	6	2480	112.49	24.58
21254471	2011	6	6	2490	114.82	24.46
21254471	2011	6	6	2500	117.56	24.3
21254471	2011	6	6	2510	120.39	24.2
21254471	2011	6	6	2520	122.72	24.05
21254471	2011	6	6	2530	124.52	23.92
21254471	2011	6	6	2540	126.16	23.72
21254471	2011	6	6	2550	128.26	23.55
21254471	2011	6	6	2560	130.94	23.42
21254471	2011	6	6	2570	134.06	23.41
21254471	2011	6	6	2580	137.21	23.34
21254471	2011	6	6	2590	140.09	23.13
21254471	2011	6	6	2600	143.42	22.96
21254471	2011	6	6	2610	146.37	22.59
21254471	2011	6	6	2620	149.37	22.31
21254471	2011	6	6	2630	151.57	22.21
21254471	2011	6	6	2640	152.38	22.21
21254471	2011	6	6	2650	152.58	22.19
21254471	2011	6	6	2660	152.56	22.11
21254471	2011	6	6	2670	152.5	22.02
21254471	2011	6	6	2680	152.33	21.92
21254471	2011	6	6	2690	152.31	21.8
21254471	2011	6	6	2700	152.25	21.64
21254471	2011	6	6	2710	152.12	21.53
21254471	2011	6	6	2720	152.03	21.42
21254471	2011	6	6	2730	151.95	21.29
21254471	2011	6	6	2740	151.86	21.14

21254471	2011	6	6	2750	152.07	21
21254471	2011	6	6	2760	152.52	20.89
21254471	2011	6	6	2770	152.87	20.78
21254471	2011	6	6	2780	152.88	20.69
21254471	2011	6	6	2790	152.58	20.6
21254471	2011	6	6	2800	152.34	20.5
21254471	2011	6	6	2810	152.07	20.4
21254471	2011	6	6	2820	151.73	20.32
21254471	2011	6	6	2830	151.35	20.25
21254471	2011	6	6	2840	150.95	20.18
21254471	2011	6	6	2850	150.52	20.13
21254471	2011	6	6	2860	150.21	20.07
21254471	2011	6	6	2870	149.47	20
21254471	2011	6	6	2880	148.75	19.94
21254471	2011	6	6	2890	148.2	19.89
21254471	2011	6	6	2900	147.86	19.85
21254471	2011	6	6	2910	147.45	19.79
21254471	2011	6	6	2920	146.57	19.74
21254471	2011	6	6	2930	146.72	19.67
21254471	2011	6	6	2940	147.19	19.63
21254471	2011	6	6	2950	147	19.6
21254471	2011	6	6	2960	146.58	19.58
21254471	2011	6	6	2970	146.56	19.55
21254471	2011	6	6	2980	146.45	19.51
21254471	2011	6	6	2990	146.1	19.49
21254471	2011	6	6	3000	145.53	19.47
21254471	2011	6	6	3010	145.02	19.44
21254471	2011	6	6	3020	144.84	19.42
21254471	2011	6	6	3030	144.63	19.39
21254471	2011	6	6	3040	144.61	19.37
21254471	2011	6	6	3050	145.1	19.33
21254471	2011	6	6	3060	145.42	19.32
21254471	2011	6	6	3070	145.08	19.29
21254471	2011	6	6	3080	145.04	19.28
21254471	2011	6	6	3090	145.1	19.27
21254471	2011	6	6	3100	145.03	19.26
21254471	2011	6	6	3110	144.97	19.25
21254471	2011	6	6	3120	144.42	19.25
21254471	2011	6	6	3130	143.52	19.23
21254471	2011	6	6	3140	143.31	19.22
21254471	2011	6	6	3150	143.37	19.21
21254471	2011	6	6	3160	143.2	19.21
21254471	2011	6	6	3170	142.79	19.21
21254471	2011	6	6	3180	141.77	19.21
21254471	2011	6	6	3190	140.62	19.2
21254471	2011	6	6	3200	139.99	19.21
21254471	2011	6	6	3210	139.42	19.21

21254471	2011	6	6	3220	139.01	19.21
21254471	2011	6	6	3230	138.49	19.23
21254471	2011	6	6	3240	138.44	19.26
21254471	2011	6	6	3250	138.19	19.29
21254471	2011	6	6	3260	137.49	19.32
21254471	2011	6	6	3270	137.69	19.35
21254471	2011	6	6	3280	137.85	19.41
21254471	2011	6	6	3290	137.68	19.45
21254471	2011	6	6	3300	137.13	19.5
21254471	2011	6	6	3310	136.4	19.54
21254471	2011	6	6	3320	135.86	19.59
21254471	2011	6	6	3330	135.07	19.67
21254471	2011	6	6	3340	134.78	19.75
21254471	2011	6	6	3350	134.66	19.82
21254471	2011	6	6	3360	134.3	19.87
21254471	2011	6	6	3370	134.79	19.95
21254471	2011	6	6	3380	135.11	20.03
21254471	2011	6	6	3390	134.72	20.06
21254471	2011	6	6	3400	134.05	20.1
21254471	2011	6	6	3410	133.05	20.14
21254471	2011	6	6	3420	132.19	20.19
21254471	2011	6	6	3430	131.68	20.26
21254471	2011	6	6	3440	131.33	20.32
21254471	2011	6	6	3450	130.33	20.36
21254471	2011	6	6	3460	129.96	20.39
21254471	2011	6	6	3470	129.77	20.45
21254471	2011	6	6	3480	129.56	20.49
21254471	2011	6	6	3490	129.51	20.54
21254471	2011	6	6	3500	129.38	20.59
21254471	2011	6	6	3510	128.65	20.64
21254471	2011	6	6	3520	128.84	20.71
21254471	2011	6	6	3530	129.62	20.75
21254471	2011	6	6	3540	129.66	20.77
21254471	2011	6	6	3550	129.53	20.78
21254471	2011	6	6	3560	129.82	20.82
21254471	2011	6	6	3570	129.28	20.85
21254471	2011	6	6	3580	128.67	20.88
21254471	2011	6	6	3590	128.27	20.91
21254471	2011	6	6	3600	127.92	20.96
21254471	2011	6	6	3610	128.62	21
21254471	2011	6	6	3620	128.58	21
21254471	2011	6	6	3630	127.5	21.03
21254471	2011	6	6	3640	126.33	21.04
21254471	2011	6	6	3650	124.89	21.09
21254471	2011	6	6	3660	123.2	21.12
21254471	2011	6	6	3670	121.61	21.16
21254471	2011	6	6	3680	119.96	21.23

21254471	2011	6	6	3690	118.89	21.31
21254471	2011	6	6	3700	117.38	21.39
21254471	2011	6	6	3710	115.71	21.47
21254471	2011	6	6	3720	114.63	21.53
21254471	2011	6	6	3730	113.2	21.62
21254471	2011	6	6	3740	112.29	21.7
21254471	2011	6	6	3750	111.94	21.77
21254471	2011	6	6	3760	111.85	21.85
21254471	2011	6	6	3770	111.2	21.94
21254471	2011	6	6	3780	110.29	22
21254471	2011	6	6	3790	109.69	22.06
21254471	2011	6	6	3800	109.22	22.12
21254471	2011	6	6	3810	109.06	22.17
21254471	2011	6	6	3820	108.9	22.24
21254471	2011	6	6	3830	108.99	22.32
21254471	2011	6	6	3840	109.16	22.38
21254471	2011	6	6	3850	108.77	22.41
21254471	2011	6	6	3860	108.65	22.46
21254471	2011	6	6	3870	108.65	22.5
21254471	2011	6	6	3880	108.16	22.55
21254471	2011	6	6	3890	107.97	22.6
21254471	2011	6	6	3900	107.26	22.64
21254471	2011	6	6	3910	106.89	22.68
21254471	2011	6	6	3920	106.55	22.71
21254471	2011	6	6	3930	105.95	22.75
21254471	2011	6	6	3940	105.23	22.78
21254471	2011	6	6	3950	104.83	22.82
21254471	2011	6	6	3960	104.9	22.85
21254471	2011	6	6	3970	104.68	22.89
21254471	2011	6	6	3980	104.35	22.9
21254471	2011	6	6	3990	103.97	22.92
21254471	2011	6	6	4000	103.45	22.96
21254471	2011	6	6	4010	103.18	22.99
21254471	2011	6	6	4020	102.95	23
21254471	2011	6	6	4030	102.24	23.04
21254471	2011	6	6	4040	101.52	23.07
21254471	2011	6	6	4050	101.52	23.07
21254471	2011	6	6	4060	100.6	23.1
21254471	2011	6	6	4070	99.23	23.11
21254471	2011	6	6	4080	98.21	23.14
21254471	2011	6	6	4090	97.59	23.16
21254471	2011	6	6	4100	97.23	23.17
21254471	2011	6	6	4110	96.9	23.2
21254471	2011	6	6	4120	96.51	23.21
21254471	2011	6	6	4130	95.4	23.27
21254471	2011	6	6	4140	93.24	23.33
21254471	2011	6	6	4150	91.83	23.35

21254471	2011	6	6	4160	90.67	23.4
21254471	2011	6	6	4170	89.76	23.46
21254471	2011	6	6	4180	88.58	23.53
21254471	2011	6	6	4190	87.27	23.6
21254471	2011	6	6	4200	86.27	23.66
21254471	2011	6	6	4210	84.69	23.73
21254471	2011	6	6	4220	83.14	23.78
21254471	2011	6	6	4230	81.47	23.89
21254471	2011	6	6	4240	79.21	23.96
21254471	2011	6	6	4250	77.14	24.01
21254471	2011	6	6	4260	75.16	24.09
21254471	2011	6	6	4270	73.25	24.16
21254471	2011	6	6	4280	71.3	24.25
21254471	2011	6	6	4290	69.33	24.35
21254471	2011	6	6	4300	67.71	24.42
21254471	2011	6	6	4310	67.44	24.53
21254471	2011	6	6	4320	65.82	24.58
21254471	2011	6	6	4330	63.34	24.64
21254471	2011	6	6	4340	60.73	24.71
21254471	2011	6	6	4350	58.15	24.78
21254471	2011	6	6	4360	55.7	24.86
21254471	2011	6	6	4370	53.75	24.96
21254471	2011	6	6	4380	52.91	25.03
21254471	2011	6	6	4390	52.6	25.09
21254471	2011	6	6	4400	52.49	25.16
21254471	2011	6	6	4410	52.65	25.2
21254471	2011	6	6	4420	52.7	25.25
21254471	2011	6	6	4430	52.45	25.28
21254471	2011	6	6	4440	51.89	25.34
21254471	2011	6	6	4450	50.91	25.39
21254471	2011	6	6	4460	49.75	25.43
21254471	2011	6	6	4470	47.92	25.5
21254471	2011	6	6	4480	46.23	25.56
21254471	2011	6	6	4490	44.08	25.63
21254471	2011	6	6	4500	42.12	25.67
21254471	2011	6	6	4510	40.18	25.71
21254471	2011	6	6	4520	38.26	25.76
21254471	2011	6	6	4530	37.22	25.8
21254471	2011	6	6	4540	36.45	25.82
21254471	2011	6	6	4550	35.94	25.85
21254471	2011	6	6	4560	35.83	25.89
21254471	2011	6	6	4570	35.92	25.92
21254471	2011	6	6	4580	35.36	25.96
21254471	2011	6	6	4590	35.05	25.99
21254471	2011	6	6	4600	34.93	26
21254471	2011	6	6	4610	33.83	26.03
21254471	2011	6	6	4620	32.28	26.07

21254471	2011	6	6	4630	31.95	26.09
21254471	2011	6	6	4640	31.92	26.11
21254471	2011	6	6	4650	31.29	26.14
21254471	2011	6	6	4660	30.77	26.19
21254471	2011	6	6	4670	30.04	26.22
21254471	2011	6	6	4680	29.76	26.26
21254471	2011	6	6	4690	29.31	26.29
21254471	2011	6	6	4700	28.85	26.34
21254471	2011	6	6	4710	28.3	26.39
21254471	2011	6	6	4720	27.54	26.45
21254471	2011	6	6	4730	26.89	26.55
21254471	2011	6	6	4740	26.55	26.63
21254471	2011	6	6	4750	26.15	26.71
21254471	2011	6	6	4760	25.9	26.81
21254471	2011	6	6	4770	26.15	26.85
21254471	2011	6	6	4780	26.63	26.93
21254471	2011	6	6	4790	26.8	27.02
21254471	2011	6	6	4800	26.54	27.12
21254471	2011	6	6	4810	26.46	27.17
21254471	2011	6	6	4820	25.91	27.24
21254471	2011	6	6	4830	25.98	27.29
21254471	2011	6	6	4840	25.94	27.35
21254471	2011	6	6	4850	25.34	27.42
21254471	2011	6	6	4860	25.13	27.47
21254471	2011	6	6	4870	24.89	27.52
21254471	2011	6	6	4880	23.87	27.59
21254471	2011	6	6	4890	23.68	27.64
21254471	2011	6	6	4900	23.61	27.69
21254471	2011	6	6	4910	23.91	27.71
21254471	2011	6	6	4920	24.12	27.75
21254471	2011	6	6	4930	24.25	27.81
21254471	2011	6	6	4940	24.43	27.84
21254471	2011	6	6	4950	24.83	27.86
21254471	2011	6	6	4960	25.39	27.88
21254471	2011	6	6	4970	25.37	27.92
21254471	2011	6	6	4980	25.31	27.95
21254471	2011	6	6	4990	25.58	27.96
21254471	2011	6	6	5000	25.42	28
21254471	2011	6	6	5010	25.86	28.02
21254471	2011	6	6	5020	26.43	28.03
21254471	2011	6	6	5030	27.09	28.06
21254471	2011	6	6	5040	26.87	28.08
21254471	2011	6	6	5050	25.61	28.11
21254471	2011	6	6	5060	25.04	28.14
21254471	2011	6	6	5070	23.52	28.17
21254471	2011	6	6	5080	23.25	28.18
21254471	2011	6	6	5090	22.65	28.21

21254471	2011	6	6	5100	22.07	28.22
21254471	2011	6	6	5110	21.51	28.25
21254471	2011	6	6	5120	21.17	28.27
21254471	2011	6	6	5130	21.01	28.28
21254471	2011	6	6	5140	20.91	28.3
21254471	2011	6	6	5150	20.97	28.32
21254471	2011	6	6	5160	21.05	28.33
21254471	2011	6	6	5170	20.66	28.35
21254471	2011	6	6	5180	20.57	28.37
21254471	2011	6	6	5190	20.47	28.37
21254471	2011	6	6	5200	20.27	28.38
21254471	2011	6	6	5210	19.68	28.4
21254471	2011	6	6	5220	18.02	28.42
21254471	2011	6	6	5230	15.59	28.45
21254471	2011	6	6	5240	13.05	28.49
21254471	2011	6	6	5250	10.54	28.52
21254471	2011	6	6	5260	7.79	28.56
21254471	2011	6	6	5270	5.25	28.6
21254471	2011	6	6	5280	3.31	28.64
21254471	2011	6	6	5290	2.81	28.67
21254471	2011	6	6	5300	2.32	28.71
21254471	2011	6	6	5310	2.26	28.75
21254471	2011	6	6	5320	2.57	28.78
21254471	2011	6	6	5330	0.66	28.82
21254471	2011	6	6	5340	0.58	28.88
21254471	2011	6	6	5350	0.59	28.89
21254471	2011	6	6	5360	0.61	28.92
21254471	2011	6	6	5370	0.62	28.94
21254471	2011	6	6	5380	0.58	29
21254471	2011	6	6	5390	0.59	29.02
21254471	2011	6	6	5400	0.6	29.06
21254471	2011	6	6	5410	0.58	29.09
21254471	2011	6	6	5420	0.58	29.12
21254471	2011	6	6	5430	0.59	29.17
21254471	2011	6	6	5440	0.59	29.2
21254471	2011	6	6	5450	0.6	29.21
21254471	2011	6	6	5460	0.58	29.24
21254471	2011	6	6	5470	0.55	29.26
21338801	2011	6	7	0	1.4	29.85
21338801	2011	6	7	10	2.38	29.85
21338801	2011	6	7	20	2.34	29.83
21338801	2011	6	7	30	2.03	29.83
21338801	2011	6	7	40	2.19	29.82
21338801	2011	6	7	50	1.97	29.82
21338801	2011	6	7	60	1.37	29.82
21338801	2011	6	7	70	0.89	29.82
21338801	2011	6	7	80	1.47	29.81

21338801	2011	6	7	90	1.78	29.81
21338801	2011	6	7	100	2.11	29.8
21338801	2011	6	7	110	2.43	29.8
21338801	2011	6	7	120	2.64	29.77
21338801	2011	6	7	130	2.9	29.75
21338801	2011	6	7	140	3.23	29.74
21338801	2011	6	7	150	3.8	29.71
21338801	2011	6	7	160	5.5	29.67
21338801	2011	6	7	170	6.19	29.64
21338801	2011	6	7	180	7.55	29.62
21338801	2011	6	7	190	9.83	29.58
21338801	2011	6	7	200	12.04	29.54
21338801	2011	6	7	210	12.94	29.52
21338801	2011	6	7	220	13.44	29.5
21338801	2011	6	7	230	14.94	29.46
21338801	2011	6	7	240	17.08	29.38
21338801	2011	6	7	250	19.64	29.32
21338801	2011	6	7	260	22.49	29.25
21338801	2011	6	7	270	25.65	29.17
21338801	2011	6	7	280	28.79	29.11
21338801	2011	6	7	290	31.72	29.04
21338801	2011	6	7	300	34.74	28.96
21338801	2011	6	7	310	37.52	28.87
21338801	2011	6	7	320	39.31	28.82
21338801	2011	6	7	330	40.67	28.78
21338801	2011	6	7	340	42.79	28.62
21338801	2011	6	7	350	45.14	28.53
21338801	2011	6	7	360	47.69	28.36
21338801	2011	6	7	370	50.01	28.25
21338801	2011	6	7	380	52.25	28.16
21338801	2011	6	7	390	54.77	28.07
21338801	2011	6	7	400	57.47	27.94
21338801	2011	6	7	410	59.9	27.85
21338801	2011	6	7	420	62.4	27.75
21338801	2011	6	7	430	65.16	27.52
21338801	2011	6	7	440	67.27	27.39
21338801	2011	6	7	450	69.28	27.23
21338801	2011	6	7	460	71.21	27.13
21338801	2011	6	7	470	73.37	27.07
21338801	2011	6	7	480	75.49	26.96
21338801	2011	6	7	490	77.57	26.89
21338801	2011	6	7	500	79.72	26.82
21338801	2011	6	7	510	81.76	26.72
21338801	2011	6	7	520	83.99	26.64
21338801	2011	6	7	530	86.3	26.52
21338801	2011	6	7	540	88.7	26.46
21338801	2011	6	7	550	90.94	26.27

21338801	2011	6	7	560	93	26.13
21338801	2011	6	7	570	95.27	26
21338801	2011	6	7	580	97.47	25.85
21338801	2011	6	7	590	99.59	25.63
21338801	2011	6	7	600	101.82	25.43
21338801	2011	6	7	610	104.81	25.38
21338801	2011	6	7	620	108.36	25.3
21338801	2011	6	7	630	111.97	25.16
21338801	2011	6	7	640	115.5	24.97
21338801	2011	6	7	650	118.4	24.74
21338801	2011	6	7	660	121.04	24.5
21338801	2011	6	7	670	123.33	23.98
21338801	2011	6	7	680	125.77	23.87
21338801	2011	6	7	690	128.36	23.67
21338801	2011	6	7	700	130.57	23.25
21338801	2011	6	7	710	132.53	22.99
21338801	2011	6	7	720	135.07	22.9
21338801	2011	6	7	730	137.93	22.71
21338801	2011	6	7	740	140.43	22.51
21338801	2011	6	7	750	142.87	22.14
21338801	2011	6	7	760	144.93	22.01
21338801	2011	6	7	770	147.1	21.9
21338801	2011	6	7	780	149.66	21.83
21338801	2011	6	7	790	152.92	21.66
21338801	2011	6	7	800	156.52	21.5
21338801	2011	6	7	810	159.88	21.41
21338801	2011	6	7	820	162.43	21.32
21338801	2011	6	7	830	164.93	21.2
21338801	2011	6	7	840	166.24	21.07
21338801	2011	6	7	850	167.18	21.01
21338801	2011	6	7	860	167.91	20.89
21338801	2011	6	7	870	168.54	20.75
21338801	2011	6	7	880	169.13	20.64
21338801	2011	6	7	890	169.77	20.48
21338801	2011	6	7	900	170.1	20.35
21338801	2011	6	7	910	170.18	20.25
21338801	2011	6	7	920	170.44	20.17
21338801	2011	6	7	930	170.87	20.1
21338801	2011	6	7	940	171.13	20
21338801	2011	6	7	950	171.54	19.92
21338801	2011	6	7	960	171.81	19.81
21338801	2011	6	7	970	172.19	19.72
21338801	2011	6	7	980	172.55	19.64
21338801	2011	6	7	990	172.88	19.53
21338801	2011	6	7	1000	173.09	19.44
21338801	2011	6	7	1010	173.21	19.39
21338801	2011	6	7	1020	173.27	19.33

21338801	2011	6	7	1030	173.4	19.25
21338801	2011	6	7	1040	174.85	19.09
21338801	2011	6	7	1050	176.76	19
21338801	2011	6	7	1060	178.41	18.91
21338801	2011	6	7	1070	179.99	18.82
21338801	2011	6	7	1080	181.65	18.72
21338801	2011	6	7	1090	181.75	18.61
21338801	2011	6	7	1100	181.45	18.57
21338801	2011	6	7	1110	180.88	18.53
21338801	2011	6	7	1120	180.78	18.47
21338801	2011	6	7	1130	181.05	18.42
21338801	2011	6	7	1140	181.21	18.35
21338801	2011	6	7	1150	181.33	18.28
21338801	2011	6	7	1160	181.68	18.23
21338801	2011	6	7	1170	182.27	18.14
21338801	2011	6	7	1180	183.12	18.08
21338801	2011	6	7	1190	183.78	18.03
21338801	2011	6	7	1200	184.18	17.95
21338801	2011	6	7	1210	184.39	17.92
21338801	2011	6	7	1220	184.44	17.87
21338801	2011	6	7	1230	184.52	17.84
21338801	2011	6	7	1240	184.57	17.81
21338801	2011	6	7	1250	184.56	17.78
21338801	2011	6	7	1260	184.42	17.75
21338801	2011	6	7	1270	184.38	17.71
21338801	2011	6	7	1280	184.54	17.67
21338801	2011	6	7	1290	184.4	17.63
21338801	2011	6	7	1300	184.26	17.6
21338801	2011	6	7	1310	184.09	17.58
21338801	2011	6	7	1320	184.22	17.54
21338801	2011	6	7	1330	184.51	17.5
21338801	2011	6	7	1340	184.44	17.47
21338801	2011	6	7	1350	184.27	17.45
21338801	2011	6	7	1360	184.14	17.42
21338801	2011	6	7	1370	184	17.39
21338801	2011	6	7	1380	183.81	17.37
21338801	2011	6	7	1390	183.57	17.35
21338801	2011	6	7	1400	183.41	17.32
21338801	2011	6	7	1410	183.38	17.3
21338801	2011	6	7	1420	183.64	17.28
21338801	2011	6	7	1430	183.38	17.25
21338801	2011	6	7	1440	182.93	17.21
21338801	2011	6	7	1450	182.72	17.21
21338801	2011	6	7	1460	182.46	17.2
21338801	2011	6	7	1470	182.16	17.19
21338801	2011	6	7	1480	181.88	17.19
21338801	2011	6	7	1490	181.61	17.17

21338801	2011	6	7	1500	181.4	17.16
21338801	2011	6	7	1510	181	17.14
21338801	2011	6	7	1520	181.08	17.13
21338801	2011	6	7	1530	181	17.11
21338801	2011	6	7	1540	180.86	17.1
21338801	2011	6	7	1550	180.8	17.09
21338801	2011	6	7	1560	180.99	17.08
21338801	2011	6	7	1570	181.13	17.06
21338801	2011	6	7	1580	180.83	17.05
21338801	2011	6	7	1590	180.86	17.05
21338801	2011	6	7	1600	181.09	17.03
21338801	2011	6	7	1610	180.6	17.02
21338801	2011	6	7	1620	180.2	17.01
21338801	2011	6	7	1630	179.94	17.01
21338801	2011	6	7	1640	179.81	17
21338801	2011	6	7	1650	179.79	16.99
21338801	2011	6	7	1660	179.74	16.98
21338801	2011	6	7	1670	179.65	16.97
21338801	2011	6	7	1680	179.73	16.97
21338801	2011	6	7	1690	180.01	16.96
21338801	2011	6	7	1700	179.77	16.92
21338801	2011	6	7	1710	179.01	16.91
21338801	2011	6	7	1720	178.93	16.91
21338801	2011	6	7	1730	178.98	16.91
21338801	2011	6	7	1740	178.86	16.9
21338801	2011	6	7	1750	179.46	16.89
21338801	2011	6	7	1760	179.66	16.89
21338801	2011	6	7	1770	179.75	16.89
21338801	2011	6	7	1780	180.01	16.89
21338801	2011	6	7	1790	180.93	16.89
21338801	2011	6	7	1800	181.98	16.89
21338801	2011	6	7	1810	182.27	16.88
21338801	2011	6	7	1820	182.15	16.88
21338801	2011	6	7	1830	181.71	16.88
21338801	2011	6	7	1840	181.78	16.87
21338801	2011	6	7	1850	182.41	16.86
21338801	2011	6	7	1860	182.84	16.87
21338801	2011	6	7	1870	182.96	16.86
21338801	2011	6	7	1880	182.86	16.85
21338801	2011	6	7	1890	182.77	16.85
21338801	2011	6	7	1900	182.66	16.85
21338801	2011	6	7	1910	182.8	16.85
21338801	2011	6	7	1920	183.53	16.85
21338801	2011	6	7	1930	183.76	16.85
21338801	2011	6	7	1940	183.77	16.84
21338801	2011	6	7	1950	183.34	16.84
21338801	2011	6	7	1960	183.04	16.84

21338801	2011	6	7	1970	182.83	16.84
21338801	2011	6	7	1980	182.82	16.85
21338801	2011	6	7	1990	182.76	16.84
21338801	2011	6	7	2000	182.73	16.83
21338801	2011	6	7	2010	182.55	16.84
21338801	2011	6	7	2020	182.36	16.84
21338801	2011	6	7	2030	182.19	16.84
21338801	2011	6	7	2040	182.09	16.83
21338801	2011	6	7	2050	181.97	16.83
21338801	2011	6	7	2060	182.03	16.84
21338801	2011	6	7	2070	182.89	16.83
21338801	2011	6	7	2080	184.1	16.82
21338801	2011	6	7	2090	183.91	16.83
21338801	2011	6	7	2100	183.39	16.84
21338801	2011	6	7	2110	183.35	16.83
21338801	2011	6	7	2120	184.06	16.83
21338801	2011	6	7	2130	185.63	16.84
21338801	2011	6	7	2140	186.38	16.83
21338801	2011	6	7	2150	187.49	16.82
21338801	2011	6	7	2160	188.68	16.83
21338801	2011	6	7	2170	189.09	16.82
21338801	2011	6	7	2180	189.78	16.82
21338801	2011	6	7	2190	190.2	16.82
21338801	2011	6	7	2200	190.46	16.81
21338801	2011	6	7	2210	190.53	16.81
21338801	2011	6	7	2220	190.25	16.82
21338801	2011	6	7	2230	189.43	16.8
21338801	2011	6	7	2240	188.99	16.79
21338801	2011	6	7	2250	188.66	16.79
21338801	2011	6	7	2260	188.51	16.79
21338801	2011	6	7	2270	188.3	16.79
21338801	2011	6	7	2280	188.6	16.78
21338801	2011	6	7	2290	189.14	16.78
21338801	2011	6	7	2300	189.33	16.78
21338801	2011	6	7	2310	189.29	16.78
21338801	2011	6	7	2320	189.78	16.78
21338801	2011	6	7	2330	189.67	16.78
21338801	2011	6	7	2340	189.39	16.77
21338801	2011	6	7	2350	189.21	16.78
21338801	2011	6	7	2360	188.92	16.77
21338801	2011	6	7	2370	188.82	16.77
21338801	2011	6	7	2380	188.92	16.77
21338801	2011	6	7	2390	189.84	16.76
21338801	2011	6	7	2400	190.8	16.77
21338801	2011	6	7	2410	191.66	16.76
21338801	2011	6	7	2420	192.27	16.75
21338801	2011	6	7	2430	193.09	16.75

21338801	2011	6	7	2440	194.29	16.74
21338801	2011	6	7	2450	194.64	16.74
21338801	2011	6	7	2460	194.71	16.74
21338801	2011	6	7	2470	194.75	16.74
21338801	2011	6	7	2480	194.82	16.73
21338801	2011	6	7	2490	194.72	16.71
21338801	2011	6	7	2500	194.64	16.72
21338801	2011	6	7	2510	194.7	16.71
21338801	2011	6	7	2520	194.61	16.71
21338801	2011	6	7	2530	194.48	16.71
21338801	2011	6	7	2540	194.59	16.69
21338801	2011	6	7	2550	194.61	16.69
21338801	2011	6	7	2560	194.45	16.68
21338801	2011	6	7	2570	195.49	16.67
21338801	2011	6	7	2580	196.28	16.65
21338801	2011	6	7	2590	196.6	16.64
21338801	2011	6	7	2600	196.89	16.62
21338801	2011	6	7	2610	197.1	16.6
21338801	2011	6	7	2620	197.3	16.6
21338801	2011	6	7	2630	197.76	16.6
21338801	2011	6	7	2640	197.74	16.59
21338801	2011	6	7	2650	197.81	16.58
21338801	2011	6	7	2660	197.42	16.57
21338801	2011	6	7	2670	197.27	16.57
21338801	2011	6	7	2680	197.57	16.55
21338801	2011	6	7	2690	198.43	16.52
21338801	2011	6	7	2700	198.33	16.53
21338801	2011	6	7	2710	197.77	16.51
21338801	2011	6	7	2720	199.07	16.5
21338801	2011	6	7	2730	200.16	16.5
21338801	2011	6	7	2740	200.57	16.47
21338801	2011	6	7	2750	201.47	16.47
21338801	2011	6	7	2760	201.98	16.46
21338801	2011	6	7	2770	202.75	16.46
21338801	2011	6	7	2780	203.56	16.46
21338801	2011	6	7	2790	205.16	16.42
21338801	2011	6	7	2800	206.86	16.41
21338801	2011	6	7	2810	208.13	16.38
21338801	2011	6	7	2820	208.72	16.35
21338801	2011	6	7	2830	209.39	16.35
21338801	2011	6	7	2840	210.53	16.31
21338801	2011	6	7	2850	211.85	16.29
21338801	2011	6	7	2860	212.18	16.22
21338801	2011	6	7	2870	212.38	16.21
21338801	2011	6	7	2880	213.09	16.17
21338801	2011	6	7	2890	213.2	16.16
21338801	2011	6	7	2900	213.13	16.16

21338801	2011	6	7	2910	212.69	16.14
21338801	2011	6	7	2920	212.92	16.13
21338801	2011	6	7	2930	211.84	16.1
21338801	2011	6	7	2940	211.67	16.08
21338801	2011	6	7	2950	212.37	16.06
21338801	2011	6	7	2960	212.55	16.03
21338801	2011	6	7	2970	212.82	16.02
21338801	2011	6	7	2980	214.2	16
21338801	2011	6	7	2990	214.63	15.97
21338801	2011	6	7	3000	214.22	15.97
21338801	2011	6	7	3010	213.97	15.96
21338801	2011	6	7	3020	214.6	15.96
21338801	2011	6	7	3030	214.92	15.95
21338801	2011	6	7	3040	214.72	15.93
21338801	2011	6	7	3050	215.12	15.89
21338801	2011	6	7	3060	215.72	15.85
21338801	2011	6	7	3070	215.56	15.83
21338801	2011	6	7	3080	215.14	15.83
21338801	2011	6	7	3090	214.66	15.82
21338801	2011	6	7	3100	214.4	15.81
21338801	2011	6	7	3110	213.96	15.8
21338801	2011	6	7	3120	213.47	15.79
21338801	2011	6	7	3130	213.08	15.78
21338801	2011	6	7	3140	213.46	15.77
21338801	2011	6	7	3150	213.46	15.75
21338801	2011	6	7	3160	213.42	15.75
21338801	2011	6	7	3170	213.43	15.75
21338801	2011	6	7	3180	213.12	15.74
21338801	2011	6	7	3190	212.86	15.75
21338801	2011	6	7	3200	212.86	15.75
21338801	2011	6	7	3210	212.87	15.75
21338801	2011	6	7	3220	212.86	15.73
21338801	2011	6	7	3230	212.81	15.74
21338801	2011	6	7	3240	212.65	15.72
21338801	2011	6	7	3250	212.49	15.73
21338801	2011	6	7	3260	212.26	15.72
21338801	2011	6	7	3270	212.06	15.71
21338801	2011	6	7	3280	212.79	15.71
21338801	2011	6	7	3290	213.54	15.72
21338801	2011	6	7	3300	214.31	15.71
21338801	2011	6	7	3310	214.69	15.71
21338801	2011	6	7	3320	214.8	15.71
21338801	2011	6	7	3330	214.55	15.69
21338801	2011	6	7	3340	214.32	15.7
21338801	2011	6	7	3350	214.12	15.7
21338801	2011	6	7	3360	213.95	15.69
21338801	2011	6	7	3370	213.91	15.69

21338801	2011	6	7	3380	214.48	15.68
21338801	2011	6	7	3390	214.84	15.68
21338801	2011	6	7	3400	214.76	15.68
21338801	2011	6	7	3410	214.29	15.68
21338801	2011	6	7	3420	213.97	15.67
21338801	2011	6	7	3430	213.75	15.67
21338801	2011	6	7	3440	213.56	15.68
21338801	2011	6	7	3450	213.78	15.68
21338801	2011	6	7	3460	213.9	15.69
21338801	2011	6	7	3470	214.08	15.7
21338801	2011	6	7	3480	214.06	15.71
21338801	2011	6	7	3490	214.09	15.71
21338801	2011	6	7	3500	214.19	15.71
21338801	2011	6	7	3510	213.82	15.72
21338801	2011	6	7	3520	213.47	15.73
21338801	2011	6	7	3530	213.71	15.74
21338801	2011	6	7	3540	214.08	15.75
21338801	2011	6	7	3550	214.25	15.76
21338801	2011	6	7	3560	214.37	15.77
21338801	2011	6	7	3570	213.99	15.78
21338801	2011	6	7	3580	213.81	15.79
21338801	2011	6	7	3590	214.01	15.79
21338801	2011	6	7	3600	214.22	15.79
21338801	2011	6	7	3610	213.69	15.81
21338801	2011	6	7	3620	213.23	15.81
21338801	2011	6	7	3630	213.71	15.82
21338801	2011	6	7	3640	214.09	15.83
21338801	2011	6	7	3650	214.32	15.82
21338801	2011	6	7	3660	214.48	15.83
21338801	2011	6	7	3670	214.62	15.83
21338801	2011	6	7	3680	214.86	15.83
21338801	2011	6	7	3690	214.89	15.84
21338801	2011	6	7	3700	214.9	15.83
21338801	2011	6	7	3710	214.91	15.84
21338801	2011	6	7	3720	215.6	15.83
21338801	2011	6	7	3730	216.07	15.84
21338801	2011	6	7	3740	216.25	15.83
21338801	2011	6	7	3750	216.06	15.85
21338801	2011	6	7	3760	215.8	15.84
21338801	2011	6	7	3770	215.67	15.84
21338801	2011	6	7	3780	215.95	15.85
21338801	2011	6	7	3790	215.24	15.83
21338801	2011	6	7	3800	214.8	15.84
21338801	2011	6	7	3810	215.04	15.84
21338801	2011	6	7	3820	216.25	15.84
21338801	2011	6	7	3830	216.66	15.83
21338801	2011	6	7	3840	216.37	15.83

21338801	2011	6	7	3850	216.41	15.82
21338801	2011	6	7	3860	217.04	15.82
21338801	2011	6	7	3870	217.7	15.81
21338801	2011	6	7	3880	217.8	15.82
21338801	2011	6	7	3890	217.81	15.82
21338801	2011	6	7	3900	217.61	15.81
21338801	2011	6	7	3910	216.8	15.8
21338801	2011	6	7	3920	216.42	15.79
21338801	2011	6	7	3930	216.81	15.78
21338801	2011	6	7	3940	217.47	15.78
21338801	2011	6	7	3950	217.99	15.77
21338801	2011	6	7	3960	218.27	15.77
21338801	2011	6	7	3970	218.47	15.77
21338801	2011	6	7	3980	218.63	15.77
21338801	2011	6	7	3990	219.58	15.77
21338801	2011	6	7	4000	219.97	15.76
21338801	2011	6	7	4010	220.22	15.76
21338801	2011	6	7	4020	220.58	15.75
21338801	2011	6	7	4030	220.42	15.74
21338801	2011	6	7	4040	220.1	15.74
21338801	2011	6	7	4050	219.43	15.73
21338801	2011	6	7	4060	219.49	15.72
21338801	2011	6	7	4070	219.53	15.71
21338801	2011	6	7	4080	219.59	15.71
21338801	2011	6	7	4090	219.47	15.71
21338801	2011	6	7	4100	218.58	15.71
21338801	2011	6	7	4110	218.25	15.71
21338801	2011	6	7	4120	218.34	15.7
21338801	2011	6	7	4130	218.5	15.7
21338801	2011	6	7	4140	218.71	15.69
21338801	2011	6	7	4150	219.01	15.7
21338801	2011	6	7	4160	219.28	15.69
21338801	2011	6	7	4170	219.92	15.69
21338801	2011	6	7	4180	221.1	15.69
21338801	2011	6	7	4190	222.85	15.68
21338801	2011	6	7	4200	223.38	15.68
21338801	2011	6	7	4210	223.37	15.67
21338801	2011	6	7	4220	223.76	15.65
21338801	2011	6	7	4230	223.97	15.64
21338801	2011	6	7	4240	224.14	15.61
21338801	2011	6	7	4250	224.23	15.6
21338801	2011	6	7	4260	224.18	15.59
21338801	2011	6	7	4270	224	15.58
21338801	2011	6	7	4280	223.71	15.57
21338801	2011	6	7	4290	223.4	15.55
21338801	2011	6	7	4300	223.03	15.53
21338801	2011	6	7	4310	223.2	15.53

21338801	2011	6	7	4320	224.21	15.5
21338801	2011	6	7	4330	224.51	15.48
21338801	2011	6	7	4340	224.48	15.48
21338801	2011	6	7	4350	224.42	15.46
21338801	2011	6	7	4360	224.46	15.45
21338801	2011	6	7	4370	224.49	15.45
21338801	2011	6	7	4380	224.54	15.45
21338801	2011	6	7	4390	224.46	15.44
21338801	2011	6	7	4400	224.67	15.43
21338801	2011	6	7	4410	224.52	15.42
21338801	2011	6	7	4420	224.3	15.42
21338801	2011	6	7	4430	224	15.42
21338801	2011	6	7	4440	224.15	15.41
21338801	2011	6	7	4450	224.26	15.4
21338801	2011	6	7	4460	224.24	15.4
21338801	2011	6	7	4470	224.11	15.4
21338801	2011	6	7	4480	224.22	15.4
21338801	2011	6	7	4490	224.32	15.39
21338801	2011	6	7	4500	224.28	15.39
21338801	2011	6	7	4510	224.39	15.39
21338801	2011	6	7	4520	224.12	15.39
21338801	2011	6	7	4530	223.82	15.39
21338801	2011	6	7	4540	223.53	15.39
21338801	2011	6	7	4550	223.34	15.39
21338801	2011	6	7	4560	223.43	15.39
21338801	2011	6	7	4570	223.67	15.4
21338801	2011	6	7	4580	223.59	15.39
21338801	2011	6	7	4590	223.53	15.4
21338801	2011	6	7	4600	223.48	15.4
21338801	2011	6	7	4610	223.53	15.4
21338801	2011	6	7	4620	223.57	15.41
21338801	2011	6	7	4630	223.54	15.41
21338801	2011	6	7	4640	223.65	15.41
21338801	2011	6	7	4650	223.67	15.41
21338801	2011	6	7	4660	223.75	15.41
21338801	2011	6	7	4670	223.78	15.41
21338801	2011	6	7	4680	223.82	15.41
21338801	2011	6	7	4690	223.92	15.42
21338801	2011	6	7	4700	223.91	15.41
21338801	2011	6	7	4710	223.98	15.42
21338801	2011	6	7	4720	223.67	15.42
21338801	2011	6	7	4730	223.8	15.42
21338801	2011	6	7	4740	223.79	15.42
21338801	2011	6	7	4750	223.85	15.42
21338801	2011	6	7	4760	223.54	15.41
21338801	2011	6	7	4770	223.3	15.41
21338801	2011	6	7	4780	223.06	15.41

21338801	2011	6	7	4790	223.23	15.41
21338801	2011	6	7	4800	223.49	15.41
21338801	2011	6	7	4810	223.49	15.4
21338801	2011	6	7	4820	223.42	15.41
21338801	2011	6	7	4830	223.35	15.4
21338801	2011	6	7	4840	223.06	15.4
21338801	2011	6	7	4850	222.67	15.4
21338801	2011	6	7	4860	222.2	15.39
21338801	2011	6	7	4870	221.77	15.4
21338801	2011	6	7	4880	221.68	15.4
21338801	2011	6	7	4890	221.87	15.39
21338801	2011	6	7	4900	223.12	15.39
21338801	2011	6	7	4910	223.3	15.39
21338801	2011	6	7	4920	223.63	15.38
21338801	2011	6	7	4930	224.26	15.37
21338801	2011	6	7	4940	223.92	15.35
21338801	2011	6	7	4950	223.78	15.35
21338801	2011	6	7	4960	223.64	15.33
21338801	2011	6	7	4970	223.73	15.32
21338801	2011	6	7	4980	223.86	15.31
21338801	2011	6	7	4990	224.03	15.29
21338801	2011	6	7	5000	223.6	15.28
21338801	2011	6	7	5010	223.36	15.26
21338801	2011	6	7	5020	223.35	15.26
21338801	2011	6	7	5030	223.43	15.25
21338801	2011	6	7	5040	223.97	15.24
21338801	2011	6	7	5050	224.25	15.23
21338801	2011	6	7	5060	224.32	15.23
21338801	2011	6	7	5070	224.32	15.21
21338801	2011	6	7	5080	224.31	15.21
21338801	2011	6	7	5090	224.49	15.21
21338801	2011	6	7	5100	223.96	15.18
21338801	2011	6	7	5110	223.56	15.17
21338801	2011	6	7	5120	223.54	15.17
21338801	2011	6	7	5130	223.75	15.16
21338801	2011	6	7	5140	223.88	15.15
21338801	2011	6	7	5150	224.95	15.15
21338801	2011	6	7	5160	225.38	15.14
21338801	2011	6	7	5170	224.47	15.14
21338801	2011	6	7	5180	223.96	15.14
21338801	2011	6	7	5190	224.01	15.13
21338801	2011	6	7	5200	223.99	15.12
21338801	2011	6	7	5210	224.12	15.12
21338801	2011	6	7	5220	224.17	15.1
21338801	2011	6	7	5230	224.24	15.1
21338801	2011	6	7	5240	223.9	15.1
21338801	2011	6	7	5250	223.98	15.09

21338801	2011	6	7	5260	224.27	15.09
21338801	2011	6	7	5270	224.27	15.09
21338801	2011	6	7	5280	224.38	15.07
21338801	2011	6	7	5290	224.27	15.07
21338801	2011	6	7	5300	224.27	15.07
21338801	2011	6	7	5310	223.49	15.06
21338801	2011	6	7	5320	222.9	15.05
21338801	2011	6	7	5330	223.08	15.05
21338801	2011	6	7	5340	223.01	15.04
21338801	2011	6	7	5350	222.82	15.05
21338801	2011	6	7	5360	222.89	15.04
21338801	2011	6	7	5370	222.64	15.04
21338801	2011	6	7	5380	222.87	15.03
21338801	2011	6	7	5390	222.72	15.03
21338801	2011	6	7	5400	222.46	15.03
21338801	2011	6	7	5410	221.9	15.03
21338801	2011	6	7	5420	221.09	15.02
21338801	2011	6	7	5430	220.34	15.02
21338801	2011	6	7	5440	220.07	15.02
21338801	2011	6	7	5450	220	15.03
21338801	2011	6	7	5460	219.86	15.03
21338801	2011	6	7	5470	219.86	15.03
21338801	2011	6	7	5480	220.05	15.03
21338801	2011	6	7	5490	219.82	15.04
21338801	2011	6	7	5500	219.51	15.04
21338801	2011	6	7	5510	219.4	15.04
21338801	2011	6	7	5520	219.65	15.04
21338801	2011	6	7	5530	219.88	15.05
21338801	2011	6	7	5540	219.84	15.06
21338801	2011	6	7	5550	219.91	15.07
21338801	2011	6	7	5560	219.64	15.07
21338801	2011	6	7	5570	219.22	15.09
21338801	2011	6	7	5580	219.28	15.08
21338801	2011	6	7	5590	219.05	15.1
21338801	2011	6	7	5600	218.71	15.12
21338801	2011	6	7	5610	218.57	15.12
21338801	2011	6	7	5620	218.33	15.14
21338801	2011	6	7	5630	218.21	15.15
21338801	2011	6	7	5640	218.04	15.16
21338801	2011	6	7	5650	217.87	15.16
21338801	2011	6	7	5660	217.73	15.16
21338801	2011	6	7	5670	217.58	15.17
21338801	2011	6	7	5680	217.38	15.18
21338801	2011	6	7	5690	217.17	15.19
21338801	2011	6	7	5700	217.32	15.19
21338801	2011	6	7	5710	217.57	15.22
21338801	2011	6	7	5720	217.7	15.25

21338801	2011	6	7	5730	217.64	15.26
21338801	2011	6	7	5740	217.62	15.28
21338801	2011	6	7	5750	217.64	15.28
21338801	2011	6	7	5760	217.62	15.3
21338801	2011	6	7	5770	217.59	15.31
21338801	2011	6	7	5780	217.43	15.32
21338801	2011	6	7	5790	217.28	15.35
21338801	2011	6	7	5800	217.24	15.35
21338801	2011	6	7	5810	217.35	15.35
21338801	2011	6	7	5820	217.43	15.37
21338801	2011	6	7	5830	217.48	15.38
21338801	2011	6	7	5840	217.2	15.39
21338801	2011	6	7	5850	217.11	15.41
21338801	2011	6	7	5860	217.02	15.42
21338801	2011	6	7	5870	216.73	15.42
21338801	2011	6	7	5880	216.72	15.43
21338801	2011	6	7	5890	216.68	15.43
21338801	2011	6	7	5900	216.68	15.44
21338801	2011	6	7	5910	216.74	15.45
21338801	2011	6	7	5920	216.77	15.46
21338801	2011	6	7	5930	216.9	15.46
21338801	2011	6	7	5940	216.99	15.47
21338801	2011	6	7	5950	216.86	15.47
21338801	2011	6	7	5960	216.82	15.48
21338801	2011	6	7	5970	216.52	15.49
21338801	2011	6	7	5980	216.49	15.5
21338801	2011	6	7	5990	216.77	15.5
21338801	2011	6	7	6000	216.82	15.5
21338801	2011	6	7	6010	216.84	15.52
21338801	2011	6	7	6020	216.8	15.52
21338801	2011	6	7	6030	216.95	15.52
21338801	2011	6	7	6040	216.89	15.53
21338801	2011	6	7	6050	216.55	15.54
21338801	2011	6	7	6060	216.12	15.54
21338801	2011	6	7	6070	215.81	15.54
21338801	2011	6	7	6080	215.65	15.55
21338801	2011	6	7	6090	215.4	15.55
21338801	2011	6	7	6100	215.08	15.56
21338801	2011	6	7	6110	214.8	15.57
21338801	2011	6	7	6120	215.23	15.58
21338801	2011	6	7	6130	215.33	15.59
21338801	2011	6	7	6140	215.28	15.61
21338801	2011	6	7	6150	215.27	15.63
21338801	2011	6	7	6160	214.63	15.64
21338801	2011	6	7	6170	214.51	15.65
21338801	2011	6	7	6180	214.76	15.67
21338801	2011	6	7	6190	214.16	15.68

21338801	2011	6	7	6200	213.81	15.71
21338801	2011	6	7	6210	213.6	15.73
21338801	2011	6	7	6220	213.49	15.75
21338801	2011	6	7	6230	213.15	15.76
21338801	2011	6	7	6240	212.98	15.78
21338801	2011	6	7	6250	212.83	15.8
21338801	2011	6	7	6260	212.85	15.82
21338801	2011	6	7	6270	212.35	15.84
21338801	2011	6	7	6280	212.08	15.85
21338801	2011	6	7	6290	212.25	15.88
21338801	2011	6	7	6300	211.26	15.89
21338801	2011	6	7	6310	211.14	15.94
21338801	2011	6	7	6320	210.91	15.95
21338801	2011	6	7	6330	210.61	15.96
21338801	2011	6	7	6340	210.12	15.98
21338801	2011	6	7	6350	208.95	15.98
21338801	2011	6	7	6360	208.09	16
21338801	2011	6	7	6370	207.96	16.01
21338801	2011	6	7	6380	208.14	16.02
21338801	2011	6	7	6390	208.28	16.02
21338801	2011	6	7	6400	208.08	16.03
21338801	2011	6	7	6410	208.55	16.04
21338801	2011	6	7	6420	208.58	16.04
21338801	2011	6	7	6430	208.43	16.05
21338801	2011	6	7	6440	208.64	16.06
21338801	2011	6	7	6450	208.02	16.07
21338801	2011	6	7	6460	206.06	16.07
21338801	2011	6	7	6470	204.31	16.07
21338801	2011	6	7	6480	202.37	16.08
21338801	2011	6	7	6490	200.67	16.09
21338801	2011	6	7	6500	199.18	16.09
21338801	2011	6	7	6510	197.74	16.09
21338801	2011	6	7	6520	196.49	16.1
21338801	2011	6	7	6530	195.88	16.11
21338801	2011	6	7	6540	195.52	16.12
21338801	2011	6	7	6550	194.9	16.13
21338801	2011	6	7	6560	193.93	16.14
21338801	2011	6	7	6570	193.18	16.14
21338801	2011	6	7	6580	192.66	16.15
21338801	2011	6	7	6590	192.17	16.16
21338801	2011	6	7	6600	191.58	16.17
21338801	2011	6	7	6610	190.56	16.19
21338801	2011	6	7	6620	189.83	16.22
21338801	2011	6	7	6630	189.45	16.25
21338801	2011	6	7	6640	187.88	16.28
21338801	2011	6	7	6650	186.43	16.31
21338801	2011	6	7	6660	185.12	16.34

21338801	2011	6	7	6670	183.4	16.39
21338801	2011	6	7	6680	182.79	16.41
21338801	2011	6	7	6690	181.49	16.42
21338801	2011	6	7	6700	179.64	16.47
21338801	2011	6	7	6710	178.6	16.5
21338801	2011	6	7	6720	177.16	16.53
21338801	2011	6	7	6730	175.42	16.57
21338801	2011	6	7	6740	174.49	16.6
21338801	2011	6	7	6750	173.88	16.63
21338801	2011	6	7	6760	173.06	16.67
21338801	2011	6	7	6770	171.77	16.7
21338801	2011	6	7	6780	170.32	16.72
21338801	2011	6	7	6790	169.54	16.76
21338801	2011	6	7	6800	168.68	16.79
21338801	2011	6	7	6810	167.03	16.84
21338801	2011	6	7	6820	165.82	16.89
21338801	2011	6	7	6830	164.63	16.98
21338801	2011	6	7	6840	162.65	17.03
21338801	2011	6	7	6850	161.76	17.08
21338801	2011	6	7	6860	160.13	17.14
21338801	2011	6	7	6870	158.09	17.19
21338801	2011	6	7	6880	157.24	17.21
21338801	2011	6	7	6890	155.94	17.25
21338801	2011	6	7	6900	154.41	17.31
21338801	2011	6	7	6910	153.38	17.34
21338801	2011	6	7	6920	152.34	17.42
21338801	2011	6	7	6930	151.76	17.44
21338801	2011	6	7	6940	151.27	17.48
21338801	2011	6	7	6950	150.06	17.53
21338801	2011	6	7	6960	149.37	17.59
21338801	2011	6	7	6970	147.67	17.65
21338801	2011	6	7	6980	146.65	17.71
21338801	2011	6	7	6990	144.93	17.78
21338801	2011	6	7	7000	143.14	17.89
21338801	2011	6	7	7010	142.27	17.92
21338801	2011	6	7	7020	142.03	18.04
21338801	2011	6	7	7030	142.21	18.14
21338801	2011	6	7	7040	141.36	18.25
21338801	2011	6	7	7050	139.31	18.28
21338801	2011	6	7	7060	138.29	18.32
21338801	2011	6	7	7070	137.79	18.37
21338801	2011	6	7	7080	135.96	18.44
21338801	2011	6	7	7090	133.95	18.5
21338801	2011	6	7	7100	132.96	18.55
21338801	2011	6	7	7110	132.44	18.6
21338801	2011	6	7	7120	130.82	18.67
21338801	2011	6	7	7130	128.88	18.79

21338801	2011	6	7	7140	126.93	18.84
21338801	2011	6	7	7150	125.01	18.93
21338801	2011	6	7	7160	122.81	19.03
21338801	2011	6	7	7170	120.1	19.1
21338801	2011	6	7	7180	117.69	19.18
21338801	2011	6	7	7190	115.56	19.3
21338801	2011	6	7	7200	113.23	19.46
21338801	2011	6	7	7210	111.35	19.71
21338801	2011	6	7	7220	109.66	19.94
21338801	2011	6	7	7230	107.46	20.1
21338801	2011	6	7	7240	105.34	20.28
21338801	2011	6	7	7250	103.35	20.46
21338801	2011	6	7	7260	101.26	20.64
21338801	2011	6	7	7270	98.95	20.78
21338801	2011	6	7	7280	96.74	21.01
21338801	2011	6	7	7290	94.6	21.25
21338801	2011	6	7	7300	92.33	21.42
21338801	2011	6	7	7310	90.08	21.72
21338801	2011	6	7	7320	87.87	21.91
21338801	2011	6	7	7330	85.73	22.21
21338801	2011	6	7	7340	83.56	22.39
21338801	2011	6	7	7350	81.15	22.5
21338801	2011	6	7	7360	78.75	22.64
21338801	2011	6	7	7370	76.34	22.82
21338801	2011	6	7	7380	74.13	23.01
21338801	2011	6	7	7390	71.61	23.19
21338801	2011	6	7	7400	69.42	23.37
21338801	2011	6	7	7410	67.02	23.54
21338801	2011	6	7	7420	64.36	23.67
21338801	2011	6	7	7430	62.13	23.82
21338801	2011	6	7	7440	59.78	23.94
21338801	2011	6	7	7450	57.98	24.02
21338801	2011	6	7	7460	57.08	24.1
21338801	2011	6	7	7470	56	24.23
21338801	2011	6	7	7480	55.27	24.33
21338801	2011	6	7	7490	54.37	24.42
21338801	2011	6	7	7500	53.45	24.53
21338801	2011	6	7	7510	52.64	24.62
21338801	2011	6	7	7520	51.64	24.76
21338801	2011	6	7	7530	51.05	24.83
21338801	2011	6	7	7540	50.48	24.91
21338801	2011	6	7	7550	50.01	24.98
21338801	2011	6	7	7560	49.58	25.05
21338801	2011	6	7	7570	49.11	25.13
21338801	2011	6	7	7580	48.64	25.19
21338801	2011	6	7	7590	48.22	25.25
21338801	2011	6	7	7600	47.75	25.35

21338801	2011	6	7	7610	47.39	25.4
21338801	2011	6	7	7620	47.11	25.46
21338801	2011	6	7	7630	46.47	25.57
21338801	2011	6	7	7640	46.08	25.62
21338801	2011	6	7	7650	45.74	25.66
21338801	2011	6	7	7660	45.19	25.72
21338801	2011	6	7	7670	44.55	25.78
21338801	2011	6	7	7680	44.01	25.82
21338801	2011	6	7	7690	43.15	25.88
21338801	2011	6	7	7700	40.87	25.93
21338801	2011	6	7	7710	38.23	26
21338801	2011	6	7	7720	35.46	26.06
21338801	2011	6	7	7730	32.48	26.12
21338801	2011	6	7	7740	29.49	26.2
21338801	2011	6	7	7750	26.62	26.26
21338801	2011	6	7	7760	23.82	26.35
21338801	2011	6	7	7770	20.81	26.47
21338801	2011	6	7	7780	17.6	26.59
21338801	2011	6	7	7790	14.47	26.7
21338801	2011	6	7	7800	11.52	26.82
21338801	2011	6	7	7810	9.49	26.93
21338801	2011	6	7	7820	8.28	27.06
21338801	2011	6	7	7830	6.18	27.2
21338801	2011	6	7	7840	4.22	27.31
21338801	2011	6	7	7850	2.77	27.56
21338801	2011	6	7	7860	2.41	27.61
21338801	2011	6	7	7870	2.03	27.67
21338801	2011	6	7	7880	2.23	27.75
21338801	2011	6	7	7890	1.79	27.87
21338801	2011	6	7	7900	0.56	28.05
21338801	2011	6	7	7910	0.58	28.07
21338801	2011	6	7	7920	0.58	28.1
21338801	2011	6	7	7930	0.57	28.14
21338801	2011	6	7	7940	0.56	28.25
21338801	2011	6	7	7950	0.58	28.37
21338801	2011	6	7	7960	0.59	28.44
21338801	2011	6	7	7970	0.58	28.5
21338801	2011	6	7	7980	0.57	28.57
21338801	2011	6	7	7990	0.59	28.67
21338801	2011	6	7	8000	0.58	28.72
21338801	2011	6	7	8010	0.49	28.77
21338801	2011	6	7	8020	0.47	28.85
21338801	2011	6	7	8030	0.46	28.89
21338801	2011	6	7	8040	0.49	28.92
21418931	2011	6	8	0	1.09	29.66
21418931	2011	6	8	10	1.51	29.64
21418931	2011	6	8	20	1.67	29.64

21418931	2011	6	8	30	1.63	29.64
21418931	2011	6	8	40	1.41	29.64
21418931	2011	6	8	50	1.62	29.64
21418931	2011	6	8	60	1.83	29.64
21418931	2011	6	8	70	2.14	29.62
21418931	2011	6	8	80	2.78	29.61
21418931	2011	6	8	90	3.19	29.6
21418931	2011	6	8	100	3.66	29.59
21418931	2011	6	8	110	5.31	29.56
21418931	2011	6	8	120	7.23	29.51
21418931	2011	6	8	130	9.44	29.47
21418931	2011	6	8	140	11.86	29.44
21418931	2011	6	8	150	14.29	29.4
21418931	2011	6	8	160	16.78	29.37
21418931	2011	6	8	170	19.81	29.31
21418931	2011	6	8	180	22.88	29.27
21418931	2011	6	8	190	25.65	29.23
21418931	2011	6	8	200	28.18	29.16
21418931	2011	6	8	210	30.59	29.07
21418931	2011	6	8	220	32.92	28.96
21418931	2011	6	8	230	35.18	28.89
21418931	2011	6	8	240	37.74	28.8
21418931	2011	6	8	250	40.24	28.75
21418931	2011	6	8	260	42.45	28.64
21418931	2011	6	8	270	43.74	28.53
21418931	2011	6	8	280	45.09	28.28
21418931	2011	6	8	290	46.67	28.24
21418931	2011	6	8	300	48.52	28.17
21418931	2011	6	8	310	50.4	28.1
21418931	2011	6	8	320	52.35	27.97
21418931	2011	6	8	330	53.86	27.87
21418931	2011	6	8	340	55.72	27.78
21418931	2011	6	8	350	58.05	27.65
21418931	2011	6	8	360	60.17	27.57
21418931	2011	6	8	370	61.87	27.48
21418931	2011	6	8	380	63.42	27.35
21418931	2011	6	8	390	65.08	27.32
21418931	2011	6	8	400	66.8	27.24
21418931	2011	6	8	410	68.48	27.1
21418931	2011	6	8	420	69.92	27.01
21418931	2011	6	8	430	71.37	26.97
21418931	2011	6	8	440	73.52	26.96
21418931	2011	6	8	450	76.5	26.94
21418931	2011	6	8	460	79.93	26.88
21418931	2011	6	8	470	83.09	26.79
21418931	2011	6	8	480	85.45	26.71
21418931	2011	6	8	490	87.6	26.62

21418931	2011	6	8	500	90.36	26.53
21418931	2011	6	8	510	93.68	26.49
21418931	2011	6	8	520	97.12	26.39
21418931	2011	6	8	530	100.39	26.28
21418931	2011	6	8	540	103.44	26.09
21418931	2011	6	8	550	106.24	25.89
21418931	2011	6	8	560	108	25.57
21418931	2011	6	8	570	109.36	25.35
21418931	2011	6	8	580	110.62	25.22
21418931	2011	6	8	590	112.08	25.07
21418931	2011	6	8	600	113.95	24.85
21418931	2011	6	8	610	115.91	24.71
21418931	2011	6	8	620	117.53	24.46
21418931	2011	6	8	630	119.1	24.38
21418931	2011	6	8	640	121.46	24.28
21418931	2011	6	8	650	123.44	24
21418931	2011	6	8	660	125.01	23.79
21418931	2011	6	8	670	126.92	23.75
21418931	2011	6	8	680	129.17	23.66
21418931	2011	6	8	690	131.13	23.5
21418931	2011	6	8	700	132.87	23.34
21418931	2011	6	8	710	135.15	23.28
21418931	2011	6	8	720	138.04	23.14
21418931	2011	6	8	730	140.72	23
21418931	2011	6	8	740	143.12	22.86
21418931	2011	6	8	750	145.85	22.76
21418931	2011	6	8	760	148.89	22.6
21418931	2011	6	8	770	152.01	22.41
21418931	2011	6	8	780	155.05	22.25
21418931	2011	6	8	790	157.59	22.07
21418931	2011	6	8	800	160.33	21.81
21418931	2011	6	8	810	162.97	21.59
21418931	2011	6	8	820	165.47	21.44
21418931	2011	6	8	830	167.68	21.28
21418931	2011	6	8	840	169.74	20.95
21418931	2011	6	8	850	170.99	20.81
21418931	2011	6	8	860	171.3	20.79
21418931	2011	6	8	870	171.47	20.71
21418931	2011	6	8	880	172.29	20.28
21418931	2011	6	8	890	173	20.28
21418931	2011	6	8	900	174.62	19.82
21418931	2011	6	8	910	176.06	19.54
21418931	2011	6	8	920	176.51	19.67
21418931	2011	6	8	930	176.67	19.71
21418931	2011	6	8	940	176.58	19.71
21418931	2011	6	8	950	178.01	19.46
21418931	2011	6	8	960	179.63	19.19

21418931	2011	6	8	970	181.18	19.04
21418931	2011	6	8	980	182.84	19.04
21418931	2011	6	8	990	184.68	18.89
21418931	2011	6	8	1000	186.82	18.89
21418931	2011	6	8	1010	188.98	18.8
21418931	2011	6	8	1020	190.11	18.72
21418931	2011	6	8	1030	191.42	18.64
21418931	2011	6	8	1040	192.27	18.63
21418931	2011	6	8	1050	192.49	18.59
21418931	2011	6	8	1060	192.18	18.53
21418931	2011	6	8	1070	192.12	18.42
21418931	2011	6	8	1080	192.69	18.32
21418931	2011	6	8	1090	193.37	18.28
21418931	2011	6	8	1100	193.35	18.25
21418931	2011	6	8	1110	193.04	18.21
21418931	2011	6	8	1120	192.53	18.16
21418931	2011	6	8	1130	191.74	18.1
21418931	2011	6	8	1140	190.93	18.04
21418931	2011	6	8	1150	190.78	17.98
21418931	2011	6	8	1160	192.15	17.83
21418931	2011	6	8	1170	193.12	17.81
21418931	2011	6	8	1180	193.16	17.76
21418931	2011	6	8	1190	193.15	17.72
21418931	2011	6	8	1200	193.44	17.65
21418931	2011	6	8	1210	195.06	17.58
21418931	2011	6	8	1220	196.44	17.54
21418931	2011	6	8	1230	197.57	17.43
21418931	2011	6	8	1240	197.38	17.4
21418931	2011	6	8	1250	197.07	17.36
21418931	2011	6	8	1260	196.94	17.28
21418931	2011	6	8	1270	196.81	17.17
21418931	2011	6	8	1280	196.9	17.09
21418931	2011	6	8	1290	196.75	16.92
21418931	2011	6	8	1300	196.55	16.9
21418931	2011	6	8	1310	197.21	16.87
21418931	2011	6	8	1320	197.68	16.85
21418931	2011	6	8	1330	197.53	16.85
21418931	2011	6	8	1340	197.2	16.84
21418931	2011	6	8	1350	196.74	16.82
21418931	2011	6	8	1360	196.16	16.77
21418931	2011	6	8	1370	195.62	16.73
21418931	2011	6	8	1380	196.19	16.71
21418931	2011	6	8	1390	196.76	16.67
21418931	2011	6	8	1400	196.86	16.63
21418931	2011	6	8	1410	198.1	16.6
21418931	2011	6	8	1420	198.87	16.57
21418931	2011	6	8	1430	198.96	16.57

21418931	2011	6	8	1440	199.78	16.54
21418931	2011	6	8	1450	199.87	16.51
21418931	2011	6	8	1460	199.88	16.5
21418931	2011	6	8	1470	199.98	16.48
21418931	2011	6	8	1480	199.95	16.47
21418931	2011	6	8	1490	199.57	16.43
21418931	2011	6	8	1500	199.13	16.42
21418931	2011	6	8	1510	198.83	16.41
21418931	2011	6	8	1520	198.46	16.4
21418931	2011	6	8	1530	199.1	16.38
21418931	2011	6	8	1540	199.01	16.36
21418931	2011	6	8	1550	198.02	16.34
21418931	2011	6	8	1560	197.61	16.33
21418931	2011	6	8	1570	198.05	16.32
21418931	2011	6	8	1580	198.55	16.32
21418931	2011	6	8	1590	199.16	16.32
21418931	2011	6	8	1600	199.55	16.31
21418931	2011	6	8	1610	199.79	16.31
21418931	2011	6	8	1620	199.65	16.3
21418931	2011	6	8	1630	199.92	16.28
21418931	2011	6	8	1640	199.87	16.29
21418931	2011	6	8	1650	199.85	16.28
21418931	2011	6	8	1660	199.76	16.28
21418931	2011	6	8	1670	199.62	16.28
21418931	2011	6	8	1680	199.91	16.28
21418931	2011	6	8	1690	199.99	16.26
21418931	2011	6	8	1700	199.84	16.26
21418931	2011	6	8	1710	199.4	16.25
21418931	2011	6	8	1720	199.53	16.25
21418931	2011	6	8	1730	200.09	16.25
21418931	2011	6	8	1740	200.3	16.24
21418931	2011	6	8	1750	200.04	16.23
21418931	2011	6	8	1760	200.17	16.22
21418931	2011	6	8	1770	200.46	16.21
21418931	2011	6	8	1780	200.66	16.21
21418931	2011	6	8	1790	200.59	16.2
21418931	2011	6	8	1800	200.37	16.19
21418931	2011	6	8	1810	200.27	16.18
21418931	2011	6	8	1820	200.38	16.17
21418931	2011	6	8	1830	199.67	16.15
21418931	2011	6	8	1840	198.96	16.14
21418931	2011	6	8	1850	199.13	16.14
21418931	2011	6	8	1860	199.7	16.14
21418931	2011	6	8	1870	201.04	16.14
21418931	2011	6	8	1880	201.38	16.13
21418931	2011	6	8	1890	201.79	16.13
21418931	2011	6	8	1900	202.42	16.13

21418931	2011	6	8	1910	203.2	16.12
21418931	2011	6	8	1920	203.87	16.1
21418931	2011	6	8	1930	204.89	16.09
21418931	2011	6	8	1940	205.48	16.06
21418931	2011	6	8	1950	205.72	16.05
21418931	2011	6	8	1960	206.21	16.01
21418931	2011	6	8	1970	206.52	16
21418931	2011	6	8	1980	206.64	15.99
21418931	2011	6	8	1990	206.54	15.98
21418931	2011	6	8	2000	206.58	15.96
21418931	2011	6	8	2010	206.29	15.95
21418931	2011	6	8	2020	205.68	15.89
21418931	2011	6	8	2030	204.94	15.89
21418931	2011	6	8	2040	205.13	15.88
21418931	2011	6	8	2050	205.83	15.85
21418931	2011	6	8	2060	206.05	15.85
21418931	2011	6	8	2070	206.03	15.85
21418931	2011	6	8	2080	206.22	15.82
21418931	2011	6	8	2090	207	15.82
21418931	2011	6	8	2100	207.38	15.81
21418931	2011	6	8	2110	208.36	15.8
21418931	2011	6	8	2120	208.89	15.78
21418931	2011	6	8	2130	208.66	15.78
21418931	2011	6	8	2140	208.55	15.78
21418931	2011	6	8	2150	208.3	15.76
21418931	2011	6	8	2160	207.74	15.75
21418931	2011	6	8	2170	207.84	15.75
21418931	2011	6	8	2180	207.8	15.75
21418931	2011	6	8	2190	208.36	15.75
21418931	2011	6	8	2200	208.43	15.75
21418931	2011	6	8	2210	208.03	15.75
21418931	2011	6	8	2220	208	15.75
21418931	2011	6	8	2230	208.04	15.75
21418931	2011	6	8	2240	208.15	15.75
21418931	2011	6	8	2250	207.98	15.75
21418931	2011	6	8	2260	207.55	15.74
21418931	2011	6	8	2270	207.9	15.75
21418931	2011	6	8	2280	208.06	15.75
21418931	2011	6	8	2290	208.25	15.75
21418931	2011	6	8	2300	208.24	15.75
21418931	2011	6	8	2310	208.16	15.75
21418931	2011	6	8	2320	208.17	15.75
21418931	2011	6	8	2330	207.79	15.75
21418931	2011	6	8	2340	207.07	15.75
21418931	2011	6	8	2350	206.67	15.75
21418931	2011	6	8	2360	206.93	15.77
21418931	2011	6	8	2370	208.12	15.76

21418931	2011	6	8	2380	208.38	15.75
21418931	2011	6	8	2390	208.6	15.75
21418931	2011	6	8	2400	208.98	15.75
21418931	2011	6	8	2410	208.98	15.74
21418931	2011	6	8	2420	208.91	15.74
21418931	2011	6	8	2430	209.9	15.74
21418931	2011	6	8	2440	210.12	15.71
21418931	2011	6	8	2450	210.42	15.71
21418931	2011	6	8	2460	210.24	15.69
21418931	2011	6	8	2470	210.61	15.67
21418931	2011	6	8	2480	210.65	15.65
21418931	2011	6	8	2490	210.76	15.65
21418931	2011	6	8	2500	210.48	15.64
21418931	2011	6	8	2510	209.95	15.63
21418931	2011	6	8	2520	210.29	15.62
21418931	2011	6	8	2530	211.96	15.6
21418931	2011	6	8	2540	212.56	15.6
21418931	2011	6	8	2550	212.53	15.58
21418931	2011	6	8	2560	213.82	15.58
21418931	2011	6	8	2570	214.41	15.58
21418931	2011	6	8	2580	214.42	15.57
21418931	2011	6	8	2590	214.49	15.57
21418931	2011	6	8	2600	214.27	15.57
21418931	2011	6	8	2610	213.96	15.57
21418931	2011	6	8	2620	214.3	15.57
21418931	2011	6	8	2630	214.69	15.57
21418931	2011	6	8	2640	215.33	15.59
21418931	2011	6	8	2650	216.85	15.6
21418931	2011	6	8	2660	217.62	15.6
21418931	2011	6	8	2670	218.15	15.61
21418931	2011	6	8	2680	218.17	15.6
21418931	2011	6	8	2690	218.05	15.6
21418931	2011	6	8	2700	217.54	15.6
21418931	2011	6	8	2710	216.95	15.6
21418931	2011	6	8	2720	216.33	15.58
21418931	2011	6	8	2730	216.03	15.58
21418931	2011	6	8	2740	216.09	15.58
21418931	2011	6	8	2750	215.98	15.57
21418931	2011	6	8	2760	216	15.57
21418931	2011	6	8	2770	216.16	15.57
21418931	2011	6	8	2780	216.1	15.57
21418931	2011	6	8	2790	216.14	15.58
21418931	2011	6	8	2800	216.17	15.57
21418931	2011	6	8	2810	216.11	15.57
21418931	2011	6	8	2820	216.15	15.57
21418931	2011	6	8	2830	215.91	15.57
21418931	2011	6	8	2840	215.52	15.57

21418931	2011	6	8	2850	215.79	15.57
21418931	2011	6	8	2860	216.44	15.59
21418931	2011	6	8	2870	216.74	15.58
21418931	2011	6	8	2880	216.78	15.59
21418931	2011	6	8	2890	216.65	15.58
21418931	2011	6	8	2900	216.65	15.6
21418931	2011	6	8	2910	216.8	15.59
21418931	2011	6	8	2920	216.61	15.59
21418931	2011	6	8	2930	216.64	15.6
21418931	2011	6	8	2940	216.78	15.59
21418931	2011	6	8	2950	216.8	15.6
21418931	2011	6	8	2960	216.58	15.6
21418931	2011	6	8	2970	216.6	15.6
21418931	2011	6	8	2980	216.86	15.6
21418931	2011	6	8	2990	217.08	15.59
21418931	2011	6	8	3000	217.19	15.6
21418931	2011	6	8	3010	216.83	15.59
21418931	2011	6	8	3020	216.66	15.58
21418931	2011	6	8	3030	216.46	15.58
21418931	2011	6	8	3040	216.1	15.58
21418931	2011	6	8	3050	215.76	15.58
21418931	2011	6	8	3060	215.31	15.58
21418931	2011	6	8	3070	215.33	15.57
21418931	2011	6	8	3080	215.54	15.58
21418931	2011	6	8	3090	215.3	15.58
21418931	2011	6	8	3100	216.22	15.59
21418931	2011	6	8	3110	216.85	15.59
21418931	2011	6	8	3120	216.82	15.59
21418931	2011	6	8	3130	216.69	15.6
21418931	2011	6	8	3140	216.71	15.6
21418931	2011	6	8	3150	216.53	15.6
21418931	2011	6	8	3160	216.28	15.6
21418931	2011	6	8	3170	215.88	15.6
21418931	2011	6	8	3180	215.3	15.59
21418931	2011	6	8	3190	215.7	15.58
21418931	2011	6	8	3200	216.51	15.59
21418931	2011	6	8	3210	216.65	15.58
21418931	2011	6	8	3220	216.25	15.58
21418931	2011	6	8	3230	215.85	15.58
21418931	2011	6	8	3240	215.8	15.57
21418931	2011	6	8	3250	217.16	15.57
21418931	2011	6	8	3260	218.18	15.57
21418931	2011	6	8	3270	218.53	15.56
21418931	2011	6	8	3280	218.56	15.56
21418931	2011	6	8	3290	218.67	15.55
21418931	2011	6	8	3300	218.67	15.54
21418931	2011	6	8	3310	218.68	15.53

21418931	2011	6	8	3320	218.67	15.54
21418931	2011	6	8	3330	218.82	15.53
21418931	2011	6	8	3340	218.7	15.52
21418931	2011	6	8	3350	218.72	15.52
21418931	2011	6	8	3360	218.92	15.51
21418931	2011	6	8	3370	218.93	15.5
21418931	2011	6	8	3380	219.02	15.5
21418931	2011	6	8	3390	219.35	15.5
21418931	2011	6	8	3400	219.11	15.49
21418931	2011	6	8	3410	218.67	15.49
21418931	2011	6	8	3420	218.8	15.49
21418931	2011	6	8	3430	218.63	15.48
21418931	2011	6	8	3440	218.46	15.48
21418931	2011	6	8	3450	218.54	15.48
21418931	2011	6	8	3460	218.88	15.47
21418931	2011	6	8	3470	218.83	15.48
21418931	2011	6	8	3480	218.92	15.48
21418931	2011	6	8	3490	218.79	15.48
21418931	2011	6	8	3500	218.82	15.47
21418931	2011	6	8	3510	218.86	15.47
21418931	2011	6	8	3520	218.59	15.47
21418931	2011	6	8	3530	218.88	15.48
21418931	2011	6	8	3540	219.07	15.47
21418931	2011	6	8	3550	218.99	15.47
21418931	2011	6	8	3560	219.03	15.47
21418931	2011	6	8	3570	219.12	15.47
21418931	2011	6	8	3580	218.88	15.47
21418931	2011	6	8	3590	218.98	15.47
21418931	2011	6	8	3600	218.81	15.48
21418931	2011	6	8	3610	218.6	15.47
21418931	2011	6	8	3620	218.15	15.47
21418931	2011	6	8	3630	217.85	15.47
21418931	2011	6	8	3640	218.8	15.47
21418931	2011	6	8	3650	219.29	15.47
21418931	2011	6	8	3660	219.28	15.47
21418931	2011	6	8	3670	219.23	15.47
21418931	2011	6	8	3680	219.2	15.47
21418931	2011	6	8	3690	219.24	15.47
21418931	2011	6	8	3700	219.14	15.46
21418931	2011	6	8	3710	218.82	15.46
21418931	2011	6	8	3720	218.57	15.45
21418931	2011	6	8	3730	218.25	15.46
21418931	2011	6	8	3740	218.21	15.45
21418931	2011	6	8	3750	217.56	15.45
21418931	2011	6	8	3760	216.73	15.44
21418931	2011	6	8	3770	216.83	15.44
21418931	2011	6	8	3780	217.13	15.44

21418931	2011	6	8	3790	216.53	15.44
21418931	2011	6	8	3800	217.66	15.43
21418931	2011	6	8	3810	217.91	15.43
21418931	2011	6	8	3820	217.36	15.44
21418931	2011	6	8	3830	217.08	15.43
21418931	2011	6	8	3840	217.2	15.43
21418931	2011	6	8	3850	217.83	15.42
21418931	2011	6	8	3860	218.1	15.42
21418931	2011	6	8	3870	218.31	15.42
21418931	2011	6	8	3880	218.34	15.42
21418931	2011	6	8	3890	218.23	15.42
21418931	2011	6	8	3900	218.25	15.42
21418931	2011	6	8	3910	218.18	15.42
21418931	2011	6	8	3920	218.01	15.41
21418931	2011	6	8	3930	218.3	15.42
21418931	2011	6	8	3940	218.27	15.4
21418931	2011	6	8	3950	217.85	15.41
21418931	2011	6	8	3960	217.6	15.4
21418931	2011	6	8	3970	218.04	15.4
21418931	2011	6	8	3980	218.31	15.4
21418931	2011	6	8	3990	218.2	15.4
21418931	2011	6	8	4000	217.91	15.41
21418931	2011	6	8	4010	218.17	15.39
21418931	2011	6	8	4020	218.27	15.39
21418931	2011	6	8	4030	217.96	15.39
21418931	2011	6	8	4040	217.49	15.39
21418931	2011	6	8	4050	216.68	15.39
21418931	2011	6	8	4060	216.34	15.39
21418931	2011	6	8	4070	216.8	15.39
21418931	2011	6	8	4080	217.07	15.39
21418931	2011	6	8	4090	216.92	15.39
21418931	2011	6	8	4100	216.28	15.39
21418931	2011	6	8	4110	216.59	15.39
21418931	2011	6	8	4120	216.68	15.39
21418931	2011	6	8	4130	216.26	15.39
21418931	2011	6	8	4140	216.42	15.38
21418931	2011	6	8	4150	216.73	15.39
21418931	2011	6	8	4160	216.21	15.38
21418931	2011	6	8	4170	215.56	15.39
21418931	2011	6	8	4180	215.54	15.38
21418931	2011	6	8	4190	215.66	15.38
21418931	2011	6	8	4200	215.4	15.38
21418931	2011	6	8	4210	215.16	15.38
21418931	2011	6	8	4220	215.27	15.38
21418931	2011	6	8	4230	215.43	15.39
21418931	2011	6	8	4240	215.74	15.39
21418931	2011	6	8	4250	216.25	15.38

21418931	2011	6	8	4260	216.57	15.38
21418931	2011	6	8	4270	216.49	15.39
21418931	2011	6	8	4280	216.15	15.39
21418931	2011	6	8	4290	216.06	15.39
21418931	2011	6	8	4300	215.44	15.38
21418931	2011	6	8	4310	215.05	15.39
21418931	2011	6	8	4320	215.04	15.39
21418931	2011	6	8	4330	215.34	15.38
21418931	2011	6	8	4340	215.58	15.38
21418931	2011	6	8	4350	215.78	15.39
21418931	2011	6	8	4360	215.88	15.38
21418931	2011	6	8	4370	215.49	15.38
21418931	2011	6	8	4380	215.3	15.38
21418931	2011	6	8	4390	214.9	15.38
21418931	2011	6	8	4400	214.81	15.38
21418931	2011	6	8	4410	214.37	15.39
21418931	2011	6	8	4420	213.68	15.38
21418931	2011	6	8	4430	212.92	15.38
21418931	2011	6	8	4440	213.54	15.38
21418931	2011	6	8	4450	214.09	15.39
21418931	2011	6	8	4460	215.4	15.38
21418931	2011	6	8	4470	215.95	15.39
21418931	2011	6	8	4480	215.89	15.39
21418931	2011	6	8	4490	215.72	15.38
21418931	2011	6	8	4500	215.3	15.39
21418931	2011	6	8	4510	216.01	15.39
21418931	2011	6	8	4520	215.39	15.39
21418931	2011	6	8	4530	215.01	15.39
21418931	2011	6	8	4540	214.43	15.39
21418931	2011	6	8	4550	213.87	15.38
21418931	2011	6	8	4560	213.51	15.38
21418931	2011	6	8	4570	214.52	15.39
21418931	2011	6	8	4580	215.56	15.39
21418931	2011	6	8	4590	215.95	15.39
21418931	2011	6	8	4600	216.59	15.38
21418931	2011	6	8	4610	217.27	15.39
21418931	2011	6	8	4620	217.77	15.38
21418931	2011	6	8	4630	217.57	15.39
21418931	2011	6	8	4640	217.57	15.39
21418931	2011	6	8	4650	218.36	15.38
21418931	2011	6	8	4660	218.64	15.39
21418931	2011	6	8	4670	218.76	15.39
21418931	2011	6	8	4680	219.52	15.39
21418931	2011	6	8	4690	220.67	15.39
21418931	2011	6	8	4700	220.98	15.38
21418931	2011	6	8	4710	220.75	15.38
21418931	2011	6	8	4720	220.44	15.38

21418931	2011	6	8	4730	220.26	15.38
21418931	2011	6	8	4740	220.19	15.38
21418931	2011	6	8	4750	220.11	15.37
21418931	2011	6	8	4760	221.35	15.38
21418931	2011	6	8	4770	221.59	15.38
21418931	2011	6	8	4780	221.7	15.39
21418931	2011	6	8	4790	222.48	15.38
21418931	2011	6	8	4800	222.47	15.38
21418931	2011	6	8	4810	222.44	15.38
21418931	2011	6	8	4820	222.43	15.37
21418931	2011	6	8	4830	222.08	15.36
21418931	2011	6	8	4840	221.82	15.35
21418931	2011	6	8	4850	221.8	15.35
21418931	2011	6	8	4860	222.9	15.34
21418931	2011	6	8	4870	223.5	15.35
21418931	2011	6	8	4880	223.2	15.34
21418931	2011	6	8	4890	222.87	15.34
21418931	2011	6	8	4900	222.58	15.33
21418931	2011	6	8	4910	222.97	15.32
21418931	2011	6	8	4920	223.2	15.3
21418931	2011	6	8	4930	223.18	15.3
21418931	2011	6	8	4940	223.54	15.28
21418931	2011	6	8	4950	224.43	15.25
21418931	2011	6	8	4960	224.82	15.25
21418931	2011	6	8	4970	224.52	15.23
21418931	2011	6	8	4980	225.68	15.21
21418931	2011	6	8	4990	226.29	15.2
21418931	2011	6	8	5000	226.71	15.19
21418931	2011	6	8	5010	227.21	15.17
21418931	2011	6	8	5020	227.09	15.14
21418931	2011	6	8	5030	226.71	15.13
21418931	2011	6	8	5040	226.12	15.11
21418931	2011	6	8	5050	225.37	15.1
21418931	2011	6	8	5060	225.3	15.08
21418931	2011	6	8	5070	226.82	15.05
21418931	2011	6	8	5080	227.35	15.04
21418931	2011	6	8	5090	227.46	15.03
21418931	2011	6	8	5100	228.23	15.01
21418931	2011	6	8	5110	228.83	14.98
21418931	2011	6	8	5120	229.41	14.94
21418931	2011	6	8	5130	230.05	14.91
21418931	2011	6	8	5140	230.16	14.84
21418931	2011	6	8	5150	230.79	14.82
21418931	2011	6	8	5160	231.06	14.79
21418931	2011	6	8	5170	231.05	14.76
21418931	2011	6	8	5180	230.81	14.72
21418931	2011	6	8	5190	230.32	14.69

21418931	2011	6	8	5200	230.05	14.68
21418931	2011	6	8	5210	229.97	14.67
21418931	2011	6	8	5220	230.19	14.66
21418931	2011	6	8	5230	230.61	14.64
21418931	2011	6	8	5240	230.77	14.61
21418931	2011	6	8	5250	231.07	14.6
21418931	2011	6	8	5260	232.02	14.59
21418931	2011	6	8	5270	232.77	14.57
21418931	2011	6	8	5280	233.54	14.57
21418931	2011	6	8	5290	233.43	14.51
21418931	2011	6	8	5300	233.59	14.5
21418931	2011	6	8	5310	233.09	14.48
21418931	2011	6	8	5320	232.4	14.47
21418931	2011	6	8	5330	232.31	14.47
21418931	2011	6	8	5340	232.23	14.46
21418931	2011	6	8	5350	231.42	14.43
21418931	2011	6	8	5360	231.98	14.43
21418931	2011	6	8	5370	231.99	14.42
21418931	2011	6	8	5380	231.8	14.42
21418931	2011	6	8	5390	231.83	14.41
21418931	2011	6	8	5400	231.79	14.4
21418931	2011	6	8	5410	231.78	14.4
21418931	2011	6	8	5420	232.01	14.39
21418931	2011	6	8	5430	231.89	14.39
21418931	2011	6	8	5440	232.19	14.38
21418931	2011	6	8	5450	232.17	14.37
21418931	2011	6	8	5460	232.08	14.37
21418931	2011	6	8	5470	231.77	14.35
21418931	2011	6	8	5480	231.47	14.35
21418931	2011	6	8	5490	231.1	14.35
21418931	2011	6	8	5500	230.97	14.35
21418931	2011	6	8	5510	230.62	14.35
21418931	2011	6	8	5520	230.38	14.35
21418931	2011	6	8	5530	231.23	14.37
21418931	2011	6	8	5540	232.34	14.38
21418931	2011	6	8	5550	232.65	14.39
21418931	2011	6	8	5560	232.27	14.39
21418931	2011	6	8	5570	231.84	14.39
21418931	2011	6	8	5580	230.81	14.4
21418931	2011	6	8	5590	229.71	14.41
21418931	2011	6	8	5600	228.4	14.41
21418931	2011	6	8	5610	227.72	14.42
21418931	2011	6	8	5620	227.64	14.45
21418931	2011	6	8	5630	226.94	14.47
21418931	2011	6	8	5640	225.81	14.49
21418931	2011	6	8	5650	224.69	14.51
21418931	2011	6	8	5660	223.35	14.53

21418931	2011	6	8	5670	222.35	14.54
21418931	2011	6	8	5680	222.95	14.58
21418931	2011	6	8	5690	223.35	14.6
21418931	2011	6	8	5700	223.53	14.61
21418931	2011	6	8	5710	223.48	14.63
21418931	2011	6	8	5720	223.43	14.64
21418931	2011	6	8	5730	223.56	14.67
21418931	2011	6	8	5740	223.44	14.69
21418931	2011	6	8	5750	223.41	14.71
21418931	2011	6	8	5760	223.48	14.73
21418931	2011	6	8	5770	223.13	14.75
21418931	2011	6	8	5780	221.23	14.77
21418931	2011	6	8	5790	219.88	14.79
21418931	2011	6	8	5800	219.02	14.82
21418931	2011	6	8	5810	217.57	14.84
21418931	2011	6	8	5820	216.14	14.92
21418931	2011	6	8	5830	214.69	14.94
21418931	2011	6	8	5840	213.08	14.96
21418931	2011	6	8	5850	211.27	15
21418931	2011	6	8	5860	209.37	15.01
21418931	2011	6	8	5870	207.32	15.05
21418931	2011	6	8	5880	206.36	15.1
21418931	2011	6	8	5890	205.29	15.13
21418931	2011	6	8	5900	203.17	15.19
21418931	2011	6	8	5910	201.69	15.24
21418931	2011	6	8	5920	200.3	15.31
21418931	2011	6	8	5930	197.9	15.37
21418931	2011	6	8	5940	196.99	15.39
21418931	2011	6	8	5950	197.02	15.57
21418931	2011	6	8	5960	195.23	15.6
21418931	2011	6	8	5970	194.32	15.64
21418931	2011	6	8	5980	194.61	15.71
21418931	2011	6	8	5990	193.96	15.79
21418931	2011	6	8	6000	193.47	15.85
21418931	2011	6	8	6010	192.65	15.97
21418931	2011	6	8	6020	191.22	16.01
21418931	2011	6	8	6030	190.01	16.01
21418931	2011	6	8	6040	188.42	16.07
21418931	2011	6	8	6050	188.13	16.12
21418931	2011	6	8	6060	188.82	16.21
21418931	2011	6	8	6070	188.77	16.22
21418931	2011	6	8	6080	188.67	16.25
21418931	2011	6	8	6090	188.15	16.29
21418931	2011	6	8	6100	186.11	16.35
21418931	2011	6	8	6110	183.83	16.41
21418931	2011	6	8	6120	182.62	16.49
21418931	2011	6	8	6130	180.96	16.53

21418931	2011	6	8	6140	179.18	16.57
21418931	2011	6	8	6150	177.81	16.6
21418931	2011	6	8	6160	176.41	16.64
21418931	2011	6	8	6170	174.08	16.74
21418931	2011	6	8	6180	171.84	16.78
21418931	2011	6	8	6190	169.26	16.87
21418931	2011	6	8	6200	166.28	16.92
21418931	2011	6	8	6210	163.37	16.99
21418931	2011	6	8	6220	160.92	17.03
21418931	2011	6	8	6230	158.23	17.1
21418931	2011	6	8	6240	155.44	17.25
21418931	2011	6	8	6250	152.98	17.32
21418931	2011	6	8	6260	150.65	17.48
21418931	2011	6	8	6270	149.17	17.57
21418931	2011	6	8	6280	147.06	17.75
21418931	2011	6	8	6290	144.6	17.85
21418931	2011	6	8	6300	142.07	17.99
21418931	2011	6	8	6310	139.48	18.09
21418931	2011	6	8	6320	136.85	18.19
21418931	2011	6	8	6330	134	18.31
21418931	2011	6	8	6340	131.37	18.44
21418931	2011	6	8	6350	128.77	18.57
21418931	2011	6	8	6360	125.95	18.72
21418931	2011	6	8	6370	123.54	18.89
21418931	2011	6	8	6380	121.19	19.04
21418931	2011	6	8	6390	118.62	19.18
21418931	2011	6	8	6400	115.94	19.32
21418931	2011	6	8	6410	113.14	19.46
21418931	2011	6	8	6420	110.33	19.61
21418931	2011	6	8	6430	107.55	19.74
21418931	2011	6	8	6440	105.24	19.91
21418931	2011	6	8	6450	103.24	20.04
21418931	2011	6	8	6460	101.22	20.17
21418931	2011	6	8	6470	99.19	20.37
21418931	2011	6	8	6480	97.22	20.47
21418931	2011	6	8	6490	95.17	20.64
21418931	2011	6	8	6500	93.17	20.83
21418931	2011	6	8	6510	91.25	20.99
21418931	2011	6	8	6520	89.48	21.23
21418931	2011	6	8	6530	87.39	21.35
21418931	2011	6	8	6540	85.51	21.63
21418931	2011	6	8	6550	83.43	22.06
21418931	2011	6	8	6560	81.11	22.21
21418931	2011	6	8	6570	78.99	22.46
21418931	2011	6	8	6580	76.69	22.53
21418931	2011	6	8	6590	74.34	22.58
21418931	2011	6	8	6600	72.26	22.73

21418931	2011	6	8	6610	69.89	22.88
21418931	2011	6	8	6620	67.38	23.07
21418931	2011	6	8	6630	64.97	23.26
21418931	2011	6	8	6640	62.75	23.44
21418931	2011	6	8	6650	60.81	23.65
21418931	2011	6	8	6660	58.77	23.74
21418931	2011	6	8	6670	56.74	23.84
21418931	2011	6	8	6680	54.67	24.03
21418931	2011	6	8	6690	52.6	24.18
21418931	2011	6	8	6700	50.42	24.31
21418931	2011	6	8	6710	48.17	24.47
21418931	2011	6	8	6720	46.05	24.57
21418931	2011	6	8	6730	43.93	24.68
21418931	2011	6	8	6740	41.86	24.79
21418931	2011	6	8	6750	39.75	24.9
21418931	2011	6	8	6760	37.62	25.07
21418931	2011	6	8	6770	35.31	25.18
21418931	2011	6	8	6780	32.8	25.36
21418931	2011	6	8	6790	30.21	25.57
21418931	2011	6	8	6800	27.93	25.67
21418931	2011	6	8	6810	25.55	25.82
21418931	2011	6	8	6820	23.23	26
21418931	2011	6	8	6830	21.06	26.2
21418931	2011	6	8	6840	18.7	26.29
21418931	2011	6	8	6850	16.39	26.45
21418931	2011	6	8	6860	14.28	26.58
21418931	2011	6	8	6870	11.93	26.71
21418931	2011	6	8	6880	9.49	26.82
21418931	2011	6	8	6890	7.5	27.07
21418931	2011	6	8	6900	5.55	27.27
21418931	2011	6	8	6910	3.57	27.4
21418931	2011	6	8	6920	2.71	27.48
21418931	2011	6	8	6930	2.18	27.62
21418931	2011	6	8	6940	1.83	27.7
21418931	2011	6	8	6950	1.8	27.75
21418931	2011	6	8	6960	0.79	27.96
21418931	2011	6	8	6970	0.59	28.02
21418931	2011	6	8	6980	0.6	28.03
21418931	2011	6	8	6990	0.61	28.09
21418931	2011	6	8	7000	0.59	28.16
21418931	2011	6	8	7010	0.61	28.3
21418931	2011	6	8	7020	0.57	28.49
21418931	2011	6	8	7030	0.61	28.53
21418931	2011	6	8	7040	0.6	28.62
21418931	2011	6	8	7050	0.6	28.66
21418931	2011	6	8	7060	0.66	28.71
21418931	2011	6	8	7070	0.6	28.76

21418931	2011	6	8	7080	0.59	28.81
21418931	2011	6	8	7090	0.56	28.87
21418931	2011	6	8	7100	0.48	28.88
21428111	2011	6	8	0	1.03	29.96
21428111	2011	6	8	10	1.72	29.94
21428111	2011	6	8	20	1.98	29.95
21428111	2011	6	8	30	2.06	29.93
21428111	2011	6	8	40	2.69	29.92
21428111	2011	6	8	50	2.76	29.92
21428111	2011	6	8	60	2.78	29.9
21428111	2011	6	8	70	2.9	29.91
21428111	2011	6	8	80	3.15	29.9
21428111	2011	6	8	90	3.6	29.88
21428111	2011	6	8	100	5.13	29.82
21428111	2011	6	8	110	7.12	29.75
21428111	2011	6	8	120	8.42	29.73
21428111	2011	6	8	130	8.51	29.67
21428111	2011	6	8	140	8.21	29.67
21428111	2011	6	8	150	8.2	29.66
21428111	2011	6	8	160	8.29	29.63
21428111	2011	6	8	170	8.34	29.61
21428111	2011	6	8	180	8.93	29.58
21428111	2011	6	8	190	8.88	29.54
21428111	2011	6	8	200	8.55	29.53
21428111	2011	6	8	210	8.99	29.51
21428111	2011	6	8	220	9.7	29.5
21428111	2011	6	8	230	9.89	29.46
21428111	2011	6	8	240	9.88	29.45
21428111	2011	6	8	250	9.42	29.44
21428111	2011	6	8	260	8.59	29.44
21428111	2011	6	8	270	7.7	29.44
21428111	2011	6	8	280	7.08	29.42
21428111	2011	6	8	290	7.76	29.41
21428111	2011	6	8	300	9.11	29.37
21428111	2011	6	8	310	9.3	29.37
21428111	2011	6	8	320	8.91	29.36
21428111	2011	6	8	330	10.27	29.35
21428111	2011	6	8	340	12.39	29.32
21428111	2011	6	8	350	14.41	29.28
21428111	2011	6	8	360	15.81	29.25
21428111	2011	6	8	370	16.89	29.23
21428111	2011	6	8	380	18.74	29.17
21428111	2011	6	8	390	20.71	29.1
21428111	2011	6	8	400	22.68	29.08
21428111	2011	6	8	410	24.98	29.03
21428111	2011	6	8	420	27.27	28.98
21428111	2011	6	8	430	29.62	28.93

21428111	2011	6	8	440	31.94	28.92
21428111	2011	6	8	450	34.58	28.89
21428111	2011	6	8	460	37.28	28.8
21428111	2011	6	8	470	39.96	28.71
21428111	2011	6	8	480	42.52	28.6
21428111	2011	6	8	490	45.01	28.52
21428111	2011	6	8	500	47.49	28.37
21428111	2011	6	8	510	49.83	28.24
21428111	2011	6	8	520	52.07	28.19
21428111	2011	6	8	530	54.13	28.15
21428111	2011	6	8	540	55.26	28.06
21428111	2011	6	8	550	56.59	28
21428111	2011	6	8	560	57.66	27.96
21428111	2011	6	8	570	57.69	27.89
21428111	2011	6	8	580	57.17	27.87
21428111	2011	6	8	590	56.3	27.85
21428111	2011	6	8	600	55.26	27.79
21428111	2011	6	8	610	55.56	27.73
21428111	2011	6	8	620	57.13	27.64
21428111	2011	6	8	630	59.08	27.52
21428111	2011	6	8	640	61.28	27.41
21428111	2011	6	8	650	63.73	27.34
21428111	2011	6	8	660	66.42	27.3
21428111	2011	6	8	670	69.37	27.18
21428111	2011	6	8	680	70.9	27.14
21428111	2011	6	8	690	70.83	27.09
21428111	2011	6	8	700	70.98	27.08
21428111	2011	6	8	710	72.79	27
21428111	2011	6	8	720	73.94	26.97
21428111	2011	6	8	730	74.46	26.95
21428111	2011	6	8	740	76.14	26.89
21428111	2011	6	8	750	77.52	26.82
21428111	2011	6	8	760	78.78	26.77
21428111	2011	6	8	770	79.89	26.74
21428111	2011	6	8	780	81.96	26.62
21428111	2011	6	8	790	82.85	26.61
21428111	2011	6	8	800	83.39	26.57
21428111	2011	6	8	810	84.93	26.49
21428111	2011	6	8	820	85.59	26.45
21428111	2011	6	8	830	86.57	26.38
21428111	2011	6	8	840	88.07	26.32
21428111	2011	6	8	850	89.63	26.26
21428111	2011	6	8	860	91.6	26.18
21428111	2011	6	8	870	92.18	26.12
21428111	2011	6	8	880	92.58	26.07
21428111	2011	6	8	890	92.56	26.04
21428111	2011	6	8	900	93.39	25.97

21428111	2011	6	8	910	93.83	25.89
21428111	2011	6	8	920	93.71	25.82
21428111	2011	6	8	930	94.64	25.75
21428111	2011	6	8	940	94.54	25.67
21428111	2011	6	8	950	94.88	25.64
21428111	2011	6	8	960	94.63	25.61
21428111	2011	6	8	970	94.65	25.53
21428111	2011	6	8	980	94.63	25.46
21428111	2011	6	8	990	94.52	25.43
21428111	2011	6	8	1000	94.04	25.4
21428111	2011	6	8	1010	94.75	25.34
21428111	2011	6	8	1020	95.27	25.28
21428111	2011	6	8	1030	96.02	25.16
21428111	2011	6	8	1040	96.89	25.08
21428111	2011	6	8	1050	97.89	25
21428111	2011	6	8	1060	99.07	24.92
21428111	2011	6	8	1070	99.6	24.85
21428111	2011	6	8	1080	100.9	24.79
21428111	2011	6	8	1090	101.14	24.76
21428111	2011	6	8	1100	101.23	24.71
21428111	2011	6	8	1110	101.2	24.64
21428111	2011	6	8	1120	101.18	24.61
21428111	2011	6	8	1130	101.45	24.57
21428111	2011	6	8	1140	101.4	24.52
21428111	2011	6	8	1150	101.92	24.48
21428111	2011	6	8	1160	102.68	24.42
21428111	2011	6	8	1170	103.69	24.35
21428111	2011	6	8	1180	104.8	24.29
21428111	2011	6	8	1190	104.69	24.26
21428111	2011	6	8	1200	104.88	24.22
21428111	2011	6	8	1210	105.5	24.17
21428111	2011	6	8	1220	105.79	24.12
21428111	2011	6	8	1230	105.89	24.09
21428111	2011	6	8	1240	105.78	24.07
21428111	2011	6	8	1250	106.27	24.03
21428111	2011	6	8	1260	106.64	23.99
21428111	2011	6	8	1270	106.83	23.96
21428111	2011	6	8	1280	106.81	23.92
21428111	2011	6	8	1290	106.99	23.87
21428111	2011	6	8	1300	106.9	23.85
21428111	2011	6	8	1310	108.4	23.79
21428111	2011	6	8	1320	110.24	23.73
21428111	2011	6	8	1330	112.04	23.71
21428111	2011	6	8	1340	113.59	23.66
21428111	2011	6	8	1350	114.84	23.64
21428111	2011	6	8	1360	116.65	23.61
21428111	2011	6	8	1370	117.25	23.58

21428111	2011	6	8	1380	118.62	23.53
21428111	2011	6	8	1390	120.25	23.47
21428111	2011	6	8	1400	120.98	23.44
21428111	2011	6	8	1410	120.92	23.42
21428111	2011	6	8	1420	121.99	23.34
21428111	2011	6	8	1430	123.6	23.22
21428111	2011	6	8	1440	124.55	23.12
21428111	2011	6	8	1450	125.9	23.03
21428111	2011	6	8	1460	127.37	22.96
21428111	2011	6	8	1470	128.88	22.91
21428111	2011	6	8	1480	130.57	22.85
21428111	2011	6	8	1490	131.48	22.77
21428111	2011	6	8	1500	133.4	22.7
21428111	2011	6	8	1510	135.51	22.58
21428111	2011	6	8	1520	136.48	22.53
21428111	2011	6	8	1530	138.01	22.43
21428111	2011	6	8	1540	138.57	22.39
21428111	2011	6	8	1550	139.06	22.35
21428111	2011	6	8	1560	139.26	22.28
21428111	2011	6	8	1570	139.75	22.21
21428111	2011	6	8	1580	140.56	22.15
21428111	2011	6	8	1590	141.82	22.07
21428111	2011	6	8	1600	143.26	22
21428111	2011	6	8	1610	143.87	21.96
21428111	2011	6	8	1620	145.39	21.84
21428111	2011	6	8	1630	146.28	21.76
21428111	2011	6	8	1640	147.15	21.72
21428111	2011	6	8	1650	147.27	21.67
21428111	2011	6	8	1660	147.35	21.61
21428111	2011	6	8	1670	147.95	21.51
21428111	2011	6	8	1680	148.07	21.46
21428111	2011	6	8	1690	148.47	21.39
21428111	2011	6	8	1700	148.97	21.33
21428111	2011	6	8	1710	150.29	21.19
21428111	2011	6	8	1720	150.68	21.14
21428111	2011	6	8	1730	151.24	21.08
21428111	2011	6	8	1740	151.78	21.01
21428111	2011	6	8	1750	151.85	20.99
21428111	2011	6	8	1760	152.22	20.93
21428111	2011	6	8	1770	153.59	20.76
21428111	2011	6	8	1780	154.7	20.65
21428111	2011	6	8	1790	155.49	20.66
21428111	2011	6	8	1800	155.55	20.65
21428111	2011	6	8	1810	155.82	20.63
21428111	2011	6	8	1820	155.96	20.59
21428111	2011	6	8	1830	156.18	20.57
21428111	2011	6	8	1840	156.29	20.52

21428111	2011	6	8	1850	156.3	20.49
21428111	2011	6	8	1860	156.27	20.45
21428111	2011	6	8	1870	156.51	20.4
21428111	2011	6	8	1880	156.42	20.36
21428111	2011	6	8	1890	156.13	20.34
21428111	2011	6	8	1900	156.68	20.32
21428111	2011	6	8	1910	156.7	20.28
21428111	2011	6	8	1920	156.75	20.26
21428111	2011	6	8	1930	156.62	20.22
21428111	2011	6	8	1940	156.83	20.19
21428111	2011	6	8	1950	157.39	20.16
21428111	2011	6	8	1960	157.92	20.14
21428111	2011	6	8	1970	158.88	20.1
21428111	2011	6	8	1980	159.5	20.05
21428111	2011	6	8	1990	159.88	19.99
21428111	2011	6	8	2000	161.03	19.9
21428111	2011	6	8	2010	161.76	19.83
21428111	2011	6	8	2020	162.86	19.67
21428111	2011	6	8	2030	163.66	19.66
21428111	2011	6	8	2040	164.92	19.55
21428111	2011	6	8	2050	165.62	19.5
21428111	2011	6	8	2060	166.37	19.46
21428111	2011	6	8	2070	167.24	19.42
21428111	2011	6	8	2080	167.78	19.39
21428111	2011	6	8	2090	168.14	19.32
21428111	2011	6	8	2100	168.22	19.23
21428111	2011	6	8	2110	168.86	19.17
21428111	2011	6	8	2120	169.11	19.13
21428111	2011	6	8	2130	169.38	19.04
21428111	2011	6	8	2140	169.7	19.01
21428111	2011	6	8	2150	169.79	18.95
21428111	2011	6	8	2160	169.75	18.9
21428111	2011	6	8	2170	169.72	18.86
21428111	2011	6	8	2180	169.84	18.82
21428111	2011	6	8	2190	169.71	18.77
21428111	2011	6	8	2200	170.04	18.72
21428111	2011	6	8	2210	170.27	18.67
21428111	2011	6	8	2220	170.04	18.64
21428111	2011	6	8	2230	170.09	18.6
21428111	2011	6	8	2240	170.11	18.56
21428111	2011	6	8	2250	169.74	18.51
21428111	2011	6	8	2260	170.22	18.48
21428111	2011	6	8	2270	170.12	18.45
21428111	2011	6	8	2280	169.75	18.43
21428111	2011	6	8	2290	170.06	18.39
21428111	2011	6	8	2300	170.1	18.37
21428111	2011	6	8	2310	169.68	18.34

21428111	2011	6	8	2320	169.55	18.3
21428111	2011	6	8	2330	169.37	18.28
21428111	2011	6	8	2340	169.41	18.26
21428111	2011	6	8	2350	168.93	18.21
21428111	2011	6	8	2360	168.27	18.2
21428111	2011	6	8	2370	168.16	18.18
21428111	2011	6	8	2380	168.15	18.17
21428111	2011	6	8	2390	168.44	18.14
21428111	2011	6	8	2400	168.89	18.13
21428111	2011	6	8	2410	169	18.11
21428111	2011	6	8	2420	168.97	18.1
21428111	2011	6	8	2430	168.59	18.08
21428111	2011	6	8	2440	167.75	18.07
21428111	2011	6	8	2450	167.27	18.07
21428111	2011	6	8	2460	166.72	18.05
21428111	2011	6	8	2470	165.82	18.04
21428111	2011	6	8	2480	165.24	18.03
21428111	2011	6	8	2490	164.91	18.01
21428111	2011	6	8	2500	164.64	18
21428111	2011	6	8	2510	164.54	18
21428111	2011	6	8	2520	164.61	18
21428111	2011	6	8	2530	164.56	17.99
21428111	2011	6	8	2540	163.47	17.98
21428111	2011	6	8	2550	162.82	17.99
21428111	2011	6	8	2560	163.6	17.99
21428111	2011	6	8	2570	164.23	18
21428111	2011	6	8	2580	164.37	17.99
21428111	2011	6	8	2590	164.3	17.98
21428111	2011	6	8	2600	164.25	17.98
21428111	2011	6	8	2610	163.67	17.96
21428111	2011	6	8	2620	163.21	17.96
21428111	2011	6	8	2630	162.99	17.95
21428111	2011	6	8	2640	162.81	17.96
21428111	2011	6	8	2650	161.47	17.95
21428111	2011	6	8	2660	161.3	17.95
21428111	2011	6	8	2670	161.11	17.98
21428111	2011	6	8	2680	159.78	18.01
21428111	2011	6	8	2690	158.49	18.04
21428111	2011	6	8	2700	156.94	18.11
21428111	2011	6	8	2710	155.54	18.2
21428111	2011	6	8	2720	154.3	18.25
21428111	2011	6	8	2730	154.17	18.3
21428111	2011	6	8	2740	154.48	18.36
21428111	2011	6	8	2750	154.78	18.44
21428111	2011	6	8	2760	154.76	18.49
21428111	2011	6	8	2770	154.83	18.53
21428111	2011	6	8	2780	154.66	18.57

21428111	2011	6	8	2790	154.56	18.6
21428111	2011	6	8	2800	154.62	18.65
21428111	2011	6	8	2810	154.56	18.69
21428111	2011	6	8	2820	154.78	18.72
21428111	2011	6	8	2830	154.88	18.78
21428111	2011	6	8	2840	155	18.81
21428111	2011	6	8	2850	154.97	18.83
21428111	2011	6	8	2860	155.07	18.88
21428111	2011	6	8	2870	155.08	18.92
21428111	2011	6	8	2880	155.1	18.94
21428111	2011	6	8	2890	154.94	18.96
21428111	2011	6	8	2900	154.94	19
21428111	2011	6	8	2910	155.04	19.02
21428111	2011	6	8	2920	154.74	19.04
21428111	2011	6	8	2930	154.69	19.07
21428111	2011	6	8	2940	154.35	19.08
21428111	2011	6	8	2950	153.38	19.11
21428111	2011	6	8	2960	152.56	19.13
21428111	2011	6	8	2970	152.29	19.15
21428111	2011	6	8	2980	150.97	19.18
21428111	2011	6	8	2990	149.13	19.2
21428111	2011	6	8	3000	147.01	19.24
21428111	2011	6	8	3010	144.91	19.27
21428111	2011	6	8	3020	143.32	19.28
21428111	2011	6	8	3030	142.65	19.3
21428111	2011	6	8	3040	141.74	19.31
21428111	2011	6	8	3050	140.05	19.35
21428111	2011	6	8	3060	137.67	19.39
21428111	2011	6	8	3070	135.39	19.49
21428111	2011	6	8	3080	133.73	19.57
21428111	2011	6	8	3090	131.67	19.69
21428111	2011	6	8	3100	129.65	19.82
21428111	2011	6	8	3110	127.95	19.97
21428111	2011	6	8	3120	126.77	20.05
21428111	2011	6	8	3130	125.16	20.13
21428111	2011	6	8	3140	124.23	20.21
21428111	2011	6	8	3150	122.72	20.3
21428111	2011	6	8	3160	121.09	20.35
21428111	2011	6	8	3170	119.56	20.47
21428111	2011	6	8	3180	117.31	20.52
21428111	2011	6	8	3190	115.16	20.6
21428111	2011	6	8	3200	114.59	20.65
21428111	2011	6	8	3210	112.97	20.74
21428111	2011	6	8	3220	110.91	20.8
21428111	2011	6	8	3230	108.91	20.88
21428111	2011	6	8	3240	107.59	21
21428111	2011	6	8	3250	106.23	21.21

21428111	2011	6	8	3260	104.24	21.27
21428111	2011	6	8	3270	102.22	21.42
21428111	2011	6	8	3280	100.08	21.52
21428111	2011	6	8	3290	98.17	21.61
21428111	2011	6	8	3300	96.43	21.72
21428111	2011	6	8	3310	94.93	21.83
21428111	2011	6	8	3320	94.36	21.96
21428111	2011	6	8	3330	93.6	22.12
21428111	2011	6	8	3340	92.46	22.25
21428111	2011	6	8	3350	92.18	22.37
21428111	2011	6	8	3360	91.83	22.57
21428111	2011	6	8	3370	91.6	22.65
21428111	2011	6	8	3380	91.33	22.71
21428111	2011	6	8	3390	90.35	22.79
21428111	2011	6	8	3400	90.21	22.89
21428111	2011	6	8	3410	89.23	22.99
21428111	2011	6	8	3420	89.32	23.04
21428111	2011	6	8	3430	88.97	23.13
21428111	2011	6	8	3440	88.32	23.21
21428111	2011	6	8	3450	86.94	23.37
21428111	2011	6	8	3460	86.53	23.43
21428111	2011	6	8	3470	86.27	23.5
21428111	2011	6	8	3480	86.19	23.6
21428111	2011	6	8	3490	86.02	23.67
21428111	2011	6	8	3500	85.75	23.75
21428111	2011	6	8	3510	85.93	23.84
21428111	2011	6	8	3520	85.87	23.89
21428111	2011	6	8	3530	85.73	23.96
21428111	2011	6	8	3540	85.7	24.03
21428111	2011	6	8	3550	86.21	24.1
21428111	2011	6	8	3560	86.38	24.17
21428111	2011	6	8	3570	86.56	24.22
21428111	2011	6	8	3580	85.24	24.31
21428111	2011	6	8	3590	84.11	24.37
21428111	2011	6	8	3600	83.18	24.43
21428111	2011	6	8	3610	82.05	24.5
21428111	2011	6	8	3620	79.38	24.57
21428111	2011	6	8	3630	76.79	24.64
21428111	2011	6	8	3640	75.38	24.71
21428111	2011	6	8	3650	73.11	24.8
21428111	2011	6	8	3660	71.28	24.87
21428111	2011	6	8	3670	69.05	24.96
21428111	2011	6	8	3680	66.03	25.01
21428111	2011	6	8	3690	62.73	25.08
21428111	2011	6	8	3700	59.55	25.14
21428111	2011	6	8	3710	57	25.21
21428111	2011	6	8	3720	56.09	25.27

21428111	2011	6	8	3730	54.67	25.34
21428111	2011	6	8	3740	53.53	25.43
21428111	2011	6	8	3750	53.19	25.49
21428111	2011	6	8	3760	51.89	25.56
21428111	2011	6	8	3770	50.88	25.6
21428111	2011	6	8	3780	51.31	25.64
21428111	2011	6	8	3790	50.93	25.7
21428111	2011	6	8	3800	50.78	25.75
21428111	2011	6	8	3810	51.08	25.79
21428111	2011	6	8	3820	51.19	25.84
21428111	2011	6	8	3830	51.59	25.87
21428111	2011	6	8	3840	51.39	25.92
21428111	2011	6	8	3850	51.36	25.96
21428111	2011	6	8	3860	51.51	26.03
21428111	2011	6	8	3870	51.47	26.05
21428111	2011	6	8	3880	51.41	26.08
21428111	2011	6	8	3890	51.4	26.1
21428111	2011	6	8	3900	51.51	26.14
21428111	2011	6	8	3910	51.63	26.2
21428111	2011	6	8	3920	51.65	26.22
21428111	2011	6	8	3930	51.59	26.24
21428111	2011	6	8	3940	51.74	26.28
21428111	2011	6	8	3950	51.49	26.29
21428111	2011	6	8	3960	51.9	26.32
21428111	2011	6	8	3970	52.01	26.34
21428111	2011	6	8	3980	52.21	26.35
21428111	2011	6	8	3990	52.6	26.36
21428111	2011	6	8	4000	52.39	26.37
21428111	2011	6	8	4010	52.02	26.39
21428111	2011	6	8	4020	52.17	26.41
21428111	2011	6	8	4030	52.22	26.45
21428111	2011	6	8	4040	52.12	26.46
21428111	2011	6	8	4050	51.96	26.46
21428111	2011	6	8	4060	52.52	26.47
21428111	2011	6	8	4070	52.3	26.49
21428111	2011	6	8	4080	51.96	26.5
21428111	2011	6	8	4090	51.62	26.52
21428111	2011	6	8	4100	51.39	26.52
21428111	2011	6	8	4110	51.29	26.53
21428111	2011	6	8	4120	51.25	26.54
21428111	2011	6	8	4130	49.94	26.55
21428111	2011	6	8	4140	49.27	26.56
21428111	2011	6	8	4150	49.12	26.57
21428111	2011	6	8	4160	49.25	26.57
21428111	2011	6	8	4170	49.29	26.58
21428111	2011	6	8	4180	49.3	26.6
21428111	2011	6	8	4190	49.3	26.61

21428111	2011	6	8	4200	49.13	26.63
21428111	2011	6	8	4210	48.23	26.64
21428111	2011	6	8	4220	47.46	26.64
21428111	2011	6	8	4230	47.12	26.65
21428111	2011	6	8	4240	46.85	26.67
21428111	2011	6	8	4250	46.52	26.7
21428111	2011	6	8	4260	46.47	26.71
21428111	2011	6	8	4270	46.46	26.73
21428111	2011	6	8	4280	46.64	26.75
21428111	2011	6	8	4290	47.07	26.75
21428111	2011	6	8	4300	46.77	26.75
21428111	2011	6	8	4310	46.27	26.75
21428111	2011	6	8	4320	45.99	26.77
21428111	2011	6	8	4330	46.17	26.77
21428111	2011	6	8	4340	45.9	26.78
21428111	2011	6	8	4350	45.6	26.78
21428111	2011	6	8	4360	45.49	26.81
21428111	2011	6	8	4370	45.38	26.84
21428111	2011	6	8	4380	45.37	26.85
21428111	2011	6	8	4390	45.26	26.85
21428111	2011	6	8	4400	44.89	26.86
21428111	2011	6	8	4410	44.62	26.89
21428111	2011	6	8	4420	44.71	26.9
21428111	2011	6	8	4430	45.18	26.91
21428111	2011	6	8	4440	45.74	26.92
21428111	2011	6	8	4450	45.88	26.93
21428111	2011	6	8	4460	46.33	26.94
21428111	2011	6	8	4470	46.23	26.94
21428111	2011	6	8	4480	45.96	26.94
21428111	2011	6	8	4490	45.72	26.95
21428111	2011	6	8	4500	45.5	26.96
21428111	2011	6	8	4510	45.52	26.95
21428111	2011	6	8	4520	46.2	26.96
21428111	2011	6	8	4530	47.06	26.96
21428111	2011	6	8	4540	47.3	26.95
21428111	2011	6	8	4550	47.21	26.95
21428111	2011	6	8	4560	47.26	26.94
21428111	2011	6	8	4570	47.22	26.93
21428111	2011	6	8	4580	46.87	26.95
21428111	2011	6	8	4590	46.77	26.94
21428111	2011	6	8	4600	46.78	26.94
21428111	2011	6	8	4610	46.71	26.94
21428111	2011	6	8	4620	46.67	26.95
21428111	2011	6	8	4630	46.56	26.94
21428111	2011	6	8	4640	46.47	26.95
21428111	2011	6	8	4650	45.78	26.96
21428111	2011	6	8	4660	45.27	26.96

21428111	2011	6	8	4670	45.2	26.97
21428111	2011	6	8	4680	44.78	27
21428111	2011	6	8	4690	44.66	27.03
21428111	2011	6	8	4700	44.49	27.04
21428111	2011	6	8	4710	44.7	27.07
21428111	2011	6	8	4720	44.81	27.1
21428111	2011	6	8	4730	44.77	27.14
21428111	2011	6	8	4740	44.08	27.17
21428111	2011	6	8	4750	43.86	27.19
21428111	2011	6	8	4760	43.97	27.21
21428111	2011	6	8	4770	43.36	27.24
21428111	2011	6	8	4780	42.62	27.28
21428111	2011	6	8	4790	42.4	27.28
21428111	2011	6	8	4800	42	27.31
21428111	2011	6	8	4810	41.74	27.33
21428111	2011	6	8	4820	41.32	27.34
21428111	2011	6	8	4830	40.67	27.36
21428111	2011	6	8	4840	40.63	27.39
21428111	2011	6	8	4850	40.62	27.42
21428111	2011	6	8	4860	40.66	27.46
21428111	2011	6	8	4870	40.56	27.5
21428111	2011	6	8	4880	40.24	27.56
21428111	2011	6	8	4890	39.62	27.58
21428111	2011	6	8	4900	38.41	27.62
21428111	2011	6	8	4910	37.87	27.65
21428111	2011	6	8	4920	37.66	27.68
21428111	2011	6	8	4930	37.34	27.74
21428111	2011	6	8	4940	35.42	27.77
21428111	2011	6	8	4950	34.05	27.78
21428111	2011	6	8	4960	32.64	27.83
21428111	2011	6	8	4970	31.61	27.86
21428111	2011	6	8	4980	31.31	27.9
21428111	2011	6	8	4990	30.67	27.95
21428111	2011	6	8	5000	30.5	27.99
21428111	2011	6	8	5010	29.67	28.06
21428111	2011	6	8	5020	28.96	28.08
21428111	2011	6	8	5030	28.47	28.12
21428111	2011	6	8	5040	27.93	28.17
21428111	2011	6	8	5050	27.07	28.21
21428111	2011	6	8	5060	26.75	28.25
21428111	2011	6	8	5070	26.29	28.28
21428111	2011	6	8	5080	26.15	28.3
21428111	2011	6	8	5090	26.07	28.34
21428111	2011	6	8	5100	26.15	28.37
21428111	2011	6	8	5110	26.26	28.4
21428111	2011	6	8	5120	26.29	28.43
21428111	2011	6	8	5130	26.35	28.44

21428111	2011	6	8	5140	25.74	28.47
21428111	2011	6	8	5150	25.85	28.5
21428111	2011	6	8	5160	25.91	28.5
21428111	2011	6	8	5170	26.11	28.52
21428111	2011	6	8	5180	26.19	28.53
21428111	2011	6	8	5190	26.38	28.55
21428111	2011	6	8	5200	26.63	28.56
21428111	2011	6	8	5210	26.96	28.57
21428111	2011	6	8	5220	26.93	28.6
21428111	2011	6	8	5230	26.92	28.62
21428111	2011	6	8	5240	27.1	28.63
21428111	2011	6	8	5250	27.29	28.64
21428111	2011	6	8	5260	27.5	28.65
21428111	2011	6	8	5270	27.67	28.65
21428111	2011	6	8	5280	27.93	28.67
21428111	2011	6	8	5290	28.35	28.67
21428111	2011	6	8	5300	28.38	28.68
21428111	2011	6	8	5310	28.55	28.69
21428111	2011	6	8	5320	28.61	28.69
21428111	2011	6	8	5330	28.1	28.71
21428111	2011	6	8	5340	27.85	28.73
21428111	2011	6	8	5350	27.79	28.73
21428111	2011	6	8	5360	27.65	28.75
21428111	2011	6	8	5370	27.74	28.75
21428111	2011	6	8	5380	28.03	28.75
21428111	2011	6	8	5390	27.74	28.75
21428111	2011	6	8	5400	27.46	28.76
21428111	2011	6	8	5410	27.6	28.78
21428111	2011	6	8	5420	27.49	28.78
21428111	2011	6	8	5430	26.46	28.79
21428111	2011	6	8	5440	26.15	28.8
21428111	2011	6	8	5450	26.36	28.8
21428111	2011	6	8	5460	26.72	28.79
21428111	2011	6	8	5470	25.34	28.82
21428111	2011	6	8	5480	23.54	28.84
21428111	2011	6	8	5490	22.12	28.86
21428111	2011	6	8	5500	20.16	28.89
21428111	2011	6	8	5510	18.3	28.9
21428111	2011	6	8	5520	17.31	28.92
21428111	2011	6	8	5530	15.86	28.92
21428111	2011	6	8	5540	13.45	28.94
21428111	2011	6	8	5550	11.06	28.96
21428111	2011	6	8	5560	8.54	29
21428111	2011	6	8	5570	5.92	29.03
21428111	2011	6	8	5580	3.69	29.06
21428111	2011	6	8	5590	2.86	29.08
21428111	2011	6	8	5600	2.64	29.11

21428111	2011	6	8	5610	2.36	29.13
21428111	2011	6	8	5620	2.74	29.15
21428111	2011	6	8	5630	2.81	29.19
21428111	2011	6	8	5640	2.5	29.21
21428111	2011	6	8	5650	1.48	29.21
21428111	2011	6	8	5660	0.59	29.28
21428111	2011	6	8	5670	0.6	29.28
21428111	2011	6	8	5680	0.58	29.31
21428111	2011	6	8	5690	0.56	29.39
21428111	2011	6	8	5700	0.57	29.4
21428111	2011	6	8	5710	0.57	29.42
21428111	2011	6	8	5720	0.57	29.45
21428111	2011	6	8	5730	0.48	29.5
21428111	2011	6	8	5740	0.49	29.54
21428111	2011	6	8	5750	0.52	29.54
21428111	2011	6	8	5760	0.49	29.56
21428111	2011	6	8	5770	0.52	29.57
21428111	2011	6	8	5780	0.5	29.59
21428111	2011	6	8	5790	0.52	29.6
21428111	2011	6	8	5800	0.52	29.61
21491371	2011	6	9	0	1.06	28.92
21491371	2011	6	9	10	1.4	28.92
21491371	2011	6	9	20	2.5	28.92
21491371	2011	6	9	30	2.61	28.92
21491371	2011	6	9	40	2.79	28.92
21491371	2011	6	9	50	2.66	28.92
21491371	2011	6	9	60	2.89	28.92
21491371	2011	6	9	70	3.45	28.92
21491371	2011	6	9	80	3.98	28.92
21491371	2011	6	9	90	5.19	28.92
21491371	2011	6	9	100	7.16	28.91
21491371	2011	6	9	110	9.49	28.9
21491371	2011	6	9	120	12.57	28.87
21491371	2011	6	9	130	15.14	28.85
21491371	2011	6	9	140	16.31	28.83
21491371	2011	6	9	150	17.7	28.82
21491371	2011	6	9	160	18.4	28.82
21491371	2011	6	9	170	17.16	28.82
21491371	2011	6	9	180	15.94	28.83
21491371	2011	6	9	190	15.89	28.83
21491371	2011	6	9	200	16.25	28.83
21491371	2011	6	9	210	16.8	28.82
21491371	2011	6	9	220	17.43	28.81
21491371	2011	6	9	230	17.77	28.82
21491371	2011	6	9	240	16.89	28.82
21491371	2011	6	9	250	15.6	28.83
21491371	2011	6	9	260	13.82	28.84

21491371	2011	6	9	270	11.95	28.85
21491371	2011	6	9	280	10.02	28.87
21491371	2011	6	9	290	7.95	28.89
21491371	2011	6	9	300	6.27	28.9
21491371	2011	6	9	310	6.12	28.9
21491371	2011	6	9	320	6.63	28.9
21491371	2011	6	9	330	7.11	28.89
21491371	2011	6	9	340	7.58	28.89
21491371	2011	6	9	350	8.16	28.89
21491371	2011	6	9	360	8.79	28.88
21491371	2011	6	9	370	8.9	28.88
21491371	2011	6	9	380	9.39	28.88
21491371	2011	6	9	390	9.71	28.88
21491371	2011	6	9	400	9.96	28.87
21491371	2011	6	9	410	9.98	28.88
21491371	2011	6	9	420	8.9	28.88
21491371	2011	6	9	430	8.32	28.89
21491371	2011	6	9	440	7.93	28.89
21491371	2011	6	9	450	7.14	28.9
21491371	2011	6	9	460	6.25	28.91
21491371	2011	6	9	470	6.3	28.91
21491371	2011	6	9	480	6.58	28.9
21491371	2011	6	9	490	7.02	28.9
21491371	2011	6	9	500	7.62	28.89
21491371	2011	6	9	510	8.22	28.89
21491371	2011	6	9	520	8.69	28.89
21491371	2011	6	9	530	9.36	28.89
21491371	2011	6	9	540	10.07	28.89
21491371	2011	6	9	550	9.92	28.88
21491371	2011	6	9	560	9.44	28.89
21491371	2011	6	9	570	9.76	28.89
21491371	2011	6	9	580	10.16	28.88
21491371	2011	6	9	590	9.85	28.89
21491371	2011	6	9	600	9.87	28.88
21491371	2011	6	9	610	10.01	28.89
21491371	2011	6	9	620	9.92	28.89
21491371	2011	6	9	630	10.02	28.88
21491371	2011	6	9	640	9.9	28.88
21491371	2011	6	9	650	9.94	28.88
21491371	2011	6	9	660	9.98	28.88
21491371	2011	6	9	670	9.9	28.89
21491371	2011	6	9	680	9.84	28.88
21491371	2011	6	9	690	10.02	28.88
21491371	2011	6	9	700	10.02	28.88
21491371	2011	6	9	710	9.84	28.89
21491371	2011	6	9	720	9.88	28.88
21491371	2011	6	9	730	9.48	28.89

21491371	2011	6	9	740	9.61	28.89
21491371	2011	6	9	750	9.7	28.88
21491371	2011	6	9	760	9.67	28.88
21491371	2011	6	9	770	10.01	28.88
21491371	2011	6	9	780	9.95	28.89
21491371	2011	6	9	790	9.93	28.88
21491371	2011	6	9	800	9.95	28.88
21491371	2011	6	9	810	9.93	28.88
21491371	2011	6	9	820	9.95	28.89
21491371	2011	6	9	830	9.96	28.88
21491371	2011	6	9	840	9.94	28.88
21491371	2011	6	9	850	9.98	28.89
21491371	2011	6	9	860	9.9	28.88
21491371	2011	6	9	870	9.93	28.88
21491371	2011	6	9	880	10.04	28.89
21491371	2011	6	9	890	10.01	28.88
21491371	2011	6	9	900	9.91	28.89
21491371	2011	6	9	910	9.98	28.89
21491371	2011	6	9	920	9.95	28.89
21491371	2011	6	9	930	10.03	28.89
21491371	2011	6	9	940	9.97	28.89
21491371	2011	6	9	950	9.98	28.89
21491371	2011	6	9	960	9.98	28.89
21491371	2011	6	9	970	9.94	28.89
21491371	2011	6	9	980	10.07	28.89
21491371	2011	6	9	990	9.96	28.89
21491371	2011	6	9	1000	9.93	28.9
21491371	2011	6	9	1010	9.94	28.89
21491371	2011	6	9	1020	10	28.89
21491371	2011	6	9	1030	10.07	28.89
21491371	2011	6	9	1040	9.84	28.89
21491371	2011	6	9	1050	9.94	28.89
21491371	2011	6	9	1060	9.98	28.9
21491371	2011	6	9	1070	9.95	28.89
21491371	2011	6	9	1080	10.01	28.9
21491371	2011	6	9	1090	10.06	28.9
21491371	2011	6	9	1100	10.02	28.9
21491371	2011	6	9	1110	9.94	28.9
21491371	2011	6	9	1120	9.99	28.9
21491371	2011	6	9	1130	10.01	28.9
21491371	2011	6	9	1140	10.09	28.9
21491371	2011	6	9	1150	9.98	28.9
21491371	2011	6	9	1160	10.06	28.91
21491371	2011	6	9	1170	9.99	28.91
21491371	2011	6	9	1180	10.26	28.91
21491371	2011	6	9	1190	10.26	28.9
21491371	2011	6	9	1200	10.03	28.91

21491371	2011	6	9	1210	10.02	28.9
21491371	2011	6	9	1220	10.23	28.9
21491371	2011	6	9	1230	10.05	28.9
21491371	2011	6	9	1240	10.3	28.89
21491371	2011	6	9	1250	10.33	28.9
21491371	2011	6	9	1260	10.23	28.9
21491371	2011	6	9	1270	10.23	28.9
21491371	2011	6	9	1280	10.12	28.9
21491371	2011	6	9	1290	10.04	28.91
21491371	2011	6	9	1300	10.05	28.9
21491371	2011	6	9	1310	9.8	28.9
21491371	2011	6	9	1320	9.25	28.91
21491371	2011	6	9	1330	9.09	28.92
21491371	2011	6	9	1340	8.54	28.91
21491371	2011	6	9	1350	7.42	28.92
21491371	2011	6	9	1360	7.76	28.92
21491371	2011	6	9	1370	8.17	28.92
21491371	2011	6	9	1380	8.04	28.92
21491371	2011	6	9	1390	7.8	28.92
21491371	2011	6	9	1400	7.76	28.92
21491371	2011	6	9	1410	7.46	28.92
21491371	2011	6	9	1420	7.66	28.92
21491371	2011	6	9	1430	7.73	28.92
21491371	2011	6	9	1440	7.46	28.92
21491371	2011	6	9	1450	6.88	28.92
21491371	2011	6	9	1460	7.44	28.92
21491371	2011	6	9	1470	8.08	28.92
21491371	2011	6	9	1480	8.37	28.92
21491371	2011	6	9	1490	8.67	28.92
21491371	2011	6	9	1500	8.77	28.92
21491371	2011	6	9	1510	9.02	28.92
21491371	2011	6	9	1520	9.19	28.92
21491371	2011	6	9	1530	9.5	28.92
21491371	2011	6	9	1540	9.54	28.92
21491371	2011	6	9	1550	9.73	28.92
21491371	2011	6	9	1560	10.17	28.91
21491371	2011	6	9	1570	10.67	28.9
21491371	2011	6	9	1580	11.27	28.9
21491371	2011	6	9	1590	12	28.89
21491371	2011	6	9	1600	12.7	28.88
21491371	2011	6	9	1610	14.27	28.87
21491371	2011	6	9	1620	16.25	28.84
21491371	2011	6	9	1630	18.22	28.82
21491371	2011	6	9	1640	20.14	28.8
21491371	2011	6	9	1650	22.09	28.78
21491371	2011	6	9	1660	24.17	28.76
21491371	2011	6	9	1670	26.55	28.73

21491371	2011	6	9	1680	28.72	28.7
21491371	2011	6	9	1690	31.59	28.67
21491371	2011	6	9	1700	34.09	28.64
21491371	2011	6	9	1710	36.85	28.63
21491371	2011	6	9	1720	38.4	28.6
21491371	2011	6	9	1730	39.78	28.6
21491371	2011	6	9	1740	41.08	28.59
21491371	2011	6	9	1750	42.22	28.57
21491371	2011	6	9	1760	43.6	28.56
21491371	2011	6	9	1770	44.85	28.54
21491371	2011	6	9	1780	45.8	28.53
21491371	2011	6	9	1790	47.3	28.5
21491371	2011	6	9	1800	48.87	28.48
21491371	2011	6	9	1810	49.73	28.46
21491371	2011	6	9	1820	50.34	28.45
21491371	2011	6	9	1830	52.02	28.36
21491371	2011	6	9	1840	53.14	28.3
21491371	2011	6	9	1850	54.8	28.17
21491371	2011	6	9	1860	57.11	28.04
21491371	2011	6	9	1870	59.86	27.93
21491371	2011	6	9	1880	62.41	27.83
21491371	2011	6	9	1890	64.62	27.75
21491371	2011	6	9	1900	66.8	27.68
21491371	2011	6	9	1910	68.95	27.59
21491371	2011	6	9	1920	71.6	27.46
21491371	2011	6	9	1930	73.77	27.35
21491371	2011	6	9	1940	75.56	27.28
21491371	2011	6	9	1950	77.33	27.24
21491371	2011	6	9	1960	79.5	27.17
21491371	2011	6	9	1970	81.69	27.11
21491371	2011	6	9	1980	83.35	27.03
21491371	2011	6	9	1990	84.29	26.94
21491371	2011	6	9	2000	85.18	26.89
21491371	2011	6	9	2010	86.71	26.73
21491371	2011	6	9	2020	87.75	26.68
21491371	2011	6	9	2030	88.44	26.64
21491371	2011	6	9	2040	89.21	26.57
21491371	2011	6	9	2050	90.69	26.52
21491371	2011	6	9	2060	92.01	26.45
21491371	2011	6	9	2070	92.98	26.36
21491371	2011	6	9	2080	92.8	26.22
21491371	2011	6	9	2090	92.91	26.17
21491371	2011	6	9	2100	92.96	26.08
21491371	2011	6	9	2110	93.01	26.02
21491371	2011	6	9	2120	93.21	25.89
21491371	2011	6	9	2130	93.97	25.82
21491371	2011	6	9	2140	94.55	25.7

21491371	2011	6	9	2150	95.15	25.63
21491371	2011	6	9	2160	96.54	25.53
21491371	2011	6	9	2170	97.52	25.46
21491371	2011	6	9	2180	98.18	25.42
21491371	2011	6	9	2190	98.37	25.38
21491371	2011	6	9	2200	98.22	25.28
21491371	2011	6	9	2210	98.08	25.25
21491371	2011	6	9	2220	98.22	25.2
21491371	2011	6	9	2230	98.29	25.14
21491371	2011	6	9	2240	98.16	25.09
21491371	2011	6	9	2250	98.28	25.03
21491371	2011	6	9	2260	98.31	24.97
21491371	2011	6	9	2270	98.31	24.91
21491371	2011	6	9	2280	98.39	24.88
21491371	2011	6	9	2290	98.34	24.85
21491371	2011	6	9	2300	98.3	24.79
21491371	2011	6	9	2310	98.2	24.74
21491371	2011	6	9	2320	98.17	24.7
21491371	2011	6	9	2330	98.21	24.65
21491371	2011	6	9	2340	98.15	24.61
21491371	2011	6	9	2350	98.02	24.57
21491371	2011	6	9	2360	98.09	24.53
21491371	2011	6	9	2370	98.24	24.46
21491371	2011	6	9	2380	98.6	24.42
21491371	2011	6	9	2390	98.71	24.37
21491371	2011	6	9	2400	98.81	24.35
21491371	2011	6	9	2410	97.98	24.3
21491371	2011	6	9	2420	96.82	24.27
21491371	2011	6	9	2430	96.61	24.25
21491371	2011	6	9	2440	96.77	24.23
21491371	2011	6	9	2450	96.59	24.19
21491371	2011	6	9	2460	96.44	24.15
21491371	2011	6	9	2470	96.33	24.14
21491371	2011	6	9	2480	96.15	24.11
21491371	2011	6	9	2490	96.18	24.1
21491371	2011	6	9	2500	96.16	24.1
21491371	2011	6	9	2510	96.26	24.1
21491371	2011	6	9	2520	96.27	24.08
21491371	2011	6	9	2530	96.16	24.07
21491371	2011	6	9	2540	96.17	24.07
21491371	2011	6	9	2550	96.28	24.07
21491371	2011	6	9	2560	96.44	24.07
21491371	2011	6	9	2570	96.62	24.06
21491371	2011	6	9	2580	97.3	24.03
21491371	2011	6	9	2590	98.39	24.01
21491371	2011	6	9	2600	99.2	23.98
21491371	2011	6	9	2610	99.77	23.96

21491371	2011	6	9	2620	100.12	23.94
21491371	2011	6	9	2630	100.54	23.92
21491371	2011	6	9	2640	100.97	23.9
21491371	2011	6	9	2650	101.89	23.87
21491371	2011	6	9	2660	102.87	23.82
21491371	2011	6	9	2670	103.22	23.78
21491371	2011	6	9	2680	104.78	23.71
21491371	2011	6	9	2690	106.12	23.64
21491371	2011	6	9	2700	107.17	23.59
21491371	2011	6	9	2710	107.82	23.54
21491371	2011	6	9	2720	107.2	23.47
21491371	2011	6	9	2730	105.78	23.46
21491371	2011	6	9	2740	104.66	23.41
21491371	2011	6	9	2750	102.87	23.35
21491371	2011	6	9	2760	100.65	23.32
21491371	2011	6	9	2770	99.02	23.32
21491371	2011	6	9	2780	97.63	23.31
21491371	2011	6	9	2790	95.94	23.3
21491371	2011	6	9	2800	93.73	23.33
21491371	2011	6	9	2810	92.25	23.36
21491371	2011	6	9	2820	90.83	23.4
21491371	2011	6	9	2830	90.07	23.46
21491371	2011	6	9	2840	90.14	23.53
21491371	2011	6	9	2850	90.45	23.6
21491371	2011	6	9	2860	90.28	23.66
21491371	2011	6	9	2870	90.13	23.76
21491371	2011	6	9	2880	89.01	23.87
21491371	2011	6	9	2890	87.37	23.91
21491371	2011	6	9	2900	86.21	23.99
21491371	2011	6	9	2910	85.66	24.1
21491371	2011	6	9	2920	85.68	24.16
21491371	2011	6	9	2930	85.83	24.21
21491371	2011	6	9	2940	86.05	24.27
21491371	2011	6	9	2950	86.01	24.37
21491371	2011	6	9	2960	86.13	24.41
21491371	2011	6	9	2970	86.51	24.45
21491371	2011	6	9	2980	87.13	24.49
21491371	2011	6	9	2990	87.66	24.53
21491371	2011	6	9	3000	87.11	24.58
21491371	2011	6	9	3010	85.88	24.62
21491371	2011	6	9	3020	84.96	24.67
21491371	2011	6	9	3030	83.34	24.74
21491371	2011	6	9	3040	81.76	24.81
21491371	2011	6	9	3050	80.4	24.88
21491371	2011	6	9	3060	78.88	24.9
21491371	2011	6	9	3070	77.58	24.96
21491371	2011	6	9	3080	76.06	24.98

21491371	2011	6	9	3090	74.24	25
21491371	2011	6	9	3100	72.73	25.07
21491371	2011	6	9	3110	71.42	25.13
21491371	2011	6	9	3120	69.92	25.18
21491371	2011	6	9	3130	68.17	25.23
21491371	2011	6	9	3140	66.24	25.3
21491371	2011	6	9	3150	64.44	25.35
21491371	2011	6	9	3160	62.52	25.39
21491371	2011	6	9	3170	60.36	25.44
21491371	2011	6	9	3180	58.13	25.49
21491371	2011	6	9	3190	56.01	25.54
21491371	2011	6	9	3200	54.13	25.64
21491371	2011	6	9	3210	53.94	25.71
21491371	2011	6	9	3220	53.78	25.82
21491371	2011	6	9	3230	52.83	25.9
21491371	2011	6	9	3240	52.07	25.92
21491371	2011	6	9	3250	51.89	25.97
21491371	2011	6	9	3260	51.79	26.01
21491371	2011	6	9	3270	52.29	26.06
21491371	2011	6	9	3280	53.04	26.07
21491371	2011	6	9	3290	53.16	26.11
21491371	2011	6	9	3300	52.71	26.15
21491371	2011	6	9	3310	52.7	26.21
21491371	2011	6	9	3320	52.7	26.25
21491371	2011	6	9	3330	52.7	26.28
21491371	2011	6	9	3340	52.77	26.29
21491371	2011	6	9	3350	52.73	26.32
21491371	2011	6	9	3360	52.69	26.35
21491371	2011	6	9	3370	52.77	26.39
21491371	2011	6	9	3380	52.92	26.39
21491371	2011	6	9	3390	53.1	26.41
21491371	2011	6	9	3400	53	26.42
21491371	2011	6	9	3410	52.69	26.45
21491371	2011	6	9	3420	52.86	26.46
21491371	2011	6	9	3430	52.79	26.48
21491371	2011	6	9	3440	52.27	26.5
21491371	2011	6	9	3450	51.4	26.53
21491371	2011	6	9	3460	51.7	26.55
21491371	2011	6	9	3470	52.16	26.56
21491371	2011	6	9	3480	52.27	26.57
21491371	2011	6	9	3490	52.48	26.57
21491371	2011	6	9	3500	52.55	26.6
21491371	2011	6	9	3510	52.04	26.61
21491371	2011	6	9	3520	51.71	26.62
21491371	2011	6	9	3530	52.05	26.63
21491371	2011	6	9	3540	52.43	26.64
21491371	2011	6	9	3550	52.1	26.65

21491371	2011	6	9	3560	52.41	26.67
21491371	2011	6	9	3570	52.36	26.67
21491371	2011	6	9	3580	52.54	26.67
21491371	2011	6	9	3590	52.46	26.68
21491371	2011	6	9	3600	51.49	26.69
21491371	2011	6	9	3610	51.11	26.7
21491371	2011	6	9	3620	51.35	26.71
21491371	2011	6	9	3630	51.25	26.72
21491371	2011	6	9	3640	51.23	26.73
21491371	2011	6	9	3650	50.48	26.74
21491371	2011	6	9	3660	49.48	26.75
21491371	2011	6	9	3670	49.13	26.77
21491371	2011	6	9	3680	48.93	26.79
21491371	2011	6	9	3690	48.74	26.83
21491371	2011	6	9	3700	47.72	26.86
21491371	2011	6	9	3710	48.08	26.92
21491371	2011	6	9	3720	49.07	26.98
21491371	2011	6	9	3730	49.59	27
21491371	2011	6	9	3740	48.76	27.03
21491371	2011	6	9	3750	48.48	27.08
21491371	2011	6	9	3760	48.66	27.13
21491371	2011	6	9	3770	48.34	27.17
21491371	2011	6	9	3780	48.16	27.23
21491371	2011	6	9	3790	49.1	27.3
21491371	2011	6	9	3800	49.23	27.34
21491371	2011	6	9	3810	48.08	27.37
21491371	2011	6	9	3820	47.17	27.4
21491371	2011	6	9	3830	46.53	27.46
21491371	2011	6	9	3840	46.17	27.5
21491371	2011	6	9	3850	46.21	27.56
21491371	2011	6	9	3860	46.28	27.64
21491371	2011	6	9	3870	45.46	27.66
21491371	2011	6	9	3880	46.04	27.71
21491371	2011	6	9	3890	46.79	27.73
21491371	2011	6	9	3900	47.32	27.75
21491371	2011	6	9	3910	47.8	27.75
21491371	2011	6	9	3920	47.8	27.78
21491371	2011	6	9	3930	47.4	27.8
21491371	2011	6	9	3940	47.89	27.82
21491371	2011	6	9	3950	49.36	27.82
21491371	2011	6	9	3960	50.6	27.79
21491371	2011	6	9	3970	51.49	27.75
21491371	2011	6	9	3980	52.48	27.68
21491371	2011	6	9	3990	53.49	27.6
21491371	2011	6	9	4000	55.25	27.5
21491371	2011	6	9	4010	57.6	27.41
21491371	2011	6	9	4020	60.12	27.35

21491371	2011	6	9	4030	62.28	27.33
21491371	2011	6	9	4040	64.59	27.28
21491371	2011	6	9	4050	65.73	27.22
21491371	2011	6	9	4060	66.42	27.14
21491371	2011	6	9	4070	67.11	27.07
21491371	2011	6	9	4080	68.44	27.02
21491371	2011	6	9	4090	69.8	26.96
21491371	2011	6	9	4100	71.22	26.88
21491371	2011	6	9	4110	72.11	26.8
21491371	2011	6	9	4120	72.45	26.78
21491371	2011	6	9	4130	73.15	26.75
21491371	2011	6	9	4140	73.62	26.7
21491371	2011	6	9	4150	73.99	26.65
21491371	2011	6	9	4160	74.5	26.62
21491371	2011	6	9	4170	75.21	26.57
21491371	2011	6	9	4180	75.34	26.54
21491371	2011	6	9	4190	75.98	26.5
21491371	2011	6	9	4200	76.28	26.46
21491371	2011	6	9	4210	76.36	26.42
21491371	2011	6	9	4220	76.94	26.39
21491371	2011	6	9	4230	77.59	26.35
21491371	2011	6	9	4240	78.3	26.31
21491371	2011	6	9	4250	79.06	26.28
21491371	2011	6	9	4260	79.88	26.23
21491371	2011	6	9	4270	80.77	26.22
21491371	2011	6	9	4280	81.36	26.17
21491371	2011	6	9	4290	81.86	26.14
21491371	2011	6	9	4300	81.6	26.11
21491371	2011	6	9	4310	81.7	26.07
21491371	2011	6	9	4320	82.17	26.05
21491371	2011	6	9	4330	82.76	26.03
21491371	2011	6	9	4340	83.34	26
21491371	2011	6	9	4350	83.47	25.97
21491371	2011	6	9	4360	84.01	25.96
21491371	2011	6	9	4370	84.66	25.95
21491371	2011	6	9	4380	84.85	25.92
21491371	2011	6	9	4390	85.3	25.89
21491371	2011	6	9	4400	85.94	25.86
21491371	2011	6	9	4410	85.71	25.82
21491371	2011	6	9	4420	85.17	25.82
21491371	2011	6	9	4430	84.97	25.81
21491371	2011	6	9	4440	85.15	25.79
21491371	2011	6	9	4450	85.85	25.77
21491371	2011	6	9	4460	86.52	25.75
21491371	2011	6	9	4470	86.5	25.73
21491371	2011	6	9	4480	86.81	25.72
21491371	2011	6	9	4490	87.96	25.71

21491371	2011	6	9	4500	88.72	25.67
21491371	2011	6	9	4510	88.65	25.66
21491371	2011	6	9	4520	88.79	25.66
21491371	2011	6	9	4530	88.95	25.64
21491371	2011	6	9	4540	88.76	25.64
21491371	2011	6	9	4550	88.66	25.59
21491371	2011	6	9	4560	89.24	25.56
21491371	2011	6	9	4570	90.01	25.5
21491371	2011	6	9	4580	90.83	25.46
21491371	2011	6	9	4590	91.23	25.39
21491371	2011	6	9	4600	91.64	25.3
21491371	2011	6	9	4610	92.15	25.22
21491371	2011	6	9	4620	92.26	25.15
21491371	2011	6	9	4630	91.85	25.07
21491371	2011	6	9	4640	91.54	24.98
21491371	2011	6	9	4650	91.45	24.91
21491371	2011	6	9	4660	90.83	24.86
21491371	2011	6	9	4670	90.89	24.83
21491371	2011	6	9	4680	90.44	24.78
21491371	2011	6	9	4690	90.48	24.74
21491371	2011	6	9	4700	90.21	24.72
21491371	2011	6	9	4710	90.17	24.68
21491371	2011	6	9	4720	90.05	24.67
21491371	2011	6	9	4730	89.99	24.65
21491371	2011	6	9	4740	89.68	24.63
21491371	2011	6	9	4750	90	24.6
21491371	2011	6	9	4760	90.41	24.58
21491371	2011	6	9	4770	90.68	24.57
21491371	2011	6	9	4780	91.03	24.54
21491371	2011	6	9	4790	90.75	24.51
21491371	2011	6	9	4800	90.47	24.5
21491371	2011	6	9	4810	90.51	24.47
21491371	2011	6	9	4820	91.13	24.45
21491371	2011	6	9	4830	91.72	24.42
21491371	2011	6	9	4840	91.78	24.38
21491371	2011	6	9	4850	92.08	24.35
21491371	2011	6	9	4860	92.68	24.32
21491371	2011	6	9	4870	92.74	24.29
21491371	2011	6	9	4880	92.73	24.27
21491371	2011	6	9	4890	93.04	24.24
21491371	2011	6	9	4900	93.58	24.22
21491371	2011	6	9	4910	94.22	24.19
21491371	2011	6	9	4920	94.83	24.17
21491371	2011	6	9	4930	95.37	24.15
21491371	2011	6	9	4940	96	24.12
21491371	2011	6	9	4950	97.21	24.1
21491371	2011	6	9	4960	98.39	24.07

21491371	2011	6	9	4970	99.2	24.06
21491371	2011	6	9	4980	99.79	24.02
21491371	2011	6	9	4990	100.54	24.01
21491371	2011	6	9	5000	101.21	24.01
21491371	2011	6	9	5010	101.82	23.98
21491371	2011	6	9	5020	102.49	23.98
21491371	2011	6	9	5030	103.12	23.96
21491371	2011	6	9	5040	103.66	23.96
21491371	2011	6	9	5050	104.18	23.93
21491371	2011	6	9	5060	104.74	23.92
21491371	2011	6	9	5070	105.34	23.91
21491371	2011	6	9	5080	105.78	23.89
21491371	2011	6	9	5090	106.05	23.88
21491371	2011	6	9	5100	106.44	23.87
21491371	2011	6	9	5110	106.96	23.85
21491371	2011	6	9	5120	107.59	23.84
21491371	2011	6	9	5130	108.05	23.83
21491371	2011	6	9	5140	108.01	23.82
21491371	2011	6	9	5150	107.95	23.8
21491371	2011	6	9	5160	107.24	23.78
21491371	2011	6	9	5170	107.03	23.78
21491371	2011	6	9	5180	106.69	23.78
21491371	2011	6	9	5190	106.05	23.76
21491371	2011	6	9	5200	105.75	23.76
21491371	2011	6	9	5210	105.83	23.75
21491371	2011	6	9	5220	105.36	23.75
21491371	2011	6	9	5230	105.17	23.74
21491371	2011	6	9	5240	104.95	23.73
21491371	2011	6	9	5250	104.66	23.73
21491371	2011	6	9	5260	104.69	23.72
21491371	2011	6	9	5270	104.96	23.72
21491371	2011	6	9	5280	105.49	23.71
21491371	2011	6	9	5290	105.69	23.71
21491371	2011	6	9	5300	105.51	23.69
21491371	2011	6	9	5310	105.78	23.69
21491371	2011	6	9	5320	106.09	23.68
21491371	2011	6	9	5330	105.72	23.67
21491371	2011	6	9	5340	105.82	23.68
21491371	2011	6	9	5350	105.5	23.67
21491371	2011	6	9	5360	105.42	23.67
21491371	2011	6	9	5370	105.91	23.65
21491371	2011	6	9	5380	106.3	23.65
21491371	2011	6	9	5390	106.71	23.65
21491371	2011	6	9	5400	107.34	23.64
21491371	2011	6	9	5410	107.92	23.64
21491371	2011	6	9	5420	108.74	23.64
21491371	2011	6	9	5430	108.56	23.63

21491371	2011	6	9	5440	108.28	23.62
21491371	2011	6	9	5450	108.83	23.62
21491371	2011	6	9	5460	109.51	23.61
21491371	2011	6	9	5470	110.04	23.6
21491371	2011	6	9	5480	109.54	23.6
21491371	2011	6	9	5490	109.4	23.59
21491371	2011	6	9	5500	108.98	23.58
21491371	2011	6	9	5510	108.77	23.58
21491371	2011	6	9	5520	108.28	23.58
21491371	2011	6	9	5530	108.46	23.58
21491371	2011	6	9	5540	108.96	23.57
21491371	2011	6	9	5550	109.6	23.57
21491371	2011	6	9	5560	110.33	23.57
21491371	2011	6	9	5570	111.03	23.55
21491371	2011	6	9	5580	111.72	23.56
21491371	2011	6	9	5590	112.34	23.53
21491371	2011	6	9	5600	113.03	23.49
21491371	2011	6	9	5610	114.25	23.41
21491371	2011	6	9	5620	115.67	23.35
21491371	2011	6	9	5630	117.45	23.28
21491371	2011	6	9	5640	119.07	23.21
21491371	2011	6	9	5650	120.3	23.12
21491371	2011	6	9	5660	121.12	23.06
21491371	2011	6	9	5670	121.46	23.03
21491371	2011	6	9	5680	121.33	22.99
21491371	2011	6	9	5690	121.3	22.94
21491371	2011	6	9	5700	121.47	22.91
21491371	2011	6	9	5710	121.57	22.86
21491371	2011	6	9	5720	121.68	22.82
21491371	2011	6	9	5730	121.89	22.76
21491371	2011	6	9	5740	122.08	22.71
21491371	2011	6	9	5750	121.33	22.65
21491371	2011	6	9	5760	120.5	22.6
21491371	2011	6	9	5770	120.14	22.59
21491371	2011	6	9	5780	119.6	22.54
21491371	2011	6	9	5790	119.43	22.52
21491371	2011	6	9	5800	119.47	22.49
21491371	2011	6	9	5810	119.7	22.46
21491371	2011	6	9	5820	119.59	22.44
21491371	2011	6	9	5830	119.47	22.42
21491371	2011	6	9	5840	119.22	22.4
21491371	2011	6	9	5850	119.11	22.38
21491371	2011	6	9	5860	119.33	22.35
21491371	2011	6	9	5870	119.26	22.32
21491371	2011	6	9	5880	119.16	22.32
21491371	2011	6	9	5890	119.1	22.29
21491371	2011	6	9	5900	119.47	22.28

21491371	2011	6	9	5910	120.11	22.25
21491371	2011	6	9	5920	122.23	22.21
21491371	2011	6	9	5930	125.16	22.18
21491371	2011	6	9	5940	127.39	22.17
21491371	2011	6	9	5950	128.35	22.15
21491371	2011	6	9	5960	129.24	22.14
21491371	2011	6	9	5970	129.61	22.1
21491371	2011	6	9	5980	129.9	22.05
21491371	2011	6	9	5990	130.38	22.02
21491371	2011	6	9	6000	131.73	21.98
21491371	2011	6	9	6010	133.24	21.93
21491371	2011	6	9	6020	133.52	21.89
21491371	2011	6	9	6030	134.04	21.85
21491371	2011	6	9	6040	135.27	21.8
21491371	2011	6	9	6050	136.47	21.74
21491371	2011	6	9	6060	137.32	21.71
21491371	2011	6	9	6070	137.63	21.67
21491371	2011	6	9	6080	137.28	21.6
21491371	2011	6	9	6090	137.6	21.54
21491371	2011	6	9	6100	137.92	21.51
21491371	2011	6	9	6110	138.17	21.46
21491371	2011	6	9	6120	138.35	21.42
21491371	2011	6	9	6130	138.5	21.38
21491371	2011	6	9	6140	138.14	21.35
21491371	2011	6	9	6150	136.56	21.32
21491371	2011	6	9	6160	135.87	21.3
21491371	2011	6	9	6170	135.57	21.28
21491371	2011	6	9	6180	135.46	21.25
21491371	2011	6	9	6190	135.79	21.23
21491371	2011	6	9	6200	136.27	21.21
21491371	2011	6	9	6210	135.62	21.19
21491371	2011	6	9	6220	133.81	21.17
21491371	2011	6	9	6230	133.01	21.15
21491371	2011	6	9	6240	132.49	21.14
21491371	2011	6	9	6250	132.3	21.13
21491371	2011	6	9	6260	132.03	21.13
21491371	2011	6	9	6270	131.68	21.13
21491371	2011	6	9	6280	131.75	21.14
21491371	2011	6	9	6290	131.38	21.14
21491371	2011	6	9	6300	131.09	21.14
21491371	2011	6	9	6310	131.26	21.14
21491371	2011	6	9	6320	131.37	21.15
21491371	2011	6	9	6330	131.58	21.16
21491371	2011	6	9	6340	131.42	21.17
21491371	2011	6	9	6350	131.55	21.17
21491371	2011	6	9	6360	131.67	21.17
21491371	2011	6	9	6370	131.63	21.17

21491371	2011	6	9	6380	131.68	21.17
21491371	2011	6	9	6390	131.64	21.19
21491371	2011	6	9	6400	132.46	21.19
21491371	2011	6	9	6410	133.32	21.18
21491371	2011	6	9	6420	134.35	21.16
21491371	2011	6	9	6430	134.77	21.15
21491371	2011	6	9	6440	134.98	21.13
21491371	2011	6	9	6450	136.22	21.11
21491371	2011	6	9	6460	136.32	21.1
21491371	2011	6	9	6470	136.06	21.09
21491371	2011	6	9	6480	136.16	21.08
21491371	2011	6	9	6490	135.97	21.07
21491371	2011	6	9	6500	136	21.07
21491371	2011	6	9	6510	136.25	21.06
21491371	2011	6	9	6520	136.1	21.04
21491371	2011	6	9	6530	136.04	21.03
21491371	2011	6	9	6540	135.93	21.02
21491371	2011	6	9	6550	135.98	21.02
21491371	2011	6	9	6560	135.96	21
21491371	2011	6	9	6570	135.4	21
21491371	2011	6	9	6580	134.37	20.98
21491371	2011	6	9	6590	133.21	20.96
21491371	2011	6	9	6600	132.78	20.96
21491371	2011	6	9	6610	132.48	20.98
21491371	2011	6	9	6620	132.29	21
21491371	2011	6	9	6630	132.14	21
21491371	2011	6	9	6640	132.07	21.01
21491371	2011	6	9	6650	132	21.02
21491371	2011	6	9	6660	131.97	21.03
21491371	2011	6	9	6670	132.5	21.03
21491371	2011	6	9	6680	132.8	21.05
21491371	2011	6	9	6690	132.78	21.07
21491371	2011	6	9	6700	132.3	21.07
21491371	2011	6	9	6710	132.08	21.08
21491371	2011	6	9	6720	131.92	21.1
21491371	2011	6	9	6730	131.79	21.1
21491371	2011	6	9	6740	131.38	21.12
21491371	2011	6	9	6750	131.47	21.12
21491371	2011	6	9	6760	131.75	21.14
21491371	2011	6	9	6770	132.05	21.15
21491371	2011	6	9	6780	132.2	21.16
21491371	2011	6	9	6790	133.18	21.15
21491371	2011	6	9	6800	133.89	21.17
21491371	2011	6	9	6810	134.43	21.17
21491371	2011	6	9	6820	134.67	21.16
21491371	2011	6	9	6830	134.84	21.16
21491371	2011	6	9	6840	135.36	21.14

21491371	2011	6	9	6850	135.81	21.13
21491371	2011	6	9	6860	136	21.11
21491371	2011	6	9	6870	136.8	21.1
21491371	2011	6	9	6880	137.82	21.07
21491371	2011	6	9	6890	138	21.06
21491371	2011	6	9	6900	138.04	21.05
21491371	2011	6	9	6910	138.14	21.03
21491371	2011	6	9	6920	138.32	21.03
21491371	2011	6	9	6930	138.52	21.02
21491371	2011	6	9	6940	138.47	21
21491371	2011	6	9	6950	138.56	20.99
21491371	2011	6	9	6960	138.32	20.98
21491371	2011	6	9	6970	137.14	20.96
21491371	2011	6	9	6980	136.86	20.95
21491371	2011	6	9	6990	138.01	20.93
21491371	2011	6	9	7000	139.54	20.91
21491371	2011	6	9	7010	140.2	20.89
21491371	2011	6	9	7020	140.59	20.89
21491371	2011	6	9	7030	140.4	20.89
21491371	2011	6	9	7040	139.88	20.89
21491371	2011	6	9	7050	139.65	20.89
21491371	2011	6	9	7060	139.62	20.88
21491371	2011	6	9	7070	139.55	20.87
21491371	2011	6	9	7080	140.18	20.85
21491371	2011	6	9	7090	140.76	20.85
21491371	2011	6	9	7100	140.76	20.84
21491371	2011	6	9	7110	140.46	20.83
21491371	2011	6	9	7120	140.1	20.83
21491371	2011	6	9	7130	139.54	20.82
21491371	2011	6	9	7140	139.17	20.82
21491371	2011	6	9	7150	139.5	20.82
21491371	2011	6	9	7160	139.75	20.81
21491371	2011	6	9	7170	139.45	20.81
21491371	2011	6	9	7180	139.57	20.81
21491371	2011	6	9	7190	139.43	20.79
21491371	2011	6	9	7200	139.71	20.79
21491371	2011	6	9	7210	140.52	20.78
21491371	2011	6	9	7220	140.09	20.78
21491371	2011	6	9	7230	140.22	20.78
21491371	2011	6	9	7240	140.41	20.78
21491371	2011	6	9	7250	139.59	20.77
21491371	2011	6	9	7260	138.18	20.78
21491371	2011	6	9	7270	138.96	20.77
21491371	2011	6	9	7280	139.35	20.76
21491371	2011	6	9	7290	138.9	20.76
21491371	2011	6	9	7300	138.98	20.75
21491371	2011	6	9	7310	139.2	20.76

21491371	2011	6	9	7320	139.11	20.76
21491371	2011	6	9	7330	138.34	20.76
21491371	2011	6	9	7340	138.64	20.76
21491371	2011	6	9	7350	138.91	20.75
21491371	2011	6	9	7360	138.34	20.77
21491371	2011	6	9	7370	137.04	20.77
21491371	2011	6	9	7380	135.53	20.77
21491371	2011	6	9	7390	135.11	20.77
21491371	2011	6	9	7400	134.61	20.78
21491371	2011	6	9	7410	133.77	20.78
21491371	2011	6	9	7420	132.29	20.8
21491371	2011	6	9	7430	131.16	20.83
21491371	2011	6	9	7440	130.43	20.89
21491371	2011	6	9	7450	129.41	20.91
21491371	2011	6	9	7460	129.34	20.92
21491371	2011	6	9	7470	128.44	20.96
21491371	2011	6	9	7480	126.96	20.99
21491371	2011	6	9	7490	125.65	21.02
21491371	2011	6	9	7500	124.37	21.07
21491371	2011	6	9	7510	122.27	21.12
21491371	2011	6	9	7520	120.82	21.15
21491371	2011	6	9	7530	120.48	21.22
21491371	2011	6	9	7540	120.58	21.3
21491371	2011	6	9	7550	120.72	21.35
21491371	2011	6	9	7560	120.19	21.39
21491371	2011	6	9	7570	120.07	21.44
21491371	2011	6	9	7580	119.75	21.48
21491371	2011	6	9	7590	119.66	21.57
21491371	2011	6	9	7600	119.82	21.6
21491371	2011	6	9	7610	120.14	21.66
21491371	2011	6	9	7620	120.35	21.7
21491371	2011	6	9	7630	119.72	21.75
21491371	2011	6	9	7640	119.06	21.83
21491371	2011	6	9	7650	118.75	21.88
21491371	2011	6	9	7660	118.76	21.92
21491371	2011	6	9	7670	118.76	21.99
21491371	2011	6	9	7680	118.74	22.07
21491371	2011	6	9	7690	119.16	22.1
21491371	2011	6	9	7700	119.58	22.15
21491371	2011	6	9	7710	119.77	22.19
21491371	2011	6	9	7720	119.97	22.21
21491371	2011	6	9	7730	120.11	22.25
21491371	2011	6	9	7740	119.21	22.29
21491371	2011	6	9	7750	117.54	22.33
21491371	2011	6	9	7760	115.78	22.37
21491371	2011	6	9	7770	113.75	22.4
21491371	2011	6	9	7780	111.91	22.46

21491371	2011	6	9	7790	110.87	22.52
21491371	2011	6	9	7800	108.95	22.56
21491371	2011	6	9	7810	109.33	22.66
21491371	2011	6	9	7820	110.14	22.69
21491371	2011	6	9	7830	110.18	22.7
21491371	2011	6	9	7840	108.81	22.74
21491371	2011	6	9	7850	106.55	22.78
21491371	2011	6	9	7860	104.11	22.82
21491371	2011	6	9	7870	101.79	22.85
21491371	2011	6	9	7880	99.73	22.88
21491371	2011	6	9	7890	97.86	22.92
21491371	2011	6	9	7900	95.97	22.96
21491371	2011	6	9	7910	94.66	23
21491371	2011	6	9	7920	93.25	23.05
21491371	2011	6	9	7930	91.18	23.09
21491371	2011	6	9	7940	88.6	23.12
21491371	2011	6	9	7950	86.02	23.15
21491371	2011	6	9	7960	83.48	23.21
21491371	2011	6	9	7970	81.1	23.25
21491371	2011	6	9	7980	79.09	23.31
21491371	2011	6	9	7990	77.43	23.37
21491371	2011	6	9	8000	75.94	23.47
21491371	2011	6	9	8010	73.73	23.57
21491371	2011	6	9	8020	71.22	23.65
21491371	2011	6	9	8030	68.83	23.75
21491371	2011	6	9	8040	67.1	23.9
21491371	2011	6	9	8050	65.87	23.99
21491371	2011	6	9	8060	65.7	24.14
21491371	2011	6	9	8070	65.85	24.19
21491371	2011	6	9	8080	65.59	24.25
21491371	2011	6	9	8090	64.96	24.35
21491371	2011	6	9	8100	65.14	24.41
21491371	2011	6	9	8110	65.6	24.48
21491371	2011	6	9	8120	65.35	24.57
21491371	2011	6	9	8130	65.42	24.64
21491371	2011	6	9	8140	65.56	24.75
21491371	2011	6	9	8150	64.42	24.81
21491371	2011	6	9	8160	63.85	24.85
21491371	2011	6	9	8170	64.11	24.94
21491371	2011	6	9	8180	64.5	24.96
21491371	2011	6	9	8190	64.9	25
21491371	2011	6	9	8200	64.82	25.03
21491371	2011	6	9	8210	64.74	25.07
21491371	2011	6	9	8220	64.78	25.12
21491371	2011	6	9	8230	63.93	25.17
21491371	2011	6	9	8240	63.71	25.21
21491371	2011	6	9	8250	63.43	25.26

21491371	2011	6	9	8260	63.38	25.29
21491371	2011	6	9	8270	63.48	25.33
21491371	2011	6	9	8280	63.49	25.36
21491371	2011	6	9	8290	63.1	25.38
21491371	2011	6	9	8300	62.41	25.42
21491371	2011	6	9	8310	61.1	25.44
21491371	2011	6	9	8320	60.91	25.47
21491371	2011	6	9	8330	60.71	25.5
21491371	2011	6	9	8340	59.13	25.52
21491371	2011	6	9	8350	57.94	25.54
21491371	2011	6	9	8360	56.24	25.59
21491371	2011	6	9	8370	54.33	25.63
21491371	2011	6	9	8380	52.73	25.68
21491371	2011	6	9	8390	50.7	25.74
21491371	2011	6	9	8400	48.83	25.82
21491371	2011	6	9	8410	47	25.89
21491371	2011	6	9	8420	45.23	25.99
21491371	2011	6	9	8430	43.7	26.12
21491371	2011	6	9	8440	42.64	26.26
21491371	2011	6	9	8450	42.96	26.4
21491371	2011	6	9	8460	42.95	26.5
21491371	2011	6	9	8470	41.79	26.61
21491371	2011	6	9	8480	40.25	26.72
21491371	2011	6	9	8490	39.01	26.82
21491371	2011	6	9	8500	38.45	26.92
21491371	2011	6	9	8510	37.09	27
21491371	2011	6	9	8520	35.7	27.11
21491371	2011	6	9	8530	34.46	27.17
21491371	2011	6	9	8540	32.87	27.25
21491371	2011	6	9	8550	31.18	27.32
21491371	2011	6	9	8560	29.27	27.4
21491371	2011	6	9	8570	27.23	27.5
21491371	2011	6	9	8580	25.07	27.57
21491371	2011	6	9	8590	23.22	27.64
21491371	2011	6	9	8600	22.67	27.71
21491371	2011	6	9	8610	22.57	27.82
21491371	2011	6	9	8620	21.5	27.85
21491371	2011	6	9	8630	19.93	27.92
21491371	2011	6	9	8640	18.23	27.96
21491371	2011	6	9	8650	17.16	28.03
21491371	2011	6	9	8660	15.48	28.06
21491371	2011	6	9	8670	13.59	28.11
21491371	2011	6	9	8680	11.8	28.16
21491371	2011	6	9	8690	10.42	28.22
21491371	2011	6	9	8700	8.97	28.27
21491371	2011	6	9	8710	7.85	28.32
21491371	2011	6	9	8720	6.54	28.36

21491371	2011	6	9	8730	6.08	28.39
21491371	2011	6	9	8740	5.08	28.44
21491371	2011	6	9	8750	3.6	28.48
21491371	2011	6	9	8760	3.32	28.5
21491371	2011	6	9	8770	3.43	28.53
21491371	2011	6	9	8780	2.74	28.55
21491371	2011	6	9	8790	2.79	28.57
21491371	2011	6	9	8800	2.93	28.59
21491371	2011	6	9	8810	2.5	28.63
21491371	2011	6	9	8820	0.62	28.69
21491371	2011	6	9	8830	0.61	28.73
21491371	2011	6	9	8840	0.6	28.74
21491371	2011	6	9	8850	0.6	28.75
21491371	2011	6	9	8860	0.59	28.76
21491371	2011	6	9	8870	0.61	28.79
21491371	2011	6	9	8880	0.59	28.82
21491371	2011	6	9	8890	0.6	28.85
21491371	2011	6	9	8900	0.64	28.85
21491371	2011	6	9	8910	0.61	28.87
21491371	2011	6	9	8920	0.66	28.89
21491371	2011	6	9	8930	0.6	28.92
21491371	2011	6	9	8940	0.51	28.92
21491371	2011	6	9	8950	0.5	28.92
21491371	2011	6	9	8960	0.53	28.94
21585781	2011	6	10	0	1.31	29.02
21585781	2011	6	10	10	2.21	29.01
21585781	2011	6	10	20	2.22	29.01
21585781	2011	6	10	30	2.38	29.01
21585781	2011	6	10	40	2.95	29
21585781	2011	6	10	50	2.79	29.01
21585781	2011	6	10	60	3.03	29
21585781	2011	6	10	70	3.92	29
21585781	2011	6	10	80	5.69	28.97
21585781	2011	6	10	90	7	28.96
21585781	2011	6	10	100	9.12	28.94
21585781	2011	6	10	110	10.23	28.93
21585781	2011	6	10	120	10.42	28.93
21585781	2011	6	10	130	10.39	28.92
21585781	2011	6	10	140	10.31	28.92
21585781	2011	6	10	150	10.13	28.92
21585781	2011	6	10	160	9.89	28.92
21585781	2011	6	10	170	9.58	28.92
21585781	2011	6	10	180	9.59	28.93
21585781	2011	6	10	190	9.71	28.92
21585781	2011	6	10	200	10.4	28.92
21585781	2011	6	10	210	10.87	28.92
21585781	2011	6	10	220	12.39	28.9

21585781	2011	6	10	230	14.64	28.89
21585781	2011	6	10	240	16.81	28.87
21585781	2011	6	10	250	18.34	28.85
21585781	2011	6	10	260	19.65	28.84
21585781	2011	6	10	270	20.69	28.83
21585781	2011	6	10	280	22.81	28.81
21585781	2011	6	10	290	24.9	28.79
21585781	2011	6	10	300	26.3	28.78
21585781	2011	6	10	310	28.29	28.76
21585781	2011	6	10	320	29.83	28.75
21585781	2011	6	10	330	30.98	28.74
21585781	2011	6	10	340	32.61	28.72
21585781	2011	6	10	350	34.92	28.7
21585781	2011	6	10	360	37.44	28.67
21585781	2011	6	10	370	40.3	28.65
21585781	2011	6	10	380	43.21	28.61
21585781	2011	6	10	390	46.09	28.55
21585781	2011	6	10	400	48.92	28.46
21585781	2011	6	10	410	51.37	28.3
21585781	2011	6	10	420	53.89	28.11
21585781	2011	6	10	430	55.7	27.96
21585781	2011	6	10	440	56.77	27.92
21585781	2011	6	10	450	57.57	27.89
21585781	2011	6	10	460	58.91	27.66
21585781	2011	6	10	470	60.94	27.52
21585781	2011	6	10	480	63.34	27.39
21585781	2011	6	10	490	65.96	27.28
21585781	2011	6	10	500	68.16	27.21
21585781	2011	6	10	510	69.36	27.16
21585781	2011	6	10	520	71.27	27.06
21585781	2011	6	10	530	72.24	27
21585781	2011	6	10	540	72.56	26.96
21585781	2011	6	10	550	72.49	26.92
21585781	2011	6	10	560	72.53	26.83
21585781	2011	6	10	570	73.36	26.77
21585781	2011	6	10	580	74.18	26.69
21585781	2011	6	10	590	75.29	26.6
21585781	2011	6	10	600	75.62	26.55
21585781	2011	6	10	610	75.67	26.52
21585781	2011	6	10	620	76.07	26.48
21585781	2011	6	10	630	77.36	26.42
21585781	2011	6	10	640	78.1	26.35
21585781	2011	6	10	650	78.58	26.32
21585781	2011	6	10	660	79.19	26.27
21585781	2011	6	10	670	79.53	26.24
21585781	2011	6	10	680	80.02	26.19
21585781	2011	6	10	690	80.57	26.16

21585781	2011	6	10	700	82.09	26.09
21585781	2011	6	10	710	82.88	26.07
21585781	2011	6	10	720	83.58	26.03
21585781	2011	6	10	730	85.56	25.96
21585781	2011	6	10	740	87.06	25.94
21585781	2011	6	10	750	87.66	25.89
21585781	2011	6	10	760	88.32	25.79
21585781	2011	6	10	770	89.83	25.67
21585781	2011	6	10	780	91.34	25.56
21585781	2011	6	10	790	93.06	25.37
21585781	2011	6	10	800	94.63	25.25
21585781	2011	6	10	810	96.82	25.07
21585781	2011	6	10	820	98.65	24.96
21585781	2011	6	10	830	99.61	24.92
21585781	2011	6	10	840	101.6	24.84
21585781	2011	6	10	850	104.03	24.75
21585781	2011	6	10	860	106.26	24.67
21585781	2011	6	10	870	108.14	24.57
21585781	2011	6	10	880	110.01	24.48
21585781	2011	6	10	890	112.08	24.37
21585781	2011	6	10	900	114.11	24.23
21585781	2011	6	10	910	116.48	24.14
21585781	2011	6	10	920	117.38	24.08
21585781	2011	6	10	930	117.62	24.07
21585781	2011	6	10	940	117.56	23.96
21585781	2011	6	10	950	117.76	23.85
21585781	2011	6	10	960	118.04	23.82
21585781	2011	6	10	970	118.48	23.82
21585781	2011	6	10	980	118.91	23.79
21585781	2011	6	10	990	119.46	23.76
21585781	2011	6	10	1000	120.07	23.71
21585781	2011	6	10	1010	120.78	23.63
21585781	2011	6	10	1020	120.76	23.59
21585781	2011	6	10	1030	120.76	23.55
21585781	2011	6	10	1040	120.99	23.5
21585781	2011	6	10	1050	121.95	23.43
21585781	2011	6	10	1060	122.29	23.39
21585781	2011	6	10	1070	122.04	23.36
21585781	2011	6	10	1080	121.67	23.34
21585781	2011	6	10	1090	121.02	23.32
21585781	2011	6	10	1100	120.49	23.27
21585781	2011	6	10	1110	120.01	23.23
21585781	2011	6	10	1120	119.95	23.18
21585781	2011	6	10	1130	119.99	23.16
21585781	2011	6	10	1140	120.94	23.14
21585781	2011	6	10	1150	122.12	23.1
21585781	2011	6	10	1160	122.31	23.07

21585781	2011	6	10	1170	123.24	23.07
21585781	2011	6	10	1180	124.97	23.02
21585781	2011	6	10	1190	125.54	23
21585781	2011	6	10	1200	125.39	22.99
21585781	2011	6	10	1210	125.5	22.99
21585781	2011	6	10	1220	126.89	22.93
21585781	2011	6	10	1230	127.44	22.92
21585781	2011	6	10	1240	128.79	22.86
21585781	2011	6	10	1250	130.49	22.84
21585781	2011	6	10	1260	132.18	22.8
21585781	2011	6	10	1270	133.27	22.77
21585781	2011	6	10	1280	133.97	22.7
21585781	2011	6	10	1290	134.35	22.66
21585781	2011	6	10	1300	134.64	22.62
21585781	2011	6	10	1310	134.46	22.57
21585781	2011	6	10	1320	134.6	22.47
21585781	2011	6	10	1330	134.55	22.45
21585781	2011	6	10	1340	134.22	22.4
21585781	2011	6	10	1350	133.46	22.39
21585781	2011	6	10	1360	133.02	22.37
21585781	2011	6	10	1370	131.97	22.35
21585781	2011	6	10	1380	130.87	22.32
21585781	2011	6	10	1390	130.14	22.29
21585781	2011	6	10	1400	130.02	22.23
21585781	2011	6	10	1410	130.26	22.19
21585781	2011	6	10	1420	130.72	22.15
21585781	2011	6	10	1430	130.96	22.13
21585781	2011	6	10	1440	130.82	22.1
21585781	2011	6	10	1450	130.68	22.1
21585781	2011	6	10	1460	130.79	22.1
21585781	2011	6	10	1470	130.68	22.08
21585781	2011	6	10	1480	130.57	22.04
21585781	2011	6	10	1490	131.19	22.04
21585781	2011	6	10	1500	131.77	22.03
21585781	2011	6	10	1510	132.75	22.01
21585781	2011	6	10	1520	132.95	22
21585781	2011	6	10	1530	132.97	21.96
21585781	2011	6	10	1540	132.71	21.96
21585781	2011	6	10	1550	132.95	21.95
21585781	2011	6	10	1560	133.26	21.92
21585781	2011	6	10	1570	133.27	21.92
21585781	2011	6	10	1580	133.53	21.91
21585781	2011	6	10	1590	133.83	21.91
21585781	2011	6	10	1600	133.58	21.89
21585781	2011	6	10	1610	133.05	21.89
21585781	2011	6	10	1620	132.88	21.89
21585781	2011	6	10	1630	132.56	21.88

21585781	2011	6	10	1640	132.21	21.86
21585781	2011	6	10	1650	132.14	21.86
21585781	2011	6	10	1660	132.04	21.86
21585781	2011	6	10	1670	131.81	21.87
21585781	2011	6	10	1680	131.68	21.87
21585781	2011	6	10	1690	131.67	21.87
21585781	2011	6	10	1700	131.43	21.88
21585781	2011	6	10	1710	131.08	21.88
21585781	2011	6	10	1720	131.2	21.87
21585781	2011	6	10	1730	131.11	21.87
21585781	2011	6	10	1740	131.05	21.88
21585781	2011	6	10	1750	131.01	21.89
21585781	2011	6	10	1760	130.86	21.88
21585781	2011	6	10	1770	130.68	21.89
21585781	2011	6	10	1780	130.4	21.89
21585781	2011	6	10	1790	130.51	21.89
21585781	2011	6	10	1800	130.85	21.9
21585781	2011	6	10	1810	130.65	21.9
21585781	2011	6	10	1820	130.89	21.9
21585781	2011	6	10	1830	131.98	21.9
21585781	2011	6	10	1840	132.35	21.9
21585781	2011	6	10	1850	132.31	21.89
21585781	2011	6	10	1860	131.99	21.91
21585781	2011	6	10	1870	131.82	21.92
21585781	2011	6	10	1880	131.97	21.91
21585781	2011	6	10	1890	131.78	21.9
21585781	2011	6	10	1900	131.46	21.91
21585781	2011	6	10	1910	130.94	21.91
21585781	2011	6	10	1920	130.25	21.9
21585781	2011	6	10	1930	130.3	21.9
21585781	2011	6	10	1940	130.11	21.89
21585781	2011	6	10	1950	129.51	21.89
21585781	2011	6	10	1960	128.76	21.89
21585781	2011	6	10	1970	127.92	21.89
21585781	2011	6	10	1980	127.32	21.9
21585781	2011	6	10	1990	127.47	21.92
21585781	2011	6	10	2000	127.41	21.92
21585781	2011	6	10	2010	127.37	21.92
21585781	2011	6	10	2020	127.75	21.95
21585781	2011	6	10	2030	127.82	21.96
21585781	2011	6	10	2040	127.85	21.97
21585781	2011	6	10	2050	127.74	21.98
21585781	2011	6	10	2060	128.12	21.98
21585781	2011	6	10	2070	127.94	21.98
21585781	2011	6	10	2080	127.86	22
21585781	2011	6	10	2090	127.76	22
21585781	2011	6	10	2100	127.84	22.01

21585781	2011	6	10	2110	127.67	22.04
21585781	2011	6	10	2120	127.6	22.04
21585781	2011	6	10	2130	127.45	22.05
21585781	2011	6	10	2140	127.2	22.06
21585781	2011	6	10	2150	127.27	22.06
21585781	2011	6	10	2160	127.19	22.07
21585781	2011	6	10	2170	127.17	22.08
21585781	2011	6	10	2180	127.3	22.1
21585781	2011	6	10	2190	127.01	22.11
21585781	2011	6	10	2200	126.12	22.12
21585781	2011	6	10	2210	125.24	22.13
21585781	2011	6	10	2220	124.16	22.14
21585781	2011	6	10	2230	123.62	22.18
21585781	2011	6	10	2240	123.72	22.21
21585781	2011	6	10	2250	123.64	22.22
21585781	2011	6	10	2260	123.51	22.24
21585781	2011	6	10	2270	123.34	22.26
21585781	2011	6	10	2280	123.11	22.3
21585781	2011	6	10	2290	122.98	22.32
21585781	2011	6	10	2300	123.1	22.33
21585781	2011	6	10	2310	122.98	22.34
21585781	2011	6	10	2320	122.78	22.37
21585781	2011	6	10	2330	122.96	22.4
21585781	2011	6	10	2340	123.07	22.42
21585781	2011	6	10	2350	123.04	22.43
21585781	2011	6	10	2360	123.41	22.44
21585781	2011	6	10	2370	123.7	22.46
21585781	2011	6	10	2380	124.06	22.46
21585781	2011	6	10	2390	124.42	22.47
21585781	2011	6	10	2400	124.93	22.49
21585781	2011	6	10	2410	124.99	22.5
21585781	2011	6	10	2420	125.06	22.5
21585781	2011	6	10	2430	125.01	22.52
21585781	2011	6	10	2440	125.12	22.52
21585781	2011	6	10	2450	125.24	22.53
21585781	2011	6	10	2460	124.98	22.53
21585781	2011	6	10	2470	124.91	22.53
21585781	2011	6	10	2480	124.55	22.54
21585781	2011	6	10	2490	124.71	22.53
21585781	2011	6	10	2500	124.72	22.53
21585781	2011	6	10	2510	124.71	22.54
21585781	2011	6	10	2520	124.5	22.54
21585781	2011	6	10	2530	124.44	22.54
21585781	2011	6	10	2540	125.2	22.55
21585781	2011	6	10	2550	126.26	22.54
21585781	2011	6	10	2560	126.71	22.53
21585781	2011	6	10	2570	126.81	22.52

21585781	2011	6	10	2580	127	22.51
21585781	2011	6	10	2590	126.49	22.51
21585781	2011	6	10	2600	125.15	22.51
21585781	2011	6	10	2610	124.54	22.53
21585781	2011	6	10	2620	123.25	22.54
21585781	2011	6	10	2630	121.86	22.55
21585781	2011	6	10	2640	120.99	22.56
21585781	2011	6	10	2650	120.73	22.56
21585781	2011	6	10	2660	119.95	22.57
21585781	2011	6	10	2670	119.24	22.6
21585781	2011	6	10	2680	118.73	22.6
21585781	2011	6	10	2690	118.48	22.61
21585781	2011	6	10	2700	118.9	22.62
21585781	2011	6	10	2710	118.86	22.64
21585781	2011	6	10	2720	118.64	22.64
21585781	2011	6	10	2730	118.37	22.65
21585781	2011	6	10	2740	118.16	22.65
21585781	2011	6	10	2750	118.22	22.66
21585781	2011	6	10	2760	118.22	22.66
21585781	2011	6	10	2770	118.04	22.68
21585781	2011	6	10	2780	117.72	22.69
21585781	2011	6	10	2790	117.55	22.7
21585781	2011	6	10	2800	117.65	22.7
21585781	2011	6	10	2810	117.75	22.7
21585781	2011	6	10	2820	118.01	22.71
21585781	2011	6	10	2830	118.17	22.72
21585781	2011	6	10	2840	118.47	22.72
21585781	2011	6	10	2850	118.73	22.73
21585781	2011	6	10	2860	118.71	22.74
21585781	2011	6	10	2870	118.7	22.73
21585781	2011	6	10	2880	118.47	22.75
21585781	2011	6	10	2890	118.11	22.75
21585781	2011	6	10	2900	117.55	22.75
21585781	2011	6	10	2910	116.86	22.75
21585781	2011	6	10	2920	116.11	22.75
21585781	2011	6	10	2930	115.95	22.77
21585781	2011	6	10	2940	115.71	22.77
21585781	2011	6	10	2950	115.18	22.77
21585781	2011	6	10	2960	114.5	22.78
21585781	2011	6	10	2970	113.83	22.78
21585781	2011	6	10	2980	113.06	22.78
21585781	2011	6	10	2990	112.29	22.8
21585781	2011	6	10	3000	111.45	22.8
21585781	2011	6	10	3010	110.64	22.81
21585781	2011	6	10	3020	110.08	22.82
21585781	2011	6	10	3030	109.8	22.82
21585781	2011	6	10	3040	109.27	22.82

21585781	2011	6	10	3050	109.24	22.83
21585781	2011	6	10	3060	109.14	22.83
21585781	2011	6	10	3070	109.14	22.84
21585781	2011	6	10	3080	109.18	22.85
21585781	2011	6	10	3090	109.05	22.84
21585781	2011	6	10	3100	108.64	22.85
21585781	2011	6	10	3110	108.33	22.85
21585781	2011	6	10	3120	108.53	22.85
21585781	2011	6	10	3130	108.68	22.86
21585781	2011	6	10	3140	108.88	22.86
21585781	2011	6	10	3150	108.86	22.86
21585781	2011	6	10	3160	109.01	22.86
21585781	2011	6	10	3170	109.01	22.86
21585781	2011	6	10	3180	108.79	22.87
21585781	2011	6	10	3190	108.87	22.88
21585781	2011	6	10	3200	108.83	22.88
21585781	2011	6	10	3210	108.79	22.89
21585781	2011	6	10	3220	108.69	22.88
21585781	2011	6	10	3230	108.4	22.88
21585781	2011	6	10	3240	108.1	22.89
21585781	2011	6	10	3250	107.51	22.89
21585781	2011	6	10	3260	106.38	22.89
21585781	2011	6	10	3270	106.14	22.9
21585781	2011	6	10	3280	104.99	22.91
21585781	2011	6	10	3290	103.81	22.92
21585781	2011	6	10	3300	102.63	22.92
21585781	2011	6	10	3310	101.99	22.92
21585781	2011	6	10	3320	101.83	22.93
21585781	2011	6	10	3330	101.38	22.94
21585781	2011	6	10	3340	100.29	22.95
21585781	2011	6	10	3350	98.99	22.96
21585781	2011	6	10	3360	96.76	22.98
21585781	2011	6	10	3370	95.46	23
21585781	2011	6	10	3380	94.34	23.02
21585781	2011	6	10	3390	93.17	23.03
21585781	2011	6	10	3400	91.57	23.07
21585781	2011	6	10	3410	90.03	23.08
21585781	2011	6	10	3420	88.72	23.1
21585781	2011	6	10	3430	87.66	23.13
21585781	2011	6	10	3440	86.74	23.14
21585781	2011	6	10	3450	86.5	23.18
21585781	2011	6	10	3460	86.69	23.26
21585781	2011	6	10	3470	86.65	23.3
21585781	2011	6	10	3480	85.87	23.33
21585781	2011	6	10	3490	85.19	23.36
21585781	2011	6	10	3500	84.52	23.41
21585781	2011	6	10	3510	83.96	23.46

21585781	2011	6	10	3520	82.72	23.5
21585781	2011	6	10	3530	82.39	23.53
21585781	2011	6	10	3540	82.22	23.56
21585781	2011	6	10	3550	82.26	23.59
21585781	2011	6	10	3560	82.21	23.61
21585781	2011	6	10	3570	82.41	23.64
21585781	2011	6	10	3580	82.79	23.69
21585781	2011	6	10	3590	82.64	23.71
21585781	2011	6	10	3600	82.25	23.75
21585781	2011	6	10	3610	81.77	23.78
21585781	2011	6	10	3620	81.27	23.82
21585781	2011	6	10	3630	81.03	23.85
21585781	2011	6	10	3640	80.24	23.91
21585781	2011	6	10	3650	78.92	23.94
21585781	2011	6	10	3660	77.31	23.97
21585781	2011	6	10	3670	75.91	24
21585781	2011	6	10	3680	74.43	24.04
21585781	2011	6	10	3690	73.56	24.09
21585781	2011	6	10	3700	73.07	24.15
21585781	2011	6	10	3710	73.43	24.19
21585781	2011	6	10	3720	73.49	24.25
21585781	2011	6	10	3730	73.33	24.29
21585781	2011	6	10	3740	73.16	24.32
21585781	2011	6	10	3750	73.42	24.35
21585781	2011	6	10	3760	73.47	24.39
21585781	2011	6	10	3770	73.2	24.43
21585781	2011	6	10	3780	73.11	24.49
21585781	2011	6	10	3790	72.79	24.51
21585781	2011	6	10	3800	72.42	24.54
21585781	2011	6	10	3810	72.08	24.57
21585781	2011	6	10	3820	71.46	24.6
21585781	2011	6	10	3830	71.12	24.63
21585781	2011	6	10	3840	71.18	24.67
21585781	2011	6	10	3850	70.97	24.71
21585781	2011	6	10	3860	70.76	24.72
21585781	2011	6	10	3870	69.95	24.74
21585781	2011	6	10	3880	69.59	24.77
21585781	2011	6	10	3890	69.51	24.79
21585781	2011	6	10	3900	69.38	24.82
21585781	2011	6	10	3910	69.22	24.83
21585781	2011	6	10	3920	69.17	24.85
21585781	2011	6	10	3930	69.08	24.87
21585781	2011	6	10	3940	69.04	24.89
21585781	2011	6	10	3950	69.02	24.91
21585781	2011	6	10	3960	68.98	24.92
21585781	2011	6	10	3970	69.14	24.94
21585781	2011	6	10	3980	69.26	24.96

21585781	2011	6	10	3990	69.3	24.97
21585781	2011	6	10	4000	69.21	24.98
21585781	2011	6	10	4010	69.34	25
21585781	2011	6	10	4020	69.21	25
21585781	2011	6	10	4030	69.34	25.02
21585781	2011	6	10	4040	69.53	25.03
21585781	2011	6	10	4050	69.46	25.05
21585781	2011	6	10	4060	69.28	25.06
21585781	2011	6	10	4070	69.04	25.08
21585781	2011	6	10	4080	68.99	25.1
21585781	2011	6	10	4090	68.96	25.1
21585781	2011	6	10	4100	69.11	25.13
21585781	2011	6	10	4110	69.08	25.14
21585781	2011	6	10	4120	68.87	25.15
21585781	2011	6	10	4130	68.83	25.16
21585781	2011	6	10	4140	68.9	25.17
21585781	2011	6	10	4150	68.82	25.18
21585781	2011	6	10	4160	68.56	25.2
21585781	2011	6	10	4170	68.25	25.21
21585781	2011	6	10	4180	68.15	25.22
21585781	2011	6	10	4190	68.58	25.24
21585781	2011	6	10	4200	68.96	25.25
21585781	2011	6	10	4210	69.08	25.26
21585781	2011	6	10	4220	69.12	25.26
21585781	2011	6	10	4230	69.11	25.27
21585781	2011	6	10	4240	68.94	25.28
21585781	2011	6	10	4250	68.76	25.28
21585781	2011	6	10	4260	68.66	25.29
21585781	2011	6	10	4270	68.64	25.28
21585781	2011	6	10	4280	68.64	25.3
21585781	2011	6	10	4290	68.53	25.3
21585781	2011	6	10	4300	68.35	25.31
21585781	2011	6	10	4310	68.05	25.32
21585781	2011	6	10	4320	66.87	25.33
21585781	2011	6	10	4330	65.83	25.34
21585781	2011	6	10	4340	65.48	25.35
21585781	2011	6	10	4350	65.23	25.38
21585781	2011	6	10	4360	65.01	25.41
21585781	2011	6	10	4370	64.92	25.42
21585781	2011	6	10	4380	65.05	25.44
21585781	2011	6	10	4390	66.38	25.46
21585781	2011	6	10	4400	67.46	25.47
21585781	2011	6	10	4410	68.06	25.47
21585781	2011	6	10	4420	69.07	25.48
21585781	2011	6	10	4430	69.22	25.48
21585781	2011	6	10	4440	68.66	25.49
21585781	2011	6	10	4450	67.79	25.5

21585781	2011	6	10	4460	65.67	25.5
21585781	2011	6	10	4470	64.12	25.51
21585781	2011	6	10	4480	62.72	25.53
21585781	2011	6	10	4490	60.5	25.54
21585781	2011	6	10	4500	58.49	25.57
21585781	2011	6	10	4510	57.08	25.6
21585781	2011	6	10	4520	56.1	25.63
21585781	2011	6	10	4530	56.15	25.65
21585781	2011	6	10	4540	56.29	25.68
21585781	2011	6	10	4550	56.25	25.7
21585781	2011	6	10	4560	56.12	25.72
21585781	2011	6	10	4570	55.93	25.75
21585781	2011	6	10	4580	55.71	25.76
21585781	2011	6	10	4590	55.73	25.78
21585781	2011	6	10	4600	55.67	25.8
21585781	2011	6	10	4610	55.6	25.82
21585781	2011	6	10	4620	55.4	25.84
21585781	2011	6	10	4630	54.13	25.87
21585781	2011	6	10	4640	52.17	25.92
21585781	2011	6	10	4650	51.16	25.94
21585781	2011	6	10	4660	49.52	25.98
21585781	2011	6	10	4670	48.32	26.03
21585781	2011	6	10	4680	46.35	26.07
21585781	2011	6	10	4690	45.78	26.1
21585781	2011	6	10	4700	45.56	26.14
21585781	2011	6	10	4710	45.56	26.21
21585781	2011	6	10	4720	45.56	26.22
21585781	2011	6	10	4730	45.69	26.25
21585781	2011	6	10	4740	45.9	26.26
21585781	2011	6	10	4750	46.27	26.28
21585781	2011	6	10	4760	46.58	26.31
21585781	2011	6	10	4770	46.83	26.33
21585781	2011	6	10	4780	46.76	26.36
21585781	2011	6	10	4790	47.28	26.41
21585781	2011	6	10	4800	47.24	26.42
21585781	2011	6	10	4810	47.2	26.43
21585781	2011	6	10	4820	46.91	26.45
21585781	2011	6	10	4830	46.3	26.46
21585781	2011	6	10	4840	46.19	26.48
21585781	2011	6	10	4850	46.32	26.5
21585781	2011	6	10	4860	46.82	26.51
21585781	2011	6	10	4870	47.71	26.52
21585781	2011	6	10	4880	48.35	26.52
21585781	2011	6	10	4890	48.78	26.54
21585781	2011	6	10	4900	48.88	26.54
21585781	2011	6	10	4910	49.04	26.56
21585781	2011	6	10	4920	49.16	26.57

21585781	2011	6	10	4930	49.19	26.57
21585781	2011	6	10	4940	49.06	26.58
21585781	2011	6	10	4950	49.11	26.58
21585781	2011	6	10	4960	49.11	26.6
21585781	2011	6	10	4970	49.28	26.6
21585781	2011	6	10	4980	49.05	26.6
21585781	2011	6	10	4990	48.67	26.62
21585781	2011	6	10	5000	48.56	26.62
21585781	2011	6	10	5010	48.74	26.63
21585781	2011	6	10	5020	48.64	26.63
21585781	2011	6	10	5030	48.27	26.64
21585781	2011	6	10	5040	48.16	26.64
21585781	2011	6	10	5050	48.16	26.65
21585781	2011	6	10	5060	47.68	26.66
21585781	2011	6	10	5070	47.83	26.67
21585781	2011	6	10	5080	47.83	26.67
21585781	2011	6	10	5090	47.84	26.67
21585781	2011	6	10	5100	47.93	26.68
21585781	2011	6	10	5110	48	26.68
21585781	2011	6	10	5120	47.93	26.69
21585781	2011	6	10	5130	47.84	26.71
21585781	2011	6	10	5140	47.86	26.7
21585781	2011	6	10	5150	47.28	26.71
21585781	2011	6	10	5160	45.92	26.72
21585781	2011	6	10	5170	44.42	26.74
21585781	2011	6	10	5180	43.36	26.75
21585781	2011	6	10	5190	42.72	26.76
21585781	2011	6	10	5200	42.45	26.76
21585781	2011	6	10	5210	42.22	26.78
21585781	2011	6	10	5220	41.12	26.78
21585781	2011	6	10	5230	40.33	26.8
21585781	2011	6	10	5240	38.59	26.82
21585781	2011	6	10	5250	37.68	26.89
21585781	2011	6	10	5260	37.19	26.97
21585781	2011	6	10	5270	36.76	27.04
21585781	2011	6	10	5280	35.2	27.12
21585781	2011	6	10	5290	33.35	27.19
21585781	2011	6	10	5300	31.77	27.33
21585781	2011	6	10	5310	30.68	27.42
21585781	2011	6	10	5320	29.24	27.5
21585781	2011	6	10	5330	28.25	27.58
21585781	2011	6	10	5340	28.43	27.67
21585781	2011	6	10	5350	27.56	27.74
21585781	2011	6	10	5360	26.32	27.8
21585781	2011	6	10	5370	24.64	27.85
21585781	2011	6	10	5380	23.15	27.94
21585781	2011	6	10	5390	21.21	27.99

21585781	2011	6	10	5400	19.28	28.04
21585781	2011	6	10	5410	17.7	28.12
21585781	2011	6	10	5420	16.84	28.17
21585781	2011	6	10	5430	14.9	28.23
21585781	2011	6	10	5440	13	28.28
21585781	2011	6	10	5450	11.67	28.35
21585781	2011	6	10	5460	9.81	28.41
21585781	2011	6	10	5470	7.48	28.47
21585781	2011	6	10	5480	5.04	28.53
21585781	2011	6	10	5490	3.31	28.58
21585781	2011	6	10	5500	2.97	28.6
21585781	2011	6	10	5510	2.62	28.64
21585781	2011	6	10	5520	2.67	28.67
21585781	2011	6	10	5530	2.51	28.69
21585781	2011	6	10	5540	1.86	28.71
21585781	2011	6	10	5550	0.64	28.76
21585781	2011	6	10	5560	0.61	28.79
21585781	2011	6	10	5570	0.61	28.82
21585781	2011	6	10	5580	0.61	28.85
21585781	2011	6	10	5590	0.61	28.88
21585781	2011	6	10	5600	0.62	28.89
21585781	2011	6	10	5610	0.58	28.92
21585781	2011	6	10	5620	0.64	28.92
21585781	2011	6	10	5630	0.62	28.94
21585781	2011	6	10	5640	0.62	28.96
21585781	2011	6	10	5650	0.58	28.97
21585781	2011	6	10	5660	0.48	28.99
21585781	2011	6	10	5670	0.49	29
21585781	2011	6	10	5680	0.49	29.03
21585781	2011	6	10	5690	0.49	29.04
21923211	2011	6	14	0	1.04	28.38
21923211	2011	6	14	10	1.8	28.37
21923211	2011	6	14	20	2.12	28.37
21923211	2011	6	14	30	2.37	28.38
21923211	2011	6	14	40	2.56	28.38
21923211	2011	6	14	50	2.36	28.37
21923211	2011	6	14	60	2.58	28.38
21923211	2011	6	14	70	2.65	28.38
21923211	2011	6	14	80	2.65	28.38
21923211	2011	6	14	90	3.15	28.38
21923211	2011	6	14	100	3.68	28.38
21923211	2011	6	14	110	4.59	28.38
21923211	2011	6	14	120	5.33	28.43
21923211	2011	6	14	130	6.78	28.42
21923211	2011	6	14	140	8.46	28.41
21923211	2011	6	14	150	10.21	28.4
21923211	2011	6	14	160	11.4	28.39

21923211	2011	6	14	170	11.67	28.39
21923211	2011	6	14	180	11.49	28.39
21923211	2011	6	14	190	11.18	28.39
21923211	2011	6	14	200	10.97	28.39
21923211	2011	6	14	210	10.63	28.39
21923211	2011	6	14	220	10.33	28.4
21923211	2011	6	14	230	10.03	28.41
21923211	2011	6	14	240	9.92	28.41
21923211	2011	6	14	250	10.14	28.42
21923211	2011	6	14	260	10.13	28.41
21923211	2011	6	14	270	10.16	28.42
21923211	2011	6	14	280	10.41	28.42
21923211	2011	6	14	290	10.3	28.41
21923211	2011	6	14	300	10.23	28.42
21923211	2011	6	14	310	9.93	28.42
21923211	2011	6	14	320	9.85	28.41
21923211	2011	6	14	330	9.73	28.42
21923211	2011	6	14	340	9.66	28.43
21923211	2011	6	14	350	9.51	28.42
21923211	2011	6	14	360	9.25	28.43
21923211	2011	6	14	370	9.26	28.43
21923211	2011	6	14	380	10.05	28.42
21923211	2011	6	14	390	10.35	28.42
21923211	2011	6	14	400	10.4	28.42
21923211	2011	6	14	410	10.22	28.42
21923211	2011	6	14	420	9.93	28.42
21923211	2011	6	14	430	10	28.42
21923211	2011	6	14	440	10.41	28.42
21923211	2011	6	14	450	10.36	28.42
21923211	2011	6	14	460	10.15	28.42
21923211	2011	6	14	470	10.1	28.42
21923211	2011	6	14	480	9.7	28.43
21923211	2011	6	14	490	10.23	28.42
21923211	2011	6	14	500	11.5	28.41
21923211	2011	6	14	510	12.85	28.4
21923211	2011	6	14	520	14.71	28.39
21923211	2011	6	14	530	16.81	28.38
21923211	2011	6	14	540	18.36	28.36
21923211	2011	6	14	550	20.59	28.35
21923211	2011	6	14	560	22.89	28.32
21923211	2011	6	14	570	25.15	28.31
21923211	2011	6	14	580	27.04	28.29
21923211	2011	6	14	590	29.13	28.27
21923211	2011	6	14	600	31.05	28.27
21923211	2011	6	14	610	33.1	28.25
21923211	2011	6	14	620	34.74	28.24
21923211	2011	6	14	630	36.03	28.22

21923211	2011	6	14	640	36.72	28.22
21923211	2011	6	14	650	38.49	28.21
21923211	2011	6	14	660	40.22	28.2
21923211	2011	6	14	670	41.56	28.19
21923211	2011	6	14	680	43.34	28.17
21923211	2011	6	14	690	45.05	28.17
21923211	2011	6	14	700	46.6	28.15
21923211	2011	6	14	710	48.23	28.14
21923211	2011	6	14	720	49.91	28.04
21923211	2011	6	14	730	51.49	27.93
21923211	2011	6	14	740	53.3	27.85
21923211	2011	6	14	750	55	27.78
21923211	2011	6	14	760	55.13	27.72
21923211	2011	6	14	770	53.41	27.64
21923211	2011	6	14	780	51.26	27.62
21923211	2011	6	14	790	50.61	27.57
21923211	2011	6	14	800	50.66	27.46
21923211	2011	6	14	810	50.5	27.42
21923211	2011	6	14	820	50.59	27.39
21923211	2011	6	14	830	51.15	27.33
21923211	2011	6	14	840	52.04	27.25
21923211	2011	6	14	850	53.08	27.18
21923211	2011	6	14	860	53.49	27.17
21923211	2011	6	14	870	53.58	27.13
21923211	2011	6	14	880	53.58	27.1
21923211	2011	6	14	890	53.87	27.07
21923211	2011	6	14	900	53.91	27.03
21923211	2011	6	14	910	53.89	27
21923211	2011	6	14	920	54.06	26.98
21923211	2011	6	14	930	53.79	26.96
21923211	2011	6	14	940	53.69	26.92
21923211	2011	6	14	950	53.9	26.89
21923211	2011	6	14	960	54.14	26.83
21923211	2011	6	14	970	53.95	26.82
21923211	2011	6	14	980	53.66	26.81
21923211	2011	6	14	990	52.99	26.79
21923211	2011	6	14	1000	51.72	26.75
21923211	2011	6	14	1010	50.92	26.75
21923211	2011	6	14	1020	49.73	26.73
21923211	2011	6	14	1030	47.29	26.73
21923211	2011	6	14	1040	45.86	26.75
21923211	2011	6	14	1050	45.23	26.76
21923211	2011	6	14	1060	44.23	26.82
21923211	2011	6	14	1070	43.41	26.87
21923211	2011	6	14	1080	43.25	26.93
21923211	2011	6	14	1090	43.36	27
21923211	2011	6	14	1100	43.25	27.07

21923211	2011	6	14	1110	43.33	27.11
21923211	2011	6	14	1120	44.08	27.16
21923211	2011	6	14	1130	44.35	27.25
21923211	2011	6	14	1140	44.22	27.31
21923211	2011	6	14	1150	44.11	27.34
21923211	2011	6	14	1160	44.22	27.39
21923211	2011	6	14	1170	44.21	27.41
21923211	2011	6	14	1180	44.24	27.43
21923211	2011	6	14	1190	44.1	27.47
21923211	2011	6	14	1200	43.83	27.5
21923211	2011	6	14	1210	43.9	27.53
21923211	2011	6	14	1220	43.65	27.58
21923211	2011	6	14	1230	41.74	27.64
21923211	2011	6	14	1240	40.83	27.67
21923211	2011	6	14	1250	40.51	27.71
21923211	2011	6	14	1260	40.71	27.73
21923211	2011	6	14	1270	41.01	27.76
21923211	2011	6	14	1280	41.53	27.78
21923211	2011	6	14	1290	41.74	27.8
21923211	2011	6	14	1300	41.57	27.81
21923211	2011	6	14	1310	41.48	27.82
21923211	2011	6	14	1320	41.47	27.84
21923211	2011	6	14	1330	41.68	27.86
21923211	2011	6	14	1340	41.92	27.87
21923211	2011	6	14	1350	42.47	27.89
21923211	2011	6	14	1360	43.35	27.9
21923211	2011	6	14	1370	43.9	27.91
21923211	2011	6	14	1380	44.53	27.93
21923211	2011	6	14	1390	44.53	27.94
21923211	2011	6	14	1400	44.49	27.94
21923211	2011	6	14	1410	44.75	27.94
21923211	2011	6	14	1420	44.74	27.95
21923211	2011	6	14	1430	44.57	27.97
21923211	2011	6	14	1440	44.87	27.97
21923211	2011	6	14	1450	44.98	27.97
21923211	2011	6	14	1460	45.15	27.96
21923211	2011	6	14	1470	45.25	27.95
21923211	2011	6	14	1480	45.17	27.95
21923211	2011	6	14	1490	44.94	27.95
21923211	2011	6	14	1500	44.28	27.95
21923211	2011	6	14	1510	42.63	27.96
21923211	2011	6	14	1520	40.29	27.98
21923211	2011	6	14	1530	38.59	28
21923211	2011	6	14	1540	36.58	28.03
21923211	2011	6	14	1550	34.42	28.05
21923211	2011	6	14	1560	32.36	28.07
21923211	2011	6	14	1570	30.25	28.1

21923211	2011	6	14	1580	28.37	28.14
21923211	2011	6	14	1590	26.71	28.15
21923211	2011	6	14	1600	24.72	28.17
21923211	2011	6	14	1610	22.72	28.2
21923211	2011	6	14	1620	20.85	28.21
21923211	2011	6	14	1630	19.1	28.25
21923211	2011	6	14	1640	17.24	28.26
21923211	2011	6	14	1650	15.35	28.28
21923211	2011	6	14	1660	13.7	28.3
21923211	2011	6	14	1670	11.89	28.32
21923211	2011	6	14	1680	10.59	28.35
21923211	2011	6	14	1690	10.15	28.35
21923211	2011	6	14	1700	9.93	28.36
21923211	2011	6	14	1710	9.57	28.37
21923211	2011	6	14	1720	9.29	28.37
21923211	2011	6	14	1730	8.77	28.39
21923211	2011	6	14	1740	8.11	28.39
21923211	2011	6	14	1750	8.25	28.39
21923211	2011	6	14	1760	8.02	28.41
21923211	2011	6	14	1770	7.81	28.41
21923211	2011	6	14	1780	8.08	28.42
21923211	2011	6	14	1790	7.99	28.41
21923211	2011	6	14	1800	7.78	28.42
21923211	2011	6	14	1810	7.93	28.42
21923211	2011	6	14	1820	7.86	28.42
21923211	2011	6	14	1830	7.92	28.42
21923211	2011	6	14	1840	7.94	28.43
21923211	2011	6	14	1850	8.05	28.43
21923211	2011	6	14	1860	7.86	28.43
21923211	2011	6	14	1870	7.87	28.44
21923211	2011	6	14	1880	7.94	28.44
21923211	2011	6	14	1890	7.87	28.45
21923211	2011	6	14	1900	7.9	28.44
21923211	2011	6	14	1910	7.87	28.44
21923211	2011	6	14	1920	7.94	28.45
21923211	2011	6	14	1930	7.83	28.44
21923211	2011	6	14	1940	7.97	28.45
21923211	2011	6	14	1950	7.97	28.45
21923211	2011	6	14	1960	7.76	28.46
21923211	2011	6	14	1970	7.92	28.46
21923211	2011	6	14	1980	7.95	28.46
21923211	2011	6	14	1990	7.81	28.46
21923211	2011	6	14	2000	7.94	28.46
21923211	2011	6	14	2010	8.01	28.46
21923211	2011	6	14	2020	7.89	28.46
21923211	2011	6	14	2030	7.94	28.46
21923211	2011	6	14	2040	7.95	28.46

21923211	2011	6	14	2050	7.86	28.47
21923211	2011	6	14	2060	8.04	28.46
21923211	2011	6	14	2070	7.9	28.46
21923211	2011	6	14	2080	7.83	28.47
21923211	2011	6	14	2090	8.05	28.46
21923211	2011	6	14	2100	7.85	28.47
21923211	2011	6	14	2110	7.74	28.47
21923211	2011	6	14	2120	8.14	28.47
21923211	2011	6	14	2130	8.14	28.47
21923211	2011	6	14	2140	8.81	28.46
21923211	2011	6	14	2150	8.71	28.47
21923211	2011	6	14	2160	8.32	28.47
21923211	2011	6	14	2170	8.58	28.46
21923211	2011	6	14	2180	8.76	28.46
21923211	2011	6	14	2190	8.44	28.47
21923211	2011	6	14	2200	8.38	28.47
21923211	2011	6	14	2210	8.55	28.47
21923211	2011	6	14	2220	8.46	28.48
21923211	2011	6	14	2230	8.43	28.47
21923211	2011	6	14	2240	8.56	28.48
21923211	2011	6	14	2250	8.55	28.47
21923211	2011	6	14	2260	8.54	28.48
21923211	2011	6	14	2270	8.45	28.48
21923211	2011	6	14	2280	8.55	28.47
21923211	2011	6	14	2290	8.7	28.47
21923211	2011	6	14	2300	8.49	28.48
21923211	2011	6	14	2310	8.47	28.48
21923211	2011	6	14	2320	8.6	28.47
21923211	2011	6	14	2330	8.54	28.47
21923211	2011	6	14	2340	8.39	28.48
21923211	2011	6	14	2350	8.48	28.47
21923211	2011	6	14	2360	9.08	28.47
21923211	2011	6	14	2370	9.14	28.47
21923211	2011	6	14	2380	9.89	28.47
21923211	2011	6	14	2390	11.38	28.46
21923211	2011	6	14	2400	12.85	28.45
21923211	2011	6	14	2410	14.34	28.43
21923211	2011	6	14	2420	15.94	28.42
21923211	2011	6	14	2430	17.82	28.39
21923211	2011	6	14	2440	20.07	28.39
21923211	2011	6	14	2450	21.85	28.37
21923211	2011	6	14	2460	23.41	28.35
21923211	2011	6	14	2470	25.01	28.34
21923211	2011	6	14	2480	26.57	28.33
21923211	2011	6	14	2490	28.83	28.32
21923211	2011	6	14	2500	30.91	28.3
21923211	2011	6	14	2510	32.55	28.28

21923211	2011	6	14	2520	34.17	28.28
21923211	2011	6	14	2530	35.92	28.26
21923211	2011	6	14	2540	37.95	28.24
21923211	2011	6	14	2550	39.77	28.23
21923211	2011	6	14	2560	41.71	28.21
21923211	2011	6	14	2570	42.83	28.2
21923211	2011	6	14	2580	43.18	28.21
21923211	2011	6	14	2590	43.59	28.2
21923211	2011	6	14	2600	43.5	28.21
21923211	2011	6	14	2610	43.29	28.21
21923211	2011	6	14	2620	43.5	28.2
21923211	2011	6	14	2630	43.5	28.2
21923211	2011	6	14	2640	43.78	28.2
21923211	2011	6	14	2650	43.47	28.2
21923211	2011	6	14	2660	43.38	28.2
21923211	2011	6	14	2670	43.61	28.21
21923211	2011	6	14	2680	43.89	28.2
21923211	2011	6	14	2690	44.44	28.2
21923211	2011	6	14	2700	44.76	28.19
21923211	2011	6	14	2710	45.05	28.19
21923211	2011	6	14	2720	45.22	28.18
21923211	2011	6	14	2730	45.13	28.18
21923211	2011	6	14	2740	44.86	28.18
21923211	2011	6	14	2750	44.8	28.18
21923211	2011	6	14	2760	44.64	28.18
21923211	2011	6	14	2770	44.6	28.18
21923211	2011	6	14	2780	45.02	28.18
21923211	2011	6	14	2790	45.14	28.17
21923211	2011	6	14	2800	45.32	28.16
21923211	2011	6	14	2810	45.21	28.17
21923211	2011	6	14	2820	45.05	28.17
21923211	2011	6	14	2830	45.34	28.16
21923211	2011	6	14	2840	46.15	28.15
21923211	2011	6	14	2850	47	28.11
21923211	2011	6	14	2860	47.08	28.09
21923211	2011	6	14	2870	47.13	28.07
21923211	2011	6	14	2880	47.39	28.04
21923211	2011	6	14	2890	47.39	28
21923211	2011	6	14	2900	47.33	27.95
21923211	2011	6	14	2910	47.42	27.91
21923211	2011	6	14	2920	47.49	27.86
21923211	2011	6	14	2930	47.39	27.82
21923211	2011	6	14	2940	46.96	27.78
21923211	2011	6	14	2950	45.78	27.75
21923211	2011	6	14	2960	45.2	27.75
21923211	2011	6	14	2970	45.15	27.73
21923211	2011	6	14	2980	46.22	27.74

21923211	2011	6	14	2990	48.44	27.73
21923211	2011	6	14	3000	50.12	27.66
21923211	2011	6	14	3010	50.75	27.64
21923211	2011	6	14	3020	51.38	27.53
21923211	2011	6	14	3030	52.23	27.46
21923211	2011	6	14	3040	52.03	27.41
21923211	2011	6	14	3050	52.35	27.39
21923211	2011	6	14	3060	52.43	27.34
21923211	2011	6	14	3070	52.38	27.32
21923211	2011	6	14	3080	52.28	27.28
21923211	2011	6	14	3090	51.78	27.25
21923211	2011	6	14	3100	51.47	27.22
21923211	2011	6	14	3110	51.22	27.18
21923211	2011	6	14	3120	51.01	27.15
21923211	2011	6	14	3130	50.87	27.12
21923211	2011	6	14	3140	51.22	27.08
21923211	2011	6	14	3150	51.69	27.03
21923211	2011	6	14	3160	51.67	26.98
21923211	2011	6	14	3170	51.56	26.95
21923211	2011	6	14	3180	51.41	26.93
21923211	2011	6	14	3190	51.42	26.89
21923211	2011	6	14	3200	50.61	26.85
21923211	2011	6	14	3210	50.06	26.83
21923211	2011	6	14	3220	50.12	26.78
21923211	2011	6	14	3230	49.89	26.73
21923211	2011	6	14	3240	49.65	26.71
21923211	2011	6	14	3250	49.35	26.7
21923211	2011	6	14	3260	49.37	26.68
21923211	2011	6	14	3270	49.33	26.66
21923211	2011	6	14	3280	49.83	26.64
21923211	2011	6	14	3290	50.59	26.65
21923211	2011	6	14	3300	51.15	26.64
21923211	2011	6	14	3310	51.43	26.62
21923211	2011	6	14	3320	51.5	26.6
21923211	2011	6	14	3330	51.49	26.58
21923211	2011	6	14	3340	51.33	26.57
21923211	2011	6	14	3350	51.07	26.54
21923211	2011	6	14	3360	49.7	26.53
21923211	2011	6	14	3370	48.61	26.53
21923211	2011	6	14	3380	48.22	26.52
21923211	2011	6	14	3390	47.54	26.52
21923211	2011	6	14	3400	46.86	26.53
21923211	2011	6	14	3410	46.44	26.57
21923211	2011	6	14	3420	47.11	26.62
21923211	2011	6	14	3430	49.13	26.69
21923211	2011	6	14	3440	51.58	26.75
21923211	2011	6	14	3450	53.65	26.75

21923211	2011	6	14	3460	55.09	26.67
21923211	2011	6	14	3470	56.56	26.63
21923211	2011	6	14	3480	58.3	26.57
21923211	2011	6	14	3490	60.17	26.49
21923211	2011	6	14	3500	61.99	26.43
21923211	2011	6	14	3510	63.75	26.39
21923211	2011	6	14	3520	65.87	26.35
21923211	2011	6	14	3530	67.93	26.28
21923211	2011	6	14	3540	69.8	26.27
21923211	2011	6	14	3550	71.27	26.27
21923211	2011	6	14	3560	73.15	26.2
21923211	2011	6	14	3570	75.56	26.1
21923211	2011	6	14	3580	77.7	26.03
21923211	2011	6	14	3590	79.69	25.96
21923211	2011	6	14	3600	81.65	25.89
21923211	2011	6	14	3610	83.56	25.54
21923211	2011	6	14	3620	85.61	25.45
21923211	2011	6	14	3630	86.88	25.35
21923211	2011	6	14	3640	86.38	25.36
21923211	2011	6	14	3650	85.93	25.36
21923211	2011	6	14	3660	85.39	25.33
21923211	2011	6	14	3670	85.16	25.28
21923211	2011	6	14	3680	85.24	25.22
21923211	2011	6	14	3690	84.88	25.17
21923211	2011	6	14	3700	84.7	25.13
21923211	2011	6	14	3710	85.05	25.05
21923211	2011	6	14	3720	84.88	25.02
21923211	2011	6	14	3730	84.65	24.98
21923211	2011	6	14	3740	84.9	24.94
21923211	2011	6	14	3750	84.39	24.85
21923211	2011	6	14	3760	83.57	24.84
21923211	2011	6	14	3770	83.96	24.78
21923211	2011	6	14	3780	84.31	24.75
21923211	2011	6	14	3790	83.93	24.73
21923211	2011	6	14	3800	83.37	24.69
21923211	2011	6	14	3810	82.43	24.64
21923211	2011	6	14	3820	81.68	24.64
21923211	2011	6	14	3830	82.91	24.6
21923211	2011	6	14	3840	84.86	24.57
21923211	2011	6	14	3850	86.89	24.52
21923211	2011	6	14	3860	88.83	24.47
21923211	2011	6	14	3870	90.53	24.43
21923211	2011	6	14	3880	91.15	24.37
21923211	2011	6	14	3890	91.49	24.31
21923211	2011	6	14	3900	93.27	24.12
21923211	2011	6	14	3910	94.92	24.03
21923211	2011	6	14	3920	96.26	23.91

21923211	2011	6	14	3930	97.31	23.82
21923211	2011	6	14	3940	98.87	23.75
21923211	2011	6	14	3950	100.91	23.64
21923211	2011	6	14	3960	103.11	23.52
21923211	2011	6	14	3970	105.55	23.39
21923211	2011	6	14	3980	107.71	23.29
21923211	2011	6	14	3990	110.07	23.22
21923211	2011	6	14	4000	112.49	23.12
21923211	2011	6	14	4010	114.84	22.99
21923211	2011	6	14	4020	116.71	22.9
21923211	2011	6	14	4030	117.57	22.78
21923211	2011	6	14	4040	118.85	22.71
21923211	2011	6	14	4050	120.74	22.63
21923211	2011	6	14	4060	122.88	22.63
21923211	2011	6	14	4070	124.76	22.6
21923211	2011	6	14	4080	125.21	22.56
21923211	2011	6	14	4090	125.68	22.53
21923211	2011	6	14	4100	126.36	22.48
21923211	2011	6	14	4110	126.22	22.42
21923211	2011	6	14	4120	125.88	22.41
21923211	2011	6	14	4130	125.37	22.37
21923211	2011	6	14	4140	125.37	22.32
21923211	2011	6	14	4150	126.35	22.24
21923211	2011	6	14	4160	127.55	22.17
21923211	2011	6	14	4170	127.38	22.17
21923211	2011	6	14	4180	127.2	22.15
21923211	2011	6	14	4190	126.67	22.08
21923211	2011	6	14	4200	127.57	21.93
21923211	2011	6	14	4210	128.96	21.74
21923211	2011	6	14	4220	130.56	21.6
21923211	2011	6	14	4230	132.03	21.49
21923211	2011	6	14	4240	133.09	21.36
21923211	2011	6	14	4250	133.78	21.25
21923211	2011	6	14	4260	134.44	21.1
21923211	2011	6	14	4270	135.33	21.06
21923211	2011	6	14	4280	136.24	20.97
21923211	2011	6	14	4290	137.37	20.88
21923211	2011	6	14	4300	138.8	20.8
21923211	2011	6	14	4310	140.42	20.69
21923211	2011	6	14	4320	141.9	20.57
21923211	2011	6	14	4330	142.2	20.51
21923211	2011	6	14	4340	141.81	20.44
21923211	2011	6	14	4350	141.94	20.37
21923211	2011	6	14	4360	142.51	20.26
21923211	2011	6	14	4370	141.8	20.22
21923211	2011	6	14	4380	140.93	20.17
21923211	2011	6	14	4390	140.31	20.13

21923211	2011	6	14	4400	139.52	20.1
21923211	2011	6	14	4410	140.29	20.02
21923211	2011	6	14	4420	141.37	19.94
21923211	2011	6	14	4430	141.13	19.91
21923211	2011	6	14	4440	140.89	19.88
21923211	2011	6	14	4450	141.11	19.85
21923211	2011	6	14	4460	141.16	19.81
21923211	2011	6	14	4470	140.92	19.78
21923211	2011	6	14	4480	139.52	19.71
21923211	2011	6	14	4490	137.24	19.69
21923211	2011	6	14	4500	135.01	19.67
21923211	2011	6	14	4510	132.31	19.67
21923211	2011	6	14	4520	129.19	19.64
21923211	2011	6	14	4530	126.04	19.62
21923211	2011	6	14	4540	123.34	19.64
21923211	2011	6	14	4550	120.85	19.67
21923211	2011	6	14	4560	118.3	19.75
21923211	2011	6	14	4570	115.73	19.85
21923211	2011	6	14	4580	113.08	19.95
21923211	2011	6	14	4590	110.27	20.05
21923211	2011	6	14	4600	107.47	20.14
21923211	2011	6	14	4610	104.57	20.23
21923211	2011	6	14	4620	101.88	20.32
21923211	2011	6	14	4630	98.98	20.44
21923211	2011	6	14	4640	95.68	20.56
21923211	2011	6	14	4650	92.28	20.73
21923211	2011	6	14	4660	89.29	20.93
21923211	2011	6	14	4670	86.44	21.17
21923211	2011	6	14	4680	83.44	21.35
21923211	2011	6	14	4690	80.47	21.51
21923211	2011	6	14	4700	78.06	21.66
21923211	2011	6	14	4710	75.82	21.81
21923211	2011	6	14	4720	73.15	21.94
21923211	2011	6	14	4730	70.23	22.14
21923211	2011	6	14	4740	67.56	22.32
21923211	2011	6	14	4750	66.26	22.5
21923211	2011	6	14	4760	64.27	22.71
21923211	2011	6	14	4770	61.45	22.89
21923211	2011	6	14	4780	58.47	23.12
21923211	2011	6	14	4790	56.47	23.24
21923211	2011	6	14	4800	55	23.34
21923211	2011	6	14	4810	52.86	23.52
21923211	2011	6	14	4820	51.83	23.7
21923211	2011	6	14	4830	51.01	23.85
21923211	2011	6	14	4840	50.08	24.02
21923211	2011	6	14	4850	50.15	24.13
21923211	2011	6	14	4860	50.27	24.23

21923211	2011	6	14	4870	50.32	24.35
21923211	2011	6	14	4880	50.38	24.54
21923211	2011	6	14	4890	50.42	24.65
21923211	2011	6	14	4900	50.44	24.76
21923211	2011	6	14	4910	50.39	24.87
21923211	2011	6	14	4920	49.38	24.96
21923211	2011	6	14	4930	48.11	25.03
21923211	2011	6	14	4940	47.28	25.14
21923211	2011	6	14	4950	46.77	25.21
21923211	2011	6	14	4960	46.43	25.28
21923211	2011	6	14	4970	46.14	25.35
21923211	2011	6	14	4980	45.82	25.42
21923211	2011	6	14	4990	45.59	25.5
21923211	2011	6	14	5000	45.51	25.6
21923211	2011	6	14	5010	45.73	25.7
21923211	2011	6	14	5020	46.06	25.81
21923211	2011	6	14	5030	44.82	25.9
21923211	2011	6	14	5040	42.74	26.01
21923211	2011	6	14	5050	40.73	26.09
21923211	2011	6	14	5060	39.16	26.2
21923211	2011	6	14	5070	37.6	26.31
21923211	2011	6	14	5080	36.46	26.42
21923211	2011	6	14	5090	34.59	26.54
21923211	2011	6	14	5100	32.35	26.64
21923211	2011	6	14	5110	29.66	26.77
21923211	2011	6	14	5120	26.94	26.86
21923211	2011	6	14	5130	24.97	26.92
21923211	2011	6	14	5140	22.47	27
21923211	2011	6	14	5150	20.86	27.08
21923211	2011	6	14	5160	18.72	27.17
21923211	2011	6	14	5170	16.83	27.24
21923211	2011	6	14	5180	16	27.32
21923211	2011	6	14	5190	16.08	27.37
21923211	2011	6	14	5200	16.19	27.44
21923211	2011	6	14	5210	16.36	27.48
21923211	2011	6	14	5220	16.23	27.53
21923211	2011	6	14	5230	16.02	27.58
21923211	2011	6	14	5240	16.08	27.6
21923211	2011	6	14	5250	16.21	27.64
21923211	2011	6	14	5260	15.52	27.67
21923211	2011	6	14	5270	15.73	27.75
21923211	2011	6	14	5280	16.58	27.77
21923211	2011	6	14	5290	16.23	27.79
21923211	2011	6	14	5300	16.01	27.82
21923211	2011	6	14	5310	16.12	27.85
21923211	2011	6	14	5320	16.24	27.88
21923211	2011	6	14	5330	16.3	27.91

21923211	2011	6	14	5340	16.24	27.93
21923211	2011	6	14	5350	16.1	27.95
21923211	2011	6	14	5360	16.24	27.97
21923211	2011	6	14	5370	16.29	27.99
21923211	2011	6	14	5380	16.16	28.01
21923211	2011	6	14	5390	16	28.03
21923211	2011	6	14	5400	16.16	28.06
21923211	2011	6	14	5410	16.32	28.07
21923211	2011	6	14	5420	16.34	28.08
21923211	2011	6	14	5430	16.26	28.1
21923211	2011	6	14	5440	16.24	28.12
21923211	2011	6	14	5450	15.67	28.14
21923211	2011	6	14	5460	16.31	28.16
21923211	2011	6	14	5470	16.4	28.17
21923211	2011	6	14	5480	16.11	28.18
21923211	2011	6	14	5490	16.15	28.19
21923211	2011	6	14	5500	16.3	28.21
21923211	2011	6	14	5510	16.25	28.23
21923211	2011	6	14	5520	16.28	28.25
21923211	2011	6	14	5530	16.2	28.25
21923211	2011	6	14	5540	16.08	28.25
21923211	2011	6	14	5550	16.14	28.27
21923211	2011	6	14	5560	16.07	28.27
21923211	2011	6	14	5570	16.18	28.28
21923211	2011	6	14	5580	16.22	28.3
21923211	2011	6	14	5590	16.23	28.3
21923211	2011	6	14	5600	16.25	28.3
21923211	2011	6	14	5610	16.29	28.32
21923211	2011	6	14	5620	16.26	28.32
21923211	2011	6	14	5630	16.21	28.32
21923211	2011	6	14	5640	16.22	28.33
21923211	2011	6	14	5650	16.29	28.34
21923211	2011	6	14	5660	16.33	28.34
21923211	2011	6	14	5670	16.14	28.35
21923211	2011	6	14	5680	16.24	28.35
21923211	2011	6	14	5690	16.26	28.35
21923211	2011	6	14	5700	16.35	28.35
21923211	2011	6	14	5710	16.42	28.36
21923211	2011	6	14	5720	16.33	28.36
21923211	2011	6	14	5730	16.25	28.38
21923211	2011	6	14	5740	16.36	28.38
21923211	2011	6	14	5750	16.47	28.38
21923211	2011	6	14	5760	16.29	28.38
21923211	2011	6	14	5770	15.86	28.39
21923211	2011	6	14	5780	15.61	28.39
21923211	2011	6	14	5790	16.61	28.39
21923211	2011	6	14	5800	16.19	28.39

21923211	2011	6	14	5810	16.18	28.39
21923211	2011	6	14	5820	16.38	28.39
21923211	2011	6	14	5830	16.21	28.39
21923211	2011	6	14	5840	16.11	28.39
21923211	2011	6	14	5850	16.1	28.4
21923211	2011	6	14	5860	14.25	28.42
21923211	2011	6	14	5870	12.1	28.44
21923211	2011	6	14	5880	10.54	28.46
21923211	2011	6	14	5890	7.73	28.48
21923211	2011	6	14	5900	5.36	28.5
21923211	2011	6	14	5910	4.34	28.52
21923211	2011	6	14	5920	3.2	28.52
21923211	2011	6	14	5930	2.12	28.53
21923211	2011	6	14	5940	1.76	28.54
21923211	2011	6	14	5950	1.63	28.54
21923211	2011	6	14	5960	1.68	28.55
21923211	2011	6	14	5970	1.62	28.55
21923211	2011	6	14	5980	0.92	28.57
21923211	2011	6	14	5990	0.66	28.56
21923211	2011	6	14	6000	0.67	28.57
21923211	2011	6	14	6010	0.62	28.57
21923211	2011	6	14	6020	0.62	28.57
21923211	2011	6	14	6030	0.68	28.57
21923211	2011	6	14	6040	0.64	28.57
21923211	2011	6	14	6050	0.68	28.57
21923211	2011	6	14	6060	0.7	28.58
21923211	2011	6	14	6070	0.56	28.59
21923211	2011	6	14	6080	0.57	28.58
21923211	2011	6	14	6090	0.57	28.6
21923211	2011	6	14	6100	0.57	28.59
21923211	2011	6	14	6110	0.57	28.6
21923211	2011	6	14	6120	0.6	28.6
21923211	2011	6	14	6130	0.56	28.6
21944731	2011	6	14	0	0.93	29.16
21944731	2011	6	14	10	1.08	29.17
21944731	2011	6	14	20	1.78	29.16
21944731	2011	6	14	30	1.59	29.16
21944731	2011	6	14	40	1.88	29.16
21944731	2011	6	14	50	2.13	29.14
21944731	2011	6	14	60	1.84	29.14
21944731	2011	6	14	70	1.73	29.12
21944731	2011	6	14	80	2.62	29.09
21944731	2011	6	14	90	2.77	29.08
21944731	2011	6	14	100	2.75	29.07
21944731	2011	6	14	110	4.03	29.03
21944731	2011	6	14	120	5.72	29
21944731	2011	6	14	130	7.03	28.96

21944731	2011	6	14	140	8.74	28.92
21944731	2011	6	14	150	10.19	28.89
21944731	2011	6	14	160	11.37	28.87
21944731	2011	6	14	170	12.66	28.86
21944731	2011	6	14	180	13.27	28.85
21944731	2011	6	14	190	13.16	28.85
21944731	2011	6	14	200	12.86	28.85
21944731	2011	6	14	210	12.97	28.83
21944731	2011	6	14	220	12.55	28.83
21944731	2011	6	14	230	12	28.82
21944731	2011	6	14	240	12.31	28.82
21944731	2011	6	14	250	12.88	28.8
21944731	2011	6	14	260	12.66	28.79
21944731	2011	6	14	270	12.37	28.79
21944731	2011	6	14	280	12.4	28.78
21944731	2011	6	14	290	12.26	28.78
21944731	2011	6	14	300	11.63	28.78
21944731	2011	6	14	310	11.08	28.78
21944731	2011	6	14	320	11.64	28.77
21944731	2011	6	14	330	12.85	28.75
21944731	2011	6	14	340	13.37	28.75
21944731	2011	6	14	350	13.69	28.74
21944731	2011	6	14	360	13.91	28.73
21944731	2011	6	14	370	13.83	28.72
21944731	2011	6	14	380	14.53	28.71
21944731	2011	6	14	390	14.64	28.71
21944731	2011	6	14	400	14.25	28.71
21944731	2011	6	14	410	13.83	28.71
21944731	2011	6	14	420	13.42	28.71
21944731	2011	6	14	430	13.3	28.71
21944731	2011	6	14	440	13.37	28.71
21944731	2011	6	14	450	13.42	28.71
21944731	2011	6	14	460	13.93	28.69
21944731	2011	6	14	470	13.97	28.68
21944731	2011	6	14	480	14.1	28.69
21944731	2011	6	14	490	15.01	28.67
21944731	2011	6	14	500	16.34	28.66
21944731	2011	6	14	510	18.12	28.64
21944731	2011	6	14	520	19.67	28.63
21944731	2011	6	14	530	20.26	28.6
21944731	2011	6	14	540	20.81	28.6
21944731	2011	6	14	550	22.45	28.6
21944731	2011	6	14	560	24.2	28.58
21944731	2011	6	14	570	26.23	28.56
21944731	2011	6	14	580	28.22	28.53
21944731	2011	6	14	590	30.06	28.52
21944731	2011	6	14	600	31.65	28.5

21944731	2011	6	14	610	33.13	28.48
21944731	2011	6	14	620	35.06	28.46
21944731	2011	6	14	630	37.1	28.43
21944731	2011	6	14	640	39.25	28.42
21944731	2011	6	14	650	41.29	28.39
21944731	2011	6	14	660	42.09	28.38
21944731	2011	6	14	670	42.55	28.39
21944731	2011	6	14	680	43.39	28.37
21944731	2011	6	14	690	44.97	28.35
21944731	2011	6	14	700	46.65	28.08
21944731	2011	6	14	710	48.31	27.91
21944731	2011	6	14	720	50	27.82
21944731	2011	6	14	730	50.74	27.83
21944731	2011	6	14	740	51.65	27.67
21944731	2011	6	14	750	52.16	27.64
21944731	2011	6	14	760	52.38	27.63
21944731	2011	6	14	770	52.13	27.6
21944731	2011	6	14	780	51.77	27.57
21944731	2011	6	14	790	51.43	27.52
21944731	2011	6	14	800	51.08	27.48
21944731	2011	6	14	810	50.96	27.41
21944731	2011	6	14	820	51.62	27.31
21944731	2011	6	14	830	52.74	27.14
21944731	2011	6	14	840	52.54	27.14
21944731	2011	6	14	850	51.61	27.13
21944731	2011	6	14	860	51.04	27.11
21944731	2011	6	14	870	49.84	27.09
21944731	2011	6	14	880	49.06	27.07
21944731	2011	6	14	890	48.94	27.03
21944731	2011	6	14	900	48.98	27
21944731	2011	6	14	910	48.89	26.96
21944731	2011	6	14	920	48.47	26.95
21944731	2011	6	14	930	48.66	26.93
21944731	2011	6	14	940	48.51	26.92
21944731	2011	6	14	950	48.55	26.91
21944731	2011	6	14	960	48.91	26.89
21944731	2011	6	14	970	48.84	26.86
21944731	2011	6	14	980	48.29	26.85
21944731	2011	6	14	990	48.58	26.83
21944731	2011	6	14	1000	49.04	26.81
21944731	2011	6	14	1010	50.06	26.77
21944731	2011	6	14	1020	51.44	26.73
21944731	2011	6	14	1030	53.13	26.68
21944731	2011	6	14	1040	53.55	26.67
21944731	2011	6	14	1050	53	26.67
21944731	2011	6	14	1060	52.54	26.67
21944731	2011	6	14	1070	52.63	26.64

21944731	2011	6	14	1080	53.22	26.63
21944731	2011	6	14	1090	53.6	26.6
21944731	2011	6	14	1100	54.02	26.59
21944731	2011	6	14	1110	54.05	26.58
21944731	2011	6	14	1120	54.13	26.57
21944731	2011	6	14	1130	53.74	26.56
21944731	2011	6	14	1140	54.22	26.54
21944731	2011	6	14	1150	54.12	26.53
21944731	2011	6	14	1160	54.3	26.52
21944731	2011	6	14	1170	54.21	26.5
21944731	2011	6	14	1180	54.27	26.49
21944731	2011	6	14	1190	53.8	26.48
21944731	2011	6	14	1200	53.74	26.47
21944731	2011	6	14	1210	53.73	26.44
21944731	2011	6	14	1220	53.74	26.42
21944731	2011	6	14	1230	53.24	26.42
21944731	2011	6	14	1240	51.94	26.42
21944731	2011	6	14	1250	50.64	26.41
21944731	2011	6	14	1260	48.98	26.42
21944731	2011	6	14	1270	47.84	26.42
21944731	2011	6	14	1280	45.98	26.44
21944731	2011	6	14	1290	44	26.45
21944731	2011	6	14	1300	43.36	26.46
21944731	2011	6	14	1310	43.03	26.49
21944731	2011	6	14	1320	42.69	26.52
21944731	2011	6	14	1330	42.38	26.54
21944731	2011	6	14	1340	42.38	26.6
21944731	2011	6	14	1350	42.79	26.67
21944731	2011	6	14	1360	43.53	26.78
21944731	2011	6	14	1370	43.55	26.87
21944731	2011	6	14	1380	43.63	26.95
21944731	2011	6	14	1390	43.62	27
21944731	2011	6	14	1400	43.67	27.05
21944731	2011	6	14	1410	43.36	27.1
21944731	2011	6	14	1420	43.52	27.16
21944731	2011	6	14	1430	43.64	27.2
21944731	2011	6	14	1440	43.48	27.26
21944731	2011	6	14	1450	43.35	27.3
21944731	2011	6	14	1460	43.67	27.35
21944731	2011	6	14	1470	43.81	27.39
21944731	2011	6	14	1480	44.24	27.44
21944731	2011	6	14	1490	44.66	27.46
21944731	2011	6	14	1500	45.73	27.43
21944731	2011	6	14	1510	46.08	27.39
21944731	2011	6	14	1520	45.86	27.39
21944731	2011	6	14	1530	45	27.38
21944731	2011	6	14	1540	45.11	27.35

21944731	2011	6	14	1550	45.34	27.32
21944731	2011	6	14	1560	45.27	27.29
21944731	2011	6	14	1570	45.41	27.27
21944731	2011	6	14	1580	45.16	27.24
21944731	2011	6	14	1590	45.68	27.22
21944731	2011	6	14	1600	45.75	27.19
21944731	2011	6	14	1610	45.98	27.17
21944731	2011	6	14	1620	46.22	27.14
21944731	2011	6	14	1630	46.34	27.11
21944731	2011	6	14	1640	46.01	27.1
21944731	2011	6	14	1650	45.93	27.07
21944731	2011	6	14	1660	46.82	27.04
21944731	2011	6	14	1670	47.18	27.01
21944731	2011	6	14	1680	47.29	27
21944731	2011	6	14	1690	46.95	26.98
21944731	2011	6	14	1700	46.8	26.96
21944731	2011	6	14	1710	46.44	26.95
21944731	2011	6	14	1720	46.48	26.94
21944731	2011	6	14	1730	47.15	26.91
21944731	2011	6	14	1740	46.93	26.89
21944731	2011	6	14	1750	46.12	26.89
21944731	2011	6	14	1760	45.93	26.87
21944731	2011	6	14	1770	46.77	26.85
21944731	2011	6	14	1780	46.88	26.84
21944731	2011	6	14	1790	46.33	26.83
21944731	2011	6	14	1800	45.6	26.82
21944731	2011	6	14	1810	45.39	26.82
21944731	2011	6	14	1820	45.7	26.78
21944731	2011	6	14	1830	46.08	26.77
21944731	2011	6	14	1840	46.27	26.75
21944731	2011	6	14	1850	47.71	26.73
21944731	2011	6	14	1860	48.12	26.71
21944731	2011	6	14	1870	47.73	26.71
21944731	2011	6	14	1880	47.12	26.7
21944731	2011	6	14	1890	46.66	26.7
21944731	2011	6	14	1900	46.36	26.69
21944731	2011	6	14	1910	46.48	26.67
21944731	2011	6	14	1920	46.34	26.67
21944731	2011	6	14	1930	46.11	26.66
21944731	2011	6	14	1940	46.23	26.64
21944731	2011	6	14	1950	46.02	26.64
21944731	2011	6	14	1960	45.71	26.64
21944731	2011	6	14	1970	45.67	26.64
21944731	2011	6	14	1980	45.99	26.63
21944731	2011	6	14	1990	46.05	26.63
21944731	2011	6	14	2000	46.11	26.62
21944731	2011	6	14	2010	46.79	26.61

21944731	2011	6	14	2020	48.39	26.58
21944731	2011	6	14	2030	49.38	26.57
21944731	2011	6	14	2040	50.16	26.54
21944731	2011	6	14	2050	50.85	26.53
21944731	2011	6	14	2060	52.25	26.51
21944731	2011	6	14	2070	52.82	26.5
21944731	2011	6	14	2080	52.75	26.5
21944731	2011	6	14	2090	52.17	26.5
21944731	2011	6	14	2100	51.93	26.5
21944731	2011	6	14	2110	51.94	26.49
21944731	2011	6	14	2120	51.91	26.47
21944731	2011	6	14	2130	51.63	26.46
21944731	2011	6	14	2140	51.73	26.46
21944731	2011	6	14	2150	51.87	26.45
21944731	2011	6	14	2160	51.78	26.42
21944731	2011	6	14	2170	52.06	26.42
21944731	2011	6	14	2180	52.03	26.42
21944731	2011	6	14	2190	51.73	26.4
21944731	2011	6	14	2200	51.89	26.39
21944731	2011	6	14	2210	51.82	26.39
21944731	2011	6	14	2220	51.82	26.38
21944731	2011	6	14	2230	51.73	26.37
21944731	2011	6	14	2240	51.83	26.37
21944731	2011	6	14	2250	51.62	26.36
21944731	2011	6	14	2260	51.24	26.36
21944731	2011	6	14	2270	51.55	26.35
21944731	2011	6	14	2280	51.58	26.34
21944731	2011	6	14	2290	52.12	26.34
21944731	2011	6	14	2300	52.51	26.32
21944731	2011	6	14	2310	53.9	26.29
21944731	2011	6	14	2320	55.98	26.27
21944731	2011	6	14	2330	57.78	26.25
21944731	2011	6	14	2340	59.77	26.21
21944731	2011	6	14	2350	61.46	26.15
21944731	2011	6	14	2360	62.99	26.1
21944731	2011	6	14	2370	64.33	26.06
21944731	2011	6	14	2380	65.51	25.97
21944731	2011	6	14	2390	67.36	25.92
21944731	2011	6	14	2400	69.66	25.89
21944731	2011	6	14	2410	71.71	25.75
21944731	2011	6	14	2420	73.21	25.66
21944731	2011	6	14	2430	74.41	25.57
21944731	2011	6	14	2440	75.91	25.49
21944731	2011	6	14	2450	77.81	25.42
21944731	2011	6	14	2460	79.39	25.35
21944731	2011	6	14	2470	79.56	25.35
21944731	2011	6	14	2480	79.96	25.3

21944731	2011	6	14	2490	80.22	25.25
21944731	2011	6	14	2500	81.52	25.15
21944731	2011	6	14	2510	82.47	25.09
21944731	2011	6	14	2520	82.42	25.09
21944731	2011	6	14	2530	82.47	25.03
21944731	2011	6	14	2540	82.33	25.01
21944731	2011	6	14	2550	82.28	24.97
21944731	2011	6	14	2560	82.41	24.94
21944731	2011	6	14	2570	83.61	24.85
21944731	2011	6	14	2580	85.13	24.8
21944731	2011	6	14	2590	85.67	24.77
21944731	2011	6	14	2600	85.69	24.76
21944731	2011	6	14	2610	85.57	24.75
21944731	2011	6	14	2620	85.67	24.73
21944731	2011	6	14	2630	85.84	24.71
21944731	2011	6	14	2640	85.86	24.69
21944731	2011	6	14	2650	85.79	24.67
21944731	2011	6	14	2660	85.38	24.66
21944731	2011	6	14	2670	85.42	24.62
21944731	2011	6	14	2680	84.96	24.6
21944731	2011	6	14	2690	84.15	24.59
21944731	2011	6	14	2700	84.28	24.56
21944731	2011	6	14	2710	85.52	24.52
21944731	2011	6	14	2720	87	24.49
21944731	2011	6	14	2730	87.46	24.47
21944731	2011	6	14	2740	87.32	24.47
21944731	2011	6	14	2750	86.82	24.46
21944731	2011	6	14	2760	86.19	24.45
21944731	2011	6	14	2770	86.82	24.42
21944731	2011	6	14	2780	87.68	24.39
21944731	2011	6	14	2790	88.5	24.35
21944731	2011	6	14	2800	89.44	24.26
21944731	2011	6	14	2810	90.59	24.2
21944731	2011	6	14	2820	90.84	24.1
21944731	2011	6	14	2830	91.69	24
21944731	2011	6	14	2840	92.61	23.91
21944731	2011	6	14	2850	93.81	23.8
21944731	2011	6	14	2860	94.88	23.71
21944731	2011	6	14	2870	96.06	23.64
21944731	2011	6	14	2880	97.93	23.57
21944731	2011	6	14	2890	100.17	23.51
21944731	2011	6	14	2900	102.51	23.41
21944731	2011	6	14	2910	104.79	23.3
21944731	2011	6	14	2920	107.1	23.2
21944731	2011	6	14	2930	109.58	23.13
21944731	2011	6	14	2940	112.18	23.03
21944731	2011	6	14	2950	114.78	22.98

21944731	2011	6	14	2960	116.73	22.89
21944731	2011	6	14	2970	118.27	22.78
21944731	2011	6	14	2980	119.86	22.64
21944731	2011	6	14	2990	121.87	22.49
21944731	2011	6	14	3000	123.86	22.38
21944731	2011	6	14	3010	125.38	22.24
21944731	2011	6	14	3020	126.65	22.07
21944731	2011	6	14	3030	127.96	22
21944731	2011	6	14	3040	129.27	21.91
21944731	2011	6	14	3050	130.6	21.82
21944731	2011	6	14	3060	132.42	21.75
21944731	2011	6	14	3070	134.55	21.67
21944731	2011	6	14	3080	136.54	21.61
21944731	2011	6	14	3090	136.87	21.54
21944731	2011	6	14	3100	136.32	21.5
21944731	2011	6	14	3110	135.09	21.5
21944731	2011	6	14	3120	133.42	21.46
21944731	2011	6	14	3130	131.88	21.41
21944731	2011	6	14	3140	132.28	21.35
21944731	2011	6	14	3150	133.79	21.3
21944731	2011	6	14	3160	135.8	21.17
21944731	2011	6	14	3170	137.29	21.08
21944731	2011	6	14	3180	137.49	21.08
21944731	2011	6	14	3190	136.94	21.08
21944731	2011	6	14	3200	135.8	21.06
21944731	2011	6	14	3210	135.07	21.03
21944731	2011	6	14	3220	134.39	20.99
21944731	2011	6	14	3230	133.73	20.96
21944731	2011	6	14	3240	134.81	20.92
21944731	2011	6	14	3250	135.32	20.87
21944731	2011	6	14	3260	134.57	20.84
21944731	2011	6	14	3270	133.65	20.8
21944731	2011	6	14	3280	132.63	20.79
21944731	2011	6	14	3290	132.4	20.73
21944731	2011	6	14	3300	131.59	20.72
21944731	2011	6	14	3310	130.54	20.7
21944731	2011	6	14	3320	129.89	20.69
21944731	2011	6	14	3330	129.28	20.67
21944731	2011	6	14	3340	128.96	20.68
21944731	2011	6	14	3350	128.58	20.7
21944731	2011	6	14	3360	127.83	20.71
21944731	2011	6	14	3370	128.46	20.71
21944731	2011	6	14	3380	128.8	20.7
21944731	2011	6	14	3390	128.23	20.71
21944731	2011	6	14	3400	127.11	20.7
21944731	2011	6	14	3410	126.67	20.69
21944731	2011	6	14	3420	126.27	20.67

21944731	2011	6	14	3430	125.86	20.67
21944731	2011	6	14	3440	125.74	20.67
21944731	2011	6	14	3450	125.07	20.66
21944731	2011	6	14	3460	125.11	20.65
21944731	2011	6	14	3470	124.79	20.64
21944731	2011	6	14	3480	124.21	20.65
21944731	2011	6	14	3490	124	20.66
21944731	2011	6	14	3500	124.3	20.67
21944731	2011	6	14	3510	124.17	20.67
21944731	2011	6	14	3520	123.65	20.67
21944731	2011	6	14	3530	122.87	20.67
21944731	2011	6	14	3540	122.4	20.69
21944731	2011	6	14	3550	121.83	20.7
21944731	2011	6	14	3560	121.7	20.71
21944731	2011	6	14	3570	122.56	20.71
21944731	2011	6	14	3580	122.29	20.72
21944731	2011	6	14	3590	121.94	20.72
21944731	2011	6	14	3600	121.66	20.73
21944731	2011	6	14	3610	121.7	20.73
21944731	2011	6	14	3620	122.05	20.73
21944731	2011	6	14	3630	121.61	20.72
21944731	2011	6	14	3640	121.11	20.73
21944731	2011	6	14	3650	121.2	20.73
21944731	2011	6	14	3660	120.59	20.73
21944731	2011	6	14	3670	119.85	20.73
21944731	2011	6	14	3680	120.46	20.72
21944731	2011	6	14	3690	122.04	20.72
21944731	2011	6	14	3700	122.53	20.71
21944731	2011	6	14	3710	122.12	20.71
21944731	2011	6	14	3720	122.1	20.72
21944731	2011	6	14	3730	121.51	20.71
21944731	2011	6	14	3740	120.84	20.71
21944731	2011	6	14	3750	120.53	20.71
21944731	2011	6	14	3760	120.23	20.71
21944731	2011	6	14	3770	120.03	20.71
21944731	2011	6	14	3780	119.84	20.72
21944731	2011	6	14	3790	119.23	20.73
21944731	2011	6	14	3800	118.57	20.73
21944731	2011	6	14	3810	118.01	20.74
21944731	2011	6	14	3820	117.58	20.78
21944731	2011	6	14	3830	117.06	20.79
21944731	2011	6	14	3840	115.27	20.82
21944731	2011	6	14	3850	112.08	20.84
21944731	2011	6	14	3860	108.91	20.87
21944731	2011	6	14	3870	106.54	20.92
21944731	2011	6	14	3880	104.42	20.96
21944731	2011	6	14	3890	102.7	21.01

21944731	2011	6	14	3900	100.4	21.09
21944731	2011	6	14	3910	98.02	21.16
21944731	2011	6	14	3920	96.45	21.22
21944731	2011	6	14	3930	94.68	21.31
21944731	2011	6	14	3940	92.37	21.39
21944731	2011	6	14	3950	89.47	21.46
21944731	2011	6	14	3960	86.68	21.6
21944731	2011	6	14	3970	84.42	21.78
21944731	2011	6	14	3980	82.35	21.89
21944731	2011	6	14	3990	80.36	22.04
21944731	2011	6	14	4000	79.09	22.17
21944731	2011	6	14	4010	77.44	22.28
21944731	2011	6	14	4020	75.71	22.38
21944731	2011	6	14	4030	74.04	22.48
21944731	2011	6	14	4040	72.31	22.6
21944731	2011	6	14	4050	70.46	22.71
21944731	2011	6	14	4060	68.1	22.82
21944731	2011	6	14	4070	66.43	22.91
21944731	2011	6	14	4080	64.29	23
21944731	2011	6	14	4090	61.76	23.12
21944731	2011	6	14	4100	59.08	23.25
21944731	2011	6	14	4110	56.46	23.41
21944731	2011	6	14	4120	53.97	23.51
21944731	2011	6	14	4130	51.53	23.66
21944731	2011	6	14	4140	49.51	23.77
21944731	2011	6	14	4150	46.95	23.89
21944731	2011	6	14	4160	44.98	24
21944731	2011	6	14	4170	43.89	24.14
21944731	2011	6	14	4180	43.27	24.27
21944731	2011	6	14	4190	42.85	24.39
21944731	2011	6	14	4200	42.14	24.53
21944731	2011	6	14	4210	40.55	24.67
21944731	2011	6	14	4220	40.68	24.97
21944731	2011	6	14	4230	40.46	25.14
21944731	2011	6	14	4240	39.51	25.25
21944731	2011	6	14	4250	38.29	25.38
21944731	2011	6	14	4260	36.14	25.6
21944731	2011	6	14	4270	34.82	25.78
21944731	2011	6	14	4280	33.68	25.96
21944731	2011	6	14	4290	32.92	26.12
21944731	2011	6	14	4300	32.21	26.21
21944731	2011	6	14	4310	31.68	26.3
21944731	2011	6	14	4320	31.22	26.4
21944731	2011	6	14	4330	31.15	26.53
21944731	2011	6	14	4340	30.33	26.64
21944731	2011	6	14	4350	29.32	26.75
21944731	2011	6	14	4360	28.79	26.86

21944731	2011	6	14	4370	28.4	26.99
21944731	2011	6	14	4380	28.47	27.03
21944731	2011	6	14	4390	28.31	27.09
21944731	2011	6	14	4400	27.75	27.14
21944731	2011	6	14	4410	27.71	27.19
21944731	2011	6	14	4420	28.07	27.29
21944731	2011	6	14	4430	27.76	27.35
21944731	2011	6	14	4440	27.27	27.4
21944731	2011	6	14	4450	26.93	27.49
21944731	2011	6	14	4460	26.64	27.57
21944731	2011	6	14	4470	26.84	27.64
21944731	2011	6	14	4480	26.71	27.71
21944731	2011	6	14	4490	26.52	27.73
21944731	2011	6	14	4500	26.99	27.77
21944731	2011	6	14	4510	26.76	27.81
21944731	2011	6	14	4520	26.64	27.86
21944731	2011	6	14	4530	26.45	27.89
21944731	2011	6	14	4540	26.37	27.9
21944731	2011	6	14	4550	26.3	27.93
21944731	2011	6	14	4560	26.12	28
21944731	2011	6	14	4570	26.15	28.04
21944731	2011	6	14	4580	25.97	28.11
21944731	2011	6	14	4590	25.58	28.13
21944731	2011	6	14	4600	25.93	28.17
21944731	2011	6	14	4610	25.86	28.17
21944731	2011	6	14	4620	25.54	28.17
21944731	2011	6	14	4630	25.89	28.17
21944731	2011	6	14	4640	26.03	28.19
21944731	2011	6	14	4650	26.29	28.21
21944731	2011	6	14	4660	26.26	28.23
21944731	2011	6	14	4670	26.25	28.24
21944731	2011	6	14	4680	26.11	28.25
21944731	2011	6	14	4690	25.6	28.28
21944731	2011	6	14	4700	25.57	28.32
21944731	2011	6	14	4710	25.76	28.35
21944731	2011	6	14	4720	25.15	28.35
21944731	2011	6	14	4730	25.12	28.36
21944731	2011	6	14	4740	25.28	28.37
21944731	2011	6	14	4750	24.45	28.38
21944731	2011	6	14	4760	23.73	28.4
21944731	2011	6	14	4770	23.48	28.41
21944731	2011	6	14	4780	23.45	28.42
21944731	2011	6	14	4790	23.34	28.44
21944731	2011	6	14	4800	23.15	28.45
21944731	2011	6	14	4810	22.87	28.46
21944731	2011	6	14	4820	22.39	28.47
21944731	2011	6	14	4830	21.85	28.49

21944731	2011	6	14	4840	21.56	28.51
21944731	2011	6	14	4850	21.61	28.5
21944731	2011	6	14	4860	21.83	28.52
21944731	2011	6	14	4870	21.78	28.53
21944731	2011	6	14	4880	21.64	28.53
21944731	2011	6	14	4890	21.62	28.53
21944731	2011	6	14	4900	21.35	28.53
21944731	2011	6	14	4910	19.92	28.55
21944731	2011	6	14	4920	18.6	28.57
21944731	2011	6	14	4930	17.48	28.57
21944731	2011	6	14	4940	15.9	28.6
21944731	2011	6	14	4950	13.46	28.63
21944731	2011	6	14	4960	11.65	28.64
21944731	2011	6	14	4970	9.36	28.67
21944731	2011	6	14	4980	6.6	28.7
21944731	2011	6	14	4990	3.94	28.72
21944731	2011	6	14	5000	2.64	28.75
21944731	2011	6	14	5010	2.68	28.75
21944731	2011	6	14	5020	2.17	28.75
21944731	2011	6	14	5030	1.79	28.76
21944731	2011	6	14	5040	2.1	28.76
21944731	2011	6	14	5050	2.14	28.78
21944731	2011	6	14	5060	1.62	28.78
21944731	2011	6	14	5070	0.67	28.79
21944731	2011	6	14	5080	0.57	28.82
21944731	2011	6	14	5090	0.59	28.82
21944731	2011	6	14	5100	0.59	28.83
21944731	2011	6	14	5110	0.6	28.85
21944731	2011	6	14	5120	0.61	28.86
21944731	2011	6	14	5130	0.59	28.89
21944731	2011	6	14	5140	0.57	28.89
21944731	2011	6	14	5150	0.57	28.91
21944731	2011	6	14	5160	0.61	28.92
21944731	2011	6	14	5170	0.55	28.94
21944731	2011	6	14	5180	0.48	28.95
21944731	2011	6	14	5190	0.47	28.96
21944731	2011	6	14	5200	0.47	28.97
21944731	2011	6	14	5210	0.53	28.98
22010671	2011	6	15	0	0.94	28.71
22010671	2011	6	15	10	1.27	28.71
22010671	2011	6	15	20	1.1	28.71
22010671	2011	6	15	30	1.76	28.71
22010671	2011	6	15	40	2.11	28.7
22010671	2011	6	15	50	2.14	28.69
22010671	2011	6	15	60	2.33	28.7
22010671	2011	6	15	70	2.59	28.7
22010671	2011	6	15	80	2.46	28.7

22010671	2011	6	15	90	2.84	28.69
22010671	2011	6	15	100	3.56	28.69
22010671	2011	6	15	110	4.03	28.68
22010671	2011	6	15	120	5.11	28.67
22010671	2011	6	15	130	6.45	28.67
22010671	2011	6	15	140	7.59	28.66
22010671	2011	6	15	150	8.3	28.65
22010671	2011	6	15	160	8.87	28.65
22010671	2011	6	15	170	8.75	28.65
22010671	2011	6	15	180	8.8	28.65
22010671	2011	6	15	190	10.53	28.64
22010671	2011	6	15	200	11.4	28.64
22010671	2011	6	15	210	11.33	28.64
22010671	2011	6	15	220	11.04	28.64
22010671	2011	6	15	230	11.04	28.64
22010671	2011	6	15	240	11.49	28.64
22010671	2011	6	15	250	11.24	28.64
22010671	2011	6	15	260	11.03	28.64
22010671	2011	6	15	270	11.27	28.64
22010671	2011	6	15	280	11.16	28.64
22010671	2011	6	15	290	11.05	28.65
22010671	2011	6	15	300	11.99	28.64
22010671	2011	6	15	310	13.49	28.63
22010671	2011	6	15	320	14.7	28.62
22010671	2011	6	15	330	15.9	28.61
22010671	2011	6	15	340	17.75	28.6
22010671	2011	6	15	350	19.33	28.58
22010671	2011	6	15	360	21.1	28.57
22010671	2011	6	15	370	22.27	28.56
22010671	2011	6	15	380	23.4	28.55
22010671	2011	6	15	390	24.64	28.54
22010671	2011	6	15	400	26	28.53
22010671	2011	6	15	410	27.94	28.52
22010671	2011	6	15	420	30.34	28.5
22010671	2011	6	15	430	33.17	28.47
22010671	2011	6	15	440	34.88	28.47
22010671	2011	6	15	450	35.86	28.45
22010671	2011	6	15	460	37.65	28.44
22010671	2011	6	15	470	39.31	28.42
22010671	2011	6	15	480	40.81	28.42
22010671	2011	6	15	490	41.64	28.4
22010671	2011	6	15	500	43.03	28.32
22010671	2011	6	15	510	44.26	28.04
22010671	2011	6	15	520	45.39	27.96
22010671	2011	6	15	530	47.08	27.9
22010671	2011	6	15	540	49.69	27.82
22010671	2011	6	15	550	52.38	27.69

22010671	2011	6	15	560	54.23	27.49
22010671	2011	6	15	570	55.72	27.38
22010671	2011	6	15	580	56.99	27.33
22010671	2011	6	15	590	57.77	27.28
22010671	2011	6	15	600	57.64	27.2
22010671	2011	6	15	610	57.8	27.1
22010671	2011	6	15	620	59.28	26.93
22010671	2011	6	15	630	60.25	26.93
22010671	2011	6	15	640	60.56	26.95
22010671	2011	6	15	650	60.94	26.94
22010671	2011	6	15	660	61.99	26.89
22010671	2011	6	15	670	63.33	26.82
22010671	2011	6	15	680	64.75	26.75
22010671	2011	6	15	690	65.47	26.68
22010671	2011	6	15	700	65.68	26.64
22010671	2011	6	15	710	65.87	26.57
22010671	2011	6	15	720	66.37	26.5
22010671	2011	6	15	730	66.82	26.46
22010671	2011	6	15	740	67.34	26.43
22010671	2011	6	15	750	67.29	26.39
22010671	2011	6	15	760	66.68	26.35
22010671	2011	6	15	770	66.38	26.29
22010671	2011	6	15	780	66.02	26.25
22010671	2011	6	15	790	65.58	26.22
22010671	2011	6	15	800	65.78	26.2
22010671	2011	6	15	810	66.18	26.15
22010671	2011	6	15	820	64.63	26.12
22010671	2011	6	15	830	63.38	26.1
22010671	2011	6	15	840	62.93	26.07
22010671	2011	6	15	850	62.19	26.05
22010671	2011	6	15	860	61.66	26.03
22010671	2011	6	15	870	61.32	26.01
22010671	2011	6	15	880	61.01	26
22010671	2011	6	15	890	60.73	25.99
22010671	2011	6	15	900	60.42	25.98
22010671	2011	6	15	910	61.42	25.94
22010671	2011	6	15	920	61.6	25.92
22010671	2011	6	15	930	60.58	25.92
22010671	2011	6	15	940	59.63	25.92
22010671	2011	6	15	950	58.75	25.92
22010671	2011	6	15	960	57.95	25.92
22010671	2011	6	15	970	57.13	25.92
22010671	2011	6	15	980	56.37	25.92
22010671	2011	6	15	990	56.14	25.92
22010671	2011	6	15	1000	55.13	25.92
22010671	2011	6	15	1010	54.52	25.92
22010671	2011	6	15	1020	54.1	25.92

22010671	2011	6	15	1030	53.82	25.93
22010671	2011	6	15	1040	54.57	25.93
22010671	2011	6	15	1050	55.1	25.92
22010671	2011	6	15	1060	56.28	25.92
22010671	2011	6	15	1070	57.09	25.92
22010671	2011	6	15	1080	56.93	25.92
22010671	2011	6	15	1090	57.07	25.92
22010671	2011	6	15	1100	56.81	25.92
22010671	2011	6	15	1110	55.47	25.93
22010671	2011	6	15	1120	54.17	25.94
22010671	2011	6	15	1130	53.65	25.95
22010671	2011	6	15	1140	52.56	25.95
22010671	2011	6	15	1150	51.98	25.96
22010671	2011	6	15	1160	51.93	25.96
22010671	2011	6	15	1170	51.88	25.96
22010671	2011	6	15	1180	52.03	25.97
22010671	2011	6	15	1190	52.25	25.97
22010671	2011	6	15	1200	52.13	25.98
22010671	2011	6	15	1210	51.93	25.99
22010671	2011	6	15	1220	51.28	26.01
22010671	2011	6	15	1230	51.51	26.01
22010671	2011	6	15	1240	51.5	26.01
22010671	2011	6	15	1250	51.32	26.03
22010671	2011	6	15	1260	51.43	26.04
22010671	2011	6	15	1270	51.5	26.05
22010671	2011	6	15	1280	51.66	26.06
22010671	2011	6	15	1290	51.69	26.07
22010671	2011	6	15	1300	52.37	26.07
22010671	2011	6	15	1310	52.49	26.08
22010671	2011	6	15	1320	52.45	26.08
22010671	2011	6	15	1330	53.12	26.09
22010671	2011	6	15	1340	53.05	26.07
22010671	2011	6	15	1350	51.76	26.08
22010671	2011	6	15	1360	52.05	26.08
22010671	2011	6	15	1370	53.18	26.06
22010671	2011	6	15	1380	53.04	26.07
22010671	2011	6	15	1390	53.07	26.06
22010671	2011	6	15	1400	53.09	26.06
22010671	2011	6	15	1410	53.03	26.06
22010671	2011	6	15	1420	52.59	26.05
22010671	2011	6	15	1430	52.82	26.06
22010671	2011	6	15	1440	52.76	26.05
22010671	2011	6	15	1450	52.8	26.05
22010671	2011	6	15	1460	53.15	26.05
22010671	2011	6	15	1470	53.1	26.05
22010671	2011	6	15	1480	53.16	26.04
22010671	2011	6	15	1490	53.25	26.04

22010671	2011	6	15	1500	53.26	26.03
22010671	2011	6	15	1510	53.3	26.03
22010671	2011	6	15	1520	53.93	26.03
22010671	2011	6	15	1530	53.9	26.02
22010671	2011	6	15	1540	53.79	26.03
22010671	2011	6	15	1550	53.92	26.01
22010671	2011	6	15	1560	53.9	26.02
22010671	2011	6	15	1570	53.42	26.03
22010671	2011	6	15	1580	53.21	26.02
22010671	2011	6	15	1590	53.93	26
22010671	2011	6	15	1600	54.08	26.01
22010671	2011	6	15	1610	53.86	26
22010671	2011	6	15	1620	54.13	26
22010671	2011	6	15	1630	53.96	26
22010671	2011	6	15	1640	53.92	26
22010671	2011	6	15	1650	54.08	26
22010671	2011	6	15	1660	53.92	26
22010671	2011	6	15	1670	54.13	26
22010671	2011	6	15	1680	54.39	25.99
22010671	2011	6	15	1690	54.82	25.98
22010671	2011	6	15	1700	54.85	25.98
22010671	2011	6	15	1710	54.36	25.98
22010671	2011	6	15	1720	54.39	26
22010671	2011	6	15	1730	54.4	25.98
22010671	2011	6	15	1740	54.02	25.98
22010671	2011	6	15	1750	52.99	25.99
22010671	2011	6	15	1760	51.58	26
22010671	2011	6	15	1770	50.6	26
22010671	2011	6	15	1780	50.04	26.01
22010671	2011	6	15	1790	49.93	26.01
22010671	2011	6	15	1800	49.91	26.02
22010671	2011	6	15	1810	49.53	26.03
22010671	2011	6	15	1820	48.96	26.03
22010671	2011	6	15	1830	48.39	26.05
22010671	2011	6	15	1840	46.56	26.08
22010671	2011	6	15	1850	46.17	26.08
22010671	2011	6	15	1860	46.17	26.1
22010671	2011	6	15	1870	46.11	26.11
22010671	2011	6	15	1880	45.73	26.14
22010671	2011	6	15	1890	45.65	26.14
22010671	2011	6	15	1900	45.12	26.17
22010671	2011	6	15	1910	45.01	26.18
22010671	2011	6	15	1920	44.9	26.2
22010671	2011	6	15	1930	44.89	26.21
22010671	2011	6	15	1940	43.89	26.24
22010671	2011	6	15	1950	43.94	26.26
22010671	2011	6	15	1960	44.06	26.27

22010671	2011	6	15	1970	43.95	26.28
22010671	2011	6	15	1980	43.93	26.32
22010671	2011	6	15	1990	44.84	26.34
22010671	2011	6	15	2000	45.48	26.36
22010671	2011	6	15	2010	45.9	26.36
22010671	2011	6	15	2020	46.04	26.37
22010671	2011	6	15	2030	45.09	26.39
22010671	2011	6	15	2040	45.52	26.39
22010671	2011	6	15	2050	45.78	26.4
22010671	2011	6	15	2060	45.64	26.41
22010671	2011	6	15	2070	45.47	26.41
22010671	2011	6	15	2080	45.07	26.42
22010671	2011	6	15	2090	45.04	26.43
22010671	2011	6	15	2100	45.1	26.44
22010671	2011	6	15	2110	45.28	26.44
22010671	2011	6	15	2120	45.11	26.45
22010671	2011	6	15	2130	45.19	26.47
22010671	2011	6	15	2140	45.24	26.48
22010671	2011	6	15	2150	45.19	26.49
22010671	2011	6	15	2160	45.36	26.5
22010671	2011	6	15	2170	45.34	26.5
22010671	2011	6	15	2180	45.21	26.52
22010671	2011	6	15	2190	45.52	26.52
22010671	2011	6	15	2200	46.15	26.51
22010671	2011	6	15	2210	46.15	26.52
22010671	2011	6	15	2220	45.28	26.53
22010671	2011	6	15	2230	45.61	26.55
22010671	2011	6	15	2240	46.59	26.57
22010671	2011	6	15	2250	46.93	26.57
22010671	2011	6	15	2260	46.57	26.59
22010671	2011	6	15	2270	46.54	26.6
22010671	2011	6	15	2280	46.84	26.6
22010671	2011	6	15	2290	46.84	26.6
22010671	2011	6	15	2300	47.16	26.6
22010671	2011	6	15	2310	47.73	26.6
22010671	2011	6	15	2320	48.13	26.59
22010671	2011	6	15	2330	48.52	26.57
22010671	2011	6	15	2340	48.67	26.57
22010671	2011	6	15	2350	49.04	26.56
22010671	2011	6	15	2360	47.98	26.57
22010671	2011	6	15	2370	47.21	26.57
22010671	2011	6	15	2380	46.92	26.57
22010671	2011	6	15	2390	46.84	26.56
22010671	2011	6	15	2400	46.76	26.56
22010671	2011	6	15	2410	46.59	26.55
22010671	2011	6	15	2420	46.32	26.54
22010671	2011	6	15	2430	45.97	26.55

22010671	2011	6	15	2440	45.93	26.54
22010671	2011	6	15	2450	46.04	26.53
22010671	2011	6	15	2460	45.9	26.54
22010671	2011	6	15	2470	45.93	26.57
22010671	2011	6	15	2480	46.34	26.58
22010671	2011	6	15	2490	46.38	26.61
22010671	2011	6	15	2500	46.2	26.61
22010671	2011	6	15	2510	45.95	26.63
22010671	2011	6	15	2520	45.64	26.64
22010671	2011	6	15	2530	44.91	26.67
22010671	2011	6	15	2540	45	26.7
22010671	2011	6	15	2550	44.32	26.72
22010671	2011	6	15	2560	44.36	26.76
22010671	2011	6	15	2570	44.57	26.79
22010671	2011	6	15	2580	44.37	26.81
22010671	2011	6	15	2590	44.62	26.82
22010671	2011	6	15	2600	44.66	26.85
22010671	2011	6	15	2610	44.37	26.85
22010671	2011	6	15	2620	43.89	26.87
22010671	2011	6	15	2630	44.36	26.9
22010671	2011	6	15	2640	44.55	26.9
22010671	2011	6	15	2650	44.63	26.92
22010671	2011	6	15	2660	44.51	26.93
22010671	2011	6	15	2670	44.82	26.94
22010671	2011	6	15	2680	45.66	26.95
22010671	2011	6	15	2690	46.79	26.92
22010671	2011	6	15	2700	47.73	26.85
22010671	2011	6	15	2710	48.69	26.83
22010671	2011	6	15	2720	49.98	26.76
22010671	2011	6	15	2730	50.73	26.75
22010671	2011	6	15	2740	52.43	26.74
22010671	2011	6	15	2750	53.23	26.71
22010671	2011	6	15	2760	53.44	26.68
22010671	2011	6	15	2770	53.35	26.67
22010671	2011	6	15	2780	53.34	26.66
22010671	2011	6	15	2790	53.49	26.64
22010671	2011	6	15	2800	53.36	26.61
22010671	2011	6	15	2810	53.33	26.6
22010671	2011	6	15	2820	53.45	26.57
22010671	2011	6	15	2830	53.59	26.57
22010671	2011	6	15	2840	53.83	26.54
22010671	2011	6	15	2850	54.03	26.53
22010671	2011	6	15	2860	53.85	26.52
22010671	2011	6	15	2870	54.19	26.5
22010671	2011	6	15	2880	54.18	26.48
22010671	2011	6	15	2890	54.5	26.46
22010671	2011	6	15	2900	54.68	26.46

22010671	2011	6	15	2910	55.01	26.44
22010671	2011	6	15	2920	55.87	26.42
22010671	2011	6	15	2930	55.8	26.41
22010671	2011	6	15	2940	55.8	26.4
22010671	2011	6	15	2950	55.75	26.39
22010671	2011	6	15	2960	55.66	26.38
22010671	2011	6	15	2970	56	26.36
22010671	2011	6	15	2980	55.8	26.35
22010671	2011	6	15	2990	55.21	26.34
22010671	2011	6	15	3000	53.61	26.35
22010671	2011	6	15	3010	51.93	26.35
22010671	2011	6	15	3020	51.79	26.34
22010671	2011	6	15	3030	51.61	26.33
22010671	2011	6	15	3040	51.26	26.33
22010671	2011	6	15	3050	51.09	26.33
22010671	2011	6	15	3060	51.24	26.32
22010671	2011	6	15	3070	51.48	26.32
22010671	2011	6	15	3080	51.72	26.32
22010671	2011	6	15	3090	51.55	26.32
22010671	2011	6	15	3100	51.47	26.32
22010671	2011	6	15	3110	51.78	26.31
22010671	2011	6	15	3120	51.78	26.31
22010671	2011	6	15	3130	51.63	26.31
22010671	2011	6	15	3140	51.27	26.31
22010671	2011	6	15	3150	50.94	26.3
22010671	2011	6	15	3160	51.45	26.3
22010671	2011	6	15	3170	51.4	26.3
22010671	2011	6	15	3180	51.28	26.3
22010671	2011	6	15	3190	51.15	26.3
22010671	2011	6	15	3200	51.06	26.29
22010671	2011	6	15	3210	51.18	26.29
22010671	2011	6	15	3220	51.07	26.29
22010671	2011	6	15	3230	50.84	26.29
22010671	2011	6	15	3240	50.91	26.28
22010671	2011	6	15	3250	50.54	26.3
22010671	2011	6	15	3260	50.83	26.29
22010671	2011	6	15	3270	51.6	26.28
22010671	2011	6	15	3280	51.62	26.28
22010671	2011	6	15	3290	51.4	26.28
22010671	2011	6	15	3300	51.26	26.28
22010671	2011	6	15	3310	51.04	26.28
22010671	2011	6	15	3320	51.01	26.28
22010671	2011	6	15	3330	50.97	26.28
22010671	2011	6	15	3340	50.76	26.28
22010671	2011	6	15	3350	51.03	26.28
22010671	2011	6	15	3360	50.91	26.28
22010671	2011	6	15	3370	51.29	26.28

22010671	2011	6	15	3380	51.72	26.27
22010671	2011	6	15	3390	51.53	26.28
22010671	2011	6	15	3400	51.53	26.28
22010671	2011	6	15	3410	51.25	26.28
22010671	2011	6	15	3420	50.96	26.28
22010671	2011	6	15	3430	51.33	26.28
22010671	2011	6	15	3440	50.93	26.28
22010671	2011	6	15	3450	50.61	26.27
22010671	2011	6	15	3460	50.73	26.28
22010671	2011	6	15	3470	50.96	26.28
22010671	2011	6	15	3480	51.3	26.27
22010671	2011	6	15	3490	52.36	26.26
22010671	2011	6	15	3500	54.07	26.25
22010671	2011	6	15	3510	56.18	26.25
22010671	2011	6	15	3520	58.17	26.22
22010671	2011	6	15	3530	60.43	26.19
22010671	2011	6	15	3540	63.35	26.17
22010671	2011	6	15	3550	64.87	26.14
22010671	2011	6	15	3560	66.39	26.11
22010671	2011	6	15	3570	67.84	26.07
22010671	2011	6	15	3580	69.77	25.99
22010671	2011	6	15	3590	71.9	25.96
22010671	2011	6	15	3600	74.13	25.9
22010671	2011	6	15	3610	76.37	25.87
22010671	2011	6	15	3620	78.72	25.83
22010671	2011	6	15	3630	80.94	25.75
22010671	2011	6	15	3640	82.4	25.65
22010671	2011	6	15	3650	83.84	25.53
22010671	2011	6	15	3660	84.63	25.46
22010671	2011	6	15	3670	84.85	25.39
22010671	2011	6	15	3680	84.8	25.32
22010671	2011	6	15	3690	85.28	25.14
22010671	2011	6	15	3700	85.25	25.1
22010671	2011	6	15	3710	85.33	25.03
22010671	2011	6	15	3720	85.42	24.97
22010671	2011	6	15	3730	85.36	24.91
22010671	2011	6	15	3740	85.38	24.86
22010671	2011	6	15	3750	85.01	24.79
22010671	2011	6	15	3760	85.39	24.6
22010671	2011	6	15	3770	85.64	24.51
22010671	2011	6	15	3780	85.73	24.46
22010671	2011	6	15	3790	87.27	24.42
22010671	2011	6	15	3800	88.65	24.3
22010671	2011	6	15	3810	88.81	24.25
22010671	2011	6	15	3820	88.76	24.18
22010671	2011	6	15	3830	89.77	24.13
22010671	2011	6	15	3840	91.33	24.05

22010671	2011	6	15	3850	91.51	23.98
22010671	2011	6	15	3860	91.06	23.93
22010671	2011	6	15	3870	91.78	23.81
22010671	2011	6	15	3880	92.17	23.72
22010671	2011	6	15	3890	92.5	23.67
22010671	2011	6	15	3900	92.62	23.64
22010671	2011	6	15	3910	92.51	23.6
22010671	2011	6	15	3920	93.2	23.53
22010671	2011	6	15	3930	93.36	23.48
22010671	2011	6	15	3940	93.2	23.42
22010671	2011	6	15	3950	93.79	23.32
22010671	2011	6	15	3960	94.88	23.25
22010671	2011	6	15	3970	95.1	23.19
22010671	2011	6	15	3980	94.28	23.16
22010671	2011	6	15	3990	94.38	23.12
22010671	2011	6	15	4000	94.6	23.08
22010671	2011	6	15	4010	95.41	23.01
22010671	2011	6	15	4020	95.74	22.97
22010671	2011	6	15	4030	95.97	22.94
22010671	2011	6	15	4040	96.27	22.91
22010671	2011	6	15	4050	96.17	22.88
22010671	2011	6	15	4060	96.51	22.85
22010671	2011	6	15	4070	97.23	22.8
22010671	2011	6	15	4080	97.72	22.77
22010671	2011	6	15	4090	98.45	22.75
22010671	2011	6	15	4100	99.04	22.71
22010671	2011	6	15	4110	100.25	22.66
22010671	2011	6	15	4120	101.12	22.64
22010671	2011	6	15	4130	102.08	22.61
22010671	2011	6	15	4140	103.52	22.56
22010671	2011	6	15	4150	104.64	22.53
22010671	2011	6	15	4160	105.54	22.52
22010671	2011	6	15	4170	106.25	22.5
22010671	2011	6	15	4180	106.47	22.49
22010671	2011	6	15	4190	106.8	22.48
22010671	2011	6	15	4200	107	22.45
22010671	2011	6	15	4210	107.75	22.43
22010671	2011	6	15	4220	108.02	22.42
22010671	2011	6	15	4230	108.07	22.4
22010671	2011	6	15	4240	108.19	22.39
22010671	2011	6	15	4250	108.14	22.38
22010671	2011	6	15	4260	107.98	22.37
22010671	2011	6	15	4270	107.87	22.35
22010671	2011	6	15	4280	108.41	22.33
22010671	2011	6	15	4290	109.17	22.27
22010671	2011	6	15	4300	109.82	22.23
22010671	2011	6	15	4310	110.67	22.14

22010671	2011	6	15	4320	111.34	22.08
22010671	2011	6	15	4330	111.85	22
22010671	2011	6	15	4340	112.41	21.93
22010671	2011	6	15	4350	113.62	21.84
22010671	2011	6	15	4360	114.29	21.78
22010671	2011	6	15	4370	115.27	21.71
22010671	2011	6	15	4380	115.53	21.64
22010671	2011	6	15	4390	116.19	21.57
22010671	2011	6	15	4400	116.37	21.52
22010671	2011	6	15	4410	116.83	21.44
22010671	2011	6	15	4420	116.64	21.39
22010671	2011	6	15	4430	116.35	21.36
22010671	2011	6	15	4440	117.05	21.31
22010671	2011	6	15	4450	117.25	21.25
22010671	2011	6	15	4460	117.98	21.19
22010671	2011	6	15	4470	119.03	21.09
22010671	2011	6	15	4480	120.34	21
22010671	2011	6	15	4490	121.02	20.99
22010671	2011	6	15	4500	121.09	20.98
22010671	2011	6	15	4510	121.05	20.94
22010671	2011	6	15	4520	121.17	20.9
22010671	2011	6	15	4530	121.42	20.85
22010671	2011	6	15	4540	121.93	20.8
22010671	2011	6	15	4550	122.28	20.74
22010671	2011	6	15	4560	123.32	20.66
22010671	2011	6	15	4570	123.67	20.64
22010671	2011	6	15	4580	124.02	20.58
22010671	2011	6	15	4590	124.33	20.52
22010671	2011	6	15	4600	125.28	20.48
22010671	2011	6	15	4610	125.99	20.44
22010671	2011	6	15	4620	127.02	20.39
22010671	2011	6	15	4630	128.39	20.35
22010671	2011	6	15	4640	129.14	20.31
22010671	2011	6	15	4650	129.3	20.28
22010671	2011	6	15	4660	130.24	20.24
22010671	2011	6	15	4670	130.1	20.21
22010671	2011	6	15	4680	130.43	20.19
22010671	2011	6	15	4690	131.72	20.13
22010671	2011	6	15	4700	132.08	20.07
22010671	2011	6	15	4710	132.8	20.03
22010671	2011	6	15	4720	133.59	19.96
22010671	2011	6	15	4730	134.55	19.88
22010671	2011	6	15	4740	135.1	19.82
22010671	2011	6	15	4750	135.83	19.78
22010671	2011	6	15	4760	135.99	19.76
22010671	2011	6	15	4770	136.04	19.71
22010671	2011	6	15	4780	136.09	19.64

22010671	2011	6	15	4790	136.02	19.62
22010671	2011	6	15	4800	136.01	19.6
22010671	2011	6	15	4810	136.97	19.53
22010671	2011	6	15	4820	136.91	19.52
22010671	2011	6	15	4830	136.46	19.49
22010671	2011	6	15	4840	136.75	19.44
22010671	2011	6	15	4850	136.52	19.42
22010671	2011	6	15	4860	136.26	19.39
22010671	2011	6	15	4870	136.57	19.35
22010671	2011	6	15	4880	136.57	19.32
22010671	2011	6	15	4890	136.03	19.28
22010671	2011	6	15	4900	135.76	19.27
22010671	2011	6	15	4910	136.17	19.21
22010671	2011	6	15	4920	135.8	19.2
22010671	2011	6	15	4930	135.65	19.19
22010671	2011	6	15	4940	135.98	19.15
22010671	2011	6	15	4950	136.13	19.14
22010671	2011	6	15	4960	135.86	19.1
22010671	2011	6	15	4970	135.94	19.1
22010671	2011	6	15	4980	135.82	19.08
22010671	2011	6	15	4990	135.53	19.07
22010671	2011	6	15	5000	135.63	19.04
22010671	2011	6	15	5010	135.54	19.03
22010671	2011	6	15	5020	135.58	19.01
22010671	2011	6	15	5030	135.64	19
22010671	2011	6	15	5040	135.57	18.97
22010671	2011	6	15	5050	135.56	18.96
22010671	2011	6	15	5060	135.48	18.95
22010671	2011	6	15	5070	134.42	18.92
22010671	2011	6	15	5080	132.52	18.92
22010671	2011	6	15	5090	130.96	18.92
22010671	2011	6	15	5100	129.31	18.91
22010671	2011	6	15	5110	128.79	18.94
22010671	2011	6	15	5120	128.13	18.97
22010671	2011	6	15	5130	127.12	19.03
22010671	2011	6	15	5140	126.3	19.06
22010671	2011	6	15	5150	125.96	19.09
22010671	2011	6	15	5160	125.49	19.14
22010671	2011	6	15	5170	123.67	19.15
22010671	2011	6	15	5180	121.77	19.2
22010671	2011	6	15	5190	119.67	19.22
22010671	2011	6	15	5200	118.99	19.26
22010671	2011	6	15	5210	118.54	19.3
22010671	2011	6	15	5220	118.05	19.36
22010671	2011	6	15	5230	117.94	19.39
22010671	2011	6	15	5240	117.65	19.42
22010671	2011	6	15	5250	117.49	19.44

22010671	2011	6	15	5260	117.56	19.48
22010671	2011	6	15	5270	117.76	19.5
22010671	2011	6	15	5280	118.02	19.53
22010671	2011	6	15	5290	117.96	19.55
22010671	2011	6	15	5300	117.98	19.58
22010671	2011	6	15	5310	118.07	19.62
22010671	2011	6	15	5320	118.27	19.65
22010671	2011	6	15	5330	118.45	19.67
22010671	2011	6	15	5340	118.75	19.69
22010671	2011	6	15	5350	118.53	19.73
22010671	2011	6	15	5360	117.4	19.75
22010671	2011	6	15	5370	115.42	19.77
22010671	2011	6	15	5380	114.19	19.79
22010671	2011	6	15	5390	112.25	19.82
22010671	2011	6	15	5400	110.37	19.86
22010671	2011	6	15	5410	108.4	19.92
22010671	2011	6	15	5420	106.21	19.96
22010671	2011	6	15	5430	103.95	20.01
22010671	2011	6	15	5440	101.53	20.08
22010671	2011	6	15	5450	99.49	20.16
22010671	2011	6	15	5460	97.41	20.26
22010671	2011	6	15	5470	96.13	20.38
22010671	2011	6	15	5480	96.03	20.49
22010671	2011	6	15	5490	96.79	20.61
22010671	2011	6	15	5500	95.7	20.7
22010671	2011	6	15	5510	94.58	20.78
22010671	2011	6	15	5520	92.78	20.89
22010671	2011	6	15	5530	91.18	21
22010671	2011	6	15	5540	90.14	21.12
22010671	2011	6	15	5550	88.79	21.21
22010671	2011	6	15	5560	86.92	21.36
22010671	2011	6	15	5570	84.48	21.43
22010671	2011	6	15	5580	82.45	21.57
22010671	2011	6	15	5590	80.1	21.72
22010671	2011	6	15	5600	77.42	21.84
22010671	2011	6	15	5610	74.85	22
22010671	2011	6	15	5620	72.81	22.17
22010671	2011	6	15	5630	70.78	22.38
22010671	2011	6	15	5640	68.56	22.54
22010671	2011	6	15	5650	66.46	22.78
22010671	2011	6	15	5660	64.21	23.04
22010671	2011	6	15	5670	63.85	23.24
22010671	2011	6	15	5680	64.93	23.33
22010671	2011	6	15	5690	63.93	23.45
22010671	2011	6	15	5700	61.85	23.57
22010671	2011	6	15	5710	60.02	23.67
22010671	2011	6	15	5720	58.13	23.81

22010671	2011	6	15	5730	57.13	23.9
22010671	2011	6	15	5740	55.5	23.99
22010671	2011	6	15	5750	55.93	24.19
22010671	2011	6	15	5760	56.73	24.34
22010671	2011	6	15	5770	57.18	24.38
22010671	2011	6	15	5780	57.35	24.42
22010671	2011	6	15	5790	56.8	24.5
22010671	2011	6	15	5800	55.4	24.58
22010671	2011	6	15	5810	54.91	24.65
22010671	2011	6	15	5820	53.87	24.72
22010671	2011	6	15	5830	52.92	24.81
22010671	2011	6	15	5840	51.51	24.92
22010671	2011	6	15	5850	49.81	25
22010671	2011	6	15	5860	49.06	25.11
22010671	2011	6	15	5870	47.52	25.24
22010671	2011	6	15	5880	46.19	25.34
22010671	2011	6	15	5890	44.61	25.53
22010671	2011	6	15	5900	42.81	25.67
22010671	2011	6	15	5910	41.5	25.8
22010671	2011	6	15	5920	41.66	25.92
22010671	2011	6	15	5930	42.71	26.07
22010671	2011	6	15	5940	42.35	26.17
22010671	2011	6	15	5950	40.94	26.35
22010671	2011	6	15	5960	39.3	26.46
22010671	2011	6	15	5970	38.06	26.55
22010671	2011	6	15	5980	36.93	26.64
22010671	2011	6	15	5990	36.8	26.73
22010671	2011	6	15	6000	35.87	26.82
22010671	2011	6	15	6010	34.89	26.91
22010671	2011	6	15	6020	34.4	27.02
22010671	2011	6	15	6030	34.27	27.07
22010671	2011	6	15	6040	32.72	27.17
22010671	2011	6	15	6050	30.52	27.24
22010671	2011	6	15	6060	28.19	27.31
22010671	2011	6	15	6070	25.97	27.37
22010671	2011	6	15	6080	23.61	27.45
22010671	2011	6	15	6090	21.22	27.52
22010671	2011	6	15	6100	18.89	27.6
22010671	2011	6	15	6110	16.54	27.66
22010671	2011	6	15	6120	14.22	27.74
22010671	2011	6	15	6130	14.28	27.84
22010671	2011	6	15	6140	14.31	27.88
22010671	2011	6	15	6150	12.85	27.92
22010671	2011	6	15	6160	11.9	27.96
22010671	2011	6	15	6170	10	28
22010671	2011	6	15	6180	7.56	28.05
22010671	2011	6	15	6190	5.58	28.1

22010671	2011	6	15	6200	4.38	28.14
22010671	2011	6	15	6210	2.81	28.18
22010671	2011	6	15	6220	2.65	28.22
22010671	2011	6	15	6230	2.94	28.25
22010671	2011	6	15	6240	2.55	28.27
22010671	2011	6	15	6250	2.78	28.29
22010671	2011	6	15	6260	2.46	28.32
22010671	2011	6	15	6270	2.39	28.35
22010671	2011	6	15	6280	0.71	28.38
22010671	2011	6	15	6290	0.66	28.41
22010671	2011	6	15	6300	0.65	28.42
22010671	2011	6	15	6310	0.6	28.44
22010671	2011	6	15	6320	0.59	28.47
22010671	2011	6	15	6330	0.66	28.5
22010671	2011	6	15	6340	0.59	28.51
22010671	2011	6	15	6350	0.61	28.53
22010671	2011	6	15	6360	0.62	28.55
22010671	2011	6	15	6370	0.66	28.57
22010671	2011	6	15	6380	0.62	28.59
22010671	2011	6	15	6390	0.73	28.6
22010671	2011	6	15	6400	0.55	28.62
22010671	2011	6	15	6410	0.57	28.64
22010671	2011	6	15	6420	0.54	28.65
22190542	2011	6	17	0	0.93	28.82
22190542	2011	6	17	10	1.23	28.82
22190542	2011	6	17	20	0.99	28.82
22190542	2011	6	17	30	1.51	28.81
22190542	2011	6	17	40	2.46	28.81
22190542	2011	6	17	50	2.65	28.81
22190542	2011	6	17	60	2.43	28.82
22190542	2011	6	17	70	1.95	28.81
22190542	2011	6	17	80	2.39	28.81
22190542	2011	6	17	90	2.67	28.8
22190542	2011	6	17	100	2.44	28.81
22190542	2011	6	17	110	2.58	28.81
22190542	2011	6	17	120	2.96	28.81
22190542	2011	6	17	130	3.57	28.81
22190542	2011	6	17	140	4.72	28.85
22190542	2011	6	17	150	6.43	28.85
22190542	2011	6	17	160	7.68	28.82
22190542	2011	6	17	170	8.18	28.82
22190542	2011	6	17	180	8.08	28.82
22190542	2011	6	17	190	8.06	28.81
22190542	2011	6	17	200	8.05	28.81
22190542	2011	6	17	210	8.18	28.8
22190542	2011	6	17	220	8.14	28.79
22190542	2011	6	17	230	8.03	28.78

22190542	2011	6	17	240	7.93	28.79
22190542	2011	6	17	250	7.78	28.79
22190542	2011	6	17	260	7.69	28.78
22190542	2011	6	17	270	7.75	28.78
22190542	2011	6	17	280	7.75	28.78
22190542	2011	6	17	290	7.79	28.78
22190542	2011	6	17	300	7.71	28.78
22190542	2011	6	17	310	7.63	28.77
22190542	2011	6	17	320	7.9	28.76
22190542	2011	6	17	330	7.99	28.76
22190542	2011	6	17	340	9.62	28.75
22190542	2011	6	17	350	11.63	28.72
22190542	2011	6	17	360	13.41	28.71
22190542	2011	6	17	370	15.24	28.69
22190542	2011	6	17	380	17.43	28.67
22190542	2011	6	17	390	19.18	28.64
22190542	2011	6	17	400	21.58	28.63
22190542	2011	6	17	410	23.01	28.6
22190542	2011	6	17	420	25.16	28.57
22190542	2011	6	17	430	27.46	28.56
22190542	2011	6	17	440	29.75	28.53
22190542	2011	6	17	450	31.87	28.5
22190542	2011	6	17	460	34.12	28.46
22190542	2011	6	17	470	36.49	28.42
22190542	2011	6	17	480	37.78	28.39
22190542	2011	6	17	490	38.04	28.38
22190542	2011	6	17	500	38.37	28.36
22190542	2011	6	17	510	40.41	28.35
22190542	2011	6	17	520	42.85	28.29
22190542	2011	6	17	530	44.62	28.27
22190542	2011	6	17	540	45.03	28.25
22190542	2011	6	17	550	46.23	28.23
22190542	2011	6	17	560	48.51	28.16
22190542	2011	6	17	570	49.72	28.12
22190542	2011	6	17	580	50.36	28.07
22190542	2011	6	17	590	50.67	28.03
22190542	2011	6	17	600	50.95	27.96
22190542	2011	6	17	610	50.9	27.87
22190542	2011	6	17	620	50.85	27.78
22190542	2011	6	17	630	50.93	27.7
22190542	2011	6	17	640	50.94	27.61
22190542	2011	6	17	650	50.88	27.53
22190542	2011	6	17	660	50.81	27.47
22190542	2011	6	17	670	50.86	27.39
22190542	2011	6	17	680	50.98	27.31
22190542	2011	6	17	690	50.75	27.25
22190542	2011	6	17	700	50.86	27.19

22190542	2011	6	17	710	50.92	27.13
22190542	2011	6	17	720	50.94	27.07
22190542	2011	6	17	730	50.86	27.03
22190542	2011	6	17	740	50.75	26.98
22190542	2011	6	17	750	50.69	26.95
22190542	2011	6	17	760	50.82	26.91
22190542	2011	6	17	770	50.88	26.86
22190542	2011	6	17	780	50.75	26.82
22190542	2011	6	17	790	50.68	26.78
22190542	2011	6	17	800	50.9	26.75
22190542	2011	6	17	810	50.02	26.71
22190542	2011	6	17	820	49.07	26.68
22190542	2011	6	17	830	48.5	26.66
22190542	2011	6	17	840	48	26.64
22190542	2011	6	17	850	47.87	26.59
22190542	2011	6	17	860	47.95	26.61
22190542	2011	6	17	870	48.01	26.61
22190542	2011	6	17	880	47.99	26.61
22190542	2011	6	17	890	47.85	26.62
22190542	2011	6	17	900	48.95	26.61
22190542	2011	6	17	910	50.93	26.57
22190542	2011	6	17	920	51.87	26.54
22190542	2011	6	17	930	51.74	26.53
22190542	2011	6	17	940	51.87	26.52
22190542	2011	6	17	950	51.76	26.5
22190542	2011	6	17	960	51.89	26.46
22190542	2011	6	17	970	52.12	26.44
22190542	2011	6	17	980	52.02	26.42
22190542	2011	6	17	990	52.13	26.41
22190542	2011	6	17	1000	52.81	26.37
22190542	2011	6	17	1010	53.18	26.34
22190542	2011	6	17	1020	53.28	26.32
22190542	2011	6	17	1030	53.23	26.29
22190542	2011	6	17	1040	53.12	26.28
22190542	2011	6	17	1050	53.14	26.26
22190542	2011	6	17	1060	53.5	26.25
22190542	2011	6	17	1070	53.6	26.23
22190542	2011	6	17	1080	53.43	26.23
22190542	2011	6	17	1090	53	26.21
22190542	2011	6	17	1100	52.7	26.2
22190542	2011	6	17	1110	52.57	26.18
22190542	2011	6	17	1120	52.11	26.17
22190542	2011	6	17	1130	51.52	26.17
22190542	2011	6	17	1140	50.82	26.16
22190542	2011	6	17	1150	50.49	26.16
22190542	2011	6	17	1160	51.25	26.14
22190542	2011	6	17	1170	52.12	26.11

22190542	2011	6	17	1180	52.86	26.11
22190542	2011	6	17	1190	51.98	26.1
22190542	2011	6	17	1200	50.32	26.11
22190542	2011	6	17	1210	48.31	26.11
22190542	2011	6	17	1220	45.91	26.13
22190542	2011	6	17	1230	44.8	26.14
22190542	2011	6	17	1240	43.99	26.14
22190542	2011	6	17	1250	43.56	26.14
22190542	2011	6	17	1260	43.32	26.15
22190542	2011	6	17	1270	42.78	26.17
22190542	2011	6	17	1280	42.17	26.18
22190542	2011	6	17	1290	42.25	26.21
22190542	2011	6	17	1300	42.3	26.27
22190542	2011	6	17	1310	42.48	26.35
22190542	2011	6	17	1320	42.61	26.42
22190542	2011	6	17	1330	42.75	26.52
22190542	2011	6	17	1340	42.72	26.63
22190542	2011	6	17	1350	42.31	26.66
22190542	2011	6	17	1360	42.79	26.71
22190542	2011	6	17	1370	43.39	26.75
22190542	2011	6	17	1380	43.35	26.81
22190542	2011	6	17	1390	43.26	26.86
22190542	2011	6	17	1400	43.18	26.89
22190542	2011	6	17	1410	43.47	26.92
22190542	2011	6	17	1420	44.43	26.96
22190542	2011	6	17	1430	45.12	26.96
22190542	2011	6	17	1440	45.58	26.98
22190542	2011	6	17	1450	46.02	27
22190542	2011	6	17	1460	46.25	27
22190542	2011	6	17	1470	46.05	27.01
22190542	2011	6	17	1480	44.96	27.02
22190542	2011	6	17	1490	44.03	27.03
22190542	2011	6	17	1500	43.67	27.02
22190542	2011	6	17	1510	44.09	27.03
22190542	2011	6	17	1520	43.95	27.07
22190542	2011	6	17	1530	43.93	27.08
22190542	2011	6	17	1540	44.61	27.1
22190542	2011	6	17	1550	44.61	27.13
22190542	2011	6	17	1560	44.39	27.17
22190542	2011	6	17	1570	44.8	27.19
22190542	2011	6	17	1580	44.98	27.18
22190542	2011	6	17	1590	45.14	27.18
22190542	2011	6	17	1600	45.52	27.19
22190542	2011	6	17	1610	46.04	27.19
22190542	2011	6	17	1620	46.26	27.18
22190542	2011	6	17	1630	46.45	27.17
22190542	2011	6	17	1640	46.8	27.17

22190542	2011	6	17	1650	47.02	27.15
22190542	2011	6	17	1660	46.99	27.14
22190542	2011	6	17	1670	47.11	27.1
22190542	2011	6	17	1680	47.01	27.07
22190542	2011	6	17	1690	47.02	27.05
22190542	2011	6	17	1700	47.02	27.03
22190542	2011	6	17	1710	47.01	27.01
22190542	2011	6	17	1720	46.92	26.96
22190542	2011	6	17	1730	46.65	26.96
22190542	2011	6	17	1740	46.46	26.94
22190542	2011	6	17	1750	46.27	26.94
22190542	2011	6	17	1760	46.53	26.92
22190542	2011	6	17	1770	46.18	26.91
22190542	2011	6	17	1780	46.43	26.89
22190542	2011	6	17	1790	46.42	26.85
22190542	2011	6	17	1800	46.1	26.82
22190542	2011	6	17	1810	45.61	26.83
22190542	2011	6	17	1820	45.09	26.82
22190542	2011	6	17	1830	44.59	26.82
22190542	2011	6	17	1840	44.4	26.81
22190542	2011	6	17	1850	46.01	26.8
22190542	2011	6	17	1860	47.21	26.78
22190542	2011	6	17	1870	48.13	26.77
22190542	2011	6	17	1880	48.6	26.75
22190542	2011	6	17	1890	48.61	26.73
22190542	2011	6	17	1900	48.42	26.71
22190542	2011	6	17	1910	48.3	26.7
22190542	2011	6	17	1920	49.37	26.66
22190542	2011	6	17	1930	50.98	26.59
22190542	2011	6	17	1940	51.61	26.57
22190542	2011	6	17	1950	51.4	26.54
22190542	2011	6	17	1960	51.12	26.53
22190542	2011	6	17	1970	51.23	26.52
22190542	2011	6	17	1980	51.19	26.49
22190542	2011	6	17	1990	51.13	26.46
22190542	2011	6	17	2000	50.97	26.44
22190542	2011	6	17	2010	50.72	26.42
22190542	2011	6	17	2020	50.8	26.39
22190542	2011	6	17	2030	50.81	26.38
22190542	2011	6	17	2040	50.52	26.36
22190542	2011	6	17	2050	50.45	26.34
22190542	2011	6	17	2060	50.57	26.32
22190542	2011	6	17	2070	50.65	26.29
22190542	2011	6	17	2080	51.26	26.28
22190542	2011	6	17	2090	52.22	26.26
22190542	2011	6	17	2100	52.38	26.24
22190542	2011	6	17	2110	52.17	26.23

22190542	2011	6	17	2120	52.11	26.22
22190542	2011	6	17	2130	51.9	26.2
22190542	2011	6	17	2140	51.4	26.19
22190542	2011	6	17	2150	51.39	26.18
22190542	2011	6	17	2160	51.6	26.17
22190542	2011	6	17	2170	52.48	26.15
22190542	2011	6	17	2180	52.92	26.14
22190542	2011	6	17	2190	52.83	26.13
22190542	2011	6	17	2200	52.42	26.12
22190542	2011	6	17	2210	52.47	26.11
22190542	2011	6	17	2220	54.49	26.07
22190542	2011	6	17	2230	57.32	26.05
22190542	2011	6	17	2240	60.49	26
22190542	2011	6	17	2250	63.84	25.91
22190542	2011	6	17	2260	67.25	25.82
22190542	2011	6	17	2270	69.77	25.72
22190542	2011	6	17	2280	70.51	25.7
22190542	2011	6	17	2290	72.39	25.67
22190542	2011	6	17	2300	74.57	25.59
22190542	2011	6	17	2310	76.26	25.46
22190542	2011	6	17	2320	78.31	25.37
22190542	2011	6	17	2330	80.75	25.29
22190542	2011	6	17	2340	82.23	25.15
22190542	2011	6	17	2350	82.81	25.07
22190542	2011	6	17	2360	83.81	25.03
22190542	2011	6	17	2370	84.33	24.96
22190542	2011	6	17	2380	84.26	24.9
22190542	2011	6	17	2390	84.26	24.85
22190542	2011	6	17	2400	84.54	24.77
22190542	2011	6	17	2410	84.58	24.69
22190542	2011	6	17	2420	85.16	24.59
22190542	2011	6	17	2430	85.37	24.52
22190542	2011	6	17	2440	85.3	24.46
22190542	2011	6	17	2450	85.34	24.4
22190542	2011	6	17	2460	85.15	24.33
22190542	2011	6	17	2470	84.82	24.28
22190542	2011	6	17	2480	84.63	24.23
22190542	2011	6	17	2490	84.79	24.16
22190542	2011	6	17	2500	84.53	24.11
22190542	2011	6	17	2510	84.25	24.05
22190542	2011	6	17	2520	83.49	24
22190542	2011	6	17	2530	82.7	23.98
22190542	2011	6	17	2540	81.9	23.94
22190542	2011	6	17	2550	81.14	23.91
22190542	2011	6	17	2560	82.58	23.85
22190542	2011	6	17	2570	85.16	23.78
22190542	2011	6	17	2580	87.97	23.74

22190542	2011	6	17	2590	89.11	23.7
22190542	2011	6	17	2600	89.54	23.64
22190542	2011	6	17	2610	91.25	23.58
22190542	2011	6	17	2620	93.1	23.52
22190542	2011	6	17	2630	95.02	23.45
22190542	2011	6	17	2640	97.11	23.39
22190542	2011	6	17	2650	99.24	23.31
22190542	2011	6	17	2660	101.23	23.21
22190542	2011	6	17	2670	103.23	23.07
22190542	2011	6	17	2680	105.16	22.93
22190542	2011	6	17	2690	107.17	22.79
22190542	2011	6	17	2700	109.36	22.67
22190542	2011	6	17	2710	111.87	22.57
22190542	2011	6	17	2720	114.45	22.31
22190542	2011	6	17	2730	116.84	22.11
22190542	2011	6	17	2740	118.83	21.86
22190542	2011	6	17	2750	120.61	21.8
22190542	2011	6	17	2760	122.42	21.55
22190542	2011	6	17	2770	124.66	21.42
22190542	2011	6	17	2780	127.06	21.19
22190542	2011	6	17	2790	129.61	21.07
22190542	2011	6	17	2800	132.12	20.91
22190542	2011	6	17	2810	133.9	20.88
22190542	2011	6	17	2820	134.36	20.83
22190542	2011	6	17	2830	135.5	20.75
22190542	2011	6	17	2840	136.3	20.67
22190542	2011	6	17	2850	137.1	20.6
22190542	2011	6	17	2860	138.88	20.44
22190542	2011	6	17	2870	140.7	20.17
22190542	2011	6	17	2880	142.59	20.1
22190542	2011	6	17	2890	143.93	20.07
22190542	2011	6	17	2900	144.68	20.05
22190542	2011	6	17	2910	144.8	19.96
22190542	2011	6	17	2920	144.49	19.92
22190542	2011	6	17	2930	144.66	19.85
22190542	2011	6	17	2940	145.35	19.8
22190542	2011	6	17	2950	146.52	19.73
22190542	2011	6	17	2960	147	19.67
22190542	2011	6	17	2970	148.69	19.54
22190542	2011	6	17	2980	150.08	19.49
22190542	2011	6	17	2990	150.75	19.42
22190542	2011	6	17	3000	151.68	19.28
22190542	2011	6	17	3010	153.22	19.17
22190542	2011	6	17	3020	153.59	19.14
22190542	2011	6	17	3030	153.07	19.12
22190542	2011	6	17	3040	152.15	19.1
22190542	2011	6	17	3050	150.9	19.07

22190542	2011	6	17	3060	149.54	19.02
22190542	2011	6	17	3070	148.05	18.96
22190542	2011	6	17	3080	146.83	18.92
22190542	2011	6	17	3090	146.29	18.86
22190542	2011	6	17	3100	147.85	18.81
22190542	2011	6	17	3110	149.92	18.75
22190542	2011	6	17	3120	151.92	18.67
22190542	2011	6	17	3130	153.88	18.61
22190542	2011	6	17	3140	155.41	18.57
22190542	2011	6	17	3150	156.5	18.53
22190542	2011	6	17	3160	157.13	18.5
22190542	2011	6	17	3170	156.81	18.49
22190542	2011	6	17	3180	156.95	18.46
22190542	2011	6	17	3190	157.82	18.4
22190542	2011	6	17	3200	159.64	18.34
22190542	2011	6	17	3210	161.91	18.26
22190542	2011	6	17	3220	163.71	18.22
22190542	2011	6	17	3230	164.37	18.24
22190542	2011	6	17	3240	164.24	18.21
22190542	2011	6	17	3250	163.74	18.21
22190542	2011	6	17	3260	162.96	18.2
22190542	2011	6	17	3270	162.72	18.17
22190542	2011	6	17	3280	164.36	18.11
22190542	2011	6	17	3290	166.08	18.07
22190542	2011	6	17	3300	168.08	18
22190542	2011	6	17	3310	169.08	17.96
22190542	2011	6	17	3320	169.4	17.92
22190542	2011	6	17	3330	170.4	17.85
22190542	2011	6	17	3340	171.46	17.78
22190542	2011	6	17	3350	173.1	17.74
22190542	2011	6	17	3360	173.71	17.7
22190542	2011	6	17	3370	173.33	17.66
22190542	2011	6	17	3380	173.28	17.64
22190542	2011	6	17	3390	173.1	17.61
22190542	2011	6	17	3400	173.18	17.58
22190542	2011	6	17	3410	173.22	17.54
22190542	2011	6	17	3420	173.26	17.52
22190542	2011	6	17	3430	173.2	17.49
22190542	2011	6	17	3440	173.18	17.46
22190542	2011	6	17	3450	173.18	17.42
22190542	2011	6	17	3460	173.13	17.39
22190542	2011	6	17	3470	173.22	17.36
22190542	2011	6	17	3480	173.06	17.34
22190542	2011	6	17	3490	173.13	17.32
22190542	2011	6	17	3500	173.25	17.28
22190542	2011	6	17	3510	173.37	17.26
22190542	2011	6	17	3520	173.25	17.25

22190542	2011	6	17	3530	173.13	17.22
22190542	2011	6	17	3540	171.21	17.21
22190542	2011	6	17	3550	169.21	17.19
22190542	2011	6	17	3560	167.35	17.17
22190542	2011	6	17	3570	165.34	17.16
22190542	2011	6	17	3580	163.39	17.16
22190542	2011	6	17	3590	161.47	17.17
22190542	2011	6	17	3600	159.41	17.17
22190542	2011	6	17	3610	157.32	17.2
22190542	2011	6	17	3620	156.12	17.25
22190542	2011	6	17	3630	155.83	17.28
22190542	2011	6	17	3640	155.92	17.31
22190542	2011	6	17	3650	156.23	17.34
22190542	2011	6	17	3660	156.22	17.36
22190542	2011	6	17	3670	154.94	17.39
22190542	2011	6	17	3680	153.82	17.41
22190542	2011	6	17	3690	152.75	17.45
22190542	2011	6	17	3700	151.03	17.47
22190542	2011	6	17	3710	149.05	17.51
22190542	2011	6	17	3720	147.19	17.53
22190542	2011	6	17	3730	145.33	17.57
22190542	2011	6	17	3740	143.44	17.62
22190542	2011	6	17	3750	141.57	17.66
22190542	2011	6	17	3760	139.15	17.72
22190542	2011	6	17	3770	136.14	17.82
22190542	2011	6	17	3780	133.62	17.86
22190542	2011	6	17	3790	131.56	17.92
22190542	2011	6	17	3800	129.77	17.96
22190542	2011	6	17	3810	128.22	18.01
22190542	2011	6	17	3820	126.8	18.05
22190542	2011	6	17	3830	125.45	18.08
22190542	2011	6	17	3840	123.23	18.14
22190542	2011	6	17	3850	120	18.2
22190542	2011	6	17	3860	116.88	18.25
22190542	2011	6	17	3870	114.18	18.29
22190542	2011	6	17	3880	111.97	18.35
22190542	2011	6	17	3890	109.92	18.44
22190542	2011	6	17	3900	108.71	18.51
22190542	2011	6	17	3910	107.62	18.57
22190542	2011	6	17	3920	106.43	18.65
22190542	2011	6	17	3930	105.16	18.71
22190542	2011	6	17	3940	102.98	18.8
22190542	2011	6	17	3950	99.98	18.91
22190542	2011	6	17	3960	96.85	19.02
22190542	2011	6	17	3970	93.94	19.15
22190542	2011	6	17	3980	91.56	19.32
22190542	2011	6	17	3990	88.76	19.5

22190542	2011	6	17	4000	85.78	19.73
22190542	2011	6	17	4010	83.01	19.93
22190542	2011	6	17	4020	80.21	20.08
22190542	2011	6	17	4030	77.25	20.24
22190542	2011	6	17	4040	75.11	20.45
22190542	2011	6	17	4050	73.19	20.6
22190542	2011	6	17	4060	71.31	20.76
22190542	2011	6	17	4070	69.67	21.03
22190542	2011	6	17	4080	69.15	21.3
22190542	2011	6	17	4090	68.5	21.5
22190542	2011	6	17	4100	66.7	21.6
22190542	2011	6	17	4110	64.85	21.74
22190542	2011	6	17	4120	62.68	21.92
22190542	2011	6	17	4130	61.03	22.08
22190542	2011	6	17	4140	60.75	22.36
22190542	2011	6	17	4150	59.7	22.56
22190542	2011	6	17	4160	57.87	22.68
22190542	2011	6	17	4170	55.85	22.82
22190542	2011	6	17	4180	53.82	22.96
22190542	2011	6	17	4190	53.07	23.1
22190542	2011	6	17	4200	51.51	23.27
22190542	2011	6	17	4210	49.64	23.43
22190542	2011	6	17	4220	47.65	23.59
22190542	2011	6	17	4230	45.94	23.76
22190542	2011	6	17	4240	44.51	23.94
22190542	2011	6	17	4250	43.15	24.24
22190542	2011	6	17	4260	41.67	24.5
22190542	2011	6	17	4270	40.2	24.67
22190542	2011	6	17	4280	39.49	24.89
22190542	2011	6	17	4290	39.22	25.09
22190542	2011	6	17	4300	38.42	25.2
22190542	2011	6	17	4310	37.88	25.32
22190542	2011	6	17	4320	38.06	25.43
22190542	2011	6	17	4330	38.38	25.65
22190542	2011	6	17	4340	38.76	25.84
22190542	2011	6	17	4350	38.52	25.96
22190542	2011	6	17	4360	37.07	26.1
22190542	2011	6	17	4370	36.72	26.19
22190542	2011	6	17	4380	36.91	26.27
22190542	2011	6	17	4390	36.78	26.42
22190542	2011	6	17	4400	36.27	26.52
22190542	2011	6	17	4410	35.61	26.6
22190542	2011	6	17	4420	35.24	26.67
22190542	2011	6	17	4430	34.69	26.77
22190542	2011	6	17	4440	34.31	26.82
22190542	2011	6	17	4450	34.5	26.96
22190542	2011	6	17	4460	34.46	26.99

22190542	2011	6	17	4470	33.93	27.12
22190542	2011	6	17	4480	33.27	27.11
22190542	2011	6	17	4490	32.96	27.16
22190542	2011	6	17	4500	32.8	27.22
22190542	2011	6	17	4510	32.97	27.28
22190542	2011	6	17	4520	33.22	27.36
22190542	2011	6	17	4530	32.19	27.43
22190542	2011	6	17	4540	30.56	27.48
22190542	2011	6	17	4550	30.61	27.51
22190542	2011	6	17	4560	30.8	27.57
22190542	2011	6	17	4570	31.24	27.61
22190542	2011	6	17	4580	31.37	27.65
22190542	2011	6	17	4590	31.16	27.67
22190542	2011	6	17	4600	31.02	27.71
22190542	2011	6	17	4610	30.22	27.74
22190542	2011	6	17	4620	30.53	27.78
22190542	2011	6	17	4630	30.11	27.79
22190542	2011	6	17	4640	30.46	27.82
22190542	2011	6	17	4650	30.45	27.83
22190542	2011	6	17	4660	30.34	27.86
22190542	2011	6	17	4670	30.35	27.9
22190542	2011	6	17	4680	28.87	27.92
22190542	2011	6	17	4690	27.12	27.94
22190542	2011	6	17	4700	25.68	27.98
22190542	2011	6	17	4710	24.38	28
22190542	2011	6	17	4720	22.82	28.03
22190542	2011	6	17	4730	21.88	28.07
22190542	2011	6	17	4740	20.88	28.1
22190542	2011	6	17	4750	19.72	28.12
22190542	2011	6	17	4760	19.74	28.14
22190542	2011	6	17	4770	19.68	28.16
22190542	2011	6	17	4780	19.68	28.17
22190542	2011	6	17	4790	19.77	28.2
22190542	2011	6	17	4800	19.65	28.21
22190542	2011	6	17	4810	19.73	28.22
22190542	2011	6	17	4820	19.68	28.25
22190542	2011	6	17	4830	19.59	28.25
22190542	2011	6	17	4840	19.68	28.27
22190542	2011	6	17	4850	19.69	28.28
22190542	2011	6	17	4860	19.69	28.29
22190542	2011	6	17	4870	19.68	28.3
22190542	2011	6	17	4880	19.78	28.32
22190542	2011	6	17	4890	19.7	28.32
22190542	2011	6	17	4900	19.7	28.33
22190542	2011	6	17	4910	19.72	28.34
22190542	2011	6	17	4920	19.73	28.35
22190542	2011	6	17	4930	19.78	28.35

22190542	2011	6	17	4940	19.72	28.36
22190542	2011	6	17	4950	19.82	28.36
22190542	2011	6	17	4960	19.79	28.36
22190542	2011	6	17	4970	19.69	28.37
22190542	2011	6	17	4980	19.73	28.38
22190542	2011	6	17	4990	19.76	28.39
22190542	2011	6	17	5000	19.87	28.39
22190542	2011	6	17	5010	19.74	28.39
22190542	2011	6	17	5020	19.7	28.4
22190542	2011	6	17	5030	19.85	28.4
22190542	2011	6	17	5040	19.98	28.4
22190542	2011	6	17	5050	19.7	28.41
22190542	2011	6	17	5060	19.82	28.42
22190542	2011	6	17	5070	19.91	28.41
22190542	2011	6	17	5080	20.07	28.4
22190542	2011	6	17	5090	20.17	28.41
22190542	2011	6	17	5100	20.42	28.41
22190542	2011	6	17	5110	20.44	28.41
22190542	2011	6	17	5120	20.45	28.41
22190542	2011	6	17	5130	20.61	28.42
22190542	2011	6	17	5140	20.4	28.42
22190542	2011	6	17	5150	20.44	28.42
22190542	2011	6	17	5160	20.41	28.42
22190542	2011	6	17	5170	20.51	28.43
22190542	2011	6	17	5180	20.43	28.42
22190542	2011	6	17	5190	20.4	28.42
22190542	2011	6	17	5200	20.39	28.42
22190542	2011	6	17	5210	20.41	28.43
22190542	2011	6	17	5220	20.41	28.42
22190542	2011	6	17	5230	20.5	28.43
22190542	2011	6	17	5240	19.17	28.45
22190542	2011	6	17	5250	17.66	28.46
22190542	2011	6	17	5260	16.31	28.47
22190542	2011	6	17	5270	14.51	28.48
22190542	2011	6	17	5280	12.14	28.5
22190542	2011	6	17	5290	9.91	28.53
22190542	2011	6	17	5300	7.64	28.55
22190542	2011	6	17	5310	5.32	28.57
22190542	2011	6	17	5320	3.53	28.6
22190542	2011	6	17	5330	2.65	28.61
22190542	2011	6	17	5340	2.63	28.64
22190542	2011	6	17	5350	2.5	28.65
22190542	2011	6	17	5360	2.49	28.65
22190542	2011	6	17	5370	2.65	28.66
22190542	2011	6	17	5380	2.56	28.67
22190542	2011	6	17	5390	2.17	28.67
22190542	2011	6	17	5400	0.71	28.71

22190542	2011	6	17	5410	0.7	28.72
22190542	2011	6	17	5420	0.68	28.73
22190542	2011	6	17	5430	0.7	28.75
22190542	2011	6	17	5440	0.7	28.75
22190542	2011	6	17	5450	0.68	28.75
22190542	2011	6	17	5460	0.68	28.78
22190542	2011	6	17	5470	0.68	28.79
22190542	2011	6	17	5480	0.69	28.81
22190542	2011	6	17	5490	0.71	28.82
22190542	2011	6	17	5500	0.75	28.83
22190542	2011	6	17	5510	0.69	28.85
22190542	2011	6	17	5520	0.62	28.86
22190542	2011	6	17	5530	0.58	28.88
22190542	2011	6	17	5540	0.55	28.89
22198142	2011	6	17	0	0.9	29.05
22198142	2011	6	17	10	1.34	29.06
22198142	2011	6	17	20	1.83	29.05
22198142	2011	6	17	30	1.84	29.04
22198142	2011	6	17	40	1.98	29.06
22198142	2011	6	17	50	1.93	29.05
22198142	2011	6	17	60	2.43	29.03
22198142	2011	6	17	70	3.1	29.02
22198142	2011	6	17	80	3.48	29.01
22198142	2011	6	17	90	4	29
22198142	2011	6	17	100	4.61	28.96
22198142	2011	6	17	110	5.84	28.94
22198142	2011	6	17	120	7.7	28.92
22198142	2011	6	17	130	8.99	28.89
22198142	2011	6	17	140	9.29	28.89
22198142	2011	6	17	150	9.18	28.89
22198142	2011	6	17	160	8.97	28.88
22198142	2011	6	17	170	8.76	28.87
22198142	2011	6	17	180	8.53	28.86
22198142	2011	6	17	190	8.45	28.85
22198142	2011	6	17	200	8.79	28.84
22198142	2011	6	17	210	9.11	28.83
22198142	2011	6	17	220	8.58	28.82
22198142	2011	6	17	230	8.51	28.82
22198142	2011	6	17	240	8.42	28.81
22198142	2011	6	17	250	8.3	28.81
22198142	2011	6	17	260	8.17	28.8
22198142	2011	6	17	270	7.94	28.79
22198142	2011	6	17	280	7.67	28.79
22198142	2011	6	17	290	7.34	28.79
22198142	2011	6	17	300	7.55	28.78
22198142	2011	6	17	310	7.64	28.78
22198142	2011	6	17	320	8.54	28.78

22198142	2011	6	17	330	8.72	28.78
22198142	2011	6	17	340	8.62	28.78
22198142	2011	6	17	350	8.75	28.78
22198142	2011	6	17	360	8.72	28.79
22198142	2011	6	17	370	8.58	28.78
22198142	2011	6	17	380	8.37	28.79
22198142	2011	6	17	390	8.18	28.8
22198142	2011	6	17	400	7.93	28.8
22198142	2011	6	17	410	7.59	28.8
22198142	2011	6	17	420	7.71	28.81
22198142	2011	6	17	430	7.77	28.81
22198142	2011	6	17	440	7.84	28.81
22198142	2011	6	17	450	7.74	28.81
22198142	2011	6	17	460	7.46	28.81
22198142	2011	6	17	470	7.16	28.82
22198142	2011	6	17	480	7.28	28.82
22198142	2011	6	17	490	7.19	28.82
22198142	2011	6	17	500	7.12	28.82
22198142	2011	6	17	510	7.27	28.82
22198142	2011	6	17	520	7.1	28.82
22198142	2011	6	17	530	6.74	28.82
22198142	2011	6	17	540	6.47	28.82
22198142	2011	6	17	550	6.12	28.83
22198142	2011	6	17	560	6.01	28.84
22198142	2011	6	17	570	5.9	28.84
22198142	2011	6	17	580	7.17	28.83
22198142	2011	6	17	590	8.82	28.82
22198142	2011	6	17	600	10.84	28.79
22198142	2011	6	17	610	12.95	28.77
22198142	2011	6	17	620	15.36	28.75
22198142	2011	6	17	630	17.52	28.71
22198142	2011	6	17	640	18.17	28.71
22198142	2011	6	17	650	18.32	28.71
22198142	2011	6	17	660	18.42	28.71
22198142	2011	6	17	670	18.53	28.7
22198142	2011	6	17	680	18.86	28.69
22198142	2011	6	17	690	18.85	28.69
22198142	2011	6	17	700	18.78	28.69
22198142	2011	6	17	710	18.93	28.68
22198142	2011	6	17	720	18.82	28.68
22198142	2011	6	17	730	18.67	28.67
22198142	2011	6	17	740	18.9	28.67
22198142	2011	6	17	750	18.89	28.67
22198142	2011	6	17	760	18.98	28.67
22198142	2011	6	17	770	18.95	28.66
22198142	2011	6	17	780	18.93	28.67
22198142	2011	6	17	790	18.95	28.66

22198142	2011	6	17	800	19.01	28.67
22198142	2011	6	17	810	18.94	28.66
22198142	2011	6	17	820	18.83	28.66
22198142	2011	6	17	830	18.79	28.66
22198142	2011	6	17	840	18.9	28.65
22198142	2011	6	17	850	18.9	28.66
22198142	2011	6	17	860	18.89	28.65
22198142	2011	6	17	870	19.04	28.65
22198142	2011	6	17	880	19.08	28.64
22198142	2011	6	17	890	19.01	28.64
22198142	2011	6	17	900	18.98	28.64
22198142	2011	6	17	910	18.85	28.65
22198142	2011	6	17	920	18.77	28.64
22198142	2011	6	17	930	18.64	28.64
22198142	2011	6	17	940	18.59	28.64
22198142	2011	6	17	950	18.63	28.64
22198142	2011	6	17	960	18.52	28.64
22198142	2011	6	17	970	18.29	28.64
22198142	2011	6	17	980	17.93	28.64
22198142	2011	6	17	990	17.45	28.65
22198142	2011	6	17	1000	16.95	28.65
22198142	2011	6	17	1010	16.4	28.65
22198142	2011	6	17	1020	15.91	28.66
22198142	2011	6	17	1030	15.7	28.66
22198142	2011	6	17	1040	16.33	28.65
22198142	2011	6	17	1050	17.17	28.64
22198142	2011	6	17	1060	18.25	28.63
22198142	2011	6	17	1070	20.88	28.6
22198142	2011	6	17	1080	23.42	28.57
22198142	2011	6	17	1090	25.58	28.55
22198142	2011	6	17	1100	27.53	28.52
22198142	2011	6	17	1110	29.52	28.5
22198142	2011	6	17	1120	31.57	28.46
22198142	2011	6	17	1130	33.61	28.44
22198142	2011	6	17	1140	35.51	28.41
22198142	2011	6	17	1150	37.58	28.39
22198142	2011	6	17	1160	40.42	28.36
22198142	2011	6	17	1170	42.66	28.33
22198142	2011	6	17	1180	44.28	28.28
22198142	2011	6	17	1190	44.9	28.27
22198142	2011	6	17	1200	46.19	28.08
22198142	2011	6	17	1210	46.53	28.04
22198142	2011	6	17	1220	46.45	28.02
22198142	2011	6	17	1230	46.19	27.99
22198142	2011	6	17	1240	45.87	27.96
22198142	2011	6	17	1250	46.16	27.89
22198142	2011	6	17	1260	46.13	27.82

22198142	2011	6	17	1270	46.1	27.75
22198142	2011	6	17	1280	46.2	27.67
22198142	2011	6	17	1290	46.18	27.62
22198142	2011	6	17	1300	46.18	27.57
22198142	2011	6	17	1310	46.28	27.49
22198142	2011	6	17	1320	47.01	27.41
22198142	2011	6	17	1330	46.82	27.34
22198142	2011	6	17	1340	46.43	27.3
22198142	2011	6	17	1350	46.57	27.25
22198142	2011	6	17	1360	46.84	27.16
22198142	2011	6	17	1370	46.92	27.12
22198142	2011	6	17	1380	47.07	27.05
22198142	2011	6	17	1390	47.16	27.01
22198142	2011	6	17	1400	46.94	26.98
22198142	2011	6	17	1410	46.41	26.94
22198142	2011	6	17	1420	45.71	26.92
22198142	2011	6	17	1430	45.06	26.9
22198142	2011	6	17	1440	44.72	26.88
22198142	2011	6	17	1450	45.47	26.84
22198142	2011	6	17	1460	46.86	26.79
22198142	2011	6	17	1470	48.86	26.75
22198142	2011	6	17	1480	50.67	26.67
22198142	2011	6	17	1490	50.92	26.67
22198142	2011	6	17	1500	51.26	26.61
22198142	2011	6	17	1510	51.93	26.58
22198142	2011	6	17	1520	51.96	26.57
22198142	2011	6	17	1530	51.96	26.54
22198142	2011	6	17	1540	51.89	26.52
22198142	2011	6	17	1550	51.88	26.49
22198142	2011	6	17	1560	51.85	26.46
22198142	2011	6	17	1570	51.78	26.43
22198142	2011	6	17	1580	51.72	26.41
22198142	2011	6	17	1590	51.95	26.39
22198142	2011	6	17	1600	51.84	26.36
22198142	2011	6	17	1610	51.96	26.34
22198142	2011	6	17	1620	51.92	26.32
22198142	2011	6	17	1630	52.04	26.3
22198142	2011	6	17	1640	51.99	26.27
22198142	2011	6	17	1650	51.93	26.24
22198142	2011	6	17	1660	51.76	26.22
22198142	2011	6	17	1670	51.55	26.2
22198142	2011	6	17	1680	51.5	26.18
22198142	2011	6	17	1690	51.46	26.17
22198142	2011	6	17	1700	52.74	26.14
22198142	2011	6	17	1710	54.71	26.1
22198142	2011	6	17	1720	55.23	26.1
22198142	2011	6	17	1730	55.14	26.09

22198142	2011	6	17	1740	54.85	26.07
22198142	2011	6	17	1750	54.23	26.07
22198142	2011	6	17	1760	55.3	26.03
22198142	2011	6	17	1770	57.61	25.99
22198142	2011	6	17	1780	60.01	25.93
22198142	2011	6	17	1790	62.3	25.88
22198142	2011	6	17	1800	64.62	25.82
22198142	2011	6	17	1810	67.29	25.76
22198142	2011	6	17	1820	70.09	25.69
22198142	2011	6	17	1830	72.63	25.6
22198142	2011	6	17	1840	74.62	25.52
22198142	2011	6	17	1850	76.38	25.37
22198142	2011	6	17	1860	78.45	25.3
22198142	2011	6	17	1870	81.08	25.17
22198142	2011	6	17	1880	83.82	25.11
22198142	2011	6	17	1890	85.9	25.04
22198142	2011	6	17	1900	86.86	24.99
22198142	2011	6	17	1910	86.5	24.94
22198142	2011	6	17	1920	85.99	24.89
22198142	2011	6	17	1930	85.75	24.83
22198142	2011	6	17	1940	85.55	24.77
22198142	2011	6	17	1950	85.46	24.73
22198142	2011	6	17	1960	85.61	24.67
22198142	2011	6	17	1970	85.45	24.64
22198142	2011	6	17	1980	85.53	24.59
22198142	2011	6	17	1990	85.42	24.56
22198142	2011	6	17	2000	85.3	24.51
22198142	2011	6	17	2010	85.16	24.48
22198142	2011	6	17	2020	85.38	24.44
22198142	2011	6	17	2030	85.24	24.37
22198142	2011	6	17	2040	85.84	24.31
22198142	2011	6	17	2050	86.27	24.28
22198142	2011	6	17	2060	87.67	24.21
22198142	2011	6	17	2070	89.66	24.13
22198142	2011	6	17	2080	91.18	24.07
22198142	2011	6	17	2090	91.51	24.03
22198142	2011	6	17	2100	92.19	23.96
22198142	2011	6	17	2110	93.87	23.78
22198142	2011	6	17	2120	95.58	23.69
22198142	2011	6	17	2130	97.34	23.64
22198142	2011	6	17	2140	99.45	23.6
22198142	2011	6	17	2150	101.8	23.57
22198142	2011	6	17	2160	102.78	23.51
22198142	2011	6	17	2170	104.43	23.43
22198142	2011	6	17	2180	106.22	23.28
22198142	2011	6	17	2190	108.1	23.19
22198142	2011	6	17	2200	110.44	23

22198142	2011	6	17	2210	113.16	22.88
22198142	2011	6	17	2220	115.97	22.7
22198142	2011	6	17	2230	118.99	22.6
22198142	2011	6	17	2240	121.78	22.47
22198142	2011	6	17	2250	123.78	22.25
22198142	2011	6	17	2260	125.75	22.19
22198142	2011	6	17	2270	127.61	22.01
22198142	2011	6	17	2280	130.07	21.93
22198142	2011	6	17	2290	132.8	21.79
22198142	2011	6	17	2300	135.66	21.67
22198142	2011	6	17	2310	138.6	21.49
22198142	2011	6	17	2320	141.52	21.3
22198142	2011	6	17	2330	144.34	21.17
22198142	2011	6	17	2340	147.14	20.94
22198142	2011	6	17	2350	149.59	20.75
22198142	2011	6	17	2360	150.84	20.72
22198142	2011	6	17	2370	151.26	20.67
22198142	2011	6	17	2380	151.06	20.56
22198142	2011	6	17	2390	150.4	20.48
22198142	2011	6	17	2400	149.41	20.33
22198142	2011	6	17	2410	149	20.21
22198142	2011	6	17	2420	149.63	20.08
22198142	2011	6	17	2430	149.73	19.97
22198142	2011	6	17	2440	149.31	19.89
22198142	2011	6	17	2450	148.75	19.79
22198142	2011	6	17	2460	148.22	19.65
22198142	2011	6	17	2470	148	19.57
22198142	2011	6	17	2480	147.89	19.53
22198142	2011	6	17	2490	147.55	19.45
22198142	2011	6	17	2500	147.38	19.37
22198142	2011	6	17	2510	147.91	19.3
22198142	2011	6	17	2520	148.43	19.24
22198142	2011	6	17	2530	148.98	19.11
22198142	2011	6	17	2540	149.55	19.07
22198142	2011	6	17	2550	149.7	19.03
22198142	2011	6	17	2560	150.4	18.96
22198142	2011	6	17	2570	150.55	18.92
22198142	2011	6	17	2580	150.36	18.89
22198142	2011	6	17	2590	149.55	18.84
22198142	2011	6	17	2600	149.07	18.78
22198142	2011	6	17	2610	149	18.71
22198142	2011	6	17	2620	148.68	18.68
22198142	2011	6	17	2630	148.03	18.64
22198142	2011	6	17	2640	147.37	18.6
22198142	2011	6	17	2650	146.9	18.56
22198142	2011	6	17	2660	146.92	18.55
22198142	2011	6	17	2670	146.39	18.53

22198142	2011	6	17	2680	145.91	18.54
22198142	2011	6	17	2690	145.46	18.55
22198142	2011	6	17	2700	145.28	18.6
22198142	2011	6	17	2710	145.01	18.63
22198142	2011	6	17	2720	145.96	18.68
22198142	2011	6	17	2730	146.17	18.68
22198142	2011	6	17	2740	145.87	18.7
22198142	2011	6	17	2750	145.68	18.72
22198142	2011	6	17	2760	145.47	18.73
22198142	2011	6	17	2770	145.2	18.74
22198142	2011	6	17	2780	145.21	18.75
22198142	2011	6	17	2790	145.65	18.76
22198142	2011	6	17	2800	146.05	18.76
22198142	2011	6	17	2810	145.86	18.77
22198142	2011	6	17	2820	145.02	18.75
22198142	2011	6	17	2830	144.59	18.75
22198142	2011	6	17	2840	144.29	18.75
22198142	2011	6	17	2850	143.58	18.75
22198142	2011	6	17	2860	142.84	18.75
22198142	2011	6	17	2870	142.06	18.75
22198142	2011	6	17	2880	140.93	18.75
22198142	2011	6	17	2890	139.95	18.77
22198142	2011	6	17	2900	139.09	18.78
22198142	2011	6	17	2910	138.26	18.78
22198142	2011	6	17	2920	137.48	18.8
22198142	2011	6	17	2930	136.74	18.81
22198142	2011	6	17	2940	135.99	18.82
22198142	2011	6	17	2950	135.43	18.84
22198142	2011	6	17	2960	135.07	18.85
22198142	2011	6	17	2970	134.61	18.86
22198142	2011	6	17	2980	134.45	18.88
22198142	2011	6	17	2990	133.41	18.89
22198142	2011	6	17	3000	132.36	18.89
22198142	2011	6	17	3010	131.62	18.89
22198142	2011	6	17	3020	131.05	18.91
22198142	2011	6	17	3030	130.61	18.92
22198142	2011	6	17	3040	130.12	18.92
22198142	2011	6	17	3050	129.65	18.93
22198142	2011	6	17	3060	129.49	18.94
22198142	2011	6	17	3070	128.97	18.94
22198142	2011	6	17	3080	128.32	18.96
22198142	2011	6	17	3090	127.67	18.96
22198142	2011	6	17	3100	127.22	19
22198142	2011	6	17	3110	126.59	19.02
22198142	2011	6	17	3120	125.83	19.03
22198142	2011	6	17	3130	125.05	19.04
22198142	2011	6	17	3140	124.38	19.07

22198142	2011	6	17	3150	123.96	19.1
22198142	2011	6	17	3160	123.61	19.12
22198142	2011	6	17	3170	123.19	19.14
22198142	2011	6	17	3180	122.83	19.17
22198142	2011	6	17	3190	122.6	19.2
22198142	2011	6	17	3200	122.27	19.21
22198142	2011	6	17	3210	121.95	19.22
22198142	2011	6	17	3220	121.75	19.24
22198142	2011	6	17	3230	121.34	19.25
22198142	2011	6	17	3240	120.77	19.26
22198142	2011	6	17	3250	120.06	19.27
22198142	2011	6	17	3260	118.76	19.29
22198142	2011	6	17	3270	116.2	19.32
22198142	2011	6	17	3280	113.51	19.35
22198142	2011	6	17	3290	111.03	19.39
22198142	2011	6	17	3300	109.58	19.46
22198142	2011	6	17	3310	107.9	19.58
22198142	2011	6	17	3320	105.57	19.67
22198142	2011	6	17	3330	103.16	19.81
22198142	2011	6	17	3340	101.03	19.92
22198142	2011	6	17	3350	98.83	20.13
22198142	2011	6	17	3360	96.45	20.25
22198142	2011	6	17	3370	95.06	20.31
22198142	2011	6	17	3380	94.37	20.53
22198142	2011	6	17	3390	93.58	20.71
22198142	2011	6	17	3400	92.07	20.78
22198142	2011	6	17	3410	91.22	20.88
22198142	2011	6	17	3420	90.85	20.98
22198142	2011	6	17	3430	90.64	21.19
22198142	2011	6	17	3440	90.24	21.33
22198142	2011	6	17	3450	89.78	21.45
22198142	2011	6	17	3460	89.25	21.6
22198142	2011	6	17	3470	87.56	21.69
22198142	2011	6	17	3480	85.9	21.78
22198142	2011	6	17	3490	84.74	21.82
22198142	2011	6	17	3500	83.63	21.87
22198142	2011	6	17	3510	82.18	21.96
22198142	2011	6	17	3520	80.31	22.09
22198142	2011	6	17	3530	78.84	22.18
22198142	2011	6	17	3540	77.36	22.27
22198142	2011	6	17	3550	76.12	22.38
22198142	2011	6	17	3560	76.03	22.57
22198142	2011	6	17	3570	75.8	22.59
22198142	2011	6	17	3580	75.57	22.64
22198142	2011	6	17	3590	75.02	22.75
22198142	2011	6	17	3600	74.39	22.83
22198142	2011	6	17	3610	73.4	22.89

22198142	2011	6	17	3620	72.6	22.96
22198142	2011	6	17	3630	71.03	23.04
22198142	2011	6	17	3640	69.42	23.12
22198142	2011	6	17	3650	67.24	23.23
22198142	2011	6	17	3660	64.54	23.32
22198142	2011	6	17	3670	61.87	23.39
22198142	2011	6	17	3680	59.19	23.5
22198142	2011	6	17	3690	56.46	23.6
22198142	2011	6	17	3700	53.79	23.71
22198142	2011	6	17	3710	51.22	23.87
22198142	2011	6	17	3720	48.65	23.98
22198142	2011	6	17	3730	46.1	24.07
22198142	2011	6	17	3740	43.82	24.2
22198142	2011	6	17	3750	42.68	24.31
22198142	2011	6	17	3760	42.02	24.54
22198142	2011	6	17	3770	41.06	24.86
22198142	2011	6	17	3780	40.26	25.02
22198142	2011	6	17	3790	39.55	25.22
22198142	2011	6	17	3800	38.67	25.39
22198142	2011	6	17	3810	38.16	25.5
22198142	2011	6	17	3820	37.99	25.63
22198142	2011	6	17	3830	38.04	25.75
22198142	2011	6	17	3840	37.87	25.88
22198142	2011	6	17	3850	37.36	26.03
22198142	2011	6	17	3860	35.99	26.12
22198142	2011	6	17	3870	34.49	26.23
22198142	2011	6	17	3880	33.24	26.33
22198142	2011	6	17	3890	32.69	26.46
22198142	2011	6	17	3900	31.69	26.55
22198142	2011	6	17	3910	31.54	26.67
22198142	2011	6	17	3920	31.24	26.78
22198142	2011	6	17	3930	30.8	26.86
22198142	2011	6	17	3940	30.49	26.93
22198142	2011	6	17	3950	30.41	26.96
22198142	2011	6	17	3960	30.11	27.03
22198142	2011	6	17	3970	29.89	27.08
22198142	2011	6	17	3980	29.83	27.15
22198142	2011	6	17	3990	29.64	27.2
22198142	2011	6	17	4000	29.26	27.25
22198142	2011	6	17	4010	29.04	27.31
22198142	2011	6	17	4020	29.04	27.42
22198142	2011	6	17	4030	28.89	27.53
22198142	2011	6	17	4040	28.82	27.56
22198142	2011	6	17	4050	28.85	27.64
22198142	2011	6	17	4060	28.7	27.71
22198142	2011	6	17	4070	28.49	27.73
22198142	2011	6	17	4080	28.48	27.76

22198142	2011	6	17	4090	28.52	27.81
22198142	2011	6	17	4100	28.36	27.83
22198142	2011	6	17	4110	28.34	27.84
22198142	2011	6	17	4120	28.37	27.87
22198142	2011	6	17	4130	28.24	27.89
22198142	2011	6	17	4140	28.03	27.92
22198142	2011	6	17	4150	28.16	27.95
22198142	2011	6	17	4160	28.26	27.99
22198142	2011	6	17	4170	28.21	27.99
22198142	2011	6	17	4180	27.99	28.03
22198142	2011	6	17	4190	27.67	28.07
22198142	2011	6	17	4200	27.39	28.06
22198142	2011	6	17	4210	27.07	28.07
22198142	2011	6	17	4220	26.92	28.1
22198142	2011	6	17	4230	27.17	28.14
22198142	2011	6	17	4240	27.32	28.14
22198142	2011	6	17	4250	26.95	28.15
22198142	2011	6	17	4260	26.06	28.17
22198142	2011	6	17	4270	25.8	28.18
22198142	2011	6	17	4280	25.64	28.18
22198142	2011	6	17	4290	25.18	28.2
22198142	2011	6	17	4300	23.89	28.21
22198142	2011	6	17	4310	23.46	28.23
22198142	2011	6	17	4320	22.71	28.24
22198142	2011	6	17	4330	21.11	28.26
22198142	2011	6	17	4340	19.84	28.29
22198142	2011	6	17	4350	18.05	28.32
22198142	2011	6	17	4360	15.86	28.34
22198142	2011	6	17	4370	13.44	28.38
22198142	2011	6	17	4380	11.29	28.4
22198142	2011	6	17	4390	8.68	28.43
22198142	2011	6	17	4400	6.11	28.46
22198142	2011	6	17	4410	4.21	28.5
22198142	2011	6	17	4420	3.09	28.53
22198142	2011	6	17	4430	2.86	28.53
22198142	2011	6	17	4440	2.24	28.56
22198142	2011	6	17	4450	2.29	28.57
22198142	2011	6	17	4460	2.2	28.58
22198142	2011	6	17	4470	1.95	28.6
22198142	2011	6	17	4480	0.78	28.63
22198142	2011	6	17	4490	0.65	28.64
22198142	2011	6	17	4500	0.64	28.64
22198142	2011	6	17	4510	0.67	28.66
22198142	2011	6	17	4520	0.62	28.67
22198142	2011	6	17	4530	0.61	28.7
22198142	2011	6	17	4540	0.6	28.73
22198142	2011	6	17	4550	0.67	28.75

22198142	2011	6	17	4560	0.61	28.78
22198142	2011	6	17	4570	0.65	28.8
22198142	2011	6	17	4580	0.65	28.82
22198142	2011	6	17	4590	0.61	28.82
22198142	2011	6	17	4600	0.67	28.84
22198142	2011	6	17	4610	0.64	28.85
22198142	2011	6	17	4620	0.65	28.85
23395865	2011	7	1	0	0.9	28.07
23395865	2011	7	1	10	0.88	28.07
23395865	2011	7	1	20	0.9	28.07
23395865	2011	7	1	30	0.89	28.07
23395865	2011	7	1	40	0.89	28.07
23395865	2011	7	1	50	0.91	28.07
23395865	2011	7	1	60	0.89	28.08
23395865	2011	7	1	70	1.09	28.08
23395865	2011	7	1	80	1.67	28.07
23395865	2011	7	1	90	2.1	28.08
23395865	2011	7	1	100	2.16	28.07
23395865	2011	7	1	110	2.15	28.09
23395865	2011	7	1	120	1.99	28.09
23395865	2011	7	1	130	2.3	28.1
23395865	2011	7	1	140	2.64	28.09
23395865	2011	7	1	150	2.83	28.09
23395865	2011	7	1	160	2.79	28.09
23395865	2011	7	1	170	2.85	28.09
23395865	2011	7	1	180	2.77	28.1
23395865	2011	7	1	190	3.2	28.1
23395865	2011	7	1	200	4.88	28.09
23395865	2011	7	1	210	7.06	28.13
23395865	2011	7	1	220	9.36	28.11
23395865	2011	7	1	230	11.5	28.1
23395865	2011	7	1	240	13.3	28.08
23395865	2011	7	1	250	14.87	28.07
23395865	2011	7	1	260	16.28	28.05
23395865	2011	7	1	270	16.6	28.04
23395865	2011	7	1	280	16.96	28.05
23395865	2011	7	1	290	18.58	28.03
23395865	2011	7	1	300	19.32	28.02
23395865	2011	7	1	310	19.76	28.02
23395865	2011	7	1	320	19.96	28.02
23395865	2011	7	1	330	20.68	28.01
23395865	2011	7	1	340	22.75	28
23395865	2011	7	1	350	24.95	27.98
23395865	2011	7	1	360	26.41	27.88
23395865	2011	7	1	370	27.46	27.82
23395865	2011	7	1	380	29.04	27.7
23395865	2011	7	1	390	30.84	27.52

23395865	2011	7	1	400	33.16	27.37
23395865	2011	7	1	410	36.04	27.29
23395865	2011	7	1	420	38.76	27.17
23395865	2011	7	1	430	41.28	27.07
23395865	2011	7	1	440	42.63	26.96
23395865	2011	7	1	450	43.43	26.92
23395865	2011	7	1	460	44.15	26.85
23395865	2011	7	1	470	44.72	26.79
23395865	2011	7	1	480	45.51	26.71
23395865	2011	7	1	490	47.53	26.46
23395865	2011	7	1	500	50.08	26.36
23395865	2011	7	1	510	52.58	26.32
23395865	2011	7	1	520	54.62	26.25
23395865	2011	7	1	530	56.53	26.18
23395865	2011	7	1	540	57.48	26.13
23395865	2011	7	1	550	59.45	26.03
23395865	2011	7	1	560	61.34	25.92
23395865	2011	7	1	570	63.23	25.83
23395865	2011	7	1	580	64.28	25.77
23395865	2011	7	1	590	64.15	25.72
23395865	2011	7	1	600	64.31	25.71
23395865	2011	7	1	610	65.08	25.67
23395865	2011	7	1	620	66.67	25.6
23395865	2011	7	1	630	68.36	25.51
23395865	2011	7	1	640	69.14	25.35
23395865	2011	7	1	650	70.29	25.28
23395865	2011	7	1	660	71.58	25.13
23395865	2011	7	1	670	72.15	25.1
23395865	2011	7	1	680	73.1	25
23395865	2011	7	1	690	73.86	24.87
23395865	2011	7	1	700	73.96	24.85
23395865	2011	7	1	710	74.38	24.8
23395865	2011	7	1	720	74.98	24.76
23395865	2011	7	1	730	75.67	24.67
23395865	2011	7	1	740	76.37	24.64
23395865	2011	7	1	750	77.13	24.55
23395865	2011	7	1	760	78.11	24.51
23395865	2011	7	1	770	78.95	24.43
23395865	2011	7	1	780	79.59	24.42
23395865	2011	7	1	790	81.21	24.24
23395865	2011	7	1	800	82.97	24.14
23395865	2011	7	1	810	84.51	24.06
23395865	2011	7	1	820	85.45	23.99
23395865	2011	7	1	830	87.38	23.87
23395865	2011	7	1	840	89.39	23.69
23395865	2011	7	1	850	91.74	23.58
23395865	2011	7	1	860	94.42	23.46

23395865	2011	7	1	870	96.32	23.39
23395865	2011	7	1	880	96.74	23.35
23395865	2011	7	1	890	96.45	23.29
23395865	2011	7	1	900	95.82	23.16
23395865	2011	7	1	910	96.38	23.04
23395865	2011	7	1	920	98.19	22.92
23395865	2011	7	1	930	100.67	22.77
23395865	2011	7	1	940	102.96	22.71
23395865	2011	7	1	950	104.82	22.58
23395865	2011	7	1	960	106.32	22.46
23395865	2011	7	1	970	108.4	22.3
23395865	2011	7	1	980	109.45	22.24
23395865	2011	7	1	990	109.65	22.2
23395865	2011	7	1	1000	111.38	22.07
23395865	2011	7	1	1010	112.66	21.96
23395865	2011	7	1	1020	113.02	21.85
23395865	2011	7	1	1030	114.08	21.74
23395865	2011	7	1	1040	116.02	21.5
23395865	2011	7	1	1050	117.08	21.45
23395865	2011	7	1	1060	117.42	21.45
23395865	2011	7	1	1070	118	21.36
23395865	2011	7	1	1080	118.63	21.27
23395865	2011	7	1	1090	118.27	21.25
23395865	2011	7	1	1100	118.02	21.21
23395865	2011	7	1	1110	117.9	21.14
23395865	2011	7	1	1120	117.94	21.08
23395865	2011	7	1	1130	117.6	21.03
23395865	2011	7	1	1140	117.35	20.91
23395865	2011	7	1	1150	117.7	20.85
23395865	2011	7	1	1160	117.57	20.83
23395865	2011	7	1	1170	117.21	20.8
23395865	2011	7	1	1180	117.22	20.75
23395865	2011	7	1	1190	117.58	20.65
23395865	2011	7	1	1200	117.77	20.64
23395865	2011	7	1	1210	118.29	20.57
23395865	2011	7	1	1220	118.39	20.53
23395865	2011	7	1	1230	118.56	20.51
23395865	2011	7	1	1240	119.61	20.46
23395865	2011	7	1	1250	120.49	20.42
23395865	2011	7	1	1260	120.83	20.4
23395865	2011	7	1	1270	120.91	20.38
23395865	2011	7	1	1280	122.14	20.33
23395865	2011	7	1	1290	123.74	20.29
23395865	2011	7	1	1300	125.58	20.25
23395865	2011	7	1	1310	126.65	20.21
23395865	2011	7	1	1320	126.88	20.19
23395865	2011	7	1	1330	127.01	20.16

23395865	2011	7	1	1340	127.32	20.13
23395865	2011	7	1	1350	127.23	20.07
23395865	2011	7	1	1360	128.35	19.99
23395865	2011	7	1	1370	130.06	19.92
23395865	2011	7	1	1380	131.05	19.87
23395865	2011	7	1	1390	131.62	19.86
23395865	2011	7	1	1400	133.42	19.81
23395865	2011	7	1	1410	135.05	19.74
23395865	2011	7	1	1420	136.91	19.64
23395865	2011	7	1	1430	138.98	19.46
23395865	2011	7	1	1440	140	19.48
23395865	2011	7	1	1450	141.09	19.4
23395865	2011	7	1	1460	141.53	19.35
23395865	2011	7	1	1470	142.06	19.28
23395865	2011	7	1	1480	141.91	19.21
23395865	2011	7	1	1490	142.42	19.17
23395865	2011	7	1	1500	142.15	19.14
23395865	2011	7	1	1510	142.58	19.09
23395865	2011	7	1	1520	142.45	19.04
23395865	2011	7	1	1530	142.2	19
23395865	2011	7	1	1540	142.44	18.91
23395865	2011	7	1	1550	141.65	18.88
23395865	2011	7	1	1560	141.59	18.83
23395865	2011	7	1	1570	141.42	18.82
23395865	2011	7	1	1580	142.01	18.78
23395865	2011	7	1	1590	143.79	18.73
23395865	2011	7	1	1600	145.4	18.7
23395865	2011	7	1	1610	145.64	18.67
23395865	2011	7	1	1620	145.78	18.64
23395865	2011	7	1	1630	145.72	18.63
23395865	2011	7	1	1640	145.79	18.6
23395865	2011	7	1	1650	145.93	18.57
23395865	2011	7	1	1660	145.71	18.55
23395865	2011	7	1	1670	145.89	18.51
23395865	2011	7	1	1680	145.36	18.5
23395865	2011	7	1	1690	144.28	18.5
23395865	2011	7	1	1700	143.24	18.49
23395865	2011	7	1	1710	142.03	18.47
23395865	2011	7	1	1720	141.08	18.46
23395865	2011	7	1	1730	140.38	18.43
23395865	2011	7	1	1740	140.74	18.41
23395865	2011	7	1	1750	141.76	18.39
23395865	2011	7	1	1760	143.18	18.37
23395865	2011	7	1	1770	143.65	18.36
23395865	2011	7	1	1780	143.8	18.35
23395865	2011	7	1	1790	143.5	18.34
23395865	2011	7	1	1800	143.86	18.32

23395865	2011	7	1	1810	144.44	18.32
23395865	2011	7	1	1820	145.5	18.28
23395865	2011	7	1	1830	145.53	18.28
23395865	2011	7	1	1840	145.56	18.28
23395865	2011	7	1	1850	145.74	18.26
23395865	2011	7	1	1860	145.3	18.26
23395865	2011	7	1	1870	145.07	18.25
23395865	2011	7	1	1880	145.03	18.25
23395865	2011	7	1	1890	145.25	18.24
23395865	2011	7	1	1900	145.53	18.23
23395865	2011	7	1	1910	145.55	18.22
23395865	2011	7	1	1920	145.52	18.22
23395865	2011	7	1	1930	145.61	18.21
23395865	2011	7	1	1940	145.62	18.2
23395865	2011	7	1	1950	145.43	18.2
23395865	2011	7	1	1960	144.59	18.19
23395865	2011	7	1	1970	143.99	18.19
23395865	2011	7	1	1980	143.04	18.18
23395865	2011	7	1	1990	143.05	18.18
23395865	2011	7	1	2000	143.47	18.17
23395865	2011	7	1	2010	143.69	18.17
23395865	2011	7	1	2020	143.68	18.17
23395865	2011	7	1	2030	143.3	18.17
23395865	2011	7	1	2040	142.89	18.17
23395865	2011	7	1	2050	143.59	18.17
23395865	2011	7	1	2060	144.33	18.17
23395865	2011	7	1	2070	144.36	18.17
23395865	2011	7	1	2080	144.84	18.17
23395865	2011	7	1	2090	144.55	18.17
23395865	2011	7	1	2100	144.94	18.17
23395865	2011	7	1	2110	146.68	18.15
23395865	2011	7	1	2120	148.29	18.15
23395865	2011	7	1	2130	150.06	18.14
23395865	2011	7	1	2140	151.42	18.14
23395865	2011	7	1	2150	151.78	18.13
23395865	2011	7	1	2160	152.07	18.12
23395865	2011	7	1	2170	153	18.1
23395865	2011	7	1	2180	153.93	18.1
23395865	2011	7	1	2190	154.17	18.1
23395865	2011	7	1	2200	155.37	18.1
23395865	2011	7	1	2210	156.1	18.09
23395865	2011	7	1	2220	155.75	18.09
23395865	2011	7	1	2230	155.8	18.08
23395865	2011	7	1	2240	157.53	18.08
23395865	2011	7	1	2250	158.49	18.07
23395865	2011	7	1	2260	159.71	18.07
23395865	2011	7	1	2270	160.09	18

23395865	2011	7	1	2280	161.51	17.91
23395865	2011	7	1	2290	162.18	17.89
23395865	2011	7	1	2300	162.34	17.85
23395865	2011	7	1	2310	163.17	17.8
23395865	2011	7	1	2320	163.28	17.77
23395865	2011	7	1	2330	163.28	17.71
23395865	2011	7	1	2340	163.67	17.68
23395865	2011	7	1	2350	163.73	17.65
23395865	2011	7	1	2360	163.59	17.61
23395865	2011	7	1	2370	164.41	17.58
23395865	2011	7	1	2380	165.22	17.53
23395865	2011	7	1	2390	165.29	17.49
23395865	2011	7	1	2400	166.65	17.47
23395865	2011	7	1	2410	167.41	17.46
23395865	2011	7	1	2420	167.66	17.45
23395865	2011	7	1	2430	167.82	17.42
23395865	2011	7	1	2440	169.1	17.39
23395865	2011	7	1	2450	169.68	17.37
23395865	2011	7	1	2460	171.02	17.35
23395865	2011	7	1	2470	171.4	17.33
23395865	2011	7	1	2480	172	17.31
23395865	2011	7	1	2490	172.56	17.29
23395865	2011	7	1	2500	173.17	17.28
23395865	2011	7	1	2510	173.15	17.28
23395865	2011	7	1	2520	173.31	17.24
23395865	2011	7	1	2530	174.14	17.21
23395865	2011	7	1	2540	174.44	17.17
23395865	2011	7	1	2550	175.27	17.12
23395865	2011	7	1	2560	175.53	17.1
23395865	2011	7	1	2570	176.64	17.08
23395865	2011	7	1	2580	177.96	17.07
23395865	2011	7	1	2590	178.26	17.06
23395865	2011	7	1	2600	179.02	17.02
23395865	2011	7	1	2610	179.42	16.99
23395865	2011	7	1	2620	180.71	16.89
23395865	2011	7	1	2630	181.43	16.84
23395865	2011	7	1	2640	181.64	16.78
23395865	2011	7	1	2650	182.28	16.7
23395865	2011	7	1	2660	182.86	16.64
23395865	2011	7	1	2670	183.84	16.59
23395865	2011	7	1	2680	184.4	16.55
23395865	2011	7	1	2690	184.52	16.5
23395865	2011	7	1	2700	185.78	16.43
23395865	2011	7	1	2710	186.19	16.4
23395865	2011	7	1	2720	187.33	16.34
23395865	2011	7	1	2730	188.06	16.32
23395865	2011	7	1	2740	188.99	16.28

23395865	2011	7	1	2750	189.9	16.24
23395865	2011	7	1	2760	191.02	16.17
23395865	2011	7	1	2770	191.78	16.14
23395865	2011	7	1	2780	192.47	16.1
23395865	2011	7	1	2790	193.36	16.03
23395865	2011	7	1	2800	194	16
23395865	2011	7	1	2810	194.98	15.94
23395865	2011	7	1	2820	195.37	15.93
23395865	2011	7	1	2830	195.82	15.85
23395865	2011	7	1	2840	195.93	15.84
23395865	2011	7	1	2850	196.27	15.81
23395865	2011	7	1	2860	196.53	15.78
23395865	2011	7	1	2870	197.18	15.73
23395865	2011	7	1	2880	196.94	15.71
23395865	2011	7	1	2890	196.28	15.68
23395865	2011	7	1	2900	196.3	15.65
23395865	2011	7	1	2910	196.76	15.6
23395865	2011	7	1	2920	196.67	15.6
23395865	2011	7	1	2930	198.03	15.58
23395865	2011	7	1	2940	199.41	15.55
23395865	2011	7	1	2950	199.76	15.53
23395865	2011	7	1	2960	199.44	15.53
23395865	2011	7	1	2970	199.06	15.5
23395865	2011	7	1	2980	199.45	15.49
23395865	2011	7	1	2990	199.45	15.47
23395865	2011	7	1	3000	199.79	15.45
23395865	2011	7	1	3010	199.64	15.44
23395865	2011	7	1	3020	199.67	15.42
23395865	2011	7	1	3030	200	15.4
23395865	2011	7	1	3040	200.71	15.39
23395865	2011	7	1	3050	200.9	15.37
23395865	2011	7	1	3060	201.63	15.35
23395865	2011	7	1	3070	202.12	15.33
23395865	2011	7	1	3080	203.11	15.32
23395865	2011	7	1	3090	204.2	15.32
23395865	2011	7	1	3100	204.54	15.29
23395865	2011	7	1	3110	204.99	15.28
23395865	2011	7	1	3120	205.51	15.27
23395865	2011	7	1	3130	205.93	15.25
23395865	2011	7	1	3140	206.04	15.25
23395865	2011	7	1	3150	206.28	15.22
23395865	2011	7	1	3160	206.8	15.17
23395865	2011	7	1	3170	207.31	15.14
23395865	2011	7	1	3180	208.2	15.1
23395865	2011	7	1	3190	208.72	15.07
23395865	2011	7	1	3200	209.26	15.05
23395865	2011	7	1	3210	209.12	15.03

23395865	2011	7	1	3220	208.91	15
23395865	2011	7	1	3230	209.16	14.97
23395865	2011	7	1	3240	209.04	14.95
23395865	2011	7	1	3250	208.87	14.91
23395865	2011	7	1	3260	208.5	14.85
23395865	2011	7	1	3270	208.29	14.81
23395865	2011	7	1	3280	207.67	14.78
23395865	2011	7	1	3290	206.87	14.77
23395865	2011	7	1	3300	206.51	14.75
23395865	2011	7	1	3310	205.72	14.75
23395865	2011	7	1	3320	205.21	14.73
23395865	2011	7	1	3330	205.54	14.71
23395865	2011	7	1	3340	205.69	14.69
23395865	2011	7	1	3350	205.62	14.69
23395865	2011	7	1	3360	205.69	14.69
23395865	2011	7	1	3370	205.43	14.71
23395865	2011	7	1	3380	205.37	14.71
23395865	2011	7	1	3390	205.44	14.71
23395865	2011	7	1	3400	205.5	14.73
23395865	2011	7	1	3410	205.45	14.73
23395865	2011	7	1	3420	205.6	14.75
23395865	2011	7	1	3430	205.45	14.75
23395865	2011	7	1	3440	205.47	14.76
23395865	2011	7	1	3450	205.59	14.77
23395865	2011	7	1	3460	205.76	14.78
23395865	2011	7	1	3470	205.49	14.78
23395865	2011	7	1	3480	205.53	14.78
23395865	2011	7	1	3490	205.75	14.79
23395865	2011	7	1	3500	205.54	14.8
23395865	2011	7	1	3510	205.48	14.81
23395865	2011	7	1	3520	205.6	14.81
23395865	2011	7	1	3530	205.56	14.81
23395865	2011	7	1	3540	205.47	14.81
23395865	2011	7	1	3550	205.47	14.8
23395865	2011	7	1	3560	205.62	14.82
23395865	2011	7	1	3570	205.54	14.81
23395865	2011	7	1	3580	205.46	14.81
23395865	2011	7	1	3590	205.71	14.81
23395865	2011	7	1	3600	205.81	14.82
23395865	2011	7	1	3610	205.65	14.82
23395865	2011	7	1	3620	205.33	14.82
23395865	2011	7	1	3630	205.29	14.82
23395865	2011	7	1	3640	205.42	14.83
23395865	2011	7	1	3650	205.66	14.84
23395865	2011	7	1	3660	205.45	14.85
23395865	2011	7	1	3670	205.45	14.9
23395865	2011	7	1	3680	205.49	14.85

23395865	2011	7	1	3690	205.39	14.91
23395865	2011	7	1	3700	205.33	14.91
23395865	2011	7	1	3710	205.36	14.92
23395865	2011	7	1	3720	205.42	14.92
23395865	2011	7	1	3730	205.6	14.92
23395865	2011	7	1	3740	205.62	14.92
23395865	2011	7	1	3750	205.47	14.92
23395865	2011	7	1	3760	205.17	14.92
23395865	2011	7	1	3770	205.3	14.92
23395865	2011	7	1	3780	205.59	14.92
23395865	2011	7	1	3790	205.6	14.92
23395865	2011	7	1	3800	205.41	14.91
23395865	2011	7	1	3810	205.45	14.91
23395865	2011	7	1	3820	205.41	14.9
23395865	2011	7	1	3830	205.42	14.85
23395865	2011	7	1	3840	205.48	14.83
23395865	2011	7	1	3850	205.45	14.82
23395865	2011	7	1	3860	205.18	14.81
23395865	2011	7	1	3870	205.69	14.79
23395865	2011	7	1	3880	207.64	14.78
23395865	2011	7	1	3890	208.83	14.78
23395865	2011	7	1	3900	209.21	14.78
23395865	2011	7	1	3910	210.05	14.78
23395865	2011	7	1	3920	210.54	14.78
23395865	2011	7	1	3930	210.72	14.76
23395865	2011	7	1	3940	210.74	14.76
23395865	2011	7	1	3950	210.8	14.75
23395865	2011	7	1	3960	210.9	14.75
23395865	2011	7	1	3970	210.9	14.74
23395865	2011	7	1	3980	210.97	14.72
23395865	2011	7	1	3990	211.06	14.72
23395865	2011	7	1	4000	210.87	14.71
23395865	2011	7	1	4010	211.05	14.71
23395865	2011	7	1	4020	211.66	14.7
23395865	2011	7	1	4030	211.71	14.7
23395865	2011	7	1	4040	211.59	14.69
23395865	2011	7	1	4050	211.57	14.67
23395865	2011	7	1	4060	211.68	14.68
23395865	2011	7	1	4070	211.8	14.67
23395865	2011	7	1	4080	211.86	14.67
23395865	2011	7	1	4090	211.57	14.65
23395865	2011	7	1	4100	211.43	14.64
23395865	2011	7	1	4110	211.68	14.64
23395865	2011	7	1	4120	211.97	14.64
23395865	2011	7	1	4130	211.75	14.64
23395865	2011	7	1	4140	211.99	14.64
23395865	2011	7	1	4150	212.46	14.64

23395865	2011	7	1	4160	212.14	14.63
23395865	2011	7	1	4170	212.26	14.62
23395865	2011	7	1	4180	212.36	14.62
23395865	2011	7	1	4190	211.85	14.62
23395865	2011	7	1	4200	210.8	14.62
23395865	2011	7	1	4210	209.47	14.6
23395865	2011	7	1	4220	208.9	14.61
23395865	2011	7	1	4230	208.94	14.6
23395865	2011	7	1	4240	209.32	14.6
23395865	2011	7	1	4250	208.1	14.6
23395865	2011	7	1	4260	207.85	14.6
23395865	2011	7	1	4270	208.06	14.6
23395865	2011	7	1	4280	208.23	14.6
23395865	2011	7	1	4290	209.54	14.59
23395865	2011	7	1	4300	211.19	14.6
23395865	2011	7	1	4310	212.95	14.58
23395865	2011	7	1	4320	213.16	14.57
23395865	2011	7	1	4330	213.05	14.57
23395865	2011	7	1	4340	212.96	14.57
23395865	2011	7	1	4350	213.62	14.57
23395865	2011	7	1	4360	214.56	14.56
23395865	2011	7	1	4370	214.96	14.56
23395865	2011	7	1	4380	215.82	14.54
23395865	2011	7	1	4390	215.49	14.54
23395865	2011	7	1	4400	215.27	14.54
23395865	2011	7	1	4410	215.31	14.53
23395865	2011	7	1	4420	215.46	14.53
23395865	2011	7	1	4430	215.54	14.52
23395865	2011	7	1	4440	214.81	14.51
23395865	2011	7	1	4450	213.73	14.51
23395865	2011	7	1	4460	212.7	14.5
23395865	2011	7	1	4470	212.01	14.5
23395865	2011	7	1	4480	210.87	14.5
23395865	2011	7	1	4490	209.81	14.49
23395865	2011	7	1	4500	209.07	14.48
23395865	2011	7	1	4510	208.01	14.47
23395865	2011	7	1	4520	206.61	14.47
23395865	2011	7	1	4530	206.01	14.48
23395865	2011	7	1	4540	205.88	14.48
23395865	2011	7	1	4550	206.51	14.48
23395865	2011	7	1	4560	206.72	14.49
23395865	2011	7	1	4570	206.32	14.49
23395865	2011	7	1	4580	205.65	14.49
23395865	2011	7	1	4590	204.92	14.49
23395865	2011	7	1	4600	204.36	14.49
23395865	2011	7	1	4610	205.65	14.5
23395865	2011	7	1	4620	207.15	14.51

23395865	2011	7	1	4630	207.01	14.51
23395865	2011	7	1	4640	205.74	14.51
23395865	2011	7	1	4650	205.14	14.51
23395865	2011	7	1	4660	204.84	14.51
23395865	2011	7	1	4670	204.41	14.51
23395865	2011	7	1	4680	203.75	14.52
23395865	2011	7	1	4690	205.43	14.53
23395865	2011	7	1	4700	207.55	14.53
23395865	2011	7	1	4710	208.45	14.53
23395865	2011	7	1	4720	208.52	14.53
23395865	2011	7	1	4730	208.57	14.53
23395865	2011	7	1	4740	208.97	14.53
23395865	2011	7	1	4750	208.95	14.52
23395865	2011	7	1	4760	208.77	14.53
23395865	2011	7	1	4770	208.94	14.53
23395865	2011	7	1	4780	209.23	14.53
23395865	2011	7	1	4790	208.97	14.53
23395865	2011	7	1	4800	208.93	14.52
23395865	2011	7	1	4810	208.75	14.53
23395865	2011	7	1	4820	208.72	14.52
23395865	2011	7	1	4830	208.75	14.53
23395865	2011	7	1	4840	208.58	14.52
23395865	2011	7	1	4850	208.54	14.52
23395865	2011	7	1	4860	208.57	14.51
23395865	2011	7	1	4870	208.9	14.52
23395865	2011	7	1	4880	209.53	14.51
23395865	2011	7	1	4890	210.47	14.52
23395865	2011	7	1	4900	210.85	14.52
23395865	2011	7	1	4910	210.9	14.52
23395865	2011	7	1	4920	210.89	14.52
23395865	2011	7	1	4930	210.82	14.51
23395865	2011	7	1	4940	210.93	14.5
23395865	2011	7	1	4950	211.04	14.51
23395865	2011	7	1	4960	210.73	14.51
23395865	2011	7	1	4970	210.98	14.5
23395865	2011	7	1	4980	211	14.5
23395865	2011	7	1	4990	210.77	14.51
23395865	2011	7	1	5000	210.77	14.5
23395865	2011	7	1	5010	210.83	14.5
23395865	2011	7	1	5020	210.95	14.5
23395865	2011	7	1	5030	210.75	14.5
23395865	2011	7	1	5040	211.05	14.5
23395865	2011	7	1	5050	211.45	14.5
23395865	2011	7	1	5060	210.48	14.49
23395865	2011	7	1	5070	209.54	14.49
23395865	2011	7	1	5080	209.3	14.49
23395865	2011	7	1	5090	208.84	14.49

23395865	2011	7	1	5100	206.82	14.49
23395865	2011	7	1	5110	205.73	14.49
23395865	2011	7	1	5120	205.6	14.49
23395865	2011	7	1	5130	205.8	14.5
23395865	2011	7	1	5140	205.67	14.5
23395865	2011	7	1	5150	205.6	14.49
23395865	2011	7	1	5160	205.72	14.5
23395865	2011	7	1	5170	205.78	14.5
23395865	2011	7	1	5180	205.92	14.52
23395865	2011	7	1	5190	205.98	14.51
23395865	2011	7	1	5200	206.04	14.52
23395865	2011	7	1	5210	205.99	14.53
23395865	2011	7	1	5220	205.99	14.53
23395865	2011	7	1	5230	205.92	14.54
23395865	2011	7	1	5240	205.65	14.56
23395865	2011	7	1	5250	205.68	14.57
23395865	2011	7	1	5260	205.66	14.59
23395865	2011	7	1	5270	205.64	14.59
23395865	2011	7	1	5280	205.87	14.6
23395865	2011	7	1	5290	205.64	14.6
23395865	2011	7	1	5300	205.67	14.61
23395865	2011	7	1	5310	205.68	14.61
23395865	2011	7	1	5320	207.2	14.64
23395865	2011	7	1	5330	208.07	14.64
23395865	2011	7	1	5340	208.5	14.64
23395865	2011	7	1	5350	208.5	14.64
23395865	2011	7	1	5360	208.56	14.65
23395865	2011	7	1	5370	208.75	14.65
23395865	2011	7	1	5380	208.49	14.65
23395865	2011	7	1	5390	208.38	14.66
23395865	2011	7	1	5400	208.39	14.66
23395865	2011	7	1	5410	208.28	14.67
23395865	2011	7	1	5420	208.17	14.67
23395865	2011	7	1	5430	208.58	14.67
23395865	2011	7	1	5440	208.67	14.67
23395865	2011	7	1	5450	208.61	14.67
23395865	2011	7	1	5460	208.83	14.67
23395865	2011	7	1	5470	208.93	14.67
23395865	2011	7	1	5480	208.77	14.67
23395865	2011	7	1	5490	208.89	14.67
23395865	2011	7	1	5500	209.01	14.67
23395865	2011	7	1	5510	209.14	14.67
23395865	2011	7	1	5520	209.05	14.67
23395865	2011	7	1	5530	209.31	14.67
23395865	2011	7	1	5540	209.02	14.67
23395865	2011	7	1	5550	209.17	14.68
23395865	2011	7	1	5560	209.14	14.67

23395865	2011	7	1	5570	209.09	14.68
23395865	2011	7	1	5580	209.23	14.67
23395865	2011	7	1	5590	208.89	14.68
23395865	2011	7	1	5600	209.1	14.68
23395865	2011	7	1	5610	209.11	14.68
23395865	2011	7	1	5620	208.46	14.68
23395865	2011	7	1	5630	208.02	14.68
23395865	2011	7	1	5640	208.76	14.69
23395865	2011	7	1	5650	209.1	14.68
23395865	2011	7	1	5660	209.27	14.69
23395865	2011	7	1	5670	209.2	14.7
23395865	2011	7	1	5680	208.78	14.7
23395865	2011	7	1	5690	208.82	14.7
23395865	2011	7	1	5700	208.6	14.7
23395865	2011	7	1	5710	208.71	14.7
23395865	2011	7	1	5720	208.24	14.7
23395865	2011	7	1	5730	207.45	14.71
23395865	2011	7	1	5740	208.24	14.7
23395865	2011	7	1	5750	208.54	14.7
23395865	2011	7	1	5760	208.21	14.71
23395865	2011	7	1	5770	207.58	14.71
23395865	2011	7	1	5780	207	14.71
23395865	2011	7	1	5790	206.52	14.71
23395865	2011	7	1	5800	205.72	14.71
23395865	2011	7	1	5810	205.21	14.71
23395865	2011	7	1	5820	205.04	14.71
23395865	2011	7	1	5830	205.87	14.71
23395865	2011	7	1	5840	206.34	14.71
23395865	2011	7	1	5850	206	14.71
23395865	2011	7	1	5860	205.81	14.71
23395865	2011	7	1	5870	205.84	14.71
23395865	2011	7	1	5880	205.98	14.71
23395865	2011	7	1	5890	205.3	14.72
23395865	2011	7	1	5900	205.24	14.71
23395865	2011	7	1	5910	205.35	14.71
23395865	2011	7	1	5920	205.37	14.71
23395865	2011	7	1	5930	205.35	14.71
23395865	2011	7	1	5940	205.33	14.72
23395865	2011	7	1	5950	205.39	14.72
23395865	2011	7	1	5960	205.31	14.72
23395865	2011	7	1	5970	205.38	14.72
23395865	2011	7	1	5980	205.22	14.72
23395865	2011	7	1	5990	205.39	14.72
23395865	2011	7	1	6000	205.23	14.71
23395865	2011	7	1	6010	205.19	14.72
23395865	2011	7	1	6020	205.4	14.71
23395865	2011	7	1	6030	205.36	14.71

23395865	2011	7	1	6040	205.2	14.73
23395865	2011	7	1	6050	205.46	14.72
23395865	2011	7	1	6060	205.23	14.72
23395865	2011	7	1	6070	205.35	14.73
23395865	2011	7	1	6080	205.39	14.74
23395865	2011	7	1	6090	205.19	14.73
23395865	2011	7	1	6100	205.15	14.74
23395865	2011	7	1	6110	205.29	14.73
23395865	2011	7	1	6120	205.28	14.74
23395865	2011	7	1	6130	204.92	14.74
23395865	2011	7	1	6140	205.24	14.73
23395865	2011	7	1	6150	205.28	14.73
23395865	2011	7	1	6160	206.63	14.73
23395865	2011	7	1	6170	207.58	14.74
23395865	2011	7	1	6180	207.63	14.73
23395865	2011	7	1	6190	207.78	14.74
23395865	2011	7	1	6200	207.49	14.74
23395865	2011	7	1	6210	207.36	14.73
23395865	2011	7	1	6220	207.85	14.73
23395865	2011	7	1	6230	207.68	14.73
23395865	2011	7	1	6240	207.3	14.72
23395865	2011	7	1	6250	207.75	14.73
23395865	2011	7	1	6260	207.74	14.73
23395865	2011	7	1	6270	207.67	14.73
23395865	2011	7	1	6280	207.49	14.73
23395865	2011	7	1	6290	207.12	14.73
23395865	2011	7	1	6300	206.64	14.74
23395865	2011	7	1	6310	206.56	14.73
23395865	2011	7	1	6320	207.29	14.74
23395865	2011	7	1	6330	207.87	14.73
23395865	2011	7	1	6340	207.79	14.74
23395865	2011	7	1	6350	207.91	14.74
23395865	2011	7	1	6360	207.96	14.73
23395865	2011	7	1	6370	208.13	14.73
23395865	2011	7	1	6380	208.06	14.72
23395865	2011	7	1	6390	208.04	14.72
23395865	2011	7	1	6400	207.96	14.71
23395865	2011	7	1	6410	208.05	14.71
23395865	2011	7	1	6420	207.76	14.71
23395865	2011	7	1	6430	208.03	14.71
23395865	2011	7	1	6440	208.04	14.71
23395865	2011	7	1	6450	207.9	14.71
23395865	2011	7	1	6460	207.96	14.71
23395865	2011	7	1	6470	208.02	14.71
23395865	2011	7	1	6480	208.16	14.71
23395865	2011	7	1	6490	208.16	14.71
23395865	2011	7	1	6500	208.16	14.71

23395865	2011	7	1	6510	208.25	14.7
23395865	2011	7	1	6520	208.17	14.7
23395865	2011	7	1	6530	207.92	14.7
23395865	2011	7	1	6540	208.44	14.7
23395865	2011	7	1	6550	208.25	14.71
23395865	2011	7	1	6560	208.08	14.7
23395865	2011	7	1	6570	207.79	14.7
23395865	2011	7	1	6580	208.22	14.7
23395865	2011	7	1	6590	208.23	14.7
23395865	2011	7	1	6600	208.18	14.7
23395865	2011	7	1	6610	208.24	14.69
23395865	2011	7	1	6620	208.14	14.69
23395865	2011	7	1	6630	208.25	14.68
23395865	2011	7	1	6640	208.18	14.69
23395865	2011	7	1	6650	208.18	14.68
23395865	2011	7	1	6660	208.01	14.69
23395865	2011	7	1	6670	208.24	14.68
23395865	2011	7	1	6680	207.94	14.68
23395865	2011	7	1	6690	207.87	14.67
23395865	2011	7	1	6700	208.01	14.67
23395865	2011	7	1	6710	207.45	14.67
23395865	2011	7	1	6720	206.49	14.67
23395865	2011	7	1	6730	205.65	14.67
23395865	2011	7	1	6740	204.88	14.67
23395865	2011	7	1	6750	204.74	14.67
23395865	2011	7	1	6760	205.03	14.67
23395865	2011	7	1	6770	205.1	14.67
23395865	2011	7	1	6780	205.08	14.67
23395865	2011	7	1	6790	205.01	14.68
23395865	2011	7	1	6800	205.15	14.68
23395865	2011	7	1	6810	205.14	14.67
23395865	2011	7	1	6820	205.02	14.67
23395865	2011	7	1	6830	205.04	14.67
23395865	2011	7	1	6840	204.92	14.67
23395865	2011	7	1	6850	205.43	14.68
23395865	2011	7	1	6860	205.69	14.67
23395865	2011	7	1	6870	205.54	14.68
23395865	2011	7	1	6880	205.51	14.67
23395865	2011	7	1	6890	205.44	14.67
23395865	2011	7	1	6900	205.4	14.67
23395865	2011	7	1	6910	205.34	14.67
23395865	2011	7	1	6920	205.33	14.67
23395865	2011	7	1	6930	205.61	14.67
23395865	2011	7	1	6940	205.47	14.67
23395865	2011	7	1	6950	205.48	14.67
23395865	2011	7	1	6960	205.42	14.67
23395865	2011	7	1	6970	205.65	14.67

23395865	2011	7	1	6980	205.59	14.67
23395865	2011	7	1	6990	205.75	14.67
23395865	2011	7	1	7000	205.54	14.67
23395865	2011	7	1	7010	205.42	14.67
23395865	2011	7	1	7020	205.92	14.67
23395865	2011	7	1	7030	205.98	14.67
23395865	2011	7	1	7040	205.92	14.66
23395865	2011	7	1	7050	206.02	14.67
23395865	2011	7	1	7060	205.84	14.67
23395865	2011	7	1	7070	206.05	14.67
23395865	2011	7	1	7080	207.54	14.67
23395865	2011	7	1	7090	209.05	14.67
23395865	2011	7	1	7100	209.77	14.67
23395865	2011	7	1	7110	211.34	14.67
23395865	2011	7	1	7120	212.2	14.64
23395865	2011	7	1	7130	212.5	14.62
23395865	2011	7	1	7140	213.77	14.59
23395865	2011	7	1	7150	214.26	14.58
23395865	2011	7	1	7160	214.97	14.57
23395865	2011	7	1	7170	215.79	14.55
23395865	2011	7	1	7180	216.71	14.53
23395865	2011	7	1	7190	218.24	14.47
23395865	2011	7	1	7200	218.75	14.46
23395865	2011	7	1	7210	220.05	14.41
23395865	2011	7	1	7220	220.61	14.41
23395865	2011	7	1	7230	221.13	14.37
23395865	2011	7	1	7240	221.61	14.32
23395865	2011	7	1	7250	222.45	14.3
23395865	2011	7	1	7260	223.79	14.25
23395865	2011	7	1	7270	224.77	14.25
23395865	2011	7	1	7280	225.7	14.21
23395865	2011	7	1	7290	226.79	14.2
23395865	2011	7	1	7300	227.06	14.18
23395865	2011	7	1	7310	228.17	14.14
23395865	2011	7	1	7320	229.26	14.13
23395865	2011	7	1	7330	229.87	14.11
23395865	2011	7	1	7340	229.75	14.1
23395865	2011	7	1	7350	229.86	14.09
23395865	2011	7	1	7360	229.95	14.07
23395865	2011	7	1	7370	230.8	14.03
23395865	2011	7	1	7380	231.64	14.02
23395865	2011	7	1	7390	232.65	14
23395865	2011	7	1	7400	233.42	13.98
23395865	2011	7	1	7410	233.96	13.96
23395865	2011	7	1	7420	235.05	13.93
23395865	2011	7	1	7430	235.76	13.91
23395865	2011	7	1	7440	236.31	13.89

23395865	2011	7	1	7450	236.47	13.88
23395865	2011	7	1	7460	236.88	13.87
23395865	2011	7	1	7470	237.75	13.8
23395865	2011	7	1	7480	238.47	13.78
23395865	2011	7	1	7490	239.24	13.76
23395865	2011	7	1	7500	239.94	13.74
23395865	2011	7	1	7510	240.63	13.71
23395865	2011	7	1	7520	241.3	13.69
23395865	2011	7	1	7530	241.97	13.66
23395865	2011	7	1	7540	242.43	13.64
23395865	2011	7	1	7550	242.96	13.63
23395865	2011	7	1	7560	243.29	13.62
23395865	2011	7	1	7570	243.61	13.62
23395865	2011	7	1	7580	244.27	13.6
23395865	2011	7	1	7590	244.74	13.59
23395865	2011	7	1	7600	244.94	13.57
23395865	2011	7	1	7610	244.87	13.54
23395865	2011	7	1	7620	244.77	13.54
23395865	2011	7	1	7630	244.77	13.53
23395865	2011	7	1	7640	244.66	13.51
23395865	2011	7	1	7650	244.82	13.48
23395865	2011	7	1	7660	245.28	13.46
23395865	2011	7	1	7670	245.74	13.45
23395865	2011	7	1	7680	245.65	13.42
23395865	2011	7	1	7690	246.63	13.39
23395865	2011	7	1	7700	246.7	13.37
23395865	2011	7	1	7710	246.92	13.35
23395865	2011	7	1	7720	246.77	13.34
23395865	2011	7	1	7730	246.3	13.33
23395865	2011	7	1	7740	244.95	13.31
23395865	2011	7	1	7750	243.72	13.3
23395865	2011	7	1	7760	243.56	13.28
23395865	2011	7	1	7770	241.74	13.28
23395865	2011	7	1	7780	240.21	13.25
23395865	2011	7	1	7790	239.42	13.25
23395865	2011	7	1	7800	238.73	13.25
23395865	2011	7	1	7810	238.47	13.26
23395865	2011	7	1	7820	238.18	13.28
23395865	2011	7	1	7830	236.41	13.29
23395865	2011	7	1	7840	234.89	13.31
23395865	2011	7	1	7850	233.62	13.32
23395865	2011	7	1	7860	233.11	13.35
23395865	2011	7	1	7870	233.18	13.37
23395865	2011	7	1	7880	233.72	13.4
23395865	2011	7	1	7890	233.81	13.41
23395865	2011	7	1	7900	234.03	13.42
23395865	2011	7	1	7910	234.08	13.42

23395865	2011	7	1	7920	234.17	13.45
23395865	2011	7	1	7930	234.11	13.46
23395865	2011	7	1	7940	233.93	13.47
23395865	2011	7	1	7950	234.02	13.47
23395865	2011	7	1	7960	234.02	13.5
23395865	2011	7	1	7970	233.88	13.5
23395865	2011	7	1	7980	233.98	13.51
23395865	2011	7	1	7990	234.2	13.53
23395865	2011	7	1	8000	234.14	13.54
23395865	2011	7	1	8010	234.05	13.54
23395865	2011	7	1	8020	233.99	13.55
23395865	2011	7	1	8030	233.4	13.56
23395865	2011	7	1	8040	233.63	13.56
23395865	2011	7	1	8050	233.95	13.58
23395865	2011	7	1	8060	233.69	13.58
23395865	2011	7	1	8070	233.71	13.58
23395865	2011	7	1	8080	233.57	13.59
23395865	2011	7	1	8090	232.89	13.59
23395865	2011	7	1	8100	232.17	13.6
23395865	2011	7	1	8110	232.62	13.62
23395865	2011	7	1	8120	232.76	13.63
23395865	2011	7	1	8130	232.33	13.63
23395865	2011	7	1	8140	231.68	13.64
23395865	2011	7	1	8150	231.46	13.65
23395865	2011	7	1	8160	232.54	13.67
23395865	2011	7	1	8170	233.19	13.67
23395865	2011	7	1	8180	233.89	13.68
23395865	2011	7	1	8190	234.36	13.68
23395865	2011	7	1	8200	234.84	13.69
23395865	2011	7	1	8210	235	13.7
23395865	2011	7	1	8220	235.01	13.69
23395865	2011	7	1	8230	234.96	13.71
23395865	2011	7	1	8240	233.66	13.71
23395865	2011	7	1	8250	232.58	13.71
23395865	2011	7	1	8260	232.18	13.71
23395865	2011	7	1	8270	231.73	13.71
23395865	2011	7	1	8280	231.1	13.71
23395865	2011	7	1	8290	230.21	13.71
23395865	2011	7	1	8300	229.33	13.71
23395865	2011	7	1	8310	229.54	13.72
23395865	2011	7	1	8320	230.19	13.73
23395865	2011	7	1	8330	230.76	13.74
23395865	2011	7	1	8340	230.95	13.74
23395865	2011	7	1	8350	231.08	13.75
23395865	2011	7	1	8360	231.29	13.75
23395865	2011	7	1	8370	231.28	13.76
23395865	2011	7	1	8380	231.27	13.78

23395865	2011	7	1	8390	231.36	13.77
23395865	2011	7	1	8400	231.15	13.78
23395865	2011	7	1	8410	231.19	13.78
23395865	2011	7	1	8420	231.14	13.78
23395865	2011	7	1	8430	231.01	13.8
23395865	2011	7	1	8440	231.18	13.85
23395865	2011	7	1	8450	231.37	13.85
23395865	2011	7	1	8460	231.29	13.86
23395865	2011	7	1	8470	231.34	13.87
23395865	2011	7	1	8480	231.15	13.88
23395865	2011	7	1	8490	231.26	13.89
23395865	2011	7	1	8500	231.49	13.89
23395865	2011	7	1	8510	231.53	13.91
23395865	2011	7	1	8520	231.36	13.9
23395865	2011	7	1	8530	230.62	13.91
23395865	2011	7	1	8540	229.57	13.92
23395865	2011	7	1	8550	228.64	13.92
23395865	2011	7	1	8560	228.25	13.92
23395865	2011	7	1	8570	227.56	13.93
23395865	2011	7	1	8580	226.32	13.94
23395865	2011	7	1	8590	225.3	13.94
23395865	2011	7	1	8600	223.34	13.95
23395865	2011	7	1	8610	221.19	13.96
23395865	2011	7	1	8620	219.47	13.96
23395865	2011	7	1	8630	218.13	13.98
23395865	2011	7	1	8640	216.23	13.99
23395865	2011	7	1	8650	215.44	14
23395865	2011	7	1	8660	215.74	14.03
23395865	2011	7	1	8670	216.79	14.06
23395865	2011	7	1	8680	216.32	14.07
23395865	2011	7	1	8690	214.41	14.08
23395865	2011	7	1	8700	213.23	14.08
23395865	2011	7	1	8710	211.99	14.1
23395865	2011	7	1	8720	211.86	14.11
23395865	2011	7	1	8730	209.93	14.12
23395865	2011	7	1	8740	208.29	14.13
23395865	2011	7	1	8750	207.1	14.14
23395865	2011	7	1	8760	206.38	14.15
23395865	2011	7	1	8770	205.38	14.17
23395865	2011	7	1	8780	204.62	14.17
23395865	2011	7	1	8790	204.89	14.2
23395865	2011	7	1	8800	204.99	14.21
23395865	2011	7	1	8810	204.92	14.21
23395865	2011	7	1	8820	204.9	14.22
23395865	2011	7	1	8830	205.01	14.24
23395865	2011	7	1	8840	204.91	14.25
23395865	2011	7	1	8850	204.84	14.25

23395865	2011	7	1	8860	204.91	14.25
23395865	2011	7	1	8870	205	14.27
23395865	2011	7	1	8880	204.95	14.28
23395865	2011	7	1	8890	204.84	14.28
23395865	2011	7	1	8900	205.03	14.29
23395865	2011	7	1	8910	204.89	14.29
23395865	2011	7	1	8920	204.94	14.31
23395865	2011	7	1	8930	204.89	14.31
23395865	2011	7	1	8940	204.94	14.32
23395865	2011	7	1	8950	205	14.32
23395865	2011	7	1	8960	204.93	14.32
23395865	2011	7	1	8970	205.03	14.34
23395865	2011	7	1	8980	204.92	14.34
23395865	2011	7	1	8990	204.95	14.35
23395865	2011	7	1	9000	204.91	14.35
23395865	2011	7	1	9010	204.87	14.36
23395865	2011	7	1	9020	203.46	14.36
23395865	2011	7	1	9030	202.79	14.37
23395865	2011	7	1	9040	203.48	14.39
23395865	2011	7	1	9050	203.48	14.39
23395865	2011	7	1	9060	203.43	14.4
23395865	2011	7	1	9070	203.41	14.4
23395865	2011	7	1	9080	203.4	14.41
23395865	2011	7	1	9090	203.43	14.42
23395865	2011	7	1	9100	203.34	14.42
23395865	2011	7	1	9110	203.48	14.42
23395865	2011	7	1	9120	203.33	14.43
23395865	2011	7	1	9130	203.45	14.43
23395865	2011	7	1	9140	203.38	14.44
23395865	2011	7	1	9150	203.42	14.44
23395865	2011	7	1	9160	203.53	14.45
23395865	2011	7	1	9170	203.26	14.45
23395865	2011	7	1	9180	203.02	14.46
23395865	2011	7	1	9190	202.85	14.46
23395865	2011	7	1	9200	201.02	14.47
23395865	2011	7	1	9210	199.4	14.48
23395865	2011	7	1	9220	199.61	14.49
23395865	2011	7	1	9230	200.41	14.5
23395865	2011	7	1	9240	200.89	14.5
23395865	2011	7	1	9250	201.85	14.5
23395865	2011	7	1	9260	202.04	14.5
23395865	2011	7	1	9270	202.17	14.51
23395865	2011	7	1	9280	202.05	14.51
23395865	2011	7	1	9290	202.02	14.51
23395865	2011	7	1	9300	201.68	14.52
23395865	2011	7	1	9310	201.69	14.52
23395865	2011	7	1	9320	201.81	14.53

23395865	2011	7	1	9330	201.4	14.53
23395865	2011	7	1	9340	202	14.53
23395865	2011	7	1	9350	202.13	14.54
23395865	2011	7	1	9360	201.97	14.53
23395865	2011	7	1	9370	202.51	14.54
23395865	2011	7	1	9380	202.26	14.54
23395865	2011	7	1	9390	202.02	14.54
23395865	2011	7	1	9400	201.76	14.55
23395865	2011	7	1	9410	201.85	14.55
23395865	2011	7	1	9420	201.98	14.54
23395865	2011	7	1	9430	202	14.55
23395865	2011	7	1	9440	202.14	14.55
23395865	2011	7	1	9450	202.13	14.55
23395865	2011	7	1	9460	203.48	14.56
23395865	2011	7	1	9470	203.49	14.56
23395865	2011	7	1	9480	203.47	14.56
23395865	2011	7	1	9490	203.51	14.56
23395865	2011	7	1	9500	203.47	14.57
23395865	2011	7	1	9510	203.43	14.56
23395865	2011	7	1	9520	203.06	14.56
23395865	2011	7	1	9530	203.22	14.56
23395865	2011	7	1	9540	202.94	14.57
23395865	2011	7	1	9550	202.5	14.56
23395865	2011	7	1	9560	202.02	14.56
23395865	2011	7	1	9570	201.91	14.57
23395865	2011	7	1	9580	202.18	14.56
23395865	2011	7	1	9590	202.35	14.56
23395865	2011	7	1	9600	202.25	14.57
23395865	2011	7	1	9610	201.85	14.57
23395865	2011	7	1	9620	201.27	14.57
23395865	2011	7	1	9630	201.14	14.57
23395865	2011	7	1	9640	201.36	14.57
23395865	2011	7	1	9650	201.32	14.57
23395865	2011	7	1	9660	200.94	14.57
23395865	2011	7	1	9670	200.45	14.57
23395865	2011	7	1	9680	199.75	14.58
23395865	2011	7	1	9690	198.94	14.58
23395865	2011	7	1	9700	198.37	14.58
23395865	2011	7	1	9710	198.58	14.59
23395865	2011	7	1	9720	198.43	14.59
23395865	2011	7	1	9730	198.48	14.59
23395865	2011	7	1	9740	199.38	14.6
23395865	2011	7	1	9750	201.33	14.61
23395865	2011	7	1	9760	202.74	14.62
23395865	2011	7	1	9770	203.48	14.61
23395865	2011	7	1	9780	204.49	14.62
23395865	2011	7	1	9790	205.46	14.61

23395865	2011	7	1	9800	205.85	14.61
23395865	2011	7	1	9810	206.69	14.61
23395865	2011	7	1	9820	207.38	14.61
23395865	2011	7	1	9830	207.82	14.61
23395865	2011	7	1	9840	207.97	14.61
23395865	2011	7	1	9850	207.97	14.61
23395865	2011	7	1	9860	207.53	14.61
23395865	2011	7	1	9870	206.15	14.6
23395865	2011	7	1	9880	205.31	14.6
23395865	2011	7	1	9890	204.19	14.6
23395865	2011	7	1	9900	203.93	14.6
23395865	2011	7	1	9910	203.28	14.6
23395865	2011	7	1	9920	203.02	14.6
23395865	2011	7	1	9930	203.01	14.59
23395865	2011	7	1	9940	202.71	14.6
23395865	2011	7	1	9950	200.95	14.6
23395865	2011	7	1	9960	199.43	14.6
23395865	2011	7	1	9970	199.41	14.6
23395865	2011	7	1	9980	200	14.6
23395865	2011	7	1	9990	199.91	14.6
23395865	2011	7	1	10000	200.26	14.6
23395865	2011	7	1	10010	200.88	14.61
23395865	2011	7	1	10020	200.93	14.61
23395865	2011	7	1	10030	200.76	14.62
23395865	2011	7	1	10040	200.74	14.61
23395865	2011	7	1	10050	200.92	14.62
23395865	2011	7	1	10060	201.7	14.62
23395865	2011	7	1	10070	203.29	14.64
23395865	2011	7	1	10080	203.81	14.64
23395865	2011	7	1	10090	203.82	14.64
23395865	2011	7	1	10100	203.76	14.64
23395865	2011	7	1	10110	203.82	14.64
23395865	2011	7	1	10120	203.82	14.65
23395865	2011	7	1	10130	203.65	14.65
23395865	2011	7	1	10140	203.81	14.65
23395865	2011	7	1	10150	203.74	14.65
23395865	2011	7	1	10160	203.8	14.65
23395865	2011	7	1	10170	203.63	14.65
23395865	2011	7	1	10180	203.38	14.66
23395865	2011	7	1	10190	203.28	14.67
23395865	2011	7	1	10200	203.25	14.68
23395865	2011	7	1	10210	203.22	14.7
23395865	2011	7	1	10220	203.19	14.71
23395865	2011	7	1	10230	201.99	14.71
23395865	2011	7	1	10240	200.73	14.72
23395865	2011	7	1	10250	200.88	14.74
23395865	2011	7	1	10260	201.39	14.75

23395865	2011	7	1	10270	201.83	14.76
23395865	2011	7	1	10280	201.87	14.77
23395865	2011	7	1	10290	201.8	14.78
23395865	2011	7	1	10300	203.02	14.78
23395865	2011	7	1	10310	204.03	14.79
23395865	2011	7	1	10320	204.7	14.78
23395865	2011	7	1	10330	205.21	14.79
23395865	2011	7	1	10340	205.24	14.79
23395865	2011	7	1	10350	205.65	14.78
23395865	2011	7	1	10360	206.37	14.78
23395865	2011	7	1	10370	205.6	14.76
23395865	2011	7	1	10380	203.93	14.75
23395865	2011	7	1	10390	202.94	14.75
23395865	2011	7	1	10400	202.88	14.74
23395865	2011	7	1	10410	203.15	14.74
23395865	2011	7	1	10420	203.14	14.74
23395865	2011	7	1	10430	203.1	14.74
23395865	2011	7	1	10440	203.09	14.74
23395865	2011	7	1	10450	202.85	14.74
23395865	2011	7	1	10460	203.07	14.74
23395865	2011	7	1	10470	203.28	14.73
23395865	2011	7	1	10480	203.02	14.72
23395865	2011	7	1	10490	203.29	14.73
23395865	2011	7	1	10500	202.95	14.73
23395865	2011	7	1	10510	202.97	14.72
23395865	2011	7	1	10520	203.05	14.71
23395865	2011	7	1	10530	203.19	14.71
23395865	2011	7	1	10540	202.95	14.72
23395865	2011	7	1	10550	202.82	14.72
23395865	2011	7	1	10560	202.79	14.71
23395865	2011	7	1	10570	203.03	14.72
23395865	2011	7	1	10580	203.27	14.73
23395865	2011	7	1	10590	203.14	14.72
23395865	2011	7	1	10600	203.29	14.73
23395865	2011	7	1	10610	203.08	14.72
23395865	2011	7	1	10620	203.12	14.73
23395865	2011	7	1	10630	203.22	14.72
23395865	2011	7	1	10640	203.13	14.71
23395865	2011	7	1	10650	202.65	14.72
23395865	2011	7	1	10660	201.42	14.71
23395865	2011	7	1	10670	200.57	14.71
23395865	2011	7	1	10680	201.43	14.71
23395865	2011	7	1	10690	202.24	14.71
23395865	2011	7	1	10700	203	14.71
23395865	2011	7	1	10710	203.63	14.71
23395865	2011	7	1	10720	204.14	14.71
23395865	2011	7	1	10730	204.27	14.71

23395865	2011	7	1	10740	203.98	14.7
23395865	2011	7	1	10750	203.65	14.69
23395865	2011	7	1	10760	203.64	14.68
23395865	2011	7	1	10770	204.06	14.68
23395865	2011	7	1	10780	205.59	14.66
23395865	2011	7	1	10790	207.04	14.65
23395865	2011	7	1	10800	208.11	14.65
23395865	2011	7	1	10810	208.81	14.64
23395865	2011	7	1	10820	208.97	14.63
23395865	2011	7	1	10830	208.87	14.63
23395865	2011	7	1	10840	209.71	14.61
23395865	2011	7	1	10850	210.01	14.61
23395865	2011	7	1	10860	210.39	14.61
23395865	2011	7	1	10870	210.14	14.6
23395865	2011	7	1	10880	209.91	14.6
23395865	2011	7	1	10890	209.97	14.6
23395865	2011	7	1	10900	210.4	14.59
23395865	2011	7	1	10910	210.89	14.58
23395865	2011	7	1	10920	211.74	14.59
23395865	2011	7	1	10930	212.1	14.58
23395865	2011	7	1	10940	212.65	14.57
23395865	2011	7	1	10950	212.33	14.58
23395865	2011	7	1	10960	212.64	14.57
23395865	2011	7	1	10970	212.03	14.57
23395865	2011	7	1	10980	211.09	14.57
23395865	2011	7	1	10990	209.48	14.55
23395865	2011	7	1	11000	207.61	14.55
23395865	2011	7	1	11010	205.82	14.55
23395865	2011	7	1	11020	205.41	14.53
23395865	2011	7	1	11030	205.14	14.54
23395865	2011	7	1	11040	204.85	14.54
23395865	2011	7	1	11050	204.91	14.54
23395865	2011	7	1	11060	205.1	14.53
23395865	2011	7	1	11070	205.4	14.53
23395865	2011	7	1	11080	205.48	14.53
23395865	2011	7	1	11090	205.56	14.53
23395865	2011	7	1	11100	205.35	14.53
23395865	2011	7	1	11110	205.37	14.53
23395865	2011	7	1	11120	204.75	14.53
23395865	2011	7	1	11130	204.78	14.53
23395865	2011	7	1	11140	205.03	14.54
23395865	2011	7	1	11150	205.13	14.54
23395865	2011	7	1	11160	204.11	14.53
23395865	2011	7	1	11170	203.93	14.54
23395865	2011	7	1	11180	203.79	14.54
23395865	2011	7	1	11190	203.66	14.55
23395865	2011	7	1	11200	203.95	14.55

23395865	2011	7	1	11210	204.43	14.55
23395865	2011	7	1	11220	204.93	14.55
23395865	2011	7	1	11230	205.55	14.55
23395865	2011	7	1	11240	206.01	14.56
23395865	2011	7	1	11250	206.28	14.56
23395865	2011	7	1	11260	206.57	14.56
23395865	2011	7	1	11270	205.72	14.56
23395865	2011	7	1	11280	204.47	14.57
23395865	2011	7	1	11290	204.32	14.56
23395865	2011	7	1	11300	204.72	14.57
23395865	2011	7	1	11310	205.43	14.57
23395865	2011	7	1	11320	205.68	14.57
23395865	2011	7	1	11330	204.64	14.57
23395865	2011	7	1	11340	204.53	14.58
23395865	2011	7	1	11350	204.26	14.59
23395865	2011	7	1	11360	203.5	14.59
23395865	2011	7	1	11370	202.22	14.59
23395865	2011	7	1	11380	202.2	14.6
23395865	2011	7	1	11390	202.29	14.6
23395865	2011	7	1	11400	201.65	14.61
23395865	2011	7	1	11410	200.99	14.62
23395865	2011	7	1	11420	198.98	14.63
23395865	2011	7	1	11430	197.48	14.63
23395865	2011	7	1	11440	196.85	14.64
23395865	2011	7	1	11450	198.29	14.66
23395865	2011	7	1	11460	199.74	14.67
23395865	2011	7	1	11470	201.83	14.7
23395865	2011	7	1	11480	203.04	14.7
23395865	2011	7	1	11490	204.08	14.7
23395865	2011	7	1	11500	204.95	14.71
23395865	2011	7	1	11510	205.49	14.71
23395865	2011	7	1	11520	204.46	14.71
23395865	2011	7	1	11530	204.25	14.72
23395865	2011	7	1	11540	204.31	14.72
23395865	2011	7	1	11550	204.14	14.71
23395865	2011	7	1	11560	204.06	14.72
23395865	2011	7	1	11570	204.42	14.71
23395865	2011	7	1	11580	204.99	14.71
23395865	2011	7	1	11590	204.7	14.71
23395865	2011	7	1	11600	204.8	14.7
23395865	2011	7	1	11610	205.47	14.69
23395865	2011	7	1	11620	206.15	14.67
23395865	2011	7	1	11630	206.98	14.66
23395865	2011	7	1	11640	207.37	14.65
23395865	2011	7	1	11650	207.79	14.64
23395865	2011	7	1	11660	208.12	14.63
23395865	2011	7	1	11670	207.96	14.64

23395865	2011	7	1	11680	207.88	14.63
23395865	2011	7	1	11690	208.09	14.63
23395865	2011	7	1	11700	208.42	14.63
23395865	2011	7	1	11710	208.84	14.61
23395865	2011	7	1	11720	209.05	14.61
23395865	2011	7	1	11730	208.98	14.6
23395865	2011	7	1	11740	209.21	14.6
23395865	2011	7	1	11750	209.3	14.6
23395865	2011	7	1	11760	209.32	14.6
23395865	2011	7	1	11770	209.81	14.6
23395865	2011	7	1	11780	211.32	14.58
23395865	2011	7	1	11790	211.83	14.58
23395865	2011	7	1	11800	211.83	14.58
23395865	2011	7	1	11810	212.03	14.57
23395865	2011	7	1	11820	212.1	14.58
23395865	2011	7	1	11830	212.25	14.57
23395865	2011	7	1	11840	211.65	14.57
23395865	2011	7	1	11850	210.71	14.57
23395865	2011	7	1	11860	210.99	14.57
23395865	2011	7	1	11870	211.09	14.56
23395865	2011	7	1	11880	210.53	14.56
23395865	2011	7	1	11890	209.76	14.56
23395865	2011	7	1	11900	209.16	14.55
23395865	2011	7	1	11910	209.16	14.55
23395865	2011	7	1	11920	209.1	14.55
23395865	2011	7	1	11930	209.11	14.56
23395865	2011	7	1	11940	209.1	14.55
23395865	2011	7	1	11950	209.56	14.55
23395865	2011	7	1	11960	209.92	14.54
23395865	2011	7	1	11970	210.16	14.55
23395865	2011	7	1	11980	210.05	14.54
23395865	2011	7	1	11990	210.14	14.54
23395865	2011	7	1	12000	210.09	14.54
23395865	2011	7	1	12010	210.14	14.54
23395865	2011	7	1	12020	209.9	14.54
23395865	2011	7	1	12030	209.86	14.54
23395865	2011	7	1	12040	209.81	14.54
23395865	2011	7	1	12050	209.75	14.53
23395865	2011	7	1	12060	209.6	14.54
23395865	2011	7	1	12070	209.32	14.54
23395865	2011	7	1	12080	208.95	14.54
23395865	2011	7	1	12090	208.56	14.53
23395865	2011	7	1	12100	208.76	14.54
23395865	2011	7	1	12110	208.3	14.53
23395865	2011	7	1	12120	208.2	14.53
23395865	2011	7	1	12130	207.19	14.53
23395865	2011	7	1	12140	206.01	14.53

23395865	2011	7	1	12150	206.96	14.53
23395865	2011	7	1	12160	207.3	14.53
23395865	2011	7	1	12170	207.17	14.53
23395865	2011	7	1	12180	207.21	14.52
23395865	2011	7	1	12190	206.94	14.53
23395865	2011	7	1	12200	206.61	14.53
23395865	2011	7	1	12210	206.61	14.53
23395865	2011	7	1	12220	207.64	14.53
23395865	2011	7	1	12230	207.77	14.53
23395865	2011	7	1	12240	208.15	14.54
23395865	2011	7	1	12250	208.09	14.53
23395865	2011	7	1	12260	208.27	14.53
23395865	2011	7	1	12270	208.14	14.53
23395865	2011	7	1	12280	208.15	14.53
23395865	2011	7	1	12290	208.23	14.53
23395865	2011	7	1	12300	208.28	14.53
23395865	2011	7	1	12310	208.07	14.54
23395865	2011	7	1	12320	207.82	14.53
23395865	2011	7	1	12330	207.53	14.55
23395865	2011	7	1	12340	207.15	14.55
23395865	2011	7	1	12350	206.53	14.55
23395865	2011	7	1	12360	205.04	14.54
23395865	2011	7	1	12370	202.67	14.54
23395865	2011	7	1	12380	200.41	14.55
23395865	2011	7	1	12390	198.77	14.54
23395865	2011	7	1	12400	197.35	14.55
23395865	2011	7	1	12410	195.98	14.55
23395865	2011	7	1	12420	194.9	14.55
23395865	2011	7	1	12430	193.78	14.56
23395865	2011	7	1	12440	192.55	14.56
23395865	2011	7	1	12450	190.35	14.56
23395865	2011	7	1	12460	188.18	14.59
23395865	2011	7	1	12470	186.75	14.62
23395865	2011	7	1	12480	185.17	14.7
23395865	2011	7	1	12490	183.79	14.75
23395865	2011	7	1	12500	182.47	14.81
23395865	2011	7	1	12510	182.45	14.96
23395865	2011	7	1	12520	182	15.03
23395865	2011	7	1	12530	182.03	15.07
23395865	2011	7	1	12540	182.8	15.14
23395865	2011	7	1	12550	182.91	15.17
23395865	2011	7	1	12560	182.52	15.2
23395865	2011	7	1	12570	180.96	15.26
23395865	2011	7	1	12580	178.77	15.29
23395865	2011	7	1	12590	176.62	15.34
23395865	2011	7	1	12600	175.72	15.36
23395865	2011	7	1	12610	174.97	15.39

23395865	2011	7	1	12620	174.27	15.42
23395865	2011	7	1	12630	173.02	15.48
23395865	2011	7	1	12640	171.25	15.52
23395865	2011	7	1	12650	170.19	15.55
23395865	2011	7	1	12660	169.75	15.58
23395865	2011	7	1	12670	169.77	15.63
23395865	2011	7	1	12680	169.67	15.66
23395865	2011	7	1	12690	169.74	15.68
23395865	2011	7	1	12700	169.48	15.71
23395865	2011	7	1	12710	168.88	15.73
23395865	2011	7	1	12720	168.12	15.75
23395865	2011	7	1	12730	167.63	15.78
23395865	2011	7	1	12740	167.09	15.8
23395865	2011	7	1	12750	166.36	15.82
23395865	2011	7	1	12760	165.53	15.85
23395865	2011	7	1	12770	165.08	15.87
23395865	2011	7	1	12780	164.54	15.93
23395865	2011	7	1	12790	163.83	15.95
23395865	2011	7	1	12800	162.97	15.97
23395865	2011	7	1	12810	162.4	16
23395865	2011	7	1	12820	162.5	16.03
23395865	2011	7	1	12830	162.82	16.07
23395865	2011	7	1	12840	162.85	16.09
23395865	2011	7	1	12850	162.38	16.12
23395865	2011	7	1	12860	161.66	16.18
23395865	2011	7	1	12870	161.05	16.21
23395865	2011	7	1	12880	161.75	16.25
23395865	2011	7	1	12890	162.9	16.3
23395865	2011	7	1	12900	162.87	16.33
23395865	2011	7	1	12910	162.91	16.35
23395865	2011	7	1	12920	162.27	16.38
23395865	2011	7	1	12930	161.35	16.4
23395865	2011	7	1	12940	160.53	16.42
23395865	2011	7	1	12950	159.92	16.44
23395865	2011	7	1	12960	159.09	16.48
23395865	2011	7	1	12970	158.21	16.51
23395865	2011	7	1	12980	157.16	16.53
23395865	2011	7	1	12990	157.15	16.57
23395865	2011	7	1	13000	157.12	16.59
23395865	2011	7	1	13010	157.23	16.6
23395865	2011	7	1	13020	157.63	16.64
23395865	2011	7	1	13030	157.15	16.68
23395865	2011	7	1	13040	156.74	16.7
23395865	2011	7	1	13050	157.18	16.71
23395865	2011	7	1	13060	157.67	16.73
23395865	2011	7	1	13070	157.31	16.75
23395865	2011	7	1	13080	156.47	16.76

23395865	2011	7	1	13090	156.78	16.79
23395865	2011	7	1	13100	156.92	16.79
23395865	2011	7	1	13110	156.9	16.79
23395865	2011	7	1	13120	157.11	16.81
23395865	2011	7	1	13130	158.22	16.82
23395865	2011	7	1	13140	158.79	16.84
23395865	2011	7	1	13150	158.69	16.85
23395865	2011	7	1	13160	158.89	16.85
23395865	2011	7	1	13170	158.85	16.86
23395865	2011	7	1	13180	159.09	16.87
23395865	2011	7	1	13190	158.9	16.88
23395865	2011	7	1	13200	157.92	16.89
23395865	2011	7	1	13210	156.65	16.9
23395865	2011	7	1	13220	155.73	16.92
23395865	2011	7	1	13230	154.54	16.92
23395865	2011	7	1	13240	154.01	16.92
23395865	2011	7	1	13250	152.22	16.97
23395865	2011	7	1	13260	151.04	16.98
23395865	2011	7	1	13270	149.95	17
23395865	2011	7	1	13280	147.97	17
23395865	2011	7	1	13290	146.16	17.02
23395865	2011	7	1	13300	143.51	17.05
23395865	2011	7	1	13310	142.23	17.07
23395865	2011	7	1	13320	142.12	17.1
23395865	2011	7	1	13330	142.33	17.14
23395865	2011	7	1	13340	141.27	17.17
23395865	2011	7	1	13350	139.54	17.22
23395865	2011	7	1	13360	137.93	17.27
23395865	2011	7	1	13370	136.68	17.31
23395865	2011	7	1	13380	136.64	17.36
23395865	2011	7	1	13390	138.26	17.45
23395865	2011	7	1	13400	139.1	17.46
23395865	2011	7	1	13410	139.01	17.49
23395865	2011	7	1	13420	138.83	17.5
23395865	2011	7	1	13430	138.77	17.52
23395865	2011	7	1	13440	139.01	17.54
23395865	2011	7	1	13450	139.07	17.57
23395865	2011	7	1	13460	138.92	17.59
23395865	2011	7	1	13470	138.28	17.64
23395865	2011	7	1	13480	136.72	17.67
23395865	2011	7	1	13490	134.76	17.69
23395865	2011	7	1	13500	132.97	17.71
23395865	2011	7	1	13510	131.51	17.75
23395865	2011	7	1	13520	130.56	17.79
23395865	2011	7	1	13530	129.33	17.85
23395865	2011	7	1	13540	127.21	17.88
23395865	2011	7	1	13550	124.89	17.93

23395865	2011	7	1	13560	123.65	18
23395865	2011	7	1	13570	121.94	18.04
23395865	2011	7	1	13580	119.91	18.08
23395865	2011	7	1	13590	117.78	18.12
23395865	2011	7	1	13600	115.68	18.15
23395865	2011	7	1	13610	113.59	18.2
23395865	2011	7	1	13620	111.43	18.24
23395865	2011	7	1	13630	109.74	18.31
23395865	2011	7	1	13640	107.61	18.35
23395865	2011	7	1	13650	105.05	18.4
23395865	2011	7	1	13660	102.3	18.47
23395865	2011	7	1	13670	100.17	18.54
23395865	2011	7	1	13680	97.97	18.62
23395865	2011	7	1	13690	95.69	18.73
23395865	2011	7	1	13700	94.01	18.81
23395865	2011	7	1	13710	92.66	19
23395865	2011	7	1	13720	90.8	19.07
23395865	2011	7	1	13730	90.02	19.18
23395865	2011	7	1	13740	90.76	19.36
23395865	2011	7	1	13750	91.17	19.51
23395865	2011	7	1	13760	91.04	19.6
23395865	2011	7	1	13770	90.91	19.66
23395865	2011	7	1	13780	90.36	19.82
23395865	2011	7	1	13790	88.34	19.91
23395865	2011	7	1	13800	86.15	20.02
23395865	2011	7	1	13810	84.06	20.17
23395865	2011	7	1	13820	82.16	20.26
23395865	2011	7	1	13830	81.41	20.36
23395865	2011	7	1	13840	80.8	20.48
23395865	2011	7	1	13850	80.27	20.57
23395865	2011	7	1	13860	80.39	20.67
23395865	2011	7	1	13870	78.98	20.78
23395865	2011	7	1	13880	78	20.87
23395865	2011	7	1	13890	77.76	21
23395865	2011	7	1	13900	77.38	21.07
23395865	2011	7	1	13910	76.6	21.15
23395865	2011	7	1	13920	76.24	21.21
23395865	2011	7	1	13930	75.53	21.27
23395865	2011	7	1	13940	75.51	21.37
23395865	2011	7	1	13950	74.24	21.43
23395865	2011	7	1	13960	72.54	21.48
23395865	2011	7	1	13970	70.53	21.56
23395865	2011	7	1	13980	68.45	21.66
23395865	2011	7	1	13990	67.66	21.77
23395865	2011	7	1	14000	67.84	21.88
23395865	2011	7	1	14010	68.31	21.98
23395865	2011	7	1	14020	67.79	22.11

23395865	2011	7	1	14030	67.84	22.22
23395865	2011	7	1	14040	67.94	22.32
23395865	2011	7	1	14050	67.7	22.39
23395865	2011	7	1	14060	67.16	22.47
23395865	2011	7	1	14070	66.81	22.52
23395865	2011	7	1	14080	66.05	22.57
23395865	2011	7	1	14090	66.1	22.66
23395865	2011	7	1	14100	66.48	22.72
23395865	2011	7	1	14110	67.18	22.77
23395865	2011	7	1	14120	66.95	22.83
23395865	2011	7	1	14130	66.2	22.89
23395865	2011	7	1	14140	66	22.96
23395865	2011	7	1	14150	65.61	23.02
23395865	2011	7	1	14160	65.43	23.07
23395865	2011	7	1	14170	65.86	23.1
23395865	2011	7	1	14180	66.1	23.14
23395865	2011	7	1	14190	65.46	23.17
23395865	2011	7	1	14200	63.83	23.21
23395865	2011	7	1	14210	62.03	23.25
23395865	2011	7	1	14220	61.11	23.31
23395865	2011	7	1	14230	61.19	23.35
23395865	2011	7	1	14240	60.74	23.39
23395865	2011	7	1	14250	59.61	23.42
23395865	2011	7	1	14260	58.98	23.47
23395865	2011	7	1	14270	58.49	23.5
23395865	2011	7	1	14280	58.35	23.53
23395865	2011	7	1	14290	58.46	23.55
23395865	2011	7	1	14300	58.02	23.57
23395865	2011	7	1	14310	58.27	23.59
23395865	2011	7	1	14320	59	23.6
23395865	2011	7	1	14330	59.46	23.62
23395865	2011	7	1	14340	58.31	23.67
23395865	2011	7	1	14350	55.86	23.68
23395865	2011	7	1	14360	53.33	23.73
23395865	2011	7	1	14370	50.54	23.78
23395865	2011	7	1	14380	48.1	23.82
23395865	2011	7	1	14390	46.09	23.93
23395865	2011	7	1	14400	43.88	24
23395865	2011	7	1	14410	41.49	24.07
23395865	2011	7	1	14420	39.44	24.14
23395865	2011	7	1	14430	36.9	24.21
23395865	2011	7	1	14440	35.38	24.32
23395865	2011	7	1	14450	33.05	24.46
23395865	2011	7	1	14460	30.34	24.52
23395865	2011	7	1	14470	27.61	24.6
23395865	2011	7	1	14480	25.28	24.69
23395865	2011	7	1	14490	23.63	24.86

23395865	2011	7	1	14500	22.16	25.07
23395865	2011	7	1	14510	20.71	25.21
23395865	2011	7	1	14520	19.36	25.42
23395865	2011	7	1	14530	17.72	25.54
23395865	2011	7	1	14540	15.99	25.7
23395865	2011	7	1	14550	14.46	25.83
23395865	2011	7	1	14560	14.61	25.95
23395865	2011	7	1	14570	14.81	26.05
23395865	2011	7	1	14580	13.44	26.24
23395865	2011	7	1	14590	11.82	26.35
23395865	2011	7	1	14600	10.42	26.45
23395865	2011	7	1	14610	8.96	26.55
23395865	2011	7	1	14620	7.31	26.66
23395865	2011	7	1	14630	5.5	26.73
23395865	2011	7	1	14640	4.1	26.84
23395865	2011	7	1	14650	3.15	26.95
23395865	2011	7	1	14660	2.47	27.03
23395865	2011	7	1	14670	2.34	27.08
23395865	2011	7	1	14680	1.72	27.16
23395865	2011	7	1	14690	1.83	27.2
23395865	2011	7	1	14700	2.18	27.25
23395865	2011	7	1	14710	2.11	27.32
23395865	2011	7	1	14720	0.66	27.47
23395865	2011	7	1	14730	0.62	27.47
23395865	2011	7	1	14740	0.63	27.49
23395865	2011	7	1	14750	0.63	27.56
23395865	2011	7	1	14760	0.61	27.71
23395865	2011	7	1	14770	0.66	27.72
23395865	2011	7	1	14780	0.67	27.74
23395865	2011	7	1	14790	0.62	27.76
23395865	2011	7	1	14800	0.63	27.8
23395865	2011	7	1	14810	0.62	27.83
23395865	2011	7	1	14820	0.59	27.87
23395865	2011	7	1	14830	0.52	27.89
23395865	2011	7	1	14840	0.52	27.92
23395865	2011	7	1	14850	0.52	27.93
23395865	2011	7	1	14860	0.51	27.96
23420135	2011	7	1	0	1.31	28.8
23420135	2011	7	1	10	1.48	28.79
23420135	2011	7	1	20	1.64	28.78
23420135	2011	7	1	30	2.26	28.78
23420135	2011	7	1	40	2.44	28.78
23420135	2011	7	1	50	2.13	28.78
23420135	2011	7	1	60	2.43	28.78
23420135	2011	7	1	70	2.67	28.76
23420135	2011	7	1	80	2.95	28.75
23420135	2011	7	1	90	4.36	28.7

23420135	2011	7	1	100	6.32	28.64
23420135	2011	7	1	110	8.5	28.59
23420135	2011	7	1	120	10.63	28.57
23420135	2011	7	1	130	11.56	28.56
23420135	2011	7	1	140	11.92	28.55
23420135	2011	7	1	150	13.07	28.53
23420135	2011	7	1	160	13.73	28.51
23420135	2011	7	1	170	14.63	28.49
23420135	2011	7	1	180	16.82	28.46
23420135	2011	7	1	190	18.54	28.42
23420135	2011	7	1	200	19.67	28.39
23420135	2011	7	1	210	20.62	28.38
23420135	2011	7	1	220	20.91	28.36
23420135	2011	7	1	230	21.82	28.35
23420135	2011	7	1	240	23.27	28.32
23420135	2011	7	1	250	24.12	28.31
23420135	2011	7	1	260	25.13	28.29
23420135	2011	7	1	270	25.6	28.28
23420135	2011	7	1	280	26.91	28.25
23420135	2011	7	1	290	29.05	28.22
23420135	2011	7	1	300	30.99	28.07
23420135	2011	7	1	310	32.37	28
23420135	2011	7	1	320	33.85	27.92
23420135	2011	7	1	330	35.29	27.79
23420135	2011	7	1	340	37.28	27.66
23420135	2011	7	1	350	38.54	27.56
23420135	2011	7	1	360	39.94	27.48
23420135	2011	7	1	370	40.81	27.4
23420135	2011	7	1	380	42.35	27.19
23420135	2011	7	1	390	44.26	27.01
23420135	2011	7	1	400	45.63	26.95
23420135	2011	7	1	410	47.17	26.9
23420135	2011	7	1	420	49.13	26.82
23420135	2011	7	1	430	50.42	26.73
23420135	2011	7	1	440	52.07	26.62
23420135	2011	7	1	450	53.76	26.51
23420135	2011	7	1	460	55.44	26.43
23420135	2011	7	1	470	57.13	26.33
23420135	2011	7	1	480	57.94	26.27
23420135	2011	7	1	490	59.55	26.15
23420135	2011	7	1	500	61.32	26.06
23420135	2011	7	1	510	62.92	25.94
23420135	2011	7	1	520	63.39	25.89
23420135	2011	7	1	530	64.05	25.83
23420135	2011	7	1	540	65.68	25.72
23420135	2011	7	1	550	67.42	25.59
23420135	2011	7	1	560	69.28	25.41

23420135	2011	7	1	570	71.1	25.35
23420135	2011	7	1	580	72.71	25.17
23420135	2011	7	1	590	73.83	25.11
23420135	2011	7	1	600	74.13	25.07
23420135	2011	7	1	610	74.2	25.02
23420135	2011	7	1	620	74.44	24.96
23420135	2011	7	1	630	75.35	24.85
23420135	2011	7	1	640	77.12	24.7
23420135	2011	7	1	650	78.64	24.63
23420135	2011	7	1	660	79.61	24.58
23420135	2011	7	1	670	80.21	24.52
23420135	2011	7	1	680	81.2	24.33
23420135	2011	7	1	690	81.68	24.21
23420135	2011	7	1	700	81.41	24.19
23420135	2011	7	1	710	81.38	24.16
23420135	2011	7	1	720	81.81	24.07
23420135	2011	7	1	730	82.44	24
23420135	2011	7	1	740	83.49	23.93
23420135	2011	7	1	750	84.18	23.87
23420135	2011	7	1	760	84.96	23.79
23420135	2011	7	1	770	86.21	23.71
23420135	2011	7	1	780	86.46	23.66
23420135	2011	7	1	790	87.34	23.6
23420135	2011	7	1	800	88.64	23.47
23420135	2011	7	1	810	89.38	23.37
23420135	2011	7	1	820	89.39	23.34
23420135	2011	7	1	830	89.32	23.32
23420135	2011	7	1	840	89.43	23.26
23420135	2011	7	1	850	89.52	23.24
23420135	2011	7	1	860	89.71	23.21
23420135	2011	7	1	870	89.38	23.13
23420135	2011	7	1	880	89.52	23.1
23420135	2011	7	1	890	90	23.07
23420135	2011	7	1	900	89.89	23.05
23420135	2011	7	1	910	90.63	23
23420135	2011	7	1	920	90.76	22.97
23420135	2011	7	1	930	90.26	22.95
23420135	2011	7	1	940	89.97	22.91
23420135	2011	7	1	950	90.17	22.88
23420135	2011	7	1	960	90.32	22.84
23420135	2011	7	1	970	90.24	22.82
23420135	2011	7	1	980	90.16	22.8
23420135	2011	7	1	990	90.1	22.78
23420135	2011	7	1	1000	90.11	22.75
23420135	2011	7	1	1010	90.2	22.72
23420135	2011	7	1	1020	90.2	22.71
23420135	2011	7	1	1030	90.05	22.68

23420135	2011	7	1	1040	90.08	22.66
23420135	2011	7	1	1050	90.07	22.64
23420135	2011	7	1	1060	90.02	22.61
23420135	2011	7	1	1070	90.6	22.6
23420135	2011	7	1	1080	90.7	22.57
23420135	2011	7	1	1090	90.46	22.57
23420135	2011	7	1	1100	90.25	22.55
23420135	2011	7	1	1110	90.42	22.53
23420135	2011	7	1	1120	90.29	22.52
23420135	2011	7	1	1130	90.36	22.5
23420135	2011	7	1	1140	90.43	22.5
23420135	2011	7	1	1150	90.49	22.49
23420135	2011	7	1	1160	90.41	22.49
23420135	2011	7	1	1170	90.52	22.47
23420135	2011	7	1	1180	90.48	22.47
23420135	2011	7	1	1190	90.54	22.46
23420135	2011	7	1	1200	90.87	22.46
23420135	2011	7	1	1210	90.84	22.46
23420135	2011	7	1	1220	91.03	22.46
23420135	2011	7	1	1230	91.26	22.46
23420135	2011	7	1	1240	91.02	22.45
23420135	2011	7	1	1250	90.38	22.46
23420135	2011	7	1	1260	90.1	22.46
23420135	2011	7	1	1270	90.37	22.45
23420135	2011	7	1	1280	91.85	22.45
23420135	2011	7	1	1290	93.16	22.43
23420135	2011	7	1	1300	93.76	22.43
23420135	2011	7	1	1310	95.43	22.42
23420135	2011	7	1	1320	96.88	22.42
23420135	2011	7	1	1330	97.15	22.41
23420135	2011	7	1	1340	97.5	22.4
23420135	2011	7	1	1350	97.75	22.39
23420135	2011	7	1	1360	97.67	22.36
23420135	2011	7	1	1370	97.6	22.35
23420135	2011	7	1	1380	97.51	22.33
23420135	2011	7	1	1390	97.74	22.31
23420135	2011	7	1	1400	99	22.28
23420135	2011	7	1	1410	99.78	22.26
23420135	2011	7	1	1420	100.72	22.23
23420135	2011	7	1	1430	100.96	22.21
23420135	2011	7	1	1440	100.89	22.18
23420135	2011	7	1	1450	100.57	22.16
23420135	2011	7	1	1460	99.9	22.15
23420135	2011	7	1	1470	99.72	22.12
23420135	2011	7	1	1480	100.42	22.09
23420135	2011	7	1	1490	101.24	22.05
23420135	2011	7	1	1500	102.37	22.01

23420135	2011	7	1	1510	103.61	21.98
23420135	2011	7	1	1520	104.46	21.93
23420135	2011	7	1	1530	105.86	21.85
23420135	2011	7	1	1540	106.57	21.79
23420135	2011	7	1	1550	107.7	21.74
23420135	2011	7	1	1560	109.24	21.64
23420135	2011	7	1	1570	110.83	21.57
23420135	2011	7	1	1580	112.03	21.52
23420135	2011	7	1	1590	112.7	21.48
23420135	2011	7	1	1600	114.37	21.4
23420135	2011	7	1	1610	115.9	21.36
23420135	2011	7	1	1620	117.83	21.32
23420135	2011	7	1	1630	119.63	21.27
23420135	2011	7	1	1640	121.25	21.21
23420135	2011	7	1	1650	122.88	21.14
23420135	2011	7	1	1660	123.12	21.07
23420135	2011	7	1	1670	123.1	21.02
23420135	2011	7	1	1680	123.4	20.93
23420135	2011	7	1	1690	123.34	20.91
23420135	2011	7	1	1700	123.48	20.85
23420135	2011	7	1	1710	123.38	20.79
23420135	2011	7	1	1720	122.94	20.75
23420135	2011	7	1	1730	123.19	20.69
23420135	2011	7	1	1740	124.13	20.61
23420135	2011	7	1	1750	124.17	20.56
23420135	2011	7	1	1760	123.7	20.5
23420135	2011	7	1	1770	124.36	20.43
23420135	2011	7	1	1780	125.86	20.37
23420135	2011	7	1	1790	127.52	20.29
23420135	2011	7	1	1800	128.63	20.27
23420135	2011	7	1	1810	128.52	20.23
23420135	2011	7	1	1820	128.64	20.2
23420135	2011	7	1	1830	128.74	20.17
23420135	2011	7	1	1840	129.07	20.13
23420135	2011	7	1	1850	128.94	20.1
23420135	2011	7	1	1860	128.63	20.03
23420135	2011	7	1	1870	128.96	20.01
23420135	2011	7	1	1880	129.61	19.99
23420135	2011	7	1	1890	130.21	19.92
23420135	2011	7	1	1900	129.97	19.9
23420135	2011	7	1	1910	129.72	19.87
23420135	2011	7	1	1920	129.8	19.84
23420135	2011	7	1	1930	129.74	19.79
23420135	2011	7	1	1940	130.24	19.75
23420135	2011	7	1	1950	129.89	19.68
23420135	2011	7	1	1960	130.07	19.67
23420135	2011	7	1	1970	129.74	19.64

23420135	2011	7	1	1980	130.27	19.63
23420135	2011	7	1	1990	130.61	19.6
23420135	2011	7	1	2000	130.54	19.57
23420135	2011	7	1	2010	130.03	19.54
23420135	2011	7	1	2020	130.1	19.5
23420135	2011	7	1	2030	131.09	19.49
23420135	2011	7	1	2040	131.65	19.47
23420135	2011	7	1	2050	131.36	19.46
23420135	2011	7	1	2060	131.25	19.45
23420135	2011	7	1	2070	130.97	19.44
23420135	2011	7	1	2080	131.23	19.42
23420135	2011	7	1	2090	131.39	19.4
23420135	2011	7	1	2100	131.35	19.39
23420135	2011	7	1	2110	131.55	19.37
23420135	2011	7	1	2120	131.37	19.35
23420135	2011	7	1	2130	131.31	19.35
23420135	2011	7	1	2140	131.07	19.32
23420135	2011	7	1	2150	132.07	19.32
23420135	2011	7	1	2160	132.42	19.32
23420135	2011	7	1	2170	131.99	19.31
23420135	2011	7	1	2180	132.49	19.3
23420135	2011	7	1	2190	132.56	19.3
23420135	2011	7	1	2200	132.56	19.3
23420135	2011	7	1	2210	132.46	19.28
23420135	2011	7	1	2220	132.1	19.28
23420135	2011	7	1	2230	132.21	19.28
23420135	2011	7	1	2240	133.25	19.28
23420135	2011	7	1	2250	133.58	19.26
23420135	2011	7	1	2260	133.76	19.26
23420135	2011	7	1	2270	133.8	19.26
23420135	2011	7	1	2280	133.94	19.25
23420135	2011	7	1	2290	133.4	19.25
23420135	2011	7	1	2300	133.64	19.24
23420135	2011	7	1	2310	133.21	19.24
23420135	2011	7	1	2320	133.67	19.22
23420135	2011	7	1	2330	133.91	19.23
23420135	2011	7	1	2340	134.5	19.21
23420135	2011	7	1	2350	134.51	19.21
23420135	2011	7	1	2360	134.48	19.2
23420135	2011	7	1	2370	134.53	19.19
23420135	2011	7	1	2380	134.2	19.19
23420135	2011	7	1	2390	133.26	19.19
23420135	2011	7	1	2400	131.9	19.18
23420135	2011	7	1	2410	131.18	19.19
23420135	2011	7	1	2420	132.3	19.19
23420135	2011	7	1	2430	133.61	19.19
23420135	2011	7	1	2440	134.05	19.18

23420135	2011	7	1	2450	134.07	19.17
23420135	2011	7	1	2460	134.14	19.17
23420135	2011	7	1	2470	134.18	19.14
23420135	2011	7	1	2480	134.09	19.14
23420135	2011	7	1	2490	134.05	19.14
23420135	2011	7	1	2500	133.76	19.14
23420135	2011	7	1	2510	133.42	19.14
23420135	2011	7	1	2520	132.93	19.13
23420135	2011	7	1	2530	132.75	19.13
23420135	2011	7	1	2540	132.49	19.13
23420135	2011	7	1	2550	132.19	19.13
23420135	2011	7	1	2560	131.71	19.12
23420135	2011	7	1	2570	131.57	19.12
23420135	2011	7	1	2580	131.84	19.13
23420135	2011	7	1	2590	131.62	19.12
23420135	2011	7	1	2600	131.47	19.13
23420135	2011	7	1	2610	130.86	19.14
23420135	2011	7	1	2620	130.02	19.14
23420135	2011	7	1	2630	129.37	19.15
23420135	2011	7	1	2640	129.17	19.17
23420135	2011	7	1	2650	128.54	19.17
23420135	2011	7	1	2660	127.54	19.19
23420135	2011	7	1	2670	127.56	19.2
23420135	2011	7	1	2680	126.8	19.21
23420135	2011	7	1	2690	125.89	19.21
23420135	2011	7	1	2700	124.96	19.23
23420135	2011	7	1	2710	122.85	19.25
23420135	2011	7	1	2720	121.72	19.26
23420135	2011	7	1	2730	120.33	19.28
23420135	2011	7	1	2740	117.84	19.31
23420135	2011	7	1	2750	115.16	19.35
23420135	2011	7	1	2760	113.71	19.38
23420135	2011	7	1	2770	111.63	19.42
23420135	2011	7	1	2780	109.89	19.44
23420135	2011	7	1	2790	107.24	19.52
23420135	2011	7	1	2800	105.09	19.56
23420135	2011	7	1	2810	102.71	19.66
23420135	2011	7	1	2820	100.09	19.72
23420135	2011	7	1	2830	97.16	19.78
23420135	2011	7	1	2840	94.98	19.89
23420135	2011	7	1	2850	92	20.05
23420135	2011	7	1	2860	89.74	20.12
23420135	2011	7	1	2870	86.94	20.3
23420135	2011	7	1	2880	84.5	20.38
23420135	2011	7	1	2890	83	20.51
23420135	2011	7	1	2900	80.86	20.64
23420135	2011	7	1	2910	78	20.75

23420135	2011	7	1	2920	74.99	20.89
23420135	2011	7	1	2930	72.42	20.96
23420135	2011	7	1	2940	69.92	21.03
23420135	2011	7	1	2950	68.64	21.14
23420135	2011	7	1	2960	68.48	21.32
23420135	2011	7	1	2970	67.55	21.45
23420135	2011	7	1	2980	67.04	21.55
23420135	2011	7	1	2990	65.9	21.67
23420135	2011	7	1	3000	65.26	21.76
23420135	2011	7	1	3010	64.53	21.85
23420135	2011	7	1	3020	63.71	21.96
23420135	2011	7	1	3030	63.66	22.09
23420135	2011	7	1	3040	63.07	22.17
23420135	2011	7	1	3050	63.17	22.25
23420135	2011	7	1	3060	63.45	22.32
23420135	2011	7	1	3070	63.75	22.42
23420135	2011	7	1	3080	63.02	22.53
23420135	2011	7	1	3090	62.83	22.64
23420135	2011	7	1	3100	61.66	22.76
23420135	2011	7	1	3110	61.17	22.85
23420135	2011	7	1	3120	60.11	22.95
23420135	2011	7	1	3130	59.89	23.01
23420135	2011	7	1	3140	59.77	23.07
23420135	2011	7	1	3150	59.57	23.17
23420135	2011	7	1	3160	58.82	23.25
23420135	2011	7	1	3170	57.85	23.31
23420135	2011	7	1	3180	56.74	23.36
23420135	2011	7	1	3190	55.94	23.42
23420135	2011	7	1	3200	54.98	23.46
23420135	2011	7	1	3210	54.3	23.5
23420135	2011	7	1	3220	54.08	23.56
23420135	2011	7	1	3230	53.9	23.61
23420135	2011	7	1	3240	51.91	23.71
23420135	2011	7	1	3250	49.11	23.81
23420135	2011	7	1	3260	46.03	23.88
23420135	2011	7	1	3270	43.78	23.96
23420135	2011	7	1	3280	41.71	24.08
23420135	2011	7	1	3290	40.28	24.12
23420135	2011	7	1	3300	38.26	24.25
23420135	2011	7	1	3310	35.52	24.32
23420135	2011	7	1	3320	32.64	24.42
23420135	2011	7	1	3330	29.83	24.52
23420135	2011	7	1	3340	26.94	24.6
23420135	2011	7	1	3350	24.28	24.71
23420135	2011	7	1	3360	22	24.92
23420135	2011	7	1	3370	19.95	25.09
23420135	2011	7	1	3380	17.67	25.22

23420135	2011	7	1	3390	16.18	25.39
23420135	2011	7	1	3400	15.33	25.73
23420135	2011	7	1	3410	13.18	25.81
23420135	2011	7	1	3420	10.87	26.04
23420135	2011	7	1	3430	9.03	26.12
23420135	2011	7	1	3440	6.93	26.26
23420135	2011	7	1	3450	4.13	26.36
23420135	2011	7	1	3460	2.51	26.46
23420135	2011	7	1	3470	2.42	26.56
23420135	2011	7	1	3480	2.5	26.72
23420135	2011	7	1	3490	2.37	26.77
23420135	2011	7	1	3500	1.76	26.85
23420135	2011	7	1	3510	1.54	26.92
23420135	2011	7	1	3520	1.33	27.01
23420135	2011	7	1	3530	0.62	27.14
23420135	2011	7	1	3540	0.59	27.17
23420135	2011	7	1	3550	0.57	27.2
23420135	2011	7	1	3560	0.58	27.25
23420135	2011	7	1	3570	0.57	27.4
23420135	2011	7	1	3580	0.56	27.45
23420135	2011	7	1	3590	0.58	27.49
23420135	2011	7	1	3600	0.59	27.53
23420135	2011	7	1	3610	0.49	27.6
23420135	2011	7	1	3620	0.48	27.66
23420135	2011	7	1	3630	0.48	27.69
23420135	2011	7	1	3640	0.47	27.74
23420135	2011	7	1	3650	0.48	27.77
23420135	2011	7	1	3660	0.48	27.82
23420135	2011	7	1	3670	0.5	27.85
23482744	2011	7	2	0	1.01	28.52
23482744	2011	7	2	10	1.45	28.51
23482744	2011	7	2	20	2.49	28.51
23482744	2011	7	2	30	2.81	28.5
23482744	2011	7	2	40	3.08	28.5
23482744	2011	7	2	50	3.07	28.5
23482744	2011	7	2	60	2.7	28.5
23482744	2011	7	2	70	2.32	28.51
23482744	2011	7	2	80	2.46	28.5
23482744	2011	7	2	90	2.75	28.51
23482744	2011	7	2	100	4.57	28.49
23482744	2011	7	2	110	5.9	28.48
23482744	2011	7	2	120	7.92	28.46
23482744	2011	7	2	130	9.94	28.43
23482744	2011	7	2	140	12.34	28.41
23482744	2011	7	2	150	14.66	28.39
23482744	2011	7	2	160	17.27	28.35
23482744	2011	7	2	170	19.29	28.33

23482744	2011	7	2	180	20.53	28.32
23482744	2011	7	2	190	21.1	28.32
23482744	2011	7	2	200	21.32	28.32
23482744	2011	7	2	210	22.89	28.3
23482744	2011	7	2	220	24.47	28.28
23482744	2011	7	2	230	26.88	28.26
23482744	2011	7	2	240	29.16	28.24
23482744	2011	7	2	250	31.23	28.18
23482744	2011	7	2	260	32.76	28.1
23482744	2011	7	2	270	33.35	28.05
23482744	2011	7	2	280	35.24	27.99
23482744	2011	7	2	290	36.19	27.92
23482744	2011	7	2	300	37.76	27.84
23482744	2011	7	2	310	38.98	27.77
23482744	2011	7	2	320	41.02	27.67
23482744	2011	7	2	330	42.02	27.6
23482744	2011	7	2	340	42.47	27.58
23482744	2011	7	2	350	43.52	27.53
23482744	2011	7	2	360	45.49	27.44
23482744	2011	7	2	370	47.66	27.35
23482744	2011	7	2	380	49.78	27.25
23482744	2011	7	2	390	51.13	27.23
23482744	2011	7	2	400	52.35	27.21
23482744	2011	7	2	410	53.01	27.17
23482744	2011	7	2	420	54.49	26.99
23482744	2011	7	2	430	56.56	26.95
23482744	2011	7	2	440	58.01	26.95
23482744	2011	7	2	450	58.59	26.92
23482744	2011	7	2	460	60.19	26.81
23482744	2011	7	2	470	62.51	26.64
23482744	2011	7	2	480	64.02	26.52
23482744	2011	7	2	490	66.04	26.45
23482744	2011	7	2	500	67.51	26.37
23482744	2011	7	2	510	69.18	26.25
23482744	2011	7	2	520	70.53	26.14
23482744	2011	7	2	530	71.28	26.09
23482744	2011	7	2	540	71.74	26.02
23482744	2011	7	2	550	73.42	25.94
23482744	2011	7	2	560	76.05	25.85
23482744	2011	7	2	570	78.41	25.74
23482744	2011	7	2	580	79.7	25.67
23482744	2011	7	2	590	81.59	25.57
23482744	2011	7	2	600	83.53	25.46
23482744	2011	7	2	610	85.87	25.36
23482744	2011	7	2	620	87.28	25.28
23482744	2011	7	2	630	88.64	25.15
23482744	2011	7	2	640	89.27	25.07

23482744	2011	7	2	650	89.67	25.01
23482744	2011	7	2	660	90	24.95
23482744	2011	7	2	670	90.04	24.86
23482744	2011	7	2	680	90.21	24.78
23482744	2011	7	2	690	90.89	24.67
23482744	2011	7	2	700	91.93	24.5
23482744	2011	7	2	710	93.4	24.35
23482744	2011	7	2	720	94.34	24.28
23482744	2011	7	2	730	96.05	24.14
23482744	2011	7	2	740	97.47	24.03
23482744	2011	7	2	750	99.26	23.85
23482744	2011	7	2	760	100.98	23.6
23482744	2011	7	2	770	102.1	23.52
23482744	2011	7	2	780	102.41	23.42
23482744	2011	7	2	790	102.51	23.35
23482744	2011	7	2	800	102.44	23.28
23482744	2011	7	2	810	102.29	23.17
23482744	2011	7	2	820	102.07	23.07
23482744	2011	7	2	830	101.71	22.98
23482744	2011	7	2	840	102.21	22.89
23482744	2011	7	2	850	102.58	22.78
23482744	2011	7	2	860	102.39	22.64
23482744	2011	7	2	870	102.17	22.57
23482744	2011	7	2	880	102.69	22.47
23482744	2011	7	2	890	102.59	22.37
23482744	2011	7	2	900	101.99	22.28
23482744	2011	7	2	910	102.53	22.22
23482744	2011	7	2	920	103.11	22.15
23482744	2011	7	2	930	103.13	22.12
23482744	2011	7	2	940	102.45	22.07
23482744	2011	7	2	950	102.49	22.01
23482744	2011	7	2	960	102.47	21.96
23482744	2011	7	2	970	102.38	21.89
23482744	2011	7	2	980	104.07	21.82
23482744	2011	7	2	990	105.19	21.74
23482744	2011	7	2	1000	106.04	21.72
23482744	2011	7	2	1010	107.49	21.62
23482744	2011	7	2	1020	109.87	21.54
23482744	2011	7	2	1030	111.33	21.51
23482744	2011	7	2	1040	112.58	21.47
23482744	2011	7	2	1050	114.39	21.39
23482744	2011	7	2	1060	116.25	21.3
23482744	2011	7	2	1070	118.08	21.17
23482744	2011	7	2	1080	120.14	21.06
23482744	2011	7	2	1090	121.97	20.96
23482744	2011	7	2	1100	124.72	20.93
23482744	2011	7	2	1110	127.83	20.85

23482744	2011	7	2	1120	130.6	20.76
23482744	2011	7	2	1130	133.06	20.69
23482744	2011	7	2	1140	135.1	20.64
23482744	2011	7	2	1150	135.95	20.58
23482744	2011	7	2	1160	136	20.47
23482744	2011	7	2	1170	134.74	20.38
23482744	2011	7	2	1180	132.74	20.31
23482744	2011	7	2	1190	132	20.19
23482744	2011	7	2	1200	131.83	20.07
23482744	2011	7	2	1210	131.74	19.98
23482744	2011	7	2	1220	131.78	19.9
23482744	2011	7	2	1230	131.77	19.83
23482744	2011	7	2	1240	131.63	19.77
23482744	2011	7	2	1250	131.63	19.72
23482744	2011	7	2	1260	131.73	19.67
23482744	2011	7	2	1270	131.78	19.66
23482744	2011	7	2	1280	131.65	19.64
23482744	2011	7	2	1290	131.66	19.6
23482744	2011	7	2	1300	131.68	19.58
23482744	2011	7	2	1310	131.68	19.57
23482744	2011	7	2	1320	131.63	19.56
23482744	2011	7	2	1330	131.68	19.54
23482744	2011	7	2	1340	131.69	19.5
23482744	2011	7	2	1350	130.72	19.49
23482744	2011	7	2	1360	130.43	19.46
23482744	2011	7	2	1370	130.18	19.45
23482744	2011	7	2	1380	129.95	19.42
23482744	2011	7	2	1390	130.2	19.41
23482744	2011	7	2	1400	130.26	19.39
23482744	2011	7	2	1410	130.43	19.36
23482744	2011	7	2	1420	130.69	19.34
23482744	2011	7	2	1430	130.59	19.32
23482744	2011	7	2	1440	130.49	19.32
23482744	2011	7	2	1450	130.44	19.3
23482744	2011	7	2	1460	130.57	19.28
23482744	2011	7	2	1470	130.5	19.28
23482744	2011	7	2	1480	130.1	19.27
23482744	2011	7	2	1490	130.02	19.27
23482744	2011	7	2	1500	129.97	19.25
23482744	2011	7	2	1510	129.37	19.25
23482744	2011	7	2	1520	129.19	19.23
23482744	2011	7	2	1530	130.32	19.2
23482744	2011	7	2	1540	131.51	19.18
23482744	2011	7	2	1550	132.75	19.17
23482744	2011	7	2	1560	134.15	19.16
23482744	2011	7	2	1570	134.98	19.15
23482744	2011	7	2	1580	134.66	19.14

23482744	2011	7	2	1590	134.47	19.13
23482744	2011	7	2	1600	134.63	19.11
23482744	2011	7	2	1610	134.46	19.1
23482744	2011	7	2	1620	134.08	19.1
23482744	2011	7	2	1630	134.02	19.07
23482744	2011	7	2	1640	134.49	19.05
23482744	2011	7	2	1650	134.64	19.01
23482744	2011	7	2	1660	135.54	18.96
23482744	2011	7	2	1670	135.86	18.92
23482744	2011	7	2	1680	135.99	18.91
23482744	2011	7	2	1690	136.19	18.87
23482744	2011	7	2	1700	136.08	18.85
23482744	2011	7	2	1710	135.9	18.82
23482744	2011	7	2	1720	135.66	18.8
23482744	2011	7	2	1730	135.43	18.77
23482744	2011	7	2	1740	135.3	18.75
23482744	2011	7	2	1750	135.2	18.73
23482744	2011	7	2	1760	135.32	18.71
23482744	2011	7	2	1770	135.76	18.68
23482744	2011	7	2	1780	136.18	18.67
23482744	2011	7	2	1790	136.63	18.66
23482744	2011	7	2	1800	137.56	18.64
23482744	2011	7	2	1810	138.62	18.6
23482744	2011	7	2	1820	138.88	18.59
23482744	2011	7	2	1830	138.95	18.56
23482744	2011	7	2	1840	138.85	18.53
23482744	2011	7	2	1850	138.99	18.51
23482744	2011	7	2	1860	139.07	18.46
23482744	2011	7	2	1870	138.69	18.45
23482744	2011	7	2	1880	138.41	18.42
23482744	2011	7	2	1890	138.96	18.39
23482744	2011	7	2	1900	139.48	18.37
23482744	2011	7	2	1910	139.85	18.35
23482744	2011	7	2	1920	140.03	18.33
23482744	2011	7	2	1930	140.3	18.3
23482744	2011	7	2	1940	139.98	18.28
23482744	2011	7	2	1950	139.6	18.27
23482744	2011	7	2	1960	139.08	18.25
23482744	2011	7	2	1970	138.98	18.25
23482744	2011	7	2	1980	138.83	18.22
23482744	2011	7	2	1990	138.69	18.22
23482744	2011	7	2	2000	138.76	18.21
23482744	2011	7	2	2010	139.08	18.19
23482744	2011	7	2	2020	139.42	18.17
23482744	2011	7	2	2030	140.06	18.17
23482744	2011	7	2	2040	140.7	18.16
23482744	2011	7	2	2050	141.4	18.15

23482744	2011	7	2	2060	142.67	18.14
23482744	2011	7	2	2070	144.13	18.11
23482744	2011	7	2	2080	145.02	18.1
23482744	2011	7	2	2090	145.77	18.09
23482744	2011	7	2	2100	146.4	18.08
23482744	2011	7	2	2110	146.81	18.07
23482744	2011	7	2	2120	147.85	18.03
23482744	2011	7	2	2130	149.59	17.98
23482744	2011	7	2	2140	150.42	17.93
23482744	2011	7	2	2150	150.72	17.92
23482744	2011	7	2	2160	151.19	17.89
23482744	2011	7	2	2170	151.88	17.86
23482744	2011	7	2	2180	153.61	17.83
23482744	2011	7	2	2190	155.16	17.78
23482744	2011	7	2	2200	157.06	17.74
23482744	2011	7	2	2210	157.69	17.71
23482744	2011	7	2	2220	158.08	17.68
23482744	2011	7	2	2230	159.04	17.64
23482744	2011	7	2	2240	159.35	17.62
23482744	2011	7	2	2250	159.54	17.61
23482744	2011	7	2	2260	160.9	17.58
23482744	2011	7	2	2270	162.56	17.53
23482744	2011	7	2	2280	163.53	17.5
23482744	2011	7	2	2290	164.64	17.46
23482744	2011	7	2	2300	164.66	17.45
23482744	2011	7	2	2310	164.53	17.43
23482744	2011	7	2	2320	165.02	17.4
23482744	2011	7	2	2330	166.43	17.32
23482744	2011	7	2	2340	166.84	17.29
23482744	2011	7	2	2350	166.97	17.28
23482744	2011	7	2	2360	167.83	17.23
23482744	2011	7	2	2370	170.19	17.14
23482744	2011	7	2	2380	171.9	17.1
23482744	2011	7	2	2390	172.98	17.04
23482744	2011	7	2	2400	174.21	17.02
23482744	2011	7	2	2410	174.96	16.92
23482744	2011	7	2	2420	175.47	16.85
23482744	2011	7	2	2430	176.16	16.79
23482744	2011	7	2	2440	176.66	16.77
23482744	2011	7	2	2450	177.07	16.74
23482744	2011	7	2	2460	177.31	16.71
23482744	2011	7	2	2470	177.6	16.67
23482744	2011	7	2	2480	177.62	16.64
23482744	2011	7	2	2490	177.52	16.61
23482744	2011	7	2	2500	177.38	16.59
23482744	2011	7	2	2510	177.52	16.55
23482744	2011	7	2	2520	177.71	16.52

23482744	2011	7	2	2530	177.77	16.49
23482744	2011	7	2	2540	177.67	16.46
23482744	2011	7	2	2550	177.44	16.43
23482744	2011	7	2	2560	177.46	16.39
23482744	2011	7	2	2570	177.57	16.35
23482744	2011	7	2	2580	177.51	16.34
23482744	2011	7	2	2590	177.33	16.32
23482744	2011	7	2	2600	177.2	16.3
23482744	2011	7	2	2610	177.14	16.28
23482744	2011	7	2	2620	177.39	16.27
23482744	2011	7	2	2630	177.74	16.26
23482744	2011	7	2	2640	178.08	16.23
23482744	2011	7	2	2650	178.32	16.21
23482744	2011	7	2	2660	178.86	16.19
23482744	2011	7	2	2670	179.14	16.17
23482744	2011	7	2	2680	179.11	16.16
23482744	2011	7	2	2690	178.89	16.14
23482744	2011	7	2	2700	178.68	16.14
23482744	2011	7	2	2710	178.6	16.12
23482744	2011	7	2	2720	178.48	16.11
23482744	2011	7	2	2730	178.38	16.1
23482744	2011	7	2	2740	178.34	16.08
23482744	2011	7	2	2750	178.65	16.07
23482744	2011	7	2	2760	178.28	16.04
23482744	2011	7	2	2770	177.78	16.03
23482744	2011	7	2	2780	176.83	16.02
23482744	2011	7	2	2790	176.11	16.01
23482744	2011	7	2	2800	175.72	16
23482744	2011	7	2	2810	175.48	16
23482744	2011	7	2	2820	175.43	15.99
23482744	2011	7	2	2830	175.34	15.98
23482744	2011	7	2	2840	175.33	15.97
23482744	2011	7	2	2850	175.57	15.96
23482744	2011	7	2	2860	175.51	15.96
23482744	2011	7	2	2870	175.42	15.94
23482744	2011	7	2	2880	175.31	15.94
23482744	2011	7	2	2890	175.16	15.94
23482744	2011	7	2	2900	175.36	15.89
23482744	2011	7	2	2910	175.25	15.93
23482744	2011	7	2	2920	175.06	15.89
23482744	2011	7	2	2930	174.88	15.89
23482744	2011	7	2	2940	174.83	15.89
23482744	2011	7	2	2950	174.9	15.88
23482744	2011	7	2	2960	174.81	15.88
23482744	2011	7	2	2970	174.74	15.88
23482744	2011	7	2	2980	174.65	15.87
23482744	2011	7	2	2990	175.02	15.86

23482744	2011	7	2	3000	175.35	15.87
23482744	2011	7	2	3010	175.39	15.85
23482744	2011	7	2	3020	175.37	15.86
23482744	2011	7	2	3030	175.26	15.85
23482744	2011	7	2	3040	175.38	15.85
23482744	2011	7	2	3050	175.3	15.85
23482744	2011	7	2	3060	175.22	15.85
23482744	2011	7	2	3070	175.21	15.84
23482744	2011	7	2	3080	175.22	15.84
23482744	2011	7	2	3090	175.18	15.85
23482744	2011	7	2	3100	175.04	15.83
23482744	2011	7	2	3110	174.97	15.84
23482744	2011	7	2	3120	175.07	15.85
23482744	2011	7	2	3130	175.17	15.85
23482744	2011	7	2	3140	175.29	15.85
23482744	2011	7	2	3150	175.57	15.85
23482744	2011	7	2	3160	175.74	15.86
23482744	2011	7	2	3170	175.91	15.87
23482744	2011	7	2	3180	175.53	15.87
23482744	2011	7	2	3190	175.03	15.88
23482744	2011	7	2	3200	173.86	15.89
23482744	2011	7	2	3210	173.8	15.89
23482744	2011	7	2	3220	174.39	15.89
23482744	2011	7	2	3230	174.64	15.93
23482744	2011	7	2	3240	174.94	15.95
23482744	2011	7	2	3250	175.55	15.96
23482744	2011	7	2	3260	175.83	15.97
23482744	2011	7	2	3270	175.83	15.97
23482744	2011	7	2	3280	175.75	15.97
23482744	2011	7	2	3290	175.75	15.97
23482744	2011	7	2	3300	175.82	15.97
23482744	2011	7	2	3310	175.84	15.96
23482744	2011	7	2	3320	175.86	15.97
23482744	2011	7	2	3330	175.77	15.97
23482744	2011	7	2	3340	175.84	15.97
23482744	2011	7	2	3350	175.82	15.97
23482744	2011	7	2	3360	175.84	15.97
23482744	2011	7	2	3370	175.77	15.97
23482744	2011	7	2	3380	175.88	15.96
23482744	2011	7	2	3390	175.79	15.96
23482744	2011	7	2	3400	175.85	15.97
23482744	2011	7	2	3410	175.71	15.96
23482744	2011	7	2	3420	175.78	15.96
23482744	2011	7	2	3430	175.78	15.96
23482744	2011	7	2	3440	175.77	15.96
23482744	2011	7	2	3450	175.93	15.96
23482744	2011	7	2	3460	175.83	15.96

23482744	2011	7	2	3470	175.92	15.97
23482744	2011	7	2	3480	175.87	15.97
23482744	2011	7	2	3490	175.97	15.97
23482744	2011	7	2	3500	175.87	15.98
23482744	2011	7	2	3510	175.83	15.98
23482744	2011	7	2	3520	175.77	15.99
23482744	2011	7	2	3530	175.65	15.99
23482744	2011	7	2	3540	175.65	16
23482744	2011	7	2	3550	175.55	16
23482744	2011	7	2	3560	175.49	16.01
23482744	2011	7	2	3570	175.8	16.01
23482744	2011	7	2	3580	175.93	16.02
23482744	2011	7	2	3590	175.81	16.03
23482744	2011	7	2	3600	175.52	16.03
23482744	2011	7	2	3610	175.06	16.03
23482744	2011	7	2	3620	175.24	16.04
23482744	2011	7	2	3630	175.79	16.05
23482744	2011	7	2	3640	175.9	16.07
23482744	2011	7	2	3650	175.86	16.07
23482744	2011	7	2	3660	175.91	16.07
23482744	2011	7	2	3670	175.79	16.07
23482744	2011	7	2	3680	175.69	16.08
23482744	2011	7	2	3690	175.8	16.08
23482744	2011	7	2	3700	175.79	16.09
23482744	2011	7	2	3710	175.81	16.09
23482744	2011	7	2	3720	175.76	16.1
23482744	2011	7	2	3730	175.72	16.1
23482744	2011	7	2	3740	175.7	16.11
23482744	2011	7	2	3750	175.68	16.11
23482744	2011	7	2	3760	175.66	16.12
23482744	2011	7	2	3770	175.73	16.13
23482744	2011	7	2	3780	175.49	16.13
23482744	2011	7	2	3790	175.23	16.13
23482744	2011	7	2	3800	175.71	16.13
23482744	2011	7	2	3810	175.91	16.13
23482744	2011	7	2	3820	176.02	16.13
23482744	2011	7	2	3830	176.05	16.14
23482744	2011	7	2	3840	175.97	16.13
23482744	2011	7	2	3850	175.7	16.14
23482744	2011	7	2	3860	175.41	16.13
23482744	2011	7	2	3870	175.18	16.12
23482744	2011	7	2	3880	175.6	16.12
23482744	2011	7	2	3890	175.76	16.12
23482744	2011	7	2	3900	175.78	16.12
23482744	2011	7	2	3910	175.79	16.11
23482744	2011	7	2	3920	175.59	16.11
23482744	2011	7	2	3930	175.79	16.12

23482744	2011	7	2	3940	175.8	16.12
23482744	2011	7	2	3950	176.31	16.11
23482744	2011	7	2	3960	176.83	16.11
23482744	2011	7	2	3970	177.1	16.11
23482744	2011	7	2	3980	177.1	16.11
23482744	2011	7	2	3990	177.05	16.11
23482744	2011	7	2	4000	177.22	16.11
23482744	2011	7	2	4010	177.13	16.11
23482744	2011	7	2	4020	177.21	16.1
23482744	2011	7	2	4030	177.05	16.1
23482744	2011	7	2	4040	176.73	16.1
23482744	2011	7	2	4050	176.42	16.1
23482744	2011	7	2	4060	176.32	16.1
23482744	2011	7	2	4070	176.33	16.09
23482744	2011	7	2	4080	176.03	16.1
23482744	2011	7	2	4090	176.28	16.09
23482744	2011	7	2	4100	176.54	16.08
23482744	2011	7	2	4110	176.43	16.07
23482744	2011	7	2	4120	176.43	16.07
23482744	2011	7	2	4130	176.35	16.07
23482744	2011	7	2	4140	176.75	16.07
23482744	2011	7	2	4150	176.59	16.04
23482744	2011	7	2	4160	176.74	16.03
23482744	2011	7	2	4170	177.11	16.03
23482744	2011	7	2	4180	177.41	16.03
23482744	2011	7	2	4190	178.21	16.02
23482744	2011	7	2	4200	178.42	16.01
23482744	2011	7	2	4210	178.43	16.01
23482744	2011	7	2	4220	178.25	16
23482744	2011	7	2	4230	178.45	16
23482744	2011	7	2	4240	178.27	15.99
23482744	2011	7	2	4250	178.13	15.98
23482744	2011	7	2	4260	178.55	15.97
23482744	2011	7	2	4270	178.78	15.96
23482744	2011	7	2	4280	179.99	15.96
23482744	2011	7	2	4290	180.64	15.95
23482744	2011	7	2	4300	181.11	15.95
23482744	2011	7	2	4310	181.5	15.94
23482744	2011	7	2	4320	181.54	15.93
23482744	2011	7	2	4330	181.73	15.93
23482744	2011	7	2	4340	181.66	15.89
23482744	2011	7	2	4350	181.56	15.89
23482744	2011	7	2	4360	181.79	15.89
23482744	2011	7	2	4370	181.7	15.87
23482744	2011	7	2	4380	181.95	15.87
23482744	2011	7	2	4390	181.89	15.86
23482744	2011	7	2	4400	181.9	15.85

23482744	2011	7	2	4410	181.97	15.86
23482744	2011	7	2	4420	181.81	15.85
23482744	2011	7	2	4430	181.73	15.85
23482744	2011	7	2	4440	181.88	15.85
23482744	2011	7	2	4450	181.84	15.84
23482744	2011	7	2	4460	181.81	15.84
23482744	2011	7	2	4470	181.89	15.83
23482744	2011	7	2	4480	181.72	15.84
23482744	2011	7	2	4490	181.96	15.82
23482744	2011	7	2	4500	181.87	15.82
23482744	2011	7	2	4510	181.92	15.82
23482744	2011	7	2	4520	181.95	15.82
23482744	2011	7	2	4530	181.83	15.82
23482744	2011	7	2	4540	181.73	15.82
23482744	2011	7	2	4550	181.64	15.82
23482744	2011	7	2	4560	181.67	15.81
23482744	2011	7	2	4570	181.94	15.81
23482744	2011	7	2	4580	181.88	15.81
23482744	2011	7	2	4590	181.87	15.8
23482744	2011	7	2	4600	181.87	15.8
23482744	2011	7	2	4610	181.97	15.81
23482744	2011	7	2	4620	181.89	15.8
23482744	2011	7	2	4630	181.98	15.8
23482744	2011	7	2	4640	181.74	15.79
23482744	2011	7	2	4650	181.49	15.79
23482744	2011	7	2	4660	180.86	15.79
23482744	2011	7	2	4670	180.96	15.78
23482744	2011	7	2	4680	181.79	15.78
23482744	2011	7	2	4690	181.81	15.79
23482744	2011	7	2	4700	181.76	15.78
23482744	2011	7	2	4710	181.95	15.79
23482744	2011	7	2	4720	181.84	15.78
23482744	2011	7	2	4730	182.15	15.78
23482744	2011	7	2	4740	181.83	15.78
23482744	2011	7	2	4750	182.12	15.78
23482744	2011	7	2	4760	181.87	15.78
23482744	2011	7	2	4770	181.51	15.78
23482744	2011	7	2	4780	181.15	15.78
23482744	2011	7	2	4790	180.54	15.78
23482744	2011	7	2	4800	180.61	15.78
23482744	2011	7	2	4810	180.61	15.78
23482744	2011	7	2	4820	180.54	15.78
23482744	2011	7	2	4830	180.54	15.78
23482744	2011	7	2	4840	180.54	15.78
23482744	2011	7	2	4850	180.48	15.78
23482744	2011	7	2	4860	180.34	15.78
23482744	2011	7	2	4870	180.21	15.78

23482744	2011	7	2	4880	180.19	15.78
23482744	2011	7	2	4890	180.08	15.78
23482744	2011	7	2	4900	179.91	15.79
23482744	2011	7	2	4910	179.76	15.79
23482744	2011	7	2	4920	179.52	15.79
23482744	2011	7	2	4930	179.33	15.78
23482744	2011	7	2	4940	179.8	15.79
23482744	2011	7	2	4950	180.17	15.79
23482744	2011	7	2	4960	180.26	15.78
23482744	2011	7	2	4970	180.41	15.79
23482744	2011	7	2	4980	180.58	15.78
23482744	2011	7	2	4990	181.37	15.78
23482744	2011	7	2	5000	181.67	15.78
23482744	2011	7	2	5010	181.93	15.78
23482744	2011	7	2	5020	182.26	15.78
23482744	2011	7	2	5030	182.38	15.78
23482744	2011	7	2	5040	183.91	15.78
23482744	2011	7	2	5050	184.36	15.77
23482744	2011	7	2	5060	184.6	15.78
23482744	2011	7	2	5070	185.6	15.76
23482744	2011	7	2	5080	186.1	15.75
23482744	2011	7	2	5090	186.41	15.75
23482744	2011	7	2	5100	186.53	15.74
23482744	2011	7	2	5110	186.35	15.73
23482744	2011	7	2	5120	186.15	15.73
23482744	2011	7	2	5130	186.32	15.71
23482744	2011	7	2	5140	186.26	15.72
23482744	2011	7	2	5150	186.28	15.71
23482744	2011	7	2	5160	185.74	15.71
23482744	2011	7	2	5170	185.47	15.71
23482744	2011	7	2	5180	185.35	15.69
23482744	2011	7	2	5190	185.41	15.7
23482744	2011	7	2	5200	186.03	15.68
23482744	2011	7	2	5210	186.16	15.68
23482744	2011	7	2	5220	186.53	15.67
23482744	2011	7	2	5230	186.87	15.67
23482744	2011	7	2	5240	188.17	15.67
23482744	2011	7	2	5250	189.08	15.65
23482744	2011	7	2	5260	189.97	15.64
23482744	2011	7	2	5270	190.84	15.63
23482744	2011	7	2	5280	191.26	15.61
23482744	2011	7	2	5290	191.48	15.6
23482744	2011	7	2	5300	192.33	15.6
23482744	2011	7	2	5310	193.19	15.59
23482744	2011	7	2	5320	193.98	15.58
23482744	2011	7	2	5330	194.64	15.57
23482744	2011	7	2	5340	195.35	15.57

23482744	2011	7	2	5350	195.88	15.56
23482744	2011	7	2	5360	195.89	15.55
23482744	2011	7	2	5370	195.7	15.53
23482744	2011	7	2	5380	195.7	15.53
23482744	2011	7	2	5390	195.69	15.52
23482744	2011	7	2	5400	195.73	15.51
23482744	2011	7	2	5410	195.59	15.5
23482744	2011	7	2	5420	195.41	15.5
23482744	2011	7	2	5430	195.19	15.48
23482744	2011	7	2	5440	195.66	15.47
23482744	2011	7	2	5450	196.31	15.46
23482744	2011	7	2	5460	196.93	15.45
23482744	2011	7	2	5470	198.03	15.45
23482744	2011	7	2	5480	198.6	15.43
23482744	2011	7	2	5490	198.74	15.42
23482744	2011	7	2	5500	199.67	15.41
23482744	2011	7	2	5510	200.1	15.4
23482744	2011	7	2	5520	200.57	15.4
23482744	2011	7	2	5530	200.57	15.39
23482744	2011	7	2	5540	200.48	15.39
23482744	2011	7	2	5550	200.57	15.38
23482744	2011	7	2	5560	200.35	15.37
23482744	2011	7	2	5570	199.48	15.37
23482744	2011	7	2	5580	199.36	15.36
23482744	2011	7	2	5590	199.5	15.35
23482744	2011	7	2	5600	200.04	15.35
23482744	2011	7	2	5610	200.39	15.35
23482744	2011	7	2	5620	200.44	15.34
23482744	2011	7	2	5630	200.73	15.34
23482744	2011	7	2	5640	201.41	15.33
23482744	2011	7	2	5650	201.99	15.33
23482744	2011	7	2	5660	202.42	15.33
23482744	2011	7	2	5670	204.75	15.32
23482744	2011	7	2	5680	205.64	15.32
23482744	2011	7	2	5690	205.67	15.32
23482744	2011	7	2	5700	205.81	15.32
23482744	2011	7	2	5710	205.79	15.3
23482744	2011	7	2	5720	205.84	15.3
23482744	2011	7	2	5730	206.02	15.29
23482744	2011	7	2	5740	206	15.29
23482744	2011	7	2	5750	206	15.28
23482744	2011	7	2	5760	206.07	15.28
23482744	2011	7	2	5770	205.98	15.28
23482744	2011	7	2	5780	205.95	15.28
23482744	2011	7	2	5790	206.1	15.28
23482744	2011	7	2	5800	205.92	15.26
23482744	2011	7	2	5810	205.96	15.27

23482744	2011	7	2	5820	206.09	15.27
23482744	2011	7	2	5830	206.08	15.26
23482744	2011	7	2	5840	206.1	15.26
23482744	2011	7	2	5850	205.41	15.26
23482744	2011	7	2	5860	205.89	15.25
23482744	2011	7	2	5870	204.72	15.25
23482744	2011	7	2	5880	203.63	15.25
23482744	2011	7	2	5890	204.1	15.25
23482744	2011	7	2	5900	205.26	15.25
23482744	2011	7	2	5910	206.3	15.26
23482744	2011	7	2	5920	206.72	15.25
23482744	2011	7	2	5930	206.67	15.26
23482744	2011	7	2	5940	206.71	15.26
23482744	2011	7	2	5950	206.43	15.26
23482744	2011	7	2	5960	206.47	15.26
23482744	2011	7	2	5970	206.76	15.26
23482744	2011	7	2	5980	206.9	15.26
23482744	2011	7	2	5990	206.83	15.26
23482744	2011	7	2	6000	206.9	15.26
23482744	2011	7	2	6010	206.98	15.27
23482744	2011	7	2	6020	207.06	15.27
23482744	2011	7	2	6030	206.14	15.26
23482744	2011	7	2	6040	205.88	15.26
23482744	2011	7	2	6050	206.41	15.27
23482744	2011	7	2	6060	207.07	15.26
23482744	2011	7	2	6070	207.45	15.26
23482744	2011	7	2	6080	207.47	15.26
23482744	2011	7	2	6090	207.61	15.27
23482744	2011	7	2	6100	208.46	15.25
23482744	2011	7	2	6110	209.18	15.26
23482744	2011	7	2	6120	209.4	15.26
23482744	2011	7	2	6130	209.54	15.26
23482744	2011	7	2	6140	209.93	15.25
23482744	2011	7	2	6150	210.84	15.26
23482744	2011	7	2	6160	210.82	15.26
23482744	2011	7	2	6170	210.74	15.25
23482744	2011	7	2	6180	210.54	15.25
23482744	2011	7	2	6190	210.22	15.25
23482744	2011	7	2	6200	209.91	15.25
23482744	2011	7	2	6210	210.8	15.25
23482744	2011	7	2	6220	210.79	15.25
23482744	2011	7	2	6230	211.01	15.23
23482744	2011	7	2	6240	210.89	15.25
23482744	2011	7	2	6250	210.6	15.24
23482744	2011	7	2	6260	210.59	15.23
23482744	2011	7	2	6270	210.05	15.24
23482744	2011	7	2	6280	210.22	15.24

23482744	2011	7	2	6290	210.58	15.24
23482744	2011	7	2	6300	210.91	15.24
23482744	2011	7	2	6310	211.32	15.24
23482744	2011	7	2	6320	211.67	15.23
23482744	2011	7	2	6330	211.53	15.24
23482744	2011	7	2	6340	211.32	15.23
23482744	2011	7	2	6350	211.21	15.23
23482744	2011	7	2	6360	211.3	15.23
23482744	2011	7	2	6370	211.71	15.24
23482744	2011	7	2	6380	212.03	15.24
23482744	2011	7	2	6390	212.35	15.24
23482744	2011	7	2	6400	212.36	15.24
23482744	2011	7	2	6410	212.37	15.23
23482744	2011	7	2	6420	212.33	15.21
23482744	2011	7	2	6430	211.92	15.21
23482744	2011	7	2	6440	211.48	15.21
23482744	2011	7	2	6450	211.97	15.18
23482744	2011	7	2	6460	212.8	15.19
23482744	2011	7	2	6470	213.1	15.17
23482744	2011	7	2	6480	214.01	15.17
23482744	2011	7	2	6490	215.07	15.15
23482744	2011	7	2	6500	216.6	15.14
23482744	2011	7	2	6510	217.86	15.12
23482744	2011	7	2	6520	218.64	15.1
23482744	2011	7	2	6530	219.1	15.07
23482744	2011	7	2	6540	220.1	15.07
23482744	2011	7	2	6550	221.2	15.05
23482744	2011	7	2	6560	221.76	15.03
23482744	2011	7	2	6570	222.02	15.01
23482744	2011	7	2	6580	221.94	14.96
23482744	2011	7	2	6590	221.96	14.93
23482744	2011	7	2	6600	222.5	14.91
23482744	2011	7	2	6610	223.32	14.82
23482744	2011	7	2	6620	223.77	14.79
23482744	2011	7	2	6630	224.03	14.77
23482744	2011	7	2	6640	224.4	14.71
23482744	2011	7	2	6650	224.66	14.67
23482744	2011	7	2	6660	224.89	14.61
23482744	2011	7	2	6670	224.98	14.57
23482744	2011	7	2	6680	225.46	14.53
23482744	2011	7	2	6690	225.74	14.42
23482744	2011	7	2	6700	226.48	14.37
23482744	2011	7	2	6710	226.96	14.33
23482744	2011	7	2	6720	227.29	14.27
23482744	2011	7	2	6730	227.43	14.18
23482744	2011	7	2	6740	227.75	14.11
23482744	2011	7	2	6750	228.15	14.04

23482744	2011	7	2	6760	229.38	14
23482744	2011	7	2	6770	230.37	13.97
23482744	2011	7	2	6780	230.88	13.94
23482744	2011	7	2	6790	231.11	13.87
23482744	2011	7	2	6800	231.15	13.85
23482744	2011	7	2	6810	231.01	13.78
23482744	2011	7	2	6820	230.77	13.76
23482744	2011	7	2	6830	230.81	13.73
23482744	2011	7	2	6840	231.95	13.7
23482744	2011	7	2	6850	233.15	13.67
23482744	2011	7	2	6860	233.75	13.64
23482744	2011	7	2	6870	233.82	13.62
23482744	2011	7	2	6880	233.59	13.59
23482744	2011	7	2	6890	233.4	13.57
23482744	2011	7	2	6900	233.29	13.55
23482744	2011	7	2	6910	233.52	13.53
23482744	2011	7	2	6920	233.94	13.51
23482744	2011	7	2	6930	234.22	13.5
23482744	2011	7	2	6940	234.34	13.46
23482744	2011	7	2	6950	234.88	13.44
23482744	2011	7	2	6960	236.28	13.42
23482744	2011	7	2	6970	236.98	13.37
23482744	2011	7	2	6980	237.24	13.34
23482744	2011	7	2	6990	237.85	13.31
23482744	2011	7	2	7000	239.34	13.28
23482744	2011	7	2	7010	239.79	13.25
23482744	2011	7	2	7020	240.08	13.23
23482744	2011	7	2	7030	240.23	13.21
23482744	2011	7	2	7040	240.45	13.18
23482744	2011	7	2	7050	240.25	13.15
23482744	2011	7	2	7060	240.08	13.14
23482744	2011	7	2	7070	240.51	13.11
23482744	2011	7	2	7080	241.17	13.08
23482744	2011	7	2	7090	241.75	13.07
23482744	2011	7	2	7100	242.16	13.04
23482744	2011	7	2	7110	242.19	13.03
23482744	2011	7	2	7120	242.18	13.01
23482744	2011	7	2	7130	242.19	12.99
23482744	2011	7	2	7140	242.23	12.98
23482744	2011	7	2	7150	242.25	12.97
23482744	2011	7	2	7160	242.25	12.96
23482744	2011	7	2	7170	242.25	12.95
23482744	2011	7	2	7180	242.2	12.93
23482744	2011	7	2	7190	242.18	12.91
23482744	2011	7	2	7200	242.16	12.9
23482744	2011	7	2	7210	242.09	12.89
23482744	2011	7	2	7220	242.07	12.89

23482744	2011	7	2	7230	242.7	12.88
23482744	2011	7	2	7240	242.82	12.88
23482744	2011	7	2	7250	242.88	12.87
23482744	2011	7	2	7260	242.93	12.85
23482744	2011	7	2	7270	242.92	12.84
23482744	2011	7	2	7280	242.87	12.83
23482744	2011	7	2	7290	242.58	12.82
23482744	2011	7	2	7300	242.3	12.82
23482744	2011	7	2	7310	241.78	12.74
23482744	2011	7	2	7320	241.6	12.74
23482744	2011	7	2	7330	241.76	12.74
23482744	2011	7	2	7340	241.74	12.73
23482744	2011	7	2	7350	241.74	12.73
23482744	2011	7	2	7360	241.76	12.73
23482744	2011	7	2	7370	241.35	12.74
23482744	2011	7	2	7380	241.12	12.72
23482744	2011	7	2	7390	241.04	12.72
23482744	2011	7	2	7400	240.71	12.73
23482744	2011	7	2	7410	239.99	12.74
23482744	2011	7	2	7420	239.96	12.73
23482744	2011	7	2	7430	240.81	12.74
23482744	2011	7	2	7440	241.71	12.82
23482744	2011	7	2	7450	241.72	12.83
23482744	2011	7	2	7460	241.45	12.84
23482744	2011	7	2	7470	241.63	12.85
23482744	2011	7	2	7480	242.06	12.87
23482744	2011	7	2	7490	241.84	12.89
23482744	2011	7	2	7500	242.68	12.92
23482744	2011	7	2	7510	244.26	12.94
23482744	2011	7	2	7520	245.62	12.96
23482744	2011	7	2	7530	246.9	12.96
23482744	2011	7	2	7540	247.33	12.97
23482744	2011	7	2	7550	247.37	12.97
23482744	2011	7	2	7560	247.76	12.96
23482744	2011	7	2	7570	247.98	12.97
23482744	2011	7	2	7580	247.89	12.96
23482744	2011	7	2	7590	247.09	12.96
23482744	2011	7	2	7600	246.51	12.95
23482744	2011	7	2	7610	246.38	12.94
23482744	2011	7	2	7620	246.43	12.95
23482744	2011	7	2	7630	246.61	12.94
23482744	2011	7	2	7640	246.69	12.95
23482744	2011	7	2	7650	246.43	12.96
23482744	2011	7	2	7660	246.65	12.96
23482744	2011	7	2	7670	246.62	12.96
23482744	2011	7	2	7680	246.55	12.95
23482744	2011	7	2	7690	246.8	12.95

23482744	2011	7	2	7700	247.16	12.95
23482744	2011	7	2	7710	246.9	12.95
23482744	2011	7	2	7720	247.06	12.94
23482744	2011	7	2	7730	248.07	12.94
23482744	2011	7	2	7740	248.22	12.94
23482744	2011	7	2	7750	248.23	12.93
23482744	2011	7	2	7760	248.22	12.92
23482744	2011	7	2	7770	248.25	12.94
23482744	2011	7	2	7780	248.28	12.92
23482744	2011	7	2	7790	248.26	12.92
23482744	2011	7	2	7800	248.32	12.92
23482744	2011	7	2	7810	248.16	12.92
23482744	2011	7	2	7820	248.24	12.91
23482744	2011	7	2	7830	248.24	12.92
23482744	2011	7	2	7840	248.19	12.9
23482744	2011	7	2	7850	248.19	12.91
23482744	2011	7	2	7860	248.19	12.9
23482744	2011	7	2	7870	248.26	12.9
23482744	2011	7	2	7880	248.23	12.89
23482744	2011	7	2	7890	248.15	12.89
23482744	2011	7	2	7900	248.27	12.89
23482744	2011	7	2	7910	248.18	12.88
23482744	2011	7	2	7920	248.16	12.89
23482744	2011	7	2	7930	248.24	12.88
23482744	2011	7	2	7940	248.42	12.88
23482744	2011	7	2	7950	248.34	12.86
23482744	2011	7	2	7960	248.38	12.86
23482744	2011	7	2	7970	248.42	12.86
23482744	2011	7	2	7980	248.32	12.85
23482744	2011	7	2	7990	248.32	12.85
23482744	2011	7	2	8000	248.33	12.85
23482744	2011	7	2	8010	248.21	12.84
23482744	2011	7	2	8020	248.07	12.84
23482744	2011	7	2	8030	248.17	12.83
23482744	2011	7	2	8040	248.1	12.82
23482744	2011	7	2	8050	248.06	12.82
23482744	2011	7	2	8060	248.01	12.82
23482744	2011	7	2	8070	247.8	12.82
23482744	2011	7	2	8080	247.57	12.82
23482744	2011	7	2	8090	247.22	12.81
23482744	2011	7	2	8100	246.41	12.81
23482744	2011	7	2	8110	246.09	12.81
23482744	2011	7	2	8120	246.27	12.74
23482744	2011	7	2	8130	246.65	12.74
23482744	2011	7	2	8140	246.62	12.74
23482744	2011	7	2	8150	246.59	12.74
23482744	2011	7	2	8160	246.62	12.74

23482744	2011	7	2	8170	246.73	12.74
23482744	2011	7	2	8180	246.79	12.74
23482744	2011	7	2	8190	246.93	12.73
23482744	2011	7	2	8200	246.96	12.72
23482744	2011	7	2	8210	247.06	12.72
23482744	2011	7	2	8220	247.09	12.71
23482744	2011	7	2	8230	246.93	12.71
23482744	2011	7	2	8240	246.85	12.71
23482744	2011	7	2	8250	246.48	12.71
23482744	2011	7	2	8260	246.1	12.71
23482744	2011	7	2	8270	246.17	12.71
23482744	2011	7	2	8280	246.21	12.71
23482744	2011	7	2	8290	246.47	12.7
23482744	2011	7	2	8300	246.94	12.7
23482744	2011	7	2	8310	247.11	12.7
23482744	2011	7	2	8320	247.1	12.7
23482744	2011	7	2	8330	247.08	12.7
23482744	2011	7	2	8340	247.02	12.69
23482744	2011	7	2	8350	247	12.68
23482744	2011	7	2	8360	247.05	12.68
23482744	2011	7	2	8370	246.98	12.67
23482744	2011	7	2	8380	246.89	12.67
23482744	2011	7	2	8390	246.81	12.67
23482744	2011	7	2	8400	246.75	12.67
23482744	2011	7	2	8410	246.84	12.66
23482744	2011	7	2	8420	246.95	12.66
23482744	2011	7	2	8430	247.14	12.66
23482744	2011	7	2	8440	247.17	12.65
23482744	2011	7	2	8450	247.15	12.65
23482744	2011	7	2	8460	247.14	12.65
23482744	2011	7	2	8470	247.17	12.65
23482744	2011	7	2	8480	247.25	12.64
23482744	2011	7	2	8490	247.11	12.65
23482744	2011	7	2	8500	247.2	12.65
23482744	2011	7	2	8510	247.12	12.65
23482744	2011	7	2	8520	247.19	12.64
23482744	2011	7	2	8530	247.08	12.65
23482744	2011	7	2	8540	246.75	12.65
23482744	2011	7	2	8550	246.68	12.64
23482744	2011	7	2	8560	246.87	12.64
23482744	2011	7	2	8570	246.72	12.65
23482744	2011	7	2	8580	246.65	12.65
23482744	2011	7	2	8590	247	12.65
23482744	2011	7	2	8600	246.81	12.66
23482744	2011	7	2	8610	246.88	12.66
23482744	2011	7	2	8620	246.78	12.65
23482744	2011	7	2	8630	246.89	12.66

23482744	2011	7	2	8640	246.87	12.66
23482744	2011	7	2	8650	247.26	12.66
23482744	2011	7	2	8660	248.32	12.67
23482744	2011	7	2	8670	249.01	12.66
23482744	2011	7	2	8680	248.97	12.67
23482744	2011	7	2	8690	249.02	12.67
23482744	2011	7	2	8700	248.91	12.67
23482744	2011	7	2	8710	248.52	12.66
23482744	2011	7	2	8720	246.94	12.66
23482744	2011	7	2	8730	246.1	12.66
23482744	2011	7	2	8740	245.55	12.66
23482744	2011	7	2	8750	245	12.66
23482744	2011	7	2	8760	244.85	12.65
23482744	2011	7	2	8770	245.21	12.67
23482744	2011	7	2	8780	246.41	12.67
23482744	2011	7	2	8790	247.27	12.67
23482744	2011	7	2	8800	247.78	12.68
23482744	2011	7	2	8810	247.79	12.67
23482744	2011	7	2	8820	247.72	12.67
23482744	2011	7	2	8830	247.72	12.67
23482744	2011	7	2	8840	247.75	12.67
23482744	2011	7	2	8850	247.72	12.67
23482744	2011	7	2	8860	247.78	12.66
23482744	2011	7	2	8870	247.74	12.66
23482744	2011	7	2	8880	247.77	12.66
23482744	2011	7	2	8890	247.76	12.66
23482744	2011	7	2	8900	247.78	12.66
23482744	2011	7	2	8910	247.8	12.66
23482744	2011	7	2	8920	247.79	12.66
23482744	2011	7	2	8930	247.74	12.65
23482744	2011	7	2	8940	247.79	12.64
23482744	2011	7	2	8950	246.56	12.64
23482744	2011	7	2	8960	245.52	12.62
23482744	2011	7	2	8970	245.65	12.63
23482744	2011	7	2	8980	245.75	12.62
23482744	2011	7	2	8990	246.23	12.61
23482744	2011	7	2	9000	247.45	12.64
23482744	2011	7	2	9010	247.84	12.64
23482744	2011	7	2	9020	248.18	12.64
23482744	2011	7	2	9030	248.31	12.64
23482744	2011	7	2	9040	248.8	12.64
23482744	2011	7	2	9050	248.76	12.64
23482744	2011	7	2	9060	248.43	12.64
23482744	2011	7	2	9070	247.6	12.64
23482744	2011	7	2	9080	247.05	12.64
23482744	2011	7	2	9090	245.58	12.64
23482744	2011	7	2	9100	244.96	12.65

23482744	2011	7	2	9110	245.43	12.66
23482744	2011	7	2	9120	245.38	12.66
23482744	2011	7	2	9130	245.43	12.67
23482744	2011	7	2	9140	246.54	12.68
23482744	2011	7	2	9150	246.62	12.69
23482744	2011	7	2	9160	245.97	12.7
23482744	2011	7	2	9170	244.89	12.71
23482744	2011	7	2	9180	244.5	12.71
23482744	2011	7	2	9190	243.58	12.71
23482744	2011	7	2	9200	243.35	12.72
23482744	2011	7	2	9210	243.37	12.74
23482744	2011	7	2	9220	243.88	12.81
23482744	2011	7	2	9230	244.23	12.82
23482744	2011	7	2	9240	244.2	12.82
23482744	2011	7	2	9250	243.65	12.84
23482744	2011	7	2	9260	243.53	12.84
23482744	2011	7	2	9270	243.42	12.84
23482744	2011	7	2	9280	243.45	12.85
23482744	2011	7	2	9290	243.65	12.86
23482744	2011	7	2	9300	244.12	12.87
23482744	2011	7	2	9310	244.63	12.88
23482744	2011	7	2	9320	244.39	12.88
23482744	2011	7	2	9330	244.54	12.88
23482744	2011	7	2	9340	244.34	12.88
23482744	2011	7	2	9350	244.49	12.89
23482744	2011	7	2	9360	244.45	12.88
23482744	2011	7	2	9370	244.42	12.89
23482744	2011	7	2	9380	244.38	12.88
23482744	2011	7	2	9390	245.13	12.88
23482744	2011	7	2	9400	245.72	12.88
23482744	2011	7	2	9410	245.62	12.88
23482744	2011	7	2	9420	245.72	12.88
23482744	2011	7	2	9430	245.79	12.88
23482744	2011	7	2	9440	245.75	12.88
23482744	2011	7	2	9450	245.75	12.87
23482744	2011	7	2	9460	245.63	12.86
23482744	2011	7	2	9470	245.8	12.87
23482744	2011	7	2	9480	245.68	12.86
23482744	2011	7	2	9490	245.73	12.86
23482744	2011	7	2	9500	245.78	12.86
23482744	2011	7	2	9510	245.7	12.86
23482744	2011	7	2	9520	245.76	12.85
23482744	2011	7	2	9530	245.65	12.86
23482744	2011	7	2	9540	245.78	12.85
23482744	2011	7	2	9550	245.69	12.85
23482744	2011	7	2	9560	245.7	12.85
23482744	2011	7	2	9570	245.8	12.85

23482744	2011	7	2	9580	245.7	12.85
23482744	2011	7	2	9590	245.6	12.85
23482744	2011	7	2	9600	245.76	12.84
23482744	2011	7	2	9610	245.72	12.85
23482744	2011	7	2	9620	245.67	12.84
23482744	2011	7	2	9630	245.7	12.84
23482744	2011	7	2	9640	245.7	12.84
23482744	2011	7	2	9650	245.79	12.84
23482744	2011	7	2	9660	245.55	12.84
23482744	2011	7	2	9670	245.8	12.85
23482744	2011	7	2	9680	245.49	12.85
23482744	2011	7	2	9690	246.17	12.85
23482744	2011	7	2	9700	247.61	12.86
23482744	2011	7	2	9710	248.43	12.86
23482744	2011	7	2	9720	248.59	12.85
23482744	2011	7	2	9730	248.72	12.85
23482744	2011	7	2	9740	248.78	12.85
23482744	2011	7	2	9750	248.4	12.84
23482744	2011	7	2	9760	248.29	12.82
23482744	2011	7	2	9770	248.16	12.82
23482744	2011	7	2	9780	247.98	12.82
23482744	2011	7	2	9790	247.78	12.82
23482744	2011	7	2	9800	247.64	12.81
23482744	2011	7	2	9810	247.44	12.71
23482744	2011	7	2	9820	247.26	12.71
23482744	2011	7	2	9830	247.04	12.71
23482744	2011	7	2	9840	246.8	12.69
23482744	2011	7	2	9850	247.15	12.68
23482744	2011	7	2	9860	247.46	12.68
23482744	2011	7	2	9870	247.77	12.67
23482744	2011	7	2	9880	248.16	12.67
23482744	2011	7	2	9890	248.38	12.66
23482744	2011	7	2	9900	248.44	12.64
23482744	2011	7	2	9910	248.46	12.64
23482744	2011	7	2	9920	248.49	12.64
23482744	2011	7	2	9930	248.46	12.62
23482744	2011	7	2	9940	248.48	12.61
23482744	2011	7	2	9950	248.36	12.61
23482744	2011	7	2	9960	247.95	12.6
23482744	2011	7	2	9970	248.32	12.6
23482744	2011	7	2	9980	248.32	12.59
23482744	2011	7	2	9990	248.36	12.59
23482744	2011	7	2	10000	247.91	12.59
23482744	2011	7	2	10010	246.65	12.57
23482744	2011	7	2	10020	245.9	12.57
23482744	2011	7	2	10030	245.44	12.57
23482744	2011	7	2	10040	245.2	12.57

23482744	2011	7	2	10050	243.49	12.56
23482744	2011	7	2	10060	241.35	12.56
23482744	2011	7	2	10070	240.68	12.55
23482744	2011	7	2	10080	239.24	12.55
23482744	2011	7	2	10090	237.54	12.56
23482744	2011	7	2	10100	236.8	12.57
23482744	2011	7	2	10110	236.63	12.58
23482744	2011	7	2	10120	235.63	12.6
23482744	2011	7	2	10130	233.73	12.6
23482744	2011	7	2	10140	232.88	12.62
23482744	2011	7	2	10150	231.31	12.64
23482744	2011	7	2	10160	229.9	12.66
23482744	2011	7	2	10170	228.82	12.67
23482744	2011	7	2	10180	227.01	12.68
23482744	2011	7	2	10190	225.58	12.7
23482744	2011	7	2	10200	225.43	12.72
23482744	2011	7	2	10210	224.15	12.84
23482744	2011	7	2	10220	222.41	12.86
23482744	2011	7	2	10230	221.69	12.89
23482744	2011	7	2	10240	219.8	12.96
23482744	2011	7	2	10250	218.15	13
23482744	2011	7	2	10260	216.52	13.02
23482744	2011	7	2	10270	214.96	13.05
23482744	2011	7	2	10280	213.29	13.1
23482744	2011	7	2	10290	211.83	13.17
23482744	2011	7	2	10300	211.5	13.24
23482744	2011	7	2	10310	209.79	13.3
23482744	2011	7	2	10320	208.22	13.36
23482744	2011	7	2	10330	207.37	13.45
23482744	2011	7	2	10340	205.73	13.52
23482744	2011	7	2	10350	204.57	13.6
23482744	2011	7	2	10360	204.27	13.72
23482744	2011	7	2	10370	203.71	13.76
23482744	2011	7	2	10380	202.96	13.87
23482744	2011	7	2	10390	202.05	13.94
23482744	2011	7	2	10400	201.03	14
23482744	2011	7	2	10410	201.03	14.07
23482744	2011	7	2	10420	201.42	14.17
23482744	2011	7	2	10430	201.38	14.22
23482744	2011	7	2	10440	201.27	14.28
23482744	2011	7	2	10450	201.28	14.32
23482744	2011	7	2	10460	201.45	14.35
23482744	2011	7	2	10470	200.52	14.42
23482744	2011	7	2	10480	199.51	14.5
23482744	2011	7	2	10490	199.38	14.53
23482744	2011	7	2	10500	199.59	14.56
23482744	2011	7	2	10510	198.65	14.61

23482744	2011	7	2	10520	196.95	14.67
23482744	2011	7	2	10530	195.79	14.71
23482744	2011	7	2	10540	194.23	14.75
23482744	2011	7	2	10550	192.18	14.76
23482744	2011	7	2	10560	191.46	14.81
23482744	2011	7	2	10570	190.05	14.92
23482744	2011	7	2	10580	187.92	14.94
23482744	2011	7	2	10590	185.79	14.96
23482744	2011	7	2	10600	184.05	15
23482744	2011	7	2	10610	181.77	15.03
23482744	2011	7	2	10620	179.66	15.06
23482744	2011	7	2	10630	178.52	15.09
23482744	2011	7	2	10640	176.36	15.13
23482744	2011	7	2	10650	173.77	15.16
23482744	2011	7	2	10660	171.85	15.24
23482744	2011	7	2	10670	170.18	15.28
23482744	2011	7	2	10680	168.66	15.35
23482744	2011	7	2	10690	166.32	15.4
23482744	2011	7	2	10700	163.93	15.46
23482744	2011	7	2	10710	162.14	15.51
23482744	2011	7	2	10720	160.94	15.55
23482744	2011	7	2	10730	159.64	15.58
23482744	2011	7	2	10740	157.56	15.63
23482744	2011	7	2	10750	154.86	15.67
23482744	2011	7	2	10760	151.83	15.72
23482744	2011	7	2	10770	149.35	15.78
23482744	2011	7	2	10780	147.62	15.86
23482744	2011	7	2	10790	145.4	15.94
23482744	2011	7	2	10800	144.6	15.99
23482744	2011	7	2	10810	143.45	16.03
23482744	2011	7	2	10820	141.44	16.1
23482744	2011	7	2	10830	139.17	16.18
23482744	2011	7	2	10840	137.17	16.25
23482744	2011	7	2	10850	135.57	16.32
23482744	2011	7	2	10860	133.96	16.41
23482744	2011	7	2	10870	131.81	16.48
23482744	2011	7	2	10880	129.5	16.57
23482744	2011	7	2	10890	126.94	16.64
23482744	2011	7	2	10900	124.76	16.8
23482744	2011	7	2	10910	122.49	16.9
23482744	2011	7	2	10920	120.13	17.05
23482744	2011	7	2	10930	119.25	17.2
23482744	2011	7	2	10940	117.82	17.36
23482744	2011	7	2	10950	116.43	17.54
23482744	2011	7	2	10960	114.59	17.66
23482744	2011	7	2	10970	112.54	17.79
23482744	2011	7	2	10980	110.32	17.93

23482744	2011	7	2	10990	108.21	18.08
23482744	2011	7	2	11000	106.13	18.22
23482744	2011	7	2	11010	103.62	18.36
23482744	2011	7	2	11020	101.14	18.5
23482744	2011	7	2	11030	99.1	18.7
23482744	2011	7	2	11040	97.38	18.84
23482744	2011	7	2	11050	95.88	19
23482744	2011	7	2	11060	94.34	19.17
23482744	2011	7	2	11070	92.51	19.31
23482744	2011	7	2	11080	90.29	19.46
23482744	2011	7	2	11090	89.6	19.64
23482744	2011	7	2	11100	87.97	19.78
23482744	2011	7	2	11110	85.91	19.86
23482744	2011	7	2	11120	85.22	20.01
23482744	2011	7	2	11130	83.99	20.14
23482744	2011	7	2	11140	82.55	20.21
23482744	2011	7	2	11150	81.03	20.31
23482744	2011	7	2	11160	80.26	20.42
23482744	2011	7	2	11170	80.34	20.54
23482744	2011	7	2	11180	79.36	20.68
23482744	2011	7	2	11190	77.7	20.81
23482744	2011	7	2	11200	75.97	20.95
23482744	2011	7	2	11210	74.66	21.09
23482744	2011	7	2	11220	74.09	21.2
23482744	2011	7	2	11230	73.15	21.31
23482744	2011	7	2	11240	71.52	21.42
23482744	2011	7	2	11250	69.85	21.55
23482744	2011	7	2	11260	68.03	21.67
23482744	2011	7	2	11270	67.54	21.82
23482744	2011	7	2	11280	67.77	21.98
23482744	2011	7	2	11290	66.76	22.1
23482744	2011	7	2	11300	65.43	22.21
23482744	2011	7	2	11310	64.33	22.33
23482744	2011	7	2	11320	62.89	22.5
23482744	2011	7	2	11330	61.26	22.62
23482744	2011	7	2	11340	59.48	22.73
23482744	2011	7	2	11350	57.6	22.82
23482744	2011	7	2	11360	55.68	22.94
23482744	2011	7	2	11370	53.43	23.02
23482744	2011	7	2	11380	51.25	23.17
23482744	2011	7	2	11390	49.19	23.27
23482744	2011	7	2	11400	47.1	23.39
23482744	2011	7	2	11410	44.77	23.55
23482744	2011	7	2	11420	42.4	23.67
23482744	2011	7	2	11430	40.82	23.82
23482744	2011	7	2	11440	38.56	23.97
23482744	2011	7	2	11450	36.43	24.15

23482744	2011	7	2	11460	34.34	24.25
23482744	2011	7	2	11470	32.18	24.38
23482744	2011	7	2	11480	30.4	24.49
23482744	2011	7	2	11490	27.96	24.65
23482744	2011	7	2	11500	25.96	24.8
23482744	2011	7	2	11510	23.34	24.99
23482744	2011	7	2	11520	20.96	25.16
23482744	2011	7	2	11530	18.69	25.32
23482744	2011	7	2	11540	16.66	25.44
23482744	2011	7	2	11550	14.6	25.57
23482744	2011	7	2	11560	12.97	25.69
23482744	2011	7	2	11570	12.07	25.8
23482744	2011	7	2	11580	10.44	25.99
23482744	2011	7	2	11590	8.32	26.19
23482744	2011	7	2	11600	6.99	26.35
23482744	2011	7	2	11610	4.67	26.49
23482744	2011	7	2	11620	2.91	26.69
23482744	2011	7	2	11630	2.37	26.76
23482744	2011	7	2	11640	1.82	26.85
23482744	2011	7	2	11650	1.56	26.91
23482744	2011	7	2	11660	0.68	26.97
23482744	2011	7	2	11670	0.59	27.07
23482744	2011	7	2	11680	0.6	27.13
23482744	2011	7	2	11690	0.62	27.16
23482744	2011	7	2	11700	0.6	27.29
23482744	2011	7	2	11710	0.59	27.35
23482744	2011	7	2	11720	0.62	27.4
23482744	2011	7	2	11730	0.59	27.46
23482744	2011	7	2	11740	0.58	27.53
23482744	2011	7	2	11750	0.62	27.57
23482744	2011	7	2	11760	0.61	27.62
23482744	2011	7	2	11770	0.52	27.67
23482744	2011	7	2	11780	0.56	27.74
23482744	2011	7	2	11790	0.53	27.78
23482744	2011	7	2	11800	0.52	27.83
23653213	2011	7	4	0	0.99	27.81
23653213	2011	7	4	10	1.34	27.81
23653213	2011	7	4	20	1.9	27.82
23653213	2011	7	4	30	1.96	27.82
23653213	2011	7	4	40	1.82	27.82
23653213	2011	7	4	50	2.17	27.82
23653213	2011	7	4	60	2.16	27.82
23653213	2011	7	4	70	2.13	27.82
23653213	2011	7	4	80	2.2	27.83
23653213	2011	7	4	90	2.58	27.83
23653213	2011	7	4	100	2.81	27.83
23653213	2011	7	4	110	2.39	27.84

23653213	2011	7	4	120	2.6	27.83
23653213	2011	7	4	130	2.97	27.83
23653213	2011	7	4	140	4.62	27.82
23653213	2011	7	4	150	5.93	27.86
23653213	2011	7	4	160	8.17	27.84
23653213	2011	7	4	170	10.21	27.82
23653213	2011	7	4	180	11.51	27.8
23653213	2011	7	4	190	13.51	27.78
23653213	2011	7	4	200	14.95	27.76
23653213	2011	7	4	210	16.57	27.75
23653213	2011	7	4	220	17.39	27.73
23653213	2011	7	4	230	18.86	27.72
23653213	2011	7	4	240	20.77	27.7
23653213	2011	7	4	250	22.62	27.68
23653213	2011	7	4	260	24.9	27.66
23653213	2011	7	4	270	27.48	27.64
23653213	2011	7	4	280	28.63	27.63
23653213	2011	7	4	290	30.35	27.6
23653213	2011	7	4	300	31.06	27.6
23653213	2011	7	4	310	32.46	27.57
23653213	2011	7	4	320	34.71	27.56
23653213	2011	7	4	330	36.25	27.53
23653213	2011	7	4	340	38.27	27.51
23653213	2011	7	4	350	39.23	27.49
23653213	2011	7	4	360	40.59	27.45
23653213	2011	7	4	370	42.21	27.34
23653213	2011	7	4	380	42.9	27.26
23653213	2011	7	4	390	44.7	27.1
23653213	2011	7	4	400	46.37	26.99
23653213	2011	7	4	410	47.39	26.94
23653213	2011	7	4	420	49.25	26.73
23653213	2011	7	4	430	49.91	26.68
23653213	2011	7	4	440	50.17	26.66
23653213	2011	7	4	450	52.15	26.5
23653213	2011	7	4	460	54.15	26.35
23653213	2011	7	4	470	55.34	26.3
23653213	2011	7	4	480	57.28	26.1
23653213	2011	7	4	490	59.53	25.91
23653213	2011	7	4	500	61.47	25.85
23653213	2011	7	4	510	62.19	25.85
23653213	2011	7	4	520	63.97	25.74
23653213	2011	7	4	530	65.8	25.64
23653213	2011	7	4	540	67.13	25.6
23653213	2011	7	4	550	68.58	25.5
23653213	2011	7	4	560	68.76	25.5
23653213	2011	7	4	570	70.06	25.37
23653213	2011	7	4	580	71.92	25.25

23653213	2011	7	4	590	73.52	25.19
23653213	2011	7	4	600	74.84	25.06
23653213	2011	7	4	610	75.6	25.02
23653213	2011	7	4	620	77.41	24.82
23653213	2011	7	4	630	78.81	24.71
23653213	2011	7	4	640	79.43	24.66
23653213	2011	7	4	650	79.8	24.53
23653213	2011	7	4	660	79.54	24.28
23653213	2011	7	4	670	79.66	24.15
23653213	2011	7	4	680	79.56	24.03
23653213	2011	7	4	690	79.53	23.92
23653213	2011	7	4	700	79.5	23.81
23653213	2011	7	4	710	79.55	23.73
23653213	2011	7	4	720	79.49	23.51
23653213	2011	7	4	730	79.51	23.43
23653213	2011	7	4	740	79.5	23.34
23653213	2011	7	4	750	79.31	23.25
23653213	2011	7	4	760	79.36	23.15
23653213	2011	7	4	770	79.43	23.07
23653213	2011	7	4	780	79.57	23.03
23653213	2011	7	4	790	79.57	22.97
23653213	2011	7	4	800	79.53	22.92
23653213	2011	7	4	810	79.5	22.86
23653213	2011	7	4	820	79.54	22.78
23653213	2011	7	4	830	79.48	22.72
23653213	2011	7	4	840	79.65	22.67
23653213	2011	7	4	850	79.52	22.59
23653213	2011	7	4	860	79.59	22.53
23653213	2011	7	4	870	79.66	22.49
23653213	2011	7	4	880	79.47	22.44
23653213	2011	7	4	890	79.42	22.4
23653213	2011	7	4	900	79.36	22.37
23653213	2011	7	4	910	79.43	22.35
23653213	2011	7	4	920	79.51	22.29
23653213	2011	7	4	930	79.48	22.25
23653213	2011	7	4	940	80.83	22.2
23653213	2011	7	4	950	81.55	22.16
23653213	2011	7	4	960	81.78	22.16
23653213	2011	7	4	970	82.65	22.13
23653213	2011	7	4	980	82.46	22.09
23653213	2011	7	4	990	82.4	22.07
23653213	2011	7	4	1000	82.68	22.04
23653213	2011	7	4	1010	82.88	22.01
23653213	2011	7	4	1020	82.76	22
23653213	2011	7	4	1030	82.84	21.96
23653213	2011	7	4	1040	82.83	21.93
23653213	2011	7	4	1050	82.73	21.89

23653213	2011	7	4	1060	82.83	21.86
23653213	2011	7	4	1070	82.77	21.82
23653213	2011	7	4	1080	82.85	21.8
23653213	2011	7	4	1090	82.65	21.79
23653213	2011	7	4	1100	82.56	21.78
23653213	2011	7	4	1110	83.11	21.74
23653213	2011	7	4	1120	84.39	21.72
23653213	2011	7	4	1130	85.06	21.71
23653213	2011	7	4	1140	85.18	21.7
23653213	2011	7	4	1150	85.18	21.68
23653213	2011	7	4	1160	85.29	21.67
23653213	2011	7	4	1170	85.1	21.65
23653213	2011	7	4	1180	85.27	21.63
23653213	2011	7	4	1190	85.29	21.61
23653213	2011	7	4	1200	85.15	21.6
23653213	2011	7	4	1210	85.34	21.6
23653213	2011	7	4	1220	85.37	21.58
23653213	2011	7	4	1230	85.08	21.57
23653213	2011	7	4	1240	85.25	21.57
23653213	2011	7	4	1250	85.29	21.55
23653213	2011	7	4	1260	85.02	21.55
23653213	2011	7	4	1270	84.95	21.55
23653213	2011	7	4	1280	85.46	21.53
23653213	2011	7	4	1290	85.95	21.53
23653213	2011	7	4	1300	85.96	21.52
23653213	2011	7	4	1310	85.89	21.52
23653213	2011	7	4	1320	86.05	21.5
23653213	2011	7	4	1330	86.49	21.5
23653213	2011	7	4	1340	87.82	21.47
23653213	2011	7	4	1350	88.53	21.46
23653213	2011	7	4	1360	90.4	21.44
23653213	2011	7	4	1370	91.13	21.43
23653213	2011	7	4	1380	91.4	21.44
23653213	2011	7	4	1390	91.58	21.42
23653213	2011	7	4	1400	91.72	21.42
23653213	2011	7	4	1410	91.79	21.42
23653213	2011	7	4	1420	91.94	21.41
23653213	2011	7	4	1430	92.24	21.39
23653213	2011	7	4	1440	92.15	21.39
23653213	2011	7	4	1450	91.73	21.37
23653213	2011	7	4	1460	91.89	21.35
23653213	2011	7	4	1470	92.25	21.34
23653213	2011	7	4	1480	92.28	21.33
23653213	2011	7	4	1490	92.1	21.33
23653213	2011	7	4	1500	92.16	21.32
23653213	2011	7	4	1510	92.19	21.32
23653213	2011	7	4	1520	92.28	21.32

23653213	2011	7	4	1530	92.43	21.31
23653213	2011	7	4	1540	92.62	21.3
23653213	2011	7	4	1550	94.32	21.28
23653213	2011	7	4	1560	95.83	21.26
23653213	2011	7	4	1570	96.06	21.26
23653213	2011	7	4	1580	96.28	21.25
23653213	2011	7	4	1590	95.92	21.24
23653213	2011	7	4	1600	96.42	21.23
23653213	2011	7	4	1610	97.82	21.21
23653213	2011	7	4	1620	98.48	21.21
23653213	2011	7	4	1630	98.95	21.21
23653213	2011	7	4	1640	99.2	21.2
23653213	2011	7	4	1650	99.38	21.21
23653213	2011	7	4	1660	100.11	21.2
23653213	2011	7	4	1670	101.8	21.18
23653213	2011	7	4	1680	102.46	21.17
23653213	2011	7	4	1690	102.19	21.16
23653213	2011	7	4	1700	102.21	21.16
23653213	2011	7	4	1710	104.14	21.14
23653213	2011	7	4	1720	105.19	21.13
23653213	2011	7	4	1730	105.8	21.12
23653213	2011	7	4	1740	107.24	21.08
23653213	2011	7	4	1750	108.06	21.07
23653213	2011	7	4	1760	108.15	21.06
23653213	2011	7	4	1770	108.19	21.05
23653213	2011	7	4	1780	108.12	21.04
23653213	2011	7	4	1790	108.05	21.02
23653213	2011	7	4	1800	107.9	20.97
23653213	2011	7	4	1810	108.32	20.85
23653213	2011	7	4	1820	108.12	20.82
23653213	2011	7	4	1830	108.27	20.75
23653213	2011	7	4	1840	108.19	20.67
23653213	2011	7	4	1850	108.54	20.59
23653213	2011	7	4	1860	108.46	20.57
23653213	2011	7	4	1870	108.41	20.54
23653213	2011	7	4	1880	107.94	20.51
23653213	2011	7	4	1890	107.03	20.47
23653213	2011	7	4	1900	106.74	20.46
23653213	2011	7	4	1910	106.55	20.42
23653213	2011	7	4	1920	107.2	20.39
23653213	2011	7	4	1930	107.17	20.38
23653213	2011	7	4	1940	107.27	20.35
23653213	2011	7	4	1950	107.3	20.35
23653213	2011	7	4	1960	107.59	20.34
23653213	2011	7	4	1970	107.73	20.33
23653213	2011	7	4	1980	107.69	20.32
23653213	2011	7	4	1990	107.03	20.32

23653213	2011	7	4	2000	106.12	20.32
23653213	2011	7	4	2010	105.54	20.32
23653213	2011	7	4	2020	105.57	20.35
23653213	2011	7	4	2030	106.85	20.38
23653213	2011	7	4	2040	106.88	20.38
23653213	2011	7	4	2050	106.9	20.39
23653213	2011	7	4	2060	106.93	20.38
23653213	2011	7	4	2070	106.86	20.38
23653213	2011	7	4	2080	106.98	20.38
23653213	2011	7	4	2090	106.71	20.36
23653213	2011	7	4	2100	106.76	20.35
23653213	2011	7	4	2110	106.28	20.34
23653213	2011	7	4	2120	105.33	20.35
23653213	2011	7	4	2130	105	20.35
23653213	2011	7	4	2140	104.8	20.35
23653213	2011	7	4	2150	104.72	20.35
23653213	2011	7	4	2160	105.04	20.35
23653213	2011	7	4	2170	105.1	20.37
23653213	2011	7	4	2180	104.75	20.38
23653213	2011	7	4	2190	104.68	20.37
23653213	2011	7	4	2200	105.02	20.36
23653213	2011	7	4	2210	105.34	20.35
23653213	2011	7	4	2220	105.74	20.35
23653213	2011	7	4	2230	105.75	20.36
23653213	2011	7	4	2240	105.61	20.36
23653213	2011	7	4	2250	105.21	20.36
23653213	2011	7	4	2260	104.73	20.37
23653213	2011	7	4	2270	104.08	20.39
23653213	2011	7	4	2280	103.47	20.39
23653213	2011	7	4	2290	102.04	20.4
23653213	2011	7	4	2300	101.12	20.42
23653213	2011	7	4	2310	101.42	20.44
23653213	2011	7	4	2320	102.13	20.46
23653213	2011	7	4	2330	103.29	20.47
23653213	2011	7	4	2340	103.83	20.47
23653213	2011	7	4	2350	104.75	20.48
23653213	2011	7	4	2360	105.56	20.48
23653213	2011	7	4	2370	106.02	20.49
23653213	2011	7	4	2380	106.64	20.5
23653213	2011	7	4	2390	106.9	20.51
23653213	2011	7	4	2400	107.28	20.5
23653213	2011	7	4	2410	108.3	20.5
23653213	2011	7	4	2420	108.42	20.5
23653213	2011	7	4	2430	108.27	20.49
23653213	2011	7	4	2440	107.88	20.49
23653213	2011	7	4	2450	107.18	20.5
23653213	2011	7	4	2460	106.55	20.5

23653213	2011	7	4	2470	106.51	20.49
23653213	2011	7	4	2480	107.61	20.48
23653213	2011	7	4	2490	108.34	20.47
23653213	2011	7	4	2500	108.41	20.46
23653213	2011	7	4	2510	108.97	20.46
23653213	2011	7	4	2520	109.45	20.45
23653213	2011	7	4	2530	110.27	20.44
23653213	2011	7	4	2540	111.45	20.42
23653213	2011	7	4	2550	111.87	20.41
23653213	2011	7	4	2560	112.45	20.37
23653213	2011	7	4	2570	113.57	20.34
23653213	2011	7	4	2580	114.43	20.33
23653213	2011	7	4	2590	115.08	20.28
23653213	2011	7	4	2600	114.41	20.26
23653213	2011	7	4	2610	114.32	20.24
23653213	2011	7	4	2620	114.53	20.21
23653213	2011	7	4	2630	114.62	20.19
23653213	2011	7	4	2640	114.45	20.17
23653213	2011	7	4	2650	114.49	20.15
23653213	2011	7	4	2660	114.64	20.11
23653213	2011	7	4	2670	114.51	20.1
23653213	2011	7	4	2680	114.66	20.07
23653213	2011	7	4	2690	114.61	20.06
23653213	2011	7	4	2700	114.32	20.04
23653213	2011	7	4	2710	113.67	20.03
23653213	2011	7	4	2720	112.77	20.03
23653213	2011	7	4	2730	112.54	20
23653213	2011	7	4	2740	111.28	20
23653213	2011	7	4	2750	110.96	20
23653213	2011	7	4	2760	111.32	20
23653213	2011	7	4	2770	111.48	20
23653213	2011	7	4	2780	111.39	19.99
23653213	2011	7	4	2790	111	19.98
23653213	2011	7	4	2800	110.78	19.99
23653213	2011	7	4	2810	111.51	20
23653213	2011	7	4	2820	111.66	19.99
23653213	2011	7	4	2830	112.01	19.99
23653213	2011	7	4	2840	112.29	20
23653213	2011	7	4	2850	112.35	19.99
23653213	2011	7	4	2860	112.33	19.99
23653213	2011	7	4	2870	112.33	19.99
23653213	2011	7	4	2880	112.83	19.98
23653213	2011	7	4	2890	113.32	19.96
23653213	2011	7	4	2900	113.92	19.96
23653213	2011	7	4	2910	114.5	19.94
23653213	2011	7	4	2920	114.69	19.92
23653213	2011	7	4	2930	114.67	19.92

23653213	2011	7	4	2940	114.56	19.92
23653213	2011	7	4	2950	114.69	19.9
23653213	2011	7	4	2960	114.58	19.86
23653213	2011	7	4	2970	114.16	19.86
23653213	2011	7	4	2980	114.15	19.85
23653213	2011	7	4	2990	114.49	19.84
23653213	2011	7	4	3000	115.63	19.82
23653213	2011	7	4	3010	117.04	19.8
23653213	2011	7	4	3020	118.77	19.74
23653213	2011	7	4	3030	119.88	19.72
23653213	2011	7	4	3040	121.43	19.67
23653213	2011	7	4	3050	122.25	19.66
23653213	2011	7	4	3060	123.01	19.64
23653213	2011	7	4	3070	123.14	19.61
23653213	2011	7	4	3080	122.76	19.6
23653213	2011	7	4	3090	122.68	19.57
23653213	2011	7	4	3100	124.27	19.47
23653213	2011	7	4	3110	125.42	19.4
23653213	2011	7	4	3120	126.33	19.38
23653213	2011	7	4	3130	127.45	19.32
23653213	2011	7	4	3140	128.09	19.32
23653213	2011	7	4	3150	128.4	19.29
23653213	2011	7	4	3160	128.34	19.26
23653213	2011	7	4	3170	128.4	19.23
23653213	2011	7	4	3180	128.47	19.19
23653213	2011	7	4	3190	128.44	19.14
23653213	2011	7	4	3200	128.64	19.1
23653213	2011	7	4	3210	128.67	19.04
23653213	2011	7	4	3220	128.69	19.03
23653213	2011	7	4	3230	128.69	19
23653213	2011	7	4	3240	128.06	18.96
23653213	2011	7	4	3250	127.68	18.93
23653213	2011	7	4	3260	127.76	18.89
23653213	2011	7	4	3270	127.69	18.85
23653213	2011	7	4	3280	127.76	18.84
23653213	2011	7	4	3290	127.76	18.82
23653213	2011	7	4	3300	127.82	18.78
23653213	2011	7	4	3310	127.64	18.78
23653213	2011	7	4	3320	127.55	18.76
23653213	2011	7	4	3330	127.5	18.75
23653213	2011	7	4	3340	127.47	18.72
23653213	2011	7	4	3350	127.25	18.71
23653213	2011	7	4	3360	127.09	18.68
23653213	2011	7	4	3370	126.23	18.67
23653213	2011	7	4	3380	125.21	18.67
23653213	2011	7	4	3390	125.03	18.66
23653213	2011	7	4	3400	124.65	18.65

23653213	2011	7	4	3410	124.07	18.64
23653213	2011	7	4	3420	124.26	18.64
23653213	2011	7	4	3430	124.83	18.63
23653213	2011	7	4	3440	124.69	18.64
23653213	2011	7	4	3450	124.58	18.64
23653213	2011	7	4	3460	124.28	18.63
23653213	2011	7	4	3470	124.34	18.63
23653213	2011	7	4	3480	124.9	18.63
23653213	2011	7	4	3490	125.84	18.62
23653213	2011	7	4	3500	126.29	18.62
23653213	2011	7	4	3510	127.08	18.61
23653213	2011	7	4	3520	127.99	18.61
23653213	2011	7	4	3530	128.58	18.6
23653213	2011	7	4	3540	130.27	18.57
23653213	2011	7	4	3550	132.08	18.55
23653213	2011	7	4	3560	132.63	18.54
23653213	2011	7	4	3570	134.11	18.5
23653213	2011	7	4	3580	135.43	18.49
23653213	2011	7	4	3590	136.18	18.47
23653213	2011	7	4	3600	137.26	18.46
23653213	2011	7	4	3610	137.61	18.43
23653213	2011	7	4	3620	138.07	18.41
23653213	2011	7	4	3630	137.92	18.39
23653213	2011	7	4	3640	137.7	18.38
23653213	2011	7	4	3650	138.1	18.36
23653213	2011	7	4	3660	138.03	18.35
23653213	2011	7	4	3670	137.92	18.35
23653213	2011	7	4	3680	138.04	18.33
23653213	2011	7	4	3690	137.86	18.32
23653213	2011	7	4	3700	137.92	18.32
23653213	2011	7	4	3710	137.83	18.29
23653213	2011	7	4	3720	137.76	18.28
23653213	2011	7	4	3730	137.84	18.28
23653213	2011	7	4	3740	137.88	18.25
23653213	2011	7	4	3750	137.95	18.25
23653213	2011	7	4	3760	137.68	18.24
23653213	2011	7	4	3770	137.95	18.23
23653213	2011	7	4	3780	138.05	18.21
23653213	2011	7	4	3790	137.86	18.21
23653213	2011	7	4	3800	137.62	18.21
23653213	2011	7	4	3810	137.75	18.2
23653213	2011	7	4	3820	138.39	18.19
23653213	2011	7	4	3830	139.12	18.18
23653213	2011	7	4	3840	139.15	18.17
23653213	2011	7	4	3850	139.23	18.17
23653213	2011	7	4	3860	139.1	18.17
23653213	2011	7	4	3870	138.8	18.16

23653213	2011	7	4	3880	138.19	18.16
23653213	2011	7	4	3890	138.18	18.15
23653213	2011	7	4	3900	138.09	18.15
23653213	2011	7	4	3910	137.58	18.15
23653213	2011	7	4	3920	136.97	18.14
23653213	2011	7	4	3930	136.98	18.14
23653213	2011	7	4	3940	137.57	18.14
23653213	2011	7	4	3950	137.91	18.13
23653213	2011	7	4	3960	137.64	18.13
23653213	2011	7	4	3970	136.99	18.13
23653213	2011	7	4	3980	136.39	18.12
23653213	2011	7	4	3990	136.77	18.11
23653213	2011	7	4	4000	137.51	18.1
23653213	2011	7	4	4010	138.42	18.1
23653213	2011	7	4	4020	138.8	18.1
23653213	2011	7	4	4030	139.1	18.1
23653213	2011	7	4	4040	140.43	18.09
23653213	2011	7	4	4050	141.7	18.08
23653213	2011	7	4	4060	142.45	18.08
23653213	2011	7	4	4070	142.73	18.08
23653213	2011	7	4	4080	143.36	18.07
23653213	2011	7	4	4090	143.5	18.07
23653213	2011	7	4	4100	143.17	18.07
23653213	2011	7	4	4110	143.17	18.07
23653213	2011	7	4	4120	142.92	18.07
23653213	2011	7	4	4130	142.94	18.06
23653213	2011	7	4	4140	142.87	18.07
23653213	2011	7	4	4150	143.1	18.06
23653213	2011	7	4	4160	143.06	18.07
23653213	2011	7	4	4170	142.84	18.06
23653213	2011	7	4	4180	142.86	18.06
23653213	2011	7	4	4190	143.1	18.05
23653213	2011	7	4	4200	144.1	18.04
23653213	2011	7	4	4210	144.81	18.03
23653213	2011	7	4	4220	145.92	18.03
23653213	2011	7	4	4230	146.44	18.03
23653213	2011	7	4	4240	146.87	18.03
23653213	2011	7	4	4250	147.52	18.03
23653213	2011	7	4	4260	148.04	18.01
23653213	2011	7	4	4270	148.44	18.01
23653213	2011	7	4	4280	148.6	18
23653213	2011	7	4	4290	148.47	18.01
23653213	2011	7	4	4300	148.66	18
23653213	2011	7	4	4310	148.46	18
23653213	2011	7	4	4320	148.14	18
23653213	2011	7	4	4330	148.48	18
23653213	2011	7	4	4340	148.87	17.99

23653213	2011	7	4	4350	149.32	18
23653213	2011	7	4	4360	150.37	17.99
23653213	2011	7	4	4370	151.18	17.98
23653213	2011	7	4	4380	152.12	17.94
23653213	2011	7	4	4390	152.84	17.93
23653213	2011	7	4	4400	153.37	17.9
23653213	2011	7	4	4410	153.97	17.85
23653213	2011	7	4	4420	154.33	17.83
23653213	2011	7	4	4430	154.84	17.81
23653213	2011	7	4	4440	155.42	17.78
23653213	2011	7	4	4450	156.08	17.75
23653213	2011	7	4	4460	156.51	17.71
23653213	2011	7	4	4470	156.94	17.69
23653213	2011	7	4	4480	157.79	17.65
23653213	2011	7	4	4490	158.87	17.6
23653213	2011	7	4	4500	160.65	17.53
23653213	2011	7	4	4510	162.26	17.52
23653213	2011	7	4	4520	163.02	17.51
23653213	2011	7	4	4530	163.5	17.5
23653213	2011	7	4	4540	164.1	17.46
23653213	2011	7	4	4550	163.82	17.44
23653213	2011	7	4	4560	162.14	17.42
23653213	2011	7	4	4570	160.31	17.39
23653213	2011	7	4	4580	159.99	17.32
23653213	2011	7	4	4590	160.74	17.26
23653213	2011	7	4	4600	161.65	17.25
23653213	2011	7	4	4610	162.05	17.25
23653213	2011	7	4	4620	162.55	17.21
23653213	2011	7	4	4630	162.82	17.19
23653213	2011	7	4	4640	162.78	17.15
23653213	2011	7	4	4650	162.73	17.14
23653213	2011	7	4	4660	164.33	17.1
23653213	2011	7	4	4670	165.73	17.08
23653213	2011	7	4	4680	165.2	17.07
23653213	2011	7	4	4690	165.82	17.03
23653213	2011	7	4	4700	167.41	16.97
23653213	2011	7	4	4710	168.47	16.96
23653213	2011	7	4	4720	169.13	16.91
23653213	2011	7	4	4730	170.23	16.85
23653213	2011	7	4	4740	170.98	16.84
23653213	2011	7	4	4750	172.35	16.76
23653213	2011	7	4	4760	172.85	16.75
23653213	2011	7	4	4770	173.95	16.71
23653213	2011	7	4	4780	174.53	16.66
23653213	2011	7	4	4790	175.16	16.58
23653213	2011	7	4	4800	176.15	16.5
23653213	2011	7	4	4810	177.26	16.43

23653213	2011	7	4	4820	177.9	16.42
23653213	2011	7	4	4830	177.84	16.4
23653213	2011	7	4	4840	177.92	16.35
23653213	2011	7	4	4850	177.81	16.3
23653213	2011	7	4	4860	177.83	16.25
23653213	2011	7	4	4870	177.63	16.21
23653213	2011	7	4	4880	177.57	16.16
23653213	2011	7	4	4890	177	16.14
23653213	2011	7	4	4900	176.5	16.1
23653213	2011	7	4	4910	176.03	16.07
23653213	2011	7	4	4920	176.74	15.98
23653213	2011	7	4	4930	177.86	15.96
23653213	2011	7	4	4940	178.36	15.93
23653213	2011	7	4	4950	178.97	15.88
23653213	2011	7	4	4960	179.53	15.85
23653213	2011	7	4	4970	180.18	15.82
23653213	2011	7	4	4980	181.63	15.78
23653213	2011	7	4	4990	182.73	15.75
23653213	2011	7	4	5000	183.25	15.74
23653213	2011	7	4	5010	183.44	15.72
23653213	2011	7	4	5020	184.1	15.69
23653213	2011	7	4	5030	184.97	15.67
23653213	2011	7	4	5040	185.29	15.64
23653213	2011	7	4	5050	186.61	15.59
23653213	2011	7	4	5060	187.57	15.56
23653213	2011	7	4	5070	187.95	15.55
23653213	2011	7	4	5080	189.23	15.48
23653213	2011	7	4	5090	190.06	15.47
23653213	2011	7	4	5100	190.54	15.46
23653213	2011	7	4	5110	191.45	15.42
23653213	2011	7	4	5120	192.57	15.4
23653213	2011	7	4	5130	193.63	15.37
23653213	2011	7	4	5140	194.32	15.35
23653213	2011	7	4	5150	194.93	15.33
23653213	2011	7	4	5160	196.42	15.3
23653213	2011	7	4	5170	196.79	15.29
23653213	2011	7	4	5180	197.05	15.28
23653213	2011	7	4	5190	198.58	15.24
23653213	2011	7	4	5200	199.56	15.22
23653213	2011	7	4	5210	200.48	15.19
23653213	2011	7	4	5220	201.1	15.18
23653213	2011	7	4	5230	202.17	15.16
23653213	2011	7	4	5240	202.8	15.15
23653213	2011	7	4	5250	203.17	15.15
23653213	2011	7	4	5260	202.97	15.14
23653213	2011	7	4	5270	202.94	15.12
23653213	2011	7	4	5280	203.16	15.12

23653213	2011	7	4	5290	203.77	15.1
23653213	2011	7	4	5300	204.44	15.08
23653213	2011	7	4	5310	205.48	15.04
23653213	2011	7	4	5320	206.28	15.03
23653213	2011	7	4	5330	206.38	15.03
23653213	2011	7	4	5340	206.67	15
23653213	2011	7	4	5350	207.34	15
23653213	2011	7	4	5360	208.02	14.99
23653213	2011	7	4	5370	209	14.96
23653213	2011	7	4	5380	210.71	14.95
23653213	2011	7	4	5390	211.37	14.93
23653213	2011	7	4	5400	211.78	14.93
23653213	2011	7	4	5410	212.19	14.92
23653213	2011	7	4	5420	213.49	14.83
23653213	2011	7	4	5430	214.31	14.82
23653213	2011	7	4	5440	214.84	14.82
23653213	2011	7	4	5450	216.22	14.79
23653213	2011	7	4	5460	217	14.78
23653213	2011	7	4	5470	218.4	14.75
23653213	2011	7	4	5480	219.05	14.72
23653213	2011	7	4	5490	219.41	14.71
23653213	2011	7	4	5500	220.19	14.69
23653213	2011	7	4	5510	220.51	14.67
23653213	2011	7	4	5520	221.53	14.62
23653213	2011	7	4	5530	223.03	14.59
23653213	2011	7	4	5540	223.8	14.59
23653213	2011	7	4	5550	224.5	14.59
23653213	2011	7	4	5560	224.98	14.57
23653213	2011	7	4	5570	225.49	14.57
23653213	2011	7	4	5580	225.99	14.54
23653213	2011	7	4	5590	226.22	14.53
23653213	2011	7	4	5600	226.69	14.5
23653213	2011	7	4	5610	227.18	14.48
23653213	2011	7	4	5620	227.76	14.46
23653213	2011	7	4	5630	228.52	14.4
23653213	2011	7	4	5640	228.87	14.4
23653213	2011	7	4	5650	229.91	14.39
23653213	2011	7	4	5660	230.11	14.38
23653213	2011	7	4	5670	230.68	14.35
23653213	2011	7	4	5680	231.69	14.34
23653213	2011	7	4	5690	231.5	14.3
23653213	2011	7	4	5700	231.03	14.29
23653213	2011	7	4	5710	230.27	14.28
23653213	2011	7	4	5720	229.88	14.26
23653213	2011	7	4	5730	229.45	14.24
23653213	2011	7	4	5740	228.97	14.22
23653213	2011	7	4	5750	230.57	14.17

23653213	2011	7	4	5760	231.61	14.17
23653213	2011	7	4	5770	232.3	14.14
23653213	2011	7	4	5780	233.35	14.12
23653213	2011	7	4	5790	234.28	14.11
23653213	2011	7	4	5800	235	14.1
23653213	2011	7	4	5810	235.7	14.07
23653213	2011	7	4	5820	237.08	14.05
23653213	2011	7	4	5830	238.89	14.03
23653213	2011	7	4	5840	240.11	14.01
23653213	2011	7	4	5850	240.98	14
23653213	2011	7	4	5860	241.79	13.99
23653213	2011	7	4	5870	242.59	13.97
23653213	2011	7	4	5880	242.99	13.96
23653213	2011	7	4	5890	242.88	13.93
23653213	2011	7	4	5900	242.7	13.89
23653213	2011	7	4	5910	243.33	13.86
23653213	2011	7	4	5920	244.07	13.85
23653213	2011	7	4	5930	244.73	13.8
23653213	2011	7	4	5940	245.87	13.77
23653213	2011	7	4	5950	246.34	13.76
23653213	2011	7	4	5960	246.47	13.75
23653213	2011	7	4	5970	247.9	13.73
23653213	2011	7	4	5980	249.09	13.71
23653213	2011	7	4	5990	249.29	13.7
23653213	2011	7	4	6000	249.51	13.67
23653213	2011	7	4	6010	250.76	13.65
23653213	2011	7	4	6020	251.51	13.63
23653213	2011	7	4	6030	252.38	13.59
23653213	2011	7	4	6040	253.38	13.57
23653213	2011	7	4	6050	253.91	13.53
23653213	2011	7	4	6060	253.97	13.51
23653213	2011	7	4	6070	254.15	13.48
23653213	2011	7	4	6080	254.88	13.46
23653213	2011	7	4	6090	255.09	13.41
23653213	2011	7	4	6100	255.15	13.39
23653213	2011	7	4	6110	256.15	13.35
23653213	2011	7	4	6120	256.6	13.31
23653213	2011	7	4	6130	257.64	13.27
23653213	2011	7	4	6140	258.6	13.26
23653213	2011	7	4	6150	258.96	13.23
23653213	2011	7	4	6160	259.03	13.2
23653213	2011	7	4	6170	259.23	13.18
23653213	2011	7	4	6180	258.83	13.16
23653213	2011	7	4	6190	258.94	13.14
23653213	2011	7	4	6200	259.52	13.12
23653213	2011	7	4	6210	259.96	13.08
23653213	2011	7	4	6220	260.95	13.04

23653213	2011	7	4	6230	261.5	13.02
23653213	2011	7	4	6240	261.44	13.02
23653213	2011	7	4	6250	260.89	13.01
23653213	2011	7	4	6260	260.42	13
23653213	2011	7	4	6270	260.93	12.97
23653213	2011	7	4	6280	260.97	12.94
23653213	2011	7	4	6290	261.57	12.94
23653213	2011	7	4	6300	262.2	12.93
23653213	2011	7	4	6310	262.18	12.92
23653213	2011	7	4	6320	262.7	12.92
23653213	2011	7	4	6330	263.67	12.92
23653213	2011	7	4	6340	263.75	12.9
23653213	2011	7	4	6350	264.07	12.89
23653213	2011	7	4	6360	264.6	12.89
23653213	2011	7	4	6370	264.59	12.89
23653213	2011	7	4	6380	264.7	12.87
23653213	2011	7	4	6390	264.82	12.87
23653213	2011	7	4	6400	264.79	12.87
23653213	2011	7	4	6410	264.52	12.85
23653213	2011	7	4	6420	263.8	12.85
23653213	2011	7	4	6430	263.52	12.84
23653213	2011	7	4	6440	263.7	12.82
23653213	2011	7	4	6450	263.84	12.81
23653213	2011	7	4	6460	264.39	12.81
23653213	2011	7	4	6470	264.96	12.82
23653213	2011	7	4	6480	265.15	12.81
23653213	2011	7	4	6490	265.39	12.81
23653213	2011	7	4	6500	265.48	12.81
23653213	2011	7	4	6510	265.52	12.74
23653213	2011	7	4	6520	265.62	12.73
23653213	2011	7	4	6530	265.38	12.72
23653213	2011	7	4	6540	264.91	12.73
23653213	2011	7	4	6550	264.68	12.72
23653213	2011	7	4	6560	265.14	12.72
23653213	2011	7	4	6570	266.05	12.71
23653213	2011	7	4	6580	266.62	12.71
23653213	2011	7	4	6590	267.26	12.71
23653213	2011	7	4	6600	267.28	12.71
23653213	2011	7	4	6610	267.63	12.7
23653213	2011	7	4	6620	268.11	12.69
23653213	2011	7	4	6630	269.16	12.69
23653213	2011	7	4	6640	270.01	12.69
23653213	2011	7	4	6650	270.88	12.67
23653213	2011	7	4	6660	270.98	12.64
23653213	2011	7	4	6670	271.67	12.64
23653213	2011	7	4	6680	272.76	12.65
23653213	2011	7	4	6690	273.13	12.64

23653213	2011	7	4	6700	274.16	12.64
23653213	2011	7	4	6710	275.2	12.62
23653213	2011	7	4	6720	275.97	12.6
23653213	2011	7	4	6730	276.98	12.61
23653213	2011	7	4	6740	277.45	12.61
23653213	2011	7	4	6750	278.49	12.6
23653213	2011	7	4	6760	279.3	12.59
23653213	2011	7	4	6770	280.67	12.57
23653213	2011	7	4	6780	281.56	12.57
23653213	2011	7	4	6790	281.78	12.57
23653213	2011	7	4	6800	282.08	12.56
23653213	2011	7	4	6810	282.11	12.55
23653213	2011	7	4	6820	281.79	12.55
23653213	2011	7	4	6830	281.57	12.55
23653213	2011	7	4	6840	281.86	12.54
23653213	2011	7	4	6850	282.87	12.53
23653213	2011	7	4	6860	284.08	12.53
23653213	2011	7	4	6870	284.75	12.53
23653213	2011	7	4	6880	285.3	12.53
23653213	2011	7	4	6890	286.05	12.53
23653213	2011	7	4	6900	286.34	12.54
23653213	2011	7	4	6910	286.36	12.53
23653213	2011	7	4	6920	286.26	12.53
23653213	2011	7	4	6930	286.13	12.53
23653213	2011	7	4	6940	286.32	12.53
23653213	2011	7	4	6950	286.65	12.53
23653213	2011	7	4	6960	286.96	12.53
23653213	2011	7	4	6970	287.21	12.53
23653213	2011	7	4	6980	287.34	12.53
23653213	2011	7	4	6990	287.35	12.53
23653213	2011	7	4	7000	287.22	12.53
23653213	2011	7	4	7010	287.12	12.53
23653213	2011	7	4	7020	287.14	12.53
23653213	2011	7	4	7030	287.42	12.53
23653213	2011	7	4	7040	287.66	12.53
23653213	2011	7	4	7050	287.72	12.53
23653213	2011	7	4	7060	287.5	12.53
23653213	2011	7	4	7070	287.47	12.53
23653213	2011	7	4	7080	288.21	12.54
23653213	2011	7	4	7090	288.24	12.54
23653213	2011	7	4	7100	288.06	12.55
23653213	2011	7	4	7110	287.6	12.55
23653213	2011	7	4	7120	286.87	12.54
23653213	2011	7	4	7130	286.34	12.54
23653213	2011	7	4	7140	286.33	12.54
23653213	2011	7	4	7150	286.1	12.54
23653213	2011	7	4	7160	286.43	12.55

23653213	2011	7	4	7170	287.08	12.54
23653213	2011	7	4	7180	287.45	12.55
23653213	2011	7	4	7190	287.47	12.55
23653213	2011	7	4	7200	287.32	12.57
23653213	2011	7	4	7210	287.54	12.57
23653213	2011	7	4	7220	287.95	12.57
23653213	2011	7	4	7230	288.17	12.57
23653213	2011	7	4	7240	287.56	12.57
23653213	2011	7	4	7250	287.28	12.57
23653213	2011	7	4	7260	287	12.57
23653213	2011	7	4	7270	286.99	12.57
23653213	2011	7	4	7280	287.51	12.57
23653213	2011	7	4	7290	288.75	12.57
23653213	2011	7	4	7300	289.35	12.58
23653213	2011	7	4	7310	289.69	12.58
23653213	2011	7	4	7320	289.58	12.58
23653213	2011	7	4	7330	289.18	12.58
23653213	2011	7	4	7340	289.01	12.59
23653213	2011	7	4	7350	289.54	12.6
23653213	2011	7	4	7360	289.83	12.6
23653213	2011	7	4	7370	290.07	12.6
23653213	2011	7	4	7380	291.58	12.6
23653213	2011	7	4	7390	292.08	12.61
23653213	2011	7	4	7400	293.03	12.61
23653213	2011	7	4	7410	293.5	12.61
23653213	2011	7	4	7420	293.76	12.6
23653213	2011	7	4	7430	293.76	12.6
23653213	2011	7	4	7440	294.52	12.6
23653213	2011	7	4	7450	294.52	12.6
23653213	2011	7	4	7460	293.97	12.6
23653213	2011	7	4	7470	293.35	12.58
23653213	2011	7	4	7480	292.98	12.59
23653213	2011	7	4	7490	292.71	12.58
23653213	2011	7	4	7500	292.37	12.58
23653213	2011	7	4	7510	292.57	12.59
23653213	2011	7	4	7520	292.6	12.58
23653213	2011	7	4	7530	292.37	12.57
23653213	2011	7	4	7540	292.38	12.57
23653213	2011	7	4	7550	293.41	12.57
23653213	2011	7	4	7560	294.69	12.58
23653213	2011	7	4	7570	295.95	12.59
23653213	2011	7	4	7580	296.33	12.58
23653213	2011	7	4	7590	296.19	12.58
23653213	2011	7	4	7600	295.93	12.58
23653213	2011	7	4	7610	296.1	12.58
23653213	2011	7	4	7620	296.13	12.57
23653213	2011	7	4	7630	296.04	12.57

23653213	2011	7	4	7640	296.16	12.57
23653213	2011	7	4	7650	297.32	12.56
23653213	2011	7	4	7660	297.81	12.57
23653213	2011	7	4	7670	298.42	12.56
23653213	2011	7	4	7680	298.4	12.55
23653213	2011	7	4	7690	299.06	12.53
23653213	2011	7	4	7700	300.66	12.53
23653213	2011	7	4	7710	301.79	12.53
23653213	2011	7	4	7720	302.36	12.53
23653213	2011	7	4	7730	302.75	12.49
23653213	2011	7	4	7740	303.25	12.46
23653213	2011	7	4	7750	303.69	12.46
23653213	2011	7	4	7760	303.91	12.43
23653213	2011	7	4	7770	303.8	12.42
23653213	2011	7	4	7780	303.49	12.39
23653213	2011	7	4	7790	303.43	12.38
23653213	2011	7	4	7800	303.83	12.36
23653213	2011	7	4	7810	303.78	12.34
23653213	2011	7	4	7820	303.16	12.32
23653213	2011	7	4	7830	301.93	12.31
23653213	2011	7	4	7840	300.75	12.29
23653213	2011	7	4	7850	299.67	12.28
23653213	2011	7	4	7860	298.79	12.28
23653213	2011	7	4	7870	298	12.26
23653213	2011	7	4	7880	297.88	12.26
23653213	2011	7	4	7890	298.22	12.25
23653213	2011	7	4	7900	297.99	12.25
23653213	2011	7	4	7910	298.29	12.25
23653213	2011	7	4	7920	298.15	12.25
23653213	2011	7	4	7930	298.1	12.24
23653213	2011	7	4	7940	297.84	12.24
23653213	2011	7	4	7950	297.53	12.24
23653213	2011	7	4	7960	297.18	12.23
23653213	2011	7	4	7970	296.84	12.23
23653213	2011	7	4	7980	296.19	12.23
23653213	2011	7	4	7990	295.55	12.22
23653213	2011	7	4	8000	294.86	12.23
23653213	2011	7	4	8010	294.02	12.22
23653213	2011	7	4	8020	293.72	12.21
23653213	2011	7	4	8030	293.63	12.21
23653213	2011	7	4	8040	293.5	12.21
23653213	2011	7	4	8050	294.16	12.21
23653213	2011	7	4	8060	294.29	12.22
23653213	2011	7	4	8070	294.17	12.22
23653213	2011	7	4	8080	293.88	12.23
23653213	2011	7	4	8090	293.63	12.22
23653213	2011	7	4	8100	294.36	12.23

23653213	2011	7	4	8110	294.94	12.23
23653213	2011	7	4	8120	294.95	12.25
23653213	2011	7	4	8130	295.05	12.25
23653213	2011	7	4	8140	295.58	12.25
23653213	2011	7	4	8150	295.71	12.26
23653213	2011	7	4	8160	295.47	12.26
23653213	2011	7	4	8170	294.89	12.25
23653213	2011	7	4	8180	294.1	12.25
23653213	2011	7	4	8190	293.16	12.25
23653213	2011	7	4	8200	292.3	12.25
23653213	2011	7	4	8210	291.87	12.25
23653213	2011	7	4	8220	291.41	12.25
23653213	2011	7	4	8230	291.02	12.25
23653213	2011	7	4	8240	290.86	12.25
23653213	2011	7	4	8250	290.78	12.25
23653213	2011	7	4	8260	290.59	12.25
23653213	2011	7	4	8270	290.97	12.26
23653213	2011	7	4	8280	291.12	12.25
23653213	2011	7	4	8290	291.08	12.27
23653213	2011	7	4	8300	291.04	12.27
23653213	2011	7	4	8310	291.03	12.27
23653213	2011	7	4	8320	290.97	12.27
23653213	2011	7	4	8330	291.19	12.27
23653213	2011	7	4	8340	291	12.28
23653213	2011	7	4	8350	291.28	12.28
23653213	2011	7	4	8360	291.17	12.28
23653213	2011	7	4	8370	290.89	12.28
23653213	2011	7	4	8380	290.95	12.28
23653213	2011	7	4	8390	290.43	12.28
23653213	2011	7	4	8400	289.84	12.28
23653213	2011	7	4	8410	288.83	12.28
23653213	2011	7	4	8420	287.74	12.28
23653213	2011	7	4	8430	287.22	12.28
23653213	2011	7	4	8440	286.89	12.28
23653213	2011	7	4	8450	286.61	12.28
23653213	2011	7	4	8460	286.23	12.28
23653213	2011	7	4	8470	285.37	12.28
23653213	2011	7	4	8480	284.4	12.28
23653213	2011	7	4	8490	283.91	12.27
23653213	2011	7	4	8500	284.01	12.28
23653213	2011	7	4	8510	283.71	12.29
23653213	2011	7	4	8520	283.91	12.31
23653213	2011	7	4	8530	284.13	12.3
23653213	2011	7	4	8540	284.1	12.31
23653213	2011	7	4	8550	283.98	12.31
23653213	2011	7	4	8560	284.15	12.32
23653213	2011	7	4	8570	284.04	12.32

23653213	2011	7	4	8580	284.31	12.33
23653213	2011	7	4	8590	284.3	12.34
23653213	2011	7	4	8600	284.18	12.34
23653213	2011	7	4	8610	284.07	12.34
23653213	2011	7	4	8620	284.17	12.35
23653213	2011	7	4	8630	283.98	12.35
23653213	2011	7	4	8640	283.99	12.35
23653213	2011	7	4	8650	283.18	12.36
23653213	2011	7	4	8660	282.75	12.36
23653213	2011	7	4	8670	282.4	12.37
23653213	2011	7	4	8680	282.23	12.37
23653213	2011	7	4	8690	282.39	12.38
23653213	2011	7	4	8700	282.47	12.39
23653213	2011	7	4	8710	282.88	12.39
23653213	2011	7	4	8720	283.36	12.39
23653213	2011	7	4	8730	283.76	12.4
23653213	2011	7	4	8740	284.26	12.4
23653213	2011	7	4	8750	284.09	12.4
23653213	2011	7	4	8760	283.99	12.4
23653213	2011	7	4	8770	283.76	12.41
23653213	2011	7	4	8780	283.13	12.4
23653213	2011	7	4	8790	282.53	12.4
23653213	2011	7	4	8800	282.13	12.41
23653213	2011	7	4	8810	280.33	12.4
23653213	2011	7	4	8820	279.51	12.4
23653213	2011	7	4	8830	279.58	12.4
23653213	2011	7	4	8840	279.51	12.41
23653213	2011	7	4	8850	278.89	12.39
23653213	2011	7	4	8860	278.08	12.4
23653213	2011	7	4	8870	277.18	12.39
23653213	2011	7	4	8880	276.1	12.39
23653213	2011	7	4	8890	274.54	12.38
23653213	2011	7	4	8900	272.4	12.38
23653213	2011	7	4	8910	270.27	12.38
23653213	2011	7	4	8920	268.57	12.38
23653213	2011	7	4	8930	266.51	12.37
23653213	2011	7	4	8940	263.94	12.38
23653213	2011	7	4	8950	261.77	12.37
23653213	2011	7	4	8960	259.58	12.37
23653213	2011	7	4	8970	258.23	12.38
23653213	2011	7	4	8980	257.38	12.39
23653213	2011	7	4	8990	255.68	12.39
23653213	2011	7	4	9000	254.9	12.39
23653213	2011	7	4	9010	253.86	12.39
23653213	2011	7	4	9020	251.78	12.39
23653213	2011	7	4	9030	250.91	12.39
23653213	2011	7	4	9040	250.75	12.4

23653213	2011	7	4	9050	249.85	12.4
23653213	2011	7	4	9060	249.66	12.41
23653213	2011	7	4	9070	248.25	12.41
23653213	2011	7	4	9080	246.73	12.43
23653213	2011	7	4	9090	245.87	12.45
23653213	2011	7	4	9100	244.08	12.46
23653213	2011	7	4	9110	243.07	12.47
23653213	2011	7	4	9120	241.85	12.5
23653213	2011	7	4	9130	240.51	12.54
23653213	2011	7	4	9140	240.25	12.55
23653213	2011	7	4	9150	239.35	12.58
23653213	2011	7	4	9160	238.62	12.6
23653213	2011	7	4	9170	238.09	12.61
23653213	2011	7	4	9180	238.2	12.63
23653213	2011	7	4	9190	238.22	12.64
23653213	2011	7	4	9200	237.93	12.68
23653213	2011	7	4	9210	237.7	12.71
23653213	2011	7	4	9220	237.71	12.74
23653213	2011	7	4	9230	238.6	12.85
23653213	2011	7	4	9240	239.52	12.85
23653213	2011	7	4	9250	240.05	12.86
23653213	2011	7	4	9260	240.18	12.88
23653213	2011	7	4	9270	240.7	12.89
23653213	2011	7	4	9280	240.82	12.89
23653213	2011	7	4	9290	240.91	12.89
23653213	2011	7	4	9300	240.86	12.91
23653213	2011	7	4	9310	240.59	12.91
23653213	2011	7	4	9320	240.99	12.93
23653213	2011	7	4	9330	241.05	12.94
23653213	2011	7	4	9340	241.02	12.94
23653213	2011	7	4	9350	241.07	12.94
23653213	2011	7	4	9360	241.05	12.95
23653213	2011	7	4	9370	241.14	12.96
23653213	2011	7	4	9380	241.18	12.96
23653213	2011	7	4	9390	241.13	12.97
23653213	2011	7	4	9400	241.29	12.98
23653213	2011	7	4	9410	241.16	12.99
23653213	2011	7	4	9420	241.29	13
23653213	2011	7	4	9430	241.31	13.01
23653213	2011	7	4	9440	241.31	13.01
23653213	2011	7	4	9450	241.2	13.02
23653213	2011	7	4	9460	240.41	13.03
23653213	2011	7	4	9470	238.68	13.03
23653213	2011	7	4	9480	237.01	13.05
23653213	2011	7	4	9490	235.19	13.07
23653213	2011	7	4	9500	233.84	13.1
23653213	2011	7	4	9510	233.13	13.13

23653213	2011	7	4	9520	231.79	13.15
23653213	2011	7	4	9530	230.81	13.18
23653213	2011	7	4	9540	229.58	13.21
23653213	2011	7	4	9550	228.14	13.25
23653213	2011	7	4	9560	226.87	13.31
23653213	2011	7	4	9570	225.48	13.32
23653213	2011	7	4	9580	223.63	13.33
23653213	2011	7	4	9590	222.3	13.37
23653213	2011	7	4	9600	220.9	13.38
23653213	2011	7	4	9610	219.88	13.39
23653213	2011	7	4	9620	218.32	13.41
23653213	2011	7	4	9630	216.32	13.44
23653213	2011	7	4	9640	214.4	13.46
23653213	2011	7	4	9650	212.88	13.5
23653213	2011	7	4	9660	211.05	13.53
23653213	2011	7	4	9670	209.2	13.57
23653213	2011	7	4	9680	208.44	13.6
23653213	2011	7	4	9690	206.66	13.62
23653213	2011	7	4	9700	204.79	13.66
23653213	2011	7	4	9710	202.7	13.68
23653213	2011	7	4	9720	201.05	13.75
23653213	2011	7	4	9730	199.15	13.78
23653213	2011	7	4	9740	197.63	13.87
23653213	2011	7	4	9750	195.69	13.9
23653213	2011	7	4	9760	194.15	13.95
23653213	2011	7	4	9770	192.2	13.99
23653213	2011	7	4	9780	190.22	14.02
23653213	2011	7	4	9790	189.46	14.07
23653213	2011	7	4	9800	187.38	14.1
23653213	2011	7	4	9810	185.6	14.14
23653213	2011	7	4	9820	183.67	14.19
23653213	2011	7	4	9830	182.55	14.21
23653213	2011	7	4	9840	180.73	14.26
23653213	2011	7	4	9850	179.79	14.29
23653213	2011	7	4	9860	178.58	14.34
23653213	2011	7	4	9870	177.97	14.37
23653213	2011	7	4	9880	177.94	14.39
23653213	2011	7	4	9890	177.9	14.41
23653213	2011	7	4	9900	177.63	14.43
23653213	2011	7	4	9910	177.22	14.46
23653213	2011	7	4	9920	176.6	14.48
23653213	2011	7	4	9930	175.77	14.5
23653213	2011	7	4	9940	173.7	14.53
23653213	2011	7	4	9950	172.42	14.58
23653213	2011	7	4	9960	171.21	14.67
23653213	2011	7	4	9970	169.74	14.71
23653213	2011	7	4	9980	169.33	14.75

23653213	2011	7	4	9990	168.83	14.78
23653213	2011	7	4	10000	166.79	14.82
23653213	2011	7	4	10010	165.28	14.9
23653213	2011	7	4	10020	163.96	14.93
23653213	2011	7	4	10030	162.66	14.95
23653213	2011	7	4	10040	161.41	14.98
23653213	2011	7	4	10050	160.07	15
23653213	2011	7	4	10060	158.85	15.03
23653213	2011	7	4	10070	158.27	15.09
23653213	2011	7	4	10080	158.11	15.14
23653213	2011	7	4	10090	157.53	15.22
23653213	2011	7	4	10100	155.69	15.32
23653213	2011	7	4	10110	153.46	15.42
23653213	2011	7	4	10120	151.39	15.53
23653213	2011	7	4	10130	149.21	15.63
23653213	2011	7	4	10140	146.93	15.71
23653213	2011	7	4	10150	145.59	15.81
23653213	2011	7	4	10160	145.19	15.88
23653213	2011	7	4	10170	143.59	16.08
23653213	2011	7	4	10180	141.66	16.17
23653213	2011	7	4	10190	140.6	16.25
23653213	2011	7	4	10200	139.68	16.35
23653213	2011	7	4	10210	139.63	16.42
23653213	2011	7	4	10220	139.34	16.5
23653213	2011	7	4	10230	139.37	16.57
23653213	2011	7	4	10240	139.1	16.63
23653213	2011	7	4	10250	138.54	16.67
23653213	2011	7	4	10260	138.76	16.74
23653213	2011	7	4	10270	138.81	16.81
23653213	2011	7	4	10280	138.39	16.88
23653213	2011	7	4	10290	137.84	16.92
23653213	2011	7	4	10300	138.24	17.03
23653213	2011	7	4	10310	139.03	17.07
23653213	2011	7	4	10320	139.03	17.11
23653213	2011	7	4	10330	139.18	17.16
23653213	2011	7	4	10340	139.35	17.18
23653213	2011	7	4	10350	139.13	17.24
23653213	2011	7	4	10360	137.5	17.28
23653213	2011	7	4	10370	135.24	17.31
23653213	2011	7	4	10380	133.78	17.36
23653213	2011	7	4	10390	131.45	17.4
23653213	2011	7	4	10400	129.16	17.45
23653213	2011	7	4	10410	126.7	17.5
23653213	2011	7	4	10420	126.29	17.53
23653213	2011	7	4	10430	127.59	17.57
23653213	2011	7	4	10440	128.45	17.63
23653213	2011	7	4	10450	127.16	17.65

23653213	2011	7	4	10460	124.41	17.71
23653213	2011	7	4	10470	121.22	17.75
23653213	2011	7	4	10480	118.25	17.79
23653213	2011	7	4	10490	115.07	17.83
23653213	2011	7	4	10500	112.34	17.89
23653213	2011	7	4	10510	109.21	17.96
23653213	2011	7	4	10520	106.48	18.06
23653213	2011	7	4	10530	103.46	18.16
23653213	2011	7	4	10540	101.03	18.24
23653213	2011	7	4	10550	99.06	18.36
23653213	2011	7	4	10560	96.7	18.54
23653213	2011	7	4	10570	93.89	18.65
23653213	2011	7	4	10580	90.88	18.78
23653213	2011	7	4	10590	88.12	18.92
23653213	2011	7	4	10600	86.13	19.08
23653213	2011	7	4	10610	84.85	19.19
23653213	2011	7	4	10620	83.45	19.31
23653213	2011	7	4	10630	82.36	19.46
23653213	2011	7	4	10640	81.27	19.62
23653213	2011	7	4	10650	80.6	19.77
23653213	2011	7	4	10660	80.19	20
23653213	2011	7	4	10670	79.39	20.11
23653213	2011	7	4	10680	79.01	20.14
23653213	2011	7	4	10690	78.88	20.25
23653213	2011	7	4	10700	78.05	20.36
23653213	2011	7	4	10710	76.24	20.5
23653213	2011	7	4	10720	74.46	20.53
23653213	2011	7	4	10730	73.33	20.63
23653213	2011	7	4	10740	72.21	20.75
23653213	2011	7	4	10750	71.13	20.82
23653213	2011	7	4	10760	70.26	20.97
23653213	2011	7	4	10770	69.74	21.09
23653213	2011	7	4	10780	68.65	21.3
23653213	2011	7	4	10790	67.49	21.54
23653213	2011	7	4	10800	66.04	21.68
23653213	2011	7	4	10810	65.62	21.77
23653213	2011	7	4	10820	64.95	21.96
23653213	2011	7	4	10830	64.3	22.1
23653213	2011	7	4	10840	63.77	22.2
23653213	2011	7	4	10850	62.75	22.46
23653213	2011	7	4	10860	62.48	22.54
23653213	2011	7	4	10870	62.09	22.65
23653213	2011	7	4	10880	62.18	22.73
23653213	2011	7	4	10890	62.4	22.78
23653213	2011	7	4	10900	62.31	22.86
23653213	2011	7	4	10910	61.64	23
23653213	2011	7	4	10920	61.13	23.12

23653213	2011	7	4	10930	60.75	23.2
23653213	2011	7	4	10940	59.7	23.27
23653213	2011	7	4	10950	59.36	23.33
23653213	2011	7	4	10960	57.93	23.42
23653213	2011	7	4	10970	55.8	23.49
23653213	2011	7	4	10980	53.53	23.55
23653213	2011	7	4	10990	51.1	23.65
23653213	2011	7	4	11000	48.66	23.69
23653213	2011	7	4	11010	47.03	23.76
23653213	2011	7	4	11020	45.12	23.87
23653213	2011	7	4	11030	43.05	23.93
23653213	2011	7	4	11040	41.55	24.03
23653213	2011	7	4	11050	40.06	24.11
23653213	2011	7	4	11060	39.13	24.25
23653213	2011	7	4	11070	38.06	24.39
23653213	2011	7	4	11080	37.17	24.5
23653213	2011	7	4	11090	36.09	24.63
23653213	2011	7	4	11100	35.57	24.71
23653213	2011	7	4	11110	34.94	24.86
23653213	2011	7	4	11120	34.91	24.94
23653213	2011	7	4	11130	35.04	25.02
23653213	2011	7	4	11140	35.44	25.17
23653213	2011	7	4	11150	36.13	25.35
23653213	2011	7	4	11160	36.51	25.43
23653213	2011	7	4	11170	36.92	25.5
23653213	2011	7	4	11180	37.1	25.53
23653213	2011	7	4	11190	37.23	25.58
23653213	2011	7	4	11200	36.68	25.67
23653213	2011	7	4	11210	36.69	25.75
23653213	2011	7	4	11220	36.68	25.83
23653213	2011	7	4	11230	36.14	25.9
23653213	2011	7	4	11240	35.16	26.03
23653213	2011	7	4	11250	33.73	26.1
23653213	2011	7	4	11260	32.69	26.21
23653213	2011	7	4	11270	32.27	26.28
23653213	2011	7	4	11280	32.08	26.36
23653213	2011	7	4	11290	31.38	26.42
23653213	2011	7	4	11300	31.32	26.43
23653213	2011	7	4	11310	31.38	26.5
23653213	2011	7	4	11320	31.37	26.55
23653213	2011	7	4	11330	30.66	26.6
23653213	2011	7	4	11340	30.34	26.65
23653213	2011	7	4	11350	29.67	26.69
23653213	2011	7	4	11360	29.56	26.75
23653213	2011	7	4	11370	28.32	26.8
23653213	2011	7	4	11380	27.77	26.86
23653213	2011	7	4	11390	27.67	26.9

23653213	2011	7	4	11400	27.32	26.92
23653213	2011	7	4	11410	27.06	26.95
23653213	2011	7	4	11420	26.6	27.02
23653213	2011	7	4	11430	25.76	27.03
23653213	2011	7	4	11440	23.72	27.08
23653213	2011	7	4	11450	21.29	27.1
23653213	2011	7	4	11460	18.73	27.15
23653213	2011	7	4	11470	16	27.18
23653213	2011	7	4	11480	14.25	27.25
23653213	2011	7	4	11490	12.14	27.29
23653213	2011	7	4	11500	10.53	27.33
23653213	2011	7	4	11510	8.62	27.37
23653213	2011	7	4	11520	6.33	27.42
23653213	2011	7	4	11530	4.31	27.47
23653213	2011	7	4	11540	2.74	27.5
23653213	2011	7	4	11550	2.18	27.53
23653213	2011	7	4	11560	2.25	27.55
23653213	2011	7	4	11570	2.08	27.57
23653213	2011	7	4	11580	2.06	27.58
23653213	2011	7	4	11590	0.97	27.6
23653213	2011	7	4	11600	0.6	27.64
23653213	2011	7	4	11610	0.62	27.64
23653213	2011	7	4	11620	0.58	27.67
23653213	2011	7	4	11630	0.59	27.7
23653213	2011	7	4	11640	0.6	27.72
23653213	2011	7	4	11650	0.57	27.74
23653213	2011	7	4	11660	0.58	27.77
23653213	2011	7	4	11670	0.63	27.78
23653213	2011	7	4	11680	0.56	27.79
23653213	2011	7	4	11690	0.56	27.8
23653213	2011	7	4	11700	0.6	27.82
23653213	2011	7	4	11710	0.53	27.85
23653213	2011	7	4	11720	0.5	27.85
23653213	2011	7	4	11730	0.53	27.86
23653213	2011	7	4	11740	0.53	27.88
23668453	2011	7	4	0	1.1	28.33
23668453	2011	7	4	10	2.33	28.32
23668453	2011	7	4	20	2.64	28.31
23668453	2011	7	4	30	2.65	28.32
23668453	2011	7	4	40	2.72	28.32
23668453	2011	7	4	50	2.77	28.31
23668453	2011	7	4	60	2.17	28.32
23668453	2011	7	4	70	2.62	28.29
23668453	2011	7	4	80	2.74	28.28
23668453	2011	7	4	90	2.58	28.27
23668453	2011	7	4	100	3.61	28.25
23668453	2011	7	4	110	4.63	28.22

23668453	2011	7	4	120	6.45	28.19
23668453	2011	7	4	130	8.18	28.15
23668453	2011	7	4	140	10	28.11
23668453	2011	7	4	150	10.48	28.1
23668453	2011	7	4	160	10.6	28.11
23668453	2011	7	4	170	10.47	28.1
23668453	2011	7	4	180	10.09	28.1
23668453	2011	7	4	190	9.91	28.1
23668453	2011	7	4	200	9.62	28.07
23668453	2011	7	4	210	9.23	28.08
23668453	2011	7	4	220	8.63	28.08
23668453	2011	7	4	230	7.87	28.08
23668453	2011	7	4	240	7.44	28.07
23668453	2011	7	4	250	7.74	28.07
23668453	2011	7	4	260	7.79	28.05
23668453	2011	7	4	270	7.65	28.05
23668453	2011	7	4	280	8.51	28.03
23668453	2011	7	4	290	10.39	28
23668453	2011	7	4	300	12.43	27.97
23668453	2011	7	4	310	13.99	27.96
23668453	2011	7	4	320	16.14	27.93
23668453	2011	7	4	330	17.85	27.92
23668453	2011	7	4	340	19.5	27.91
23668453	2011	7	4	350	21	27.89
23668453	2011	7	4	360	21.62	27.89
23668453	2011	7	4	370	22.68	27.86
23668453	2011	7	4	380	24.33	27.84
23668453	2011	7	4	390	26.14	27.82
23668453	2011	7	4	400	28.1	27.8
23668453	2011	7	4	410	29.75	27.78
23668453	2011	7	4	420	31.25	27.76
23668453	2011	7	4	430	33.37	27.74
23668453	2011	7	4	440	35.79	27.72
23668453	2011	7	4	450	37.72	27.69
23668453	2011	7	4	460	38.94	27.68
23668453	2011	7	4	470	40.65	27.66
23668453	2011	7	4	480	42.75	27.64
23668453	2011	7	4	490	44.43	27.6
23668453	2011	7	4	500	46.29	27.54
23668453	2011	7	4	510	47.08	27.48
23668453	2011	7	4	520	46.63	27.41
23668453	2011	7	4	530	46.2	27.39
23668453	2011	7	4	540	46.1	27.32
23668453	2011	7	4	550	45.97	27.28
23668453	2011	7	4	560	47.02	27.1
23668453	2011	7	4	570	48.56	27.03
23668453	2011	7	4	580	48.92	27.03

23668453	2011	7	4	590	48.85	27.02
23668453	2011	7	4	600	48.91	26.93
23668453	2011	7	4	610	48.51	26.86
23668453	2011	7	4	620	48.21	26.82
23668453	2011	7	4	630	48.05	26.77
23668453	2011	7	4	640	47.84	26.67
23668453	2011	7	4	650	48.76	26.6
23668453	2011	7	4	660	49.19	26.54
23668453	2011	7	4	670	49.19	26.46
23668453	2011	7	4	680	50.56	26.28
23668453	2011	7	4	690	52.55	26.17
23668453	2011	7	4	700	54.85	26.07
23668453	2011	7	4	710	57.17	25.98
23668453	2011	7	4	720	59.69	25.86
23668453	2011	7	4	730	62.11	25.75
23668453	2011	7	4	740	63.8	25.71
23668453	2011	7	4	750	64.64	25.71
23668453	2011	7	4	760	65.66	25.64
23668453	2011	7	4	770	67.51	25.53
23668453	2011	7	4	780	68.8	25.46
23668453	2011	7	4	790	68.76	25.46
23668453	2011	7	4	800	69.58	25.37
23668453	2011	7	4	810	71.86	25.1
23668453	2011	7	4	820	73.59	24.9
23668453	2011	7	4	830	74.08	24.9
23668453	2011	7	4	840	74.86	24.77
23668453	2011	7	4	850	75.73	24.71
23668453	2011	7	4	860	76.91	24.6
23668453	2011	7	4	870	77.18	24.53
23668453	2011	7	4	880	77.29	24.48
23668453	2011	7	4	890	78.46	24.34
23668453	2011	7	4	900	78.69	24.3
23668453	2011	7	4	910	79.16	24.23
23668453	2011	7	4	920	79.52	24.14
23668453	2011	7	4	930	79.97	24.09
23668453	2011	7	4	940	80.23	24.03
23668453	2011	7	4	950	80.53	23.96
23668453	2011	7	4	960	81.05	23.85
23668453	2011	7	4	970	81.67	23.81
23668453	2011	7	4	980	83.11	23.69
23668453	2011	7	4	990	83.59	23.64
23668453	2011	7	4	1000	83.78	23.6
23668453	2011	7	4	1010	85.2	23.5
23668453	2011	7	4	1020	86.16	23.46
23668453	2011	7	4	1030	87.78	23.33
23668453	2011	7	4	1040	88.42	23.3
23668453	2011	7	4	1050	88.8	23.28

23668453	2011	7	4	1060	88.88	23.25
23668453	2011	7	4	1070	89	23.23
23668453	2011	7	4	1080	88.99	23.17
23668453	2011	7	4	1090	88.97	23.15
23668453	2011	7	4	1100	89.13	23.11
23668453	2011	7	4	1110	89.56	23.06
23668453	2011	7	4	1120	90.34	22.99
23668453	2011	7	4	1130	92.32	22.89
23668453	2011	7	4	1140	93.54	22.84
23668453	2011	7	4	1150	94.39	22.79
23668453	2011	7	4	1160	95.06	22.75
23668453	2011	7	4	1170	95.67	22.69
23668453	2011	7	4	1180	95.84	22.66
23668453	2011	7	4	1190	95.87	22.62
23668453	2011	7	4	1200	95.77	22.58
23668453	2011	7	4	1210	95.64	22.55
23668453	2011	7	4	1220	95.56	22.52
23668453	2011	7	4	1230	95.59	22.46
23668453	2011	7	4	1240	96.12	22.42
23668453	2011	7	4	1250	97.43	22.32
23668453	2011	7	4	1260	98.31	22.29
23668453	2011	7	4	1270	100.11	22.23
23668453	2011	7	4	1280	101.15	22.17
23668453	2011	7	4	1290	101.54	22.15
23668453	2011	7	4	1300	102.45	22.12
23668453	2011	7	4	1310	103.09	22.05
23668453	2011	7	4	1320	103.75	22
23668453	2011	7	4	1330	104.78	21.92
23668453	2011	7	4	1340	106.16	21.88
23668453	2011	7	4	1350	106.96	21.85
23668453	2011	7	4	1360	108.5	21.76
23668453	2011	7	4	1370	109	21.73
23668453	2011	7	4	1380	109.14	21.71
23668453	2011	7	4	1390	110.04	21.67
23668453	2011	7	4	1400	111.17	21.6
23668453	2011	7	4	1410	112.39	21.53
23668453	2011	7	4	1420	112.82	21.5
23668453	2011	7	4	1430	113.73	21.42
23668453	2011	7	4	1440	114.73	21.39
23668453	2011	7	4	1450	116.18	21.32
23668453	2011	7	4	1460	116.7	21.32
23668453	2011	7	4	1470	117.06	21.29
23668453	2011	7	4	1480	118.1	21.19
23668453	2011	7	4	1490	119.75	21.1
23668453	2011	7	4	1500	120.62	21.07
23668453	2011	7	4	1510	121.97	20.99
23668453	2011	7	4	1520	122.81	20.93

23668453	2011	7	4	1530	123.8	20.87
23668453	2011	7	4	1540	125.43	20.65
23668453	2011	7	4	1550	126.22	20.58
23668453	2011	7	4	1560	127.38	20.5
23668453	2011	7	4	1570	127.37	20.39
23668453	2011	7	4	1580	126.99	20.37
23668453	2011	7	4	1590	127.11	20.28
23668453	2011	7	4	1600	127.79	20.22
23668453	2011	7	4	1610	129.36	20.13
23668453	2011	7	4	1620	130.42	20.03
23668453	2011	7	4	1630	131.65	19.94
23668453	2011	7	4	1640	133.44	19.85
23668453	2011	7	4	1650	134.43	19.78
23668453	2011	7	4	1660	135.64	19.75
23668453	2011	7	4	1670	136.54	19.68
23668453	2011	7	4	1680	137.37	19.61
23668453	2011	7	4	1690	138.04	19.53
23668453	2011	7	4	1700	138.52	19.49
23668453	2011	7	4	1710	138.84	19.44
23668453	2011	7	4	1720	139.63	19.37
23668453	2011	7	4	1730	139.61	19.32
23668453	2011	7	4	1740	139.52	19.25
23668453	2011	7	4	1750	139.5	19.2
23668453	2011	7	4	1760	139.28	19.14
23668453	2011	7	4	1770	138.97	19.08
23668453	2011	7	4	1780	139.25	19.02
23668453	2011	7	4	1790	138.95	18.96
23668453	2011	7	4	1800	138.47	18.92
23668453	2011	7	4	1810	139.31	18.84
23668453	2011	7	4	1820	140.13	18.79
23668453	2011	7	4	1830	141.56	18.71
23668453	2011	7	4	1840	142.11	18.66
23668453	2011	7	4	1850	142.52	18.65
23668453	2011	7	4	1860	142.92	18.62
23668453	2011	7	4	1870	143.34	18.57
23668453	2011	7	4	1880	143.46	18.54
23668453	2011	7	4	1890	143.62	18.52
23668453	2011	7	4	1900	144.6	18.46
23668453	2011	7	4	1910	145.76	18.34
23668453	2011	7	4	1920	146.28	18.35
23668453	2011	7	4	1930	147.56	18.28
23668453	2011	7	4	1940	148.05	18.26
23668453	2011	7	4	1950	147.97	18.25
23668453	2011	7	4	1960	148.23	18.22
23668453	2011	7	4	1970	148.65	18.18
23668453	2011	7	4	1980	148.74	18.16
23668453	2011	7	4	1990	148.72	18.14

23668453	2011	7	4	2000	148.84	18.11
23668453	2011	7	4	2010	148.81	18.06
23668453	2011	7	4	2020	148.73	18.03
23668453	2011	7	4	2030	148.73	17.96
23668453	2011	7	4	2040	148.83	17.93
23668453	2011	7	4	2050	148.86	17.91
23668453	2011	7	4	2060	148.79	17.88
23668453	2011	7	4	2070	148.57	17.86
23668453	2011	7	4	2080	148.89	17.83
23668453	2011	7	4	2090	149.26	17.8
23668453	2011	7	4	2100	149.29	17.76
23668453	2011	7	4	2110	149.37	17.75
23668453	2011	7	4	2120	149.38	17.72
23668453	2011	7	4	2130	149.29	17.69
23668453	2011	7	4	2140	149.15	17.68
23668453	2011	7	4	2150	149.92	17.62
23668453	2011	7	4	2160	150.34	17.61
23668453	2011	7	4	2170	150.98	17.57
23668453	2011	7	4	2180	151.23	17.57
23668453	2011	7	4	2190	151.45	17.54
23668453	2011	7	4	2200	151.42	17.53
23668453	2011	7	4	2210	151.5	17.5
23668453	2011	7	4	2220	151.15	17.48
23668453	2011	7	4	2230	150.78	17.47
23668453	2011	7	4	2240	150.75	17.44
23668453	2011	7	4	2250	150.95	17.42
23668453	2011	7	4	2260	151.06	17.41
23668453	2011	7	4	2270	151.14	17.38
23668453	2011	7	4	2280	151.07	17.36
23668453	2011	7	4	2290	151.05	17.35
23668453	2011	7	4	2300	150.53	17.35
23668453	2011	7	4	2310	149.94	17.33
23668453	2011	7	4	2320	150.24	17.32
23668453	2011	7	4	2330	150.18	17.31
23668453	2011	7	4	2340	149.47	17.3
23668453	2011	7	4	2350	148.97	17.29
23668453	2011	7	4	2360	149.13	17.28
23668453	2011	7	4	2370	148.16	17.29
23668453	2011	7	4	2380	146.5	17.28
23668453	2011	7	4	2390	145.05	17.3
23668453	2011	7	4	2400	143.97	17.31
23668453	2011	7	4	2410	143.09	17.32
23668453	2011	7	4	2420	142.34	17.33
23668453	2011	7	4	2430	141.2	17.34
23668453	2011	7	4	2440	140.85	17.34
23668453	2011	7	4	2450	140.04	17.35
23668453	2011	7	4	2460	138.92	17.37

23668453	2011	7	4	2470	137.88	17.38
23668453	2011	7	4	2480	137.79	17.39
23668453	2011	7	4	2490	137.87	17.41
23668453	2011	7	4	2500	136.57	17.42
23668453	2011	7	4	2510	135.59	17.44
23668453	2011	7	4	2520	135.39	17.46
23668453	2011	7	4	2530	135.3	17.5
23668453	2011	7	4	2540	135.15	17.51
23668453	2011	7	4	2550	134.39	17.53
23668453	2011	7	4	2560	134	17.56
23668453	2011	7	4	2570	133.79	17.6
23668453	2011	7	4	2580	133.54	17.62
23668453	2011	7	4	2590	132.12	17.66
23668453	2011	7	4	2600	130.74	17.69
23668453	2011	7	4	2610	128.93	17.73
23668453	2011	7	4	2620	126.66	17.76
23668453	2011	7	4	2630	125.04	17.8
23668453	2011	7	4	2640	124.52	17.85
23668453	2011	7	4	2650	124.59	17.91
23668453	2011	7	4	2660	123.6	17.99
23668453	2011	7	4	2670	121.69	18.05
23668453	2011	7	4	2680	120.28	18.1
23668453	2011	7	4	2690	119.51	18.16
23668453	2011	7	4	2700	119.6	18.22
23668453	2011	7	4	2710	119.97	18.28
23668453	2011	7	4	2720	119.3	18.34
23668453	2011	7	4	2730	117.44	18.42
23668453	2011	7	4	2740	115.15	18.5
23668453	2011	7	4	2750	113.38	18.58
23668453	2011	7	4	2760	111.04	18.63
23668453	2011	7	4	2770	108.72	18.73
23668453	2011	7	4	2780	106.49	18.82
23668453	2011	7	4	2790	104.28	18.9
23668453	2011	7	4	2800	102.75	19.11
23668453	2011	7	4	2810	102.05	19.27
23668453	2011	7	4	2820	100.48	19.36
23668453	2011	7	4	2830	99.1	19.46
23668453	2011	7	4	2840	98.51	19.57
23668453	2011	7	4	2850	97.68	19.66
23668453	2011	7	4	2860	97.77	19.79
23668453	2011	7	4	2870	97.21	19.96
23668453	2011	7	4	2880	97.18	20.01
23668453	2011	7	4	2890	95.97	20.15
23668453	2011	7	4	2900	94.96	20.24
23668453	2011	7	4	2910	94.19	20.35
23668453	2011	7	4	2920	93.49	20.44
23668453	2011	7	4	2930	93.06	20.52

23668453	2011	7	4	2940	92.89	20.59
23668453	2011	7	4	2950	92.41	20.65
23668453	2011	7	4	2960	91.9	20.71
23668453	2011	7	4	2970	90.71	20.75
23668453	2011	7	4	2980	90.07	20.79
23668453	2011	7	4	2990	88.73	20.85
23668453	2011	7	4	3000	88.37	20.91
23668453	2011	7	4	3010	88.44	20.94
23668453	2011	7	4	3020	87.94	20.99
23668453	2011	7	4	3030	87.96	21.02
23668453	2011	7	4	3040	88.18	21.08
23668453	2011	7	4	3050	87.95	21.13
23668453	2011	7	4	3060	87.98	21.14
23668453	2011	7	4	3070	87.41	21.2
23668453	2011	7	4	3080	86.93	21.22
23668453	2011	7	4	3090	86.33	21.27
23668453	2011	7	4	3100	85.61	21.31
23668453	2011	7	4	3110	85.56	21.33
23668453	2011	7	4	3120	85.67	21.36
23668453	2011	7	4	3130	85.48	21.37
23668453	2011	7	4	3140	85.75	21.42
23668453	2011	7	4	3150	85.67	21.42
23668453	2011	7	4	3160	85.57	21.44
23668453	2011	7	4	3170	85.66	21.46
23668453	2011	7	4	3180	85.48	21.47
23668453	2011	7	4	3190	85.57	21.5
23668453	2011	7	4	3200	85.33	21.52
23668453	2011	7	4	3210	84.95	21.54
23668453	2011	7	4	3220	84.65	21.56
23668453	2011	7	4	3230	84.15	21.57
23668453	2011	7	4	3240	82.88	21.61
23668453	2011	7	4	3250	80.74	21.64
23668453	2011	7	4	3260	78.75	21.66
23668453	2011	7	4	3270	78.12	21.7
23668453	2011	7	4	3280	77.01	21.73
23668453	2011	7	4	3290	75.08	21.75
23668453	2011	7	4	3300	74.24	21.77
23668453	2011	7	4	3310	72.87	21.81
23668453	2011	7	4	3320	70.96	21.85
23668453	2011	7	4	3330	69.11	21.91
23668453	2011	7	4	3340	67.16	21.96
23668453	2011	7	4	3350	65.35	22.06
23668453	2011	7	4	3360	63.39	22.12
23668453	2011	7	4	3370	61.39	22.2
23668453	2011	7	4	3380	59.33	22.35
23668453	2011	7	4	3390	56.96	22.46
23668453	2011	7	4	3400	55.47	22.63

23668453	2011	7	4	3410	53.67	22.73
23668453	2011	7	4	3420	52.46	22.86
23668453	2011	7	4	3430	51.98	22.95
23668453	2011	7	4	3440	51.65	23.01
23668453	2011	7	4	3450	51.42	23.08
23668453	2011	7	4	3460	51.1	23.2
23668453	2011	7	4	3470	51.19	23.27
23668453	2011	7	4	3480	51.38	23.34
23668453	2011	7	4	3490	51.43	23.4
23668453	2011	7	4	3500	51.46	23.46
23668453	2011	7	4	3510	51.42	23.53
23668453	2011	7	4	3520	51.27	23.58
23668453	2011	7	4	3530	51.09	23.64
23668453	2011	7	4	3540	50.85	23.68
23668453	2011	7	4	3550	51.63	23.86
23668453	2011	7	4	3560	52.03	23.9
23668453	2011	7	4	3570	52.04	23.96
23668453	2011	7	4	3580	51.77	24.01
23668453	2011	7	4	3590	51.54	24.04
23668453	2011	7	4	3600	51.76	24.1
23668453	2011	7	4	3610	51.74	24.12
23668453	2011	7	4	3620	51.52	24.16
23668453	2011	7	4	3630	51.71	24.19
23668453	2011	7	4	3640	51.81	24.21
23668453	2011	7	4	3650	51.79	24.25
23668453	2011	7	4	3660	51.7	24.28
23668453	2011	7	4	3670	51.68	24.3
23668453	2011	7	4	3680	51.62	24.32
23668453	2011	7	4	3690	51.84	24.36
23668453	2011	7	4	3700	51.81	24.42
23668453	2011	7	4	3710	51.98	24.42
23668453	2011	7	4	3720	52.02	24.45
23668453	2011	7	4	3730	51.21	24.47
23668453	2011	7	4	3740	50.68	24.49
23668453	2011	7	4	3750	49.69	24.52
23668453	2011	7	4	3760	49.21	24.53
23668453	2011	7	4	3770	48.11	24.61
23668453	2011	7	4	3780	47.22	24.67
23668453	2011	7	4	3790	47.5	24.74
23668453	2011	7	4	3800	47.5	24.87
23668453	2011	7	4	3810	47.01	24.94
23668453	2011	7	4	3820	47.01	25.02
23668453	2011	7	4	3830	47.06	25.11
23668453	2011	7	4	3840	47	25.17
23668453	2011	7	4	3850	46.93	25.23
23668453	2011	7	4	3860	46.48	25.28
23668453	2011	7	4	3870	46.32	25.36

23668453	2011	7	4	3880	46.43	25.42
23668453	2011	7	4	3890	46.43	25.47
23668453	2011	7	4	3900	45.31	25.58
23668453	2011	7	4	3910	45.21	25.66
23668453	2011	7	4	3920	45.58	25.75
23668453	2011	7	4	3930	45.98	25.77
23668453	2011	7	4	3940	45.75	25.8
23668453	2011	7	4	3950	45.25	25.85
23668453	2011	7	4	3960	45.11	25.88
23668453	2011	7	4	3970	45.05	25.95
23668453	2011	7	4	3980	44.99	25.97
23668453	2011	7	4	3990	45.09	26.01
23668453	2011	7	4	4000	45.16	26.06
23668453	2011	7	4	4010	45.01	26.1
23668453	2011	7	4	4020	43.84	26.14
23668453	2011	7	4	4030	43.1	26.23
23668453	2011	7	4	4040	43.35	26.36
23668453	2011	7	4	4050	44.58	26.44
23668453	2011	7	4	4060	45.79	26.47
23668453	2011	7	4	4070	46.61	26.51
23668453	2011	7	4	4080	46.77	26.56
23668453	2011	7	4	4090	47.09	26.6
23668453	2011	7	4	4100	47.23	26.64
23668453	2011	7	4	4110	47.08	26.67
23668453	2011	7	4	4120	47.31	26.71
23668453	2011	7	4	4130	47.99	26.7
23668453	2011	7	4	4140	48.55	26.64
23668453	2011	7	4	4150	49.04	26.6
23668453	2011	7	4	4160	49.31	26.57
23668453	2011	7	4	4170	49.52	26.51
23668453	2011	7	4	4180	49.7	26.48
23668453	2011	7	4	4190	49.86	26.44
23668453	2011	7	4	4200	50.17	26.39
23668453	2011	7	4	4210	50.47	26.31
23668453	2011	7	4	4220	50.73	26.28
23668453	2011	7	4	4230	50.75	26.25
23668453	2011	7	4	4240	51.04	26.2
23668453	2011	7	4	4250	51.05	26.19
23668453	2011	7	4	4260	51.01	26.18
23668453	2011	7	4	4270	51.27	26.14
23668453	2011	7	4	4280	51.98	26.13
23668453	2011	7	4	4290	52.64	26.09
23668453	2011	7	4	4300	53.01	26.03
23668453	2011	7	4	4310	52.91	25.99
23668453	2011	7	4	4320	52.67	25.94
23668453	2011	7	4	4330	52.91	25.9
23668453	2011	7	4	4340	53.03	25.85

23668453	2011	7	4	4350	53.12	25.81
23668453	2011	7	4	4360	54.2	25.72
23668453	2011	7	4	4370	55.47	25.64
23668453	2011	7	4	4380	55.96	25.62
23668453	2011	7	4	4390	56.09	25.59
23668453	2011	7	4	4400	56.19	25.57
23668453	2011	7	4	4410	56.7	25.5
23668453	2011	7	4	4420	57.48	25.46
23668453	2011	7	4	4430	57.78	25.43
23668453	2011	7	4	4440	57.71	25.39
23668453	2011	7	4	4450	57.44	25.35
23668453	2011	7	4	4460	56.94	25.33
23668453	2011	7	4	4470	55.41	25.32
23668453	2011	7	4	4480	54.47	25.29
23668453	2011	7	4	4490	52.89	25.28
23668453	2011	7	4	4500	51.59	25.26
23668453	2011	7	4	4510	51.37	25.25
23668453	2011	7	4	4520	51.44	25.22
23668453	2011	7	4	4530	51.15	25.25
23668453	2011	7	4	4540	51.36	25.25
23668453	2011	7	4	4550	52.13	25.22
23668453	2011	7	4	4560	53.25	25.21
23668453	2011	7	4	4570	53.75	25.18
23668453	2011	7	4	4580	53.9	25.18
23668453	2011	7	4	4590	53.95	25.17
23668453	2011	7	4	4600	54.09	25.17
23668453	2011	7	4	4610	54.03	25.15
23668453	2011	7	4	4620	53.82	25.13
23668453	2011	7	4	4630	53.74	25.12
23668453	2011	7	4	4640	54.4	25.1
23668453	2011	7	4	4650	54.47	25.09
23668453	2011	7	4	4660	54.35	25.09
23668453	2011	7	4	4670	53.95	25.07
23668453	2011	7	4	4680	53.5	25.07
23668453	2011	7	4	4690	53.33	25.06
23668453	2011	7	4	4700	53.32	25.05
23668453	2011	7	4	4710	52.82	25.04
23668453	2011	7	4	4720	52.05	25.04
23668453	2011	7	4	4730	50.63	25.03
23668453	2011	7	4	4740	48.79	25.05
23668453	2011	7	4	4750	46.64	25.08
23668453	2011	7	4	4760	44.71	25.15
23668453	2011	7	4	4770	42.51	25.23
23668453	2011	7	4	4780	39.99	25.33
23668453	2011	7	4	4790	37.56	25.47
23668453	2011	7	4	4800	34.79	25.6
23668453	2011	7	4	4810	32.27	25.74

23668453	2011	7	4	4820	29.85	25.85
23668453	2011	7	4	4830	27.54	25.97
23668453	2011	7	4	4840	25.57	26.08
23668453	2011	7	4	4850	24.52	26.19
23668453	2011	7	4	4860	23.23	26.33
23668453	2011	7	4	4870	22.21	26.4
23668453	2011	7	4	4880	21.68	26.5
23668453	2011	7	4	4890	21.47	26.57
23668453	2011	7	4	4900	21.35	26.67
23668453	2011	7	4	4910	21.29	26.74
23668453	2011	7	4	4920	20.5	26.82
23668453	2011	7	4	4930	20.4	26.86
23668453	2011	7	4	4940	20.69	26.9
23668453	2011	7	4	4950	20.76	26.96
23668453	2011	7	4	4960	20.97	26.97
23668453	2011	7	4	4970	20.91	27
23668453	2011	7	4	4980	20.83	27.06
23668453	2011	7	4	4990	20.96	27.1
23668453	2011	7	4	5000	21.2	27.12
23668453	2011	7	4	5010	21.39	27.15
23668453	2011	7	4	5020	21.42	27.19
23668453	2011	7	4	5030	21.64	27.22
23668453	2011	7	4	5040	22.09	27.28
23668453	2011	7	4	5050	21.9	27.31
23668453	2011	7	4	5060	21.54	27.32
23668453	2011	7	4	5070	21.76	27.38
23668453	2011	7	4	5080	21.85	27.4
23668453	2011	7	4	5090	21.63	27.42
23668453	2011	7	4	5100	21.43	27.42
23668453	2011	7	4	5110	21.07	27.44
23668453	2011	7	4	5120	21.1	27.46
23668453	2011	7	4	5130	21.22	27.47
23668453	2011	7	4	5140	21.24	27.49
23668453	2011	7	4	5150	21.46	27.5
23668453	2011	7	4	5160	21.91	27.52
23668453	2011	7	4	5170	21.97	27.53
23668453	2011	7	4	5180	21.99	27.56
23668453	2011	7	4	5190	22.24	27.57
23668453	2011	7	4	5200	22.55	27.57
23668453	2011	7	4	5210	22.95	27.58
23668453	2011	7	4	5220	23.58	27.59
23668453	2011	7	4	5230	23.75	27.61
23668453	2011	7	4	5240	22.52	27.63
23668453	2011	7	4	5250	22.37	27.64
23668453	2011	7	4	5260	22.59	27.64
23668453	2011	7	4	5270	22.6	27.66
23668453	2011	7	4	5280	22.19	27.66

23668453	2011	7	4	5290	21.95	27.67
23668453	2011	7	4	5300	21.04	27.68
23668453	2011	7	4	5310	19.04	27.71
23668453	2011	7	4	5320	16.72	27.73
23668453	2011	7	4	5330	14.82	27.76
23668453	2011	7	4	5340	12.93	27.78
23668453	2011	7	4	5350	11.15	27.81
23668453	2011	7	4	5360	9.77	27.82
23668453	2011	7	4	5370	7.97	27.85
23668453	2011	7	4	5380	5.47	27.88
23668453	2011	7	4	5390	3.89	27.92
23668453	2011	7	4	5400	2.94	27.93
23668453	2011	7	4	5410	2.41	27.94
23668453	2011	7	4	5420	2.37	27.95
23668453	2011	7	4	5430	2.42	27.95
23668453	2011	7	4	5440	2.6	27.96
23668453	2011	7	4	5450	2.16	27.97
23668453	2011	7	4	5460	0.58	28.01
23668453	2011	7	4	5470	0.57	28.02
23668453	2011	7	4	5480	0.59	28.02
23668453	2011	7	4	5490	0.57	28.04
23668453	2011	7	4	5500	0.58	28.07
23668453	2011	7	4	5510	0.58	28.07
23668453	2011	7	4	5520	0.54	28.1
23668453	2011	7	4	5530	0.54	28.11
23668453	2011	7	4	5540	0.56	28.12
23668453	2011	7	4	5550	0.52	28.14
23668453	2011	7	4	5560	0.5	28.16
23668453	2011	7	4	5570	0.49	28.17
23668453	2011	7	4	5580	0.46	28.18
23668453	2011	7	4	5590	0.44	28.2
23668453	2011	7	4	5600	0.46	28.21
23739515	2011	7	5	0	1.05	27.92
23739515	2011	7	5	10	1.19	27.91
23739515	2011	7	5	20	2.07	27.9
23739515	2011	7	5	30	2.49	27.9
23739515	2011	7	5	40	2.68	27.9
23739515	2011	7	5	50	2.7	27.9
23739515	2011	7	5	60	2.27	27.89
23739515	2011	7	5	70	2.45	27.89
23739515	2011	7	5	80	2.82	27.89
23739515	2011	7	5	90	4.52	27.87
23739515	2011	7	5	100	6.85	27.85
23739515	2011	7	5	110	8.41	27.82
23739515	2011	7	5	120	10.28	27.8
23739515	2011	7	5	130	11.68	27.78
23739515	2011	7	5	140	13.14	27.78

23739515	2011	7	5	150	14.65	27.75
23739515	2011	7	5	160	15.44	27.75
23739515	2011	7	5	170	17.13	27.71
23739515	2011	7	5	180	18.82	27.69
23739515	2011	7	5	190	20.54	27.67
23739515	2011	7	5	200	21.16	27.67
23739515	2011	7	5	210	21.73	27.66
23739515	2011	7	5	220	23.45	27.64
23739515	2011	7	5	230	25.23	27.62
23739515	2011	7	5	240	26.98	27.6
23739515	2011	7	5	250	27.63	27.6
23739515	2011	7	5	260	29.77	27.57
23739515	2011	7	5	270	32.59	27.54
23739515	2011	7	5	280	34.54	27.53
23739515	2011	7	5	290	35.91	27.51
23739515	2011	7	5	300	37.96	27.5
23739515	2011	7	5	310	39.24	27.48
23739515	2011	7	5	320	40.08	27.47
23739515	2011	7	5	330	41.92	27.43
23739515	2011	7	5	340	44.32	27.35
23739515	2011	7	5	350	46.92	27.23
23739515	2011	7	5	360	48.71	27.14
23739515	2011	7	5	370	49.45	27.1
23739515	2011	7	5	380	50.62	26.91
23739515	2011	7	5	390	51.3	26.85
23739515	2011	7	5	400	51.89	26.76
23739515	2011	7	5	410	54.05	26.55
23739515	2011	7	5	420	56.87	26.28
23739515	2011	7	5	430	58.68	26.13
23739515	2011	7	5	440	59.6	26.06
23739515	2011	7	5	450	60.15	26
23739515	2011	7	5	460	60.78	25.89
23739515	2011	7	5	470	62.58	25.56
23739515	2011	7	5	480	64.89	25.3
23739515	2011	7	5	490	67.15	25.21
23739515	2011	7	5	500	68.69	25.2
23739515	2011	7	5	510	69.61	25.17
23739515	2011	7	5	520	70.56	25.14
23739515	2011	7	5	530	71.94	24.98
23739515	2011	7	5	540	72.74	24.94
23739515	2011	7	5	550	73.25	24.9
23739515	2011	7	5	560	74.23	24.79
23739515	2011	7	5	570	75.73	24.7
23739515	2011	7	5	580	77.21	24.62
23739515	2011	7	5	590	77.8	24.56
23739515	2011	7	5	600	77.9	24.52
23739515	2011	7	5	610	78.05	24.47

23739515	2011	7	5	620	78.14	24.42
23739515	2011	7	5	630	78.38	24.35
23739515	2011	7	5	640	78.55	24.31
23739515	2011	7	5	650	78.65	24.25
23739515	2011	7	5	660	78.98	24.2
23739515	2011	7	5	670	79.27	24.15
23739515	2011	7	5	680	79.61	24.09
23739515	2011	7	5	690	80.79	24
23739515	2011	7	5	700	81.37	23.92
23739515	2011	7	5	710	81.27	23.85
23739515	2011	7	5	720	81.15	23.82
23739515	2011	7	5	730	80.98	23.74
23739515	2011	7	5	740	80.99	23.72
23739515	2011	7	5	750	80.9	23.68
23739515	2011	7	5	760	81.2	23.65
23739515	2011	7	5	770	81.28	23.6
23739515	2011	7	5	780	81.21	23.55
23739515	2011	7	5	790	81.24	23.53
23739515	2011	7	5	800	81.06	23.47
23739515	2011	7	5	810	81.01	23.42
23739515	2011	7	5	820	80.97	23.39
23739515	2011	7	5	830	80.95	23.38
23739515	2011	7	5	840	81.22	23.36
23739515	2011	7	5	850	82.1	23.33
23739515	2011	7	5	860	83.69	23.26
23739515	2011	7	5	870	84.32	23.24
23739515	2011	7	5	880	85.02	23.21
23739515	2011	7	5	890	85.61	23.17
23739515	2011	7	5	900	85.92	23.15
23739515	2011	7	5	910	86.44	23.06
23739515	2011	7	5	920	87.07	23.03
23739515	2011	7	5	930	87.29	23.02
23739515	2011	7	5	940	87.48	22.99
23739515	2011	7	5	950	87.83	22.96
23739515	2011	7	5	960	88.16	22.89
23739515	2011	7	5	970	88.32	22.79
23739515	2011	7	5	980	88.28	22.75
23739515	2011	7	5	990	87.94	22.7
23739515	2011	7	5	1000	86.93	22.66
23739515	2011	7	5	1010	87.05	22.63
23739515	2011	7	5	1020	86.98	22.53
23739515	2011	7	5	1030	86.6	22.49
23739515	2011	7	5	1040	86.21	22.46
23739515	2011	7	5	1050	85.46	22.44
23739515	2011	7	5	1060	85.5	22.42
23739515	2011	7	5	1070	85.69	22.42
23739515	2011	7	5	1080	85.84	22.41

23739515	2011	7	5	1090	86.05	22.41
23739515	2011	7	5	1100	86.25	22.4
23739515	2011	7	5	1110	86.28	22.4
23739515	2011	7	5	1120	86.36	22.4
23739515	2011	7	5	1130	86.48	22.4
23739515	2011	7	5	1140	86.69	22.4
23739515	2011	7	5	1150	86.8	22.39
23739515	2011	7	5	1160	86.82	22.39
23739515	2011	7	5	1170	86.87	22.39
23739515	2011	7	5	1180	86.99	22.38
23739515	2011	7	5	1190	86.88	22.33
23739515	2011	7	5	1200	87.01	22.29
23739515	2011	7	5	1210	87.14	22.25
23739515	2011	7	5	1220	87.28	22.21
23739515	2011	7	5	1230	87.7	22.16
23739515	2011	7	5	1240	88.15	22.12
23739515	2011	7	5	1250	87.89	22.04
23739515	2011	7	5	1260	87.55	22
23739515	2011	7	5	1270	86.37	21.96
23739515	2011	7	5	1280	85.62	21.92
23739515	2011	7	5	1290	84.53	21.87
23739515	2011	7	5	1300	84.38	21.86
23739515	2011	7	5	1310	83.74	21.86
23739515	2011	7	5	1320	83.27	21.92
23739515	2011	7	5	1330	82.85	21.96
23739515	2011	7	5	1340	82.53	22
23739515	2011	7	5	1350	82.86	22
23739515	2011	7	5	1360	83.38	22.03
23739515	2011	7	5	1370	84.04	22.06
23739515	2011	7	5	1380	84.74	22.09
23739515	2011	7	5	1390	85.48	22.14
23739515	2011	7	5	1400	86.15	22.17
23739515	2011	7	5	1410	86.32	22.21
23739515	2011	7	5	1420	86.29	22.24
23739515	2011	7	5	1430	86.01	22.28
23739515	2011	7	5	1440	85.87	22.31
23739515	2011	7	5	1450	85.75	22.33
23739515	2011	7	5	1460	85.6	22.36
23739515	2011	7	5	1470	85.45	22.38
23739515	2011	7	5	1480	85.15	22.41
23739515	2011	7	5	1490	85.44	22.46
23739515	2011	7	5	1500	85.57	22.46
23739515	2011	7	5	1510	85.78	22.49
23739515	2011	7	5	1520	85.91	22.5
23739515	2011	7	5	1530	86.36	22.53
23739515	2011	7	5	1540	87.26	22.53
23739515	2011	7	5	1550	87.8	22.55

23739515	2011	7	5	1560	88.05	22.56
23739515	2011	7	5	1570	88.17	22.57
23739515	2011	7	5	1580	88.05	22.56
23739515	2011	7	5	1590	88.12	22.54
23739515	2011	7	5	1600	88.04	22.52
23739515	2011	7	5	1610	87.8	22.51
23739515	2011	7	5	1620	88.12	22.47
23739515	2011	7	5	1630	88.35	22.42
23739515	2011	7	5	1640	88.39	22.39
23739515	2011	7	5	1650	88.67	22.35
23739515	2011	7	5	1660	88.96	22.28
23739515	2011	7	5	1670	88.92	22.23
23739515	2011	7	5	1680	88.42	22.14
23739515	2011	7	5	1690	88.24	22.07
23739515	2011	7	5	1700	89.35	21.83
23739515	2011	7	5	1710	91.17	21.78
23739515	2011	7	5	1720	91.9	21.8
23739515	2011	7	5	1730	92.71	21.68
23739515	2011	7	5	1740	94.5	21.58
23739515	2011	7	5	1750	95.67	21.54
23739515	2011	7	5	1760	97.75	21.5
23739515	2011	7	5	1770	100.3	21.42
23739515	2011	7	5	1780	101.87	21.42
23739515	2011	7	5	1790	102.76	21.4
23739515	2011	7	5	1800	103.88	21.35
23739515	2011	7	5	1810	105.12	21.28
23739515	2011	7	5	1820	107.89	21.22
23739515	2011	7	5	1830	110.44	21.17
23739515	2011	7	5	1840	111.98	21.17
23739515	2011	7	5	1850	114.01	21.14
23739515	2011	7	5	1860	115.3	21.1
23739515	2011	7	5	1870	116.5	21.07
23739515	2011	7	5	1880	117.21	21.07
23739515	2011	7	5	1890	117.54	21.04
23739515	2011	7	5	1900	117.63	21.02
23739515	2011	7	5	1910	117.6	21
23739515	2011	7	5	1920	117.64	20.98
23739515	2011	7	5	1930	117.59	20.96
23739515	2011	7	5	1940	117.72	20.94
23739515	2011	7	5	1950	117.68	20.92
23739515	2011	7	5	1960	117.55	20.91
23739515	2011	7	5	1970	117.8	20.88
23739515	2011	7	5	1980	117.83	20.86
23739515	2011	7	5	1990	117.74	20.85
23739515	2011	7	5	2000	117.52	20.83
23739515	2011	7	5	2010	117.37	20.82
23739515	2011	7	5	2020	117.69	20.8

23739515	2011	7	5	2030	117.84	20.78
23739515	2011	7	5	2040	117.8	20.76
23739515	2011	7	5	2050	117.78	20.75
23739515	2011	7	5	2060	117.7	20.75
23739515	2011	7	5	2070	117.64	20.74
23739515	2011	7	5	2080	117.66	20.72
23739515	2011	7	5	2090	117.69	20.71
23739515	2011	7	5	2100	117.76	20.7
23739515	2011	7	5	2110	117.73	20.69
23739515	2011	7	5	2120	117.61	20.68
23739515	2011	7	5	2130	117.63	20.67
23739515	2011	7	5	2140	117.71	20.67
23739515	2011	7	5	2150	117.73	20.65
23739515	2011	7	5	2160	117.73	20.64
23739515	2011	7	5	2170	117.64	20.64
23739515	2011	7	5	2180	117.67	20.64
23739515	2011	7	5	2190	117.7	20.63
23739515	2011	7	5	2200	117.83	20.63
23739515	2011	7	5	2210	117.74	20.62
23739515	2011	7	5	2220	117.77	20.62
23739515	2011	7	5	2230	117.78	20.63
23739515	2011	7	5	2240	117.75	20.61
23739515	2011	7	5	2250	117.86	20.6
23739515	2011	7	5	2260	117.75	20.61
23739515	2011	7	5	2270	117.79	20.61
23739515	2011	7	5	2280	117.92	20.61
23739515	2011	7	5	2290	117.59	20.61
23739515	2011	7	5	2300	117.29	20.6
23739515	2011	7	5	2310	116.79	20.61
23739515	2011	7	5	2320	116.48	20.6
23739515	2011	7	5	2330	117.17	20.6
23739515	2011	7	5	2340	119.65	20.58
23739515	2011	7	5	2350	121.7	20.57
23739515	2011	7	5	2360	122.55	20.57
23739515	2011	7	5	2370	122.97	20.57
23739515	2011	7	5	2380	123.35	20.57
23739515	2011	7	5	2390	123.53	20.56
23739515	2011	7	5	2400	124.86	20.54
23739515	2011	7	5	2410	125.59	20.54
23739515	2011	7	5	2420	126.62	20.53
23739515	2011	7	5	2430	127.43	20.52
23739515	2011	7	5	2440	127.79	20.51
23739515	2011	7	5	2450	127.97	20.5
23739515	2011	7	5	2460	127.96	20.48
23739515	2011	7	5	2470	127.8	20.44
23739515	2011	7	5	2480	128.5	20.37
23739515	2011	7	5	2490	130.35	20.28

23739515	2011	7	5	2500	131.49	20.25
23739515	2011	7	5	2510	132.76	20.21
23739515	2011	7	5	2520	134.04	20.13
23739515	2011	7	5	2530	135.56	20.01
23739515	2011	7	5	2540	136.39	19.98
23739515	2011	7	5	2550	137.42	19.85
23739515	2011	7	5	2560	138.17	19.83
23739515	2011	7	5	2570	138.7	19.79
23739515	2011	7	5	2580	139.46	19.71
23739515	2011	7	5	2590	140.51	19.64
23739515	2011	7	5	2600	141.07	19.59
23739515	2011	7	5	2610	141.78	19.57
23739515	2011	7	5	2620	142.53	19.52
23739515	2011	7	5	2630	142.81	19.5
23739515	2011	7	5	2640	142.91	19.42
23739515	2011	7	5	2650	143.73	19.33
23739515	2011	7	5	2660	144.08	19.3
23739515	2011	7	5	2670	144.14	19.28
23739515	2011	7	5	2680	144.6	19.24
23739515	2011	7	5	2690	144.85	19.2
23739515	2011	7	5	2700	144.83	19.15
23739515	2011	7	5	2710	144.76	19.1
23739515	2011	7	5	2720	144.47	19.04
23739515	2011	7	5	2730	144.83	18.91
23739515	2011	7	5	2740	146.58	18.82
23739515	2011	7	5	2750	147.61	18.81
23739515	2011	7	5	2760	149.2	18.76
23739515	2011	7	5	2770	150.42	18.71
23739515	2011	7	5	2780	151.36	18.67
23739515	2011	7	5	2790	152.22	18.61
23739515	2011	7	5	2800	152.65	18.56
23739515	2011	7	5	2810	153.25	18.48
23739515	2011	7	5	2820	153.74	18.45
23739515	2011	7	5	2830	154.48	18.39
23739515	2011	7	5	2840	155.13	18.35
23739515	2011	7	5	2850	155.59	18.32
23739515	2011	7	5	2860	156.04	18.3
23739515	2011	7	5	2870	156.3	18.27
23739515	2011	7	5	2880	156.7	18.22
23739515	2011	7	5	2890	158.02	18.16
23739515	2011	7	5	2900	158.52	18.13
23739515	2011	7	5	2910	158.72	18.1
23739515	2011	7	5	2920	159	18.08
23739515	2011	7	5	2930	159.36	18.07
23739515	2011	7	5	2940	160.28	18.02
23739515	2011	7	5	2950	160.62	18
23739515	2011	7	5	2960	161.17	17.96

23739515	2011	7	5	2970	161.41	17.93
23739515	2011	7	5	2980	161.84	17.9
23739515	2011	7	5	2990	161.66	17.87
23739515	2011	7	5	3000	161.39	17.84
23739515	2011	7	5	3010	161.55	17.82
23739515	2011	7	5	3020	161.99	17.79
23739515	2011	7	5	3030	161.94	17.77
23739515	2011	7	5	3040	162.15	17.71
23739515	2011	7	5	3050	163.22	17.66
23739515	2011	7	5	3060	164.01	17.65
23739515	2011	7	5	3070	164.53	17.64
23739515	2011	7	5	3080	164.97	17.6
23739515	2011	7	5	3090	165.28	17.58
23739515	2011	7	5	3100	165.9	17.55
23739515	2011	7	5	3110	165.76	17.5
23739515	2011	7	5	3120	165.82	17.49
23739515	2011	7	5	3130	165.68	17.46
23739515	2011	7	5	3140	165.56	17.42
23739515	2011	7	5	3150	165.7	17.37
23739515	2011	7	5	3160	165.74	17.36
23739515	2011	7	5	3170	166.09	17.33
23739515	2011	7	5	3180	166.78	17.32
23739515	2011	7	5	3190	167.31	17.29
23739515	2011	7	5	3200	168.23	17.25
23739515	2011	7	5	3210	169.18	17.21
23739515	2011	7	5	3220	169.94	17.17
23739515	2011	7	5	3230	171.03	17.1
23739515	2011	7	5	3240	171.64	17.03
23739515	2011	7	5	3250	171.62	17
23739515	2011	7	5	3260	171.44	16.97
23739515	2011	7	5	3270	171.25	16.92
23739515	2011	7	5	3280	171.03	16.91
23739515	2011	7	5	3290	170.54	16.89
23739515	2011	7	5	3300	170.97	16.82
23739515	2011	7	5	3310	171.13	16.8
23739515	2011	7	5	3320	171	16.78
23739515	2011	7	5	3330	171.08	16.74
23739515	2011	7	5	3340	171.24	16.71
23739515	2011	7	5	3350	171.48	16.68
23739515	2011	7	5	3360	171.42	16.66
23739515	2011	7	5	3370	171.23	16.64
23739515	2011	7	5	3380	171.34	16.62
23739515	2011	7	5	3390	171.4	16.6
23739515	2011	7	5	3400	172.06	16.57
23739515	2011	7	5	3410	173.87	16.53
23739515	2011	7	5	3420	175.5	16.5
23739515	2011	7	5	3430	175.8	16.48

23739515	2011	7	5	3440	175.99	16.41
23739515	2011	7	5	3450	175.97	16.4
23739515	2011	7	5	3460	175.32	16.35
23739515	2011	7	5	3470	174.68	16.35
23739515	2011	7	5	3480	173.93	16.33
23739515	2011	7	5	3490	172.87	16.32
23739515	2011	7	5	3500	173.72	16.28
23739515	2011	7	5	3510	175.23	16.25
23739515	2011	7	5	3520	176.13	16.25
23739515	2011	7	5	3530	176.87	16.24
23739515	2011	7	5	3540	177.17	16.22
23739515	2011	7	5	3550	177.63	16.19
23739515	2011	7	5	3560	177.76	16.17
23739515	2011	7	5	3570	178.15	16.14
23739515	2011	7	5	3580	178.36	16.13
23739515	2011	7	5	3590	178.75	16.1
23739515	2011	7	5	3600	179.22	16.1
23739515	2011	7	5	3610	179.48	16.1
23739515	2011	7	5	3620	179.96	16.09
23739515	2011	7	5	3630	180.56	16.08
23739515	2011	7	5	3640	180.94	16.07
23739515	2011	7	5	3650	181.24	16.07
23739515	2011	7	5	3660	181.53	16.07
23739515	2011	7	5	3670	181.76	16.09
23739515	2011	7	5	3680	181.62	16.09
23739515	2011	7	5	3690	181.73	16.07
23739515	2011	7	5	3700	181.9	16.07
23739515	2011	7	5	3710	181.9	16.06
23739515	2011	7	5	3720	181.87	16.01
23739515	2011	7	5	3730	181.73	15.97
23739515	2011	7	5	3740	181.39	15.96
23739515	2011	7	5	3750	181.71	15.94
23739515	2011	7	5	3760	181.93	15.89
23739515	2011	7	5	3770	181.98	15.87
23739515	2011	7	5	3780	181.96	15.85
23739515	2011	7	5	3790	181.96	15.84
23739515	2011	7	5	3800	182.05	15.82
23739515	2011	7	5	3810	182.05	15.8
23739515	2011	7	5	3820	182.05	15.78
23739515	2011	7	5	3830	182.12	15.78
23739515	2011	7	5	3840	182.21	15.78
23739515	2011	7	5	3850	182.11	15.76
23739515	2011	7	5	3860	181.99	15.74
23739515	2011	7	5	3870	181.94	15.73
23739515	2011	7	5	3880	181.93	15.73
23739515	2011	7	5	3890	181.89	15.72
23739515	2011	7	5	3900	181.84	15.71

23739515	2011	7	5	3910	181.97	15.7
23739515	2011	7	5	3920	182.15	15.69
23739515	2011	7	5	3930	181.95	15.68
23739515	2011	7	5	3940	182.06	15.67
23739515	2011	7	5	3950	182.14	15.67
23739515	2011	7	5	3960	182.01	15.66
23739515	2011	7	5	3970	182.05	15.67
23739515	2011	7	5	3980	181.99	15.64
23739515	2011	7	5	3990	182.14	15.65
23739515	2011	7	5	4000	182.09	15.64
23739515	2011	7	5	4010	182.15	15.64
23739515	2011	7	5	4020	182.28	15.64
23739515	2011	7	5	4030	182.24	15.65
23739515	2011	7	5	4040	182.21	15.65
23739515	2011	7	5	4050	182.73	15.64
23739515	2011	7	5	4060	182.8	15.64
23739515	2011	7	5	4070	182.83	15.64
23739515	2011	7	5	4080	183	15.64
23739515	2011	7	5	4090	183.05	15.63
23739515	2011	7	5	4100	182.85	15.64
23739515	2011	7	5	4110	181.56	15.64
23739515	2011	7	5	4120	180.11	15.64
23739515	2011	7	5	4130	177.92	15.64
23739515	2011	7	5	4140	176.57	15.64
23739515	2011	7	5	4150	175.37	15.65
23739515	2011	7	5	4160	174.97	15.69
23739515	2011	7	5	4170	174.93	15.73
23739515	2011	7	5	4180	175.12	15.82
23739515	2011	7	5	4190	173.93	15.87
23739515	2011	7	5	4200	173.05	15.89
23739515	2011	7	5	4210	172.77	15.98
23739515	2011	7	5	4220	172.53	16.04
23739515	2011	7	5	4230	171.52	16.1
23739515	2011	7	5	4240	170.92	16.16
23739515	2011	7	5	4250	170.12	16.27
23739515	2011	7	5	4260	169.82	16.33
23739515	2011	7	5	4270	169.82	16.37
23739515	2011	7	5	4280	169.48	16.43
23739515	2011	7	5	4290	169.57	16.46
23739515	2011	7	5	4300	170.06	16.52
23739515	2011	7	5	4310	170.55	16.55
23739515	2011	7	5	4320	170.69	16.56
23739515	2011	7	5	4330	170.58	16.59
23739515	2011	7	5	4340	170.64	16.61
23739515	2011	7	5	4350	170.11	16.63
23739515	2011	7	5	4360	169.79	16.64
23739515	2011	7	5	4370	169.74	16.65

23739515	2011	7	5	4380	169.7	16.65
23739515	2011	7	5	4390	169.44	16.66
23739515	2011	7	5	4400	167.92	16.66
23739515	2011	7	5	4410	167.65	16.67
23739515	2011	7	5	4420	166.93	16.67
23739515	2011	7	5	4430	166.98	16.69
23739515	2011	7	5	4440	167.68	16.72
23739515	2011	7	5	4450	167.58	16.74
23739515	2011	7	5	4460	168	16.74
23739515	2011	7	5	4470	167.8	16.74
23739515	2011	7	5	4480	166.99	16.74
23739515	2011	7	5	4490	165.47	16.75
23739515	2011	7	5	4500	165.18	16.76
23739515	2011	7	5	4510	165.46	16.76
23739515	2011	7	5	4520	165.92	16.78
23739515	2011	7	5	4530	166.44	16.78
23739515	2011	7	5	4540	167.05	16.8
23739515	2011	7	5	4550	166.73	16.81
23739515	2011	7	5	4560	164.53	16.82
23739515	2011	7	5	4570	162.59	16.82
23739515	2011	7	5	4580	162.32	16.82
23739515	2011	7	5	4590	162.81	16.81
23739515	2011	7	5	4600	163.41	16.81
23739515	2011	7	5	4610	164.01	16.81
23739515	2011	7	5	4620	164.8	16.81
23739515	2011	7	5	4630	165.26	16.8
23739515	2011	7	5	4640	165.42	16.8
23739515	2011	7	5	4650	166.48	16.79
23739515	2011	7	5	4660	167.08	16.79
23739515	2011	7	5	4670	167.35	16.77
23739515	2011	7	5	4680	167.44	16.78
23739515	2011	7	5	4690	167.25	16.77
23739515	2011	7	5	4700	167.19	16.76
23739515	2011	7	5	4710	167.24	16.76
23739515	2011	7	5	4720	167.54	16.76
23739515	2011	7	5	4730	167.17	16.75
23739515	2011	7	5	4740	167.35	16.75
23739515	2011	7	5	4750	167.19	16.75
23739515	2011	7	5	4760	166.15	16.75
23739515	2011	7	5	4770	164.29	16.75
23739515	2011	7	5	4780	162.78	16.75
23739515	2011	7	5	4790	160.97	16.75
23739515	2011	7	5	4800	159.68	16.75
23739515	2011	7	5	4810	158.52	16.75
23739515	2011	7	5	4820	157.47	16.76
23739515	2011	7	5	4830	156.78	16.77
23739515	2011	7	5	4840	155.8	16.78

23739515	2011	7	5	4850	154.99	16.79
23739515	2011	7	5	4860	154.8	16.81
23739515	2011	7	5	4870	153.63	16.83
23739515	2011	7	5	4880	151.37	16.85
23739515	2011	7	5	4890	149.71	16.88
23739515	2011	7	5	4900	149.45	16.91
23739515	2011	7	5	4910	149.69	16.98
23739515	2011	7	5	4920	148.8	17.03
23739515	2011	7	5	4930	146.88	17.07
23739515	2011	7	5	4940	145.41	17.13
23739515	2011	7	5	4950	144.76	17.17
23739515	2011	7	5	4960	143.79	17.26
23739515	2011	7	5	4970	143.66	17.3
23739515	2011	7	5	4980	142.25	17.34
23739515	2011	7	5	4990	140.6	17.39
23739515	2011	7	5	5000	138.53	17.42
23739515	2011	7	5	5010	136.81	17.48
23739515	2011	7	5	5020	136.07	17.55
23739515	2011	7	5	5030	135.5	17.61
23739515	2011	7	5	5040	133.47	17.67
23739515	2011	7	5	5050	131.68	17.72
23739515	2011	7	5	5060	129.97	17.79
23739515	2011	7	5	5070	127.61	17.86
23739515	2011	7	5	5080	124.9	17.93
23739515	2011	7	5	5090	122.31	18.01
23739515	2011	7	5	5100	120.19	18.1
23739515	2011	7	5	5110	118.57	18.17
23739515	2011	7	5	5120	116.9	18.28
23739515	2011	7	5	5130	115.47	18.42
23739515	2011	7	5	5140	113.44	18.54
23739515	2011	7	5	5150	111.74	18.62
23739515	2011	7	5	5160	109.65	18.72
23739515	2011	7	5	5170	107.72	18.82
23739515	2011	7	5	5180	105.88	18.92
23739515	2011	7	5	5190	104.23	19.06
23739515	2011	7	5	5200	103.49	19.14
23739515	2011	7	5	5210	102.28	19.22
23739515	2011	7	5	5220	101.75	19.27
23739515	2011	7	5	5230	101.35	19.39
23739515	2011	7	5	5240	99.65	19.48
23739515	2011	7	5	5250	97.73	19.53
23739515	2011	7	5	5260	96.8	19.6
23739515	2011	7	5	5270	96.12	19.65
23739515	2011	7	5	5280	95.19	19.71
23739515	2011	7	5	5290	94.13	19.73
23739515	2011	7	5	5300	91.65	19.8
23739515	2011	7	5	5310	90.1	19.85

23739515	2011	7	5	5320	87.87	19.98
23739515	2011	7	5	5330	86.12	20.02
23739515	2011	7	5	5340	85.4	20.16
23739515	2011	7	5	5350	83.88	20.23
23739515	2011	7	5	5360	82.05	20.29
23739515	2011	7	5	5370	80.7	20.38
23739515	2011	7	5	5380	78.84	20.41
23739515	2011	7	5	5390	76.84	20.46
23739515	2011	7	5	5400	75.36	20.58
23739515	2011	7	5	5410	73.38	20.71
23739515	2011	7	5	5420	71.52	20.82
23739515	2011	7	5	5430	70.27	21.02
23739515	2011	7	5	5440	68.48	21.18
23739515	2011	7	5	5450	67.59	21.35
23739515	2011	7	5	5460	66.23	21.5
23739515	2011	7	5	5470	64.73	21.59
23739515	2011	7	5	5480	64.04	21.8
23739515	2011	7	5	5490	63.29	21.89
23739515	2011	7	5	5500	62.41	22.08
23739515	2011	7	5	5510	61.4	22.14
23739515	2011	7	5	5520	60.8	22.21
23739515	2011	7	5	5530	60.09	22.28
23739515	2011	7	5	5540	59.54	22.36
23739515	2011	7	5	5550	58.79	22.5
23739515	2011	7	5	5560	58.15	22.53
23739515	2011	7	5	5570	57.83	22.62
23739515	2011	7	5	5580	58.04	22.66
23739515	2011	7	5	5590	57.62	22.77
23739515	2011	7	5	5600	57.39	22.83
23739515	2011	7	5	5610	56.87	22.89
23739515	2011	7	5	5620	57.19	22.93
23739515	2011	7	5	5630	57.78	22.94
23739515	2011	7	5	5640	58.03	23.01
23739515	2011	7	5	5650	57.95	23.07
23739515	2011	7	5	5660	57.83	23.12
23739515	2011	7	5	5670	57.6	23.17
23739515	2011	7	5	5680	57.84	23.19
23739515	2011	7	5	5690	58.4	23.21
23739515	2011	7	5	5700	58.78	23.24
23739515	2011	7	5	5710	58.26	23.3
23739515	2011	7	5	5720	57.7	23.32
23739515	2011	7	5	5730	57.34	23.35
23739515	2011	7	5	5740	57.29	23.38
23739515	2011	7	5	5750	57.69	23.4
23739515	2011	7	5	5760	57.2	23.44
23739515	2011	7	5	5770	56.72	23.47
23739515	2011	7	5	5780	55.7	23.5

23739515	2011	7	5	5790	54.73	23.53
23739515	2011	7	5	5800	54.39	23.56
23739515	2011	7	5	5810	53.7	23.6
23739515	2011	7	5	5820	52.97	23.61
23739515	2011	7	5	5830	52.96	23.63
23739515	2011	7	5	5840	52.07	23.67
23739515	2011	7	5	5850	51.59	23.69
23739515	2011	7	5	5860	50.27	23.75
23739515	2011	7	5	5870	48.18	23.81
23739515	2011	7	5	5880	46.06	23.89
23739515	2011	7	5	5890	44.16	24.02
23739515	2011	7	5	5900	42.9	24.14
23739515	2011	7	5	5910	41.61	24.33
23739515	2011	7	5	5920	40.24	24.39
23739515	2011	7	5	5930	38.31	24.46
23739515	2011	7	5	5940	36.74	24.57
23739515	2011	7	5	5950	35.46	24.64
23739515	2011	7	5	5960	33.77	24.77
23739515	2011	7	5	5970	32.86	24.83
23739515	2011	7	5	5980	31.8	24.97
23739515	2011	7	5	5990	30.08	25.07
23739515	2011	7	5	6000	29.37	25.21
23739515	2011	7	5	6010	28.07	25.37
23739515	2011	7	5	6020	26.28	25.46
23739515	2011	7	5	6030	25.48	25.57
23739515	2011	7	5	6040	24.73	25.69
23739515	2011	7	5	6050	23.81	25.84
23739515	2011	7	5	6060	23.22	25.94
23739515	2011	7	5	6070	21.82	26.04
23739515	2011	7	5	6080	20.28	26.09
23739515	2011	7	5	6090	19.84	26.13
23739515	2011	7	5	6100	19.91	26.17
23739515	2011	7	5	6110	19.96	26.22
23739515	2011	7	5	6120	20.15	26.27
23739515	2011	7	5	6130	20.07	26.33
23739515	2011	7	5	6140	19.76	26.4
23739515	2011	7	5	6150	20.06	26.43
23739515	2011	7	5	6160	19.53	26.53
23739515	2011	7	5	6170	18.38	26.57
23739515	2011	7	5	6180	17.74	26.65
23739515	2011	7	5	6190	16.39	26.69
23739515	2011	7	5	6200	14.98	26.77
23739515	2011	7	5	6210	14.59	26.78
23739515	2011	7	5	6220	14.23	26.82
23739515	2011	7	5	6230	13.3	26.88
23739515	2011	7	5	6240	13.18	26.9
23739515	2011	7	5	6250	13.57	26.92

23739515	2011	7	5	6260	14.16	26.95
23739515	2011	7	5	6270	14.31	26.99
23739515	2011	7	5	6280	14.53	27
23739515	2011	7	5	6290	14.73	27.02
23739515	2011	7	5	6300	14.55	27.03
23739515	2011	7	5	6310	13.96	27.08
23739515	2011	7	5	6320	13.94	27.1
23739515	2011	7	5	6330	14.13	27.12
23739515	2011	7	5	6340	13.75	27.15
23739515	2011	7	5	6350	12.69	27.21
23739515	2011	7	5	6360	11.48	27.22
23739515	2011	7	5	6370	10.06	27.27
23739515	2011	7	5	6380	8.91	27.3
23739515	2011	7	5	6390	7.46	27.33
23739515	2011	7	5	6400	5.32	27.36
23739515	2011	7	5	6410	3.81	27.4
23739515	2011	7	5	6420	2.73	27.42
23739515	2011	7	5	6430	2.63	27.43
23739515	2011	7	5	6440	2.19	27.45
23739515	2011	7	5	6450	1.98	27.48
23739515	2011	7	5	6460	1.98	27.49
23739515	2011	7	5	6470	2.42	27.5
23739515	2011	7	5	6480	2.39	27.52
23739515	2011	7	5	6490	0.64	27.55
23739515	2011	7	5	6500	0.58	27.57
23739515	2011	7	5	6510	0.6	27.59
23739515	2011	7	5	6520	0.62	27.6
23739515	2011	7	5	6530	0.6	27.64
23739515	2011	7	5	6540	0.62	27.64
23739515	2011	7	5	6550	0.61	27.65
23739515	2011	7	5	6560	0.62	27.67
23739515	2011	7	5	6570	0.62	27.68
23739515	2011	7	5	6580	0.61	27.7
23739515	2011	7	5	6590	0.58	27.73
23739515	2011	7	5	6600	0.6	27.75
23739515	2011	7	5	6610	0.6	27.76
23739515	2011	7	5	6620	0.61	27.78
23739515	2011	7	5	6630	0.6	27.79
23835115	2011	7	6	0	1.04	28.14
23835115	2011	7	6	10	1.67	28.13
23835115	2011	7	6	20	2.5	28.11
23835115	2011	7	6	30	2.5	28.11
23835115	2011	7	6	40	2.58	28.12
23835115	2011	7	6	50	2.69	28.11
23835115	2011	7	6	60	2.9	28.12
23835115	2011	7	6	70	3.22	28.1
23835115	2011	7	6	80	3.51	28.1

23835115	2011	7	6	90	4.47	28.07
23835115	2011	7	6	100	5.7	28.06
23835115	2011	7	6	110	7.41	28.03
23835115	2011	7	6	120	8.94	28
23835115	2011	7	6	130	10.81	27.97
23835115	2011	7	6	140	11.11	27.96
23835115	2011	7	6	150	10.66	27.96
23835115	2011	7	6	160	10.7	27.96
23835115	2011	7	6	170	10.55	27.94
23835115	2011	7	6	180	10.2	27.95
23835115	2011	7	6	190	9.99	27.94
23835115	2011	7	6	200	10.18	27.93
23835115	2011	7	6	210	10.09	27.94
23835115	2011	7	6	220	9.74	27.94
23835115	2011	7	6	230	9.85	27.94
23835115	2011	7	6	240	9.8	27.92
23835115	2011	7	6	250	9.48	27.92
23835115	2011	7	6	260	8.78	27.92
23835115	2011	7	6	270	7.9	27.92
23835115	2011	7	6	280	7.63	27.93
23835115	2011	7	6	290	8.11	27.92
23835115	2011	7	6	300	7.91	27.92
23835115	2011	7	6	310	7.82	27.91
23835115	2011	7	6	320	8.73	27.89
23835115	2011	7	6	330	10.37	27.87
23835115	2011	7	6	340	12.43	27.85
23835115	2011	7	6	350	14.63	27.83
23835115	2011	7	6	360	17.19	27.81
23835115	2011	7	6	370	19.36	27.79
23835115	2011	7	6	380	21.28	27.77
23835115	2011	7	6	390	23.45	27.75
23835115	2011	7	6	400	26.29	27.73
23835115	2011	7	6	410	28.19	27.71
23835115	2011	7	6	420	29.26	27.7
23835115	2011	7	6	430	30.73	27.67
23835115	2011	7	6	440	32.43	27.67
23835115	2011	7	6	450	33.44	27.66
23835115	2011	7	6	460	34.92	27.64
23835115	2011	7	6	470	36.29	27.63
23835115	2011	7	6	480	37.58	27.61
23835115	2011	7	6	490	39.24	27.58
23835115	2011	7	6	500	40.97	27.55
23835115	2011	7	6	510	42.43	27.5
23835115	2011	7	6	520	43.95	27.4
23835115	2011	7	6	530	44.97	27.24
23835115	2011	7	6	540	45.14	27.16
23835115	2011	7	6	550	45.28	27.1

23835115	2011	7	6	560	45.81	27.01
23835115	2011	7	6	570	47.02	26.92
23835115	2011	7	6	580	47.89	26.82
23835115	2011	7	6	590	49.4	26.67
23835115	2011	7	6	600	50.38	26.57
23835115	2011	7	6	610	51.45	26.48
23835115	2011	7	6	620	52.32	26.39
23835115	2011	7	6	630	52.6	26.37
23835115	2011	7	6	640	52.8	26.35
23835115	2011	7	6	650	53.12	26.3
23835115	2011	7	6	660	52.59	26.23
23835115	2011	7	6	670	51.64	26.15
23835115	2011	7	6	680	51.54	26.08
23835115	2011	7	6	690	51.84	26.04
23835115	2011	7	6	700	52.08	26.02
23835115	2011	7	6	710	52.23	25.96
23835115	2011	7	6	720	52.17	25.94
23835115	2011	7	6	730	52.16	25.92
23835115	2011	7	6	740	52.36	25.88
23835115	2011	7	6	750	52.6	25.85
23835115	2011	7	6	760	52.71	25.81
23835115	2011	7	6	770	52.45	25.77
23835115	2011	7	6	780	52.42	25.72
23835115	2011	7	6	790	52.27	25.67
23835115	2011	7	6	800	51.98	25.64
23835115	2011	7	6	810	51.95	25.62
23835115	2011	7	6	820	52.19	25.61
23835115	2011	7	6	830	52.42	25.6
23835115	2011	7	6	840	52.66	25.57
23835115	2011	7	6	850	52.88	25.56
23835115	2011	7	6	860	53.1	25.53
23835115	2011	7	6	870	53.31	25.51
23835115	2011	7	6	880	51.98	25.5
23835115	2011	7	6	890	50.91	25.48
23835115	2011	7	6	900	50.77	25.48
23835115	2011	7	6	910	51.62	25.46
23835115	2011	7	6	920	52.13	25.44
23835115	2011	7	6	930	52.55	25.43
23835115	2011	7	6	940	53	25.41
23835115	2011	7	6	950	53.71	25.4
23835115	2011	7	6	960	53.66	25.39
23835115	2011	7	6	970	53.83	25.38
23835115	2011	7	6	980	53.9	25.37
23835115	2011	7	6	990	53.74	25.36
23835115	2011	7	6	1000	53.69	25.35
23835115	2011	7	6	1010	53.79	25.35
23835115	2011	7	6	1020	53.85	25.35

23835115	2011	7	6	1030	53.73	25.34
23835115	2011	7	6	1040	53.83	25.33
23835115	2011	7	6	1050	53.89	25.33
23835115	2011	7	6	1060	53.84	25.32
23835115	2011	7	6	1070	53.6	25.31
23835115	2011	7	6	1080	53.44	25.3
23835115	2011	7	6	1090	54.38	25.26
23835115	2011	7	6	1100	55.43	25.25
23835115	2011	7	6	1110	57.09	25.21
23835115	2011	7	6	1120	58.65	25.19
23835115	2011	7	6	1130	60.61	25.17
23835115	2011	7	6	1140	62.98	25.14
23835115	2011	7	6	1150	65.48	25.05
23835115	2011	7	6	1160	67.4	24.96
23835115	2011	7	6	1170	69	24.78
23835115	2011	7	6	1180	70.65	24.67
23835115	2011	7	6	1190	72.41	24.62
23835115	2011	7	6	1200	74.22	24.49
23835115	2011	7	6	1210	76.17	24.41
23835115	2011	7	6	1220	78.18	24.28
23835115	2011	7	6	1230	79.67	24
23835115	2011	7	6	1240	81.13	23.83
23835115	2011	7	6	1250	83.22	23.79
23835115	2011	7	6	1260	85.03	23.63
23835115	2011	7	6	1270	87.31	23.57
23835115	2011	7	6	1280	88.82	23.58
23835115	2011	7	6	1290	89.57	23.6
23835115	2011	7	6	1300	89.76	23.6
23835115	2011	7	6	1310	88.57	23.57
23835115	2011	7	6	1320	86.68	23.46
23835115	2011	7	6	1330	85.19	23.42
23835115	2011	7	6	1340	84.69	23.37
23835115	2011	7	6	1350	84.87	23.28
23835115	2011	7	6	1360	85.22	23.25
23835115	2011	7	6	1370	85.62	23.21
23835115	2011	7	6	1380	85.87	23.21
23835115	2011	7	6	1390	86.03	23.17
23835115	2011	7	6	1400	86.2	23.12
23835115	2011	7	6	1410	86.36	23.08
23835115	2011	7	6	1420	86.38	23.07
23835115	2011	7	6	1430	86.3	23.02
23835115	2011	7	6	1440	86.14	23
23835115	2011	7	6	1450	86.19	22.97
23835115	2011	7	6	1460	86.39	22.96
23835115	2011	7	6	1470	86.54	22.92
23835115	2011	7	6	1480	86.06	22.89
23835115	2011	7	6	1490	85.12	22.85

23835115	2011	7	6	1500	84.81	22.85
23835115	2011	7	6	1510	84.91	22.82
23835115	2011	7	6	1520	84.76	22.8
23835115	2011	7	6	1530	84.68	22.78
23835115	2011	7	6	1540	85.54	22.75
23835115	2011	7	6	1550	86.23	22.74
23835115	2011	7	6	1560	85.88	22.72
23835115	2011	7	6	1570	85.99	22.71
23835115	2011	7	6	1580	85.9	22.68
23835115	2011	7	6	1590	86.13	22.67
23835115	2011	7	6	1600	86.32	22.65
23835115	2011	7	6	1610	86.58	22.63
23835115	2011	7	6	1620	86.59	22.62
23835115	2011	7	6	1630	86.53	22.6
23835115	2011	7	6	1640	86.45	22.59
23835115	2011	7	6	1650	86.36	22.58
23835115	2011	7	6	1660	86.46	22.57
23835115	2011	7	6	1670	86.39	22.57
23835115	2011	7	6	1680	86.44	22.54
23835115	2011	7	6	1690	86.44	22.54
23835115	2011	7	6	1700	86.39	22.53
23835115	2011	7	6	1710	86.8	22.52
23835115	2011	7	6	1720	87.18	22.5
23835115	2011	7	6	1730	87.41	22.5
23835115	2011	7	6	1740	87.29	22.5
23835115	2011	7	6	1750	87.04	22.49
23835115	2011	7	6	1760	86.77	22.48
23835115	2011	7	6	1770	86.65	22.46
23835115	2011	7	6	1780	86.52	22.46
23835115	2011	7	6	1790	86.52	22.46
23835115	2011	7	6	1800	86.64	22.43
23835115	2011	7	6	1810	86.72	22.42
23835115	2011	7	6	1820	86.7	22.4
23835115	2011	7	6	1830	86.66	22.4
23835115	2011	7	6	1840	86.45	22.39
23835115	2011	7	6	1850	85.99	22.39
23835115	2011	7	6	1860	85.99	22.37
23835115	2011	7	6	1870	85.82	22.36
23835115	2011	7	6	1880	85.44	22.36
23835115	2011	7	6	1890	85.3	22.35
23835115	2011	7	6	1900	85.29	22.35
23835115	2011	7	6	1910	85.7	22.35
23835115	2011	7	6	1920	85.53	22.35
23835115	2011	7	6	1930	85.3	22.34
23835115	2011	7	6	1940	85.08	22.34
23835115	2011	7	6	1950	85.1	22.34
23835115	2011	7	6	1960	85.02	22.34

23835115	2011	7	6	1970	84.98	22.33
23835115	2011	7	6	1980	85.49	22.33
23835115	2011	7	6	1990	86.3	22.32
23835115	2011	7	6	2000	87.64	22.32
23835115	2011	7	6	2010	89.63	22.31
23835115	2011	7	6	2020	91.37	22.28
23835115	2011	7	6	2030	93.72	22.26
23835115	2011	7	6	2040	94.89	22.25
23835115	2011	7	6	2050	95.31	22.23
23835115	2011	7	6	2060	95.96	22.19
23835115	2011	7	6	2070	98.03	22.16
23835115	2011	7	6	2080	99.8	22.12
23835115	2011	7	6	2090	101.12	21.95
23835115	2011	7	6	2100	102.58	21.71
23835115	2011	7	6	2110	104.58	21.56
23835115	2011	7	6	2120	106.82	21.49
23835115	2011	7	6	2130	109.16	21.34
23835115	2011	7	6	2140	110.16	21.32
23835115	2011	7	6	2150	110.23	21.31
23835115	2011	7	6	2160	110.72	21.18
23835115	2011	7	6	2170	111.49	21.08
23835115	2011	7	6	2180	113.28	20.81
23835115	2011	7	6	2190	115.54	20.75
23835115	2011	7	6	2200	118.18	20.59
23835115	2011	7	6	2210	121.01	20.43
23835115	2011	7	6	2220	123.56	20.42
23835115	2011	7	6	2230	126.35	20.26
23835115	2011	7	6	2240	129.2	20.14
23835115	2011	7	6	2250	132.08	20.03
23835115	2011	7	6	2260	134.72	19.96
23835115	2011	7	6	2270	136.01	19.93
23835115	2011	7	6	2280	136.54	19.89
23835115	2011	7	6	2290	137.42	19.71
23835115	2011	7	6	2300	139.09	19.48
23835115	2011	7	6	2310	140.76	19.31
23835115	2011	7	6	2320	142.73	19.3
23835115	2011	7	6	2330	145.31	19.25
23835115	2011	7	6	2340	148.11	19.19
23835115	2011	7	6	2350	150.67	19.13
23835115	2011	7	6	2360	153.13	19.1
23835115	2011	7	6	2370	155.38	19.03
23835115	2011	7	6	2380	157.53	18.92
23835115	2011	7	6	2390	158.25	18.91
23835115	2011	7	6	2400	158.44	18.89
23835115	2011	7	6	2410	158.57	18.85
23835115	2011	7	6	2420	158.63	18.8
23835115	2011	7	6	2430	158.91	18.75

23835115	2011	7	6	2440	158.72	18.72
23835115	2011	7	6	2450	158.54	18.68
23835115	2011	7	6	2460	158.67	18.65
23835115	2011	7	6	2470	158.55	18.61
23835115	2011	7	6	2480	158.89	18.52
23835115	2011	7	6	2490	159.25	18.45
23835115	2011	7	6	2500	159.76	18.41
23835115	2011	7	6	2510	161	18.36
23835115	2011	7	6	2520	162.17	18.33
23835115	2011	7	6	2530	164.29	18.3
23835115	2011	7	6	2540	165.84	18.23
23835115	2011	7	6	2550	166.72	18.2
23835115	2011	7	6	2560	167.59	18.18
23835115	2011	7	6	2570	167.99	18.14
23835115	2011	7	6	2580	167.95	18.09
23835115	2011	7	6	2590	168.07	18.07
23835115	2011	7	6	2600	168.39	18.04
23835115	2011	7	6	2610	168.31	18.02
23835115	2011	7	6	2620	168.13	17.96
23835115	2011	7	6	2630	168.14	17.92
23835115	2011	7	6	2640	169.02	17.86
23835115	2011	7	6	2650	169.99	17.82
23835115	2011	7	6	2660	170.71	17.78
23835115	2011	7	6	2670	171	17.76
23835115	2011	7	6	2680	172.26	17.73
23835115	2011	7	6	2690	172.43	17.69
23835115	2011	7	6	2700	172.08	17.68
23835115	2011	7	6	2710	171.29	17.67
23835115	2011	7	6	2720	170.98	17.65
23835115	2011	7	6	2730	171.03	17.63
23835115	2011	7	6	2740	172.13	17.56
23835115	2011	7	6	2750	173.27	17.53
23835115	2011	7	6	2760	174.14	17.5
23835115	2011	7	6	2770	175.22	17.49
23835115	2011	7	6	2780	176.34	17.46
23835115	2011	7	6	2790	176.84	17.42
23835115	2011	7	6	2800	178.57	17.4
23835115	2011	7	6	2810	179.52	17.37
23835115	2011	7	6	2820	179.52	17.37
23835115	2011	7	6	2830	178.14	17.35
23835115	2011	7	6	2840	176.73	17.32
23835115	2011	7	6	2850	176.4	17.29
23835115	2011	7	6	2860	176.19	17.26
23835115	2011	7	6	2870	176.2	17.24
23835115	2011	7	6	2880	176.59	17.2
23835115	2011	7	6	2890	176.52	17.19
23835115	2011	7	6	2900	176.44	17.19

23835115	2011	7	6	2910	176.53	17.18
23835115	2011	7	6	2920	176.86	17.17
23835115	2011	7	6	2930	177.82	17.16
23835115	2011	7	6	2940	178.12	17.15
23835115	2011	7	6	2950	177.46	17.15
23835115	2011	7	6	2960	176.31	17.13
23835115	2011	7	6	2970	175.87	17.13
23835115	2011	7	6	2980	175.59	17.11
23835115	2011	7	6	2990	175.7	17.1
23835115	2011	7	6	3000	175.46	17.09
23835115	2011	7	6	3010	175.58	17.09
23835115	2011	7	6	3020	175.45	17.08
23835115	2011	7	6	3030	175.34	17.07
23835115	2011	7	6	3040	175.69	17.06
23835115	2011	7	6	3050	175.58	17.06
23835115	2011	7	6	3060	175.29	17.05
23835115	2011	7	6	3070	174.74	17.05
23835115	2011	7	6	3080	174.62	17.04
23835115	2011	7	6	3090	175.59	17.03
23835115	2011	7	6	3100	177.1	17.03
23835115	2011	7	6	3110	177.99	17.01
23835115	2011	7	6	3120	177.98	17.02
23835115	2011	7	6	3130	177.68	17
23835115	2011	7	6	3140	177.9	16.97
23835115	2011	7	6	3150	177.47	16.92
23835115	2011	7	6	3160	176.77	16.92
23835115	2011	7	6	3170	177.07	16.92
23835115	2011	7	6	3180	176.98	16.9
23835115	2011	7	6	3190	176.95	16.9
23835115	2011	7	6	3200	176.78	16.89
23835115	2011	7	6	3210	176.76	16.88
23835115	2011	7	6	3220	176.54	16.86
23835115	2011	7	6	3230	176.47	16.86
23835115	2011	7	6	3240	177.21	16.84
23835115	2011	7	6	3250	177.79	16.83
23835115	2011	7	6	3260	178.68	16.79
23835115	2011	7	6	3270	180.41	16.75
23835115	2011	7	6	3280	182.42	16.71
23835115	2011	7	6	3290	184.18	16.65
23835115	2011	7	6	3300	186.5	16.63
23835115	2011	7	6	3310	187.96	16.64
23835115	2011	7	6	3320	188.61	16.62
23835115	2011	7	6	3330	189.27	16.62
23835115	2011	7	6	3340	189.87	16.59
23835115	2011	7	6	3350	190.45	16.57
23835115	2011	7	6	3360	192.38	16.46
23835115	2011	7	6	3370	194.07	16.41

23835115	2011	7	6	3380	194.18	16.39
23835115	2011	7	6	3390	194	16.39
23835115	2011	7	6	3400	194.78	16.32
23835115	2011	7	6	3410	195.73	16.27
23835115	2011	7	6	3420	196.89	16.23
23835115	2011	7	6	3430	199.13	16.17
23835115	2011	7	6	3440	200.46	16.14
23835115	2011	7	6	3450	201.71	16.01
23835115	2011	7	6	3460	203.25	15.85
23835115	2011	7	6	3470	204.63	15.82
23835115	2011	7	6	3480	206.74	15.71
23835115	2011	7	6	3490	207.96	15.64
23835115	2011	7	6	3500	207.35	15.64
23835115	2011	7	6	3510	207.36	15.6
23835115	2011	7	6	3520	207.62	15.54
23835115	2011	7	6	3530	208	15.46
23835115	2011	7	6	3540	207.97	15.44
23835115	2011	7	6	3550	207.66	15.41
23835115	2011	7	6	3560	207.92	15.37
23835115	2011	7	6	3570	207.56	15.33
23835115	2011	7	6	3580	207.04	15.28
23835115	2011	7	6	3590	207.24	15.19
23835115	2011	7	6	3600	207.52	15.17
23835115	2011	7	6	3610	208.07	15.14
23835115	2011	7	6	3620	208.08	15.11
23835115	2011	7	6	3630	207.95	15.07
23835115	2011	7	6	3640	207.83	15.01
23835115	2011	7	6	3650	207.5	14.97
23835115	2011	7	6	3660	207.75	14.92
23835115	2011	7	6	3670	207.71	14.85
23835115	2011	7	6	3680	208.39	14.8
23835115	2011	7	6	3690	209.27	14.79
23835115	2011	7	6	3700	210.56	14.74
23835115	2011	7	6	3710	210.6	14.73
23835115	2011	7	6	3720	210.61	14.71
23835115	2011	7	6	3730	210.68	14.68
23835115	2011	7	6	3740	211.19	14.64
23835115	2011	7	6	3750	212.13	14.6
23835115	2011	7	6	3760	212.56	14.61
23835115	2011	7	6	3770	212.58	14.58
23835115	2011	7	6	3780	213.14	14.57
23835115	2011	7	6	3790	213.33	14.52
23835115	2011	7	6	3800	213.31	14.5
23835115	2011	7	6	3810	213.88	14.47
23835115	2011	7	6	3820	214.54	14.43
23835115	2011	7	6	3830	215.26	14.42
23835115	2011	7	6	3840	215.72	14.42

23835115	2011	7	6	3850	216.15	14.4
23835115	2011	7	6	3860	216.23	14.39
23835115	2011	7	6	3870	215.93	14.36
23835115	2011	7	6	3880	217.06	14.32
23835115	2011	7	6	3890	217.74	14.31
23835115	2011	7	6	3900	218.25	14.29
23835115	2011	7	6	3910	219.28	14.27
23835115	2011	7	6	3920	220.28	14.25
23835115	2011	7	6	3930	220.6	14.23
23835115	2011	7	6	3940	220.65	14.22
23835115	2011	7	6	3950	221.26	14.19
23835115	2011	7	6	3960	221.76	14.17
23835115	2011	7	6	3970	221.71	14.15
23835115	2011	7	6	3980	221.57	14.13
23835115	2011	7	6	3990	221.13	14.12
23835115	2011	7	6	4000	220.81	14.11
23835115	2011	7	6	4010	220.76	14.09
23835115	2011	7	6	4020	221.6	14.07
23835115	2011	7	6	4030	222.27	14.07
23835115	2011	7	6	4040	222.72	14.05
23835115	2011	7	6	4050	222.82	14.03
23835115	2011	7	6	4060	223.3	14.03
23835115	2011	7	6	4070	223.87	14.02
23835115	2011	7	6	4080	224.6	14.01
23835115	2011	7	6	4090	225.43	13.98
23835115	2011	7	6	4100	224.92	13.96
23835115	2011	7	6	4110	224.69	13.94
23835115	2011	7	6	4120	224.17	13.93
23835115	2011	7	6	4130	222.65	13.92
23835115	2011	7	6	4140	221.62	13.92
23835115	2011	7	6	4150	220.98	13.92
23835115	2011	7	6	4160	220.36	13.9
23835115	2011	7	6	4170	219.28	13.9
23835115	2011	7	6	4180	218.54	13.89
23835115	2011	7	6	4190	217.96	13.89
23835115	2011	7	6	4200	217.23	13.9
23835115	2011	7	6	4210	216.8	13.9
23835115	2011	7	6	4220	216.54	13.91
23835115	2011	7	6	4230	216.52	13.91
23835115	2011	7	6	4240	216.31	13.92
23835115	2011	7	6	4250	216.71	13.93
23835115	2011	7	6	4260	216.75	13.93
23835115	2011	7	6	4270	216.46	13.94
23835115	2011	7	6	4280	215.86	13.94
23835115	2011	7	6	4290	215.39	13.95
23835115	2011	7	6	4300	213.54	13.95
23835115	2011	7	6	4310	212.06	13.95

23835115	2011	7	6	4320	211.27	13.95
23835115	2011	7	6	4330	209.96	13.96
23835115	2011	7	6	4340	208.83	13.97
23835115	2011	7	6	4350	207.77	13.97
23835115	2011	7	6	4360	206.7	13.97
23835115	2011	7	6	4370	206.25	13.99
23835115	2011	7	6	4380	206	14
23835115	2011	7	6	4390	204.35	14.01
23835115	2011	7	6	4400	203	14.01
23835115	2011	7	6	4410	202.82	14.09
23835115	2011	7	6	4420	203.6	14.14
23835115	2011	7	6	4430	203.49	14.17
23835115	2011	7	6	4440	202.77	14.19
23835115	2011	7	6	4450	203.64	14.26
23835115	2011	7	6	4460	204.09	14.28
23835115	2011	7	6	4470	203.83	14.29
23835115	2011	7	6	4480	202.61	14.32
23835115	2011	7	6	4490	201.58	14.38
23835115	2011	7	6	4500	201.78	14.42
23835115	2011	7	6	4510	202.93	14.46
23835115	2011	7	6	4520	203.09	14.46
23835115	2011	7	6	4530	202.77	14.47
23835115	2011	7	6	4540	202.12	14.5
23835115	2011	7	6	4550	202.12	14.54
23835115	2011	7	6	4560	202.43	14.57
23835115	2011	7	6	4570	202.22	14.58
23835115	2011	7	6	4580	201.92	14.6
23835115	2011	7	6	4590	201.78	14.64
23835115	2011	7	6	4600	201.16	14.64
23835115	2011	7	6	4610	200.14	14.64
23835115	2011	7	6	4620	199.01	14.64
23835115	2011	7	6	4630	198.12	14.67
23835115	2011	7	6	4640	196.77	14.68
23835115	2011	7	6	4650	195.34	14.7
23835115	2011	7	6	4660	194.13	14.72
23835115	2011	7	6	4670	193.62	14.75
23835115	2011	7	6	4680	193.81	14.81
23835115	2011	7	6	4690	193.75	14.85
23835115	2011	7	6	4700	194.11	14.94
23835115	2011	7	6	4710	194.64	14.96
23835115	2011	7	6	4720	194.41	14.96
23835115	2011	7	6	4730	193.89	15
23835115	2011	7	6	4740	193.78	15.01
23835115	2011	7	6	4750	194.02	15.03
23835115	2011	7	6	4760	193.9	15.04
23835115	2011	7	6	4770	193.91	15.07
23835115	2011	7	6	4780	192.85	15.1

23835115	2011	7	6	4790	192.04	15.11
23835115	2011	7	6	4800	191.26	15.15
23835115	2011	7	6	4810	191.56	15.24
23835115	2011	7	6	4820	192.07	15.27
23835115	2011	7	6	4830	191.77	15.28
23835115	2011	7	6	4840	191.16	15.29
23835115	2011	7	6	4850	190.78	15.3
23835115	2011	7	6	4860	190.27	15.32
23835115	2011	7	6	4870	189.49	15.34
23835115	2011	7	6	4880	188.4	15.35
23835115	2011	7	6	4890	188.23	15.42
23835115	2011	7	6	4900	187.5	15.45
23835115	2011	7	6	4910	187.51	15.46
23835115	2011	7	6	4920	186.79	15.49
23835115	2011	7	6	4930	186.28	15.53
23835115	2011	7	6	4940	186.02	15.53
23835115	2011	7	6	4950	186.08	15.56
23835115	2011	7	6	4960	185.82	15.58
23835115	2011	7	6	4970	185.3	15.6
23835115	2011	7	6	4980	184.41	15.61
23835115	2011	7	6	4990	184.4	15.67
23835115	2011	7	6	5000	184.91	15.72
23835115	2011	7	6	5010	184.71	15.72
23835115	2011	7	6	5020	184.48	15.73
23835115	2011	7	6	5030	184.33	15.74
23835115	2011	7	6	5040	184.31	15.77
23835115	2011	7	6	5050	184.08	15.78
23835115	2011	7	6	5060	184.12	15.79
23835115	2011	7	6	5070	184.09	15.82
23835115	2011	7	6	5080	183.91	15.83
23835115	2011	7	6	5090	184.01	15.85
23835115	2011	7	6	5100	183.91	15.86
23835115	2011	7	6	5110	183.95	15.88
23835115	2011	7	6	5120	183.79	15.93
23835115	2011	7	6	5130	183.75	15.94
23835115	2011	7	6	5140	183.15	15.96
23835115	2011	7	6	5150	182.11	15.98
23835115	2011	7	6	5160	181.28	16
23835115	2011	7	6	5170	180.06	16.03
23835115	2011	7	6	5180	179.29	16.03
23835115	2011	7	6	5190	178.35	16.04
23835115	2011	7	6	5200	178.25	16.07
23835115	2011	7	6	5210	177.71	16.1
23835115	2011	7	6	5220	176.04	16.12
23835115	2011	7	6	5230	174.94	16.14
23835115	2011	7	6	5240	173.5	16.17
23835115	2011	7	6	5250	171.84	16.21

23835115	2011	7	6	5260	170.32	16.24
23835115	2011	7	6	5270	169.07	16.28
23835115	2011	7	6	5280	168.03	16.33
23835115	2011	7	6	5290	166.27	16.38
23835115	2011	7	6	5300	165.2	16.41
23835115	2011	7	6	5310	163.81	16.46
23835115	2011	7	6	5320	163.29	16.53
23835115	2011	7	6	5330	162.97	16.56
23835115	2011	7	6	5340	162.71	16.59
23835115	2011	7	6	5350	162.35	16.62
23835115	2011	7	6	5360	162.05	16.64
23835115	2011	7	6	5370	161.68	16.66
23835115	2011	7	6	5380	161.58	16.7
23835115	2011	7	6	5390	161.58	16.73
23835115	2011	7	6	5400	161.45	16.75
23835115	2011	7	6	5410	160.56	16.78
23835115	2011	7	6	5420	159.9	16.82
23835115	2011	7	6	5430	159.04	16.83
23835115	2011	7	6	5440	158.44	16.85
23835115	2011	7	6	5450	157.71	16.88
23835115	2011	7	6	5460	156.5	16.91
23835115	2011	7	6	5470	156.05	16.96
23835115	2011	7	6	5480	155.43	16.97
23835115	2011	7	6	5490	154.48	16.99
23835115	2011	7	6	5500	154.1	17.03
23835115	2011	7	6	5510	153.86	17.05
23835115	2011	7	6	5520	153.61	17.07
23835115	2011	7	6	5530	152.7	17.1
23835115	2011	7	6	5540	151.64	17.11
23835115	2011	7	6	5550	150.24	17.14
23835115	2011	7	6	5560	148.64	17.17
23835115	2011	7	6	5570	147.52	17.17
23835115	2011	7	6	5580	146.65	17.2
23835115	2011	7	6	5590	145.74	17.23
23835115	2011	7	6	5600	144.99	17.27
23835115	2011	7	6	5610	144.21	17.29
23835115	2011	7	6	5620	143.08	17.35
23835115	2011	7	6	5630	142.24	17.37
23835115	2011	7	6	5640	141.15	17.42
23835115	2011	7	6	5650	139.97	17.44
23835115	2011	7	6	5660	138.22	17.47
23835115	2011	7	6	5670	136.47	17.51
23835115	2011	7	6	5680	135.5	17.54
23835115	2011	7	6	5690	134.46	17.58
23835115	2011	7	6	5700	133.1	17.62
23835115	2011	7	6	5710	132.03	17.65
23835115	2011	7	6	5720	130.75	17.7

23835115	2011	7	6	5730	129.13	17.75
23835115	2011	7	6	5740	127.44	17.79
23835115	2011	7	6	5750	125.36	17.82
23835115	2011	7	6	5760	123.78	17.85
23835115	2011	7	6	5770	122.02	17.89
23835115	2011	7	6	5780	120.14	17.95
23835115	2011	7	6	5790	118.32	18.05
23835115	2011	7	6	5800	116.29	18.12
23835115	2011	7	6	5810	114.29	18.19
23835115	2011	7	6	5820	112.8	18.28
23835115	2011	7	6	5830	110.82	18.39
23835115	2011	7	6	5840	110.68	18.53
23835115	2011	7	6	5850	110.63	18.6
23835115	2011	7	6	5860	110.48	18.66
23835115	2011	7	6	5870	109.43	18.72
23835115	2011	7	6	5880	107.79	18.78
23835115	2011	7	6	5890	105.83	18.85
23835115	2011	7	6	5900	105.7	18.94
23835115	2011	7	6	5910	104.52	19.06
23835115	2011	7	6	5920	102.31	19.11
23835115	2011	7	6	5930	100.09	19.17
23835115	2011	7	6	5940	97.79	19.25
23835115	2011	7	6	5950	95.45	19.36
23835115	2011	7	6	5960	93.28	19.46
23835115	2011	7	6	5970	90.77	19.68
23835115	2011	7	6	5980	88.34	19.85
23835115	2011	7	6	5990	86.9	19.93
23835115	2011	7	6	6000	85.86	20.07
23835115	2011	7	6	6010	84.24	20.16
23835115	2011	7	6	6020	83.09	20.32
23835115	2011	7	6	6030	83.82	20.46
23835115	2011	7	6	6040	84.1	20.6
23835115	2011	7	6	6050	84.41	20.63
23835115	2011	7	6	6060	84.5	20.69
23835115	2011	7	6	6070	84.56	20.77
23835115	2011	7	6	6080	84.34	20.84
23835115	2011	7	6	6090	84.38	20.94
23835115	2011	7	6	6100	84.23	21.06
23835115	2011	7	6	6110	84.32	21.1
23835115	2011	7	6	6120	84.39	21.14
23835115	2011	7	6	6130	84.41	21.18
23835115	2011	7	6	6140	84.4	21.25
23835115	2011	7	6	6150	83.71	21.32
23835115	2011	7	6	6160	81.7	21.39
23835115	2011	7	6	6170	80.05	21.46
23835115	2011	7	6	6180	78.17	21.53
23835115	2011	7	6	6190	76.57	21.6

23835115	2011	7	6	6200	74.73	21.69
23835115	2011	7	6	6210	72.93	21.74
23835115	2011	7	6	6220	71.7	21.78
23835115	2011	7	6	6230	70.79	21.83
23835115	2011	7	6	6240	69.68	21.92
23835115	2011	7	6	6250	67.63	22.04
23835115	2011	7	6	6260	65.38	22.12
23835115	2011	7	6	6270	62.88	22.21
23835115	2011	7	6	6280	60.3	22.28
23835115	2011	7	6	6290	58.06	22.41
23835115	2011	7	6	6300	55.39	22.6
23835115	2011	7	6	6310	52.37	22.71
23835115	2011	7	6	6320	49.3	22.84
23835115	2011	7	6	6330	46.3	23.03
23835115	2011	7	6	6340	44.55	23.16
23835115	2011	7	6	6350	45.25	23.36
23835115	2011	7	6	6360	45.57	23.48
23835115	2011	7	6	6370	46.08	23.53
23835115	2011	7	6	6380	46.86	23.68
23835115	2011	7	6	6390	47.1	23.75
23835115	2011	7	6	6400	47.15	23.78
23835115	2011	7	6	6410	47.03	23.82
23835115	2011	7	6	6420	46.79	23.89
23835115	2011	7	6	6430	46.81	23.96
23835115	2011	7	6	6440	47.35	24.03
23835115	2011	7	6	6450	46.95	24.12
23835115	2011	7	6	6460	46.88	24.14
23835115	2011	7	6	6470	45.32	24.21
23835115	2011	7	6	6480	44.66	24.32
23835115	2011	7	6	6490	44.8	24.34
23835115	2011	7	6	6500	45.22	24.42
23835115	2011	7	6	6510	45.94	24.48
23835115	2011	7	6	6520	45.84	24.48
23835115	2011	7	6	6530	45.53	24.5
23835115	2011	7	6	6540	46.03	24.51
23835115	2011	7	6	6550	46.57	24.52
23835115	2011	7	6	6560	46.7	24.53
23835115	2011	7	6	6570	47.07	24.57
23835115	2011	7	6	6580	47.67	24.6
23835115	2011	7	6	6590	48.45	24.66
23835115	2011	7	6	6600	47.91	24.68
23835115	2011	7	6	6610	47.24	24.72
23835115	2011	7	6	6620	46.89	24.76
23835115	2011	7	6	6630	45.65	24.8
23835115	2011	7	6	6640	44.81	24.83
23835115	2011	7	6	6650	45.66	24.91
23835115	2011	7	6	6660	46.24	24.96

23835115	2011	7	6	6670	46.73	24.96
23835115	2011	7	6	6680	46.81	24.99
23835115	2011	7	6	6690	46.6	25
23835115	2011	7	6	6700	46.37	25.03
23835115	2011	7	6	6710	46.47	25.05
23835115	2011	7	6	6720	46.36	25.06
23835115	2011	7	6	6730	46.58	25.07
23835115	2011	7	6	6740	46.95	25.07
23835115	2011	7	6	6750	46.82	25.07
23835115	2011	7	6	6760	46.74	25.09
23835115	2011	7	6	6770	46.91	25.1
23835115	2011	7	6	6780	47.3	25.1
23835115	2011	7	6	6790	47.7	25.1
23835115	2011	7	6	6800	48.03	25.11
23835115	2011	7	6	6810	48.42	25.11
23835115	2011	7	6	6820	48.54	25.11
23835115	2011	7	6	6830	48.58	25.1
23835115	2011	7	6	6840	48.71	25.11
23835115	2011	7	6	6850	48.94	25.11
23835115	2011	7	6	6860	49.22	25.11
23835115	2011	7	6	6870	49.5	25.12
23835115	2011	7	6	6880	49.57	25.12
23835115	2011	7	6	6890	49.68	25.13
23835115	2011	7	6	6900	49.87	25.12
23835115	2011	7	6	6910	50.19	25.12
23835115	2011	7	6	6920	50.13	25.12
23835115	2011	7	6	6930	49.8	25.11
23835115	2011	7	6	6940	49.65	25.11
23835115	2011	7	6	6950	49.67	25.11
23835115	2011	7	6	6960	49.81	25.11
23835115	2011	7	6	6970	49.42	25.12
23835115	2011	7	6	6980	48.79	25.12
23835115	2011	7	6	6990	48.83	25.12
23835115	2011	7	6	7000	47.96	25.14
23835115	2011	7	6	7010	46.35	25.14
23835115	2011	7	6	7020	46.03	25.16
23835115	2011	7	6	7030	45.09	25.17
23835115	2011	7	6	7040	43.3	25.2
23835115	2011	7	6	7050	42.4	25.25
23835115	2011	7	6	7060	42.44	25.29
23835115	2011	7	6	7070	41.67	25.34
23835115	2011	7	6	7080	40.31	25.39
23835115	2011	7	6	7090	39.55	25.46
23835115	2011	7	6	7100	37.88	25.5
23835115	2011	7	6	7110	36.72	25.58
23835115	2011	7	6	7120	34.93	25.67
23835115	2011	7	6	7130	33.11	25.81

23835115	2011	7	6	7140	31.21	25.9
23835115	2011	7	6	7150	29.23	25.96
23835115	2011	7	6	7160	26.92	26.1
23835115	2011	7	6	7170	24.59	26.18
23835115	2011	7	6	7180	22.36	26.28
23835115	2011	7	6	7190	20.18	26.41
23835115	2011	7	6	7200	18.32	26.5
23835115	2011	7	6	7210	18.13	26.71
23835115	2011	7	6	7220	17.92	26.78
23835115	2011	7	6	7230	17.99	26.86
23835115	2011	7	6	7240	18.16	26.89
23835115	2011	7	6	7250	18.29	26.92
23835115	2011	7	6	7260	18.55	26.99
23835115	2011	7	6	7270	19.07	27.05
23835115	2011	7	6	7280	19.38	27.07
23835115	2011	7	6	7290	19.36	27.07
23835115	2011	7	6	7300	18.27	27.12
23835115	2011	7	6	7310	16.31	27.18
23835115	2011	7	6	7320	14.03	27.21
23835115	2011	7	6	7330	13.52	27.25
23835115	2011	7	6	7340	13.83	27.27
23835115	2011	7	6	7350	13.89	27.27
23835115	2011	7	6	7360	14.07	27.28
23835115	2011	7	6	7370	12.87	27.33
23835115	2011	7	6	7380	10.79	27.37
23835115	2011	7	6	7390	8.63	27.42
23835115	2011	7	6	7400	6.19	27.46
23835115	2011	7	6	7410	4.88	27.51
23835115	2011	7	6	7420	4.31	27.54
23835115	2011	7	6	7430	3.71	27.56
23835115	2011	7	6	7440	3.17	27.59
23835115	2011	7	6	7450	2.72	27.63
23835115	2011	7	6	7460	2.28	27.65
23835115	2011	7	6	7470	2.47	27.67
23835115	2011	7	6	7480	2.08	27.69
23835115	2011	7	6	7490	2.34	27.7
23835115	2011	7	6	7500	0.64	27.73
23835115	2011	7	6	7510	0.6	27.75
23835115	2011	7	6	7520	0.6	27.76
23835115	2011	7	6	7530	0.6	27.82
23835115	2011	7	6	7540	0.61	27.84
23835115	2011	7	6	7550	0.61	27.86
23835115	2011	7	6	7560	0.6	27.89
23835115	2011	7	6	7570	0.6	27.92
23835115	2011	7	6	7580	0.63	27.92
23835115	2011	7	6	7590	0.64	27.94
23835115	2011	7	6	7600	0.51	27.96

23835115	2011	7	6	7610	0.51	27.99
23835115	2011	7	6	7620	0.49	28
23835115	2011	7	6	7630	0.48	28.03
23835115	2011	7	6	7640	0.48	28.03
23930415	2011	7	7	0	1.01	28.47
23930415	2011	7	7	10	1.2	28.48
23930415	2011	7	7	20	1.77	28.46
23930415	2011	7	7	30	2.55	28.46
23930415	2011	7	7	40	2.77	28.46
23930415	2011	7	7	50	2.71	28.46
23930415	2011	7	7	60	2.68	28.46
23930415	2011	7	7	70	2.78	28.46
23930415	2011	7	7	80	2.72	28.46
23930415	2011	7	7	90	2.57	28.45
23930415	2011	7	7	100	2.69	28.44
23930415	2011	7	7	110	2.85	28.42
23930415	2011	7	7	120	3.1	28.42
23930415	2011	7	7	130	3.24	28.39
23930415	2011	7	7	140	4.39	28.36
23930415	2011	7	7	150	6.59	28.33
23930415	2011	7	7	160	7.48	28.3
23930415	2011	7	7	170	7.6	28.27
23930415	2011	7	7	180	8.72	28.25
23930415	2011	7	7	190	10.82	28.21
23930415	2011	7	7	200	12.05	28.19
23930415	2011	7	7	210	12.21	28.17
23930415	2011	7	7	220	12.12	28.16
23930415	2011	7	7	230	11.91	28.15
23930415	2011	7	7	240	11.65	28.14
23930415	2011	7	7	250	11.7	28.13
23930415	2011	7	7	260	11.9	28.12
23930415	2011	7	7	270	11.84	28.1
23930415	2011	7	7	280	11.69	28.1
23930415	2011	7	7	290	11.77	28.09
23930415	2011	7	7	300	11.81	28.08
23930415	2011	7	7	310	11.73	28.07
23930415	2011	7	7	320	11.68	28.06
23930415	2011	7	7	330	11.77	28.04
23930415	2011	7	7	340	11.85	28.03
23930415	2011	7	7	350	11.77	28.03
23930415	2011	7	7	360	11.7	28.02
23930415	2011	7	7	370	11.71	28.02
23930415	2011	7	7	380	11.77	28
23930415	2011	7	7	390	11.74	28
23930415	2011	7	7	400	11.72	27.99
23930415	2011	7	7	410	11.77	27.98
23930415	2011	7	7	420	11.8	27.97

23930415	2011	7	7	430	11.73	27.97
23930415	2011	7	7	440	12.05	27.96
23930415	2011	7	7	450	12.22	27.96
23930415	2011	7	7	460	11.95	27.95
23930415	2011	7	7	470	11.9	27.94
23930415	2011	7	7	480	13.55	27.94
23930415	2011	7	7	490	15.83	27.92
23930415	2011	7	7	500	16.9	27.9
23930415	2011	7	7	510	17.69	27.88
23930415	2011	7	7	520	18.03	27.88
23930415	2011	7	7	530	19.17	27.87
23930415	2011	7	7	540	21.63	27.83
23930415	2011	7	7	550	23.17	27.82
23930415	2011	7	7	560	23.96	27.81
23930415	2011	7	7	570	24.22	27.8
23930415	2011	7	7	580	24.27	27.8
23930415	2011	7	7	590	25.93	27.78
23930415	2011	7	7	600	28.28	27.75
23930415	2011	7	7	610	29.37	27.73
23930415	2011	7	7	620	30.38	27.71
23930415	2011	7	7	630	32.93	27.69
23930415	2011	7	7	640	35.67	27.67
23930415	2011	7	7	650	38.05	27.64
23930415	2011	7	7	660	38.39	27.62
23930415	2011	7	7	670	38.51	27.63
23930415	2011	7	7	680	38.63	27.62
23930415	2011	7	7	690	38.71	27.61
23930415	2011	7	7	700	39.77	27.61
23930415	2011	7	7	710	41.82	27.59
23930415	2011	7	7	720	42.96	27.57
23930415	2011	7	7	730	43.79	27.53
23930415	2011	7	7	740	46.06	27.51
23930415	2011	7	7	750	49.03	27.47
23930415	2011	7	7	760	50.9	27.44
23930415	2011	7	7	770	51.75	27.42
23930415	2011	7	7	780	53.86	27.32
23930415	2011	7	7	790	56.27	27.28
23930415	2011	7	7	800	57.38	27.25
23930415	2011	7	7	810	59.07	27.11
23930415	2011	7	7	820	61.26	27
23930415	2011	7	7	830	62.25	26.93
23930415	2011	7	7	840	62.77	26.85
23930415	2011	7	7	850	62.89	26.74
23930415	2011	7	7	860	62.46	26.69
23930415	2011	7	7	870	61.88	26.56
23930415	2011	7	7	880	61.37	26.54
23930415	2011	7	7	890	60.9	26.51

23930415	2011	7	7	900	60.46	26.47
23930415	2011	7	7	910	60.01	26.43
23930415	2011	7	7	920	59.66	26.39
23930415	2011	7	7	930	60.07	26.36
23930415	2011	7	7	940	62.03	26.25
23930415	2011	7	7	950	63.16	26.2
23930415	2011	7	7	960	65.45	26.1
23930415	2011	7	7	970	67.25	26.03
23930415	2011	7	7	980	68.19	25.99
23930415	2011	7	7	990	68.6	25.95
23930415	2011	7	7	1000	69.01	25.89
23930415	2011	7	7	1010	70.07	25.78
23930415	2011	7	7	1020	70.61	25.68
23930415	2011	7	7	1030	70.86	25.65
23930415	2011	7	7	1040	71.05	25.57
23930415	2011	7	7	1050	71.05	25.48
23930415	2011	7	7	1060	70.99	25.41
23930415	2011	7	7	1070	71.9	25.31
23930415	2011	7	7	1080	72.17	25.23
23930415	2011	7	7	1090	72.07	25.19
23930415	2011	7	7	1100	71.94	25.11
23930415	2011	7	7	1110	71.53	25.05
23930415	2011	7	7	1120	71.04	25.06
23930415	2011	7	7	1130	70.75	25.03
23930415	2011	7	7	1140	71.04	24.99
23930415	2011	7	7	1150	71.41	24.97
23930415	2011	7	7	1160	71.31	24.93
23930415	2011	7	7	1170	70.89	24.91
23930415	2011	7	7	1180	71.31	24.86
23930415	2011	7	7	1190	71.31	24.85
23930415	2011	7	7	1200	71.75	24.83
23930415	2011	7	7	1210	72.16	24.8
23930415	2011	7	7	1220	72.09	24.79
23930415	2011	7	7	1230	72.21	24.75
23930415	2011	7	7	1240	72.46	24.72
23930415	2011	7	7	1250	74.12	24.67
23930415	2011	7	7	1260	75.37	24.64
23930415	2011	7	7	1270	75.25	24.6
23930415	2011	7	7	1280	75.33	24.58
23930415	2011	7	7	1290	75.47	24.57
23930415	2011	7	7	1300	75.39	24.53
23930415	2011	7	7	1310	75.29	24.49
23930415	2011	7	7	1320	75.11	24.46
23930415	2011	7	7	1330	74.77	24.43
23930415	2011	7	7	1340	74.51	24.42
23930415	2011	7	7	1350	74.3	24.39
23930415	2011	7	7	1360	74.04	24.37

23930415	2011	7	7	1370	73.97	24.33
23930415	2011	7	7	1380	73.85	24.32
23930415	2011	7	7	1390	73.92	24.32
23930415	2011	7	7	1400	74.08	24.29
23930415	2011	7	7	1410	73.96	24.28
23930415	2011	7	7	1420	73.89	24.25
23930415	2011	7	7	1430	73.87	24.25
23930415	2011	7	7	1440	73.97	24.24
23930415	2011	7	7	1450	73.93	24.24
23930415	2011	7	7	1460	74.02	24.23
23930415	2011	7	7	1470	74	24.21
23930415	2011	7	7	1480	74.25	24.19
23930415	2011	7	7	1490	76.06	24.17
23930415	2011	7	7	1500	77.5	24.14
23930415	2011	7	7	1510	78.7	24.1
23930415	2011	7	7	1520	80.98	24.05
23930415	2011	7	7	1530	83.4	23.98
23930415	2011	7	7	1540	85.3	23.79
23930415	2011	7	7	1550	87.8	23.52
23930415	2011	7	7	1560	89.81	23.3
23930415	2011	7	7	1570	91.38	23.03
23930415	2011	7	7	1580	93.68	22.96
23930415	2011	7	7	1590	96.4	22.75
23930415	2011	7	7	1600	99.36	22.61
23930415	2011	7	7	1610	102.47	22.41
23930415	2011	7	7	1620	104.99	22.38
23930415	2011	7	7	1630	106.96	22.2
23930415	2011	7	7	1640	107.75	22.03
23930415	2011	7	7	1650	107.59	21.98
23930415	2011	7	7	1660	107.65	21.92
23930415	2011	7	7	1670	108.21	21.72
23930415	2011	7	7	1680	108.64	21.46
23930415	2011	7	7	1690	109.12	21.22
23930415	2011	7	7	1700	110.49	21.21
23930415	2011	7	7	1710	112.56	21.07
23930415	2011	7	7	1720	114.64	21
23930415	2011	7	7	1730	116.26	20.9
23930415	2011	7	7	1740	116.56	20.78
23930415	2011	7	7	1750	117.39	20.66
23930415	2011	7	7	1760	117.48	20.54
23930415	2011	7	7	1770	116.96	20.52
23930415	2011	7	7	1780	118.24	20.39
23930415	2011	7	7	1790	120.5	20.33
23930415	2011	7	7	1800	121.51	20.29
23930415	2011	7	7	1810	121.3	20.25
23930415	2011	7	7	1820	120.25	20.17
23930415	2011	7	7	1830	119.51	20.14

23930415	2011	7	7	1840	119.89	20.07
23930415	2011	7	7	1850	120.07	20.01
23930415	2011	7	7	1860	120.61	19.81
23930415	2011	7	7	1870	122.21	19.77
23930415	2011	7	7	1880	124.62	19.67
23930415	2011	7	7	1890	126	19.63
23930415	2011	7	7	1900	125.52	19.59
23930415	2011	7	7	1910	125.3	19.53
23930415	2011	7	7	1920	125.56	19.5
23930415	2011	7	7	1930	125.38	19.47
23930415	2011	7	7	1940	125.28	19.44
23930415	2011	7	7	1950	125.35	19.41
23930415	2011	7	7	1960	125.46	19.39
23930415	2011	7	7	1970	125.39	19.35
23930415	2011	7	7	1980	125.41	19.32
23930415	2011	7	7	1990	127.02	19.24
23930415	2011	7	7	2000	128.16	19.18
23930415	2011	7	7	2010	128.3	19.14
23930415	2011	7	7	2020	128.02	19.12
23930415	2011	7	7	2030	128.16	19.08
23930415	2011	7	7	2040	128.84	19.05
23930415	2011	7	7	2050	128.95	19.02
23930415	2011	7	7	2060	128.81	19
23930415	2011	7	7	2070	128.66	18.95
23930415	2011	7	7	2080	128.09	18.92
23930415	2011	7	7	2090	126.99	18.89
23930415	2011	7	7	2100	124.4	18.85
23930415	2011	7	7	2110	122.31	18.82
23930415	2011	7	7	2120	120.67	18.82
23930415	2011	7	7	2130	119.37	18.81
23930415	2011	7	7	2140	118.19	18.8
23930415	2011	7	7	2150	117.46	18.8
23930415	2011	7	7	2160	117.31	18.8
23930415	2011	7	7	2170	117.27	18.79
23930415	2011	7	7	2180	116.71	18.79
23930415	2011	7	7	2190	115.62	18.8
23930415	2011	7	7	2200	115.09	18.8
23930415	2011	7	7	2210	114.45	18.82
23930415	2011	7	7	2220	113.72	18.83
23930415	2011	7	7	2230	113.07	18.84
23930415	2011	7	7	2240	112.4	18.85
23930415	2011	7	7	2250	111.71	18.87
23930415	2011	7	7	2260	111.46	18.89
23930415	2011	7	7	2270	111.52	18.89
23930415	2011	7	7	2280	111.75	18.91
23930415	2011	7	7	2290	112.08	18.92
23930415	2011	7	7	2300	112.7	18.92

23930415	2011	7	7	2310	113.63	18.94
23930415	2011	7	7	2320	113.91	18.95
23930415	2011	7	7	2330	114.2	18.95
23930415	2011	7	7	2340	115.51	18.95
23930415	2011	7	7	2350	116.64	18.95
23930415	2011	7	7	2360	118.32	18.95
23930415	2011	7	7	2370	119.95	18.92
23930415	2011	7	7	2380	120.64	18.92
23930415	2011	7	7	2390	120.39	18.9
23930415	2011	7	7	2400	120.41	18.89
23930415	2011	7	7	2410	120.39	18.87
23930415	2011	7	7	2420	120.33	18.85
23930415	2011	7	7	2430	120.36	18.82
23930415	2011	7	7	2440	120.35	18.8
23930415	2011	7	7	2450	120.28	18.78
23930415	2011	7	7	2460	120.33	18.77
23930415	2011	7	7	2470	120.36	18.75
23930415	2011	7	7	2480	120.46	18.73
23930415	2011	7	7	2490	120.4	18.72
23930415	2011	7	7	2500	120.32	18.69
23930415	2011	7	7	2510	120.31	18.68
23930415	2011	7	7	2520	120.38	18.67
23930415	2011	7	7	2530	120.23	18.65
23930415	2011	7	7	2540	120.33	18.64
23930415	2011	7	7	2550	120.34	18.63
23930415	2011	7	7	2560	120.29	18.61
23930415	2011	7	7	2570	120.43	18.6
23930415	2011	7	7	2580	120.4	18.6
23930415	2011	7	7	2590	120.39	18.59
23930415	2011	7	7	2600	120.4	18.57
23930415	2011	7	7	2610	120.42	18.57
23930415	2011	7	7	2620	120.2	18.56
23930415	2011	7	7	2630	119.69	18.55
23930415	2011	7	7	2640	119.67	18.55
23930415	2011	7	7	2650	119.5	18.54
23930415	2011	7	7	2660	119.4	18.53
23930415	2011	7	7	2670	119.47	18.54
23930415	2011	7	7	2680	119.78	18.53
23930415	2011	7	7	2690	120.17	18.53
23930415	2011	7	7	2700	120.59	18.52
23930415	2011	7	7	2710	120.96	18.52
23930415	2011	7	7	2720	121.22	18.52
23930415	2011	7	7	2730	121.21	18.53
23930415	2011	7	7	2740	120.86	18.52
23930415	2011	7	7	2750	120.69	18.52
23930415	2011	7	7	2760	120.69	18.52
23930415	2011	7	7	2770	120.59	18.52

23930415	2011	7	7	2780	120.54	18.53
23930415	2011	7	7	2790	120.5	18.53
23930415	2011	7	7	2800	120.38	18.53
23930415	2011	7	7	2810	120.31	18.53
23930415	2011	7	7	2820	120.2	18.52
23930415	2011	7	7	2830	120.06	18.53
23930415	2011	7	7	2840	120.02	18.52
23930415	2011	7	7	2850	120	18.53
23930415	2011	7	7	2860	119.71	18.53
23930415	2011	7	7	2870	117.98	18.53
23930415	2011	7	7	2880	115.91	18.53
23930415	2011	7	7	2890	113.55	18.53
23930415	2011	7	7	2900	111.28	18.54
23930415	2011	7	7	2910	109.06	18.57
23930415	2011	7	7	2920	106.85	18.6
23930415	2011	7	7	2930	104.92	18.64
23930415	2011	7	7	2940	103.54	18.66
23930415	2011	7	7	2950	101.44	18.71
23930415	2011	7	7	2960	99.6	18.76
23930415	2011	7	7	2970	98.23	18.82
23930415	2011	7	7	2980	96.52	18.87
23930415	2011	7	7	2990	95.78	18.93
23930415	2011	7	7	3000	95.86	18.96
23930415	2011	7	7	3010	96.02	19.01
23930415	2011	7	7	3020	96.24	19.04
23930415	2011	7	7	3030	95.18	19.11
23930415	2011	7	7	3040	93.24	19.19
23930415	2011	7	7	3050	91.51	19.29
23930415	2011	7	7	3060	90.45	19.33
23930415	2011	7	7	3070	89.59	19.39
23930415	2011	7	7	3080	88.9	19.43
23930415	2011	7	7	3090	88.8	19.51
23930415	2011	7	7	3100	88.68	19.59
23930415	2011	7	7	3110	88.78	19.66
23930415	2011	7	7	3120	88.77	19.71
23930415	2011	7	7	3130	88.68	19.85
23930415	2011	7	7	3140	88.72	19.93
23930415	2011	7	7	3150	88.72	19.96
23930415	2011	7	7	3160	88.71	19.99
23930415	2011	7	7	3170	88.78	20.03
23930415	2011	7	7	3180	88.07	20.08
23930415	2011	7	7	3190	87.01	20.15
23930415	2011	7	7	3200	86.42	20.19
23930415	2011	7	7	3210	86.54	20.21
23930415	2011	7	7	3220	86.65	20.27
23930415	2011	7	7	3230	86.55	20.29
23930415	2011	7	7	3240	86.62	20.32

23930415	2011	7	7	3250	86.58	20.35
23930415	2011	7	7	3260	86.55	20.39
23930415	2011	7	7	3270	86.6	20.39
23930415	2011	7	7	3280	86.62	20.43
23930415	2011	7	7	3290	86.66	20.44
23930415	2011	7	7	3300	86.51	20.46
23930415	2011	7	7	3310	86.35	20.5
23930415	2011	7	7	3320	86.6	20.51
23930415	2011	7	7	3330	86.75	20.54
23930415	2011	7	7	3340	86.54	20.57
23930415	2011	7	7	3350	86.66	20.59
23930415	2011	7	7	3360	86.5	20.6
23930415	2011	7	7	3370	86.63	20.63
23930415	2011	7	7	3380	86.69	20.64
23930415	2011	7	7	3390	86.53	20.64
23930415	2011	7	7	3400	86.52	20.66
23930415	2011	7	7	3410	86.64	20.67
23930415	2011	7	7	3420	86.63	20.68
23930415	2011	7	7	3430	86.71	20.71
23930415	2011	7	7	3440	86.55	20.72
23930415	2011	7	7	3450	86.51	20.73
23930415	2011	7	7	3460	86.6	20.73
23930415	2011	7	7	3470	86.62	20.74
23930415	2011	7	7	3480	86.55	20.75
23930415	2011	7	7	3490	86.57	20.75
23930415	2011	7	7	3500	86.43	20.77
23930415	2011	7	7	3510	87.38	20.78
23930415	2011	7	7	3520	88.15	20.78
23930415	2011	7	7	3530	88.53	20.78
23930415	2011	7	7	3540	88.55	20.78
23930415	2011	7	7	3550	88.22	20.8
23930415	2011	7	7	3560	87.77	20.8
23930415	2011	7	7	3570	87.94	20.81
23930415	2011	7	7	3580	87.85	20.81
23930415	2011	7	7	3590	87.46	20.82
23930415	2011	7	7	3600	86.84	20.82
23930415	2011	7	7	3610	86.39	20.82
23930415	2011	7	7	3620	86.18	20.82
23930415	2011	7	7	3630	86.32	20.82
23930415	2011	7	7	3640	86.44	20.83
23930415	2011	7	7	3650	86.33	20.83
23930415	2011	7	7	3660	86.13	20.84
23930415	2011	7	7	3670	85.71	20.85
23930415	2011	7	7	3680	85.07	20.85
23930415	2011	7	7	3690	84.28	20.85
23930415	2011	7	7	3700	83.4	20.85
23930415	2011	7	7	3710	82.66	20.86

23930415	2011	7	7	3720	82.8	20.87
23930415	2011	7	7	3730	83.02	20.89
23930415	2011	7	7	3740	82.99	20.9
23930415	2011	7	7	3750	83.1	20.94
23930415	2011	7	7	3760	82.97	20.95
23930415	2011	7	7	3770	82.81	20.97
23930415	2011	7	7	3780	82.91	21
23930415	2011	7	7	3790	82.88	21.01
23930415	2011	7	7	3800	82.97	21.03
23930415	2011	7	7	3810	83.09	21.07
23930415	2011	7	7	3820	82.97	21.1
23930415	2011	7	7	3830	82.93	21.16
23930415	2011	7	7	3840	82.94	21.21
23930415	2011	7	7	3850	83.01	21.27
23930415	2011	7	7	3860	83.07	21.31
23930415	2011	7	7	3870	82.97	21.35
23930415	2011	7	7	3880	83.07	21.39
23930415	2011	7	7	3890	83.03	21.45
23930415	2011	7	7	3900	83	21.5
23930415	2011	7	7	3910	82.99	21.54
23930415	2011	7	7	3920	82.92	21.56
23930415	2011	7	7	3930	82.88	21.57
23930415	2011	7	7	3940	82.91	21.58
23930415	2011	7	7	3950	82.93	21.6
23930415	2011	7	7	3960	83.18	21.61
23930415	2011	7	7	3970	83.09	21.61
23930415	2011	7	7	3980	82.68	21.63
23930415	2011	7	7	3990	82.08	21.64
23930415	2011	7	7	4000	81.67	21.65
23930415	2011	7	7	4010	82.43	21.67
23930415	2011	7	7	4020	82.9	21.67
23930415	2011	7	7	4030	82.84	21.67
23930415	2011	7	7	4040	82.46	21.69
23930415	2011	7	7	4050	82.49	21.7
23930415	2011	7	7	4060	82.79	21.71
23930415	2011	7	7	4070	82.95	21.71
23930415	2011	7	7	4080	82.9	21.71
23930415	2011	7	7	4090	82.84	21.71
23930415	2011	7	7	4100	82.18	21.74
23930415	2011	7	7	4110	80.87	21.75
23930415	2011	7	7	4120	80.79	21.75
23930415	2011	7	7	4130	80.54	21.77
23930415	2011	7	7	4140	80.64	21.78
23930415	2011	7	7	4150	80.43	21.81
23930415	2011	7	7	4160	80.28	21.82
23930415	2011	7	7	4170	80.39	21.84
23930415	2011	7	7	4180	80.83	21.84

23930415	2011	7	7	4190	81.19	21.85
23930415	2011	7	7	4200	81.4	21.85
23930415	2011	7	7	4210	81.73	21.85
23930415	2011	7	7	4220	83.39	21.85
23930415	2011	7	7	4230	84.49	21.85
23930415	2011	7	7	4240	84.9	21.82
23930415	2011	7	7	4250	85.34	21.82
23930415	2011	7	7	4260	85.34	21.79
23930415	2011	7	7	4270	85.34	21.77
23930415	2011	7	7	4280	84.93	21.75
23930415	2011	7	7	4290	85.29	21.72
23930415	2011	7	7	4300	86.04	21.68
23930415	2011	7	7	4310	85.71	21.66
23930415	2011	7	7	4320	85.13	21.65
23930415	2011	7	7	4330	85	21.65
23930415	2011	7	7	4340	85.26	21.65
23930415	2011	7	7	4350	85.12	21.64
23930415	2011	7	7	4360	84.93	21.64
23930415	2011	7	7	4370	85.12	21.63
23930415	2011	7	7	4380	85.01	21.62
23930415	2011	7	7	4390	84.77	21.62
23930415	2011	7	7	4400	82.88	21.61
23930415	2011	7	7	4410	80.92	21.62
23930415	2011	7	7	4420	78.86	21.65
23930415	2011	7	7	4430	76.55	21.67
23930415	2011	7	7	4440	74.69	21.76
23930415	2011	7	7	4450	72.63	21.86
23930415	2011	7	7	4460	71.52	21.96
23930415	2011	7	7	4470	70.45	22.13
23930415	2011	7	7	4480	68.22	22.18
23930415	2011	7	7	4490	65.6	22.28
23930415	2011	7	7	4500	63.49	22.38
23930415	2011	7	7	4510	63.11	22.43
23930415	2011	7	7	4520	62.52	22.57
23930415	2011	7	7	4530	60.88	22.68
23930415	2011	7	7	4540	59.67	22.82
23930415	2011	7	7	4550	58.01	23.01
23930415	2011	7	7	4560	55.91	23.1
23930415	2011	7	7	4570	53.17	23.25
23930415	2011	7	7	4580	51.7	23.39
23930415	2011	7	7	4590	51.15	23.53
23930415	2011	7	7	4600	51.52	23.73
23930415	2011	7	7	4610	52.15	23.85
23930415	2011	7	7	4620	52.44	24.1
23930415	2011	7	7	4630	51.98	24.25
23930415	2011	7	7	4640	51.24	24.27
23930415	2011	7	7	4650	51.15	24.42

23930415	2011	7	7	4660	51.23	24.63
23930415	2011	7	7	4670	51.26	24.94
23930415	2011	7	7	4680	51.23	25.03
23930415	2011	7	7	4690	51.1	25.07
23930415	2011	7	7	4700	51.01	25.03
23930415	2011	7	7	4710	51.27	25.06
23930415	2011	7	7	4720	51.03	25.13
23930415	2011	7	7	4730	51.22	25.19
23930415	2011	7	7	4740	50.44	25.28
23930415	2011	7	7	4750	48.82	25.36
23930415	2011	7	7	4760	46.95	25.68
23930415	2011	7	7	4770	45.44	25.87
23930415	2011	7	7	4780	45.3	25.85
23930415	2011	7	7	4790	45.35	25.87
23930415	2011	7	7	4800	45.57	25.95
23930415	2011	7	7	4810	45.94	25.98
23930415	2011	7	7	4820	46.28	26.07
23930415	2011	7	7	4830	46.57	26.17
23930415	2011	7	7	4840	46.63	26.21
23930415	2011	7	7	4850	46.75	26.27
23930415	2011	7	7	4860	46.65	26.33
23930415	2011	7	7	4870	46.39	26.4
23930415	2011	7	7	4880	46.19	26.43
23930415	2011	7	7	4890	45.95	26.46
23930415	2011	7	7	4900	45.89	26.52
23930415	2011	7	7	4910	45.89	26.59
23930415	2011	7	7	4920	45.56	26.65
23930415	2011	7	7	4930	45.45	26.75
23930415	2011	7	7	4940	45.37	26.78
23930415	2011	7	7	4950	45.54	26.78
23930415	2011	7	7	4960	45.68	26.79
23930415	2011	7	7	4970	45.84	26.82
23930415	2011	7	7	4980	46.05	26.84
23930415	2011	7	7	4990	46.31	26.85
23930415	2011	7	7	5000	46.65	26.88
23930415	2011	7	7	5010	46.22	26.94
23930415	2011	7	7	5020	45.3	26.99
23930415	2011	7	7	5030	44.92	27.02
23930415	2011	7	7	5040	44.8	27.03
23930415	2011	7	7	5050	44.83	27.06
23930415	2011	7	7	5060	45.08	27.07
23930415	2011	7	7	5070	45.71	27.08
23930415	2011	7	7	5080	46.43	27.1
23930415	2011	7	7	5090	46.7	27.15
23930415	2011	7	7	5100	45.45	27.19
23930415	2011	7	7	5110	45.2	27.2
23930415	2011	7	7	5120	44.89	27.22

23930415	2011	7	7	5130	43.53	27.25
23930415	2011	7	7	5140	43.91	27.28
23930415	2011	7	7	5150	44.02	27.29
23930415	2011	7	7	5160	43.6	27.31
23930415	2011	7	7	5170	43.12	27.33
23930415	2011	7	7	5180	43.47	27.34
23930415	2011	7	7	5190	44.13	27.34
23930415	2011	7	7	5200	44.76	27.33
23930415	2011	7	7	5210	45.42	27.35
23930415	2011	7	7	5220	46.94	27.37
23930415	2011	7	7	5230	48.76	27.36
23930415	2011	7	7	5240	50.63	27.35
23930415	2011	7	7	5250	51.43	27.34
23930415	2011	7	7	5260	51.3	27.34
23930415	2011	7	7	5270	51.48	27.33
23930415	2011	7	7	5280	51.3	27.33
23930415	2011	7	7	5290	52.37	27.32
23930415	2011	7	7	5300	52.95	27.32
23930415	2011	7	7	5310	53.36	27.31
23930415	2011	7	7	5320	53.61	27.29
23930415	2011	7	7	5330	53.62	27.26
23930415	2011	7	7	5340	53.54	27.22
23930415	2011	7	7	5350	53.62	27.2
23930415	2011	7	7	5360	53.66	27.16
23930415	2011	7	7	5370	52.53	27.14
23930415	2011	7	7	5380	52	27.16
23930415	2011	7	7	5390	51.56	27.17
23930415	2011	7	7	5400	51.68	27.17
23930415	2011	7	7	5410	51.79	27.18
23930415	2011	7	7	5420	51.9	27.2
23930415	2011	7	7	5430	52.49	27.21
23930415	2011	7	7	5440	53.14	27.21
23930415	2011	7	7	5450	53.5	27.21
23930415	2011	7	7	5460	53.97	27.21
23930415	2011	7	7	5470	55.27	27.21
23930415	2011	7	7	5480	56.08	27.14
23930415	2011	7	7	5490	56.29	27.1
23930415	2011	7	7	5500	54.87	27.03
23930415	2011	7	7	5510	53.95	27.01
23930415	2011	7	7	5520	53.56	27
23930415	2011	7	7	5530	53.46	26.97
23930415	2011	7	7	5540	53.39	26.98
23930415	2011	7	7	5550	52.07	27.02
23930415	2011	7	7	5560	50.29	27.03
23930415	2011	7	7	5570	48.69	27.11
23930415	2011	7	7	5580	47.03	27.16
23930415	2011	7	7	5590	45.34	27.2

23930415	2011	7	7	5600	43.69	27.25
23930415	2011	7	7	5610	42.18	27.27
23930415	2011	7	7	5620	40.54	27.3
23930415	2011	7	7	5630	38.84	27.33
23930415	2011	7	7	5640	37.27	27.36
23930415	2011	7	7	5650	35.87	27.4
23930415	2011	7	7	5660	34.26	27.41
23930415	2011	7	7	5670	32.74	27.45
23930415	2011	7	7	5680	31.41	27.47
23930415	2011	7	7	5690	30.08	27.5
23930415	2011	7	7	5700	28.44	27.52
23930415	2011	7	7	5710	26.91	27.53
23930415	2011	7	7	5720	25.46	27.55
23930415	2011	7	7	5730	24.17	27.57
23930415	2011	7	7	5740	22.91	27.6
23930415	2011	7	7	5750	22.99	27.6
23930415	2011	7	7	5760	23.21	27.6
23930415	2011	7	7	5770	23.02	27.61
23930415	2011	7	7	5780	23.21	27.62
23930415	2011	7	7	5790	23.13	27.62
23930415	2011	7	7	5800	23.17	27.62
23930415	2011	7	7	5810	23.08	27.64
23930415	2011	7	7	5820	21.88	27.65
23930415	2011	7	7	5830	21.76	27.64
23930415	2011	7	7	5840	22.26	27.64
23930415	2011	7	7	5850	22.9	27.64
23930415	2011	7	7	5860	23.16	27.66
23930415	2011	7	7	5870	23.03	27.67
23930415	2011	7	7	5880	23.11	27.67
23930415	2011	7	7	5890	23.14	27.66
23930415	2011	7	7	5900	23.02	27.67
23930415	2011	7	7	5910	23.13	27.67
23930415	2011	7	7	5920	23.13	27.67
23930415	2011	7	7	5930	23.11	27.67
23930415	2011	7	7	5940	23.15	27.68
23930415	2011	7	7	5950	23.06	27.68
23930415	2011	7	7	5960	23.17	27.68
23930415	2011	7	7	5970	23.08	27.69
23930415	2011	7	7	5980	23.06	27.7
23930415	2011	7	7	5990	23.04	27.7
23930415	2011	7	7	6000	22.6	27.7
23930415	2011	7	7	6010	22.71	27.71
23930415	2011	7	7	6020	22.97	27.71
23930415	2011	7	7	6030	23.09	27.7
23930415	2011	7	7	6040	23.18	27.7
23930415	2011	7	7	6050	21.97	27.71
23930415	2011	7	7	6060	19.77	27.74

23930415	2011	7	7	6070	17.96	27.75
23930415	2011	7	7	6080	17.25	27.75
23930415	2011	7	7	6090	16.73	27.76
23930415	2011	7	7	6100	16.36	27.77
23930415	2011	7	7	6110	15.07	27.78
23930415	2011	7	7	6120	12.74	27.81
23930415	2011	7	7	6130	10.36	27.82
23930415	2011	7	7	6140	7.63	27.85
23930415	2011	7	7	6150	4.93	27.86
23930415	2011	7	7	6160	3.68	27.88
23930415	2011	7	7	6170	2.76	27.89
23930415	2011	7	7	6180	2.59	27.9
23930415	2011	7	7	6190	2.74	27.9
23930415	2011	7	7	6200	2.45	27.91
23930415	2011	7	7	6210	2.45	27.92
23930415	2011	7	7	6220	2.57	27.92
23930415	2011	7	7	6230	2.58	27.92
23930415	2011	7	7	6240	2.43	27.92
23930415	2011	7	7	6250	2.46	27.93
23930415	2011	7	7	6260	1.91	27.94
23930415	2011	7	7	6270	0.57	27.95
23930415	2011	7	7	6280	0.58	27.95
23930415	2011	7	7	6290	0.57	27.97
23930415	2011	7	7	6300	0.58	27.98
23930415	2011	7	7	6310	0.59	28
23930415	2011	7	7	6320	0.6	28.01
23930415	2011	7	7	6330	0.57	28.04
23930415	2011	7	7	6340	0.6	28.05
23930415	2011	7	7	6350	0.61	28.07
23930415	2011	7	7	6360	0.6	28.08
23930415	2011	7	7	6370	0.57	28.1
23930415	2011	7	7	6380	0.54	28.11
23930415	2011	7	7	6390	0.48	28.12
23930415	2011	7	7	6400	0.5	28.14
23930415	2011	7	7	6410	0.51	28.14
51486355	2012	5	21	0	1.11	27.6
51486355	2012	5	21	10	1.59	27.6
51486355	2012	5	21	20	2.73	27.59
51486355	2012	5	21	30	3.19	27.59
51486355	2012	5	21	40	3.27	27.58
51486355	2012	5	21	50	3.17	27.58
51486355	2012	5	21	60	2.91	27.59
51486355	2012	5	21	70	3.01	27.59
51486355	2012	5	21	80	2.93	27.58
51486355	2012	5	21	90	3.55	27.57
51486355	2012	5	21	100	5	27.6
51486355	2012	5	21	110	5.72	27.58

51486355	2012	5	21	120	7.48	27.55
51486355	2012	5	21	130	9.77	27.52
51486355	2012	5	21	140	11.19	27.5
51486355	2012	5	21	150	11.74	27.48
51486355	2012	5	21	160	11.93	27.46
51486355	2012	5	21	170	11.93	27.46
51486355	2012	5	21	180	11.65	27.45
51486355	2012	5	21	190	11.31	27.43
51486355	2012	5	21	200	11.14	27.42
51486355	2012	5	21	210	11.15	27.42
51486355	2012	5	21	220	11.11	27.42
51486355	2012	5	21	230	11.14	27.42
51486355	2012	5	21	240	11.26	27.4
51486355	2012	5	21	250	12.12	27.39
51486355	2012	5	21	260	12.82	27.38
51486355	2012	5	21	270	13.48	27.37
51486355	2012	5	21	280	14.05	27.35
51486355	2012	5	21	290	14.51	27.34
51486355	2012	5	21	300	15.32	27.33
51486355	2012	5	21	310	16.19	27.32
51486355	2012	5	21	320	17.33	27.3
51486355	2012	5	21	330	19.28	27.28
51486355	2012	5	21	340	21.2	27.25
51486355	2012	5	21	350	22.42	27.24
51486355	2012	5	21	360	22.59	27.24
51486355	2012	5	21	370	22.96	27.23
51486355	2012	5	21	380	23.47	27.22
51486355	2012	5	21	390	23.94	27.22
51486355	2012	5	21	400	24.19	27.21
51486355	2012	5	21	410	24.55	27.21
51486355	2012	5	21	420	24.82	27.2
51486355	2012	5	21	430	25.31	27.19
51486355	2012	5	21	440	26.38	27.19
51486355	2012	5	21	450	27.5	27.17
51486355	2012	5	21	460	28.58	27.16
51486355	2012	5	21	470	30.41	27.14
51486355	2012	5	21	480	31.86	27.13
51486355	2012	5	21	490	33.23	27.11
51486355	2012	5	21	500	34.53	27.11
51486355	2012	5	21	510	36.72	27.08
51486355	2012	5	21	520	39.06	27.07
51486355	2012	5	21	530	41.05	27.06
51486355	2012	5	21	540	43.11	27.05
51486355	2012	5	21	550	44.53	27.04
51486355	2012	5	21	560	46.28	27.03
51486355	2012	5	21	570	47.97	27.03
51486355	2012	5	21	580	49.07	27.01

51486355	2012	5	21	590	50.37	27.01
51486355	2012	5	21	600	52.43	27
51486355	2012	5	21	610	54.21	26.98
51486355	2012	5	21	620	54.61	26.99
51486355	2012	5	21	630	54.02	27
51486355	2012	5	21	640	54.27	26.99
51486355	2012	5	21	650	54.28	26.99
51486355	2012	5	21	660	54.58	26.99
51486355	2012	5	21	670	54.6	27
51486355	2012	5	21	680	54.99	26.99
51486355	2012	5	21	690	54.79	26.99
51486355	2012	5	21	700	54.58	27
51486355	2012	5	21	710	54.9	26.99
51486355	2012	5	21	720	55.11	27
51486355	2012	5	21	730	55.1	27
51486355	2012	5	21	740	55.02	27
51486355	2012	5	21	750	54.85	27
51486355	2012	5	21	760	54.54	27
51486355	2012	5	21	770	54.55	27
51486355	2012	5	21	780	54.39	27.01
51486355	2012	5	21	790	54.34	27.01
51486355	2012	5	21	800	54.43	27
51486355	2012	5	21	810	54.03	27.01
51486355	2012	5	21	820	53.97	27.01
51486355	2012	5	21	830	54.06	27
51486355	2012	5	21	840	54.46	27.01
51486355	2012	5	21	850	54.71	27.01
51486355	2012	5	21	860	55.04	27.01
51486355	2012	5	21	870	55.54	27
51486355	2012	5	21	880	56.54	27
51486355	2012	5	21	890	57.61	26.99
51486355	2012	5	21	900	58.71	26.98
51486355	2012	5	21	910	59.82	26.97
51486355	2012	5	21	920	60.67	26.96
51486355	2012	5	21	930	61.45	26.96
51486355	2012	5	21	940	62.3	26.95
51486355	2012	5	21	950	63	26.95
51486355	2012	5	21	960	64.97	26.96
51486355	2012	5	21	970	67.74	26.93
51486355	2012	5	21	980	69.39	26.91
51486355	2012	5	21	990	70.54	26.85
51486355	2012	5	21	1000	71.52	26.68
51486355	2012	5	21	1010	72.48	26.47
51486355	2012	5	21	1020	73.38	26.33
51486355	2012	5	21	1030	73.99	26.26
51486355	2012	5	21	1040	74.24	26.2
51486355	2012	5	21	1050	74.15	26.14

51486355	2012	5	21	1060	74.08	26.12
51486355	2012	5	21	1070	74.25	26.1
51486355	2012	5	21	1080	74.2	26.05
51486355	2012	5	21	1090	74.31	25.98
51486355	2012	5	21	1100	73.98	25.85
51486355	2012	5	21	1110	74.18	25.82
51486355	2012	5	21	1120	74.5	25.74
51486355	2012	5	21	1130	75.03	25.67
51486355	2012	5	21	1140	75.6	25.64
51486355	2012	5	21	1150	76.02	25.61
51486355	2012	5	21	1160	76.23	25.57
51486355	2012	5	21	1170	76.64	25.51
51486355	2012	5	21	1180	77.32	25.42
51486355	2012	5	21	1190	78.16	25.33
51486355	2012	5	21	1200	78.66	25.27
51486355	2012	5	21	1210	78.76	25.23
51486355	2012	5	21	1220	78.38	25.21
51486355	2012	5	21	1230	78.27	25.21
51486355	2012	5	21	1240	78.68	25.19
51486355	2012	5	21	1250	78.69	25.17
51486355	2012	5	21	1260	78.83	25.16
51486355	2012	5	21	1270	78.8	25.15
51486355	2012	5	21	1280	78.76	25.11
51486355	2012	5	21	1290	78.63	25.09
51486355	2012	5	21	1300	78.64	25.07
51486355	2012	5	21	1310	78.88	25.05
51486355	2012	5	21	1320	79.16	25.03
51486355	2012	5	21	1330	79.58	24.99
51486355	2012	5	21	1340	80.22	24.93
51486355	2012	5	21	1350	80.9	24.89
51486355	2012	5	21	1360	81.65	24.85
51486355	2012	5	21	1370	82.47	24.79
51486355	2012	5	21	1380	83.13	24.75
51486355	2012	5	21	1390	83.78	24.72
51486355	2012	5	21	1400	84.29	24.7
51486355	2012	5	21	1410	85.01	24.65
51486355	2012	5	21	1420	85.87	24.6
51486355	2012	5	21	1430	86.81	24.51
51486355	2012	5	21	1440	88.41	24.49
51486355	2012	5	21	1450	90.38	24.35
51486355	2012	5	21	1460	92.54	24.22
51486355	2012	5	21	1470	94.19	24.14
51486355	2012	5	21	1480	95.31	23.99
51486355	2012	5	21	1490	96.09	23.93
51486355	2012	5	21	1500	97.35	23.68
51486355	2012	5	21	1510	97.72	23.61
51486355	2012	5	21	1520	99.2	23.37

51486355	2012	5	21	1530	100.8	23.23
51486355	2012	5	21	1540	102.13	23.1
51486355	2012	5	21	1550	104.58	23.12
51486355	2012	5	21	1560	107.68	23.06
51486355	2012	5	21	1570	110.92	22.95
51486355	2012	5	21	1580	114.19	22.81
51486355	2012	5	21	1590	117.05	22.59
51486355	2012	5	21	1600	118.55	22.5
51486355	2012	5	21	1610	120.97	22.28
51486355	2012	5	21	1620	122.51	22.18
51486355	2012	5	21	1630	124.24	22.07
51486355	2012	5	21	1640	126.47	21.94
51486355	2012	5	21	1650	128.06	21.64
51486355	2012	5	21	1660	129.22	21.48
51486355	2012	5	21	1670	130.71	21.25
51486355	2012	5	21	1680	132.9	21.22
51486355	2012	5	21	1690	135.29	21.22
51486355	2012	5	21	1700	137.48	21.21
51486355	2012	5	21	1710	139.77	21.15
51486355	2012	5	21	1720	141.88	21.09
51486355	2012	5	21	1730	143.31	20.95
51486355	2012	5	21	1740	144.68	20.89
51486355	2012	5	21	1750	146.41	20.68
51486355	2012	5	21	1760	148.24	20.63
51486355	2012	5	21	1770	150.14	20.56
51486355	2012	5	21	1780	152.52	20.49
51486355	2012	5	21	1790	155.46	20.3
51486355	2012	5	21	1800	157.46	20.16
51486355	2012	5	21	1810	158.55	20.13
51486355	2012	5	21	1820	160.32	19.96
51486355	2012	5	21	1830	161.47	19.89
51486355	2012	5	21	1840	162.6	19.65
51486355	2012	5	21	1850	162.9	19.65
51486355	2012	5	21	1860	164.34	19.49
51486355	2012	5	21	1870	165.45	19.41
51486355	2012	5	21	1880	165.81	19.4
51486355	2012	5	21	1890	166.29	19.32
51486355	2012	5	21	1900	166.39	19.27
51486355	2012	5	21	1910	166.44	19.21
51486355	2012	5	21	1920	166.17	19.19
51486355	2012	5	21	1930	166.66	19.15
51486355	2012	5	21	1940	166.98	19.1
51486355	2012	5	21	1950	167.56	19.06
51486355	2012	5	21	1960	167.68	18.96
51486355	2012	5	21	1970	168.05	18.8
51486355	2012	5	21	1980	169.1	18.78
51486355	2012	5	21	1990	169.71	18.75

51486355	2012	5	21	2000	170	18.67
51486355	2012	5	21	2010	170.43	18.61
51486355	2012	5	21	2020	170.97	18.58
51486355	2012	5	21	2030	171.47	18.54
51486355	2012	5	21	2040	172.05	18.48
51486355	2012	5	21	2050	172.89	18.42
51486355	2012	5	21	2060	173.55	18.37
51486355	2012	5	21	2070	173.91	18.32
51486355	2012	5	21	2080	174.13	18.28
51486355	2012	5	21	2090	175.09	18.24
51486355	2012	5	21	2100	175.9	18.2
51486355	2012	5	21	2110	177.05	18.16
51486355	2012	5	21	2120	177.89	18.11
51486355	2012	5	21	2130	177.69	18.07
51486355	2012	5	21	2140	178.67	18.04
51486355	2012	5	21	2150	178.93	18
51486355	2012	5	21	2160	178.82	17.94
51486355	2012	5	21	2170	178.7	17.91
51486355	2012	5	21	2180	178.7	17.85
51486355	2012	5	21	2190	179.41	17.83
51486355	2012	5	21	2200	179.95	17.78
51486355	2012	5	21	2210	180.18	17.75
51486355	2012	5	21	2220	181.86	17.72
51486355	2012	5	21	2230	183.33	17.69
51486355	2012	5	21	2240	184.92	17.62
51486355	2012	5	21	2250	186.36	17.6
51486355	2012	5	21	2260	187.43	17.55
51486355	2012	5	21	2270	189.24	17.51
51486355	2012	5	21	2280	190.53	17.45
51486355	2012	5	21	2290	191.03	17.39
51486355	2012	5	21	2300	191.73	17.35
51486355	2012	5	21	2310	192.88	17.3
51486355	2012	5	21	2320	194.16	17.23
51486355	2012	5	21	2330	194.94	17.14
51486355	2012	5	21	2340	195.47	17.09
51486355	2012	5	21	2350	196.33	17.03
51486355	2012	5	21	2360	197.02	16.99
51486355	2012	5	21	2370	197.73	16.96
51486355	2012	5	21	2380	197.62	16.89
51486355	2012	5	21	2390	198.11	16.85
51486355	2012	5	21	2400	197.81	16.82
51486355	2012	5	21	2410	197.81	16.78
51486355	2012	5	21	2420	197.74	16.69
51486355	2012	5	21	2430	198	16.61
51486355	2012	5	21	2440	198.21	16.54
51486355	2012	5	21	2450	198.34	16.5
51486355	2012	5	21	2460	198.21	16.46

51486355	2012	5	21	2470	198.41	16.35
51486355	2012	5	21	2480	198.49	16.34
51486355	2012	5	21	2490	198.29	16.31
51486355	2012	5	21	2500	198.08	16.27
51486355	2012	5	21	2510	197.87	16.22
51486355	2012	5	21	2520	197.66	16.17
51486355	2012	5	21	2530	197.65	16.13
51486355	2012	5	21	2540	197.64	16.09
51486355	2012	5	21	2550	197.65	16.06
51486355	2012	5	21	2560	197.76	16.03
51486355	2012	5	21	2570	197.73	16
51486355	2012	5	21	2580	197.54	15.97
51486355	2012	5	21	2590	197.18	15.94
51486355	2012	5	21	2600	197.11	15.89
51486355	2012	5	21	2610	197.19	15.87
51486355	2012	5	21	2620	197.05	15.85
51486355	2012	5	21	2630	197.03	15.83
51486355	2012	5	21	2640	197.32	15.8
51486355	2012	5	21	2650	198.2	15.78
51486355	2012	5	21	2660	198.51	15.75
51486355	2012	5	21	2670	199.38	15.73
51486355	2012	5	21	2680	199.84	15.71
51486355	2012	5	21	2690	199.96	15.68
51486355	2012	5	21	2700	200.04	15.67
51486355	2012	5	21	2710	200.21	15.64
51486355	2012	5	21	2720	199.95	15.62
51486355	2012	5	21	2730	199.89	15.6
51486355	2012	5	21	2740	199.95	15.58
51486355	2012	5	21	2750	199.91	15.57
51486355	2012	5	21	2760	200	15.57
51486355	2012	5	21	2770	199.88	15.56
51486355	2012	5	21	2780	199.84	15.55
51486355	2012	5	21	2790	199.87	15.54
51486355	2012	5	21	2800	200.21	15.53
51486355	2012	5	21	2810	200.88	15.53
51486355	2012	5	21	2820	201.08	15.51
51486355	2012	5	21	2830	200.9	15.52
51486355	2012	5	21	2840	201.2	15.51
51486355	2012	5	21	2850	201.13	15.51
51486355	2012	5	21	2860	201.2	15.5
51486355	2012	5	21	2870	201.23	15.5
51486355	2012	5	21	2880	201.13	15.49
51486355	2012	5	21	2890	201.05	15.5
51486355	2012	5	21	2900	201.06	15.49
51486355	2012	5	21	2910	200.86	15.47
51486355	2012	5	21	2920	200.9	15.46
51486355	2012	5	21	2930	201.3	15.46

51486355	2012	5	21	2940	202.14	15.46
51486355	2012	5	21	2950	203.21	15.46
51486355	2012	5	21	2960	203.77	15.46
51486355	2012	5	21	2970	204.22	15.46
51486355	2012	5	21	2980	204.26	15.45
51486355	2012	5	21	2990	204.35	15.45
51486355	2012	5	21	3000	204.17	15.44
51486355	2012	5	21	3010	204.05	15.43
51486355	2012	5	21	3020	204.59	15.42
51486355	2012	5	21	3030	205.18	15.42
51486355	2012	5	21	3040	205.48	15.41
51486355	2012	5	21	3050	206.51	15.39
51486355	2012	5	21	3060	207.21	15.38
51486355	2012	5	21	3070	207.63	15.37
51486355	2012	5	21	3080	207.92	15.35
51486355	2012	5	21	3090	207.93	15.35
51486355	2012	5	21	3100	208.73	15.34
51486355	2012	5	21	3110	209.32	15.33
51486355	2012	5	21	3120	209.89	15.29
51486355	2012	5	21	3130	210.99	15.28
51486355	2012	5	21	3140	212.34	15.25
51486355	2012	5	21	3150	213.83	15.23
51486355	2012	5	21	3160	214.47	15.21
51486355	2012	5	21	3170	214.76	15.17
51486355	2012	5	21	3180	215.22	15.13
51486355	2012	5	21	3190	215.41	15.1
51486355	2012	5	21	3200	215.49	15.07
51486355	2012	5	21	3210	215.64	15.01
51486355	2012	5	21	3220	215.77	15
51486355	2012	5	21	3230	215.92	15
51486355	2012	5	21	3240	216.01	14.99
51486355	2012	5	21	3250	216.09	14.98
51486355	2012	5	21	3260	216.17	14.96
51486355	2012	5	21	3270	216.38	14.95
51486355	2012	5	21	3280	216.48	14.92
51486355	2012	5	21	3290	216.31	14.91
51486355	2012	5	21	3300	215.74	14.82
51486355	2012	5	21	3310	215.86	14.81
51486355	2012	5	21	3320	215.91	14.78
51486355	2012	5	21	3330	215.98	14.77
51486355	2012	5	21	3340	215.85	14.75
51486355	2012	5	21	3350	216.33	14.66
51486355	2012	5	21	3360	215.95	14.64
51486355	2012	5	21	3370	215.31	14.64
51486355	2012	5	21	3380	214.49	14.62
51486355	2012	5	21	3390	213.61	14.62
51486355	2012	5	21	3400	212.48	14.61

51486355	2012	5	21	3410	211.21	14.6
51486355	2012	5	21	3420	209.92	14.6
51486355	2012	5	21	3430	208.73	14.6
51486355	2012	5	21	3440	208.57	14.59
51486355	2012	5	21	3450	208.5	14.57
51486355	2012	5	21	3460	208.08	14.58
51486355	2012	5	21	3470	207.4	14.58
51486355	2012	5	21	3480	206.89	14.58
51486355	2012	5	21	3490	206.51	14.58
51486355	2012	5	21	3500	207.62	14.61
51486355	2012	5	21	3510	208.4	14.66
51486355	2012	5	21	3520	208.99	14.66
51486355	2012	5	21	3530	209.32	14.67
51486355	2012	5	21	3540	209.7	14.67
51486355	2012	5	21	3550	210.14	14.67
51486355	2012	5	21	3560	210.37	14.67
51486355	2012	5	21	3570	210.46	14.68
51486355	2012	5	21	3580	210.64	14.69
51486355	2012	5	21	3590	210.93	14.71
51486355	2012	5	21	3600	211.73	14.71
51486355	2012	5	21	3610	212.12	14.73
51486355	2012	5	21	3620	212.33	14.71
51486355	2012	5	21	3630	213.22	14.7
51486355	2012	5	21	3640	214.08	14.7
51486355	2012	5	21	3650	214.55	14.69
51486355	2012	5	21	3660	214.53	14.69
51486355	2012	5	21	3670	214.34	14.68
51486355	2012	5	21	3680	214.55	14.67
51486355	2012	5	21	3690	214.91	14.66
51486355	2012	5	21	3700	215	14.64
51486355	2012	5	21	3710	215.7	14.63
51486355	2012	5	21	3720	217.01	14.64
51486355	2012	5	21	3730	217.55	14.63
51486355	2012	5	21	3740	218.52	14.61
51486355	2012	5	21	3750	219.15	14.6
51486355	2012	5	21	3760	219.36	14.6
51486355	2012	5	21	3770	219.55	14.57
51486355	2012	5	21	3780	219.57	14.57
51486355	2012	5	21	3790	218.86	14.54
51486355	2012	5	21	3800	218.83	14.53
51486355	2012	5	21	3810	218.57	14.51
51486355	2012	5	21	3820	218.81	14.48
51486355	2012	5	21	3830	219.03	14.44
51486355	2012	5	21	3840	218.94	14.42
51486355	2012	5	21	3850	218.71	14.42
51486355	2012	5	21	3860	220.21	14.43
51486355	2012	5	21	3870	220.93	14.42

51486355	2012	5	21	3880	221	14.41
51486355	2012	5	21	3890	221.07	14.41
51486355	2012	5	21	3900	220.85	14.39
51486355	2012	5	21	3910	220.95	14.37
51486355	2012	5	21	3920	221.04	14.35
51486355	2012	5	21	3930	221.07	14.35
51486355	2012	5	21	3940	221.34	14.34
51486355	2012	5	21	3950	221.34	14.33
51486355	2012	5	21	3960	221.32	14.32
51486355	2012	5	21	3970	221.17	14.31
51486355	2012	5	21	3980	221.11	14.3
51486355	2012	5	21	3990	221.07	14.29
51486355	2012	5	21	4000	221.08	14.26
51486355	2012	5	21	4010	221	14.25
51486355	2012	5	21	4020	221.28	14.23
51486355	2012	5	21	4030	221.19	14.22
51486355	2012	5	21	4040	221.3	14.21
51486355	2012	5	21	4050	221.17	14.2
51486355	2012	5	21	4060	221.23	14.16
51486355	2012	5	21	4070	221.37	14.13
51486355	2012	5	21	4080	221.43	14.1
51486355	2012	5	21	4090	221.87	14.08
51486355	2012	5	21	4100	222.06	14.07
51486355	2012	5	21	4110	223.04	14.05
51486355	2012	5	21	4120	223.01	14.02
51486355	2012	5	21	4130	223.2	14
51486355	2012	5	21	4140	223.88	13.98
51486355	2012	5	21	4150	224.58	13.94
51486355	2012	5	21	4160	224.82	13.92
51486355	2012	5	21	4170	224.62	13.89
51486355	2012	5	21	4180	224.49	13.88
51486355	2012	5	21	4190	224.53	13.85
51486355	2012	5	21	4200	224.34	13.8
51486355	2012	5	21	4210	224.29	13.78
51486355	2012	5	21	4220	224.88	13.73
51486355	2012	5	21	4230	225.03	13.72
51486355	2012	5	21	4240	225.19	13.71
51486355	2012	5	21	4250	224.96	13.71
51486355	2012	5	21	4260	225.02	13.7
51486355	2012	5	21	4270	224.84	13.69
51486355	2012	5	21	4280	224.86	13.67
51486355	2012	5	21	4290	224.87	13.67
51486355	2012	5	21	4300	224.94	13.65
51486355	2012	5	21	4310	225.35	13.65
51486355	2012	5	21	4320	225.16	13.65
51486355	2012	5	21	4330	224.84	13.65
51486355	2012	5	21	4340	224.92	13.65

51486355	2012	5	21	4350	224.85	13.65
51486355	2012	5	21	4360	224.94	13.64
51486355	2012	5	21	4370	225.05	13.65
51486355	2012	5	21	4380	225.02	13.65
51486355	2012	5	21	4390	225.04	13.65
51486355	2012	5	21	4400	225.15	13.65
51486355	2012	5	21	4410	225.68	13.64
51486355	2012	5	21	4420	225.66	13.64
51486355	2012	5	21	4430	225.95	13.64
51486355	2012	5	21	4440	225.61	13.64
51486355	2012	5	21	4450	225.76	13.64
51486355	2012	5	21	4460	225.65	13.65
51486355	2012	5	21	4470	225.92	13.67
51486355	2012	5	21	4480	225.91	13.67
51486355	2012	5	21	4490	225.94	13.67
51486355	2012	5	21	4500	225.87	13.68
51486355	2012	5	21	4510	225.95	13.71
51486355	2012	5	21	4520	225.8	13.72
51486355	2012	5	21	4530	226.41	13.75
51486355	2012	5	21	4540	226.2	13.76
51486355	2012	5	21	4550	225.86	13.85
51486355	2012	5	21	4560	226.47	13.9
51486355	2012	5	21	4570	226.8	13.9
51486355	2012	5	21	4580	227.67	13.9
51486355	2012	5	21	4590	228.23	13.92
51486355	2012	5	21	4600	228.84	13.92
51486355	2012	5	21	4610	228.86	13.92
51486355	2012	5	21	4620	228.76	13.92
51486355	2012	5	21	4630	228.56	13.92
51486355	2012	5	21	4640	228.65	13.92
51486355	2012	5	21	4650	228.44	13.93
51486355	2012	5	21	4660	227.8	13.95
51486355	2012	5	21	4670	228.5	13.95
51486355	2012	5	21	4680	228.59	13.96
51486355	2012	5	21	4690	228.19	13.97
51486355	2012	5	21	4700	227.49	13.98
51486355	2012	5	21	4710	226.81	13.99
51486355	2012	5	21	4720	226.32	14
51486355	2012	5	21	4730	226	14
51486355	2012	5	21	4740	225.3	14
51486355	2012	5	21	4750	224.35	14
51486355	2012	5	21	4760	224.76	14.01
51486355	2012	5	21	4770	226.47	14.03
51486355	2012	5	21	4780	228.24	14.03
51486355	2012	5	21	4790	228.84	14.06
51486355	2012	5	21	4800	228.38	14.07
51486355	2012	5	21	4810	228.28	14.09

51486355	2012	5	21	4820	229.73	14.13
51486355	2012	5	21	4830	230.28	14.13
51486355	2012	5	21	4840	230.26	14.14
51486355	2012	5	21	4850	230.53	14.17
51486355	2012	5	21	4860	231.07	14.16
51486355	2012	5	21	4870	231.29	14.16
51486355	2012	5	21	4880	231.82	14.14
51486355	2012	5	21	4890	232.23	14.14
51486355	2012	5	21	4900	231.79	14.14
51486355	2012	5	21	4910	231.66	14.09
51486355	2012	5	21	4920	231.71	14.07
51486355	2012	5	21	4930	231.54	14.05
51486355	2012	5	21	4940	232.06	14.04
51486355	2012	5	21	4950	232.23	14
51486355	2012	5	21	4960	232.4	13.93
51486355	2012	5	21	4970	233.04	13.9
51486355	2012	5	21	4980	232.64	13.89
51486355	2012	5	21	4990	232.59	13.88
51486355	2012	5	21	5000	232.33	13.85
51486355	2012	5	21	5010	232.42	13.78
51486355	2012	5	21	5020	232.8	13.68
51486355	2012	5	21	5030	232.81	13.67
51486355	2012	5	21	5040	233.29	13.63
51486355	2012	5	21	5050	233.25	13.6
51486355	2012	5	21	5060	233.03	13.6
51486355	2012	5	21	5070	233.15	13.57
51486355	2012	5	21	5080	233.93	13.52
51486355	2012	5	21	5090	234.39	13.52
51486355	2012	5	21	5100	235.29	13.52
51486355	2012	5	21	5110	235.89	13.51
51486355	2012	5	21	5120	236.7	13.46
51486355	2012	5	21	5130	238.19	13.45
51486355	2012	5	21	5140	239.09	13.43
51486355	2012	5	21	5150	239.87	13.42
51486355	2012	5	21	5160	241.38	13.4
51486355	2012	5	21	5170	241.55	13.4
51486355	2012	5	21	5180	241.98	13.4
51486355	2012	5	21	5190	242.15	13.39
51486355	2012	5	21	5200	241.71	13.38
51486355	2012	5	21	5210	241.15	13.37
51486355	2012	5	21	5220	241.97	13.35
51486355	2012	5	21	5230	241.88	13.33
51486355	2012	5	21	5240	242.24	13.32
51486355	2012	5	21	5250	242.11	13.32
51486355	2012	5	21	5260	242.2	13.28
51486355	2012	5	21	5270	242.33	13.26
51486355	2012	5	21	5280	242.97	13.25

51486355	2012	5	21	5290	243.73	13.25
51486355	2012	5	21	5300	245.23	13.25
51486355	2012	5	21	5310	246.54	13.24
51486355	2012	5	21	5320	247.68	13.24
51486355	2012	5	21	5330	248.85	13.23
51486355	2012	5	21	5340	250.02	13.22
51486355	2012	5	21	5350	250.12	13.21
51486355	2012	5	21	5360	249.62	13.21
51486355	2012	5	21	5370	249.59	13.21
51486355	2012	5	21	5380	249.78	13.19
51486355	2012	5	21	5390	249.77	13.18
51486355	2012	5	21	5400	249.87	13.17
51486355	2012	5	21	5410	249.86	13.17
51486355	2012	5	21	5420	249.84	13.17
51486355	2012	5	21	5430	249.85	13.16
51486355	2012	5	21	5440	250	13.13
51486355	2012	5	21	5450	250.06	13.11
51486355	2012	5	21	5460	249.74	13.08
51486355	2012	5	21	5470	250.22	13.07
51486355	2012	5	21	5480	250.33	13.03
51486355	2012	5	21	5490	251.6	13.02
51486355	2012	5	21	5500	252.56	13
51486355	2012	5	21	5510	253.34	12.98
51486355	2012	5	21	5520	254.15	12.96
51486355	2012	5	21	5530	254.29	12.93
51486355	2012	5	21	5540	255.31	12.92
51486355	2012	5	21	5550	256.33	12.9
51486355	2012	5	21	5560	256.29	12.89
51486355	2012	5	21	5570	255.93	12.85
51486355	2012	5	21	5580	255.57	12.74
51486355	2012	5	21	5590	256.41	12.71
51486355	2012	5	21	5600	257.06	12.71
51486355	2012	5	21	5610	257.51	12.7
51486355	2012	5	21	5620	257.79	12.68
51486355	2012	5	21	5630	257.7	12.67
51486355	2012	5	21	5640	257.53	12.66
51486355	2012	5	21	5650	257.65	12.64
51486355	2012	5	21	5660	257.47	12.64
51486355	2012	5	21	5670	257.64	12.63
51486355	2012	5	21	5680	257.79	12.62
51486355	2012	5	21	5690	257.95	12.6
51486355	2012	5	21	5700	257.91	12.6
51486355	2012	5	21	5710	257.68	12.57
51486355	2012	5	21	5720	257.48	12.56
51486355	2012	5	21	5730	257.22	12.54
51486355	2012	5	21	5740	257.35	12.53
51486355	2012	5	21	5750	256.65	12.51

51486355	2012	5	21	5760	256.34	12.52
51486355	2012	5	21	5770	256.62	12.51
51486355	2012	5	21	5780	256.15	12.49
51486355	2012	5	21	5790	255.11	12.49
51486355	2012	5	21	5800	255.66	12.48
51486355	2012	5	21	5810	256.52	12.47
51486355	2012	5	21	5820	257.42	12.46
51486355	2012	5	21	5830	258.79	12.46
51486355	2012	5	21	5840	259.2	12.46
51486355	2012	5	21	5850	259.31	12.45
51486355	2012	5	21	5860	259.08	12.44
51486355	2012	5	21	5870	258.53	12.43
51486355	2012	5	21	5880	259.52	12.43
51486355	2012	5	21	5890	260.05	12.43
51486355	2012	5	21	5900	260.27	12.42
51486355	2012	5	21	5910	261.14	12.42
51486355	2012	5	21	5920	261.32	12.42
51486355	2012	5	21	5930	261.88	12.42
51486355	2012	5	21	5940	262.79	12.41
51486355	2012	5	21	5950	263.45	12.41
51486355	2012	5	21	5960	263.55	12.4
51486355	2012	5	21	5970	264.22	12.4
51486355	2012	5	21	5980	264.15	12.4
51486355	2012	5	21	5990	264.47	12.39
51486355	2012	5	21	6000	264.13	12.37
51486355	2012	5	21	6010	263.48	12.35
51486355	2012	5	21	6020	264.05	12.34
51486355	2012	5	21	6030	264.42	12.33
51486355	2012	5	21	6040	264.3	12.32
51486355	2012	5	21	6050	264.25	12.31
51486355	2012	5	21	6060	263.97	12.28
51486355	2012	5	21	6070	263.58	12.27
51486355	2012	5	21	6080	263.37	12.25
51486355	2012	5	21	6090	263.5	12.25
51486355	2012	5	21	6100	263.32	12.25
51486355	2012	5	21	6110	263.24	12.24
51486355	2012	5	21	6120	263	12.25
51486355	2012	5	21	6130	262.83	12.25
51486355	2012	5	21	6140	263.11	12.25
51486355	2012	5	21	6150	263.83	12.25
51486355	2012	5	21	6160	264.02	12.24
51486355	2012	5	21	6170	264.46	12.24
51486355	2012	5	21	6180	264.44	12.21
51486355	2012	5	21	6190	264.51	12.18
51486355	2012	5	21	6200	264.33	12.16
51486355	2012	5	21	6210	264.51	12.14
51486355	2012	5	21	6220	264.26	12.13

51486355	2012	5	21	6230	264.2	12.1
51486355	2012	5	21	6240	264.31	12.1
51486355	2012	5	21	6250	264.43	12.1
51486355	2012	5	21	6260	263.97	12.07
51486355	2012	5	21	6270	263.67	12.04
51486355	2012	5	21	6280	263.83	12.05
51486355	2012	5	21	6290	264.37	12.05
51486355	2012	5	21	6300	264.56	12.03
51486355	2012	5	21	6310	265.31	12.03
51486355	2012	5	21	6320	265.38	12.02
51486355	2012	5	21	6330	265.24	12
51486355	2012	5	21	6340	265.2	11.97
51486355	2012	5	21	6350	264.77	11.95
51486355	2012	5	21	6360	264.71	11.94
51486355	2012	5	21	6370	264.91	11.92
51486355	2012	5	21	6380	264.76	11.9
51486355	2012	5	21	6390	264.58	11.89
51486355	2012	5	21	6400	264.65	11.88
51486355	2012	5	21	6410	264.72	11.87
51486355	2012	5	21	6420	264.75	11.85
51486355	2012	5	21	6430	264.82	11.85
51486355	2012	5	21	6440	264.63	11.83
51486355	2012	5	21	6450	264.83	11.83
51486355	2012	5	21	6460	264.45	11.82
51486355	2012	5	21	6470	264.78	11.81
51486355	2012	5	21	6480	264.69	11.8
51486355	2012	5	21	6490	264.63	11.79
51486355	2012	5	21	6500	264.71	11.78
51486355	2012	5	21	6510	264.5	11.78
51486355	2012	5	21	6520	265.23	11.78
51486355	2012	5	21	6530	265.19	11.78
51486355	2012	5	21	6540	265.38	11.77
51486355	2012	5	21	6550	265.4	11.76
51486355	2012	5	21	6560	265.82	11.75
51486355	2012	5	21	6570	266.67	11.75
51486355	2012	5	21	6580	266.7	11.75
51486355	2012	5	21	6590	267.24	11.67
51486355	2012	5	21	6600	267.09	11.66
51486355	2012	5	21	6610	267.4	11.65
51486355	2012	5	21	6620	267.55	11.64
51486355	2012	5	21	6630	268.08	11.63
51486355	2012	5	21	6640	268.13	11.61
51486355	2012	5	21	6650	267.85	11.6
51486355	2012	5	21	6660	266.48	11.6
51486355	2012	5	21	6670	266.5	11.59
51486355	2012	5	21	6680	266.57	11.59
51486355	2012	5	21	6690	265.88	11.6

51486355	2012	5	21	6700	265.98	11.6
51486355	2012	5	21	6710	267.09	11.62
51486355	2012	5	21	6720	267	11.63
51486355	2012	5	21	6730	266.59	11.64
51486355	2012	5	21	6740	266.53	11.64
51486355	2012	5	21	6750	266.39	11.64
51486355	2012	5	21	6760	266.2	11.67
51486355	2012	5	21	6770	265.7	11.76
51486355	2012	5	21	6780	264.6	11.76
51486355	2012	5	21	6790	264.98	11.77
51486355	2012	5	21	6800	265.4	11.78
51486355	2012	5	21	6810	266.78	11.81
51486355	2012	5	21	6820	268.32	11.85
51486355	2012	5	21	6830	270.48	11.89
51486355	2012	5	21	6840	272.21	11.89
51486355	2012	5	21	6850	272.63	11.87
51486355	2012	5	21	6860	273.29	11.85
51486355	2012	5	21	6870	274.78	11.67
51486355	2012	5	21	6880	275.65	11.65
51486355	2012	5	21	6890	276.33	11.64
51486355	2012	5	21	6900	276.51	11.63
51486355	2012	5	21	6910	276.8	11.6
51486355	2012	5	21	6920	276.24	11.59
51486355	2012	5	21	6930	276.13	11.57
51486355	2012	5	21	6940	276.38	11.53
51486355	2012	5	21	6950	276.54	11.5
51486355	2012	5	21	6960	276.88	11.47
51486355	2012	5	21	6970	277.23	11.46
51486355	2012	5	21	6980	278.4	11.4
51486355	2012	5	21	6990	278.51	11.39
51486355	2012	5	21	7000	278.69	11.37
51486355	2012	5	21	7010	278.63	11.35
51486355	2012	5	21	7020	279.36	11.35
51486355	2012	5	21	7030	279.82	11.33
51486355	2012	5	21	7040	280.12	11.32
51486355	2012	5	21	7050	280.25	11.3
51486355	2012	5	21	7060	280.4	11.25
51486355	2012	5	21	7070	280.79	11.24
51486355	2012	5	21	7080	281.18	11.24
51486355	2012	5	21	7090	280.87	11.21
51486355	2012	5	21	7100	279.91	11.18
51486355	2012	5	21	7110	279.7	11.17
51486355	2012	5	21	7120	279.99	11.17
51486355	2012	5	21	7130	279.56	11.17
51486355	2012	5	21	7140	279.73	11.16
51486355	2012	5	21	7150	280.77	11.17
51486355	2012	5	21	7160	281.31	11.15

51486355	2012	5	21	7170	281.4	11.14
51486355	2012	5	21	7180	281.93	11.13
51486355	2012	5	21	7190	282.66	11.11
51486355	2012	5	21	7200	283.29	11.1
51486355	2012	5	21	7210	283.52	11.11
51486355	2012	5	21	7220	284.11	11.1
51486355	2012	5	21	7230	283.88	11.1
51486355	2012	5	21	7240	284.17	11.08
51486355	2012	5	21	7250	283.86	11.06
51486355	2012	5	21	7260	283.87	11.06
51486355	2012	5	21	7270	284.1	11.06
51486355	2012	5	21	7280	284.02	11.05
51486355	2012	5	21	7290	283.78	11.04
51486355	2012	5	21	7300	283.71	11.03
51486355	2012	5	21	7310	283.15	11.01
51486355	2012	5	21	7320	282.95	11
51486355	2012	5	21	7330	283.29	11
51486355	2012	5	21	7340	283.33	11
51486355	2012	5	21	7350	283.45	11
51486355	2012	5	21	7360	283.38	11
51486355	2012	5	21	7370	283.67	11
51486355	2012	5	21	7380	283.55	11
51486355	2012	5	21	7390	283.24	10.99
51486355	2012	5	21	7400	283.45	10.99
51486355	2012	5	21	7410	283.24	10.98
51486355	2012	5	21	7420	283.53	10.98
51486355	2012	5	21	7430	283.48	10.98
51486355	2012	5	21	7440	283.87	10.97
51486355	2012	5	21	7450	283.74	10.97
51486355	2012	5	21	7460	283.58	10.96
51486355	2012	5	21	7470	283.51	10.96
51486355	2012	5	21	7480	283.85	10.96
51486355	2012	5	21	7490	283.6	10.95
51486355	2012	5	21	7500	283.71	10.96
51486355	2012	5	21	7510	283.7	10.96
51486355	2012	5	21	7520	283.85	10.94
51486355	2012	5	21	7530	283.72	10.94
51486355	2012	5	21	7540	283.62	10.94
51486355	2012	5	21	7550	283.39	10.95
51486355	2012	5	21	7560	283.21	10.94
51486355	2012	5	21	7570	282.99	10.93
51486355	2012	5	21	7580	282.92	10.94
51486355	2012	5	21	7590	283.1	10.94
51486355	2012	5	21	7600	283.12	10.93
51486355	2012	5	21	7610	282.65	10.92
51486355	2012	5	21	7620	282.19	10.93
51486355	2012	5	21	7630	281.96	10.93

51486355	2012	5	21	7640	281.52	10.93
51486355	2012	5	21	7650	280.99	10.92
51486355	2012	5	21	7660	280.29	10.93
51486355	2012	5	21	7670	279.62	10.92
51486355	2012	5	21	7680	280.54	10.93
51486355	2012	5	21	7690	281.09	10.92
51486355	2012	5	21	7700	282.02	10.94
51486355	2012	5	21	7710	283.76	10.95
51486355	2012	5	21	7720	285.57	10.95
51486355	2012	5	21	7730	286.45	10.96
51486355	2012	5	21	7740	287.25	10.95
51486355	2012	5	21	7750	288.06	10.96
51486355	2012	5	21	7760	288.74	10.96
51486355	2012	5	21	7770	290.31	10.96
51486355	2012	5	21	7780	291.33	10.96
51486355	2012	5	21	7790	292.14	10.96
51486355	2012	5	21	7800	292.75	10.96
51486355	2012	5	21	7810	293.58	10.96
51486355	2012	5	21	7820	294.26	10.96
51486355	2012	5	21	7830	294.99	10.96
51486355	2012	5	21	7840	294.97	10.95
51486355	2012	5	21	7850	295.41	10.95
51486355	2012	5	21	7860	295.43	10.95
51486355	2012	5	21	7870	295.85	10.94
51486355	2012	5	21	7880	296.69	10.93
51486355	2012	5	21	7890	297.1	10.92
51486355	2012	5	21	7900	297.38	10.91
51486355	2012	5	21	7910	298.26	10.9
51486355	2012	5	21	7920	299.58	10.87
51486355	2012	5	21	7930	300.16	10.87
51486355	2012	5	21	7940	300.44	10.85
51486355	2012	5	21	7950	300.45	10.85
51486355	2012	5	21	7960	300.68	10.84
51486355	2012	5	21	7970	300.54	10.82
51486355	2012	5	21	7980	300.5	10.82
51486355	2012	5	21	7990	300.88	10.8
51486355	2012	5	21	8000	301.13	10.79
51486355	2012	5	21	8010	301.16	10.78
51486355	2012	5	21	8020	301.34	10.77
51486355	2012	5	21	8030	301.84	10.76
51486355	2012	5	21	8040	302.31	10.75
51486355	2012	5	21	8050	302.17	10.75
51486355	2012	5	21	8060	302.8	10.75
51486355	2012	5	21	8070	303.98	10.75
51486355	2012	5	21	8080	304.89	10.75
51486355	2012	5	21	8090	305.39	10.75
51486355	2012	5	21	8100	305.68	10.74

51486355	2012	5	21	8110	305.8	10.73
51486355	2012	5	21	8120	306.06	10.71
51486355	2012	5	21	8130	306.29	10.71
51486355	2012	5	21	8140	306.42	10.7
51486355	2012	5	21	8150	305.7	10.68
51486355	2012	5	21	8160	306.02	10.68
51486355	2012	5	21	8170	306.04	10.59
51486355	2012	5	21	8180	306.36	10.6
51486355	2012	5	21	8190	306.5	10.58
51486355	2012	5	21	8200	306.81	10.57
51486355	2012	5	21	8210	306.45	10.57
51486355	2012	5	21	8220	306.25	10.57
51486355	2012	5	21	8230	306.2	10.56
51486355	2012	5	21	8240	306.2	10.56
51486355	2012	5	21	8250	306.2	10.55
51486355	2012	5	21	8260	305.86	10.54
51486355	2012	5	21	8270	305.2	10.53
51486355	2012	5	21	8280	305.42	10.53
51486355	2012	5	21	8290	305.12	10.53
51486355	2012	5	21	8300	304.79	10.52
51486355	2012	5	21	8310	304.27	10.51
51486355	2012	5	21	8320	303.93	10.5
51486355	2012	5	21	8330	303.27	10.51
51486355	2012	5	21	8340	302.08	10.5
51486355	2012	5	21	8350	301.33	10.49
51486355	2012	5	21	8360	301	10.49
51486355	2012	5	21	8370	301.23	10.49
51486355	2012	5	21	8380	301.2	10.49
51486355	2012	5	21	8390	301.04	10.49
51486355	2012	5	21	8400	300.86	10.49
51486355	2012	5	21	8410	300.39	10.49
51486355	2012	5	21	8420	299.61	10.49
51486355	2012	5	21	8430	299.37	10.49
51486355	2012	5	21	8440	299.2	10.48
51486355	2012	5	21	8450	298.32	10.48
51486355	2012	5	21	8460	297.1	10.48
51486355	2012	5	21	8470	295.87	10.46
51486355	2012	5	21	8480	294.39	10.46
51486355	2012	5	21	8490	292.92	10.46
51486355	2012	5	21	8500	291.7	10.46
51486355	2012	5	21	8510	290.31	10.45
51486355	2012	5	21	8520	288.68	10.45
51486355	2012	5	21	8530	288.35	10.45
51486355	2012	5	21	8540	288.55	10.46
51486355	2012	5	21	8550	287.87	10.46
51486355	2012	5	21	8560	288.06	10.46
51486355	2012	5	21	8570	289.12	10.48

51486355	2012	5	21	8580	289.01	10.49
51486355	2012	5	21	8590	288.14	10.5
51486355	2012	5	21	8600	287.19	10.49
51486355	2012	5	21	8610	285.89	10.49
51486355	2012	5	21	8620	284.1	10.48
51486355	2012	5	21	8630	281.93	10.49
51486355	2012	5	21	8640	280.37	10.5
51486355	2012	5	21	8650	280.48	10.56
51486355	2012	5	21	8660	281.32	10.58
51486355	2012	5	21	8670	282.19	10.68
51486355	2012	5	21	8680	283.11	10.71
51486355	2012	5	21	8690	283.41	10.73
51486355	2012	5	21	8700	281.98	10.74
51486355	2012	5	21	8710	281.24	10.75
51486355	2012	5	21	8720	281.11	10.77
51486355	2012	5	21	8730	280.67	10.78
51486355	2012	5	21	8740	280.56	10.79
51486355	2012	5	21	8750	280.01	10.8
51486355	2012	5	21	8760	278.68	10.79
51486355	2012	5	21	8770	277.78	10.81
51486355	2012	5	21	8780	276.75	10.82
51486355	2012	5	21	8790	276.07	10.84
51486355	2012	5	21	8800	275.43	10.85
51486355	2012	5	21	8810	274.94	10.87
51486355	2012	5	21	8820	273.25	10.89
51486355	2012	5	21	8830	272.4	10.92
51486355	2012	5	21	8840	271.62	10.96
51486355	2012	5	21	8850	270.58	10.97
51486355	2012	5	21	8860	269.58	11
51486355	2012	5	21	8870	269.37	11.01
51486355	2012	5	21	8880	269.16	11.06
51486355	2012	5	21	8890	269.74	11.1
51486355	2012	5	21	8900	270.71	11.11
51486355	2012	5	21	8910	271.46	11.12
51486355	2012	5	21	8920	271.64	11.13
51486355	2012	5	21	8930	271.82	11.14
51486355	2012	5	21	8940	271.75	11.14
51486355	2012	5	21	8950	271.93	11.15
51486355	2012	5	21	8960	271.86	11.15
51486355	2012	5	21	8970	271.81	11.15
51486355	2012	5	21	8980	271.63	11.15
51486355	2012	5	21	8990	271.76	11.16
51486355	2012	5	21	9000	271.46	11.17
51486355	2012	5	21	9010	270.33	11.17
51486355	2012	5	21	9020	270.45	11.17
51486355	2012	5	21	9030	270.47	11.17
51486355	2012	5	21	9040	270.62	11.18

51486355	2012	5	21	9050	270.32	11.19
51486355	2012	5	21	9060	270.6	11.19
51486355	2012	5	21	9070	270.66	11.2
51486355	2012	5	21	9080	270.68	11.21
51486355	2012	5	21	9090	269.45	11.2
51486355	2012	5	21	9100	268.21	11.2
51486355	2012	5	21	9110	267.3	11.22
51486355	2012	5	21	9120	266.15	11.22
51486355	2012	5	21	9130	265.05	11.21
51486355	2012	5	21	9140	265.17	11.22
51486355	2012	5	21	9150	266.15	11.28
51486355	2012	5	21	9160	266.23	11.3
51486355	2012	5	21	9170	265.46	11.31
51486355	2012	5	21	9180	264.44	11.32
51486355	2012	5	21	9190	263.68	11.33
51486355	2012	5	21	9200	263.08	11.35
51486355	2012	5	21	9210	261.55	11.38
51486355	2012	5	21	9220	260.97	11.4
51486355	2012	5	21	9230	259.91	11.44
51486355	2012	5	21	9240	258.96	11.44
51486355	2012	5	21	9250	257.61	11.46
51486355	2012	5	21	9260	257.16	11.5
51486355	2012	5	21	9270	257.37	11.55
51486355	2012	5	21	9280	257.62	11.6
51486355	2012	5	21	9290	257.96	11.63
51486355	2012	5	21	9300	257.89	11.63
51486355	2012	5	21	9310	257.73	11.63
51486355	2012	5	21	9320	257.75	11.64
51486355	2012	5	21	9330	258	11.65
51486355	2012	5	21	9340	257.93	11.67
51486355	2012	5	21	9350	258.17	11.75
51486355	2012	5	21	9360	257.67	11.78
51486355	2012	5	21	9370	257.91	11.8
51486355	2012	5	21	9380	258.05	11.82
51486355	2012	5	21	9390	256.43	11.83
51486355	2012	5	21	9400	255.03	11.85
51486355	2012	5	21	9410	254.02	11.86
51486355	2012	5	21	9420	253.65	11.89
51486355	2012	5	21	9430	252.96	11.92
51486355	2012	5	21	9440	253.14	11.92
51486355	2012	5	21	9450	253.45	11.96
51486355	2012	5	21	9460	253.79	11.98
51486355	2012	5	21	9470	253.6	11.99
51486355	2012	5	21	9480	253.43	12
51486355	2012	5	21	9490	252.58	12.01
51486355	2012	5	21	9500	252.8	12.05
51486355	2012	5	21	9510	253.75	12.06

51486355	2012	5	21	9520	254.22	12.08
51486355	2012	5	21	9530	254.16	12.1
51486355	2012	5	21	9540	254.11	12.11
51486355	2012	5	21	9550	253.87	12.13
51486355	2012	5	21	9560	254.04	12.14
51486355	2012	5	21	9570	253.98	12.15
51486355	2012	5	21	9580	253.96	12.17
51486355	2012	5	21	9590	253.18	12.17
51486355	2012	5	21	9600	253.34	12.2
51486355	2012	5	21	9610	253.35	12.21
51486355	2012	5	21	9620	252.86	12.22
51486355	2012	5	21	9630	251.34	12.22
51486355	2012	5	21	9640	250.13	12.27
51486355	2012	5	21	9650	248.8	12.29
51486355	2012	5	21	9660	247.66	12.32
51486355	2012	5	21	9670	246.21	12.34
51486355	2012	5	21	9680	245.28	12.42
51486355	2012	5	21	9690	243.94	12.43
51486355	2012	5	21	9700	242.8	12.45
51486355	2012	5	21	9710	241.01	12.46
51486355	2012	5	21	9720	239.47	12.47
51486355	2012	5	21	9730	239.01	12.53
51486355	2012	5	21	9740	237.35	12.53
51486355	2012	5	21	9750	236.49	12.56
51486355	2012	5	21	9760	236.1	12.61
51486355	2012	5	21	9770	236.21	12.6
51486355	2012	5	21	9780	236.78	12.6
51486355	2012	5	21	9790	237.99	12.63
51486355	2012	5	21	9800	239.17	12.64
51486355	2012	5	21	9810	240.63	12.68
51486355	2012	5	21	9820	240.36	12.69
51486355	2012	5	21	9830	239.02	12.7
51486355	2012	5	21	9840	238.5	12.71
51486355	2012	5	21	9850	239.15	12.72
51486355	2012	5	21	9860	239.33	12.73
51486355	2012	5	21	9870	239.35	12.81
51486355	2012	5	21	9880	239.32	12.82
51486355	2012	5	21	9890	239.43	12.83
51486355	2012	5	21	9900	239.32	12.84
51486355	2012	5	21	9910	239.57	12.86
51486355	2012	5	21	9920	239.49	12.86
51486355	2012	5	21	9930	239.41	12.88
51486355	2012	5	21	9940	239.51	12.89
51486355	2012	5	21	9950	239.33	12.89
51486355	2012	5	21	9960	239.59	12.9
51486355	2012	5	21	9970	239.29	12.91
51486355	2012	5	21	9980	238.33	12.92

51486355	2012	5	21	9990	236.87	12.92
51486355	2012	5	21	10000	235.26	12.92
51486355	2012	5	21	10010	234.17	12.92
51486355	2012	5	21	10020	232.6	12.92
51486355	2012	5	21	10030	230.65	12.94
51486355	2012	5	21	10040	229.84	12.94
51486355	2012	5	21	10050	230.44	12.95
51486355	2012	5	21	10060	230.79	12.96
51486355	2012	5	21	10070	230.77	12.96
51486355	2012	5	21	10080	230.69	12.96
51486355	2012	5	21	10090	230.93	12.96
51486355	2012	5	21	10100	231.13	12.98
51486355	2012	5	21	10110	230.64	12.98
51486355	2012	5	21	10120	229.83	12.98
51486355	2012	5	21	10130	229.99	13
51486355	2012	5	21	10140	229.52	13
51486355	2012	5	21	10150	227.9	13
51486355	2012	5	21	10160	226.81	13
51486355	2012	5	21	10170	226.91	13.02
51486355	2012	5	21	10180	227.59	13.03
51486355	2012	5	21	10190	227.32	13.03
51486355	2012	5	21	10200	226.67	13.03
51486355	2012	5	21	10210	227.1	13.05
51486355	2012	5	21	10220	227.08	13.05
51486355	2012	5	21	10230	227.11	13.07
51486355	2012	5	21	10240	227.26	13.09
51486355	2012	5	21	10250	226.6	13.09
51486355	2012	5	21	10260	226.97	13.1
51486355	2012	5	21	10270	226.69	13.1
51486355	2012	5	21	10280	227.18	13.11
51486355	2012	5	21	10290	227.14	13.11
51486355	2012	5	21	10300	226.85	13.11
51486355	2012	5	21	10310	226.95	13.11
51486355	2012	5	21	10320	227.2	13.12
51486355	2012	5	21	10330	227.04	13.13
51486355	2012	5	21	10340	226.95	13.14
51486355	2012	5	21	10350	227.11	13.15
51486355	2012	5	21	10360	227.06	13.16
51486355	2012	5	21	10370	227.44	13.16
51486355	2012	5	21	10380	227.4	13.15
51486355	2012	5	21	10390	227.58	13.16
51486355	2012	5	21	10400	227.36	13.17
51486355	2012	5	21	10410	227.51	13.17
51486355	2012	5	21	10420	227.02	13.17
51486355	2012	5	21	10430	227.43	13.16
51486355	2012	5	21	10440	227.4	13.16
51486355	2012	5	21	10450	227.24	13.16

51486355	2012	5	21	10460	226.55	13.16
51486355	2012	5	21	10470	226.99	13.16
51486355	2012	5	21	10480	226.83	13.15
51486355	2012	5	21	10490	226.4	13.16
51486355	2012	5	21	10500	225.63	13.15
51486355	2012	5	21	10510	224.55	13.15
51486355	2012	5	21	10520	224.39	13.16
51486355	2012	5	21	10530	223.55	13.15
51486355	2012	5	21	10540	222.76	13.15
51486355	2012	5	21	10550	222.01	13.16
51486355	2012	5	21	10560	221.25	13.17
51486355	2012	5	21	10570	220.32	13.18
51486355	2012	5	21	10580	219.14	13.2
51486355	2012	5	21	10590	218.38	13.21
51486355	2012	5	21	10600	217.31	13.23
51486355	2012	5	21	10610	216.36	13.26
51486355	2012	5	21	10620	215.6	13.29
51486355	2012	5	21	10630	214.8	13.32
51486355	2012	5	21	10640	214.31	13.34
51486355	2012	5	21	10650	214.47	13.39
51486355	2012	5	21	10660	214.26	13.42
51486355	2012	5	21	10670	214.37	13.42
51486355	2012	5	21	10680	214.53	13.46
51486355	2012	5	21	10690	214.38	13.47
51486355	2012	5	21	10700	214.26	13.5
51486355	2012	5	21	10710	213.27	13.5
51486355	2012	5	21	10720	211.34	13.5
51486355	2012	5	21	10730	210.44	13.56
51486355	2012	5	21	10740	210.64	13.66
51486355	2012	5	21	10750	209.77	13.67
51486355	2012	5	21	10760	208.95	13.7
51486355	2012	5	21	10770	208.25	13.74
51486355	2012	5	21	10780	207.43	13.77
51486355	2012	5	21	10790	207.45	13.88
51486355	2012	5	21	10800	206.04	13.91
51486355	2012	5	21	10810	204.3	13.97
51486355	2012	5	21	10820	202.91	14.04
51486355	2012	5	21	10830	201.77	14.1
51486355	2012	5	21	10840	199.74	14.2
51486355	2012	5	21	10850	197.91	14.27
51486355	2012	5	21	10860	196.6	14.38
51486355	2012	5	21	10870	196.34	14.48
51486355	2012	5	21	10880	195.46	14.54
51486355	2012	5	21	10890	193.5	14.61
51486355	2012	5	21	10900	192.14	14.84
51486355	2012	5	21	10910	190.54	14.92
51486355	2012	5	21	10920	189.17	15

51486355	2012	5	21	10930	187.66	15.06
51486355	2012	5	21	10940	187.3	15.21
51486355	2012	5	21	10950	186.34	15.26
51486355	2012	5	21	10960	184.69	15.3
51486355	2012	5	21	10970	182.83	15.35
51486355	2012	5	21	10980	182.72	15.66
51486355	2012	5	21	10990	183.51	15.71
51486355	2012	5	21	11000	183.92	15.74
51486355	2012	5	21	11010	182.68	15.77
51486355	2012	5	21	11020	181.17	15.85
51486355	2012	5	21	11030	180.99	16.09
51486355	2012	5	21	11040	180.38	16.09
51486355	2012	5	21	11050	179.38	16.1
51486355	2012	5	21	11060	178.17	16.12
51486355	2012	5	21	11070	178.21	16.17
51486355	2012	5	21	11080	177.91	16.24
51486355	2012	5	21	11090	177.35	16.29
51486355	2012	5	21	11100	175.86	16.33
51486355	2012	5	21	11110	174.28	16.38
51486355	2012	5	21	11120	174.48	16.56
51486355	2012	5	21	11130	175.01	16.58
51486355	2012	5	21	11140	175.99	16.68
51486355	2012	5	21	11150	176.15	16.71
51486355	2012	5	21	11160	175.33	16.74
51486355	2012	5	21	11170	173.7	16.75
51486355	2012	5	21	11180	172.98	16.76
51486355	2012	5	21	11190	173.08	16.83
51486355	2012	5	21	11200	173.11	16.85
51486355	2012	5	21	11210	173.44	16.87
51486355	2012	5	21	11220	173.5	16.92
51486355	2012	5	21	11230	173.62	17
51486355	2012	5	21	11240	174.47	17
51486355	2012	5	21	11250	174.21	17.03
51486355	2012	5	21	11260	173.88	17.06
51486355	2012	5	21	11270	173.24	17.09
51486355	2012	5	21	11280	172.69	17.13
51486355	2012	5	21	11290	172.89	17.14
51486355	2012	5	21	11300	172.88	17.17
51486355	2012	5	21	11310	173.03	17.18
51486355	2012	5	21	11320	173.05	17.21
51486355	2012	5	21	11330	172.98	17.23
51486355	2012	5	21	11340	172.96	17.25
51486355	2012	5	21	11350	172.94	17.27
51486355	2012	5	21	11360	172.86	17.28
51486355	2012	5	21	11370	172.31	17.3
51486355	2012	5	21	11380	171.17	17.34
51486355	2012	5	21	11390	170.3	17.39

51486355	2012	5	21	11400	169.6	17.39
51486355	2012	5	21	11410	169.26	17.42
51486355	2012	5	21	11420	168.35	17.43
51486355	2012	5	21	11430	168.28	17.46
51486355	2012	5	21	11440	168.28	17.48
51486355	2012	5	21	11450	167.69	17.48
51486355	2012	5	21	11460	167.38	17.5
51486355	2012	5	21	11470	166.8	17.5
51486355	2012	5	21	11480	166.78	17.5
51486355	2012	5	21	11490	166.6	17.52
51486355	2012	5	21	11500	166.65	17.52
51486355	2012	5	21	11510	166.54	17.53
51486355	2012	5	21	11520	166.96	17.54
51486355	2012	5	21	11530	168.07	17.55
51486355	2012	5	21	11540	168.66	17.57
51486355	2012	5	21	11550	168.85	17.57
51486355	2012	5	21	11560	169.13	17.58
51486355	2012	5	21	11570	169.06	17.6
51486355	2012	5	21	11580	169.02	17.61
51486355	2012	5	21	11590	168.83	17.6
51486355	2012	5	21	11600	169.51	17.61
51486355	2012	5	21	11610	170.86	17.63
51486355	2012	5	21	11620	171.84	17.64
51486355	2012	5	21	11630	171.65	17.64
51486355	2012	5	21	11640	172.1	17.64
51486355	2012	5	21	11650	173.33	17.64
51486355	2012	5	21	11660	174.47	17.64
51486355	2012	5	21	11670	175.63	17.65
51486355	2012	5	21	11680	176.05	17.66
51486355	2012	5	21	11690	176.32	17.64
51486355	2012	5	21	11700	176.98	17.64
51486355	2012	5	21	11710	176.76	17.64
51486355	2012	5	21	11720	176.64	17.64
51486355	2012	5	21	11730	176.64	17.63
51486355	2012	5	21	11740	176.51	17.63
51486355	2012	5	21	11750	176.22	17.63
51486355	2012	5	21	11760	175.52	17.62
51486355	2012	5	21	11770	175.66	17.62
51486355	2012	5	21	11780	175.68	17.62
51486355	2012	5	21	11790	175.55	17.61
51486355	2012	5	21	11800	175.49	17.6
51486355	2012	5	21	11810	175.47	17.59
51486355	2012	5	21	11820	175.51	17.58
51486355	2012	5	21	11830	175.31	17.57
51486355	2012	5	21	11840	175.23	17.57
51486355	2012	5	21	11850	175.35	17.56
51486355	2012	5	21	11860	174.91	17.56

51486355	2012	5	21	11870	175.14	17.56
51486355	2012	5	21	11880	174.75	17.56
51486355	2012	5	21	11890	175.32	17.55
51486355	2012	5	21	11900	175.32	17.55
51486355	2012	5	21	11910	175.26	17.55
51486355	2012	5	21	11920	175.51	17.56
51486355	2012	5	21	11930	175.52	17.56
51486355	2012	5	21	11940	175.56	17.56
51486355	2012	5	21	11950	175.74	17.56
51486355	2012	5	21	11960	175.75	17.57
51486355	2012	5	21	11970	175.84	17.57
51486355	2012	5	21	11980	175.61	17.57
51486355	2012	5	21	11990	175.86	17.57
51486355	2012	5	21	12000	175.62	17.57
51486355	2012	5	21	12010	175.26	17.59
51486355	2012	5	21	12020	174.24	17.59
51486355	2012	5	21	12030	174.74	17.58
51486355	2012	5	21	12040	174.68	17.59
51486355	2012	5	21	12050	174.33	17.59
51486355	2012	5	21	12060	173.72	17.59
51486355	2012	5	21	12070	173.45	17.6
51486355	2012	5	21	12080	173.56	17.59
51486355	2012	5	21	12090	173.54	17.59
51486355	2012	5	21	12100	173.15	17.59
51486355	2012	5	21	12110	173.43	17.59
51486355	2012	5	21	12120	173.22	17.58
51486355	2012	5	21	12130	173.05	17.58
51486355	2012	5	21	12140	172.94	17.58
51486355	2012	5	21	12150	172.65	17.57
51486355	2012	5	21	12160	172.56	17.58
51486355	2012	5	21	12170	172.54	17.58
51486355	2012	5	21	12180	172.04	17.58
51486355	2012	5	21	12190	171.13	17.59
51486355	2012	5	21	12200	169.27	17.6
51486355	2012	5	21	12210	167.55	17.6
51486355	2012	5	21	12220	166.94	17.63
51486355	2012	5	21	12230	166.45	17.64
51486355	2012	5	21	12240	165.4	17.64
51486355	2012	5	21	12250	164.48	17.64
51486355	2012	5	21	12260	163.72	17.66
51486355	2012	5	21	12270	163.15	17.67
51486355	2012	5	21	12280	162.56	17.67
51486355	2012	5	21	12290	162.48	17.69
51486355	2012	5	21	12300	162.47	17.7
51486355	2012	5	21	12310	162.12	17.7
51486355	2012	5	21	12320	161.33	17.7
51486355	2012	5	21	12330	160.78	17.71

51486355	2012	5	21	12340	160.96	17.72
51486355	2012	5	21	12350	160.37	17.71
51486355	2012	5	21	12360	159.6	17.72
51486355	2012	5	21	12370	159.54	17.71
51486355	2012	5	21	12380	159.29	17.72
51486355	2012	5	21	12390	159.19	17.72
51486355	2012	5	21	12400	159.72	17.73
51486355	2012	5	21	12410	159.52	17.73
51486355	2012	5	21	12420	159.84	17.73
51486355	2012	5	21	12430	159.63	17.74
51486355	2012	5	21	12440	160.01	17.75
51486355	2012	5	21	12450	159.97	17.75
51486355	2012	5	21	12460	159.73	17.75
51486355	2012	5	21	12470	159.61	17.75
51486355	2012	5	21	12480	159.36	17.75
51486355	2012	5	21	12490	159.48	17.75
51486355	2012	5	21	12500	159.06	17.75
51486355	2012	5	21	12510	158.47	17.75
51486355	2012	5	21	12520	157.68	17.78
51486355	2012	5	21	12530	157.79	17.79
51486355	2012	5	21	12540	158.97	17.78
51486355	2012	5	21	12550	159.74	17.78
51486355	2012	5	21	12560	159.49	17.78
51486355	2012	5	21	12570	158.28	17.78
51486355	2012	5	21	12580	156.95	17.78
51486355	2012	5	21	12590	156.62	17.78
51486355	2012	5	21	12600	156.12	17.79
51486355	2012	5	21	12610	157	17.79
51486355	2012	5	21	12620	156.76	17.79
51486355	2012	5	21	12630	155.45	17.8
51486355	2012	5	21	12640	155.53	17.8
51486355	2012	5	21	12650	156.15	17.8
51486355	2012	5	21	12660	155.67	17.8
51486355	2012	5	21	12670	155.75	17.8
51486355	2012	5	21	12680	155.81	17.8
51486355	2012	5	21	12690	155.98	17.81
51486355	2012	5	21	12700	155.99	17.8
51486355	2012	5	21	12710	156.09	17.81
51486355	2012	5	21	12720	155.84	17.8
51486355	2012	5	21	12730	154.93	17.8
51486355	2012	5	21	12740	154.58	17.81
51486355	2012	5	21	12750	153.34	17.81
51486355	2012	5	21	12760	153.14	17.81
51486355	2012	5	21	12770	152.76	17.81
51486355	2012	5	21	12780	152.7	17.81
51486355	2012	5	21	12790	152.03	17.82
51486355	2012	5	21	12800	152.45	17.82

51486355	2012	5	21	12810	151.91	17.84
51486355	2012	5	21	12820	151.63	17.86
51486355	2012	5	21	12830	151.36	17.87
51486355	2012	5	21	12840	150.66	17.88
51486355	2012	5	21	12850	149.83	17.89
51486355	2012	5	21	12860	148.64	17.91
51486355	2012	5	21	12870	147.61	17.92
51486355	2012	5	21	12880	146.97	17.94
51486355	2012	5	21	12890	146.04	17.98
51486355	2012	5	21	12900	144.95	17.98
51486355	2012	5	21	12910	143.6	18
51486355	2012	5	21	12920	143	18.01
51486355	2012	5	21	12930	142.52	18.03
51486355	2012	5	21	12940	141.63	18.08
51486355	2012	5	21	12950	140.24	18.1
51486355	2012	5	21	12960	139.24	18.14
51486355	2012	5	21	12970	138.39	18.17
51486355	2012	5	21	12980	136.8	18.21
51486355	2012	5	21	12990	134.08	18.25
51486355	2012	5	21	13000	132.31	18.28
51486355	2012	5	21	13010	131.11	18.32
51486355	2012	5	21	13020	129.9	18.36
51486355	2012	5	21	13030	128.28	18.42
51486355	2012	5	21	13040	126.15	18.46
51486355	2012	5	21	13050	125.17	18.47
51486355	2012	5	21	13060	123.93	18.55
51486355	2012	5	21	13070	121.57	18.6
51486355	2012	5	21	13080	120.26	18.64
51486355	2012	5	21	13090	119.13	18.69
51486355	2012	5	21	13100	117.26	18.76
51486355	2012	5	21	13110	115.9	18.81
51486355	2012	5	21	13120	115.05	18.84
51486355	2012	5	21	13130	114.22	18.92
51486355	2012	5	21	13140	113.61	18.96
51486355	2012	5	21	13150	112.49	19.02
51486355	2012	5	21	13160	110.59	19.07
51486355	2012	5	21	13170	108.85	19.11
51486355	2012	5	21	13180	107.79	19.15
51486355	2012	5	21	13190	107.21	19.22
51486355	2012	5	21	13200	106.24	19.28
51486355	2012	5	21	13210	105.11	19.37
51486355	2012	5	21	13220	104.09	19.47
51486355	2012	5	21	13230	103.22	19.58
51486355	2012	5	21	13240	101.7	19.67
51486355	2012	5	21	13250	99.76	19.72
51486355	2012	5	21	13260	98.38	19.81
51486355	2012	5	21	13270	97.26	19.9

51486355	2012	5	21	13280	95.86	20
51486355	2012	5	21	13290	94.23	20.14
51486355	2012	5	21	13300	92.91	20.31
51486355	2012	5	21	13310	92.14	20.73
51486355	2012	5	21	13320	91.62	20.9
51486355	2012	5	21	13330	90.42	21.03
51486355	2012	5	21	13340	88.75	21.18
51486355	2012	5	21	13350	87.55	21.35
51486355	2012	5	21	13360	86.93	21.45
51486355	2012	5	21	13370	85.51	21.58
51486355	2012	5	21	13380	83.98	21.77
51486355	2012	5	21	13390	83.28	21.89
51486355	2012	5	21	13400	82.29	22.04
51486355	2012	5	21	13410	80.51	22.19
51486355	2012	5	21	13420	79.65	22.39
51486355	2012	5	21	13430	78.73	22.5
51486355	2012	5	21	13440	77.02	22.56
51486355	2012	5	21	13450	74.96	22.68
51486355	2012	5	21	13460	73.83	22.84
51486355	2012	5	21	13470	73.11	22.98
51486355	2012	5	21	13480	72.36	23.12
51486355	2012	5	21	13490	71.43	23.35
51486355	2012	5	21	13500	69.75	23.51
51486355	2012	5	21	13510	68.12	23.64
51486355	2012	5	21	13520	66.82	23.85
51486355	2012	5	21	13530	65.99	24.14
51486355	2012	5	21	13540	64.89	24.23
51486355	2012	5	21	13550	63.17	24.28
51486355	2012	5	21	13560	61	24.35
51486355	2012	5	21	13570	59.15	24.47
51486355	2012	5	21	13580	58.2	24.68
51486355	2012	5	21	13590	57.53	24.84
51486355	2012	5	21	13600	56.49	24.91
51486355	2012	5	21	13610	56.06	25.12
51486355	2012	5	21	13620	55.97	25.28
51486355	2012	5	21	13630	55.04	25.29
51486355	2012	5	21	13640	54.94	25.35
51486355	2012	5	21	13650	55.34	25.52
51486355	2012	5	21	13660	55.02	25.56
51486355	2012	5	21	13670	55.36	25.63
51486355	2012	5	21	13680	54.86	25.68
51486355	2012	5	21	13690	53.82	25.74
51486355	2012	5	21	13700	53.65	25.89
51486355	2012	5	21	13710	53.3	25.96
51486355	2012	5	21	13720	52.93	26
51486355	2012	5	21	13730	52.24	26.03
51486355	2012	5	21	13740	51.1	26.05

51486355	2012	5	21	13750	50.07	26.08
51486355	2012	5	21	13760	48.82	26.1
51486355	2012	5	21	13770	47.71	26.13
51486355	2012	5	21	13780	46.27	26.2
51486355	2012	5	21	13790	43.9	26.26
51486355	2012	5	21	13800	42.14	26.35
51486355	2012	5	21	13810	40.67	26.39
51486355	2012	5	21	13820	38.19	26.45
51486355	2012	5	21	13830	35.71	26.53
51486355	2012	5	21	13840	34.3	26.59
51486355	2012	5	21	13850	32.4	26.63
51486355	2012	5	21	13860	29.53	26.69
51486355	2012	5	21	13870	27.08	26.74
51486355	2012	5	21	13880	25.26	26.78
51486355	2012	5	21	13890	23.8	26.82
51486355	2012	5	21	13900	21.75	26.85
51486355	2012	5	21	13910	18.72	26.89
51486355	2012	5	21	13920	16.24	26.94
51486355	2012	5	21	13930	13.92	26.99
51486355	2012	5	21	13940	11.34	27.05
51486355	2012	5	21	13950	9.57	27.1
51486355	2012	5	21	13960	7.82	27.13
51486355	2012	5	21	13970	5.19	27.16
51486355	2012	5	21	13980	3.08	27.2
51486355	2012	5	21	13990	2.49	27.25
51486355	2012	5	21	14000	1.91	27.28
51486355	2012	5	21	14010	1.72	27.31
51486355	2012	5	21	14020	1.69	27.34
51486355	2012	5	21	14030	1.86	27.38
51486355	2012	5	21	14040	0.72	27.37
51486355	2012	5	21	14050	0.6	27.37
51486355	2012	5	21	14060	0.62	27.37
51486355	2012	5	21	14070	0.68	27.41
51486355	2012	5	21	14080	0.62	27.48
51486355	2012	5	21	14090	0.63	27.5
51486355	2012	5	21	14100	0.61	27.51
51486355	2012	5	21	14110	0.68	27.53
51486355	2012	5	21	14120	0.58	27.57
51486355	2012	5	21	14130	0.57	27.57
51486355	2012	5	21	14140	0.55	27.59
51486355	2012	5	21	14150	0.53	27.61
51486355	2012	5	21	14160	0.56	27.62
51486355	2012	5	21	14170	0.56	27.63
51486355	2012	5	21	14180	0.56	27.65
51559905	2012	5	22	0	1.28	27.6
51559905	2012	5	22	10	2.23	27.59
51559905	2012	5	22	20	2.75	27.58

51559905	2012	5	22	30	2.88	27.58
51559905	2012	5	22	40	2.7	27.59
51559905	2012	5	22	50	2.83	27.58
51559905	2012	5	22	60	3.08	27.58
51559905	2012	5	22	70	3.74	27.58
51559905	2012	5	22	80	5.55	27.57
51559905	2012	5	22	90	6.5	27.56
51559905	2012	5	22	100	8.01	27.55
51559905	2012	5	22	110	8.92	27.54
51559905	2012	5	22	120	9.77	27.53
51559905	2012	5	22	130	10.74	27.52
51559905	2012	5	22	140	11.64	27.52
51559905	2012	5	22	150	12.18	27.51
51559905	2012	5	22	160	12.76	27.51
51559905	2012	5	22	170	13.31	27.5
51559905	2012	5	22	180	13.93	27.5
51559905	2012	5	22	190	13.88	27.5
51559905	2012	5	22	200	14.09	27.5
51559905	2012	5	22	210	14.41	27.5
51559905	2012	5	22	220	14.92	27.5
51559905	2012	5	22	230	15.15	27.5
51559905	2012	5	22	240	16.29	27.48
51559905	2012	5	22	250	17.46	27.47
51559905	2012	5	22	260	18.28	27.46
51559905	2012	5	22	270	19.18	27.46
51559905	2012	5	22	280	20.75	27.44
51559905	2012	5	22	290	21.81	27.44
51559905	2012	5	22	300	23.18	27.42
51559905	2012	5	22	310	24.97	27.41
51559905	2012	5	22	320	25.45	27.41
51559905	2012	5	22	330	25.73	27.42
51559905	2012	5	22	340	25.82	27.41
51559905	2012	5	22	350	25.93	27.4
51559905	2012	5	22	360	25.83	27.41
51559905	2012	5	22	370	25.61	27.41
51559905	2012	5	22	380	26.48	27.4
51559905	2012	5	22	390	27.05	27.4
51559905	2012	5	22	400	28.08	27.39
51559905	2012	5	22	410	30.46	27.37
51559905	2012	5	22	420	32.95	27.35
51559905	2012	5	22	430	35.71	27.33
51559905	2012	5	22	440	38.6	27.31
51559905	2012	5	22	450	41.67	27.29
51559905	2012	5	22	460	43.5	27.28
51559905	2012	5	22	470	45.11	27.28
51559905	2012	5	22	480	46.54	27.26
51559905	2012	5	22	490	47.35	27.25

51559905	2012	5	22	500	48.5	27.25
51559905	2012	5	22	510	49.74	27.24
51559905	2012	5	22	520	52.01	27.22
51559905	2012	5	22	530	54.98	27.21
51559905	2012	5	22	540	57.89	27.18
51559905	2012	5	22	550	61.18	27.17
51559905	2012	5	22	560	62.94	27.15
51559905	2012	5	22	570	63.27	27.15
51559905	2012	5	22	580	64.68	27.14
51559905	2012	5	22	590	66.55	27.14
51559905	2012	5	22	600	67.35	27.02
51559905	2012	5	22	610	68.06	26.96
51559905	2012	5	22	620	69.52	26.89
51559905	2012	5	22	630	70.88	26.81
51559905	2012	5	22	640	71.37	26.71
51559905	2012	5	22	650	71.65	26.6
51559905	2012	5	22	660	73.23	26.43
51559905	2012	5	22	670	74.51	26.32
51559905	2012	5	22	680	75.57	26.22
51559905	2012	5	22	690	77.2	26.04
51559905	2012	5	22	700	78.61	25.99
51559905	2012	5	22	710	79.05	25.92
51559905	2012	5	22	720	81.01	25.85
51559905	2012	5	22	730	83.48	25.74
51559905	2012	5	22	740	85.37	25.58
51559905	2012	5	22	750	87.34	25.49
51559905	2012	5	22	760	90.11	25.35
51559905	2012	5	22	770	92.31	25.18
51559905	2012	5	22	780	94.15	25.08
51559905	2012	5	22	790	95.95	24.93
51559905	2012	5	22	800	98.28	24.85
51559905	2012	5	22	810	100.45	24.72
51559905	2012	5	22	820	101.88	24.56
51559905	2012	5	22	830	102.87	24.35
51559905	2012	5	22	840	104.59	24.27
51559905	2012	5	22	850	106.28	24.21
51559905	2012	5	22	860	107.83	24.08
51559905	2012	5	22	870	109.85	23.98
51559905	2012	5	22	880	111.19	23.85
51559905	2012	5	22	890	112.19	23.74
51559905	2012	5	22	900	113.54	23.72
51559905	2012	5	22	910	114.23	23.6
51559905	2012	5	22	920	115.54	23.57
51559905	2012	5	22	930	116.17	23.53
51559905	2012	5	22	940	116.54	23.46
51559905	2012	5	22	950	117.11	23.32
51559905	2012	5	22	960	118.53	23.28

51559905	2012	5	22	970	118.91	23.24
51559905	2012	5	22	980	120.33	23.21
51559905	2012	5	22	990	122.18	23.17
51559905	2012	5	22	1000	123.17	23.14
51559905	2012	5	22	1010	123.78	23.07
51559905	2012	5	22	1020	125.26	23.03
51559905	2012	5	22	1030	126.96	23
51559905	2012	5	22	1040	127.67	22.96
51559905	2012	5	22	1050	128.85	22.9
51559905	2012	5	22	1060	129.92	22.84
51559905	2012	5	22	1070	131.12	22.71
51559905	2012	5	22	1080	132.71	22.5
51559905	2012	5	22	1090	133.42	22.29
51559905	2012	5	22	1100	133.69	22.14
51559905	2012	5	22	1110	135.3	21.95
51559905	2012	5	22	1120	135.81	21.84
51559905	2012	5	22	1130	135.5	21.75
51559905	2012	5	22	1140	135.66	21.69
51559905	2012	5	22	1150	135.62	21.63
51559905	2012	5	22	1160	135.59	21.35
51559905	2012	5	22	1170	135.88	21.32
51559905	2012	5	22	1180	137.39	21.25
51559905	2012	5	22	1190	138.2	21.11
51559905	2012	5	22	1200	139.85	21.03
51559905	2012	5	22	1210	141.64	21
51559905	2012	5	22	1220	141.76	20.92
51559905	2012	5	22	1230	143.11	20.77
51559905	2012	5	22	1240	145.12	20.67
51559905	2012	5	22	1250	146.98	20.59
51559905	2012	5	22	1260	148.8	20.47
51559905	2012	5	22	1270	150.72	20.37
51559905	2012	5	22	1280	152.1	20.19
51559905	2012	5	22	1290	154.23	20.05
51559905	2012	5	22	1300	155.97	20
51559905	2012	5	22	1310	157.85	19.94
51559905	2012	5	22	1320	158.76	19.86
51559905	2012	5	22	1330	160.13	19.78
51559905	2012	5	22	1340	161.33	19.71
51559905	2012	5	22	1350	161.07	19.57
51559905	2012	5	22	1360	162.08	19.39
51559905	2012	5	22	1370	163.35	19.36
51559905	2012	5	22	1380	165.25	19.3
51559905	2012	5	22	1390	166.83	19.25
51559905	2012	5	22	1400	168.72	19.12
51559905	2012	5	22	1410	169.63	18.93
51559905	2012	5	22	1420	170.05	18.78
51559905	2012	5	22	1430	170.62	18.73

51559905	2012	5	22	1440	172.28	18.67
51559905	2012	5	22	1450	173.25	18.64
51559905	2012	5	22	1460	173.33	18.57
51559905	2012	5	22	1470	173.28	18.42
51559905	2012	5	22	1480	174.25	18.39
51559905	2012	5	22	1490	175.49	18.32
51559905	2012	5	22	1500	176.47	18.17
51559905	2012	5	22	1510	178.19	18.08
51559905	2012	5	22	1520	179.89	18
51559905	2012	5	22	1530	181.28	17.95
51559905	2012	5	22	1540	182.47	17.8
51559905	2012	5	22	1550	184.52	17.82
51559905	2012	5	22	1560	185.3	17.8
51559905	2012	5	22	1570	185.35	17.76
51559905	2012	5	22	1580	186.61	17.64
51559905	2012	5	22	1590	188.24	17.59
51559905	2012	5	22	1600	188.65	17.5
51559905	2012	5	22	1610	189.68	17.35
51559905	2012	5	22	1620	190.75	17.19
51559905	2012	5	22	1630	192.71	17.2
51559905	2012	5	22	1640	193.4	17.13
51559905	2012	5	22	1650	193.2	17.07
51559905	2012	5	22	1660	194.32	17.03
51559905	2012	5	22	1670	196.05	16.99
51559905	2012	5	22	1680	197.33	16.88
51559905	2012	5	22	1690	198.47	16.79
51559905	2012	5	22	1700	199.63	16.67
51559905	2012	5	22	1710	200.25	16.61
51559905	2012	5	22	1720	201.32	16.48
51559905	2012	5	22	1730	201.62	16.42
51559905	2012	5	22	1740	202.36	16.39
51559905	2012	5	22	1750	202.11	16.35
51559905	2012	5	22	1760	201.89	16.31
51559905	2012	5	22	1770	201.55	16.24
51559905	2012	5	22	1780	201.97	16.18
51559905	2012	5	22	1790	202.19	16.14
51559905	2012	5	22	1800	202.4	15.97
51559905	2012	5	22	1810	202.45	15.94
51559905	2012	5	22	1820	203.34	15.94
51559905	2012	5	22	1830	203.15	15.88
51559905	2012	5	22	1840	202.43	15.85
51559905	2012	5	22	1850	201.81	15.82
51559905	2012	5	22	1860	201.25	15.8
51559905	2012	5	22	1870	200.63	15.77
51559905	2012	5	22	1880	200.5	15.73
51559905	2012	5	22	1890	200.37	15.65
51559905	2012	5	22	1900	200.42	15.62

51559905	2012	5	22	1910	200.28	15.6
51559905	2012	5	22	1920	199.6	15.59
51559905	2012	5	22	1930	198.47	15.57
51559905	2012	5	22	1940	197.79	15.57
51559905	2012	5	22	1950	197.3	15.56
51559905	2012	5	22	1960	196.71	15.54
51559905	2012	5	22	1970	196.37	15.53
51559905	2012	5	22	1980	196.38	15.51
51559905	2012	5	22	1990	197.59	15.51
51559905	2012	5	22	2000	198.68	15.51
51559905	2012	5	22	2010	199.13	15.49
51559905	2012	5	22	2020	198.51	15.5
51559905	2012	5	22	2030	198.37	15.48
51559905	2012	5	22	2040	198.16	15.47
51559905	2012	5	22	2050	197.8	15.47
51559905	2012	5	22	2060	197.37	15.46
51559905	2012	5	22	2070	197.16	15.46
51559905	2012	5	22	2080	196.95	15.46
51559905	2012	5	22	2090	197.54	15.46
51559905	2012	5	22	2100	199.14	15.47
51559905	2012	5	22	2110	199.91	15.47
51559905	2012	5	22	2120	199.96	15.47
51559905	2012	5	22	2130	199.64	15.46
51559905	2012	5	22	2140	199.63	15.46
51559905	2012	5	22	2150	199.87	15.45
51559905	2012	5	22	2160	200.52	15.43
51559905	2012	5	22	2170	200.47	15.44
51559905	2012	5	22	2180	199.95	15.42
51559905	2012	5	22	2190	199.51	15.42
51559905	2012	5	22	2200	199.81	15.42
51559905	2012	5	22	2210	199.92	15.41
51559905	2012	5	22	2220	199.76	15.41
51559905	2012	5	22	2230	199.94	15.41
51559905	2012	5	22	2240	200.67	15.41
51559905	2012	5	22	2250	200.4	15.41
51559905	2012	5	22	2260	200.59	15.4
51559905	2012	5	22	2270	200.93	15.39
51559905	2012	5	22	2280	200.79	15.39
51559905	2012	5	22	2290	200.66	15.39
51559905	2012	5	22	2300	200.55	15.39
51559905	2012	5	22	2310	200.51	15.39
51559905	2012	5	22	2320	200.33	15.39
51559905	2012	5	22	2330	200.53	15.38
51559905	2012	5	22	2340	200.7	15.37
51559905	2012	5	22	2350	200.44	15.37
51559905	2012	5	22	2360	199.83	15.37
51559905	2012	5	22	2370	199.05	15.36

51559905	2012	5	22	2380	199.18	15.36
51559905	2012	5	22	2390	200.34	15.35
51559905	2012	5	22	2400	200.71	15.36
51559905	2012	5	22	2410	200.56	15.35
51559905	2012	5	22	2420	200.18	15.35
51559905	2012	5	22	2430	200.9	15.35
51559905	2012	5	22	2440	200.97	15.36
51559905	2012	5	22	2450	200.57	15.36
51559905	2012	5	22	2460	200.61	15.37
51559905	2012	5	22	2470	200.42	15.37
51559905	2012	5	22	2480	199.97	15.39
51559905	2012	5	22	2490	200.55	15.4
51559905	2012	5	22	2500	200.78	15.4
51559905	2012	5	22	2510	200.66	15.41
51559905	2012	5	22	2520	200.32	15.41
51559905	2012	5	22	2530	199.73	15.42
51559905	2012	5	22	2540	198.99	15.42
51559905	2012	5	22	2550	198.18	15.42
51559905	2012	5	22	2560	197.26	15.43
51559905	2012	5	22	2570	196.3	15.44
51559905	2012	5	22	2580	195.44	15.45
51559905	2012	5	22	2590	195.34	15.46
51559905	2012	5	22	2600	196.35	15.47
51559905	2012	5	22	2610	197.6	15.48
51559905	2012	5	22	2620	198.21	15.5
51559905	2012	5	22	2630	198.65	15.5
51559905	2012	5	22	2640	199.18	15.53
51559905	2012	5	22	2650	199.62	15.54
51559905	2012	5	22	2660	199.89	15.54
51559905	2012	5	22	2670	199.98	15.54
51559905	2012	5	22	2680	200.1	15.55
51559905	2012	5	22	2690	200.1	15.55
51559905	2012	5	22	2700	199.79	15.55
51559905	2012	5	22	2710	199.2	15.56
51559905	2012	5	22	2720	198.52	15.56
51559905	2012	5	22	2730	197.81	15.57
51559905	2012	5	22	2740	197.37	15.57
51559905	2012	5	22	2750	197.02	15.57
51559905	2012	5	22	2760	197.35	15.57
51559905	2012	5	22	2770	198.09	15.58
51559905	2012	5	22	2780	198.55	15.58
51559905	2012	5	22	2790	198.79	15.58
51559905	2012	5	22	2800	199.01	15.58
51559905	2012	5	22	2810	198.42	15.6
51559905	2012	5	22	2820	197.93	15.6
51559905	2012	5	22	2830	197.76	15.63
51559905	2012	5	22	2840	197.29	15.64

51559905	2012	5	22	2850	195.86	15.66
51559905	2012	5	22	2860	195.42	15.68
51559905	2012	5	22	2870	195.2	15.7
51559905	2012	5	22	2880	195.15	15.71
51559905	2012	5	22	2890	194.62	15.72
51559905	2012	5	22	2900	193.94	15.76
51559905	2012	5	22	2910	193.12	15.79
51559905	2012	5	22	2920	192.12	15.82
51559905	2012	5	22	2930	191.94	15.85
51559905	2012	5	22	2940	191.08	15.94
51559905	2012	5	22	2950	190.4	15.98
51559905	2012	5	22	2960	189.9	16
51559905	2012	5	22	2970	189.26	16.03
51559905	2012	5	22	2980	189.03	16.07
51559905	2012	5	22	2990	188.22	16.1
51559905	2012	5	22	3000	187.57	16.13
51559905	2012	5	22	3010	187.13	16.14
51559905	2012	5	22	3020	186.78	16.18
51559905	2012	5	22	3030	186.62	16.23
51559905	2012	5	22	3040	186.46	16.27
51559905	2012	5	22	3050	186.91	16.29
51559905	2012	5	22	3060	187.3	16.32
51559905	2012	5	22	3070	187.26	16.34
51559905	2012	5	22	3080	187.11	16.36
51559905	2012	5	22	3090	187.62	16.41
51559905	2012	5	22	3100	188.25	16.42
51559905	2012	5	22	3110	188.59	16.46
51559905	2012	5	22	3120	189.3	16.49
51559905	2012	5	22	3130	189.73	16.5
51559905	2012	5	22	3140	189.7	16.51
51559905	2012	5	22	3150	189.39	16.52
51559905	2012	5	22	3160	189.44	16.53
51559905	2012	5	22	3170	190.04	16.56
51559905	2012	5	22	3180	189.94	16.57
51559905	2012	5	22	3190	189.41	16.58
51559905	2012	5	22	3200	189.32	16.6
51559905	2012	5	22	3210	189.95	16.62
51559905	2012	5	22	3220	190.66	16.64
51559905	2012	5	22	3230	191.12	16.64
51559905	2012	5	22	3240	191.12	16.64
51559905	2012	5	22	3250	191.17	16.66
51559905	2012	5	22	3260	191.06	16.67
51559905	2012	5	22	3270	190.48	16.67
51559905	2012	5	22	3280	190.95	16.68
51559905	2012	5	22	3290	191.24	16.69
51559905	2012	5	22	3300	191.44	16.71
51559905	2012	5	22	3310	191.46	16.71

51559905	2012	5	22	3320	191.11	16.7
51559905	2012	5	22	3330	190.59	16.71
51559905	2012	5	22	3340	190.36	16.71
51559905	2012	5	22	3350	190.36	16.72
51559905	2012	5	22	3360	190.6	16.72
51559905	2012	5	22	3370	190.54	16.73
51559905	2012	5	22	3380	190.23	16.74
51559905	2012	5	22	3390	190.27	16.73
51559905	2012	5	22	3400	190.09	16.75
51559905	2012	5	22	3410	189.88	16.76
51559905	2012	5	22	3420	189.85	16.76
51559905	2012	5	22	3430	190.23	16.77
51559905	2012	5	22	3440	189.86	16.78
51559905	2012	5	22	3450	189.64	16.78
51559905	2012	5	22	3460	190.23	16.78
51559905	2012	5	22	3470	190.47	16.78
51559905	2012	5	22	3480	190.06	16.79
51559905	2012	5	22	3490	189.65	16.78
51559905	2012	5	22	3500	190.29	16.8
51559905	2012	5	22	3510	190.14	16.79
51559905	2012	5	22	3520	189.9	16.79
51559905	2012	5	22	3530	189.62	16.8
51559905	2012	5	22	3540	189.76	16.8
51559905	2012	5	22	3550	190.03	16.81
51559905	2012	5	22	3560	189.82	16.81
51559905	2012	5	22	3570	189.59	16.81
51559905	2012	5	22	3580	189.21	16.81
51559905	2012	5	22	3590	188.7	16.81
51559905	2012	5	22	3600	188.32	16.82
51559905	2012	5	22	3610	188.16	16.82
51559905	2012	5	22	3620	188.57	16.82
51559905	2012	5	22	3630	188.63	16.82
51559905	2012	5	22	3640	188.41	16.82
51559905	2012	5	22	3650	188.4	16.83
51559905	2012	5	22	3660	188.08	16.83
51559905	2012	5	22	3670	187.97	16.82
51559905	2012	5	22	3680	187.95	16.84
51559905	2012	5	22	3690	188.02	16.83
51559905	2012	5	22	3700	188.16	16.85
51559905	2012	5	22	3710	187.22	16.84
51559905	2012	5	22	3720	186.79	16.85
51559905	2012	5	22	3730	186.66	16.85
51559905	2012	5	22	3740	186.62	16.87
51559905	2012	5	22	3750	186.25	16.87
51559905	2012	5	22	3760	184.96	16.87
51559905	2012	5	22	3770	184.61	16.87
51559905	2012	5	22	3780	183.88	16.87

51559905	2012	5	22	3790	183.21	16.88
51559905	2012	5	22	3800	183.26	16.89
51559905	2012	5	22	3810	182.54	16.89
51559905	2012	5	22	3820	181.71	16.9
51559905	2012	5	22	3830	181.39	16.9
51559905	2012	5	22	3840	181.37	16.91
51559905	2012	5	22	3850	181.13	16.92
51559905	2012	5	22	3860	180.58	16.97
51559905	2012	5	22	3870	179.56	16.98
51559905	2012	5	22	3880	179.25	17
51559905	2012	5	22	3890	178.84	17.02
51559905	2012	5	22	3900	179.07	17.04
51559905	2012	5	22	3910	178.6	17.05
51559905	2012	5	22	3920	177.98	17.07
51559905	2012	5	22	3930	178.19	17.09
51559905	2012	5	22	3940	177.44	17.1
51559905	2012	5	22	3950	177.22	17.12
51559905	2012	5	22	3960	177.64	17.12
51559905	2012	5	22	3970	177.83	17.15
51559905	2012	5	22	3980	178.22	17.16
51559905	2012	5	22	3990	178.8	17.17
51559905	2012	5	22	4000	178.79	17.19
51559905	2012	5	22	4010	179.55	17.2
51559905	2012	5	22	4020	179.8	17.21
51559905	2012	5	22	4030	179.98	17.21
51559905	2012	5	22	4040	180.07	17.25
51559905	2012	5	22	4050	180.05	17.25
51559905	2012	5	22	4060	180.25	17.25
51559905	2012	5	22	4070	180.3	17.25
51559905	2012	5	22	4080	180.23	17.27
51559905	2012	5	22	4090	180	17.26
51559905	2012	5	22	4100	179.97	17.28
51559905	2012	5	22	4110	179.63	17.28
51559905	2012	5	22	4120	179.26	17.28
51559905	2012	5	22	4130	179.1	17.29
51559905	2012	5	22	4140	179.02	17.3
51559905	2012	5	22	4150	178.41	17.3
51559905	2012	5	22	4160	177.75	17.3
51559905	2012	5	22	4170	176.82	17.31
51559905	2012	5	22	4180	176.23	17.32
51559905	2012	5	22	4190	175.71	17.32
51559905	2012	5	22	4200	174.8	17.32
51559905	2012	5	22	4210	174.08	17.34
51559905	2012	5	22	4220	173.57	17.34
51559905	2012	5	22	4230	173.11	17.35
51559905	2012	5	22	4240	172.24	17.35
51559905	2012	5	22	4250	170.96	17.36

51559905	2012	5	22	4260	169.43	17.38
51559905	2012	5	22	4270	168.43	17.39
51559905	2012	5	22	4280	168.7	17.39
51559905	2012	5	22	4290	167.8	17.42
51559905	2012	5	22	4300	166.51	17.44
51559905	2012	5	22	4310	165.72	17.46
51559905	2012	5	22	4320	163.86	17.46
51559905	2012	5	22	4330	162.87	17.49
51559905	2012	5	22	4340	161.79	17.51
51559905	2012	5	22	4350	160.8	17.53
51559905	2012	5	22	4360	159.55	17.57
51559905	2012	5	22	4370	158.18	17.6
51559905	2012	5	22	4380	157.06	17.64
51559905	2012	5	22	4390	156.21	17.66
51559905	2012	5	22	4400	154.91	17.68
51559905	2012	5	22	4410	153.95	17.7
51559905	2012	5	22	4420	152.93	17.72
51559905	2012	5	22	4430	152.09	17.74
51559905	2012	5	22	4440	151.06	17.76
51559905	2012	5	22	4450	150.23	17.78
51559905	2012	5	22	4460	149.34	17.81
51559905	2012	5	22	4470	148.27	17.82
51559905	2012	5	22	4480	147.56	17.85
51559905	2012	5	22	4490	146.84	17.87
51559905	2012	5	22	4500	146.32	17.88
51559905	2012	5	22	4510	146.04	17.9
51559905	2012	5	22	4520	144.75	17.92
51559905	2012	5	22	4530	143.04	17.98
51559905	2012	5	22	4540	141.87	18.04
51559905	2012	5	22	4550	140.38	18.14
51559905	2012	5	22	4560	138.63	18.2
51559905	2012	5	22	4570	138.97	18.26
51559905	2012	5	22	4580	138.14	18.32
51559905	2012	5	22	4590	136.81	18.35
51559905	2012	5	22	4600	135.02	18.41
51559905	2012	5	22	4610	133.76	18.47
51559905	2012	5	22	4620	132.14	18.53
51559905	2012	5	22	4630	131.39	18.62
51559905	2012	5	22	4640	130.05	18.64
51559905	2012	5	22	4650	129.09	18.7
51559905	2012	5	22	4660	128.62	18.73
51559905	2012	5	22	4670	128.04	18.74
51559905	2012	5	22	4680	127.26	18.76
51559905	2012	5	22	4690	126.42	18.78
51559905	2012	5	22	4700	125.47	18.82
51559905	2012	5	22	4710	124.65	18.85
51559905	2012	5	22	4720	124.52	18.89

51559905	2012	5	22	4730	124.25	18.94
51559905	2012	5	22	4740	124.14	18.96
51559905	2012	5	22	4750	124.22	18.97
51559905	2012	5	22	4760	123.02	19.03
51559905	2012	5	22	4770	121.35	19.06
51559905	2012	5	22	4780	119.34	19.1
51559905	2012	5	22	4790	117.54	19.11
51559905	2012	5	22	4800	115.5	19.17
51559905	2012	5	22	4810	113.56	19.2
51559905	2012	5	22	4820	111.83	19.25
51559905	2012	5	22	4830	109.83	19.34
51559905	2012	5	22	4840	108.45	19.6
51559905	2012	5	22	4850	107.54	19.64
51559905	2012	5	22	4860	106.97	19.71
51559905	2012	5	22	4870	106.74	19.76
51559905	2012	5	22	4880	106.66	19.82
51559905	2012	5	22	4890	106.59	19.85
51559905	2012	5	22	4900	106.63	19.89
51559905	2012	5	22	4910	106.52	19.92
51559905	2012	5	22	4920	106.17	19.96
51559905	2012	5	22	4930	106.43	20.13
51559905	2012	5	22	4940	106.6	20.21
51559905	2012	5	22	4950	106.73	20.3
51559905	2012	5	22	4960	106.58	20.36
51559905	2012	5	22	4970	106.62	20.42
51559905	2012	5	22	4980	106.84	20.47
51559905	2012	5	22	4990	107.08	20.53
51559905	2012	5	22	5000	106.78	20.58
51559905	2012	5	22	5010	106.46	20.62
51559905	2012	5	22	5020	106.41	20.63
51559905	2012	5	22	5030	106.05	20.68
51559905	2012	5	22	5040	105.83	20.83
51559905	2012	5	22	5050	105.51	20.89
51559905	2012	5	22	5060	105.37	20.92
51559905	2012	5	22	5070	105.31	20.97
51559905	2012	5	22	5080	105.04	21
51559905	2012	5	22	5090	103.84	21.03
51559905	2012	5	22	5100	102.76	21.09
51559905	2012	5	22	5110	101.86	21.15
51559905	2012	5	22	5120	101.19	21.23
51559905	2012	5	22	5130	100.45	21.31
51559905	2012	5	22	5140	99.48	21.38
51559905	2012	5	22	5150	98.92	21.42
51559905	2012	5	22	5160	97.89	21.45
51559905	2012	5	22	5170	96.85	21.5
51559905	2012	5	22	5180	96.75	21.58
51559905	2012	5	22	5190	95.82	21.64

51559905	2012	5	22	5200	94.59	21.67
51559905	2012	5	22	5210	93.21	21.7
51559905	2012	5	22	5220	92.24	21.73
51559905	2012	5	22	5230	91.9	21.76
51559905	2012	5	22	5240	91.65	21.8
51559905	2012	5	22	5250	91.61	21.84
51559905	2012	5	22	5260	91.69	21.87
51559905	2012	5	22	5270	91.4	21.91
51559905	2012	5	22	5280	91.01	21.95
51559905	2012	5	22	5290	90.62	21.98
51559905	2012	5	22	5300	90.31	22.02
51559905	2012	5	22	5310	89.78	22.05
51559905	2012	5	22	5320	89.27	22.1
51559905	2012	5	22	5330	89.17	22.11
51559905	2012	5	22	5340	89.01	22.14
51559905	2012	5	22	5350	88.71	22.16
51559905	2012	5	22	5360	88.65	22.2
51559905	2012	5	22	5370	88.75	22.23
51559905	2012	5	22	5380	88.71	22.26
51559905	2012	5	22	5390	88.85	22.26
51559905	2012	5	22	5400	88.75	22.27
51559905	2012	5	22	5410	88.64	22.3
51559905	2012	5	22	5420	88.41	22.32
51559905	2012	5	22	5430	88.07	22.32
51559905	2012	5	22	5440	88.17	22.33
51559905	2012	5	22	5450	88.17	22.35
51559905	2012	5	22	5460	87.99	22.36
51559905	2012	5	22	5470	87.85	22.36
51559905	2012	5	22	5480	88.39	22.37
51559905	2012	5	22	5490	88.6	22.38
51559905	2012	5	22	5500	88.37	22.39
51559905	2012	5	22	5510	88.48	22.39
51559905	2012	5	22	5520	88.5	22.4
51559905	2012	5	22	5530	88.24	22.42
51559905	2012	5	22	5540	89.07	22.42
51559905	2012	5	22	5550	89.5	22.42
51559905	2012	5	22	5560	89.63	22.42
51559905	2012	5	22	5570	89.64	22.43
51559905	2012	5	22	5580	90.07	22.44
51559905	2012	5	22	5590	90.85	22.44
51559905	2012	5	22	5600	92.3	22.46
51559905	2012	5	22	5610	93.97	22.45
51559905	2012	5	22	5620	95.95	22.44
51559905	2012	5	22	5630	98.35	22.43
51559905	2012	5	22	5640	99.44	22.42
51559905	2012	5	22	5650	98.7	22.42
51559905	2012	5	22	5660	96.91	22.43

51559905	2012	5	22	5670	94.84	22.44
51559905	2012	5	22	5680	92.47	22.45
51559905	2012	5	22	5690	89.9	22.46
51559905	2012	5	22	5700	87.65	22.48
51559905	2012	5	22	5710	85.48	22.5
51559905	2012	5	22	5720	82.99	22.52
51559905	2012	5	22	5730	81.89	22.54
51559905	2012	5	22	5740	81.79	22.55
51559905	2012	5	22	5750	81.95	22.57
51559905	2012	5	22	5760	82.16	22.58
51559905	2012	5	22	5770	82.21	22.59
51559905	2012	5	22	5780	82.36	22.6
51559905	2012	5	22	5790	82.44	22.6
51559905	2012	5	22	5800	81.97	22.62
51559905	2012	5	22	5810	81.72	22.64
51559905	2012	5	22	5820	81.61	22.66
51559905	2012	5	22	5830	81.77	22.7
51559905	2012	5	22	5840	81.81	22.69
51559905	2012	5	22	5850	81.83	22.71
51559905	2012	5	22	5860	81.35	22.71
51559905	2012	5	22	5870	81.45	22.75
51559905	2012	5	22	5880	81.61	22.75
51559905	2012	5	22	5890	81.56	22.75
51559905	2012	5	22	5900	81.27	22.75
51559905	2012	5	22	5910	80.79	22.75
51559905	2012	5	22	5920	81	22.76
51559905	2012	5	22	5930	81.38	22.78
51559905	2012	5	22	5940	82.19	22.78
51559905	2012	5	22	5950	82.95	22.79
51559905	2012	5	22	5960	82.89	22.81
51559905	2012	5	22	5970	82.66	22.83
51559905	2012	5	22	5980	82.69	22.85
51559905	2012	5	22	5990	82.55	22.87
51559905	2012	5	22	6000	82.27	22.89
51559905	2012	5	22	6010	81.84	22.9
51559905	2012	5	22	6020	81.55	22.92
51559905	2012	5	22	6030	81.43	22.93
51559905	2012	5	22	6040	81.27	22.94
51559905	2012	5	22	6050	81.1	22.96
51559905	2012	5	22	6060	81.31	22.96
51559905	2012	5	22	6070	81.36	22.96
51559905	2012	5	22	6080	81.21	22.97
51559905	2012	5	22	6090	80.98	22.99
51559905	2012	5	22	6100	80.84	23
51559905	2012	5	22	6110	80.74	23
51559905	2012	5	22	6120	80.54	23.01
51559905	2012	5	22	6130	80.25	23.03

51559905	2012	5	22	6140	80.11	23.03
51559905	2012	5	22	6150	79.92	23.05
51559905	2012	5	22	6160	79.65	23.1
51559905	2012	5	22	6170	80	23.12
51559905	2012	5	22	6180	80.5	23.12
51559905	2012	5	22	6190	80.78	23.14
51559905	2012	5	22	6200	81.66	23.14
51559905	2012	5	22	6210	82.12	23.14
51559905	2012	5	22	6220	82.78	23.17
51559905	2012	5	22	6230	82.5	23.18
51559905	2012	5	22	6240	82.17	23.19
51559905	2012	5	22	6250	82.25	23.2
51559905	2012	5	22	6260	82.36	23.21
51559905	2012	5	22	6270	82.47	23.21
51559905	2012	5	22	6280	82.69	23.21
51559905	2012	5	22	6290	82.94	23.21
51559905	2012	5	22	6300	83.44	23.22
51559905	2012	5	22	6310	83.76	23.22
51559905	2012	5	22	6320	83.95	23.22
51559905	2012	5	22	6330	83.94	23.21
51559905	2012	5	22	6340	83.79	23.2
51559905	2012	5	22	6350	83.45	23.21
51559905	2012	5	22	6360	83.44	23.2
51559905	2012	5	22	6370	83.23	23.19
51559905	2012	5	22	6380	82.97	23.17
51559905	2012	5	22	6390	83.07	23.17
51559905	2012	5	22	6400	83.05	23.17
51559905	2012	5	22	6410	83.13	23.15
51559905	2012	5	22	6420	82.85	23.15
51559905	2012	5	22	6430	82.68	23.14
51559905	2012	5	22	6440	82.61	23.13
51559905	2012	5	22	6450	82.64	23.13
51559905	2012	5	22	6460	82.8	23.13
51559905	2012	5	22	6470	82.75	23.12
51559905	2012	5	22	6480	82.57	23.14
51559905	2012	5	22	6490	82.32	23.14
51559905	2012	5	22	6500	82.34	23.14
51559905	2012	5	22	6510	82.45	23.14
51559905	2012	5	22	6520	82.34	23.14
51559905	2012	5	22	6530	81.91	23.15
51559905	2012	5	22	6540	81.83	23.15
51559905	2012	5	22	6550	82.12	23.15
51559905	2012	5	22	6560	82.42	23.15
51559905	2012	5	22	6570	82.7	23.16
51559905	2012	5	22	6580	82.53	23.17
51559905	2012	5	22	6590	82.16	23.18
51559905	2012	5	22	6600	82.53	23.18

51559905	2012	5	22	6610	82.37	23.19
51559905	2012	5	22	6620	82.25	23.2
51559905	2012	5	22	6630	82.46	23.19
51559905	2012	5	22	6640	82.71	23.2
51559905	2012	5	22	6650	82.74	23.2
51559905	2012	5	22	6660	82.58	23.19
51559905	2012	5	22	6670	82.85	23.2
51559905	2012	5	22	6680	82.24	23.19
51559905	2012	5	22	6690	81.63	23.18
51559905	2012	5	22	6700	80.88	23.16
51559905	2012	5	22	6710	80.1	23.14
51559905	2012	5	22	6720	79.53	23.14
51559905	2012	5	22	6730	79.22	23.12
51559905	2012	5	22	6740	78.7	23.12
51559905	2012	5	22	6750	77.64	23.12
51559905	2012	5	22	6760	76.38	23.14
51559905	2012	5	22	6770	75.87	23.14
51559905	2012	5	22	6780	75.8	23.18
51559905	2012	5	22	6790	75.25	23.21
51559905	2012	5	22	6800	74.21	23.23
51559905	2012	5	22	6810	73.83	23.27
51559905	2012	5	22	6820	73.43	23.28
51559905	2012	5	22	6830	72.63	23.31
51559905	2012	5	22	6840	72.9	23.34
51559905	2012	5	22	6850	72.91	23.35
51559905	2012	5	22	6860	72.98	23.37
51559905	2012	5	22	6870	72.91	23.39
51559905	2012	5	22	6880	73.07	23.44
51559905	2012	5	22	6890	73	23.47
51559905	2012	5	22	6900	73.08	23.59
51559905	2012	5	22	6910	73.48	23.69
51559905	2012	5	22	6920	73.87	23.73
51559905	2012	5	22	6930	74.17	23.77
51559905	2012	5	22	6940	74.83	23.88
51559905	2012	5	22	6950	75.18	23.89
51559905	2012	5	22	6960	75.48	23.92
51559905	2012	5	22	6970	75.96	23.92
51559905	2012	5	22	6980	76.92	23.92
51559905	2012	5	22	6990	77.12	23.92
51559905	2012	5	22	7000	77.21	23.91
51559905	2012	5	22	7010	78	23.89
51559905	2012	5	22	7020	78.88	23.88
51559905	2012	5	22	7030	79.94	23.85
51559905	2012	5	22	7040	80.68	23.83
51559905	2012	5	22	7050	81.11	23.83
51559905	2012	5	22	7060	81.48	23.8
51559905	2012	5	22	7070	81.99	23.79

51559905	2012	5	22	7080	81.85	23.78
51559905	2012	5	22	7090	82.4	23.75
51559905	2012	5	22	7100	82.07	23.73
51559905	2012	5	22	7110	81.82	23.71
51559905	2012	5	22	7120	81.46	23.69
51559905	2012	5	22	7130	81.11	23.69
51559905	2012	5	22	7140	80.7	23.68
51559905	2012	5	22	7150	80.19	23.67
51559905	2012	5	22	7160	80.2	23.67
51559905	2012	5	22	7170	79.77	23.66
51559905	2012	5	22	7180	78.43	23.67
51559905	2012	5	22	7190	77.68	23.67
51559905	2012	5	22	7200	77.64	23.67
51559905	2012	5	22	7210	77.71	23.66
51559905	2012	5	22	7220	76.99	23.67
51559905	2012	5	22	7230	76.69	23.67
51559905	2012	5	22	7240	77.06	23.67
51559905	2012	5	22	7250	77.25	23.67
51559905	2012	5	22	7260	77.11	23.67
51559905	2012	5	22	7270	76.87	23.67
51559905	2012	5	22	7280	76.65	23.67
51559905	2012	5	22	7290	76.11	23.67
51559905	2012	5	22	7300	75.22	23.67
51559905	2012	5	22	7310	74.24	23.69
51559905	2012	5	22	7320	74.15	23.69
51559905	2012	5	22	7330	72.84	23.7
51559905	2012	5	22	7340	72.09	23.71
51559905	2012	5	22	7350	71.18	23.72
51559905	2012	5	22	7360	69.96	23.76
51559905	2012	5	22	7370	69.7	23.88
51559905	2012	5	22	7380	70.24	23.98
51559905	2012	5	22	7390	71.01	24.08
51559905	2012	5	22	7400	71.54	24.15
51559905	2012	5	22	7410	71.93	24.25
51559905	2012	5	22	7420	71.93	24.33
51559905	2012	5	22	7430	71.43	24.46
51559905	2012	5	22	7440	70.87	24.54
51559905	2012	5	22	7450	70.01	24.62
51559905	2012	5	22	7460	70.14	24.64
51559905	2012	5	22	7470	70.21	24.67
51559905	2012	5	22	7480	69.29	24.77
51559905	2012	5	22	7490	68.31	24.85
51559905	2012	5	22	7500	68.75	24.94
51559905	2012	5	22	7510	68.93	24.97
51559905	2012	5	22	7520	69.46	24.99
51559905	2012	5	22	7530	70.06	25.02
51559905	2012	5	22	7540	70.43	25.03

51559905	2012	5	22	7550	70.31	25.07
51559905	2012	5	22	7560	70.29	25.1
51559905	2012	5	22	7570	70.21	25.13
51559905	2012	5	22	7580	70.17	25.15
51559905	2012	5	22	7590	70.19	25.18
51559905	2012	5	22	7600	70.14	25.21
51559905	2012	5	22	7610	70.28	25.24
51559905	2012	5	22	7620	70.48	25.26
51559905	2012	5	22	7630	70.45	25.29
51559905	2012	5	22	7640	70.22	25.32
51559905	2012	5	22	7650	70.07	25.34
51559905	2012	5	22	7660	70.09	25.35
51559905	2012	5	22	7670	69.87	25.37
51559905	2012	5	22	7680	69.5	25.39
51559905	2012	5	22	7690	69.28	25.41
51559905	2012	5	22	7700	69.27	25.43
51559905	2012	5	22	7710	69.41	25.46
51559905	2012	5	22	7720	69.21	25.48
51559905	2012	5	22	7730	69.12	25.5
51559905	2012	5	22	7740	68.8	25.52
51559905	2012	5	22	7750	68.17	25.54
51559905	2012	5	22	7760	67.07	25.57
51559905	2012	5	22	7770	66.62	25.59
51559905	2012	5	22	7780	66.07	25.63
51559905	2012	5	22	7790	65.94	25.66
51559905	2012	5	22	7800	66.18	25.68
51559905	2012	5	22	7810	65	25.7
51559905	2012	5	22	7820	64.54	25.71
51559905	2012	5	22	7830	64.53	25.72
51559905	2012	5	22	7840	63.21	25.78
51559905	2012	5	22	7850	62	25.85
51559905	2012	5	22	7860	60.63	25.92
51559905	2012	5	22	7870	59.33	25.98
51559905	2012	5	22	7880	58.18	26.02
51559905	2012	5	22	7890	56.37	26.08
51559905	2012	5	22	7900	55.12	26.2
51559905	2012	5	22	7910	54.14	26.25
51559905	2012	5	22	7920	53	26.28
51559905	2012	5	22	7930	51.17	26.34
51559905	2012	5	22	7940	50.14	26.4
51559905	2012	5	22	7950	48.85	26.46
51559905	2012	5	22	7960	47.51	26.51
51559905	2012	5	22	7970	45.98	26.55
51559905	2012	5	22	7980	44.57	26.61
51559905	2012	5	22	7990	42.94	26.66
51559905	2012	5	22	8000	41.21	26.69
51559905	2012	5	22	8010	39.62	26.75

51559905	2012	5	22	8020	38.09	26.78
51559905	2012	5	22	8030	36.82	26.81
51559905	2012	5	22	8040	35.03	26.85
51559905	2012	5	22	8050	33.31	26.88
51559905	2012	5	22	8060	31.68	26.92
51559905	2012	5	22	8070	29.89	26.95
51559905	2012	5	22	8080	28.43	26.98
51559905	2012	5	22	8090	26.54	27.02
51559905	2012	5	22	8100	24.48	27.05
51559905	2012	5	22	8110	22.88	27.08
51559905	2012	5	22	8120	21.08	27.11
51559905	2012	5	22	8130	19.03	27.14
51559905	2012	5	22	8140	17.15	27.17
51559905	2012	5	22	8150	16.04	27.19
51559905	2012	5	22	8160	15.19	27.21
51559905	2012	5	22	8170	13.7	27.25
51559905	2012	5	22	8180	12.23	27.28
51559905	2012	5	22	8190	10.31	27.29
51559905	2012	5	22	8200	8.21	27.32
51559905	2012	5	22	8210	6.09	27.36
51559905	2012	5	22	8220	4.42	27.39
51559905	2012	5	22	8230	3.15	27.43
51559905	2012	5	22	8240	3.05	27.46
51559905	2012	5	22	8250	2.38	27.47
51559905	2012	5	22	8260	2.44	27.5
51559905	2012	5	22	8270	2.25	27.5
51559905	2012	5	22	8280	1.72	27.5
51559905	2012	5	22	8290	0.74	27.53
51559905	2012	5	22	8300	0.64	27.52
51559905	2012	5	22	8310	0.67	27.54
51559905	2012	5	22	8320	0.67	27.57
51559905	2012	5	22	8330	0.63	27.57
51559905	2012	5	22	8340	0.61	27.61
51559905	2012	5	22	8350	0.63	27.63
51559905	2012	5	22	8360	0.62	27.63
51559905	2012	5	22	8370	0.7	27.66
51559905	2012	5	22	8380	0.59	27.66
51559905	2012	5	22	8390	0.56	27.68
51559905	2012	5	22	8400	0.56	27.68
51559905	2012	5	22	8410	0.57	27.69
51559905	2012	5	22	8420	0.59	27.69
51559905	2012	5	22	8430	0.58	27.71
51574415	2012	5	22	0	1.12	28.05
51574415	2012	5	22	10	1.47	28.04
51574415	2012	5	22	20	2.58	28.03
51574415	2012	5	22	30	2.66	28.03
51574415	2012	5	22	40	2.58	28.03

51574415	2012	5	22	50	2.51	28.03
51574415	2012	5	22	60	2.24	28.03
51574415	2012	5	22	70	2.46	28.01
51574415	2012	5	22	80	2.7	27.98
51574415	2012	5	22	90	3.55	27.95
51574415	2012	5	22	100	4.87	27.93
51574415	2012	5	22	110	6.02	27.9
51574415	2012	5	22	120	6.51	27.89
51574415	2012	5	22	130	7.7	27.86
51574415	2012	5	22	140	9.32	27.82
51574415	2012	5	22	150	11.17	27.81
51574415	2012	5	22	160	12.7	27.78
51574415	2012	5	22	170	14.79	27.76
51574415	2012	5	22	180	16.65	27.74
51574415	2012	5	22	190	18.7	27.71
51574415	2012	5	22	200	20.83	27.69
51574415	2012	5	22	210	22.63	27.67
51574415	2012	5	22	220	24.36	27.64
51574415	2012	5	22	230	26.32	27.64
51574415	2012	5	22	240	28.48	27.61
51574415	2012	5	22	250	30.62	27.6
51574415	2012	5	22	260	32.12	27.57
51574415	2012	5	22	270	33.45	27.57
51574415	2012	5	22	280	34.89	27.56
51574415	2012	5	22	290	35.87	27.54
51574415	2012	5	22	300	36.86	27.53
51574415	2012	5	22	310	38.48	27.51
51574415	2012	5	22	320	39.98	27.5
51574415	2012	5	22	330	41.27	27.49
51574415	2012	5	22	340	42.8	27.47
51574415	2012	5	22	350	44.1	27.46
51574415	2012	5	22	360	45.16	27.45
51574415	2012	5	22	370	46.89	27.44
51574415	2012	5	22	380	49.3	27.42
51574415	2012	5	22	390	51.92	27.4
51574415	2012	5	22	400	54.08	27.38
51574415	2012	5	22	410	56.06	27.37
51574415	2012	5	22	420	57.97	27.35
51574415	2012	5	22	430	59.92	27.33
51574415	2012	5	22	440	61.1	27.32
51574415	2012	5	22	450	62.27	27.3
51574415	2012	5	22	460	63.85	27.28
51574415	2012	5	22	470	65.81	27.28
51574415	2012	5	22	480	67.9	27.26
51574415	2012	5	22	490	69.06	27.23
51574415	2012	5	22	500	69.6	27.1
51574415	2012	5	22	510	69.73	26.97

51574415	2012	5	22	520	69.84	26.89
51574415	2012	5	22	530	69.86	26.78
51574415	2012	5	22	540	69.95	26.68
51574415	2012	5	22	550	70	26.65
51574415	2012	5	22	560	69.92	26.55
51574415	2012	5	22	570	69.61	26.42
51574415	2012	5	22	580	70.4	26.14
51574415	2012	5	22	590	71.42	26.06
51574415	2012	5	22	600	73.56	26.03
51574415	2012	5	22	610	75.13	25.94
51574415	2012	5	22	620	76.94	25.65
51574415	2012	5	22	630	79.19	25.63
51574415	2012	5	22	640	81.51	25.57
51574415	2012	5	22	650	84.13	25.44
51574415	2012	5	22	660	86.21	25.3
51574415	2012	5	22	670	88.03	25.16
51574415	2012	5	22	680	90.25	25.04
51574415	2012	5	22	690	92.91	24.92
51574415	2012	5	22	700	95.72	24.8
51574415	2012	5	22	710	98.46	24.66
51574415	2012	5	22	720	101.06	24.5
51574415	2012	5	22	730	103.47	24.4
51574415	2012	5	22	740	106.05	24.29
51574415	2012	5	22	750	108.92	24.18
51574415	2012	5	22	760	111.96	24.06
51574415	2012	5	22	770	114.5	23.98
51574415	2012	5	22	780	116.64	23.91
51574415	2012	5	22	790	119.24	23.82
51574415	2012	5	22	800	121.4	23.75
51574415	2012	5	22	810	123.57	23.66
51574415	2012	5	22	820	125.69	23.5
51574415	2012	5	22	830	127.4	23.42
51574415	2012	5	22	840	129.49	23.3
51574415	2012	5	22	850	131.4	23.14
51574415	2012	5	22	860	132.35	22.63
51574415	2012	5	22	870	133.93	22.34
51574415	2012	5	22	880	135.24	22.35
51574415	2012	5	22	890	137.14	22.24
51574415	2012	5	22	900	137.96	22.14
51574415	2012	5	22	910	137.93	22.01
51574415	2012	5	22	920	138.1	21.87
51574415	2012	5	22	930	138.39	21.71
51574415	2012	5	22	940	138.75	21.6
51574415	2012	5	22	950	139.04	21.52
51574415	2012	5	22	960	138.71	21.43
51574415	2012	5	22	970	138.28	21.34
51574415	2012	5	22	980	137.93	21.23

51574415	2012	5	22	990	138.63	21.18
51574415	2012	5	22	1000	139.8	21.08
51574415	2012	5	22	1010	140.3	21.02
51574415	2012	5	22	1020	141.07	20.85
51574415	2012	5	22	1030	142.58	20.66
51574415	2012	5	22	1040	144.09	20.66
51574415	2012	5	22	1050	146.52	20.65
51574415	2012	5	22	1060	149.13	20.54
51574415	2012	5	22	1070	152.15	20.52
51574415	2012	5	22	1080	154.97	20.48
51574415	2012	5	22	1090	156.2	20.41
51574415	2012	5	22	1100	157.57	20.19
51574415	2012	5	22	1110	159.03	20.02
51574415	2012	5	22	1120	159.37	19.99
51574415	2012	5	22	1130	159.52	19.94
51574415	2012	5	22	1140	159.64	19.82
51574415	2012	5	22	1150	160.16	19.78
51574415	2012	5	22	1160	160.32	19.75
51574415	2012	5	22	1170	160.11	19.64
51574415	2012	5	22	1180	159.49	19.55
51574415	2012	5	22	1190	160.08	19.46
51574415	2012	5	22	1200	161.25	19.32
51574415	2012	5	22	1210	162.34	19.22
51574415	2012	5	22	1220	163.94	19.17
51574415	2012	5	22	1230	165.95	19.19
51574415	2012	5	22	1240	167.03	19.15
51574415	2012	5	22	1250	167.8	19.12
51574415	2012	5	22	1260	168.75	19.07
51574415	2012	5	22	1270	169.85	19.06
51574415	2012	5	22	1280	170.87	18.96
51574415	2012	5	22	1290	171.88	18.89
51574415	2012	5	22	1300	172.93	18.79
51574415	2012	5	22	1310	174.38	18.73
51574415	2012	5	22	1320	175.44	18.73
51574415	2012	5	22	1330	176.18	18.64
51574415	2012	5	22	1340	177.37	18.58
51574415	2012	5	22	1350	177.67	18.55
51574415	2012	5	22	1360	178.07	18.51
51574415	2012	5	22	1370	178.52	18.49
51574415	2012	5	22	1380	178.76	18.45
51574415	2012	5	22	1390	179.07	18.39
51574415	2012	5	22	1400	179.4	18.37
51574415	2012	5	22	1410	179.35	18.35
51574415	2012	5	22	1420	179.76	18.32
51574415	2012	5	22	1430	180.02	18.28
51574415	2012	5	22	1440	179.98	18.25
51574415	2012	5	22	1450	179.92	18.23

51574415	2012	5	22	1460	180.27	18.22
51574415	2012	5	22	1470	179.78	18.2
51574415	2012	5	22	1480	179.24	18.17
51574415	2012	5	22	1490	179.62	18.12
51574415	2012	5	22	1500	180.07	18.1
51574415	2012	5	22	1510	179.82	18.07
51574415	2012	5	22	1520	179.98	18.05
51574415	2012	5	22	1530	179.27	18.03
51574415	2012	5	22	1540	179.15	18.01
51574415	2012	5	22	1550	179.22	17.93
51574415	2012	5	22	1560	179.32	17.89
51574415	2012	5	22	1570	180.25	17.88
51574415	2012	5	22	1580	180.76	17.87
51574415	2012	5	22	1590	181.32	17.87
51574415	2012	5	22	1600	181.83	17.83
51574415	2012	5	22	1610	182.38	17.81
51574415	2012	5	22	1620	183.41	17.79
51574415	2012	5	22	1630	183.57	17.78
51574415	2012	5	22	1640	184.6	17.76
51574415	2012	5	22	1650	185.67	17.73
51574415	2012	5	22	1660	186.87	17.71
51574415	2012	5	22	1670	188.95	17.71
51574415	2012	5	22	1680	190.67	17.69
51574415	2012	5	22	1690	191.76	17.59
51574415	2012	5	22	1700	192.88	17.55
51574415	2012	5	22	1710	193.97	17.41
51574415	2012	5	22	1720	194.55	17.35
51574415	2012	5	22	1730	195.16	17.35
51574415	2012	5	22	1740	196.02	17.31
51574415	2012	5	22	1750	196.79	17.15
51574415	2012	5	22	1760	197.88	17.08
51574415	2012	5	22	1770	199.18	17.1
51574415	2012	5	22	1780	199.94	17.09
51574415	2012	5	22	1790	199.96	17
51574415	2012	5	22	1800	200.22	16.88
51574415	2012	5	22	1810	200.43	16.6
51574415	2012	5	22	1820	200.35	16.56
51574415	2012	5	22	1830	199.98	16.53
51574415	2012	5	22	1840	200.51	16.49
51574415	2012	5	22	1850	200.6	16.39
51574415	2012	5	22	1860	201.39	16.28
51574415	2012	5	22	1870	201.75	16.25
51574415	2012	5	22	1880	201.57	16.22
51574415	2012	5	22	1890	201.57	16.03
51574415	2012	5	22	1900	201.58	15.95
51574415	2012	5	22	1910	202.04	15.88
51574415	2012	5	22	1920	202.77	15.84

51574415	2012	5	22	1930	204.05	15.77
51574415	2012	5	22	1940	203.97	15.78
51574415	2012	5	22	1950	203.5	15.75
51574415	2012	5	22	1960	203.58	15.67
51574415	2012	5	22	1970	203.39	15.67
51574415	2012	5	22	1980	203.36	15.66
51574415	2012	5	22	1990	202.93	15.64
51574415	2012	5	22	2000	202.61	15.57
51574415	2012	5	22	2010	202.52	15.54
51574415	2012	5	22	2020	203.2	15.48
51574415	2012	5	22	2030	203.84	15.44
51574415	2012	5	22	2040	205.31	15.45
51574415	2012	5	22	2050	206.59	15.46
51574415	2012	5	22	2060	207.26	15.45
51574415	2012	5	22	2070	207.8	15.39
51574415	2012	5	22	2080	208.29	15.34
51574415	2012	5	22	2090	209.26	15.34
51574415	2012	5	22	2100	210.15	15.32
51574415	2012	5	22	2110	210.4	15.31
51574415	2012	5	22	2120	211.25	15.3
51574415	2012	5	22	2130	212.27	15.29
51574415	2012	5	22	2140	212.86	15.28
51574415	2012	5	22	2150	212.44	15.24
51574415	2012	5	22	2160	213.17	15.21
51574415	2012	5	22	2170	213.96	15.19
51574415	2012	5	22	2180	214.67	15.17
51574415	2012	5	22	2190	215.46	15.14
51574415	2012	5	22	2200	216.11	15.14
51574415	2012	5	22	2210	217.44	15.14
51574415	2012	5	22	2220	218.15	15.14
51574415	2012	5	22	2230	218.67	15.14
51574415	2012	5	22	2240	219.43	15.1
51574415	2012	5	22	2250	220.06	15.04
51574415	2012	5	22	2260	221.3	15
51574415	2012	5	22	2270	222.06	15
51574415	2012	5	22	2280	222.39	15
51574415	2012	5	22	2290	222.54	15
51574415	2012	5	22	2300	222.01	14.96
51574415	2012	5	22	2310	221.6	14.94
51574415	2012	5	22	2320	222.74	14.92
51574415	2012	5	22	2330	223.64	14.92
51574415	2012	5	22	2340	224.65	14.85
51574415	2012	5	22	2350	225.3	14.85
51574415	2012	5	22	2360	226.11	14.83
51574415	2012	5	22	2370	225.97	14.8
51574415	2012	5	22	2380	227.01	14.73
51574415	2012	5	22	2390	228.42	14.73

51574415	2012	5	22	2400	228.87	14.71
51574415	2012	5	22	2410	229.41	14.71
51574415	2012	5	22	2420	230	14.7
51574415	2012	5	22	2430	230.13	14.69
51574415	2012	5	22	2440	229.98	14.66
51574415	2012	5	22	2450	229.56	14.62
51574415	2012	5	22	2460	229.78	14.6
51574415	2012	5	22	2470	229.99	14.57
51574415	2012	5	22	2480	230.07	14.54
51574415	2012	5	22	2490	229.85	14.51
51574415	2012	5	22	2500	229.46	14.5
51574415	2012	5	22	2510	229.5	14.5
51574415	2012	5	22	2520	229.74	14.47
51574415	2012	5	22	2530	229.36	14.46
51574415	2012	5	22	2540	229.05	14.45
51574415	2012	5	22	2550	229.39	14.42
51574415	2012	5	22	2560	229.44	14.42
51574415	2012	5	22	2570	229.59	14.41
51574415	2012	5	22	2580	227.96	14.39
51574415	2012	5	22	2590	227.06	14.36
51574415	2012	5	22	2600	226.79	14.35
51574415	2012	5	22	2610	226.73	14.34
51574415	2012	5	22	2620	226.33	14.33
51574415	2012	5	22	2630	225.5	14.33
51574415	2012	5	22	2640	224.96	14.32
51574415	2012	5	22	2650	225.12	14.32
51574415	2012	5	22	2660	225.29	14.33
51574415	2012	5	22	2670	224.79	14.33
51574415	2012	5	22	2680	223.83	14.33
51574415	2012	5	22	2690	223.71	14.33
51574415	2012	5	22	2700	224.24	14.35
51574415	2012	5	22	2710	223.99	14.35
51574415	2012	5	22	2720	223.71	14.36
51574415	2012	5	22	2730	223.73	14.36
51574415	2012	5	22	2740	224.11	14.37
51574415	2012	5	22	2750	223.99	14.38
51574415	2012	5	22	2760	224.12	14.38
51574415	2012	5	22	2770	224.72	14.38
51574415	2012	5	22	2780	224.89	14.39
51574415	2012	5	22	2790	224.42	14.39
51574415	2012	5	22	2800	224.1	14.4
51574415	2012	5	22	2810	224.03	14.41
51574415	2012	5	22	2820	223.75	14.41
51574415	2012	5	22	2830	223.85	14.42
51574415	2012	5	22	2840	223.31	14.42
51574415	2012	5	22	2850	223.34	14.42
51574415	2012	5	22	2860	223.79	14.42

51574415	2012	5	22	2870	224.44	14.42
51574415	2012	5	22	2880	224.94	14.41
51574415	2012	5	22	2890	225.9	14.4
51574415	2012	5	22	2900	227.21	14.4
51574415	2012	5	22	2910	228.2	14.39
51574415	2012	5	22	2920	228.45	14.39
51574415	2012	5	22	2930	229.38	14.39
51574415	2012	5	22	2940	229.58	14.35
51574415	2012	5	22	2950	230.18	14.31
51574415	2012	5	22	2960	231.57	14.28
51574415	2012	5	22	2970	231.9	14.27
51574415	2012	5	22	2980	232.62	14.2
51574415	2012	5	22	2990	233.48	14.15
51574415	2012	5	22	3000	234.25	14.12
51574415	2012	5	22	3010	234.55	14.12
51574415	2012	5	22	3020	235.31	14.1
51574415	2012	5	22	3030	235.76	14.03
51574415	2012	5	22	3040	236.16	13.97
51574415	2012	5	22	3050	236.91	13.92
51574415	2012	5	22	3060	237.43	13.91
51574415	2012	5	22	3070	238.45	13.91
51574415	2012	5	22	3080	239.28	13.86
51574415	2012	5	22	3090	239.86	13.79
51574415	2012	5	22	3100	240.64	13.78
51574415	2012	5	22	3110	241.5	13.78
51574415	2012	5	22	3120	241.63	13.75
51574415	2012	5	22	3130	242.2	13.71
51574415	2012	5	22	3140	242.62	13.71
51574415	2012	5	22	3150	243	13.71
51574415	2012	5	22	3160	243.37	13.71
51574415	2012	5	22	3170	243.11	13.69
51574415	2012	5	22	3180	243.09	13.67
51574415	2012	5	22	3190	242.51	13.64
51574415	2012	5	22	3200	242.47	13.63
51574415	2012	5	22	3210	242.83	13.63
51574415	2012	5	22	3220	242.54	13.61
51574415	2012	5	22	3230	242.34	13.61
51574415	2012	5	22	3240	242.36	13.6
51574415	2012	5	22	3250	241.7	13.58
51574415	2012	5	22	3260	241.94	13.57
51574415	2012	5	22	3270	241.85	13.56
51574415	2012	5	22	3280	241.78	13.55
51574415	2012	5	22	3290	241.66	13.54
51574415	2012	5	22	3300	241.66	13.53
51574415	2012	5	22	3310	241.55	13.52
51574415	2012	5	22	3320	241.46	13.51
51574415	2012	5	22	3330	241.57	13.5

51574415	2012	5	22	3340	241.85	13.49
51574415	2012	5	22	3350	241.93	13.49
51574415	2012	5	22	3360	242.13	13.46
51574415	2012	5	22	3370	242.83	13.45
51574415	2012	5	22	3380	243.08	13.45
51574415	2012	5	22	3390	243.26	13.46
51574415	2012	5	22	3400	243.05	13.45
51574415	2012	5	22	3410	242.91	13.44
51574415	2012	5	22	3420	242.8	13.43
51574415	2012	5	22	3430	242.65	13.42
51574415	2012	5	22	3440	243.1	13.43
51574415	2012	5	22	3450	243.53	13.43
51574415	2012	5	22	3460	243.49	13.42
51574415	2012	5	22	3470	243.77	13.42
51574415	2012	5	22	3480	243.43	13.42
51574415	2012	5	22	3490	243.69	13.42
51574415	2012	5	22	3500	244.16	13.42
51574415	2012	5	22	3510	244.02	13.42
51574415	2012	5	22	3520	244.4	13.41
51574415	2012	5	22	3530	244.62	13.42
51574415	2012	5	22	3540	245.2	13.42
51574415	2012	5	22	3550	246.3	13.42
51574415	2012	5	22	3560	246.81	13.42
51574415	2012	5	22	3570	247.56	13.42
51574415	2012	5	22	3580	247.66	13.42
51574415	2012	5	22	3590	248.56	13.42
51574415	2012	5	22	3600	248.86	13.42
51574415	2012	5	22	3610	249.18	13.42
51574415	2012	5	22	3620	249	13.42
51574415	2012	5	22	3630	249.33	13.42
51574415	2012	5	22	3640	249.65	13.41
51574415	2012	5	22	3650	250.51	13.41
51574415	2012	5	22	3660	250.41	13.41
51574415	2012	5	22	3670	250.58	13.41
51574415	2012	5	22	3680	251.09	13.4
51574415	2012	5	22	3690	251.34	13.4
51574415	2012	5	22	3700	251.57	13.4
51574415	2012	5	22	3710	251.19	13.4
51574415	2012	5	22	3720	251.36	13.39
51574415	2012	5	22	3730	250.85	13.39
51574415	2012	5	22	3740	250.69	13.38
51574415	2012	5	22	3750	250.66	13.38
51574415	2012	5	22	3760	251.59	13.38
51574415	2012	5	22	3770	251.55	13.38
51574415	2012	5	22	3780	251.27	13.37
51574415	2012	5	22	3790	251.36	13.35
51574415	2012	5	22	3800	251.53	13.37

51574415	2012	5	22	3810	250.53	13.35
51574415	2012	5	22	3820	249.86	13.36
51574415	2012	5	22	3830	250.19	13.35
51574415	2012	5	22	3840	250.35	13.35
51574415	2012	5	22	3850	250.64	13.35
51574415	2012	5	22	3860	250.99	13.35
51574415	2012	5	22	3870	250.95	13.35
51574415	2012	5	22	3880	250.77	13.35
51574415	2012	5	22	3890	251.21	13.34
51574415	2012	5	22	3900	251.14	13.33
51574415	2012	5	22	3910	250.88	13.34
51574415	2012	5	22	3920	250.51	13.34
51574415	2012	5	22	3930	250.49	13.33
51574415	2012	5	22	3940	250.35	13.34
51574415	2012	5	22	3950	250.16	13.33
51574415	2012	5	22	3960	249.89	13.32
51574415	2012	5	22	3970	250.75	13.32
51574415	2012	5	22	3980	251.25	13.32
51574415	2012	5	22	3990	251.15	13.32
51574415	2012	5	22	4000	249.91	13.32
51574415	2012	5	22	4010	249.29	13.32
51574415	2012	5	22	4020	250.06	13.32
51574415	2012	5	22	4030	250.41	13.32
51574415	2012	5	22	4040	250.75	13.32
51574415	2012	5	22	4050	250.99	13.32
51574415	2012	5	22	4060	251.13	13.32
51574415	2012	5	22	4070	251.54	13.32
51574415	2012	5	22	4080	251.83	13.31
51574415	2012	5	22	4090	252.14	13.32
51574415	2012	5	22	4100	252.48	13.31
51574415	2012	5	22	4110	252.93	13.31
51574415	2012	5	22	4120	253.4	13.3
51574415	2012	5	22	4130	253.64	13.3
51574415	2012	5	22	4140	253.77	13.31
51574415	2012	5	22	4150	253.51	13.31
51574415	2012	5	22	4160	252.9	13.3
51574415	2012	5	22	4170	252.14	13.3
51574415	2012	5	22	4180	252.02	13.29
51574415	2012	5	22	4190	251.63	13.29
51574415	2012	5	22	4200	252.08	13.29
51574415	2012	5	22	4210	252.15	13.28
51574415	2012	5	22	4220	251.58	13.28
51574415	2012	5	22	4230	251.27	13.28
51574415	2012	5	22	4240	250.64	13.28
51574415	2012	5	22	4250	251.19	13.28
51574415	2012	5	22	4260	251.47	13.28
51574415	2012	5	22	4270	251.19	13.28

51574415	2012	5	22	4280	251.32	13.28
51574415	2012	5	22	4290	251.45	13.28
51574415	2012	5	22	4300	252.16	13.28
51574415	2012	5	22	4310	252.33	13.28
51574415	2012	5	22	4320	252.32	13.28
51574415	2012	5	22	4330	252.48	13.28
51574415	2012	5	22	4340	252.36	13.28
51574415	2012	5	22	4350	252.37	13.27
51574415	2012	5	22	4360	252.64	13.28
51574415	2012	5	22	4370	251.99	13.28
51574415	2012	5	22	4380	251.23	13.27
51574415	2012	5	22	4390	250.82	13.27
51574415	2012	5	22	4400	250.31	13.26
51574415	2012	5	22	4410	250.44	13.27
51574415	2012	5	22	4420	251.54	13.27
51574415	2012	5	22	4430	252.55	13.26
51574415	2012	5	22	4440	252.92	13.26
51574415	2012	5	22	4450	252.45	13.25
51574415	2012	5	22	4460	251.79	13.24
51574415	2012	5	22	4470	252.01	13.25
51574415	2012	5	22	4480	251.98	13.23
51574415	2012	5	22	4490	252.06	13.22
51574415	2012	5	22	4500	251.81	13.21
51574415	2012	5	22	4510	252.07	13.22
51574415	2012	5	22	4520	252.25	13.22
51574415	2012	5	22	4530	252.24	13.22
51574415	2012	5	22	4540	252.27	13.21
51574415	2012	5	22	4550	252.19	13.21
51574415	2012	5	22	4560	252.28	13.21
51574415	2012	5	22	4570	251.86	13.21
51574415	2012	5	22	4580	250.48	13.2
51574415	2012	5	22	4590	250.03	13.2
51574415	2012	5	22	4600	249.33	13.2
51574415	2012	5	22	4610	249.11	13.19
51574415	2012	5	22	4620	249.98	13.2
51574415	2012	5	22	4630	250.16	13.2
51574415	2012	5	22	4640	250.17	13.21
51574415	2012	5	22	4650	250.15	13.21
51574415	2012	5	22	4660	249.98	13.21
51574415	2012	5	22	4670	250.41	13.21
51574415	2012	5	22	4680	250.51	13.21
51574415	2012	5	22	4690	250.04	13.21
51574415	2012	5	22	4700	249.88	13.21
51574415	2012	5	22	4710	250.56	13.22
51574415	2012	5	22	4720	250.54	13.22
51574415	2012	5	22	4730	250.25	13.21
51574415	2012	5	22	4740	250.73	13.23

51574415	2012	5	22	4750	250.4	13.22
51574415	2012	5	22	4760	250.14	13.21
51574415	2012	5	22	4770	250.22	13.22
51574415	2012	5	22	4780	250.31	13.23
51574415	2012	5	22	4790	250.55	13.22
51574415	2012	5	22	4800	250.53	13.22
51574415	2012	5	22	4810	251.28	13.22
51574415	2012	5	22	4820	251.22	13.22
51574415	2012	5	22	4830	250.83	13.21
51574415	2012	5	22	4840	250.64	13.22
51574415	2012	5	22	4850	250.44	13.23
51574415	2012	5	22	4860	250.97	13.23
51574415	2012	5	22	4870	251.49	13.22
51574415	2012	5	22	4880	251.77	13.22
51574415	2012	5	22	4890	250.85	13.22
51574415	2012	5	22	4900	251.22	13.22
51574415	2012	5	22	4910	251.31	13.22
51574415	2012	5	22	4920	251.18	13.23
51574415	2012	5	22	4930	250.2	13.23
51574415	2012	5	22	4940	249.62	13.22
51574415	2012	5	22	4950	249.86	13.23
51574415	2012	5	22	4960	249.92	13.23
51574415	2012	5	22	4970	250.23	13.23
51574415	2012	5	22	4980	250.17	13.23
51574415	2012	5	22	4990	249.51	13.23
51574415	2012	5	22	5000	249.92	13.23
51574415	2012	5	22	5010	250.28	13.23
51574415	2012	5	22	5020	249.98	13.23
51574415	2012	5	22	5030	250.64	13.24
51574415	2012	5	22	5040	250.83	13.24
51574415	2012	5	22	5050	250.5	13.24
51574415	2012	5	22	5060	248.43	13.23
51574415	2012	5	22	5070	246.63	13.23
51574415	2012	5	22	5080	246.02	13.24
51574415	2012	5	22	5090	244.74	13.23
51574415	2012	5	22	5100	243.88	13.23
51574415	2012	5	22	5110	244.46	13.24
51574415	2012	5	22	5120	245.12	13.24
51574415	2012	5	22	5130	245.73	13.25
51574415	2012	5	22	5140	245.3	13.25
51574415	2012	5	22	5150	244.91	13.25
51574415	2012	5	22	5160	244.71	13.25
51574415	2012	5	22	5170	245.34	13.25
51574415	2012	5	22	5180	246.06	13.25
51574415	2012	5	22	5190	245.85	13.26
51574415	2012	5	22	5200	244.96	13.25
51574415	2012	5	22	5210	245.16	13.26

51574415	2012	5	22	5220	245.26	13.26
51574415	2012	5	22	5230	244.88	13.26
51574415	2012	5	22	5240	244.79	13.26
51574415	2012	5	22	5250	244.1	13.26
51574415	2012	5	22	5260	243.32	13.26
51574415	2012	5	22	5270	243.95	13.27
51574415	2012	5	22	5280	244.18	13.28
51574415	2012	5	22	5290	244.24	13.28
51574415	2012	5	22	5300	244.13	13.28
51574415	2012	5	22	5310	243.86	13.29
51574415	2012	5	22	5320	244.18	13.3
51574415	2012	5	22	5330	244.63	13.3
51574415	2012	5	22	5340	244.58	13.31
51574415	2012	5	22	5350	244.06	13.32
51574415	2012	5	22	5360	244.19	13.32
51574415	2012	5	22	5370	244.34	13.32
51574415	2012	5	22	5380	244.74	13.32
51574415	2012	5	22	5390	243.94	13.32
51574415	2012	5	22	5400	243.46	13.32
51574415	2012	5	22	5410	244.16	13.34
51574415	2012	5	22	5420	244.13	13.34
51574415	2012	5	22	5430	244.07	13.35
51574415	2012	5	22	5440	243.61	13.35
51574415	2012	5	22	5450	242.71	13.35
51574415	2012	5	22	5460	243.05	13.35
51574415	2012	5	22	5470	244.1	13.35
51574415	2012	5	22	5480	244.27	13.36
51574415	2012	5	22	5490	243.96	13.36
51574415	2012	5	22	5500	243.61	13.37
51574415	2012	5	22	5510	243.09	13.37
51574415	2012	5	22	5520	242.97	13.38
51574415	2012	5	22	5530	242.51	13.38
51574415	2012	5	22	5540	241.87	13.38
51574415	2012	5	22	5550	241.1	13.39
51574415	2012	5	22	5560	241.07	13.39
51574415	2012	5	22	5570	240.82	13.39
51574415	2012	5	22	5580	240.47	13.39
51574415	2012	5	22	5590	239.59	13.39
51574415	2012	5	22	5600	238.8	13.39
51574415	2012	5	22	5610	237.97	13.4
51574415	2012	5	22	5620	237.64	13.4
51574415	2012	5	22	5630	237.25	13.41
51574415	2012	5	22	5640	236.55	13.42
51574415	2012	5	22	5650	236.95	13.42
51574415	2012	5	22	5660	236.24	13.42
51574415	2012	5	22	5670	235.65	13.42
51574415	2012	5	22	5680	234.88	13.42

51574415	2012	5	22	5690	234.33	13.42
51574415	2012	5	22	5700	233.37	13.42
51574415	2012	5	22	5710	232.28	13.43
51574415	2012	5	22	5720	231.37	13.42
51574415	2012	5	22	5730	230.36	13.42
51574415	2012	5	22	5740	229.76	13.44
51574415	2012	5	22	5750	229.73	13.45
51574415	2012	5	22	5760	229.58	13.44
51574415	2012	5	22	5770	228.72	13.46
51574415	2012	5	22	5780	227.73	13.45
51574415	2012	5	22	5790	226.71	13.46
51574415	2012	5	22	5800	225.39	13.46
51574415	2012	5	22	5810	224.6	13.47
51574415	2012	5	22	5820	223.83	13.48
51574415	2012	5	22	5830	223.63	13.5
51574415	2012	5	22	5840	222.88	13.53
51574415	2012	5	22	5850	221.74	13.56
51574415	2012	5	22	5860	220.67	13.6
51574415	2012	5	22	5870	219.5	13.66
51574415	2012	5	22	5880	218.2	13.73
51574415	2012	5	22	5890	216.97	13.78
51574415	2012	5	22	5900	215.96	13.88
51574415	2012	5	22	5910	214.55	13.93
51574415	2012	5	22	5920	213.16	14
51574415	2012	5	22	5930	212.33	14.03
51574415	2012	5	22	5940	211.79	14.08
51574415	2012	5	22	5950	210.61	14.11
51574415	2012	5	22	5960	210.22	14.13
51574415	2012	5	22	5970	210.26	14.16
51574415	2012	5	22	5980	210.1	14.18
51574415	2012	5	22	5990	209.49	14.22
51574415	2012	5	22	6000	208.87	14.27
51574415	2012	5	22	6010	208.6	14.32
51574415	2012	5	22	6020	208.35	14.33
51574415	2012	5	22	6030	206.96	14.35
51574415	2012	5	22	6040	206.44	14.39
51574415	2012	5	22	6050	205.18	14.41
51574415	2012	5	22	6060	204.22	14.43
51574415	2012	5	22	6070	203.5	14.45
51574415	2012	5	22	6080	202.03	14.47
51574415	2012	5	22	6090	200.83	14.53
51574415	2012	5	22	6100	199.86	14.58
51574415	2012	5	22	6110	198.68	14.62
51574415	2012	5	22	6120	198.31	14.67
51574415	2012	5	22	6130	197.58	14.71
51574415	2012	5	22	6140	196.18	14.78
51574415	2012	5	22	6150	194.54	14.81

51574415	2012	5	22	6160	194.28	14.92
51574415	2012	5	22	6170	195.22	15
51574415	2012	5	22	6180	193.98	15.07
51574415	2012	5	22	6190	192.2	15.12
51574415	2012	5	22	6200	190.77	15.17
51574415	2012	5	22	6210	189.84	15.23
51574415	2012	5	22	6220	187.79	15.28
51574415	2012	5	22	6230	186.62	15.41
51574415	2012	5	22	6240	185.84	15.46
51574415	2012	5	22	6250	184.72	15.47
51574415	2012	5	22	6260	183.63	15.52
51574415	2012	5	22	6270	182.58	15.6
51574415	2012	5	22	6280	181.68	15.64
51574415	2012	5	22	6290	180.98	15.69
51574415	2012	5	22	6300	179.66	15.72
51574415	2012	5	22	6310	179.26	15.78
51574415	2012	5	22	6320	179.41	15.84
51574415	2012	5	22	6330	178.46	15.94
51574415	2012	5	22	6340	177.77	15.99
51574415	2012	5	22	6350	177.39	16.04
51574415	2012	5	22	6360	176.64	16.1
51574415	2012	5	22	6370	176.28	16.14
51574415	2012	5	22	6380	176.04	16.17
51574415	2012	5	22	6390	175.08	16.21
51574415	2012	5	22	6400	174.04	16.26
51574415	2012	5	22	6410	172.87	16.28
51574415	2012	5	22	6420	172.34	16.32
51574415	2012	5	22	6430	171.51	16.39
51574415	2012	5	22	6440	170.97	16.44
51574415	2012	5	22	6450	170.85	16.5
51574415	2012	5	22	6460	169.95	16.54
51574415	2012	5	22	6470	169.06	16.59
51574415	2012	5	22	6480	167.99	16.61
51574415	2012	5	22	6490	166.5	16.65
51574415	2012	5	22	6500	165.27	16.71
51574415	2012	5	22	6510	163.71	16.77
51574415	2012	5	22	6520	162.74	16.82
51574415	2012	5	22	6530	161.34	16.87
51574415	2012	5	22	6540	160.34	16.92
51574415	2012	5	22	6550	159.21	16.98
51574415	2012	5	22	6560	157.69	17.03
51574415	2012	5	22	6570	156.83	17.07
51574415	2012	5	22	6580	155.58	17.13
51574415	2012	5	22	6590	155.23	17.16
51574415	2012	5	22	6600	155.42	17.19
51574415	2012	5	22	6610	156.1	17.26
51574415	2012	5	22	6620	156.26	17.28

51574415	2012	5	22	6630	155.77	17.3
51574415	2012	5	22	6640	155.22	17.32
51574415	2012	5	22	6650	155.19	17.35
51574415	2012	5	22	6660	155.13	17.36
51574415	2012	5	22	6670	155.36	17.38
51574415	2012	5	22	6680	155.26	17.42
51574415	2012	5	22	6690	155.38	17.44
51574415	2012	5	22	6700	155.47	17.45
51574415	2012	5	22	6710	155.38	17.46
51574415	2012	5	22	6720	155.52	17.46
51574415	2012	5	22	6730	155.38	17.49
51574415	2012	5	22	6740	155.54	17.49
51574415	2012	5	22	6750	155.39	17.51
51574415	2012	5	22	6760	155.32	17.52
51574415	2012	5	22	6770	154.97	17.53
51574415	2012	5	22	6780	155.67	17.57
51574415	2012	5	22	6790	155.68	17.58
51574415	2012	5	22	6800	155.66	17.59
51574415	2012	5	22	6810	155.78	17.59
51574415	2012	5	22	6820	155.83	17.6
51574415	2012	5	22	6830	155.78	17.61
51574415	2012	5	22	6840	155.66	17.61
51574415	2012	5	22	6850	155.84	17.63
51574415	2012	5	22	6860	155.77	17.64
51574415	2012	5	22	6870	155.66	17.65
51574415	2012	5	22	6880	154.85	17.66
51574415	2012	5	22	6890	153.62	17.7
51574415	2012	5	22	6900	153.34	17.71
51574415	2012	5	22	6910	153.5	17.74
51574415	2012	5	22	6920	153.34	17.75
51574415	2012	5	22	6930	153.21	17.78
51574415	2012	5	22	6940	152.77	17.82
51574415	2012	5	22	6950	152.57	17.85
51574415	2012	5	22	6960	151.85	17.86
51574415	2012	5	22	6970	151.04	17.88
51574415	2012	5	22	6980	150.7	17.89
51574415	2012	5	22	6990	150.14	17.9
51574415	2012	5	22	7000	150.19	17.92
51574415	2012	5	22	7010	149.87	17.95
51574415	2012	5	22	7020	149.85	18
51574415	2012	5	22	7030	149.31	18.04
51574415	2012	5	22	7040	148.33	18.06
51574415	2012	5	22	7050	147.38	18.08
51574415	2012	5	22	7060	147.8	18.1
51574415	2012	5	22	7070	148.03	18.14
51574415	2012	5	22	7080	147.02	18.19
51574415	2012	5	22	7090	145.88	18.22

51574415	2012	5	22	7100	145.5	18.26
51574415	2012	5	22	7110	145.35	18.28
51574415	2012	5	22	7120	145.56	18.32
51574415	2012	5	22	7130	145.62	18.35
51574415	2012	5	22	7140	145.12	18.39
51574415	2012	5	22	7150	143.96	18.42
51574415	2012	5	22	7160	144.41	18.44
51574415	2012	5	22	7170	144.57	18.5
51574415	2012	5	22	7180	144.15	18.54
51574415	2012	5	22	7190	144.54	18.64
51574415	2012	5	22	7200	145.43	18.63
51574415	2012	5	22	7210	146.46	18.62
51574415	2012	5	22	7220	146.95	18.64
51574415	2012	5	22	7230	146.22	18.64
51574415	2012	5	22	7240	146.67	18.65
51574415	2012	5	22	7250	146.89	18.67
51574415	2012	5	22	7260	145.43	18.67
51574415	2012	5	22	7270	143.79	18.69
51574415	2012	5	22	7280	143.31	18.72
51574415	2012	5	22	7290	142.39	18.75
51574415	2012	5	22	7300	141.78	18.78
51574415	2012	5	22	7310	141.13	18.8
51574415	2012	5	22	7320	140.92	18.83
51574415	2012	5	22	7330	140.46	18.85
51574415	2012	5	22	7340	139.83	18.88
51574415	2012	5	22	7350	138.67	18.91
51574415	2012	5	22	7360	137.95	18.94
51574415	2012	5	22	7370	137.7	18.96
51574415	2012	5	22	7380	137.36	19
51574415	2012	5	22	7390	136.58	19.02
51574415	2012	5	22	7400	136.45	19.05
51574415	2012	5	22	7410	136.18	19.07
51574415	2012	5	22	7420	135.29	19.1
51574415	2012	5	22	7430	134.58	19.1
51574415	2012	5	22	7440	134.26	19.11
51574415	2012	5	22	7450	133.78	19.14
51574415	2012	5	22	7460	133.44	19.14
51574415	2012	5	22	7470	133.46	19.15
51574415	2012	5	22	7480	132.85	19.17
51574415	2012	5	22	7490	132.45	19.19
51574415	2012	5	22	7500	132.21	19.21
51574415	2012	5	22	7510	132.02	19.22
51574415	2012	5	22	7520	132.13	19.23
51574415	2012	5	22	7530	131.74	19.25
51574415	2012	5	22	7540	131.17	19.25
51574415	2012	5	22	7550	130.36	19.27
51574415	2012	5	22	7560	129.78	19.28

51574415	2012	5	22	7570	128.92	19.29
51574415	2012	5	22	7580	127.58	19.32
51574415	2012	5	22	7590	125.58	19.32
51574415	2012	5	22	7600	123.76	19.33
51574415	2012	5	22	7610	122.05	19.36
51574415	2012	5	22	7620	121.1	19.39
51574415	2012	5	22	7630	121.59	19.52
51574415	2012	5	22	7640	122.87	19.64
51574415	2012	5	22	7650	122.87	19.7
51574415	2012	5	22	7660	123.21	19.75
51574415	2012	5	22	7670	123.15	19.82
51574415	2012	5	22	7680	123.15	19.89
51574415	2012	5	22	7690	123.22	19.97
51574415	2012	5	22	7700	123.1	20.05
51574415	2012	5	22	7710	123.3	20.11
51574415	2012	5	22	7720	123.15	20.18
51574415	2012	5	22	7730	122.86	20.31
51574415	2012	5	22	7740	121.64	20.37
51574415	2012	5	22	7750	120.66	20.42
51574415	2012	5	22	7760	119.22	20.46
51574415	2012	5	22	7770	118.43	20.52
51574415	2012	5	22	7780	118.28	20.56
51574415	2012	5	22	7790	118.29	20.61
51574415	2012	5	22	7800	118.13	20.67
51574415	2012	5	22	7810	117.95	20.7
51574415	2012	5	22	7820	117.58	20.75
51574415	2012	5	22	7830	117.07	20.8
51574415	2012	5	22	7840	117.21	20.86
51574415	2012	5	22	7850	116.52	20.92
51574415	2012	5	22	7860	115.68	20.99
51574415	2012	5	22	7870	115.1	21.04
51574415	2012	5	22	7880	114.19	21.09
51574415	2012	5	22	7890	113.85	21.11
51574415	2012	5	22	7900	113.18	21.17
51574415	2012	5	22	7910	112.47	21.21
51574415	2012	5	22	7920	112.16	21.25
51574415	2012	5	22	7930	112.68	21.28
51574415	2012	5	22	7940	113.78	21.35
51574415	2012	5	22	7950	113.74	21.4
51574415	2012	5	22	7960	113.73	21.42
51574415	2012	5	22	7970	113.3	21.45
51574415	2012	5	22	7980	113.51	21.47
51574415	2012	5	22	7990	114.1	21.52
51574415	2012	5	22	8000	113.89	21.56
51574415	2012	5	22	8010	114.14	21.57
51574415	2012	5	22	8020	114.16	21.6
51574415	2012	5	22	8030	113.97	21.63

51574415	2012	5	22	8040	113.95	21.66
51574415	2012	5	22	8050	113.61	21.78
51574415	2012	5	22	8060	113.98	21.81
51574415	2012	5	22	8070	114.21	21.81
51574415	2012	5	22	8080	114.33	21.81
51574415	2012	5	22	8090	114.45	21.83
51574415	2012	5	22	8100	114.36	21.88
51574415	2012	5	22	8110	114.21	21.88
51574415	2012	5	22	8120	114.22	21.9
51574415	2012	5	22	8130	114.15	21.93
51574415	2012	5	22	8140	114.3	21.94
51574415	2012	5	22	8150	113.57	21.96
51574415	2012	5	22	8160	112.82	21.97
51574415	2012	5	22	8170	112.29	21.99
51574415	2012	5	22	8180	112.09	22
51574415	2012	5	22	8190	111.6	22.02
51574415	2012	5	22	8200	111.86	22.03
51574415	2012	5	22	8210	111.98	22.04
51574415	2012	5	22	8220	111.97	22.07
51574415	2012	5	22	8230	112.4	22.07
51574415	2012	5	22	8240	112.53	22.08
51574415	2012	5	22	8250	112.65	22.09
51574415	2012	5	22	8260	112.23	22.1
51574415	2012	5	22	8270	112.32	22.1
51574415	2012	5	22	8280	112.16	22.12
51574415	2012	5	22	8290	112.03	22.14
51574415	2012	5	22	8300	112.05	22.14
51574415	2012	5	22	8310	111.77	22.14
51574415	2012	5	22	8320	111.84	22.15
51574415	2012	5	22	8330	111.58	22.16
51574415	2012	5	22	8340	111.89	22.17
51574415	2012	5	22	8350	111.68	22.17
51574415	2012	5	22	8360	111.56	22.18
51574415	2012	5	22	8370	112.16	22.18
51574415	2012	5	22	8380	110.83	22.19
51574415	2012	5	22	8390	108.74	22.21
51574415	2012	5	22	8400	107.91	22.21
51574415	2012	5	22	8410	106.49	22.23
51574415	2012	5	22	8420	104.67	22.25
51574415	2012	5	22	8430	103.43	22.25
51574415	2012	5	22	8440	103.47	22.25
51574415	2012	5	22	8450	103.57	22.26
51574415	2012	5	22	8460	103.53	22.28
51574415	2012	5	22	8470	103.74	22.28
51574415	2012	5	22	8480	103.74	22.28
51574415	2012	5	22	8490	103.82	22.29
51574415	2012	5	22	8500	103.83	22.3

51574415	2012	5	22	8510	103.62	22.31
51574415	2012	5	22	8520	103.66	22.32
51574415	2012	5	22	8530	103.56	22.32
51574415	2012	5	22	8540	103.55	22.32
51574415	2012	5	22	8550	103.97	22.33
51574415	2012	5	22	8560	104.01	22.33
51574415	2012	5	22	8570	104.03	22.33
51574415	2012	5	22	8580	103.95	22.34
51574415	2012	5	22	8590	104.05	22.35
51574415	2012	5	22	8600	103.77	22.34
51574415	2012	5	22	8610	103.92	22.35
51574415	2012	5	22	8620	103.78	22.35
51574415	2012	5	22	8630	104.17	22.35
51574415	2012	5	22	8640	103.63	22.35
51574415	2012	5	22	8650	103.09	22.36
51574415	2012	5	22	8660	103.25	22.36
51574415	2012	5	22	8670	102.83	22.37
51574415	2012	5	22	8680	102.83	22.37
51574415	2012	5	22	8690	102.39	22.37
51574415	2012	5	22	8700	102.15	22.37
51574415	2012	5	22	8710	101.9	22.38
51574415	2012	5	22	8720	101.47	22.38
51574415	2012	5	22	8730	101.5	22.39
51574415	2012	5	22	8740	101.25	22.39
51574415	2012	5	22	8750	100.73	22.4
51574415	2012	5	22	8760	101.15	22.4
51574415	2012	5	22	8770	101.57	22.4
51574415	2012	5	22	8780	101.54	22.4
51574415	2012	5	22	8790	101.42	22.42
51574415	2012	5	22	8800	100.22	22.42
51574415	2012	5	22	8810	99.72	22.43
51574415	2012	5	22	8820	99.19	22.43
51574415	2012	5	22	8830	99.13	22.44
51574415	2012	5	22	8840	98.57	22.44
51574415	2012	5	22	8850	98.12	22.46
51574415	2012	5	22	8860	98.16	22.45
51574415	2012	5	22	8870	97.83	22.45
51574415	2012	5	22	8880	97.4	22.46
51574415	2012	5	22	8890	96.98	22.46
51574415	2012	5	22	8900	96.96	22.46
51574415	2012	5	22	8910	96.64	22.46
51574415	2012	5	22	8920	96.42	22.47
51574415	2012	5	22	8930	95.87	22.47
51574415	2012	5	22	8940	95.96	22.47
51574415	2012	5	22	8950	95.48	22.49
51574415	2012	5	22	8960	94.63	22.49
51574415	2012	5	22	8970	94.15	22.49

51574415	2012	5	22	8980	94.03	22.5
51574415	2012	5	22	8990	93.68	22.5
51574415	2012	5	22	9000	93.14	22.5
51574415	2012	5	22	9010	92.91	22.5
51574415	2012	5	22	9020	93.48	22.5
51574415	2012	5	22	9030	93.2	22.51
51574415	2012	5	22	9040	92.82	22.51
51574415	2012	5	22	9050	92.13	22.51
51574415	2012	5	22	9060	91.84	22.52
51574415	2012	5	22	9070	91.82	22.51
51574415	2012	5	22	9080	91.62	22.52
51574415	2012	5	22	9090	91.65	22.52
51574415	2012	5	22	9100	91.02	22.53
51574415	2012	5	22	9110	90.57	22.53
51574415	2012	5	22	9120	90.67	22.53
51574415	2012	5	22	9130	90.75	22.54
51574415	2012	5	22	9140	90.41	22.55
51574415	2012	5	22	9150	90.18	22.56
51574415	2012	5	22	9160	89.81	22.57
51574415	2012	5	22	9170	88.72	22.58
51574415	2012	5	22	9180	86.92	22.6
51574415	2012	5	22	9190	85.21	22.63
51574415	2012	5	22	9200	83.3	22.67
51574415	2012	5	22	9210	81.01	22.71
51574415	2012	5	22	9220	79.33	22.74
51574415	2012	5	22	9230	77.5	22.77
51574415	2012	5	22	9240	75.94	22.8
51574415	2012	5	22	9250	74.01	22.84
51574415	2012	5	22	9260	72.43	22.89
51574415	2012	5	22	9270	70.6	22.91
51574415	2012	5	22	9280	68.86	22.95
51574415	2012	5	22	9290	67.31	23.02
51574415	2012	5	22	9300	65.89	23.14
51574415	2012	5	22	9310	63.67	23.35
51574415	2012	5	22	9320	61.68	23.76
51574415	2012	5	22	9330	59.3	23.99
51574415	2012	5	22	9340	57.06	24.14
51574415	2012	5	22	9350	54.88	24.35
51574415	2012	5	22	9360	53.21	24.5
51574415	2012	5	22	9370	52.02	24.68
51574415	2012	5	22	9380	50.44	24.84
51574415	2012	5	22	9390	48.87	25.03
51574415	2012	5	22	9400	47.4	25.17
51574415	2012	5	22	9410	46.23	25.34
51574415	2012	5	22	9420	45.82	25.46
51574415	2012	5	22	9430	45.68	25.54
51574415	2012	5	22	9440	45.84	25.63

51574415	2012	5	22	9450	46.36	25.77
51574415	2012	5	22	9460	46.72	25.81
51574415	2012	5	22	9470	46.7	25.85
51574415	2012	5	22	9480	47.26	25.91
51574415	2012	5	22	9490	47.75	25.96
51574415	2012	5	22	9500	47.67	26.02
51574415	2012	5	22	9510	48	26.1
51574415	2012	5	22	9520	48.56	26.13
51574415	2012	5	22	9530	49.52	26.22
51574415	2012	5	22	9540	49.69	26.25
51574415	2012	5	22	9550	50.24	26.35
51574415	2012	5	22	9560	51.08	26.42
51574415	2012	5	22	9570	52.17	26.43
51574415	2012	5	22	9580	53.48	26.42
51574415	2012	5	22	9590	54.74	26.48
51574415	2012	5	22	9600	55.89	26.53
51574415	2012	5	22	9610	56.94	26.55
51574415	2012	5	22	9620	58.18	26.53
51574415	2012	5	22	9630	59.39	26.53
51574415	2012	5	22	9640	60.21	26.57
51574415	2012	5	22	9650	60.98	26.58
51574415	2012	5	22	9660	62.04	26.6
51574415	2012	5	22	9670	62.81	26.6
51574415	2012	5	22	9680	62.8	26.6
51574415	2012	5	22	9690	63.45	26.6
51574415	2012	5	22	9700	63.07	26.63
51574415	2012	5	22	9710	63.86	26.66
51574415	2012	5	22	9720	64.81	26.67
51574415	2012	5	22	9730	64.92	26.67
51574415	2012	5	22	9740	64.93	26.65
51574415	2012	5	22	9750	65.51	26.6
51574415	2012	5	22	9760	66.36	26.54
51574415	2012	5	22	9770	67.19	26.5
51574415	2012	5	22	9780	68.12	26.39
51574415	2012	5	22	9790	68.95	26.24
51574415	2012	5	22	9800	69.15	26.18
51574415	2012	5	22	9810	69.05	26.1
51574415	2012	5	22	9820	69.31	25.94
51574415	2012	5	22	9830	69.58	25.84
51574415	2012	5	22	9840	69.72	25.77
51574415	2012	5	22	9850	69.76	25.71
51574415	2012	5	22	9860	69.8	25.66
51574415	2012	5	22	9870	69.91	25.61
51574415	2012	5	22	9880	69.8	25.54
51574415	2012	5	22	9890	69.58	25.49
51574415	2012	5	22	9900	69.67	25.43
51574415	2012	5	22	9910	69.95	25.38

51574415	2012	5	22	9920	70.51	25.35
51574415	2012	5	22	9930	70.35	25.35
51574415	2012	5	22	9940	69.83	25.32
51574415	2012	5	22	9950	69.69	25.3
51574415	2012	5	22	9960	70.34	25.27
51574415	2012	5	22	9970	70.58	25.23
51574415	2012	5	22	9980	71.07	25.1
51574415	2012	5	22	9990	71.32	25.03
51574415	2012	5	22	10000	71.93	24.93
51574415	2012	5	22	10010	71.56	24.84
51574415	2012	5	22	10020	69.53	24.82
51574415	2012	5	22	10030	67.26	24.82
51574415	2012	5	22	10040	64.96	24.95
51574415	2012	5	22	10050	62.64	25.07
51574415	2012	5	22	10060	59.92	25.17
51574415	2012	5	22	10070	57.47	25.28
51574415	2012	5	22	10080	55.48	25.4
51574415	2012	5	22	10090	53.54	25.51
51574415	2012	5	22	10100	51.24	25.65
51574415	2012	5	22	10110	48.76	25.79
51574415	2012	5	22	10120	47.31	25.9
51574415	2012	5	22	10130	47.05	26.06
51574415	2012	5	22	10140	45.88	26.1
51574415	2012	5	22	10150	45.5	26.18
51574415	2012	5	22	10160	44.76	26.25
51574415	2012	5	22	10170	43.57	26.35
51574415	2012	5	22	10180	43.05	26.37
51574415	2012	5	22	10190	43.72	26.5
51574415	2012	5	22	10200	43.8	26.56
51574415	2012	5	22	10210	43.73	26.56
51574415	2012	5	22	10220	44.34	26.64
51574415	2012	5	22	10230	44.91	26.67
51574415	2012	5	22	10240	45.37	26.7
51574415	2012	5	22	10250	46.33	26.78
51574415	2012	5	22	10260	47.21	26.82
51574415	2012	5	22	10270	48.44	26.83
51574415	2012	5	22	10280	49.19	26.82
51574415	2012	5	22	10290	50.29	26.84
51574415	2012	5	22	10300	51.06	26.87
51574415	2012	5	22	10310	51.16	26.86
51574415	2012	5	22	10320	51.44	26.88
51574415	2012	5	22	10330	51.62	26.91
51574415	2012	5	22	10340	51.35	26.92
51574415	2012	5	22	10350	51.08	26.93
51574415	2012	5	22	10360	51.05	26.96
51574415	2012	5	22	10370	50.83	26.96
51574415	2012	5	22	10380	50.67	26.99

51574415	2012	5	22	10390	50.59	27
51574415	2012	5	22	10400	50.38	27.03
51574415	2012	5	22	10410	50.03	27.04
51574415	2012	5	22	10420	48.59	27.06
51574415	2012	5	22	10430	47.59	27.08
51574415	2012	5	22	10440	47.18	27.1
51574415	2012	5	22	10450	46.76	27.11
51574415	2012	5	22	10460	46.31	27.13
51574415	2012	5	22	10470	45.97	27.14
51574415	2012	5	22	10480	45.71	27.16
51574415	2012	5	22	10490	45.27	27.17
51574415	2012	5	22	10500	44.07	27.19
51574415	2012	5	22	10510	42.89	27.21
51574415	2012	5	22	10520	42.23	27.22
51574415	2012	5	22	10530	41.78	27.23
51574415	2012	5	22	10540	41.38	27.24
51574415	2012	5	22	10550	41.1	27.25
51574415	2012	5	22	10560	40.45	27.27
51574415	2012	5	22	10570	40.4	27.28
51574415	2012	5	22	10580	40.26	27.29
51574415	2012	5	22	10590	40.03	27.31
51574415	2012	5	22	10600	40.4	27.3
51574415	2012	5	22	10610	39.83	27.32
51574415	2012	5	22	10620	37.8	27.33
51574415	2012	5	22	10630	35.35	27.35
51574415	2012	5	22	10640	33.05	27.37
51574415	2012	5	22	10650	30.43	27.39
51574415	2012	5	22	10660	28.4	27.42
51574415	2012	5	22	10670	26.76	27.44
51574415	2012	5	22	10680	24.69	27.46
51574415	2012	5	22	10690	22.25	27.49
51574415	2012	5	22	10700	19.65	27.5
51574415	2012	5	22	10710	17.88	27.52
51574415	2012	5	22	10720	17.04	27.53
51574415	2012	5	22	10730	15.54	27.55
51574415	2012	5	22	10740	13.92	27.57
51574415	2012	5	22	10750	11.97	27.6
51574415	2012	5	22	10760	9.36	27.62
51574415	2012	5	22	10770	7.07	27.65
51574415	2012	5	22	10780	5.21	27.67
51574415	2012	5	22	10790	3.13	27.7
51574415	2012	5	22	10800	2.61	27.72
51574415	2012	5	22	10810	2.52	27.73
51574415	2012	5	22	10820	2.32	27.75
51574415	2012	5	22	10830	2.28	27.78
51574415	2012	5	22	10840	2.43	27.82
51574415	2012	5	22	10850	2.1	27.83

51574415	2012	5	22	10860	1.19	27.85
51574415	2012	5	22	10870	0.6	27.85
51574415	2012	5	22	10880	0.67	27.85
51574415	2012	5	22	10890	0.64	27.86
51574415	2012	5	22	10900	0.66	27.89
51574415	2012	5	22	10910	0.62	27.93
51574415	2012	5	22	10920	0.64	27.97
51574415	2012	5	22	10930	0.62	28
51574415	2012	5	22	10940	0.67	28.01
51574415	2012	5	22	10950	0.66	28.03
51574415	2012	5	22	10960	0.61	28.04
51574415	2012	5	22	10970	0.63	28.06
51574415	2012	5	22	10980	0.48	28.1
51574415	2012	5	22	10990	0.51	28.1
51574415	2012	5	22	11000	0.5	28.11
51574415	2012	5	22	11010	0.5	28.14
51646515	2012	5	23	0	1.04	27.59
51646515	2012	5	23	10	2.29	27.57
51646515	2012	5	23	20	3.08	27.57
51646515	2012	5	23	30	3.05	27.58
51646515	2012	5	23	40	3.02	27.58
51646515	2012	5	23	50	2.53	27.59
51646515	2012	5	23	60	2.72	27.6
51646515	2012	5	23	70	2.87	27.59
51646515	2012	5	23	80	3.51	27.58
51646515	2012	5	23	90	5.33	27.57
51646515	2012	5	23	100	7.59	27.55
51646515	2012	5	23	110	9.28	27.53
51646515	2012	5	23	120	10	27.53
51646515	2012	5	23	130	11.01	27.53
51646515	2012	5	23	140	12.71	27.52
51646515	2012	5	23	150	13.32	27.51
51646515	2012	5	23	160	13.54	27.51
51646515	2012	5	23	170	13.78	27.51
51646515	2012	5	23	180	13.96	27.5
51646515	2012	5	23	190	14.06	27.52
51646515	2012	5	23	200	14.11	27.51
51646515	2012	5	23	210	14.41	27.51
51646515	2012	5	23	220	14.8	27.51
51646515	2012	5	23	230	15.12	27.5
51646515	2012	5	23	240	15.36	27.51
51646515	2012	5	23	250	15.51	27.51
51646515	2012	5	23	260	15.66	27.5
51646515	2012	5	23	270	15.86	27.5
51646515	2012	5	23	280	15.85	27.5
51646515	2012	5	23	290	14.84	27.51
51646515	2012	5	23	300	14.58	27.53

51646515	2012	5	23	310	15.4	27.51
51646515	2012	5	23	320	17.68	27.5
51646515	2012	5	23	330	18.97	27.49
51646515	2012	5	23	340	19.79	27.48
51646515	2012	5	23	350	22.07	27.46
51646515	2012	5	23	360	24.75	27.44
51646515	2012	5	23	370	26.62	27.42
51646515	2012	5	23	380	28	27.41
51646515	2012	5	23	390	29.24	27.4
51646515	2012	5	23	400	31.44	27.38
51646515	2012	5	23	410	33.23	27.36
51646515	2012	5	23	420	34.37	27.35
51646515	2012	5	23	430	35.64	27.35
51646515	2012	5	23	440	37.14	27.32
51646515	2012	5	23	450	38.84	27.31
51646515	2012	5	23	460	40.8	27.28
51646515	2012	5	23	470	41.96	27.27
51646515	2012	5	23	480	42.88	27.26
51646515	2012	5	23	490	43.71	27.25
51646515	2012	5	23	500	44.85	27.23
51646515	2012	5	23	510	46.8	27.21
51646515	2012	5	23	520	49.09	27.18
51646515	2012	5	23	530	51.37	27.17
51646515	2012	5	23	540	53.25	27.13
51646515	2012	5	23	550	54.44	27.08
51646515	2012	5	23	560	55.74	27.05
51646515	2012	5	23	570	56.95	27
51646515	2012	5	23	580	58.67	26.93
51646515	2012	5	23	590	60.22	26.83
51646515	2012	5	23	600	61.71	26.77
51646515	2012	5	23	610	63.85	26.67
51646515	2012	5	23	620	65.44	26.64
51646515	2012	5	23	630	66.63	26.56
51646515	2012	5	23	640	67.81	26.49
51646515	2012	5	23	650	68.94	26.45
51646515	2012	5	23	660	69.95	26.4
51646515	2012	5	23	670	70.81	26.38
51646515	2012	5	23	680	72.15	26.34
51646515	2012	5	23	690	73.41	26.3
51646515	2012	5	23	700	74.26	26.28
51646515	2012	5	23	710	74.88	26.25
51646515	2012	5	23	720	76.34	26.21
51646515	2012	5	23	730	77.81	26.17
51646515	2012	5	23	740	79.37	26.14
51646515	2012	5	23	750	81.11	26.1
51646515	2012	5	23	760	83.16	26.07
51646515	2012	5	23	770	84.32	26.04

51646515	2012	5	23	780	84.71	26.03
51646515	2012	5	23	790	86.01	25.98
51646515	2012	5	23	800	87.27	25.9
51646515	2012	5	23	810	89.51	25.53
51646515	2012	5	23	820	90.54	25.5
51646515	2012	5	23	830	91.03	25.44
51646515	2012	5	23	840	91.1	25.26
51646515	2012	5	23	850	91.62	25.2
51646515	2012	5	23	860	92.42	25.14
51646515	2012	5	23	870	92.94	25.07
51646515	2012	5	23	880	93.53	25.02
51646515	2012	5	23	890	94.13	24.96
51646515	2012	5	23	900	94.89	24.85
51646515	2012	5	23	910	96.04	24.78
51646515	2012	5	23	920	96.8	24.72
51646515	2012	5	23	930	97.34	24.65
51646515	2012	5	23	940	98.25	24.59
51646515	2012	5	23	950	99.15	24.53
51646515	2012	5	23	960	99.98	24.46
51646515	2012	5	23	970	100.56	24.39
51646515	2012	5	23	980	100.79	24.34
51646515	2012	5	23	990	101.53	24.28
51646515	2012	5	23	1000	102.06	24.24
51646515	2012	5	23	1010	102.23	24.17
51646515	2012	5	23	1020	103.11	24.09
51646515	2012	5	23	1030	103.96	24.02
51646515	2012	5	23	1040	105.82	23.94
51646515	2012	5	23	1050	108.4	23.73
51646515	2012	5	23	1060	111.12	23.53
51646515	2012	5	23	1070	112.07	23.46
51646515	2012	5	23	1080	112.51	23.38
51646515	2012	5	23	1090	112.96	23.35
51646515	2012	5	23	1100	112.88	23.31
51646515	2012	5	23	1110	112.68	23.25
51646515	2012	5	23	1120	112.64	23.2
51646515	2012	5	23	1130	112.47	23.16
51646515	2012	5	23	1140	112.46	23.12
51646515	2012	5	23	1150	112.98	23.07
51646515	2012	5	23	1160	113.62	23.03
51646515	2012	5	23	1170	114.37	22.99
51646515	2012	5	23	1180	115.13	22.94
51646515	2012	5	23	1190	116.29	22.81
51646515	2012	5	23	1200	117.21	22.72
51646515	2012	5	23	1210	118.63	22.67
51646515	2012	5	23	1220	120.8	22.53
51646515	2012	5	23	1230	122.51	22.46
51646515	2012	5	23	1240	124.24	22.41

51646515	2012	5	23	1250	125.41	22.39
51646515	2012	5	23	1260	126.67	22.37
51646515	2012	5	23	1270	128.04	22.34
51646515	2012	5	23	1280	128.84	22.32
51646515	2012	5	23	1290	129.68	22.29
51646515	2012	5	23	1300	130.62	22.26
51646515	2012	5	23	1310	132.24	22.22
51646515	2012	5	23	1320	133.59	22.17
51646515	2012	5	23	1330	134.47	22.07
51646515	2012	5	23	1340	134.82	21.91
51646515	2012	5	23	1350	135.01	21.85
51646515	2012	5	23	1360	135.81	21.79
51646515	2012	5	23	1370	136.56	21.71
51646515	2012	5	23	1380	137.36	21.61
51646515	2012	5	23	1390	138.46	21.53
51646515	2012	5	23	1400	139.75	21.42
51646515	2012	5	23	1410	140.23	21.28
51646515	2012	5	23	1420	140.11	21.16
51646515	2012	5	23	1430	140.21	21.04
51646515	2012	5	23	1440	140.13	20.91
51646515	2012	5	23	1450	140.19	20.78
51646515	2012	5	23	1460	140.03	20.68
51646515	2012	5	23	1470	139.14	20.59
51646515	2012	5	23	1480	138.51	20.5
51646515	2012	5	23	1490	137.93	20.4
51646515	2012	5	23	1500	137.35	20.32
51646515	2012	5	23	1510	136.93	20.22
51646515	2012	5	23	1520	136.89	20.13
51646515	2012	5	23	1530	137.4	20.02
51646515	2012	5	23	1540	137.01	19.97
51646515	2012	5	23	1550	136.71	19.92
51646515	2012	5	23	1560	136.9	19.89
51646515	2012	5	23	1570	137.29	19.78
51646515	2012	5	23	1580	137.56	19.74
51646515	2012	5	23	1590	138.82	19.72
51646515	2012	5	23	1600	140.07	19.67
51646515	2012	5	23	1610	141.31	19.62
51646515	2012	5	23	1620	143.15	19.6
51646515	2012	5	23	1630	144.47	19.58
51646515	2012	5	23	1640	144.6	19.51
51646515	2012	5	23	1650	146.19	19.45
51646515	2012	5	23	1660	148.48	19.38
51646515	2012	5	23	1670	150.05	19.32
51646515	2012	5	23	1680	152.67	19.25
51646515	2012	5	23	1690	155.48	19.19
51646515	2012	5	23	1700	157.52	19.14
51646515	2012	5	23	1710	158.92	19.06

51646515	2012	5	23	1720	159.78	19
51646515	2012	5	23	1730	160.08	18.94
51646515	2012	5	23	1740	160.92	18.9
51646515	2012	5	23	1750	161.57	18.85
51646515	2012	5	23	1760	161.63	18.79
51646515	2012	5	23	1770	161.61	18.67
51646515	2012	5	23	1780	161.3	18.61
51646515	2012	5	23	1790	162.56	18.57
51646515	2012	5	23	1800	164.29	18.53
51646515	2012	5	23	1810	165.06	18.46
51646515	2012	5	23	1820	164.93	18.37
51646515	2012	5	23	1830	165.67	18.28
51646515	2012	5	23	1840	166.85	18.25
51646515	2012	5	23	1850	168.55	18.14
51646515	2012	5	23	1860	169.5	18.07
51646515	2012	5	23	1870	170.12	18.05
51646515	2012	5	23	1880	170.95	18.03
51646515	2012	5	23	1890	172.12	17.99
51646515	2012	5	23	1900	172.95	17.92
51646515	2012	5	23	1910	173.61	17.87
51646515	2012	5	23	1920	175	17.82
51646515	2012	5	23	1930	175.46	17.78
51646515	2012	5	23	1940	176.2	17.75
51646515	2012	5	23	1950	177.51	17.7
51646515	2012	5	23	1960	178.26	17.65
51646515	2012	5	23	1970	178.75	17.6
51646515	2012	5	23	1980	178.82	17.49
51646515	2012	5	23	1990	179.51	17.45
51646515	2012	5	23	2000	179.79	17.42
51646515	2012	5	23	2010	179.55	17.39
51646515	2012	5	23	2020	180.95	17.35
51646515	2012	5	23	2030	182.13	17.29
51646515	2012	5	23	2040	183.91	17.26
51646515	2012	5	23	2050	185.37	17.23
51646515	2012	5	23	2060	186.57	17.19
51646515	2012	5	23	2070	187.31	17.16
51646515	2012	5	23	2080	187.78	17.09
51646515	2012	5	23	2090	187.62	17.03
51646515	2012	5	23	2100	187.32	16.97
51646515	2012	5	23	2110	187.08	16.96
51646515	2012	5	23	2120	186.78	16.92
51646515	2012	5	23	2130	186.66	16.9
51646515	2012	5	23	2140	187.55	16.88
51646515	2012	5	23	2150	188.14	16.83
51646515	2012	5	23	2160	189.06	16.8
51646515	2012	5	23	2170	189.5	16.77
51646515	2012	5	23	2180	189.63	16.75

51646515	2012	5	23	2190	189.69	16.71
51646515	2012	5	23	2200	189.98	16.66
51646515	2012	5	23	2210	189.81	16.64
51646515	2012	5	23	2220	189.64	16.63
51646515	2012	5	23	2230	190.34	16.62
51646515	2012	5	23	2240	191.78	16.6
51646515	2012	5	23	2250	192.86	16.58
51646515	2012	5	23	2260	194.98	16.56
51646515	2012	5	23	2270	196.3	16.53
51646515	2012	5	23	2280	196.96	16.51
51646515	2012	5	23	2290	197.31	16.49
51646515	2012	5	23	2300	197.53	16.46
51646515	2012	5	23	2310	198.23	16.4
51646515	2012	5	23	2320	198.23	16.35
51646515	2012	5	23	2330	198.66	16.24
51646515	2012	5	23	2340	200.09	16.17
51646515	2012	5	23	2350	200.71	16.16
51646515	2012	5	23	2360	201.16	16.14
51646515	2012	5	23	2370	201.52	16.07
51646515	2012	5	23	2380	202.67	15.99
51646515	2012	5	23	2390	204.41	16
51646515	2012	5	23	2400	205.67	16
51646515	2012	5	23	2410	206.89	15.96
51646515	2012	5	23	2420	208.62	15.89
51646515	2012	5	23	2430	209.88	15.83
51646515	2012	5	23	2440	211.37	15.78
51646515	2012	5	23	2450	213.47	15.76
51646515	2012	5	23	2460	216.17	15.74
51646515	2012	5	23	2470	218.4	15.72
51646515	2012	5	23	2480	220.09	15.69
51646515	2012	5	23	2490	220.92	15.67
51646515	2012	5	23	2500	220.67	15.62
51646515	2012	5	23	2510	220.52	15.57
51646515	2012	5	23	2520	220.47	15.51
51646515	2012	5	23	2530	220.38	15.49
51646515	2012	5	23	2540	220.46	15.46
51646515	2012	5	23	2550	220.31	15.42
51646515	2012	5	23	2560	220.88	15.36
51646515	2012	5	23	2570	222	15.35
51646515	2012	5	23	2580	223.01	15.33
51646515	2012	5	23	2590	224.12	15.32
51646515	2012	5	23	2600	224.41	15.29
51646515	2012	5	23	2610	225.42	15.23
51646515	2012	5	23	2620	226.68	15.17
51646515	2012	5	23	2630	227.27	15.16
51646515	2012	5	23	2640	227.87	15.15
51646515	2012	5	23	2650	228.54	15.14

51646515	2012	5	23	2660	228.69	15.09
51646515	2012	5	23	2670	229.86	15.05
51646515	2012	5	23	2680	230.62	15.04
51646515	2012	5	23	2690	231.25	15.03
51646515	2012	5	23	2700	232.22	15
51646515	2012	5	23	2710	233.07	14.98
51646515	2012	5	23	2720	233.78	14.95
51646515	2012	5	23	2730	234.4	14.91
51646515	2012	5	23	2740	235.76	14.83
51646515	2012	5	23	2750	237.18	14.79
51646515	2012	5	23	2760	238.54	14.76
51646515	2012	5	23	2770	238.82	14.71
51646515	2012	5	23	2780	239.39	14.67
51646515	2012	5	23	2790	240.26	14.64
51646515	2012	5	23	2800	241.35	14.6
51646515	2012	5	23	2810	242.6	14.58
51646515	2012	5	23	2820	243.44	14.56
51646515	2012	5	23	2830	243.99	14.53
51646515	2012	5	23	2840	244.22	14.5
51646515	2012	5	23	2850	244.59	14.49
51646515	2012	5	23	2860	245.16	14.47
51646515	2012	5	23	2870	245.38	14.45
51646515	2012	5	23	2880	244.98	14.42
51646515	2012	5	23	2890	245.21	14.41
51646515	2012	5	23	2900	245.4	14.4
51646515	2012	5	23	2910	245.6	14.37
51646515	2012	5	23	2920	245.63	14.36
51646515	2012	5	23	2930	245.65	14.35
51646515	2012	5	23	2940	245.63	14.33
51646515	2012	5	23	2950	245.48	14.32
51646515	2012	5	23	2960	245.41	14.31
51646515	2012	5	23	2970	245.6	14.3
51646515	2012	5	23	2980	245.75	14.28
51646515	2012	5	23	2990	245.42	14.28
51646515	2012	5	23	3000	245.51	14.26
51646515	2012	5	23	3010	245.76	14.26
51646515	2012	5	23	3020	245.87	14.25
51646515	2012	5	23	3030	245.96	14.24
51646515	2012	5	23	3040	246.07	14.23
51646515	2012	5	23	3050	246.14	14.22
51646515	2012	5	23	3060	246.09	14.21
51646515	2012	5	23	3070	245.99	14.21
51646515	2012	5	23	3080	245.97	14.2
51646515	2012	5	23	3090	245.95	14.2
51646515	2012	5	23	3100	245.82	14.19
51646515	2012	5	23	3110	245.57	14.17
51646515	2012	5	23	3120	245.44	14.17

51646515	2012	5	23	3130	245.56	14.17
51646515	2012	5	23	3140	245.82	14.15
51646515	2012	5	23	3150	246.05	14.14
51646515	2012	5	23	3160	246.18	14.13
51646515	2012	5	23	3170	246.03	14.11
51646515	2012	5	23	3180	246.45	14.11
51646515	2012	5	23	3190	247.62	14.1
51646515	2012	5	23	3200	249.25	14.1
51646515	2012	5	23	3210	250.86	14.09
51646515	2012	5	23	3220	252.14	14.07
51646515	2012	5	23	3230	253.35	14.07
51646515	2012	5	23	3240	255.15	14.05
51646515	2012	5	23	3250	255.61	14.04
51646515	2012	5	23	3260	256.36	14.04
51646515	2012	5	23	3270	257.08	14.03
51646515	2012	5	23	3280	257.7	14.01
51646515	2012	5	23	3290	258.28	14.01
51646515	2012	5	23	3300	258.77	14
51646515	2012	5	23	3310	258.75	14
51646515	2012	5	23	3320	258.51	13.99
51646515	2012	5	23	3330	258.58	13.97
51646515	2012	5	23	3340	259.07	13.97
51646515	2012	5	23	3350	259.44	13.96
51646515	2012	5	23	3360	259.66	13.96
51646515	2012	5	23	3370	259.74	13.96
51646515	2012	5	23	3380	260.36	13.96
51646515	2012	5	23	3390	260.88	13.96
51646515	2012	5	23	3400	261.17	13.96
51646515	2012	5	23	3410	261.15	13.94
51646515	2012	5	23	3420	260.56	13.93
51646515	2012	5	23	3430	261.03	13.93
51646515	2012	5	23	3440	260.93	13.93
51646515	2012	5	23	3450	260.76	13.92
51646515	2012	5	23	3460	260.58	13.92
51646515	2012	5	23	3470	260.6	13.91
51646515	2012	5	23	3480	260.75	13.91
51646515	2012	5	23	3490	260.72	13.91
51646515	2012	5	23	3500	260.73	13.9
51646515	2012	5	23	3510	260.54	13.9
51646515	2012	5	23	3520	260.57	13.89
51646515	2012	5	23	3530	260.7	13.89
51646515	2012	5	23	3540	260.62	13.89
51646515	2012	5	23	3550	260.68	13.89
51646515	2012	5	23	3560	260.58	13.9
51646515	2012	5	23	3570	260.56	13.89
51646515	2012	5	23	3580	259.93	13.89
51646515	2012	5	23	3590	260.43	13.89

51646515	2012	5	23	3600	260.94	13.89
51646515	2012	5	23	3610	260.95	13.89
51646515	2012	5	23	3620	260.9	13.89
51646515	2012	5	23	3630	260.98	13.89
51646515	2012	5	23	3640	261.14	13.89
51646515	2012	5	23	3650	261.48	13.89
51646515	2012	5	23	3660	262.43	13.89
51646515	2012	5	23	3670	263.1	13.89
51646515	2012	5	23	3680	263.52	13.88
51646515	2012	5	23	3690	263.76	13.88
51646515	2012	5	23	3700	264.07	13.88
51646515	2012	5	23	3710	263.58	13.88
51646515	2012	5	23	3720	263.78	13.88
51646515	2012	5	23	3730	263.87	13.88
51646515	2012	5	23	3740	263.93	13.88
51646515	2012	5	23	3750	263.98	13.86
51646515	2012	5	23	3760	263.96	13.86
51646515	2012	5	23	3770	263.87	13.86
51646515	2012	5	23	3780	263.83	13.86
51646515	2012	5	23	3790	263.89	13.86
51646515	2012	5	23	3800	264.01	13.86
51646515	2012	5	23	3810	264.02	13.86
51646515	2012	5	23	3820	263.92	13.85
51646515	2012	5	23	3830	263.84	13.85
51646515	2012	5	23	3840	263.91	13.85
51646515	2012	5	23	3850	263.78	13.85
51646515	2012	5	23	3860	263.71	13.8
51646515	2012	5	23	3870	263.38	13.8
51646515	2012	5	23	3880	262.95	13.8
51646515	2012	5	23	3890	263.21	13.8
51646515	2012	5	23	3900	263.8	13.85
51646515	2012	5	23	3910	263.61	13.85
51646515	2012	5	23	3920	263.38	13.8
51646515	2012	5	23	3930	262.93	13.8
51646515	2012	5	23	3940	262.67	13.79
51646515	2012	5	23	3950	262.95	13.8
51646515	2012	5	23	3960	263.1	13.85
51646515	2012	5	23	3970	263.08	13.8
51646515	2012	5	23	3980	263.02	13.8
51646515	2012	5	23	3990	262.88	13.79
51646515	2012	5	23	4000	262.73	13.8
51646515	2012	5	23	4010	262.68	13.8
51646515	2012	5	23	4020	262.24	13.79
51646515	2012	5	23	4030	262.21	13.79
51646515	2012	5	23	4040	261.93	13.79
51646515	2012	5	23	4050	262.39	13.79
51646515	2012	5	23	4060	262.98	13.8

51646515	2012	5	23	4070	263.17	13.8
51646515	2012	5	23	4080	263.03	13.8
51646515	2012	5	23	4090	262.59	13.8
51646515	2012	5	23	4100	262.48	13.79
51646515	2012	5	23	4110	262.4	13.79
51646515	2012	5	23	4120	262.29	13.8
51646515	2012	5	23	4130	262.54	13.79
51646515	2012	5	23	4140	262.73	13.79
51646515	2012	5	23	4150	263.19	13.79
51646515	2012	5	23	4160	263.71	13.8
51646515	2012	5	23	4170	264.62	13.8
51646515	2012	5	23	4180	265.9	13.8
51646515	2012	5	23	4190	266.54	13.85
51646515	2012	5	23	4200	267	13.85
51646515	2012	5	23	4210	267.45	13.86
51646515	2012	5	23	4220	267.37	13.8
51646515	2012	5	23	4230	267.8	13.85
51646515	2012	5	23	4240	267.96	13.85
51646515	2012	5	23	4250	267.98	13.85
51646515	2012	5	23	4260	267.94	13.8
51646515	2012	5	23	4270	267.91	13.85
51646515	2012	5	23	4280	267.59	13.8
51646515	2012	5	23	4290	267.24	13.85
51646515	2012	5	23	4300	267.09	13.8
51646515	2012	5	23	4310	267.26	13.8
51646515	2012	5	23	4320	267.35	13.8
51646515	2012	5	23	4330	267.52	13.8
51646515	2012	5	23	4340	267.34	13.85
51646515	2012	5	23	4350	266.78	13.8
51646515	2012	5	23	4360	266.8	13.8
51646515	2012	5	23	4370	266.77	13.8
51646515	2012	5	23	4380	266.39	13.8
51646515	2012	5	23	4390	266.04	13.79
51646515	2012	5	23	4400	265.54	13.79
51646515	2012	5	23	4410	264.94	13.79
51646515	2012	5	23	4420	264.48	13.8
51646515	2012	5	23	4430	264.23	13.79
51646515	2012	5	23	4440	263.8	13.79
51646515	2012	5	23	4450	263.38	13.79
51646515	2012	5	23	4460	263.18	13.79
51646515	2012	5	23	4470	263.08	13.79
51646515	2012	5	23	4480	263.08	13.78
51646515	2012	5	23	4490	263.04	13.79
51646515	2012	5	23	4500	263.06	13.78
51646515	2012	5	23	4510	262.65	13.79
51646515	2012	5	23	4520	262.41	13.79
51646515	2012	5	23	4530	262.07	13.79

51646515	2012	5	23	4540	261.34	13.79
51646515	2012	5	23	4550	260.72	13.78
51646515	2012	5	23	4560	259.71	13.78
51646515	2012	5	23	4570	258.62	13.78
51646515	2012	5	23	4580	257.33	13.78
51646515	2012	5	23	4590	256.53	13.77
51646515	2012	5	23	4600	256.23	13.77
51646515	2012	5	23	4610	255.15	13.78
51646515	2012	5	23	4620	253.89	13.77
51646515	2012	5	23	4630	253.1	13.77
51646515	2012	5	23	4640	251.65	13.75
51646515	2012	5	23	4650	250.89	13.76
51646515	2012	5	23	4660	251.61	13.78
51646515	2012	5	23	4670	251.83	13.77
51646515	2012	5	23	4680	251.77	13.77
51646515	2012	5	23	4690	251.82	13.77
51646515	2012	5	23	4700	252.01	13.77
51646515	2012	5	23	4710	252	13.78
51646515	2012	5	23	4720	251.91	13.78
51646515	2012	5	23	4730	252.03	13.78
51646515	2012	5	23	4740	251.74	13.78
51646515	2012	5	23	4750	251.68	13.76
51646515	2012	5	23	4760	251.89	13.77
51646515	2012	5	23	4770	251.19	13.78
51646515	2012	5	23	4780	251.05	13.78
51646515	2012	5	23	4790	251.3	13.78
51646515	2012	5	23	4800	251.42	13.78
51646515	2012	5	23	4810	251.46	13.78
51646515	2012	5	23	4820	251.54	13.78
51646515	2012	5	23	4830	251.08	13.78
51646515	2012	5	23	4840	250.73	13.78
51646515	2012	5	23	4850	250.7	13.78
51646515	2012	5	23	4860	251.11	13.78
51646515	2012	5	23	4870	251.38	13.78
51646515	2012	5	23	4880	251.28	13.78
51646515	2012	5	23	4890	250.87	13.78
51646515	2012	5	23	4900	250.77	13.78
51646515	2012	5	23	4910	250.72	13.78
51646515	2012	5	23	4920	250.63	13.78
51646515	2012	5	23	4930	250.67	13.78
51646515	2012	5	23	4940	251.59	13.78
51646515	2012	5	23	4950	251.75	13.78
51646515	2012	5	23	4960	251.74	13.78
51646515	2012	5	23	4970	251.63	13.78
51646515	2012	5	23	4980	251.65	13.79
51646515	2012	5	23	4990	251.72	13.79
51646515	2012	5	23	5000	251.75	13.79

51646515	2012	5	23	5010	251.73	13.79
51646515	2012	5	23	5020	251.19	13.78
51646515	2012	5	23	5030	249.8	13.78
51646515	2012	5	23	5040	248.29	13.78
51646515	2012	5	23	5050	247.62	13.78
51646515	2012	5	23	5060	246	13.77
51646515	2012	5	23	5070	245.72	13.78
51646515	2012	5	23	5080	245.85	13.78
51646515	2012	5	23	5090	245.64	13.78
51646515	2012	5	23	5100	245.13	13.78
51646515	2012	5	23	5110	245.42	13.78
51646515	2012	5	23	5120	245.64	13.78
51646515	2012	5	23	5130	245.8	13.79
51646515	2012	5	23	5140	245.8	13.78
51646515	2012	5	23	5150	245.45	13.78
51646515	2012	5	23	5160	245.4	13.79
51646515	2012	5	23	5170	245.69	13.85
51646515	2012	5	23	5180	244.66	13.8
51646515	2012	5	23	5190	243.73	13.85
51646515	2012	5	23	5200	242.77	13.85
51646515	2012	5	23	5210	242.44	13.86
51646515	2012	5	23	5220	241.08	13.86
51646515	2012	5	23	5230	239.89	13.87
51646515	2012	5	23	5240	239.33	13.88
51646515	2012	5	23	5250	239.12	13.89
51646515	2012	5	23	5260	238.1	13.88
51646515	2012	5	23	5270	237.57	13.88
51646515	2012	5	23	5280	236.64	13.89
51646515	2012	5	23	5290	236.26	13.89
51646515	2012	5	23	5300	236.88	13.9
51646515	2012	5	23	5310	235.78	13.9
51646515	2012	5	23	5320	234.62	13.91
51646515	2012	5	23	5330	233.56	13.93
51646515	2012	5	23	5340	233.09	13.94
51646515	2012	5	23	5350	233.93	13.98
51646515	2012	5	23	5360	233.51	13.99
51646515	2012	5	23	5370	233.8	14
51646515	2012	5	23	5380	233.69	14.01
51646515	2012	5	23	5390	233.6	14.03
51646515	2012	5	23	5400	234.08	14.05
51646515	2012	5	23	5410	234.09	14.07
51646515	2012	5	23	5420	233.92	14.08
51646515	2012	5	23	5430	233.68	14.08
51646515	2012	5	23	5440	234.04	14.1
51646515	2012	5	23	5450	233.61	14.1
51646515	2012	5	23	5460	233.2	14.13
51646515	2012	5	23	5470	233.66	14.14

51646515	2012	5	23	5480	233.12	14.14
51646515	2012	5	23	5490	232.9	14.15
51646515	2012	5	23	5500	232.77	14.17
51646515	2012	5	23	5510	232.84	14.17
51646515	2012	5	23	5520	232.84	14.19
51646515	2012	5	23	5530	232.76	14.2
51646515	2012	5	23	5540	232.79	14.21
51646515	2012	5	23	5550	233.01	14.21
51646515	2012	5	23	5560	233.06	14.22
51646515	2012	5	23	5570	233.02	14.22
51646515	2012	5	23	5580	233.25	14.25
51646515	2012	5	23	5590	233.38	14.26
51646515	2012	5	23	5600	233.21	14.27
51646515	2012	5	23	5610	232.85	14.27
51646515	2012	5	23	5620	232.43	14.28
51646515	2012	5	23	5630	231.97	14.28
51646515	2012	5	23	5640	231.74	14.28
51646515	2012	5	23	5650	231.45	14.3
51646515	2012	5	23	5660	231.33	14.3
51646515	2012	5	23	5670	231.32	14.32
51646515	2012	5	23	5680	231.45	14.32
51646515	2012	5	23	5690	231.63	14.32
51646515	2012	5	23	5700	231.78	14.33
51646515	2012	5	23	5710	231.79	14.33
51646515	2012	5	23	5720	231.67	14.33
51646515	2012	5	23	5730	231.52	14.33
51646515	2012	5	23	5740	231.44	14.34
51646515	2012	5	23	5750	231.5	14.34
51646515	2012	5	23	5760	231.44	14.35
51646515	2012	5	23	5770	231.36	14.35
51646515	2012	5	23	5780	231.47	14.35
51646515	2012	5	23	5790	231.49	14.35
51646515	2012	5	23	5800	231.56	14.35
51646515	2012	5	23	5810	231.51	14.36
51646515	2012	5	23	5820	231.24	14.36
51646515	2012	5	23	5830	231.5	14.36
51646515	2012	5	23	5840	231.53	14.37
51646515	2012	5	23	5850	231.49	14.37
51646515	2012	5	23	5860	231.55	14.38
51646515	2012	5	23	5870	231.09	14.38
51646515	2012	5	23	5880	230.59	14.38
51646515	2012	5	23	5890	230.03	14.39
51646515	2012	5	23	5900	229.54	14.39
51646515	2012	5	23	5910	229.02	14.39
51646515	2012	5	23	5920	228.67	14.39
51646515	2012	5	23	5930	228.27	14.39
51646515	2012	5	23	5940	227.95	14.39

51646515	2012	5	23	5950	227.8	14.39
51646515	2012	5	23	5960	227.24	14.4
51646515	2012	5	23	5970	226.94	14.4
51646515	2012	5	23	5980	227.11	14.41
51646515	2012	5	23	5990	227.13	14.42
51646515	2012	5	23	6000	227	14.42
51646515	2012	5	23	6010	226.81	14.42
51646515	2012	5	23	6020	226.62	14.42
51646515	2012	5	23	6030	226.73	14.42
51646515	2012	5	23	6040	226.68	14.44
51646515	2012	5	23	6050	226.65	14.44
51646515	2012	5	23	6060	226.59	14.43
51646515	2012	5	23	6070	226.6	14.43
51646515	2012	5	23	6080	226.49	14.43
51646515	2012	5	23	6090	225.92	14.44
51646515	2012	5	23	6100	224.98	14.43
51646515	2012	5	23	6110	224.02	14.44
51646515	2012	5	23	6120	223.04	14.44
51646515	2012	5	23	6130	222.07	14.44
51646515	2012	5	23	6140	221.29	14.44
51646515	2012	5	23	6150	220.28	14.43
51646515	2012	5	23	6160	218.96	14.43
51646515	2012	5	23	6170	217.32	14.42
51646515	2012	5	23	6180	216.16	14.42
51646515	2012	5	23	6190	215.12	14.42
51646515	2012	5	23	6200	213.45	14.42
51646515	2012	5	23	6210	212.03	14.42
51646515	2012	5	23	6220	211.38	14.43
51646515	2012	5	23	6230	211.66	14.46
51646515	2012	5	23	6240	211.4	14.49
51646515	2012	5	23	6250	211.61	14.5
51646515	2012	5	23	6260	210.77	14.51
51646515	2012	5	23	6270	210.19	14.54
51646515	2012	5	23	6280	209.43	14.57
51646515	2012	5	23	6290	208.67	14.57
51646515	2012	5	23	6300	207.99	14.59
51646515	2012	5	23	6310	206.87	14.6
51646515	2012	5	23	6320	206.09	14.61
51646515	2012	5	23	6330	205.35	14.63
51646515	2012	5	23	6340	205	14.64
51646515	2012	5	23	6350	204.81	14.67
51646515	2012	5	23	6360	204.84	14.68
51646515	2012	5	23	6370	205.07	14.71
51646515	2012	5	23	6380	205.01	14.73
51646515	2012	5	23	6390	204.39	14.73
51646515	2012	5	23	6400	204.05	14.73
51646515	2012	5	23	6410	203.49	14.75

51646515	2012	5	23	6420	202.03	14.75
51646515	2012	5	23	6430	201.21	14.78
51646515	2012	5	23	6440	200.52	14.79
51646515	2012	5	23	6450	200.16	14.8
51646515	2012	5	23	6460	199.58	14.8
51646515	2012	5	23	6470	198.71	14.81
51646515	2012	5	23	6480	197.53	14.82
51646515	2012	5	23	6490	196.53	14.83
51646515	2012	5	23	6500	195.93	14.85
51646515	2012	5	23	6510	194.99	14.84
51646515	2012	5	23	6520	194.69	14.9
51646515	2012	5	23	6530	194.26	14.92
51646515	2012	5	23	6540	193.49	14.92
51646515	2012	5	23	6550	192.93	14.93
51646515	2012	5	23	6560	192.38	14.94
51646515	2012	5	23	6570	191.96	14.96
51646515	2012	5	23	6580	191.68	14.97
51646515	2012	5	23	6590	191.31	14.98
51646515	2012	5	23	6600	190.81	14.99
51646515	2012	5	23	6610	190.15	15
51646515	2012	5	23	6620	189.55	15
51646515	2012	5	23	6630	189.16	15.02
51646515	2012	5	23	6640	188.39	15.03
51646515	2012	5	23	6650	186.97	15.04
51646515	2012	5	23	6660	186.33	15.05
51646515	2012	5	23	6670	185.48	15.06
51646515	2012	5	23	6680	184.1	15.07
51646515	2012	5	23	6690	183.17	15.08
51646515	2012	5	23	6700	182.31	15.1
51646515	2012	5	23	6710	181.69	15.12
51646515	2012	5	23	6720	180.82	15.12
51646515	2012	5	23	6730	179.82	15.14
51646515	2012	5	23	6740	179.31	15.14
51646515	2012	5	23	6750	179.07	15.15
51646515	2012	5	23	6760	178.17	15.18
51646515	2012	5	23	6770	177.54	15.21
51646515	2012	5	23	6780	177.1	15.25
51646515	2012	5	23	6790	176.78	15.28
51646515	2012	5	23	6800	176.52	15.32
51646515	2012	5	23	6810	175.99	15.33
51646515	2012	5	23	6820	175.5	15.35
51646515	2012	5	23	6830	175.33	15.37
51646515	2012	5	23	6840	174.61	15.4
51646515	2012	5	23	6850	173.73	15.42
51646515	2012	5	23	6860	173.68	15.45
51646515	2012	5	23	6870	173.73	15.49
51646515	2012	5	23	6880	173.53	15.51

51646515	2012	5	23	6890	173.16	15.53
51646515	2012	5	23	6900	173.07	15.57
51646515	2012	5	23	6910	173.61	15.58
51646515	2012	5	23	6920	173.38	15.61
51646515	2012	5	23	6930	173.38	15.62
51646515	2012	5	23	6940	173.4	15.64
51646515	2012	5	23	6950	173.38	15.67
51646515	2012	5	23	6960	173.22	15.67
51646515	2012	5	23	6970	172.92	15.71
51646515	2012	5	23	6980	172.87	15.72
51646515	2012	5	23	6990	172.8	15.73
51646515	2012	5	23	7000	172.24	15.75
51646515	2012	5	23	7010	172.14	15.77
51646515	2012	5	23	7020	171.55	15.78
51646515	2012	5	23	7030	170.23	15.82
51646515	2012	5	23	7040	169.36	15.82
51646515	2012	5	23	7050	168.65	15.85
51646515	2012	5	23	7060	166.93	15.85
51646515	2012	5	23	7070	165.32	15.88
51646515	2012	5	23	7080	164.91	15.94
51646515	2012	5	23	7090	165.06	15.96
51646515	2012	5	23	7100	164.51	15.96
51646515	2012	5	23	7110	164.45	16
51646515	2012	5	23	7120	164.07	16.03
51646515	2012	5	23	7130	163.5	16.06
51646515	2012	5	23	7140	163.1	16.1
51646515	2012	5	23	7150	162.69	16.11
51646515	2012	5	23	7160	162.04	16.12
51646515	2012	5	23	7170	161.96	16.14
51646515	2012	5	23	7180	161.38	16.15
51646515	2012	5	23	7190	159.49	16.17
51646515	2012	5	23	7200	158.25	16.19
51646515	2012	5	23	7210	157.47	16.23
51646515	2012	5	23	7220	156.69	16.25
51646515	2012	5	23	7230	155.61	16.26
51646515	2012	5	23	7240	155	16.28
51646515	2012	5	23	7250	154.29	16.3
51646515	2012	5	23	7260	152.73	16.32
51646515	2012	5	23	7270	151.35	16.35
51646515	2012	5	23	7280	151.07	16.39
51646515	2012	5	23	7290	149.74	16.42
51646515	2012	5	23	7300	148.13	16.46
51646515	2012	5	23	7310	146.65	16.48
51646515	2012	5	23	7320	145.67	16.54
51646515	2012	5	23	7330	144.96	16.58
51646515	2012	5	23	7340	144.57	16.62
51646515	2012	5	23	7350	144.55	16.66

51646515	2012	5	23	7360	144.62	16.69
51646515	2012	5	23	7370	144.09	16.72
51646515	2012	5	23	7380	143.68	16.77
51646515	2012	5	23	7390	143.52	16.85
51646515	2012	5	23	7400	143.23	17.01
51646515	2012	5	23	7410	143.24	17.04
51646515	2012	5	23	7420	143.67	17.07
51646515	2012	5	23	7430	143.63	17.09
51646515	2012	5	23	7440	143.42	17.12
51646515	2012	5	23	7450	143.21	17.14
51646515	2012	5	23	7460	143.19	17.17
51646515	2012	5	23	7470	142.97	17.19
51646515	2012	5	23	7480	143.28	17.21
51646515	2012	5	23	7490	143.48	17.24
51646515	2012	5	23	7500	142.72	17.26
51646515	2012	5	23	7510	142.54	17.29
51646515	2012	5	23	7520	142.79	17.42
51646515	2012	5	23	7530	143.57	17.46
51646515	2012	5	23	7540	143.61	17.49
51646515	2012	5	23	7550	143.99	17.51
51646515	2012	5	23	7560	144.47	17.54
51646515	2012	5	23	7570	145.37	17.58
51646515	2012	5	23	7580	145.93	17.6
51646515	2012	5	23	7590	145.81	17.62
51646515	2012	5	23	7600	145.78	17.64
51646515	2012	5	23	7610	145.48	17.67
51646515	2012	5	23	7620	144.87	17.67
51646515	2012	5	23	7630	144.82	17.71
51646515	2012	5	23	7640	143.88	17.74
51646515	2012	5	23	7650	143.31	17.75
51646515	2012	5	23	7660	142.36	17.76
51646515	2012	5	23	7670	140.89	17.78
51646515	2012	5	23	7680	140.3	17.82
51646515	2012	5	23	7690	139.15	17.86
51646515	2012	5	23	7700	139.33	17.9
51646515	2012	5	23	7710	138.51	17.95
51646515	2012	5	23	7720	138.21	18.01
51646515	2012	5	23	7730	138.34	18.04
51646515	2012	5	23	7740	138.49	18.08
51646515	2012	5	23	7750	138.31	18.12
51646515	2012	5	23	7760	138.16	18.16
51646515	2012	5	23	7770	138.22	18.18
51646515	2012	5	23	7780	137.04	18.21
51646515	2012	5	23	7790	136.39	18.23
51646515	2012	5	23	7800	136.03	18.27
51646515	2012	5	23	7810	134.78	18.3
51646515	2012	5	23	7820	132.92	18.34

51646515	2012	5	23	7830	132.06	18.38
51646515	2012	5	23	7840	130.76	18.4
51646515	2012	5	23	7850	130.34	18.42
51646515	2012	5	23	7860	129.6	18.46
51646515	2012	5	23	7870	128.92	18.49
51646515	2012	5	23	7880	128.65	18.53
51646515	2012	5	23	7890	127.52	18.56
51646515	2012	5	23	7900	126.52	18.59
51646515	2012	5	23	7910	125.94	18.62
51646515	2012	5	23	7920	125.65	18.64
51646515	2012	5	23	7930	125.02	18.67
51646515	2012	5	23	7940	125.07	18.7
51646515	2012	5	23	7950	124.9	18.73
51646515	2012	5	23	7960	124.28	18.75
51646515	2012	5	23	7970	123.37	18.78
51646515	2012	5	23	7980	121.9	18.81
51646515	2012	5	23	7990	120.85	18.84
51646515	2012	5	23	8000	120.71	18.94
51646515	2012	5	23	8010	119.79	19.03
51646515	2012	5	23	8020	119	19.1
51646515	2012	5	23	8030	118.7	19.2
51646515	2012	5	23	8040	118.73	19.31
51646515	2012	5	23	8050	119	19.37
51646515	2012	5	23	8060	118.98	19.46
51646515	2012	5	23	8070	118.91	19.53
51646515	2012	5	23	8080	118.85	19.61
51646515	2012	5	23	8090	117.39	19.71
51646515	2012	5	23	8100	116.15	19.78
51646515	2012	5	23	8110	115.07	19.86
51646515	2012	5	23	8120	113.7	19.92
51646515	2012	5	23	8130	112.97	19.97
51646515	2012	5	23	8140	112.09	20.03
51646515	2012	5	23	8150	111.59	20.21
51646515	2012	5	23	8160	112.03	20.23
51646515	2012	5	23	8170	112.3	20.29
51646515	2012	5	23	8180	111.67	20.34
51646515	2012	5	23	8190	110.47	20.38
51646515	2012	5	23	8200	109.38	20.44
51646515	2012	5	23	8210	107.97	20.5
51646515	2012	5	23	8220	107.11	20.53
51646515	2012	5	23	8230	106.47	20.57
51646515	2012	5	23	8240	105.87	20.6
51646515	2012	5	23	8250	104.52	20.66
51646515	2012	5	23	8260	103.09	20.72
51646515	2012	5	23	8270	101.89	20.78
51646515	2012	5	23	8280	101.74	20.82
51646515	2012	5	23	8290	101.66	20.86

51646515	2012	5	23	8300	101.18	20.92
51646515	2012	5	23	8310	100.43	20.96
51646515	2012	5	23	8320	98.79	21.01
51646515	2012	5	23	8330	97.89	21.05
51646515	2012	5	23	8340	97.17	21.1
51646515	2012	5	23	8350	96.93	21.14
51646515	2012	5	23	8360	96.04	21.19
51646515	2012	5	23	8370	95.58	21.25
51646515	2012	5	23	8380	95	21.28
51646515	2012	5	23	8390	94.33	21.32
51646515	2012	5	23	8400	94.12	21.35
51646515	2012	5	23	8410	93.42	21.4
51646515	2012	5	23	8420	93.22	21.43
51646515	2012	5	23	8430	92.81	21.48
51646515	2012	5	23	8440	91.8	21.53
51646515	2012	5	23	8450	91.58	21.61
51646515	2012	5	23	8460	90.8	21.67
51646515	2012	5	23	8470	90.44	21.73
51646515	2012	5	23	8480	90.42	21.82
51646515	2012	5	23	8490	90.82	21.86
51646515	2012	5	23	8500	91.31	21.9
51646515	2012	5	23	8510	91.49	21.94
51646515	2012	5	23	8520	91.39	22.02
51646515	2012	5	23	8530	91.33	22.07
51646515	2012	5	23	8540	91.2	22.1
51646515	2012	5	23	8550	91.47	22.13
51646515	2012	5	23	8560	91.19	22.19
51646515	2012	5	23	8570	90.75	22.24
51646515	2012	5	23	8580	91	22.28
51646515	2012	5	23	8590	91.77	22.32
51646515	2012	5	23	8600	92.03	22.34
51646515	2012	5	23	8610	92.53	22.35
51646515	2012	5	23	8620	93.74	22.37
51646515	2012	5	23	8630	94.73	22.36
51646515	2012	5	23	8640	94.98	22.36
51646515	2012	5	23	8650	95.07	22.38
51646515	2012	5	23	8660	95.48	22.39
51646515	2012	5	23	8670	96.18	22.39
51646515	2012	5	23	8680	96.42	22.39
51646515	2012	5	23	8690	96.32	22.37
51646515	2012	5	23	8700	96.5	22.34
51646515	2012	5	23	8710	96.77	22.31
51646515	2012	5	23	8720	96.78	22.3
51646515	2012	5	23	8730	96.72	22.28
51646515	2012	5	23	8740	96.31	22.28
51646515	2012	5	23	8750	95.69	22.26
51646515	2012	5	23	8760	95.49	22.25

51646515	2012	5	23	8770	94.58	22.25
51646515	2012	5	23	8780	93.88	22.25
51646515	2012	5	23	8790	92.71	22.27
51646515	2012	5	23	8800	91.59	22.29
51646515	2012	5	23	8810	91.38	22.37
51646515	2012	5	23	8820	91.6	22.41
51646515	2012	5	23	8830	91.16	22.45
51646515	2012	5	23	8840	90.52	22.5
51646515	2012	5	23	8850	90.15	22.53
51646515	2012	5	23	8860	90.08	22.58
51646515	2012	5	23	8870	89.64	22.62
51646515	2012	5	23	8880	89.53	22.67
51646515	2012	5	23	8890	88.85	22.71
51646515	2012	5	23	8900	88.27	22.77
51646515	2012	5	23	8910	88.24	22.78
51646515	2012	5	23	8920	88.3	22.82
51646515	2012	5	23	8930	87.75	22.86
51646515	2012	5	23	8940	87.15	22.87
51646515	2012	5	23	8950	86.39	22.89
51646515	2012	5	23	8960	86.05	22.92
51646515	2012	5	23	8970	85.78	22.96
51646515	2012	5	23	8980	85.86	22.99
51646515	2012	5	23	8990	86.06	23
51646515	2012	5	23	9000	86.11	23.03
51646515	2012	5	23	9010	86.16	23.04
51646515	2012	5	23	9020	86.47	23.05
51646515	2012	5	23	9030	86.92	23.07
51646515	2012	5	23	9040	86.34	23.07
51646515	2012	5	23	9050	86.16	23.09
51646515	2012	5	23	9060	86.12	23.1
51646515	2012	5	23	9070	86.19	23.1
51646515	2012	5	23	9080	85.73	23.13
51646515	2012	5	23	9090	85.67	23.14
51646515	2012	5	23	9100	85.85	23.14
51646515	2012	5	23	9110	84.99	23.17
51646515	2012	5	23	9120	84.25	23.17
51646515	2012	5	23	9130	84.47	23.18
51646515	2012	5	23	9140	84.44	23.19
51646515	2012	5	23	9150	84.26	23.19
51646515	2012	5	23	9160	83.41	23.21
51646515	2012	5	23	9170	81.59	23.23
51646515	2012	5	23	9180	80.2	23.23
51646515	2012	5	23	9190	78.63	23.29
51646515	2012	5	23	9200	78.68	23.49
51646515	2012	5	23	9210	79.37	23.71
51646515	2012	5	23	9220	79.25	23.75
51646515	2012	5	23	9230	79.07	23.77

51646515	2012	5	23	9240	79	23.81
51646515	2012	5	23	9250	79.24	23.85
51646515	2012	5	23	9260	79.69	23.89
51646515	2012	5	23	9270	80.3	23.96
51646515	2012	5	23	9280	79.23	24.02
51646515	2012	5	23	9290	78.15	24.07
51646515	2012	5	23	9300	76.76	24.14
51646515	2012	5	23	9310	75.73	24.23
51646515	2012	5	23	9320	74.3	24.32
51646515	2012	5	23	9330	72.73	24.4
51646515	2012	5	23	9340	71.07	24.49
51646515	2012	5	23	9350	69.48	24.61
51646515	2012	5	23	9360	68.64	24.63
51646515	2012	5	23	9370	67.68	24.67
51646515	2012	5	23	9380	66.7	24.77
51646515	2012	5	23	9390	67.12	24.85
51646515	2012	5	23	9400	66.44	24.9
51646515	2012	5	23	9410	66.22	24.95
51646515	2012	5	23	9420	66.23	24.96
51646515	2012	5	23	9430	66.52	24.98
51646515	2012	5	23	9440	66.61	25.03
51646515	2012	5	23	9450	66.93	25.07
51646515	2012	5	23	9460	67.31	25.07
51646515	2012	5	23	9470	67.1	25.1
51646515	2012	5	23	9480	66.97	25.14
51646515	2012	5	23	9490	67.02	25.17
51646515	2012	5	23	9500	67.1	25.23
51646515	2012	5	23	9510	67.78	25.26
51646515	2012	5	23	9520	67.87	25.28
51646515	2012	5	23	9530	67.94	25.3
51646515	2012	5	23	9540	67.44	25.32
51646515	2012	5	23	9550	67.65	25.34
51646515	2012	5	23	9560	67.37	25.37
51646515	2012	5	23	9570	66.8	25.39
51646515	2012	5	23	9580	67.01	25.39
51646515	2012	5	23	9590	67.42	25.41
51646515	2012	5	23	9600	67.35	25.42
51646515	2012	5	23	9610	67.25	25.45
51646515	2012	5	23	9620	67.61	25.46
51646515	2012	5	23	9630	67.87	25.47
51646515	2012	5	23	9640	67.86	25.49
51646515	2012	5	23	9650	67.78	25.5
51646515	2012	5	23	9660	68.41	25.51
51646515	2012	5	23	9670	69.18	25.51
51646515	2012	5	23	9680	68.95	25.53
51646515	2012	5	23	9690	69.52	25.53
51646515	2012	5	23	9700	70.45	25.54

51646515	2012	5	23	9710	70.38	25.54
51646515	2012	5	23	9720	69.86	25.56
51646515	2012	5	23	9730	68.85	25.57
51646515	2012	5	23	9740	68.28	25.57
51646515	2012	5	23	9750	68.43	25.58
51646515	2012	5	23	9760	68.35	25.59
51646515	2012	5	23	9770	68.35	25.6
51646515	2012	5	23	9780	68.13	25.61
51646515	2012	5	23	9790	67.81	25.61
51646515	2012	5	23	9800	67.28	25.62
51646515	2012	5	23	9810	66.45	25.62
51646515	2012	5	23	9820	65.41	25.63
51646515	2012	5	23	9830	64.41	25.64
51646515	2012	5	23	9840	62.41	25.66
51646515	2012	5	23	9850	60.6	25.67
51646515	2012	5	23	9860	59.54	25.69
51646515	2012	5	23	9870	58.25	25.71
51646515	2012	5	23	9880	56.39	25.72
51646515	2012	5	23	9890	54.79	25.73
51646515	2012	5	23	9900	53.08	25.75
51646515	2012	5	23	9910	51.3	25.77
51646515	2012	5	23	9920	49.61	25.78
51646515	2012	5	23	9930	47.8	25.79
51646515	2012	5	23	9940	46.05	25.81
51646515	2012	5	23	9950	44.44	25.83
51646515	2012	5	23	9960	42.78	25.85
51646515	2012	5	23	9970	41.01	25.87
51646515	2012	5	23	9980	38.79	25.92
51646515	2012	5	23	9990	36.91	26
51646515	2012	5	23	10000	35.1	26.05
51646515	2012	5	23	10010	32.77	26.13
51646515	2012	5	23	10020	30.57	26.21
51646515	2012	5	23	10030	28.4	26.26
51646515	2012	5	23	10040	26.24	26.32
51646515	2012	5	23	10050	24.43	26.37
51646515	2012	5	23	10060	22.57	26.43
51646515	2012	5	23	10070	20.43	26.52
51646515	2012	5	23	10080	17.9	26.57
51646515	2012	5	23	10090	15.29	26.63
51646515	2012	5	23	10100	12.72	26.72
51646515	2012	5	23	10110	9.8	26.78
51646515	2012	5	23	10120	6.86	26.85
51646515	2012	5	23	10130	5.05	26.96
51646515	2012	5	23	10140	3.59	26.98
51646515	2012	5	23	10150	2.93	27.02
51646515	2012	5	23	10160	2.53	27.07
51646515	2012	5	23	10170	2.63	27.1

51646515	2012	5	23	10180	2.52	27.13
51646515	2012	5	23	10190	2.32	27.14
51646515	2012	5	23	10200	0.6	27.15
51646515	2012	5	23	10210	0.57	27.17
51646515	2012	5	23	10220	0.68	27.19
51646515	2012	5	23	10230	0.67	27.24
51646515	2012	5	23	10240	0.67	27.28
51646515	2012	5	23	10250	0.61	27.33
51646515	2012	5	23	10260	0.61	27.36
51646515	2012	5	23	10270	0.67	27.38
51646515	2012	5	23	10280	0.67	27.4
51646515	2012	5	23	10290	0.64	27.42
51646515	2012	5	23	10300	0.57	27.44
51646515	2012	5	23	10310	0.57	27.44
51646515	2012	5	23	10320	0.61	27.46
51646515	2012	5	23	10330	0.57	27.48
51646515	2012	5	23	10340	0.59	27.49
51659515	2012	5	23	0	0.99	27.86
51659515	2012	5	23	10	1.41	27.87
51659515	2012	5	23	20	2.43	27.86
51659515	2012	5	23	30	2.58	27.86
51659515	2012	5	23	40	2.43	27.85
51659515	2012	5	23	50	2.54	27.85
51659515	2012	5	23	60	2.51	27.85
51659515	2012	5	23	70	2.64	27.85
51659515	2012	5	23	80	2.61	27.84
51659515	2012	5	23	90	2.64	27.82
51659515	2012	5	23	100	3.51	27.81
51659515	2012	5	23	110	4.87	27.79
51659515	2012	5	23	120	5.26	27.78
51659515	2012	5	23	130	5.22	27.78
51659515	2012	5	23	140	5.17	27.77
51659515	2012	5	23	150	6.78	27.75
51659515	2012	5	23	160	8.08	27.74
51659515	2012	5	23	170	8.43	27.73
51659515	2012	5	23	180	8.55	27.72
51659515	2012	5	23	190	8.51	27.72
51659515	2012	5	23	200	8.66	27.71
51659515	2012	5	23	210	8.81	27.71
51659515	2012	5	23	220	8.69	27.71
51659515	2012	5	23	230	8.54	27.71
51659515	2012	5	23	240	8.25	27.71
51659515	2012	5	23	250	8.29	27.7
51659515	2012	5	23	260	8.55	27.7
51659515	2012	5	23	270	8.23	27.69
51659515	2012	5	23	280	8.14	27.69
51659515	2012	5	23	290	8.82	27.68

51659515	2012	5	23	300	8.98	27.67
51659515	2012	5	23	310	8.84	27.67
51659515	2012	5	23	320	8.82	27.67
51659515	2012	5	23	330	9.56	27.66
51659515	2012	5	23	340	10.53	27.64
51659515	2012	5	23	350	12.1	27.62
51659515	2012	5	23	360	13.71	27.6
51659515	2012	5	23	370	15.13	27.57
51659515	2012	5	23	380	16.62	27.56
51659515	2012	5	23	390	18.44	27.54
51659515	2012	5	23	400	20.28	27.52
51659515	2012	5	23	410	21.92	27.5
51659515	2012	5	23	420	24.15	27.47
51659515	2012	5	23	430	26.94	27.45
51659515	2012	5	23	440	29.05	27.42
51659515	2012	5	23	450	31.12	27.4
51659515	2012	5	23	460	33.12	27.38
51659515	2012	5	23	470	35.17	27.35
51659515	2012	5	23	480	37.31	27.32
51659515	2012	5	23	490	39.54	27.3
51659515	2012	5	23	500	41.78	27.28
51659515	2012	5	23	510	44.07	27.25
51659515	2012	5	23	520	46.58	27.2
51659515	2012	5	23	530	48.84	27.09
51659515	2012	5	23	540	49.82	26.94
51659515	2012	5	23	550	51.41	26.76
51659515	2012	5	23	560	52.91	26.67
51659515	2012	5	23	570	53.04	26.66
51659515	2012	5	23	580	52.63	26.65
51659515	2012	5	23	590	52.18	26.62
51659515	2012	5	23	600	52.44	26.58
51659515	2012	5	23	610	52.56	26.55
51659515	2012	5	23	620	52.77	26.5
51659515	2012	5	23	630	53.2	26.46
51659515	2012	5	23	640	53.59	26.43
51659515	2012	5	23	650	52.89	26.41
51659515	2012	5	23	660	52.74	26.38
51659515	2012	5	23	670	53.09	26.36
51659515	2012	5	23	680	53.28	26.34
51659515	2012	5	23	690	53.24	26.32
51659515	2012	5	23	700	53.13	26.31
51659515	2012	5	23	710	52.96	26.29
51659515	2012	5	23	720	52.91	26.26
51659515	2012	5	23	730	52.58	26.25
51659515	2012	5	23	740	51.81	26.23
51659515	2012	5	23	750	51.42	26.23
51659515	2012	5	23	760	51.72	26.21

51659515	2012	5	23	770	51.39	26.2
51659515	2012	5	23	780	51.26	26.18
51659515	2012	5	23	790	51.84	26.16
51659515	2012	5	23	800	52.17	26.12
51659515	2012	5	23	810	51.62	26.1
51659515	2012	5	23	820	51.87	26.08
51659515	2012	5	23	830	52.08	26.07
51659515	2012	5	23	840	52.06	26.05
51659515	2012	5	23	850	51.99	26.04
51659515	2012	5	23	860	52.06	26.03
51659515	2012	5	23	870	52.02	26.02
51659515	2012	5	23	880	51.95	26.01
51659515	2012	5	23	890	51.71	26
51659515	2012	5	23	900	51.71	25.99
51659515	2012	5	23	910	51.98	25.99
51659515	2012	5	23	920	52.13	25.97
51659515	2012	5	23	930	51.83	25.97
51659515	2012	5	23	940	51.74	25.96
51659515	2012	5	23	950	51.73	25.96
51659515	2012	5	23	960	51.75	25.95
51659515	2012	5	23	970	51.86	25.94
51659515	2012	5	23	980	51.85	25.93
51659515	2012	5	23	990	51.93	25.93
51659515	2012	5	23	1000	52.85	25.92
51659515	2012	5	23	1010	53.22	25.92
51659515	2012	5	23	1020	53.3	25.92
51659515	2012	5	23	1030	53.69	25.92
51659515	2012	5	23	1040	53.86	25.9
51659515	2012	5	23	1050	53.74	25.92
51659515	2012	5	23	1060	53.81	25.91
51659515	2012	5	23	1070	53.82	25.9
51659515	2012	5	23	1080	53.86	25.9
51659515	2012	5	23	1090	53.81	25.9
51659515	2012	5	23	1100	53.69	25.9
51659515	2012	5	23	1110	53.96	25.89
51659515	2012	5	23	1120	53.97	25.89
51659515	2012	5	23	1130	54.08	25.89
51659515	2012	5	23	1140	54.29	25.89
51659515	2012	5	23	1150	54.28	25.89
51659515	2012	5	23	1160	54.27	25.88
51659515	2012	5	23	1170	54.26	25.88
51659515	2012	5	23	1180	54.24	25.87
51659515	2012	5	23	1190	54.24	25.87
51659515	2012	5	23	1200	54.21	25.87
51659515	2012	5	23	1210	54.16	25.86
51659515	2012	5	23	1220	54.05	25.87
51659515	2012	5	23	1230	54.07	25.87

51659515	2012	5	23	1240	54.08	25.86
51659515	2012	5	23	1250	52.71	25.87
51659515	2012	5	23	1260	50.5	25.89
51659515	2012	5	23	1270	49.26	25.92
51659515	2012	5	23	1280	49.27	25.95
51659515	2012	5	23	1290	49.38	25.98
51659515	2012	5	23	1300	49.61	26
51659515	2012	5	23	1310	49.55	26.03
51659515	2012	5	23	1320	49.52	26.04
51659515	2012	5	23	1330	49.38	26.06
51659515	2012	5	23	1340	49.08	26.1
51659515	2012	5	23	1350	49.2	26.13
51659515	2012	5	23	1360	49.72	26.14
51659515	2012	5	23	1370	49.87	26.15
51659515	2012	5	23	1380	49.66	26.16
51659515	2012	5	23	1390	49.8	26.17
51659515	2012	5	23	1400	49.7	26.18
51659515	2012	5	23	1410	49.66	26.19
51659515	2012	5	23	1420	49.33	26.21
51659515	2012	5	23	1430	48.77	26.22
51659515	2012	5	23	1440	48.84	26.25
51659515	2012	5	23	1450	48.27	26.28
51659515	2012	5	23	1460	47.66	26.31
51659515	2012	5	23	1470	46.99	26.35
51659515	2012	5	23	1480	46.64	26.36
51659515	2012	5	23	1490	46.29	26.4
51659515	2012	5	23	1500	46.03	26.41
51659515	2012	5	23	1510	46.14	26.44
51659515	2012	5	23	1520	46.35	26.46
51659515	2012	5	23	1530	46.46	26.46
51659515	2012	5	23	1540	46.77	26.49
51659515	2012	5	23	1550	46.74	26.5
51659515	2012	5	23	1560	46.69	26.5
51659515	2012	5	23	1570	46.89	26.5
51659515	2012	5	23	1580	47.06	26.51
51659515	2012	5	23	1590	47.02	26.53
51659515	2012	5	23	1600	47.09	26.53
51659515	2012	5	23	1610	47.14	26.54
51659515	2012	5	23	1620	47.17	26.55
51659515	2012	5	23	1630	47.19	26.55
51659515	2012	5	23	1640	47.05	26.56
51659515	2012	5	23	1650	46.92	26.56
51659515	2012	5	23	1660	46.91	26.57
51659515	2012	5	23	1670	46.52	26.58
51659515	2012	5	23	1680	46.21	26.6
51659515	2012	5	23	1690	45.5	26.6
51659515	2012	5	23	1700	45.24	26.61

51659515	2012	5	23	1710	45.16	26.62
51659515	2012	5	23	1720	45.11	26.64
51659515	2012	5	23	1730	45.1	26.63
51659515	2012	5	23	1740	45.31	26.62
51659515	2012	5	23	1750	45.65	26.64
51659515	2012	5	23	1760	45.61	26.64
51659515	2012	5	23	1770	45.57	26.64
51659515	2012	5	23	1780	46	26.64
51659515	2012	5	23	1790	45.98	26.64
51659515	2012	5	23	1800	46.01	26.65
51659515	2012	5	23	1810	46.4	26.65
51659515	2012	5	23	1820	46.48	26.65
51659515	2012	5	23	1830	46.52	26.66
51659515	2012	5	23	1840	46.44	26.66
51659515	2012	5	23	1850	46.63	26.66
51659515	2012	5	23	1860	47.13	26.66
51659515	2012	5	23	1870	47.54	26.66
51659515	2012	5	23	1880	48.15	26.67
51659515	2012	5	23	1890	47.98	26.67
51659515	2012	5	23	1900	47.88	26.67
51659515	2012	5	23	1910	48.06	26.67
51659515	2012	5	23	1920	47.54	26.67
51659515	2012	5	23	1930	47.43	26.67
51659515	2012	5	23	1940	47.35	26.68
51659515	2012	5	23	1950	47.52	26.67
51659515	2012	5	23	1960	47.78	26.68
51659515	2012	5	23	1970	48.13	26.68
51659515	2012	5	23	1980	48.38	26.67
51659515	2012	5	23	1990	48.35	26.68
51659515	2012	5	23	2000	48.28	26.67
51659515	2012	5	23	2010	47.85	26.68
51659515	2012	5	23	2020	46.6	26.7
51659515	2012	5	23	2030	46.04	26.7
51659515	2012	5	23	2040	45.46	26.7
51659515	2012	5	23	2050	45.3	26.71
51659515	2012	5	23	2060	45.57	26.71
51659515	2012	5	23	2070	46.09	26.71
51659515	2012	5	23	2080	46.85	26.71
51659515	2012	5	23	2090	47.39	26.71
51659515	2012	5	23	2100	47.67	26.71
51659515	2012	5	23	2110	48.75	26.69
51659515	2012	5	23	2120	50	26.7
51659515	2012	5	23	2130	50.68	26.68
51659515	2012	5	23	2140	51.06	26.68
51659515	2012	5	23	2150	51.04	26.68
51659515	2012	5	23	2160	51	26.68
51659515	2012	5	23	2170	50.95	26.68

51659515	2012	5	23	2180	51.26	26.68
51659515	2012	5	23	2190	51.64	26.68
51659515	2012	5	23	2200	51.72	26.67
51659515	2012	5	23	2210	52	26.67
51659515	2012	5	23	2220	52.27	26.67
51659515	2012	5	23	2230	52.46	26.66
51659515	2012	5	23	2240	52.59	26.66
51659515	2012	5	23	2250	52.44	26.65
51659515	2012	5	23	2260	52.4	26.64
51659515	2012	5	23	2270	52.04	26.65
51659515	2012	5	23	2280	51.82	26.65
51659515	2012	5	23	2290	51.86	26.66
51659515	2012	5	23	2300	52.01	26.65
51659515	2012	5	23	2310	52.13	26.65
51659515	2012	5	23	2320	52.21	26.65
51659515	2012	5	23	2330	52.2	26.65
51659515	2012	5	23	2340	51.98	26.64
51659515	2012	5	23	2350	51.59	26.66
51659515	2012	5	23	2360	51.49	26.65
51659515	2012	5	23	2370	51.62	26.66
51659515	2012	5	23	2380	52.3	26.65
51659515	2012	5	23	2390	53	26.65
51659515	2012	5	23	2400	53.04	26.64
51659515	2012	5	23	2410	52.8	26.65
51659515	2012	5	23	2420	52.88	26.64
51659515	2012	5	23	2430	52.64	26.65
51659515	2012	5	23	2440	52.55	26.65
51659515	2012	5	23	2450	52.47	26.66
51659515	2012	5	23	2460	52.36	26.66
51659515	2012	5	23	2470	52.48	26.66
51659515	2012	5	23	2480	51.53	26.67
51659515	2012	5	23	2490	51.06	26.67
51659515	2012	5	23	2500	51.02	26.67
51659515	2012	5	23	2510	51.72	26.67
51659515	2012	5	23	2520	52.4	26.67
51659515	2012	5	23	2530	52.86	26.67
51659515	2012	5	23	2540	53.71	26.66
51659515	2012	5	23	2550	54.83	26.64
51659515	2012	5	23	2560	56.69	26.61
51659515	2012	5	23	2570	58.83	26.55
51659515	2012	5	23	2580	60.97	26.51
51659515	2012	5	23	2590	62.46	26.46
51659515	2012	5	23	2600	64.67	26.34
51659515	2012	5	23	2610	66.66	26.28
51659515	2012	5	23	2620	68.98	26.27
51659515	2012	5	23	2630	70.95	26.24
51659515	2012	5	23	2640	72.04	26.21

51659515	2012	5	23	2650	72.71	26.16
51659515	2012	5	23	2660	72.95	26.12
51659515	2012	5	23	2670	73	26.11
51659515	2012	5	23	2680	72.89	26.09
51659515	2012	5	23	2690	73.8	26.06
51659515	2012	5	23	2700	75.26	25.99
51659515	2012	5	23	2710	76.93	25.95
51659515	2012	5	23	2720	78.41	25.93
51659515	2012	5	23	2730	79.86	25.86
51659515	2012	5	23	2740	80.94	25.82
51659515	2012	5	23	2750	82.17	25.81
51659515	2012	5	23	2760	83.49	25.79
51659515	2012	5	23	2770	84.03	25.78
51659515	2012	5	23	2780	84.29	25.75
51659515	2012	5	23	2790	84.69	25.75
51659515	2012	5	23	2800	85.42	25.7
51659515	2012	5	23	2810	85.87	25.7
51659515	2012	5	23	2820	85.8	25.69
51659515	2012	5	23	2830	85.71	25.68
51659515	2012	5	23	2840	85.68	25.67
51659515	2012	5	23	2850	85.52	25.67
51659515	2012	5	23	2860	85.64	25.67
51659515	2012	5	23	2870	85.81	25.65
51659515	2012	5	23	2880	85.78	25.64
51659515	2012	5	23	2890	85.82	25.63
51659515	2012	5	23	2900	85.66	25.58
51659515	2012	5	23	2910	85.59	25.57
51659515	2012	5	23	2920	86.44	25.56
51659515	2012	5	23	2930	87.48	25.54
51659515	2012	5	23	2940	88.07	25.48
51659515	2012	5	23	2950	88.86	25.21
51659515	2012	5	23	2960	89.32	25.08
51659515	2012	5	23	2970	89.32	25.07
51659515	2012	5	23	2980	89.2	25.07
51659515	2012	5	23	2990	89.3	25.04
51659515	2012	5	23	3000	89.31	25
51659515	2012	5	23	3010	89.37	24.98
51659515	2012	5	23	3020	89.33	24.93
51659515	2012	5	23	3030	89.26	24.86
51659515	2012	5	23	3040	89.14	24.82
51659515	2012	5	23	3050	89.17	24.79
51659515	2012	5	23	3060	89.03	24.74
51659515	2012	5	23	3070	89.32	24.6
51659515	2012	5	23	3080	89.8	24.48
51659515	2012	5	23	3090	90.18	24.41
51659515	2012	5	23	3100	90.88	24.41
51659515	2012	5	23	3110	92.01	24.32

51659515	2012	5	23	3120	93.47	24.29
51659515	2012	5	23	3130	94.08	24.25
51659515	2012	5	23	3140	94.19	24.19
51659515	2012	5	23	3150	94.62	24.12
51659515	2012	5	23	3160	96.04	24.1
51659515	2012	5	23	3170	97.98	24.07
51659515	2012	5	23	3180	99.02	24
51659515	2012	5	23	3190	100.31	23.91
51659515	2012	5	23	3200	101.42	23.74
51659515	2012	5	23	3210	102.63	23.66
51659515	2012	5	23	3220	104.13	23.57
51659515	2012	5	23	3230	105.22	23.5
51659515	2012	5	23	3240	106.37	23.38
51659515	2012	5	23	3250	107.6	23.32
51659515	2012	5	23	3260	109.23	23.06
51659515	2012	5	23	3270	111.09	22.86
51659515	2012	5	23	3280	112.68	22.78
51659515	2012	5	23	3290	114.06	22.8
51659515	2012	5	23	3300	115.51	22.79
51659515	2012	5	23	3310	116.73	22.75
51659515	2012	5	23	3320	118.39	22.7
51659515	2012	5	23	3330	119.21	22.62
51659515	2012	5	23	3340	120	22.59
51659515	2012	5	23	3350	120.85	22.53
51659515	2012	5	23	3360	121.33	22.46
51659515	2012	5	23	3370	122.39	22.39
51659515	2012	5	23	3380	123.51	22.27
51659515	2012	5	23	3390	124.44	22.08
51659515	2012	5	23	3400	124.7	22
51659515	2012	5	23	3410	124.82	21.95
51659515	2012	5	23	3420	124.85	21.82
51659515	2012	5	23	3430	124.85	21.74
51659515	2012	5	23	3440	125.03	21.69
51659515	2012	5	23	3450	125.29	21.63
51659515	2012	5	23	3460	126.45	21.53
51659515	2012	5	23	3470	127.23	21.38
51659515	2012	5	23	3480	127.89	21.22
51659515	2012	5	23	3490	128.06	21.14
51659515	2012	5	23	3500	127.67	21.05
51659515	2012	5	23	3510	127.32	20.96
51659515	2012	5	23	3520	126.88	20.8
51659515	2012	5	23	3530	126.7	20.75
51659515	2012	5	23	3540	126.89	20.61
51659515	2012	5	23	3550	127.48	20.53
51659515	2012	5	23	3560	128.75	20.39
51659515	2012	5	23	3570	130.47	20.23
51659515	2012	5	23	3580	132.21	20.13

51659515	2012	5	23	3590	132.87	20.08
51659515	2012	5	23	3600	132.88	20.05
51659515	2012	5	23	3610	132.69	20.03
51659515	2012	5	23	3620	132.42	19.96
51659515	2012	5	23	3630	132.18	19.95
51659515	2012	5	23	3640	132.38	19.86
51659515	2012	5	23	3650	132.87	19.81
51659515	2012	5	23	3660	133.89	19.71
51659515	2012	5	23	3670	134.63	19.64
51659515	2012	5	23	3680	136	19.5
51659515	2012	5	23	3690	136.9	19.44
51659515	2012	5	23	3700	137.55	19.43
51659515	2012	5	23	3710	138.56	19.28
51659515	2012	5	23	3720	138.78	19.26
51659515	2012	5	23	3730	138.6	19.24
51659515	2012	5	23	3740	138.79	19.22
51659515	2012	5	23	3750	138.5	19.21
51659515	2012	5	23	3760	138.64	19.17
51659515	2012	5	23	3770	139.2	19.14
51659515	2012	5	23	3780	139.25	19.1
51659515	2012	5	23	3790	139.35	19.07
51659515	2012	5	23	3800	139.42	19.04
51659515	2012	5	23	3810	139.28	19
51659515	2012	5	23	3820	139.12	18.91
51659515	2012	5	23	3830	139.07	18.86
51659515	2012	5	23	3840	138.91	18.84
51659515	2012	5	23	3850	139.23	18.8
51659515	2012	5	23	3860	139.95	18.76
51659515	2012	5	23	3870	140.48	18.73
51659515	2012	5	23	3880	141.16	18.7
51659515	2012	5	23	3890	141.42	18.67
51659515	2012	5	23	3900	141.7	18.64
51659515	2012	5	23	3910	142.09	18.62
51659515	2012	5	23	3920	142.21	18.6
51659515	2012	5	23	3930	142.22	18.59
51659515	2012	5	23	3940	142.21	18.55
51659515	2012	5	23	3950	141.91	18.53
51659515	2012	5	23	3960	141.43	18.5
51659515	2012	5	23	3970	141.37	18.47
51659515	2012	5	23	3980	141.22	18.46
51659515	2012	5	23	3990	140.37	18.44
51659515	2012	5	23	4000	139.76	18.42
51659515	2012	5	23	4010	139.41	18.41
51659515	2012	5	23	4020	139.21	18.39
51659515	2012	5	23	4030	139.72	18.38
51659515	2012	5	23	4040	139.66	18.37
51659515	2012	5	23	4050	139.48	18.36

51659515	2012	5	23	4060	139.77	18.35
51659515	2012	5	23	4070	139.92	18.35
51659515	2012	5	23	4080	140.25	18.33
51659515	2012	5	23	4090	140.04	18.32
51659515	2012	5	23	4100	139.77	18.32
51659515	2012	5	23	4110	140.11	18.3
51659515	2012	5	23	4120	140.35	18.28
51659515	2012	5	23	4130	140.05	18.28
51659515	2012	5	23	4140	140.57	18.28
51659515	2012	5	23	4150	140.71	18.27
51659515	2012	5	23	4160	140.66	18.27
51659515	2012	5	23	4170	140.6	18.26
51659515	2012	5	23	4180	140.21	18.27
51659515	2012	5	23	4190	139.87	18.25
51659515	2012	5	23	4200	140.34	18.25
51659515	2012	5	23	4210	140.52	18.25
51659515	2012	5	23	4220	140.58	18.24
51659515	2012	5	23	4230	140.61	18.22
51659515	2012	5	23	4240	140.52	18.23
51659515	2012	5	23	4250	140.3	18.22
51659515	2012	5	23	4260	139.56	18.22
51659515	2012	5	23	4270	139.51	18.21
51659515	2012	5	23	4280	139.77	18.21
51659515	2012	5	23	4290	140.32	18.21
51659515	2012	5	23	4300	140.4	18.21
51659515	2012	5	23	4310	140.51	18.2
51659515	2012	5	23	4320	140.49	18.2
51659515	2012	5	23	4330	139.8	18.2
51659515	2012	5	23	4340	138.36	18.2
51659515	2012	5	23	4350	137.1	18.21
51659515	2012	5	23	4360	136.19	18.21
51659515	2012	5	23	4370	135.16	18.23
51659515	2012	5	23	4380	133.51	18.25
51659515	2012	5	23	4390	131.94	18.27
51659515	2012	5	23	4400	130.94	18.29
51659515	2012	5	23	4410	129.79	18.32
51659515	2012	5	23	4420	128.43	18.36
51659515	2012	5	23	4430	127.03	18.4
51659515	2012	5	23	4440	124.96	18.44
51659515	2012	5	23	4450	123.39	18.49
51659515	2012	5	23	4460	121.89	18.56
51659515	2012	5	23	4470	120.57	18.69
51659515	2012	5	23	4480	119.26	18.85
51659515	2012	5	23	4490	116.89	18.96
51659515	2012	5	23	4500	114.4	19.06
51659515	2012	5	23	4510	113.34	19.17
51659515	2012	5	23	4520	114.17	19.33

51659515	2012	5	23	4530	114.46	19.42
51659515	2012	5	23	4540	113.81	19.55
51659515	2012	5	23	4550	112.82	19.74
51659515	2012	5	23	4560	113.43	19.89
51659515	2012	5	23	4570	113.17	19.93
51659515	2012	5	23	4580	113.01	19.99
51659515	2012	5	23	4590	113.05	20.04
51659515	2012	5	23	4600	112.59	20.1
51659515	2012	5	23	4610	111.8	20.18
51659515	2012	5	23	4620	110.79	20.23
51659515	2012	5	23	4630	109.6	20.28
51659515	2012	5	23	4640	108.03	20.36
51659515	2012	5	23	4650	106.91	20.46
51659515	2012	5	23	4660	105.43	20.53
51659515	2012	5	23	4670	103.55	20.59
51659515	2012	5	23	4680	102.39	20.64
51659515	2012	5	23	4690	101.11	20.71
51659515	2012	5	23	4700	98.98	20.75
51659515	2012	5	23	4710	97.86	20.84
51659515	2012	5	23	4720	97.75	20.9
51659515	2012	5	23	4730	97.53	20.94
51659515	2012	5	23	4740	96.65	21.03
51659515	2012	5	23	4750	94.58	21.14
51659515	2012	5	23	4760	92.05	21.28
51659515	2012	5	23	4770	90.07	21.36
51659515	2012	5	23	4780	87.85	21.49
51659515	2012	5	23	4790	85.96	21.59
51659515	2012	5	23	4800	84.92	21.7
51659515	2012	5	23	4810	83.1	21.91
51659515	2012	5	23	4820	81.95	22.17
51659515	2012	5	23	4830	82.2	22.51
51659515	2012	5	23	4840	82.05	22.59
51659515	2012	5	23	4850	81.01	22.66
51659515	2012	5	23	4860	80.84	22.76
51659515	2012	5	23	4870	80.84	22.96
51659515	2012	5	23	4880	80.85	23.06
51659515	2012	5	23	4890	80.82	23.14
51659515	2012	5	23	4900	80.69	23.2
51659515	2012	5	23	4910	79.59	23.3
51659515	2012	5	23	4920	77.9	23.46
51659515	2012	5	23	4930	76.75	23.6
51659515	2012	5	23	4940	74.74	23.75
51659515	2012	5	23	4950	74.17	23.82
51659515	2012	5	23	4960	73.03	23.92
51659515	2012	5	23	4970	71.27	24.02
51659515	2012	5	23	4980	68.96	24.15
51659515	2012	5	23	4990	66.88	24.22

51659515	2012	5	23	5000	65.25	24.28
51659515	2012	5	23	5010	62.99	24.37
51659515	2012	5	23	5020	61.01	24.46
51659515	2012	5	23	5030	59.06	24.57
51659515	2012	5	23	5040	57.24	24.76
51659515	2012	5	23	5050	56.13	24.82
51659515	2012	5	23	5060	54.65	24.87
51659515	2012	5	23	5070	53.66	24.95
51659515	2012	5	23	5080	52.79	25.02
51659515	2012	5	23	5090	51.21	25.15
51659515	2012	5	23	5100	50.08	25.25
51659515	2012	5	23	5110	49.39	25.32
51659515	2012	5	23	5120	49.3	25.39
51659515	2012	5	23	5130	49.6	25.42
51659515	2012	5	23	5140	49.73	25.44
51659515	2012	5	23	5150	49.48	25.48
51659515	2012	5	23	5160	49.21	25.54
51659515	2012	5	23	5170	48.99	25.61
51659515	2012	5	23	5180	48.75	25.67
51659515	2012	5	23	5190	49.11	25.73
51659515	2012	5	23	5200	49.1	25.81
51659515	2012	5	23	5210	49.4	25.85
51659515	2012	5	23	5220	49.47	25.9
51659515	2012	5	23	5230	49.49	25.94
51659515	2012	5	23	5240	50.09	26.01
51659515	2012	5	23	5250	50.43	26.04
51659515	2012	5	23	5260	50.43	26.08
51659515	2012	5	23	5270	50.32	26.1
51659515	2012	5	23	5280	50.27	26.14
51659515	2012	5	23	5290	49.55	26.17
51659515	2012	5	23	5300	48.15	26.22
51659515	2012	5	23	5310	47.1	26.26
51659515	2012	5	23	5320	46.76	26.29
51659515	2012	5	23	5330	46.53	26.31
51659515	2012	5	23	5340	46.07	26.33
51659515	2012	5	23	5350	45.95	26.35
51659515	2012	5	23	5360	45.39	26.37
51659515	2012	5	23	5370	45.19	26.39
51659515	2012	5	23	5380	44.86	26.41
51659515	2012	5	23	5390	44.57	26.45
51659515	2012	5	23	5400	44.45	26.46
51659515	2012	5	23	5410	44.5	26.48
51659515	2012	5	23	5420	44.81	26.49
51659515	2012	5	23	5430	45.25	26.5
51659515	2012	5	23	5440	44.91	26.52
51659515	2012	5	23	5450	44.95	26.53
51659515	2012	5	23	5460	44.58	26.55

51659515	2012	5	23	5470	44.49	26.55
51659515	2012	5	23	5480	43.97	26.57
51659515	2012	5	23	5490	42.2	26.6
51659515	2012	5	23	5500	41.74	26.62
51659515	2012	5	23	5510	41.18	26.63
51659515	2012	5	23	5520	39.76	26.64
51659515	2012	5	23	5530	38.43	26.66
51659515	2012	5	23	5540	36.78	26.69
51659515	2012	5	23	5550	34.95	26.71
51659515	2012	5	23	5560	33.51	26.73
51659515	2012	5	23	5570	31.73	26.75
51659515	2012	5	23	5580	29.99	26.77
51659515	2012	5	23	5590	29.03	26.79
51659515	2012	5	23	5600	27.36	26.81
51659515	2012	5	23	5610	26.72	26.82
51659515	2012	5	23	5620	26.73	26.84
51659515	2012	5	23	5630	26.83	26.84
51659515	2012	5	23	5640	26.11	26.85
51659515	2012	5	23	5650	24.39	26.88
51659515	2012	5	23	5660	22.32	26.92
51659515	2012	5	23	5670	20.8	26.94
51659515	2012	5	23	5680	19.15	26.96
51659515	2012	5	23	5690	18.05	26.96
51659515	2012	5	23	5700	17.06	26.98
51659515	2012	5	23	5710	15.43	27.02
51659515	2012	5	23	5720	13.25	27.05
51659515	2012	5	23	5730	11.75	27.08
51659515	2012	5	23	5740	9.82	27.11
51659515	2012	5	23	5750	7.52	27.15
51659515	2012	5	23	5760	5.9	27.19
51659515	2012	5	23	5770	4.44	27.24
51659515	2012	5	23	5780	3.12	27.27
51659515	2012	5	23	5790	2.87	27.31
51659515	2012	5	23	5800	2.43	27.33
51659515	2012	5	23	5810	2.51	27.38
51659515	2012	5	23	5820	2.67	27.39
51659515	2012	5	23	5830	2.86	27.4
51659515	2012	5	23	5840	2.6	27.43
51659515	2012	5	23	5850	1.63	27.44
51659515	2012	5	23	5860	0.59	27.43
51659515	2012	5	23	5870	0.62	27.43
51659515	2012	5	23	5880	0.6	27.46
51659515	2012	5	23	5890	0.58	27.48
51659515	2012	5	23	5900	0.6	27.53
51659515	2012	5	23	5910	0.61	27.57
51659515	2012	5	23	5920	0.63	27.61
51659515	2012	5	23	5930	0.62	27.63

51659515	2012	5	23	5940	0.58	27.66
51659515	2012	5	23	5950	0.63	27.67
51659515	2012	5	23	5960	0.6	27.67
51659515	2012	5	23	5970	0.58	27.69
51659515	2012	5	23	5980	0.51	27.71
51659515	2012	5	23	5990	0.5	27.73
51659515	2012	5	23	6000	0.52	27.73
51667855	2012	5	23	0	1.11	28
51667855	2012	5	23	10	1.69	28
51667855	2012	5	23	20	2.04	27.99
51667855	2012	5	23	30	2.39	27.98
51667855	2012	5	23	40	2.51	27.97
51667855	2012	5	23	50	2.42	27.96
51667855	2012	5	23	60	2.43	27.96
51667855	2012	5	23	70	2.82	27.92
51667855	2012	5	23	80	2.65	27.89
51667855	2012	5	23	90	2.86	27.87
51667855	2012	5	23	100	3.5	27.85
51667855	2012	5	23	110	5.2	27.84
51667855	2012	5	23	120	6.76	27.82
51667855	2012	5	23	130	7.56	27.82
51667855	2012	5	23	140	8.26	27.81
51667855	2012	5	23	150	8.98	27.81
51667855	2012	5	23	160	9.48	27.8
51667855	2012	5	23	170	10.02	27.79
51667855	2012	5	23	180	10.74	27.78
51667855	2012	5	23	190	11.25	27.77
51667855	2012	5	23	200	11.53	27.76
51667855	2012	5	23	210	11.69	27.75
51667855	2012	5	23	220	12.02	27.75
51667855	2012	5	23	230	12.77	27.74
51667855	2012	5	23	240	13.45	27.74
51667855	2012	5	23	250	14.22	27.72
51667855	2012	5	23	260	14.91	27.71
51667855	2012	5	23	270	15.41	27.71
51667855	2012	5	23	280	15.68	27.7
51667855	2012	5	23	290	14.47	27.71
51667855	2012	5	23	300	14.23	27.7
51667855	2012	5	23	310	14.46	27.69
51667855	2012	5	23	320	14.99	27.68
51667855	2012	5	23	330	16.69	27.65
51667855	2012	5	23	340	18.61	27.63
51667855	2012	5	23	350	20.62	27.6
51667855	2012	5	23	360	23.14	27.58
51667855	2012	5	23	370	25.84	27.53
51667855	2012	5	23	380	28.55	27.5
51667855	2012	5	23	390	30.4	27.48

51667855	2012	5	23	400	31.85	27.46
51667855	2012	5	23	410	32.86	27.44
51667855	2012	5	23	420	34.19	27.4
51667855	2012	5	23	430	35.48	27.39
51667855	2012	5	23	440	36.44	27.37
51667855	2012	5	23	450	37.27	27.35
51667855	2012	5	23	460	37.44	27.34
51667855	2012	5	23	470	38	27.33
51667855	2012	5	23	480	39.11	27.32
51667855	2012	5	23	490	39.69	27.28
51667855	2012	5	23	500	40.49	27.25
51667855	2012	5	23	510	41.12	27.21
51667855	2012	5	23	520	41.31	27.21
51667855	2012	5	23	530	41.99	27.17
51667855	2012	5	23	540	42.57	27.15
51667855	2012	5	23	550	43.14	27.11
51667855	2012	5	23	560	43.58	27.09
51667855	2012	5	23	570	44.71	27.06
51667855	2012	5	23	580	45.42	27.04
51667855	2012	5	23	590	46.38	27
51667855	2012	5	23	600	47.28	26.95
51667855	2012	5	23	610	48.32	26.92
51667855	2012	5	23	620	49.17	26.89
51667855	2012	5	23	630	49.84	26.89
51667855	2012	5	23	640	50.39	26.88
51667855	2012	5	23	650	50.59	26.87
51667855	2012	5	23	660	50.97	26.85
51667855	2012	5	23	670	51.38	26.85
51667855	2012	5	23	680	51.57	26.84
51667855	2012	5	23	690	51.22	26.83
51667855	2012	5	23	700	51.23	26.82
51667855	2012	5	23	710	51.51	26.82
51667855	2012	5	23	720	51.87	26.8
51667855	2012	5	23	730	52.39	26.76
51667855	2012	5	23	740	52.56	26.73
51667855	2012	5	23	750	52.7	26.72
51667855	2012	5	23	760	52.74	26.71
51667855	2012	5	23	770	52.99	26.69
51667855	2012	5	23	780	53.08	26.64
51667855	2012	5	23	790	53.46	26.6
51667855	2012	5	23	800	53.62	26.58
51667855	2012	5	23	810	52.98	26.53
51667855	2012	5	23	820	53.34	26.49
51667855	2012	5	23	830	53.79	26.44
51667855	2012	5	23	840	53.94	26.44
51667855	2012	5	23	850	52.99	26.44
51667855	2012	5	23	860	51.44	26.44

51667855	2012	5	23	870	50.05	26.46
51667855	2012	5	23	880	49.53	26.46
51667855	2012	5	23	890	49.02	26.46
51667855	2012	5	23	900	49.06	26.47
51667855	2012	5	23	910	49.16	26.48
51667855	2012	5	23	920	49.18	26.48
51667855	2012	5	23	930	49.14	26.49
51667855	2012	5	23	940	48.94	26.5
51667855	2012	5	23	950	48.92	26.5
51667855	2012	5	23	960	49.03	26.53
51667855	2012	5	23	970	51.01	26.53
51667855	2012	5	23	980	52.75	26.52
51667855	2012	5	23	990	53.09	26.5
51667855	2012	5	23	1000	52.99	26.49
51667855	2012	5	23	1010	52.63	26.49
51667855	2012	5	23	1020	52.91	26.48
51667855	2012	5	23	1030	53.13	26.47
51667855	2012	5	23	1040	53.58	26.45
51667855	2012	5	23	1050	53.57	26.44
51667855	2012	5	23	1060	53.68	26.42
51667855	2012	5	23	1070	54.1	26.41
51667855	2012	5	23	1080	54.12	26.39
51667855	2012	5	23	1090	54.23	26.39
51667855	2012	5	23	1100	54.19	26.37
51667855	2012	5	23	1110	54.28	26.37
51667855	2012	5	23	1120	54.21	26.35
51667855	2012	5	23	1130	54.42	26.35
51667855	2012	5	23	1140	54.37	26.35
51667855	2012	5	23	1150	54.65	26.35
51667855	2012	5	23	1160	55.01	26.34
51667855	2012	5	23	1170	55.72	26.32
51667855	2012	5	23	1180	57.2	26.29
51667855	2012	5	23	1190	58.23	26.28
51667855	2012	5	23	1200	58.99	26.25
51667855	2012	5	23	1210	59.71	26.23
51667855	2012	5	23	1220	60.6	26.2
51667855	2012	5	23	1230	61.52	26.15
51667855	2012	5	23	1240	62.3	26.13
51667855	2012	5	23	1250	62.93	26.11
51667855	2012	5	23	1260	63.34	26.09
51667855	2012	5	23	1270	63.61	26.07
51667855	2012	5	23	1280	63.82	26.06
51667855	2012	5	23	1290	64.38	26.03
51667855	2012	5	23	1300	66.29	26.01
51667855	2012	5	23	1310	67.78	25.99
51667855	2012	5	23	1320	68.71	25.96
51667855	2012	5	23	1330	68.94	25.96

51667855	2012	5	23	1340	70.27	25.94
51667855	2012	5	23	1350	71.28	25.92
51667855	2012	5	23	1360	72.06	25.91
51667855	2012	5	23	1370	72.51	25.89
51667855	2012	5	23	1380	73.31	25.88
51667855	2012	5	23	1390	75.21	25.86
51667855	2012	5	23	1400	76.79	25.84
51667855	2012	5	23	1410	77.15	25.82
51667855	2012	5	23	1420	77.41	25.82
51667855	2012	5	23	1430	77.82	25.81
51667855	2012	5	23	1440	79.83	25.79
51667855	2012	5	23	1450	82.31	25.76
51667855	2012	5	23	1460	83.81	25.74
51667855	2012	5	23	1470	86.45	25.69
51667855	2012	5	23	1480	87.52	25.68
51667855	2012	5	23	1490	87.95	25.67
51667855	2012	5	23	1500	89.81	25.66
51667855	2012	5	23	1510	91.41	25.64
51667855	2012	5	23	1520	92.48	25.61
51667855	2012	5	23	1530	93.32	25.6
51667855	2012	5	23	1540	95.38	25.57
51667855	2012	5	23	1550	97.52	25.53
51667855	2012	5	23	1560	99.92	25.28
51667855	2012	5	23	1570	101.49	25.26
51667855	2012	5	23	1580	103.38	25.18
51667855	2012	5	23	1590	104.7	25
51667855	2012	5	23	1600	104.6	24.93
51667855	2012	5	23	1610	104.6	24.91
51667855	2012	5	23	1620	104.79	24.83
51667855	2012	5	23	1630	105.58	24.75
51667855	2012	5	23	1640	105.99	24.68
51667855	2012	5	23	1650	106.02	24.61
51667855	2012	5	23	1660	106.05	24.53
51667855	2012	5	23	1670	105.81	24.48
51667855	2012	5	23	1680	105.88	24.42
51667855	2012	5	23	1690	105.96	24.37
51667855	2012	5	23	1700	107.69	24.31
51667855	2012	5	23	1710	109.33	24.24
51667855	2012	5	23	1720	109.9	24.15
51667855	2012	5	23	1730	110.35	24.13
51667855	2012	5	23	1740	112.3	23.97
51667855	2012	5	23	1750	113.79	23.92
51667855	2012	5	23	1760	114.48	23.9
51667855	2012	5	23	1770	114.93	23.82
51667855	2012	5	23	1780	116.1	23.6
51667855	2012	5	23	1790	116.43	23.57
51667855	2012	5	23	1800	116.19	23.53

51667855	2012	5	23	1810	116.83	23.47
51667855	2012	5	23	1820	117.81	23.39
51667855	2012	5	23	1830	118.25	23.31
51667855	2012	5	23	1840	118.92	23.23
51667855	2012	5	23	1850	119.55	23.17
51667855	2012	5	23	1860	119.87	23.13
51667855	2012	5	23	1870	120.3	23.1
51667855	2012	5	23	1880	120.45	23.05
51667855	2012	5	23	1890	121.33	23
51667855	2012	5	23	1900	122.01	22.92
51667855	2012	5	23	1910	122.24	22.87
51667855	2012	5	23	1920	122.42	22.79
51667855	2012	5	23	1930	122.56	22.73
51667855	2012	5	23	1940	122.64	22.66
51667855	2012	5	23	1950	122.94	22.57
51667855	2012	5	23	1960	123.2	22.51
51667855	2012	5	23	1970	123.49	22.44
51667855	2012	5	23	1980	123.73	22.36
51667855	2012	5	23	1990	123.91	22.32
51667855	2012	5	23	2000	124.07	22.27
51667855	2012	5	23	2010	125.29	22.22
51667855	2012	5	23	2020	126.08	22.18
51667855	2012	5	23	2030	126.67	22.15
51667855	2012	5	23	2040	127.43	22.11
51667855	2012	5	23	2050	127.8	22.09
51667855	2012	5	23	2060	127.91	22.05
51667855	2012	5	23	2070	128.07	22.01
51667855	2012	5	23	2080	128.24	21.95
51667855	2012	5	23	2090	128.46	21.92
51667855	2012	5	23	2100	128.56	21.89
51667855	2012	5	23	2110	128.89	21.84
51667855	2012	5	23	2120	129.11	21.79
51667855	2012	5	23	2130	128.98	21.74
51667855	2012	5	23	2140	129.52	21.67
51667855	2012	5	23	2150	129.18	21.6
51667855	2012	5	23	2160	128.4	21.51
51667855	2012	5	23	2170	128.02	21.35
51667855	2012	5	23	2180	128.42	21.19
51667855	2012	5	23	2190	128.55	21.15
51667855	2012	5	23	2200	128.53	21.13
51667855	2012	5	23	2210	128.63	21.1
51667855	2012	5	23	2220	128.72	21.08
51667855	2012	5	23	2230	128.7	21.03
51667855	2012	5	23	2240	128.69	21.01
51667855	2012	5	23	2250	128.71	21
51667855	2012	5	23	2260	128.93	20.97
51667855	2012	5	23	2270	128.91	20.96

51667855	2012	5	23	2280	128.96	20.91
51667855	2012	5	23	2290	129.18	20.84
51667855	2012	5	23	2300	129.35	20.78
51667855	2012	5	23	2310	129.35	20.71
51667855	2012	5	23	2320	129.62	20.66
51667855	2012	5	23	2330	129.93	20.56
51667855	2012	5	23	2340	129.97	20.48
51667855	2012	5	23	2350	129.89	20.44
51667855	2012	5	23	2360	129.73	20.42
51667855	2012	5	23	2370	129.71	20.39
51667855	2012	5	23	2380	130.14	20.32
51667855	2012	5	23	2390	130.41	20.28
51667855	2012	5	23	2400	130.42	20.24
51667855	2012	5	23	2410	130.6	20.18
51667855	2012	5	23	2420	130.79	20.14
51667855	2012	5	23	2430	131.06	20.1
51667855	2012	5	23	2440	131.56	20.07
51667855	2012	5	23	2450	131.82	20
51667855	2012	5	23	2460	131.9	19.96
51667855	2012	5	23	2470	131.75	19.92
51667855	2012	5	23	2480	131.47	19.89
51667855	2012	5	23	2490	131.27	19.86
51667855	2012	5	23	2500	131.19	19.84
51667855	2012	5	23	2510	130.97	19.81
51667855	2012	5	23	2520	131.21	19.77
51667855	2012	5	23	2530	131.52	19.74
51667855	2012	5	23	2540	131.63	19.72
51667855	2012	5	23	2550	131.55	19.7
51667855	2012	5	23	2560	130.59	19.67
51667855	2012	5	23	2570	129.23	19.66
51667855	2012	5	23	2580	128.59	19.64
51667855	2012	5	23	2590	127.06	19.64
51667855	2012	5	23	2600	126.17	19.67
51667855	2012	5	23	2610	125.38	19.71
51667855	2012	5	23	2620	124.1	19.78
51667855	2012	5	23	2630	122.64	19.89
51667855	2012	5	23	2640	121.73	20
51667855	2012	5	23	2650	120.45	20.05
51667855	2012	5	23	2660	119	20.12
51667855	2012	5	23	2670	118.22	20.19
51667855	2012	5	23	2680	117.09	20.28
51667855	2012	5	23	2690	115.76	20.35
51667855	2012	5	23	2700	115.5	20.41
51667855	2012	5	23	2710	115.42	20.46
51667855	2012	5	23	2720	115.28	20.55
51667855	2012	5	23	2730	114.57	20.62
51667855	2012	5	23	2740	113.92	20.68

51667855	2012	5	23	2750	113.42	20.73
51667855	2012	5	23	2760	112.29	20.78
51667855	2012	5	23	2770	110.64	20.85
51667855	2012	5	23	2780	109.06	20.91
51667855	2012	5	23	2790	107.8	21
51667855	2012	5	23	2800	106.91	21.08
51667855	2012	5	23	2810	105.77	21.15
51667855	2012	5	23	2820	104.53	21.23
51667855	2012	5	23	2830	103	21.29
51667855	2012	5	23	2840	101.89	21.36
51667855	2012	5	23	2850	100.47	21.46
51667855	2012	5	23	2860	98.86	21.54
51667855	2012	5	23	2870	97.17	21.6
51667855	2012	5	23	2880	95.57	21.68
51667855	2012	5	23	2890	94.48	21.76
51667855	2012	5	23	2900	93.61	21.89
51667855	2012	5	23	2910	93.04	22.03
51667855	2012	5	23	2920	91.8	22.13
51667855	2012	5	23	2930	91.29	22.22
51667855	2012	5	23	2940	90.56	22.41
51667855	2012	5	23	2950	90.04	22.58
51667855	2012	5	23	2960	88.79	22.68
51667855	2012	5	23	2970	88.17	22.89
51667855	2012	5	23	2980	87.09	23
51667855	2012	5	23	2990	86.66	23.15
51667855	2012	5	23	3000	86.78	23.17
51667855	2012	5	23	3010	87.1	23.35
51667855	2012	5	23	3020	86.57	23.44
51667855	2012	5	23	3030	86.86	23.46
51667855	2012	5	23	3040	86.53	23.52
51667855	2012	5	23	3050	85.98	23.59
51667855	2012	5	23	3060	86.04	23.67
51667855	2012	5	23	3070	85.91	23.75
51667855	2012	5	23	3080	86.01	23.84
51667855	2012	5	23	3090	85.91	23.88
51667855	2012	5	23	3100	85.82	23.92
51667855	2012	5	23	3110	85.82	23.97
51667855	2012	5	23	3120	86.04	24.01
51667855	2012	5	23	3130	86.03	24.06
51667855	2012	5	23	3140	85.99	24.1
51667855	2012	5	23	3150	85.82	24.14
51667855	2012	5	23	3160	84.64	24.2
51667855	2012	5	23	3170	83.15	24.27
51667855	2012	5	23	3180	81.65	24.31
51667855	2012	5	23	3190	80.22	24.39
51667855	2012	5	23	3200	78.71	24.45
51667855	2012	5	23	3210	76.76	24.5

51667855	2012	5	23	3220	75.21	24.55
51667855	2012	5	23	3230	73.66	24.59
51667855	2012	5	23	3240	71.19	24.64
51667855	2012	5	23	3250	68.44	24.69
51667855	2012	5	23	3260	66.7	24.75
51667855	2012	5	23	3270	65.79	24.81
51667855	2012	5	23	3280	63.94	24.86
51667855	2012	5	23	3290	62.96	24.92
51667855	2012	5	23	3300	63.01	25.05
51667855	2012	5	23	3310	62.81	25.12
51667855	2012	5	23	3320	61.9	25.17
51667855	2012	5	23	3330	61.15	25.21
51667855	2012	5	23	3340	59.55	25.29
51667855	2012	5	23	3350	58.03	25.38
51667855	2012	5	23	3360	56.66	25.45
51667855	2012	5	23	3370	55.47	25.51
51667855	2012	5	23	3380	54.4	25.55
51667855	2012	5	23	3390	53.86	25.68
51667855	2012	5	23	3400	53.41	25.75
51667855	2012	5	23	3410	52.84	25.78
51667855	2012	5	23	3420	52.95	25.85
51667855	2012	5	23	3430	53.6	25.84
51667855	2012	5	23	3440	53.41	25.85
51667855	2012	5	23	3450	51.89	25.89
51667855	2012	5	23	3460	51.07	25.94
51667855	2012	5	23	3470	51.08	26
51667855	2012	5	23	3480	51.37	26.03
51667855	2012	5	23	3490	51.4	26.07
51667855	2012	5	23	3500	51.26	26.08
51667855	2012	5	23	3510	51.14	26.1
51667855	2012	5	23	3520	50.76	26.13
51667855	2012	5	23	3530	50.65	26.16
51667855	2012	5	23	3540	51.3	26.18
51667855	2012	5	23	3550	51.49	26.21
51667855	2012	5	23	3560	51.37	26.25
51667855	2012	5	23	3570	51.41	26.27
51667855	2012	5	23	3580	51.49	26.29
51667855	2012	5	23	3590	51.63	26.31
51667855	2012	5	23	3600	51.89	26.31
51667855	2012	5	23	3610	51.44	26.34
51667855	2012	5	23	3620	51.77	26.34
51667855	2012	5	23	3630	51.7	26.35
51667855	2012	5	23	3640	51.98	26.37
51667855	2012	5	23	3650	52.1	26.4
51667855	2012	5	23	3660	52.13	26.4
51667855	2012	5	23	3670	52.29	26.42
51667855	2012	5	23	3680	52.07	26.43

51667855	2012	5	23	3690	52.05	26.46
51667855	2012	5	23	3700	52.21	26.46
51667855	2012	5	23	3710	52.51	26.45
51667855	2012	5	23	3720	52.6	26.46
51667855	2012	5	23	3730	52	26.46
51667855	2012	5	23	3740	51.76	26.46
51667855	2012	5	23	3750	51.71	26.47
51667855	2012	5	23	3760	51.69	26.47
51667855	2012	5	23	3770	51.17	26.5
51667855	2012	5	23	3780	49.82	26.5
51667855	2012	5	23	3790	49.28	26.52
51667855	2012	5	23	3800	49.14	26.53
51667855	2012	5	23	3810	47.75	26.55
51667855	2012	5	23	3820	47.03	26.57
51667855	2012	5	23	3830	47.12	26.58
51667855	2012	5	23	3840	47.35	26.58
51667855	2012	5	23	3850	47.53	26.59
51667855	2012	5	23	3860	46.99	26.6
51667855	2012	5	23	3870	46.78	26.61
51667855	2012	5	23	3880	46.45	26.62
51667855	2012	5	23	3890	45.75	26.64
51667855	2012	5	23	3900	44.83	26.64
51667855	2012	5	23	3910	44.45	26.65
51667855	2012	5	23	3920	43.03	26.67
51667855	2012	5	23	3930	41.3	26.69
51667855	2012	5	23	3940	39.92	26.71
51667855	2012	5	23	3950	38.37	26.72
51667855	2012	5	23	3960	36.58	26.75
51667855	2012	5	23	3970	35.45	26.76
51667855	2012	5	23	3980	35.12	26.77
51667855	2012	5	23	3990	35.38	26.78
51667855	2012	5	23	4000	34.81	26.78
51667855	2012	5	23	4010	33.35	26.79
51667855	2012	5	23	4020	31.72	26.81
51667855	2012	5	23	4030	29.69	26.82
51667855	2012	5	23	4040	28.14	26.85
51667855	2012	5	23	4050	27.2	26.86
51667855	2012	5	23	4060	27.18	26.86
51667855	2012	5	23	4070	26.28	26.87
51667855	2012	5	23	4080	26.17	26.88
51667855	2012	5	23	4090	26.41	26.89
51667855	2012	5	23	4100	26.45	26.89
51667855	2012	5	23	4110	25.73	26.89
51667855	2012	5	23	4120	25.62	26.89
51667855	2012	5	23	4130	24.77	26.91
51667855	2012	5	23	4140	23.8	26.93
51667855	2012	5	23	4150	23.73	26.93

51667855	2012	5	23	4160	23.91	26.92
51667855	2012	5	23	4170	23.95	26.93
51667855	2012	5	23	4180	23.96	26.93
51667855	2012	5	23	4190	24.48	26.93
51667855	2012	5	23	4200	24.95	26.93
51667855	2012	5	23	4210	25.23	26.93
51667855	2012	5	23	4220	25.39	26.92
51667855	2012	5	23	4230	25.52	26.93
51667855	2012	5	23	4240	24.99	26.94
51667855	2012	5	23	4250	24.65	26.94
51667855	2012	5	23	4260	24.99	26.94
51667855	2012	5	23	4270	24.91	26.94
51667855	2012	5	23	4280	25.09	26.94
51667855	2012	5	23	4290	25.1	26.95
51667855	2012	5	23	4300	24.64	26.95
51667855	2012	5	23	4310	24.23	26.95
51667855	2012	5	23	4320	24.11	26.95
51667855	2012	5	23	4330	24.42	26.95
51667855	2012	5	23	4340	24.17	26.96
51667855	2012	5	23	4350	24.03	26.95
51667855	2012	5	23	4360	23.58	26.95
51667855	2012	5	23	4370	23.11	26.96
51667855	2012	5	23	4380	22.31	26.96
51667855	2012	5	23	4390	21.18	26.96
51667855	2012	5	23	4400	20.82	26.98
51667855	2012	5	23	4410	20.66	26.97
51667855	2012	5	23	4420	20.9	26.99
51667855	2012	5	23	4430	20.91	26.98
51667855	2012	5	23	4440	20.6	26.98
51667855	2012	5	23	4450	20.22	26.99
51667855	2012	5	23	4460	20.15	26.99
51667855	2012	5	23	4470	19.94	27
51667855	2012	5	23	4480	19.93	26.99
51667855	2012	5	23	4490	19.83	27
51667855	2012	5	23	4500	19.28	27
51667855	2012	5	23	4510	18.7	27
51667855	2012	5	23	4520	18.87	27.01
51667855	2012	5	23	4530	19.08	27.01
51667855	2012	5	23	4540	19.05	27.01
51667855	2012	5	23	4550	18.87	27.01
51667855	2012	5	23	4560	18.71	27.02
51667855	2012	5	23	4570	18.85	27.01
51667855	2012	5	23	4580	19.09	27.01
51667855	2012	5	23	4590	18.18	27.02
51667855	2012	5	23	4600	17.11	27.03
51667855	2012	5	23	4610	15.79	27.03
51667855	2012	5	23	4620	15.58	27.04

51667855	2012	5	23	4630	15.33	27.05
51667855	2012	5	23	4640	15.1	27.06
51667855	2012	5	23	4650	15.01	27.07
51667855	2012	5	23	4660	15.01	27.07
51667855	2012	5	23	4670	14.13	27.07
51667855	2012	5	23	4680	12.36	27.1
51667855	2012	5	23	4690	10.44	27.11
51667855	2012	5	23	4700	8.46	27.15
51667855	2012	5	23	4710	6.66	27.2
51667855	2012	5	23	4720	5.08	27.23
51667855	2012	5	23	4730	3.54	27.25
51667855	2012	5	23	4740	3	27.28
51667855	2012	5	23	4750	2.47	27.3
51667855	2012	5	23	4760	2.46	27.34
51667855	2012	5	23	4770	2.86	27.36
51667855	2012	5	23	4780	0.88	27.39
51667855	2012	5	23	4790	0.63	27.38
51667855	2012	5	23	4800	0.59	27.4
51667855	2012	5	23	4810	0.54	27.47
51667855	2012	5	23	4820	0.62	27.53
51667855	2012	5	23	4830	0.68	27.55
51667855	2012	5	23	4840	0.62	27.58
51667855	2012	5	23	4850	0.61	27.62
51667855	2012	5	23	4860	0.61	27.66
51667855	2012	5	23	4870	0.63	27.66
51667855	2012	5	23	4880	0.59	27.67
51667855	2012	5	23	4890	0.62	27.67
51667855	2012	5	23	4900	0.57	27.71
51667855	2012	5	23	4910	0.53	27.71
51667855	2012	5	23	4920	0.57	27.73
51734345	2012	5	24	0	0.91	27.55
51734345	2012	5	24	10	1.58	27.55
51734345	2012	5	24	20	1.88	27.55
51734345	2012	5	24	30	2.12	27.54
51734345	2012	5	24	40	2.54	27.53
51734345	2012	5	24	50	3.16	27.53
51734345	2012	5	24	60	3.26	27.53
51734345	2012	5	24	70	3.14	27.53
51734345	2012	5	24	80	2.83	27.53
51734345	2012	5	24	90	3.03	27.53
51734345	2012	5	24	100	3.21	27.53
51734345	2012	5	24	110	3.15	27.53
51734345	2012	5	24	120	3.15	27.53
51734345	2012	5	24	130	3.15	27.53
51734345	2012	5	24	140	3.15	27.53
51734345	2012	5	24	150	3.08	27.53
51734345	2012	5	24	160	2.72	27.54

51734345	2012	5	24	170	2.86	27.53
51734345	2012	5	24	180	3.08	27.54
51734345	2012	5	24	190	2.36	27.54
51734345	2012	5	24	200	0.77	27.56
51734345	2012	5	24	210	0.7	27.55
51734345	2012	5	24	220	0.66	27.56
51734345	2012	5	24	230	0.74	27.56
51734345	2012	5	24	240	0.71	27.55
51734345	2012	5	24	250	0.72	27.56
51734345	2012	5	24	260	0.73	27.56
51734345	2012	5	24	270	0.67	27.56
51734345	2012	5	24	280	0.75	27.56
51734345	2012	5	24	290	0.73	27.56
51734345	2012	5	24	300	0.71	27.56
51734345	2012	5	24	310	0.7	27.56
51734345	2012	5	24	320	0.69	27.56
51734345	2012	5	24	330	0.73	27.56
51734345	2012	5	24	340	1.07	27.56
51734345	2012	5	24	350	1.88	27.54
51734345	2012	5	24	360	2.5	27.54
51734345	2012	5	24	370	2.98	27.53
51734345	2012	5	24	380	3.21	27.53
51734345	2012	5	24	390	2.62	27.54
51734345	2012	5	24	400	1.82	27.55
51734345	2012	5	24	410	1.77	27.56
51734345	2012	5	24	420	1.12	27.55
51734345	2012	5	24	430	1.99	27.55
51734345	2012	5	24	440	2.89	27.54
51734345	2012	5	24	450	4.7	27.53
51734345	2012	5	24	460	7.21	27.5
51734345	2012	5	24	470	9.61	27.48
51734345	2012	5	24	480	11.79	27.46
51734345	2012	5	24	490	13.06	27.45
51734345	2012	5	24	500	13.97	27.45
51734345	2012	5	24	510	14.86	27.43
51734345	2012	5	24	520	14.9	27.43
51734345	2012	5	24	530	14.64	27.43
51734345	2012	5	24	540	15.18	27.43
51734345	2012	5	24	550	14.93	27.43
51734345	2012	5	24	560	14.47	27.44
51734345	2012	5	24	570	14.45	27.44
51734345	2012	5	24	580	15.71	27.42
51734345	2012	5	24	590	16.01	27.42
51734345	2012	5	24	600	16.08	27.43
51734345	2012	5	24	610	16.22	27.42
51734345	2012	5	24	620	16.77	27.42
51734345	2012	5	24	630	17.28	27.42

51734345	2012	5	24	640	17.97	27.41
51734345	2012	5	24	650	19.91	27.39
51734345	2012	5	24	660	21.29	27.39
51734345	2012	5	24	670	22.46	27.37
51734345	2012	5	24	680	23.81	27.35
51734345	2012	5	24	690	25.41	27.35
51734345	2012	5	24	700	27.28	27.33
51734345	2012	5	24	710	29.03	27.32
51734345	2012	5	24	720	31.32	27.3
51734345	2012	5	24	730	33.68	27.28
51734345	2012	5	24	740	36.03	27.26
51734345	2012	5	24	750	37.88	27.25
51734345	2012	5	24	760	39.79	27.23
51734345	2012	5	24	770	41.85	27.22
51734345	2012	5	24	780	43.29	27.21
51734345	2012	5	24	790	43.43	27.21
51734345	2012	5	24	800	44.67	27.21
51734345	2012	5	24	810	46.51	27.19
51734345	2012	5	24	820	48.53	27.17
51734345	2012	5	24	830	50.47	27.16
51734345	2012	5	24	840	52.26	27.15
51734345	2012	5	24	850	54.22	27.14
51734345	2012	5	24	860	56.47	27.13
51734345	2012	5	24	870	58.44	27.1
51734345	2012	5	24	880	60.57	27.08
51734345	2012	5	24	890	62.68	27.07
51734345	2012	5	24	900	64.81	27.07
51734345	2012	5	24	910	66.94	27.03
51734345	2012	5	24	920	69.03	26.98
51734345	2012	5	24	930	70.66	26.92
51734345	2012	5	24	940	72.24	26.71
51734345	2012	5	24	950	73.98	26.54
51734345	2012	5	24	960	74.99	26.39
51734345	2012	5	24	970	75.25	26.35
51734345	2012	5	24	980	76.1	26.32
51734345	2012	5	24	990	77.12	26.29
51734345	2012	5	24	1000	78.02	26.25
51734345	2012	5	24	1010	79.6	26.2
51734345	2012	5	24	1020	82.1	25.98
51734345	2012	5	24	1030	84.11	25.79
51734345	2012	5	24	1040	85.67	25.68
51734345	2012	5	24	1050	86.82	25.64
51734345	2012	5	24	1060	87.72	25.59
51734345	2012	5	24	1070	88.41	25.42
51734345	2012	5	24	1080	89.32	25.37
51734345	2012	5	24	1090	90.33	25.23
51734345	2012	5	24	1100	91.3	25.11

51734345	2012	5	24	1110	92.12	24.99
51734345	2012	5	24	1120	92.97	24.9
51734345	2012	5	24	1130	94.32	24.51
51734345	2012	5	24	1140	95.85	24.35
51734345	2012	5	24	1150	96.99	24.28
51734345	2012	5	24	1160	97.68	24.25
51734345	2012	5	24	1170	98.5	24.19
51734345	2012	5	24	1180	99.59	24.11
51734345	2012	5	24	1190	101.18	23.78
51734345	2012	5	24	1200	102.46	23.75
51734345	2012	5	24	1210	104.17	23.66
51734345	2012	5	24	1220	106.05	23.52
51734345	2012	5	24	1230	108.02	23.34
51734345	2012	5	24	1240	110.14	23.28
51734345	2012	5	24	1250	112.08	23.21
51734345	2012	5	24	1260	113.7	23.18
51734345	2012	5	24	1270	114.98	23.17
51734345	2012	5	24	1280	116.71	23.15
51734345	2012	5	24	1290	120.1	23.05
51734345	2012	5	24	1300	122.79	22.99
51734345	2012	5	24	1310	124.75	22.96
51734345	2012	5	24	1320	126.33	22.91
51734345	2012	5	24	1330	129.14	22.76
51734345	2012	5	24	1340	131.59	22.67
51734345	2012	5	24	1350	133.51	22.65
51734345	2012	5	24	1360	135.26	22.51
51734345	2012	5	24	1370	136.7	22.35
51734345	2012	5	24	1380	138.33	22.26
51734345	2012	5	24	1390	139.89	22.21
51734345	2012	5	24	1400	142.69	21.93
51734345	2012	5	24	1410	144.43	21.69
51734345	2012	5	24	1420	145.39	21.5
51734345	2012	5	24	1430	146.59	21.41
51734345	2012	5	24	1440	147.52	21.32
51734345	2012	5	24	1450	148.98	21.17
51734345	2012	5	24	1460	150.28	21
51734345	2012	5	24	1470	151.45	20.95
51734345	2012	5	24	1480	152.44	20.86
51734345	2012	5	24	1490	153.32	20.85
51734345	2012	5	24	1500	155.05	20.76
51734345	2012	5	24	1510	157.03	20.58
51734345	2012	5	24	1520	158.02	20.5
51734345	2012	5	24	1530	159.05	20.41
51734345	2012	5	24	1540	160.31	20.32
51734345	2012	5	24	1550	161.77	20.25
51734345	2012	5	24	1560	162.75	20.15
51734345	2012	5	24	1570	163.81	20.07

51734345	2012	5	24	1580	164.4	19.91
51734345	2012	5	24	1590	164.6	19.78
51734345	2012	5	24	1600	164.62	19.67
51734345	2012	5	24	1610	164.8	19.59
51734345	2012	5	24	1620	165.26	19.5
51734345	2012	5	24	1630	165.75	19.39
51734345	2012	5	24	1640	166.58	19.3
51734345	2012	5	24	1650	167.17	19.14
51734345	2012	5	24	1660	168.29	19.03
51734345	2012	5	24	1670	169.54	18.96
51734345	2012	5	24	1680	170.34	18.89
51734345	2012	5	24	1690	170.89	18.79
51734345	2012	5	24	1700	171.53	18.75
51734345	2012	5	24	1710	172.39	18.69
51734345	2012	5	24	1720	173.19	18.62
51734345	2012	5	24	1730	174.17	18.53
51734345	2012	5	24	1740	175.04	18.46
51734345	2012	5	24	1750	175.54	18.39
51734345	2012	5	24	1760	176.38	18.32
51734345	2012	5	24	1770	177.25	18.27
51734345	2012	5	24	1780	178.56	18.21
51734345	2012	5	24	1790	179.08	18.15
51734345	2012	5	24	1800	179.9	18.09
51734345	2012	5	24	1810	180.48	18.04
51734345	2012	5	24	1820	181.13	17.96
51734345	2012	5	24	1830	181.64	17.89
51734345	2012	5	24	1840	182.28	17.8
51734345	2012	5	24	1850	182.28	17.73
51734345	2012	5	24	1860	182.39	17.65
51734345	2012	5	24	1870	182.82	17.63
51734345	2012	5	24	1880	183.49	17.62
51734345	2012	5	24	1890	184.25	17.6
51734345	2012	5	24	1900	184.87	17.58
51734345	2012	5	24	1910	185.11	17.53
51734345	2012	5	24	1920	185.73	17.5
51734345	2012	5	24	1930	186.5	17.47
51734345	2012	5	24	1940	187.22	17.45
51734345	2012	5	24	1950	187.99	17.41
51734345	2012	5	24	1960	188.78	17.38
51734345	2012	5	24	1970	189.61	17.35
51734345	2012	5	24	1980	190.46	17.31
51734345	2012	5	24	1990	191.99	17.25
51734345	2012	5	24	2000	193.14	17.2
51734345	2012	5	24	2010	194.06	17.17
51734345	2012	5	24	2020	193.96	17.14
51734345	2012	5	24	2030	193.75	17.1
51734345	2012	5	24	2040	194.21	17.06

51734345	2012	5	24	2050	194.34	17.02
51734345	2012	5	24	2060	194.82	16.97
51734345	2012	5	24	2070	195.47	16.9
51734345	2012	5	24	2080	195.23	16.86
51734345	2012	5	24	2090	195.31	16.77
51734345	2012	5	24	2100	195.27	16.72
51734345	2012	5	24	2110	195.52	16.71
51734345	2012	5	24	2120	196.08	16.67
51734345	2012	5	24	2130	196.44	16.65
51734345	2012	5	24	2140	196.68	16.63
51734345	2012	5	24	2150	196.93	16.57
51734345	2012	5	24	2160	197.23	16.52
51734345	2012	5	24	2170	197.51	16.51
51734345	2012	5	24	2180	197.74	16.5
51734345	2012	5	24	2190	197.92	16.48
51734345	2012	5	24	2200	198.08	16.45
51734345	2012	5	24	2210	198.4	16.42
51734345	2012	5	24	2220	199.09	16.4
51734345	2012	5	24	2230	199.7	16.37
51734345	2012	5	24	2240	200.14	16.34
51734345	2012	5	24	2250	200.55	16.32
51734345	2012	5	24	2260	201.23	16.28
51734345	2012	5	24	2270	201.37	16.25
51734345	2012	5	24	2280	201.76	16.21
51734345	2012	5	24	2290	202.3	16.19
51734345	2012	5	24	2300	202.64	16.18
51734345	2012	5	24	2310	202.69	16.15
51734345	2012	5	24	2320	202.75	16.13
51734345	2012	5	24	2330	202.95	16.12
51734345	2012	5	24	2340	203.1	16.1
51734345	2012	5	24	2350	202.93	16.1
51734345	2012	5	24	2360	203.02	16.08
51734345	2012	5	24	2370	203.37	16.06
51734345	2012	5	24	2380	203.58	16.05
51734345	2012	5	24	2390	203.57	16.03
51734345	2012	5	24	2400	203.97	16.03
51734345	2012	5	24	2410	204.63	16
51734345	2012	5	24	2420	205.22	16
51734345	2012	5	24	2430	205.74	15.99
51734345	2012	5	24	2440	206.24	15.96
51734345	2012	5	24	2450	207.54	15.96
51734345	2012	5	24	2460	208.37	15.95
51734345	2012	5	24	2470	209.01	15.89
51734345	2012	5	24	2480	209.63	15.87
51734345	2012	5	24	2490	210.82	15.85
51734345	2012	5	24	2500	211.82	15.84
51734345	2012	5	24	2510	212.47	15.82

51734345	2012	5	24	2520	212.11	15.81
51734345	2012	5	24	2530	212.83	15.78
51734345	2012	5	24	2540	213.93	15.76
51734345	2012	5	24	2550	214.76	15.75
51734345	2012	5	24	2560	216.11	15.72
51734345	2012	5	24	2570	217.71	15.7
51734345	2012	5	24	2580	218.54	15.67
51734345	2012	5	24	2590	218.71	15.65
51734345	2012	5	24	2600	219.34	15.65
51734345	2012	5	24	2610	220.2	15.64
51734345	2012	5	24	2620	221	15.64
51734345	2012	5	24	2630	221.58	15.62
51734345	2012	5	24	2640	221.89	15.61
51734345	2012	5	24	2650	222.28	15.6
51734345	2012	5	24	2660	222.97	15.57
51734345	2012	5	24	2670	223.65	15.54
51734345	2012	5	24	2680	223.79	15.51
51734345	2012	5	24	2690	224.17	15.51
51734345	2012	5	24	2700	225.74	15.47
51734345	2012	5	24	2710	226.9	15.46
51734345	2012	5	24	2720	227.39	15.39
51734345	2012	5	24	2730	227.79	15.39
51734345	2012	5	24	2740	228.32	15.37
51734345	2012	5	24	2750	229.07	15.35
51734345	2012	5	24	2760	229.96	15.35
51734345	2012	5	24	2770	230.84	15.32
51734345	2012	5	24	2780	231.75	15.3
51734345	2012	5	24	2790	232.54	15.28
51734345	2012	5	24	2800	233.2	15.25
51734345	2012	5	24	2810	233.82	15.19
51734345	2012	5	24	2820	234.51	15.17
51734345	2012	5	24	2830	235.42	15.11
51734345	2012	5	24	2840	237.05	15.1
51734345	2012	5	24	2850	238.75	15.07
51734345	2012	5	24	2860	240.23	15.04
51734345	2012	5	24	2870	242.05	15.01
51734345	2012	5	24	2880	244.03	14.99
51734345	2012	5	24	2890	245.11	14.96
51734345	2012	5	24	2900	246.56	14.93
51734345	2012	5	24	2910	247.89	14.9
51734345	2012	5	24	2920	248.93	14.82
51734345	2012	5	24	2930	249.72	14.81
51734345	2012	5	24	2940	250.16	14.77
51734345	2012	5	24	2950	250.37	14.71
51734345	2012	5	24	2960	251.14	14.69
51734345	2012	5	24	2970	251.97	14.67
51734345	2012	5	24	2980	252.61	14.66

51734345	2012	5	24	2990	253.6	14.64
51734345	2012	5	24	3000	254.74	14.62
51734345	2012	5	24	3010	255.51	14.6
51734345	2012	5	24	3020	256.7	14.58
51734345	2012	5	24	3030	257.83	14.57
51734345	2012	5	24	3040	259.24	14.55
51734345	2012	5	24	3050	259.72	14.51
51734345	2012	5	24	3060	260.48	14.47
51734345	2012	5	24	3070	262.48	14.46
51734345	2012	5	24	3080	264.44	14.43
51734345	2012	5	24	3090	265.65	14.42
51734345	2012	5	24	3100	266.32	14.38
51734345	2012	5	24	3110	266.93	14.37
51734345	2012	5	24	3120	268.21	14.35
51734345	2012	5	24	3130	269.48	14.32
51734345	2012	5	24	3140	270.73	14.31
51734345	2012	5	24	3150	271.7	14.28
51734345	2012	5	24	3160	273.15	14.25
51734345	2012	5	24	3170	274.22	14.2
51734345	2012	5	24	3180	274.63	14.17
51734345	2012	5	24	3190	275.09	14.14
51734345	2012	5	24	3200	275.98	14.13
51734345	2012	5	24	3210	276.74	14.1
51734345	2012	5	24	3220	277.65	14.03
51734345	2012	5	24	3230	278.3	14
51734345	2012	5	24	3240	279.11	13.96
51734345	2012	5	24	3250	279.73	13.94
51734345	2012	5	24	3260	280.38	13.92
51734345	2012	5	24	3270	280.49	13.89
51734345	2012	5	24	3280	280.82	13.8
51734345	2012	5	24	3290	281.28	13.75
51734345	2012	5	24	3300	282.19	13.75
51734345	2012	5	24	3310	282.71	13.75
51734345	2012	5	24	3320	283.79	13.74
51734345	2012	5	24	3330	283.98	13.73
51734345	2012	5	24	3340	284.4	13.72
51734345	2012	5	24	3350	285.51	13.71
51734345	2012	5	24	3360	286.36	13.7
51734345	2012	5	24	3370	287	13.68
51734345	2012	5	24	3380	287.59	13.67
51734345	2012	5	24	3390	288.46	13.66
51734345	2012	5	24	3400	289.99	13.64
51734345	2012	5	24	3410	290.96	13.63
51734345	2012	5	24	3420	291.15	13.59
51734345	2012	5	24	3430	291.08	13.56
51734345	2012	5	24	3440	290.77	13.53
51734345	2012	5	24	3450	291.2	13.52

51734345	2012	5	24	3460	292.53	13.51
51734345	2012	5	24	3470	293.34	13.5
51734345	2012	5	24	3480	294.21	13.48
51734345	2012	5	24	3490	294.93	13.46
51734345	2012	5	24	3500	296.28	13.46
51734345	2012	5	24	3510	297.71	13.44
51734345	2012	5	24	3520	298.78	13.42
51734345	2012	5	24	3530	299.77	13.42
51734345	2012	5	24	3540	300.76	13.41
51734345	2012	5	24	3550	301.49	13.39
51734345	2012	5	24	3560	302.52	13.39
51734345	2012	5	24	3570	303.05	13.38
51734345	2012	5	24	3580	303.17	13.36
51734345	2012	5	24	3590	303.22	13.36
51734345	2012	5	24	3600	303.29	13.35
51734345	2012	5	24	3610	303.21	13.33
51734345	2012	5	24	3620	303.19	13.32
51734345	2012	5	24	3630	303.22	13.32
51734345	2012	5	24	3640	303.23	13.31
51734345	2012	5	24	3650	303.31	13.3
51734345	2012	5	24	3660	303.27	13.29
51734345	2012	5	24	3670	303.31	13.28
51734345	2012	5	24	3680	303.33	13.27
51734345	2012	5	24	3690	303.29	13.26
51734345	2012	5	24	3700	303.33	13.25
51734345	2012	5	24	3710	303.31	13.25
51734345	2012	5	24	3720	303.32	13.24
51734345	2012	5	24	3730	303.34	13.23
51734345	2012	5	24	3740	303.34	13.23
51734345	2012	5	24	3750	303.34	13.22
51734345	2012	5	24	3760	303.27	13.21
51734345	2012	5	24	3770	303.28	13.21
51734345	2012	5	24	3780	303.29	13.21
51734345	2012	5	24	3790	303.28	13.21
51734345	2012	5	24	3800	303.29	13.2
51734345	2012	5	24	3810	303.29	13.19
51734345	2012	5	24	3820	303.3	13.18
51734345	2012	5	24	3830	303.29	13.19
51734345	2012	5	24	3840	303.3	13.18
51734345	2012	5	24	3850	303.3	13.18
51734345	2012	5	24	3860	303.31	13.17
51734345	2012	5	24	3870	303.3	13.17
51734345	2012	5	24	3880	303.31	13.17
51734345	2012	5	24	3890	303.31	13.17
51734345	2012	5	24	3900	303.37	13.17
51734345	2012	5	24	3910	303.36	13.17
51734345	2012	5	24	3920	303.57	13.16

51734345	2012	5	24	3930	303.75	13.15
51734345	2012	5	24	3940	303.49	13.15
51734345	2012	5	24	3950	303.25	13.15
51734345	2012	5	24	3960	303.31	13.14
51734345	2012	5	24	3970	303.33	13.14
51734345	2012	5	24	3980	303.29	13.13
51734345	2012	5	24	3990	303.16	13.13
51734345	2012	5	24	4000	302.98	13.12
51734345	2012	5	24	4010	303.1	13.11
51734345	2012	5	24	4020	303.14	13.11
51734345	2012	5	24	4030	302.8	13.1
51734345	2012	5	24	4040	303.24	13.1
51734345	2012	5	24	4050	302.8	13.1
51734345	2012	5	24	4060	302.73	13.1
51734345	2012	5	24	4070	303.29	13.09
51734345	2012	5	24	4080	303.13	13.08
51734345	2012	5	24	4090	303.1	13.08
51734345	2012	5	24	4100	303.12	13.07
51734345	2012	5	24	4110	303.1	13.07
51734345	2012	5	24	4120	302.99	13.07
51734345	2012	5	24	4130	302.67	13.06
51734345	2012	5	24	4140	301.81	13.05
51734345	2012	5	24	4150	300.77	13.03
51734345	2012	5	24	4160	299.64	13.03
51734345	2012	5	24	4170	299.2	13
51734345	2012	5	24	4180	298.39	13
51734345	2012	5	24	4190	298.3	12.99
51734345	2012	5	24	4200	296.95	12.99
51734345	2012	5	24	4210	296.79	12.98
51734345	2012	5	24	4220	297.03	12.99
51734345	2012	5	24	4230	296.64	12.98
51734345	2012	5	24	4240	297.13	12.99
51734345	2012	5	24	4250	297.3	12.98
51734345	2012	5	24	4260	296.64	12.98
51734345	2012	5	24	4270	295.52	12.98
51734345	2012	5	24	4280	294.58	12.97
51734345	2012	5	24	4290	293.55	12.97
51734345	2012	5	24	4300	292.39	12.96
51734345	2012	5	24	4310	291.39	12.96
51734345	2012	5	24	4320	291.51	12.96
51734345	2012	5	24	4330	291.73	12.97
51734345	2012	5	24	4340	291.8	12.96
51734345	2012	5	24	4350	291.96	12.97
51734345	2012	5	24	4360	291.92	12.97
51734345	2012	5	24	4370	291.99	12.98
51734345	2012	5	24	4380	291.72	12.97
51734345	2012	5	24	4390	291.05	12.97

51734345	2012	5	24	4400	289.8	12.96
51734345	2012	5	24	4410	288.79	12.96
51734345	2012	5	24	4420	287.88	12.96
51734345	2012	5	24	4430	287.09	12.96
51734345	2012	5	24	4440	286.17	12.96
51734345	2012	5	24	4450	285.61	12.96
51734345	2012	5	24	4460	285.8	12.96
51734345	2012	5	24	4470	285.67	12.96
51734345	2012	5	24	4480	285.6	12.96
51734345	2012	5	24	4490	285.73	12.96
51734345	2012	5	24	4500	285.34	12.96
51734345	2012	5	24	4510	285.7	12.96
51734345	2012	5	24	4520	285.48	12.96
51734345	2012	5	24	4530	285.6	12.96
51734345	2012	5	24	4540	285.26	12.96
51734345	2012	5	24	4550	285.18	12.96
51734345	2012	5	24	4560	285.4	12.96
51734345	2012	5	24	4570	285.61	12.97
51734345	2012	5	24	4580	285.57	12.97
51734345	2012	5	24	4590	285.57	12.98
51734345	2012	5	24	4600	285.34	12.98
51734345	2012	5	24	4610	285.44	12.97
51734345	2012	5	24	4620	285.48	12.98
51734345	2012	5	24	4630	285.3	12.98
51734345	2012	5	24	4640	285.39	12.97
51734345	2012	5	24	4650	285.44	12.98
51734345	2012	5	24	4660	284.9	12.98
51734345	2012	5	24	4670	285.05	12.99
51734345	2012	5	24	4680	284.93	12.98
51734345	2012	5	24	4690	284.76	12.98
51734345	2012	5	24	4700	285.26	12.99
51734345	2012	5	24	4710	284.68	12.99
51734345	2012	5	24	4720	283	12.98
51734345	2012	5	24	4730	282.06	12.98
51734345	2012	5	24	4740	282.01	12.98
51734345	2012	5	24	4750	280.82	12.97
51734345	2012	5	24	4760	279.69	12.97
51734345	2012	5	24	4770	278.41	12.97
51734345	2012	5	24	4780	277.85	12.97
51734345	2012	5	24	4790	277.98	12.98
51734345	2012	5	24	4800	278.18	12.98
51734345	2012	5	24	4810	278.11	12.98
51734345	2012	5	24	4820	277.93	12.99
51734345	2012	5	24	4830	277.91	12.98
51734345	2012	5	24	4840	277.83	13
51734345	2012	5	24	4850	278.2	12.99
51734345	2012	5	24	4860	278	13

51734345	2012	5	24	4870	277.97	13
51734345	2012	5	24	4880	278.17	13
51734345	2012	5	24	4890	278.11	13
51734345	2012	5	24	4900	277.96	13
51734345	2012	5	24	4910	277.54	13
51734345	2012	5	24	4920	277.28	13
51734345	2012	5	24	4930	276.92	13
51734345	2012	5	24	4940	276.75	13.01
51734345	2012	5	24	4950	276.92	13
51734345	2012	5	24	4960	277.28	13.01
51734345	2012	5	24	4970	277.21	13.01
51734345	2012	5	24	4980	276.44	13.01
51734345	2012	5	24	4990	275.87	13.01
51734345	2012	5	24	5000	275.32	13.01
51734345	2012	5	24	5010	275.06	13.01
51734345	2012	5	24	5020	275.48	13.01
51734345	2012	5	24	5030	276.11	13.01
51734345	2012	5	24	5040	276.02	13.02
51734345	2012	5	24	5050	276.28	13.02
51734345	2012	5	24	5060	276.69	13.02
51734345	2012	5	24	5070	276.54	13.02
51734345	2012	5	24	5080	277.52	13.02
51734345	2012	5	24	5090	279.52	13.03
51734345	2012	5	24	5100	280.45	13.03
51734345	2012	5	24	5110	281.04	13.02
51734345	2012	5	24	5120	282.19	13.03
51734345	2012	5	24	5130	282.98	13.03
51734345	2012	5	24	5140	283.98	13.03
51734345	2012	5	24	5150	285.07	13.03
51734345	2012	5	24	5160	286.53	13.03
51734345	2012	5	24	5170	287.79	13.04
51734345	2012	5	24	5180	288.75	13.04
51734345	2012	5	24	5190	290.38	13.04
51734345	2012	5	24	5200	291.72	13.04
51734345	2012	5	24	5210	292.83	13.04
51734345	2012	5	24	5220	294.36	13.05
51734345	2012	5	24	5230	295.72	13.06
51734345	2012	5	24	5240	296.46	13.06
51734345	2012	5	24	5250	297.61	13.05
51734345	2012	5	24	5260	298.86	13.06
51734345	2012	5	24	5270	299.79	13.07
51734345	2012	5	24	5280	300.42	13.07
51734345	2012	5	24	5290	301.27	13.06
51734345	2012	5	24	5300	301.95	13.07
51734345	2012	5	24	5310	302.41	13.07
51734345	2012	5	24	5320	302.83	13.07
51734345	2012	5	24	5330	302.88	13.06

51734345	2012	5	24	5340	302.82	13.06
51734345	2012	5	24	5350	302.94	13.05
51734345	2012	5	24	5360	302.79	13.05
51734345	2012	5	24	5370	303.07	13.05
51734345	2012	5	24	5380	303.29	13.04
51734345	2012	5	24	5390	302.97	13.05
51734345	2012	5	24	5400	303.21	13.04
51734345	2012	5	24	5410	303.15	13.04
51734345	2012	5	24	5420	303.09	13.04
51734345	2012	5	24	5430	303.14	13.05
51734345	2012	5	24	5440	303.05	13.04
51734345	2012	5	24	5450	303.25	13.04
51734345	2012	5	24	5460	302.95	13.03
51734345	2012	5	24	5470	302.75	13.04
51734345	2012	5	24	5480	302.88	13.03
51734345	2012	5	24	5490	303.1	13.04
51734345	2012	5	24	5500	303.21	13.04
51734345	2012	5	24	5510	302.59	13.03
51734345	2012	5	24	5520	301.67	13.03
51734345	2012	5	24	5530	301.71	13.03
51734345	2012	5	24	5540	301.41	13.03
51734345	2012	5	24	5550	301.19	13.03
51734345	2012	5	24	5560	300.29	13.03
51734345	2012	5	24	5570	300.44	13.03
51734345	2012	5	24	5580	299.9	13.02
51734345	2012	5	24	5590	299.54	13.02
51734345	2012	5	24	5600	298.77	13.03
51734345	2012	5	24	5610	298.64	13.02
51734345	2012	5	24	5620	298.35	13.02
51734345	2012	5	24	5630	297.17	13.01
51734345	2012	5	24	5640	296.67	13.01
51734345	2012	5	24	5650	296.98	13.02
51734345	2012	5	24	5660	296.87	13.02
51734345	2012	5	24	5670	296.86	13.02
51734345	2012	5	24	5680	296.31	13.01
51734345	2012	5	24	5690	295.75	13.01
51734345	2012	5	24	5700	295.71	13.02
51734345	2012	5	24	5710	295.57	13.02
51734345	2012	5	24	5720	295.41	13.02
51734345	2012	5	24	5730	295.27	13.02
51734345	2012	5	24	5740	295.36	13.02
51734345	2012	5	24	5750	295.4	13.02
51734345	2012	5	24	5760	295.12	13.02
51734345	2012	5	24	5770	295.1	13.03
51734345	2012	5	24	5780	294.94	13.02
51734345	2012	5	24	5790	294.84	13.02
51734345	2012	5	24	5800	294.99	13.03

51734345	2012	5	24	5810	294.33	13.02
51734345	2012	5	24	5820	293.37	13.02
51734345	2012	5	24	5830	293.13	13.01
51734345	2012	5	24	5840	293.49	13.02
51734345	2012	5	24	5850	292.82	13.01
51734345	2012	5	24	5860	292.1	13.01
51734345	2012	5	24	5870	291.48	13.01
51734345	2012	5	24	5880	291.1	13.01
51734345	2012	5	24	5890	291.08	13.02
51734345	2012	5	24	5900	289.77	13
51734345	2012	5	24	5910	288.91	13.01
51734345	2012	5	24	5920	289.15	13
51734345	2012	5	24	5930	289.44	13.02
51734345	2012	5	24	5940	289.53	13.02
51734345	2012	5	24	5950	290.02	13.02
51734345	2012	5	24	5960	289.65	13.02
51734345	2012	5	24	5970	288.2	13.01
51734345	2012	5	24	5980	287.59	13.02
51734345	2012	5	24	5990	287.52	13.02
51734345	2012	5	24	6000	287.04	13.02
51734345	2012	5	24	6010	286.42	13.01
51734345	2012	5	24	6020	286.41	13.02
51734345	2012	5	24	6030	285.93	13.01
51734345	2012	5	24	6040	285.82	13.01
51734345	2012	5	24	6050	286.18	13.01
51734345	2012	5	24	6060	286.3	13.02
51734345	2012	5	24	6070	286.56	13.02
51734345	2012	5	24	6080	286.26	13.02
51734345	2012	5	24	6090	285.74	13.02
51734345	2012	5	24	6100	285.63	13.02
51734345	2012	5	24	6110	284.66	13.02
51734345	2012	5	24	6120	283.83	13.02
51734345	2012	5	24	6130	283.72	13.02
51734345	2012	5	24	6140	283.58	13.02
51734345	2012	5	24	6150	283.58	13.02
51734345	2012	5	24	6160	283.82	13.02
51734345	2012	5	24	6170	283.89	13.03
51734345	2012	5	24	6180	283.82	13.03
51734345	2012	5	24	6190	283.71	13.02
51734345	2012	5	24	6200	283.72	13.03
51734345	2012	5	24	6210	283.97	13.03
51734345	2012	5	24	6220	283.42	13.03
51734345	2012	5	24	6230	282.48	13.02
51734345	2012	5	24	6240	282.16	13.02
51734345	2012	5	24	6250	282.28	13.02
51734345	2012	5	24	6260	282.82	13.03
51734345	2012	5	24	6270	283.24	13.03

51734345	2012	5	24	6280	283.45	13.03
51734345	2012	5	24	6290	283.2	13.03
51734345	2012	5	24	6300	282.88	13.03
51734345	2012	5	24	6310	283.18	13.03
51734345	2012	5	24	6320	283.29	13.03
51734345	2012	5	24	6330	283.14	13.03
51734345	2012	5	24	6340	282.89	13.03
51734345	2012	5	24	6350	283.1	13.03
51734345	2012	5	24	6360	283.28	13.04
51734345	2012	5	24	6370	283.19	13.04
51734345	2012	5	24	6380	283.22	13.04
51734345	2012	5	24	6390	283.23	13.05
51734345	2012	5	24	6400	283.19	13.04
51734345	2012	5	24	6410	283.2	13.05
51734345	2012	5	24	6420	283.01	13.05
51734345	2012	5	24	6430	282.9	13.06
51734345	2012	5	24	6440	282.58	13.06
51734345	2012	5	24	6450	282.49	13.06
51734345	2012	5	24	6460	282.57	13.06
51734345	2012	5	24	6470	282.68	13.06
51734345	2012	5	24	6480	282.75	13.06
51734345	2012	5	24	6490	282.43	13.06
51734345	2012	5	24	6500	282.53	13.06
51734345	2012	5	24	6510	282.73	13.06
51734345	2012	5	24	6520	282.9	13.07
51734345	2012	5	24	6530	283.15	13.07
51734345	2012	5	24	6540	283.51	13.07
51734345	2012	5	24	6550	284.01	13.07
51734345	2012	5	24	6560	284.56	13.07
51734345	2012	5	24	6570	284.95	13.07
51734345	2012	5	24	6580	284.39	13.06
51734345	2012	5	24	6590	282.98	13.07
51734345	2012	5	24	6600	282.38	13.06
51734345	2012	5	24	6610	282.68	13.06
51734345	2012	5	24	6620	283.23	13.07
51734345	2012	5	24	6630	282.62	13.06
51734345	2012	5	24	6640	281.75	13.06
51734345	2012	5	24	6650	282.02	13.07
51734345	2012	5	24	6660	281.94	13.07
51734345	2012	5	24	6670	281.03	13.07
51734345	2012	5	24	6680	279.91	13.07
51734345	2012	5	24	6690	279.65	13.07
51734345	2012	5	24	6700	279.58	13.07
51734345	2012	5	24	6710	279.06	13.07
51734345	2012	5	24	6720	278.88	13.07
51734345	2012	5	24	6730	279.21	13.08
51734345	2012	5	24	6740	278.32	13.07

51734345	2012	5	24	6750	277.87	13.07
51734345	2012	5	24	6760	276.72	13.07
51734345	2012	5	24	6770	275.57	13.07
51734345	2012	5	24	6780	274.38	13.07
51734345	2012	5	24	6790	273.66	13.08
51734345	2012	5	24	6800	273.09	13.09
51734345	2012	5	24	6810	272.14	13.1
51734345	2012	5	24	6820	270.96	13.1
51734345	2012	5	24	6830	270.51	13.1
51734345	2012	5	24	6840	269.7	13.12
51734345	2012	5	24	6850	268.67	13.12
51734345	2012	5	24	6860	268.29	13.14
51734345	2012	5	24	6870	267.11	13.14
51734345	2012	5	24	6880	265.77	13.17
51734345	2012	5	24	6890	263.8	13.17
51734345	2012	5	24	6900	262.25	13.17
51734345	2012	5	24	6910	260.35	13.17
51734345	2012	5	24	6920	258.38	13.18
51734345	2012	5	24	6930	256.92	13.2
51734345	2012	5	24	6940	257.38	13.23
51734345	2012	5	24	6950	256.36	13.25
51734345	2012	5	24	6960	254.42	13.26
51734345	2012	5	24	6970	252.31	13.28
51734345	2012	5	24	6980	250.8	13.3
51734345	2012	5	24	6990	250.92	13.34
51734345	2012	5	24	7000	250.95	13.36
51734345	2012	5	24	7010	250.24	13.38
51734345	2012	5	24	7020	249.02	13.4
51734345	2012	5	24	7030	248.44	13.42
51734345	2012	5	24	7040	248.08	13.44
51734345	2012	5	24	7050	247.33	13.47
51734345	2012	5	24	7060	247.18	13.48
51734345	2012	5	24	7070	247.12	13.5
51734345	2012	5	24	7080	246.75	13.5
51734345	2012	5	24	7090	246.76	13.55
51734345	2012	5	24	7100	246.69	13.57
51734345	2012	5	24	7110	245.95	13.56
51734345	2012	5	24	7120	246.57	13.57
51734345	2012	5	24	7130	247.24	13.58
51734345	2012	5	24	7140	246.77	13.59
51734345	2012	5	24	7150	246.42	13.6
51734345	2012	5	24	7160	246.93	13.64
51734345	2012	5	24	7170	246.52	13.64
51734345	2012	5	24	7180	246.05	13.64
51734345	2012	5	24	7190	246.08	13.64
51734345	2012	5	24	7200	246.19	13.66
51734345	2012	5	24	7210	246.07	13.67

51734345	2012	5	24	7220	246.26	13.67
51734345	2012	5	24	7230	246.14	13.67
51734345	2012	5	24	7240	246	13.68
51734345	2012	5	24	7250	246.05	13.69
51734345	2012	5	24	7260	246.22	13.69
51734345	2012	5	24	7270	246.15	13.71
51734345	2012	5	24	7280	246.1	13.71
51734345	2012	5	24	7290	246.11	13.71
51734345	2012	5	24	7300	245.74	13.72
51734345	2012	5	24	7310	245.54	13.72
51734345	2012	5	24	7320	245.75	13.73
51734345	2012	5	24	7330	245.66	13.74
51734345	2012	5	24	7340	244.63	13.74
51734345	2012	5	24	7350	243.6	13.74
51734345	2012	5	24	7360	242.96	13.74
51734345	2012	5	24	7370	242.61	13.75
51734345	2012	5	24	7380	241.53	13.75
51734345	2012	5	24	7390	240.82	13.75
51734345	2012	5	24	7400	240.81	13.75
51734345	2012	5	24	7410	240.2	13.75
51734345	2012	5	24	7420	239.5	13.75
51734345	2012	5	24	7430	239.27	13.75
51734345	2012	5	24	7440	238.68	13.75
51734345	2012	5	24	7450	238.12	13.75
51734345	2012	5	24	7460	237.77	13.76
51734345	2012	5	24	7470	237.65	13.77
51734345	2012	5	24	7480	236.44	13.76
51734345	2012	5	24	7490	235.5	13.77
51734345	2012	5	24	7500	234.67	13.78
51734345	2012	5	24	7510	233.69	13.78
51734345	2012	5	24	7520	232.67	13.79
51734345	2012	5	24	7530	231.98	13.85
51734345	2012	5	24	7540	231.19	13.86
51734345	2012	5	24	7550	230.57	13.89
51734345	2012	5	24	7560	230.01	13.9
51734345	2012	5	24	7570	229.3	13.92
51734345	2012	5	24	7580	229.1	13.93
51734345	2012	5	24	7590	229	13.96
51734345	2012	5	24	7600	228.03	13.98
51734345	2012	5	24	7610	227.68	13.99
51734345	2012	5	24	7620	227.61	14.01
51734345	2012	5	24	7630	228.51	14.03
51734345	2012	5	24	7640	228.59	14.07
51734345	2012	5	24	7650	228.15	14.08
51734345	2012	5	24	7660	228.11	14.09
51734345	2012	5	24	7670	228.04	14.1
51734345	2012	5	24	7680	226.54	14.12

51734345	2012	5	24	7690	226.08	14.14
51734345	2012	5	24	7700	226.64	14.16
51734345	2012	5	24	7710	226.69	14.17
51734345	2012	5	24	7720	226.55	14.18
51734345	2012	5	24	7730	226.65	14.19
51734345	2012	5	24	7740	226.66	14.21
51734345	2012	5	24	7750	226.27	14.21
51734345	2012	5	24	7760	226.22	14.22
51734345	2012	5	24	7770	226.39	14.23
51734345	2012	5	24	7780	226.43	14.24
51734345	2012	5	24	7790	225.91	14.25
51734345	2012	5	24	7800	225.11	14.26
51734345	2012	5	24	7810	224.47	14.28
51734345	2012	5	24	7820	224.07	14.28
51734345	2012	5	24	7830	223.84	14.28
51734345	2012	5	24	7840	223.73	14.29
51734345	2012	5	24	7850	223.66	14.3
51734345	2012	5	24	7860	223.7	14.31
51734345	2012	5	24	7870	223.75	14.32
51734345	2012	5	24	7880	223.85	14.32
51734345	2012	5	24	7890	224.04	14.32
51734345	2012	5	24	7900	223.94	14.33
51734345	2012	5	24	7910	222.95	14.34
51734345	2012	5	24	7920	222.87	14.35
51734345	2012	5	24	7930	223.26	14.35
51734345	2012	5	24	7940	223.65	14.36
51734345	2012	5	24	7950	224.01	14.37
51734345	2012	5	24	7960	223.79	14.36
51734345	2012	5	24	7970	223.6	14.37
51734345	2012	5	24	7980	222.45	14.38
51734345	2012	5	24	7990	221.71	14.37
51734345	2012	5	24	8000	221.03	14.37
51734345	2012	5	24	8010	220.51	14.38
51734345	2012	5	24	8020	220.03	14.37
51734345	2012	5	24	8030	219.32	14.37
51734345	2012	5	24	8040	218.16	14.37
51734345	2012	5	24	8050	217.43	14.38
51734345	2012	5	24	8060	217.12	14.37
51734345	2012	5	24	8070	217.09	14.38
51734345	2012	5	24	8080	217.13	14.38
51734345	2012	5	24	8090	217.23	14.38
51734345	2012	5	24	8100	217.06	14.39
51734345	2012	5	24	8110	216.98	14.39
51734345	2012	5	24	8120	217.15	14.39
51734345	2012	5	24	8130	215.86	14.4
51734345	2012	5	24	8140	214.85	14.4
51734345	2012	5	24	8150	213.86	14.41

51734345	2012	5	24	8160	213.64	14.42
51734345	2012	5	24	8170	213.54	14.42
51734345	2012	5	24	8180	212.88	14.43
51734345	2012	5	24	8190	212.77	14.44
51734345	2012	5	24	8200	212.73	14.44
51734345	2012	5	24	8210	211.98	14.45
51734345	2012	5	24	8220	211.18	14.46
51734345	2012	5	24	8230	211.99	14.47
51734345	2012	5	24	8240	212.04	14.49
51734345	2012	5	24	8250	211.81	14.5
51734345	2012	5	24	8260	212.21	14.5
51734345	2012	5	24	8270	211.43	14.5
51734345	2012	5	24	8280	210.35	14.52
51734345	2012	5	24	8290	209.5	14.53
51734345	2012	5	24	8300	209.46	14.54
51734345	2012	5	24	8310	208.94	14.55
51734345	2012	5	24	8320	208.22	14.56
51734345	2012	5	24	8330	208.23	14.58
51734345	2012	5	24	8340	208.26	14.59
51734345	2012	5	24	8350	208.36	14.6
51734345	2012	5	24	8360	208.2	14.6
51734345	2012	5	24	8370	207.53	14.62
51734345	2012	5	24	8380	207.52	14.63
51734345	2012	5	24	8390	207.24	14.64
51734345	2012	5	24	8400	206.96	14.66
51734345	2012	5	24	8410	206.68	14.67
51734345	2012	5	24	8420	206.72	14.71
51734345	2012	5	24	8430	206.64	14.71
51734345	2012	5	24	8440	206.49	14.71
51734345	2012	5	24	8450	206.11	14.73
51734345	2012	5	24	8460	206.18	14.74
51734345	2012	5	24	8470	205.41	14.75
51734345	2012	5	24	8480	204.06	14.75
51734345	2012	5	24	8490	202.57	14.78
51734345	2012	5	24	8500	201.65	14.8
51734345	2012	5	24	8510	201.26	14.84
51734345	2012	5	24	8520	200.89	14.94
51734345	2012	5	24	8530	200.2	14.96
51734345	2012	5	24	8540	199.38	14.98
51734345	2012	5	24	8550	198.5	15
51734345	2012	5	24	8560	197.92	15.01
51734345	2012	5	24	8570	197.63	15.03
51734345	2012	5	24	8580	197.25	15.04
51734345	2012	5	24	8590	196.34	15.07
51734345	2012	5	24	8600	195.52	15.08
51734345	2012	5	24	8610	194.86	15.1
51734345	2012	5	24	8620	194.51	15.15

51734345	2012	5	24	8630	193.43	15.17
51734345	2012	5	24	8640	192.74	15.21
51734345	2012	5	24	8650	192.64	15.25
51734345	2012	5	24	8660	192.38	15.3
51734345	2012	5	24	8670	191.54	15.33
51734345	2012	5	24	8680	190.2	15.38
51734345	2012	5	24	8690	189.11	15.43
51734345	2012	5	24	8700	188.97	15.51
51734345	2012	5	24	8710	189.12	15.6
51734345	2012	5	24	8720	188.31	15.68
51734345	2012	5	24	8730	187.43	15.76
51734345	2012	5	24	8740	186.93	15.83
51734345	2012	5	24	8750	187.17	15.95
51734345	2012	5	24	8760	187.57	16
51734345	2012	5	24	8770	187.72	16.07
51734345	2012	5	24	8780	187.74	16.15
51734345	2012	5	24	8790	188.07	16.21
51734345	2012	5	24	8800	188	16.26
51734345	2012	5	24	8810	187.8	16.3
51734345	2012	5	24	8820	187.69	16.35
51734345	2012	5	24	8830	187.63	16.41
51734345	2012	5	24	8840	187.37	16.48
51734345	2012	5	24	8850	186.29	16.51
51734345	2012	5	24	8860	184.99	16.54
51734345	2012	5	24	8870	184.17	16.57
51734345	2012	5	24	8880	183.97	16.6
51734345	2012	5	24	8890	183.39	16.64
51734345	2012	5	24	8900	183.24	16.67
51734345	2012	5	24	8910	183.22	16.68
51734345	2012	5	24	8920	182.61	16.69
51734345	2012	5	24	8930	181.19	16.72
51734345	2012	5	24	8940	180.66	16.78
51734345	2012	5	24	8950	179.68	16.83
51734345	2012	5	24	8960	179.15	16.85
51734345	2012	5	24	8970	178.72	16.87
51734345	2012	5	24	8980	178.66	16.9
51734345	2012	5	24	8990	178.55	16.92
51734345	2012	5	24	9000	177.51	16.97
51734345	2012	5	24	9010	176.71	16.98
51734345	2012	5	24	9020	176.84	17.01
51734345	2012	5	24	9030	176.86	17.04
51734345	2012	5	24	9040	177.09	17.07
51734345	2012	5	24	9050	176.77	17.09
51734345	2012	5	24	9060	176.7	17.1
51734345	2012	5	24	9070	176.78	17.14
51734345	2012	5	24	9080	176.9	17.17
51734345	2012	5	24	9090	176.3	17.18

51734345	2012	5	24	9100	176.16	17.21
51734345	2012	5	24	9110	175.55	17.22
51734345	2012	5	24	9120	174.74	17.24
51734345	2012	5	24	9130	174.74	17.26
51734345	2012	5	24	9140	174.98	17.28
51734345	2012	5	24	9150	175.33	17.29
51734345	2012	5	24	9160	175.71	17.3
51734345	2012	5	24	9170	175.88	17.32
51734345	2012	5	24	9180	175.36	17.32
51734345	2012	5	24	9190	175.04	17.34
51734345	2012	5	24	9200	175.14	17.35
51734345	2012	5	24	9210	174.73	17.36
51734345	2012	5	24	9220	174.46	17.38
51734345	2012	5	24	9230	174.53	17.38
51734345	2012	5	24	9240	174.71	17.4
51734345	2012	5	24	9250	174.78	17.4
51734345	2012	5	24	9260	173.59	17.41
51734345	2012	5	24	9270	172.46	17.42
51734345	2012	5	24	9280	170.94	17.44
51734345	2012	5	24	9290	170.22	17.45
51734345	2012	5	24	9300	169.86	17.48
51734345	2012	5	24	9310	169.44	17.5
51734345	2012	5	24	9320	168.76	17.54
51734345	2012	5	24	9330	168.72	17.58
51734345	2012	5	24	9340	169.03	17.6
51734345	2012	5	24	9350	169.26	17.63
51734345	2012	5	24	9360	169.3	17.65
51734345	2012	5	24	9370	169.23	17.67
51734345	2012	5	24	9380	169.28	17.69
51734345	2012	5	24	9390	169.3	17.71
51734345	2012	5	24	9400	169.25	17.75
51734345	2012	5	24	9410	169.23	17.76
51734345	2012	5	24	9420	168.36	17.78
51734345	2012	5	24	9430	167.44	17.8
51734345	2012	5	24	9440	166.91	17.82
51734345	2012	5	24	9450	166.51	17.85
51734345	2012	5	24	9460	166.86	17.86
51734345	2012	5	24	9470	167.16	17.89
51734345	2012	5	24	9480	166.53	17.9
51734345	2012	5	24	9490	167.02	17.91
51734345	2012	5	24	9500	167.98	17.92
51734345	2012	5	24	9510	168.3	17.92
51734345	2012	5	24	9520	169.52	17.92
51734345	2012	5	24	9530	170.33	17.93
51734345	2012	5	24	9540	171.48	17.93
51734345	2012	5	24	9550	172.45	17.93
51734345	2012	5	24	9560	172.85	17.92

51734345	2012	5	24	9570	172.95	17.91
51734345	2012	5	24	9580	173.33	17.91
51734345	2012	5	24	9590	173.53	17.9
51734345	2012	5	24	9600	173.68	17.9
51734345	2012	5	24	9610	173.57	17.9
51734345	2012	5	24	9620	173.61	17.89
51734345	2012	5	24	9630	173.57	17.87
51734345	2012	5	24	9640	173.56	17.87
51734345	2012	5	24	9650	173.59	17.85
51734345	2012	5	24	9660	172.99	17.85
51734345	2012	5	24	9670	172.5	17.85
51734345	2012	5	24	9680	172.45	17.82
51734345	2012	5	24	9690	172.8	17.8
51734345	2012	5	24	9700	173.14	17.78
51734345	2012	5	24	9710	173.4	17.75
51734345	2012	5	24	9720	173.08	17.74
51734345	2012	5	24	9730	173.06	17.73
51734345	2012	5	24	9740	173.14	17.73
51734345	2012	5	24	9750	173.51	17.72
51734345	2012	5	24	9760	173.99	17.71
51734345	2012	5	24	9770	174.46	17.7
51734345	2012	5	24	9780	174.74	17.68
51734345	2012	5	24	9790	175.16	17.67
51734345	2012	5	24	9800	175.53	17.65
51734345	2012	5	24	9810	175.42	17.64
51734345	2012	5	24	9820	175.54	17.64
51734345	2012	5	24	9830	176.1	17.62
51734345	2012	5	24	9840	176.28	17.62
51734345	2012	5	24	9850	176.27	17.61
51734345	2012	5	24	9860	176.47	17.6
51734345	2012	5	24	9870	176.58	17.59
51734345	2012	5	24	9880	176.42	17.58
51734345	2012	5	24	9890	175.19	17.57
51734345	2012	5	24	9900	173.91	17.57
51734345	2012	5	24	9910	172.25	17.56
51734345	2012	5	24	9920	171.09	17.56
51734345	2012	5	24	9930	170.82	17.53
51734345	2012	5	24	9940	169.87	17.53
51734345	2012	5	24	9950	170.04	17.57
51734345	2012	5	24	9960	170	17.57
51734345	2012	5	24	9970	169.89	17.6
51734345	2012	5	24	9980	169.05	17.61
51734345	2012	5	24	9990	168.41	17.62
51734345	2012	5	24	10000	167.78	17.64
51734345	2012	5	24	10010	166.57	17.7
51734345	2012	5	24	10020	165.29	17.71
51734345	2012	5	24	10030	164.93	17.76

51734345	2012	5	24	10040	164.62	17.77
51734345	2012	5	24	10050	163.2	17.79
51734345	2012	5	24	10060	161.97	17.83
51734345	2012	5	24	10070	160.28	17.85
51734345	2012	5	24	10080	158.86	17.87
51734345	2012	5	24	10090	158.1	17.9
51734345	2012	5	24	10100	156.75	17.93
51734345	2012	5	24	10110	156.11	17.96
51734345	2012	5	24	10120	155.51	17.99
51734345	2012	5	24	10130	155.46	18
51734345	2012	5	24	10140	154.96	18.01
51734345	2012	5	24	10150	155.03	18.03
51734345	2012	5	24	10160	154.57	18.04
51734345	2012	5	24	10170	153.48	18.05
51734345	2012	5	24	10180	152.92	18.07
51734345	2012	5	24	10190	152.33	18.09
51734345	2012	5	24	10200	151.93	18.1
51734345	2012	5	24	10210	151.9	18.13
51734345	2012	5	24	10220	152.31	18.14
51734345	2012	5	24	10230	152.16	18.15
51734345	2012	5	24	10240	151.84	18.17
51734345	2012	5	24	10250	152.09	18.2
51734345	2012	5	24	10260	152.15	18.22
51734345	2012	5	24	10270	151.84	18.24
51734345	2012	5	24	10280	151.54	18.25
51734345	2012	5	24	10290	151.36	18.25
51734345	2012	5	24	10300	151.1	18.28
51734345	2012	5	24	10310	151.3	18.3
51734345	2012	5	24	10320	151.47	18.31
51734345	2012	5	24	10330	151.18	18.31
51734345	2012	5	24	10340	151.56	18.31
51734345	2012	5	24	10350	151.46	18.32
51734345	2012	5	24	10360	151.22	18.33
51734345	2012	5	24	10370	151.22	18.35
51734345	2012	5	24	10380	151.45	18.35
51734345	2012	5	24	10390	150.97	18.35
51734345	2012	5	24	10400	149.44	18.37
51734345	2012	5	24	10410	147.51	18.39
51734345	2012	5	24	10420	146.44	18.4
51734345	2012	5	24	10430	145.66	18.41
51734345	2012	5	24	10440	145.15	18.41
51734345	2012	5	24	10450	145.02	18.42
51734345	2012	5	24	10460	144.87	18.42
51734345	2012	5	24	10470	143.47	18.43
51734345	2012	5	24	10480	142.75	18.44
51734345	2012	5	24	10490	141.35	18.44
51734345	2012	5	24	10500	140.83	18.46

51734345	2012	5	24	10510	140.04	18.46
51734345	2012	5	24	10520	138.99	18.47
51734345	2012	5	24	10530	137.24	18.49
51734345	2012	5	24	10540	136.19	18.5
51734345	2012	5	24	10550	135.35	18.53
51734345	2012	5	24	10560	134.89	18.55
51734345	2012	5	24	10570	134.06	18.58
51734345	2012	5	24	10580	133.46	18.61
51734345	2012	5	24	10590	131.87	18.64
51734345	2012	5	24	10600	130.63	18.67
51734345	2012	5	24	10610	130.41	18.68
51734345	2012	5	24	10620	129.17	18.71
51734345	2012	5	24	10630	128.21	18.72
51734345	2012	5	24	10640	127.37	18.75
51734345	2012	5	24	10650	126.49	18.77
51734345	2012	5	24	10660	125.57	18.81
51734345	2012	5	24	10670	125.1	18.85
51734345	2012	5	24	10680	124.75	18.86
51734345	2012	5	24	10690	124.36	18.89
51734345	2012	5	24	10700	124.16	18.91
51734345	2012	5	24	10710	124.47	18.92
51734345	2012	5	24	10720	124.47	18.95
51734345	2012	5	24	10730	124.18	18.97
51734345	2012	5	24	10740	123.86	19.01
51734345	2012	5	24	10750	123.82	19.03
51734345	2012	5	24	10760	123.56	19.07
51734345	2012	5	24	10770	123.62	19.09
51734345	2012	5	24	10780	123.69	19.12
51734345	2012	5	24	10790	123.41	19.15
51734345	2012	5	24	10800	123.06	19.2
51734345	2012	5	24	10810	122.95	19.21
51734345	2012	5	24	10820	122.74	19.22
51734345	2012	5	24	10830	122.94	19.24
51734345	2012	5	24	10840	122.93	19.25
51734345	2012	5	24	10850	122.51	19.26
51734345	2012	5	24	10860	121.81	19.28
51734345	2012	5	24	10870	120.7	19.3
51734345	2012	5	24	10880	118.59	19.33
51734345	2012	5	24	10890	116.45	19.39
51734345	2012	5	24	10900	114.72	19.45
51734345	2012	5	24	10910	112.64	19.52
51734345	2012	5	24	10920	111.1	19.59
51734345	2012	5	24	10930	109.42	19.67
51734345	2012	5	24	10940	107.56	19.75
51734345	2012	5	24	10950	106.53	19.8
51734345	2012	5	24	10960	104.86	19.92
51734345	2012	5	24	10970	103.62	20.03

51734345	2012	5	24	10980	102.49	20.13
51734345	2012	5	24	10990	101.01	20.27
51734345	2012	5	24	11000	99.23	20.32
51734345	2012	5	24	11010	97.93	20.37
51734345	2012	5	24	11020	96.71	20.46
51734345	2012	5	24	11030	94.86	20.54
51734345	2012	5	24	11040	93.57	20.65
51734345	2012	5	24	11050	91.86	20.75
51734345	2012	5	24	11060	90.04	20.83
51734345	2012	5	24	11070	87.43	20.92
51734345	2012	5	24	11080	86.55	21.04
51734345	2012	5	24	11090	86.53	21.16
51734345	2012	5	24	11100	85.06	21.21
51734345	2012	5	24	11110	84.55	21.28
51734345	2012	5	24	11120	84.18	21.59
51734345	2012	5	24	11130	83.28	21.85
51734345	2012	5	24	11140	82.68	21.91
51734345	2012	5	24	11150	82.06	22.1
51734345	2012	5	24	11160	81.29	22.14
51734345	2012	5	24	11170	80.69	22.22
51734345	2012	5	24	11180	80.14	22.35
51734345	2012	5	24	11190	79.3	22.46
51734345	2012	5	24	11200	78.42	22.64
51734345	2012	5	24	11210	77	22.69
51734345	2012	5	24	11220	76.07	22.76
51734345	2012	5	24	11230	75.48	22.85
51734345	2012	5	24	11240	74.52	22.92
51734345	2012	5	24	11250	73.62	22.99
51734345	2012	5	24	11260	72.76	23.05
51734345	2012	5	24	11270	71.57	23.19
51734345	2012	5	24	11280	69.61	23.27
51734345	2012	5	24	11290	68.64	23.39
51734345	2012	5	24	11300	67.29	23.44
51734345	2012	5	24	11310	66.88	23.58
51734345	2012	5	24	11320	66.74	23.7
51734345	2012	5	24	11330	66.42	23.95
51734345	2012	5	24	11340	66.56	24.21
51734345	2012	5	24	11350	66.75	24.3
51734345	2012	5	24	11360	66	24.42
51734345	2012	5	24	11370	64.84	24.63
51734345	2012	5	24	11380	64.3	24.8
51734345	2012	5	24	11390	63.54	24.85
51734345	2012	5	24	11400	62.75	24.96
51734345	2012	5	24	11410	62.19	25.07
51734345	2012	5	24	11420	62.25	25.15
51734345	2012	5	24	11430	62.25	25.25
51734345	2012	5	24	11440	62.55	25.35

51734345	2012	5	24	11450	62.72	25.46
51734345	2012	5	24	11460	62.77	25.57
51734345	2012	5	24	11470	63.23	25.62
51734345	2012	5	24	11480	63.88	25.67
51734345	2012	5	24	11490	64.42	25.72
51734345	2012	5	24	11500	64.49	25.79
51734345	2012	5	24	11510	64.11	25.84
51734345	2012	5	24	11520	63.66	25.89
51734345	2012	5	24	11530	62.47	25.9
51734345	2012	5	24	11540	60.9	25.95
51734345	2012	5	24	11550	59.43	25.99
51734345	2012	5	24	11560	57.14	26.06
51734345	2012	5	24	11570	54.57	26.12
51734345	2012	5	24	11580	51.99	26.21
51734345	2012	5	24	11590	49.74	26.24
51734345	2012	5	24	11600	48.2	26.28
51734345	2012	5	24	11610	45.48	26.34
51734345	2012	5	24	11620	43.37	26.4
51734345	2012	5	24	11630	41.73	26.45
51734345	2012	5	24	11640	40.51	26.46
51734345	2012	5	24	11650	38.1	26.51
51734345	2012	5	24	11660	35.49	26.55
51734345	2012	5	24	11670	32.71	26.6
51734345	2012	5	24	11680	30.13	26.67
51734345	2012	5	24	11690	27	26.73
51734345	2012	5	24	11700	23.54	26.82
51734345	2012	5	24	11710	20.91	26.9
51734345	2012	5	24	11720	18.61	26.95
51734345	2012	5	24	11730	16.03	26.97
51734345	2012	5	24	11740	13.65	27.03
51734345	2012	5	24	11750	11.14	27.08
51734345	2012	5	24	11760	10.88	27.1
51734345	2012	5	24	11770	10	27.11
51734345	2012	5	24	11780	8.53	27.14
51734345	2012	5	24	11790	6.77	27.15
51734345	2012	5	24	11800	5.13	27.17
51734345	2012	5	24	11810	3.83	27.22
51734345	2012	5	24	11820	2.96	27.26
51734345	2012	5	24	11830	2.5	27.28
51734345	2012	5	24	11840	2.34	27.31
51734345	2012	5	24	11850	2.55	27.33
51734345	2012	5	24	11860	1.93	27.35
51734345	2012	5	24	11870	0.7	27.37
51734345	2012	5	24	11880	0.64	27.38
51734345	2012	5	24	11890	0.69	27.38
51734345	2012	5	24	11900	0.64	27.42
51734345	2012	5	24	11910	0.63	27.45

51734345	2012	5	24	11920	0.71	27.49
51734345	2012	5	24	11930	0.63	27.5
51734345	2012	5	24	11940	0.67	27.53
51734345	2012	5	24	11950	0.64	27.53
51734345	2012	5	24	11960	0.71	27.54
51734345	2012	5	24	11970	0.68	27.56
51734345	2012	5	24	11980	0.69	27.57
51734345	2012	5	24	11990	0.67	27.57
51734345	2012	5	24	12000	0.64	27.59
51734345	2012	5	24	12010	0.7	27.59
51750865	2012	5	24	0	1.32	27.88
51750865	2012	5	24	10	1.89	27.89
51750865	2012	5	24	20	2.54	27.87
51750865	2012	5	24	30	2.58	27.88
51750865	2012	5	24	40	2.75	27.87
51750865	2012	5	24	50	2.73	27.88
51750865	2012	5	24	60	2.44	27.87
51750865	2012	5	24	70	2.67	27.87
51750865	2012	5	24	80	2.73	27.85
51750865	2012	5	24	90	3.49	27.82
51750865	2012	5	24	100	4.51	27.79
51750865	2012	5	24	110	5.69	27.77
51750865	2012	5	24	120	6.17	27.75
51750865	2012	5	24	130	6.74	27.75
51750865	2012	5	24	140	7.66	27.72
51750865	2012	5	24	150	8.57	27.69
51750865	2012	5	24	160	9.13	27.67
51750865	2012	5	24	170	10.76	27.65
51750865	2012	5	24	180	12.55	27.63
51750865	2012	5	24	190	14.11	27.6
51750865	2012	5	24	200	15.44	27.6
51750865	2012	5	24	210	16.66	27.57
51750865	2012	5	24	220	17.32	27.57
51750865	2012	5	24	230	18.32	27.55
51750865	2012	5	24	240	19.51	27.53
51750865	2012	5	24	250	21.5	27.5
51750865	2012	5	24	260	24.17	27.48
51750865	2012	5	24	270	26.76	27.46
51750865	2012	5	24	280	29.01	27.44
51750865	2012	5	24	290	31.34	27.42
51750865	2012	5	24	300	34.18	27.39
51750865	2012	5	24	310	36.61	27.38
51750865	2012	5	24	320	39.01	27.35
51750865	2012	5	24	330	41.94	27.32
51750865	2012	5	24	340	44.43	27.29
51750865	2012	5	24	350	46.8	27.28
51750865	2012	5	24	360	48.45	27.26

51750865	2012	5	24	370	49.64	27.25
51750865	2012	5	24	380	51.27	27.23
51750865	2012	5	24	390	52.74	27.21
51750865	2012	5	24	400	53.36	27.19
51750865	2012	5	24	410	52.91	27.2
51750865	2012	5	24	420	52.01	27.2
51750865	2012	5	24	430	52.54	27.19
51750865	2012	5	24	440	53.8	27.17
51750865	2012	5	24	450	54.14	27.16
51750865	2012	5	24	460	54.24	27.16
51750865	2012	5	24	470	53.16	27.16
51750865	2012	5	24	480	52.58	27.17
51750865	2012	5	24	490	52.5	27.16
51750865	2012	5	24	500	52.73	27.14
51750865	2012	5	24	510	52.65	27.14
51750865	2012	5	24	520	52.62	27.14
51750865	2012	5	24	530	52.7	27.14
51750865	2012	5	24	540	53.27	27.13
51750865	2012	5	24	550	53.01	27.12
51750865	2012	5	24	560	52.52	27.12
51750865	2012	5	24	570	53.03	27.1
51750865	2012	5	24	580	54.11	27.09
51750865	2012	5	24	590	54.03	27.09
51750865	2012	5	24	600	52.93	27.1
51750865	2012	5	24	610	52.03	27.1
51750865	2012	5	24	620	52.37	27.1
51750865	2012	5	24	630	52.23	27.09
51750865	2012	5	24	640	51.72	27.09
51750865	2012	5	24	650	52.03	27.1
51750865	2012	5	24	660	52.58	27.09
51750865	2012	5	24	670	53.06	27.09
51750865	2012	5	24	680	53.49	27.07
51750865	2012	5	24	690	52.85	27.08
51750865	2012	5	24	700	53.27	27.08
51750865	2012	5	24	710	53.61	27.08
51750865	2012	5	24	720	53.51	27.08
51750865	2012	5	24	730	53.79	27.08
51750865	2012	5	24	740	53.72	27.08
51750865	2012	5	24	750	54	27.08
51750865	2012	5	24	760	53.93	27.09
51750865	2012	5	24	770	54.02	27.08
51750865	2012	5	24	780	54.35	27.08
51750865	2012	5	24	790	54.12	27.09
51750865	2012	5	24	800	54.12	27.08
51750865	2012	5	24	810	54.34	27.07
51750865	2012	5	24	820	54.11	27.09
51750865	2012	5	24	830	54.14	27.08

51750865	2012	5	24	840	54.23	27.09
51750865	2012	5	24	850	54.31	27.08
51750865	2012	5	24	860	53.83	27.08
51750865	2012	5	24	870	53.93	27.07
51750865	2012	5	24	880	54.02	27.07
51750865	2012	5	24	890	54.06	27.07
51750865	2012	5	24	900	54.12	27.07
51750865	2012	5	24	910	54.16	27.07
51750865	2012	5	24	920	54.28	27.07
51750865	2012	5	24	930	54.69	27.07
51750865	2012	5	24	940	54.27	27.07
51750865	2012	5	24	950	53.99	27.07
51750865	2012	5	24	960	54.07	27.06
51750865	2012	5	24	970	54.51	27.06
51750865	2012	5	24	980	54.5	27.06
51750865	2012	5	24	990	53.53	27.07
51750865	2012	5	24	1000	52.66	27.07
51750865	2012	5	24	1010	51.78	27.07
51750865	2012	5	24	1020	50.53	27.08
51750865	2012	5	24	1030	49.19	27.1
51750865	2012	5	24	1040	48.02	27.1
51750865	2012	5	24	1050	46.95	27.12
51750865	2012	5	24	1060	46.25	27.14
51750865	2012	5	24	1070	46	27.14
51750865	2012	5	24	1080	44.99	27.15
51750865	2012	5	24	1090	44.43	27.16
51750865	2012	5	24	1100	44.15	27.17
51750865	2012	5	24	1110	44.4	27.18
51750865	2012	5	24	1120	45.53	27.17
51750865	2012	5	24	1130	45.34	27.19
51750865	2012	5	24	1140	44.56	27.19
51750865	2012	5	24	1150	44.52	27.2
51750865	2012	5	24	1160	44.62	27.2
51750865	2012	5	24	1170	44.68	27.21
51750865	2012	5	24	1180	44.36	27.21
51750865	2012	5	24	1190	44.3	27.21
51750865	2012	5	24	1200	44.21	27.22
51750865	2012	5	24	1210	44.41	27.21
51750865	2012	5	24	1220	44.61	27.22
51750865	2012	5	24	1230	44.72	27.22
51750865	2012	5	24	1240	44.28	27.22
51750865	2012	5	24	1250	43.69	27.23
51750865	2012	5	24	1260	43.43	27.24
51750865	2012	5	24	1270	43.37	27.24
51750865	2012	5	24	1280	44.25	27.25
51750865	2012	5	24	1290	45.27	27.23
51750865	2012	5	24	1300	45.52	27.23

51750865	2012	5	24	1310	45.83	27.24
51750865	2012	5	24	1320	46.2	27.24
51750865	2012	5	24	1330	46.71	27.23
51750865	2012	5	24	1340	47.27	27.23
51750865	2012	5	24	1350	47.79	27.22
51750865	2012	5	24	1360	48.2	27.23
51750865	2012	5	24	1370	48.07	27.22
51750865	2012	5	24	1380	47.87	27.23
51750865	2012	5	24	1390	47.7	27.23
51750865	2012	5	24	1400	47.37	27.25
51750865	2012	5	24	1410	47.38	27.24
51750865	2012	5	24	1420	47.37	27.24
51750865	2012	5	24	1430	46.95	27.25
51750865	2012	5	24	1440	47.14	27.25
51750865	2012	5	24	1450	46.96	27.25
51750865	2012	5	24	1460	46.6	27.25
51750865	2012	5	24	1470	46.23	27.25
51750865	2012	5	24	1480	46.72	27.24
51750865	2012	5	24	1490	47.34	27.25
51750865	2012	5	24	1500	47.29	27.25
51750865	2012	5	24	1510	47.72	27.25
51750865	2012	5	24	1520	47.89	27.24
51750865	2012	5	24	1530	47.9	27.25
51750865	2012	5	24	1540	47.72	27.25
51750865	2012	5	24	1550	47.76	27.24
51750865	2012	5	24	1560	47.72	27.25
51750865	2012	5	24	1570	47.63	27.25
51750865	2012	5	24	1580	47.32	27.25
51750865	2012	5	24	1590	46.85	27.26
51750865	2012	5	24	1600	46.6	27.25
51750865	2012	5	24	1610	46.28	27.26
51750865	2012	5	24	1620	46.23	27.26
51750865	2012	5	24	1630	46.19	27.26
51750865	2012	5	24	1640	46.41	27.26
51750865	2012	5	24	1650	47.3	27.25
51750865	2012	5	24	1660	47.91	27.25
51750865	2012	5	24	1670	48.5	27.25
51750865	2012	5	24	1680	48.51	27.25
51750865	2012	5	24	1690	48.1	27.25
51750865	2012	5	24	1700	49.12	27.25
51750865	2012	5	24	1710	50.63	27.24
51750865	2012	5	24	1720	52.15	27.22
51750865	2012	5	24	1730	53.39	27.22
51750865	2012	5	24	1740	53.88	27.21
51750865	2012	5	24	1750	54.63	27.21
51750865	2012	5	24	1760	54.27	27.22
51750865	2012	5	24	1770	53.77	27.21

51750865	2012	5	24	1780	53	27.21
51750865	2012	5	24	1790	52.2	27.22
51750865	2012	5	24	1800	50.84	27.24
51750865	2012	5	24	1810	50.59	27.24
51750865	2012	5	24	1820	51.22	27.22
51750865	2012	5	24	1830	52.11	27.23
51750865	2012	5	24	1840	51.56	27.23
51750865	2012	5	24	1850	51.02	27.23
51750865	2012	5	24	1860	51.31	27.22
51750865	2012	5	24	1870	51.72	27.23
51750865	2012	5	24	1880	51.78	27.21
51750865	2012	5	24	1890	51.83	27.21
51750865	2012	5	24	1900	51.97	27.21
51750865	2012	5	24	1910	52.04	27.21
51750865	2012	5	24	1920	52.01	27.21
51750865	2012	5	24	1930	52.16	27.21
51750865	2012	5	24	1940	52.22	27.21
51750865	2012	5	24	1950	52.26	27.21
51750865	2012	5	24	1960	52.18	27.21
51750865	2012	5	24	1970	51.86	27.21
51750865	2012	5	24	1980	51.8	27.21
51750865	2012	5	24	1990	51.58	27.21
51750865	2012	5	24	2000	51.64	27.21
51750865	2012	5	24	2010	51.93	27.22
51750865	2012	5	24	2020	52.06	27.22
51750865	2012	5	24	2030	52.81	27.21
51750865	2012	5	24	2040	53.92	27.21
51750865	2012	5	24	2050	55.23	27.2
51750865	2012	5	24	2060	56.71	27.19
51750865	2012	5	24	2070	58.51	27.17
51750865	2012	5	24	2080	59.83	27.17
51750865	2012	5	24	2090	61.37	27.14
51750865	2012	5	24	2100	62.85	27.13
51750865	2012	5	24	2110	64.19	27.05
51750865	2012	5	24	2120	65.71	26.93
51750865	2012	5	24	2130	67.36	26.91
51750865	2012	5	24	2140	68.88	26.86
51750865	2012	5	24	2150	70.41	26.75
51750865	2012	5	24	2160	72.35	26.64
51750865	2012	5	24	2170	74.55	26.51
51750865	2012	5	24	2180	76.28	26.32
51750865	2012	5	24	2190	77.84	26.07
51750865	2012	5	24	2200	80.07	25.98
51750865	2012	5	24	2210	82.21	25.92
51750865	2012	5	24	2220	84.44	25.89
51750865	2012	5	24	2230	85.2	25.82
51750865	2012	5	24	2240	85.3	25.78

51750865	2012	5	24	2250	85.51	25.64
51750865	2012	5	24	2260	85.78	25.62
51750865	2012	5	24	2270	85.46	25.49
51750865	2012	5	24	2280	85.52	25.46
51750865	2012	5	24	2290	85.73	25.42
51750865	2012	5	24	2300	85.6	25.36
51750865	2012	5	24	2310	85.52	25.3
51750865	2012	5	24	2320	85.36	25.27
51750865	2012	5	24	2330	85.11	25.21
51750865	2012	5	24	2340	84.75	25.05
51750865	2012	5	24	2350	85.39	25.03
51750865	2012	5	24	2360	85.95	24.99
51750865	2012	5	24	2370	86.47	24.96
51750865	2012	5	24	2380	87.61	24.92
51750865	2012	5	24	2390	89.09	24.85
51750865	2012	5	24	2400	90.67	24.75
51750865	2012	5	24	2410	91.93	24.65
51750865	2012	5	24	2420	93.29	24.48
51750865	2012	5	24	2430	94.74	24.39
51750865	2012	5	24	2440	96.53	24.17
51750865	2012	5	24	2450	98.27	23.98
51750865	2012	5	24	2460	99.86	23.93
51750865	2012	5	24	2470	101.93	23.87
51750865	2012	5	24	2480	103.76	23.78
51750865	2012	5	24	2490	105.63	23.67
51750865	2012	5	24	2500	107.9	23.59
51750865	2012	5	24	2510	110.18	23.46
51750865	2012	5	24	2520	112.24	23.38
51750865	2012	5	24	2530	114.25	23.3
51750865	2012	5	24	2540	116.3	23.13
51750865	2012	5	24	2550	118.68	22.96
51750865	2012	5	24	2560	120.26	22.9
51750865	2012	5	24	2570	120.9	22.85
51750865	2012	5	24	2580	121.96	22.79
51750865	2012	5	24	2590	123.52	22.7
51750865	2012	5	24	2600	124.28	22.64
51750865	2012	5	24	2610	124.5	22.56
51750865	2012	5	24	2620	124.79	22.44
51750865	2012	5	24	2630	125.38	22.37
51750865	2012	5	24	2640	125.54	22.33
51750865	2012	5	24	2650	125.5	22.28
51750865	2012	5	24	2660	125.73	22.25
51750865	2012	5	24	2670	125.78	22.19
51750865	2012	5	24	2680	125.61	22.15
51750865	2012	5	24	2690	125.59	22.1
51750865	2012	5	24	2700	125.45	22.07
51750865	2012	5	24	2710	125.4	22.04

51750865	2012	5	24	2720	125.57	22
51750865	2012	5	24	2730	125.7	22
51750865	2012	5	24	2740	125.41	21.92
51750865	2012	5	24	2750	125.12	21.84
51750865	2012	5	24	2760	125.38	21.81
51750865	2012	5	24	2770	126.18	21.78
51750865	2012	5	24	2780	127.53	21.75
51750865	2012	5	24	2790	129.53	21.64
51750865	2012	5	24	2800	130.86	21.56
51750865	2012	5	24	2810	131.8	21.53
51750865	2012	5	24	2820	133.65	21.44
51750865	2012	5	24	2830	135.59	21.39
51750865	2012	5	24	2840	136.39	21.32
51750865	2012	5	24	2850	136.7	21.28
51750865	2012	5	24	2860	136.84	21.24
51750865	2012	5	24	2870	136.76	21.17
51750865	2012	5	24	2880	136.56	21.1
51750865	2012	5	24	2890	136.38	21.07
51750865	2012	5	24	2900	136.8	21.02
51750865	2012	5	24	2910	137.46	20.92
51750865	2012	5	24	2920	138.62	20.82
51750865	2012	5	24	2930	139.56	20.72
51750865	2012	5	24	2940	140.24	20.64
51750865	2012	5	24	2950	140.8	20.55
51750865	2012	5	24	2960	141.56	20.43
51750865	2012	5	24	2970	142.28	20.31
51750865	2012	5	24	2980	142.9	20.28
51750865	2012	5	24	2990	143.23	20.24
51750865	2012	5	24	3000	144.08	20.19
51750865	2012	5	24	3010	144.11	20.17
51750865	2012	5	24	3020	143.75	20.14
51750865	2012	5	24	3030	143.8	20.1
51750865	2012	5	24	3040	143.24	20.06
51750865	2012	5	24	3050	143.63	20.03
51750865	2012	5	24	3060	143.42	19.96
51750865	2012	5	24	3070	143.32	19.93
51750865	2012	5	24	3080	143.64	19.92
51750865	2012	5	24	3090	143.54	19.91
51750865	2012	5	24	3100	143.09	19.89
51750865	2012	5	24	3110	142.55	19.88
51750865	2012	5	24	3120	142.88	19.77
51750865	2012	5	24	3130	142.91	19.74
51750865	2012	5	24	3140	142.73	19.74
51750865	2012	5	24	3150	142.74	19.71
51750865	2012	5	24	3160	142.61	19.67
51750865	2012	5	24	3170	142.4	19.66
51750865	2012	5	24	3180	142.53	19.64

51750865	2012	5	24	3190	142.18	19.62
51750865	2012	5	24	3200	141.68	19.6
51750865	2012	5	24	3210	141.77	19.57
51750865	2012	5	24	3220	141.9	19.55
51750865	2012	5	24	3230	141.93	19.53
51750865	2012	5	24	3240	142	19.51
51750865	2012	5	24	3250	141.97	19.5
51750865	2012	5	24	3260	141.84	19.49
51750865	2012	5	24	3270	141.68	19.46
51750865	2012	5	24	3280	141.77	19.43
51750865	2012	5	24	3290	141.39	19.42
51750865	2012	5	24	3300	141.15	19.39
51750865	2012	5	24	3310	140.61	19.38
51750865	2012	5	24	3320	140.08	19.38
51750865	2012	5	24	3330	139.62	19.35
51750865	2012	5	24	3340	139.89	19.32
51750865	2012	5	24	3350	139.33	19.32
51750865	2012	5	24	3360	139.09	19.31
51750865	2012	5	24	3370	139.02	19.28
51750865	2012	5	24	3380	139.04	19.28
51750865	2012	5	24	3390	139.05	19.28
51750865	2012	5	24	3400	139.01	19.27
51750865	2012	5	24	3410	138.63	19.26
51750865	2012	5	24	3420	137.89	19.25
51750865	2012	5	24	3430	137.47	19.24
51750865	2012	5	24	3440	137.95	19.23
51750865	2012	5	24	3450	137.82	19.22
51750865	2012	5	24	3460	138.04	19.22
51750865	2012	5	24	3470	138.41	19.22
51750865	2012	5	24	3480	138.43	19.22
51750865	2012	5	24	3490	138.28	19.21
51750865	2012	5	24	3500	138.52	19.21
51750865	2012	5	24	3510	138.42	19.21
51750865	2012	5	24	3520	138.26	19.21
51750865	2012	5	24	3530	137.35	19.21
51750865	2012	5	24	3540	136.61	19.2
51750865	2012	5	24	3550	135.73	19.21
51750865	2012	5	24	3560	135.05	19.25
51750865	2012	5	24	3570	134.4	19.31
51750865	2012	5	24	3580	133.26	19.35
51750865	2012	5	24	3590	131.62	19.42
51750865	2012	5	24	3600	130.27	19.5
51750865	2012	5	24	3610	128.88	19.6
51750865	2012	5	24	3620	127.08	19.67
51750865	2012	5	24	3630	126.1	19.74
51750865	2012	5	24	3640	125.17	19.84
51750865	2012	5	24	3650	123.88	19.91

51750865	2012	5	24	3660	123.3	19.98
51750865	2012	5	24	3670	122.35	20.07
51750865	2012	5	24	3680	121.39	20.13
51750865	2012	5	24	3690	120.96	20.18
51750865	2012	5	24	3700	119.69	20.27
51750865	2012	5	24	3710	118.68	20.36
51750865	2012	5	24	3720	117.07	20.41
51750865	2012	5	24	3730	115.24	20.46
51750865	2012	5	24	3740	114.35	20.53
51750865	2012	5	24	3750	113.81	20.58
51750865	2012	5	24	3760	112.36	20.65
51750865	2012	5	24	3770	110.64	20.71
51750865	2012	5	24	3780	109.02	20.78
51750865	2012	5	24	3790	108.07	20.87
51750865	2012	5	24	3800	107.54	20.95
51750865	2012	5	24	3810	107.59	21.04
51750865	2012	5	24	3820	107.65	21.07
51750865	2012	5	24	3830	107.27	21.08
51750865	2012	5	24	3840	105.59	21.12
51750865	2012	5	24	3850	104.94	21.17
51750865	2012	5	24	3860	104.89	21.22
51750865	2012	5	24	3870	104.83	21.25
51750865	2012	5	24	3880	104.6	21.27
51750865	2012	5	24	3890	104.65	21.32
51750865	2012	5	24	3900	104.87	21.38
51750865	2012	5	24	3910	104.44	21.42
51750865	2012	5	24	3920	102.95	21.46
51750865	2012	5	24	3930	100.54	21.5
51750865	2012	5	24	3940	98.14	21.53
51750865	2012	5	24	3950	95.79	21.57
51750865	2012	5	24	3960	93.51	21.6
51750865	2012	5	24	3970	92.18	21.64
51750865	2012	5	24	3980	90.95	21.7
51750865	2012	5	24	3990	89.1	21.74
51750865	2012	5	24	4000	86.93	21.82
51750865	2012	5	24	4010	85.01	21.92
51750865	2012	5	24	4020	83.44	22.1
51750865	2012	5	24	4030	81.99	22.22
51750865	2012	5	24	4040	79.89	22.32
51750865	2012	5	24	4050	77.67	22.44
51750865	2012	5	24	4060	75.84	22.58
51750865	2012	5	24	4070	74.76	22.69
51750865	2012	5	24	4080	73.96	22.81
51750865	2012	5	24	4090	73.07	22.89
51750865	2012	5	24	4100	71.46	22.97
51750865	2012	5	24	4110	70.17	23.05
51750865	2012	5	24	4120	69.74	23.14

51750865	2012	5	24	4130	69.52	23.23
51750865	2012	5	24	4140	68.19	23.28
51750865	2012	5	24	4150	66.61	23.37
51750865	2012	5	24	4160	65.4	23.45
51750865	2012	5	24	4170	63.94	23.62
51750865	2012	5	24	4180	62.66	23.81
51750865	2012	5	24	4190	61.12	24
51750865	2012	5	24	4200	59.76	24.19
51750865	2012	5	24	4210	58.56	24.35
51750865	2012	5	24	4220	57.26	24.48
51750865	2012	5	24	4230	56.17	24.58
51750865	2012	5	24	4240	54.66	24.78
51750865	2012	5	24	4250	53.28	24.92
51750865	2012	5	24	4260	51.59	25.06
51750865	2012	5	24	4270	50.06	25.25
51750865	2012	5	24	4280	48.55	25.35
51750865	2012	5	24	4290	47.38	25.54
51750865	2012	5	24	4300	46.4	25.65
51750865	2012	5	24	4310	45.69	25.75
51750865	2012	5	24	4320	44.6	25.8
51750865	2012	5	24	4330	43.68	25.88
51750865	2012	5	24	4340	42.68	25.96
51750865	2012	5	24	4350	41.68	26
51750865	2012	5	24	4360	41.15	26.08
51750865	2012	5	24	4370	41.53	26.15
51750865	2012	5	24	4380	41.84	26.19
51750865	2012	5	24	4390	41.85	26.24
51750865	2012	5	24	4400	42.15	26.28
51750865	2012	5	24	4410	42.17	26.33
51750865	2012	5	24	4420	42.28	26.37
51750865	2012	5	24	4430	41.9	26.41
51750865	2012	5	24	4440	40.74	26.46
51750865	2012	5	24	4450	39.84	26.5
51750865	2012	5	24	4460	39.12	26.54
51750865	2012	5	24	4470	37.92	26.57
51750865	2012	5	24	4480	36.86	26.62
51750865	2012	5	24	4490	35.79	26.65
51750865	2012	5	24	4500	34.39	26.68
51750865	2012	5	24	4510	33.24	26.72
51750865	2012	5	24	4520	31.89	26.76
51750865	2012	5	24	4530	30.52	26.8
51750865	2012	5	24	4540	29.19	26.83
51750865	2012	5	24	4550	27.87	26.86
51750865	2012	5	24	4560	26.56	26.89
51750865	2012	5	24	4570	25.37	26.92
51750865	2012	5	24	4580	25.61	26.95
51750865	2012	5	24	4590	26.12	27

51750865	2012	5	24	4600	26.16	27
51750865	2012	5	24	4610	26.04	27.02
51750865	2012	5	24	4620	26.1	27.01
51750865	2012	5	24	4630	25.98	27.02
51750865	2012	5	24	4640	24.83	27.03
51750865	2012	5	24	4650	23.23	27.07
51750865	2012	5	24	4660	21.57	27.09
51750865	2012	5	24	4670	20.21	27.12
51750865	2012	5	24	4680	18.62	27.14
51750865	2012	5	24	4690	16.94	27.17
51750865	2012	5	24	4700	15.46	27.21
51750865	2012	5	24	4710	14.08	27.24
51750865	2012	5	24	4720	12.98	27.26
51750865	2012	5	24	4730	12.75	27.28
51750865	2012	5	24	4740	12.64	27.31
51750865	2012	5	24	4750	12.2	27.32
51750865	2012	5	24	4760	10.73	27.34
51750865	2012	5	24	4770	8.93	27.36
51750865	2012	5	24	4780	7.34	27.39
51750865	2012	5	24	4790	5.88	27.41
51750865	2012	5	24	4800	4.69	27.42
51750865	2012	5	24	4810	3.41	27.44
51750865	2012	5	24	4820	3.38	27.46
51750865	2012	5	24	4830	2.83	27.48
51750865	2012	5	24	4840	2.5	27.49
51750865	2012	5	24	4850	2.8	27.51
51750865	2012	5	24	4860	2.78	27.51
51750865	2012	5	24	4870	2.56	27.54
51750865	2012	5	24	4880	2.17	27.57
51750865	2012	5	24	4890	0.63	27.57
51750865	2012	5	24	4900	0.61	27.58
51750865	2012	5	24	4910	0.64	27.6
51750865	2012	5	24	4920	0.64	27.63
51750865	2012	5	24	4930	0.66	27.69
51750865	2012	5	24	4940	0.6	27.71
51750865	2012	5	24	4950	0.64	27.71
51750865	2012	5	24	4960	0.58	27.72
51750865	2012	5	24	4970	0.59	27.75
51750865	2012	5	24	4980	0.58	27.76
51750865	2012	5	24	4990	0.61	27.78
51750865	2012	5	24	5000	0.56	27.78
51750865	2012	5	24	5010	0.57	27.79
51750865	2012	5	24	5020	0.59	27.8
51750865	2012	5	24	5030	0.56	27.82
51817505	2012	5	25	0	1.34	27.54
51817505	2012	5	25	10	1.9	27.55
51817505	2012	5	25	20	2.48	27.54

51817505	2012	5	25	30	2.77	27.54
51817505	2012	5	25	40	2.93	27.54
51817505	2012	5	25	50	2.71	27.54
51817505	2012	5	25	60	2.6	27.55
51817505	2012	5	25	70	3.11	27.54
51817505	2012	5	25	80	3.2	27.54
51817505	2012	5	25	90	4.11	27.54
51817505	2012	5	25	100	5.36	27.53
51817505	2012	5	25	110	6.19	27.52
51817505	2012	5	25	120	6.69	27.51
51817505	2012	5	25	130	8.83	27.5
51817505	2012	5	25	140	10.51	27.49
51817505	2012	5	25	150	12.15	27.46
51817505	2012	5	25	160	13.51	27.46
51817505	2012	5	25	170	15.46	27.43
51817505	2012	5	25	180	17.51	27.42
51817505	2012	5	25	190	19.11	27.4
51817505	2012	5	25	200	21.32	27.38
51817505	2012	5	25	210	23.93	27.36
51817505	2012	5	25	220	25.96	27.35
51817505	2012	5	25	230	27.65	27.33
51817505	2012	5	25	240	29.28	27.31
51817505	2012	5	25	250	31.51	27.3
51817505	2012	5	25	260	33.56	27.27
51817505	2012	5	25	270	35.83	27.26
51817505	2012	5	25	280	37.67	27.25
51817505	2012	5	25	290	39.38	27.23
51817505	2012	5	25	300	41.38	27.21
51817505	2012	5	25	310	43.36	27.21
51817505	2012	5	25	320	45.26	27.17
51817505	2012	5	25	330	47.33	27.16
51817505	2012	5	25	340	49.43	27.14
51817505	2012	5	25	350	51.85	27.13
51817505	2012	5	25	360	54.02	27.12
51817505	2012	5	25	370	56.51	27.09
51817505	2012	5	25	380	59.12	27.07
51817505	2012	5	25	390	61.79	27.03
51817505	2012	5	25	400	63.04	27
51817505	2012	5	25	410	63.54	26.99
51817505	2012	5	25	420	64.09	26.96
51817505	2012	5	25	430	65.41	26.95
51817505	2012	5	25	440	66.8	26.9
51817505	2012	5	25	450	67.83	26.89
51817505	2012	5	25	460	68.99	26.85
51817505	2012	5	25	470	70.16	26.83
51817505	2012	5	25	480	71.7	26.8
51817505	2012	5	25	490	73.27	26.78

51817505	2012	5	25	500	75.39	26.78
51817505	2012	5	25	510	77.41	26.75
51817505	2012	5	25	520	79.22	26.7
51817505	2012	5	25	530	81.38	26.63
51817505	2012	5	25	540	82.76	26.59
51817505	2012	5	25	550	84.42	26.46
51817505	2012	5	25	560	86.38	26.32
51817505	2012	5	25	570	88.45	26.21
51817505	2012	5	25	580	90.75	26.11
51817505	2012	5	25	590	93.25	25.99
51817505	2012	5	25	600	95.44	25.86
51817505	2012	5	25	610	97.93	25.5
51817505	2012	5	25	620	100.28	25.38
51817505	2012	5	25	630	102.72	25.19
51817505	2012	5	25	640	104.06	25.11
51817505	2012	5	25	650	104.82	25.07
51817505	2012	5	25	660	105.6	24.98
51817505	2012	5	25	670	106.22	24.8
51817505	2012	5	25	680	106.54	24.67
51817505	2012	5	25	690	106.5	24.53
51817505	2012	5	25	700	106.5	24.4
51817505	2012	5	25	710	106.88	24.3
51817505	2012	5	25	720	107.38	24.19
51817505	2012	5	25	730	107.92	24.07
51817505	2012	5	25	740	108.34	23.98
51817505	2012	5	25	750	108.31	23.92
51817505	2012	5	25	760	108.03	23.82
51817505	2012	5	25	770	107.86	23.75
51817505	2012	5	25	780	107.74	23.68
51817505	2012	5	25	790	107.02	23.59
51817505	2012	5	25	800	106.08	23.5
51817505	2012	5	25	810	105.53	23.45
51817505	2012	5	25	820	105.18	23.39
51817505	2012	5	25	830	105.02	23.34
51817505	2012	5	25	840	104.99	23.29
51817505	2012	5	25	850	104.97	23.25
51817505	2012	5	25	860	105.13	23.2
51817505	2012	5	25	870	105.3	23.15
51817505	2012	5	25	880	105.54	23.11
51817505	2012	5	25	890	106.14	23.06
51817505	2012	5	25	900	106.5	23.01
51817505	2012	5	25	910	106.63	22.98
51817505	2012	5	25	920	106.76	22.93
51817505	2012	5	25	930	107.11	22.89
51817505	2012	5	25	940	107.5	22.85
51817505	2012	5	25	950	107.46	22.8
51817505	2012	5	25	960	107.04	22.76

51817505	2012	5	25	970	106.92	22.72
51817505	2012	5	25	980	107.42	22.68
51817505	2012	5	25	990	107.73	22.66
51817505	2012	5	25	1000	107.85	22.64
51817505	2012	5	25	1010	107.54	22.6
51817505	2012	5	25	1020	107.29	22.57
51817505	2012	5	25	1030	106.94	22.55
51817505	2012	5	25	1040	106.73	22.52
51817505	2012	5	25	1050	106.6	22.5
51817505	2012	5	25	1060	106.75	22.47
51817505	2012	5	25	1070	106.94	22.46
51817505	2012	5	25	1080	106.91	22.44
51817505	2012	5	25	1090	106.67	22.42
51817505	2012	5	25	1100	106.43	22.43
51817505	2012	5	25	1110	106.45	22.42
51817505	2012	5	25	1120	106.33	22.4
51817505	2012	5	25	1130	106.5	22.39
51817505	2012	5	25	1140	106.7	22.38
51817505	2012	5	25	1150	106.9	22.37
51817505	2012	5	25	1160	106.45	22.36
51817505	2012	5	25	1170	106.09	22.36
51817505	2012	5	25	1180	106.17	22.34
51817505	2012	5	25	1190	106.66	22.34
51817505	2012	5	25	1200	107.06	22.33
51817505	2012	5	25	1210	106.41	22.34
51817505	2012	5	25	1220	105.86	22.33
51817505	2012	5	25	1230	105.86	22.33
51817505	2012	5	25	1240	105.78	22.33
51817505	2012	5	25	1250	105.69	22.32
51817505	2012	5	25	1260	105.7	22.32
51817505	2012	5	25	1270	105.41	22.32
51817505	2012	5	25	1280	105.61	22.32
51817505	2012	5	25	1290	105.95	22.32
51817505	2012	5	25	1300	106.2	22.33
51817505	2012	5	25	1310	106.39	22.32
51817505	2012	5	25	1320	106.57	22.32
51817505	2012	5	25	1330	106.69	22.32
51817505	2012	5	25	1340	106.78	22.32
51817505	2012	5	25	1350	106.91	22.32
51817505	2012	5	25	1360	107.15	22.32
51817505	2012	5	25	1370	107.52	22.32
51817505	2012	5	25	1380	107.64	22.32
51817505	2012	5	25	1390	107.67	22.31
51817505	2012	5	25	1400	107.25	22.31
51817505	2012	5	25	1410	106.65	22.31
51817505	2012	5	25	1420	106.73	22.3
51817505	2012	5	25	1430	106.94	22.28

51817505	2012	5	25	1440	107.13	22.26
51817505	2012	5	25	1450	107.26	22.25
51817505	2012	5	25	1460	107.19	22.25
51817505	2012	5	25	1470	107.32	22.25
51817505	2012	5	25	1480	107.39	22.24
51817505	2012	5	25	1490	107.36	22.23
51817505	2012	5	25	1500	107.13	22.22
51817505	2012	5	25	1510	106.97	22.21
51817505	2012	5	25	1520	106.97	22.21
51817505	2012	5	25	1530	106.8	22.22
51817505	2012	5	25	1540	106.47	22.21
51817505	2012	5	25	1550	106.51	22.21
51817505	2012	5	25	1560	106.12	22.21
51817505	2012	5	25	1570	105.87	22.21
51817505	2012	5	25	1580	105.59	22.23
51817505	2012	5	25	1590	105.27	22.23
51817505	2012	5	25	1600	105.04	22.24
51817505	2012	5	25	1610	104.71	22.24
51817505	2012	5	25	1620	104.53	22.25
51817505	2012	5	25	1630	104.38	22.25
51817505	2012	5	25	1640	104.14	22.25
51817505	2012	5	25	1650	104.12	22.26
51817505	2012	5	25	1660	104.05	22.27
51817505	2012	5	25	1670	104	22.27
51817505	2012	5	25	1680	104.2	22.28
51817505	2012	5	25	1690	104.25	22.28
51817505	2012	5	25	1700	103.92	22.28
51817505	2012	5	25	1710	103.69	22.28
51817505	2012	5	25	1720	103.8	22.28
51817505	2012	5	25	1730	104.12	22.28
51817505	2012	5	25	1740	103.88	22.29
51817505	2012	5	25	1750	104.01	22.29
51817505	2012	5	25	1760	104.42	22.29
51817505	2012	5	25	1770	104.75	22.31
51817505	2012	5	25	1780	105.16	22.32
51817505	2012	5	25	1790	105.47	22.32
51817505	2012	5	25	1800	105.9	22.32
51817505	2012	5	25	1810	106.27	22.32
51817505	2012	5	25	1820	106.69	22.32
51817505	2012	5	25	1830	106.69	22.32
51817505	2012	5	25	1840	106.58	22.31
51817505	2012	5	25	1850	106.55	22.3
51817505	2012	5	25	1860	106.67	22.29
51817505	2012	5	25	1870	106.54	22.28
51817505	2012	5	25	1880	106.68	22.28
51817505	2012	5	25	1890	106.9	22.28
51817505	2012	5	25	1900	106.81	22.27

51817505	2012	5	25	1910	106.71	22.27
51817505	2012	5	25	1920	106.76	22.27
51817505	2012	5	25	1930	106.82	22.25
51817505	2012	5	25	1940	107.04	22.25
51817505	2012	5	25	1950	107.26	22.25
51817505	2012	5	25	1960	106.88	22.25
51817505	2012	5	25	1970	106.79	22.25
51817505	2012	5	25	1980	106.9	22.25
51817505	2012	5	25	1990	107.12	22.23
51817505	2012	5	25	2000	107.38	22.23
51817505	2012	5	25	2010	107.13	22.21
51817505	2012	5	25	2020	107.11	22.21
51817505	2012	5	25	2030	107.27	22.2
51817505	2012	5	25	2040	107.4	22.2
51817505	2012	5	25	2050	107.73	22.19
51817505	2012	5	25	2060	107.77	22.17
51817505	2012	5	25	2070	107.56	22.17
51817505	2012	5	25	2080	107.81	22.15
51817505	2012	5	25	2090	107.94	22.14
51817505	2012	5	25	2100	107.77	22.13
51817505	2012	5	25	2110	107.77	22.13
51817505	2012	5	25	2120	107.5	22.1
51817505	2012	5	25	2130	106.97	22.11
51817505	2012	5	25	2140	106.88	22.1
51817505	2012	5	25	2150	106.42	22.09
51817505	2012	5	25	2160	106.35	22.08
51817505	2012	5	25	2170	106.36	22.07
51817505	2012	5	25	2180	106.2	22.07
51817505	2012	5	25	2190	105.99	22.07
51817505	2012	5	25	2200	105.35	22.06
51817505	2012	5	25	2210	105.08	22.06
51817505	2012	5	25	2220	105.1	22.06
51817505	2012	5	25	2230	105.31	22.05
51817505	2012	5	25	2240	105.42	22.03
51817505	2012	5	25	2250	105.26	22.03
51817505	2012	5	25	2260	105.25	22.03
51817505	2012	5	25	2270	105.41	22.03
51817505	2012	5	25	2280	105.43	22.02
51817505	2012	5	25	2290	105.16	22.02
51817505	2012	5	25	2300	105	22
51817505	2012	5	25	2310	105.17	22
51817505	2012	5	25	2320	105.39	22
51817505	2012	5	25	2330	105.29	22
51817505	2012	5	25	2340	105.19	21.99
51817505	2012	5	25	2350	105.22	21.99
51817505	2012	5	25	2360	105.57	21.98
51817505	2012	5	25	2370	105.83	21.97

51817505	2012	5	25	2380	105.68	21.96
51817505	2012	5	25	2390	105.51	21.96
51817505	2012	5	25	2400	104.74	21.96
51817505	2012	5	25	2410	103.71	21.97
51817505	2012	5	25	2420	102.64	21.96
51817505	2012	5	25	2430	101.77	21.97
51817505	2012	5	25	2440	101.65	21.97
51817505	2012	5	25	2450	101.99	21.98
51817505	2012	5	25	2460	101.99	22
51817505	2012	5	25	2470	101.95	22.02
51817505	2012	5	25	2480	101.99	22.04
51817505	2012	5	25	2490	101.77	22.06
51817505	2012	5	25	2500	101.81	22.08
51817505	2012	5	25	2510	101.81	22.1
51817505	2012	5	25	2520	101.85	22.14
51817505	2012	5	25	2530	101.52	22.16
51817505	2012	5	25	2540	101.73	22.2
51817505	2012	5	25	2550	101.88	22.25
51817505	2012	5	25	2560	101.55	22.27
51817505	2012	5	25	2570	101.66	22.29
51817505	2012	5	25	2580	101.03	22.32
51817505	2012	5	25	2590	100.36	22.34
51817505	2012	5	25	2600	99.27	22.37
51817505	2012	5	25	2610	98.55	22.42
51817505	2012	5	25	2620	97.81	22.46
51817505	2012	5	25	2630	97.02	22.5
51817505	2012	5	25	2640	96.52	22.55
51817505	2012	5	25	2650	96.65	22.59
51817505	2012	5	25	2660	96.84	22.62
51817505	2012	5	25	2670	96.81	22.67
51817505	2012	5	25	2680	96.78	22.67
51817505	2012	5	25	2690	97.04	22.7
51817505	2012	5	25	2700	97.08	22.72
51817505	2012	5	25	2710	97.16	22.76
51817505	2012	5	25	2720	96.81	22.79
51817505	2012	5	25	2730	96.39	22.83
51817505	2012	5	25	2740	95.92	22.87
51817505	2012	5	25	2750	96.07	22.89
51817505	2012	5	25	2760	96.63	22.91
51817505	2012	5	25	2770	96.74	22.94
51817505	2012	5	25	2780	96.53	22.96
51817505	2012	5	25	2790	96.55	22.99
51817505	2012	5	25	2800	96.29	23
51817505	2012	5	25	2810	95.57	23.03
51817505	2012	5	25	2820	95	23.06
51817505	2012	5	25	2830	94.6	23.09
51817505	2012	5	25	2840	94.34	23.12

51817505	2012	5	25	2850	94.24	23.14
51817505	2012	5	25	2860	94.84	23.16
51817505	2012	5	25	2870	95.78	23.17
51817505	2012	5	25	2880	96.72	23.17
51817505	2012	5	25	2890	97.58	23.18
51817505	2012	5	25	2900	97.55	23.19
51817505	2012	5	25	2910	97.73	23.2
51817505	2012	5	25	2920	98.07	23.21
51817505	2012	5	25	2930	98.26	23.22
51817505	2012	5	25	2940	98.47	23.24
51817505	2012	5	25	2950	98.45	23.25
51817505	2012	5	25	2960	98.5	23.27
51817505	2012	5	25	2970	98.53	23.27
51817505	2012	5	25	2980	98.59	23.28
51817505	2012	5	25	2990	97.9	23.29
51817505	2012	5	25	3000	97.67	23.31
51817505	2012	5	25	3010	97.91	23.32
51817505	2012	5	25	3020	97.59	23.32
51817505	2012	5	25	3030	97.9	23.34
51817505	2012	5	25	3040	97.94	23.35
51817505	2012	5	25	3050	97.92	23.35
51817505	2012	5	25	3060	97.56	23.36
51817505	2012	5	25	3070	97.86	23.37
51817505	2012	5	25	3080	98.27	23.38
51817505	2012	5	25	3090	98.41	23.39
51817505	2012	5	25	3100	98.42	23.39
51817505	2012	5	25	3110	98.54	23.39
51817505	2012	5	25	3120	98.56	23.39
51817505	2012	5	25	3130	98.73	23.4
51817505	2012	5	25	3140	99.07	23.41
51817505	2012	5	25	3150	99.24	23.41
51817505	2012	5	25	3160	99.37	23.42
51817505	2012	5	25	3170	99.6	23.42
51817505	2012	5	25	3180	99.46	23.42
51817505	2012	5	25	3190	98.37	23.43
51817505	2012	5	25	3200	97.02	23.44
51817505	2012	5	25	3210	96.18	23.45
51817505	2012	5	25	3220	95.17	23.46
51817505	2012	5	25	3230	95.45	23.46
51817505	2012	5	25	3240	94.41	23.48
51817505	2012	5	25	3250	93.59	23.48
51817505	2012	5	25	3260	93.01	23.5
51817505	2012	5	25	3270	93.22	23.5
51817505	2012	5	25	3280	93.07	23.51
51817505	2012	5	25	3290	92.9	23.52
51817505	2012	5	25	3300	93.02	23.53
51817505	2012	5	25	3310	93.37	23.53

51817505	2012	5	25	3320	93.74	23.54
51817505	2012	5	25	3330	93.84	23.54
51817505	2012	5	25	3340	94.3	23.55
51817505	2012	5	25	3350	94.65	23.56
51817505	2012	5	25	3360	94.97	23.57
51817505	2012	5	25	3370	94.86	23.57
51817505	2012	5	25	3380	94.77	23.57
51817505	2012	5	25	3390	94.8	23.58
51817505	2012	5	25	3400	94.98	23.58
51817505	2012	5	25	3410	95.34	23.59
51817505	2012	5	25	3420	95.55	23.58
51817505	2012	5	25	3430	95.04	23.59
51817505	2012	5	25	3440	95.03	23.59
51817505	2012	5	25	3450	94.84	23.6
51817505	2012	5	25	3460	94.6	23.6
51817505	2012	5	25	3470	94.79	23.6
51817505	2012	5	25	3480	95.08	23.6
51817505	2012	5	25	3490	95.36	23.6
51817505	2012	5	25	3500	95.37	23.6
51817505	2012	5	25	3510	95.51	23.6
51817505	2012	5	25	3520	95.61	23.6
51817505	2012	5	25	3530	95.79	23.6
51817505	2012	5	25	3540	95.97	23.6
51817505	2012	5	25	3550	96.3	23.6
51817505	2012	5	25	3560	96.66	23.6
51817505	2012	5	25	3570	96.62	23.6
51817505	2012	5	25	3580	96.64	23.6
51817505	2012	5	25	3590	96.88	23.6
51817505	2012	5	25	3600	96.9	23.6
51817505	2012	5	25	3610	96.76	23.6
51817505	2012	5	25	3620	96.33	23.6
51817505	2012	5	25	3630	96.33	23.6
51817505	2012	5	25	3640	96.51	23.6
51817505	2012	5	25	3650	96.64	23.6
51817505	2012	5	25	3660	96.11	23.6
51817505	2012	5	25	3670	95.93	23.6
51817505	2012	5	25	3680	95.9	23.6
51817505	2012	5	25	3690	96.09	23.6
51817505	2012	5	25	3700	96.27	23.6
51817505	2012	5	25	3710	96.39	23.6
51817505	2012	5	25	3720	96.64	23.59
51817505	2012	5	25	3730	96.38	23.59
51817505	2012	5	25	3740	96.03	23.59
51817505	2012	5	25	3750	95.39	23.6
51817505	2012	5	25	3760	95	23.6
51817505	2012	5	25	3770	94.51	23.6
51817505	2012	5	25	3780	93.2	23.6

51817505	2012	5	25	3790	92.49	23.61
51817505	2012	5	25	3800	92.09	23.6
51817505	2012	5	25	3810	91.01	23.62
51817505	2012	5	25	3820	89.88	23.63
51817505	2012	5	25	3830	89.2	23.64
51817505	2012	5	25	3840	88.64	23.64
51817505	2012	5	25	3850	88.47	23.64
51817505	2012	5	25	3860	87.96	23.65
51817505	2012	5	25	3870	87.47	23.67
51817505	2012	5	25	3880	87.36	23.67
51817505	2012	5	25	3890	87.13	23.67
51817505	2012	5	25	3900	87.27	23.67
51817505	2012	5	25	3910	86.99	23.68
51817505	2012	5	25	3920	86.83	23.69
51817505	2012	5	25	3930	86.45	23.69
51817505	2012	5	25	3940	85.61	23.71
51817505	2012	5	25	3950	85.7	23.71
51817505	2012	5	25	3960	86.13	23.72
51817505	2012	5	25	3970	86.26	23.75
51817505	2012	5	25	3980	86.63	23.77
51817505	2012	5	25	3990	86.86	23.8
51817505	2012	5	25	4000	87.15	23.84
51817505	2012	5	25	4010	87.08	23.86
51817505	2012	5	25	4020	86.8	23.92
51817505	2012	5	25	4030	86.75	23.98
51817505	2012	5	25	4040	87.16	24.07
51817505	2012	5	25	4050	87.71	24.1
51817505	2012	5	25	4060	87.35	24.14
51817505	2012	5	25	4070	86.27	24.19
51817505	2012	5	25	4080	85.18	24.25
51817505	2012	5	25	4090	85.33	24.29
51817505	2012	5	25	4100	85.73	24.32
51817505	2012	5	25	4110	86.21	24.38
51817505	2012	5	25	4120	86.71	24.42
51817505	2012	5	25	4130	86.04	24.51
51817505	2012	5	25	4140	85.7	24.56
51817505	2012	5	25	4150	85.13	24.64
51817505	2012	5	25	4160	84.39	24.65
51817505	2012	5	25	4170	84.01	24.69
51817505	2012	5	25	4180	83.8	24.77
51817505	2012	5	25	4190	83.92	24.82
51817505	2012	5	25	4200	84.25	24.89
51817505	2012	5	25	4210	84.44	24.93
51817505	2012	5	25	4220	84.83	24.97
51817505	2012	5	25	4230	84.97	25.01
51817505	2012	5	25	4240	84.95	25.05
51817505	2012	5	25	4250	84.78	25.08

51817505	2012	5	25	4260	84.64	25.1
51817505	2012	5	25	4270	84.19	25.14
51817505	2012	5	25	4280	84.81	25.19
51817505	2012	5	25	4290	85.43	25.24
51817505	2012	5	25	4300	86.27	25.26
51817505	2012	5	25	4310	86.89	25.3
51817505	2012	5	25	4320	87.62	25.32
51817505	2012	5	25	4330	88.69	25.29
51817505	2012	5	25	4340	89.32	25.25
51817505	2012	5	25	4350	90.04	25.19
51817505	2012	5	25	4360	90.43	25.15
51817505	2012	5	25	4370	90.95	25.1
51817505	2012	5	25	4380	91.61	25.05
51817505	2012	5	25	4390	92.07	25
51817505	2012	5	25	4400	92.75	24.95
51817505	2012	5	25	4410	93.31	24.9
51817505	2012	5	25	4420	92.93	24.85
51817505	2012	5	25	4430	92.28	24.78
51817505	2012	5	25	4440	91.1	24.71
51817505	2012	5	25	4450	90.11	24.64
51817505	2012	5	25	4460	89.32	24.6
51817505	2012	5	25	4470	87.79	24.54
51817505	2012	5	25	4480	86.49	24.55
51817505	2012	5	25	4490	84.95	24.64
51817505	2012	5	25	4500	83.55	24.69
51817505	2012	5	25	4510	82.66	24.76
51817505	2012	5	25	4520	81.73	24.85
51817505	2012	5	25	4530	81.21	24.93
51817505	2012	5	25	4540	80.54	25.02
51817505	2012	5	25	4550	80.26	25.08
51817505	2012	5	25	4560	79.53	25.14
51817505	2012	5	25	4570	79.39	25.25
51817505	2012	5	25	4580	78.89	25.28
51817505	2012	5	25	4590	78.37	25.34
51817505	2012	5	25	4600	78.4	25.37
51817505	2012	5	25	4610	78.8	25.4
51817505	2012	5	25	4620	78.9	25.44
51817505	2012	5	25	4630	79.4	25.48
51817505	2012	5	25	4640	80.16	25.53
51817505	2012	5	25	4650	81.28	25.54
51817505	2012	5	25	4660	81.86	25.58
51817505	2012	5	25	4670	82.1	25.6
51817505	2012	5	25	4680	82.15	25.62
51817505	2012	5	25	4690	82.27	25.64
51817505	2012	5	25	4700	82.91	25.66
51817505	2012	5	25	4710	83.56	25.67
51817505	2012	5	25	4720	84.07	25.68

51817505	2012	5	25	4730	84.4	25.71
51817505	2012	5	25	4740	85.05	25.72
51817505	2012	5	25	4750	85.24	25.74
51817505	2012	5	25	4760	85.16	25.76
51817505	2012	5	25	4770	85.09	25.77
51817505	2012	5	25	4780	85.3	25.78
51817505	2012	5	25	4790	86.41	25.8
51817505	2012	5	25	4800	87.51	25.81
51817505	2012	5	25	4810	88.38	25.81
51817505	2012	5	25	4820	88.93	25.79
51817505	2012	5	25	4830	88.41	25.62
51817505	2012	5	25	4840	88.55	25.55
51817505	2012	5	25	4850	89.55	25.32
51817505	2012	5	25	4860	89.39	25.27
51817505	2012	5	25	4870	87.87	25.2
51817505	2012	5	25	4880	86.39	25.14
51817505	2012	5	25	4890	85.6	25.09
51817505	2012	5	25	4900	84.85	25.07
51817505	2012	5	25	4910	83.89	25.09
51817505	2012	5	25	4920	83.55	25.14
51817505	2012	5	25	4930	83.85	25.21
51817505	2012	5	25	4940	83.77	25.25
51817505	2012	5	25	4950	82.95	25.3
51817505	2012	5	25	4960	83.85	25.34
51817505	2012	5	25	4970	85.02	25.36
51817505	2012	5	25	4980	85.4	25.4
51817505	2012	5	25	4990	84.69	25.45
[truncated: 524,582 more chars]
